# Current trends and projections for potential acupuncture needs globally and in China: evidence from the Global Burden of Disease Study 2021

**DOI:** 10.1186/s13020-025-01286-9

**Published:** 2026-01-07

**Authors:** Zihan Yin, Ziwen Chen, Fanrong Liang, Ling Zhao

**Affiliations:** 1 School of Acu-Mox and Tuina, Chengdu University of Traditional Chinese Medicine, 37 Shierqiao Road, Chengdu, 610075 Sichuan China; 2 Acupuncture Clinical Research Center of Sichuan Province, Chengdu, China

**Keywords:** Acupuncture, Global Burden of Disease, Global, China

## Abstract

**Background:**

Acupuncture plays a vital role in managing musculoskeletal, neurological, gastrointestinal, and cancer-related conditions and significantly improves individual quality of life and societal well-being. However, despite its demonstrated benefits, it remains under-prioritized and under-resourced globally and in China. Thus, the present study aimed to explore global and Chinese data on the number of patients who would benefit from acupuncture at least once during the course of their illness.

**Methods:**

To estimate the potential need for acupuncture, data from the Global Burden of Disease (GBD) 2021 database were used to calculate the prevalence, years lived with disability (YLDs), and estimated annual percentage change (EAPC) for eight disease categories (including 20 health conditions) identified as amenable to acupuncture, both globally and in China, thereby assessing current needs. Decomposition analysis was conducted to identify the key contributors to changes in acupuncture needs, and a Bayesian age-period-cohort model was applied to forecast future needs.

**Results:**

In 2021, an estimated 6.50 (95% uncertainty intervals [UI] 5.64 to 7.48) billion individuals globally had conditions that would benefit from acupuncture, contributing to 306.47 (95% UI 185.17 to 469.90) million YLDs—representing increases of 70.65% and 79.83% from 1990, with EAPCs of 0.02 and − 0.04, respectively. In China, 1.06 (95% UI 0.91 to 1.24) billion individuals had such conditions, accounting for 55.68 (95% UI 33.80 to 85.13) million YLDs—up 55.63% and 65.13% from 1990, with EAPCs of 0.10 and − 0.13, respectively. Globally and in China, neurological disorders represent the greatest need for acupuncture, with tension-type headaches being the leading specific condition. Decomposition analyses revealed an increased need for acupuncture, with a positive contribution from population growth and aging, both worldwide and in China. By 2045, the number of individuals projected to require acupuncture services is expected to reach 9.68 billion globally and 1.35 billion in China, contributing to 657.81 million and 80.76 million YLDs, respectively.

**Conclusions:**

Global and Chinese needs for acupuncture have risen markedly since 1990 and are projected to continue increasing through 2045. This challenges the traditional perception that acupuncture serves only a small portion of the population. Thus, this study emphasizes the urgent need to integrate acupuncture into modern primary healthcare systems to meet the increasing health needs of an aging and growing populations.

**Supplementary Information:**

The online version contains supplementary material available at 10.1186/s13020-025-01286-9.

## Background

Amid global demographic transitions and the increasing burden of diseases, acupuncture has gained prominence as a non-pharmacological, low-risk, and cost-effective therapeutic approach [1, 2]. As a fundamental element of traditional Chinese medicine, acupuncture has gained widespread clinical application globally and in China, underpinned by an increasing volume of high-quality randomized controlled trials, systematic reviews, and neurobiological research [3, 4]. Its clinical applications include musculoskeletal pain [5, 6], neurological disorders [7–10] and mental health disorders [11, 12], and even supportive care in cancer [13–15], substance use disorders [16], and infectious diseases [1, 12]. The growing inclusion of acupuncture in national guidelines [17–21] and health insurance schemes [22, 23] reflects its well-established therapeutic efficacy and, more importantly, underscores its critical role in alleviating pressure on health systems. By effectively managing chronic, function-limiting diseases through holistic, patient-centered treatment, acupuncture offers a sustainable and strategic solution to the growing needs of modern healthcare, especially in aging and growing populations.

Although the clinical applications and institutional support for acupuncture have expanded and its benefits are well established, it remains under-prioritized and under-resourced globally [1, 24]. By contrast, China has integrated acupuncture as a core component of its national healthcare system through dedicated policies, training programs, and infrastructure development, which has promoted its widespread application and sustained growth. However, both globally and in China, most existing studies have focused primarily on patient-reported utilization or isolated clinical outcomes, thus providing limited insights into the broader disease burden that could benefit from acupuncture. Few studies have examined long-term trends or projected future needs [1, 25]. Robust, data-driven estimates of acupuncture needs, grounded in standardized epidemiological indicators, are therefore urgently needed to inform effective service planning, workforce training, and health policy development [26, 27].

To address these gaps, the Global Burden of Disease (GBD) approach offers a valuable and objective way of assessing acupuncture needs at the population level. GBD Study is a widely recognized resource that quantifies disease prevalence, and disability across countries and populations [28]. Using data from the GBD 2021 dataset, which covers 371 conditions including key non-fatal outcomes such as prevalence and years lived with disability (YLDs), we can estimate the potential need for acupuncture by analyzing the prevalence, YLDs, and estimated annual percentage change (EAPC) of diseases amenable to acupuncture, both globally and in China [29]. Furthermore, the methodological transparency of the GBD and its data harmonization enable advanced statistical analyses, decomposition of disease burden drivers, and long-term projections [30]. These capabilities enable a robust, multidimensional assessment of the evolving landscape of potential need for acupuncture.

Building on data from the GBD 2021 dataset, the present study aimed to quantify and project the potential global and domestic needs for acupuncture services from 1990 to 2045. The study selected 20 health conditions spanning eight major disease categories—neurological, musculoskeletal, digestive, mental, gynecological, substance use disorders, infectious disorders, and neoplasms—based on robust clinical evidence and the World Health Organization guidelines on the advantages of acupuncture. Using standardized metrics such as prevalence and YLDs, we examined temporal trends from 1990 to 2021, performed decomposition analyses to identify key demographic and epidemiological drivers, and conducted stratified analyses according to age and sex. To estimate the future burden, this study applied the Bayesian age-period-cohort (BAPC). To our knowledge, this is the first study to offer a comprehensive population-based projection of acupuncture needs at both the global and national levels, providing timely evidence to inform strategic planning and guide the sustainable development of acupuncture services during ongoing global health system reforms.

## Methods

### Study population and data collection

This study analyzed acupuncture needs using data from the GBD 2021 dataset [31, 32], focusing on the prevalence and YLDs across eight disease categories and 20 health conditions identified as amenable to acupuncture. The data were obtained from the Global Health Data Exchange platform (https://vizhub.healthdata.org/gbd-results/). The Institutional Review Board waived the requirement for informed consent for this research, and all procedures adhered to the Strengthening the Reporting of Cohort Studies in Surgery guidelines [33] and the Guidelines for Accurate and Transparent Health Estimates Reporting [34].

### Selection of eight disease categories and 20 specific health conditions

Conditions were selected based on established evidence for acupuncture efficacy, drawing on World Health Organization benchmarks [26], high-quality reviews [1, 12, 14, 35–37], meta-analyses [13, 38, 39], and randomized controlled trials [7–10, 15, 40]. A total of 20 conditions based on eight disease categories were mapped to GBD cause codes: musculoskeletal disorders (neck pain [NP], low back pain [LBP], hip osteoarthritis [HOA], knee osteoarthritis [KOA], and rheumatoid arthritis [RA]); neurological disorders (tension-type headache [TTH]), migraine, stroke, Alzheimer’s disease [AD] and other dementias [ADOD], Parkinson’s disease [PD]), digestive disorders (upper digestive system diseases [UDSD], inflammatory bowel disease [IBD], pancreatitis); gynecological disorders (male and female infertility); mental disorders (depressive and anxiety disorders); substance use disorders (opioid use disorders); infectious disorders (herpes zoster); and neoplasms. Detailed definitions of the eight disease categories and 20 specific health conditions requiring acupuncture are provided in Supplementary Materials and Methods, Part I.

### Statistical analysis

Statistical analyses were performed using R version 4.4.2 (R Core Team, R Foundation for Statistical Computing, Vienna, Austria), and differences with *P* < 0.05 were considered to be statistically significant. The findings are expressed with detailed interpretations, incorporating effect sizes, confidence intervals (CIs), uncertainty intervals (UIs), and p*-*values to enhance the clarity and reliability of the results. To minimize potential biases, prevalence and YLDs were estimated using the Bayesian meta-regression framework implemented in IHME DisMod-MR 2.1, thereby optimizing the use of available evidence.

This study first analyzed trends from 1990 to 2021, both globally and in China, in (1) the overall need (maximum potential addressable burden) for acupuncture, estimated by aggregating the prevalence and YLDs for eight disease categories and 20 specific health conditions amenable to acupuncture, and (2) the prevalence and YLDs of each category and condition individually. Customized aggregations for this study were conducted at the draw level, and the 2.5th and 97.5th percentiles were applied to calculate the 95% UIs. Age-standardized rates (ASRs) and estimated annual percentage changes (EAPC) were calculated to assess temporal trends. The EAPCs and the corresponding 95% CIs were calculated by fitting a linear regression model to the logarithm of the ASRs. A trend was considered increasing if the EAPC was > 0 with a 95% CI lower bound > 0, decreasing if the EAPC was < 0 with a 95% CI upper bound < 0, and stable if the CI included zero. Subgroup analyses were also performed by age and sex to explore potential variations across populations [41].

Additionally, the decomposition method [42, 43] based on the Das-Gupta method was conducted to quantify the relative contributions of population aging, population growth, and epidemiological changes to the temporal variations in acupuncture needs, prevalence and YLDs across eight disease categories and 20 specific health conditions. Specifically, population aging represents shifts in the age distribution of the population, population growth captures changes in the total population size, and epidemiological changes reflect variations in age-specific disease rates arising from altered incidence, diagnosis, or treatment patterns. This approach, implemented in R software, enables the assessment of how demographic and epidemiological dynamics jointly influence the evolving burden of disease, and whether these changes have contributed to an overall increase or reduction in the burden. This method provides insights into the relative contributions of each factor to the evolving disease burden and clarifies the effects these changes have on the overall burden.

Finally, the overall acupuncture needs across eight categories, and 20 health condition burden trends from 2022 to 2045, both globally and in China, were predicted using the BAPC model. The model, implemented via the Integrated Nested Laplace Approximation in R software, extends the traditional generalized linear framework by dynamically integrating age, period, and cohort effects modeled as second-order random walks [44]. Weakly informative gamma (1, 0.00005) priors were assigned to the second-order random walks precision parameters to ensure a smooth temporal evolution while preventing overfitting. Model convergence and stability were evaluated by inspecting the effective sample sizes and trace plots. Predictive validity was assessed through 5-year backcasting, which showed good agreement between the observed and predicted values, with all observations falling within the 95% prediction intervals. Owing to its robustness and computational efficiency, the BAPC model has been widely applied in disease burden forecasting and is well-suited for estimating long-term trends.

## Results

### Trends in overall acupuncture needs globally and in China

**Fig. 1:**
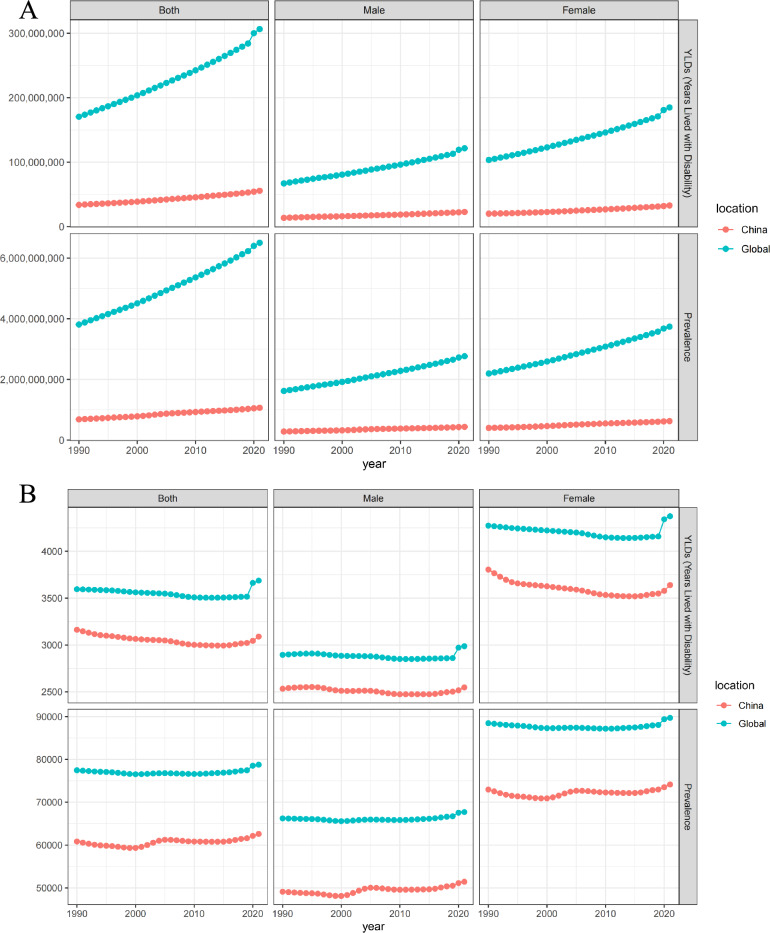
**A** Trends in acupuncture needs based on the number of prevalent cases and years lived with disability from 1990 to 2021; **B** Trends in acupuncture needs based on age-standardized rates of prevalence and years lived with disability from 1990 to 2021

Globally, the number of individuals with 20 health conditions that could benefit from acupuncture has significantly increased. From 1990 to 2021, the estimated number of prevalent cases surged by 70.65%, escalating from 3.81 (95% UI 3.29 to 4.41) billion in 1990 to 6.50 (95% UI 5.64 to 7.48) billion in 2021. YLD exhibited a parallel trend, increasing by 79.83%, from 170.42 (95% UI 101.15 to 264.80) million in 1990 to 306.47 (95% UI 185.17 to 469.90) million in 2021. The EAPCs were calculated to be 0.02 (95% CI -0.01 to 0.04) for age-specific prevalence rate (ASPR) and -0.04 (95% CI -0.09 to 0.00) for age-standardized YLD rate (Table 1, Table S1, Fig. 1).

**Table 1:** Estimated prevalence and years of life lived with disability for health conditions in need of acupuncture in 1990 and 2021 in the worldwide

Disease type	Prevalence				Years of life lived with disability			
	1990		2021		1990		2021	
	Number	ASR, per 100,000 persons	Number	ASR, per 100,000 persons	Number	ASR, per 100,000 persons	Number	ASR, per 100,000 persons
**Total**	3,810,752,632.38 (3,287,011,491.51, 4,409,268,074.94)	77,452.42 (67,279.15, 88,696.69)	6,503,158,089.04 (5,637,830,692.21, 7,476,087,549.08)	78,757.04 (68,246.25, 90,618.97)	170,422,166.71 (101,146,597.94, 264,800,842.69)	3594.8 (2167.97, 5519.73)	306,468,686.48 (185,172,954.93, 469,904,305.95)	3686.65 (2221.46, 5663.82)
**Musculoskeletal disorders**	684,487,785.38 (586,539,658.46, 787,290,426.95)	15,367.24 (13,170.82, 17,600.42)	1,263,417,793.78 (1,079,053,915.16, 1,448,146,719.04)	14,825.32 (12,701.33, 17,000.96)	61,522,957.64 (42,152,161.98, 87,121,953.53)	1342.94 (915.69, 1910.06)	106,085,664.63 (71,833,864.01, 151,770,401.34)	1252.72 (850.47, 1789.35)
Neck pain	114,601,451.02 (88,840,737.56, 141,520,154.71)	2436.71 (1912.98, 2992.64)	206,029,628.55 (161,756,682.55, 252,863,254.4)	2443.02 (1923.04, 3002.33)	11,442,356.21 (7,608,943.12, 16,334,312.96)	241.96 (162.05, 343.53)	20,415,496.55 (13,638,705.32, 28,856,642.59)	242.3 (162.6, 342.76)
Low back pain	386,731,360.72 (341,581,662.21, 434,164,619.58)	8391.58 (7381.14, 9367.39)	628,838,475.1 (551,834,407.36, 700,881,341)	7463.13 (6575.68, 8321.8)	43,386,225.77 (31,083,936.7, 58,355,209.83)	937.34 (669.13, 1261)	70,156,962.19 (50,194,204.95, 94,104,688.44)	832.18 (595.85, 1115.24)
Hip osteoarthritis	15,397,009.44 (11,798,401.89, 19,637,630.4)	391.66 (303.26, 496.51)	35,886,278.68 (27,630,665.5, 45,745,600.75)	416.01 (321.23, 529.74)	491,843.53 (231,455.91, 994,652.97)	12.43 (5.86, 25.21)	1,139,666.93 (535,835.99, 2,306,309.68)	13.19 (6.2, 26.65)
Knee osteoarthritis	159,798,909.45 (137,277,437.41, 182,882,553.69)	3964.75 (3411.86, 4536.4)	374,738,744.12 (321,858,981.94, 428,353,219.52)	4294.27 (3695.04, 4910.76)	5,145,338.7 (2,507,481.29, 9,953,258.48)	127.14 (62.17, 246.99)	12,019,069.9 (5,858,107.88, 23,267,858.34)	137.59 (67.08, 266.87)
Rheumatoid arthritis	7,959,054.74 (7,041,419.4, 9,085,468.56)	182.54 (161.59, 207.48)	17,924,667.32 (15,973,177.81, 20,303,303.37)	208.9 (186.34, 236.33)	1,057,193.43 (720,344.96, 1,484,519.29)	24.07 (16.48, 33.33)	2,354,469.06 (1,607,009.86, 3,234,902.28)	27.45 (18.73, 37.82)
**Neurological disorders**	2,094,294,892.04 (1,816,672,564.87, 2,395,912,780.99)	40,892.11 (35,825.88, 46,251.02)	3,332,486,075.59 (2,921,256,918.22, 3,779,905,998.53)	40,943.27 (35,827.85, 46,447.25)	43,131,216.57 (14,021,845.74, 86,492,169.47)	927.21 (343.16, 1775.99)	76,438,503.79 (28,200,646.86, 147,020,952.93)	928.63 (337.14, 1793.57)
Tension-type headache	1,286,366,671.75 (1,122,503,420.79, 1,467,160,187.52)	24,904.85 (21,960.05, 28,038.8)	2,011,612,877.49 (1,776,544,390.83, 2,270,860,638.85)	24,764.77 (21,863.62, 27,954.74)	2,848,687.62 (820,890.75, 9,563,745.41)	56.99 (16.79, 186.13)	4,596,785.25 (1,347,300.84, 15,012,932.75)	55.69 (16.13, 185.07)
Migraine	732,564,462.68 (624,559,243.92, 847,058,436.26)	14,027.65 (12,063.37, 16,078.07)	1,158,432,823.82 (995,861,966.38, 1,331,312,506.13)	14,246.55 (12,194.12, 16,378.7)	27,412,196.29 (4,076,605.01, 60,325,805.84)	526.76 (83.36, 1145.92)	43,378,889.81 (6,732,642.21, 95,079,454.09)	532.7 (80.57, 1167.71)
Stroke	50,415,602.15 (47,793,499.16, 53,227,185.77)	1201.11 (1137.99, 1271.32)	93,816,414.1 (89,030,218.63, 99,335,466.96)	1099.31 (1044.17, 1162.11)	8,010,313.68 (5,777,993.46, 10,180,918.82)	192.93 (139.37, 245.29)	15,210,424.48 (10,985,667.17, 19,424,864.47)	178.67 (128.85, 227.7)
Alzheimer's disease and other dementias	21,799,760.9 (19,067,087.2, 24,837,693.05)	672.22 (588.73, 763.95)	56,856,688.21 (49,382,064.01, 64,977,511.92)	694.01 (602.88, 794.08)	4,409,783.37 (3,029,438.98, 5,820,653.8)	138.31 (94.96, 182.44)	11,582,108.01 (7,961,941.52, 15,296,793.45)	141.95 (97.72, 187.2)
Parkinson's disease	3,148,394.56 (2,749,313.8, 3,629,278.39)	86.28 (75.74, 98.88)	11,767,271.97 (10,438,278.37, 13,419,874.68)	138.63 (123.06, 157.62)	450,235.6 (316,917.53, 601,045.6)	12.22 (8.68, 16.22)	1,670,296.23 (1,173,095.12, 2,206,908.19)	19.62 (13.86, 25.88)
**Digestive disorders**	6,318,548.46 (4,934,850.81, 8,162,130.96)	141.76 (110.25, 182.86)	9,730,459.46 (7,688,877.2, 12,350,352.09)	113.87 (90.06, 144.15)	584,187.72 (343,752.37, 898,364.8)	12.94 (7.63, 19.92)	946,201.6 (569,168.6, 1,430,736.39)	11.1 (6.68, 16.76)
Upper digestive system diseases	477,417,883.22 (425,347,947.18, 537,885,606.73)	10,084.4 (8992.81, 11,230.74)	864,284,077.51 (771,564,187.07, 963,733,781.73)	10,299.18 (9218.78, 11,519.63)	4,901,507.24 (2,901,026.64, 7,988,584.92)	103.49 (61.07, 168.16)	8,379,627.39 (4,817,164.19, 13,840,560.14)	99.92 (57.25, 165.16)
Inflammatory bowel disease	2,170,243.3 (1,892,401.81, 2,522,561.3)	48.02 (41.94, 55.78)	3,830,119.27 (3,312,834, 4,511,554.52)	44.88 (38.8, 52.86)	330,876.36 (222,761.23, 456,115.44)	7.27 (4.93, 10.01)	579,202.54 (391,900.57, 799,563.71)	6.79 (4.59, 9.34)
Pancreatitis	4,148,305.16 (3,042,449, 5,639,569.66)	93.74 (68.31, 127.08)	5,900,340.2 (4,376,043.2, 7,838,797.57)	68.99 (51.26, 91.29)	253,311.36 (120,991.13, 442,249.37)	5.66 (2.7, 9.9)	366,999.06 (177,268.03, 631,172.69)	4.31 (2.09, 7.42)
**Genecological disorders**	91,180,381.62 (51,350,652.04, 154,779,554.72)	1705.44 (954.67, 2900.06)	165,090,277.54 (91,220,072.36, 283,753,538.26)	2052.24 (1135.78, 3504.87)	507,805.76 (181,355.5, 1,233,325.94)	9.45 (3.41, 23.15)	918,747.11 (329,446.08, 2,221,233.24)	11.44 (4.09, 27.62)
Male infertility	31,490,381.81 (18,725,068.36, 50,165,061.42)	587.15 (352.91, 941.53)	55,000,818.26 (32,611,257.3, 88,727,953.05)	684.89 (405.44, 1099.04)	181,868.97 (66,532.49, 425,578.94)	3.37 (1.25, 7.91)	317,613.61 (116,288.11, 752,758.23)	3.96 (1.44, 9.38)
Female infertility	59,689,999.8 (32,625,583.68, 104,614,493.31)	1118.29 (601.76, 1958.53)	110,089,459.27 (58,608,815.06, 195,025,585.22)	1367.36 (730.34, 2405.83)	325,936.79 (114,823.01, 807,747)	6.08 (2.16, 15.24)	601,133.5 (213,157.97, 1,468,475.01)	7.48 (2.65, 18.23)
**Mental disorders**	369,263,347.55 (323,571,363.98, 425,898,535.12)	7346.02 (6486.6, 8391.71)	691,623,603.49 (604,927,466.1, 796,069,018.05)	8428.69 (7349.54, 9721.09)	52,671,397.71 (36,562,858.08, 71,753,183.6)	1044.24 (727.15, 1421.73)	98,840,006.43 (68,736,713.81, 134,267,991.37)	1205.48 (838.25, 1640.08)
Depressive disorders	176,327,213.74 (158,931,450.32, 199,374,726.19)	3599.67 (3251.91, 4023.21)	332,410,333.27 (297,742,040.84, 376,102,441.53)	4006.82 (3581.26, 4539.01)	29,674,665.8 (20,748,742.32, 40,205,907.65)	600.52 (420.94, 818.45)	56,330,361.15 (39,339,990.83, 76,538,170.96)	681.14 (475.19, 923.83)
Anxiety disorders	192,936,133.81 (164,639,913.66, 226,523,808.92)	3746.36 (3234.69, 4368.51)	359,213,270.22 (307,185,425.26, 419,966,576.52)	4421.87 (3768.28, 5182.08)	22,996,731.91 (15,814,115.76, 31,547,275.96)	443.73 (306.21, 603.28)	42,509,645.28 (29,396,722.99, 57,729,820.42)	524.33 (363.06, 716.25)
**Substance use disorders**								
Opioid use disorders	8,120,813.52 (6,801,332.78, 9,596,422.15)	154.59 (131.06, 181.26)	16,164,875.92 (14,133,119.75, 18,431,509.71)	198.49 (173.42, 227.22)	3,374,229.36 (2,241,241.82, 4,470,626.88)	63.95 (42.76, 83.3)	6,636,467.31 (4,606,665.22, 8,617,061.59)	81.59 (56.77, 106.19)
**Infectious disorders**								
Varicella and herpes zoster	3,371,208.15 (2,914,533.62, 3,885,486.4)	66.67 (56.81, 77.22)	5,282,097.14 (4,407,183.35, 6,182,739.63)	67.16 (56.99, 77.81)	126,545 (73,891.43, 195,810.36)	2.69 (1.56, 4.16)	224,886.33 (128,628.98, 346,705.04)	2.71 (1.56, 4.18)
**Neoplasms**	76,297,772.45 (68,878,587.77, 85,857,130.92)	1694.19 (1550.25, 1881.39)	155,078,828.61 (143,578,953, 167,513,892.03)	1828.81 (1692.5, 1975.98)	3,602,319.71 (2,668,464.38, 4,646,823.18)	87.9 (65.54, 113.26)	7,998,581.89 (5,950,657.17, 10,388,663.9)	93.07 (69.25, 120.92)

ASR: age-standardized rates

In China, the number of individuals with 20 health conditions that could benefit from acupuncture has significantly increased. From 1990 to 2021, estimated prevalent cases surged by 55.63%, escalating from 683.42 (95% UI 578.02 to 805.05) million in 1990 to 1.07 (95% UI 0.91 to 1.24) billion in 2021. YLD exhibited a parallel trend, increasing by 65.13%, from 33.72 (95% UI 20.05 to 52.56) million in 1990 to 55.69 (95% UI 33.80 to 85.13) million in 2021. The EAPCs were calculated at 0.1 (95% CI 0.07 to 0.14) for ASPR, -0.13 (95% CI -0.17 to -0.1) for age-standardized YLDs rate (Table 2, Table S2, Fig. 1). The global need for acupuncture services contributed by China was approximately 16.31%.

**Table 2:** Estimated prevalence and years of life lived with disability for health conditions in need of acupuncture in 1990 and 2021 in China

Disease type	Prevalence				Years of life lived with disability			
	1990		2021		1990		2021	
	Number	ASR, per 100,000 persons	Number	ASR, per 100,000 persons	Number	ASR, per 100,000 persons	Number	ASR, per 100,000 persons
**Total**	683,422,557.3 (578,018,993.61, 805,051,958.13)	60,832.79 (52,018.18, 70,854.31)	1,063,602,486 (910,225,098.83, 1,241,368,008.82)	62,596.45 (53,502.85, 72,999.92)	33,722,644.71 (20,053,136.29, 52,555,481.57)	3162.04 (1919.78, 4836.99)	55,685,183.44 (33,799,983.51, 85,125,647.63)	3089.74 (1849.63, 4760.45)
**Musculoskeletal disorders**	139,617,024.96 (117,206,824.12, 162,781,060.66)	14,190.24 (12,042.19, 16,373.01)	268,271,622.61 (225,845,643.82, 312,274,196.95)	13,409.29 (11,344.24, 15,489.32)	12,118,970.77 (8,114,432.62, 17,549,140.17)	1182.21 (788.41, 1710.54)	20,467,287.53 (13,312,777.64, 30,309,991.12)	1060.73 (698.38, 1543.71)
Neck pain	26,530,054.74 (20,347,966.07, 32,917,308.73)	2479.42 (1942.66, 3042.93)	48,377,403.9 (37,665,090.51, 60,063,295.51)	2549.87 (2007.89, 3141.64)	2,675,172.13 (1,740,002.21, 3,911,198.5)	248 (163.93, 353.44)	4,807,593.39 (3,155,339.53, 6,903,309.57)	254.77 (166.89, 357.93)
Low back pain	68,281,006.5 (59,158,022.43, 77,853,897.03)	6635.49 (5770.68, 7472.8)	100,093,745.61 (87,128,172.52, 113,014,315.76)	5342.1 (4660.41, 5976.28)	7,772,957.79 (5,520,145.03, 10,545,675.58)	749.03 (530.01, 1013.84)	11,297,804.86 (7,931,467.76, 15,328,056.41)	603.03 (427.63, 810.16)
Hip osteoarthritis	1,720,272.75 (1,317,266.12, 2,211,917.4)	202.34 (155.23, 259.25)	5,469,514.13 (4,187,810.74, 7,105,044.47)	260.1 (199.95, 335.08)	56,039.43 (25,701.03, 113,555.13)	6.52 (3, 13.05)	175,922.03 (80,877.37, 351,640.74)	8.35 (3.87, 16.62)
Knee osteoarthritis	41,044,009.16 (34,636,656.81, 47,406,915.14)	4667.29 (3996.06, 5359.85)	109,575,472.44 (92,723,350.57, 126,639,048.86)	5016.52 (4265.22, 5758.38)	1,339,566.29 (644,355.7, 2,583,887.25)	151.24 (72.96, 291.47)	3,554,153.43 (1,715,777.15, 6,842,993.79)	162.44 (78.35, 314.13)
Rheumatoid arthritis	2,041,681.8 (1,746,912.69, 2,391,022.36)	205.7 (177.56, 238.18)	4,755,486.53 (4,141,219.49, 5,452,492.35)	240.7 (210.77, 277.95)	275,235.12 (184,228.65, 394,823.72)	27.42 (18.52, 38.74)	631,813.82 (429,315.83, 883,990.62)	32.14 (21.65, 44.86)
**Neurological disorders**	352,946,298.35 (305,093,981.41, 404,003,047.79)	30,085.34 (26,281.79, 34,147.53)	516,969,717.98 (455,196,399.93, 588,413,609.16)	32,750.55 (28,698.25, 37,235.46)	8,493,828.56 (3,004,504.59, 16,813,996.4)	842.46 (349.29, 1556.23)	16,983,022.88 (7,882,764.7, 29,561,678.3)	960.42 (411.86, 1732.52)
Tension-type headache	204,064,313.18 (176,898,604.49, 233,568,232.22)	17,174.48 (15,086.74, 19,379.66)	283,814,151.04 (251,438,661.81, 320,431,556.78)	18,525.07 (16,380.87, 20,958.7)	489,462.66 (151,736.85, 1,687,075.44)	41.79 (13.09, 141.41)	716,164.94 (224,403.14, 2,174,717.29)	43.5 (13.07, 141.34)
Migraine	133,474,536.55 (114,199,443.66, 153,482,597.68)	10,948.52 (9428.76, 12,586.13)	184,752,280.11 (160,836,524.72, 213,633,958.29)	11,777.51 (10,137.56, 13,538.56)	5,028,787.46 (767,668.49, 11,262,271.45)	412.97 (66.16, 911.02)	6,988,198.6 (1,133,318.67, 15,186,289.26)	443.65 (66.93, 971.68)
Stroke	10,731,080.12 (10,003,053.91, 11,542,610.14)	1167.42 (1082.04, 1262.59)	26,335,402.63 (24,154,963.05, 28,625,610.86)	1301.42 (1200.61, 1405.73)	2,072,204.4 (1,474,566.11, 2,652,676.54)	228.81 (161.78, 292.76)	5,089,856.76 (3,624,862.57, 6,591,427.31)	252.64 (179.42, 325.87)
Alzheimer's disease and other dementias	4,024,535.83 (3,446,397.5, 4,623,086.05)	703.14 (608.36, 809.51)	16,990,827.32 (14,488,494.04, 19,672,741.19)	900.82 (770.92, 1043.22)	808,456.04 (545,684.24, 1,082,807.54)	145.78 (99.22, 193.23)	3,460,323.73 (2,394,267.39, 4,632,167.12)	185.63 (127.98, 246.72)
Parkinson's disease	651,832.67 (546,481.86, 786,521.7)	91.77 (75.88, 109.65)	5,077,056.87 (4,277,756.31, 6,049,742.04)	245.73 (208.28, 289.24)	94,918 (64,848.91, 129,165.43)	13.11 (9.05, 17.82)	728,478.85 (505,912.92, 977,077.32)	34.99 (24.45, 46.9)
**Digestive disorders**	414,107.96 (290,877.31, 583,206.63)	40.92 (28.5, 57.69)	656,204.84 (485,557.15, 894,464.15)	33.31 (25.13, 44.3)	37,615.91 (21,641.04, 60,024.12)	3.63 (2.08, 5.7)	63,436.17 (37,448.53, 98,935.84)	3.32 (1.98, 5.13)
Upper digestive system diseases	61,664,961.52 (54,580,738.54, 68,957,129.71)	5652.87 (5052.39, 6273.02)	94,895,509.22 (83,448,886.5, 105,877,276.91)	5239.87 (4629.88, 5860.38)	922,965.8 (583,843.98, 1,416,626.24)	87.69 (55.02, 133.16)	1,238,810.67 (784,848.01, 1,929,115.64)	66.68 (42.13, 102.4)
Inflammatory bowel disease	62,097.93 (52,445.94, 75,050.25)	5.59 (4.73, 6.69)	168,076.67 (141,521.15, 201,684.43)	9.16 (7.8, 11.01)	10,149.81 (6667.52, 14,629.82)	0.91 (0.6, 1.3)	26,930.14 (17,903.7, 38,405.99)	1.47 (0.97, 2.09)
Pancreatitis	352,010.03 (238,431.37, 508,156.38)	35.33 (23.78, 50.99)	488,128.17 (344,036, 692,779.72)	24.15 (17.33, 33.29)	27,466.1 (14,973.51, 45,394.3)	2.71 (1.48, 4.39)	36,506.02 (19,544.83, 60,529.85)	1.85 (1.01, 3.03)
**Genecological disorders**	35,052,907.86 (18,992,529.84, 60,585,847.9)	2699.44 (1419.55, 4705.03)	41,162,804.58 (21,057,893.42, 72,854,862.89)	2825.15 (1491.93, 4888.21)	184,630.87 (61,865.12, 467,889.99)	14.17 (4.77, 36.23)	217,182.47 (72,331.35, 552,160.87)	14.99 (5.15, 37.36)
Male infertility	10,256,626.69 (5,659,915.5, 17,534,174.17)	786.86 (432.8, 1348.1)	11,845,804.49 (6,488,726.4, 20,756,170.65)	824.98 (459.59, 1401.32)	55,154.62 (18,884.11, 135,265.76)	4.21 (1.46, 10.37)	63,930.85 (21,751.83, 155,613.8)	4.49 (1.54, 10.92)
Female infertility	24,796,281.17 (13,332,614.34, 43,051,673.73)	1912.58 (986.75, 3356.93)	29,317,000.09 (14,569,167.02, 52,098,692.24)	2000.17 (1032.34, 3486.89)	129,476.24 (42,981.01, 332,624.22)	9.96 (3.32, 25.87)	153,251.63 (50,579.52, 396,547.08)	10.5 (3.61, 26.43)
**Mental disorders**	74,975,662.7 (65,922,275.55, 85,440,302.71)	6484.14 (5742.32, 7321.32)	106,247,043.19 (92,979,858.04, 120,978,859.08)	6357.42 (5566.16, 7247.9)	10,322,611.61 (7,151,861.12, 14,073,364.99)	882.56 (617.46, 1199.41)	14,180,392.18 (9,928,183.05, 19,263,451.09)	849.55 (596.76, 1159.38)
Depressive disorders	34,479,389.55 (31,145,101.72, 38,431,728.11)	3071.84 (2779.1, 3404.49)	53,114,656.12 (47,435,175.6, 59,334,612.53)	2875.68 (2589.96, 3203.43)	5,426,668.66 (3,756,628.73, 7,330,296.01)	473.32 (331.33, 639.58)	7,865,942.53 (5,560,590.87, 10,696,832.64)	430.61 (305.24, 586.21)
Anxiety disorders	40,496,273.15 (34,777,173.83, 47,008,574.6)	3412.3 (2963.21, 3916.83)	53,132,387.07 (45,544,682.45, 61,644,246.55)	3481.74 (2976.2, 4044.48)	4,895,942.94 (3,395,232.39, 6,743,068.98)	409.25 (286.13, 559.83)	6,314,449.65 (4,367,592.18, 8,566,618.45)	418.95 (291.52, 573.17)
**Substance use disorders**								
Opioid use disorders	2,397,758.42 (2,019,080.63, 2,833,937.29)	192.51 (164.43, 223.98)	1,472,267.66 (1,222,899.67, 1,734,651.87)	94.35 (77.16, 112.47)	1,007,172.62 (671,830.95, 1,332,324.67)	80.25 (54.19, 104.75)	611,735.75 (421,460.08, 799,545.88)	39.55 (26.69, 51.84)
**Infectious disorders**								
Varicella and herpes zoster	754,741.95 (635,860.09, 893,850.99)	72.27 (61.17, 85.11)	1,116,219.49 (905,616.84, 1,360,148.98)	72.03 (60.92, 84.93)	31,079.25 (17,909.73, 48,268.94)	3 (1.75, 4.64)	53,600.41 (30,114.79, 82,722.83)	3 (1.74, 4.67)
**Neoplasms**	15,599,093.58 (13,276,826.11, 18,973,574.44)	1415.07 (1225.85, 1667.63)	32,811,096.43 (29,082,343.45, 36,979,938.83)	1814.49 (1609.18, 2036.94)	603,769.33 (425,247.14, 793,846.05)	66.07 (46.8, 86.31)	1,869,715.4 (1,330,055.37, 2,528,046.04)	91.5 (64.96, 123.45)

ASR: age-standardized rates

Compared with the global trend, China showed a faster increase in prevalence (EAPC = 0.10 vs. 0.02) but a more favorable decline in YLDs (EAPC = − 0.13 vs. − 0.04).

### Acupuncture needs across eight categories

Globally in 2021, across the eight categories, neurological disorders accounted for the highest number of prevalent cases, affecting approximately 3.33 billion individuals (51.19%), followed by musculoskeletal disorders (1.26 billion, 19.38%) and mental disorders (691 million, 10.83%). Gynecological disorders, substance use disorders, neoplasms, and infectious disorders accounted for increased trends in ASPRs in the past 30 years, with EAPC values of 0.64, 0.5, 0.25 and 0.05, respectively. In addition, musculoskeletal disorders accounted for the highest number of YLDs, contributing 106.09 million (34.62%), followed by mental disorders (98.84 million, 32.26%) and neurological disorders (76.44 million, 24.95%). Gynecological disorders, substance use disorders, neoplasms, and infectious disorders represented increased trends in age-standardized YLD rates in the past 30 years, with EAPC values of 0.65, 0.49, 0.17 and 0.06, respectively (Table 1, Table S1, Figures S1-S8).

Across the eight categories in China, neurological disorders accounted for the highest number of prevalent cases, accounting for 48.51% of all cases (516 million individuals), followed by musculoskeletal disorders (25.20%, 268 million), and mental disorders (9.97%, 106 million). Neoplasms and neurological disorders accounted for increased trends in ASPRs in the past 30 years, with EAPC values of 0.88 and 0.3, respectively. In addition, musculoskeletal disorders accounted for the highest number of YLDs, contributing to 36.74% (20.47 million), followed by neurological disorders (30.49%, 16.98 million), and mental disorders (25.56%, 14.18 million). Neoplasms and neurological disorders represented increased trends in age-standardized YLD rates in the past 30 years, with EAPC values of 1.16 and 0.38, respectively (Table 2, Table S2, Figures S1-S8).

Compared with the global trend, China exhibited a broadly similar distribution pattern of eight categories, with neurological, musculoskeletal, and mental disorders ranking as the top three contributors to both prevalence and YLDs. In terms of temporal trends, China showed higher increases in both ASPRs and age-standardized YLD rates for neoplasms (EAPC = 0.88 vs. 0.25; 1.16 vs. 0.17) and neurological disorders (EAPC = 0.30 vs. 0.05; 0.38 vs. 0.06), indicating more pronounced growth in the burden of these conditions over the past three decades.

### Acupuncture needs across 20 health conditions

Globally, for 20 health conditions, the top five conditions with the highest need for acupuncture included TTH, migraine, UDSD, LBP, and KOA, accounting for 77.47% of all prevalent cases (Table 1). TTH caused the highest burden, with nearly 2.01 billion prevalent cases and 4.60 million YLDs. Notably, TTH was consistently a leading contributor to the prevalence of 20 health conditions worldwide between 1990 and 2021 (Table 1). According to the EAPC values, the top five conditions with the highest increasing trends in ASPRs and age-standardized YLD rates over the past 30 years were PD, female infertility, RA, opioid use disorders, and male infertility (Table 1, Table S1, Figures S9-27).

In China, of the 20 health conditions, the top five conditions with the highest need for acupuncture were TTH, migraine, LBP, KOA, and UDSD, accounting for 72.69% of all prevalent cases (Table 2). TTH caused the highest burden, with 283.81 million prevalent cases and 0.72 million YLDs. Notably, TTH was consistently a leading contributor to the prevalence of 20 health conditions worldwide between 1990 and 2021 (Table 2). According to the EAPC values, the top five conditions with the highest increasing trends in ASPRs and age-standardized YLD rates over the past 30 years were PD, IBD, HOA, RA, and KOA (Tables 2 and S2, Figures S9-27).

The distribution of the 20 conditions in China was similar to global trends. Temporal trends differed, with PD, IBD, HOA, RA, and KOA showing the most rapid increases in China. The fastest-growing conditions were PD, female infertility, RA, opioid use disorders, and male infertility.

### Acupuncture needs in the two sexes

Worldwide and in China, significant sex differences are evident in acupuncture needs, with females consistently exhibiting a higher prevalence and greater YLDs. However, when assessing trends over time, males demonstrated a more pronounced increase in both the number of prevalent cases and YLDs, surpassing the growth rate observed in females globally and in China (Fig. 2, Tables S3-58, Figures S28-83).

**Fig. 2:**
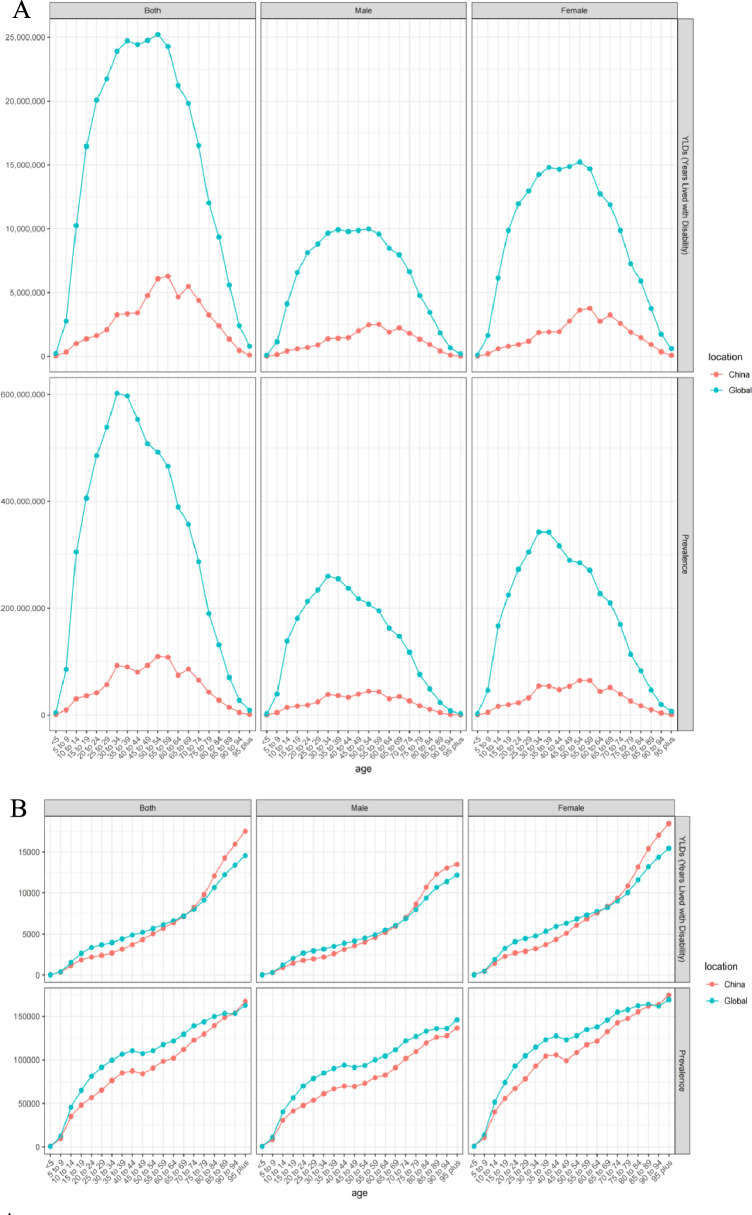
**A** The acupuncture need based on the number of prevalent cases and years lived with disability in different ages in 2021; **B** The acupuncture need based on age-standardized rates of prevalence and years lived with disability in different ages in 2021

Across the eight disease categories, females also consistently exhibited a higher prevalence of cases and YLDs of acupuncture needs compared to males, with the exception of digestive disorders and substance use disorders worldwide, which consistently exhibited a higher prevalence of cases and YLDs of acupuncture needs compared to males, with the exception of digestive disorders and neoplasms in China. In the trend of prevalent cases and YLDs of acupuncture needs, females showed more notable upward trends for gynecological disorders, whereas for neurological disorders and neoplasms, males had more significant increasing trends of prevalent cases and YLDs worldwide and in China.

Across the 20 health conditions for prevalent cases, females consistently exhibited a higher prevalence of cases and YLDs of acupuncture needs than males, with the exception of opioid use disorders worldwide, which consistently exhibited a higher prevalence of cases and YLDs of acupuncture needs than males, except for PD, pancreatitis, and HOA in China. Regarding the trend in acupuncture needs, females exhibited more notable upward trends for KOA, whereas for RA, migraine, and PD, males exhibited more significant increasing trends in prevalent cases and YLDs worldwide and in China.

### Acupuncture needs in different age groups

The need for acupuncture varied across the age groups of prevalent cases and YLDs worldwide and in China (Fig. 2, Tables S3-58, Figures S84-110). Individuals 15 to 64 years of age were the main contributors to acupuncture. As individuals age, the need for acupuncture has gradually shifted, both worldwide and in China. Furthermore, across the eight disease categories, the main contributors to acupuncture needs were mental and infectious disorders in children < 15 years old; neurological, digestive, musculoskeletal, gynecological, and substance use disorders in adults 15 to 29 years old; neurological, digestive, musculoskeletal, mental, and gynecological disorders in adults 30 to 44 years old; neurological, musculoskeletal, mental, digestive disorders, and neoplasms in adults 45 to 64 years old; and neoplasms in older individuals. Among the 20 health conditions analyzed, the top three contributors to the need for acupuncture were TTH, migraine, and UDSD in adults aged 15 to 29, 30 to 44, and 45 to 64 years; AD, PD, and stroke in older adults; and depressive disorders, anxiety disorders, varicella, and herpes zoster in children under 15 years of age.

### Factors contributing to acupuncture needs

In China and globally, the need for acupuncture has increased from 1990 to 2021. Although population growth is the main driver worldwide, population aging also plays a prominent role in China. Population growth, aging, and epidemiological changes contributed positively to the prevalence and YLD. In addition, both sexes exhibited the greatest increases in prevalence and YLDs, driven primarily by population growth and aging (Fig. 3). Across the eight categories and 20 health conditions, the findings highlighted that population growth was the primary contributing factor to the prevalence and increase in YLDs (Figures S111-S136).

**Fig. 3:**
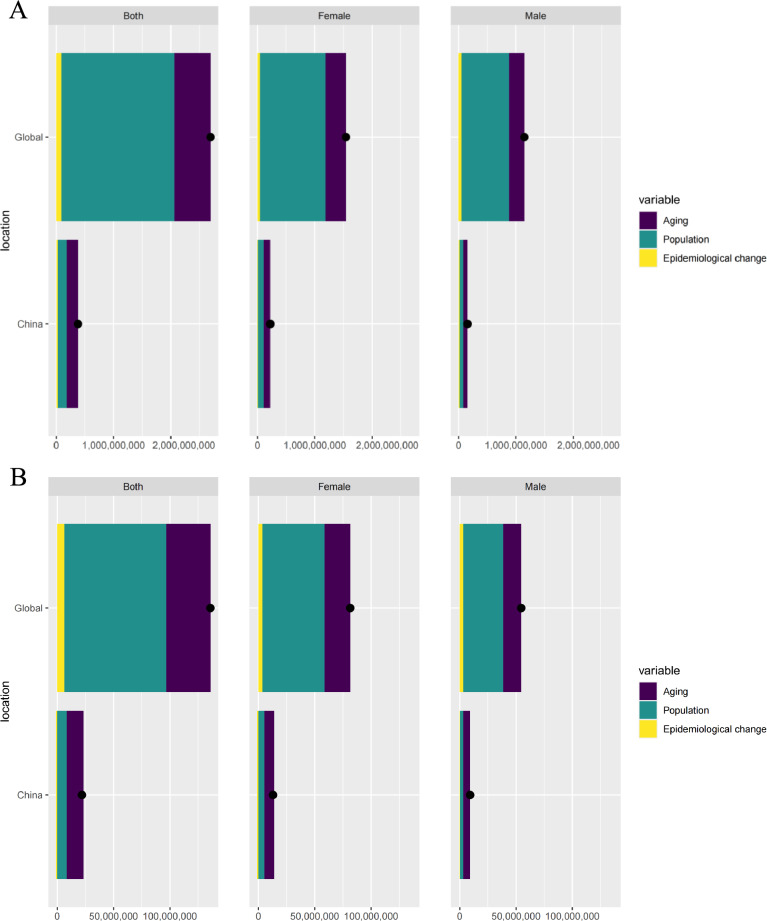
**A** Decomposition analysis of acupuncture need change based on prevalence from 1990 to 2021; **B** Decomposition analysis of acupuncture need change based on years lived with disability from 1990 to 2021. *Notes*: Black dots represent the total change contributed by all three components. A positive value for each component indicates a corresponding positive contribution, and a negative value indicates a corresponding negative contribution

### Forecasting future need for acupuncture

**Fig. 4:**
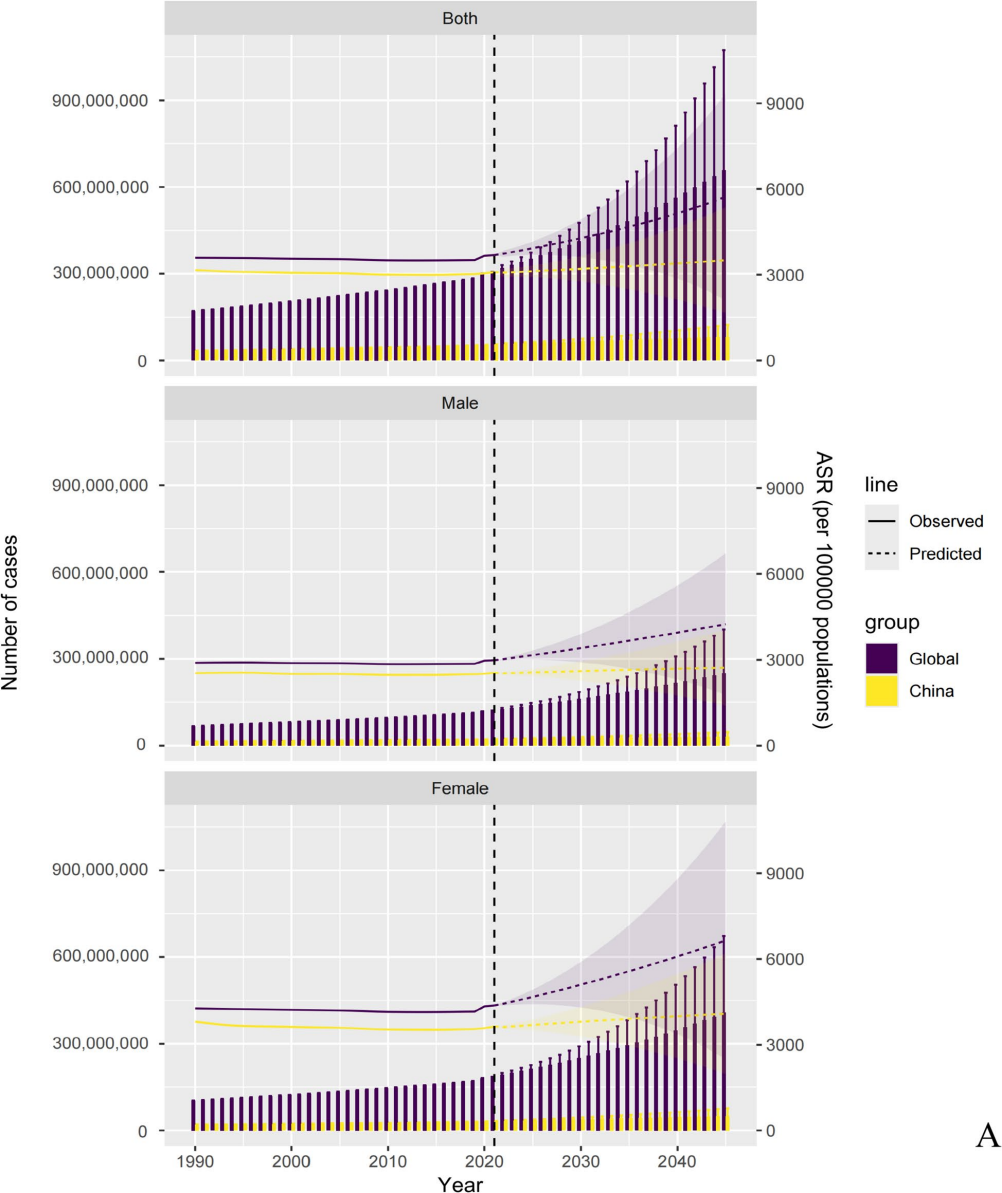

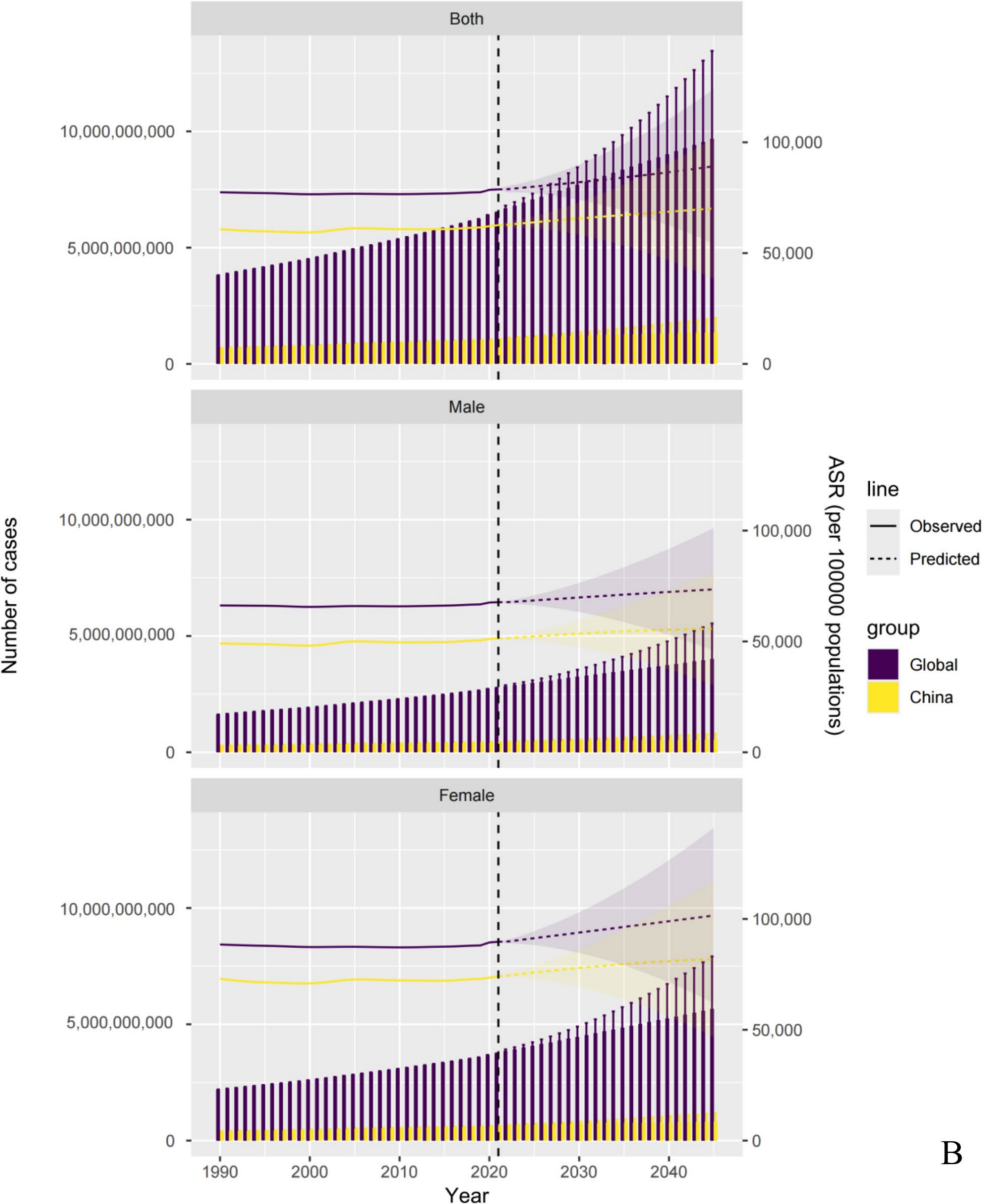
**A** Future forecasts of Global Burden of Disease in acupuncture need based on prevalence using bayesian age-period-cohort model; **B** Future forecasts of Global Burden of Disease in acupuncture need based on years lived with disability using bayesian age-period-cohort model. *Notes*: The line graphs show the change of age-standardized rates (ASR), and the bar graphs show the change of burden.

Global and Chinese trends in the need for acupuncture from 1990 to 2045 are illustrated in Fig. 4, Tables S59-114, and Figures S137-S163. The plots reveal a marked upward trend in the number of individuals affected by the prevalence and YLDs, along with steadily increasing age-standardized rates, both globally and in China. Globally, the number of prevalent cases is projected to reach 9.68 (95% UI 5.90 to 13.46) billion and YLDs are expected to rise to 0.66 (95% UI 0.24 to 1.07) billion by 2045. In China, prevalent cases are expected to reach 1.35 (95% UI 0.74 to 1.96) billion and YLDs to 80.76 (95% UI 38.33 to 123.18) million. Across both global and Chinese populations, females also consistently exhibited higher numbers and rates of prevalence and YLDs related to acupuncture needs than those for males. The need for acupuncture is expected to increase significantly for neurological disorders, musculoskeletal disorders, mental disorders, and neoplasms. Notably, TTH is projected to remain the leading contributor to the prevalence of cases among the 20 health conditions analyzed.

## Discussion

This study offers the first comprehensive population-level assessment of the global and national potential need for acupuncture, revealing a marked and accelerating rise in the burden of conditions with therapeutic indications for acupuncture, particularly driven by neurological and musculoskeletal disorders that are predominantly chronic and age-related. These trends underscore the growing alignment of acupuncture with global public health priorities, especially in aging societies that are increasingly burdened by long-term, degenerative, and function-limiting conditions. The findings not only provide empirical support for integrating acupuncture into evidence-based, person-centered care models, but also affirm the strategic value of acupuncture in contributing to the development of inclusive, sustainable, and globally relevant health systems.

### Principal findings

This study systematically detailed the potential need for acupuncture, both globally and in China. The total acupuncture needs increased significantly from 1990 to 2021 in terms of prevalent cases and YLDs worldwide and in China. In 2021, the global prevalence of acupuncture and YLDs increased by 70.65% and 79.83%, respectively, whereas in China, the corresponding increases are 55.63% and 65.13%, respectively. The need for acupuncture is strikingly high, and continues to increase worldwide, particularly in China. This growing need is largely driven by population growth and aging both globally and in China. The majority of acupuncture needs, both globally and in China, are attributable to females and those aged 15 to 64 years. Moreover, the need for acupuncture is projected to continue to rise over the next 24 years, reflecting a persistent and significant health need in both global and Chinese contexts. Moreover, from 2021 to 2045, the greatest contributors to the need for acupuncture were neurological disorders across the eight categories and TTH in neurological disorders among the 20 health conditions. These findings provide key insights into the needs of acupuncture both globally and in China, highlighting the urgency of expanding acupuncture services and developing relevant policies.

### Acupuncture needs in categories and health conditions

Between 1990 and 2021, neurological disorders were consistently the leading contributors to acupuncture needs both globally and in China, a trend projected to continue through 2045. Compared with the global trend, China showed higher increases in both ASPRs and age-standardized YLD rates, indicating an urgent need for strengthened acupuncture strategies. In addition, TTH and migraine consistently ranked first and second among the 20 health conditions [45, 46], reflecting their high prevalence, disabling nature, and substantial number of YLDs. Acupuncture, proven effective for TTH [8, 36] and migraine [9, 10], offers significant pain relief, reduced attack frequency, and improved function, supporting its role as a key non-pharmacological strategy in long-term management. Moreover, stroke, ADOD, and PD impose a disproportionate YLD burden due to high disability weights despite their relatively low prevalence, underscoring the need to address disability as a priority within acupuncture care. Notably, PD showed the fastest increase in prevalence and YLDs, with the highest EAPC among the 20 conditions, likely linked to aging, environmental factors, and lifestyle risks [47]. As the second most common neurodegenerative disorder, PD may benefit from integrated acupuncture and conventional care, which can alleviate motor and non-motor symptoms through neurochemical and neuroimmune pathways, reduce medication reliance, and improve mental health and quality of life [7, 39, 40].

From 1990 to 2021, musculoskeletal disorders consistently ranked as the second leading contributor to acupuncture needs globally. In China, this trend is expected to persist through 2045. LBP, KOA, and NP accounted for the majority of the cases. LBP ranks fourth globally, and third among acupuncture needs in China. Numerous studies have identified a range of factors that contribute to chronic and disabling LBP, including physical dysfunction, psychological influences, and social determinants [50]. KOA is a major cause of activity limitation and work absenteeism worldwide, with onset and progression linked to obesity, prior injury, muscle weakness, and joint instability, imposing substantial economic burdens [51]. NP, the fourth-leading cause of global disability [52], arises from a multifactorial interplay of muscle strain, inactivity, and inflammatory or degenerative changes in spinal and neural structures [53]. In addition, RA, as a chronic autoimmune inflammatory disease associated with disability and premature death, has shown a rising trend in prevalence and YLD rates [54]. Collectively, these musculoskeletal conditions impose substantial and growing personal, societal, and healthcare burdens. Although often progressive and irreversible, targeted acupuncture can relieve pain, address modifiable risk factors, and improve quality of life [37].

Although digestive and mental disorders have imposed an increasing overall burden, both have exhibited a declining trend in prevalence and YLDs rates both globally and in China, suggesting that these conditions have been increasingly brought under effective control. This trend may reflect the combined effects of improved disease prevention, early detection, and expanded access to healthcare services. In China, national initiatives such as the Healthy China 2030 Strategy and the Traditional Chinese Medicine Development Strategy Plan (2016–2030) have played important roles in enhancing screening coverage, optimizing management pathways, and promoting health literacy. Improvements in primary care capacity along with the integration of traditional Chinese medicine into gastrointestinal and psychological care have further contributed to a reduction in the overall disability burden of these conditions. Acupuncture has been shown to benefit the management of a range of symptoms associated with digestive and mental health conditions [1, 12]. In digestive disorders, acupuncture has demonstrated efficacy in relieving abdominal pain, regulating gastrointestinal motility, and improving overall digestive function. In the context of mental disorders, such as anxiety, depression, and insomnia, acupuncture has been found to modulate neuroendocrine function and promote emotional regulation, contributing to improved psychological well-being. Future efforts should focus on strengthening acupuncture interventions to sustain and enhance this positive trend, with the aim of further reducing both the prevalence of and YLDs associated with digestive and mental disorders.

Neoplasms have shown an increasing trend in prevalence and YLDs rates both globally and in China, which is potentially driven by the degree of economic development and associated social and lifestyle factors. Currently, acupuncture has demonstrated promising effects in alleviating cancer-related pain, fatigue, and insomnia, as well as in improving overall quality of life for patients. It has also shown therapeutic potential for the management of nausea and vomiting, bone marrow suppression, menopausal symptoms, arthralgia, and dysphagia. Moreover, emerging evidence supports its possible benefits in the treatment of lymphedema, gastrointestinal dysfunction, and xerostomia [1, 12]. Compared with the global trend, China has shown higher increases in both ASPRs and age-standardized YLD rates, underscoring the urgency of promoting evidence-based research and policy initiatives to strengthen the role of acupuncture in oncology rehabilitation. Future efforts should focus on expanding the spectrum of cancer-related conditions identified as amenable to acupuncture, elucidating the underlying biological mechanism(s), and reinforcing evidence through rigorous large-scale clinical trials. Such initiatives are critical to establish acupuncture as a scientifically grounded and integral component of multidisciplinary cancer care.

Infertility, opioid use disorders, and herpes zoster have increased globally, but China has seen a decline in their prevalence and YLDs, likely due to national initiatives, primary care capacity, improved care access, and integration of acupuncture [55, 56]. In particular, acupuncture has been increasingly used to manage symptoms associated with gynecological disorders, reduce dependency on individuals with opioid use disorders, and relieve neuralgia related to herpes zoster [1, 12]. This intervention has contributed to better clinical outcomes and reduced the overall disease burden. Therefore, it is essential to promote the application of acupuncture in the management of a broader range of symptoms associated with gynecological disorders, opioid use disorders, and herpes zoster in China. Internationally, healthcare systems may benefit from drawing on China’s experience by exploring the integration of evidence-based traditional therapies such as acupuncture into conventional care pathways.

### Factors influencing acupuncture needs

In our study, we observed that worldwide and in China, females and males presented different characteristics regarding the need for acupuncture services. Across the eight disease categories, females also consistently exhibited higher acupuncture needs than males, except for digestive disorders. Among the 20 health conditions, females had higher acupuncture needs than males for TTH, migraine, UDSD, LBP, and KOA, except for opioid use disorders, PD, pancreatitis, and HOA. These sex differences in acupuncture needs may be partially attributed to physiological characteristics, hormonal fluctuations, occupational exposure, and mechanical load disparities [57]. Moreover, when examining trends in acupuncture needs, males exhibited more notable upward trends than females, both globally and in China. Thus, although females currently account for higher acupuncture needs for several conditions, the faster increase observed among males suggests a shifting pattern in health-seeking behaviors or disease burden, underscoring the importance of designing acupuncture service models to ensure that both men and women can access timely, appropriate, and effective acupuncture based on their specific needs.

From 1990 to 2021, a substantial increase in the number of prevalent cases and YLDs associated with acupuncture needs was observed among individuals aged 15 to 64 years, indicating an increasing burden of pain-related neurological conditions and digestive disorders (such as TTH, migraines, and UDSD) in the working-age population. This trend may be attributed to occupational stress, sedentary behavior, and lifestyle-related risk factors [58–60]. Among older adults, the need for acupuncture is predominantly driven by age-associated neurodegenerative disorders, including ADOD and PD, highlighting the potential role of acupuncture in geriatric care and functional rehabilitation. In contrast, children aged < 15 years of age demonstrated a distinct profile of mental disorders and infectious diseases, particularly depressive disorders, anxiety, and varicella/herpes zoster, which are the primary contributors to acupuncture use. These findings underscore the critical importance of age as a determinant of acupuncture needs, and suggest that integrating age-specific considerations into acupuncture service planning and policy frameworks may enhance the precision and effectiveness of traditional medical interventions across populations.

We found that increases in population growth and aging have collectively driven a significant increase in the total prevalence and YLDs, underscoring the escalating need for acupuncture services both globally and in China from 1990 to 2021. The impact of these demographic forces varies across development contexts [61]. In many developing countries, population growth is the primary driver, increasing burdens from infectious diseases, maternal and child health issues, and injuries, which often require scalable and equitable primary care solutions. Integrating acupuncture into expanding health service networks may improve accessibility and cost-effectiveness. In contrast, in aging societies such as China, the rising proportion of older adults drives the prevalence of non-communicable diseases, multimorbidity, and disability. Health systems must transition toward long-term, integrated care, where acupuncture can serve as a non-pharmacologic intervention to reduce polypharmacy and disability burden for aging population. Policy responses should therefore be stage-specific: focusing on workforce training and service decentralization where population growth predominates, and prioritizing geriatric-specific protocols, early disability screening, and community-based acupuncture programs where ageing is dominant. Such measures should be accompanied by regular monitoring of the ageing population, population management strategies, and the establishment of early warning systems, with acupuncture-based interventions implemented to slow disability progression and improve the quality of life of the elderly, particularly in China.

### Forecasting acupuncture need and strategic expansion of service provision

This study underscores a pronounced upward trajectory in acupuncture needs projected through 2045, highlighting the persistent and considerable challenges encountered globally and in China. This requires the development of systematic and integrated strategies to meet future needs [1].

First, the global development of acupuncture has been driven by international strategies, strong government policies, and broad societal engagement. Guided by the WHO’s Traditional and Complementary Medicine Development Strategy (2014–2023) [26], efforts have focused on enhancing its safety, effectiveness, and relevance, thereby integrating acupuncture into health systems as a safe, cost-effective, and culturally accepted therapy. In China, sustained investments in research, education, and regulation, along with physician recognition and continuing education, have strengthened professional capacity. However, the disease-based departmental structure of conventional medicine conflicts with acupuncture’s treatment-based approach, restricting its integration and clinical use [62, 63] To effectively address this pressing priority, it is essential to establish disease-oriented acupuncture subspecialties—such as Acupuncture for Neurological Conditions, Acupuncture for Musculoskeletal Management, and Acupuncture for Digestive Disorders and the like. Furthermore, with the rising need in an aging population, expanding the acupuncture workforce (such as integrating acupuncture modules into continuing medical education for general practitioners; establishing a tiered licensing system that enables acupuncturists with advanced training to specialize in fields like neurology, pain medicine, and geriatrics; establishing multidisciplinary “disease-oriented acupuncture subspecialties” to align with major chronic disease management pathways) is imperative. Priorities include establishing specialized training centers, increasing enrollment in related academic programs, and fostering international and cross-disciplinary collaboration supported by adequate funding and policy initiatives [64]. Moreover, to optimize acupuncture delivery, it is essential to embed such services within all levels of the healthcare system, ranging from primary to tertiary care. Integrating acupuncture into primary care allows for timely interventions that substantially slow disease progression, reduce disability, prevent complications, improve quality of life, and reduce medication overuse [65, 66]. Furthermore, supportive technologies such as need-forecasting models, clinical databases, and tele-acupuncture services will help meet growing needs [57, 67]. Finally, based on high-quality clinical evidence, this study included officially recognized 20 health conditions. However, this represents only a small proportion of the 377 health conditions currently reported to be suitable for acupuncture interventions [68]. Therefore, there is a pressing need for high-quality evidence-based clinical research to expand the officially recognized spectrum of acupuncture.

### Strengths and limitations

To our knowledge, this study represents the first and most recent and comprehensive secondary analysis to assess the need for acupuncture worldwide and in China from 1990 to 2021 and to project future needs from 2022 to 2045 based on data from the 2021 GBD study. Unlike previous investigations that primarily examined acupuncture utilization or need within single diseases (e.g., KOA, LBP, migraine) or within specific countries (e.g., Japan, Korea, or Western populations), this study established an integrated global–China and multi-disease analytical framework that enhances the generalizability of findings. This study revealed both shared and distinctive patterns of acupuncture needs between Chinese and global averages. Females and middle-aged to older adults showed a higher prevalence of YLDs, with neurological and musculoskeletal disorders as the main contributors. In China, overall need grew faster, largely driven by population aging and increasing cases among the 45 to 64 and ≥ 65-year-old groups, which carry the highest burden of chronic, neurological, and neoplastic conditions. Male needs increased more steeply than female needs, narrowing the gender gap and likely reflecting the relative growth of the male population in key age groups and the rising prevalence of acupuncture-responsive conditions among men. The pronounced rise in neurological and neoplastic disorders likely reflects aging, a higher prevalence and improved diagnosis, whereas the slower growth in mental disorder–related needs align with lower epidemiological growth, limited integration of psychological care in traditional medicine, and sociocultural barriers to diagnosis. Collectively, these findings provide robust evidence on global acupuncture needs, reveal China-specific trends and drivers, and demonstrate the value of a comprehensive global–China and multi-disease framework, offering a foundation for future research, resource allocation, and integrative healthcare policies.

However, this study had several limitations. First, the analysis equated disease prevalence and YLDs with the potential need for acupuncture, which represents a theoretical rather than an actual service need. This estimation did not consider factors such as the real-world availability of acupuncture services, cultural acceptance, or insurance coverage, all of which may substantially influence utilization. Therefore, the results should be interpreted as reflecting the maximum potentially addressable burden rather than the effective need for acupuncture. Future research incorporating service utilization or accessibility data would help to refine these estimates. Second, due to the inherent characteristics of the GBD data, the present analysis did not account for contraindications or individual factors that may limit the applicability of acupuncture. Certain populations such as those with bleeding disorders, severe psychiatric disorders, or acute infections are not suitable for acupuncture. It must be acknowledged that this omission may have slightly overestimated the population eligible for acupuncture treatment. In addition, by assuming that co-occurring diseases were independently distributed within the population, our study may have overestimated the true prevalence. Finally, although the BAPC model currently represents one of the most suitable approaches for projecting future trends, the cross-sectional nature of GBD data and the inherent limitations of the model restrict its ability to capture long-term or dynamic changes. The projections were based solely on current patterns, without accounting for future shifts in risk factors, therapeutic advances, or healthcare policies, nor did they consider the potential impacts of unforeseen events such as pandemics, wars, or climate change on the disease burden, which could substantially alter future trajectories. This omission may reduce the accuracy of the long-term forecasts.

Importantly, the present analysis likely underestimated the true global need for acupuncture. First, the GBD database lacks data regarding many conditions for which acupuncture is commonly used, such as insomnia, constipation, chronic fatigue syndrome, fibromyalgia, allergic rhinitis, irritable bowel syndrome, and stress urinary incontinence, among others [1, 12, 35]. As a result, these conditions were not included in the analysis, further contributing to the potential underestimation. Secondly, while 20 major health conditions were included based on WHO benchmarks, high-quality reviews, meta-analyses, and randomized controlled trials, acupuncture is widely applied to over 377 health conditions worldwide [68]. Given this extensive spectrum, the population potentially benefiting from acupuncture is likely to be much larger than the current estimates. However, many of these conditions lacked high-quality evidence, which was why they were not included in the analysis. Future research should first generate higher-quality clinical evidence to support the inclusion of a broader range of conditions and incorporate additional international data, which could, in turn, enable frameworks such as the GBD to better capture acupuncture-relevant disorders and provide a more comprehensive assessment of global acupuncture needs.

## Conclusions

In summary, this study demonstrated the substantial and increasing global and Chinese need for acupuncture, driven by population aging and growth, with a higher need among females and variation across age groups. The increasing burden of neurological disorders highlights the increasing therapeutic relevance of acupuncture. To address these trends, health authorities should actively integrate acupuncture into primary care and health planning, medical institutions should expand training and establish disease-specific clinics, and research institutions should focus on long-term effectiveness studies. Together, these actions can ensure population-specific needs are met and enhance acupuncture’s impact on public health.

## Supplementary Information

Additional file 1 (PDF 72738 KB)

## Data Availability

The data sets generated and/or analyzed during the current study are available in the GBD repository (https://vizhub.healthdata.org/gbd-results/). Data will be made available on request.

## Abbreviations

ADOD: Alzheimer’s disease and other dementias
ASPR: Age-specific prevalence rate
ASRs: Age-standardized rates
BAPC: Bayesian age-period-cohort
CIs: Confidence intervals
EAPC: Estimated annual percentage change
GBD: Global Burden of Disease
HOA: Hip osteoarthritis
IBD: Inflammatory bowel disease
KOA: Knee osteoarthritis
LBP: Low back pain
NP: Neck pain
PD: Parkinson’s disease
RA: Rheumatoid arthritis
TTH: Tension-type headache
UDSD: Upper digestive system diseases
UI: Uncertainty intervals
YLDs: Years lived with disability
