# Supplementary material for: Current trends and projections for potential acupuncture needs globally and in China: evidence from the Global Burden of Disease Study 2021
Source: Chin Med. 2026 Jan 7;21:12. doi: 10.1186/s13020-025-01286-9 (PMC12777211; doi:10.1186/s13020-025-01286-9)
Supplement: Supplementary file 1 — Additional file 1 (PDF 72738 KB) [file 13020_2025_1286_MOESM1_ESM.pdf]

Trends and Projections of Potential Acupuncture Needs in the worldwide and China: Evidence from the Global Burden of Disease Study 2021

Supplementary Materials and Methods*

Content List

Part I. Detailed definitions of 20 specific health conditions 1
Table S1. Estimated annual percentage changes of prevalence and years of life lived with disability for health conditions in need of acupuncture in 1990 and 2021 in the worldwide. 10
Table S2. Estimated annual percentage changes of prevalence and years of life lived with disability for health conditions in need of acupuncture in 1990 and 2021 in China. 11
Table S3. Acupuncture demands based on prevalence and YLDs and its temporal trends from 1990, 2021 by gender, and age in the worldwide. 12
Table S4. Prevalence and YLDs of musculoskeletal disorders and its temporal trends from 1990, 2021 by gender, and age in the worldwide. 14
Table S5 Prevalence and YLDs of neurological disorders and its temporal trends from 1990, 2021 by gender, and age in the worldwide. 16
Table S6. Prevalence and YLDs of digestive disorders and its temporal trends from 1990, 2021 by gender, and age in the worldwide. 18
Table S7. Prevalence and YLDs of genecological disorders and its temporal trends from 1990, 2021 by gender, and age in the worldwide. 20
Table S8. Prevalence and YLDs of mental disorders and its temporal trends from 1990, 2021 by gender, and age in the worldwide. 21
Table S9. Prevalence and YLDs of substance use disorders and its temporal trends from 1990, 2021 by gender, and age in the worldwide. 23
Table S10. Prevalence and YLDs of infectious disorders and its temporal trends from 1990, 2021 by gender, and age in the worldwide. 25
Table S11. Prevalence and YLDs of neoplasms and its temporal trends from 1990, 2021 by gender, and age in the worldwide. 27
Table S12. Prevalence and YLDs of neck pain and its temporal trends from 1990, 2021 by gender, and age in the worldwide. 29
Table S13. Prevalence and YLDs of low back pain and its temporal trends from 1990, 2021 by gender, and age in the worldwide. 31
Table S14. Prevalence and YLDs of hip osteoarthritis and its temporal trends from 1990, 2021 by gender, and age in the worldwide. 33
Table S15. Prevalence and YLDs of knee osteoarthritis and its temporal trends from 1990, 2021 by gender, and age in the worldwide. 35
Table S16. Prevalence and YLDs of rheumatoid arthritis and its temporal trends from 1990, 2021 by gender, and age in the worldwide. 37
Table S17. Prevalence and YLDs of tension-type headache and its temporal trends from 1990, 2021 by gender, and age in the worldwide. 39
Table S18. Prevalence and YLDs of migraine and its temporal trends from 1990, 2021 by gender, and age in the worldwide. 41
Table S19. Prevalence and YLDs of stroke and its temporal trends from 1990, 2021 by gender, and age in the worldwide. 43
Table S20. Prevalence and YLDs of Alzheimer's disease and other dementias and its temporal trends from 1990, 2021 by gender, and age in the worldwide. 45
Table S21. Prevalence and YLDs of Parkinson's disease and its temporal trends from 1990, 2021 by gender, and age in the worldwide. 47
Table S22. Prevalence and YLDs of upper digestive system diseases and its temporal trends from 1990,

2021 by gender, and age in the worldwide. 49
Table S23. Prevalence and YLDs of inflammatory bowel disease and its temporal trends from 1990, 2021 by gender, and age in the worldwide. 51
Table S24. Prevalence and YLDs of pancreatitis and its temporal trends from 1990, 2021 by gender, and age in the worldwide. 53
Table S25. Prevalence and YLDs of male infertility and its temporal trends from 1990, 2021 by gender, and age in the worldwide. 55
Table S26. Prevalence and YLDs of female infertility and its temporal trends from 1990, 2021 by gender, and age in the worldwide. 56
Table S27. Prevalence and YLDs of depressive disorders and its temporal trends from 1990, 2021 by gender, and age in the worldwide. 57
Table S28. Prevalence and YLDs of anxiety disorders and its temporal trends from 1990, 2021 by gender, and age in the worldwide. 59
Table S29. Prevalence and YLDs of opioid use disorders and its temporal trends from 1990, 2021 by gender, and age in the worldwide. 61
Table S30. Prevalence and YLDs of varicella and herpes zoster and its temporal trends from 1990, 2021 by gender, and age in the worldwide. 64
Table S31. Acupuncture demands based on prevalence and YLDs and its temporal trends from 1990, 2021 by gender, and age in China. 67
Table S32. Prevalence and YLDs of musculoskeletal disorders and its temporal trends from 1990, 2021 by gender, and age in China. 69
Table S33 Prevalence and YLDs of neurological disorders and its temporal trends from 1990, 2021 by gender, and age in China. 71
Table S34. Prevalence and YLDs of digestive disorders and its temporal trends from 1990, 2021 by gender, and age in China. 73
Table S35. Prevalence and YLDs of genecological disorders and its temporal trends from 1990, 2021 by gender, and age in China. 75
Table S36. Prevalence and YLDs of mental disorders and its temporal trends from 1990, 2021 by gender, and age in China. 77
Table S37. Prevalence and YLDs of substance use disorders and its temporal trends from 1990, 2021 by gender, and age in China. 79
Table S38. Prevalence and YLDs of infectious disorders and its temporal trends from 1990, 2021 by gender, and age in China. 81
Table S39. Prevalence and YLDs of neoplasms and its temporal trends from 1990, 2021 by gender, and age in China. 83
Table S40. Prevalence and YLDs of neck pain and its temporal trends from 1990, 2021 by gender, and age in China. 85
Table S41. Prevalence and YLDs of low back pain and its temporal trends from 1990, 2021 by gender, and age in China. 87
Table S42. Prevalence and YLDs of hip osteoarthritis and its temporal trends from 1990, 2021 by gender, and age in China. 89
Table S43. Prevalence and YLDs of knee osteoarthritis and its temporal trends from 1990, 2021 by gender, and age in China. 91
Table S44. Prevalence and YLDs of rheumatoid arthritis and its temporal trends from 1990, 2021 by gender, and age in China. 93
Table S45 Prevalence and YLDs of tension-type headache and its temporal trends from 1990, 2021 by gender, and age in China. 95
Table S46. Prevalence and YLDs of migraine and its temporal trends from 1990, 2021 by gender, and age in China. 97
Table S47. Prevalence and YLDs of stroke and its temporal trends from 1990, 2021 by gender, and age in China. 99
Table S48. Prevalence and YLDs of Alzheimer's disease and other dementias and its temporal trends from 1990, 2021 by gender, and age in China. 101
Table S49. Prevalence and YLDs of Parkinson's disease and its temporal trends from 1990, 2021 by

gender, and age in China. 103
Table S50. Prevalence and YLDs of upper digestive system diseases and its temporal trends from 1990, 2021 by gender, and age in China. 105
Table S51. Prevalence and YLDs of inflammatory bowel disease and its temporal trends from 1990, 2021 by gender, and age in China. 107
Table S52. Prevalence and YLDs of pancreatitis and its temporal trends from 1990, 2021 by gender, and age in China. 109
Table S53. Prevalence and YLDs of male infertility and its temporal trends from 1990, 2021 by gender, and age in China. 111
Table S54. Prevalence and YLDs of female infertility and its temporal trends from 1990, 2021 by gender, and age in China. 112
Table S55. Prevalence and YLDs of depressive disorders and its temporal trends from 1990, 2021 by gender, and age in China. 113
Table S56. Prevalence and YLDs of anxiety disorders and its temporal trends from 1990, 2021 by gender, and age in China. 115
Table S57. Prevalence and YLDs of opioid use disorders and its temporal trends from 1990, 2021 by gender, and age in China. 117
Table S58. Prevalence and YLDs of varicella and herpes zoster and its temporal trends from 1990, 2021 by gender, and age in China. 120
Table S59. Acupuncture demands based on prevalence and YLDs in the worldwide of future forecasts using bayesian age-period-cohort model. 123
Table S60. Prevalence and YLDs of musculoskeletal disorders in the worldwide of future forecasts using bayesian age-period-cohort model. 129
Table S61. Prevalence and YLDs of neurological disorders in the worldwide of future forecasts using bayesian age-period-cohort model. 135
Table S62. Prevalence and YLDs of digestive disorders in the worldwide of future forecasts using bayesian age-period-cohort model. 141
Table S63. Prevalence and YLDs of genecological disorders in the worldwide of future forecasts using bayesian age-period-cohort model. 147
Table S64. Prevalence and YLDs of mental disorders in the worldwide of future forecasts using bayesian age-period-cohort model. 153
Table S65. Prevalence and YLDs of substance use disorders in the worldwide of future forecasts using bayesian age-period-cohort model. 159
Table S66. Prevalence and YLDs of infectious disorders in the worldwide of future forecasts using bayesian age-period-cohort model. 165
Table S67. Prevalence and YLDs of neoplasms in the worldwide of future forecasts using bayesian age-period-cohort model. 171
Table S68. Prevalence and YLDs of neck pain in the worldwide of future forecasts using bayesian age-period-cohort model. 177
Table S69. Prevalence and YLDs of low back pain in the worldwide of future forecasts using bayesian age-period-cohort model. 183
Table S70. Prevalence and YLDs of hip osteoarthritis in the worldwide of future forecasts using bayesian age-period-cohort model. 189
Table S71. Prevalence and YLDs of knee osteoarthritis in the worldwide of future forecasts using bayesian age-period-cohort model. 195
Table S72. Prevalence and YLDs of rheumatoid arthritis in the worldwide of future forecasts using bayesian age-period-cohort model. 201
Table S73. Prevalence and YLDs of tension-type headache in the worldwide of future forecasts using bayesian age-period-cohort model. 207
Table S74. Prevalence and YLDs of migraine in the worldwide of future forecasts using bayesian age-period-cohort model. 213
Table S75. Prevalence and YLDs of stroke in the worldwide of future forecasts using bayesian age-period-cohort model. 219
Table S76. Prevalence and YLDs of Alzheimer's disease and other dementias in the worldwide of future

forecasts using bayesian age-period-cohort model. 225
Table S77. Prevalence and YLDs of Parkinson's disease in the worldwide of future forecasts using bayesian age-period-cohort model. 231
Table S78. Prevalence and YLDs of upper digestive system diseases in the worldwide of future forecasts using bayesian age-period-cohort model. 237
Table S79. Prevalence and YLDs of inflammatory bowel disease in the worldwide of future forecasts using bayesian age-period-cohort model. 243
Table S80. Prevalence and YLDs of pancreatitis in the worldwide of future forecasts using bayesian age-period-cohort model. 249
Table S81. Prevalence and YLDs of male infertility in the worldwide of future forecasts using bayesian age-period-cohort model. 255
Table S82. Prevalence and YLDs of female infertility in the worldwide of future forecasts using bayesian age-period-cohort model. 257
Table S83. Prevalence and YLDs of depressive disorders in the worldwide of future forecasts using bayesian age-period-cohort model. 259
Table S84. Prevalence and YLDs of anxiety disorders in the worldwide of future forecasts using bayesian age-period-cohort model. 265
Table S85. Prevalence and YLDs of opioid use disorders in the worldwide of future forecasts using bayesian age-period-cohort model. 271
Table S86. Prevalence and YLDs of varicella and herpes zoster in the worldwide of future forecasts using bayesian age-period-cohort model. 277
Table S87. Acupuncture demands based on prevalence and YLDs in China of future forecasts using bayesian age-period-cohort model. 283
Table S88. Prevalence and YLDs of musculoskeletal disorders in China of future forecasts using bayesian age-period-cohort model. 289
Table S89. Prevalence and YLDs of neurological disorders in China of future forecasts using bayesian age-period-cohort model. 295
Table S90. Prevalence and YLDs of digestive disorders in China of future forecasts using bayesian age-period-cohort model. 301
Table S91. Prevalence and YLDs of genecological disorders in China of future forecasts using bayesian age-period-cohort model. 307
Table S92. Prevalence and YLDs of mental disorders in China of future forecasts using bayesian age-period-cohort model. 313
Table S93. Prevalence and YLDs of substance use disorders in China of future forecasts using bayesian age-period-cohort model. 319
Table S94. Prevalence and YLDs of infectious disorders in China of future forecasts using bayesian age-period-cohort model. 325
Table S95. Prevalence and YLDs of neoplasms in China of future forecasts using bayesian age-period-cohort model. 331
Table S96. Prevalence and YLDs of neck pain in China of future forecasts using bayesian age-period-cohort model. 337
Table S97. Prevalence and YLDs of low back pain in China of future forecasts using bayesian age-period-cohort model. 343
Table S98. Prevalence and YLDs of hip osteoarthritis in China of future forecasts using bayesian age-period-cohort model. 349
Table S99. Prevalence and YLDs of knee osteoarthritis in China of future forecasts using bayesian age-period-cohort model. 355
Table S100. Prevalence and YLDs of rheumatoid arthritis in China of future forecasts using bayesian age-period-cohort model. 361
Table S101. Prevalence and YLDs of tension-type headache in China of future forecasts using bayesian age-period-cohort model. 367
Table S102. Prevalence and YLDs of migraine in China of future forecasts using bayesian age-period-cohort model. 373
Table S103. Prevalence and YLDs of stroke in China of future forecasts using bayesian

age-period-cohort model. 379
Table S104. Prevalence and YLDs of Alzheimer's disease and other dementias in China of future forecasts using bayesian age-period-cohort model. 385
Table S105. Prevalence and YLDs of Parkinson's disease in China of future forecasts using bayesian age-period-cohort model. 391
Table S106. Prevalence and YLDs of upper digestive system diseases in China of future forecasts using bayesian age-period-cohort model. 397
Table S107. Prevalence and YLDs of inflammatory bowel disease in China of future forecasts using bayesian age-period-cohort model. 403
Table S108. Prevalence and YLDs of pancreatitis in China of future forecasts using bayesian age-period-cohort model. 409
Table S109. Prevalence and YLDs of male infertility in China of future forecasts using bayesian age-period-cohort model. 415
Table S110. Prevalence and YLDs of female infertility in China of future forecasts using bayesian age-period-cohort model. 417
Table S111. Prevalence and YLDs of depressive disorders in China of future forecasts using bayesian age-period-cohort model. 419
Table S112. Prevalence and YLDs of anxiety disorders in China of future forecasts using bayesian age-period-cohort model. 425
Table S113. Prevalence and YLDs of opioid use disorders in China of future forecasts using bayesian age-period-cohort model. 431
Table S114. Prevalence and YLDs of varicella and herpes zoster in China of future forecasts using bayesian age-period-cohort model. 437
Fig. S1 (A) Trends in the number of prevalent cases and years lived with disability of musculoskeletal disorders from 1990 to 2021; (B) Trends in the age-standardized rates of prevalence and years lived with disability of musculoskeletal disorders from 1990 to 2021. 443
Fig. S2 (A) Trends in the number of prevalent cases and years lived with disability of neurological disorders from 1990 to 2021; (B) Trends in the age-standardized rates of prevalence and years lived with disability of neurological disorders from 1990 to 2021. 444
Fig. S3 (A) Trends in the number of prevalent cases and years lived with disability of digestive disorders from 1990 to 2021; (B) Trends in the age-standardized rates of prevalence and years lived with disability of digestive disorders from 1990 to 2021. 445
Fig. S4 (A) Trends in the number of prevalent cases and years lived with disability of genecological disorders from 1990 to 2021; (B) Trends in the age-standardized rates of prevalence and years lived with disability of genecological disorders from 1990 to 2021. 446
Fig. S5 (A) Trends in the number of prevalent cases and years lived with disability of mental disorders from 1990 to 2021; (B) Trends in the age-standardized rates of prevalence and years lived with disability of mental disorders from 1990 to 2021. 447
Fig. S6 (A) Trends in the number of prevalent cases and years lived with disability of substance use disorders from 1990 to 2021; (B) Trends in the age-standardized rates of prevalence and years lived with disability of substance use disorders from 1990 to 2021. 448
Fig. S7 (A) Trends in the number of prevalent cases and years lived with disability of infectious disorders from 1990 to 2021; (B) Trends in the age-standardized rates of prevalence and years lived with disability of infectious disorders from 1990 to 2021. 449
Fig. S8 (A) Trends in the number of prevalent cases and years lived with disability of neoplasms from 1990 to 2021; (B) Trends in the age-standardized rates of prevalence and years lived with disability of neoplasms from 1990 to 2021. 450
Fig. S9 (A) Trends in the number of prevalent cases and years lived with disability of neck pain from 1990 to 2021; (B) Trends in the age-standardized rates of prevalence and years lived with disability of neck pain from 1990 to 2021. 451
Fig. S10 (A) Trends in the number of prevalent cases and years lived with disability of low back pain from 1990 to 2021; (B) Trends in the age-standardized rates of prevalence and years lived with disability of low back pain from 1990 to 2021. 452
Fig. S11 (A) Trends in the number of prevalent cases and years lived with disability of hip osteoarthritis

from 1990 to 2021; (B) Trends in the age-standardized rates of prevalence and years lived with disability of hip osteoarthritis from 1990 to 2021. 453
Fig. S12 (A) Trends in the number of prevalent cases and years lived with disability of knee osteoarthritis from 1990 to 2021; (B) Trends in the age-standardized rates of prevalence and years lived with disability of knee osteoarthritis from 1990 to 2021. 454
Fig. S13 (A) Trends in the number of prevalent cases and years lived with disability of rheumatoid arthritis from 1990 to 2021; (B) Trends in the age-standardized rates of prevalence and years lived with disability of rheumatoid arthritis from 1990 to 2021. 455
Fig. S14 (A) Trends in the number of prevalent cases and years lived with disability of tension-type headache from 1990 to 2021; (B) Trends in the age-standardized rates of prevalence and years lived with disability of tension-type headache from 1990 to 2021. 456
Fig. S15 (A) Trends in the number of prevalent cases and years lived with disability of migraine from 1990 to 2021; (B) Trends in the age-standardized rates of prevalence and years lived with disability of migraine from 1990 to 2021. 457
Fig. S16 (A) Trends in the number of prevalent cases and years lived with disability of stroke from 1990 to 2021; (B) Trends in the age-standardized rates of prevalence and years lived with disability of stroke from 1990 to 2021. 458
Fig. S17 (A) Trends in the number of prevalent cases and years lived with disability of Alzheimer's disease and other dementias from 1990 to 2021; (B) Trends in the age-standardized rates of prevalence and years lived with disability of Alzheimer's disease and other dementias from 1990 to 2021. 459
Fig. S18 (A) Trends in the number of prevalent cases and years lived with disability of Parkinson's disease from 1990 to 2021; (B) Trends in the age-standardized rates of prevalence and years lived with disability of Parkinson's disease from 1990 to 2021. 460
Fig. S20 (A) Trends in the number of prevalent cases and years lived with disability of inflammatory bowel disease from 1990 to 2021; (B) Trends in the age-standardized rates of prevalence and years lived with disability of inflammatory bowel disease from 1990 to 2021. 462
Fig. S21 (A) Trends in the number of prevalent cases and years lived with disability of pancreatitis from 1990 to 2021; (B) Trends in the age-standardized rates of prevalence and years lived with disability of pancreatitis from 1990 to 2021. 463
Fig. S22 (A) Trends in the number of prevalent cases and years lived with disability of male infertility from 1990 to 2021; (B) Trends in the age-standardized rates of prevalence and years lived with disability of male infertility from 1990 to 2021. 464
Fig. S23 (A) Trends in the number of prevalent cases and years lived with disability of female infertility from 1990 to 2021; (B) Trends in the age-standardized rates of prevalence and years lived with disability of female infertility from 1990 to 2021. 465
Fig. S24 (A) Trends in the number of prevalent cases and years lived with disability of depressive disorders from 1990 to 2021; (B) Trends in the age-standardized rates of prevalence and years lived with disability of depressive disorders from 1990 to 2021. 466
Fig. S25 (A) Trends in the number of prevalent cases and years lived with disability of anxiety disorders from 1990 to 2021; (B) Trends in the age-standardized rates of prevalence and years lived with disability of anxiety disorders from 1990 to 2021. 467
Fig. S26 (A) Trends in the number of prevalent cases and years lived with disability of opioid use disorders from 1990 to 2021; (B) Trends in the age-standardized rates of prevalence and years lived with disability of opioid use disorders from 1990 to 2021. 468
Fig. S27 (A) Trends in the number of prevalent cases and years lived with disability of varicella and herpes zoster from 1990 to 2021; (B) Trends in the age-standardized rates of prevalence and years lived with disability of varicella and herpes zoster from 1990 to 2021. 469
Fig. S28 (A) Acupuncture demands based on prevalence cases in different ages from 1990 to 2021 in the worldwide; (B) Acupuncture demands based on prevalence rates in different ages from 1990 to 2021 in the worldwide; (C) Acupuncture demands based on years lived with disability in different ages from 1990 to 2021 in the worldwide; (D) Acupuncture demands based on years lived with disability rates in different ages from 1990 to 2021 in the worldwide. 473
Fig. S29 (A) The prevalence cases of musculoskeletal disorders in different ages from 1990 to 2021 in the worldwide; (B) The prevalence rates of musculoskeletal disorders in different ages from 1990 to

2021 in the worldwide; (C) The years lived with disability of musculoskeletal disorders in different ages from 1990 to 2021 in the worldwide; (D) The years lived with disability rates of musculoskeletal disorders in different ages from 1990 to 2021 in the worldwide. 477
Fig. S30 (A) The prevalence cases of neurological disorders in different ages from 1990 to 2021 in the worldwide; (B) The prevalence rates of neurological disorders in different ages from 1990 to 2021 in the worldwide; (C) The years lived with disability of neurological disorders in different ages from 1990 to 2021 in the worldwide; (D) The years lived with disability rates of neurological disorders in different ages from 1990 to 2021 in the worldwide. 481
Fig. S31 (A) The prevalence cases of digestive disorders in different ages from 1990 to 2021 in the worldwide; (B) The prevalence rates of digestive disorders in different ages from 1990 to 2021 in the worldwide; (C) The years lived with disability of digestive disorders in different ages from 1990 to 2021 in the worldwide; (D) The years lived with disability rates of digestive disorders in different ages from 1990 to 2021 in the worldwide. 485
Fig. S32 (A) The prevalence cases of genecological disorders in different ages from 1990 to 2021 in the worldwide; (B) The prevalence rates of genecological disorders in different ages from 1990 to 2021 in the worldwide; (C) The years lived with disability of genecological disorders in different ages from 1990 to 2021 in the worldwide; (D) The years lived with disability rates of genecological disorders in different ages from 1990 to 2021 in the worldwide. 489
Fig. S33 (A) The prevalence cases of mental disorders in different ages from 1990 to 2021 in the worldwide; (B) The prevalence rates of mental disorders in different ages from 1990 to 2021 in the worldwide; (C) The years lived with disability of mental disorders in different ages from 1990 to 2021 in the worldwide; (D) The years lived with disability rates of mental disorders in different ages from 1990 to 2021 in the worldwide. 493
Fig. S34 (A) The prevalence cases of substance use disorders in different ages from 1990 to 2021 in the worldwide; (B) The prevalence rates of substance use disorders in different ages from 1990 to 2021 in the worldwide; (C) The years lived with disability of substance use disorders in different ages from 1990 to 2021 in the worldwide; (D) The years lived with disability rates of substance use disorders in different ages from 1990 to 2021 in the worldwide. 497
Fig. S35 (A) The prevalence cases of infectious disorders in different ages from 1990 to 2021 in the worldwide; (B) The prevalence rates of infectious disorders in different ages from 1990 to 2021 in the worldwide; (C) The years lived with disability of infectious disorders in different ages from 1990 to 2021 in the worldwide; (D) The years lived with disability rates of infectious disorders in different ages from 1990 to 2021 in the worldwide. 501
Fig. S36 (A) The prevalence cases of neoplasms in different ages from 1990 to 2021 in the worldwide; (B) The prevalence rates of neoplasms in different ages from 1990 to 2021 in the worldwide; (C) The years lived with disability of neoplasms in different ages from 1990 to 2021 in the worldwide; (D) The years lived with disability rates of neoplasms in different ages from 1990 to 2021 in the worldwide. ... 505
Fig. S37 (A) The prevalence cases of neck pain in different ages from 1990 to 2021 in the worldwide; (B) The prevalence rates of neck pain in different ages from 1990 to 2021 in the worldwide; (C) The years lived with disability of neck pain in different ages from 1990 to 2021 in the worldwide; (D) The years lived with disability rates of neck pain in different ages from 1990 to 2021 in the worldwide. 509
Fig. S38 (A) The prevalence cases of low back pain in different ages from 1990 to 2021 in the worldwide; (B) The prevalence rates of low back pain in different ages from 1990 to 2021 in the worldwide; (C) The years lived with disability of low back pain in different ages from 1990 to 2021 in the worldwide; (D) The years lived with disability rates of low back pain in different ages from 1990 to 2021 in the worldwide. 513
Fig. S39 (A) The prevalence cases of hip osteoarthritis in different ages from 1990 to 2021 in the worldwide; (B) The prevalence rates of hip osteoarthritis in different ages from 1990 to 2021 in the worldwide; (C) The years lived with disability of hip osteoarthritis in different ages from 1990 to 2021 in the worldwide; (D) The years lived with disability rates of hip osteoarthritis in different ages from 1990 to 2021 in the worldwide. 517
Fig. S40 (A) The prevalence cases of knee osteoarthritis in different ages from 1990 to 2021 in the worldwide; (B) The prevalence rates of knee osteoarthritis in different ages from 1990 to 2021 in the worldwide; (C) The years lived with disability of knee osteoarthritis in different ages from 1990 to 2021

in the worldwide; (D) The years lived with disability rates of knee osteoarthritis in different ages from 1990 to 2021 in the worldwide. 521
Fig. S41 (A) The prevalence cases of rheumatoid arthritis in different ages from 1990 to 2021 in the worldwide; (B) The prevalence rates of rheumatoid arthritis in different ages from 1990 to 2021 in the worldwide; (C) The years lived with disability of rheumatoid arthritis in different ages from 1990 to 2021 in the worldwide; (D) The years lived with disability rates of rheumatoid arthritis in different ages from 1990 to 2021 in the worldwide. 525
Fig. S42 (A) The prevalence cases of tension-type headache in different ages from 1990 to 2021 in the worldwide; (B) The prevalence rates of tension-type headache in different ages from 1990 to 2021 in the worldwide; (C) The years lived with disability of tension-type headache in different ages from 1990 to 2021 in the worldwide; (D) The years lived with disability rates of tension-type headache in different ages from 1990 to 2021 in the worldwide. 529
Fig. S43 (A) The prevalence cases of migraine in different ages from 1990 to 2021 in the worldwide; (B) The prevalence rates of migraine in different ages from 1990 to 2021 in the worldwide; (C) The years lived with disability of migraine in different ages from 1990 to 2021 in the worldwide; (D) The years lived with disability rates of migraine in different ages from 1990 to 2021 in the worldwide. 533
Fig. S44 (A) The prevalence cases of stroke in different ages from 1990 to 2021 in the worldwide; (B) The prevalence rates of stroke in different ages from 1990 to 2021 in the worldwide; (C) The years lived with disability of stroke in different ages from 1990 to 2021 in the worldwide; (D) The years lived with disability rates of stroke in different ages from 1990 to 2021 in the worldwide. 537
Fig. S45 (A) The prevalence cases of Alzheimer's disease and other dementias in different ages from 1990 to 2021 in the worldwide; (B) The prevalence rates of Alzheimer's disease and other dementias in different ages from 1990 to 2021 in the worldwide; (C) The years lived with disability of Alzheimer's disease and other dementias in different ages from 1990 to 2021 in the worldwide; (D) The years lived with disability rates of Alzheimer's disease and other dementias in different ages from 1990 to 2021 in the worldwide. 541
Fig. S46 (A) The prevalence cases of Parkinson's disease in different ages from 1990 to 2021 in the worldwide; (B) The prevalence rates of Parkinson's disease in different ages from 1990 to 2021 in the worldwide; (C) The years lived with disability of Parkinson's disease in different ages from 1990 to 2021 in the worldwide; (D) The years lived with disability rates of Parkinson's disease in different ages from 1990 to 2021 in the worldwide. 545
Fig. S47 (A) The prevalence cases of upper digestive system diseases in different ages from 1990 to 2021 in the worldwide; (B) The prevalence rates of upper digestive system diseases in different ages from 1990 to 2021 in the worldwide; (C) The years lived with disability of upper digestive system diseases in different ages from 1990 to 2021 in the worldwide; (D) The years lived with disability rates of upper digestive system diseases in different ages from 1990 to 2021 in the worldwide. 549
Fig. S48 (A) The prevalence cases of inflammatory bowel disease in different ages from 1990 to 2021 in the worldwide; (B) The prevalence rates of inflammatory bowel disease in different ages from 1990 to 2021 in the worldwide; (C) The years lived with disability of inflammatory bowel disease in different ages from 1990 to 2021 in the worldwide; (D) The years lived with disability rates of inflammatory bowel disease in different ages from 1990 to 2021 in the worldwide. 553
Fig. S49 (A) The prevalence cases of pancreatitis in different ages from 1990 to 2021 in the worldwide; (B) The prevalence rates of pancreatitis in different ages from 1990 to 2021 in the worldwide; (C) The years lived with disability of pancreatitis in different ages from 1990 to 2021 in the worldwide; (D) The years lived with disability rates of pancreatitis in different ages from 1990 to 2021 in the worldwide.. 557
Fig. S50 (A) The prevalence cases of male infertility in different ages from 1990 to 2021 in the worldwide; (B) The prevalence rates of male infertility in different ages from 1990 to 2021 in the worldwide; (C) The years lived with disability of male infertility in different ages from 1990 to 2021 in the worldwide; (D) The years lived with disability rates of male infertility in different ages from 1990 to 2021 in the worldwide. 561
Fig. S51 (A) The prevalence cases of female infertility in different ages from 1990 to 2021 in the worldwide; (B) The prevalence rates of female infertility in different ages from 1990 to 2021 in the worldwide; (C) The years lived with disability of female infertility in different ages from 1990 to 2021 in the worldwide; (D) The years lived with disability rates of female infertility in different ages from 1990

to 2021 in the worldwide. 565
Fig. S52 (A) The prevalence cases of depressive disorders in different ages from 1990 to 2021 in the worldwide; (B) The prevalence rates of depressive disorders in different ages from 1990 to 2021 in the worldwide; (C) The years lived with disability of depressive disorders in different ages from 1990 to 2021 in the worldwide; (D) The years lived with disability rates of depressive disorders in different ages from 1990 to 2021 in the worldwide. 569
Fig. S53 (A) The prevalence cases of anxiety disorders in different ages from 1990 to 2021 in the worldwide; (B) The prevalence rates of anxiety disorders in different ages from 1990 to 2021 in the worldwide; (C) The years lived with disability of anxiety disorders in different ages from 1990 to 2021 in the worldwide; (D) The years lived with disability rates of anxiety disorders in different ages from 1990 to 2021 in the worldwide. 573
Fig. S54 (A) The prevalence cases of opioid use disorders in different ages from 1990 to 2021 in the worldwide; (B) The prevalence rates of opioid use disorders in different ages from 1990 to 2021 in the worldwide; (C) The years lived with disability of opioid use disorders in different ages from 1990 to 2021 in the worldwide; (D) The years lived with disability rates of opioid use disorders in different ages from 1990 to 2021 in the worldwide. 577
Fig. S55 (A) The prevalence cases of varicella and herpes zoster in different ages from 1990 to 2021 in the worldwide; (B) The prevalence rates of varicella and herpes zoster in different ages from 1990 to 2021 in the worldwide; (C) The years lived with disability of varicella and herpes zoster in different ages from 1990 to 2021 in the worldwide; (D) The years lived with disability rates of varicella and herpes zoster in different ages from 1990 to 2021 in the worldwide. 581
Fig. S56 (A) Acupuncture demands based on prevalence cases in different ages from 1990 to 2021 in China; (B) Acupuncture demands based on prevalence rates in different ages from 1990 to 2021 in China; (C) Acupuncture demands based on years lived with disability in different ages from 1990 to 2021 in China; (D) Acupuncture demands based on years lived with disability rates in different ages from 1990 to 2021 in China. 585
Fig. S57 (A) The prevalence cases of musculoskeletal disorders in different ages from 1990 to 2021 in China; (B) The prevalence rates of musculoskeletal disorders in different ages from 1990 to 2021 in China; (C) The years lived with disability of musculoskeletal disorders in different ages from 1990 to 2021 in China; (D) The years lived with disability rates of musculoskeletal disorders in different ages from 1990 to 2021 in China. 589
Fig. S58 (A) The prevalence cases of neurological disorders in different ages from 1990 to 2021 in China; (B) The prevalence rates of neurological disorders in different ages from 1990 to 2021 in China; (C) The years lived with disability of neurological disorders in different ages from 1990 to 2021 in China; (D) The years lived with disability rates of neurological disorders in different ages from 1990 to 2021 in China. 593
Fig. S59 (A) The prevalence cases of digestive disorders in different ages from 1990 to 2021 in China; (B) The prevalence rates of digestive disorders in different ages from 1990 to 2021 in China; (C) The years lived with disability of digestive disorders in different ages from 1990 to 2021 in China; (D) The years lived with disability rates of digestive disorders in different ages from 1990 to 2021 in China. 597
Fig. S60 (A) The prevalence cases of genecological disorders in different ages from 1990 to 2021 in China; (B) The prevalence rates of genecological disorders in different ages from 1990 to 2021 in China; (C) The years lived with disability of genecological disorders in different ages from 1990 to 2021 in China; (D) The years lived with disability rates of genecological disorders in different ages from 1990 to 2021 in China. 601
Fig. S61 (A) The prevalence cases of mental disorders in different ages from 1990 to 2021 in China; (B) The prevalence rates of mental disorders in different ages from 1990 to 2021 in China; (C) The years lived with disability of mental disorders in different ages from 1990 to 2021 in China; (D) The years lived with disability rates of mental disorders in different ages from 1990 to 2021 in China. 605
Fig. S62 (A) The prevalence cases of substance use disorders in different ages from 1990 to 2021 in China; (B) The prevalence rates of substance use disorders in different ages from 1990 to 2021 in China; (C) The years lived with disability of substance use disorders in different ages from 1990 to 2021 in China; (D) The years lived with disability rates of substance use disorders in different ages from 1990 to 2021 in China. 609

Fig. S63 (A) The prevalence cases of infectious disorders in different ages from 1990 to 2021 in China; (B) The prevalence rates of infectious disorders in different ages from 1990 to 2021 in China; (C) The years lived with disability of infectious disorders in different ages from 1990 to 2021 in China; (D) The years lived with disability rates of infectious disorders in different ages from 1990 to 2021 in China...613
Fig. S64 (A) The prevalence cases of neoplasms in different ages from 1990 to 2021 in China; (B) The prevalence rates of neoplasms in different ages from 1990 to 2021 in China; (C) The years lived with disability of neoplasms in different ages from 1990 to 2021 in China; (D) The years lived with disability rates of neoplasms in different ages from 1990 to 2021 in China. 617
Fig. S65 (A) The prevalence cases of neck pain in different ages from 1990 to 2021 in China; (B) The prevalence rates of neck pain in different ages from 1990 to 2021 in China; (C) The years lived with disability of neck pain in different ages from 1990 to 2021 in China; (D) The years lived with disability rates of neck pain in different ages from 1990 to 2021 in China. 621
Fig. S66 (A) The prevalence cases of low back pain in different ages from 1990 to 2021 in China; (B) The prevalence rates of low back pain in different ages from 1990 to 2021 in China; (C) The years lived with disability of low back pain in different ages from 1990 to 2021 in China; (D) The years lived with disability rates of low back pain in different ages from 1990 to 2021 in China. 625
Fig. S67 (A) The prevalence cases of hip osteoarthritis in different ages from 1990 to 2021 in China; (B) The prevalence rates of hip osteoarthritis in different ages from 1990 to 2021 in China; (C) The years lived with disability of hip osteoarthritis in different ages from 1990 to 2021 in China; (D) The years lived with disability rates of hip osteoarthritis in different ages from 1990 to 2021 in China. 629
Fig. S68 (A) The prevalence cases of knee osteoarthritis in different ages from 1990 to 2021 in China; (B) The prevalence rates of knee osteoarthritis in different ages from 1990 to 2021 in China; (C) The years lived with disability of knee osteoarthritis in different ages from 1990 to 2021 in China; (D) The years lived with disability rates of knee osteoarthritis in different ages from 1990 to 2021 in China. 633
Fig. S69 (A) The prevalence cases of rheumatoid arthritis in different ages from 1990 to 2021 in China; (B) The prevalence rates of rheumatoid arthritis in different ages from 1990 to 2021 in China; (C) The years lived with disability of rheumatoid arthritis in different ages from 1990 to 2021 in China; (D) The years lived with disability rates of rheumatoid arthritis in different ages from 1990 to 2021 in China.. 637
Fig. S70 (A) The prevalence cases of tension-type headache in different ages from 1990 to 2021 in China; (B) The prevalence rates of tension-type headache in different ages from 1990 to 2021 in China; (C) The years lived with disability of tension-type headache in different ages from 1990 to 2021 in China; (D) The years lived with disability rates of tension-type headache in different ages from 1990 to 2021 in China. 641
Fig. S71 (A) The prevalence cases of migraine in different ages from 1990 to 2021 in China; (B) The prevalence rates of migraine in different ages from 1990 to 2021 in China; (C) The years lived with disability of migraine in different ages from 1990 to 2021 in China; (D) The years lived with disability rates of migraine in different ages from 1990 to 2021 in China. 645
Fig. S72 (A) The prevalence cases of stroke in different ages from 1990 to 2021 in China; (B) The prevalence rates of stroke in different ages from 1990 to 2021 in China; (C) The years lived with disability of stroke in different ages from 1990 to 2021 in China; (D) The years lived with disability rates of stroke in different ages from 1990 to 2021 in China. 649
Fig. S73 (A) The prevalence cases of Alzheimer's disease and other dementias in different ages from 1990 to 2021 in China; (B) The prevalence rates of Alzheimer's disease and other dementias in different ages from 1990 to 2021 in China; (C) The years lived with disability of Alzheimer's disease and other dementias in different ages from 1990 to 2021 in China; (D) The years lived with disability rates of Alzheimer's disease and other dementias in different ages from 1990 to 2021 in China. 653
Fig. S74 (A) The prevalence cases of Parkinson's disease in different ages from 1990 to 2021 in China; (B) The prevalence rates of Parkinson's disease in different ages from 1990 to 2021 in China; (C) The years lived with disability of Parkinson's disease in different ages from 1990 to 2021 in China; (D) The years lived with disability rates of Parkinson's disease in different ages from 1990 to 2021 in China... 657
Fig. S75 (A) The prevalence cases of upper digestive system diseases in different ages from 1990 to 2021 in China; (B) The prevalence rates of upper digestive system diseases in different ages from 1990 to 2021 in China; (C) The years lived with disability of upper digestive system diseases in different ages from 1990 to 2021 in China; (D) The years lived with disability rates of upper digestive system diseases

in different ages from 1990 to 2021 in China. 661
Fig. S76 (A) The prevalence cases of inflammatory bowel disease in different ages from 1990 to 2021 in China; (B) The prevalence rates of inflammatory bowel disease in different ages from 1990 to 2021 in China; (C) The years lived with disability of inflammatory bowel disease in different ages from 1990 to 2021 in China; (D) The years lived with disability rates of inflammatory bowel disease in different ages from 1990 to 2021 in China. 665
Fig. S77 (A) The prevalence cases of pancreatitis in different ages from 1990 to 2021 in China; (B) The prevalence rates of pancreatitis in different ages from 1990 to 2021 in China; (C) The years lived with disability of pancreatitis in different ages from 1990 to 2021 in China; (D) The years lived with disability rates of pancreatitis in different ages from 1990 to 2021 in China. 669
Fig. S78 (A) The prevalence cases of male infertility in different ages from 1990 to 2021 in China; (B) The prevalence rates of male infertility in different ages from 1990 to 2021 in China; (C) The years lived with disability of male infertility in different ages from 1990 to 2021 in China; (D) The years lived with disability rates of male infertility in different ages from 1990 to 2021 in China. 673
Fig. S79 (A) The prevalence cases of female infertility in different ages from 1990 to 2021 in China; (B) The prevalence rates of female infertility in different ages from 1990 to 2021 in China; (C) The years lived with disability of female infertility in different ages from 1990 to 2021 in China; (D) The years lived with disability rates of female infertility in different ages from 1990 to 2021 in China. 677
Fig. S80 (A) The prevalence cases of depressive disorders in different ages from 1990 to 2021 in China; (B) The prevalence rates of depressive disorders in different ages from 1990 to 2021 in China; (C) The years lived with disability of depressive disorders in different ages from 1990 to 2021 in China; (D) The years lived with disability rates of depressive disorders in different ages from 1990 to 2021 in China. .681
Fig. S81 (A) The prevalence cases of anxiety disorders in different ages from 1990 to 2021 in China; (B) The prevalence rates of anxiety disorders in different ages from 1990 to 2021 in China; (C) The years lived with disability of anxiety disorders in different ages from 1990 to 2021 in China; (D) The years lived with disability rates of anxiety disorders in different ages from 1990 to 2021 in China. 685
Fig. S82 (A) The prevalence cases of opioid use disorders in different ages from 1990 to 2021 in China; (B) The prevalence rates of opioid use disorders in different ages from 1990 to 2021 in China; (C) The years lived with disability of opioid use disorders in different ages from 1990 to 2021 in China; (D) The years lived with disability rates of opioid use disorders in different ages from 1990 to 2021 in China. . 689
Fig. S83 (A) The prevalence cases of varicella and herpes zoster in different ages from 1990 to 2021 in China; (B) The prevalence rates of varicella and herpes zoster in different ages from 1990 to 2021 in China; (C) The years lived with disability of varicella and herpes zoster in different ages from 1990 to 2021 in China; (D) The years lived with disability rates of varicella and herpes zoster in different ages from 1990 to 2021 in China. 693
Fig. S84 (A) The number of prevalent cases and years lived with disability of musculoskeletal disorders in different ages in 2021; (B) The age-standardized rates of prevalent cases and years lived with disability of musculoskeletal disorders in different ages in 2021. 694
Fig. S85 (A) The number of prevalent cases and years lived with disability of neurological disorders in different ages in 2021; (B) The age-standardized rates of prevalent cases and years lived with disability of neurological disorders in different ages in 2021. 695
Fig. S86 (A) The number of prevalent cases and years lived with disability of digestive disorders in different ages in 2021; (B) The age-standardized rates of prevalent cases and years lived with disability of digestive disorders in different ages in 2021. 696
Fig. S87 (A) The number of prevalent cases and years lived with disability of genecological disorders in different ages in 2021; (B) The age-standardized rates of prevalent cases and years lived with disability of genecological disorders in different ages in 2021. 697
Fig. S88 (A) The number of prevalent cases and years lived with disability of mental disorders in different ages in 2021; (B) The age-standardized rates of prevalent cases and years lived with disability of mental disorders in different ages in 2021. 698
Fig. S89 (A) The number of prevalent cases and years lived with disability of substance use disorders in different ages in 2021; (B) The age-standardized rates of prevalent cases and years lived with disability of substance use disorders in different ages in 2021. 699
Fig. S90 (A) The number of prevalent cases and years lived with disability of infectious disorders in

different ages in 2021; (B) The age-standardized rates of prevalent cases and years lived with disability of infectious disorders in different ages in 2021. 700
Fig. S91 (A) The number of prevalent cases and years lived with disability of neoplasms in different ages in 2021; (B) The age-standardized rates of prevalent cases and years lived with disability of neoplasms in different ages in 2021. 701
Fig. S92 (A) The number of prevalent cases and years lived with disability of neck pain in different ages in 2021; (B) The age-standardized rates of prevalent cases and years lived with disability of neck pain in different ages in 2021. 702
Fig. S93 (A) The number of prevalent cases and years lived with disability of low back pain in different ages in 2021; (B) The age-standardized rates of prevalent cases and years lived with disability of low back pain in different ages in 2021. 703
Fig. S94 (A) The number of prevalent cases and years lived with disability of hip osteoarthritis in different ages in 2021; (B) The age-standardized rates of prevalent cases and years lived with disability of hip osteoarthritis in different ages in 2021. 704
Fig. S95 (A) The number of prevalent cases and years lived with disability of knee osteoarthritis in different ages in 2021; (B) The age-standardized rates of prevalent cases and years lived with disability of knee osteoarthritis in different ages in 2021. 705
Fig. S96 (A) The number of prevalent cases and years lived with disability of rheumatoid arthritis in different ages in 2021; (B) The age-standardized rates of prevalent cases and years lived with disability of rheumatoid arthritis in different ages in 2021. 706
Fig. S97 (A) The number of prevalent cases and years lived with disability of tension-type headache in different ages in 2021; (B) The age-standardized rates of prevalent cases and years lived with disability of tension-type headache in different ages in 2021. 707
Fig. S98 (A) The number of prevalent cases and years lived with disability of migraine in different ages in 2021; (B) The age-standardized rates of prevalent cases and years lived with disability of migraine in different ages in 2021. 708
Fig. S99 (A) The number of prevalent cases and years lived with disability of stroke in different ages in 2021; (B) The age-standardized rates of prevalent cases and years lived with disability of stroke in different ages in 2021. 709
Fig. S100 (A) The number of prevalent cases and years lived with disability of Alzheimer's disease and other dementias in different ages in 2021; (B) The age-standardized rates of prevalent cases and years lived with disability of Alzheimer's disease and other dementias in different ages in 2021. 710
Fig. S101 (A) The number of prevalent cases and years lived with disability of Parkinson's disease in different ages in 2021; (B) The age-standardized rates of prevalent cases and years lived with disability of Parkinson's disease in different ages in 2021. 711
Fig. S102 (A) The number of prevalent cases and years lived with disability of upper digestive system diseases in different ages in 2021; (B) The age-standardized rates of prevalent cases and years lived with disability of upper digestive system diseases in different ages in 2021. 712
Fig. S103 (A) The number of prevalent cases and years lived with disability of inflammatory bowel disease in different ages in 2021; (B) The age-standardized rates of prevalent cases and years lived with disability of inflammatory bowel disease in different ages in 2021. 713
Fig. S104 (A) The number of prevalent cases and years lived with disability of pancreatitis in different ages in 2021; (B) The age-standardized rates of prevalent cases and years lived with disability of pancreatitis in different ages in 2021. 714
Fig. S105 (A) The number of prevalent cases and years lived with disability of male infertility in different ages in 2021; (B) The age-standardized rates of prevalent cases and years lived with disability of male infertility in different ages in 2021. 715
Fig. S106 (A) The number of prevalent cases and years lived with disability of female infertility in different ages in 2021; (B) The age-standardized rates of prevalent cases and years lived with disability of female infertility in different ages in 2021. 716
Fig. S107 (A) The number of prevalent cases and years lived with disability of depressive disorders in different ages in 2021; (B) The age-standardized rates of prevalent cases and years lived with disability of depressive disorders in different ages in 2021. 717
Fig. S108 (A) The number of prevalent cases and years lived with disability of anxiety disorders in

different ages in 2021; (B) The age-standardized rates of prevalent cases and years lived with disability of anxiety disorders in different ages in 2021. 718
Fig. S109 (A) The number of prevalent cases and years lived with disability of opioid use disorders in different ages in 2021; (B) The age-standardized rates of prevalent cases and years lived with disability of opioid use disorders in different ages in 2021. 719
Fig. S110 (A) The number of prevalent cases and years lived with disability of varicella and herpes zoster in different ages in 2021; (B) The age-standardized rates of prevalent cases and years lived with disability of varicella and herpes zoster in different agesin different ages in 2021. 720
Fig. S111 (A) Decomposition analysis of musculoskeletal disorders change in prevalence from 1990 to 2021; (B) Decomposition analysis of musculoskeletal disorders change in years lived with disability from 1990 to 2021. 721
Fig. S112 (A) Decomposition analysis of digestive disorders change in prevalence from 1990 to 2021; (B) Decomposition analysis of digestive disorders change in years lived with disability from 1990 to 2021.722
Fig. S113 (A) Decomposition analysis of genecological disorders disorders change in prevalence from 1990 to 2021; (B) Decomposition analysis of genecological disorders change in years lived with disability from 1990 to 2021. 723
Fig. S114 (A) Decomposition analysis of mental disorders change in prevalence from 1990 to 2021; (B) Decomposition analysis of mental disorders change in years lived with disability from 1990 to 2021. . 724
Fig. S115 (A) Decomposition analysis of substance use disorders change in prevalence from 1990 to 2021; (B) Decomposition analysis of substance use disorders change in years lived with disability from 1990 to 2021. 725
Fig. S116 (A) Decomposition analysis of infectious disorders change in prevalence from 1990 to 2021; (B) Decomposition analysis of infectious disorders change in years lived with disability from 1990 to 2021. 726
Fig. S117 (A) Decomposition analysis of neoplasms change in prevalence from 1990 to 2021; (B) Decomposition analysis of neoplasms change in years lived with disability from 1990 to 2021. 727
Fig. S118 (A) Decomposition analysis of neck pain change in prevalence from 1990 to 2021; (B) Decomposition analysis of neck pain change in years lived with disability from 1990 to 2021. 728
Fig. S119 (A) Decomposition analysis of low back pain change in prevalence from 1990 to 2021; (B) Decomposition analysis of low back pain change in years lived with disability from 1990 to 2021. 729
Fig. S120 (A) Decomposition analysis of hip osteoarthritis change in prevalence from 1990 to 2021; (B) Decomposition analysis of hip osteoarthritis change in years lived with disability from 1990 to 2021. 730
Fig. S121 (A) Decomposition analysis of knee osteoarthritis change in prevalence from 1990 to 2021; (B) Decomposition analysis of knee osteoarthritis change in years lived with disability from 1990 to 2021.731
Fig. S122 (A) Decomposition analysis of rheumatoid arthritis change in prevalence from 1990 to 2021; (B) Decomposition analysis of rheumatoid arthritis change in years lived with disability from 1990 to 2021. 732
Fig. S123 (A) Decomposition analysis of tension-type headache change in prevalence from 1990 to 2021; (B) Decomposition analysis of tension-type headache change in years lived with disability from 1990 to 2021. 733
Fig. S124 (A) Decomposition analysis of migraine change in prevalence from 1990 to 2021; (B) Decomposition analysis of migraine change in years lived with disability from 1990 to 2021. 734
Fig. S125 (A) Decomposition analysis of stroke change in prevalence from 1990 to 2021; (B) Decomposition analysis of stroke change in years lived with disability from 1990 to 2021. 735
Fig. S126 (A) Decomposition analysis of Alzheimer's disease and other dementias change in prevalence from 1990 to 2021; (B) Decomposition analysis of Alzheimer's disease and other dementias change in years lived with disability from 1990 to 2021. 736
Fig. S127 (A) Decomposition analysis of Parkinson's disease change in prevalence from 1990 to 2021; (B) Decomposition analysis of Parkinson's disease change in years lived with disability from 1990 to 2021. 737
Fig. S128 (A) Decomposition analysis of upper digestive system diseases change in prevalence from 1990 to 2021; (B) Decomposition analysis of upper digestive system diseases change in years lived with disability from 1990 to 2021. 738
Fig. S129 (A) Decomposition analysis of inflammatory bowel disease change in prevalence from 1990 to

2021; (B) Decomposition analysis of inflammatory bowel disease change in years lived with disability from 1990 to 2021. 739
Fig. S130 (A) Decomposition analysis of pancreatitis change in prevalence from 1990 to 2021; (B) Decomposition analysis of pancreatitis change in years lived with disability from 1990 to 2021. 740
Fig. S131 (A) Decomposition analysis of male infertility change in prevalence from 1990 to 2021; (B) Decomposition analysis of male infertility change in years lived with disability from 1990 to 2021. 741
Fig. S132 (A) Decomposition analysis of female infertility change in prevalence from 1990 to 2021; (B) Decomposition analysis of female infertility change in years lived with disability from 1990 to 2021. 742
Fig. S133 (A) Decomposition analysis of depressive disorders change in prevalence from 1990 to 2021; (B) Decomposition analysis of depressive disorders change in years lived with disability from 1990 to 2021. 743
Fig. S134 (A) Decomposition analysis of anxiety disorders change in prevalence from 1990 to 2021; (B) Decomposition analysis of anxiety disorders change in years lived with disability from 1990 to 2021. 744
Fig. S135 (A) Decomposition analysis of opioid use disorders change in prevalence from 1990 to 2021; (B) Decomposition analysis of opioid use disorders change in years lived with disability from 1990 to 2021. 745
Fig. S136 (A) Decomposition analysis of varicella and herpes zoster change in prevalence from 1990 to 2021; (B) Decomposition analysis of varicella and herpes zoster change in years lived with disability from 1990 to 2021. 746
Fig. S137 (A) Future forecasts of Global Burden of Disease in musculoskeletal disorders burden based on prevalence using bayesian age-period-cohort model; (B) Future forecasts of Global Burden of Disease in musculoskeletal disorders burden based on years lived with disability using bayesian age-period-cohort model. 747
Fig. S138 (A) Future forecasts of Global Burden of Disease in neurological disorders burden based on prevalence using bayesian age-period-cohort model; (B) Future forecasts of Global Burden of Disease in neurological disorders burden based on years lived with disability using bayesian age-period-cohort model. 748
Fig. S139 (A) Future forecasts of Global Burden of Disease in digestive disorders burden based on prevalence using bayesian age-period-cohort model; (B) Future forecasts of Global Burden of Disease in digestive disorders burden based on years lived with disability using bayesian age-period-cohort model.749
Fig. S140 (A) Future forecasts of Global Burden of Disease in genecological disorders burden based on prevalence using bayesian age-period-cohort model; (B) Future forecasts of Global Burden of Disease in genecological disorders burden based on years lived with disability using bayesian age-period-cohort model. 750
Fig. S141 (A) Future forecasts of Global Burden of Disease in mental disorders burden based on prevalence using bayesian age-period-cohort model; (B) Future forecasts of Global Burden of Disease in mental disorders burden based on years lived with disability using bayesian age-period-cohort model. 751
Fig. S142 (A) Future forecasts of Global Burden of Disease in substance use disorders burden based on prevalence using bayesian age-period-cohort model; (B) Future forecasts of Global Burden of Disease in substance use disorders burden based on years lived with disability using bayesian age-period-cohort model. 752
Fig. S143 (A) Future forecasts of Global Burden of Disease in infectious disorders burden based on prevalence using bayesian age-period-cohort model; (B) Future forecasts of Global Burden of Disease in infectious disorders burden based on years lived with disability using bayesian age-period-cohort model.753
Fig. S146 (A) Future forecasts of Global Burden of Disease in low back pain burden based on prevalence using bayesian age-period-cohort model; (B) Future forecasts of Global Burden of Disease in low back pain burden based on years lived with disability using bayesian age-period-cohort model. 756
Fig. S147 (A) Future forecasts of Global Burden of Disease in hip osteoarthritis burden based on prevalence using bayesian age-period-cohort model; (B) Future forecasts of Global Burden of Disease in hip osteoarthritis burden based on years lived with disability using bayesian age-period-cohort model.757
Fig. S148 (A) Future forecasts of Global Burden of Disease in knee osteoarthritis burden based on prevalence using bayesian age-period-cohort model; (B) Future forecasts of Global Burden of Disease in knee osteoarthritis burden based on years lived with disability using bayesian age-period-cohort model.758
Fig. S149 (A) Future forecasts of Global Burden of Disease in rheumatoid arthritis burden based on

prevalence using bayesian age-period-cohort model; (B) Future forecasts of Global Burden of Disease in rheumatoid arthritis burden based on years lived with disability using bayesian age-period-cohort model.759
Fig. S150 (A) Future forecasts of Global Burden of Disease in tension-type headache burden based on prevalence using bayesian age-period-cohort model; (B) Future forecasts of Global Burden of Disease in tension-type headache burden based on years lived with disability using bayesian age-period-cohort model. 760
Fig. S151 (A) Future forecasts of Global Burden of Disease in migraine burden based on prevalence using bayesian age-period-cohort model; (B) Future forecasts of Global Burden of Disease in migraine burden based on years lived with disability using bayesian age-period-cohort model. 761
Fig. S152 (A) Future forecasts of Global Burden of Disease in stroke burden based on prevalence using bayesian age-period-cohort model; (B) Future forecasts of Global Burden of Disease in stroke burden based on years lived with disability using bayesian age-period-cohort model. 762
Fig. S153 (A) Future forecasts of Global Burden of Disease in Alzheimer's disease and other dementias burden based on prevalence using bayesian age-period-cohort model; (B) Future forecasts of Global Burden of Disease in Alzheimer's disease and other dementias burden based on years lived with disability using bayesian age-period-cohort model. 763
Fig. S154 (A) Future forecasts of Global Burden of Disease in Parkinson's disease burden based on prevalence using bayesian age-period-cohort model; (B) Future forecasts of Global Burden of Disease in Parkinson's disease burden based on years lived with disability using bayesian age-period-cohort model.764
Fig. S155 (A) Future forecasts of Global Burden of Disease in upper digestive system diseases burden based on prevalence using bayesian age-period-cohort model; (B) Future forecasts of Global Burden of Disease in upper digestive system diseases burden based on years lived with disability using bayesian age-period-cohort model. 765
Fig. S156 (A) Future forecasts of Global Burden of Disease in inflammatory bowel disease burden based on prevalence using bayesian age-period-cohort model; (B) Future forecasts of Global Burden of Disease in inflammatory bowel disease burden based on years lived with disability using bayesian age-period-cohort model. 766
Fig. S157 (A) Future forecasts of Global Burden of Disease in pancreatitis burden based on prevalence using bayesian age-period-cohort model; (B) Future forecasts of Global Burden of Disease in pancreatitis burden based on years lived with disability using bayesian age-period-cohort model. 767
Fig. S158 (A) Future forecasts of Global Burden of Disease in male infertility burden based on prevalence using bayesian age-period-cohort model; (B) Future forecasts of Global Burden of Disease in male infertility burden based on years lived with disability using bayesian age-period-cohort model... 768
Fig. S159 (A) Future forecasts of Global Burden of Disease in female infertility burden based on prevalence using bayesian age-period-cohort model; (B) Future forecasts of Global Burden of Disease in female infertility burden based on years lived with disability using bayesian age-period-cohort model.769
Fig. S160 (A) Future forecasts of Global Burden of Disease in depressive disorders burden based on prevalence using bayesian age-period-cohort model; (B) Future forecasts of Global Burden of Disease in depressive disorders burden based on years lived with disability using bayesian age-period-cohort model.770
Fig. S161 (A) Future forecasts of Global Burden of Disease in anxiety disorders burden based on prevalence using bayesian age-period-cohort model; (B) Future forecasts of Global Burden of Disease in anxiety disorders burden based on years lived with disability using bayesian age-period-cohort model.771
Fig. S162 (A) Future forecasts of Global Burden of Disease in opioid use disorders burden based on prevalence using bayesian age-period-cohort model; (B) Future forecasts of Global Burden of Disease in opioid use disorders burden based on years lived with disability using bayesian age-period-cohort model.772
Fig. S163 (A) Future forecasts of Global Burden of Disease in varicella and herpes zoster burden based on prevalence using bayesian age-period-cohort model; (B) Future forecasts of Global Burden of Disease in varicella and herpes zoster burden based on years lived with disability using bayesian age-period-cohort model. 773

Part I. Detailed definitions of 20 specific health conditions

There are musculoskeletal disorders [neck pain, low back pain, hip osteoarthritis, knee osteoarthritis, and rheumatoid arthritis], neurological disorders [tension-type headache, migraine, stroke, Alzheimer's disease and other dementias, Parkinson's disease], digestive disorders [upper digestive system diseases, inflammatory bowel disease, pancreatitis], genecological disorders (male infertility, and female infertility), mental disorders (depressive disorders and anxiety disorders), substance use disorders (opioid use disorders), infectious disorders (herpes zoster) and neoplasms - all of which have been identified as acupuncture-responsive or acupuncture-preferred conditions based on clinical evidence and international guidelines.

1. Neck Pain

1.1 Code: ME84.0

1.2 Description: This is a condition which is usually characterised by pain or discomfort in the neck region and can be caused by numerous spinal problems. It may be a feature of virtually every disorder and disease that occurs above the shoulder blades.

1.3.1 Finding: Individualized acupuncture interventions using high- or low-sensitivity acupuncture points were more effective in reducing chronic neck pain than sham acupoints and waiting list control groups sustained through 24 weeks [1].

1.3.2 Finding: This study results suggest that 4-week optimized acupuncture treatment alleviates cervical spondylosis-related neck pain and improves the quality of life, with the effects persisting for minimum 3 months [2].

1.3.3 Finding: Acupuncture sessions and Alexander Technique lessons both led to significant reductions in neck pain and associated disability compared with usual care at 12 months [3].

2. Low Back Pain

2.1 Code: ME84.2

2.2 Description: This is a condition which is defined as pain and discomfort, localised below the costal margin and above the inferior gluteal folds, with or without leg pain.

2.3.1 Findings: This randomized sham-controlled trial suggests that acupuncture treatment shows better effect on the reduction of the bothersomeness and pain intensity than sham control in participants with chronic low back pain [4].

2.3.2 Findings: Acupuncture probably improves function slightly for people with chronic low back pain, compared to sham acupuncture. Acupuncture probably reduces pain intensity, and improves function slightly for people with chronic low back pain, compared to no treatment. Acupuncture probably improves function slightly for people with chronic low back pain, compared to usual care [5].

2.3.3 Findings: Recommendation 1: Given that most patients with acute or subacute low back pain improve over time regardless of treatment, clinicians and patients should select non-pharmacologic treatment with superficial heat (moderate-quality evidence), massage, acupuncture, or spinal manipulation (low-quality evidence). (Grade: strong recommendation); **Recommendation 2:** For patients with chronic low back pain, clinicians and patients should initially select non-pharmacologic treatment with exercise, multidisciplinary rehabilitation, acupuncture, mindfulness-based stress reduction (moderate-quality evidence), (Grade: strong recommendation) [6].

3. Hip Osteoarthritis

3.1 Code: FA00

3.2 Description: Hip osteoarthritis is a degenerative hip joint disease that leads to progressive damage of articular cartilage, and structural changes underlying the subchondral bone that clinically manifest with overall changes in the (i) shape of the femoral head, (ii) loss of joint space, (iii) frequent severe pain, and loss of joint function.

3.3 Finding: Acupuncture is an effective conservative rehabilitation strategy to reduce pain and improve

quality of life in subjects with severe hip osteoarthritis [7].

4. Knee Osteoarthritis

4.1 Code: FA01

4.2 Description: Primary osteoarthritis occurring in an otherwise intact knee joint, involving genetically related, age-related or use-related degeneration with microscopic and macroscopic anatomical changes, which ultimately limit motion in one or more joints. Changes to the joint include increasing cartilage loss and osseous transformation such as sclerosis, osteophyte formation and cysts as well as potential inflammatory changes in surrounding soft tissue structures.

4.3.1 Finding: In this randomized clinical trial, self-administered acupressure with a brief knee health education program was efficacious and cost-effective in relieving knee pain and improving mobility in middle-aged and older adults with probable knee osteoarthritis [8].

4.3.2 Finding: The findings suggest that acupuncture may provide clinically important effects in reducing pain and improving physical function in patients with knee osteoarthritis [9].

4.3.3 Finding: Recommendations: We suggest acupuncture rather than no treatment in adult knee osteoarthritis (weak recommendation, moderate certainty evidence), and acupuncture combined with nonsteroidal anti-inflammatory drugs rather than acupuncture alone when knee osteoarthritis symptoms are severe (weak recommendation, moderate certainty evidence), with duration of acupuncture for 4-8 weeks depending on knee osteoarthritis severity and treatment response (weak recommendation, moderate certainty evidence), and discussing with patients in shared decision-making [10].

4.3.4 Finding: Among patients with knee osteoarthritis, intensive electroacupuncture resulted in less pain and better function at week 8, compared with sham acupuncture, and these effects persisted though week 26. Intensive manual acupuncture had no benefit for knee osteoarthritis at week 8, although it showed benefits during follow-up [11].

5. Rheumatoid Arthritis

5.1 Code: FA20

5.2 Description: Rheumatoid arthritis (RA) is persistent and/or erosive disease that is defined as the confirmed presence of synovitis in at least 1 joint, absence of an alternative diagnosis that better explains the synovitis, and achievement of a total score of 6 or greater (of a possible 10) from the individual scores in 4 domains: number and site of involved joints, serologic abnormality, elevated acute-phase response, and symptom duration.

5.3.1 Finding: This study found that acupuncture significantly improved symptoms of RA. Symptomatic improvement was not restricted to pain relief and was most significant for fatigue and anxiety [12].

5.3.2 Finding: Yoga, acupuncture, massage therapy and tai chi have been proved for their capability in RA treatment [13].

6. Tension-type Headache

6.1 Code: 8A81

6.2 Description: A primary and highly prevalent headache disorder, in most cases episodic. Attacks of highly variable frequency and duration are characterised by mild-to-moderate headache without associated symptoms, although pericranial tenderness may be present. In a minority of cases the disorder evolves, with increasingly frequent headache and sometimes loss of episodicity.

6.3.1 Finding: The 8-week true acupuncture treatment was effective for the prophylaxis of chronic tension-type headache [14].

6.3.2 Finding: Based on identified benefits, certainty of evidence, and patient preferences, manual joint mobilisation techniques, supervised physical activity, psychological treatment, acupuncture, and patient education can be considered as non-pharmacological treatment approaches for tension-type headache [15].

7. Migraine

7.1 Code: 8A80

7.2 Description: A primary headache disorder, in most cases episodic. Disabling attacks lasting 4-72 hours are characterised by moderate or severe headache, usually accompanied by nausea, vomiting and/or photophobia and phonophobia, and sometimes preceded by a short-lasting aura of unilateral fully-reversible visual, sensory

or other central nervous system symptoms. In a small minority of cases headache, but not necessarily the associated symptoms, becomes very frequent, with loss of episodicity.

7.3.1 Finding: Among patients with migraine without aura, true acupuncture may be associated with long-term reduction in migraine recurrence compared with sham acupuncture or assigned to a waiting list [16].

7.3.2 Finding: Twenty sessions of manual acupuncture was superior to sham acupuncture and usual care for the prophylaxis of episodic migraine without aura. These results support the use of manual acupuncture in patients who are reluctant to use prophylactic drugs or when prophylactic drugs are ineffective, and it should be considered in future guidelines [17].

8. Stroke

8.1 Code: 8B20

8.1.1 Description: Fulfills criteria for stroke in acute symptoms of focal brain injury that have lasted 24 hours or more (or led to death before 24 hours), but subtype of stroke (ischemic or haemorrhagic) has not been determined by neuroimaging or other techniques.

8.1.2.1 Finding: In this randomized clinical trial, patients with poststroke motor aphasia who received 6 weeks of manual acupuncture compared with those who received sham acupuncture demonstrated statistically significant improvements in language function, quality of life, and neurological impairment from week 6 of treatment to the end of follow-up at 6 months after onset [18].

8.1.2.2 Finding: The results suggested that acupuncture on points of the Governor vessel enhanced cognitive function in stroke survivors [19].

8.1.2.3 Finding: Positive recommendations on the use of acupuncture were identified for 15 symptom areas from 11 countries for: stroke rehabilitation, dysphagia, shoulder pain, motor recovery, walking, balance, spasticity, upper limb extremity impairment, post-stroke pain, central post stroke pain, cognitive disorder, depression, and sleep problems [20].

8.2 Code: 8B11 Cerebral Ischaemic Stroke

8.2.1 Description: Acute focal neurological dysfunction caused by focal infarction at single or multiple sites of the brain. Evidence of acute infarction may come either from a) symptom duration lasting more than 24 hours, or b) neuroimaging or other technique in the clinically relevant area of the brain. The term does not include infarction of the retina.

8.2.2.1 Finding: There was a tendency of fewer patients being dead or dependent in acupuncture group (80/385, 20.7%) than in control group (102/396, 25.8%) at 6 months (odds ratio, 0.75; 95% confidence interval, 0.54-1.05). The benefit was noted in subgroup receiving ≥10 sessions of acupuncture (odds ratio, 0.68; 95% confidence interval, 0.47-0.98) [21].

8.2.2.2 Finding: The results of this clinical trial showed a clinically relevant decrease of relapse in patients treated with resuscitating acupuncture intervention by the end of six months, compared with needling at the sham-acupoints. The resuscitating acupuncture intervention could also improve self-care ability and quality of life, evaluated with BI, NIHSS, CSS, Oxford Handicap Scale (OHS), and SS-QOL [22].

9. Alzheimer's disease and other dementias

9.1 Code: 6D80 Dementia due to Alzheimer disease

9.2 Description: Dementia due to Alzheimer disease is the most common form of dementia. Onset is insidious with memory impairment typically reported as the initial presenting complaint. The characteristic course is a slow but steady decline from a previous level of cognitive functioning with impairment in additional cognitive domains (such as executive functions, attention, language, social cognition and judgment, psychomotor speed, visuoperceptual or visuospatial abilities) emerging with disease progression. Dementia due to Alzheimer disease may be accompanied by mental and behavioural symptoms such as depressed mood and apathy in the initial stages of the disease and may be accompanied by psychotic symptoms, irritability, aggression, confusion, abnormalities of gait and mobility, and seizures at later stages. Positive genetic testing, family history and gradual cognitive decline are suggestive of Dementia due to Alzheimer disease.

9.3.1 Finding: Acupuncture is safe, well tolerated and effective in improving the cognitive function, global clinical status of AD [23].

9.3.2 Finding: Electro-acupuncture has demonstrated potential in improving mild-to-moderate dementia in

clinics [24].

10. Parkinson's Disease

10.1 Code: 8A00.0

10.2 Description: Parkinson Disease is a gradual onset progressive degenerative disease whose cardinal manifestations include bradykinesia plus one of the following-tremor, rigidity or postural instability. Nonmotor manifestations include autonomic dysfunction and neuropsychiatric features.

10.3.1 Finding: Electroacupuncture therapy can effectively improve skeletal muscle pain in patients with Parkinson's disease, reduce the muscle hardness of patients, improve patients' daily life ability, and improve patients' emotional disorders. The degree of skeletal muscle pain in PD patients is correlated with motor ability and emotional disorders, but there is no significant correlation between the degree of skeletal muscle pain and the muscle tone of PD patients [25].

10.3.2 Finding: In this randomized clinical trial, acupuncture proved beneficial in improving sleep quality and quality of life among patients with PD. These findings suggest that the therapeutic effects of acupuncture could continue for up to 4 weeks [26].

11. Upper Digestive System Diseases

11.1 Code: including DD90.3 Functional dyspepsia, DA22 Gastro-oesophageal reflux disease, DA42 Gastritis, DA61 Peptic ulcer, site unspecified, DA24 Oesophagitis.

11.2 Description: Functional dyspepsia is a disorder defined as the presence of dyspepsia symptoms thought to originate from the gastroduodenal region, in the absence of any organic, systemic, or metabolic disease that is likely to explain the symptoms such as epigastric pain, epigastric burning, postprandial fullness, and early satiation. Gastro-oesophageal reflux disease: A condition which develops when the reflux of stomach contents causes troublesome symptoms and/or complications.

11.3.1 Finding: Among patients with postprandial distress syndrome, acupuncture resulted in increased response rate and elimination rate of all 3 cardinal symptoms compared with sham acupuncture, with sustained efficacy over 12 weeks in patients who received thrice-weekly acupuncture for 4 weeks [27].

11.3.2 Finding: Adding acupuncture is more effective than doubling the proton pump inhibitor dose in controlling gastro-oesophageal reflux disease-related symptoms in patients who failed standard-dose proton pump inhibitors [28].

12. Inflammatory Bowel Disease

12.1 Code: including DD70 Crohn disease, DD71 Ulcerative colitis, DD72 Indeterminate colitis, DD7Y Other specified inflammatory bowel diseases, DD7Z Inflammatory bowel diseases, unspecified.

12.2 Description: Inflammatory bowel disease is a group of inflammatory conditions of the intestine of unknown aetiology. Regarding its pathogenesis, it is hypothesized that the mucosal immune system shows an aberrant response towards luminal antigens such as dietary factors and commensal microbiota in genetically susceptible individuals.

12.3.1 Finding: Moxibustion with acupuncture provided significant therapeutic benefits in patients with active Crohn's disease beyond the placebo effect and is therefore an effective and safe treatment for active Crohn's disease [29].

12.3.2 Finding: Acupuncture was effective in inducing and maintaining remission in patients with active Crohn's disease, which was associated with increased abundance of intestinal anti-inflammatory bacteria, enhanced intestinal barrier, and regulation of circulating Th1/Th17-related cytokines [30].

13. Pancreatitis

13.1 Code: DC31 Acute pancreatitis, DC32 Chronic pancreatitis

13.2 Description: Inflammation of the pancreas with sudden onset. Pathological changes range from oedema to necrosis. While mild cases often recover without complications, severe cases have high mortality due to systemic complications despite intensive treatment.

13.3 Finding: The study presents proof-of-concept for the analgesic effect of acupuncture in pancreatic pain. Although the effect was short lasting, the framework may be used to conceptualize future trials of acupuncture in visceral pain [31].

14. Male Infertility
14.1 Code: GB04
14.2 Description: Any disorder of the reproductive system affecting males, characterised by dysfunctionalities in the ejection of semen or an abnormal absence in the measurable level of sperm in semen.
14.3.1 Finding: The treatment of idiopathic male infertility could benefit from employing acupuncture. A general improvement of sperm quality, specifically in the ultrastructural integrity of spermatozoa, was seen after acupuncture, although we did not identify specific sperm pathologies that could be particularly sensitive to this therapy [32].
14.3.2 Finding: Acupuncture treatment in primary infertile varicocele patients with semen abnormalities seems to be effective and has comparable results with the varicocelectomy treatment [33].
14.3.3 Finding: We conclude that a functional down-regulation of CatSper channel in the sperm may be a contributor or a downstream indicator for a portion of asthenozoospermia (AZS), especially idiopathic asthenozoospermia (iAZS), while 2 Hz-transcutaneous electrical acupoint stimulation or electroacupuncture treatment has a therapeutic effect on iAZS through inducing the functional up-regulation of CatSper channels in the sperm. This study provides a novel mechanism for the pathogenesis of some AZS especially iAZS, and presents a potential therapeutic target of CatSper for iAZS treatment. Acupuncture treatment like TEAS may be used as a promising complementary and alternative medicine therapy for male infertility caused by iAZS in clinical practice [34].

15. Female infertility
15.1 Code: GA31
15.2 Description: Disease of the reproductive system defined by the failure to achieve a clinical pregnancy after 12 months or more of regular unprotected sexual intercourse.
15.3.1 Finding: This study finds a benefit of acupuncture for outcomes in women with infertility, and the number of acupuncture treatments is a potential influential factor [35].
15.3.2 Finding: Transcutaneous electrical acupoint stimulation significantly improved the clinical pregnancy rate in women undergoing IVF-ET, especially in women of older age. It might be due to improved endometrial receptivity [36].

16. Depressive Disorders
16.1 Code: 6A70 Single episode depressive disorder; Code: 6A71 Recurrent depressive disorder
16.2 Description: Depressive disorders are characterised by depressive mood (e.g., sad, irritable, empty) or loss of pleasure accompanied by other cognitive, behavioural, or neurovegetative symptoms that significantly affect the individual's ability to function. A depressive disorder should not be diagnosed in individuals who have ever experienced a manic, mixed or hypomanic episode, which would indicate the presence of a bipolar disorder.
16.3.1 Finding: The results of this randomized clinical trial suggest that specific auricular acupuncture over 6 weeks is safe. Although there was no statistically significant difference between groups for the primary efficacy outcome, patients receiving specific auricular acupuncture did experience greater symptom remission at 3 months [37].
16.3.2 Finding: Intradermal acupuncture as adjunctive therapy provides clinical efficacy and safety for major depressive disorder, and it may exert antidepressant effects by modulating striatal functional connectivity [38].

17. Anxiety Disorders
17.1 Code: including 6B00 Generalised anxiety disorder, 6B01 Panic disorder, 6B02 Agoraphobia, 6B03 Specific phobia, 6B04 Social anxiety disorder, 6B05 Separation anxiety disorder, 6B06 Selective mutism
17.2 Description: Anxiety and fear-related disorders are characterised by excessive fear and anxiety and related behavioural disturbances, with symptoms that are severe enough to result in significant distress or significant impairment in personal, family, social, educational, occupational, or other important areas of functioning. Fear and anxiety are closely related phenomena; fear represents a reaction to perceived imminent threat in the present, whereas anxiety is more future-oriented, referring to perceived anticipated threat. A key differentiating feature among the Anxiety and fear-related disorders are disorder-specific foci of apprehension, that is, the stimulus or situation that triggers the fear or anxiety. The clinical presentation of Anxiety and fear-related disorders typically includes specific associated cognitions that can assist in differentiating among

the disorders by clarifying the focus of apprehension.

17.3.1 Finding: This study found acupuncture to be an effective treatment for anxiety in patients with PD. These findings suggest that acupuncture may enhance the wellbeing of patients who have Parkinson disease and anxiety [39].

17.3.2 Finding: This study finds that a volume of literature, consistency of statistically significant results, wide range of conditions treated and use of animal test subjects suggests very real, positive outcomes using a treatment method preferred by a population of individuals who tend to be resistant to conventional medicine [40].

18. Opioid Use Disorders

18.1 Code: 6C43

18.2 Description: Disorders due to use of opioids are characterised by the pattern and consequences of opioid use. Opioids is a generic term that encompasses the constituents or derivatives of the opium poppy Papaver somniferum as well as a range of synthetic and semisynthetic compounds, some related to morphine and others chemically distinct but all having their primary actions on the μ opioid receptor. Examples of opioids include morphine, diacetylmorphine (heroin), fentanyl, pethidine, oxycodone, hydromorphone, methadone, buprenorphine, codeine and d-propoxyphene. The opioids all have analgesic properties of different potencies and are primarily central nervous system depressants. They suppress respiration as well as other vital functions and are a common cause of overdose and related deaths. Certain opioids are used or administered parenterally, including heroin, a common and potent opioid that is primarily used non-medically. Therapeutic opioids are prescribed for a range of indications worldwide, and are essential for pain management in cancer pain and palliative care, although they are also used for non-therapeutic reasons. In some countries morbidity and mortality related to therapeutic opioids is greater than that related to heroin. All opioids may result in Opioid Intoxication, Opioid Dependence and Opioid Withdrawal. A range of Opioid-Induced Disorders occur, some of which occur following Opioid Withdrawal.

18.3 Finding: Eight weeks of acupuncture were superior to sham acupuncture in reducing methadone dose and decreasing opioid craving [41].

19. Herpes Zoster

19.1 Code: 1E91

19.2 Description: A disease caused by the reactivation of a latent infection with varicella zoster virus. This disease commonly presents with a rash (typically within one or two adjacent dermatomes), cutaneous hyperaesthesia, or fever.

19.3.1 Finding: Wrist-ankle acupuncture combined with standard pharmacological treatment may potentially improve the pain cure rate at 7 days post-treatment. This suggests a potential new strategy for alleviating pain in patients in the acute phase of herpes zoster [42].

19.3.2 Finding: Acupuncture therapy could accelerate healing and reduce pain and incidence of post-herpetic neuralgia in vesicular phase. In post-herpetic neuralgia phase, it can reduce pain and discomfort in most patients and remove pain and discomfort in some patients [43].

20. Neoplasms

20.1 Code: MG30.1 Chronic cancer related pain,

20.2 Description: Chronic cancer-related pain is pain caused by the primary cancer itself or metastases (chronic cancer pain) or its treatment (chronic post-cancer treatment pain). It is distinct from pain caused by co-morbid disease. It should be highly probable that the pain is due to cancer or its treatment; if its genesis is vague, consider using codes in the section of Primary pain.

20.3 Finding: Evidence from SRs showed that acupuncture is beneficial to cancer survivors with cancer-related pain, fatigue, insomnia, improved quality of life, nausea and vomiting, bone marrow suppression, menopausal symptoms, arthralgia, and dysphagia, and may also be potential for lymphoedema, gastrointestinal function, and xerostomia. For neuropathy, depression and anxiety, acupuncture should be used as an option based on individual conditions. Acupuncture is relatively safe without serious adverse events [44].

Reference:

[1] Zhao L, Sun M, Yin Z, Cui J, Wang R, Ji L, Geng G, Chen J, Cai D, Liu Q, Zheng H, Liang F. Long-Term Effects of Individualized Acupuncture for Chronic Neck Pain: A Randomized Controlled Trial. Ann Intern Med. 2024;177(10):1330-1338. doi: 10.7326/M23-2425.
[2] Chen L, Li M, Fan L, Zhu X, Liu J, Li H, Xu Z, Chen J, Liang Z, Liu Z, Feng L, Chen X, He Q, Chen X, Ou A, He J, Ma R, Ning B, Jiang L, Li S, Fu W. Optimized acupuncture treatment (acupuncture and intradermal needling) for cervical spondylosis-related neck pain: a multicenter randomized controlled trial. Pain. 2021;162(3):728-739. doi: 10.1097/j.pain.0000000000002071.
[3] MacPherson H, Tilbrook H, Richmond S, Woodman J, Ballard K, Atkin K, Bland M, Eldred J, Essex H, Hewitt C, Hopton A, Keding A, Lansdown H, Parrott S, Torgerson D, Wenham A, Watt I. Alexander Technique Lessons or Acupuncture Sessions for Persons With Chronic Neck Pain: A Randomized Trial. Ann Intern Med. 2015;163(9):653-62. doi: 10.7326/M15-0667.
[4] Cho YJ, Song YK, Cha YY, Shin BC, Shin IH, Park HJ, Lee HS, Kim KW, Cho JH, Chung WS, Lee JH, Song MY. Acupuncture for chronic low back pain: a multicenter, randomized, patient-assessor blind, sham-controlled clinical trial. Spine (Phila Pa 1976). 2013;38(7):549-57. doi: 10.1097/BRS.0b013e318275e601.
[5] Rizzo RR, Cashin AG, Wand BM, Ferraro MC, Sharma S, Lee H, O'Hagan E, Maher CG, Furlan AD, van Tulder MW, McAuley JH. Non-pharmacological and non-surgical treatments for low back pain in adults: an overview of Cochrane reviews. Cochrane Database Syst Rev. 2025;3(3):CD014691. doi: 10.1002/14651858.CD014691.
[6] Qaseem A, Wilt TJ, McLean RM, Forciea MA; Clinical Guidelines Committee of the American College of Physicians; Denberg TD, Barry MJ, Boyd C, Chow RD, Fitterman N, Harris RP, Humphrey LL, Vijan S. Noninvasive Treatments for Acute, Subacute, and Chronic Low Back Pain: A Clinical Practice Guideline From the American College of Physicians. Ann Intern Med. 2017;166(7):514-530. doi: 10.7326/M16-2367.
[7] Astini R, Riberto M. Acupuncture in the Treatment of a Series of Patients with Chronic Pain Associated with Hip Osteoarthritis. Rev Bras Ortop (Sao Paulo). 2023;58(5):e750-e754. doi: 10.1055/s-0043-1776134.
[8] Yeung WF, Chen SC, Cheung DST, Wong CK, Chong TC, Ho YS, Suen LKP, Ho LM, Lao L. Self-Administered Acupressure for Probable Knee Osteoarthritis in Middle-Aged and Older Adults: A Randomized Clinical Trial. JAMA Netw Open. 2024;7(4):e245830. doi: 10.1001/jamanetworkopen.2024.5830.
[9] Liu CY, Duan YS, Zhou H, Wang Y, Tu JF, Bao XY, Yang JW, Lee MS, Wang LQ. Clinical effect and contributing factors of acupuncture for knee osteoarthritis: a systematic review and pairwise and exploratory network meta-analysis. BMJ Evid Based Med. 2024;29(6):374-384. doi: 10.1136/bmjebm-2023-112626.
[10] Luo X, Liu J, Li Q, Zhao J, Hao Q, Zhao L, Chen Y, Yin P, Li L, Liang F, Sun X. Acupuncture for treatment of knee osteoarthritis: A clinical practice guideline. J Evid Based Med. 2023;16(2):237-245. doi: 10.1111/jebm.12526.
[11] Tu JF, Yang JW, Shi GX, Yu ZS, Li JL, Lin LL, Du YZ, Yu XG, Hu H, Liu ZS, Jia CS, Wang LQ, Zhao JJ, Wang J, Wang T, Wang Y, Wang TQ, Zhang N, Zou X, Wang Y, Shao JK, Liu CZ. Efficacy of Intensive Acupuncture Versus Sham Acupuncture in Knee Osteoarthritis: A Randomized Controlled Trial. Arthritis Rheumatol. 2021;73(3):448-458. doi: 10.1002/art.41584.
[12] Mishra, Pragnyaban & Trivedi, Vikas. THU0136 Acupuncture in the Treatment of Rheumatoid Arthritis: Results of A Randomized Controlled Trial. Annals of the Rheumatic Diseases. 2014;73. 226-226. doi: 10.1136/annrheumdis-2014-eular.2199.
[13] Kumar LD, Karthik R, Gayathri N, Sivasudha T. Advancement in contemporary diagnostic and therapeutic approaches for rheumatoid arthritis. Biomed Pharmacother. 2016;79:52-61. doi: 10.1016/j.biopha.2016.02.001.
[14] Zheng H, Gao T, Zheng QH, Lu LY, Hou TH, Zhang SS, Zhou SY, Hao XY, Wang L, Zhao L, Liang FR, Li Y. Acupuncture for Patients With Chronic Tension-Type Headache: A Randomized Controlled Trial. Neurology. 2022;99(14):e1560-e1569. doi: 10.1212/WNL.0000000000200670.
[15] Krøll LS, Callesen HE, Carlsen LN, Birkefoss K, Beier D, Christensen HW, Jensen M, Tómasdóttir H, Würtzen H, Høst CV, Hansen JM. Manual joint mobilisation techniques, supervised physical activity, psychological treatment, acupuncture and patient education for patients with tension-type headache. A

systematic review and meta-analysis. J Headache Pain. 2021;22(1):96. doi: 10.1186/s10194-021-01298-4.
[16] Zhao L, Chen J, Li Y, Sun X, Chang X, Zheng H, Gong B, Huang Y, Yang M, Wu X, Li X, Liang F. The Long-term Effect of Acupuncture for Migraine Prophylaxis: A Randomized Clinical Trial. JAMA Intern Med. 2017;177(4):508-515. doi: 10.1001/jamainternmed.2016.9378.
[17] Xu S, Yu L, Luo X, Wang M, Chen G, Zhang Q, Liu W, Zhou Z, Song J, Jing H, Huang G, Liang F, Wang H, Wang W. Manual acupuncture versus sham acupuncture and usual care for prophylaxis of episodic migraine without aura: multicentre, randomised clinical trial. BMJ. 2020;368:m697. doi: 10.1136/bmj.m697.
[18] Li B, Deng S, Zhuo B, Sang B, Chen J, Zhang M, Tian G, Zhang L, Du Y, Zheng P, Yue G, Meng Z. Effect of Acupuncture vs Sham Acupuncture on Patients With Poststroke Motor Aphasia: A Randomized Clinical Trial. JAMA Netw Open. 2024;7(1):e2352580. doi: 10.1001/jamanetworkopen.2023.52580.
[19] Han JZ, Yang Y, Wang YF, Feng JH, Song CN, Wu WJ, Lin HB. Effectiveness and safety of Governor vessel acupuncture therapy for post-stroke cognitive impairment: A meta-analysis of randomized controlled trials. Ageing Res Rev. 2024;99:102355. doi: 10.1016/j.arr.2024.102355.
[20] Birch S, Robinson N. Acupuncture as a post-stroke treatment option: A narrative review of clinical guideline recommendations. Phytomedicine. 2022;104:154297. doi: 10.1016/j.phymed.2022.154297.
[21] Zhang S, Wu B, Liu M, Li N, Zeng X, Liu H, Yang Q, Han Z, Rao P, Wang D; all Investigators. Acupuncture efficacy on ischemic stroke recovery: multicenter randomized controlled trial in China. Stroke. 2015;46(5):1301-6. doi: 10.1161/STROKEAHA.114.007659.
[22] Shen PF, Kong L, Ni LW, Guo HL, Yang S, Zhang LL, Zhang ZL, Guo JK, Xiong J, Zhen Z, Shi XM. Acupuncture intervention in ischemic stroke: a randomized controlled prospective study. Am J Chin Med;40(4):685-93. doi: 10.1142/S0192415X12500516.
[23] Jia Y, Zhang X, Yu J, Han J, Yu T, Shi J, Zhao L, Nie K. Acupuncture for patients with mild to moderate Alzheimer's disease: a randomized controlled trial. BMC Complement Altern Med. 2017;17(1):556. doi: 10.1186/s12906-017-2064-x.
[24] Wang L, Bi L, Qiu Y, Huang G, Ye P, Liu Y, Li A, Yang X, Shen P, Wang J, Zeng Q, Zhang H, Li S, Jin H. Effectiveness of electro-acupuncture for cognitive improvement on Alzheimer's disease quantified via PET imaging of sphingosine-1-phosphate receptor 1. Alzheimers Dement. 2024;20(12):8331-8345. doi: 10.1002/alz.14260.
[25] Shaosong W, Jingqing S, Qingyin F, Bin LI, Xin W, Fan Y, Yingxue C. Effectivenss of electroacupuncture for skeletal muscle pain in Parkinson's disease: a Clinical randomized controlled trial. J Tradit Chin Med. 2024;44(2):388-395. doi: 10.19852/j.cnki.jtcm.20240203.004.
[26] Yan M, Fan J, Liu X, Li Y, Wang Y, Tan W, Chen Y, He J, Zhuang L. Acupuncture and Sleep Quality Among Patients With Parkinson Disease: A Randomized Clinical Trial. JAMA Netw Open. 2024;7(6):e2417862. doi: 10.1001/jamanetworkopen.2024.17862.
[27] Yang JW, Wang LQ, Zou X, Yan SY, Wang Y, Zhao JJ, Tu JF, Wang J, Shi GX, Hu H, Zhou W, Du Y, Liu CZ. Effect of Acupuncture for Postprandial Distress Syndrome: A Randomized Clinical Trial. Ann Intern Med. 2020;172(12):777-785. doi: 10.7326/M19-2880.
[28] Dickman R, Schiff E, Holland A, Wright C, Sarela SR, Han B, Fass R. Clinical trial: acupuncture vs. doubling the proton pump inhibitor dose in refractory heartburn. Aliment Pharmacol Ther. 2007;26(10):1333-44. doi: 10.1111/j.1365-2036.2007.03520.x.
[29] Bao CH, Zhao JM, Liu HR, Lu Y, Zhu YF, Shi Y, Weng ZJ, Feng H, Guan X, Li J, Chen WF, Wu LY, Jin XM, Dou CZ, Wu HG. Randomized controlled trial: moxibustion and acupuncture for the treatment of Crohn's disease. World J Gastroenterol. 2014;20(31):11000-11. doi: 10.3748/wjg.v20.i31.11000.
[30] Bao C, Wu L, Wang D, Chen L, Jin X, Shi Y, Li G, Zhang J, Zeng X, Chen J, Liu H, Wu H. Acupuncture improves the symptoms, intestinal microbiota, and inflammation of patients with mild to moderate Crohn's disease: A randomized controlled trial. EClinicalMedicine. 2022;45:101300. doi: 10.1016/j.eclinm.2022.101300.
[31] Juel J, Liguori S, Liguori A, Poulsen JL, Valeriani M, Graversen C, Olesen SS, Drewes AM. Acupuncture for Pain in Chronic Pancreatitis: A Single-Blinded Randomized Crossover Trial. Pancreas. 2017;46(2):170-176. doi: 10.1097/MPA.0000000000000749.
[32] Pei J, Strehler E, Noss U, Abt M, Piomboni P, Baccetti B, Sterzik K. Quantitative evaluation of spermatozoa ultrastructure after acupuncture treatment for idiopathic male infertility. Fertil Steril.

2005;84(1):141-7. doi: 10.1016/j.fertnstert.2004.12.056.
[33] Kucuk EV, Bindayi A, Boylu U, Onol FF, Gumus E. Randomised clinical trial of comparing effects of acupuncture and varicocelectomy on sperm parameters in infertile varicocele patients. Andrologia. 2016;48(10):1080-1085. doi: 10.1111/and.12541.
[34] Jin ZR, Fang D, Liu BH, Cai J, Tang WH, Jiang H, Xing GG. Roles of CatSper channels in the pathogenesis of asthenozoospermia and the therapeutic effects of acupuncture-like treatment on asthenozoospermia. Theranostics. 2021;11(6):2822-2844. doi: 10.7150/thno.51869.
[35] Quan K, Yu C, Wen X, Lin Q, Wang N, Ma H. Acupuncture as Treatment for Female Infertility: A Systematic Review and Meta-Analysis of Randomized Controlled Trials. Evid Based Complement Alternat Med. 2022;2022:3595033. doi: 10.1155/2022/3595033.
[36] Feng X, Zhu N, Yang S, Wang L, Sun W, Li R, Gong F, Han S, Zhang R, Han J. Transcutaneous electrical acupoint stimulation improves endometrial receptivity resulting in improved IVF-ET pregnancy outcomes in older women: a multicenter, randomized, controlled clinical trial. Reprod Biol Endocrinol. 2022;20(1):127. doi: 10.1186/s12958-022-00997-0.
[37] de Oliveira Rodrigues DM, Menezes PR, Machado Ribeiro Silotto AE, Heps A, Pereira Sanches NM, Schveitzer MC, Faisal-Cury A. Efficacy and Safety of Auricular Acupuncture for Depression: A Randomized Clinical Trial. JAMA Netw Open. 2023;6(11):e2345138. doi: 10.1001/jamanetworkopen.2023.45138.
[38] Wu X, Tu M, Yu Z, Cao Z, Qu S, Chen N, Jin J, Xiong S, Yang J, Pei S, Xu M, Wang J, Shi Y, Gao L, Xie J, Li X, Fang J, Shao X. The efficacy and cerebral mechanism of intradermal acupuncture for major depressive disorder: a multicenter randomized controlled trial. Neuropsychopharmacology. 2025;50(7):1075-1083. doi: 10.1038/s41386-024-02036-5.
[39] Fan JQ, Lu WJ, Tan WQ, Liu X, Wang YT, Wang NB, Zhuang LX. Effectiveness of Acupuncture for Anxiety Among Patients With Parkinson Disease: A Randomized Clinical Trial. JAMA Netw Open. 2022;5(9):e2232133. doi: 10.1001/jamanetworkopen.2022.32133.
[40] Errington-Evans N. Acupuncture for anxiety. CNS Neurosci Ther. 2012;18(4):277-84. doi: 10.1111/j.1755-5949.2011.00254.x.
[41] Lu L, Chen C, Chen Y, Dong Y, Chen R, Wei X, Tao C, Li C, Wang Y, Fan B, Tang X, Xu S, He Z, Mo G, Liu Y, Gu H, Li X, Cao F, Xu H, Zhang Y, Li G, Liu X, Zeng J, Tang C, Xu N. Effect of Acupuncture for Methadone Reduction : A Randomized Clinical Trial. Ann Intern Med. 2024;177(8):1039-1047. doi: 10.7326/M23-2721.
[42] Pu J, Li D, Luo X, Wang J, Li Y, Lei L, Zhao X, Du H, Yang X, Du X. Wrist-ankle acupuncture alleviates pain in the acute phase of herpes zoster: A randomized controlled trial. PLoS One. 2025;20(5):e0318386. doi: 10.1371/journal.pone.0318386.
[43] Liu ZS, Peng WN, Liu BY, Wang J, Wang Y, Mao M, Deng YH, Yu JN, Liaw Y, Mu Y, Luo Y, Xiao XL, Wu XD, Zi MJ. Clinical practice guideline of acupuncture for herpes zoster. Chin J Integr Med. 2013;19(1):58-67. doi: 10.1007/s11655-013-1191-y.
[44] Zhang XW, Hou WB, Pu FL, Wang XF, Wang YR, Yang M, Cheng K, Wang Y, Robinson N, Liu JP. Acupuncture for cancer-related conditions: An overview of systematic reviews. Phytomedicine. 2022;106:154430. doi: 10.1016/j.phymed.2022.154430.

Table S1. Estimated annual percentage changes of prevalence and years of life lived with disability for health conditions in need of acupuncture in 1990 and 2021 in the worldwide.

Disease type	EAPC (95%CI)	
	Prevalence	Years of life lived with disability
**Total**	0.02 (-0.01, 0.04)	-0.04 (-0.09, 0)
**Musculoskeletal disorders**	-0.07 (-0.1, -0.04)	-0.18 (-0.21, -0.14)
Neck pain	-0.05 (-0.12, 0.03)	-0.04 (-0.12, 0.03)
Low back pain	-0.32 (-0.35, -0.28)	-0.32 (-0.35, -0.28)
Hip osteoarthritis	0.26 (0.24, 0.28)	0.26 (0.24, 0.28)
Knee osteoarthritis	0.33 (0.3, 0.35)	0.33 (0.3, 0.36)
Rheumatoid arthritis	0.53 (0.5, 0.57)	0.53 (0.49, 0.56)
**Neurological disorders**	0 (-0.01, 0.02)	-0.01 (-0.02, -0.01)
Tension-type headache	-0.02 (-0.03, 0)	-0.08 (-0.09, -0.07)
Migraine	0.06 (0.05, 0.07)	0.05 (0.04, 0.07)
Stroke	-0.37 (-0.4, -0.35)	-0.33 (-0.35, -0.3)
Alzheimer's disease and other dementias	0 (-0.02, 0.03)	-0.01 (-0.03, 0.01)
Parkinson's disease	1.52 (1.49, 1.54)	1.52 (1.49, 1.54)
**Digestive disorders**	-0.74 (-0.81, -0.66)	-0.47 (-0.55, -0.39)
Upper digestive system diseases	0.04 (0, 0.07)	-0.14 (-0.16, -0.11)
Inflammatory bowel disease	-0.13 (-0.25, 0)	-0.12 (-0.24, 0)
Pancreatitis	-1.09 (-1.15, -1.03)	-0.97 (-1.01, -0.92)
**Genecological disorders**	0.64 (0.48, 0.79)	0.65 (0.49, 0.8)
Male infertility	0.48 (0.34, 0.63)	0.5 (0.36, 0.64)
Female infertility	0.71 (0.55, 0.88)	0.73 (0.56, 0.89)
**Mental disorders**	0.05 (-0.07, 0.17)	0.03 (-0.1, 0.17)
Depressive disorders	-0.03 (-0.15, 0.08)	-0.04 (-0.18, 0.1)
Anxiety disorders	0.13 (-0.01, 0.27)	0.14 (0, 0.27)
**Substance use disorders**		
Opioid use disorders	0.5 (0.33, 0.68)	0.49 (0.32, 0.66)
**Infectious disorders**		
Varicella and herpes zoster	0.05 (0.04, 0.07)	0.06 (0.04, 0.09)
**Neoplasms**	0.25 (0.21, 0.29)	0.17 (0.11, 0.22)

Notes: CI: confidence interval; EAPCs: estimated annual percentage changes.

Table S2. Estimated annual percentage changes of prevalence and years of life lived with disability for health conditions in need of acupuncture in 1990 and 2021 in China.

Disease type	EAPC (95%CI)	
	Prevalence	Years of life lived with disability
**Total**	0.1 (0.07, 0.14)	-0.13 (-0.17, -0.1)
**Musculoskeletal disorders**	0.02 (-0.05, 0.09)	-0.17 (-0.24, -0.1)
Neck pain	0.13 (0.1, 0.16)	0.13 (0.1, 0.16)
Low back pain	-0.5 (-0.6, -0.39)	-0.49 (-0.6, -0.39)
Hip osteoarthritis	0.95 (0.86, 1.03)	0.93 (0.85, 1.02)
Knee osteoarthritis	0.5 (0.39, 0.61)	0.5 (0.39, 0.62)
Rheumatoid arthritis	0.59 (0.55, 0.62)	0.6 (0.56, 0.63)
**Neurological disorders**	0.3 (0.25, 0.35)	0.38 (0.35, 0.4)
Tension-type headache	0.27 (0.22, 0.33)	0.09 (0.04, 0.13)
Migraine	0.28 (0.24, 0.33)	0.27 (0.23, 0.32)
Stroke	0.34 (0.31, 0.38)	0.32 (0.29, 0.36)
Alzheimer's disease and other dementias	0.44 (0.36, 0.52)	0.42 (0.33, 0.5)
Parkinson's disease	3.16 (3.03, 3.29)	3.14 (3, 3.28)
**Digestive disorders**	-0.48 (-0.85, -0.11)	0.08 (-0.24, 0.4)
Upper digestive system diseases	-0.34 (-0.46, -0.21)	-1.02 (-1.14, -0.89)
Inflammatory bowel disease	2.54 (1.91, 3.17)	2.46 (1.84, 3.09)
Pancreatitis	-1.36 (-1.84, -0.87)	-1.36 (-1.78, -0.94)
**Genecological disorders**	0.03 (-0.02, 0.08)	0.05 (-0.01, 0.1)
Male infertility	-0.04 (-0.09, 0.01)	-0.01 (-0.08, 0.05)
Female infertility	0.06 (0.01, 0.11)	0.07 (0.01, 0.13)
**Mental disorders**	-0.41 (-0.49, -0.33)	-0.45 (-0.53, -0.37)
Depressive disorders	-0.44 (-0.5, -0.38)	-0.53 (-0.62, -0.43)
Anxiety disorders	-0.39 (-0.51, -0.26)	-0.37 (-0.49, -0.24)
**Substance use disorders**		
Opioid use disorders	-3.31 (-3.69, -2.94)	-3.3 (-3.67, -2.92)
**Infectious disorders**		
Varicella and herpes zoster	-0.08 (-0.15, 0)	-0.09 (-0.19, 0.01)
**Neoplasms**	0.88 (0.84, 0.92)	1.16 (1.1, 1.22)

Notes: CI: confidence interval; EAPCs: estimated annual percentage changes.

Table S3. Acupuncture demands based on prevalence and YLDs and its temporal trends from 1990, 2021 by gender, and age in the worldwide.

	1990		2021		EAPC 95%CI
	Number	ASR, per 100,000 persons	Number	ASR, per 100,000 persons	
**Prevalence**					
**Gender**					
Female	2192906116.85 (1893062292.62, 2542041272.8)	88454.09 (76649.07, 101716.42)	3739194831.64 (3237651520.1, 4316004491.32)	89690.25 (77558.77, 103699.61)	0.01 (-0.02, 0.03)
Male	1617846515.53 (1394486946.11, 1867999133.77)	66233.58 (57613.75, 75560.53)	2763963257.4 (2395105203.27, 3168694229.35)	67699.41 (58803.56, 77519.74)	0.04 (0.02, 0.06)
**Age**					
<5 years	4157624.93 (3313422.79, 5187896.29)	670.65 (534.48, 836.84)	4149466.52 (3275925.1, 5239008.74)	630.45 (497.73, 795.99)	-0.17 (-0.26, -0.08)
5-9 years	71560296.47 (45450419.62, 103869464.64)	12263.33 (7788.86, 17800.17)	85396172.09 (54193341.95, 124256691.58)	12429.33 (7887.79, 18085.44)	0.02 (-0.01, 0.04)
10-14 years	240256567.07 (174775408.81, 318231264.13)	44850.54 (32626.67, 59406.68)	304994064.02 (221194515.64, 407085041.35)	45751.22 (33180.71, 61065.57)	0.04 (0.02, 0.06)
15-19 years	323512839.41 (224820564.22, 431722125.01)	62283.06 (43282.71, 83115.64)	405761965.61 (282467840.8, 543286832.5)	65028.05 (45268.74, 87068.01)	0.06 (0.03, 0.09)
20-24 years	375213544 (271451177.98, 509897102.01)	76249.29 (55163.15, 103619.11)	485272938.79 (349284058.52, 665407915.85)	81263.72 (58491.05, 111429.1)	0.11 (0.07, 0.16)
25-29 years	387224500.88 (281122657.74, 526739900.64)	87484.71 (63513.37, 119005.09)	539008965.6 (387077586.6, 738756634.7)	91614.72 (65791.12, 125565.59)	0.12 (0.08, 0.17)
30-34 years	377512310.65 (268825427.34, 513021900.78)	97947.66 (69748.25, 133106.37)	602217714.23 (426134867.7, 829032297.81)	99625.72 (70496.09, 137147.97)	0.05 (0, 0.11)
35-39 years	366168093.47 (257808031.67, 498269686.49)	103952.83 (73190.09, 141455.64)	597053061.35 (416777062.03, 818854340.67)	106451.98 (74309.55, 145998.19)	0.03 (-0.02, 0.09)
40-44 years	311949235.75 (226032833.24, 425342270.73)	108889.78 (78899.59, 148471.04)	553408977.73 (399176050.35, 755874722.56)	110626.31 (79795.19, 151099.15)	-0.01 (-0.06, 0.03)
45-49 years	248659503.92 (185708670.3, 322581439.32)	107090.58 (79979.45, 138926.66)	507332147.24 (378570459.76, 659506712.06)	107144.07 (79950.74, 139282)	-0.05 (-0.07, -0.02)
50-54 years	237160862.88 (176487932.23, 310115384.6)	111567.59 (83025.22, 145887.59)	492232742.39 (367646409.86, 644069047.37)	110633.25 (82631.47, 144759.67)	-0.05 (-0.06, -0.03)
55-59 years	218397370.63 (165037795.4, 284306248.62)	117925.17 (89113.3, 153513.13)	465756043.48 (353871465.67, 605304694.97)	117696 (89422.9, 152959.78)	-0.01 (-0.02, 0)
60-64 years	194857209.39 (147665496.76, 254035251.61)	121323.79 (91940.85, 158169.76)	389396814.92 (296349950.24, 506180456.58)	121668.31 (92595.51, 158157.74)	-0.01 (-0.03, 0.01)
65-69 years	161508681.11 (124448772.24, 208837302.17)	130660.46 (100679.01, 168949.29)	357038465.8 (277199217.08, 459063241.77)	129435.79 (100491.97, 166422.44)	-0.04 (-0.05, -0.02)
70-74 years	118283972.58 (91464894.08, 152515558.38)	139714.58 (108036.44, 180148.21)	286832251.71 (222506782.49, 367313924.18)	139347.72 (108097.37, 178447.01)	-0.04 (-0.06, -0.02)
75-79 years	89832645.91 (68545459.66, 114919507.59)	145937.61 (111355.51, 186692.47)	189559033.8 (145301835.95, 241601541.78)	143731.2 (110173.63, 183191.9)	-0.04 (-0.06, -0.03)
80-84 years	53193526.89 (41198081.67, 67989571.95)	150366.45 (116457.96, 192191.63)	131312440.66 (102185994.08, 167234370.88)	149929.04 (116673.24, 190943.75)	-0.02 (-0.04, -0.01)
85-89 years	23107865.86 (17732353.06, 29680993.04)	152920.09 (117346.75, 196418.84)	70125743.57 (54049741, 89663233.72)	153374.88 (118214.4, 196106.13)	0.01 (0, 0.01)

90-94 years	6533595.1 (5010091.01, 8365959.6)	152469.29 (116916.49, 195229.72)	27442972.37 (21147573.62, 35057540.12)	153403.76 (118213.05, 195968.52)	0.03 (0.02, 0.03)
95 plus years	1662385.47 (1239944.48, 2165426.2)	163285.29 (121791.66, 212695.7)	8866107.17 (6652563.54, 11539738.98)	162671.65 (122058.47, 211726.34)	0 (-0.01, 0.01)
**YLDs**					
**Gender**					
Female	103376572.94 (61154395.55, 160293419.82)	4274.12 (2556.84, 6573.37)	184870186.09 (111322192.68, 282547212.57)	4373.89 (2614.81, 6711.89)	-0.3 (-0.41, -0.18)
Male	67045593.77 (40067136.46, 104718907.86)	2894.79 (1762.27, 4440.77)	121598500.39 (73859581.73, 186859284.68)	2986.98 (1815.01, 4583.04)	0.11 (0.02, 0.2)
**Age**					
<5 years	207450.73 (128502.42, 309087.7)	33.46 (20.73, 49.86)	210040.12 (126345.31, 322954.21)	31.91 (19.2, 49.07)	0.13 (0.06, 0.2)
5-9 years	2106712.59 (911022.31, 4118365.06)	361.03 (156.12, 705.77)	2765786.61 (1215809.43, 5311391.47)	402.56 (176.96, 773.07)	0.07 (-0.01, 0.15)
10-14 years	7446519.57 (3126236.32, 14296584.32)	1390.1 (583.6, 2668.85)	10247583.9 (4430637.25, 19363503.93)	1537.21 (664.63, 2904.66)	0.01 (-0.09, 0.11)
15-19 years	12395703.41 (5790322.61, 22423793.86)	2386.44 (1114.76, 4317.05)	16449599.05 (7809857.91, 29589479.19)	2636.24 (1251.62, 4742.06)	0 (-0.09, 0.09)
20-24 years	15139783.46 (7410543.76, 26857104.57)	3076.64 (1505.94, 5457.79)	20067566.62 (9937171.44, 35247305.35)	3360.51 (1664.08, 5902.51)	-0.06 (-0.13, 0.02)
25-29 years	15376133.49 (7667284.56, 26705813.34)	3473.89 (1732.25, 6033.58)	21751757.86 (10965742.14, 37472652.59)	3697.12 (1863.83, 6369.18)	-0.07 (-0.14, 0.01)
30-34 years	14838006.22 (7523173.07, 25268390.89)	3849.8 (1951.93, 6556.02)	23900379.59 (12193881.97, 40561063.18)	3953.87 (2017.25, 6710.07)	-0.1 (-0.16, -0.03)
35-39 years	15074144.6 (7929799.32, 24937026.29)	4279.46 (2251.22, 7079.47)	24719538.84 (13035681.01, 40956477.69)	4407.39 (2324.21, 7302.36)	-0.1 (-0.14, -0.06)
40-44 years	13700041.19 (7290881.35, 22699515.32)	4782.17 (2544.97, 7923.55)	24427887.69 (12968549.73, 40536667.02)	4883.13 (2592.41, 8103.27)	-0.09 (-0.12, -0.07)
45-49 years	12052927.93 (6692394.88, 19685585.96)	5190.85 (2882.22, 8478.02)	24745053.75 (13662414.46, 40460634.1)	5225.94 (2885.38, 8544.93)	-0.07 (-0.09, -0.05)
50-54 years	12116217.3 (6861481.45, 19360867.25)	5699.83 (3227.85, 9107.93)	25210473.54 (14217653.21, 40441476.78)	5666.26 (3195.53, 9089.55)	-0.07 (-0.08, -0.05)
55-59 years	11391324.01 (6617735.97, 18028960.89)	6150.82 (3573.29, 9734.86)	24274496.85 (14022819.87, 38595402.09)	6134.14 (3543.55, 9753.01)	-0.08 (-0.1, -0.07)
60-64 years	10716353.78 (6397294.77, 16768316.1)	6672.31 (3983.14, 10440.44)	21218105.41 (12631841.18, 33173685.71)	6629.67 (3946.86, 10365.23)	-0.09 (-0.1, -0.07)
65-69 years	8988081.03 (5445049.02, 13876991.35)	7271.35 (4405.04, 11226.48)	19823369.76 (12021328.13, 30545014.05)	7186.49 (4358.05, 11073.37)	-0.06 (-0.07, -0.05)
70-74 years	6841302.5 (4254254.12, 10382466.89)	8080.8 (5025.04, 12263.55)	16516566.62 (10301237.93, 25089815.65)	8024.01 (5004.51, 12189.04)	-0.02 (-0.03, -0.01)
75-79 years	5720459.87 (3621341.83, 8582389.02)	9293.17 (5883.05, 13942.52)	12019203.79 (7650546.22, 18068587.55)	9113.44 (5800.95, 13700.32)	0 (-0.01, 0.01)
80-84 years	3761812.49 (2422127.53, 5526047.5)	10633.82 (6846.82, 15620.93)	9344298.08 (5969911.04, 13763853.57)	10669.07 (6816.29, 15715.2)	-0.01 (-0.02, 0)
85-89 years	1828680.03 (1195643.18, 2676643.03)	12101.59 (7912.36, 17713.12)	5591550.05 (3657961.97, 8176496.83)	12229.51 (8000.48, 17883.15)	-0.06 (-0.08, -0.04)
90-94 years	570766.15 (376503, 812505.15)	13319.51 (8786.15, 18960.78)	2393770.77 (1575656.86, 3419705.19)	13380.96 (8807.78, 19115.85)	-0.3 (-0.41, -0.18)
95 plus years	149746.36 (98674.78, 215772.22)	14708.61 (9692.18, 21193.9)	791657.58 (523187.64, 1138074.99)	14525 (9599.23, 20880.93)	0.11 (0.02, 0.2)

Notes: ASR: age-standardized rates; YLDs: years lived with disability; EAPCs: estimated annual percentage changes.

Table S4. Prevalence and YLDs of musculoskeletal disorders and its temporal trends from 1990, 2021 by gender, and age in the worldwide.

	1990		2021		
	Number	ASR, per 100,000 persons	Number	ASR, per 100,000 persons	EAPC 95%CI
**Prevalence**					
**Gender**					
Female	421998748.92 (363363817.19, 484189031.23)	18372.2 (15784.16, 21068.19)	782244778.3 (669845992.17, 895856913.37)	17835.05 (15296.7, 20442.6)	-0.04 (-0.07, -0.01)
Male	262489036.46 (222932249.49, 303856033.24)	12139.11 (10373.37, 13958.79)	481173015.48 (408737814.23, 554846453.68)	11655.37 (9950.31, 13415.45)	-0.1 (-0.13, -0.07)
**Age**					
5-9 years	343840.08 (171057.14, 604624.68)	329.73 (164.04, 579.82)	312495.48 (153657.38, 543474.84)	326.3 (160.44, 567.48)	-0.04 (-0.06, -0.01)
10-14 years	1757433.66 (1191327.77, 2528233.66)	1718.05 (1164.63, 2471.57)	1502784.03 (1032344.06, 2132647.91)	1743.51 (1197.71, 2474.27)	-0.11 (-0.12, -0.09)
15-19 years	4421770.65 (2954352.73, 6265386.18)	3490.91 (2332.41, 4946.41)	2674827.74 (1804943.11, 3772584.79)	3582.1 (2417.16, 5052.2)	-0.18 (-0.22, -0.14)
20-24 years	6471856.82 (4177196.24, 9644173.81)	4902.89 (3164.52, 7306.14)	3521289.07 (2254217.37, 5226689.14)	4812.2 (3080.62, 7142.81)	-0.19 (-0.23, -0.14)
25-29 years	7089956.36 (4510592.37, 10498119.06)	6451.9 (4104.66, 9553.35)	4824717.98 (3072888.23, 7169209.93)	5578.86 (3553.21, 8289.82)	-0.2 (-0.25, -0.14)
30-34 years	7700215.2 (5147797.48, 11175926.89)	8726.02 (5833.57, 12664.75)	8255717.45 (5541218.39, 12058780.75)	6814.27 (4573.73, 9953.33)	-0.27 (-0.35, -0.18)
35-39 years	11431241.61 (7737345.56, 16182147.09)	12515.17 (8471.02, 17716.57)	10734545.48 (7287521.19, 15268325.41)	10130.44 (6877.4, 14409.08)	-0.23 (-0.31, -0.16)
40-44 years	11783006.3 (8188051.65, 16299383.31)	17561.85 (12203.79, 24293.23)	14291075.82 (9950708.28, 19894110.34)	15612.91 (10871.09, 21734.2)	-0.15 (-0.2, -0.11)
45-49 years	12504124.34 (9011849.05, 17002798.69)	24223.9 (17458.41, 32939.06)	25429572.14 (18472758.46, 34616248.92)	23050.41 (16744.47, 31377.59)	-0.05 (-0.09, -0.02)
50-54 years	15034656.3 (10737756.13, 20480091.72)	31512.01 (22505.89, 42925.42)	37182521.83 (26519913.51, 50285379.95)	30765.2 (21942.85, 41606.64)	0 (-0.03, 0.03)
55-59 years	15998157.11 (11722748.38, 21328345.94)	36888.38 (27030.18, 49178.67)	40077033.24 (29548920.21, 53151357.81)	36452.76 (26876.73, 48344.74)	0.03 (0, 0.05)
60-64 years	14364177 (10660826.32, 18797736.52)	40648.58 (30168.62, 53194.93)	29270494.24 (21833727.05, 38234450.79)	40093.73 (29907.1, 52372.25)	0.03 (0.01, 0.06)
65-69 years	11998680.45 (9088585.97, 15638096.69)	43980.51 (33313.72, 57320.6)	32988109.13 (25131489.51, 42638332.44)	43007.3 (32764.46, 55588.5)	-0.01 (-0.04, 0.02)
70-74 years	8976139.35 (6727365.27, 11807957.4)	47700.64 (35750.29, 62749.37)	24194275.94 (18301291.84, 31733685.26)	45395.67 (34338.67, 59541.85)	-0.05 (-0.07, -0.03)
75-79 years	5839814.92 (4462822.78, 7702642.37)	51313.33 (39213.97, 67681.64)	16034272.62 (12290207.14, 20859797.14)	48414.21 (37109.3, 62984.5)	-0.08 (-0.11, -0.06)
80-84 years	2821557.98 (2191458.65, 3619446.12)	53265.83 (41370.71, 68328.49)	10123298.32 (7906617.66, 13025067.84)	51148.81 (39948.84, 65810.24)	-0.06 (-0.08, -0.04)
85-89 years	903981.36 (690608.55, 1176507.08)	53589.73 (40940.59, 69745.57)	5017120.7 (3880988.78, 6452208.67)	52669.03 (40742.08, 67734.38)	0.01 (0, 0.02)
90-94 years	156504.52 (122291.26, 200531.56)	51008.23 (39857.39, 65357.61)	1516174.47 (1178978.47, 1940245.74)	51711.49 (40210.9, 66175.1)	0.09 (0.09, 0.1)

95 plus years	19910.94 (15134.58, 25840.57)	49172.42 (37376.64, 63816.36)	321296.94 (244890.32, 417568.09)	50273.47 (38318.09, 65337.05)	0.17 (0.16, 0.18)
**YLDs**					
**Gender**					
Female	37831772.74 (26016080.35, 53453170.18)	1616.31 (1105.67, 2291.09)	65712592.73 (44612633.76, 93831220.5)	1518.28 (1033.29, 2163.16)	-0.14 (-0.18, -0.11)
Male	23691184.9 (16142820.29, 33628671.81)	1053.8 (713.47, 1508.58)	40373071.9 (27202718.37, 58258800.5)	976.47 (659.24, 1405.51)	-0.21 (-0.24, -0.18)
**Age**					
5-9 years	37659.38 (16929.9, 69922.99)	36.11 (16.24, 67.05)	34329.54 (15661.21, 63075.25)	35.85 (16.35, 65.86)	-0.03 (-0.05, -0.01)
10-14 years	191040.8 (112073.7, 303693.44)	186.76 (109.56, 296.89)	163948.72 (96495.99, 257235.76)	190.21 (111.95, 298.44)	-0.1 (-0.12, -0.09)
15-19 years	486775.55 (272395.85, 763707.85)	384.3 (215.05, 602.93)	295448.65 (164918.57, 461726.82)	395.66 (220.86, 618.34)	-0.18 (-0.22, -0.14)
20-24 years	722911.97 (405816.51, 1191403.69)	547.66 (307.43, 902.57)	395434.35 (223906.63, 649037.23)	540.4 (305.99, 886.98)	-0.18 (-0.22, -0.14)
25-29 years	798273.66 (435584.3, 1292847.2)	726.43 (396.38, 1176.5)	544596.86 (298669.35, 898451.58)	629.72 (345.35, 1038.89)	-0.19 (-0.25, -0.13)
30-34 years	854481.59 (483422.83, 1359599.64)	968.31 (547.82, 1540.72)	909865.7 (516139.58, 1458373.87)	751 (426.02, 1203.74)	-0.27 (-0.36, -0.18)
35-39 years	1194231.95 (683467.92, 1905181.11)	1307.47 (748.28, 2085.83)	1093580.06 (625066.84, 1767350.76)	1032.04 (589.89, 1667.89)	-0.27 (-0.35, -0.19)
40-44 years	1122399.23 (649826.31, 1777032.78)	1672.87 (968.53, 2648.56)	1311607.13 (758292.48, 2089935.39)	1432.92 (828.43, 2283.24)	-0.24 (-0.29, -0.19)
45-49 years	1069020.61 (619033.91, 1765033.35)	2070.98 (1199.24, 3419.35)	2075559.93 (1183444.58, 3446287.67)	1881.37 (1072.72, 3123.86)	-0.18 (-0.21, -0.15)
50-54 years	1179673.69 (667475.44, 1961053.9)	2472.55 (1399, 4110.29)	2763766.39 (1545291.51, 4686812.16)	2286.77 (1278.59, 3877.92)	-0.15 (-0.17, -0.12)
55-59 years	1202379.42 (683857.03, 1975469.94)	2772.43 (1576.83, 4555.02)	2841326.98 (1580301.29, 4768062.97)	2584.38 (1437.39, 4336.87)	-0.12 (-0.14, -0.09)
60-64 years	1059643.81 (604709.78, 1729785.54)	2998.64 (1711.24, 4895.05)	2040560.34 (1163971.83, 3343873.08)	2795.09 (1594.37, 4580.32)	-0.11 (-0.14, -0.08)
65-69 years	876248.8 (496870.48, 1409015.78)	3211.84 (1821.25, 5164.67)	2259562.82 (1271172.22, 3655032.75)	2945.84 (1657.25, 4765.14)	-0.15 (-0.18, -0.12)
70-74 years	649591.61 (379118.69, 1050431.11)	3452.03 (2014.7, 5582.16)	1629680.46 (936844.81, 2681203.81)	3057.77 (1757.8, 5030.74)	-0.17 (-0.2, -0.15)
75-79 years	413665.2 (239156.84, 669062.94)	3634.8 (2101.43, 5878.93)	1054805.18 (603277.91, 1722323.9)	3184.9 (1821.55, 5200.42)	-0.19 (-0.22, -0.17)
80-84 years	191798.77 (115323.65, 308310.27)	3620.81 (2177.1, 5820.33)	643046.01 (381602.82, 1046773.44)	3249.04 (1928.08, 5288.91)	-0.16 (-0.19, -0.13)
85-89 years	58568.41 (35532.52, 96522.59)	3472.05 (2106.44, 5722.04)	305989.04 (183444.92, 507629.59)	3212.23 (1925.78, 5329.02)	-0.1 (-0.12, -0.08)
90-94 years	9475.69 (5657.57, 15042.11)	3088.33 (1843.93, 4902.55)	86903.25 (52080.23, 138946.67)	2963.97 (1776.28, 4738.99)	-0.01 (-0.03, 0)
95 plus years	1130.61 (656.26, 1877.04)	2792.18 (1620.71, 4635.56)	17276.13 (10054.89, 28651.67)	2703.2 (1573.29, 4483.14)	0.06 (0.05, 0.08)

Notes: ASR: age-standardized rates; YLDs: years lived with disability; EAPCs: estimated annual percentage changes.

Table S5 Prevalence and YLDs of neurological disorders and its temporal trends from 1990, 2021 by gender, and age in the worldwide.

	1990		2021		EAPC 95%CI
	Number	ASR, per 100,000 persons	Number	ASR, per 100,000 persons	
**Prevalence**					
**Gender**					
Female	1173841827.71 (1021223764.8, 1340749237.7)	45828.56 (40146.31, 51841.4)	1854915962.68 (1630405648.21, 2103463169.65)	45449.13 (39824.58, 51556.72)	-0.03 (-0.04, -0.02)
Male	920453064.33 (796379981.23, 1054820736.66)	35951.67 (31459.65, 40684.73)	1477570112.91 (1289290711.9, 1679320279.9)	36447.06 (31865.79, 41354.96)	0.05 (0.03, 0.06)
**Age**					
<5 years	232431.02 (210355.13, 257715.52)	37.49 (33.93, 41.57)	197464.12 (181284.28, 214517.29)	30 (27.54, 32.59)	-0.92 (-0.98, -0.85)
5-9 years	57166871.07 (36055465.11, 82394606.24)	9796.72 (6178.85, 14120.01)	66516711.27 (41890113.74, 96306290.99)	9681.44 (6097.07, 14017.29)	0 (-0.03, 0.03)
10-14 years	196275319.66 (144086119.32, 257643758.47)	36640.22 (26897.66, 48096.34)	244211260.43 (179056396.13, 322697611.45)	36633.38 (26859.7, 48406.87)	0.03 (0.01, 0.05)
15-19 years	237276938.14 (162678289.88, 314267583.62)	45680.83 (31319.01, 60503.16)	292054334.3 (200790978.14, 388269405.94)	46805.09 (32179.08, 62224.67)	0.06 (0.04, 0.08)
20-24 years	242508022.21 (180305924.22, 321274720.23)	49281.44 (36641, 65288.07)	305036458.83 (226366727.97, 406868074.9)	51081.35 (37907.33, 68134.06)	0.09 (0.07, 0.11)
25-29 years	234875667.44 (175020781.12, 306955722.42)	53064.9 (39542.03, 69349.77)	317020139.23 (234027340.36, 414965126.66)	53883.54 (39777.35, 70531.13)	0.07 (0.05, 0.1)
30-34 years	218263483.18 (160578222.47, 283595194.56)	56629.67 (41662.91, 73580.34)	339293352.39 (249652023.31, 442551938.24)	56129.77 (41300.28, 73211.99)	0.01 (-0.04, 0.05)
35-39 years	195535671.92 (141196306.36, 252310707.61)	55511.35 (40084.75, 71629.43)	312444949.51 (224659629.09, 405946932.58)	55707.58 (40055.84, 72378.58)	0 (-0.04, 0.03)
40-44 years	159366603.76 (120524530.1, 210581051.9)	55628.91 (42070.6, 73505.95)	277863494.55 (209364825.34, 367033284.37)	55544.84 (41851.97, 73369.86)	-0.03 (-0.06, 0)
45-49 years	121004239.08 (90564203.16, 154994298.65)	52113.09 (39003.43, 66751.64)	244103479.47 (181837627.02, 314209922.69)	51552.5 (38402.5, 66358.36)	-0.05 (-0.08, -0.02)
50-54 years	104800986.06 (78092872.43, 136418598.16)	49301.53 (36737.23, 64175.4)	214069880.31 (159655402.78, 280674005.76)	48113.92 (35883.83, 63083.73)	-0.07 (-0.09, -0.05)
55-59 years	89248235.77 (67151005.65, 118098450.32)	48190.2 (36258.65, 63768.08)	187250322.01 (140959984.37, 248747876.49)	47317.94 (35620.42, 62858.3)	-0.04 (-0.06, -0.02)
60-64 years	72381681.66 (53602825.49, 96407709.35)	45066.95 (33374.68, 60026.25)	142045662.19 (104995787.73, 189407613.47)	44382.63 (32806.28, 59181.03)	-0.06 (-0.09, -0.04)
65-69 years	58794276.37 (44715683.76, 77336098.93)	47564.55 (36174.97, 62564.87)	128064800.97 (97934758.9, 168368364.84)	46426.84 (35503.91, 61037.94)	-0.09 (-0.11, -0.08)
70-74 years	42699416.65 (32556243.14, 56929342.41)	50435.67 (38454.76, 67243.76)	102400184.06 (77871574.75, 135788730.95)	49747.65 (37831.26, 65968.35)	-0.07 (-0.09, -0.06)
75-79 years	31219043.5 (22449027.35, 40681949.31)	50716.89 (36469.56, 66089.85)	66312112.09 (47926986.26, 86206756.48)	50280.48 (36340.15, 65365.39)	-0.04 (-0.05, -0.02)
80-84 years	19664984.61 (14837942.43, 25708416.48)	55588.61 (41943.61, 72672.06)	48953549.48 (37050390.06, 63711512.99)	55893.86 (42303.15, 72744.11)	-0.01 (-0.02, 0.01)
85-89 years	9280069.02 (6952910.47, 12066375.66)	61412.38 (46012.03, 79851.22)	28303816.86 (21281512.67, 36777682.56)	61904.44 (46545.67, 80437.97)	-0.01 (-0.02, 0)

90-94 years	2877190.65 (2167307.67, 3724894.57)	67142.7 (50576.73, 86924.89)	11997211.24 (9026626.38, 15576242.06)	67063.34 (50458.03, 87069.8)	-0.02 (-0.03, -0.01)
95 plus years	823760.26 (605838.95, 1077713.83)	80912.6 (59507.61, 105856.8)	4346892.27 (3190430.63, 5716685.5)	79754.97 (58536.7, 104887.37)	-0.06 (-0.07, -0.05)
**YLDs**					
**Gender**					
Female	26307069.17 (8189820.63, 52633166.38)	1091.28 (373.04, 2117.16)	45690325.88 (16141604.58, 87605237.27)	1078.38 (361.25, 2098.87)	-0.06 (-0.07, -0.05)
Male	16824147.4 (5820919.42, 34039087.97)	757.21 (307.75, 1425.26)	30748177.91 (12065628.64, 58484977.03)	774.95 (309.02, 1462.56)	0.06 (0.05, 0.07)
**Age**					
<5 years	37081.76 (26179.03, 48351.08)	5.98 (4.22, 7.8)	29747.04 (21127.9, 38867.91)	4.52 (3.21, 5.91)	-1.1 (-1.17, -1.02)
5-9 years	627390.1 (73741.32, 1764443.72)	107.52 (12.64, 302.37)	720256.39 (72641.05, 2039143.14)	104.83 (10.57, 296.8)	-0.05 (-0.08, -0.01)
10-14 years	2591591.26 (209178.2, 6970095.79)	483.79 (39.05, 1301.16)	3230981.5 (235976.63, 8670998.82)	484.67 (35.4, 1300.71)	0.06 (0.03, 0.09)
15-19 years	3691955.61 (457919.07, 9245369.13)	710.78 (88.16, 1779.93)	4520718.75 (522662.66, 11489917.38)	724.5 (83.76, 1841.39)	0.06 (0.04, 0.07)
20-24 years	3956211.45 (572968.04, 9598726.96)	803.96 (116.44, 1950.61)	4875412.19 (666883.94, 11913497.14)	816.44 (111.68, 1995.03)	0.04 (0.02, 0.05)
25-29 years	3817811.96 (571046.79, 8959545.34)	862.55 (129.02, 2024.21)	5078240.89 (704798.6, 11968192.02)	863.14 (119.79, 2034.22)	0.02 (0, 0.04)
30-34 years	3588934.35 (637905.13, 8167774.15)	931.17 (165.51, 2119.17)	5563463.4 (953572.99, 12811176.15)	920.37 (157.75, 2119.37)	-0.01 (-0.04, 0.01)
35-39 years	3473984.36 (785588.74, 7504797.76)	986.24 (223.02, 2130.57)	5498307.69 (1172835.06, 12054188.34)	980.32 (209.11, 2149.21)	-0.03 (-0.06, -0.01)
40-44 years	3011056.99 (791890.18, 6501235.47)	1051.05 (276.42, 2269.34)	5192470.75 (1295226.01, 11393154.89)	1037.97 (258.92, 2277.49)	-0.06 (-0.08, -0.05)
45-49 years	2516119.71 (773379.98, 5050848.04)	1083.62 (333.07, 2175.26)	5018126.06 (1481642.13, 10263333.98)	1059.78 (312.91, 2167.53)	-0.08 (-0.1, -0.07)
50-54 years	2480766.89 (928131.81, 4711766.21)	1167.03 (436.62, 2216.56)	4993728.14 (1766543.88, 9654085.35)	1122.38 (397.04, 2169.83)	-0.11 (-0.12, -0.09)
55-59 years	2333702.93 (1028881.63, 4245057.1)	1260.1 (555.55, 2292.15)	4860929.09 (2052169.3, 9009826.41)	1228.35 (518.58, 2276.77)	-0.09 (-0.11, -0.07)
60-64 years	2299907.73 (1179788.67, 3912390.32)	1431.99 (734.57, 2435.97)	4470674.17 (2221232.8, 7674810.25)	1396.88 (694.03, 2398.02)	-0.08 (-0.11, -0.05)
65-69 years	2079254.08 (1179164.35, 3401719.27)	1682.12 (953.94, 2751.99)	4694327.49 (2628503.51, 7662718.67)	1701.82 (952.9, 2777.94)	-0.05 (-0.08, -0.02)
70-74 years	1824845.24 (1107911.87, 2836909.12)	2155.47 (1308.64, 3350.9)	4544505.11 (2758728.04, 7072317.69)	2207.79 (1340.23, 3435.85)	-0.01 (-0.03, 0.01)
75-79 years	1842088.92 (1186215.28, 2706716.46)	2992.57 (1927.07, 4397.2)	4109826.11 (2670693.06, 6028683.7)	3116.23 (2025.03, 4571.19)	0.07 (0.06, 0.08)
80-84 years	1572822.95 (1017086.87, 2219511.64)	4446.03 (2875.08, 6274.07)	4098331.67 (2636663.29, 5804396.09)	4679.37 (3010.47, 6627.3)	0.09 (0.06, 0.11)
85-89 years	938393.92 (625231.19, 1315451.86)	6209.98 (4137.57, 8705.22)	2948043.65 (1962505.06, 4128508.76)	6447.79 (4292.28, 9029.63)	0.03 (0.01, 0.06)
90-94 years	345417.55 (233190.58, 474420.63)	8060.73 (5441.78, 11071.18)	1454309.45 (975270.98, 2006133.81)	8129.46 (5451.68, 11214.11)	-0.02 (-0.04, -0.01)
95 plus years	101878.81 (69529.07, 141365.4)	10006.89 (6829.39, 13885.4)	536104.23 (366966.28, 740160.08)	9836.22 (6732.94, 13580.15)	-0.09 (-0.11, -0.07)

Notes: ASR: age-standardized rates; YLDs: years lived with disability; EAPCs: estimated annual percentage changes.

Table S6. Prevalence and YLDs of digestive disorders and its temporal trends from 1990, 2021 by gender, and age in the worldwide.

	1990		2021		EAPC 95%CI
	Number	ASR, per 100,000 persons	Number	ASR, per 100,000 persons	
**Prevalence**					
**Gender**					
Female	2978157.59 (2337698.48, 3837397.74)	131.06 (102.38, 169.57)	4621374.91 (3666916.48, 5884627.09)	105.24 (83.95, 133.48)	-0.73 (-0.81, -0.65)
Male	3340390.86 (2580693.29, 4409807.89)	151.57 (117.14, 200.29)	5109084.55 (3998091.82, 6587782.98)	122.48 (95.99, 157.37)	-0.73 (-0.79, -0.66)
**Age**					
<5 years	14000.62 (7403.63, 24507.59)	2.26 (1.19, 3.95)	13342.27 (6861.98, 23731.03)	2.03 (1.04, 3.61)	-0.21 (-0.31, -0.11)
5-9 years	45746.48 (25456.96, 74747.68)	7.84 (4.36, 12.81)	47565.29 (25809.97, 79031.44)	6.92 (3.76, 11.5)	-0.35 (-0.42, -0.28)
10-14 years	77172.92 (45732.88, 121745.19)	14.41 (8.54, 22.73)	85860.79 (49444.88, 134841.74)	12.88 (7.42, 20.23)	-0.41 (-0.45, -0.38)
15-19 years	128404.37 (82368.58, 194191.21)	24.72 (15.86, 37.39)	138781.63 (88330.91, 212395.78)	22.24 (14.16, 34.04)	-0.42 (-0.53, -0.3)
20-24 years	206015.27 (142765.51, 302961.05)	41.87 (29.01, 61.57)	222151.47 (152476.13, 330404.97)	37.2 (25.53, 55.33)	-0.37 (-0.55, -0.19)
25-29 years	321447.4 (225798.74, 457147.44)	72.62 (51.01, 103.28)	351831.25 (242319.05, 510863.31)	59.8 (41.19, 86.83)	-0.35 (-0.54, -0.17)
30-34 years	442056.78 (306354.65, 632272.08)	114.69 (79.49, 164.05)	542017.72 (372705.2, 784031.32)	89.67 (61.66, 129.7)	-0.57 (-0.73, -0.4)
35-39 years	547480.61 (370321.1, 830041.15)	155.43 (105.13, 235.64)	713908.03 (485161.1, 1049994.84)	127.29 (86.5, 187.21)	-0.73 (-0.86, -0.61)
40-44 years	590642.66 (416895.81, 874356.25)	206.17 (145.52, 305.2)	833730.11 (590578.98, 1207303.71)	166.66 (118.06, 241.34)	-0.94 (-1.05, -0.82)
45-49 years	569506.92 (407241.6, 797148.83)	245.27 (175.39, 343.31)	942455 (673477.7, 1294684.75)	199.04 (142.23, 273.43)	-0.86 (-1.09, -0.62)
50-54 years	708655.6 (497424.68, 1013982.29)	333.37 (234, 477.01)	1045605.51 (761780.98, 1454304.32)	235.01 (171.22, 326.87)	-0.89 (-1.12, -0.66)
55-59 years	665671.82 (456959.96, 952812.79)	359.43 (246.74, 514.48)	1119016.08 (792912.91, 1562129.83)	282.77 (200.37, 394.75)	-0.67 (-0.84, -0.49)
60-64 years	680724.06 (455644.62, 951777.65)	423.84 (283.7, 592.6)	1089222.61 (764274.07, 1497904.57)	340.33 (238.8, 468.03)	-0.77 (-0.91, -0.62)
65-69 years	502445.97 (339005.17, 715609.27)	406.48 (274.26, 578.93)	933775.17 (652382.72, 1299908.17)	338.52 (236.51, 471.25)	-0.89 (-1.04, -0.73)
70-74 years	335161.8 (226311.45, 481469.24)	395.89 (267.31, 568.7)	720079.15 (502904.67, 999849.22)	349.83 (244.32, 485.74)	-0.72 (-0.94, -0.51)
75-79 years	265045.28 (175503.99, 373808.69)	430.58 (285.11, 607.27)	417631.23 (294856.86, 567442.35)	316.66 (223.57, 430.26)	-0.71 (-0.87, -0.55)
80-84 years	142331.1 (99764.36, 198666.86)	402.34 (282.01, 561.59)	298281.29 (215836.48, 401679.61)	340.57 (246.44, 458.63)	-0.64 (-0.76, -0.51)
85-89 years	57025.26 (40314.85, 79563.93)	377.37 (266.79, 526.53)	142016.95 (104526.5, 192715.52)	310.61 (228.61, 421.5)	-0.58 (-0.68, -0.48)

90-94 years	15417.31 (10805.76, 21295.6)	359.78 (252.17, 496.96)	56980.33 (40762.12, 77625.48)	318.51 (227.86, 433.92)	-0.45 (-0.53, -0.38)
95 plus years	3596.25 (2502.59, 5109.44)	353.24 (245.81, 501.87)	16207.57 (11339.62, 23015.16)	297.37 (208.05, 422.27)	-0.62 (-0.72, -0.52)
**YLDs**					
**Gender**					
Female	288980.06 (173554.03, 437986.4)	12.57 (7.56, 19.05)	471297.97 (288398.7, 702498.29)	10.82 (6.62, 16.09)	-0.45 (-0.53, -0.36)
Male	295207.66 (169751.67, 466733.98)	13.26 (7.66, 20.89)	474903.63 (281741.43, 728981.03)	11.39 (6.78, 17.45)	-0.47 (-0.55, -0.4)
**Age**					
<5 years	1067.5 (524.74, 1983.35)	0.17 (0.08, 0.32)	1023.06 (501.93, 1877.82)	0.16 (0.08, 0.29)	-0.24 (-0.32, -0.15)
5-9 years	3532.12 (1704.57, 6570.08)	0.61 (0.29, 1.13)	3751.97 (1811.74, 6938.71)	0.55 (0.26, 1.01)	-0.28 (-0.32, -0.24)
10-14 years	6545.49 (3459.37, 11179.25)	1.22 (0.65, 2.09)	7523.85 (4074.42, 12766.33)	1.13 (0.61, 1.92)	-0.26 (-0.35, -0.17)
15-19 years	12426.75 (7108.45, 20337.46)	2.39 (1.37, 3.92)	13830.52 (7867.5, 22643.27)	2.22 (1.26, 3.63)	-0.26 (-0.43, -0.1)
20-24 years	22297.54 (12960.28, 35174.41)	4.53 (2.63, 7.15)	24513.51 (14122.6, 38522.35)	4.11 (2.36, 6.45)	-0.27 (-0.47, -0.07)
25-29 years	35852.31 (20963.67, 55771.07)	8.1 (4.74, 12.6)	40626.02 (23602.6, 64011.14)	6.91 (4.01, 10.88)	-0.29 (-0.48, -0.09)
30-34 years	48339.94 (28356.28, 78242.22)	12.54 (7.36, 20.3)	61971.71 (36414.5, 101302.13)	10.25 (6.02, 16.76)	-0.46 (-0.62, -0.29)
35-39 years	57521.69 (33444.15, 92186.58)	16.33 (9.49, 26.17)	79020.43 (45317.08, 125208.78)	14.09 (8.08, 22.32)	-0.5 (-0.61, -0.4)
40-44 years	60003.57 (34561.59, 96541.45)	20.94 (12.06, 33.7)	89010.94 (51556.14, 141374.91)	17.79 (10.31, 28.26)	-0.63 (-0.71, -0.55)
45-49 years	55660.93 (31397.08, 91026.69)	23.97 (13.52, 39.2)	96434.99 (55198.34, 154503.94)	20.37 (11.66, 32.63)	-0.59 (-0.76, -0.41)
50-54 years	61675.98 (33799.71, 102249.43)	29.01 (15.9, 48.1)	102894.25 (59144.86, 162724.09)	23.13 (13.29, 36.57)	-0.56 (-0.74, -0.37)
55-59 years	56317.3 (30497.57, 93393.62)	30.41 (16.47, 50.43)	104363.81 (59380.57, 167735.91)	26.37 (15.01, 42.39)	-0.33 (-0.46, -0.2)
60-64 years	54651.64 (30190.64, 90780.28)	34.03 (18.8, 56.52)	96403.17 (56414.59, 153345.64)	30.12 (17.63, 47.91)	-0.39 (-0.47, -0.3)
65-69 years	41563.58 (23391.39, 66361.15)	33.62 (18.92, 53.69)	81306.2 (47221.88, 127097.62)	29.48 (17.12, 46.08)	-0.5 (-0.58, -0.42)
70-74 years	27633.27 (15593.37, 43681.53)	32.64 (18.42, 51.6)	62122.46 (35428.74, 97253.84)	30.18 (17.21, 47.25)	-0.44 (-0.55, -0.32)
75-79 years	21110.2 (12003.06, 34098.23)	34.29 (19.5, 55.39)	37131.32 (21675.93, 57917.96)	28.15 (16.44, 43.92)	-0.51 (-0.58, -0.43)
80-84 years	11554.21 (6751.6, 18173.84)	32.66 (19.09, 51.37)	25340.04 (15214.51, 39548.63)	28.93 (17.37, 45.16)	-0.51 (-0.59, -0.43)
85-89 years	4746.78 (2804.02, 7504.71)	31.41 (18.56, 49.66)	12383.56 (7563.85, 19268.91)	27.08 (16.54, 42.14)	-0.46 (-0.54, -0.39)
90-94 years	1358.95 (825.65, 2129.25)	31.71 (19.27, 49.69)	5061.05 (3099.37, 7859.2)	28.29 (17.33, 43.93)	-0.38 (-0.43, -0.33)
95 plus years	327.95 (196.08, 495.5)	32.21 (19.26, 48.67)	1488.74 (907.51, 2271.85)	27.31 (16.65, 41.68)	-0.6 (-0.68, -0.53)

Notes: ASR: age-standardized rates; YLDs: years lived with disability; EAPCs: estimated annual percentage changes.

Table S7. Prevalence and YLDs of genecological disorders and its temporal trends from 1990, 2021 by gender, and age in the worldwide.

	1990		2021		
	Number	ASR, per 100,000 persons	Number	ASR, per 100,000 persons	EAPC 95%CI
**Prevalence**					
**Gender**					
Female	59689999.8 (32625583.68, 104614493.31)	2267.26 (1219.63, 3969.94)	110089459.27 (58608815.06, 195025585.22)	2764.62 (1476.33, 4862.57)	0.7 (0.53, 0.87)
Male	31490381.81 (18725068.36, 50165061.42)	1158.86 (696.62, 1858.35)	55000818.26 (32611257.3, 88727953.05)	1354.76 (802.12, 2174.77)	0.5 (0.36, 0.64)
**Age**					
15-19 years	1580871.67 (327359.62, 4393120.97)	304.35 (63.02, 845.77)	2118150.48 (358625.06, 6009051.52)	339.46 (57.47, 963.02)	0.05 (-0.18, 0.28)
20-24 years	12890639.11 (4961874.01, 25611553.72)	2619.58 (1008.33, 5204.67)	20878682.92 (7790058.7, 41977203.53)	3496.34 (1304.52, 7029.5)	0.96 (0.72, 1.21)
25-29 years	16545982.86 (5818514.7, 38780512.88)	3738.19 (1314.56, 8761.59)	29312891.47 (10130902.72, 67513260.89)	4982.28 (1721.94, 11475.15)	1.1 (0.85, 1.35)
30-34 years	19869581.02 (6098485.75, 43898281.64)	5155.27 (1582.29, 11389.65)	39275050.13 (11715792.23, 90009669.4)	6497.33 (1938.16, 14890.43)	0.77 (0.57, 0.97)
35-39 years	24783191.17 (8418620.08, 53561275.87)	7035.79 (2389.99, 15205.71)	43909253.69 (13881453.62, 96097605.58)	7828.83 (2475, 17133.79)	0.4 (0.26, 0.54)
40-44 years	14901617.85 (4916318.74, 32064202.61)	5201.6 (1716.1, 11192.41)	28291519.78 (8769041.24, 61765874.65)	5655.47 (1752.93, 12346.98)	0.24 (0.16, 0.31)
45-49 years	608497.93 (173361.22, 1724917.33)	262.06 (74.66, 742.87)	1304729.08 (338802.71, 4102129.56)	275.55 (71.55, 866.33)	-0.25 (-0.44, -0.05)
**YLDs**					
**Gender**					
Female	325936.79 (114823.01, 807747)	12.32 (4.39, 30.93)	601133.5 (213157.97, 1468475.01)	15.12 (5.35, 36.88)	0.71 (0.54, 0.88)
Male	181868.97 (66532.49, 425578.94)	6.65 (2.47, 15.61)	317613.61 (116288.11, 752758.23)	7.84 (2.85, 18.56)	0.51 (0.38, 0.65)
**Age**					
15-19 years	10334.99 (1505.68, 36375.15)	1.99 (0.29, 7)	13259.35 (1592.32, 48998.03)	2.12 (0.26, 7.85)	-0.13 (-0.31, 0.06)
20-24 years	80637.6 (20194.6, 219286.33)	16.39 (4.1, 44.56)	128305.46 (32056.85, 340464.28)	21.49 (5.37, 57.01)	0.86 (0.64, 1.07)
25-29 years	96220.25 (21725.69, 259695.5)	21.74 (4.91, 58.67)	171146.19 (38689.31, 462859.52)	29.09 (6.58, 78.67)	1.09 (0.84, 1.33)
30-34 years	106704.89 (23390.65, 289269.72)	27.69 (6.07, 75.05)	212135.37 (44610.21, 571405.54)	35.09 (7.38, 94.53)	0.79 (0.59, 0.99)
35-39 years	130909.68 (33900.61, 370633.22)	37.16 (9.62, 105.22)	233969.25 (58148.55, 659134.47)	41.72 (10.37, 117.52)	0.43 (0.29, 0.57)
40-44 years	79652.98 (19051.06, 233906.26)	27.8 (6.65, 81.65)	152699.83 (35849.26, 446561.02)	30.52 (7.17, 89.27)	0.27 (0.19, 0.35)
45-49 years	3345.36 (740.55, 11254.94)	1.44 (0.32, 4.85)	7231.65 (1484.56, 25556.84)	1.53 (0.31, 5.4)	-0.21 (-0.39, -0.02)

Notes: ASR: age-standardized rates; YLDs: years lived with disability; EAPCs: estimated annual percentage changes.

Table S8. Prevalence and YLDs of mental disorders and its temporal trends from 1990, 2021 by gender, and age in the worldwide.

	1990		2021		EAPC 95%CI
	Number	ASR, per 100,000 persons	Number	ASR, per 100,000 persons	
**Prevalence**					
**Gender**					
Female	227903228.21 (199882835.03, 262853899.49)	9032.34 (7956.99, 10335.49)	426075866.78 (372102935.64, 491506803.14)	10357.73 (9046.8, 11978.64)	0.03 (-0.1, 0.16)
Male	141360119.34 (123814477.61, 163181557.57)	5640.4 (4961.66, 6448.45)	265547736.7 (232101180.7, 305788424.9)	6494.95 (5683.8, 7492.08)	0.08 (-0.03, 0.2)
**Age**					
<5 years	538940.1 (365225.57, 778097.55)	86.93 (58.91, 125.51)	751125.17 (497361.96, 1094860.94)	114.12 (75.57, 166.35)	0.38 (0.18, 0.57)
5-9 years	8121421.42 (5576839.69, 11707121.29)	1391.77 (955.71, 2006.26)	12020620.43 (8071475.19, 17420599.17)	1749.59 (1174.8, 2535.55)	0.26 (0.1, 0.43)
10-14 years	23015308.68 (16010166.35, 31485325.25)	4296.44 (2988.74, 5877.61)	35509000 (24313893.9, 49348414.01)	5326.6 (3647.25, 7402.6)	0.24 (0.08, 0.4)
15-19 years	36867926.22 (28243931.29, 47474542.69)	7097.86 (5437.55, 9139.85)	54135313.36 (40762499.92, 70471310.76)	8675.81 (6532.66, 11293.84)	0.17 (0.01, 0.33)
20-24 years	42347096.62 (31825844.36, 56248623.8)	8605.6 (6467.51, 11430.6)	61791644.71 (45998330.37, 83277260.21)	10347.62 (7702.87, 13945.6)	0.06 (-0.12, 0.24)
25-29 years	40309229.03 (31372606.04, 51533184.59)	9106.97 (7087.94, 11642.77)	63174115.25 (48728060.95, 81815471.17)	10737.63 (8282.25, 13906.08)	0.05 (-0.12, 0.22)
30-34 years	36667834.1 (28722742.65, 45981316.42)	9513.67 (7452.27, 11930.11)	65893081.07 (51217932.2, 83719765.23)	10900.78 (8473.05, 13849.88)	0.02 (-0.13, 0.16)
35-39 years	34831197.58 (28161932.1, 42309514.11)	9888.36 (7995, 12011.41)	64432553.83 (51265860.43, 78937581.57)	11488.05 (9140.48, 14074.23)	0.05 (-0.09, 0.19)
40-44 years	29607566.04 (22934179.99, 36913923.25)	10334.89 (8005.46, 12885.27)	59408831.89 (45338986.63, 74206037.59)	11875.81 (9063.25, 14833.77)	0.02 (-0.12, 0.16)
45-49 years	24395734.83 (19471071.56, 30090871.22)	10506.55 (8385.64, 12959.28)	55653250.27 (44064484.43, 68641206.26)	11753.48 (9306.03, 14496.42)	-0.01 (-0.13, 0.1)
50-54 years	22321970.09 (18511980.49, 26898595.05)	10500.92 (8708.59, 12653.91)	51943463.18 (42942535.34, 62803688.3)	11674.71 (9651.68, 14115.63)	0.01 (-0.09, 0.11)
55-59 years	19485220.01 (16011194.29, 23573039.88)	10521.18 (8645.36, 12728.43)	46205510.04 (37969185.65, 55880700.6)	11676.08 (9594.77, 14120.99)	0.03 (-0.07, 0.12)
60-64 years	16968822.15 (13542378.25, 21421360.22)	10565.28 (8431.88, 13337.56)	37145860.1 (29660908.72, 46648482.42)	11606.35 (9267.65, 14575.47)	0.01 (-0.09, 0.1)
65-69 years	13077364.49 (10474436.57, 16600172.21)	10579.58 (8473.81, 13429.53)	31308302.11 (25135932.55, 39688828.2)	11350.08 (9112.43, 14388.24)	-0.01 (-0.09, 0.07)
70-74 years	8816120.05 (7109637.43, 10954504.6)	10413.42 (8397.76, 12939.23)	22603219.02 (18134535.27, 28277358.41)	10981.01 (8810.05, 13737.59)	-0.02 (-0.07, 0.04)
75-79 years	6328004.52 (5038472.15, 7866247.92)	10280.16 (8185.25, 12779.11)	14055415.96 (11172178.34, 17570851.53)	10657.38 (8471.19, 13322.92)	-0.01 (-0.04, 0.03)
80-84 years	3569301.34 (2850542.97, 4423636.34)	10089.63 (8057.86, 12504.65)	9007059.44 (7144276.09, 11212225.76)	10284.02 (8157.14, 12801.82)	-0.02 (-0.04, 0.01)
85-89 years	1492828.74 (1200248.31, 1854069.26)	9879.04 (7942.84, 12269.61)	4473139.4 (3601793.52, 5509365.22)	9783.39 (7877.63, 12049.76)	-0.08 (-0.1, -0.06)

90-94 years	408477.11 (317663.16, 517589.03)	9532.3 (7413.05, 12078.56)	1647751.64 (1271721.86, 2089977.95)	9210.78 (7108.81, 11682.79)	-0.13 (-0.15, -0.11)
95 plus years	92984.42 (65626.09, 124780.68)	9133.25 (6446.02, 12256.39)	464346.63 (326538.63, 623009.34)	8519.64 (5991.2, 11430.72)	-0.2 (-0.23, -0.17)
**YLDs**					
**Gender**					
Female	32446215.03 (22595517.72, 44036395)	1282.41 (895.34, 1742.19)	60515410.03 (42185258.17, 81930340.53)	1473.33 (1024.4, 1997.58)	0.01 (-0.13, 0.15)
Male	20225182.68 (14026437.49, 27777225.65)	803.91 (557.43, 1101.13)	38324596.39 (26565333.53, 52479486.02)	937.41 (649.59, 1283.14)	0.08 (-0.05, 0.21)
**Age**					
<5 years	67515.11 (39415.93, 103277.83)	10.89 (6.36, 16.66)	94534.54 (53622.95, 149193.43)	14.36 (8.15, 22.67)	0.4 (0.2, 0.59)
5-9 years	1047253.78 (613244.69, 1601142.26)	179.47 (105.09, 274.39)	1564342.74 (890225.89, 2438607.57)	227.69 (129.57, 354.94)	0.29 (0.12, 0.46)
10-14 years	3165514.53 (1903790.26, 4729335.12)	590.93 (355.4, 882.86)	4987658.17 (2971565.11, 7567368.81)	748.18 (445.76, 1135.16)	0.3 (0.13, 0.47)
15-19 years	5381305.68 (3411778.64, 8024295.83)	1036.02 (656.84, 1544.85)	8033246.59 (5039705.5, 12053327.75)	1287.42 (807.67, 1931.69)	0.19 (0.02, 0.37)
20-24 years	6300790.82 (4001301.18, 9372671.91)	1280.42 (813.13, 1904.67)	9221006.48 (5798139.63, 13761998.22)	1544.15 (970.96, 2304.58)	0.02 (-0.18, 0.21)
25-29 years	5891353.02 (3814910.98, 8670715.5)	1331.02 (861.89, 1958.95)	9242005.95 (5900590.72, 13620691.53)	1570.85 (1002.92, 2315.09)	-0.01 (-0.19, 0.18)
30-34 years	5294462.02 (3419725.41, 7773894.61)	1373.68 (887.27, 2016.98)	9521310.33 (6091087.37, 13963303.97)	1575.12 (1007.66, 2309.97)	-0.03 (-0.19, 0.12)
35-39 years	4998156.53 (3285956.96, 7027216.17)	1418.95 (932.86, 1994.98)	9300870.84 (6081837.83, 13173084.72)	1658.31 (1084.37, 2348.7)	0.02 (-0.14, 0.18)
40-44 years	4224160.29 (2665125.16, 6116840.35)	1474.5 (930.3, 2135.16)	8541284.94 (5321265, 12404317.74)	1707.4 (1063.72, 2479.62)	0 (-0.16, 0.15)
45-49 years	3463573.67 (2258231.43, 4901620.12)	1491.66 (972.56, 2110.99)	7937592.81 (5139846.33, 11229471.15)	1676.35 (1085.49, 2371.57)	-0.04 (-0.16, 0.09)
50-54 years	3150494.86 (2098971.09, 4367466.48)	1482.09 (987.42, 2054.59)	7354430.98 (4887106.35, 10216822.61)	1652.97 (1098.42, 2296.31)	-0.01 (-0.11, 0.1)
55-59 years	2728772.5 (1830943.16, 3821084.97)	1473.42 (988.63, 2063.22)	6495951 (4340561.12, 9039896.65)	1641.52 (1096.85, 2284.37)	0.01 (-0.09, 0.12)
60-64 years	2358726.7 (1580364.15, 3344159.67)	1468.61 (983.98, 2082.17)	5191497.26 (3469924.86, 7345474.09)	1622.1 (1084.19, 2295.12)	0 (-0.1, 0.1)
65-69 years	1799004.82 (1201484.08, 2526453.54)	1455.39 (972, 2043.9)	4318713.77 (2903689.18, 6022775.72)	1565.65 (1052.66, 2183.41)	0 (-0.08, 0.07)
70-74 years	1202396.61 (806725.37, 1665209.69)	1420.25 (952.89, 1966.91)	3085184.77 (2087047.53, 4266918.24)	1498.83 (1013.92, 2072.94)	0 (-0.05, 0.05)
75-79 years	855874.29 (578446.88, 1206679.85)	1390.41 (939.72, 1960.31)	1896024.66 (1294080.21, 2668240.38)	1437.64 (981.22, 2023.17)	0.01 (-0.01, 0.04)
80-84 years	478118.02 (326928.85, 675638.47)	1351.53 (924.16, 1909.88)	1198171.25 (813102.84, 1697448.46)	1368.04 (928.38, 1938.1)	-0.01 (-0.03, 0.01)
85-89 years	198027.66 (136249.69, 275693.35)	1310.48 (901.65, 1824.45)	584333.51 (400806.49, 815637.69)	1278.02 (876.62, 1783.91)	-0.1 (-0.13, -0.08)
90-94 years	53759.53 (36053.56, 76319.07)	1254.54 (841.35, 1781)	212545.78 (143128.9, 302163.61)	1188.11 (800.08, 1689.07)	-0.17 (-0.2, -0.14)
95 plus years	12137.28 (7462.32, 18126.24)	1192.17 (732.98, 1780.42)	59300.07 (36161.29, 88293.74)	1088.01 (663.47, 1619.98)	-0.25 (-0.29, -0.2)

Notes: ASR: age-standardized rates; YLDs: years lived with disability; EAPCs: estimated annual percentage changes.

Table S9. Prevalence and YLDs of substance use disorders and its temporal trends from 1990, 2021 by gender, and age in the worldwide.

	1990		2021		
	Number	ASR, per 100,000 persons	Number	ASR, per 100,000 persons	EAPC 95%CI
**Prevalence**					
**Gender**					
Female	4008774.15 (3293734.29, 4771654.68)	153.99 (128.53, 181.17)	7988655.62 (6896383, 9215842.89)	196.11 (168.4, 226.88)	0.56 (0.27, 0.85)
Male	4112039.37 (3496117.73, 4815001.11)	154.58 (132.54, 180.63)	8176220.31 (7238339.72, 9254557.11)	200.23 (176.76, 226.9)	0.46 (0.29, 0.63)
**Age**					
15-19 years	362227.65 (250991.3, 497586.43)	69.74 (48.32, 95.8)	539794.28 (402222.38, 704377.77)	86.51 (64.46, 112.88)	0.24 (-0.04, 0.51)
20-24 years	1575248.18 (1151673.02, 2123600.47)	320.12 (234.04, 431.55)	2427331.46 (1895535.22, 3118136.86)	406.48 (317.43, 522.16)	0.38 (0.16, 0.6)
25-29 years	1790047.17 (1387905.46, 2262293.92)	404.42 (313.57, 511.11)	3081386.2 (2537623.54, 3759825.03)	523.74 (431.32, 639.05)	0.54 (0.36, 0.73)
30-34 years	1296283.43 (1012834.51, 1661734.05)	336.33 (262.79, 431.15)	2697915.67 (2246890.9, 3291168.66)	446.32 (371.71, 544.46)	0.63 (0.46, 0.79)
35-39 years	939812.87 (747326.76, 1217532.64)	266.81 (212.16, 345.65)	2031387.27 (1682416.94, 2487428.49)	362.19 (299.97, 443.5)	0.66 (0.45, 0.87)
40-44 years	610769.07 (462550.3, 774905.25)	213.2 (161.46, 270.49)	1484656.53 (1223164.91, 1765534.15)	296.78 (244.51, 352.93)	0.6 (0.39, 0.82)
45-49 years	419513.73 (322196.88, 538215.99)	180.67 (138.76, 231.79)	1167532.07 (970653.38, 1403748.11)	246.57 (204.99, 296.46)	0.68 (0.52, 0.85)
50-54 years	368712.71 (274858.55, 487237.05)	173.45 (129.3, 229.21)	981724.71 (778053.47, 1208817.79)	220.65 (174.87, 271.69)	0.72 (0.56, 0.88)
55-59 years	268538.7 (199532.9, 345813.67)	145 (107.74, 186.72)	710363.11 (563876.52, 874885.7)	179.51 (142.49, 221.08)	0.66 (0.45, 0.87)
60-64 years	192787.93 (142247.13, 263825.08)	120.04 (88.57, 164.27)	428424.1 (334255.21, 551159.77)	133.86 (104.44, 172.21)	0.27 (0.1, 0.45)
65-69 years	123824.59 (94206.27, 159673.92)	100.17 (76.21, 129.18)	262215.53 (207105.86, 335624.02)	95.06 (75.08, 121.67)	-0.3 (-0.4, -0.21)
70-74 years	78920.3 (57725.77, 106288.56)	93.22 (68.18, 125.55)	153586.74 (116864.57, 201858.47)	74.61 (56.77, 98.07)	-0.91 (-0.98, -0.83)
75-79 years	51810.25 (39270.94, 68126.39)	84.17 (63.8, 110.67)	85583.29 (66487.45, 108339.96)	64.89 (50.41, 82.15)	-1.12 (-1.22, -1.03)
80-84 years	27074.83 (19300.15, 36121.15)	76.53 (54.56, 102.11)	57139.81 (43159.98, 72878.18)	65.24 (49.28, 83.21)	-0.78 (-0.91, -0.64)
85-89 years	11128.29 (8660.44, 14004.26)	73.64 (57.31, 92.68)	33773.5 (27746.61, 40813.19)	73.87 (60.69, 89.26)	-0.09 (-0.21, 0.03)
90-94 years	3256.09 (2539.79, 4137.54)	75.98 (59.27, 96.55)	16387.64 (13722.4, 19439.27)	91.61 (76.71, 108.66)	0.57 (0.45, 0.7)
95 plus years	857.72 (634.68, 1104.64)	84.25 (62.34, 108.5)	5674.03 (4555.49, 6895.57)	104.1 (83.58, 126.52)	0.51 (0.38, 0.64)
**YLDs**					

**Gender**					
Female	1646211.24 (1097683.12, 2178466.76)	62.98 (41.91, 82.52)	3226975.06 (2221519.52, 4183467.7)	79.37 (54.78, 103.57)	0.53 (0.25, 0.82)
Male	1728018.13 (1158454.86, 2289908.92)	64.69 (43.62, 84.19)	3409492.25 (2377710.31, 4420570.07)	83.55 (58.24, 108.57)	0.46 (0.29, 0.62)
**Age**					
15-19 years	155506.39 (94083.7, 228877.23)	29.94 (18.11, 44.06)	230302.22 (144879.14, 332712.36)	36.91 (23.22, 53.32)	0.22 (-0.05, 0.49)
20-24 years	670226.82 (412567.65, 980704.93)	136.2 (83.84, 199.29)	1022516.99 (655011.34, 1430910.99)	171.23 (109.69, 239.62)	0.36 (0.15, 0.58)
25-29 years	754315.38 (480914.08, 1039484.46)	170.42 (108.65, 234.85)	1287738.07 (877390.47, 1743357.34)	218.88 (149.13, 296.32)	0.52 (0.34, 0.71)
30-34 years	541523.33 (360514.46, 756799.21)	140.5 (93.54, 196.36)	1120136.95 (757363.18, 1519870.77)	185.31 (125.29, 251.43)	0.61 (0.45, 0.78)
35-39 years	389807.53 (258980.28, 552425.09)	110.66 (73.52, 156.83)	835588.69 (565861.05, 1147002.61)	148.98 (100.89, 204.51)	0.64 (0.44, 0.85)
40-44 years	251009.67 (165951.59, 350123.05)	87.62 (57.93, 122.21)	604763.94 (416100.65, 810364.3)	120.89 (83.18, 161.99)	0.58 (0.37, 0.79)
45-49 years	170834.24 (115946.77, 242206.21)	73.57 (49.93, 104.31)	471383.61 (321280.86, 625159.03)	99.55 (67.85, 132.03)	0.66 (0.5, 0.82)
50-54 years	148241.3 (91974.32, 208879.56)	69.74 (43.27, 98.26)	391589.99 (260737.13, 521693.36)	88.01 (58.6, 117.25)	0.7 (0.54, 0.85)
55-59 years	106595.59 (65970.92, 148081.11)	57.56 (35.62, 79.96)	279464.26 (186638.22, 370226.48)	70.62 (47.16, 93.56)	0.64 (0.44, 0.84)
60-64 years	75399.91 (47837.58, 108626.85)	46.95 (29.79, 67.63)	165843.11 (108675.52, 231359.88)	51.82 (33.96, 72.29)	0.25 (0.08, 0.42)
65-69 years	47409.57 (30792.1, 66573.26)	38.35 (24.91, 53.86)	99589.35 (66281.77, 138307.22)	36.1 (24.03, 50.14)	-0.32 (-0.42, -0.23)
70-74 years	29539.83 (18654.45, 44111.71)	34.89 (22.03, 52.1)	57165.42 (37236, 82544.32)	27.77 (18.09, 40.1)	-0.93 (-1, -0.85)
75-79 years	18926.78 (12485.52, 27214.82)	30.75 (20.28, 44.21)	31118.52 (20892.53, 43542.21)	23.6 (15.84, 33.02)	-1.14 (-1.23, -1.04)
80-84 years	9668.77 (6198.22, 13915.72)	27.33 (17.52, 39.34)	20297.14 (13307.32, 28630.19)	23.17 (15.19, 32.69)	-0.8 (-0.93, -0.66)
85-89 years	3851.06 (2533.85, 5359.55)	25.49 (16.77, 35.47)	11637.78 (8016.43, 15541.96)	25.45 (17.53, 33.99)	-0.1 (-0.22, 0.02)
90-94 years	1094.7 (739.72, 1486.7)	25.55 (17.26, 34.69)	5489.5 (3846.12, 7222.85)	30.69 (21.5, 40.38)	0.56 (0.44, 0.68)
95 plus years	278.5 (186.47, 384.75)	27.36 (18.32, 37.79)	1841.77 (1291.97, 2445.07)	33.79 (23.7, 44.86)	0.51 (0.38, 0.64)

Notes: ASR: age-standardized rates; YLDs: years lived with disability; EAPCs: estimated annual percentage changes.

Table S10. Prevalence and YLDs of infectious disorders and its temporal trends from 1990, 2021 by gender, and age in the worldwide.

	1990		2021		EAPC 95%CI
	Number	ASR, per 100,000 persons	Number	ASR, per 100,000 persons	
**Prevalence**					
**Gender**					
Female	1755922.63 (1514760.77, 2033827.82)	69.17 (58.78, 80.43)	2792712.95 (2321664.07, 3298954.96)	69.83 (59.26, 81.31)	0.07 (0.04, 0.09)
Male	1615285.52 (1402126.77, 1859332.86)	63.95 (54.41, 73.78)	2489384.19 (2081824.64, 2898608.03)	64.35 (54.64, 74.35)	0.04 (0.03, 0.05)
**Age**					
<5 years	1031112.14 (906804.4, 1136810.32)	166.33 (146.27, 183.37)	1059671.62 (930775.74, 1173640.07)	161 (141.42, 178.32)	-0.02 (-0.05, 0)
5-9 years	179811.32 (118666.67, 246947.58)	30.81 (20.34, 42.32)	213533.73 (143759.25, 293674.56)	31.08 (20.92, 42.74)	-0.06 (-0.11, -0.01)
10-14 years	132984.75 (89893.59, 189196.41)	24.83 (16.78, 35.32)	165760.11 (111364.08, 235853.05)	24.87 (16.71, 35.38)	-0.04 (-0.08, -0.01)
15-19 years	141152.08 (95214.76, 196152.44)	27.17 (18.33, 37.76)	169661.27 (114418.05, 236039.06)	27.19 (18.34, 37.83)	0.01 (-0.02, 0.04)
20-24 years	155533.85 (90484.03, 239529.28)	31.61 (18.39, 48.68)	189354.58 (110287.54, 292705.31)	31.71 (18.47, 49.02)	0.04 (0.02, 0.07)
25-29 years	158240.85 (97763.8, 242413.46)	35.75 (22.09, 54.77)	211748.19 (129749.58, 324624.83)	35.99 (22.05, 55.18)	0.05 (0.03, 0.07)
30-34 years	150846.89 (106325.14, 209004.3)	39.14 (27.59, 54.23)	238938.9 (167290.14, 330247.14)	39.53 (27.68, 54.63)	0.06 (0.03, 0.08)
35-39 years	151841.18 (106178.04, 211163.71)	43.11 (30.14, 59.95)	242861.87 (168761.49, 337418.16)	43.3 (30.09, 60.16)	0.06 (0.03, 0.1)
40-44 years	135349.92 (82955.4, 204534.82)	47.25 (28.96, 71.4)	237716.61 (146232.98, 359680.87)	47.52 (29.23, 71.9)	0.09 (0.05, 0.12)
45-49 years	132656.5 (86319.1, 195405.04)	57.13 (37.18, 84.16)	274239.71 (177828.84, 406584.8)	57.92 (37.56, 85.87)	0.09 (0.07, 0.12)
50-54 years	156746.66 (110257.1, 211929.22)	73.74 (51.87, 99.7)	338956.55 (236969.73, 456509.37)	76.18 (53.26, 102.6)	0.11 (0.07, 0.14)
55-59 years	171019.74 (113161.24, 243175.44)	92.34 (61.1, 131.3)	375123.63 (247569.36, 535820.58)	94.79 (62.56, 135.4)	0.09 (0.04, 0.14)
60-64 years	177648.89 (107978.02, 262340.61)	110.61 (67.23, 163.34)	362152.36 (217981.69, 536556.99)	113.16 (68.11, 167.65)	0.09 (0.04, 0.14)
65-69 years	162086.91 (105418.94, 231980.31)	131.13 (85.28, 187.67)	367836 (237665.82, 528126.38)	133.35 (86.16, 191.46)	0.1 (0.06, 0.14)
70-74 years	126253.79 (91707.18, 166957.59)	149.13 (108.32, 197.21)	308873.45 (224000.43, 407814.98)	150.06 (108.82, 198.12)	0.07 (0.03, 0.12)
75-79 years	101252.76 (70454.41, 136210.95)	164.49 (114.46, 221.28)	221589.4 (152811.1, 298452.1)	168.02 (115.87, 226.3)	0.05 (0.03, 0.08)
80-84 years	64790.79 (40901.65, 94464.58)	183.15 (115.62, 267.03)	161040.3 (100766.94, 237050.75)	183.87 (115.05, 270.66)	0.05 (0.02, 0.09)
85-89 years	30326.59 (19054.73, 44459.06)	200.69 (126.1, 294.22)	92145.92 (58893.36, 135148.97)	201.54 (128.81, 295.59)	0.1 (0.06, 0.14)

90-94 years	9209.26 (6045.63, 14324.91)	214.91 (141.08, 334.29)	38363 (25029.22, 60928.82)	214.45 (139.91, 340.59)	0.16 (0.1, 0.22)
95 plus years	2343.28 (1188.68, 4208.89)	230.17 (116.76, 413.41)	12529.93 (6260.91, 22671.7)	229.89 (114.87, 415.97)	0.15 (0.07, 0.23)
**YLDs**					
**Gender**					
Female	67475.26 (39542.83, 104927.04)	2.81 (1.62, 4.39)	120739.63 (68396.19, 187130.47)	2.84 (1.62, 4.43)	0.08 (0.05, 0.11)
Male	59069.74 (34614.31, 88969.31)	2.56 (1.49, 3.85)	104146.7 (60183.33, 157322.24)	2.58 (1.5, 3.92)	0.05 (0.04, 0.07)
**Age**					
<5 years	7066.92 (3242.47, 13780.11)	1.14 (0.52, 2.22)	7332.09 (3343.96, 14402.34)	1.11 (0.51, 2.19)	-0.01 (-0.03, 0.01)
5-9 years	4723.74 (2420.18, 8215.65)	0.81 (0.41, 1.41)	5550.81 (2884.54, 9923.82)	0.81 (0.42, 1.44)	-0.04 (-0.07, -0.02)
10-14 years	6261.08 (3196.64, 11046.8)	1.17 (0.6, 2.06)	7676.56 (3991.98, 13676.6)	1.15 (0.6, 2.05)	-0.03 (-0.06, -0.01)
15-19 years	7448.93 (3793.8, 12580.99)	1.43 (0.73, 2.42)	8840.09 (4480.26, 15285.72)	1.42 (0.72, 2.45)	0.01 (-0.02, 0.04)
20-24 years	8397.44 (4076.68, 14700.4)	1.71 (0.83, 2.99)	10132.74 (4842.23, 17629.42)	1.7 (0.81, 2.95)	0.04 (0.01, 0.07)
25-29 years	8630.14 (4162.88, 14901.81)	1.95 (0.94, 3.37)	11494.69 (5701, 19690.1)	1.95 (0.97, 3.35)	0.05 (0.03, 0.07)
30-34 years	8256.84 (4434.6, 13508.33)	2.14 (1.15, 3.5)	13084.75 (6981.45, 21332.31)	2.16 (1.15, 3.53)	0.06 (0.03, 0.08)
35-39 years	8324.59 (4563.25, 13732.1)	2.36 (1.3, 3.9)	13270.63 (7354.01, 21733.89)	2.37 (1.31, 3.88)	0.06 (0.03, 0.1)
40-44 years	7391.95 (3679.02, 12825.79)	2.58 (1.28, 4.48)	12932.7 (6447.65, 22452.55)	2.59 (1.29, 4.49)	0.09 (0.05, 0.13)
45-49 years	7219.02 (3783.73, 12075.39)	3.11 (1.63, 5.2)	14891.4 (7783.73, 25162.22)	3.14 (1.64, 5.31)	0.09 (0.07, 0.12)
50-54 years	8510.35 (4544.72, 13300.72)	4 (2.14, 6.26)	18348.26 (9933.44, 28829.57)	4.12 (2.23, 6.48)	0.11 (0.07, 0.14)
55-59 years	9210.01 (4660.04, 14842.24)	4.97 (2.52, 8.01)	20188.23 (10319.91, 32569.53)	5.1 (2.61, 8.23)	0.09 (0.04, 0.14)
60-64 years	9495.5 (4381.07, 15876.8)	5.91 (2.73, 9.89)	19324.63 (8846.73, 32219.19)	6.04 (2.76, 10.07)	0.09 (0.04, 0.14)
65-69 years	8546.27 (4098.74, 13817.44)	6.91 (3.32, 11.18)	19377.31 (9189.03, 31256.89)	7.02 (3.33, 11.33)	0.1 (0.06, 0.14)
70-74 years	6564.89 (3401.83, 10367.62)	7.75 (4.02, 12.25)	16042.1 (8379.2, 25486.27)	7.79 (4.07, 12.38)	0.07 (0.03, 0.11)
75-79 years	5185.56 (2729.96, 8648.31)	8.42 (4.43, 14.05)	11327.08 (5967.98, 19160.07)	8.59 (4.53, 14.53)	0.05 (0.02, 0.07)
80-84 years	3260.8 (1656.12, 5769.09)	9.22 (4.68, 16.31)	8091.32 (4031.58, 14382.94)	9.24 (4.6, 16.42)	0.05 (0.01, 0.08)
85-89 years	1494.71 (780.02, 2685.34)	9.89 (5.16, 17.77)	4536.77 (2356.52, 7994.72)	9.92 (5.15, 17.49)	0.1 (0.06, 0.14)
90-94 years	445.37 (229.3, 829.59)	10.39 (5.35, 19.36)	1852.24 (955.43, 3472.72)	10.35 (5.34, 19.41)	0.16 (0.09, 0.22)
95 plus years	110.89 (48.79, 229.47)	10.89 (4.79, 22.54)	591.94 (255.18, 1259.18)	10.86 (4.68, 23.1)	0.14 (0.06, 0.22)

Notes: ASR: age-standardized rates; YLDs: years lived with disability; EAPCs: estimated annual percentage changes.

Table S11. Prevalence and YLDs of neoplasms and its temporal trends from 1990, 2021 by gender, and age in the worldwide.

	1990		2021		EAPC 95%CI
	Number	ASR, per 100,000 persons	Number	ASR, per 100,000 persons	
**Prevalence**					
**Gender**					
Female	49596801.12 (44170709.33, 56957173.73)	2105.3 (1893.72, 2373.49)	93954059.09 (86011960.6, 103235681.03)	2173.64 (1983.27, 2393.51)	0.11 (0.07, 0.15)
Male	26700971.33 (24703229.86, 28943005.83)	1304.89 (1220.52, 1403.94)	61124769.52 (57101254.97, 65395160.57)	1507.28 (1410.94, 1609.1)	0.45 (0.4, 0.51)
**Age**					
<5 years	1513931.82 (1258848.26, 1810060.28)	244.21 (203.06, 291.97)	1493915.1 (1224933.2, 1836104.68)	226.98 (186.11, 278.97)	0.06 (-0.1, 0.22)
5-9 years	1702365.98 (1231516.86, 2418079.55)	291.74 (211.05, 414.39)	1866836.34 (1369077.22, 2574979.73)	271.72 (199.27, 374.79)	-0.17 (-0.27, -0.06)
10-14 years	1987205.48 (1285593.9, 3043638.16)	370.97 (239.99, 568.18)	2356213.62 (1589477.01, 3461152.6)	353.45 (238.43, 519.2)	-0.19 (-0.25, -0.13)
15-19 years	2853766.18 (1863436.36, 4481538.65)	549.41 (358.75, 862.79)	3228379.7 (2219867.74, 4768855.78)	517.39 (355.76, 764.26)	-0.12 (-0.18, -0.05)
20-24 years	3762041.23 (2321813.75, 5832490.61)	764.51 (471.83, 1185.25)	4304358.81 (2856959.34, 6307410.74)	720.81 (478.43, 1056.24)	-0.05 (-0.17, 0.08)
25-29 years	4430857.69 (3055904.64, 6353481.91)	1001.05 (690.41, 1435.43)	5596732.13 (4059180.87, 7515431.34)	951.27 (689.93, 1277.39)	-0.08 (-0.16, 0)
30-34 years	4979066.95 (3467698.9, 7373056.72)	1291.85 (899.71, 1912.98)	7688069.25 (5613438.42, 10539224.01)	1271.85 (928.64, 1743.52)	-0.11 (-0.16, -0.06)
35-39 years	5576377.37 (4362292.67, 7446587.05)	1583.1 (1238.43, 2114.04)	8898781.33 (7260290.33, 11442975.63)	1586.61 (1294.48, 2040.23)	-0.03 (-0.08, 0.02)
40-44 years	5484448.41 (4237924.21, 7510166.27)	1914.42 (1479.3, 2621.52)	9925943.72 (7956931.56, 12894493.45)	1984.19 (1590.59, 2577.61)	0.09 (0.03, 0.14)
45-49 years	5265138.79 (4228642.1, 6704198.49)	2267.55 (1821.16, 2887.31)	11467212.01 (9535231.37, 14099717.35)	2421.77 (2013.76, 2977.74)	0.13 (0.06, 0.19)
50-54 years	6023251.53 (4827598.87, 7808436.86)	2833.52 (2271.05, 3673.32)	13590335 (11369588.72, 16849977.49)	3054.54 (2555.41, 3787.17)	0.18 (0.11, 0.25)
55-59 years	6344859.37 (5492649.72, 7512367.16)	3425.95 (2965.79, 4056.35)	15212243.22 (13435615, 17466221.29)	3844.12 (3395.16, 4413.69)	0.4 (0.33, 0.48)
60-64 years	6917833.88 (5989407.2, 8204869.7)	4307.25 (3729.18, 5108.59)	15880283.7 (14132478.31, 18270749.31)	4961.85 (4415.74, 5708.76)	0.53 (0.47, 0.59)
65-69 years	6611474 (5970477.08, 7549313.26)	5348.68 (4830.11, 6107.39)	16759041.63 (15427904.53, 18570118.5)	6075.59 (5593.02, 6732.15)	0.49 (0.41, 0.56)
70-74 years	5082272.75 (4542180.18, 5884729.71)	6003.08 (5365.13, 6950.92)	15063734.65 (13703110.64, 17124543.77)	7318.2 (6657.19, 8319.38)	0.48 (0.41, 0.54)
75-79 years	4120972.68 (3758940.74, 4559817.47)	6694.73 (6106.59, 7407.65)	10140315.72 (9365163.66, 11036045.47)	7688.79 (7101.04, 8367.97)	0.4 (0.36, 0.43)
80-84 years	2310464.06 (2086768.22, 2583755.75)	6531.18 (5898.84, 7303.71)	6552068.42 (5881002.95, 7219370.47)	7480.98 (6714.77, 8242.88)	0.42 (0.34, 0.51)
85-89 years	990840.8 (895258.75, 1097965.81)	6557.05 (5924.52, 7265.97)	3480866.01 (3086842.36, 3795898.28)	7613.14 (6751.36, 8302.16)	0.65 (0.55, 0.74)

90-94 years	271126.99 (236331.43, 310540.12)	6327.07 (5515.08, 7246.83)	1236966.29 (1116049.65, 1365232.38)	6914.53 (6238.62, 7631.53)	0.6 (0.49, 0.71)
95 plus years	69476.48 (57375.16, 83280.43)	6824.22 (5635.59, 8180.09)	336531.97 (294422.22, 383594.88)	6174.55 (5401.94, 7038.04)	-0.09 (-0.28, 0.11)
**YLDs**					
**Gender**					
Female	1850269.73 (1368415.44, 2407806.59)	84.42 (62.59, 109.61)	4008418.84 (2960702.44, 5259587.61)	89.23 (65.8, 117.1)	0.16 (0.12, 0.19)
Male	1752049.98 (1305537.71, 2247900.83)	94.88 (71.04, 121.55)	3990163.06 (3003335.17, 5148093.48)	99.56 (75.07, 128.25)	0.13 (0.06, 0.2)
**Age**					
<5 years	51805.87 (35469.78, 73017.32)	8.36 (5.72, 11.78)	45107.15 (29797.4, 67551.83)	6.85 (4.53, 10.26)	-0.23 (-0.42, -0.04)
5-9 years	25947.66 (18185.23, 35426.58)	4.45 (3.12, 6.07)	27273.04 (19311.7, 37725.55)	3.97 (2.81, 5.49)	-0.27 (-0.36, -0.17)
10-14 years	22462.59 (15789.07, 30351.35)	4.19 (2.95, 5.67)	28375.48 (20082.49, 38850.33)	4.26 (3.01, 5.83)	0.07 (0.02, 0.13)
15-19 years	30829.31 (21716.96, 41507.75)	5.94 (4.18, 7.99)	39056.83 (27374.23, 53829.55)	6.26 (4.39, 8.63)	0.1 (0.07, 0.14)
20-24 years	38485.9 (26956.18, 51594.77)	7.82 (5.48, 10.48)	54151.36 (38071.4, 73723.42)	9.07 (6.38, 12.35)	0.45 (0.42, 0.49)
25-29 years	61298.59 (43115.26, 82743.99)	13.85 (9.74, 18.69)	93804.61 (65670.67, 128319.96)	15.94 (11.16, 21.81)	0.55 (0.51, 0.59)
30-34 years	94527.98 (66545.17, 127631.19)	24.53 (17.27, 33.11)	166541.56 (117527.48, 227016.86)	27.55 (19.44, 37.56)	0.34 (0.27, 0.42)
35-39 years	143006.71 (101693.35, 192140.71)	40.6 (28.87, 54.55)	240359.1 (170635.75, 326162.3)	42.85 (30.42, 58.15)	0.07 (0, 0.14)
40-44 years	185613.73 (133035.36, 245570.94)	64.79 (46.44, 85.72)	335118.56 (239900.45, 452489.13)	66.99 (47.96, 90.45)	0.02 (-0.03, 0.06)
45-49 years	220981.65 (160633.09, 289413.7)	95.17 (69.18, 124.64)	467659.85 (337129.17, 629137.45)	98.77 (71.2, 132.87)	0.06 (-0.02, 0.14)
50-54 years	307851.99 (224205.72, 398997.96)	144.82 (105.47, 187.7)	665268.44 (480848.57, 880323.18)	149.52 (108.07, 197.86)	0.05 (-0.04, 0.14)
55-59 years	384134.58 (281267.26, 494674.77)	207.42 (151.87, 267.1)	860162.45 (630246.01, 1120391.13)	217.36 (159.26, 283.12)	0.19 (0.1, 0.28)
60-64 years	468042.13 (346492.07, 601260.48)	291.42 (215.74, 374.36)	981734.34 (724298.58, 1274646.45)	306.75 (226.31, 398.27)	0.28 (0.2, 0.35)
65-69 years	484756.96 (361197.69, 624203.44)	392.17 (292.21, 504.98)	1120201.54 (836610.33, 1447601.06)	406.1 (303.29, 524.79)	0.16 (0.11, 0.21)
70-74 years	401800.67 (303355.53, 515202.25)	474.6 (358.32, 608.55)	1073736.58 (810534.41, 1386120.39)	521.64 (393.77, 673.4)	0.12 (0.05, 0.19)
75-79 years	352925.48 (267731.49, 448465.17)	573.35 (434.94, 728.55)	790983.14 (600555.54, 1016945.21)	599.75 (455.37, 771.09)	0.08 (0, 0.16)
80-84 years	207826.02 (155729.02, 262466.05)	587.48 (440.21, 741.93)	549828.17 (400890.53, 705904.31)	627.78 (457.73, 805.98)	0.17 (0.06, 0.27)
85-89 years	92282.71 (67508.55, 116913.94)	610.7 (446.75, 773.7)	321315.39 (234033.34, 412230.26)	702.76 (511.86, 901.61)	0.5 (0.38, 0.61)
90-94 years	23086.64 (17031.79, 29239.58)	538.75 (397.46, 682.34)	111496.57 (81234.72, 141994.29)	623.26 (454.09, 793.74)	0.61 (0.54, 0.68)
95 plus years	4652.54 (3404.28, 6025.55)	456.99 (334.38, 591.85)	26407.74 (19334.27, 33748.82)	484.52 (354.74, 619.21)	0.24 (0.15, 0.34)

Notes: ASR: age-standardized rates; YLDs: years lived with disability; EAPCs: estimated annual percentage changes.

Table S12. Prevalence and YLDs of neck pain and its temporal trends from 1990, 2021 by gender, and age in the worldwide.

	1990		2021		EAPC 95%CI
	Number	ASR, per 100,000 persons	Number	ASR, per 100,000 persons	
**Prevalence**					
**Gender**					
Female	67256733.43 (52138492.46, 83455855.07)	2835.08 (2224.58, 3517.55)	123424737.3 (97219218.2, 151771401.46)	2886.98 (2260.2, 3563.8)	0 (-0.08, 0.07)
Male	47344717.6 (36696133.18, 58921351.01)	2032.53 (1599.18, 2499.59)	82604891.24 (64494827.34, 101493796.81)	1992.59 (1564.97, 2449.69)	-0.11 (-0.18, -0.03)
**Age**					
5-9 years	557128.24 (268489.53, 1055769.22)	95.48 (46.01, 180.93)	669471.26 (322825.55, 1263640.03)	97.44 (46.99, 183.92)	0.12 (0.1, 0.14)
10-14 years	2656335.33 (1455274.26, 4318237.23)	495.88 (271.67, 806.12)	3336182.1 (1835634.61, 5413796.04)	500.45 (275.36, 812.11)	0.08 (0.02, 0.14)
15-19 years	5273779.38 (2915903.67, 8844790.35)	1015.31 (561.37, 1702.81)	6314842.31 (3465171.93, 10691655.84)	1012.03 (555.33, 1713.46)	-0.01 (-0.1, 0.07)
20-24 years	8161934.71 (4319917.88, 14289541.33)	1658.63 (877.88, 2903.86)	9698585.05 (5126948.72, 16991536.95)	1624.12 (858.56, 2845.4)	-0.08 (-0.14, -0.02)
25-29 years	9850128.77 (5319146.54, 16456988.37)	2225.42 (1201.74, 3718.09)	12562355.33 (6766317.26, 21047198.28)	2135.21 (1150.06, 3577.37)	-0.13 (-0.2, -0.06)
30-34 years	10573911.9 (6479332.66, 16507871.51)	2743.46 (1681.1, 4283.06)	15908718.47 (9631817.9, 24924884.48)	2631.8 (1593.41, 4123.36)	-0.19 (-0.3, -0.09)
35-39 years	11829111.45 (6823257.21, 18535110.45)	3358.21 (1937.08, 5262)	18358351.22 (10561803.29, 28642857.73)	3273.21 (1883.12, 5106.9)	-0.19 (-0.3, -0.08)
40-44 years	11920803.58 (7190318.28, 18323650.45)	4161.11 (2509.87, 6396.1)	20816329.92 (12531966.83, 32372495.21)	4161.18 (2505.14, 6471.25)	-0.1 (-0.19, -0.01)
45-49 years	11099292.26 (6721058, 17473139.68)	4780.15 (2894.57, 7525.18)	23066405.16 (13993896.28, 36605021.3)	4871.42 (2955.39, 7730.66)	0 (-0.07, 0.06)
50-54 years	10276616.03 (6062714.09, 15884638.04)	4834.43 (2852.08, 7472.61)	22153485.66 (12990127.24, 34350747.79)	4979.17 (2919.64, 7720.61)	0.04 (-0.01, 0.09)
55-59 years	9193922.23 (5286473.61, 15079009.15)	4964.32 (2854.47, 8142.02)	20434841.38 (11884191.71, 33757857.86)	5163.86 (3003.12, 8530.57)	0.04 (-0.02, 0.1)
60-64 years	7701052.27 (4624112.79, 12224519.76)	4794.9 (2879.11, 7611.34)	15741655.56 (9327810.98, 24915706.34)	4918.53 (2914.51, 7784.99)	0.03 (-0.04, 0.11)
65-69 years	5653979.26 (3350563.28, 8677986.68)	4574.07 (2710.6, 7020.49)	12970720.12 (7578195.85, 19872104.27)	4702.23 (2747.29, 7204.16)	0.02 (-0.06, 0.11)
70-74 years	4196130.29 (2398894.48, 6686573.54)	4956.38 (2833.52, 7898.04)	10292675.61 (5807028.54, 16276746.24)	5000.35 (2821.15, 7907.5)	0 (-0.09, 0.1)
75-79 years	3085440.26 (1738160.47, 5024064.77)	5012.45 (2823.73, 8161.84)	6600448.16 (3689602.59, 10748561.33)	5004.72 (2797.6, 8149.99)	0 (-0.09, 0.09)
80-84 years	1700970.89 (1077184.81, 2744696.34)	4808.27 (3044.97, 7758.66)	4204816.98 (2653813.5, 6787459.24)	4800.95 (3030.05, 7749.74)	0.01 (-0.06, 0.08)
85-89 years	654047.79 (404078.33, 1041012.48)	4328.27 (2674.05, 6889.07)	1960603.99 (1212746.64, 3143725.03)	4288.12 (2652.45, 6875.77)	-0.04 (-0.1, 0.03)
90-94 years	173208.56 (96855.09, 286417.26)	4042.03 (2260.23, 6683.89)	709881.69 (399187.18, 1177900.56)	3968.18 (2231.42, 6584.36)	-0.08 (-0.13, -0.03)

95 plus years	43657.83 (24848.71, 72864.51)	4288.22 (2440.73, 7157.01)	229258.56 (131009.92, 386827.94)	4206.34 (2403.72, 7097.36)	-0.08 (-0.1, -0.06)
**YLDs**					
**Gender**					
Female	6668697.18 (4447191.13, 9522447.88)	280.1 (187.38, 399.33)	12143076.07 (8136076.28, 17101009.43)	284.68 (190.15, 401.71)	0 (-0.08, 0.07)
Male	4773659.03 (3161758.48, 6799981.05)	203.35 (135.34, 287.77)	8272420.49 (5526592.87, 11818471.25)	199.38 (133.59, 284.34)	-0.1 (-0.18, -0.02)
**Age**					
5-9 years	58871.07 (25208.93, 117188.58)	10.09 (4.32, 20.08)	70773.85 (31013.77, 142138.55)	10.3 (4.51, 20.69)	0.12 (0.11, 0.14)
10-14 years	277703.25 (131311.14, 488277.59)	51.84 (24.51, 91.15)	348740.12 (165452.28, 617880.08)	52.31 (24.82, 92.69)	0.09 (0.02, 0.15)
15-19 years	548000.3 (256592.29, 1034865.69)	105.5 (49.4, 199.23)	655849.39 (306171.5, 1225013.51)	105.11 (49.07, 196.32)	-0.01 (-0.09, 0.07)
20-24 years	843275.17 (425160.27, 1604167.43)	171.37 (86.4, 325.99)	1001053.89 (502942.63, 1895791.73)	167.64 (84.22, 317.47)	-0.07 (-0.14, -0.01)
25-29 years	1009090.31 (490667.86, 1824834.91)	227.98 (110.86, 412.28)	1286333.9 (624028.16, 2323791.98)	218.64 (106.07, 394.97)	-0.13 (-0.2, -0.05)
30-34 years	1075552.49 (579155.66, 1834847.43)	279.06 (150.27, 476.06)	1618122.57 (874538.97, 2718153.64)	267.69 (144.68, 449.67)	-0.19 (-0.29, -0.09)
35-39 years	1196419.04 (595302.08, 2076018.97)	339.66 (169, 589.37)	1855342.02 (918743.25, 3235585.84)	330.8 (163.81, 576.89)	-0.19 (-0.29, -0.08)
40-44 years	1199986.33 (647016.57, 1948113.26)	418.87 (225.85, 680.01)	2092929.57 (1146480.62, 3407446.12)	418.38 (229.18, 681.15)	-0.1 (-0.18, -0.01)
45-49 years	1111015.95 (600361.39, 1935796)	478.48 (258.56, 833.69)	2308368.38 (1240464.25, 4074636.35)	487.51 (261.98, 860.53)	0 (-0.06, 0.07)
50-54 years	1024207.84 (541697.72, 1804900.3)	481.82 (254.83, 849.08)	2207718.51 (1169816.03, 3939920.85)	496.2 (262.93, 885.53)	0.05 (-0.01, 0.1)
55-59 years	905526.28 (479800.38, 1627114.42)	488.95 (259.07, 878.57)	2011648.62 (1065294.3, 3647226.43)	508.34 (269.2, 921.65)	0.04 (-0.01, 0.1)
60-64 years	746824.66 (403846.78, 1252060.27)	464.99 (251.45, 779.57)	1524242.89 (827906.78, 2533943.9)	476.25 (258.68, 791.74)	0.04 (-0.04, 0.11)
65-69 years	540036.86 (291688.87, 864201.32)	436.89 (235.98, 699.14)	1236684.77 (664456.15, 1969168.69)	448.33 (240.88, 713.88)	0.02 (-0.06, 0.11)
70-74 years	392629.32 (210913.66, 648576.16)	463.77 (249.13, 766.08)	961408.08 (501367.92, 1595830.9)	467.07 (243.57, 775.28)	0 (-0.09, 0.1)
75-79 years	283305.17 (152094.15, 468162.23)	460.24 (247.08, 760.55)	604524.26 (319821.68, 1014253.11)	458.37 (242.5, 769.05)	-0.01 (-0.09, 0.08)
80-84 years	153580.04 (83565.28, 257972.77)	434.14 (236.22, 729.23)	378802.83 (205061.01, 637132.19)	432.51 (234.13, 727.46)	0 (-0.07, 0.08)
85-89 years	57750.6 (31503.2, 95662.97)	382.17 (208.48, 633.07)	172698.93 (93831.54, 286787.17)	377.72 (205.22, 627.24)	-0.04 (-0.11, 0.03)
90-94 years	14918.55 (7924.56, 25077.99)	348.14 (184.93, 585.23)	61043.31 (32255.08, 101580.7)	341.23 (180.3, 567.83)	-0.08 (-0.13, -0.03)
95 plus years	3662.99 (1969.89, 6348.27)	359.79 (193.49, 623.55)	19210.65 (10283.64, 33524.86)	352.47 (188.68, 615.1)	-0.08 (-0.11, -0.05)

Notes: ASR: age-standardized rates; YLDs: years lived with disability; EAPCs: estimated annual percentage changes.

Table S13. Prevalence and YLDs of low back pain and its temporal trends from 1990, 2021 by gender, and age in the worldwide.

	1990		2021		EAPC 95%CI
	Number	ASR, per 100,000 persons	Number	ASR, per 100,000 persons	
**Prevalence**					
**Gender**					
Female	242102829.97 (214937265.43, 271065504.4)	10272.63 (9053.81, 11491.92)	396747971.85 (348340471.06, 442511662.33)	9212.46 (8122.92, 10285.14)	-0.28 (-0.32, -0.24)
Male	144628530.75 (126723498.9, 163120515.09)	6393.26 (5615.58, 7160.79)	232090503.25 (203351349.85, 259931952.95)	5640.23 (4965.63, 6300.61)	-0.36 (-0.38, -0.34)
**Age**					
5-9 years	2595081.92 (1431890.17, 4069487.91)	444.72 (245.38, 697.39)	2958718.98 (1659796.35, 4628650.81)	430.64 (241.58, 673.7)	-0.07 (-0.1, -0.04)
10-14 years	11920254.31 (8797881.35, 15886526.32)	2225.25 (1642.37, 2965.66)	14178911.51 (10531790.9, 18925844.97)	2126.93 (1579.84, 2839.01)	-0.15 (-0.17, -0.14)
15-19 years	21356546.42 (15422303.16, 28134066.81)	4111.59 (2969.12, 5416.4)	24297914.35 (17619299.58, 31876412.23)	3894.02 (2823.7, 5108.56)	-0.23 (-0.26, -0.2)
20-24 years	24891378.97 (17932170.89, 33859070.82)	5058.32 (3644.1, 6880.7)	28264042.52 (20432758.35, 38206774.16)	4733.09 (3421.67, 6398.1)	-0.23 (-0.28, -0.19)
25-29 years	27605243.82 (19871555.41, 36872089.65)	6236.79 (4489.53, 8330.42)	32974313.49 (23808036.56, 44101022.31)	5604.61 (4046.62, 7495.8)	-0.24 (-0.3, -0.17)
30-34 years	30234754.91 (21708141.62, 40681784.71)	7844.57 (5632.3, 10555.11)	40469160.01 (29368914.76, 54200101.91)	6694.87 (4858.54, 8966.4)	-0.33 (-0.43, -0.23)
35-39 years	34265988.5 (25575535.51, 44820626.15)	9727.9 (7260.73, 12724.3)	47385924.27 (35227212.26, 62200741.93)	8448.71 (6280.86, 11090.12)	-0.35 (-0.44, -0.27)
40-44 years	33481062.26 (24247395, 44165412.97)	11686.98 (8463.86, 15416.49)	51400902.6 (37564314.27, 67867420.84)	10275.03 (7509.1, 13566.68)	-0.38 (-0.44, -0.31)
45-49 years	30722252.97 (22260318.71, 40895295.44)	13231.2 (9586.89, 17612.44)	55462912.83 (40506735.33, 74148989.83)	11713.28 (8554.67, 15659.61)	-0.37 (-0.4, -0.33)
50-54 years	32652367.14 (23553165.99, 44296273.33)	15360.65 (11080.12, 20838.3)	59765617.54 (43183511.24, 81457391.18)	13432.8 (9705.84, 18308.2)	-0.36 (-0.39, -0.33)
55-59 years	31259590.35 (22844127.39, 41798217.78)	16878.83 (12334.84, 22569.24)	58963608.82 (43259709.18, 78865827.03)	14900.03 (10931.68, 19929.3)	-0.32 (-0.35, -0.28)
60-64 years	30173774.29 (21789776.22, 39882727.97)	18787.07 (13566.95, 24832.15)	53862394.06 (39362646.33, 71471627.77)	16829.48 (12298.99, 22331.54)	-0.31 (-0.34, -0.27)
65-69 years	25720212.45 (18961992.76, 34271717.13)	20807.64 (15340.24, 27725.81)	50825854.97 (37740718.34, 67494985.35)	18425.7 (13682, 24468.7)	-0.34 (-0.37, -0.31)
70-74 years	19390757.1 (14169188.38, 25408844.85)	22903.96 (16736.35, 30012.4)	42173146.01 (30943169.51, 54775716.98)	20488.39 (15032.69, 26610.92)	-0.35 (-0.37, -0.33)
75-79 years	16076198.91 (12115414.94, 20992687.44)	26116.59 (19682.1, 34103.67)	29508892.57 (22168667.55, 38524820.28)	22374.82 (16809.17, 29211.05)	-0.35 (-0.39, -0.31)
80-84 years	9326697.38 (7069414.12, 11888955.95)	26364.53 (19983.69, 33607.48)	20648664.52 (15816086.54, 26419482.13)	23576.09 (18058.39, 30165.06)	-0.31 (-0.35, -0.27)
85-89 years	3886562.85 (2835673.05, 5138068.47)	25719.97 (18765.53, 34002.01)	10750479.26 (7853606.89, 14192771.5)	23512.81 (17176.94, 31041.59)	-0.23 (-0.26, -0.2)
90-94 years	973058.89 (729208.1, 1265429.79)	22707.5 (17016.95, 29530.32)	3888465.42 (2935704.02, 5042619.87)	21736.17 (16410.32, 28187.79)	-0.14 (-0.15, -0.12)

95 plus years	199577.28 (142258.77, 268595.42)	19603.18 (13973.15, 26382.38)	1058551.36 (766407.14, 1412141.66)	19421.86 (14061.72, 25909.39)	-0.05 (-0.08, -0.02)
**YLDs**					
**Gender**					
Female	26974140.37 (19377113.2, 36203736.44)	1142.28 (817.03, 1533.18)	43934954.85 (31447685.06, 58945142.75)	1021.52 (732.39, 1370.45)	-0.28 (-0.32, -0.24)
Male	16412085.4 (11713623.21, 22118911.42)	720.6 (512.4, 974.5)	26222007.33 (18702285.02, 35396347.63)	635.48 (453.91, 854.29)	-0.35 (-0.38, -0.33)
**Age**					
5-9 years	282652.34 (148420.25, 483109.7)	48.44 (25.43, 82.79)	322998.54 (172035.02, 546268.46)	47.01 (25.04, 79.51)	-0.06 (-0.09, -0.04)
10-14 years	1291278.94 (809976.07, 1918014.42)	241.05 (151.2, 358.05)	1537404.82 (970907.84, 2280860.7)	230.62 (145.64, 342.14)	-0.15 (-0.16, -0.13)
15-19 years	2345953.21 (1418740.67, 3429036.15)	451.65 (273.14, 660.16)	2668590.42 (1610051.24, 3906594.54)	427.67 (258.03, 626.08)	-0.23 (-0.26, -0.2)
20-24 years	2811758.89 (1717189.24, 4278070.94)	571.39 (348.96, 869.37)	3192076.46 (1943526.14, 4845421.64)	534.54 (325.46, 811.41)	-0.23 (-0.27, -0.18)
25-29 years	3171326.17 (1932993.17, 4865643.37)	716.49 (436.72, 1099.28)	3785696.64 (2324086.97, 5800595.74)	643.45 (395.02, 985.92)	-0.23 (-0.29, -0.16)
30-34 years	3485823.79 (2083957.81, 5187977.32)	904.42 (540.69, 1346.05)	4662316.63 (2793017.31, 6932083.99)	771.29 (462.05, 1146.78)	-0.32 (-0.42, -0.23)
35-39 years	3950186.45 (2438495.3, 5782980.94)	1121.43 (692.27, 1641.75)	5455696.85 (3370953.14, 7999316.45)	972.73 (601.03, 1426.24)	-0.35 (-0.43, -0.26)
40-44 years	3835722.57 (2382455.47, 5651541.42)	1338.91 (831.63, 1972.74)	5880449.65 (3658350.29, 8639810.7)	1175.5 (731.3, 1727.1)	-0.37 (-0.43, -0.31)
45-49 years	3527882.34 (2237831.79, 5388179.37)	1519.36 (963.77, 2320.54)	6364181.05 (4010153.41, 9688964.7)	1344.06 (846.91, 2046.22)	-0.36 (-0.39, -0.33)
50-54 years	3726949.7 (2320970.08, 5512378.1)	1753.27 (1091.85, 2593.19)	6816592.28 (4239946.43, 10077365.68)	1532.08 (952.96, 2264.97)	-0.36 (-0.39, -0.32)
55-59 years	3543075.74 (2211274.71, 5187127.79)	1913.11 (1193.99, 2800.83)	6673088.05 (4158521.59, 9810460.62)	1686.28 (1050.85, 2479.09)	-0.31 (-0.35, -0.28)
60-64 years	3375353.81 (2108626.69, 5024034.28)	2101.59 (1312.89, 3128.11)	6011464.79 (3784031.89, 8889762.34)	1878.3 (1182.33, 2777.64)	-0.31 (-0.34, -0.27)
65-69 years	2829635.44 (1744611.2, 4174415.09)	2289.17 (1411.39, 3377.1)	5577566.03 (3462952.02, 8198945.81)	2022.01 (1255.41, 2972.33)	-0.34 (-0.37, -0.31)
70-74 years	2092094.55 (1336220.2, 3017158.63)	2471.14 (1578.32, 3563.81)	4538764.28 (2930586.33, 6495869.04)	2205 (1423.73, 3155.8)	-0.35 (-0.37, -0.33)
75-79 years	1683248.32 (1066170.92, 2434743.49)	2734.52 (1732.05, 3955.36)	3080130.71 (1958824.59, 4441638.72)	2335.48 (1485.26, 3367.83)	-0.36 (-0.4, -0.31)
80-84 years	946714.06 (627119.23, 1359475.3)	2676.15 (1772.73, 3842.94)	2090831.71 (1377472.03, 2998239.25)	2387.26 (1572.76, 3423.31)	-0.31 (-0.35, -0.27)
85-89 years	378421.16 (248735.75, 566697.26)	2504.26 (1646.05, 3750.21)	1044191.73 (691417.45, 1563592.44)	2283.79 (1512.23, 3419.8)	-0.24 (-0.27, -0.21)
90-94 years	90365.13 (59470.67, 125999.05)	2108.78 (1387.82, 2940.34)	360623.75 (240425.53, 502026.68)	2015.85 (1343.96, 2806.28)	-0.14 (-0.16, -0.12)
95 plus years	17783.16 (11214.26, 25827.7)	1746.72 (1101.5, 2536.89)	94297.8 (60415.21, 136198.77)	1730.14 (1108.47, 2498.92)	-0.05 (-0.08, -0.02)

Notes: ASR: age-standardized rates; YLDs: years lived with disability; EAPCs: estimated annual percentage changes.

Table S14. Prevalence and YLDs of hip osteoarthritis and its temporal trends from 1990, 2021 by gender, and age in the worldwide.

	1990		2021		EAPC 95%CI
	Number	ASR, per 100,000 persons	Number	ASR, per 100,000 persons	
**Prevalence**					
**Gender**					
Female	8309468.97 (6423683.4, 10552218.64)	393.28 (304.32, 499.27)	18907637.35 (14587639.8, 24103796.67)	412.44 (318.18, 523.89)	0.25 (0.22, 0.28)
Male	7087540.48 (5405198.18, 9058811.77)	387.08 (297.61, 495.26)	16978641.34 (12979722.49, 21896693.26)	419.51 (322.25, 538.04)	0.3 (0.27, 0.32)
**Age**					
30-34 years	334315.65 (201774.4, 515799.89)	86.74 (52.35, 133.83)	585793.31 (354836.1, 891189.13)	96.91 (58.7, 147.43)	0.43 (0.39, 0.47)
35-39 years	526008.97 (334387.53, 762666.25)	149.33 (94.93, 216.52)	950427.9 (608637.65, 1362081.97)	169.46 (108.52, 242.85)	0.46 (0.41, 0.51)
40-44 years	738932.41 (470980.62, 1106035.26)	257.93 (164.4, 386.08)	1442559.72 (922227.04, 2150926.27)	288.37 (184.35, 429.97)	0.41 (0.36, 0.46)
45-49 years	989986.54 (655239.22, 1376660.77)	426.36 (282.19, 592.89)	2212761.95 (1453184.63, 3071304.22)	467.32 (306.9, 648.63)	0.35 (0.31, 0.39)
50-54 years	1415347.57 (924916.58, 1987368.44)	665.82 (435.11, 934.92)	3200456.67 (2091571.24, 4475969.85)	719.33 (470.1, 1006.01)	0.35 (0.3, 0.4)
55-59 years	1772713.1 (1238500.16, 2410596.14)	957.19 (668.74, 1301.62)	4125599.74 (2871401.04, 5623050.56)	1042.53 (725.6, 1420.94)	0.44 (0.4, 0.48)
60-64 years	2148828.51 (1586065.2, 2824843.6)	1337.92 (987.53, 1758.83)	4673772.36 (3419673.09, 6177851.17)	1460.34 (1068.49, 1930.29)	0.4 (0.35, 0.46)
65-69 years	2184510.6 (1621479.67, 2836263.32)	1767.27 (1311.78, 2294.54)	5042734.35 (3718323.71, 6590237.51)	1828.12 (1347.99, 2389.13)	0.27 (0.2, 0.33)
70-74 years	1824078.47 (1387451.82, 2369934.77)	2154.56 (1638.83, 2799.32)	4760088.07 (3602152.48, 6222547.48)	2312.53 (1749.98, 3023.01)	0.19 (0.13, 0.24)
75-79 years	1634075.44 (1238278.12, 2124220.54)	2654.64 (2011.64, 3450.9)	3540381.88 (2698456.35, 4608268.26)	2684.46 (2046.08, 3494.17)	0.06 (0.02, 0.11)
80-84 years	1079457.55 (817147.74, 1383457.19)	3051.39 (2309.9, 3910.73)	2720579.92 (2053830.3, 3511147.31)	3106.29 (2345.01, 4008.93)	0.03 (-0.01, 0.06)
85-89 years	529103.73 (407800.67, 673798.21)	3501.43 (2698.69, 4458.97)	1628208.59 (1242325.1, 2082380.83)	3561.12 (2717.14, 4554.46)	0.08 (0.03, 0.12)
90-94 years	172418.76 (135360.73, 219470.52)	4023.6 (3158.81, 5121.61)	745891.19 (582249.49, 950382.74)	4169.47 (3254.72, 5312.55)	0.1 (0.05, 0.14)
95 plus years	47232.13 (37349.3, 59685.54)	4639.3 (3668.58, 5862.52)	257023.05 (203223.06, 326550.45)	4715.75 (3728.65, 5991.41)	-0.01 (-0.06, 0.04)
**YLDs**					
**Gender**					
Female	263454.1 (123284.82, 532648.3)	12.43 (5.81, 25.12)	595549.43 (280680.81, 1203819.45)	13.01 (6.12, 26.28)	0.25 (0.22, 0.28)
Male	228389.43 (107391.92, 464405.31)	12.35 (5.84, 25.08)	544117.49 (254794.12, 1104113.12)	13.39 (6.27, 27.11)	0.3 (0.27, 0.33)

**Age**					
30-34 years	11467.15 (4592.58, 23705.86)	2.98 (1.19, 6.15)	20044.73 (8215.23, 41953.42)	3.32 (1.36, 6.94)	0.42 (0.39, 0.46)
35-39 years	17668.48 (7398.88, 37719.56)	5.02 (2.1, 10.71)	31884.01 (13599.56, 67958.86)	5.68 (2.42, 12.12)	0.45 (0.41, 0.5)
40-44 years	24617.3 (10445.81, 52781.42)	8.59 (3.65, 18.42)	48048.03 (20441.42, 103819.57)	9.6 (4.09, 20.75)	0.41 (0.36, 0.47)
45-49 years	32773.28 (14467.79, 69351.4)	14.11 (6.23, 29.87)	73285.13 (32408.81, 157127.94)	15.48 (6.84, 33.18)	0.36 (0.32, 0.39)
50-54 years	46563.72 (19675.89, 96304.46)	21.9 (9.26, 45.3)	105188.83 (44690.43, 218456.16)	23.64 (10.04, 49.1)	0.36 (0.31, 0.4)
55-59 years	57796.43 (25626.2, 114328.65)	31.21 (13.84, 61.73)	134304.3 (60394.76, 266178.17)	33.94 (15.26, 67.26)	0.44 (0.39, 0.48)
60-64 years	69247.87 (32527.71, 141394.48)	43.12 (20.25, 88.04)	150354.14 (70766.94, 307353.38)	46.98 (22.11, 96.03)	0.4 (0.35, 0.46)
65-69 years	69530.65 (33150.27, 143203.67)	56.25 (26.82, 115.85)	160083.71 (75424.5, 330160.24)	58.03 (27.34, 119.69)	0.26 (0.2, 0.33)
70-74 years	57166.92 (26785.06, 116858.79)	67.52 (31.64, 138.03)	148764.58 (69877.61, 304088.07)	72.27 (33.95, 147.73)	0.18 (0.13, 0.23)
75-79 years	50349.46 (23905.97, 101139.59)	81.8 (38.84, 164.31)	108716.58 (51163.24, 219215.28)	82.43 (38.79, 166.22)	0.06 (0.01, 0.1)
80-84 years	32645.87 (15634.22, 64178.76)	92.28 (44.19, 181.42)	82032.52 (39178.19, 161295.91)	93.66 (44.73, 184.16)	0.02 (-0.02, 0.05)
85-89 years	15675.54 (7552.92, 30451.13)	103.74 (49.98, 201.52)	48100.04 (23136.88, 93900.38)	105.2 (50.6, 205.37)	0.07 (0.02, 0.12)
90-94 years	5000.56 (2461.21, 9810.39)	116.69 (57.44, 228.94)	21588.52 (10594.43, 42496.6)	120.68 (59.22, 237.55)	0.09 (0.04, 0.13)
95 plus years	1340.29 (658.35, 2636.79)	131.65 (64.67, 258.99)	7271.81 (3603.55, 14304.26)	133.42 (66.12, 262.45)	-0.02 (-0.07, 0.03)

Notes: ASR: age-standardized rates; YLDs: years lived with disability; EAPCs: estimated annual percentage changes.

Table S15. Prevalence and YLDs of knee osteoarthritis and its temporal trends from 1990, 2021 by gender, and age in the worldwide.

	1990		2021		EAPC 95%CI
	Number	ASR, per 100,000 persons	Number	ASR, per 100,000 persons	
**Prevalence**					
**Gender**					
Female	98498537.54 (84692265.3, 112495598.25)	4613.23 (3972.13, 5267.02)	230189285.45 (198051497.86, 262858482.11)	5029.51 (4331.89, 5738.43)	0.36 (0.33, 0.4)
Male	61300371.91 (52267190.68, 70269784.32)	3224.33 (2771.36, 3685.31)	144549458.67 (123542115.6, 165818687.2)	3483.37 (2991.52, 3989.67)	0.3 (0.27, 0.32)
**Age**					
30-34 years	391331.22 (285245.54, 526392.91)	101.53 (74.01, 136.58)	664479.13 (483746.02, 890861.5)	109.93 (80.03, 147.38)	0.4 (0.29, 0.5)
35-39 years	3437596.98 (2483463.11, 4608306.7)	975.91 (705.04, 1308.27)	5841634.15 (4231517.39, 7801647.33)	1041.54 (754.46, 1391)	0.42 (0.28, 0.57)
40-44 years	8158515.43 (6352088.38, 10457870.05)	2847.83 (2217.28, 3650.45)	15196204.79 (11810709.19, 19415585.07)	3037.72 (2360.96, 3881.17)	0.46 (0.31, 0.61)
45-49 years	13525080.74 (10953998.24, 16262928.41)	5824.87 (4717.58, 7003.98)	30059813.31 (24323835.67, 36045160.5)	6348.37 (5136.98, 7612.42)	0.49 (0.39, 0.58)
50-54 years	19917033.64 (15455198.19, 24946924.13)	9369.57 (7270.59, 11735.78)	46619708.01 (36141663.6, 58291310.17)	10478.15 (8123.13, 13101.44)	0.47 (0.42, 0.51)
55-59 years	23544189.61 (18973462.29, 28153011.44)	12712.85 (10244.85, 15201.41)	55621238.86 (45079915.97, 66454321.54)	14055.42 (11391.64, 16792.93)	0.38 (0.34, 0.43)
60-64 years	24885704.66 (20354931.97, 29857001.58)	15494.57 (12673.57, 18589.84)	53626680.5 (43844244.89, 64102640.33)	16755.83 (13699.28, 20029.08)	0.35 (0.32, 0.39)
65-69 years	22480857.87 (18685104.09, 27196811.89)	18187 (15116.24, 22002.21)	53790238.72 (44848663.21, 64334841.65)	19500.37 (16258.81, 23323.06)	0.28 (0.26, 0.31)
70-74 years	17325613.34 (14578086.3, 20641994.25)	20464.66 (17219.33, 24381.9)	44819021.51 (37668997.74, 53407317.19)	21773.8 (18300.2, 25946.13)	0.2 (0.17, 0.22)
75-79 years	13598101.29 (11524297.9, 16146012.03)	22090.79 (18721.8, 26230)	30826348.54 (26164394.26, 36565376.78)	23373.76 (19838.88, 27725.32)	0.14 (0.11, 0.17)
80-84 years	8071702.97 (6853440.53, 9564993.98)	22816.94 (19373.18, 27038.14)	21264233.01 (18065993.86, 25136546.7)	24278.93 (20627.27, 28700.24)	0.16 (0.14, 0.18)
85-89 years	3374042.78 (2857030.73, 3970582.92)	22328.28 (18906.87, 26275.98)	11092546.2 (9414233.54, 13026388.07)	24260.96 (20590.26, 28490.55)	0.24 (0.22, 0.26)
90-94 years	895554.34 (765761.3, 1051790.76)	20898.84 (17869.96, 24544.8)	4130842.24 (3529798.59, 4858914.37)	23091.04 (19731.26, 27160.9)	0.34 (0.32, 0.36)
95 plus years	193584.58 (165116.83, 228448.86)	19014.55 (16218.35, 22439.04)	1185755.15 (1012252.46, 1397534.03)	21755.74 (18572.39, 25641.37)	0.45 (0.44, 0.47)
**YLDs**					
**Gender**					
Female	3155785.82 (1543267.63, 6114836.17)	147.59 (72.23, 286.21)	7344350.74 (3585648.27, 14249003.54)	160.61 (78.3, 311.53)	0.36 (0.33, 0.4)
Male	1989552.88 (966778.16, 3838422.31)	103.89 (50.66, 202.11)	4674719.16 (2271425.61, 9018854.81)	112.3 (54.71, 217.62)	0.31 (0.28, 0.33)

**Age**					
30-34 years	13417.81 (5981.24, 28967.36)	3.48 (1.55, 7.52)	22761.59 (10284.59, 48340.99)	3.77 (1.7, 8)	0.39 (0.29, 0.5)
35-39 years	115706.93 (53472.73, 244353.69)	32.85 (15.18, 69.37)	196770.27 (92136.96, 414117.69)	35.08 (16.43, 73.84)	0.43 (0.29, 0.57)
40-44 years	273109.54 (130078.82, 572482.65)	95.33 (45.41, 199.83)	508637.78 (242653.66, 1060241.96)	101.68 (48.51, 211.94)	0.47 (0.32, 0.62)
45-49 years	449552.5 (213071.28, 879903.44)	193.61 (91.76, 378.95)	999524.97 (471177.03, 1959034)	211.09 (99.51, 413.73)	0.49 (0.39, 0.59)
50-54 years	657495.43 (304248.41, 1323760.98)	309.31 (143.13, 622.74)	1539134.4 (709506.02, 3079501.24)	345.93 (159.47, 692.14)	0.47 (0.43, 0.51)
55-59 years	770406.73 (361595.35, 1477015.76)	415.99 (195.25, 797.52)	1818905.72 (854416.1, 3485633.57)	459.64 (215.91, 880.82)	0.38 (0.34, 0.43)
60-64 years	805153.62 (383779.97, 1551197.38)	501.31 (238.95, 965.82)	1732661.4 (825738.51, 3327841.56)	541.38 (258, 1039.79)	0.35 (0.31, 0.39)
65-69 years	717874.38 (343737.2, 1407969.89)	580.76 (278.08, 1139.05)	1714937.34 (823039.02, 3354470.79)	621.71 (298.37, 1216.08)	0.28 (0.26, 0.31)
70-74 years	544834.69 (263995.76, 1083001.15)	643.55 (311.83, 1279.22)	1407034.24 (682030.14, 2808604.52)	683.56 (331.34, 1364.47)	0.19 (0.17, 0.22)
75-79 years	420764.93 (203794.88, 867849.72)	683.55 (331.07, 1409.87)	951389.69 (459300.27, 1961105.67)	721.38 (348.26, 1486.99)	0.14 (0.11, 0.17)
80-84 years	245168.01 (119283.76, 498035.74)	693.04 (337.19, 1407.84)	644624.89 (312180.22, 1305882.39)	736.02 (356.44, 1491.02)	0.16 (0.14, 0.18)
85-89 years	100319.57 (49270.67, 201461.76)	663.88 (326.06, 1333.21)	329198.99 (162446.23, 654621.6)	720 (355.29, 1431.75)	0.24 (0.22, 0.26)
90-94 years	26038.45 (12906.71, 52529.64)	607.64 (301.19, 1225.84)	119876.46 (60009.53, 242154.11)	670.1 (335.45, 1353.62)	0.34 (0.32, 0.35)
95 plus years	5496.1 (2740.3, 11035.82)	539.85 (269.16, 1083.98)	33612.16 (16822.11, 67894.3)	616.7 (308.65, 1245.7)	0.45 (0.43, 0.46)

Notes: ASR: age-standardized rates; YLDs: years lived with disability; EAPCs: estimated annual percentage changes.

Table S16. Prevalence and YLDs of rheumatoid arthritis and its temporal trends from 1990, 2021 by gender, and age in the worldwide.

	1990		2021		
	Number	ASR, per 100,000 persons	Number	ASR, per 100,000 persons	EAPC 95%CI
**Prevalence**					
**Gender**					
Female	5831179.02 (5172110.6, 6619854.86)	257.98 (229.32, 292.43)	12975146.35 (11647165.24, 14611570.8)	293.66 (263.52, 331.33)	0.52 (0.48, 0.55)
Male	2127875.72 (1840228.54, 2485571.05)	101.91 (89.65, 117.83)	4949520.98 (4369798.94, 5705323.46)	119.68 (105.95, 137.45)	0.61 (0.58, 0.64)
**Age**					
5-9 years	7125.27 (3408.3, 12256.77)	1.22 (0.58, 2.1)	9149.57 (4289.4, 15647.72)	1.33 (0.62, 2.28)	0.4 (0.36, 0.44)
10-14 years	47416.47 (29452.47, 72969.81)	8.85 (5.5, 13.62)	65643.21 (41441.99, 99406.35)	9.85 (6.22, 14.91)	0.46 (0.42, 0.5)
15-19 years	134681.31 (88770.05, 185164.42)	25.93 (17.09, 35.65)	182782.07 (122075.42, 252470.18)	29.29 (19.56, 40.46)	0.53 (0.49, 0.58)
20-24 years	257926.23 (174609.22, 361662.34)	52.41 (35.48, 73.5)	359830.27 (248461.24, 500289.52)	60.26 (41.61, 83.78)	0.61 (0.56, 0.65)
25-29 years	381229.44 (273409.72, 506744.63)	86.13 (61.77, 114.49)	589490.87 (427870.27, 776598.15)	100.2 (72.72, 132)	0.61 (0.57, 0.65)
30-34 years	485268.5 (369628.82, 651219.34)	125.91 (95.9, 168.96)	901929.43 (683037.81, 1196066.94)	149.21 (113, 197.87)	0.59 (0.57, 0.62)
35-39 years	621930.81 (470861.66, 804707.89)	176.56 (133.67, 228.45)	1134994.62 (873492.65, 1457969.97)	202.36 (155.74, 259.95)	0.52 (0.48, 0.56)
40-44 years	669390.56 (514000.87, 835496.01)	233.66 (179.42, 291.64)	1313109.11 (1024530.72, 1620788.5)	262.49 (204.8, 324)	0.5 (0.45, 0.55)
45-49 years	680330.36 (532138.08, 847526.77)	293 (229.18, 365.01)	1587897.62 (1259938.67, 1948963.18)	335.35 (266.09, 411.6)	0.52 (0.48, 0.55)
50-54 years	771771.56 (625356.5, 933805.66)	363.06 (294.19, 439.29)	1849034.21 (1502673.97, 2227738.21)	415.59 (337.74, 500.7)	0.51 (0.49, 0.53)
55-59 years	827686.45 (696406.54, 984390.34)	446.92 (376.03, 531.53)	2011670.8 (1700393.29, 2377347.84)	508.35 (429.69, 600.75)	0.5 (0.45, 0.56)
60-64 years	870117.66 (745799.89, 1024431.77)	541.76 (464.36, 637.84)	1948104.81 (1678737.37, 2265242.8)	608.69 (524.53, 707.78)	0.53 (0.46, 0.6)
65-69 years	802511.79 (687002.46, 942253.48)	649.23 (555.78, 762.28)	1997898.73 (1722725.34, 2323991.47)	724.29 (624.53, 842.51)	0.56 (0.49, 0.64)
70-74 years	591207.64 (512997.99, 684295.7)	698.32 (605.94, 808.28)	1659305.98 (1447190.41, 1907390.98)	806.12 (703.07, 926.64)	0.57 (0.5, 0.63)
75-79 years	437816.84 (385534.97, 498469.15)	711.26 (626.32, 809.79)	1102839.68 (971034.73, 1250100.17)	836.22 (736.28, 947.88)	0.5 (0.47, 0.53)
80-84 years	246421.07 (218806.42, 275805.62)	696.58 (618.52, 779.64)	708713.87 (632280.97, 793786.49)	809.19 (721.92, 906.32)	0.48 (0.44, 0.52)
85-89 years	97273.58 (87065.83, 108402.08)	643.72 (576.17, 717.37)	350080.91 (314329.96, 390470.94)	765.68 (687.48, 854.01)	0.56 (0.48, 0.65)
90-94 years	24360.05 (21787.88, 27305.63)	568.47 (508.45, 637.21)	121383.93 (108995.42, 135527.99)	678.53 (609.27, 757.59)	0.68 (0.56, 0.79)

95 plus years	4589.13 (4038.45, 5221.32)	450.76 (396.67, 512.86)	30807.64 (27244.72, 35051.68)	565.25 (499.87, 643.11)	0.79 (0.69, 0.89)
**YLDs**					
**Gender**					
Female	769695.27 (525223.57, 1079501.39)	33.91 (23.22, 47.25)	1694661.63 (1162543.35, 2332245.33)	38.46 (26.32, 53.19)	0.51 (0.47, 0.55)
Male	287498.16 (193268.51, 406951.71)	13.6 (9.24, 19.13)	659807.43 (447620.76, 921013.7)	15.92 (10.76, 22.15)	0.6 (0.57, 0.63)
**Age**					
5-9 years	1046.09 (447.58, 2088.33)	0.18 (0.08, 0.36)	1343.28 (582.03, 2712.17)	0.2 (0.08, 0.39)	0.4 (0.36, 0.44)
10-14 years	6947.79 (3655.24, 12291.73)	1.3 (0.68, 2.29)	9617.95 (5158.76, 16941.85)	1.44 (0.77, 2.54)	0.46 (0.42, 0.5)
15-19 years	19548.55 (11200.82, 31523.66)	3.76 (2.16, 6.07)	26480.17 (15229.89, 42954.67)	4.24 (2.44, 6.88)	0.53 (0.48, 0.57)
20-24 years	36987.05 (20672.41, 59556.21)	7.52 (4.2, 12.1)	51333.75 (29408.99, 80994.96)	8.6 (4.92, 13.56)	0.59 (0.54, 0.64)
25-29 years	53666.09 (32752.77, 83279.87)	12.12 (7.4, 18.82)	82679.27 (50254.11, 128484.67)	14.05 (8.54, 21.84)	0.6 (0.56, 0.64)
30-34 years	67481.83 (43094.12, 104624.83)	17.51 (11.18, 27.15)	125217.47 (81098.89, 193563.52)	20.71 (13.42, 32.02)	0.59 (0.57, 0.61)
35-39 years	85789.8 (53683.72, 129460.89)	24.36 (15.24, 36.75)	156037.66 (99961.02, 235676.87)	27.82 (17.82, 42.02)	0.51 (0.47, 0.55)
40-44 years	91287.06 (56553.19, 136585.91)	31.86 (19.74, 47.68)	178782.74 (111223.69, 266566.45)	35.74 (22.23, 53.29)	0.5 (0.45, 0.56)
45-49 years	91917.72 (59265.63, 134319.38)	39.59 (25.52, 57.85)	214213.78 (137479.63, 312985.28)	45.24 (29.03, 66.1)	0.52 (0.48, 0.55)
50-54 years	103393.49 (67567.17, 148999.17)	48.64 (31.79, 70.09)	247434.91 (160245.25, 359076.45)	55.61 (36.02, 80.71)	0.51 (0.49, 0.54)
55-59 years	109547.24 (75380.9, 154376.29)	59.15 (40.7, 83.36)	265818 (182296.86, 373320.82)	67.17 (46.07, 94.34)	0.5 (0.45, 0.56)
60-64 years	113668.43 (78154.14, 159478.55)	70.77 (48.66, 99.3)	253580.83 (173847.01, 354091.25)	79.23 (54.32, 110.64)	0.52 (0.45, 0.6)
65-69 years	102975.4 (71016.22, 143198.08)	83.31 (57.45, 115.85)	255386.68 (177737.97, 356241.89)	92.58 (64.43, 129.15)	0.56 (0.48, 0.63)
70-74 years	74443.37 (50568.27, 102808.17)	87.93 (59.73, 121.43)	207929.33 (140791.46, 283135.96)	101.02 (68.4, 137.55)	0.55 (0.49, 0.62)
75-79 years	54046.03 (37252.22, 73170.32)	87.8 (60.52, 118.87)	135320.54 (94739.26, 183114.58)	102.61 (71.84, 138.84)	0.48 (0.45, 0.51)
80-84 years	29689.08 (20479.08, 39492.98)	83.92 (57.89, 111.64)	85052.6 (58632.67, 112333.49)	97.11 (66.95, 128.26)	0.47 (0.43, 0.51)
85-89 years	11444.17 (7914.41, 15083.52)	75.73 (52.37, 99.82)	40955.52 (28534.57, 54020.04)	89.58 (62.41, 118.15)	0.54 (0.46, 0.63)
90-94 years	2798.98 (1922.77, 3697.69)	65.32 (44.87, 86.29)	13852.8 (9613.62, 18452.1)	77.44 (53.74, 103.15)	0.66 (0.54, 0.77)
95 plus years	515.25 (354.46, 692.46)	50.61 (34.82, 68.02)	3431.78 (2360.75, 4603.75)	62.96 (43.31, 84.47)	0.77 (0.67, 0.87)

Notes: ASR: age-standardized rates; YLDs: years lived with disability; EAPCs: estimated annual percentage changes.

Table S17. Prevalence and YLDs of tension-type headache and its temporal trends from 1990, 2021 by gender, and age in the worldwide.

	1990		2021		EAPC 95%CI
	Number	ASR, per 100,000 persons	Number	ASR, per 100,000 persons	
**Prevalence**					
**Gender**					
Female	669543694.94 (586174254.21, 763312519.53)	25998.32 (22971.7, 29282.6)	1042235881.11 (923030957.86, 1176685749.4)	25634.41 (22631.48, 28974.28)	-0.05 (-0.06, -0.04)
Male	616822976.81 (537230447.65, 705261999.9)	23782.48 (20933.54, 26745.2)	969376996.38 (851723604.5, 1095945313.3)	23880.79 (21046.24, 26935.06)	0.02 (0, 0.03)
**Age**					
5-9 years	41828970.78 (25734431.15, 61152461.94)	7168.26 (4410.12, 10479.73)	48516984.84 (29768981.58, 71212810.64)	7061.6 (4332.85, 10364.96)	-0.01 (-0.04, 0.01)
10-14 years	131962838.36 (95959607.64, 172587248.71)	24634.52 (17913.52, 32218.19)	163395842.88 (118540839.92, 215698218.13)	24510.51 (17781.95, 32356.22)	0 (-0.02, 0.02)
15-19 years	148724492.13 (96025700.64, 201941439.74)	28632.61 (18486.98, 38878)	182630211.04 (118262019.38, 248862752.67)	29268.61 (18952.86, 39883.14)	0.04 (0.02, 0.06)
20-24 years	148380198.18 (107250704.71, 203469692.37)	30153.19 (21795.03, 41348.24)	187767477.93 (135943894.77, 260071630.85)	31443.51 (22765.14, 43551.55)	0.1 (0.08, 0.13)
25-29 years	143667559.06 (102344064.73, 193891416.14)	32458.47 (23122.35, 43805.42)	194233133.64 (136635928.53, 262219572.44)	33013.58 (23223.85, 44569.15)	0.09 (0.05, 0.12)
30-34 years	134993641.23 (94490122.83, 179263004.1)	35024.85 (24515.99, 46510.78)	208549144.19 (145971723.06, 277947832.05)	34500.58 (24148.31, 45981.3)	-0.01 (-0.06, 0.04)
35-39 years	118490551.24 (78395239.37, 157562574.78)	33638.73 (22255.92, 44731.03)	188272545.67 (123875465.11, 252958374.58)	33568.18 (22086.46, 45101.38)	-0.02 (-0.07, 0.02)
40-44 years	95600212.16 (68628290.08, 131376728.59)	33370.45 (23955.56, 45858.69)	165571577.48 (117905770.29, 226683906.97)	33097.71 (23569.33, 45314.05)	-0.05 (-0.09, -0.01)
45-49 years	71778816.49 (50498255.52, 93990038.88)	30913.1 (21748.16, 40478.84)	143708543.05 (101012981.75, 189325728.49)	30349.98 (21333.05, 39983.92)	-0.08 (-0.12, -0.04)
50-54 years	61359655.58 (42239329.11, 82615515.18)	28865.42 (19870.65, 38864.82)	123568462.69 (85121544.49, 167310801.52)	27773 (19131.75, 37604.44)	-0.12 (-0.15, -0.1)
55-59 years	53165152.22 (37409662.01, 73465722.05)	28706.89 (20199.61, 39668.33)	109866566.16 (77806366.54, 152208646.46)	27763.15 (19661.58, 38462.95)	-0.08 (-0.1, -0.06)
60-64 years	42150809.53 (28759823.71, 59334516.59)	26244.32 (17906.71, 36943.4)	81601381.09 (55456884.19, 114714480.56)	25496.62 (17327.69, 35842.92)	-0.12 (-0.15, -0.09)
65-69 years	35933396.2 (25925275.04, 49466668.87)	29070.1 (20973.54, 40018.51)	76564079.52 (55848754.88, 105244212.2)	27756.48 (20246.63, 38153.78)	-0.15 (-0.17, -0.14)
70-74 years	26279568.05 (19059766.35, 36757271.28)	31040.88 (22513, 43416.93)	61780155.19 (44690070.06, 85622422.15)	30013.79 (21711.15, 41596.75)	-0.12 (-0.15, -0.1)
75-79 years	17167247.21 (10770534.63, 23684582.13)	27889.05 (17497.27, 38476.78)	35240754.03 (22285523.07, 48629105.64)	26720.94 (16897.77, 36872.52)	-0.13 (-0.15, -0.11)
80-84 years	9496369.61 (6407380.67, 13305589.69)	26844.16 (18112.26, 37611.99)	22614317.2 (15303837, 31513862.63)	25820.42 (17473.51, 35981.69)	-0.11 (-0.12, -0.1)
85-89 years	3939779.27 (2602138.36, 5594484.34)	26072.14 (17220.08, 37022.42)	11473159.77 (7594628.02, 16283598.8)	25093.42 (16610.52, 35614.53)	-0.11 (-0.12, -0.1)
90-94 years	1104209.24 (737350.77, 1551664.35)	25768.05 (17206.97, 36209.95)	4465921.44 (2984841.9, 6290048.51)	24964.1 (16685, 35160.81)	-0.09 (-0.1, -0.09)

95 plus years	343205.22 (226411.72, 477942.58)	33710.81 (22238.95, 46945.18)	1792619.68 (1181405.96, 2503917.73)	32890.24 (21675.95, 45940.84)	-0.07 (-0.08, -0.07)
**YLDs**					
**Gender**					
Female	1598917.95 (484063.04, 4995532.38)	64.13 (19.76, 193.9)	2567596.81 (788724.96, 7674299.2)	62 (18.8, 189.03)	-0.12 (-0.13, -0.11)
Male	1249769.67 (336750.01, 4481442.73)	49.75 (13.78, 171.18)	2029188.44 (558294.12, 7054295.17)	49.35 (13.44, 172.38)	-0.03 (-0.04, -0.02)
**Age**					
5-9 years	46948.99 (2314.6, 273833.56)	8.05 (0.4, 46.93)	55027.91 (2749.26, 326787.51)	8.01 (0.4, 47.56)	0.02 (0.01, 0.04)
10-14 years	167723.65 (20231.68, 1020319.66)	31.31 (3.78, 190.47)	209536.64 (24441.62, 1236171.66)	31.43 (3.67, 185.43)	0.03 (0.01, 0.05)
15-19 years	262358.72 (52288.29, 1120501.75)	50.51 (10.07, 215.72)	322125.08 (62289.71, 1385527.03)	51.62 (9.98, 222.05)	0.04 (0.02, 0.05)
20-24 years	297280.11 (69419.32, 1126243.91)	60.41 (14.11, 228.87)	371434.8 (85578.16, 1450304.05)	62.2 (14.33, 242.87)	0.06 (0.04, 0.08)
25-29 years	285820.24 (66486.26, 983382.33)	64.57 (15.02, 222.17)	382517.01 (88173.54, 1340266.8)	65.02 (14.99, 227.8)	0.06 (0.04, 0.09)
30-34 years	288737.97 (69772.43, 1058354.74)	74.91 (18.1, 274.6)	444057.69 (108955.3, 1641260.52)	73.46 (18.02, 271.52)	-0.01 (-0.06, 0.03)
35-39 years	294304.59 (77259.3, 944509.91)	83.55 (21.93, 268.14)	461944.66 (118383.99, 1494804.07)	82.36 (21.11, 266.52)	-0.07 (-0.11, -0.02)
40-44 years	258989.36 (82134.15, 777148.63)	90.4 (28.67, 271.27)	440412.61 (137621.56, 1314748.38)	88.04 (27.51, 262.82)	-0.14 (-0.18, -0.09)
45-49 years	211963.18 (63535.94, 585385.17)	91.29 (27.36, 252.11)	417972.98 (122421.65, 1184650)	88.27 (25.85, 250.19)	-0.16 (-0.19, -0.12)
50-54 years	195309.79 (58705.28, 500294.71)	91.88 (27.62, 235.35)	380548.26 (111365.82, 998579.85)	85.53 (25.03, 224.44)	-0.2 (-0.24, -0.17)
55-59 years	164302.02 (48559.9, 439620.56)	88.72 (26.22, 237.38)	330292.51 (95262.39, 895197.33)	83.46 (24.07, 226.22)	-0.16 (-0.2, -0.13)
60-64 years	142967.18 (44796.92, 372839.34)	89.02 (27.89, 232.14)	267566.76 (82482.93, 727207.47)	83.6 (25.77, 227.22)	-0.21 (-0.25, -0.17)
65-69 years	104109.03 (31499.62, 301589.25)	84.22 (25.48, 243.99)	219061.18 (65165.46, 652695.6)	79.42 (23.62, 236.62)	-0.23 (-0.26, -0.21)
70-74 years	60584.26 (17055.3, 202515.02)	71.56 (20.15, 239.21)	141686.3 (39578.18, 478419.12)	68.83 (19.23, 232.42)	-0.17 (-0.2, -0.15)
75-79 years	37648.37 (10277.54, 128165.73)	61.16 (16.7, 208.21)	75230.02 (20463.02, 262272.26)	57.04 (15.52, 198.87)	-0.16 (-0.19, -0.14)
80-84 years	19589.84 (5129.95, 66142.12)	55.38 (14.5, 186.97)	45936.66 (11750.9, 157482.57)	52.45 (13.42, 179.81)	-0.16 (-0.18, -0.14)
85-89 years	7850.83 (2011.5, 27737.64)	51.95 (13.31, 183.56)	22255.58 (5452.9, 79686.11)	48.68 (11.93, 174.28)	-0.18 (-0.2, -0.16)
90-94 years	1817.15 (388.16, 6502.97)	42.41 (9.06, 151.75)	7205.39 (1530.74, 26044.77)	40.28 (8.56, 145.59)	-0.17 (-0.18, -0.15)
95 plus years	382.33 (34.81, 1860.57)	37.55 (3.42, 182.75)	1973.21 (173.14, 9601.47)	36.2 (3.18, 176.16)	-0.12 (-0.13, -0.12)

Notes: ASR: age-standardized rates; YLDs: years lived with disability; EAPCs: estimated annual percentage changes.

Table S18. Prevalence and YLDs of migraine and its temporal trends from 1990, 2021 by gender, and age in the worldwide.

	1990		2021		EAPC 95%CI
	Number	ASR, per 100,000 persons	Number	ASR, per 100,000 persons	
**Prevalence**					
**Gender**					
Female	462896609.35 (396950116.14, 532406329.56)	17864.62 (15368.19, 20418.65)	725242191.97 (627617457.67, 830850946.92)	17902.6 (15445.99, 20487.01)	0.02 (0.01, 0.03)
Male	269667853.33 (227787523.42, 312882000.72)	10229.25 (8743.81, 11822.93)	433190631.84 (368788829.32, 501602102.35)	10624.2 (9039.46, 12297.27)	0.13 (0.11, 0.15)
**Age**					
5-9 years	14767041.23 (9823783.66, 20585697.12)	2530.64 (1683.51, 3527.78)	17455195.54 (11640912.01, 24478806.8)	2540.59 (1694.32, 3562.87)	0.08 (0.03, 0.14)
10-14 years	63492597.52 (47413936.62, 84121631.43)	11852.65 (8851.12, 15703.63)	79974614.55 (59777743.44, 106050718.12)	11996.74 (8967.07, 15908.34)	0.1 (0.06, 0.14)
15-19 years	87427388.92 (65664063.48, 111053195.43)	16831.62 (12641.72, 21380.09)	108299424.96 (81527056.48, 138151358.33)	17356.24 (13065.65, 22140.35)	0.1 (0.07, 0.12)
20-24 years	92702395.12 (71784722.88, 116204849.11)	18838.58 (14587.78, 23614.65)	115809740.29 (89103284.99, 145179519.69)	19393.48 (14921.22, 24311.74)	0.08 (0.06, 0.1)
25-29 years	89517694.59 (71159768.4, 111182825.69)	20224.52 (16076.96, 25119.27)	120861267.01 (95650514.64, 150629444.65)	20542.65 (16257.6, 25602.31)	0.07 (0.04, 0.09)
30-34 years	81321481.97 (64338433.26, 102185149.3)	21099.31 (16692.96, 26512.5)	128086793.79 (101265584.88, 161691639.5)	21189.58 (16752.51, 26748.87)	0.04 (0.01, 0.07)
35-39 years	74634932.49 (60625524.43, 92096760.52)	21188.39 (17211.21, 26145.69)	120786757.33 (97701173.3, 149277443.47)	21535.76 (17419.7, 26615.52)	0.04 (0.02, 0.07)
40-44 years	60993744.09 (49415166.89, 76120238.88)	21290.63 (17248.98, 26570.72)	107953780.6 (87558299.51, 135549148.06)	21579.93 (17502.88, 27096.24)	0.03 (0.01, 0.05)
45-49 years	45640333.02 (36872623.15, 56966354.78)	19655.99 (15879.99, 24533.79)	93847929.9 (74995723.77, 117496654.94)	19819.85 (15838.44, 24814.26)	0.03 (0.01, 0.05)
50-54 years	38325026.32 (31354632.88, 48015761.14)	18029.24 (14750.16, 22588.06)	80895579.36 (66115228.15, 102422980.03)	18181.93 (14859.93, 23020.38)	0.06 (0.04, 0.08)
55-59 years	29740350.13 (24132043.62, 37540787.1)	16058.51 (13030.26, 20270.41)	64703876.87 (51991279.78, 82222466.95)	16350.59 (13138.14, 20777.52)	0.08 (0.06, 0.11)
60-64 years	22546232.88 (18149407.81, 28370132.11)	14037.94 (11300.35, 17664.07)	45753173.13 (36759041.59, 57868829.22)	14295.73 (11485.48, 18081.31)	0.07 (0.06, 0.09)
65-69 years	14495292.5 (11511079.01, 18271466.06)	11726.69 (9312.46, 14781.61)	32724361.11 (25885728.08, 41521359.33)	11863.44 (9384.25, 15052.58)	0.05 (0.03, 0.08)
70-74 years	8117863.04 (6416174.79, 10433902.24)	9588.65 (7578.65, 12324.31)	19987935.98 (15713240.41, 25850252.99)	9710.46 (7633.74, 12558.47)	0.05 (0.04, 0.07)
75-79 years	5025137.1 (3953199.63, 6459583.04)	8163.59 (6422.17, 10493.91)	10794541.21 (8429389.82, 13881770.61)	8184.85 (6391.5, 10525.71)	0.06 (0.04, 0.08)
80-84 years	2569486.86 (2028308.07, 3359547.73)	7263.38 (5733.58, 9496.71)	6385715.45 (5036566.84, 8369713.4)	7291.04 (5750.62, 9556.32)	0.05 (0.03, 0.08)
85-89 years	978880.28 (760018.83, 1249751.37)	6477.9 (5029.55, 8270.43)	2954856.8 (2277253.27, 3814786.02)	6462.69 (4980.67, 8343.47)	0.03 (0, 0.05)
90-94 years	227900.51 (176615.77, 292865.46)	5318.33 (4121.54, 6834.37)	945477.02 (733659.74, 1226625.81)	5285.13 (4101.09, 6856.73)	0.01 (-0.01, 0.03)

95 plus years	40684.12 (30042.18, 55702.32)	3996.14 (2950.85, 5471.28)	211802.92 (153698.36, 292505.65)	3886.07 (2819.99, 5366.77)	-0.07 (-0.1, -0.04)
**YLDs**					
**Gender**					
Female	17179627.7 (2400079.7, 37859718.47)	664.92 (98.1, 1453.28)	26883943.36 (3930570.58, 58848028.71)	662.76 (93.66, 1450.83)	0.01 (0, 0.02)
Male	10232568.59 (1675317.81, 22688369.4)	389.98 (68.44, 844.94)	16494946.45 (2802071.63, 35551602.46)	403.88 (67.39, 872.77)	0.13 (0.11, 0.14)
**Age**					
5-9 years	492448.45 (10460.27, 1374370.9)	84.39 (1.79, 235.53)	584845.33 (13180.58, 1607282.64)	85.12 (1.92, 233.94)	0.1 (0.05, 0.16)
10-14 years	2297212.25 (100959.27, 5782523.33)	428.84 (18.85, 1079.47)	2897557.37 (125710.61, 7274035.55)	434.65 (18.86, 1091.16)	0.11 (0.07, 0.15)
15-19 years	3253115.27 (283586.66, 7894740.82)	626.29 (54.6, 1519.9)	4033372.61 (345474.04, 9888373.84)	646.39 (55.37, 1584.73)	0.1 (0.08, 0.12)
20-24 years	3433909.7 (345338.81, 8175473.81)	697.82 (70.18, 1661.38)	4289113.77 (432044.07, 10181153.16)	718.25 (72.35, 1704.93)	0.09 (0.07, 0.1)
25-29 years	3266500.95 (319415.09, 7626788.4)	737.99 (72.16, 1723.1)	4408485.01 (417385.13, 10254464.54)	749.3 (70.94, 1742.94)	0.08 (0.06, 0.1)
30-34 years	2996220.44 (359714.59, 6712563.56)	777.39 (93.33, 1741.61)	4714571.85 (567721.53, 10643665)	779.94 (93.92, 1760.8)	0.05 (0.02, 0.08)
35-39 years	2805888.34 (446636.34, 6073871.5)	796.57 (126.8, 1724.33)	4527647.18 (701860.49, 9891420.63)	807.26 (125.14, 1763.6)	0.04 (0.02, 0.07)
40-44 years	2331743.04 (417837.93, 5165838.23)	813.92 (145.85, 1803.2)	4106009.41 (702527.36, 9226433.9)	820.79 (140.44, 1844.36)	0.02 (0, 0.04)
45-49 years	1766226.27 (333934.72, 3752713.16)	760.66 (143.82, 1616.19)	3613338.21 (665253.9, 7760720.73)	763.11 (140.5, 1639)	0.02 (0, 0.04)
50-54 years	1498010.03 (318811.91, 3167560.5)	704.71 (149.98, 1490.12)	3122973.74 (614242, 6666749.71)	701.91 (138.06, 1498.41)	0.03 (0, 0.05)
55-59 years	1160738.53 (272013.82, 2475445.12)	626.75 (146.88, 1336.63)	2491133.51 (534564.66, 5415757.03)	629.51 (135.08, 1368.56)	0.05 (0.02, 0.07)
60-64 years	899525.89 (248411.75, 1863255.17)	560.07 (154.67, 1160.12)	1792017.59 (457897.01, 3719823.19)	559.92 (143.07, 1162.27)	0.02 (0, 0.04)
65-69 years	575547.83 (163584.63, 1215780.32)	465.62 (132.34, 983.57)	1278140.75 (338167.34, 2677152.52)	463.36 (122.59, 970.54)	-0.01 (-0.03, 0.01)
70-74 years	309481.24 (86906.29, 661440.8)	365.55 (102.65, 781.28)	752494.88 (202119.13, 1627115.77)	365.57 (98.19, 790.48)	0 (-0.01, 0.02)
75-79 years	187132.04 (49265.89, 395236.17)	304.01 (80.03, 642.08)	393916.33 (95782.82, 848808.48)	298.68 (72.63, 643.6)	0.01 (-0.01, 0.03)
80-84 years	93868.14 (27337.43, 200318.92)	265.34 (77.28, 566.26)	229649.1 (63418.57, 495371.46)	262.21 (72.41, 565.6)	0 (-0.02, 0.03)
85-89 years	35534.94 (11701.19, 71870.22)	235.16 (77.43, 475.61)	105139.7 (32895.4, 217228.24)	229.96 (71.95, 475.11)	-0.03 (-0.06, -0.01)
90-94 years	7844.19 (2086.53, 16252.3)	183.05 (48.69, 379.27)	32050.52 (8082.48, 67494.37)	179.16 (45.18, 377.29)	-0.05 (-0.07, -0.03)
95 plus years	1248.78 (203.69, 2858.83)	122.66 (20.01, 280.8)	6432.96 (1004.62, 14851.81)	118.03 (18.43, 272.49)	-0.1 (-0.13, -0.08)

Notes: ASR: age-standardized rates; YLDs: years lived with disability; EAPCs: estimated annual percentage changes.

Table S19. Prevalence and YLDs of stroke and its temporal trends from 1990, 2021 by gender, and age in the worldwide.

	1990		2021		EAPC 95%CI
	Number	ASR, per 100,000 persons	Number	ASR, per 100,000 persons	
**Prevalence**					
**Gender**					
Female	25668650.71 (24340826.87, 27098207.48)	1152.39 (1092.47, 1217.79)	46005877.66 (43540264.24, 48766466.68)	1027.71 (974.35, 1088.08)	-0.49 (-0.54, -0.45)
Male	24746951.45 (23390373.11, 26147609.13)	1269.25 (1197.97, 1348.48)	47810536.44 (45336514.44, 50574533.95)	1184.35 (1124.19, 1252.12)	-0.27 (-0.29, -0.25)
**Age**					
<5 years	232431.02 (210355.13, 257715.52)	37.49 (33.93, 41.57)	197464.12 (181284.28, 214517.29)	30 (27.54, 32.59)	-0.92 (-0.98, -0.85)
5-9 years	570859.06 (497250.3, 656447.18)	97.83 (85.21, 112.5)	544530.9 (480220.15, 614673.55)	79.26 (69.9, 89.47)	-0.85 (-0.9, -0.8)
10-14 years	819883.78 (712575.06, 934878.33)	153.05 (133.02, 174.52)	840803.01 (737812.77, 948675.21)	126.13 (110.68, 142.31)	-0.78 (-0.84, -0.73)
15-19 years	1125057.09 (988525.76, 1272948.45)	216.6 (190.31, 245.07)	1124698.29 (1001902.28, 1255294.94)	180.25 (160.57, 201.18)	-0.73 (-0.77, -0.68)
20-24 years	1424380.72 (1270226.63, 1598131.81)	289.46 (258.13, 324.77)	1457862.53 (1319203.23, 1614246.16)	244.13 (220.91, 270.32)	-0.66 (-0.69, -0.63)
25-29 years	1683918.13 (1515267.38, 1868791.12)	380.44 (342.34, 422.21)	1916463.26 (1738579.58, 2098086.63)	325.74 (295.5, 356.61)	-0.6 (-0.63, -0.57)
30-34 years	1932108.09 (1742581.9, 2118522.13)	501.3 (452.12, 549.66)	2629455.47 (2401494.56, 2864697.14)	434.99 (397.28, 473.91)	-0.56 (-0.59, -0.53)
35-39 years	2377258.89 (2155450.08, 2603528.06)	674.89 (611.92, 739.13)	3324240.94 (3044948.65, 3622103.51)	592.7 (542.9, 645.81)	-0.54 (-0.58, -0.51)
40-44 years	2667730.39 (2423615.84, 2916166.85)	931.2 (845.99, 1017.92)	4134056.67 (3783369.89, 4481927.83)	826.4 (756.29, 895.94)	-0.51 (-0.56, -0.47)
45-49 years	3223640.99 (2956028.83, 3512532.06)	1388.33 (1273.08, 1512.75)	5731876.18 (5287270.31, 6216817.01)	1210.52 (1116.62, 1312.94)	-0.53 (-0.57, -0.49)
50-54 years	4352823.04 (3959571.53, 4758113.1)	2047.7 (1862.7, 2238.36)	7763892.94 (7097006.26, 8461176.35)	1745 (1595.11, 1901.72)	-0.56 (-0.59, -0.54)
55-59 years	5082243.81 (4630756.35, 5497228.08)	2744.19 (2500.41, 2968.27)	9498132.29 (8682336.34, 10290040.5)	2400.17 (2194.02, 2600.28)	-0.51 (-0.53, -0.49)
60-64 years	5723110 (5175442.66, 6223833.26)	3563.38 (3222.38, 3875.14)	10169596.35 (9270509.21, 11076985.89)	3177.52 (2896.6, 3461.04)	-0.44 (-0.47, -0.4)
65-69 years	5680050.37 (5167036.19, 6228140.38)	4595.16 (4180.13, 5038.56)	11716144.86 (10631125.64, 12819736.37)	4247.41 (3854.06, 4647.49)	-0.38 (-0.42, -0.34)
70-74 years	4937777.54 (4438023.53, 5481747.94)	5832.4 (5242.1, 6474.93)	11268398.18 (10136013.81, 12501447.26)	5474.37 (4924.24, 6073.4)	-0.3 (-0.33, -0.28)
75-79 years	4291936.58 (3909615.73, 4748992.62)	6972.46 (6351.37, 7714.98)	9000947.69 (8158516.69, 9914094.88)	6824.88 (6186.11, 7517.26)	-0.12 (-0.15, -0.1)
80-84 years	2702654.03 (2444985.95, 2994913.26)	7639.81 (6911.44, 8465.96)	6833164.71 (6201635.34, 7576861.99)	7801.93 (7080.86, 8651.06)	0.02 (-0.01, 0.05)
85-89 years	1165153.58 (1035212.64, 1300881.84)	7710.59 (6850.69, 8608.8)	3699339.07 (3307961.36, 4132619.42)	8090.98 (7234.98, 9038.62)	0.08 (0.06, 0.11)

90-94 years	339168.27 (292981.58, 390581.33)	7914.9 (6837.08, 9114.68)	1481505.61 (1285032.21, 1680872.75)	8281.48 (7183.22, 9395.93)	0.13 (0.11, 0.14)
95 plus years	83416.76 (70052.5, 98711.4)	8193.48 (6880.8, 9695.78)	483841.04 (415047.85, 563175.82)	8877.31 (7615.13, 10332.92)	0.2 (0.16, 0.24)
**YLDs**					
**Gender**					
Female	4344002.86 (3122691.41, 5534877.03)	195.76 (140.92, 248.94)	7889067.55 (5689932.36, 10023828.29)	175.63 (126.73, 223.09)	-0.47 (-0.51, -0.43)
Male	3666310.83 (2648046.99, 4668981.5)	192.41 (138.68, 244.44)	7321356.93 (5299334.17, 9396089.49)	183.4 (132.67, 234.7)	-0.19 (-0.21, -0.17)
**Age**					
<5 years	37081.76 (26179.03, 48351.08)	5.98 (4.22, 7.8)	29747.04 (21127.9, 38867.91)	4.52 (3.21, 5.91)	-1.1 (-1.17, -1.02)
5-9 years	87992.65 (60966.45, 116239.26)	15.08 (10.45, 19.92)	80383.15 (56711.21, 105072.99)	11.7 (8.25, 15.29)	-1.04 (-1.11, -0.96)
10-14 years	126655.36 (87987.25, 167252.81)	23.64 (16.43, 31.22)	123887.49 (85824.39, 160791.62)	18.58 (12.87, 24.12)	-0.98 (-1.06, -0.9)
15-19 years	176481.62 (122044.12, 230126.56)	33.98 (23.5, 44.3)	165221.06 (114898.91, 216016.51)	26.48 (18.41, 34.62)	-0.93 (-0.99, -0.88)
20-24 years	224845.6 (158164.71, 296662.78)	45.69 (32.14, 60.29)	214632.25 (149203.73, 281586.92)	35.94 (24.99, 47.15)	-0.84 (-0.87, -0.82)
25-29 years	264399.81 (184864.21, 347232.12)	59.74 (41.77, 78.45)	285681.5 (198851.07, 370411.45)	48.56 (33.8, 62.96)	-0.75 (-0.78, -0.73)
30-34 years	301248.52 (207286.87, 391962.84)	78.16 (53.78, 101.7)	400143.25 (274862.08, 517997.81)	66.2 (45.47, 85.69)	-0.67 (-0.72, -0.62)
35-39 years	368273.52 (258778.24, 477646.09)	104.55 (73.47, 135.6)	498430.32 (346915.43, 651427.43)	88.87 (61.85, 116.15)	-0.64 (-0.68, -0.6)
40-44 years	401728.14 (282935.15, 526172.29)	140.23 (98.76, 183.67)	610362.19 (437235.69, 790730.66)	122.01 (87.4, 158.07)	-0.56 (-0.59, -0.52)
45-49 years	474455.64 (339525.47, 611012.25)	204.33 (146.22, 263.15)	845996.35 (612391.78, 1091788.15)	178.67 (129.33, 230.58)	-0.49 (-0.55, -0.44)
50-54 years	650975.54 (468337.69, 838544.67)	306.24 (220.32, 394.48)	1167834.2 (843669.76, 1506013.26)	262.48 (189.62, 338.49)	-0.51 (-0.54, -0.48)
55-59 years	778397.84 (564580.09, 1003724.65)	420.3 (304.85, 541.97)	1470463.67 (1065444.86, 1886933.8)	371.58 (269.24, 476.83)	-0.47 (-0.5, -0.44)
60-64 years	892546.95 (651677.63, 1151058.05)	555.73 (405.75, 716.68)	1586275.4 (1151691.7, 2048987.7)	495.64 (359.85, 640.21)	-0.4 (-0.44, -0.35)
65-69 years	891935.65 (648488, 1152222.42)	721.58 (524.63, 932.15)	1890756.53 (1365396.19, 2455152.18)	685.45 (494.99, 890.06)	-0.32 (-0.37, -0.27)
70-74 years	805956.14 (576469.06, 1045867.12)	951.98 (680.91, 1235.36)	1880274.87 (1347752.11, 2443773.73)	913.47 (654.76, 1187.22)	-0.24 (-0.27, -0.21)
75-79 years	735027.63 (523436.05, 960068.3)	1194.09 (850.35, 1559.68)	1577624.85 (1141266.67, 2056531.22)	1196.22 (865.35, 1559.34)	-0.04 (-0.05, -0.02)
80-84 years	487391.14 (349136.45, 624738.92)	1377.75 (986.93, 1766)	1266648.56 (899293.9, 1626407.72)	1446.23 (1026.79, 1856.99)	0.12 (0.09, 0.15)
85-89 years	220868.47 (156819.8, 285675.13)	1461.63 (1037.78, 1890.5)	717441.99 (505279.9, 926791.02)	1569.15 (1105.12, 2027.02)	0.15 (0.13, 0.18)
90-94 years	66918.42 (46597.09, 87043.92)	1561.62 (1087.4, 2031.27)	297716.06 (208125.41, 387434.72)	1664.21 (1163.4, 2165.73)	0.17 (0.15, 0.2)
95 plus years	17133.27 (11974.7, 22268.26)	1682.89 (1176.2, 2187.27)	100903.75 (71203.5, 129623.77)	1851.34 (1306.41, 2378.28)	0.22 (0.17, 0.27)

Notes: ASR: age-standardized rates; YLDs: years lived with disability; EAPCs: estimated annual percentage changes.

Table S20. Prevalence and YLDs of Alzheimer's disease and other dementias and its temporal trends from 1990, 2021 by gender, and age in the worldwide.

	1990		2021		EAPC 95%CI
	Number	ASR, per 100,000 persons	Number	ASR, per 100,000 persons	
**Prevalence**					
**Gender**					
Female	14143445.64 (12361842.01, 16105291.71)	736.15 (646.05, 834.27)	36103379.82 (31468184.99, 41117469.75)	769.94 (670.71, 877.57)	0.04 (0.01, 0.06)
Male	7656315.26 (6611275.56, 8728099.19)	571.47 (497.63, 654.31)	20753308.39 (17769420.13, 23796797.51)	589.47 (507.48, 678.79)	0.03 (0.01, 0.05)
**Age**					
40-44 years	52557.99 (23182.71, 88251.89)	18.35 (8.09, 30.81)	86217.1 (37443.55, 146910.18)	17.23 (7.48, 29.37)	-0.22 (-0.24, -0.2)
45-49 years	280046.25 (178917.27, 415799.84)	120.61 (77.05, 179.07)	549138.48 (346656.83, 817086.23)	115.97 (73.21, 172.56)	-0.15 (-0.17, -0.13)
50-54 years	630632.31 (445284.45, 848763.32)	296.67 (209.48, 399.28)	1316046.58 (930375.96, 1775693.46)	295.79 (209.11, 399.1)	-0.03 (-0.05, -0.02)
55-59 years	1058762.08 (826971.83, 1336788.63)	571.69 (446.53, 721.81)	2345032.54 (1828789.78, 2960594.02)	592.59 (462.13, 748.14)	0.08 (0.06, 0.09)
60-64 years	1653349.59 (1281677.67, 2079461.71)	1029.42 (798.01, 1294.73)	3461247.53 (2683722.22, 4380526.69)	1081.48 (838.54, 1368.71)	0.15 (0.13, 0.17)
65-69 years	2234917.95 (1747451.39, 2812708.74)	1808.05 (1413.69, 2275.48)	5383792.63 (4185913.08, 6722380.19)	1951.77 (1517.5, 2437.04)	0.14 (0.12, 0.16)
70-74 years	2835286.72 (2219993.31, 3602656.05)	3348.98 (2622.21, 4255.38)	7364245.23 (5701395.92, 9397560.88)	3577.67 (2769.83, 4565.49)	0.08 (0.05, 0.1)
75-79 years	4128476.26 (3309816.55, 5065911.14)	6706.92 (5376.96, 8229.83)	9325099.49 (7423397.81, 11476764.31)	7070.66 (5628.72, 8702.14)	0.04 (0.01, 0.07)
80-84 years	4440386.28 (3587025.46, 5500308.94)	12552 (10139.74, 15548.17)	11387765.6 (9080701.96, 14175059.43)	13002.25 (10368.1, 16184.71)	-0.01 (-0.04, 0.02)
85-89 years	2990482.4 (2386113.02, 3673439.71)	19790.01 (15790.49, 24309.59)	9159172.08 (7253558.28, 11346546.18)	20032.4 (15864.55, 24816.5)	-0.06 (-0.09, -0.03)
90-94 years	1150861.68 (916046.9, 1421175.8)	26856.74 (21377.05, 33164.84)	4724243.74 (3711195.04, 5911668.66)	26408.1 (20745.25, 33045.7)	-0.11 (-0.13, -0.1)
95 plus years	344001.38 (270198.83, 428440.06)	33789.01 (26539.87, 42082.87)	1754687.21 (1360064.53, 2222942.38)	32194.27 (24953.9, 40785.62)	-0.18 (-0.2, -0.16)
**YLDs**					
**Gender**					
Female	2959271.49 (2024969.79, 3942681.72)	155.6 (106.59, 206.6)	7602653.88 (5202893.44, 10072814.04)	161.92 (110.67, 214.71)	0.02 (0, 0.05)
Male	1450511.88 (1002181.59, 1898467.88)	110.97 (76.86, 146.11)	3979454.13 (2759473.47, 5262333.47)	114.38 (78.55, 151.18)	0.03 (0.01, 0.05)
**Age**					
40-44 years	9857.01 (3998.17, 18145.73)	3.44 (1.4, 6.33)	16193.45 (6489.28, 30560.45)	3.24 (1.3, 6.11)	-0.22 (-0.23, -0.2)

45-49 years	50305.55 (28220.49, 82261.81)	21.67 (12.15, 35.43)	98653.03 (54650.55, 164093.95)	20.83 (11.54, 34.66)	-0.14 (-0.16, -0.12)
50-54 years	115436.57 (69638.17, 174052.1)	54.3 (32.76, 81.88)	241119 (146753.22, 363061.16)	54.19 (32.98, 81.6)	-0.02 (-0.04, -0.01)
55-59 years	199944.31 (124464.96, 282708.24)	107.96 (67.21, 152.65)	443030.33 (273166.13, 631248.07)	111.95 (69.03, 159.52)	0.09 (0.07, 0.1)
60-64 years	319468.81 (205248.21, 460272.94)	198.91 (127.79, 286.58)	667958.72 (426335.49, 958650.8)	208.71 (133.21, 299.53)	0.15 (0.13, 0.18)
65-69 years	442413.22 (291273.02, 643410.45)	357.91 (235.64, 520.52)	1063102.44 (694820.82, 1549608.5)	385.4 (251.89, 561.77)	0.14 (0.11, 0.16)
70-74 years	573841.98 (376814.6, 821626.62)	677.81 (445.09, 970.49)	1486345.44 (976579.01, 2130737.73)	722.09 (474.44, 1035.15)	0.07 (0.04, 0.1)
75-79 years	798146.91 (543382.65, 1110986.39)	1296.63 (882.75, 1804.85)	1792578.21 (1222617.17, 2498002.31)	1359.2 (927.04, 1894.08)	0.02 (0, 0.05)
80-84 years	910100.14 (592631.62, 1243943.9)	2572.65 (1675.24, 3516.36)	2321558.41 (1499740.79, 3210564.94)	2650.69 (1712.36, 3665.74)	-0.03 (-0.06, 0)
85-89 years	646955.24 (435749.38, 893339.44)	4281.33 (2883.64, 5911.82)	1969185.34 (1323169.82, 2726479.07)	4306.89 (2893.96, 5963.19)	-0.08 (-0.11, -0.06)
90-94 years	261756.96 (179075.06, 355048.77)	6108.41 (4178.93, 8285.49)	1068544.36 (722726.71, 1459102.29)	5973.07 (4039.98, 8156.25)	-0.13 (-0.15, -0.11)
95 plus years	81556.69 (56257.57, 112130.02)	8010.78 (5525.81, 11013.8)	413839.28 (285509.37, 568057.55)	7592.95 (5238.41, 10422.48)	-0.2 (-0.22, -0.18)

Notes: ASR: age-standardized rates; YLDs: years lived with disability; EAPCs: estimated annual percentage changes.

Table S21. Prevalence and YLDs of Parkinson's disease and its temporal trends from 1990, 2021 by gender, and age in the worldwide.

	1990		2021		EAPC 95%CI
	Number	ASR, per 100,000 persons	Number	ASR, per 100,000 persons	
**Prevalence**					
**Gender**					
Female	1589427.07 (1396725.56, 1826889.42)	77.09 (67.9, 88.09)	5328632.12 (4748783.45, 6042536.89)	114.47 (102.05, 129.78)	1.25 (1.23, 1.27)
Male	1558967.49 (1360361.5, 1801027.72)	99.22 (86.7, 113.81)	6438639.86 (5672343.51, 7401532.78)	168.24 (148.41, 191.71)	1.7 (1.67, 1.73)
**Age**					
20-24 years	1048.2 (270, 2046.93)	0.21 (0.05, 0.42)	1378.07 (344.98, 2678.19)	0.23 (0.06, 0.45)	0.35 (0.3, 0.39)
25-29 years	6495.65 (1680.62, 12689.47)	1.47 (0.38, 2.87)	9275.32 (2317.62, 18022.94)	1.58 (0.39, 3.06)	0.31 (0.27, 0.35)
30-34 years	16251.89 (7084.48, 28519.03)	4.22 (1.84, 7.4)	27958.95 (13220.81, 47769.55)	4.63 (2.19, 7.9)	0.39 (0.35, 0.42)
35-39 years	32929.31 (20092.48, 47844.25)	9.35 (5.7, 13.58)	61405.57 (38042.04, 89011.02)	10.95 (6.78, 15.87)	0.6 (0.58, 0.63)
40-44 years	52359.13 (34274.57, 79665.69)	18.28 (11.96, 27.81)	117862.7 (79942.1, 171391.33)	23.56 (15.98, 34.26)	0.9 (0.84, 0.95)
45-49 years	81402.33 (58378.39, 109573.09)	35.06 (25.14, 47.19)	265991.86 (194994.36, 353636.02)	56.18 (41.18, 74.68)	1.45 (1.32, 1.59)
50-54 years	132848.81 (94054.46, 180445.42)	62.5 (44.25, 84.89)	525898.74 (391247.93, 703354.4)	118.2 (87.94, 158.08)	1.82 (1.65, 1.98)
55-59 years	201727.53 (151571.84, 257924.45)	108.92 (81.84, 139.27)	836714.14 (651211.93, 1066128.56)	211.44 (164.56, 269.41)	1.83 (1.69, 1.97)
60-64 years	308179.66 (236473.64, 399765.69)	191.88 (147.24, 248.91)	1060264.09 (825630.52, 1366791.11)	331.28 (257.97, 427.06)	1.77 (1.67, 1.88)
65-69 years	450619.35 (364842.13, 557114.89)	364.55 (295.16, 450.71)	1676422.85 (1383237.23, 2060676.76)	607.75 (501.46, 747.05)	1.6 (1.57, 1.63)
70-74 years	528921.29 (422285.15, 653764.9)	624.75 (498.79, 772.21)	1999449.48 (1630854.56, 2417047.66)	971.36 (792.3, 1174.24)	1.42 (1.4, 1.44)
75-79 years	606246.36 (505860.81, 722880.39)	984.88 (821.8, 1174.36)	1950769.68 (1630158.86, 2305021.04)	1479.15 (1236.05, 1747.76)	1.38 (1.35, 1.41)
80-84 years	456087.83 (370242.27, 548056.86)	1289.26 (1046.59, 1549.24)	1732586.51 (1427648.92, 2076015.54)	1978.22 (1630.05, 2370.34)	1.42 (1.38, 1.47)
85-89 years	205773.48 (169427.61, 247818.4)	1361.74 (1121.21, 1639.98)	1017289.14 (848111.74, 1200132.14)	2224.95 (1854.94, 2624.86)	1.56 (1.54, 1.59)
90-94 years	55050.96 (44312.66, 68607.63)	1284.68 (1034.09, 1601.04)	380063.43 (311897.5, 467026.34)	2124.52 (1743.48, 2610.64)	1.66 (1.63, 1.69)
95 plus years	12452.78 (9133.73, 16917.46)	1223.16 (897.15, 1661.69)	103941.42 (80213.92, 134143.92)	1907.07 (1471.73, 2461.22)	1.54 (1.48, 1.59)
**YLDs**					
**Gender**					

Female	225249.18 (158016.69, 300356.78)	10.87 (7.68, 14.44)	747064.28 (529483.24, 986267.03)	16.07 (11.38, 21.21)	1.24 (1.22, 1.26)
Male	224986.42 (158623.02, 301826.46)	14.09 (9.99, 18.59)	923231.96 (646455.25, 1220656.45)	23.95 (16.97, 31.52)	1.71 (1.68, 1.74)
**Age**					
20-24 years	176.05 (45.21, 346.47)	0.04 (0.01, 0.07)	231.38 (57.97, 453.01)	0.04 (0.01, 0.08)	0.35 (0.3, 0.39)
25-29 years	1090.96 (281.22, 2142.49)	0.25 (0.06, 0.48)	1557.37 (388.86, 3049.22)	0.26 (0.07, 0.52)	0.31 (0.27, 0.35)
30-34 years	2727.42 (1131.24, 4893.02)	0.71 (0.29, 1.27)	4690.61 (2034.08, 8252.82)	0.78 (0.34, 1.37)	0.39 (0.35, 0.42)
35-39 years	5517.91 (2914.86, 8770.26)	1.57 (0.83, 2.49)	10285.53 (5675.15, 16536.21)	1.83 (1.01, 2.95)	0.6 (0.58, 0.63)
40-44 years	8739.44 (4984.78, 13930.6)	3.05 (1.74, 4.86)	19493.1 (11352.11, 30681.5)	3.9 (2.27, 6.13)	0.86 (0.8, 0.91)
45-49 years	13169.07 (8163.34, 19475.64)	5.67 (3.52, 8.39)	42165.49 (26924.25, 62081.15)	8.9 (5.69, 13.11)	1.39 (1.26, 1.53)
50-54 years	21034.96 (12638.77, 31314.24)	9.9 (5.95, 14.73)	81252.95 (50513.07, 119681.36)	18.26 (11.35, 26.9)	1.76 (1.6, 1.92)
55-59 years	30320.22 (19262.87, 43558.54)	16.37 (10.4, 23.52)	126009.07 (83731.26, 180690.17)	31.84 (21.16, 45.66)	1.84 (1.69, 1.98)
60-64 years	45398.91 (29654.16, 64964.81)	28.27 (18.46, 40.45)	156855.7 (102825.66, 220141.09)	49.01 (32.13, 68.78)	1.79 (1.69, 1.9)
65-69 years	65248.35 (44319.08, 88716.84)	52.79 (35.85, 71.77)	243266.58 (164953.7, 328109.87)	88.19 (59.8, 118.95)	1.62 (1.58, 1.65)
70-74 years	74981.63 (50666.62, 105459.56)	88.57 (59.85, 124.57)	283703.62 (192699.61, 392271.33)	137.83 (93.62, 190.57)	1.43 (1.4, 1.45)
75-79 years	84133.98 (59853.15, 112259.86)	136.68 (97.23, 182.37)	270476.7 (190563.39, 363069.44)	205.09 (144.49, 275.29)	1.38 (1.35, 1.41)
80-84 years	61873.7 (42851.43, 84367.78)	174.9 (121.13, 238.49)	234538.94 (162459.13, 314569.39)	267.79 (185.49, 359.17)	1.42 (1.37, 1.46)
85-89 years	27184.45 (18949.33, 36829.44)	179.9 (125.4, 243.72)	134021.04 (95707.03, 178324.31)	293.12 (209.32, 390.02)	1.56 (1.53, 1.58)
90-94 years	7080.83 (5043.75, 9572.67)	165.24 (117.7, 223.39)	48793.12 (34805.64, 66057.66)	272.75 (194.56, 369.26)	1.65 (1.62, 1.69)
95 plus years	1557.73 (1058.29, 2247.72)	153.01 (103.95, 220.78)	12955.04 (9075.65, 18025.49)	237.69 (166.52, 330.72)	1.53 (1.47, 1.59)

Notes: ASR: age-standardized rates; YLDs: years lived with disability; EAPCs: estimated annual percentage changes.

Table S22. Prevalence and YLDs of upper digestive system diseases and its temporal trends from 1990, 2021 by gender, and age in the worldwide.

	1990		2021		EAPC 95%CI
	Number	ASR, per 100,000 persons	Number	ASR, per 100,000 persons	
**Prevalence**					
**Gender**					
Female	251132656.71 (224649389.05, 282034557.11)	10494.22 (9358.56, 11696.76)	456511962.03 (407791204.87, 508516913.99)	10738.92 (9619.49, 12023.88)	0.04 (-0.01, 0.08)
Male	226285226.51 (200453001.78, 255948597.2)	9668.55 (8597.85, 10751.58)	407772115.48 (361944727.99, 455875009.12)	9852.94 (8763.22, 11014.76)	0.04 (0.01, 0.07)
**Age**					
<5 years	827209.24 (564785.8, 1180705.02)	133.43 (91.1, 190.46)	633948.24 (434707.93, 896154.74)	96.32 (66.05, 136.16)	-1.1 (-1.25, -0.95)
5-9 years	1184744.78 (738686.33, 1890448.4)	203.03 (126.59, 323.97)	1093565.23 (706195.28, 1674177.15)	159.17 (102.79, 243.67)	-0.93 (-0.99, -0.86)
10-14 years	4144569.46 (2975294.69, 5469867.28)	773.7 (555.42, 1021.1)	5085232.25 (3665072.13, 6768121.15)	762.82 (549.79, 1015.26)	-0.01 (-0.03, 0.01)
15-19 years	17536545.97 (12851995.56, 23053387.42)	3376.16 (2474.28, 4438.26)	22582011.87 (16524351.66, 29794857.64)	3619.03 (2648.22, 4774.97)	0.18 (0.15, 0.22)
20-24 years	38457707.62 (28224101.08, 49753348.36)	7815.21 (5735.58, 10110.66)	52100498.18 (38305514.93, 67538118.71)	8724.74 (6414.64, 11309.92)	0.26 (0.19, 0.33)
25-29 years	50956426.42 (38679271.55, 66319321.38)	11512.46 (8738.72, 14983.37)	74133962.18 (56220185.45, 96427212.73)	12600.46 (9555.68, 16389.62)	0.28 (0.23, 0.33)
30-34 years	53823576.13 (39488640.22, 70787972.66)	13964.83 (10245.55, 18366.33)	88059208.75 (64626442.69, 115703149.85)	14567.76 (10691.24, 19140.93)	0.21 (0.16, 0.27)
35-39 years	53121884.05 (38757549.55, 70851446.9)	15080.97 (11003.03, 20114.28)	90708033.66 (65870825.8, 121089104.89)	16172.85 (11744.48, 21589.66)	0.21 (0.12, 0.3)
40-44 years	46283533.8 (33682695.55, 61530665.63)	16155.85 (11757.37, 21478.05)	85193978.39 (61932540.67, 113215297.88)	17030.25 (12380.3, 22631.71)	0.08 (-0.02, 0.18)
45-49 years	39247273.28 (29332882.43, 50680832.7)	16902.69 (12632.84, 21826.79)	80029458.76 (59434763.71, 103529279.51)	16901.52 (12552.1, 21864.47)	-0.08 (-0.15, -0.02)
50-54 years	37747404.28 (27551588.76, 49227596.37)	17757.51 (12961.09, 23158.14)	76674475.04 (55992531.56, 99818587.14)	17233.2 (12584.77, 22435.03)	-0.14 (-0.18, -0.11)
55-59 years	35615723.46 (26574321.65, 45155364.51)	19230.96 (14348.99, 24381.95)	73726505.81 (55106710.67, 93158655.65)	18630.6 (13925.4, 23541.08)	-0.11 (-0.17, -0.06)
60-64 years	31758233.44 (24724329.98, 40709844.29)	19773.6 (15394.09, 25347.14)	62592602.59 (48611151.85, 80334921.63)	19557.26 (15188.71, 25100.91)	-0.12 (-0.17, -0.07)
65-69 years	25395136.81 (19443402.19, 32319421.78)	20544.66 (15729.7, 26146.4)	54715047.51 (41994840.25, 69656111.41)	19835.64 (15224.23, 25252.16)	-0.13 (-0.15, -0.11)
70-74 years	17818040.39 (13834469.96, 22200623.14)	21046.3 (16340.99, 26222.92)	41878337.45 (32485253.47, 51924049.51)	20345.17 (15781.86, 25225.54)	-0.11 (-0.13, -0.08)
75-79 years	12914884.17 (10012103.68, 16447892.93)	20980.87 (16265.16, 26720.42)	26747475.27 (20631196.8, 34116527.07)	20281 (15643.39, 25868.51)	-0.11 (-0.14, -0.07)
80-84 years	6989330.29 (5226868.27, 9086601.7)	19757.31 (14775.21, 25685.83)	16736293.63 (12528556.41, 21731231.25)	19109.05 (14304.77, 24812.14)	-0.13 (-0.16, -0.1)
85-89 years	2704616.43 (2024256.91, 3592690.89)	17898.24 (13395.85, 23775.22)	7818065.96 (5851183.86, 10375873.61)	17099.21 (12797.36, 22693.5)	-0.15 (-0.18, -0.13)

90-94 years	710317.08 (520424.46, 922763.87)	16576.1 (12144.73, 21533.8)	2852847.75 (2097727.28, 3702748.63)	15947.16 (11726.11, 20698.03)	-0.13 (-0.15, -0.12)
95 plus years	180726.11 (133166.28, 234412.65)	17751.55 (13080.06, 23024.83)	922529 (678878.75, 1205761.08)	16926.18 (12455.79, 22122.8)	-0.1 (-0.13, -0.07)
**YLDs**					
**Gender**					
Female	2612642.93 (1558958.43, 4233754.47)	109.03 (64.73, 176.44)	4523292.45 (2630521.36, 7379255.19)	106.52 (61.69, 174.2)	-0.09 (-0.11, -0.07)
Male	2288864.31 (1342068.22, 3754830.45)	97.84 (57.34, 159.7)	3856334.94 (2186642.83, 6428296.08)	93.22 (52.72, 155.08)	-0.19 (-0.22, -0.16)
**Age**					
<5 years	42913.57 (23670.47, 68678)	6.92 (3.82, 11.08)	32296.25 (17951.17, 51060.89)	4.91 (2.73, 7.76)	-1.17 (-1.3, -1.04)
5-9 years	55295.68 (27649.56, 100180.17)	9.48 (4.74, 17.17)	49495.98 (25303.69, 87933.5)	7.2 (3.68, 12.8)	-0.96 (-1.01, -0.92)
10-14 years	78214.66 (45880.31, 125992.27)	14.6 (8.56, 23.52)	89605.45 (53427.74, 144160.4)	13.44 (8.01, 21.63)	-0.18 (-0.23, -0.14)
15-19 years	192393.7 (105882.53, 319024.82)	37.04 (20.38, 61.42)	239424.71 (129843.69, 398202.4)	38.37 (20.81, 63.82)	0.12 (0.09, 0.14)
20-24 years	370714.77 (196497.23, 642450.27)	75.34 (39.93, 130.56)	487063.8 (252165.68, 848351.2)	81.56 (42.23, 142.06)	0.18 (0.12, 0.24)
25-29 years	476569.27 (254031.41, 849197.51)	107.67 (57.39, 191.86)	671991.63 (350929.53, 1212658.59)	114.22 (59.65, 206.11)	0.17 (0.12, 0.22)
30-34 years	501513.81 (265519.96, 881148.67)	130.12 (68.89, 228.62)	793272.51 (419169.8, 1411559.88)	131.23 (69.34, 233.52)	0.09 (0.05, 0.12)
35-39 years	506662.81 (277319.28, 913360.6)	143.84 (78.73, 259.3)	822421.39 (438297.75, 1497306.86)	146.63 (78.15, 266.96)	0.05 (-0.01, 0.11)
40-44 years	456429.19 (251037.53, 780967.32)	159.32 (87.63, 272.61)	790758.23 (423054.9, 1388067.68)	158.07 (84.57, 277.47)	-0.1 (-0.17, -0.03)
45-49 years	402051.55 (223284.38, 679591.29)	173.15 (96.16, 292.68)	772160.05 (426366.19, 1315561.22)	163.07 (90.04, 277.83)	-0.26 (-0.3, -0.21)
50-54 years	400065.74 (225694.81, 671863.89)	188.2 (106.17, 316.06)	768144.55 (429134.81, 1302678.25)	172.65 (96.45, 292.79)	-0.31 (-0.33, -0.3)
55-59 years	386238.67 (221837.86, 651864.16)	208.55 (119.78, 351.98)	749673.33 (422581.14, 1271936.37)	189.44 (106.79, 321.42)	-0.33 (-0.36, -0.3)
60-64 years	339881.78 (201305.29, 567056.76)	211.62 (125.34, 353.07)	620324.67 (360156.99, 1048837.78)	193.82 (112.53, 327.71)	-0.33 (-0.37, -0.3)
65-69 years	267493.01 (160716.93, 444875.19)	216.4 (130.02, 359.9)	545195.58 (326222.79, 906269.44)	197.65 (118.26, 328.55)	-0.3 (-0.32, -0.28)
70-74 years	187353.12 (110128.76, 298582.06)	221.3 (130.08, 352.68)	413909.68 (239230.54, 671646.41)	201.08 (116.22, 326.3)	-0.29 (-0.31, -0.27)
75-79 years	132634.73 (78511.5, 205500.81)	215.47 (127.55, 333.85)	262711.19 (152831.94, 414770.65)	199.2 (115.88, 314.5)	-0.28 (-0.31, -0.26)
80-84 years	70764.66 (41695.28, 111417.14)	200.04 (117.86, 314.95)	162893.96 (94176.85, 258659.71)	185.99 (107.53, 295.33)	-0.28 (-0.32, -0.23)
85-89 years	26272.15 (15558.92, 43677.64)	173.86 (102.96, 289.04)	74154.19 (43313.6, 124392.91)	162.19 (94.73, 272.06)	-0.26 (-0.3, -0.21)
90-94 years	6481.75 (3746.49, 10965.58)	151.26 (87.43, 255.9)	26031.34 (15223.15, 44148.54)	145.51 (85.1, 246.79)	-0.14 (-0.18, -0.11)
95 plus years	1562.61 (910.51, 2604.28)	153.48 (89.43, 255.8)	8098.89 (4785.87, 13370.31)	148.6 (87.81, 245.31)	-0.11 (-0.14, -0.09)

Notes: ASR: age-standardized rates; YLDs: years lived with disability; EAPCs: estimated annual percentage changes.

Table S23. Prevalence and YLDs of inflammatory bowel disease and its temporal trends from 1990, 2021 by gender, and age in the worldwide.

	1990		2021		EAPC 95%CI
	Number	ASR, per 100,000 persons	Number	ASR, per 100,000 persons	
**Prevalence**					
**Gender**					
Female	1137825.28 (991143.41, 1324604.71)	49.43 (43.2, 57.62)	2000477.89 (1731570.92, 2351092.31)	45.9 (39.71, 53.97)	-0.15 (-0.27, -0.02)
Male	1032418.02 (898681.43, 1193482.57)	46.65 (40.72, 53.79)	1829641.37 (1577325.45, 2155695.95)	43.91 (37.88, 51.62)	-0.1 (-0.22, 0.02)
**Age**					
<5 years	108.67 (70.7, 151.08)	0.02 (0.01, 0.02)	120.38 (77.88, 167.13)	0.02 (0.01, 0.03)	0.14 (0.05, 0.23)
5-9 years	2550.38 (1889.38, 3197.76)	0.44 (0.32, 0.55)	2917.55 (2140.19, 3690.29)	0.42 (0.31, 0.54)	-0.06 (-0.33, 0.21)
10-14 years	11914.01 (9559.92, 14568.16)	2.22 (1.78, 2.72)	13795.05 (10789.96, 17213.73)	2.07 (1.62, 2.58)	-0.2 (-0.54, 0.14)
15-19 years	36978.3 (30183.59, 44764.52)	7.12 (5.81, 8.62)	40161.82 (31670.59, 51100.25)	6.44 (5.08, 8.19)	-0.28 (-0.62, 0.06)
20-24 years	84824.69 (69442.88, 104570.36)	17.24 (14.11, 21.25)	91188.62 (71859.43, 116652.82)	15.27 (12.03, 19.53)	-0.3 (-0.59, 0)
25-29 years	148844.44 (122564.59, 182812.98)	33.63 (27.69, 41.3)	168730.36 (132129.18, 216194.42)	28.68 (22.46, 36.75)	-0.3 (-0.56, -0.05)
30-34 years	201104.42 (163409.57, 247792.99)	52.18 (42.4, 64.29)	262930.65 (203605.84, 340248.91)	43.5 (33.68, 56.29)	-0.42 (-0.62, -0.21)
35-39 years	233397.09 (190130.89, 290792.26)	66.26 (53.98, 82.55)	336260.66 (260311.13, 426957.68)	59.95 (46.41, 76.12)	-0.29 (-0.42, -0.16)
40-44 years	243137.15 (197483.55, 295403.16)	84.87 (68.93, 103.11)	379362.71 (298055.36, 476550.96)	75.83 (59.58, 95.26)	-0.31 (-0.43, -0.19)
45-49 years	222195.24 (182266.22, 270954.86)	95.69 (78.5, 116.69)	405981.54 (325007.43, 501172.94)	85.74 (68.64, 105.84)	-0.28 (-0.46, -0.1)
50-54 years	216406.33 (174652.73, 262153.11)	101.8 (82.16, 123.32)	428381.61 (341764.9, 523916.71)	96.28 (76.81, 117.75)	-0.09 (-0.28, 0.1)
55-59 years	195279.87 (162568.15, 233313.09)	105.44 (87.78, 125.98)	422094.03 (346980.76, 510022.47)	106.66 (87.68, 128.88)	0.21 (0.09, 0.34)
60-64 years	181269.36 (149612.84, 217261.63)	112.86 (93.15, 135.27)	379809.53 (311989.77, 456677.66)	118.67 (97.48, 142.69)	0.28 (0.16, 0.4)
65-69 years	149275.42 (122450.49, 179019.9)	120.76 (99.06, 144.83)	317528.77 (255786.8, 379438.49)	115.11 (92.73, 137.56)	0.15 (0.01, 0.29)
70-74 years	100979.38 (82120.98, 121184.66)	119.27 (97, 143.14)	247278.61 (199176.59, 297141.98)	120.13 (96.76, 144.36)	0.02 (-0.1, 0.14)
75-79 years	74573.7 (59542.55, 91480.84)	121.15 (96.73, 148.62)	154506.02 (122742.69, 192024.88)	117.15 (93.07, 145.6)	-0.23 (-0.32, -0.14)
80-84 years	42407.59 (34154.26, 52385.77)	119.88 (96.55, 148.08)	100192.39 (80397.2, 123616.87)	114.4 (91.8, 141.14)	-0.35 (-0.42, -0.27)
85-89 years	18008.27 (14579.12, 22381.56)	119.17 (96.48, 148.11)	51092.66 (41367.53, 63361.48)	111.75 (90.48, 138.58)	-0.26 (-0.38, -0.14)

90-94 years	5564.22 (4368.36, 6931.19)	129.85 (101.94, 161.75)	21265.32 (16703.53, 26496.68)	118.87 (93.37, 148.11)	-0.24 (-0.32, -0.16)
95 plus years	1424.77 (1091.12, 1830.97)	139.95 (107.17, 179.84)	6521.01 (4863.68, 8521.26)	119.64 (89.24, 156.34)	-0.6 (-0.68, -0.52)
**YLDs**					
**Gender**					
Female	176404.63 (119546.98, 243200.99)	7.62 (5.2, 10.51)	307741.14 (208255.9, 423747.41)	7.09 (4.8, 9.73)	-0.13 (-0.25, -0.01)
Male	154471.73 (102974.78, 214567.47)	6.93 (4.66, 9.57)	271461.39 (183519.3, 376559.23)	6.51 (4.41, 8.99)	-0.11 (-0.23, 0.02)
**Age**					
<5 years	18.9 (11.02, 30.88)	0 (0, 0)	20.8 (12.05, 33.61)	0 (0, 0.01)	0.11 (0.03, 0.19)
5-9 years	441.34 (279.55, 666.39)	0.08 (0.05, 0.11)	504.72 (315.92, 770.11)	0.07 (0.05, 0.11)	-0.04 (-0.3, 0.22)
10-14 years	1980.83 (1297.43, 2856.93)	0.37 (0.24, 0.53)	2305.72 (1511.02, 3341.33)	0.35 (0.23, 0.5)	-0.17 (-0.5, 0.16)
15-19 years	6037.19 (3947.47, 8508.42)	1.16 (0.76, 1.64)	6605.33 (4215.36, 9401.76)	1.06 (0.68, 1.51)	-0.25 (-0.57, 0.08)
20-24 years	13715.39 (8760.67, 19306.65)	2.79 (1.78, 3.92)	14819.72 (9389.98, 20926.91)	2.48 (1.57, 3.5)	-0.28 (-0.56, 0.01)
25-29 years	23742.13 (15226.35, 33360.93)	5.36 (3.44, 7.54)	27132.75 (17165.68, 39539.88)	4.61 (2.92, 6.72)	-0.27 (-0.52, -0.01)
30-34 years	31839.27 (20300.52, 47020.14)	8.26 (5.27, 12.2)	41847.03 (26383.66, 62490.81)	6.92 (4.36, 10.34)	-0.39 (-0.59, -0.19)
35-39 years	36646.05 (23339.11, 53509.56)	10.4 (6.63, 15.19)	52976 (32350.87, 77740.83)	9.45 (5.77, 13.86)	-0.28 (-0.4, -0.15)
40-44 years	37820.81 (24086.94, 54251.8)	13.2 (8.41, 18.94)	59006.49 (37193.52, 85228.95)	11.8 (7.43, 17.04)	-0.3 (-0.41, -0.19)
45-49 years	34040.52 (21588.2, 48890.72)	14.66 (9.3, 21.06)	62166.41 (39250.9, 89218.4)	13.13 (8.29, 18.84)	-0.27 (-0.44, -0.1)
50-54 years	32781.41 (21676.64, 47483.26)	15.42 (10.2, 22.34)	64849.71 (42253.58, 92822.7)	14.58 (9.5, 20.86)	-0.08 (-0.27, 0.1)
55-59 years	29246.03 (19223.29, 41637.92)	15.79 (10.38, 22.48)	63169.18 (41738.05, 90898.61)	15.96 (10.55, 22.97)	0.21 (0.09, 0.34)
60-64 years	26710.81 (18211.3, 39059.63)	16.63 (11.34, 24.32)	55890.16 (38489.25, 79903.69)	17.46 (12.03, 24.97)	0.27 (0.16, 0.39)
65-69 years	21629.49 (14310.57, 29855.19)	17.5 (11.58, 24.15)	46140.19 (30762.91, 63367.79)	16.73 (11.15, 22.97)	0.15 (0.02, 0.29)
70-74 years	14387.27 (9458.75, 20062.47)	16.99 (11.17, 23.7)	35226.77 (22847.23, 48759.38)	17.11 (11.1, 23.69)	0.03 (-0.08, 0.15)
75-79 years	10485.07 (6962.78, 14994.27)	17.03 (11.31, 24.36)	21788.93 (14078.18, 31210.34)	16.52 (10.67, 23.66)	-0.2 (-0.29, -0.11)
80-84 years	5900.08 (3957.69, 8352.43)	16.68 (11.19, 23.61)	13935.96 (9437.22, 19808.61)	15.91 (10.78, 22.62)	-0.33 (-0.39, -0.26)
85-89 years	2484.48 (1628.58, 3509.69)	16.44 (10.78, 23.23)	6984.96 (4703.63, 9874.55)	15.28 (10.29, 21.6)	-0.29 (-0.4, -0.18)
90-94 years	772.17 (520.72, 1077.08)	18.02 (12.15, 25.13)	2930.83 (1977.67, 4051.49)	16.38 (11.06, 22.65)	-0.27 (-0.35, -0.2)
95 plus years	197.12 (128.41, 276)	19.36 (12.61, 27.11)	900.88 (592.93, 1262.43)	16.53 (10.88, 23.16)	-0.62 (-0.69, -0.54)

Notes: ASR: age-standardized rates; YLDs: years lived with disability; EAPCs: estimated annual percentage changes.

Table S24. Prevalence and YLDs of pancreatitis and its temporal trends from 1990, 2021 by gender, and age in the worldwide.

	1990		2021		EAPC 95%CI
	Number	ASR, per 100,000 persons	Number	ASR, per 100,000 persons	
**Prevalence**					
**Gender**					
Female	1840332.31 (1346555.06, 2512793.02)	81.63 (59.19, 111.94)	2620897.02 (1935345.56, 3533534.78)	59.34 (44.23, 79.51)	-1.12 (-1.19, -1.06)
Male	2307972.85 (1682011.86, 3216325.32)	104.92 (76.41, 146.5)	3279443.18 (2420766.36, 4432087.03)	78.57 (58.11, 105.75)	-1.04 (-1.09, -0.98)
**Age**					
<5 years	13891.94 (7332.93, 24356.51)	2.24 (1.18, 3.93)	13221.89 (6784.09, 23563.89)	2.01 (1.03, 3.58)	-0.21 (-0.32, -0.11)
5-9 years	43196.1 (23567.57, 71549.92)	7.4 (4.04, 12.26)	44647.74 (23669.78, 75341.15)	6.5 (3.45, 10.97)	-0.37 (-0.46, -0.28)
10-14 years	65258.9 (36172.95, 107177.03)	12.18 (6.75, 20.01)	72065.74 (38654.92, 117628.01)	10.81 (5.8, 17.65)	-0.46 (-0.52, -0.39)
15-19 years	91426.07 (52184.99, 149426.69)	17.6 (10.05, 28.77)	98619.81 (56660.32, 161295.53)	15.8 (9.08, 25.85)	-0.48 (-0.53, -0.44)
20-24 years	121190.58 (73322.64, 198390.69)	24.63 (14.9, 40.32)	130962.85 (80616.7, 213752.14)	21.93 (13.5, 35.79)	-0.42 (-0.52, -0.31)
25-29 years	172602.96 (103234.15, 274334.45)	39 (23.32, 61.98)	183100.89 (110189.88, 294668.89)	31.12 (18.73, 50.08)	-0.4 (-0.53, -0.27)
30-34 years	240952.35 (142945.09, 384479.09)	62.52 (37.09, 99.76)	279087.07 (169099.37, 443782.41)	46.17 (27.97, 73.42)	-0.7 (-0.9, -0.51)
35-39 years	314083.51 (180190.21, 539248.89)	89.17 (51.15, 153.09)	377647.37 (224849.97, 623037.16)	67.33 (40.09, 111.08)	-1.1 (-1.3, -0.9)
40-44 years	347505.51 (219412.26, 578953.09)	121.3 (76.59, 202.09)	454367.41 (292523.62, 730752.75)	90.83 (58.48, 146.08)	-1.41 (-1.6, -1.21)
45-49 years	347311.68 (224975.38, 526193.97)	149.58 (96.89, 226.62)	536473.45 (348470.28, 793511.81)	113.3 (73.59, 167.58)	-1.25 (-1.57, -0.94)
50-54 years	492249.27 (322771.95, 751829.18)	231.57 (151.84, 353.68)	617223.91 (420016.08, 930387.62)	138.73 (94.4, 209.11)	-1.35 (-1.64, -1.06)
55-59 years	470391.96 (294391.81, 719499.7)	253.99 (158.96, 388.5)	696922.05 (445932.15, 1052107.36)	176.11 (112.69, 265.87)	-1.09 (-1.33, -0.85)
60-64 years	499454.7 (306031.78, 734516.02)	310.98 (190.54, 457.33)	709413.08 (452284.31, 1041226.92)	221.66 (141.32, 325.33)	-1.23 (-1.46, -1)
65-69 years	353170.55 (216554.67, 536589.38)	285.71 (175.19, 434.1)	616246.4 (396595.92, 920469.68)	223.41 (143.78, 333.69)	-1.35 (-1.61, -1.09)
70-74 years	234182.42 (144190.47, 360284.58)	276.61 (170.31, 425.56)	472800.55 (303728.07, 702707.24)	229.69 (147.56, 341.39)	-1.07 (-1.41, -0.73)
75-79 years	190471.58 (115961.45, 282327.85)	309.43 (188.39, 458.66)	263125.21 (172114.17, 375417.46)	199.51 (130.5, 284.66)	-0.94 (-1.2, -0.67)
80-84 years	99923.51 (65610.09, 146281.08)	282.46 (185.47, 413.5)	198088.89 (135439.28, 278062.74)	226.17 (154.64, 317.48)	-0.77 (-0.96, -0.59)
85-89 years	39017 (25735.73, 57182.37)	258.2 (170.31, 378.41)	90924.29 (63158.97, 129354.04)	198.86 (138.14, 282.92)	-0.74 (-0.87, -0.61)

90-94 years	9853.09 (6437.4, 14364.41)	229.93 (150.22, 335.21)	35715.02 (24058.59, 51128.79)	199.64 (134.49, 285.81)	-0.59 (-0.73, -0.45)
95 plus years	2171.48 (1411.47, 3278.47)	213.29 (138.64, 322.02)	9686.57 (6475.93, 14493.9)	177.73 (118.82, 265.93)	-0.63 (-0.76, -0.5)
**YLDs**					
**Gender**					
Female	112575.42 (54007.05, 194785.41)	4.95 (2.36, 8.54)	163556.83 (80142.8, 278750.89)	3.73 (1.83, 6.37)	-1 (-1.04, -0.95)
Male	140735.93 (66776.89, 252166.52)	6.32 (2.99, 11.32)	203442.24 (98222.13, 352421.8)	4.88 (2.37, 8.46)	-0.92 (-0.96, -0.88)
**Age**					
<5 years	1048.6 (513.72, 1952.47)	0.17 (0.08, 0.31)	1002.26 (489.89, 1844.21)	0.15 (0.07, 0.28)	-0.24 (-0.33, -0.15)
5-9 years	3090.78 (1425.02, 5903.69)	0.53 (0.24, 1.01)	3247.25 (1495.82, 6168.59)	0.47 (0.22, 0.9)	-0.32 (-0.39, -0.25)
10-14 years	4564.65 (2161.95, 8322.32)	0.85 (0.4, 1.55)	5218.14 (2563.4, 9425)	0.78 (0.38, 1.41)	-0.3 (-0.35, -0.26)
15-19 years	6389.56 (3160.97, 11829.04)	1.23 (0.61, 2.28)	7225.19 (3652.13, 13241.51)	1.16 (0.59, 2.12)	-0.29 (-0.32, -0.26)
20-24 years	8582.15 (4199.62, 15867.76)	1.74 (0.85, 3.22)	9693.79 (4732.61, 17595.44)	1.62 (0.79, 2.95)	-0.27 (-0.35, -0.2)
25-29 years	12110.18 (5737.32, 22410.14)	2.74 (1.3, 5.06)	13493.27 (6436.92, 24471.27)	2.29 (1.09, 4.16)	-0.33 (-0.43, -0.23)
30-34 years	16500.67 (8055.76, 31222.08)	4.28 (2.09, 8.1)	20124.68 (10030.83, 38811.33)	3.33 (1.66, 6.42)	-0.6 (-0.77, -0.42)
35-39 years	20875.65 (10105.04, 38677.02)	5.93 (2.87, 10.98)	26044.43 (12966.21, 47467.95)	4.64 (2.31, 8.46)	-0.94 (-1.13, -0.76)
40-44 years	22182.77 (10474.65, 42289.65)	7.74 (3.66, 14.76)	30004.45 (14362.61, 56145.96)	6 (2.87, 11.22)	-1.23 (-1.39, -1.07)
45-49 years	21620.41 (9808.88, 42135.97)	9.31 (4.22, 18.15)	34268.58 (15947.44, 65285.54)	7.24 (3.37, 13.79)	-1.13 (-1.39, -0.86)
50-54 years	28894.57 (12123.07, 54766.17)	13.59 (5.7, 25.76)	38044.54 (16891.28, 69901.39)	8.55 (3.8, 15.71)	-1.23 (-1.47, -0.99)
55-59 years	27071.27 (11274.28, 51755.7)	14.62 (6.09, 27.95)	41194.63 (17642.52, 76837.3)	10.41 (4.46, 19.42)	-1.01 (-1.23, -0.79)
60-64 years	27940.83 (11979.34, 51720.65)	17.4 (7.46, 32.2)	40513.01 (17925.34, 73441.95)	12.66 (5.6, 22.95)	-1.14 (-1.35, -0.94)
65-69 years	19934.09 (9080.82, 36505.96)	16.13 (7.35, 29.53)	35166.01 (16458.97, 63729.83)	12.75 (5.97, 23.1)	-1.25 (-1.48, -1.02)
70-74 years	13246.01 (6134.62, 23619.06)	15.65 (7.25, 27.9)	26895.69 (12581.51, 48494.47)	13.07 (6.11, 23.56)	-1 (-1.29, -0.71)
75-79 years	10625.13 (5040.29, 19103.97)	17.26 (8.19, 31.04)	15342.39 (7597.75, 26707.62)	11.63 (5.76, 20.25)	-0.87 (-1.09, -0.65)
80-84 years	5654.13 (2793.91, 9821.41)	15.98 (7.9, 27.76)	11404.08 (5777.3, 19740.02)	13.02 (6.6, 22.54)	-0.71 (-0.87, -0.56)
85-89 years	2262.3 (1175.44, 3995.02)	14.97 (7.78, 26.44)	5398.6 (2860.23, 9394.36)	11.81 (6.26, 20.55)	-0.68 (-0.78, -0.57)
90-94 years	586.78 (304.92, 1052.17)	13.69 (7.12, 24.55)	2130.22 (1121.69, 3807.71)	11.91 (6.27, 21.28)	-0.54 (-0.65, -0.42)
95 plus years	130.83 (67.67, 219.5)	12.85 (6.65, 21.56)	587.86 (314.58, 1009.42)	10.79 (5.77, 18.52)	-0.58 (-0.69, -0.48)

Notes: ASR: age-standardized rates; YLDs: years lived with disability; EAPCs: estimated annual percentage changes.

Table S25. Prevalence and YLDs of male infertility and its temporal trends from 1990, 2021 by gender, and age in the worldwide.

	1990		2021		EAPC 95%CI
	Number	ASR, per 100,000 persons	Number	ASR, per 100,000 persons	
**Prevalence**					
**Age**					
15-19 years	789213.42 (229366.37, 2054624.29)	151.94 (44.16, 395.56)	1103161.94 (289609.12, 2738023.32)	176.79 (46.41, 438.8)	0.26 (0.13, 0.38)
20-24 years	4933388.1 (2098271.61, 9607440.61)	1002.54 (426.4, 1952.38)	7796075.36 (3173774.77, 15566067.83)	1305.53 (531.48, 2606.69)	0.84 (0.66, 1.02)
25-29 years	5927405.09 (2465728.22, 12773838.59)	1339.16 (557.08, 2885.96)	10142512.02 (4001135.11, 21323101.97)	1723.91 (680.07, 3624.26)	0.91 (0.7, 1.12)
30-34 years	6589597.13 (2669096.64, 13569423.34)	1709.71 (692.51, 3520.66)	12408567.21 (4783468.41, 25934087.55)	2052.77 (791.34, 4290.31)	0.59 (0.41, 0.76)
35-39 years	7693315.89 (3261568.03, 15211674.84)	2184.08 (925.94, 4318.5)	13309850.27 (5389565.88, 26758106.28)	2373.09 (960.94, 4770.85)	0.26 (0.14, 0.38)
40-44 years	5067051.52 (2317183.09, 9769300.28)	1768.72 (808.84, 3410.1)	9220680.83 (4005545.4, 18023513.37)	1843.21 (800.71, 3602.9)	0.06 (-0.03, 0.14)
45-49 years	490410.65 (149828.93, 1104059.92)	211.21 (64.53, 475.49)	1019970.64 (290558.19, 2415134.78)	215.41 (61.36, 510.06)	-0.27 (-0.43, -0.11)
**YLDs**					
**Age**					
15-19 years	5260.62 (1036.89, 18423.51)	1.01 (0.2, 3.55)	7097.17 (1276.13, 25833.67)	1.14 (0.2, 4.14)	0.11 (0, 0.22)
20-24 years	31974.53 (8371.11, 85934.76)	6.5 (1.7, 17.46)	49823.75 (12556.24, 132617.94)	8.34 (2.1, 22.21)	0.76 (0.6, 0.92)
25-29 years	35814.04 (9968.54, 87214.14)	8.09 (2.25, 19.7)	61530.89 (16354.86, 153022.61)	10.46 (2.78, 26.01)	0.9 (0.7, 1.09)
30-34 years	36585.68 (10050.06, 99454.42)	9.49 (2.61, 25.8)	69320.19 (18177.22, 187144.6)	11.47 (3.01, 30.96)	0.61 (0.43, 0.78)
35-39 years	41733.65 (13045.55, 109036.49)	11.85 (3.7, 30.95)	72974.64 (22358.65, 194032.52)	13.01 (3.99, 34.6)	0.29 (0.17, 0.42)
40-44 years	27785.35 (8758.42, 69286.71)	9.7 (3.06, 24.19)	51164.94 (16139.02, 129124.37)	10.23 (3.23, 25.81)	0.09 (0, 0.19)
45-49 years	2715.11 (645.34, 7899.83)	1.17 (0.28, 3.4)	5702.02 (1275.79, 16913.03)	1.2 (0.27, 3.57)	-0.22 (-0.38, -0.06)

Notes: ASR: age-standardized rates; YLDs: years lived with disability; EAPCs: estimated annual percentage changes.

Table S26. Prevalence and YLDs of female infertility and its temporal trends from 1990, 2021 by gender, and age in the worldwide.

	1990		2021		EAPC 95%CI
	Number	ASR, per 100,000 persons	Number	ASR, per 100,000 persons	
**Prevalence**					
**Age**					
15-19 years	791658.25 (97993.25, 2338496.68)	152.41 (18.87, 450.21)	1014988.54 (69015.93, 3271028.2)	162.66 (11.06, 524.22)	-0.17 (-0.53, 0.18)
20-24 years	7957251.01 (2863602.4, 16004113.11)	1617.04 (581.93, 3252.29)	13082607.56 (4616283.93, 26411135.7)	2190.81 (773.04, 4422.8)	1.04 (0.76, 1.32)
25-29 years	10618577.77 (3352786.48, 26006674.29)	2399.03 (757.49, 5875.63)	19170379.45 (6129767.61, 46190158.91)	3258.37 (1041.87, 7850.89)	1.2 (0.92, 1.48)
30-34 years	13279983.89 (3429389.11, 30328858.3)	3445.57 (889.77, 7868.99)	26866482.92 (6932323.82, 64075581.85)	4444.56 (1146.82, 10600.11)	0.86 (0.65, 1.06)
35-39 years	17089875.28 (5157052.04, 38349601.04)	4851.71 (1464.05, 10887.21)	30599403.42 (8491887.74, 69339499.29)	5455.74 (1514.07, 12362.93)	0.46 (0.31, 0.62)
40-44 years	9834566.32 (2599135.64, 22294902.33)	3432.88 (907.26, 7782.31)	19070838.95 (4763495.84, 43742361.28)	3812.26 (952.22, 8744.09)	0.32 (0.24, 0.4)
45-49 years	118087.28 (23532.29, 620857.42)	50.86 (10.13, 267.39)	284758.44 (48244.52, 1686994.78)	60.14 (10.19, 356.28)	-0.16 (-0.55, 0.23)
**YLDs**					
**Age**					
15-19 years	5074.38 (468.79, 17951.64)	0.98 (0.09, 3.46)	6162.18 (316.19, 23164.36)	0.99 (0.05, 3.71)	-0.39 (-0.69, -0.09)
20-24 years	48663.08 (11823.48, 133351.57)	9.89 (2.4, 27.1)	78481.71 (19500.61, 207846.34)	13.14 (3.27, 34.81)	0.92 (0.67, 1.17)
25-29 years	60406.22 (11757.15, 172481.36)	13.65 (2.66, 38.97)	109615.3 (22334.45, 309836.91)	18.63 (3.8, 52.66)	1.19 (0.93, 1.46)
30-34 years	70119.21 (13340.58, 189815.29)	18.19 (3.46, 49.25)	142815.18 (26432.98, 384260.94)	23.63 (4.37, 63.57)	0.88 (0.67, 1.09)
35-39 years	89176.03 (20855.05, 261596.73)	25.32 (5.92, 74.27)	160994.61 (35789.9, 465101.96)	28.7 (6.38, 82.93)	0.49 (0.34, 0.65)
40-44 years	51867.62 (10292.63, 164619.55)	18.11 (3.59, 57.46)	101534.89 (19710.24, 317436.65)	20.3 (3.94, 63.46)	0.36 (0.28, 0.44)
45-49 years	630.25 (95.21, 3355.1)	0.27 (0.04, 1.44)	1529.64 (208.76, 8643.82)	0.32 (0.04, 1.83)	-0.14 (-0.52, 0.25)

Notes: ASR: age-standardized rates; YLDs: years lived with disability; EAPCs: estimated annual percentage changes.

Table S27. Prevalence and YLDs of depressive disorders and its temporal trends from 1990, 2021 by gender, and age in the worldwide.

	1990		2021		EAPC 95%CI
	Number	ASR, per 100,000 persons	Number	ASR, per 100,000 persons	
**Prevalence**					
**Gender**					
Female	107675384.54 (97003874.93, 121555309.67)	4358.15 (3938.97, 4883.83)	201266251.45 (179872069.06, 228454650.51)	4822.12 (4316.38, 5483.35)	-0.05 (-0.17, 0.06)
Male	68651829.2 (61818421.07, 77334211.75)	2833 (2551.62, 3163.66)	131144081.82 (117402031.62, 147879574.78)	3186.43 (2853.4, 3604.27)	0 (-0.12, 0.11)
**Age**					
<5 years	6406.46 (3323.23, 10283.6)	1.03 (0.54, 1.66)	9201.92 (4772.7, 14857.09)	1.4 (0.73, 2.26)	0.48 (0.28, 0.67)
5-9 years	564579.37 (354638.37, 856220.09)	96.75 (60.77, 146.73)	892933.3 (533562.47, 1380695.39)	129.97 (77.66, 200.96)	0.64 (0.47, 0.81)
10-14 years	4947885.11 (3353922.84, 6849777.48)	923.66 (626.1, 1278.7)	8432585.23 (5583087.39, 11830958.04)	1264.95 (837.5, 1774.73)	0.66 (0.49, 0.83)
15-19 years	13814766.07 (10482518.49, 17853459.64)	2659.63 (2018.11, 3437.17)	21090635.89 (15607278.44, 27678620.32)	3380.02 (2501.25, 4435.82)	0.27 (0.1, 0.45)
20-24 years	19714032.13 (14989838.55, 26466621.83)	4006.2 (3046.17, 5378.43)	27965580.4 (21041568.38, 38573570.8)	4683.11 (3523.62, 6459.52)	-0.08 (-0.28, 0.12)
25-29 years	19659113.34 (15964558.08, 24101141.73)	4441.54 (3606.83, 5445.11)	29282843.33 (23648832.46, 36802143.23)	4977.17 (4019.56, 6255.22)	-0.14 (-0.32, 0.03)
30-34 years	18467293.85 (14667260.7, 22645600.68)	4791.44 (3805.5, 5875.53)	31383926.22 (24599622.6, 39151547.51)	5191.89 (4069.55, 6476.9)	-0.18 (-0.33, -0.04)
35-39 years	18222578.72 (15082460.1, 21552913.12)	5173.28 (4281.82, 6118.74)	32113543.1 (26063428.1, 38451400.7)	5725.71 (4647, 6855.72)	-0.12 (-0.27, 0.02)
40-44 years	15848848.52 (12679730.78, 19236079.56)	5532.24 (4426.02, 6714.59)	30617205.26 (24028578.05, 37684899.09)	6120.37 (4803.31, 7533.2)	-0.13 (-0.27, 0.01)
45-49 years	13305266 (11328898.82, 15701451.25)	5730.2 (4879.03, 6762.17)	29476915.98 (24959704.75, 35040854.72)	6225.26 (5271.27, 7400.32)	-0.12 (-0.23, -0.01)
50-54 years	12361386.96 (10755255.13, 14295789.5)	5815.17 (5059.59, 6725.17)	28093554.44 (24331838.31, 32602775.51)	6314.25 (5468.78, 7327.73)	-0.06 (-0.15, 0.03)
55-59 years	10892269.88 (9172198.69, 12793292.39)	5881.36 (4952.59, 6907.83)	25419499.26 (21225536.58, 29961379.06)	6423.48 (5363.67, 7571.21)	-0.02 (-0.11, 0.07)
60-64 years	9541450.59 (7830139.84, 11557517.35)	5940.79 (4875.27, 7196.05)	20657380.06 (16971512.43, 24852103.14)	6454.47 (5302.8, 7765.12)	0.01 (-0.07, 0.1)
65-69 years	7314216.63 (6171202.05, 8773923.51)	5917.2 (4992.5, 7098.1)	17593575.78 (14873237.69, 21109041.57)	6378.13 (5391.94, 7652.58)	0.05 (-0.02, 0.13)
70-74 years	4951237.62 (4145860.3, 5795623.03)	5848.3 (4897, 6845.67)	12651141.98 (10529029.09, 14972456.06)	6146.13 (5115.17, 7273.86)	0.07 (0.02, 0.12)
75-79 years	3527250.63 (2871957.73, 4267549.97)	5730.19 (4665.64, 6932.85)	7862706.94 (6358776.41, 9597057.08)	5961.82 (4821.48, 7276.87)	0.1 (0.08, 0.11)
80-84 years	2002758.19 (1621903.56, 2438235.42)	5661.36 (4584.77, 6892.36)	5020820.71 (4028483.91, 6178369.14)	5732.64 (4599.62, 7054.3)	0.07 (0.04, 0.09)
85-89 years	870383.21 (733349.18, 1055870.06)	5759.9 (4853.06, 6987.39)	2551683.28 (2150557.53, 3062974.14)	5580.89 (4703.57, 6699.16)	-0.07 (-0.11, -0.03)

90-94 years	253256.37 (203516.83, 311037.91)	5910.04 (4749.31, 7258.44)	992246.33 (787532.06, 1213128.33)	5546.57 (4402.23, 6781.28)	-0.16 (-0.21, -0.11)
95 plus years	62234.1 (44620.07, 82016.79)	6112.85 (4382.74, 8055.97)	302353.85 (215331.58, 397907.28)	5547.46 (3950.81, 7300.64)	-0.23 (-0.29, -0.18)
**YLDs**					
**Gender**					
Female	18209724.17 (12739285.93, 24659287.89)	731.7 (512.35, 996.7)	34119422.2 (23794824.67, 46219577.35)	821.17 (570.96, 1110.43)	-0.07 (-0.21, 0.07)
Male	11464941.63 (8009456.4, 15605994.78)	468.06 (326.2, 640.14)	22210938.94 (15498042.12, 30314533.58)	540.51 (377.31, 735.48)	0 (-0.13, 0.14)
**Age**					
<5 years	1164.81 (503.74, 2192.27)	0.19 (0.08, 0.35)	1751.68 (745.78, 3416.95)	0.27 (0.11, 0.52)	0.54 (0.31, 0.77)
5-9 years	104606.11 (51232.18, 184679.04)	17.93 (8.78, 31.65)	171685.87 (79510.19, 306877.76)	24.99 (11.57, 44.67)	0.71 (0.51, 0.9)
10-14 years	926879.73 (553471.23, 1402112.01)	173.03 (103.32, 261.74)	1629553.55 (958147.44, 2495215.31)	244.44 (143.73, 374.3)	0.72 (0.53, 0.91)
15-19 years	2550150 (1624371.18, 3808419.38)	490.96 (312.73, 733.2)	3979118.44 (2494527.69, 6048541.94)	637.7 (399.78, 969.35)	0.28 (0.08, 0.48)
20-24 years	3548059.67 (2262643.7, 5269856.6)	721.02 (459.8, 1070.92)	5109522.82 (3217184.32, 7651378.41)	855.64 (538.75, 1281.3)	-0.12 (-0.34, 0.1)
25-29 years	3401918.75 (2252493.13, 4968966.03)	768.59 (508.9, 1122.63)	5157309.36 (3365033.11, 7577529.6)	876.58 (571.95, 1287.94)	-0.18 (-0.39, 0.02)
30-34 years	3115511.98 (2017169.59, 4621755.82)	808.34 (523.37, 1199.14)	5387844.66 (3420056.67, 8011824.73)	891.32 (565.78, 1325.41)	-0.21 (-0.38, -0.04)
35-39 years	3022602.27 (1988462.75, 4224774.83)	858.1 (564.51, 1199.39)	5459259.65 (3559902.62, 7735847.63)	973.36 (634.72, 1379.27)	-0.12 (-0.3, 0.05)
40-44 years	2599935.24 (1674074.5, 3727685.59)	907.54 (584.36, 1301.2)	5147257.11 (3254703.67, 7448012.07)	1028.94 (650.61, 1488.86)	-0.13 (-0.29, 0.04)
45-49 years	2164257.79 (1448448.44, 2989110.7)	932.08 (623.81, 1287.33)	4873116.79 (3232648.97, 6757603.52)	1029.16 (682.71, 1427.15)	-0.13 (-0.27, 0)
50-54 years	1993133.83 (1347813.01, 2720214.3)	937.63 (634.05, 1279.67)	4585046.39 (3083192.01, 6302889.79)	1030.53 (692.97, 1416.62)	-0.08 (-0.18, 0.03)
55-59 years	1740451.19 (1163841.63, 2410020.34)	939.77 (628.42, 1301.31)	4108532.81 (2727727.02, 5639378.52)	1038.22 (689.29, 1425.06)	-0.03 (-0.13, 0.08)
60-64 years	1515974.38 (1028510.61, 2103818.33)	943.89 (640.38, 1309.9)	3325196.81 (2243108.92, 4607166.49)	1038.97 (700.87, 1439.52)	0 (-0.1, 0.1)
65-69 years	1156354.04 (787459.38, 1572544.89)	935.49 (637.05, 1272.19)	2792754.61 (1912536.14, 3786190.3)	1012.45 (693.34, 1372.59)	0.04 (-0.03, 0.12)
70-74 years	780187.19 (526452.02, 1055804.27)	921.54 (621.83, 1247.09)	2000821.35 (1363384.31, 2710353.55)	972.03 (662.35, 1316.73)	0.07 (0.03, 0.11)
75-79 years	556445.25 (371800.22, 777965)	903.97 (604.01, 1263.84)	1236578.44 (841610.46, 1738534.76)	937.62 (638.14, 1318.23)	0.1 (0.08, 0.11)
80-84 years	314605.98 (214096.01, 445618.53)	889.32 (605.2, 1259.67)	783402.05 (531635.97, 1113253.64)	894.47 (607.01, 1271.08)	0.06 (0.02, 0.1)
85-89 years	134832.81 (95036.54, 186004.8)	892.28 (628.92, 1230.92)	389849.47 (275344.93, 540314.26)	852.66 (602.22, 1181.74)	-0.1 (-0.15, -0.05)
90-94 years	38413.41 (26229.57, 54787.44)	896.42 (612.1, 1278.53)	147970.61 (101545.88, 211544.37)	827.14 (567.63, 1182.51)	-0.21 (-0.26, -0.15)
95 plus years	9181.38 (5600.39, 13816.31)	901.83 (550.09, 1357.09)	43788.66 (26321.91, 65968.91)	803.42 (482.94, 1210.37)	-0.28 (-0.35, -0.22)

Notes: ASR: age-standardized rates; YLDs: years lived with disability; EAPCs: estimated annual percentage changes.

Table S28. Prevalence and YLDs of anxiety disorders and its temporal trends from 1990, 2021 by gender, and age in the worldwide.

	1990		2021		EAPC 95%CI
	Number	ASR, per 100,000 persons	Number	ASR, per 100,000 persons	
**Prevalence**					
**Gender**					
Female	120227843.67 (102878960.1, 141298589.83)	4674.19 (4018.03, 5451.66)	224809615.34 (192230866.58, 263052152.62)	5535.61 (4730.42, 6495.3)	0.11 (-0.03, 0.26)
Male	72708290.14 (61996056.54, 85847345.82)	2807.4 (2410.04, 3284.79)	134403654.88 (114699149.08, 157908850.13)	3308.52 (2830.39, 3887.81)	0.17 (0.04, 0.31)
**Age**					
<5 years	532533.64 (361902.34, 767813.95)	85.9 (58.38, 123.85)	741923.25 (492589.26, 1080003.85)	112.72 (74.84, 164.09)	0.37 (0.18, 0.57)
5-9 years	7556842.05 (5222201.32, 10850901.2)	1295.02 (894.93, 1859.53)	11127687.13 (7537912.72, 16039903.78)	1619.62 (1097.14, 2334.59)	0.23 (0.07, 0.4)
10-14 years	18067423.57 (12656243.51, 24635547.77)	3372.79 (2362.64, 4598.91)	27076414.76 (18730806.51, 37517455.97)	4061.65 (2809.75, 5627.88)	0.11 (-0.05, 0.27)
15-19 years	23053160.15 (17761412.8, 29621083.05)	4438.22 (3419.45, 5702.68)	33044677.47 (25155221.48, 42792690.44)	5295.79 (4031.42, 6858.02)	0.11 (-0.05, 0.27)
20-24 years	22633064.49 (16836005.81, 29782001.97)	4599.39 (3421.34, 6052.17)	33826064.31 (24956761.99, 44703689.4)	5664.51 (4179.26, 7486.07)	0.18 (0.01, 0.36)
25-29 years	20650115.69 (15408047.95, 27432042.86)	4665.43 (3481.1, 6197.66)	33891271.93 (25079228.49, 45013327.94)	5760.46 (4262.69, 7650.86)	0.22 (0.05, 0.4)
30-34 years	18200540.25 (14055481.95, 23335715.73)	4722.23 (3646.77, 6054.58)	34509154.85 (26618309.6, 44568217.73)	5708.9 (4403.5, 7372.98)	0.21 (0.06, 0.37)
35-39 years	16608618.86 (13079472, 20756600.99)	4715.08 (3713.18, 5892.67)	32319010.74 (25202432.33, 40486180.86)	5762.34 (4493.48, 7218.51)	0.24 (0.09, 0.38)
40-44 years	13758717.52 (10254449.22, 17677843.69)	4802.65 (3579.44, 6170.67)	28791626.64 (21310408.58, 36521138.5)	5755.44 (4259.94, 7300.57)	0.19 (0.04, 0.34)
45-49 years	11090468.82 (8142172.74, 14389419.96)	4776.35 (3506.6, 6197.11)	26176334.28 (19104779.68, 33600351.54)	5528.21 (4034.76, 7096.1)	0.12 (-0.01, 0.24)
50-54 years	9960583.14 (7756725.35, 12602805.55)	4685.76 (3649, 5928.74)	23849908.74 (18610697.03, 30200912.79)	5360.46 (4182.9, 6787.9)	0.1 (-0.02, 0.22)
55-59 years	8592950.14 (6838995.59, 10779747.49)	4639.82 (3692.76, 5820.6)	20786010.77 (16743649.07, 25919321.54)	5252.6 (4231.1, 6549.78)	0.08 (-0.04, 0.2)
60-64 years	7427371.56 (5712238.41, 9863842.87)	4624.5 (3556.61, 6141.52)	16488480.04 (12689396.29, 21796379.28)	5151.88 (3964.84, 6810.35)	0 (-0.12, 0.13)
65-69 years	5763147.86 (4303234.52, 7826248.69)	4662.38 (3481.31, 6331.43)	13714726.33 (10262694.86, 18579786.63)	4971.95 (3720.5, 6735.66)	-0.09 (-0.2, 0.02)
70-74 years	3864882.44 (2963777.13, 5158881.57)	4565.12 (3500.75, 6093.56)	9952077.04 (7605506.18, 13304902.36)	4834.88 (3694.88, 6463.74)	-0.13 (-0.23, -0.04)
75-79 years	2800753.89 (2166514.42, 3598697.96)	4549.96 (3519.61, 5846.26)	6192709.02 (4813401.93, 7973794.45)	4695.56 (3649.71, 6046.05)	-0.15 (-0.23, -0.06)
80-84 years	1566543.15 (1228639.4, 1985400.92)	4428.27 (3473.09, 5612.29)	3986238.73 (3115792.18, 5033856.62)	4551.38 (3557.53, 5747.52)	-0.13 (-0.2, -0.06)
85-89 years	622445.53 (466899.13, 798199.21)	4119.14 (3089.78, 5282.21)	1921456.12 (1451235.98, 2446391.08)	4202.5 (3174.06, 5350.6)	-0.1 (-0.17, -0.03)

90-94 years	155220.74 (114146.32, 206551.12)	3622.26 (2663.74, 4820.12)	655505.31 (484189.79, 876849.62)	3664.22 (2706.58, 4901.51)	-0.08 (-0.13, -0.02)
95 plus years	30750.32 (21006.01, 42763.89)	3020.4 (2063.28, 4200.42)	161992.78 (111207.05, 225102.06)	2972.18 (2040.38, 4130.08)	-0.14 (-0.18, -0.1)
**YLDs**					
**Gender**					
Female	14236490.86 (9856231.79, 19377107.11)	550.7 (382.99, 745.49)	26395987.83 (18390433.5, 35710763.19)	652.17 (453.44, 887.15)	0.11 (-0.03, 0.26)
Male	8760241.05 (6016981.1, 12171230.87)	335.85 (231.23, 460.99)	16113657.45 (11067291.41, 22164952.44)	396.91 (272.28, 547.66)	0.18 (0.05, 0.32)
**Age**					
<5 years	66350.3 (38912.19, 101085.55)	10.7 (6.28, 16.31)	92782.86 (52877.16, 145776.48)	14.1 (8.03, 22.15)	0.39 (0.2, 0.59)
5-9 years	942647.67 (562012.5, 1416463.21)	161.54 (96.31, 242.74)	1392656.86 (810715.71, 2131729.82)	202.7 (118, 310.27)	0.24 (0.07, 0.41)
10-14 years	2238634.8 (1350319.03, 3327223.1)	417.9 (252.07, 621.12)	3358104.61 (2013417.67, 5072153.5)	503.74 (302.03, 760.86)	0.12 (-0.04, 0.28)
15-19 years	2831155.67 (1787407.46, 4215876.45)	545.06 (344.11, 811.65)	4054128.15 (2545177.81, 6004785.8)	649.72 (407.89, 962.34)	0.11 (-0.04, 0.27)
20-24 years	2752731.15 (1738657.47, 4102815.31)	559.4 (353.32, 833.76)	4111483.66 (2580955.31, 6110619.81)	688.51 (432.21, 1023.28)	0.19 (0.02, 0.36)
25-29 years	2489434.27 (1562417.85, 3701749.47)	562.43 (352.99, 836.33)	4084696.59 (2535557.61, 6043161.94)	694.27 (430.97, 1027.15)	0.23 (0.06, 0.4)
30-34 years	2178950.04 (1402555.82, 3152138.78)	565.34 (363.9, 817.84)	4133465.66 (2671030.7, 5951479.24)	683.81 (441.87, 984.56)	0.22 (0.07, 0.37)
35-39 years	1975554.26 (1297494.21, 2802441.33)	560.85 (368.35, 795.6)	3841611.19 (2521935.21, 5437237.09)	684.94 (449.65, 969.44)	0.24 (0.1, 0.38)
40-44 years	1624225.06 (991050.66, 2389154.76)	566.96 (345.94, 833.96)	3394027.83 (2066561.33, 4956305.67)	678.47 (413.11, 990.76)	0.19 (0.05, 0.34)
45-49 years	1299315.88 (809782.99, 1912509.41)	559.58 (348.75, 823.66)	3064476.02 (1907197.37, 4471867.63)	647.19 (402.78, 944.42)	0.12 (0, 0.25)
50-54 years	1157361.04 (751158.08, 1647252.18)	544.46 (353.37, 774.92)	2769384.59 (1803914.34, 3913932.82)	622.44 (405.44, 879.69)	0.11 (-0.01, 0.22)
55-59 years	988321.31 (667101.54, 1411064.64)	533.65 (360.21, 761.91)	2387418.19 (1612834.1, 3400518.14)	603.3 (407.56, 859.31)	0.08 (-0.03, 0.2)
60-64 years	842752.32 (551853.54, 1240341.34)	524.72 (343.6, 772.27)	1866300.45 (1226815.94, 2738307.6)	583.13 (383.32, 855.59)	0 (-0.12, 0.12)
65-69 years	642650.79 (414024.69, 953908.65)	519.9 (334.95, 771.71)	1525959.16 (991153.04, 2236585.41)	553.2 (359.32, 810.82)	-0.09 (-0.2, 0.01)
70-74 years	422209.42 (280273.35, 609405.43)	498.71 (331.05, 719.82)	1084363.42 (723663.21, 1556564.69)	526.8 (351.57, 756.2)	-0.14 (-0.23, -0.05)
75-79 years	299429.04 (206646.66, 428714.85)	486.44 (335.71, 696.47)	659446.22 (452469.76, 929705.61)	500.02 (343.08, 704.94)	-0.15 (-0.24, -0.07)
80-84 years	163512.03 (112832.84, 230019.94)	462.21 (318.95, 650.22)	414769.2 (281466.87, 584194.82)	473.57 (321.37, 667.02)	-0.13 (-0.2, -0.07)
85-89 years	63194.85 (41213.15, 89688.55)	418.2 (272.73, 593.53)	194484.04 (125461.57, 275323.43)	425.36 (274.4, 602.17)	-0.11 (-0.17, -0.04)
90-94 years	15346.11 (9823.99, 21531.62)	358.12 (229.25, 502.47)	64575.16 (41583.03, 90619.23)	360.97 (232.45, 506.55)	-0.08 (-0.13, -0.03)
95 plus years	2955.9 (1861.93, 4309.93)	290.34 (182.89, 423.34)	15511.41 (9839.38, 22324.82)	284.6 (180.53, 409.61)	-0.15 (-0.19, -0.11)

Notes: ASR: age-standardized rates; YLDs: years lived with disability; EAPCs: estimated annual percentage changes.

Table S29. Prevalence and YLDs of opioid use disorders and its temporal trends from 1990, 2021 by gender, and age in the worldwide.

	1990		2021		EAPC 95%CI
	Number	ASR, per 100,000 persons	Number	ASR, per 100,000 persons	
**Prevalence**					
**Gender**					
Female	4008774.15 (3293734.29, 4771654.68)	153.99 (128.53, 181.17)	7988655.62 (6896383, 9215842.89)	196.11 (168.4, 226.88)	0.56 (0.27, 0.85)
Male	4112039.37 (3496117.73, 4815001.11)	154.58 (132.54, 180.63)	8176220.31 (7238339.72, 9254557.11)	200.23 (176.76, 226.9)	0.46 (0.29, 0.63)
**Age**					
<5 years	362227.65 (250991.3, 497586.43)	69.74 (48.32, 95.8)	539794.28 (402222.38, 704377.77)	86.51 (64.46, 112.88)	0.24 (-0.04, 0.51)
5-9 years	1575248.18 (1151673.02, 2123600.47)	320.12 (234.04, 431.55)	2427331.46 (1895535.22, 3118136.86)	406.48 (317.43, 522.16)	0.38 (0.16, 0.6)
10-14 years	1790047.17 (1387905.46, 2262293.92)	404.42 (313.57, 511.11)	3081386.2 (2537623.54, 3759825.03)	523.74 (431.32, 639.05)	0.54 (0.36, 0.73)
15-19 years	1296283.43 (1012834.51, 1661734.05)	336.33 (262.79, 431.15)	2697915.67 (2246890.9, 3291168.66)	446.32 (371.71, 544.46)	0.63 (0.46, 0.79)
20-24 years	939812.87 (747326.76, 1217532.64)	266.81 (212.16, 345.65)	2031387.27 (1682416.94, 2487428.49)	362.19 (299.97, 443.5)	0.66 (0.45, 0.87)
25-29 years	610769.07 (462550.3, 774905.25)	213.2 (161.46, 270.49)	1484656.53 (1223164.91, 1765534.15)	296.78 (244.51, 352.93)	0.6 (0.39, 0.82)
30-34 years	419513.73 (322196.88, 538215.99)	180.67 (138.76, 231.79)	1167532.07 (970653.38, 1403748.11)	246.57 (204.99, 296.46)	0.68 (0.52, 0.85)
35-39 years	368712.71 (274858.55, 487237.05)	173.45 (129.3, 229.21)	981724.71 (778053.47, 1208817.79)	220.65 (174.87, 271.69)	0.72 (0.56, 0.88)
40-44 years	268538.7 (199532.9, 345813.67)	145 (107.74, 186.72)	710363.11 (563876.52, 874885.7)	179.51 (142.49, 221.08)	0.66 (0.45, 0.87)
45-49 years	192787.93 (142247.13, 263825.08)	120.04 (88.57, 164.27)	428424.1 (334255.21, 551159.77)	133.86 (104.44, 172.21)	0.27 (0.1, 0.45)
50-54 years	123824.59 (94206.27, 159673.92)	100.17 (76.21, 129.18)	262215.53 (207105.86, 335624.02)	95.06 (75.08, 121.67)	-0.3 (-0.4, -0.21)
55-59 years	78920.3 (57725.77, 106288.56)	93.22 (68.18, 125.55)	153586.74 (116864.57, 201858.47)	74.61 (56.77, 98.07)	-0.91 (-0.98, -0.83)
60-64 years	51810.25 (39270.94, 68126.39)	84.17 (63.8, 110.67)	85583.29 (66487.45, 108339.96)	64.89 (50.41, 82.15)	-1.12 (-1.22, -1.03)
65-69 years	27074.83 (19300.15, 36121.15)	76.53 (54.56, 102.11)	57139.81 (43159.98, 72878.18)	65.24 (49.28, 83.21)	-0.78 (-0.91, -0.64)
70-74 years	11128.29 (8660.44, 14004.26)	73.64 (57.31, 92.68)	33773.5 (27746.61, 40813.19)	73.87 (60.69, 89.26)	-0.09 (-0.21, 0.03)
75-79 years	3256.09 (2539.79, 4137.54)	75.98 (59.27, 96.55)	16387.64 (13722.4, 19439.27)	91.61 (76.71, 108.66)	0.57 (0.45, 0.7)

80-84 years	857.72 (634.68, 1104.64)	84.25 (62.34, 108.5)	5674.03 (4555.49, 6895.57)	104.1 (83.58, 126.52)	0.51 (0.38, 0.64)
85-89 years					
90-94 years					
95 plus years	1646211.24 (1097683.12, 2178466.76)	62.98 (41.91, 82.52)	3226975.06 (2221519.52, 4183467.7)	79.37 (54.78, 103.57)	0.53 (0.25, 0.82)
**YLDs**	1728018.13 (1158454.86, 2289908.92)	64.69 (43.62, 84.19)	3409492.25 (2377710.31, 4420570.07)	83.55 (58.24, 108.57)	0.46 (0.29, 0.62)
**Gender**					
Female	155506.39 (94083.7, 228877.23)	29.94 (18.11, 44.06)	230302.22 (144879.14, 332712.36)	36.91 (23.22, 53.32)	0.22 (-0.05, 0.49)
Male	670226.82 (412567.65, 980704.93)	136.2 (83.84, 199.29)	1022516.99 (655011.34, 1430910.99)	171.23 (109.69, 239.62)	0.36 (0.15, 0.58)
**Age**	754315.38 (480914.08, 1039484.46)	170.42 (108.65, 234.85)	1287738.07 (877390.47, 1743357.34)	218.88 (149.13, 296.32)	0.52 (0.34, 0.71)
<5 years	541523.33 (360514.46, 756799.21)	140.5 (93.54, 196.36)	1120136.95 (757363.18, 1519870.77)	185.31 (125.29, 251.43)	0.61 (0.45, 0.78)
5-9 years	389807.53 (258980.28, 552425.09)	110.66 (73.52, 156.83)	835588.69 (565861.05, 1147002.61)	148.98 (100.89, 204.51)	0.64 (0.44, 0.85)
10-14 years	251009.67 (165951.59, 350123.05)	87.62 (57.93, 122.21)	604763.94 (416100.65, 810364.3)	120.89 (83.18, 161.99)	0.58 (0.37, 0.79)
15-19 years	170834.24 (115946.77, 242206.21)	73.57 (49.93, 104.31)	471383.61 (321280.86, 625159.03)	99.55 (67.85, 132.03)	0.66 (0.5, 0.82)
20-24 years	148241.3 (91974.32, 208879.56)	69.74 (43.27, 98.26)	391589.99 (260737.13, 521693.36)	88.01 (58.6, 117.25)	0.7 (0.54, 0.85)
25-29 years	106595.59 (65970.92, 148081.11)	57.56 (35.62, 79.96)	279464.26 (186638.22, 370226.48)	70.62 (47.16, 93.56)	0.64 (0.44, 0.84)
30-34 years	75399.91 (47837.58, 108626.85)	46.95 (29.79, 67.63)	165843.11 (108675.52, 231359.88)	51.82 (33.96, 72.29)	0.25 (0.08, 0.42)
35-39 years	47409.57 (30792.1, 66573.26)	38.35 (24.91, 53.86)	99589.35 (66281.77, 138307.22)	36.1 (24.03, 50.14)	-0.32 (-0.42, -0.23)
40-44 years	29539.83 (18654.45, 44111.71)	34.89 (22.03, 52.1)	57165.42 (37236, 82544.32)	27.77 (18.09, 40.1)	-0.93 (-1, -0.85)
45-49 years	18926.78 (12485.52, 27214.82)	30.75 (20.28, 44.21)	31118.52 (20892.53, 43542.21)	23.6 (15.84, 33.02)	-1.14 (-1.23, -1.04)
50-54 years	9668.77 (6198.22, 13915.72)	27.33 (17.52, 39.34)	20297.14 (13307.32, 28630.19)	23.17 (15.19, 32.69)	-0.8 (-0.93, -0.66)
55-59 years	3851.06 (2533.85, 5359.55)	25.49 (16.77, 35.47)	11637.78 (8016.43, 15541.96)	25.45 (17.53, 33.99)	-0.1 (-0.22, 0.02)
60-64 years	1094.7 (739.72, 1486.7)	25.55 (17.26, 34.69)	5489.5 (3846.12, 7222.85)	30.69 (21.5, 40.38)	0.56 (0.44, 0.68)
65-69 years	278.5 (186.47, 384.75)	27.36 (18.32, 37.79)	1841.77 (1291.97, 2445.07)	33.79 (23.7, 44.86)	0.51 (0.38, 0.64)
70-74 years	4008774.15 (3293734.29, 4771654.68)	153.99 (128.53, 181.17)	7988655.62 (6896383, 9215842.89)	196.11 (168.4, 226.88)	0.56 (0.27, 0.85)
75-79 years	4112039.37 (3496117.73, 4815001.11)	154.58 (132.54, 180.63)	8176220.31 (7238339.72, 9254557.11)	200.23 (176.76, 226.9)	0.46 (0.29, 0.63)
80-84 years					

85-89 years	362227.65 (250991.3, 497586.43)	69.74 (48.32, 95.8)	539794.28 (402222.38, 704377.77)	86.51 (64.46, 112.88)	0.24 (-0.04, 0.51)
90-94 years	1575248.18 (1151673.02, 2123600.47)	320.12 (234.04, 431.55)	2427331.46 (1895535.22, 3118136.86)	406.48 (317.43, 522.16)	0.38 (0.16, 0.6)
95 plus years	1790047.17 (1387905.46, 2262293.92)	404.42 (313.57, 511.11)	3081386.2 (2537623.54, 3759825.03)	523.74 (431.32, 639.05)	0.54 (0.36, 0.73)

Notes: ASR: age-standardized rates; YLDs: years lived with disability; EAPCs: estimated annual percentage changes.

Table S30. Prevalence and YLDs of varicella and herpes zoster and its temporal trends from 1990, 2021 by gender, and age in the worldwide.

	1990		2021		EAPC 95%CI
	Number	ASR, per 100,000 persons	Number	ASR, per 100,000 persons	
**Prevalence**					
**Gender**					
Female	1755922.63 (1514760.77, 2033827.82)	69.17 (58.78, 80.43)	2792712.95 (2321664.07, 3298954.96)	69.83 (59.26, 81.31)	0.07 (0.04, 0.09)
Male	1615285.52 (1402126.77, 1859332.86)	63.95 (54.41, 73.78)	2489384.19 (2081824.64, 2898608.03)	64.35 (54.64, 74.35)	0.04 (0.03, 0.05)
**Age**					
<5 years	1031112.14 (906804.4, 1136810.32)	166.33 (146.27, 183.37)	1059671.62 (930775.74, 1173640.07)	161 (141.42, 178.32)	-0.02 (-0.05, 0)
5-9 years	179811.32 (118666.67, 246947.58)	30.81 (20.34, 42.32)	213533.73 (143759.25, 293674.56)	31.08 (20.92, 42.74)	-0.06 (-0.11, -0.01)
10-14 years	132984.75 (89893.59, 189196.41)	24.83 (16.78, 35.32)	165760.11 (111364.08, 235853.05)	24.87 (16.71, 35.38)	-0.04 (-0.08, -0.01)
15-19 years	141152.08 (95214.76, 196152.44)	27.17 (18.33, 37.76)	169661.27 (114418.05, 236039.06)	27.19 (18.34, 37.83)	0.01 (-0.02, 0.04)
20-24 years	155533.85 (90484.03, 239529.28)	31.61 (18.39, 48.68)	189354.58 (110287.54, 292705.31)	31.71 (18.47, 49.02)	0.04 (0.02, 0.07)
25-29 years	158240.85 (97763.8, 242413.46)	35.75 (22.09, 54.77)	211748.19 (129749.58, 324624.83)	35.99 (22.05, 55.18)	0.05 (0.03, 0.07)
30-34 years	150846.89 (106325.14, 209004.3)	39.14 (27.59, 54.23)	238938.9 (167290.14, 330247.14)	39.53 (27.68, 54.63)	0.06 (0.03, 0.08)
35-39 years	151841.18 (106178.04, 211163.71)	43.11 (30.14, 59.95)	242861.87 (168761.49, 337418.16)	43.3 (30.09, 60.16)	0.06 (0.03, 0.1)
40-44 years	135349.92 (82955.4, 204534.82)	47.25 (28.96, 71.4)	237716.61 (146232.98, 359680.87)	47.52 (29.23, 71.9)	0.09 (0.05, 0.12)
45-49 years	132656.5 (86319.1, 195405.04)	57.13 (37.18, 84.16)	274239.71 (177828.84, 406584.8)	57.92 (37.56, 85.87)	0.09 (0.07, 0.12)
50-54 years	156746.66 (110257.1, 211929.22)	73.74 (51.87, 99.7)	338956.55 (236969.73, 456509.37)	76.18 (53.26, 102.6)	0.11 (0.07, 0.14)
55-59 years	171019.74 (113161.24, 243175.44)	92.34 (61.1, 131.3)	375123.63 (247569.36, 535820.58)	94.79 (62.56, 135.4)	0.09 (0.04, 0.14)
60-64 years	177648.89 (107978.02, 262340.61)	110.61 (67.23, 163.34)	362152.36 (217981.69, 536556.99)	113.16 (68.11, 167.65)	0.09 (0.04, 0.14)
65-69 years	162086.91 (105418.94, 231980.31)	131.13 (85.28, 187.67)	367836 (237665.82, 528126.38)	133.35 (86.16, 191.46)	0.1 (0.06, 0.14)
70-74 years	126253.79 (91707.18, 166957.59)	149.13 (108.32, 197.21)	308873.45 (224000.43, 407814.98)	150.06 (108.82, 198.12)	0.07 (0.03, 0.12)
75-79 years	101252.76 (70454.41, 136210.95)	164.49 (114.46, 221.28)	221589.4 (152811.1, 298452.1)	168.02 (115.87, 226.3)	0.05 (0.03, 0.08)

80-84 years	64790.79 (40901.65, 94464.58)	183.15 (115.62, 267.03)	161040.3 (100766.94, 237050.75)	183.87 (115.05, 270.66)	0.05 (0.02, 0.09)
85-89 years	30326.59 (19054.73, 44459.06)	200.69 (126.1, 294.22)	92145.92 (58893.36, 135148.97)	201.54 (128.81, 295.59)	0.1 (0.06, 0.14)
90-94 years	9209.26 (6045.63, 14324.91)	214.91 (141.08, 334.29)	38363 (25029.22, 60928.82)	214.45 (139.91, 340.59)	0.16 (0.1, 0.22)
95 plus years	2343.28 (1188.68, 4208.89)	230.17 (116.76, 413.41)	12529.93 (6260.91, 22671.7)	229.89 (114.87, 415.97)	0.15 (0.07, 0.23)
**YLDs**					
**Gender**					
Female	67475.26 (39542.83, 104927.04)	2.81 (1.62, 4.39)	120739.63 (68396.19, 187130.47)	2.84 (1.62, 4.43)	0.08 (0.05, 0.11)
Male	59069.74 (34614.31, 88969.31)	2.56 (1.49, 3.85)	104146.7 (60183.33, 157322.24)	2.58 (1.5, 3.92)	0.05 (0.04, 0.07)
**Age**					
<5 years	7066.92 (3242.47, 13780.11)	1.14 (0.52, 2.22)	7332.09 (3343.96, 14402.34)	1.11 (0.51, 2.19)	-0.01 (-0.03, 0.01)
5-9 years	4723.74 (2420.18, 8215.65)	0.81 (0.41, 1.41)	5550.81 (2884.54, 9923.82)	0.81 (0.42, 1.44)	-0.04 (-0.07, -0.02)
10-14 years	6261.08 (3196.64, 11046.8)	1.17 (0.6, 2.06)	7676.56 (3991.98, 13676.6)	1.15 (0.6, 2.05)	-0.03 (-0.06, -0.01)
15-19 years	7448.93 (3793.8, 12580.99)	1.43 (0.73, 2.42)	8840.09 (4480.26, 15285.72)	1.42 (0.72, 2.45)	0.01 (-0.02, 0.04)
20-24 years	8397.44 (4076.68, 14700.4)	1.71 (0.83, 2.99)	10132.74 (4842.23, 17629.42)	1.7 (0.81, 2.95)	0.04 (0.01, 0.07)
25-29 years	8630.14 (4162.88, 14901.81)	1.95 (0.94, 3.37)	11494.69 (5701, 19690.1)	1.95 (0.97, 3.35)	0.05 (0.03, 0.07)
30-34 years	8256.84 (4434.6, 13508.33)	2.14 (1.15, 3.5)	13084.75 (6981.45, 21332.31)	2.16 (1.15, 3.53)	0.06 (0.03, 0.08)
35-39 years	8324.59 (4563.25, 13732.1)	2.36 (1.3, 3.9)	13270.63 (7354.01, 21733.89)	2.37 (1.31, 3.88)	0.06 (0.03, 0.1)
40-44 years	7391.95 (3679.02, 12825.79)	2.58 (1.28, 4.48)	12932.7 (6447.65, 22452.55)	2.59 (1.29, 4.49)	0.09 (0.05, 0.13)
45-49 years	7219.02 (3783.73, 12075.39)	3.11 (1.63, 5.2)	14891.4 (7783.73, 25162.22)	3.14 (1.64, 5.31)	0.09 (0.07, 0.12)
50-54 years	8510.35 (4544.72, 13300.72)	4 (2.14, 6.26)	18348.26 (9933.44, 28829.57)	4.12 (2.23, 6.48)	0.11 (0.07, 0.14)
55-59 years	9210.01 (4660.04, 14842.24)	4.97 (2.52, 8.01)	20188.23 (10319.91, 32569.53)	5.1 (2.61, 8.23)	0.09 (0.04, 0.14)
60-64 years	9495.5 (4381.07, 15876.8)	5.91 (2.73, 9.89)	19324.63 (8846.73, 32219.19)	6.04 (2.76, 10.07)	0.09 (0.04, 0.14)
65-69 years	8546.27 (4098.74, 13817.44)	6.91 (3.32, 11.18)	19377.31 (9189.03, 31256.89)	7.02 (3.33, 11.33)	0.1 (0.06, 0.14)
70-74 years	6564.89 (3401.83, 10367.62)	7.75 (4.02, 12.25)	16042.1 (8379.2, 25486.27)	7.79 (4.07, 12.38)	0.07 (0.03, 0.11)
75-79 years	5185.56 (2729.96, 8648.31)	8.42 (4.43, 14.05)	11327.08 (5967.98, 19160.07)	8.59 (4.53, 14.53)	0.05 (0.02, 0.07)
80-84 years	3260.8 (1656.12, 5769.09)	9.22 (4.68, 16.31)	8091.32 (4031.58, 14382.94)	9.24 (4.6, 16.42)	0.05 (0.01, 0.08)

85-89 years	1494.71 (780.02, 2685.34)	9.89 (5.16, 17.77)	4536.77 (2356.52, 7994.72)	9.92 (5.15, 17.49)	0.1 (0.06, 0.14)
90-94 years	445.37 (229.3, 829.59)	10.39 (5.35, 19.36)	1852.24 (955.43, 3472.72)	10.35 (5.34, 19.41)	0.16 (0.09, 0.22)
95 plus years	110.89 (48.79, 229.47)	10.89 (4.79, 22.54)	591.94 (255.18, 1259.18)	10.86 (4.68, 23.1)	0.14 (0.06, 0.22)

Notes: ASR: age-standardized rates; YLDs: years lived with disability; EAPCs: estimated annual percentage changes.

Table S31. Acupuncture demands based on prevalence and YLDs and its temporal trends from 1990, 2021 by gender, and age in China.

	1990		2021		EAPC 95%CI
	Number	ASR, per 100,000 persons	Number	ASR, per 100,000 persons	
**Prevalence**					
**Gender**					
Female	401594681.84 (337649358.34, 475766580.07)	72966.28 (61891.46, 85531.69)	626467861.06 (531660338.91, 734479506.01)	74140.61 (62957.06, 87165.77)	0.07 (0.03, 0.1)
Male	281827875.46 (239661811.51, 330218925.26)	49102.52 (42202.77, 56909.75)	437134624.95 (373675553.64, 509578182.92)	51417.23 (44083.84, 59752.31)	0.14 (0.1, 0.18)
**Age**					
<5 years	1138723.56 (878506, 1453381.54)	1018.48 (785.74, 1299.91)	711327.38 (536770.4, 929265.49)	915.85 (691.11, 1196.45)	-0.29 (-0.36, -0.23)
5-9 years	10351722.23 (6784364.43, 15112290.45)	9927.05 (6506.04, 14492.32)	9443563.38 (6213438.04, 13474325.31)	9860.63 (6487.85, 14069.41)	0 (-0.04, 0.04)
10-14 years	34769177.18 (25479501.54, 46762060.8)	33989.98 (24908.49, 45714.1)	30258378.71 (22233060.47, 40381880.31)	35105.4 (25794.52, 46850.56)	0.12 (0.09, 0.15)
15-19 years	57633915.52 (40218836.33, 77982546.4)	45500.98 (31752.08, 61565.88)	35857950.04 (25036507.66, 48599772.05)	48020.51 (33528.57, 65084.2)	0.16 (0.12, 0.21)
20-24 years	72026005.67 (50770247.85, 100665176.54)	54564.78 (38462.04, 76260.97)	41493542.84 (29318707.01, 58287341.04)	56705.19 (40067.02, 79655.64)	0.08 (0.04, 0.13)
25-29 years	69951268.88 (48503851.81, 100526865.06)	63656.03 (44138.77, 91479.99)	56479206.67 (39397207.57, 80619147.69)	65307.42 (45555.35, 93220.65)	0.06 (0.01, 0.11)
30-34 years	66095631.01 (43971465.21, 96085064.69)	74900.72 (49829.23, 108885.27)	92574486.55 (62049337.81, 134799559.79)	76411.04 (51215.57, 111263.65)	0.03 (-0.03, 0.08)
35-39 years	76913224.32 (51010005.57, 111653997.19)	84206.28 (55846.87, 122241.24)	90155983.37 (59784897.79, 131336394.57)	85082.31 (56420.4, 123945.22)	0.03 (-0.02, 0.07)
40-44 years	57371488.99 (39790838.76, 81790943.31)	85508.69 (59305.81, 121904.39)	79984740.78 (55187279.45, 113978917.89)	87382.85 (60291.77, 124521.28)	0.08 (0.04, 0.11)
45-49 years	42008069.94 (31277629.72, 55764166.96)	81381.09 (60593.3, 108030.4)	92750989.23 (69103185.87, 123280769.69)	84073.31 (62637.97, 111746.76)	0.15 (0.11, 0.19)
50-54 years	41885819.81 (31116920.24, 55184413.61)	87790.93 (65219.76, 115664.22)	109528794.72 (81840157.28, 144759443.97)	90625.24 (67715.38, 119775.44)	0.16 (0.12, 0.2)
55-59 years	41089173.97 (30866727.11, 53677996.04)	94742.97 (71172.16, 123770.13)	108269817.94 (81929931.89, 141194388.49)	98478.7 (74520.8, 128425.81)	0.18 (0.14, 0.23)
60-64 years	34931730.22 (26380503.53, 45317928.53)	98851.84 (74653.08, 128243.3)	74439253.27 (56333662.79, 96621768.54)	101964.36 (77163.94, 132349.22)	0.16 (0.12, 0.2)
65-69 years	30150227.77 (23017402.73, 39181511.91)	110514.02 (84369.04, 143617.71)	85983361.85 (66296605.55, 111008470.82)	112098.34 (86432.29, 144724.1)	0.07 (0.04, 0.1)
70-74 years	22970306.68 (17454732.85, 30021605.41)	122067.88 (92757.24, 159539.6)	65392462.54 (50182397.47, 84460789.25)	122695.74 (94157.12, 158473.6)	0.03 (-0.01, 0.07)
75-79 years	14396536.47 (10827054.33, 18774555.4)	126499.6 (95135.25, 164968.41)	42922235.87 (32754314.42, 55175751.15)	129600.28 (98899.05, 166598.8)	0.08 (0.05, 0.12)
80-84 years	6958588 (5323212.63, 9054865.87)	131365.36 (100492.48, 170939.24)	27617848.21 (21498239.93, 35496168.14)	139541.48 (108621.65, 179347.34)	0.19 (0.17, 0.21)
85-89 years	2296189.35 (1742814.49, 2990701.63)	136122.46 (103317.35, 177294.47)	14163456.73 (10933384.53, 18209393.2)	148685.98 (114777.14, 191159.66)	0.25 (0.24, 0.27)

90-94 years	423884.36 (322692.48, 550114.9)	138153.15 (105172.51, 179294.44)	4507089.61 (3432254.17, 5805324.22)	153721.31 (117062.37, 197999.62)	0.31 (0.29, 0.32)
95 plus years	60873.36 (44564.99, 80272.46)	150333.98 (110058.52, 198242.33)	1067996.31 (787829.72, 1400630.35)	167109.83 (123272.04, 219157.21)	0.34 (0.31, 0.36)
**YLDs**					
**Gender**					
Female	20095179.54 (11857871.88, 31344307.2)	3804.81 (2288, 5854.14)	32920028.38 (19719318.91, 50461705.12)	3639.11 (2137.37, 5651.35)	-0.38 (-0.47, -0.29)
Male	13627465.17 (8189192.6, 21191029.64)	2532.65 (1558.71, 3876.31)	22765155.06 (14040255.77, 34607260.94)	2547.23 (1556.9, 3910.42)	0.01 (-0.13, 0.15)
**Age**					0.03 (-0.08, 0.14)
<5 years	63797.85 (39335.41, 96085.92)	57.06 (35.18, 85.94)	38846.1 (22117.9, 61098.63)	50.02 (28.48, 78.67)	-0.28 (-0.34, -0.22)
5-9 years	375316.76 (186705.39, 686936.03)	359.92 (179.05, 658.75)	340310.25 (169703.35, 623002.74)	355.34 (177.2, 650.52)	-0.64 (-0.76, -0.52)
10-14 years	1188130.48 (558484.9, 2172546.79)	1161.5 (545.97, 2123.86)	1011007.79 (458263.75, 1858842.73)	1172.96 (531.67, 2156.61)	-0.66 (-0.79, -0.53)
15-19 years	2439016.36 (1194522.52, 4293509.35)	1925.56 (943.05, 3389.65)	1378445.11 (642209.1, 2465268.18)	1846 (860.04, 3301.46)	-0.61 (-0.72, -0.5)
20-24 years	3258918.98 (1621551.05, 5769511.29)	2468.86 (1228.44, 4370.81)	1617637.34 (752022.67, 2948646.05)	2210.67 (1027.72, 4029.63)	-0.51 (-0.59, -0.43)
25-29 years	3039104.43 (1492405.07, 5275740.07)	2765.6 (1358.1, 4800.95)	2085771.76 (961616.16, 3748218.17)	2411.8 (1111.93, 4334.1)	-0.34 (-0.39, -0.29)
30-34 years	2740828.61 (1374497.77, 4719909.42)	3105.95 (1557.6, 5348.68)	3261575.14 (1563227.39, 5766487.26)	2692.11 (1290.29, 4759.66)	-0.19 (-0.21, -0.16)
35-39 years	3282083.54 (1716520.86, 5499516.17)	3593.3 (1879.28, 6020.99)	3332830.19 (1653254.41, 5785709.95)	3145.27 (1560.21, 5460.11)	-0.09 (-0.11, -0.08)
40-44 years	2729324.88 (1441485.94, 4550471.31)	4067.89 (2148.45, 6782.2)	3398940.3 (1734337.29, 5815231.53)	3713.32 (1894.75, 6353.11)	0 (-0.02, 0.02)
45-49 years	2345098.41 (1300506.23, 3847554.41)	4543.1 (2519.44, 7453.76)	4771134.38 (2580189.64, 7960251.22)	4324.75 (2338.79, 7215.5)	0.11 (0.09, 0.12)
50-54 years	2460636.32 (1383833.98, 4008954.18)	5157.39 (2900.46, 8402.6)	6083418.93 (3377775.78, 10058270.32)	5033.48 (2794.81, 8322.32)	0.15 (0.12, 0.17)
55-59 years	2470014.43 (1421061.13, 3945923.14)	5695.33 (3276.67, 9098.47)	6273885.73 (3568130.24, 10156951.63)	5706.52 (3245.45, 9238.43)	0.2 (0.17, 0.23)
60-64 years	2189321.15 (1284989.77, 3464139.68)	6195.47 (3636.34, 9803.02)	4656192.9 (2747445, 7350765.35)	6377.9 (3763.36, 10068.83)	0.32 (0.3, 0.35)
65-69 years	1870801.1 (1114572.44, 2909001.54)	6857.32 (4085.4, 10662.79)	5483399.83 (3304297.7, 8467670.75)	7148.82 (4307.88, 11039.48)	0.44 (0.43, 0.46)
70-74 years	1459798.02 (898551.17, 2243793.62)	7757.6 (4775.04, 11923.88)	4377426.71 (2707731.4, 6696748.39)	8213.36 (5080.51, 12565.09)	0.46 (0.44, 0.48)
75-79 years	1011658.12 (632127.32, 1531979.62)	8889.25 (5554.38, 13461.21)	3248143.66 (2052342.22, 4863098.02)	9807.51 (6196.88, 14683.74)	0.47 (0.44, 0.51)
80-84 years	550924.5 (347550.46, 820483.78)	10400.44 (6561.11, 15489.23)	2390534.3 (1527787.31, 3493569.08)	12078.37 (7719.27, 17651.55)	0.52 (0.49, 0.54)
85-89 years	201572.86 (129773.4, 299846.81)	11949.62 (7693.21, 17775.49)	1357138.08 (886912.45, 1982127.56)	14247.05 (9310.68, 20808.1)	-0.38 (-0.47, -0.29)
90-94 years	40421.41 (25857.53, 58732.23)	13174.22 (8427.53, 19142.11)	466707.04 (305199.85, 670396.81)	15917.77 (10409.32, 22864.93)	0.01 (-0.13, 0.15)
95 plus years	5876.5 (3822.4, 8624.96)	14512.7 (9439.87, 21300.37)	111837.91 (73275.95, 161764.65)	17499.32 (11465.52, 25311.38)	0.03 (-0.08, 0.14)

Notes: ASR: age-standardized rates; YLDs: years lived with disability; EAPCs: estimated annual percentage changes.

Table S32. Prevalence and YLDs of musculoskeletal disorders and its temporal trends from 1990, 2021 by gender, and age in China.

	1990		2021		
	Number	ASR, per 100,000 persons	Number	ASR, per 100,000 persons	EAPC 95%CI
**Prevalence**					
**Gender**					
Female	84676879.83 (71465489.86, 98268742.17)	17435.83 (14815.75, 20097.42)	165245937.54 (139266165.68, 191628822.75)	16196.8 (13709.44, 18709.21)	0.01 (-0.08, 0.09)
Male	54940145.13 (45779427.75, 64751103.51)	10902.38 (9201.02, 12687.27)	103025685.08 (86220307.79, 120653504.92)	10543.13 (8928.09, 12251.15)	0.02 (-0.03, 0.07)
**Age**					
5-9 years	3159335.43 (1703788, 5137513.89)	541.42 (291.98, 880.42)	3637339.81 (1986911.3, 5907938.55)	529.41 (289.19, 859.89)	-0.18 (-0.24, -0.12)
10-14 years	14624006.11 (10282608.08, 20277733.36)	2729.98 (1919.53, 3785.4)	17580736.81 (12408867.5, 24439047.35)	2637.23 (1861.42, 3666.03)	-0.14 (-0.2, -0.07)
15-19 years	26765007.12 (18426976.88, 37164021.58)	5152.83 (3547.58, 7154.86)	30795538.73 (21206546.94, 42820538.24)	4935.34 (3398.59, 6862.49)	-0.09 (-0.19, 0.01)
20-24 years	33311239.92 (22426697.99, 48510274.49)	6769.37 (4557.46, 9858.05)	38322457.84 (25808168.32, 55698600.63)	6417.47 (4321.83, 9327.28)	-0.09 (-0.27, 0.09)
25-29 years	37836602.03 (25464111.68, 53835822.64)	8548.33 (5753.05, 12163)	46126159.7 (31002224.09, 65924818.74)	7840.01 (5269.41, 11205.16)	-0.31 (-0.5, -0.12)
30-34 years	42019582.17 (29044123.04, 58883068.36)	10902.21 (7535.66, 15277.54)	58530080.35 (40522352.59, 82103103.96)	9682.71 (6703.67, 13582.43)	-0.54 (-0.69, -0.4)
35-39 years	50680636.72 (35687505.02, 69531417.45)	14387.92 (10131.46, 19739.53)	73671332.17 (51502663.23, 101465298.93)	13135.28 (9182.7, 18090.82)	-0.43 (-0.52, -0.33)
40-44 years	54968704.24 (38774783.14, 74888464.74)	19187.51 (13534.82, 26140.76)	90169106.14 (63853748.05, 123427215.89)	18024.78 (12764.35, 24673.07)	-0.14 (-0.22, -0.06)
45-49 years	57016942.87 (41122752.25, 76855551.05)	24555.58 (17710.4, 33099.5)	112389790.87 (81537590.58, 151819439.03)	23735.73 (17220.02, 32062.93)	0.07 (-0.01, 0.15)
50-54 years	65033135.95 (46621351.35, 88049009.59)	30593.54 (21932.08, 41420.9)	133588302.08 (95909547.28, 180803157.21)	30025.04 (21556.44, 40636.96)	0.15 (0.08, 0.23)
55-59 years	66598101.75 (49038970, 88425224.85)	35960.11 (26478.93, 47745.81)	141156959.59 (104795611.2, 187078404.82)	35670.2 (26481.73, 47274.49)	0.19 (0.11, 0.27)
60-64 years	65779477.38 (49100686.07, 85813524.69)	40956.22 (30571.52, 53430.01)	129852607.27 (97633112.66, 168933068.41)	40572.87 (30505.79, 52783.69)	0.21 (0.12, 0.29)
65-69 years	56842071.97 (43306142.26, 73925032.49)	45985.21 (35034.65, 59805.32)	124627446.89 (95608626.46, 160616160.25)	45180.71 (34660.63, 58227.56)	0.16 (0.08, 0.24)
70-74 years	43327786.84 (33046618.98, 55791643.11)	51177.88 (39033.98, 65899.93)	103704237.18 (79468538.69, 132589718.87)	50381.18 (38607.09, 64414.22)	0.05 (-0.03, 0.14)
75-79 years	34831632.75 (27001686.4, 44785453.93)	56585.72 (43865.58, 72756.2)	71578910.84 (55692155.49, 91697126.82)	54273.98 (42228.01, 69528.41)	-0.02 (-0.11, 0.06)
80-84 years	20425249.87 (16035993.62, 25857909.08)	57737.71 (45330.24, 73094.65)	49547008.3 (39222005.18, 62648421.86)	56571.45 (44782.64, 71530.3)	-0.01 (-0.06, 0.05)
85-89 years	8541030.73 (6591648.61, 10931864.16)	56521.67 (43621.32, 72343.4)	25781918.97 (20037242.13, 32835736.37)	56388.69 (43824.27, 71816.38)	0.05 (0.02, 0.09)
90-94 years	2238600.6 (1748973.11, 2850413.97)	52240.43 (40814.39, 66517.83)	9596464.47 (7555934.7, 12165345.53)	53643.38 (42237, 68003.19)	0.15 (0.1, 0.2)

95 plus years	488640.94 (373612.06, 634815.65)	47996.01 (36697.48, 62353.8)	2761395.76 (2140137.29, 3558105.76)	50664.94 (39266.35, 65282.65)	0.21 (0.14, 0.28)
**YLDs**					
**Gender**					
Female	7260259.14 (4880783.71, 10502898.01)	1443.14 (965.77, 2089.88)	12390446.36 (8045042.34, 18303186.89)	1264.17 (830.62, 1844.88)	-0.19 (-0.28, -0.1)
Male	4858711.63 (3236513.58, 7008203.36)	922.04 (612.89, 1340.96)	8076841.17 (5284511.02, 11884324.89)	854.8 (567.68, 1243.66)	-0.15 (-0.2, -0.1)
**Age**					
5-9 years	342569.5 (174076.76, 602386.61)	58.71 (29.83, 103.23)	395115.68 (203630.82, 691119.18)	57.51 (29.64, 100.59)	-0.17 (-0.22, -0.11)
10-14 years	1575929.97 (944942.46, 2418583.75)	294.19 (176.4, 451.5)	1895762.89 (1141518.88, 2915682.63)	284.38 (171.24, 437.37)	-0.12 (-0.18, -0.06)
15-19 years	2913502.06 (1686533.78, 4495425.5)	560.91 (324.69, 865.46)	3350919.98 (1931452.63, 5174562.73)	537.02 (309.54, 829.28)	-0.08 (-0.18, 0.03)
20-24 years	3692021.11 (2163021.92, 5941794.59)	750.28 (439.56, 1207.47)	4244464.09 (2475877.76, 6822208.33)	710.78 (414.61, 1142.45)	-0.07 (-0.26, 0.12)
25-29 years	4234082.57 (2456413.8, 6773758.15)	956.6 (554.97, 1530.38)	5154709.81 (2998369.24, 8252872.38)	876.14 (509.63, 1402.73)	-0.29 (-0.49, -0.1)
30-34 years	4653743.07 (2716781.41, 7180122.81)	1207.44 (704.88, 1862.92)	6448462.99 (3767155, 9934095.57)	1066.78 (623.21, 1643.41)	-0.55 (-0.7, -0.4)
35-39 years	5365770.7 (3148352.7, 8270534.06)	1523.31 (893.8, 2347.95)	7695730.81 (4495393.93, 11952655.71)	1372.12 (801.51, 2131.11)	-0.51 (-0.61, -0.4)
40-44 years	5424722.81 (3226549.88, 8361504.67)	1893.57 (1126.27, 2918.69)	8708847.78 (5179149.68, 13477884.8)	1740.9 (1035.31, 2694.23)	-0.29 (-0.36, -0.21)
45-49 years	5213141.8 (3124997.86, 8407549.59)	2245.15 (1345.85, 3620.89)	9959573.32 (5891683.14, 16192748.27)	2103.37 (1244.27, 3419.77)	-0.12 (-0.18, -0.06)
50-54 years	5558610.19 (3254159.27, 8886343.01)	2614.94 (1530.85, 4180.4)	10916068.93 (6324204.15, 17674320.38)	2453.47 (1421.42, 3972.44)	-0.06 (-0.12, -0.01)
55-59 years	5386352.42 (3153677.53, 8559962.91)	2908.4 (1702.85, 4622.01)	10903764.68 (6320923.61, 17582819.6)	2755.37 (1597.29, 4443.16)	-0.03 (-0.1, 0.03)
60-64 years	5110248.39 (3006935.29, 8128164.95)	3181.79 (1872.21, 5060.83)	9672304.06 (5682291.12, 15412992.43)	3022.14 (1775.45, 4815.84)	-0.01 (-0.08, 0.05)
65-69 years	4260052.74 (2484203.75, 6732988.05)	3446.38 (2009.72, 5446.98)	8944658.53 (5203609.66, 14208987.42)	3242.67 (1886.44, 5151.13)	-0.06 (-0.13, 0.01)
70-74 years	3161168.86 (1888482.95, 4968402.9)	3733.91 (2230.64, 5868.57)	7263900.51 (4324653.46, 11487528.49)	3528.92 (2100.99, 5580.83)	-0.15 (-0.24, -0.06)
75-79 years	2491713.92 (1483218.14, 3945065.36)	4047.91 (2409.56, 6408.96)	4880081.78 (2883849.03, 7819327.37)	3700.27 (2186.65, 5928.92)	-0.22 (-0.3, -0.13)
80-84 years	1407797.07 (866081.57, 2219155.55)	3979.53 (2448.22, 6273.07)	3281344.55 (1992524.12, 5214883.23)	3746.55 (2275.01, 5954.21)	-0.19 (-0.25, -0.13)
85-89 years	563611.04 (344976.95, 909356.65)	3729.79 (2282.94, 6017.82)	1635145.21 (999366.66, 2652921.62)	3576.29 (2185.76, 5802.31)	-0.13 (-0.17, -0.1)
90-94 years	139121.67 (84685.92, 217114.76)	3246.57 (1976.25, 5066.63)	576984.84 (352898.19, 906710.19)	3225.29 (1972.67, 5068.43)	-0.05 (-0.09, -0.01)
95 plus years	28797.79 (16937.26, 46541.04)	2828.62 (1663.64, 4571.42)	157824.2 (93485.26, 256525.94)	2895.69 (1715.23, 4706.63)	0 (-0.06, 0.05)

Notes: ASR: age-standardized rates; YLDs: years lived with disability; EAPCs: estimated annual percentage changes.

Table S33 Prevalence and YLDs of neurological disorders and its temporal trends from 1990, 2021 by gender, and age in China.

	1990		2021		EAPC 95%CI
	Number	ASR, per 100,000 persons	Number	ASR, per 100,000 persons	
**Prevalence**					
**Gender**					
Female	201197112.15 (173528588.39, 230265893.82)	35116.96 (30563.94, 39837.94)	293920288.46 (257426253.8, 334846544.78)	37690.43 (33025.82, 42920.68)	0.25 (0.21, 0.29)
Male	151749186.2 (131194014.75, 174252109.76)	25285.77 (22097.52, 28831.45)	223049429.52 (194472198.73, 255065307.35)	28042.94 (24407.81, 32008.54)	0.36 (0.31, 0.41)
**Age**					
<5 years	55882.05 (48723.59, 64316.48)	49.98 (43.58, 57.53)	23148.03 (20335.25, 26325.5)	29.8 (26.18, 33.89)	-2.19 (-2.34, -2.04)
5-9 years	7402783.87 (4831235.61, 10724560.72)	7099.09 (4633.04, 10284.59)	6883981.33 (4473005.72, 9844256.27)	7188.01 (4670.55, 10279.02)	0.07 (0.05, 0.09)
10-14 years	26989989.73 (20007286.87, 36048378.35)	26385.13 (19558.91, 35240.52)	23762845.3 (17598603.29, 31509042.57)	27569.36 (20417.68, 36556.4)	0.18 (0.15, 0.21)
15-19 years	40762030.1 (28010996.84, 55019589.36)	32180.92 (22114.2, 43437.02)	26243340.05 (18101449.33, 35543478.17)	35144.75 (24241.23, 47599.37)	0.34 (0.28, 0.4)
20-24 years	44858583.78 (32945622.28, 60398592.9)	33983.54 (24958.63, 45756.2)	27324989.42 (20160465.83, 37005769.66)	37342.4 (27551.35, 50572.18)	0.36 (0.29, 0.43)
25-29 years	40915408.72 (29991719.42, 54371230.71)	37233.24 (27292.63, 49478.11)	35245521.39 (26082680.38, 46327247.41)	40754.71 (30159.64, 53568.61)	0.35 (0.28, 0.42)
30-34 years	36052707.11 (25942419.63, 48137123.15)	40855.55 (29398.4, 54549.83)	54298025.33 (39703987.53, 71731800.53)	44817.63 (32771.7, 59207.48)	0.31 (0.25, 0.38)
35-39 years	37551926.18 (26818920.04, 49724651.69)	41112.67 (29361.94, 54439.64)	47426490.35 (34113445.17, 62470842.25)	44757.49 (32193.66, 58955.19)	0.3 (0.24, 0.37)
40-44 years	26950263.52 (20187469.14, 36202311.13)	40167.72 (30088.19, 53957.33)	40215660.7 (29771834.42, 53750160.39)	43935.37 (32525.55, 58721.73)	0.33 (0.27, 0.38)
45-49 years	18964575.96 (14229494.5, 24766678.34)	36739.56 (27566.41, 47979.81)	44959583.28 (33636117.41, 58801786.52)	40753.22 (30489.16, 53300.36)	0.39 (0.32, 0.46)
50-54 years	16521356.66 (12289046.73, 21654337.78)	34628.07 (25757.33, 45386.59)	45999986.08 (34672904.99, 61343254.68)	38060.86 (28688.72, 50756.04)	0.38 (0.31, 0.45)
55-59 years	14828772.75 (11061501.78, 19564396.85)	34192.02 (25505.49, 45111.37)	41674502.24 (31449108.57, 54990341.22)	37905.77 (28605.08, 50017.42)	0.39 (0.33, 0.46)
60-64 years	11812906.04 (8823185.38, 15366669.39)	33428.85 (24968.36, 43485.49)	26361894.17 (19559866.12, 34687169.14)	36109.63 (26792.44, 47513.31)	0.26 (0.22, 0.3)
65-69 years	11016945.55 (8312218.8, 14429053.97)	40382.02 (30467.99, 52888.91)	31934655.99 (24438210.28, 41776579.27)	41633.89 (31860.61, 54465.01)	0.03 (-0.01, 0.07)
70-74 years	8885605.03 (6651956.26, 11786378.38)	47219.52 (35349.56, 62634.7)	25855169.28 (19570129.48, 33629009.74)	48512 (36719.39, 63098.04)	0 (-0.04, 0.04)
75-79 years	5457938.34 (3896926.56, 7164222.8)	47957.86 (34241.55, 62950.65)	17321538.76 (12773619.39, 22346631.04)	52301.01 (38568.93, 67473.88)	0.18 (0.15, 0.22)
80-84 years	2734313.68 (2027348.37, 3676657.27)	51618.82 (38272.61, 69408.54)	11997063.91 (9257444.89, 15585951.54)	60616.16 (46774.01, 78749.32)	0.43 (0.4, 0.45)
85-89 years	962135.81 (713440.17, 1266974.48)	57037.24 (42294.09, 75108.65)	6612982.66 (5030371.25, 8551822.05)	69422.17 (52808.13, 89775.83)	0.49 (0.46, 0.53)

90-94 years	191631.35 (141561.3, 252716.2)	62456.83 (46137.91, 82365.72)	2247473.43 (1673909.98, 2921592.74)	76653.58 (57091.31, 99645.47)	0.51 (0.47, 0.55)
95 plus years	30542.11 (21878.8, 40472.02)	75427.35 (54032.28, 99950.44)	580866.28 (422591.26, 761960.12)	90888.39 (66123.03, 119224.22)	0.5 (0.47, 0.53)
**YLDs**					
**Gender**					
Female	4964620.45 (1608193.92, 9889731.19)	973.94 (368.27, 1842.67)	9751836.9 (4241254.86, 17234372.19)	1090.09 (420.03, 2019.6)	0.29 (0.26, 0.32)
Male	3529208.11 (1385753.77, 6920691.17)	710.69 (327.55, 1293.42)	7231185.98 (3615684.88, 12400729.83)	828.16 (396.14, 1466.34)	0.48 (0.46, 0.51)
**Age**					
<5 years	10780.15 (7460.98, 14441.4)	9.64 (6.67, 12.92)	4465.34 (3053.15, 5952.53)	5.75 (3.93, 7.66)	-2.19 (-2.34, -2.04)
5-9 years	83338.39 (18970.64, 232497.27)	79.92 (18.19, 222.96)	71932.62 (12012.61, 206930.52)	75.11 (12.54, 216.07)	-0.21 (-0.24, -0.18)
10-14 years	339869.79 (44641.19, 899357.42)	332.25 (43.64, 879.2)	295800.3 (30717.34, 781104.25)	343.18 (35.64, 906.23)	0.13 (0.1, 0.17)
15-19 years	681102.01 (109720.4, 1649141.18)	537.72 (86.62, 1301.97)	418287.63 (59101.25, 1010840.58)	560.17 (79.15, 1353.7)	0.15 (0.11, 0.19)
20-24 years	844296.46 (149008.88, 2053538.17)	639.61 (112.88, 1555.7)	486788.78 (75726.55, 1182430.72)	665.25 (103.49, 1615.91)	0.14 (0.1, 0.18)
25-29 years	776836.21 (140138.94, 1786890.84)	706.93 (127.53, 1626.08)	632498.01 (99738.47, 1466367.89)	731.36 (115.33, 1695.57)	0.14 (0.1, 0.18)
30-34 years	686879.76 (143693.43, 1537133.23)	778.38 (162.84, 1741.91)	978966.06 (187957, 2201620.73)	808.04 (155.14, 1817.22)	0.13 (0.09, 0.17)
35-39 years	770138.51 (193878.72, 1639517.26)	843.16 (212.26, 1794.98)	926908.57 (210923.52, 2023914.27)	874.75 (199.05, 1910.02)	0.12 (0.08, 0.15)
40-44 years	602546 (171428.94, 1281419.01)	898.06 (255.5, 1909.88)	856749.7 (222431.99, 1858680.54)	935.99 (243.01, 2030.6)	0.12 (0.08, 0.15)
45-49 years	490652.77 (167103.69, 962218.5)	950.53 (323.73, 1864.08)	1089234.52 (342162.88, 2210790.5)	987.33 (310.15, 2003.95)	0.11 (0.08, 0.14)
50-54 years	508261.16 (201882.47, 953671.56)	1065.29 (423.14, 1998.86)	1341868.97 (506351.28, 2553410.68)	1110.28 (418.96, 2112.72)	0.13 (0.1, 0.17)
55-59 years	521396.54 (242039.8, 920006.23)	1202.23 (558.09, 2121.34)	1438544.46 (652194.12, 2567436.32)	1308.45 (593.21, 2335.26)	0.28 (0.25, 0.31)
60-64 years	494386.5 (258533.2, 825887.95)	1399.04 (731.61, 2337.15)	1185194.18 (620703.89, 1965503.02)	1623.44 (850.22, 2692.28)	0.47 (0.44, 0.5)
65-69 years	478731.9 (272811.2, 768165.54)	1754.77 (999.97, 2815.67)	1635602.63 (946803.23, 2572206.05)	2132.37 (1234.37, 3353.44)	0.57 (0.54, 0.6)
70-74 years	439274.45 (267908.1, 676542.38)	2334.37 (1423.71, 3595.25)	1584855.16 (972869.99, 2399636.63)	2973.66 (1825.39, 4502.43)	0.67 (0.64, 0.71)
75-79 years	371413.55 (238185.21, 546096.83)	3263.54 (2092.89, 4798.45)	1460907.48 (949125.01, 2117534.04)	4411.09 (2865.81, 6393.73)	0.86 (0.82, 0.9)
80-84 years	255015.45 (161597.78, 366448.45)	4814.22 (3050.67, 6917.87)	1320317.92 (852359.11, 1850933.43)	6671.02 (4306.62, 9352)	0.89 (0.86, 0.93)
85-89 years	109876.45 (71652.88, 157281.34)	6513.68 (4247.72, 9323.94)	848524.29 (562717.33, 1194141.8)	8907.69 (5907.33, 12535.93)	0.78 (0.73, 0.84)
90-94 years	25014.55 (16198.92, 35329.09)	8152.79 (5279.58, 11514.52)	322308.22 (213776.66, 450951.23)	10992.82 (7291.19, 15380.39)	0.73 (0.67, 0.79)
95 plus years	4017.97 (2701.37, 5681.4)	9922.85 (6671.35, 14030.88)	83268.02 (56120.48, 116621.95)	13028.98 (8781.19, 18247.89)	0.73 (0.68, 0.77)

Notes: ASR: age-standardized rates; YLDs: years lived with disability; EAPCs: estimated annual percentage changes.

Table S34. Prevalence and YLDs of digestive disorders and its temporal trends from 1990, 2021 by gender, and age in China.

	1990		2021		EAPC 95%CI
	Number	ASR, per 100,000 persons	Number	ASR, per 100,000 persons	
**Prevalence**					
**Gender**					
Female	173054.63 (120787.32, 244502.96)	34.66 (24.07, 49.11)	281780.09 (211599.28, 379663.29)	28.59 (21.85, 37.74)	-0.36 (-0.74, 0.03)
Male	241053.34 (169021.87, 331111.98)	47.12 (32.66, 65.28)	374424.75 (273550.88, 512637.56)	38.08 (28.35, 51.03)	-0.56 (-0.93, -0.19)
**Age**					
<5 years	1545.7 (702.03, 3051.72)	1.38 (0.63, 2.73)	815.93 (400.38, 1562.19)	1.05 (0.52, 2.01)	-1.36 (-1.93, -0.79)
5-9 years	4582.36 (2166.16, 8400.56)	4.39 (2.08, 8.06)	2563.69 (1309.63, 4408.47)	2.68 (1.37, 4.6)	-1.69 (-2.26, -1.1)
10-14 years	7694.71 (4164.47, 13569.75)	7.52 (4.07, 13.27)	3963.35 (2246.96, 6621)	4.6 (2.61, 7.68)	-1.48 (-2.04, -0.92)
15-19 years	14886.25 (7853.45, 25115.25)	11.75 (6.2, 19.83)	5661.07 (3621.09, 8968.31)	7.58 (4.85, 12.01)	-1.08 (-1.64, -0.53)
20-24 years	22641.07 (12720.9, 37131.79)	17.15 (9.64, 28.13)	8642.71 (5763.74, 12769.51)	11.81 (7.88, 17.45)	-0.72 (-1.28, -0.16)
25-29 years	26251.36 (15156.94, 43666.67)	23.89 (13.79, 39.74)	15005.36 (10011.75, 22256.99)	17.35 (11.58, 25.74)	-0.51 (-1.04, 0.04)
30-34 years	27725.61 (16255.78, 46615.38)	31.42 (18.42, 52.83)	28778.68 (19187.76, 44088.41)	23.75 (15.84, 36.39)	-0.41 (-0.9, 0.08)
35-39 years	36178.49 (21095.61, 61578.34)	39.61 (23.1, 67.42)	33448.68 (21963.75, 51327.11)	31.57 (20.73, 48.44)	-0.34 (-0.78, 0.11)
40-44 years	34080.37 (20847.93, 56682.99)	50.79 (31.07, 84.48)	38169.66 (25009.87, 57393.79)	41.7 (27.32, 62.7)	-0.32 (-0.72, 0.08)
45-49 years	33314.48 (19514.15, 53295.22)	64.54 (37.8, 103.25)	59519.07 (37295.23, 90334.76)	53.95 (33.81, 81.88)	-0.33 (-0.7, 0.04)
50-54 years	39256.83 (23201.46, 65841.64)	82.28 (48.63, 138)	83330.69 (53154.87, 129458.43)	68.95 (43.98, 107.12)	-0.4 (-0.76, -0.05)
55-59 years	43598.63 (25680.87, 73111.99)	100.53 (59.21, 168.58)	92765.59 (59271.34, 144107.14)	84.38 (53.91, 131.08)	-0.46 (-0.81, -0.11)
60-64 years	40197.2 (23357.76, 65172.7)	113.75 (66.1, 184.43)	70434.47 (44720.26, 106823.61)	96.48 (61.26, 146.32)	-0.47 (-0.81, -0.14)
65-69 years	33475.76 (20106.95, 53417.02)	122.7 (73.7, 195.8)	81068.14 (50504.75, 124121.35)	105.69 (65.84, 161.82)	-0.46 (-0.78, -0.14)
70-74 years	24252.6 (14693.15, 38831.24)	128.88 (78.08, 206.36)	59421.84 (37629.33, 88490.73)	111.49 (70.6, 166.03)	-0.45 (-0.76, -0.14)
75-79 years	15009.42 (9345.2, 22958.49)	131.88 (82.11, 201.73)	37502.43 (24362.95, 54901.58)	113.24 (73.56, 165.77)	-0.48 (-0.8, -0.15)
80-84 years	6872.17 (4525.68, 10357.41)	129.73 (85.44, 195.53)	21719.37 (14912.05, 31997.1)	109.74 (75.34, 161.67)	-0.56 (-0.9, -0.21)
85-89 years	2122.23 (1355.47, 3212.65)	125.81 (80.35, 190.45)	9877.08 (6648.11, 14823.76)	103.69 (69.79, 155.62)	-0.66 (-1.03, -0.28)

90-94 years	375.09 (228.8, 584.48)	122.25 (74.57, 190.49)	2909.48 (1864.31, 4451.79)	99.23 (63.59, 151.84)	-0.72 (-1.12, -0.32)
95 plus years	47.62 (28.29, 81.01)	117.6 (69.87, 200.07)	607.53 (372.27, 990.43)	95.06 (58.25, 154.97)	-0.76 (-1.15, -0.36)
**YLDs**					
**Gender**					
Female	16631.56 (9779.27, 25833.75)	3.26 (1.93, 5.02)	29235.26 (17467.22, 45010.01)	3.06 (1.85, 4.67)	0.24 (-0.1, 0.59)
Male	20984.35 (11767.28, 33509.08)	3.98 (2.23, 6.34)	34200.91 (19916.45, 54284.35)	3.58 (2.12, 5.6)	-0.05 (-0.35, 0.25)
**Age**					
<5 years	148.53 (72.63, 277.06)	0.13 (0.06, 0.25)	80.47 (40.25, 144.27)	0.1 (0.05, 0.19)	-1.27 (-1.74, -0.8)
5-9 years	404.22 (184.62, 779.38)	0.39 (0.18, 0.75)	239.29 (118.77, 435.83)	0.25 (0.12, 0.46)	-1.42 (-1.88, -0.97)
10-14 years	691.22 (361.08, 1217.77)	0.68 (0.35, 1.19)	410.01 (228.03, 697.47)	0.48 (0.26, 0.81)	-0.85 (-1.29, -0.41)
15-19 years	1407.25 (719, 2406.16)	1.11 (0.57, 1.9)	654.83 (376.94, 1040.6)	0.88 (0.5, 1.39)	-0.22 (-0.73, 0.28)
20-24 years	2286.62 (1234.79, 3769.75)	1.73 (0.94, 2.86)	1086.7 (636.7, 1684.32)	1.49 (0.87, 2.3)	0.18 (-0.36, 0.73)
25-29 years	2795.64 (1502.66, 4725.34)	2.54 (1.37, 4.3)	1963.33 (1173.36, 3077.1)	2.27 (1.36, 3.56)	0.33 (-0.2, 0.86)
30-34 years	3049.01 (1652.88, 5215.61)	3.46 (1.87, 5.91)	3780.09 (2152.26, 6111.86)	3.12 (1.78, 5.04)	0.32 (-0.15, 0.79)
35-39 years	3887.46 (2104.61, 6546.22)	4.26 (2.3, 7.17)	4216.83 (2435.44, 6734.8)	3.98 (2.3, 6.36)	0.33 (-0.07, 0.73)
40-44 years	3424.57 (1820.63, 5926.29)	5.1 (2.71, 8.83)	4433.28 (2493.07, 7287.6)	4.84 (2.72, 7.96)	0.31 (-0.05, 0.66)
45-49 years	3119.74 (1639.98, 5556.82)	6.04 (3.18, 10.77)	6370.01 (3523.68, 10719.92)	5.77 (3.19, 9.72)	0.26 (-0.06, 0.59)
50-54 years	3403.02 (1702.72, 6101.19)	7.13 (3.57, 12.79)	8157.78 (4426.88, 13863.76)	6.75 (3.66, 11.47)	0.16 (-0.14, 0.46)
55-59 years	3516.49 (1679.3, 6524.88)	8.11 (3.87, 15.05)	8363.21 (4361.47, 14602.3)	7.61 (3.97, 13.28)	0.06 (-0.22, 0.34)
60-64 years	3131.17 (1601.21, 5472.87)	8.86 (4.53, 15.49)	6010.63 (3139.44, 10180.34)	8.23 (4.3, 13.94)	-0.03 (-0.31, 0.24)
65-69 years	2577.82 (1361.21, 4508.73)	9.45 (4.99, 16.53)	6750.71 (3553.14, 11245.32)	8.8 (4.63, 14.66)	-0.08 (-0.33, 0.18)
70-74 years	1860.11 (953.09, 3152.92)	9.88 (5.06, 16.76)	4846.07 (2557.82, 8024.3)	9.09 (4.8, 15.06)	-0.16 (-0.39, 0.08)
75-79 years	1168.56 (621.38, 1900.86)	10.27 (5.46, 16.7)	3070.21 (1658.89, 5058.92)	9.27 (5.01, 15.28)	-0.24 (-0.48, -0.01)
80-84 years	542.37 (299.51, 899.88)	10.24 (5.65, 16.99)	1829.12 (1042.35, 3007.71)	9.24 (5.27, 15.2)	-0.28 (-0.5, -0.05)
85-89 years	168.25 (92.71, 292.55)	9.97 (5.5, 17.34)	850.68 (474.23, 1408.56)	8.93 (4.98, 14.79)	-0.31 (-0.54, -0.08)
90-94 years	30.12 (15.85, 50.69)	9.82 (5.17, 16.52)	265.53 (144.97, 437.74)	9.06 (4.94, 14.93)	-0.23 (-0.47, 0)
95 plus years	3.74 (1.95, 6.39)	9.23 (4.81, 15.78)	57.38 (30.69, 95.2)	8.98 (4.8, 14.9)	-0.09 (-0.29, 0.11)

Notes: ASR: age-standardized rates; YLDs: years lived with disability; EAPCs: estimated annual percentage changes.

Table S35. Prevalence and YLDs of genecological disorders and its temporal trends from 1990, 2021 by gender, and age in China.

	1990		2021		EAPC 95%CI
	Number	ASR, per 100,000 persons	Number	ASR, per 100,000 persons	
**Prevalence**					
**Gender**					
Female	59689999.8 (32625583.68, 104614493.31)	2267.26 (1219.63, 3969.94)	110089459.27 (58608815.06, 195025585.22)	2764.62 (1476.33, 4862.57)	0.7 (0.53, 0.87)
Male	31490381.81 (18725068.36, 50165061.42)	1158.86 (696.62, 1858.35)	55000818.26 (32611257.3, 88727953.05)	1354.76 (802.12, 2174.77)	0.5 (0.36, 0.64)
**Age**					
15-19 years	229512.21 (29323.42, 773843.28)	181.2 (23.15, 610.94)	112107.73 (18789.35, 406472.27)	150.13 (25.16, 544.34)	-0.68 (-0.77, -0.58)
20-24 years	3542564.17 (1062056.15, 7866287.67)	2683.74 (804.58, 5959.27)	2029680.12 (576748.6, 4551140.14)	2773.77 (788.19, 6219.6)	0 (-0.08, 0.09)
25-29 years	5674832.48 (1781333.12, 14397892.3)	5164.13 (1621.02, 13102.16)	4733894.68 (1475757.93, 11845154.92)	5473.85 (1706.43, 13696.66)	0.09 (0.02, 0.17)
30-34 years	7959154.64 (2052734.01, 17706101.05)	9019.45 (2326.19, 20064.86)	11497774.49 (2851059.73, 26563765.46)	9490.27 (2353.27, 21925.75)	0.04 (0, 0.08)
35-39 years	11659380.08 (3903371.1, 24559395.31)	12764.94 (4273.5, 26888.16)	13979442.97 (4540194.68, 30083410.61)	13192.73 (4284.69, 28390.42)	0.03 (0.01, 0.05)
40-44 years	5824826.43 (1775923.7, 12507950.26)	8681.55 (2646.91, 18642.33)	8429036.1 (2661322.87, 18033806.41)	9208.67 (2907.48, 19701.83)	0.02 (-0.07, 0.11)
45-49 years	162637.85 (27397.35, 700685.96)	315.07 (53.08, 1357.42)	380868.5 (65758.91, 1692636.44)	345.23 (59.61, 1534.28)	-0.29 (-0.65, 0.06)
**YLDs**					
**Gender**					
Female	325936.79 (114823.01, 807747)	12.32 (4.39, 30.93)	601133.5 (213157.97, 1468475.01)	15.12 (5.35, 36.88)	0.71 (0.54, 0.88)
Male	181868.97 (66532.49, 425578.94)	6.65 (2.47, 15.61)	317613.61 (116288.11, 752758.23)	7.84 (2.85, 18.56)	0.51 (0.38, 0.65)
**Age**					
15-19 years	1370.66 (113.94, 5552.34)	1.08 (0.09, 4.38)	684.57 (75.26, 3017.88)	0.92 (0.1, 4.04)	-0.65 (-0.75, -0.54)
20-24 years	20334.64 (4394.26, 60077.78)	15.4 (3.33, 45.51)	11971.04 (2353.76, 34252.38)	16.36 (3.22, 46.81)	0.04 (-0.06, 0.14)
25-29 years	30645.3 (6318.19, 88618.24)	27.89 (5.75, 80.64)	26136.61 (5325.03, 77503.21)	30.22 (6.16, 89.62)	0.12 (0.03, 0.21)
30-34 years	40907.08 (8675.82, 114559.93)	46.36 (9.83, 129.82)	59771.81 (12363.39, 170378.03)	49.34 (10.2, 140.63)	0.06 (0.01, 0.11)
35-39 years	60123.39 (15740.92, 166568.44)	65.82 (17.23, 182.36)	72427.42 (18224.96, 205146.03)	68.35 (17.2, 193.6)	0.04 (0.02, 0.06)
40-44 years	30382.24 (6806.5, 89915.27)	45.28 (10.14, 134.01)	44156.05 (10202.01, 130613.51)	48.24 (11.15, 142.69)	0.03 (-0.07, 0.12)
45-49 years	867.57 (123.23, 4174.86)	1.68 (0.24, 8.09)	2034.98 (280.38, 9570.56)	1.84 (0.25, 8.68)	-0.3 (-0.65, 0.05)

Notes: ASR: age-standardized rates; YLDs: years lived with disability; EAPCs: estimated annual percentage changes.

Table S36. Prevalence and YLDs of mental disorders and its temporal trends from 1990, 2021 by gender, and age in China.

	1990		2021		EAPC 95%CI
	Number	ASR, per 100,000 persons	Number	ASR, per 100,000 persons	
**Prevalence**					
**Gender**					
Female	46174874.44 (40557663.65, 52582761.53)	8156.54 (7215.38, 9217.17)	65243313.64 (56997164.1, 74010730.13)	7924.35 (6928.86, 9055.86)	-0.44 (-0.53, -0.35)
Male	28800788.26 (25248945.34, 32886473.41)	4877.74 (4291.63, 5518.83)	41003729.55 (35874975.39, 46904995.87)	4855.37 (4235.64, 5552.12)	-0.36 (-0.44, -0.28)
**Age**					
<5 years	125675.36 (88000.79, 174368)	112.4 (78.71, 155.96)	103003.4 (72501.21, 141474.49)	132.62 (93.35, 182.15)	0.38 (0.17, 0.58)
5-9 years	1779961.75 (1271765.2, 2465983.45)	1706.94 (1219.59, 2364.82)	1699430.65 (1204694.98, 2302733.64)	1774.48 (1257.9, 2404.43)	0.26 (0.03, 0.48)
10-14 years	4777562.64 (3434021.83, 6411291.79)	4670.5 (3357.06, 6267.61)	4081173.89 (2957474.26, 5493738.98)	4734.93 (3431.23, 6373.77)	0.07 (-0.14, 0.27)
15-19 years	8306762.32 (6489915.29, 10492372.22)	6558.05 (5123.68, 8283.55)	4617004.13 (3548906.09, 5858298.15)	6183.03 (4752.65, 7845.36)	-0.43 (-0.54, -0.31)
20-24 years	9419666.4 (7203683.99, 12190755.73)	7136.06 (5457.3, 9235.36)	4530847.6 (3454764.1, 5933294.93)	6191.87 (4721.29, 8108.46)	-1.02 (-1.17, -0.87)
25-29 years	7826124.53 (6070619.9, 9937698.68)	7121.82 (5524.3, 9043.36)	5361772.24 (4124487.93, 6904451.05)	6199.87 (4769.18, 7983.68)	-1.04 (-1.24, -0.84)
30-34 years	6478250.82 (5159202.67, 8048565.75)	7341.27 (5846.5, 9120.77)	8050967.08 (6409117.39, 10029840.95)	6645.27 (5290.09, 8278.64)	-0.88 (-1.07, -0.68)
35-39 years	7205211.02 (5880488.5, 8741116.6)	7888.42 (6438.09, 9569.97)	7661426.64 (6172599.08, 9381306.1)	7230.27 (5825.23, 8853.36)	-0.72 (-0.86, -0.58)
40-44 years	5649590.05 (4419651.29, 7056262.51)	8420.37 (6587.22, 10516.93)	7289788.58 (5664091.52, 9214380.7)	7964.05 (6187.99, 10066.66)	-0.53 (-0.63, -0.43)
45-49 years	4555285.19 (3662819.85, 5592236.18)	8824.83 (7095.88, 10833.69)	9736104.76 (7762250.81, 12010373.32)	8825.21 (7036.02, 10886.7)	-0.34 (-0.43, -0.26)
50-54 years	4388910.2 (3673592.25, 5275401.54)	9198.97 (7699.7, 11057.02)	11617594.97 (9611032.49, 13977108.32)	9612.52 (7952.27, 11564.8)	-0.22 (-0.31, -0.13)
55-59 years	4154823.09 (3454067.67, 4977519.67)	9580.15 (7964.35, 11477.11)	11412480.99 (9466005.12, 13689025.57)	10380.42 (8609.97, 12451.09)	-0.15 (-0.26, -0.04)
60-64 years	3531194.24 (2829528.32, 4433876.34)	9992.78 (8007.16, 12547.24)	7972437.96 (6440810.08, 9898737.56)	10920.38 (8822.4, 13558.95)	-0.11 (-0.23, 0.02)
65-69 years	2823484.65 (2276843.78, 3564189.52)	10349.33 (8345.65, 13064.34)	8621084.52 (7010135.92, 10802036.36)	11239.49 (9139.26, 14082.84)	-0.09 (-0.21, 0.04)
70-74 years	1973960.48 (1604439.76, 2444454.42)	10489.94 (8526.25, 12990.22)	6045272.81 (4915726.33, 7476433.57)	11342.73 (9223.37, 14028.02)	-0.07 (-0.19, 0.05)
75-79 years	1199218.55 (974603.02, 1466618.22)	10537.3 (8563.65, 12886.89)	3753740.18 (3063006.33, 4595896.34)	11334.12 (9248.5, 13876.94)	-0.02 (-0.13, 0.1)
80-84 years	562543.36 (457594.52, 684024.78)	10619.79 (8638.54, 12913.13)	2237983.11 (1801261.77, 2746983.13)	11307.6 (9101.03, 13879.36)	0.04 (-0.08, 0.15)
85-89 years	180512.25 (147311.61, 219697.73)	10701.11 (8732.91, 13024.1)	1065634.16 (869838.38, 1294007.31)	11186.88 (9131.44, 13584.31)	0.04 (-0.08, 0.16)

90-94 years	32633.59 (26016.54, 40612.6)	10636 (8479.36, 13236.53)	321403.32 (255088.1, 397624.89)	10961.96 (8700.18, 13561.62)	0.04 (-0.1, 0.17)
95 plus years	4292.18 (3150.93, 5615.59)	10600.05 (7781.6, 13868.36)	67892.2 (48828.71, 89314.49)	10623.12 (7640.25, 13975.08)	0.02 (-0.09, 0.13)
**YLDs**					
**Gender**					
Female	6394080.33 (4437549.18, 8719291.4)	1116.78 (781.09, 1516.51)	8706763.29 (6123534.44, 11799329.69)	1058.62 (745.45, 1439.82)	-0.48 (-0.56, -0.4)
Male	3928531.27 (2724968.46, 5368336.18)	658.3 (461.41, 897.33)	5473628.88 (3835313.39, 7439378)	649.39 (452.36, 888.97)	-0.42 (-0.51, -0.33)
**Age**					
<5 years	15831.68 (9559.87, 23987.2)	14.16 (8.55, 21.45)	13066.88 (7715.77, 19839.7)	16.82 (9.93, 25.54)	0.41 (0.2, 0.62)
5-9 years	227185.73 (136418.64, 336584.41)	217.87 (130.82, 322.78)	217827.14 (132881.47, 326255.1)	227.45 (138.75, 340.66)	0.27 (0.05, 0.5)
10-14 years	632786.63 (387590.69, 929619.38)	618.61 (378.9, 908.79)	535772.12 (321884.28, 797018.08)	621.6 (373.45, 924.69)	0.06 (-0.15, 0.27)
15-19 years	1178965.36 (760278.42, 1736911.57)	930.77 (600.23, 1371.26)	627687.11 (398335.21, 932394.32)	840.59 (533.45, 1248.65)	-0.54 (-0.65, -0.43)
20-24 years	1389916.32 (896377.83, 2041259.86)	1052.96 (679.07, 1546.4)	627060.55 (396168.51, 927248.93)	856.94 (541.4, 1267.18)	-1.22 (-1.38, -1.06)
25-29 years	1136313.48 (727062.14, 1669923.49)	1034.05 (661.63, 1519.64)	732077.39 (466458.13, 1076965.07)	846.51 (539.37, 1245.3)	-1.22 (-1.44, -1.01)
30-34 years	920143.18 (590352.78, 1349375.53)	1042.72 (669, 1529.14)	1080841.27 (704230.13, 1577690.66)	892.13 (581.27, 1302.23)	-1.04 (-1.25, -0.83)
35-39 years	1003290.25 (662221.76, 1406611.68)	1098.42 (725.01, 1539.99)	1016346.78 (659767.29, 1443903.32)	959.15 (622.64, 1362.65)	-0.86 (-1.03, -0.7)
40-44 years	771164.75 (487114.85, 1104215.62)	1149.37 (726.01, 1645.77)	962011.39 (604301.97, 1394830.71)	1050.99 (660.2, 1523.84)	-0.63 (-0.75, -0.51)
45-49 years	610994.41 (403068.13, 861220.13)	1183.66 (780.85, 1668.42)	1295586.62 (860070, 1829711.59)	1174.37 (779.6, 1658.53)	-0.37 (-0.47, -0.27)
50-54 years	579835.12 (393249.36, 801270.14)	1215.31 (824.23, 1679.43)	1556886.76 (1059735.5, 2168396.56)	1288.18 (876.84, 1794.15)	-0.17 (-0.26, -0.08)
55-59 years	540189.21 (366049.81, 742511.29)	1245.56 (844.03, 1712.07)	1533136.41 (1040010.2, 2132410.19)	1394.49 (945.96, 1939.57)	-0.04 (-0.14, 0.06)
60-64 years	452928.91 (304686.6, 643159.32)	1281.72 (862.22, 1820.05)	1067675.21 (721973.36, 1510976.93)	1462.47 (988.93, 2069.68)	0.04 (-0.07, 0.15)
65-69 years	360722.77 (243264, 507426.8)	1322.21 (891.67, 1859.95)	1146962.75 (786401.87, 1608184.76)	1495.32 (1025.25, 2096.62)	0.07 (-0.03, 0.17)
70-74 years	251274.3 (171845.33, 345656.56)	1335.31 (913.21, 1836.87)	799827.16 (549955.26, 1102551.72)	1500.71 (1031.88, 2068.72)	0.1 (0.01, 0.19)
75-79 years	152347.61 (104934.95, 210548.29)	1338.65 (922.04, 1850.05)	493337.14 (335742.25, 688735.2)	1489.59 (1013.75, 2079.58)	0.14 (0.06, 0.22)
80-84 years	71230.82 (48879.34, 99187.09)	1344.71 (922.75, 1872.47)	290200.85 (200512.44, 405133.72)	1466.26 (1013.11, 2046.97)	0.17 (0.1, 0.25)
85-89 years	22822.38 (15811.23, 31509.64)	1352.95 (937.32, 1867.95)	135583.01 (94745.49, 188057.12)	1423.33 (994.62, 1974.2)	0.12 (0.05, 0.2)
90-94 years	4130.35 (2789.49, 5835.55)	1346.17 (909.16, 1901.93)	40178.96 (27598.96, 56490.77)	1370.37 (941.31, 1926.71)	0.07 (-0.03, 0.16)
95 plus years	538.32 (340.31, 796.26)	1329.46 (840.43, 1966.46)	8326.69 (5178.54, 12250.98)	1302.88 (810.29, 1916.92)	0.01 (-0.08, 0.09)

Notes: ASR: age-standardized rates; YLDs: years lived with disability; EAPCs: estimated annual percentage changes.

Table S37. Prevalence and YLDs of substance use disorders and its temporal trends from 1990, 2021 by gender, and age in China.

	1990		2021		EAPC 95%CI
	Number	ASR, per 100,000 persons	Number	ASR, per 100,000 persons	
**Prevalence**					
**Gender**					
Female	1329304.68 (1106812.23, 1568985.75)	218.94 (186.15, 254.5)	790717.34 (646483.56, 942570.3)	102.29 (82.22, 124)	-3.26 (-3.61, -2.92)
Male	1068453.75 (897710.38, 1254084.99)	166.96 (143.03, 193.44)	681550.32 (579199.51, 793300.4)	86.63 (72.3, 102.07)	-3.36 (-3.81, -2.9)
**Age**					
15-19 years	101964.35 (69615.18, 142367.97)	80.5 (54.96, 112.4)	29651.87 (18872.13, 43457.03)	39.71 (25.27, 58.2)	-3.35 (-3.79, -2.91)
20-24 years	473171.17 (334452.34, 646429.73)	358.46 (253.37, 489.72)	131263.08 (88365.55, 189784.55)	179.38 (120.76, 259.36)	-3.38 (-3.83, -2.92)
25-29 years	488336.46 (372744.85, 622367.57)	444.39 (339.2, 566.36)	195860.76 (143416.01, 257959.7)	226.48 (165.83, 298.28)	-3.35 (-3.8, -2.89)
30-34 years	340419.64 (257572.28, 439823.59)	385.77 (291.89, 498.42)	242518.15 (176699.51, 324744.16)	200.17 (145.85, 268.04)	-3.26 (-3.7, -2.82)
35-39 years	297430.02 (231203.07, 387824.3)	325.63 (253.13, 424.6)	175808.65 (132546.49, 231031.56)	165.91 (125.09, 218.03)	-3.25 (-3.66, -2.84)
40-44 years	185907.49 (136499.61, 243034.45)	277.08 (203.44, 362.23)	124435.27 (87730.72, 168434.59)	135.94 (95.85, 184.01)	-3.31 (-3.68, -2.93)
45-49 years	130745.82 (99006.45, 170416.58)	253.29 (191.8, 330.14)	131586.38 (96017.26, 174911.44)	119.28 (87.03, 158.55)	-3.33 (-3.66, -3)
50-54 years	111991.17 (82844.13, 149305.64)	234.73 (173.64, 312.94)	132111.37 (90257.19, 180629.62)	109.31 (74.68, 149.45)	-3.28 (-3.57, -3)
55-59 years	88399.34 (66141.9, 115519.34)	203.83 (152.51, 266.36)	107010.53 (76470.42, 143947.27)	97.33 (69.56, 130.93)	-3.06 (-3.29, -2.83)
60-64 years	62043.99 (45208.33, 85351.74)	175.58 (127.93, 241.53)	62001.3 (42622.59, 89457.69)	84.93 (58.38, 122.54)	-2.84 (-3.01, -2.68)
65-69 years	47768.74 (36710.93, 60870.66)	175.09 (134.56, 223.12)	59242.45 (42277.04, 78591.23)	77.24 (55.12, 102.46)	-3.16 (-3.33, -2.99)
70-74 years	35703.75 (25858.51, 47936.81)	189.74 (137.42, 254.74)	38601.95 (27825.78, 52623.31)	72.43 (52.21, 98.74)	-3.74 (-3.95, -3.53)
75-79 years	21571.27 (16279.88, 28373.28)	189.54 (143.05, 249.31)	22464.1 (16777.48, 29350.02)	67.83 (50.66, 88.62)	-4.05 (-4.29, -3.81)
80-84 years	9268.25 (6675.84, 12306.02)	174.97 (126.03, 232.32)	12479.51 (8983.87, 16557.55)	63.05 (45.39, 83.66)	-4.13 (-4.39, -3.86)
85-89 years	2594.4 (1966.98, 3371.54)	153.8 (116.61, 199.87)	5449.67 (4164.08, 6967.31)	57.21 (43.71, 73.14)	-4.07 (-4.37, -3.78)
90-94 years	396.6 (297.9, 518.79)	129.26 (97.09, 169.09)	1485.06 (1131.46, 1928.13)	50.65 (38.59, 65.76)	-3.9 (-4.19, -3.61)
95 plus years	45.97 (31.8, 62.5)	113.52 (78.53, 154.36)	297.56 (210.99, 396.13)	46.56 (33.01, 61.98)	-3.55 (-3.76, -3.34)
**YLDs**					

**Gender**					
Female	553614.68 (369028.19, 732751.71)	90.52 (61.34, 118.95)	325016.12 (222018.29, 419138.67)	42.49 (28.53, 56.03)	-3.25 (-3.6, -2.91)
Male	453557.94 (301255.41, 596860.7)	70.34 (47.79, 91.53)	286719.62 (196447.38, 370077.97)	36.69 (24.86, 48.34)	-3.34 (-3.8, -2.88)
**Age**					
15-19 years	44218.22 (25017.97, 65527.57)	34.91 (19.75, 51.73)	12919.84 (6771.84, 20465.9)	17.3 (9.07, 27.41)	-3.33 (-3.76, -2.89)
20-24 years	203675.48 (122173.25, 294239.58)	154.3 (92.55, 222.91)	56752.57 (31962.85, 88678.65)	77.56 (43.68, 121.19)	-3.36 (-3.81, -2.9)
25-29 years	208554.38 (132916.67, 291547.05)	189.79 (120.95, 265.31)	83988.84 (53651.1, 118920.2)	97.12 (62.04, 137.51)	-3.33 (-3.78, -2.87)
30-34 years	143969.67 (93680.96, 204883.28)	163.15 (106.16, 232.18)	103274.62 (66368.5, 148778.14)	85.24 (54.78, 122.8)	-3.25 (-3.69, -2.8)
35-39 years	125032.95 (82137.1, 178791.32)	136.89 (89.93, 195.74)	74144.96 (48053.44, 109126.59)	69.97 (45.35, 102.99)	-3.23 (-3.64, -2.82)
40-44 years	77423.28 (49945.41, 109197.8)	115.39 (74.44, 162.75)	51941.69 (33008.66, 76551.56)	56.75 (36.06, 83.63)	-3.29 (-3.66, -2.92)
45-49 years	53999.24 (36358.76, 76541)	104.61 (70.44, 148.28)	54515.16 (35079.48, 78663)	49.41 (31.8, 71.3)	-3.32 (-3.65, -3)
50-54 years	45788.94 (28831.57, 65793.93)	95.97 (60.43, 137.9)	54039.8 (32096, 80549.7)	44.71 (26.56, 66.65)	-3.28 (-3.56, -3)
55-59 years	35638.08 (22061.33, 50225.86)	82.17 (50.87, 115.81)	43130.39 (26369.72, 61529.64)	39.23 (23.99, 55.97)	-3.06 (-3.29, -2.83)
60-64 years	24660.18 (15298.87, 36205.16)	69.78 (43.29, 102.46)	24522.13 (14473.11, 37872.07)	33.59 (19.82, 51.88)	-2.86 (-3.02, -2.69)
65-69 years	18463.45 (11627.63, 26429.77)	67.68 (42.62, 96.88)	22859.87 (14307.25, 33111.06)	29.8 (18.65, 43.17)	-3.16 (-3.33, -2.99)
70-74 years	13475.05 (8570.21, 20237.66)	71.61 (45.54, 107.55)	14542.49 (9005.41, 21878.33)	27.29 (16.9, 41.05)	-3.75 (-3.96, -3.54)
75-79 years	7907.36 (5271.98, 11300.4)	69.48 (46.32, 99.29)	8213.65 (5241.44, 11595.28)	24.8 (15.83, 35.01)	-4.07 (-4.31, -3.83)
80-84 years	3315.57 (2153.28, 4781.46)	62.59 (40.65, 90.27)	4424.95 (2882.06, 6272.91)	22.36 (14.56, 31.69)	-4.16 (-4.43, -3.89)
85-89 years	900.98 (590.74, 1271.71)	53.41 (35.02, 75.39)	1876.29 (1230.54, 2659.57)	19.7 (12.92, 27.92)	-4.11 (-4.4, -3.82)
90-94 years	134.7 (89.52, 190.7)	43.9 (29.18, 62.15)	493.89 (327.04, 708.23)	16.84 (11.15, 24.16)	-3.96 (-4.24, -3.67)
95 plus years	15.08 (9.47, 22.32)	37.24 (23.38, 55.13)	94.62 (59.47, 136.56)	14.81 (9.3, 21.37)	-3.63 (-3.84, -3.42)

Notes: ASR: age-standardized rates; YLDs: years lived with disability; EAPCs: estimated annual percentage changes.

Table S38. Prevalence and YLDs of infectious disorders and its temporal trends from 1990, 2021 by gender, and age in China.

	1990		2021		EAPC 95%CI
	Number	ASR, per 100,000 persons	Number	ASR, per 100,000 persons	
**Prevalence**					
**Gender**					
Female	386097.62 (322866.41, 461117.32)	75.9 (63.93, 89.89)	591998.44 (476190.04, 723166.65)	75.67 (63.79, 89.64)	-0.07 (-0.16, 0.01)
Male	368644.33 (312810.8, 436274.61)	68.6 (58.12, 80.69)	524221.04 (422353.9, 641319.14)	68.35 (57.9, 80.36)	-0.08 (-0.15, -0.01)
**Age**					
<5 years	195586.36 (173694.01, 213252.18)	174.93 (155.35, 190.73)	123405.64 (108776.58, 140018.77)	158.89 (140.05, 180.28)	-0.1 (-0.18, -0.02)
5-9 years	28288.01 (16057.81, 43845.02)	27.13 (15.4, 42.05)	26905.46 (15380.96, 41158.1)	28.09 (16.06, 42.98)	0.13 (0.11, 0.14)
10-14 years	23691.64 (15019.15, 34333.46)	23.16 (14.68, 33.56)	20000.7 (12728.78, 29106.39)	23.2 (14.77, 33.77)	0.01 (0, 0.02)
15-19 years	33916.77 (22136.01, 47710.19)	26.78 (17.48, 37.67)	19917.38 (13058.34, 27963.56)	26.67 (17.49, 37.45)	-0.01 (-0.02, -0.01)
20-24 years	41898.71 (23062.62, 64760.38)	31.74 (17.47, 49.06)	23045.62 (12742.88, 35640.88)	31.49 (17.41, 48.71)	-0.02 (-0.03, -0.01)
25-29 years	40692.8 (24340.69, 62577.23)	37.03 (22.15, 56.95)	31733.44 (18873.64, 49141.13)	36.69 (21.82, 56.82)	-0.02 (-0.03, -0.02)
30-34 years	37155.7 (26126.44, 52259.83)	42.11 (29.61, 59.22)	50772.29 (35580.27, 71449.85)	41.91 (29.37, 58.97)	-0.04 (-0.05, -0.02)
35-39 years	43347.57 (30168.26, 61051.97)	47.46 (33.03, 66.84)	49978.96 (34796.43, 70527.53)	47.17 (32.84, 66.56)	-0.05 (-0.08, -0.02)
40-44 years	35530.91 (21504.64, 54528.52)	52.96 (32.05, 81.27)	48270.81 (29200.97, 73973.73)	52.74 (31.9, 80.82)	-0.05 (-0.09, -0.01)
45-49 years	33440.01 (21523.2, 50330.86)	64.78 (41.7, 97.5)	71379.5 (45882.95, 107303.24)	64.7 (41.59, 97.26)	-0.09 (-0.17, -0.01)
50-54 years	40454.81 (28353.73, 55930.61)	84.79 (59.43, 117.23)	102582.9 (71916.26, 141753.76)	84.88 (59.5, 117.29)	-0.13 (-0.27, 0.01)
55-59 years	45857.86 (30028.69, 64885.97)	105.74 (69.24, 149.61)	116513.01 (76338.49, 164940.41)	105.98 (69.43, 150.02)	-0.16 (-0.34, 0.01)
60-64 years	45207.81 (27546.64, 67421.24)	127.93 (77.95, 190.79)	93460.31 (56908, 139250.69)	128.02 (77.95, 190.74)	-0.18 (-0.37, 0.01)
65-69 years	40839.59 (26651.7, 58755.96)	149.7 (97.69, 215.37)	114779.4 (74912.28, 165118.88)	149.64 (97.66, 215.27)	-0.18 (-0.38, 0.01)
70-74 years	31665.43 (22737.13, 42409.35)	168.28 (120.83, 225.37)	89443.57 (64120.25, 119821.84)	167.82 (120.31, 224.82)	-0.17 (-0.36, 0.02)
75-79 years	21353.32 (14942.52, 29269.37)	187.63 (131.3, 257.18)	61889.96 (43282.2, 84768.17)	186.87 (130.69, 255.95)	-0.16 (-0.35, 0.02)
80-84 years	11036 (6828.83, 16182.08)	208.34 (128.92, 305.49)	41015.67 (25409.4, 60490.47)	207.24 (128.38, 305.63)	-0.15 (-0.34, 0.03)
85-89 years	3886.82 (2432.11, 5717.21)	230.42 (144.18, 338.93)	21884.24 (13703.51, 32240.42)	229.74 (143.86, 338.46)	0 (-0.18, 0.18)

90-94 years	777.56 (498.78, 1235.17)	253.42 (162.56, 402.57)	7444.16 (4777.7, 11813.87)	253.89 (162.95, 402.93)	0.26 (0.02, 0.51)
95 plus years	114.26 (57.18, 211.71)	282.17 (141.21, 522.84)	1796.48 (893.88, 3341.54)	281.1 (139.87, 522.85)	0.43 (0.11, 0.76)
**YLDs**					
**Gender**					
Female	16224.24 (9404.55, 25178.23)	3.18 (1.86, 4.91)	28693.08 (16285.3, 44211.89)	3.18 (1.84, 4.91)	-0.08 (-0.19, 0.03)
Male	14855.01 (8533.22, 23447.01)	2.82 (1.63, 4.34)	24907.33 (14341.69, 39162.22)	2.81 (1.62, 4.34)	-0.1 (-0.19, -0.01)
**Age**					
<5 years	1349.61 (630.64, 2601.13)	1.21 (0.56, 2.33)	880.49 (411.19, 1680.12)	1.13 (0.53, 2.16)	-0.07 (-0.12, -0.02)
5-9 years	866.33 (427.74, 1514.29)	0.83 (0.41, 1.45)	798.56 (400.6, 1393.69)	0.83 (0.42, 1.46)	0.03 (0, 0.05)
10-14 years	1245.86 (597.87, 2226.06)	1.22 (0.58, 2.18)	1043.99 (530.85, 1852.02)	1.21 (0.62, 2.15)	0.01 (-0.01, 0.03)
15-19 years	1881.89 (936.09, 3188.68)	1.49 (0.74, 2.52)	1109.2 (545.67, 1963.81)	1.49 (0.73, 2.63)	0 (-0.01, 0.01)
20-24 years	2332.87 (1096.62, 4256.86)	1.77 (0.83, 3.22)	1286.87 (597.33, 2302.69)	1.76 (0.82, 3.15)	-0.01 (-0.03, 0.02)
25-29 years	2276.48 (1117.9, 4041.46)	2.07 (1.02, 3.68)	1779.54 (895.69, 3248.43)	2.06 (1.04, 3.76)	-0.02 (-0.03, -0.01)
30-34 years	2074.34 (1083.65, 3506)	2.35 (1.23, 3.97)	2843.18 (1454.14, 4926.91)	2.35 (1.2, 4.07)	-0.03 (-0.05, 0)
35-39 years	2401.75 (1301.23, 4002.21)	2.63 (1.42, 4.38)	2778.7 (1458.62, 4713.15)	2.62 (1.38, 4.45)	-0.03 (-0.07, 0)
40-44 years	1955.3 (983.4, 3462.28)	2.91 (1.47, 5.16)	2659.23 (1293.14, 4846.51)	2.91 (1.41, 5.29)	-0.05 (-0.09, -0.01)
45-49 years	1834.91 (915.99, 3081.02)	3.55 (1.77, 5.97)	3922.69 (2005.69, 6610.16)	3.56 (1.82, 5.99)	-0.08 (-0.16, 0)
50-54 years	2215.7 (1147.98, 3527.97)	4.64 (2.41, 7.39)	5604.07 (2933.99, 8793.44)	4.64 (2.43, 7.28)	-0.13 (-0.27, 0)
55-59 years	2484.03 (1265.47, 4010.42)	5.73 (2.92, 9.25)	6325.52 (3241.96, 10206.55)	5.75 (2.95, 9.28)	-0.16 (-0.33, 0.01)
60-64 years	2438.4 (1090.48, 3989.92)	6.9 (3.09, 11.29)	5029.7 (2340.96, 8305.93)	6.89 (3.21, 11.38)	-0.18 (-0.37, 0.01)
65-69 years	2165.9 (997.01, 3556.9)	7.94 (3.65, 13.04)	6085.23 (2942.36, 9975.42)	7.93 (3.84, 13.01)	-0.19 (-0.38, 0.01)
70-74 years	1657.54 (856.58, 2707.17)	8.81 (4.55, 14.39)	4674.56 (2414.1, 7742.44)	8.77 (4.53, 14.53)	-0.17 (-0.36, 0.02)
75-79 years	1102.22 (571.22, 1923.07)	9.68 (5.02, 16.9)	3184.63 (1663.75, 5482.55)	9.62 (5.02, 16.55)	-0.18 (-0.36, 0.01)
80-84 years	559.61 (281.35, 1017.15)	10.56 (5.31, 19.2)	2072.29 (1050.07, 3792.79)	10.47 (5.31, 19.16)	-0.17 (-0.35, 0.02)
85-89 years	193.1 (99.8, 345.17)	11.45 (5.92, 20.46)	1079.18 (565.58, 1948.1)	11.33 (5.94, 20.45)	-0.02 (-0.2, 0.16)
90-94 years	37.95 (19.77, 70.16)	12.37 (6.44, 22.87)	358.86 (180.01, 667.29)	12.24 (6.14, 22.76)	0.23 (-0.02, 0.48)
95 plus years	5.46 (2.27, 11.76)	13.48 (5.6, 29.04)	83.93 (36.32, 174.16)	13.13 (5.68, 27.25)	0.39 (0.05, 0.72)

Notes: ASR: age-standardized rates; YLDs: years lived with disability; EAPCs: estimated annual percentage changes.

Table S39. Prevalence and YLDs of neoplasms and its temporal trends from 1990, 2021 by gender, and age in China.

	1990		2021		EAPC 95%CI
	Number	ASR, per 100,000 persons	Number	ASR, per 100,000 persons	
**Prevalence**					
**Gender**					
Female	10793635.63 (8886495.25, 13436191.18)	1935.51 (1630.68, 2323.42)	20079253.22 (17258275.36, 23109775.97)	2352.32 (2021.31, 2718.27)	0.68 (0.64, 0.71)
Male	4805457.95 (4181484.6, 5499555.7)	941.51 (824.71, 1063.27)	12731843.21 (10831091.39, 15043654.75)	1329.66 (1146.46, 1548.6)	1.22 (1.16, 1.27)
**Age**					
<5 years	353881.67 (289179.31, 420256.57)	316.51 (258.64, 375.88)	279563.47 (208444.59, 365441.17)	359.95 (268.38, 470.52)	0.79 (0.61, 0.97)
5-9 years	339814.22 (235508.69, 495262.45)	325.87 (225.85, 474.94)	294263.5 (223359.43, 390463.38)	307.26 (233.22, 407.71)	-0.36 (-0.46, -0.26)
10-14 years	468274.37 (296428.24, 722221)	457.78 (289.79, 706.04)	390226.53 (275744.6, 542566.28)	452.74 (319.92, 629.48)	-0.15 (-0.21, -0.1)
15-19 years	931921.83 (575088.97, 1480249.68)	735.74 (454.02, 1168.63)	610384.54 (411070.89, 863740.6)	817.42 (550.5, 1156.71)	0.41 (0.35, 0.47)
20-24 years	1395590.98 (817373.29, 2236847.09)	1057.26 (619.22, 1694.57)	878226.78 (575809.67, 1284803.95)	1200.19 (786.9, 1755.82)	0.58 (0.51, 0.65)
25-29 years	1475231.05 (951966.91, 2191242.21)	1342.47 (866.3, 1994.04)	1257877.42 (895432.31, 1697668.24)	1454.5 (1035.4, 1963.03)	0.39 (0.34, 0.43)
30-34 years	1464889.79 (935986.35, 2333504.12)	1660.04 (1060.68, 2644.37)	2205223.4 (1559480.81, 3127440.09)	1820.19 (1287.2, 2581.39)	0.34 (0.31, 0.37)
35-39 years	1696921.13 (1248261.44, 2367731)	1857.83 (1366.62, 2592.24)	2350227.98 (1852393.57, 3043065.72)	2217.97 (1748.15, 2871.81)	0.62 (0.56, 0.67)
40-44 years	1338634.11 (964924.11, 1948090.02)	1995.15 (1438.16, 2903.51)	2394894.18 (1856459.84, 3144931.13)	2616.41 (2028.17, 3435.82)	0.94 (0.87, 1)
45-49 years	1044732.59 (790131.76, 1401258.22)	2023.93 (1530.7, 2714.62)	2903905.55 (2322686.19, 3581906.91)	2632.22 (2105.38, 3246.79)	0.94 (0.88, 1)
50-54 years	1103881.67 (843046, 1493413.7)	2313.69 (1766.99, 3130.13)	3605915.52 (2934588.96, 4474011.82)	2983.57 (2428.11, 3701.84)	0.8 (0.72, 0.87)
55-59 years	1112082.38 (908600.87, 1355226.98)	2564.23 (2095.04, 3124.87)	3695405.19 (3134273.3, 4372405.24)	3361.22 (2850.83, 3977)	0.98 (0.92, 1.04)
60-64 years	966045.72 (790286.2, 1196745.52)	2733.77 (2236.4, 3386.62)	2901963.68 (2471070.91, 3420554.36)	3975.01 (3384.79, 4685.36)	1.37 (1.28, 1.47)
65-69 years	809177.47 (692012.8, 951138.49)	2966 (2536.54, 3486.35)	3453795.68 (3006429.3, 3981392.52)	4502.79 (3919.55, 5190.63)	1.43 (1.36, 1.5)
70-74 years	582650.34 (495982.27, 707308.27)	3096.3 (2635.73, 3758.75)	2691906.09 (2336746.95, 3125448.58)	5050.82 (4384.43, 5864.27)	1.67 (1.6, 1.75)
75-79 years	326778.65 (287703.44, 376474.62)	2871.34 (2527.99, 3308.01)	1604957.35 (1407269.71, 1825645.36)	4846.04 (4249.14, 5512.39)	1.88 (1.8, 1.96)
80-84 years	135835.29 (118086.97, 159734.02)	2564.32 (2229.27, 3015.48)	818236.66 (721368.11, 919718.85)	4134.21 (3644.77, 4646.96)	1.76 (1.67, 1.86)
85-89 years	43497.19 (37800.87, 50187.49)	2578.6 (2240.91, 2975.21)	364834.11 (321473.62, 403967.18)	3829.98 (3374.79, 4240.79)	1.38 (1.29, 1.47)

90-94 years	8209.16 (6965.36, 9702.24)	2675.54 (2270.16, 3162.17)	91970.99 (80056.38, 102837.24)	3136.81 (2730.45, 3507.42)	0.51 (0.43, 0.59)
95 plus years	1044 (816.77, 1331.96)	2578.27 (2017.11, 3289.43)	17317.8 (14305.69, 21078.67)	2709.72 (2238.42, 3298.19)	0.15 (0.03, 0.27)
**YLDs**					
**Gender**					
Female	279766.79 (197354.38, 374875.44)	60.08 (42.56, 80.23)	855070.61 (573314.92, 1169466.48)	83.14 (55.75, 114.2)	1.07 (1, 1.14)
Male	324002.54 (220470.48, 429196.85)	74.08 (50.58, 97.78)	1014644.79 (696496.81, 1390746.43)	102.25 (70.47, 138.68)	1.21 (1.14, 1.29)
**Age**					
<5 years	16504.92 (11170.79, 23693.1)	14.76 (9.99, 21.19)	12495.45 (6556.54, 21014.83)	16.09 (8.44, 27.06)	0.94 (0.61, 1.27)
5-9 years	5591.25 (3946.1, 7587.14)	5.36 (3.78, 7.28)	6283.01 (4201.01, 9105.22)	6.56 (4.39, 9.51)	0.76 (0.48, 1.04)
10-14 years	4588.81 (3235, 6219.63)	4.49 (3.16, 6.08)	5125.14 (3468.46, 7082.91)	5.95 (4.02, 8.22)	0.95 (0.76, 1.15)
15-19 years	7505.47 (5201.53, 10117.76)	5.93 (4.11, 7.99)	5599.14 (3773.69, 7818.21)	7.5 (5.05, 10.47)	0.57 (0.4, 0.74)
20-24 years	9399.09 (6399.87, 12872.69)	7.12 (4.85, 9.75)	8326.1 (5674.26, 11850.95)	11.38 (7.75, 16.2)	1.56 (1.39, 1.73)
25-29 years	12241.6 (8394.07, 16929.59)	11.14 (7.64, 15.41)	16191.17 (10993.34, 22974.29)	18.72 (12.71, 26.57)	1.86 (1.67, 2.05)
30-34 years	19285.97 (13138.84, 26352.11)	21.86 (14.89, 29.86)	41804.8 (28149.25, 59321.23)	34.51 (23.23, 48.96)	1.59 (1.4, 1.78)
35-39 years	35464.07 (24449.19, 47598.58)	38.83 (26.77, 52.11)	58430.73 (39470.88, 81356.73)	55.14 (37.25, 76.78)	0.99 (0.81, 1.18)
40-44 years	41299.53 (28393.79, 55635.2)	61.55 (42.32, 82.92)	80722.69 (54770.68, 113527.42)	88.19 (59.84, 124.03)	1.1 (0.95, 1.24)
45-49 years	42472.05 (29221.8, 56463.58)	82.28 (56.61, 109.39)	125773.57 (85167.54, 177750.44)	114.01 (77.2, 161.12)	1.23 (1.1, 1.35)
50-54 years	62009.8 (43186.73, 82422.84)	129.97 (90.52, 172.75)	201985.95 (138510.12, 279426.5)	167.13 (114.6, 231.2)	0.77 (0.65, 0.88)
55-59 years	77161.71 (53616.96, 101461.79)	177.92 (123.63, 233.95)	240852.15 (166615.84, 335281.24)	219.07 (151.55, 304.96)	0.79 (0.71, 0.87)
60-64 years	76553.54 (53327.03, 100435.34)	216.64 (150.91, 284.22)	214803.28 (151875.27, 294967.98)	294.23 (208.03, 404.04)	1.22 (1.08, 1.36)
65-69 years	72424.11 (50866.36, 95302.54)	265.47 (186.45, 349.33)	281001.5 (201578.48, 378458.68)	366.35 (262.8, 493.4)	1.14 (1.05, 1.23)
70-74 years	59582.74 (42597.34, 78192.63)	316.63 (226.37, 415.53)	246826.67 (177315.66, 332824.11)	463.12 (332.7, 624.48)	1.3 (1.22, 1.39)
75-79 years	37728.05 (27357.22, 49134.73)	331.51 (240.38, 431.74)	166194.81 (120533.81, 222115.36)	501.81 (363.94, 670.66)	1.51 (1.42, 1.61)
80-84 years	16801.84 (12030.28, 21679.03)	317.19 (227.11, 409.26)	94808.74 (68106.59, 123560.67)	479.03 (344.11, 624.3)	1.58 (1.44, 1.71)
85-89 years	5932.05 (4197.8, 7659.02)	351.66 (248.85, 454.04)	48766.41 (35027.58, 63449.95)	511.94 (367.71, 666.09)	1.3 (1.16, 1.44)
90-94 years	1117.4 (791.43, 1434.68)	364.19 (257.95, 467.59)	11964.07 (8424.64, 15517.4)	408.05 (287.34, 529.25)	0.29 (0.22, 0.36)
95 plus years	105.33 (75.13, 134.86)	260.13 (185.55, 333.06)	1760 (1219.07, 2310.14)	275.39 (190.75, 361.47)	0.04 (-0.08, 0.16)

Notes: ASR: age-standardized rates; YLDs: years lived with disability; EAPCs: estimated annual percentage changes.

Table S40. Prevalence and YLDs of neck pain and its temporal trends from 1990, 2021 by gender, and age in China.

	1990		2021		EAPC 95%CI
	Number	ASR, per 100,000 persons	Number	ASR, per 100,000 persons	
**Prevalence**					
**Gender**					
Female	14885765.8 (11515444.26, 18433543.94)	2855.52 (2245.42, 3512.27)	28277014.46 (21872002.61, 35243385.56)	2957.42 (2302.4, 3672.13)	0.16 (0.13, 0.18)
Male	11644288.93 (8887146.42, 14697208.21)	2123.91 (1673.28, 2638.36)	20100389.44 (15686712.21, 25117319.44)	2148.84 (1700.57, 2623.78)	0.07 (0.04, 0.11)
**Age**					
5-9 years	64056.48 (29766.3, 125351.92)	61.43 (28.55, 120.21)	58303.22 (27042.96, 109092.79)	60.88 (28.24, 113.91)	-0.04 (-0.05, -0.03)
10-14 years	336640.27 (173661.34, 576978.28)	329.1 (169.77, 564.05)	282645.04 (150276.47, 483850.58)	327.92 (174.35, 561.36)	-0.02 (-0.02, -0.01)
15-19 years	953500.04 (504621.96, 1614382.24)	752.77 (398.39, 1274.53)	560573.88 (303733.16, 962362.63)	750.71 (406.76, 1288.78)	-0.01 (-0.02, -0.01)
20-24 years	1855795.06 (983726.94, 3219485.39)	1405.9 (745.24, 2438.99)	1011702.23 (521935.89, 1764770.73)	1382.59 (713.28, 2411.74)	-0.05 (-0.06, -0.05)
25-29 years	2315508.73 (1194515.35, 3954363.31)	2107.13 (1087.02, 3598.49)	1712636.08 (894260.51, 2922871.17)	1980.34 (1034.04, 3379.74)	-0.21 (-0.26, -0.16)
30-34 years	2498960.53 (1483132.19, 4084380.16)	2831.87 (1680.71, 4628.49)	3060812.16 (1846927.61, 4963612.74)	2526.4 (1524.46, 4096.97)	-0.4 (-0.51, -0.3)
35-39 years	3222827.68 (1818562.22, 5208132.84)	3528.42 (1991, 5701.98)	3340169.16 (1922401.53, 5431646.2)	3152.2 (1814.22, 5125.97)	-0.4 (-0.5, -0.3)
40-44 years	2844030.74 (1644088.01, 4393434.16)	4238.85 (2450.41, 6548.14)	3745024.74 (2179859.15, 5886437.17)	4091.42 (2381.48, 6430.9)	-0.12 (-0.16, -0.08)
45-49 years	2471351.01 (1466176.09, 3962947.33)	4787.68 (2840.38, 7677.31)	5576779.87 (3328170.09, 8970203.72)	5055.02 (3016.79, 8130.96)	0.19 (0.15, 0.23)
50-54 years	2340127.86 (1354685.65, 3785599.12)	4904.81 (2839.36, 7934.46)	6589812.46 (3760951.28, 10620871.81)	5452.48 (3111.85, 8787.82)	0.39 (0.31, 0.46)
55-59 years	2233164.52 (1218152.29, 3748439.08)	5149.21 (2808.8, 8643.11)	6468478.07 (3507624.47, 10890557.74)	5883.52 (3190.42, 9905.7)	0.5 (0.41, 0.58)
60-64 years	1811544.32 (1034794.75, 2936210.14)	5126.41 (2928.32, 8309.06)	4308389.98 (2512621.63, 6925141)	5901.49 (3441.7, 9485.82)	0.57 (0.51, 0.63)
65-69 years	1400313.25 (797071.22, 2143666.33)	5132.77 (2921.62, 7857.49)	4455103.5 (2542737.54, 6914918.25)	5808.21 (3315.02, 9015.13)	0.55 (0.47, 0.64)
70-74 years	1106669.93 (595922.97, 1799741.13)	5881.02 (3166.83, 9564.11)	3391742.31 (1833159.33, 5477321.39)	6363.92 (3439.55, 10277.09)	0.43 (0.27, 0.58)
75-79 years	683012.44 (380504.47, 1138486.99)	6001.5 (3343.42, 10003.67)	2064600.22 (1138560.37, 3374725.82)	6233.9 (3437.79, 10189.72)	0.27 (0.1, 0.43)
80-84 years	298069.43 (182607.16, 480193.15)	5627 (3447.29, 9065.16)	1144752.24 (705459.18, 1859672.4)	5783.96 (3564.39, 9396.15)	0.21 (0.08, 0.33)
85-89 years	80894.11 (49618.95, 130722.63)	4795.56 (2941.51, 7749.49)	464272.54 (289646.02, 745816)	4873.87 (3040.66, 7829.47)	0.11 (0.05, 0.18)
90-94 years	12138.52 (6909.6, 20179.75)	3956.21 (2251.99, 6577.02)	118416.23 (67267.48, 198955.4)	4038.77 (2294.26, 6785.68)	0.09 (0.08, 0.11)

95 plus years	1449.83 (795.34, 2538.38)	3580.52 (1964.18, 6268.83)	23189.96 (12745.46, 40617.53)	3628.54 (1994.29, 6355.44)	0.04 (0.02, 0.06)
**YLDs**					
**Gender**					
Female	1490376.44 (977310.71, 2174460.7)	284.09 (188.02, 405.92)	2793902.52 (1844448.93, 3931333.74)	294.19 (193.52, 414.36)	0.16 (0.13, 0.18)
Male	1184795.7 (774026.15, 1719942.69)	214.03 (142.11, 307.82)	2013690.87 (1329395.62, 2863368.9)	216.09 (144.9, 307.55)	0.07 (0.03, 0.1)
**Age**					
5-9 years	6876.19 (2743.31, 14537.37)	6.59 (2.63, 13.94)	6268.9 (2584.52, 12930.26)	6.55 (2.7, 13.5)	-0.04 (-0.04, -0.03)
10-14 years	35514.44 (16126.87, 64502.08)	34.72 (15.77, 63.06)	29960.2 (13629.44, 54109.87)	34.76 (15.81, 62.78)	0 (0, 0.01)
15-19 years	100100.16 (45007.35, 192092.62)	79.03 (35.53, 151.65)	58895.83 (25993.36, 112157.03)	78.87 (34.81, 150.2)	-0.01 (-0.01, 0)
20-24 years	193641.54 (94163.17, 371066.56)	146.7 (71.34, 281.11)	105861.47 (50686.71, 196909.07)	144.67 (69.27, 269.1)	-0.04 (-0.05, -0.04)
25-29 years	239523.73 (109155.46, 435146.59)	217.97 (99.33, 395.99)	177731.47 (82958.8, 324622.43)	205.51 (95.93, 375.36)	-0.19 (-0.24, -0.15)
30-34 years	257105.94 (134848.78, 454179.89)	291.36 (152.81, 514.68)	316078.87 (166883.02, 539419.87)	260.89 (137.75, 445.24)	-0.39 (-0.49, -0.29)
35-39 years	329373.84 (158632.63, 583108.07)	360.61 (173.67, 638.4)	342657.88 (170680.58, 615583.03)	323.37 (161.08, 580.94)	-0.38 (-0.48, -0.29)
40-44 years	289341.14 (153349.39, 469258.82)	431.25 (228.56, 699.4)	382259.5 (205632.28, 628724.73)	417.62 (224.65, 686.88)	-0.11 (-0.15, -0.06)
45-49 years	250302.41 (134762.56, 447954.16)	484.9 (261.07, 867.81)	565371.45 (298102.35, 1004537.1)	512.48 (270.21, 910.55)	0.2 (0.15, 0.24)
50-54 years	235992.53 (124378.56, 433846.43)	494.63 (260.69, 909.32)	664033.5 (341390.54, 1224700.4)	549.43 (282.47, 1013.33)	0.38 (0.31, 0.46)
55-59 years	222249.23 (112365.57, 412079.95)	512.46 (259.09, 950.17)	642928.62 (328104.28, 1187967.43)	584.79 (298.43, 1080.54)	0.49 (0.41, 0.58)
60-64 years	177322.01 (92516.3, 302417.63)	501.8 (261.81, 855.8)	421012.92 (223762.76, 713357.74)	576.69 (306.5, 977.13)	0.56 (0.5, 0.63)
65-69 years	134923.61 (70987.35, 217247.04)	494.56 (260.2, 796.31)	428426.82 (227601.53, 681013.79)	558.55 (296.73, 887.85)	0.55 (0.46, 0.63)
70-74 years	104295.35 (52524.64, 174299.81)	554.24 (279.12, 926.26)	319311.51 (163442.85, 533015.07)	599.12 (306.67, 1000.1)	0.42 (0.27, 0.58)
75-79 years	63134.76 (33155.55, 106189.83)	554.75 (291.33, 933.07)	190398.15 (100071.04, 316560.05)	574.89 (302.16, 955.83)	0.26 (0.1, 0.42)
80-84 years	27083.64 (14131.71, 46723.57)	511.29 (266.78, 882.06)	103412.37 (56188.16, 176207.56)	522.5 (283.9, 890.3)	0.19 (0.06, 0.31)
85-89 years	7210.86 (3841.27, 12250.74)	427.47 (227.72, 726.25)	40901.96 (21689.41, 68458.62)	429.38 (227.69, 718.67)	0.08 (0.02, 0.15)
90-94 years	1057.93 (566.77, 1801.02)	344.8 (184.72, 586.99)	10158.25 (5470.65, 16938.38)	346.46 (186.58, 577.71)	0.06 (0.04, 0.08)
95 plus years	122.84 (66.84, 217.22)	303.36 (165.07, 536.46)	1923.71 (1053.65, 3411.43)	301 (164.87, 533.79)	0 (-0.02, 0.01)

Notes: ASR: age-standardized rates; YLDs: years lived with disability; EAPCs: estimated annual percentage changes.

Table S41. Prevalence and YLDs of low back pain and its temporal trends from 1990, 2021 by gender, and age in China.

	1990		2021		EAPC 95%CI
	Number	ASR, per 100,000 persons	Number	ASR, per 100,000 persons	
**Prevalence**					
**Gender**					
Female	41762395.25 (36226090.34, 47518758.14)	8247.28 (7164.44, 9292.73)	60945208.4 (52941430.71, 68505910.77)	6381.38 (5567.92, 7153.22)	-0.54 (-0.68, -0.41)
Male	26518611.26 (22932827.52, 30437569.16)	5007.6 (4329.54, 5663.31)	39148537.2 (33850360.66, 44326366.95)	4282.3 (3759.62, 4838.55)	-0.43 (-0.5, -0.36)
**Age**					
5-9 years	278572.3 (140844.83, 477006.59)	267.14 (135.07, 457.44)	252727.01 (125993.46, 431728.48)	263.89 (131.56, 450.8)	-0.22 (-0.29, -0.14)
10-14 years	1411538.1 (1012509.65, 1936381.89)	1379.9 (989.82, 1892.99)	1209757.3 (876063.74, 1632868.48)	1403.55 (1016.4, 1894.43)	-0.17 (-0.25, -0.1)
15-19 years	3431844.08 (2427009.15, 4598299.08)	2709.38 (1916.08, 3630.28)	2085247.22 (1482449.81, 2768510.37)	2792.54 (1985.28, 3707.55)	-0.12 (-0.26, 0.01)
20-24 years	4536053.23 (3142204.06, 6309559.75)	3436.38 (2380.44, 4779.94)	2450135.58 (1692860.96, 3377771.14)	3348.36 (2313.47, 4616.07)	-0.14 (-0.41, 0.13)
25-29 years	4661032.38 (3238028.62, 6389119.13)	4241.56 (2946.62, 5814.13)	2997352.77 (2098087.2, 4091804.08)	3465.87 (2426.04, 4731.39)	-0.42 (-0.71, -0.12)
30-34 years	4894704.53 (3451084.54, 6657695.58)	5546.76 (3910.83, 7544.62)	4704681.85 (3348836.7, 6412582.83)	3883.25 (2764.13, 5292.95)	-0.73 (-0.94, -0.52)
35-39 years	6823014.86 (4934305.14, 9099534.96)	7469.99 (5402.19, 9962.37)	5636488.34 (4112130.82, 7466522.45)	5319.29 (3880.71, 7046.33)	-0.71 (-0.86, -0.56)
40-44 years	6213687.71 (4442159.85, 8414809.85)	9261.12 (6620.77, 12541.76)	6521865.4 (4660484.11, 8860950.31)	7125.1 (5091.55, 9680.53)	-0.57 (-0.68, -0.46)
45-49 years	5592766.43 (3972264.5, 7609312.52)	10834.71 (7695.36, 14741.31)	9572816.79 (6905685.1, 13115211.41)	8677.19 (6259.6, 11888.17)	-0.51 (-0.6, -0.42)
50-54 years	6064709.83 (4261444.52, 8393949.16)	12711.38 (8931.81, 17593.37)	12367108 (8760649.56, 16826835.79)	10232.67 (7248.65, 13922.7)	-0.52 (-0.6, -0.43)
55-59 years	6155377.6 (4399012.42, 8389102.79)	14193 (10143.19, 19343.5)	12562313.63 (8993975.71, 16993052.54)	11426.27 (8180.63, 15456.33)	-0.52 (-0.6, -0.43)
60-64 years	5603375.4 (3961672.42, 7580389.14)	15856.76 (11210.97, 21451.43)	9413798.06 (6684880.31, 12728370.72)	12894.7 (9156.72, 17434.89)	-0.48 (-0.55, -0.4)
65-69 years	4845002.45 (3522142.24, 6585621.21)	17759.09 (12910.22, 24139.24)	10878645.6 (7968522.89, 14596788.81)	14182.72 (10388.73, 19030.14)	-0.5 (-0.59, -0.42)
70-74 years	3684034.4 (2624802.02, 4910222.36)	19577.55 (13948.62, 26093.71)	7957404.29 (5689804.03, 10612383.04)	14930.46 (10675.77, 19911.99)	-0.58 (-0.69, -0.47)
75-79 years	2459197.54 (1793753.61, 3314664.66)	21608.5 (15761.37, 29125.32)	5461185.7 (3975489.34, 7266728.29)	16489.62 (12003.67, 21941.31)	-0.59 (-0.7, -0.48)
80-84 years	1178859.34 (875968.8, 1539578.61)	22254.7 (16536.68, 29064.42)	3537877.25 (2630934.61, 4680686.8)	17875.42 (13293.02, 23649.56)	-0.5 (-0.58, -0.43)
85-89 years	377214.63 (267834.7, 515266)	22362 (15877.75, 30545.95)	1828220.3 (1304727.93, 2468064.97)	19192.4 (13696.85, 25909.4)	-0.37 (-0.41, -0.33)
90-94 years	62451.57 (46131.93, 82880.52)	20354.33 (15035.4, 27012.57)	544514.38 (394243.41, 723507.71)	18571.51 (13446.29, 24676.36)	-0.26 (-0.28, -0.23)

95 plus years	7570.14 (5253.69, 10344.35)	18695.36 (12974.61, 25546.59)	111606.14 (77292.89, 154041.08)	17463.06 (12094.05, 24102.87)	-0.18 (-0.22, -0.15)
**YLDs**					
**Gender**					
Female	4717264.2 (3360731.09, 6387684.31)	925.83 (657.88, 1253.35)	6823284.3 (4775816.16, 9218929.38)	716.15 (506.26, 959.84)	-0.54 (-0.67, -0.41)
Male	3055693.59 (2153539.73, 4136119.18)	571.73 (402.17, 779.32)	4474520.56 (3156562.37, 6067442.17)	488.36 (346.52, 657.58)	-0.43 (-0.5, -0.36)
**Age**					
5-9 years	30605.44 (14124.02, 55003.47)	29.35 (13.54, 52.75)	27845.57 (12994.21, 49689.34)	29.08 (13.57, 51.88)	-0.2 (-0.27, -0.13)
10-14 years	154166.52 (95277.02, 236755.96)	150.71 (93.14, 231.45)	132462.93 (82074.77, 200396.39)	153.68 (95.22, 232.5)	-0.16 (-0.24, -0.09)
15-19 years	381361.97 (224508.61, 562829.82)	301.08 (177.25, 444.34)	232338.39 (136512.04, 342687.35)	311.14 (182.82, 458.92)	-0.11 (-0.25, 0.02)
20-24 years	517748.54 (305317.63, 801587.14)	392.23 (231.3, 607.26)	280994.08 (168516.13, 438353.96)	384.01 (230.29, 599.06)	-0.12 (-0.39, 0.15)
25-29 years	542686.33 (316825.73, 832398.87)	493.85 (288.31, 757.49)	350578.88 (206109.33, 548140.11)	405.38 (238.33, 633.82)	-0.4 (-0.69, -0.1)
30-34 years	572373.7 (334215.69, 862516.41)	648.62 (378.74, 977.42)	552193.5 (324764.91, 848229.14)	455.78 (268.06, 700.13)	-0.72 (-0.92, -0.51)
35-39 years	796862.17 (489937.62, 1194799.29)	872.42 (536.39, 1308.09)	661783.27 (406723.16, 985896.05)	624.54 (383.83, 930.41)	-0.69 (-0.84, -0.55)
40-44 years	720876.36 (439973.49, 1088196.96)	1074.42 (655.75, 1621.89)	760758.88 (467496.27, 1136826.44)	831.12 (510.74, 1241.98)	-0.55 (-0.66, -0.45)
45-49 years	650702.73 (399983, 999739.14)	1260.59 (774.88, 1936.77)	1117478.76 (690092.38, 1705640.73)	1012.93 (625.53, 1546.06)	-0.5 (-0.59, -0.41)
50-54 years	701532.92 (426643.78, 1054697.11)	1470.38 (894.23, 2210.6)	1431766.12 (882140.93, 2161134.07)	1184.66 (729.89, 1788.14)	-0.51 (-0.59, -0.43)
55-59 years	706459.71 (437755.81, 1049288.73)	1628.95 (1009.37, 2419.44)	1440541.07 (880782.68, 2157602.62)	1310.27 (801.13, 1962.49)	-0.52 (-0.6, -0.44)
60-64 years	634759.86 (388529.51, 960526.96)	1796.28 (1099.48, 2718.15)	1064877.02 (662801.62, 1587317.48)	1458.63 (907.88, 2174.25)	-0.48 (-0.55, -0.41)
65-69 years	538788.69 (323712.56, 812833.93)	1974.9 (1186.55, 2979.4)	1208478.46 (730931.07, 1802687.42)	1575.52 (952.93, 2350.2)	-0.51 (-0.59, -0.42)
70-74 years	400802.86 (253563.33, 594266.59)	2129.93 (1347.48, 3158.03)	864659.62 (547654.54, 1277692.05)	1622.36 (1027.56, 2397.33)	-0.58 (-0.69, -0.47)
75-79 years	259314.11 (160168.04, 382709.96)	2278.54 (1407.37, 3362.8)	574602.91 (357034.63, 837523.32)	1734.97 (1078.04, 2528.84)	-0.6 (-0.7, -0.49)
80-84 years	120352.71 (78934.55, 176224.56)	2272.04 (1490.14, 3326.8)	359020.94 (234189.12, 524566.23)	1813.98 (1183.26, 2650.41)	-0.52 (-0.59, -0.45)
85-89 years	37023.83 (24356.8, 56557.97)	2194.84 (1443.92, 3352.86)	177383.22 (116754.1, 270071.68)	1862.14 (1225.67, 2835.17)	-0.4 (-0.44, -0.36)
90-94 years	5858.97 (3752.6, 8246.7)	1909.56 (1223.05, 2687.78)	50232.33 (32631.51, 70415.76)	1713.25 (1112.95, 2401.64)	-0.29 (-0.33, -0.26)
95 plus years	680.36 (421.14, 1008.3)	1680.24 (1040.05, 2490.11)	9808.9 (6124.99, 14328.48)	1534.8 (958.38, 2241.98)	-0.24 (-0.28, -0.2)

Notes: ASR: age-standardized rates; YLDs: years lived with disability; EAPCs: estimated annual percentage changes.

Table S42. Prevalence and YLDs of hip osteoarthritis and its temporal trends from 1990, 2021 by gender, and age in China.

	1990		2021		EAPC 95%CI
	Number	ASR, per 100,000 persons	Number	ASR, per 100,000 persons	
**Prevalence**					
**Gender**					
Female	787353.94 (605332.65, 1011252.81)	183.58 (141.47, 234.35)	2532082.19 (1940614.38, 3273771.14)	233.39 (180.52, 300.48)	0.97 (0.87, 1.08)
Male	932918.81 (708345.97, 1200664.6)	223.53 (172.18, 287.28)	2937431.94 (2241383.63, 3810535.53)	289.38 (222.37, 372.44)	0.92 (0.85, 0.98)
**Age**					
30-34 years	61052.96 (35349.2, 96808.21)	69.19 (40.06, 109.7)	96086.8 (56247.83, 151136.72)	79.31 (46.43, 124.75)	0.44 (0.37, 0.5)
35-39 years	102185.06 (62355.79, 149887.39)	111.87 (68.27, 164.1)	139178.93 (87921.52, 203049.71)	131.35 (82.97, 191.62)	0.5 (0.44, 0.57)
40-44 years	117087.08 (72242.48, 176917.69)	174.51 (107.67, 263.68)	192776.91 (121902.27, 288777.81)	210.61 (133.18, 315.49)	0.6 (0.53, 0.67)
45-49 years	136337.03 (89259, 188654.95)	264.12 (172.92, 365.48)	360872.95 (233249.05, 496188.67)	327.11 (211.43, 449.77)	0.72 (0.65, 0.8)
50-54 years	181721.37 (119312.62, 260307.74)	380.88 (250.07, 545.59)	583322.01 (384966.66, 828846.6)	482.65 (318.53, 685.8)	0.84 (0.76, 0.92)
55-59 years	224019.66 (156526.86, 310361.33)	516.54 (360.92, 715.63)	728539.01 (504607.88, 1005972.84)	662.66 (458.97, 915)	0.93 (0.84, 1.02)
60-64 years	236178.66 (170498.09, 314677.1)	668.35 (482.49, 890.49)	633914.12 (454884.25, 843965.77)	868.31 (623.09, 1156.04)	1 (0.91, 1.09)
65-69 years	227794.64 (167843.3, 303416.35)	834.97 (615.22, 1112.16)	839216.27 (615834.63, 1115556.52)	1094.1 (802.88, 1454.37)	1.05 (0.95, 1.14)
70-74 years	190653.19 (141859.53, 253782.42)	1013.16 (753.86, 1348.64)	712473.15 (528252.67, 947896.12)	1336.81 (991.16, 1778.54)	1.09 (0.99, 1.18)
75-79 years	135753.71 (101609.91, 179449.17)	1192.84 (892.83, 1576.79)	523558.61 (391054.7, 689329.67)	1580.84 (1180.76, 2081.38)	1.11 (1.02, 1.21)
80-84 years	73238.09 (53963.71, 97301.31)	1382.6 (1018.74, 1836.87)	362137.3 (268750.19, 483276.76)	1829.73 (1357.88, 2441.8)	1.11 (1.02, 1.21)
85-89 years	27316.42 (20034.32, 36261.27)	1619.37 (1187.67, 2149.64)	203313.11 (149240.49, 270288.82)	2134.35 (1566.71, 2837.45)	1.1 (1.01, 1.19)
90-94 years	5951.42 (4454.42, 7903.55)	1939.7 (1451.79, 2575.94)	74244.81 (55781.14, 97723.28)	2532.23 (1902.5, 3333)	1.05 (0.96, 1.15)
95 plus years	983.48 (711.06, 1303.67)	2428.82 (1756.06, 3219.58)	19880.14 (14327.94, 26291.8)	3110.65 (2241.9, 4113.89)	0.95 (0.87, 1.03)
**YLDs**					
**Gender**					
Female	25427.37 (11808.09, 50837.51)	5.87 (2.7, 11.74)	80779.06 (37566.26, 161224.8)	7.45 (3.48, 14.85)	0.96 (0.86, 1.07)
Male	30612.06 (14082.02, 61980.79)	7.24 (3.34, 14.5)	95142.97 (43485.18, 190673.21)	9.33 (4.28, 18.69)	0.9 (0.84, 0.97)

**Age**					
30-34 years	2115.62 (815.95, 4438.89)	2.4 (0.92, 5.03)	3336.98 (1268.12, 7002.33)	2.75 (1.05, 5.78)	0.44 (0.38, 0.5)
35-39 years	3477.34 (1344.55, 7390.71)	3.81 (1.47, 8.09)	4742.16 (1911.22, 10161.65)	4.48 (1.8, 9.59)	0.49 (0.43, 0.55)
40-44 years	3957.64 (1588.64, 8465.86)	5.9 (2.37, 12.62)	6509.87 (2701.73, 14110)	7.11 (2.95, 15.42)	0.61 (0.54, 0.68)
45-49 years	4552.32 (1981.42, 9372.79)	8.82 (3.84, 18.16)	12069.33 (5205.05, 25323.15)	10.94 (4.72, 22.95)	0.73 (0.66, 0.8)
50-54 years	6050.8 (2537.04, 12512.38)	12.68 (5.32, 26.23)	19370.31 (7849.11, 41125.76)	16.03 (6.49, 34.03)	0.83 (0.75, 0.91)
55-59 years	7378.67 (3233.06, 15082.79)	17.01 (7.45, 34.78)	23918.77 (10644.5, 48335.59)	21.76 (9.68, 43.96)	0.92 (0.84, 1)
60-64 years	7693.85 (3596.52, 15598.76)	21.77 (10.18, 44.14)	20589.35 (9650.4, 41803.34)	28.2 (13.22, 57.26)	0.99 (0.9, 1.08)
65-69 years	7322.86 (3403.7, 15060.68)	26.84 (12.48, 55.2)	26933.98 (12490.91, 56036.35)	35.11 (16.28, 73.06)	1.04 (0.95, 1.14)
70-74 years	6027.1 (2699.04, 12423.5)	32.03 (14.34, 66.02)	22514.56 (10171.92, 46603.13)	42.24 (19.09, 87.44)	1.08 (0.99, 1.17)
75-79 years	4215.66 (1963.2, 8426.77)	37.04 (17.25, 74.04)	16232.91 (7536.66, 32529.31)	49.01 (22.76, 98.22)	1.11 (1.01, 1.2)
80-84 years	2228.95 (1064.4, 4483.15)	42.08 (20.09, 84.63)	10979.64 (5152.15, 22227.76)	55.48 (26.03, 112.31)	1.1 (1.01, 1.2)
85-89 years	816.06 (385.96, 1641.23)	48.38 (22.88, 97.29)	6018.01 (2833.36, 12045.52)	63.18 (29.74, 126.45)	1.08 (0.98, 1.17)
90-94 years	174.47 (81.78, 348.35)	56.86 (26.65, 113.53)	2148.68 (1010.46, 4242.43)	73.28 (34.46, 144.69)	1.03 (0.93, 1.12)
95 plus years	28.1 (13.03, 56.59)	69.39 (32.18, 139.77)	557.48 (263.62, 1123.57)	87.23 (41.25, 175.81)	0.9 (0.82, 0.99)

Notes: ASR: age-standardized rates; YLDs: years lived with disability; EAPCs: estimated annual percentage changes.

Table S43. Prevalence and YLDs of knee osteoarthritis and its temporal trends from 1990, 2021 by gender, and age in China.

	1990		2021		EAPC 95%CI
	Number	ASR, per 100,000 persons	Number	ASR, per 100,000 persons	
**Prevalence**					
**Gender**					
Female	25828310.27 (21907784.27, 29658343.35)	5863.22 (5016.52, 6725.58)	70294935.85 (59713904.72, 80953334.68)	6302.93 (5378.56, 7213.7)	0.5 (0.39, 0.61)
Male	15215698.89 (12720237.75, 17663215.11)	3419.35 (2916.18, 3948.18)	39280536.59 (33096647.86, 45575847)	3661.85 (3106.11, 4228.87)	0.48 (0.35, 0.6)
**Age**					
30-34 years	108868.25 (78434.6, 146715.36)	123.37 (88.88, 166.26)	161366.73 (116562.41, 218239.53)	133.19 (96.21, 180.14)	0.52 (0.29, 0.75)
35-39 years	1083157.23 (773447.53, 1460202.76)	1185.86 (846.79, 1598.66)	1341055.4 (955240.32, 1806339.33)	1265.59 (901.48, 1704.68)	0.54 (0.3, 0.79)
40-44 years	2412842.04 (1885587.5, 3066208.75)	3596.19 (2810.35, 4570)	3519608.13 (2750428.16, 4467340.41)	3845.15 (3004.83, 4880.54)	0.59 (0.37, 0.82)
45-49 years	4114401.63 (3339955.93, 5003696.93)	7970.72 (6470.41, 9693.53)	9454876.19 (7645245.85, 11457544.97)	8570.29 (6929.97, 10385.59)	0.61 (0.43, 0.79)
50-54 years	6237450.11 (4834768.54, 7783220.96)	13073.43 (10133.47, 16313.31)	17040192.42 (13131899.12, 21281444.91)	14099.23 (10865.47, 17608.49)	0.58 (0.41, 0.74)
55-59 years	7161002.6 (5762991.02, 8610187.19)	16511.76 (13288.24, 19853.28)	19677851.57 (16008379.87, 23497974.16)	17898.33 (14560.7, 21372.99)	0.57 (0.43, 0.71)
60-64 years	6502955.7 (5317232.04, 7715185.69)	18402.44 (15047.01, 21832.88)	14432022.41 (11772794.63, 17165867.37)	19768.49 (16125.97, 23513.22)	0.57 (0.43, 0.71)
65-69 years	5346302.34 (4450617.99, 6391546.1)	19596.58 (16313.5, 23427.87)	16249609.32 (13521407.9, 19343691.66)	21184.96 (17628.15, 25218.78)	0.51 (0.38, 0.63)
70-74 years	3867530.04 (3257531.83, 4693532.92)	20552.67 (17311.04, 24942.18)	11717344.49 (9898645.06, 14208888.23)	21985.23 (18572.81, 26660.11)	0.37 (0.26, 0.49)
75-79 years	2483465.97 (2119946.34, 2978371.14)	21821.74 (18627.56, 26170.38)	7711071.51 (6549433.24, 9211828.51)	23282.97 (19775.49, 27814.38)	0.27 (0.16, 0.38)
80-84 years	1234731.55 (1047109.18, 1460073.53)	23309.46 (19767.5, 27563.51)	4908767.86 (4152612.57, 5807410.29)	24801.96 (20981.42, 29342.42)	0.25 (0.17, 0.33)
85-89 years	407426.23 (343613.01, 481311.82)	24153 (20370.03, 28533.08)	2441895.33 (2068055.28, 2876512.88)	25634.68 (21710.15, 30197.23)	0.29 (0.23, 0.36)
90-94 years	74158.81 (63266.8, 87444.45)	24169.97 (20620.03, 28500.05)	757123.22 (642836.9, 894619.65)	25822.87 (21924.95, 30512.4)	0.37 (0.29, 0.46)
95 plus years	9716.67 (8219.28, 11422.53)	23996.46 (20298.48, 28209.3)	162687.85 (137205.29, 191981.64)	25455.84 (21468.57, 30039.45)	0.42 (0.3, 0.54)
**YLDs**					
**Gender**					
Female	837866.8 (403306.58, 1618226.26)	189.3 (91.27, 365.09)	2269290.85 (1097550.01, 4397449.61)	203.49 (98.22, 395.51)	0.5 (0.39, 0.61)
Male	501699.49 (238695.84, 967197.92)	111.82 (53.84, 215.06)	1284862.58 (614830.46, 2467313.99)	119.45 (57.51, 229.15)	0.47 (0.35, 0.6)

**Age**					
30-34 years	3731.18 (1599.53, 7876.46)	4.23 (1.81, 8.93)	5521.57 (2458.24, 11921.88)	4.56 (2.03, 9.84)	0.53 (0.3, 0.76)
35-39 years	36710.42 (16699.87, 76844.24)	40.19 (18.28, 84.13)	45647.85 (20982.54, 97420.82)	43.08 (19.8, 91.94)	0.56 (0.32, 0.81)
40-44 years	81373.83 (38579.8, 170220.9)	121.28 (57.5, 253.7)	119084.74 (56542.66, 245807.83)	130.1 (61.77, 268.54)	0.61 (0.38, 0.83)
45-49 years	137754.28 (65886.3, 268975.98)	266.87 (127.64, 521.08)	317138.43 (150194.93, 617301.93)	287.47 (136.14, 559.55)	0.62 (0.43, 0.8)
50-54 years	207542.59 (95754.78, 418467.05)	435 (200.7, 877.09)	567014 (261406.3, 1139702.43)	469.15 (216.29, 943)	0.58 (0.42, 0.74)
55-59 years	236260.1 (110242.92, 456065.02)	544.77 (254.2, 1051.59)	648485.44 (303339, 1251946.71)	589.84 (275.91, 1138.73)	0.57 (0.43, 0.71)
60-64 years	212163.83 (101421.17, 411718)	600.39 (287.01, 1165.1)	470508.51 (225311.87, 910808.94)	644.49 (308.62, 1247.6)	0.57 (0.43, 0.71)
65-69 years	172026.11 (83025.33, 331271.2)	630.55 (304.32, 1214.26)	522506.15 (249900.88, 1011856.28)	681.2 (325.8, 1319.18)	0.51 (0.38, 0.63)
70-74 years	122357.13 (59402.48, 246810.35)	650.23 (315.67, 1311.59)	370643.22 (180132.86, 751431.66)	695.44 (337.98, 1409.91)	0.37 (0.25, 0.48)
75-79 years	77290.88 (37332.87, 158292.12)	679.14 (328.04, 1390.88)	239641.2 (115325.35, 489143.37)	723.58 (348.22, 1476.93)	0.26 (0.15, 0.38)
80-84 years	37701.96 (18175.76, 74956.99)	711.74 (343.13, 1415.05)	149152.39 (72043.98, 296483.24)	753.6 (364.01, 1498.01)	0.23 (0.16, 0.31)
85-89 years	12200.48 (6050.72, 24296.49)	723.27 (358.7, 1440.34)	72379 (35845.5, 144566.85)	759.82 (376.3, 1517.64)	0.27 (0.21, 0.33)
90-94 years	2175.67 (1114.35, 4363.63)	709.1 (363.19, 1422.2)	21876.72 (11255.42, 43958.28)	746.14 (383.88, 1499.27)	0.34 (0.25, 0.43)
95 plus years	277.84 (141.01, 565.6)	686.16 (348.23, 1396.81)	4554.21 (2318.57, 9191.52)	712.6 (362.79, 1438.2)	0.37 (0.24, 0.5)

Notes: ASR: age-standardized rates; YLDs: years lived with disability; EAPCs: estimated annual percentage changes.

Table S44. Prevalence and YLDs of rheumatoid arthritis and its temporal trends from 1990, 2021 by gender, and age in China.

	1990		2021		
	Number	ASR, per 100,000 persons	Number	ASR, per 100,000 persons	EAPC 95%CI
**Prevalence**					
**Gender**					
Female	1413054.56 (1210838.34, 1646843.93)	286.22 (247.9, 332.48)	3196696.63 (2798213.26, 3652420.59)	321.69 (280.04, 369.68)	0.47 (0.43, 0.52)
Male	628627.24 (530870.08, 752446.44)	127.99 (109.84, 150.14)	1558789.9 (1345203.43, 1823436)	160.76 (139.42, 187.51)	0.76 (0.74, 0.79)
**Age**					
5-9 years	1211.29 (446.01, 2266.17)	1.16 (0.43, 2.17)	1465.26 (620.96, 2653.56)	1.53 (0.65, 2.77)	0.93 (0.89, 0.97)
10-14 years	9255.3 (5156.78, 14873.49)	9.05 (5.04, 14.54)	10381.69 (6003.85, 15928.85)	12.04 (6.97, 18.48)	0.98 (0.94, 1.01)
15-19 years	36426.53 (22721.61, 52704.86)	28.76 (17.94, 41.61)	29006.63 (18760.15, 41711.79)	38.85 (25.12, 55.86)	1.02 (0.98, 1.05)
20-24 years	80008.53 (51265.23, 115128.67)	60.61 (38.84, 87.22)	59451.26 (39420.52, 84147.27)	81.25 (53.87, 115)	1.02 (0.97, 1.06)
25-29 years	113415.26 (78048.39, 154636.62)	103.21 (71.02, 140.72)	114729.13 (80540.52, 154534.68)	132.66 (93.13, 178.69)	0.92 (0.87, 0.97)
30-34 years	136628.95 (99796.95, 190327.58)	154.83 (113.09, 215.68)	232769.91 (172643.83, 313208.93)	192.13 (142.5, 258.52)	0.79 (0.75, 0.84)
35-39 years	200056.78 (148674.88, 264389.15)	219.03 (162.77, 289.46)	277653.64 (209827, 360767.72)	262.03 (198.02, 340.46)	0.7 (0.65, 0.74)
40-44 years	195358.73 (143973.82, 248012.86)	291.17 (214.58, 369.65)	311800.65 (238034.58, 390604.63)	340.64 (260.05, 426.73)	0.62 (0.57, 0.67)
45-49 years	189268.24 (144193.52, 238186.97)	366.66 (279.34, 461.43)	464226.33 (360408.37, 577100.14)	420.79 (326.69, 523.11)	0.54 (0.49, 0.6)
50-54 years	210647.14 (167544.79, 257014.73)	441.51 (351.17, 538.69)	602086.94 (481446.88, 727380.84)	498.17 (398.35, 601.84)	0.48 (0.43, 0.52)
55-59 years	224592.73 (186065.78, 270255.56)	517.86 (429.03, 623.15)	639850.96 (534332.28, 763800.52)	581.99 (486.01, 694.73)	0.43 (0.39, 0.47)
60-64 years	210122.92 (176629.01, 251274.45)	594.62 (499.84, 711.07)	482369.67 (408546.23, 571105.92)	660.73 (559.61, 782.28)	0.41 (0.37, 0.45)
65-69 years	179267.77 (150911.21, 213846.7)	657.1 (553.16, 783.84)	565534.43 (482986.55, 667377.2)	737.3 (629.68, 870.07)	0.46 (0.42, 0.5)
70-74 years	127251.79 (107248.92, 150678.56)	676.24 (569.94, 800.73)	415311.69 (351430.74, 487196.48)	779.25 (659.39, 914.13)	0.54 (0.5, 0.59)
75-79 years	78385.28 (67008.45, 91670.41)	688.76 (588.79, 805.49)	273856.59 (235669.49, 317184.85)	826.89 (711.59, 957.71)	0.63 (0.6, 0.65)
80-84 years	36659.57 (31809.79, 42299.51)	692.07 (600.51, 798.54)	169763.66 (148861.11, 194021.58)	857.75 (752.13, 980.31)	0.72 (0.71, 0.73)
85-89 years	11129.98 (9507.57, 12945.38)	659.81 (563.63, 767.43)	79419.41 (69319.06, 91525.99)	833.73 (727.7, 960.83)	0.78 (0.75, 0.82)
90-94 years	1804.21 (1528.5, 2123.29)	588.03 (498.17, 692.03)	21875.82 (18849.53, 25439.7)	746.11 (642.89, 867.66)	0.79 (0.75, 0.84)

95 plus years	190.83 (155.21, 231.64)	471.27 (383.32, 572.06)	3932.85 (3318.73, 4636.04)	615.38 (519.28, 725.4)	0.83 (0.78, 0.88)
**YLDs**					
**Gender**					
Female	189324.33 (127627.24, 271689.23)	38.04 (25.9, 53.78)	423189.64 (289660.99, 594249.36)	42.89 (29.15, 60.32)	0.48 (0.44, 0.53)
Male	85910.8 (56169.84, 122962.77)	17.22 (11.42, 24.25)	208624.18 (140237.39, 295526.61)	21.57 (14.47, 30.69)	0.76 (0.73, 0.78)
**Age**					
5-9 years	177.75 (62.56, 382.15)	0.17 (0.06, 0.37)	215.06 (82.49, 455.66)	0.22 (0.09, 0.48)	0.93 (0.89, 0.97)
10-14 years	1359.84 (669.81, 2435.4)	1.33 (0.65, 2.38)	1525.58 (791.78, 2729.5)	1.77 (0.92, 3.17)	0.98 (0.94, 1.01)
15-19 years	5313.42 (2879.89, 8785.41)	4.19 (2.27, 6.94)	4214.43 (2413.16, 6882.43)	5.64 (3.23, 9.22)	1.01 (0.98, 1.05)
20-24 years	11521.89 (6335.71, 18750)	8.73 (4.8, 14.2)	8578.8 (4703.79, 13774.2)	11.72 (6.43, 18.82)	1.02 (0.98, 1.06)
25-29 years	16063.6 (9603.11, 25301.74)	14.62 (8.74, 23.02)	16286.52 (9601.22, 25689.05)	18.83 (11.1, 29.7)	0.93 (0.87, 0.98)
30-34 years	19155.16 (11942.87, 30588)	21.71 (13.53, 34.66)	32734.78 (20765.29, 51800.66)	27.02 (17.14, 42.76)	0.81 (0.76, 0.86)
35-39 years	27808.2 (16853.25, 43038.79)	30.45 (18.45, 47.12)	38748.9 (24769.34, 58289.21)	36.57 (23.38, 55.01)	0.71 (0.66, 0.76)
40-44 years	26850.26 (16335, 40890.23)	40.02 (24.35, 60.94)	42994.13 (25919.54, 64466.38)	46.97 (28.32, 70.43)	0.64 (0.58, 0.69)
45-49 years	25708.87 (16420.62, 38991.29)	49.81 (31.81, 75.54)	63501.96 (39849.87, 93484.76)	57.56 (36.12, 84.74)	0.56 (0.5, 0.61)
50-54 years	28554.86 (18161.27, 41530.94)	59.85 (38.07, 87.05)	81582.47 (52504.64, 120149.5)	67.5 (43.44, 99.41)	0.49 (0.44, 0.54)
55-59 years	30031.71 (20259.68, 42953.45)	69.25 (46.71, 99.04)	85453.08 (57430.83, 122210.62)	77.73 (52.24, 111.16)	0.43 (0.39, 0.47)
60-64 years	27704.27 (18646.28, 39524.19)	78.4 (52.77, 111.85)	63572.54 (42445.19, 90585.58)	87.08 (58.14, 124.08)	0.41 (0.37, 0.45)
65-69 years	23187.52 (15741.55, 32602.93)	84.99 (57.7, 119.5)	73217.41 (50247.83, 103438.92)	95.46 (65.51, 134.86)	0.46 (0.42, 0.5)
70-74 years	16109.17 (10929.19, 22630.86)	85.61 (58.08, 120.26)	52551.54 (35442.64, 72461.9)	98.6 (66.5, 135.96)	0.55 (0.5, 0.59)
75-79 years	9709.79 (6537.18, 13444.26)	85.32 (57.44, 118.13)	33930.01 (23310.23, 46567.84)	102.45 (70.38, 140.61)	0.62 (0.6, 0.65)
80-84 years	4431.51 (3017.23, 5921.99)	83.66 (56.96, 111.8)	20480.67 (14029.41, 27288.65)	103.48 (70.88, 137.88)	0.71 (0.7, 0.72)
85-89 years	1317.18 (897.76, 1776.17)	78.09 (53.22, 105.29)	9306.86 (6322.55, 12486.93)	97.7 (66.37, 131.09)	0.76 (0.73, 0.79)
90-94 years	208.66 (142.08, 282.43)	68.01 (46.31, 92.05)	2487.26 (1712.19, 3391.83)	84.83 (58.4, 115.68)	0.76 (0.71, 0.8)
95 plus years	21.47 (14.24, 29.32)	53.03 (35.17, 72.41)	431.83 (294.07, 596.67)	67.57 (46.01, 93.36)	0.77 (0.72, 0.82)

Notes: ASR: age-standardized rates; YLDs: years lived with disability; EAPCs: estimated annual percentage changes.

Table S45 Prevalence and YLDs of tension-type headache and its temporal trends from 1990, 2021 by gender, and age in China.

	1990		2021		EAPC 95%CI
	Number	ASR, per 100,000 persons	Number	ASR, per 100,000 persons	
**Prevalence**					
**Gender**					
Female	110367560.79 (95575144.26, 126490494.43)	19107.02 (16708.49, 21546.14)	153608982.83 (135965647.39, 173917944.89)	20292.27 (18007.36, 22987.63)	0.21 (0.17, 0.26)
Male	93696752.39 (81301112.69, 107220269.21)	15321.49 (13462.78, 17389.81)	130205168.22 (113609949.78, 148215066.5)	16837.13 (14750.15, 19169.77)	0.34 (0.27, 0.41)
**Age**					
5-9 years	5711535.94 (3679463.12, 8358652.74)	5477.22 (3528.52, 8015.74)	5263943.67 (3361166.32, 7557281.72)	5496.42 (3509.61, 7891.04)	0.03 (0.02, 0.04)
10-14 years	19118492.53 (14136933.57, 25473352.47)	18690.04 (13820.12, 24902.48)	16702187.61 (12333545.04, 22187764.45)	19377.67 (14309.23, 25741.97)	0.15 (0.12, 0.18)
15-19 years	25210646.86 (16333802, 34838519.67)	19903.37 (12895.26, 27504.41)	16509357.08 (10921752.8, 22909581.8)	22109.12 (14626.27, 30680.22)	0.41 (0.33, 0.49)
20-24 years	25634927.77 (17938392.18, 36226151.99)	19420.27 (13589.6, 27443.87)	16045206.25 (11478627.22, 22778308.11)	21927.42 (15686.72, 31128.9)	0.47 (0.38, 0.57)
25-29 years	23216018.69 (16149862.14, 32343778.66)	21126.7 (14696.46, 29433.01)	20576073.52 (14508763.66, 27907954.75)	23792.3 (16776.61, 32270.22)	0.46 (0.36, 0.55)
30-34 years	20890316.17 (14068863.56, 29179145.11)	23673.27 (15943.08, 33066.31)	32243427.09 (22073630.58, 44283123.28)	26613.75 (18219.59, 36551.32)	0.39 (0.31, 0.47)
35-39 years	21278835.36 (13726652.56, 29389611.41)	23296.54 (15028.24, 32176.39)	27400077.32 (18091603.39, 37669435.38)	25858.09 (17073.47, 35549.53)	0.37 (0.29, 0.45)
40-44 years	14894380.24 (10398165.17, 21129782.57)	22199.16 (15497.83, 31492.65)	22605580.81 (15694138.57, 31578273.28)	24696.46 (17145.75, 34499.07)	0.39 (0.31, 0.47)
45-49 years	10053260.72 (6975676.9, 13773197.03)	19475.91 (13513.79, 26682.44)	24468107.82 (17326330.62, 32983155.12)	22178.9 (15705.3, 29897.29)	0.5 (0.4, 0.6)
50-54 years	8344999.86 (5564540.95, 11569233.99)	17490.77 (11663.04, 24248.63)	23619583.1 (16282656.84, 33045304.33)	19543.08 (13472.44, 27342.02)	0.46 (0.36, 0.55)
55-59 years	7509721.02 (5086618.5, 10587866.06)	17315.83 (11728.67, 24413.39)	21219438.73 (14898865.78, 29573460.07)	19300.51 (13551.52, 26899.06)	0.41 (0.33, 0.5)
60-64 years	5940886.89 (4077035.88, 8144138.96)	16811.87 (11537.43, 23046.76)	12549556.61 (8373422.33, 17591407.31)	17189.96 (11469.63, 24096.11)	0.05 (0, 0.1)
65-69 years	6371965.08 (4552721.44, 8657545.87)	23356.09 (16687.75, 31733.76)	16573966.38 (12086574.14, 22811784.72)	21607.83 (15757.52, 29740.21)	-0.39 (-0.47, -0.3)
70-74 years	5370617.77 (3837482.08, 7413048.06)	28540.32 (20392.99, 39394.12)	13610213.74 (9771428.79, 18408811.67)	25536.81 (18334.11, 34540.41)	-0.49 (-0.58, -0.39)
75-79 years	2883406.44 (1828288.46, 3990162.28)	25335.94 (16064.82, 35060.79)	7497849.46 (4775175.08, 10342252.5)	22639.16 (14418.26, 31227.61)	-0.5 (-0.59, -0.41)
80-84 years	1191805.12 (798140.83, 1741363.54)	22499.09 (15067.43, 32873.75)	4148399.02 (2876503.68, 5888626.54)	20960.13 (14533.77, 29752.78)	-0.29 (-0.34, -0.25)
85-89 years	363148.23 (245284.52, 514164.12)	21528.12 (14540.93, 30480.63)	1979413.06 (1340992.58, 2824256.86)	20779.6 (14077.55, 29648.65)	-0.19 (-0.23, -0.15)
90-94 years	67292.25 (44799.38, 93762.96)	21932.01 (14601.09, 30559.39)	617026.04 (397599.32, 871133.1)	21044.63 (13560.74, 29711.35)	-0.18 (-0.21, -0.15)

95 plus years	12056.26 (7872.25, 16550.12)	29774.35 (19441.45, 40872.48)	184743.73 (120889.46, 258447.89)	28906.93 (18915.62, 40439.45)	-0.13 (-0.15, -0.1)
**YLDs**					
**Gender**					
Female	248793.57 (72186.71, 842240.17)	43.72 (12.86, 144.46)	367259.18 (108105.56, 1130637.9)	45.47 (13.03, 152.73)	0.06 (0.01, 0.11)
Male	240669.09 (78456.76, 873200.78)	39.92 (13.17, 140.4)	348905.76 (115344.24, 1113501.96)	41.63 (13.33, 143.34)	0.12 (0.08, 0.15)
**Age**					
5-9 years	6469.72 (376.75, 39276.65)	6.2 (0.36, 37.67)	6042.1 (345.36, 34111.86)	6.31 (0.36, 35.62)	0.1 (0.08, 0.12)
10-14 years	24601.2 (3255.74, 164005.93)	24.05 (3.18, 160.33)	21650.45 (2760.71, 141003.61)	25.12 (3.2, 163.59)	0.16 (0.13, 0.18)
15-19 years	47716.75 (10942.9, 206142.32)	37.67 (8.64, 162.75)	29976.21 (6480.57, 126377.73)	40.14 (8.68, 169.24)	0.22 (0.18, 0.27)
20-24 years	58548.07 (15593.36, 221967.4)	44.35 (11.81, 168.16)	34690.19 (8472.23, 127743.06)	47.41 (11.58, 174.57)	0.2 (0.15, 0.25)
25-29 years	52515.83 (14271.81, 180099.12)	47.79 (12.99, 163.89)	44095.26 (11076.99, 148653.29)	50.99 (12.81, 171.89)	0.19 (0.14, 0.24)
30-34 years	49372.07 (13805.6, 191624.61)	55.95 (15.64, 217.15)	71923.33 (19431.32, 261730.65)	59.37 (16.04, 216.03)	0.14 (0.09, 0.2)
35-39 years	57331.15 (16625.53, 191527.42)	62.77 (18.2, 209.69)	69946.5 (18373.2, 230646.02)	66.01 (17.34, 217.67)	0.1 (0.04, 0.16)
40-44 years	44321.56 (14184.33, 142218.23)	66.06 (21.14, 211.97)	63202.93 (19851.69, 196768.01)	69.05 (21.69, 214.97)	0.08 (0.02, 0.13)
45-49 years	33528.95 (10574.16, 92141.52)	64.95 (20.49, 178.5)	75239.39 (22578.9, 213735.31)	68.2 (20.47, 193.74)	0.09 (0.04, 0.15)
50-54 years	30224.03 (9356.8, 77690.25)	63.35 (19.61, 162.84)	79526.79 (23480.8, 206784.33)	65.8 (19.43, 171.1)	0.06 (0, 0.12)
55-59 years	26676.87 (8395.91, 66461.34)	61.51 (19.36, 153.25)	70059.71 (21268.18, 181529.32)	63.72 (19.34, 165.11)	0.06 (0.01, 0.11)
60-64 years	21499.51 (6891.58, 52123.18)	60.84 (19.5, 147.5)	44856.35 (14389.82, 111281.26)	61.44 (19.71, 152.43)	-0.03 (-0.08, 0.01)
65-69 years	17098.09 (4963.28, 49298.03)	62.67 (18.19, 180.7)	47080.21 (14488.57, 135032.19)	61.38 (18.89, 176.04)	-0.15 (-0.2, -0.11)
70-74 years	10968.5 (2723.83, 37303.66)	58.29 (14.47, 198.24)	29635.73 (7532.97, 100663.35)	55.61 (14.13, 188.87)	-0.23 (-0.27, -0.18)
75-79 years	5612.93 (1379.26, 20148.01)	49.32 (12.12, 177.04)	15558.06 (4003.57, 55026.17)	46.98 (12.09, 166.15)	-0.22 (-0.26, -0.18)
80-84 years	2234.22 (549.39, 7737.57)	42.18 (10.37, 146.07)	8097.89 (2076.43, 27336.2)	40.92 (10.49, 138.12)	-0.11 (-0.13, -0.1)
85-89 years	636.02 (144.58, 2350.47)	37.7 (8.57, 139.34)	3520.55 (810.02, 13410.36)	36.96 (8.5, 140.78)	-0.09 (-0.11, -0.08)
90-94 years	95.28 (16.9, 408.38)	31.05 (5.51, 133.1)	881.35 (165.62, 3650.76)	30.06 (5.65, 124.51)	-0.12 (-0.13, -0.1)
95 plus years	11.9 (0.72, 68.28)	29.38 (1.78, 168.63)	181.95 (11.05, 986.92)	28.47 (1.73, 154.42)	-0.12 (-0.14, -0.1)

Notes: ASR: age-standardized rates; YLDs: years lived with disability; EAPCs: estimated annual percentage changes.

Table S46. Prevalence and YLDs of migraine and its temporal trends from 1990, 2021 by gender, and age in China.

	1990		2021		EAPC 95%CI
	Number	ASR, per 100,000 persons	Number	ASR, per 100,000 persons	
**Prevalence**					
**Gender**					
Female	82587175.18 (70478899.45, 94673001.54)	13992.18 (12040.43, 16050.32)	114716057.16 (98856230.57, 132076637.56)	14959.04 (12857.78, 17190.35)	0.27 (0.22, 0.31)
Male	50887361.36 (43387428.18, 59129685.48)	8077.76 (6940.56, 9333.35)	70036222.95 (60652046.08, 81324228.2)	8781.96 (7497.4, 10129.33)	0.3 (0.25, 0.34)
**Age**					
5-9 years	1560250.31 (1041271.22, 2211851.64)	1496.24 (998.55, 2121.11)	1545079.14 (1047732.92, 2198506.91)	1613.32 (1094.01, 2295.6)	0.33 (0.28, 0.38)
10-14 years	7680796.27 (5708866.28, 10353123.41)	7508.67 (5580.93, 10121.11)	6948972.86 (5169830.21, 9191581.87)	8062.11 (5997.97, 10663.96)	0.28 (0.24, 0.33)
15-19 years	15235295.78 (11404519.29, 19819262.87)	12028 (9003.67, 15646.97)	9593758.4 (7057822.65, 12473183.11)	12847.84 (9451.75, 16703.93)	0.25 (0.2, 0.3)
20-24 years	18798155.33 (14634712.36, 23685160.82)	14240.93 (11086.83, 17943.18)	11095010.76 (8518890.08, 14017594.34)	15162.47 (11641.94, 19156.48)	0.23 (0.19, 0.28)
25-29 years	17244153.26 (13439548.97, 21513852.79)	15692.27 (12230.06, 19577.72)	14381787 (11321783.06, 18094785.54)	16629.79 (13091.48, 20923.16)	0.23 (0.18, 0.28)
30-34 years	14692565.06 (11463047.64, 18428144.32)	16649.87 (12990.12, 20883.09)	21513987.26 (17154202.83, 26840962.66)	17757.66 (14159.09, 22154.55)	0.24 (0.19, 0.28)
35-39 years	15635738.53 (12530556.12, 19619772.15)	17118.35 (13718.73, 21480.16)	19386094.18 (15453352.87, 24082365.03)	18295.11 (14583.69, 22727.09)	0.24 (0.2, 0.29)
40-44 years	11406962.46 (9224182.21, 14333018.1)	17001.38 (13748.08, 21362.49)	16814132.67 (13382492.23, 21264175.98)	18369.34 (14620.29, 23230.98)	0.28 (0.24, 0.33)
45-49 years	8124676.62 (6572617.36, 10088247.5)	15739.72 (12732.95, 19543.69)	18912346.58 (14962006.56, 23966920.87)	17142.93 (13562.18, 21724.6)	0.32 (0.27, 0.36)
50-54 years	7057304.84 (5766077.87, 8785790.13)	14791.82 (12085.46, 18414.65)	19555855.14 (16030093.04, 24949111.21)	16180.71 (13263.46, 20643.15)	0.36 (0.3, 0.41)
55-59 years	5890471.98 (4751993.45, 7329405.83)	13582.19 (10957.09, 16900.06)	16425701.87 (13128715.84, 20706397.58)	14940.28 (11941.45, 18833.86)	0.39 (0.33, 0.45)
60-64 years	4264563.05 (3400415.37, 5324846.65)	12068.11 (9622.69, 15068.56)	9686345.39 (7769072.88, 12162945.26)	13268.03 (10641.81, 16660.39)	0.39 (0.34, 0.45)
65-69 years	2833439.56 (2252734.21, 3615066.88)	10385.82 (8257.28, 13250.83)	8692513.25 (6824875.41, 11054590.47)	11332.61 (8897.74, 14412.1)	0.38 (0.32, 0.43)
70-74 years	1667032.95 (1318569.23, 2104309.53)	8858.88 (7007.09, 11182.64)	5106191.15 (4009224.95, 6559621.33)	9580.74 (7522.5, 12307.8)	0.34 (0.28, 0.4)
75-79 years	880460.01 (682370.09, 1124138.83)	7736.43 (5995.85, 9877.59)	2742435.48 (2129949.14, 3563500.83)	8280.57 (6431.21, 10759.71)	0.32 (0.25, 0.38)
80-84 years	375702.83 (295531.23, 491967.24)	7092.58 (5579.09, 9287.44)	1493495.75 (1173779.95, 1971899.99)	7546.01 (5930.62, 9963.19)	0.31 (0.25, 0.38)
85-89 years	108864.16 (84586.63, 138263.23)	6453.67 (5014.46, 8196.51)	658884.23 (505872.48, 840986.42)	6916.87 (5310.58, 8828.56)	0.34 (0.27, 0.42)
90-94 years	16450.62 (12424.62, 21411.25)	5361.62 (4049.45, 6978.39)	170998.05 (130643.48, 219728.57)	5832.15 (4455.8, 7494.19)	0.39 (0.29, 0.48)

95 plus years	1652.92 (1195.75, 2240.49)	4082.08 (2953.06, 5533.15)	28690.95 (20375.93, 40138.14)	4489.28 (3188.23, 6280.43)	0.44 (0.34, 0.53)
**YLDs**					
**Gender**					
Female	3041524.19 (360236.18, 6866451.03)	515.35 (64.62, 1157.38)	4242877.87 (548619.6, 9335915.43)	552.65 (64.68, 1221.52)	0.27 (0.22, 0.31)
Male	1987263.27 (394504.68, 4365126.26)	316.56 (65.91, 692.31)	2745320.72 (581676.86, 5851443.22)	341.31 (67.72, 739.24)	0.27 (0.22, 0.31)
**Age**					
5-9 years	51805.78 (1707.05, 159292.54)	49.68 (1.64, 152.76)	51441.42 (1806.83, 153376.49)	53.71 (1.89, 160.15)	0.34 (0.29, 0.39)
10-14 years	278537.15 (16424, 686111.79)	272.3 (16.06, 670.74)	252598.84 (13597.95, 611459.57)	293.06 (15.78, 709.41)	0.29 (0.25, 0.34)
15-19 years	572729.01 (57889.57, 1362488.6)	452.16 (45.7, 1075.66)	361195.04 (33973.66, 848761.66)	483.71 (45.5, 1136.65)	0.26 (0.21, 0.3)
20-24 years	704115.17 (77747.33, 1722498.79)	533.42 (58.9, 1304.91)	416428.92 (42963.7, 1007271.59)	569.09 (58.71, 1376.54)	0.24 (0.2, 0.29)
25-29 years	637117.76 (66147.41, 1489403.97)	579.78 (60.19, 1355.37)	533143.03 (51294.28, 1242799.03)	616.48 (59.31, 1437.06)	0.24 (0.2, 0.29)
30-34 years	547722.93 (68688.84, 1226795.5)	620.69 (77.84, 1390.23)	803666.89 (97700.99, 1802154.43)	663.35 (80.64, 1487.5)	0.24 (0.2, 0.29)
35-39 years	592704.99 (94651.95, 1290795.84)	648.91 (103.63, 1413.19)	736255.43 (110747.11, 1632749.82)	694.82 (104.51, 1540.86)	0.25 (0.2, 0.29)
40-44 years	438541.28 (75825.38, 978599.27)	653.62 (113.01, 1458.54)	646641.41 (102411.54, 1465627.37)	706.45 (111.88, 1601.19)	0.27 (0.23, 0.32)
45-49 years	315481.69 (58335.49, 679087.87)	611.17 (113.01, 1315.58)	731511.9 (126438.92, 1612978.55)	663.07 (114.61, 1462.07)	0.29 (0.25, 0.33)
50-54 years	275005.43 (52642.28, 604719.74)	576.4 (110.34, 1267.47)	755765.18 (135827.97, 1656213.18)	625.33 (112.39, 1370.37)	0.31 (0.26, 0.36)
55-59 years	228228.95 (47702.62, 498960.18)	526.25 (109.99, 1150.5)	629902.5 (121444.97, 1389014.12)	572.94 (110.46, 1263.4)	0.34 (0.28, 0.39)
60-64 years	166457.28 (37572.06, 356657.37)	471.05 (106.32, 1009.29)	373478.16 (79107.72, 810729.42)	511.58 (108.36, 1110.51)	0.33 (0.28, 0.38)
65-69 years	109508.95 (25373.9, 237011.8)	401.4 (93.01, 868.75)	331568.51 (71213.26, 708380.87)	432.27 (92.84, 923.53)	0.31 (0.26, 0.36)
70-74 years	61739.19 (14095.66, 133584.57)	328.09 (74.91, 709.89)	187300.94 (40341.78, 410472.94)	351.43 (75.69, 770.17)	0.29 (0.24, 0.34)
75-79 years	31699.18 (6991.41, 67832.2)	278.53 (61.43, 596.03)	97707.26 (20006.03, 219258.01)	295.02 (60.41, 662.03)	0.27 (0.21, 0.32)
80-84 years	13115.31 (2833.44, 28856.3)	247.59 (53.49, 544.75)	51460.77 (10403.29, 115034.24)	260.01 (52.56, 581.22)	0.26 (0.2, 0.33)
85-89 years	3699.68 (894.61, 8021.47)	219.32 (53.03, 475.53)	21949.94 (4943.49, 48927.21)	230.43 (51.9, 513.63)	0.27 (0.2, 0.34)
90-94 years	529.58 (93.2, 1193.28)	172.6 (30.38, 388.91)	5370.15 (874.24, 12169.63)	183.16 (29.82, 415.06)	0.31 (0.22, 0.4)
95 plus years	48.17 (4.65, 114.63)	118.97 (11.49, 283.1)	812.3 (66.01, 1963.35)	127.1 (10.33, 307.21)	0.36 (0.26, 0.46)

Notes: ASR: age-standardized rates; YLDs: years lived with disability; EAPCs: estimated annual percentage changes.

Table S47. Prevalence and YLDs of stroke and its temporal trends from 1990, 2021 by gender, and age in China.

	1990		2021		EAPC 95%CI
	Number	ASR, per 100,000 persons	Number	ASR, per 100,000 persons	
**Prevalence**					
**Gender**					
Female	5409632.82 (5041940.45, 5826048.11)	1149.59 (1064.73, 1241.73)	12616023.66 (11451736.97, 13793510.61)	1218.3 (1115.07, 1324.55)	0.11 (0.05, 0.17)
Male	5321447.3 (4946271.72, 5762679.57)	1205.87 (1112.53, 1315.03)	13719378.97 (12615636.63, 14899172.67)	1385.9 (1282.65, 1498.26)	0.51 (0.46, 0.55)
**Age**					
<5 years	55882.05 (48723.59, 64316.48)	49.98 (43.58, 57.53)	23148.03 (20335.25, 26325.5)	29.8 (26.18, 33.89)	-2.19 (-2.34, -2.04)
5-9 years	130997.62 (110501.27, 154056.34)	125.62 (105.97, 147.74)	74958.52 (64106.47, 88467.64)	78.27 (66.94, 92.37)	-1.79 (-1.87, -1.71)
10-14 years	190700.93 (161487.02, 221902.47)	186.43 (157.87, 216.93)	111684.83 (95228.04, 129696.25)	129.58 (110.48, 150.47)	-1.42 (-1.5, -1.33)
15-19 years	316087.47 (272675.54, 361806.81)	249.55 (215.27, 285.64)	140224.57 (121873.88, 160713.26)	187.79 (163.21, 215.23)	-1.13 (-1.2, -1.06)
20-24 years	425248.4 (372459.79, 486786.58)	322.16 (282.16, 368.78)	184620.89 (162920.27, 209560.78)	252.3 (222.65, 286.39)	-0.93 (-0.97, -0.89)
25-29 years	453797.67 (401976.72, 510785.26)	412.96 (365.8, 464.82)	286435.16 (251902.61, 322018.85)	331.21 (291.28, 372.35)	-0.79 (-0.82, -0.76)
30-34 years	466504.08 (409161.55, 523969.3)	528.65 (463.67, 593.77)	535297.59 (473876.26, 598171.45)	441.83 (391.14, 493.73)	-0.7 (-0.74, -0.65)
35-39 years	629729.24 (557217.87, 703719.7)	689.44 (610.05, 770.45)	628457.77 (561564.24, 700982.14)	593.09 (529.96, 661.53)	-0.62 (-0.66, -0.58)
40-44 years	623866.4 (551761.24, 698993.64)	929.83 (822.37, 1041.81)	749178.38 (667063.01, 836103.93)	818.47 (728.76, 913.44)	-0.55 (-0.58, -0.51)
45-49 years	699297.57 (625456.71, 777697.03)	1354.73 (1211.68, 1506.61)	1321855.36 (1175163.68, 1480017.48)	1198.18 (1065.22, 1341.55)	-0.48 (-0.52, -0.44)
50-54 years	928420.77 (823795.26, 1039718.47)	1945.93 (1726.64, 2179.21)	2125704.75 (1861182.77, 2404570.3)	1758.83 (1539.96, 1989.57)	-0.35 (-0.39, -0.31)
55-59 years	1092292.33 (964858.84, 1219185.43)	2518.6 (2224.76, 2811.18)	2760427.23 (2436694.63, 3078150.49)	2510.79 (2216.34, 2799.79)	0.01 (-0.03, 0.05)
60-64 years	1112935.87 (966136.2, 1263241.43)	3149.45 (2734.03, 3574.79)	2584288.39 (2241991.25, 2940336.31)	3539.87 (3071, 4027.57)	0.4 (0.35, 0.46)
65-69 years	1162343.31 (1005077.88, 1334859.83)	4260.51 (3684.06, 4892.86)	3909443.19 (3408716.24, 4442523.1)	5096.82 (4444.02, 5791.81)	0.56 (0.52, 0.61)
70-74 years	1076811.69 (910689.97, 1269352.82)	5722.35 (4839.55, 6745.54)	3854925.72 (3301148.24, 4425078.33)	7232.99 (6193.94, 8302.76)	0.74 (0.69, 0.8)
75-79 years	816508.88 (699983.82, 955657.73)	7174.51 (6150.62, 8397.18)	3285286.19 (2888367.86, 3732534.97)	9919.66 (8721.2, 11270.09)	1.15 (1.08, 1.21)
80-84 years	413158.68 (347926.9, 489772.09)	7799.68 (6568.22, 9246)	2340301.48 (2051664.73, 2661008.32)	11824.57 (10366.21, 13444.97)	1.49 (1.41, 1.57)
85-89 years	115863.77 (93454.09, 141718.07)	6868.62 (5540.14, 8401.32)	1064008.92 (902790.83, 1248929.51)	11169.82 (9477.37, 13111.09)	1.69 (1.61, 1.78)

90-94 years	18342.03 (14303.24, 23496.94)	5978.07 (4661.74, 7658.17)	293896.63 (231017.79, 363805.74)	10023.8 (7879.22, 12408.16)	1.71 (1.61, 1.81)
95 plus years	2291.34 (1742.86, 2977.63)	5658.74 (4304.2, 7353.61)	61259.04 (46343.99, 78610.07)	9585.23 (7251.46, 12300.15)	1.62 (1.55, 1.69)
**YLDs**					
**Gender**					
Female	1106550.76 (789757.98, 1419795.2)	236.49 (168.57, 302.43)	2562183.42 (1814853.87, 3309157.59)	248.04 (174.88, 319.53)	0.08 (0.03, 0.14)
Male	965653.65 (684752.66, 1238887.41)	225.81 (160.49, 290.75)	2527673.34 (1810587.76, 3274605.43)	257.55 (185.01, 333.03)	0.5 (0.44, 0.55)
**Age**					
<5 years	10780.15 (7460.98, 14441.4)	9.64 (6.67, 12.92)	4465.34 (3053.15, 5952.53)	5.75 (3.93, 7.66)	-2.19 (-2.34, -2.04)
5-9 years	25062.89 (16886.85, 33928.07)	24.03 (16.19, 32.54)	14449.1 (9860.41, 19442.18)	15.09 (10.3, 20.3)	-1.78 (-1.86, -1.69)
10-14 years	36731.44 (24961.45, 49239.7)	35.91 (24.4, 48.14)	21551.02 (14358.69, 28641.07)	25 (16.66, 33.23)	-1.41 (-1.49, -1.33)
15-19 years	60656.25 (40887.93, 80510.26)	47.89 (32.28, 63.56)	27116.38 (18647.02, 35701.2)	36.31 (24.97, 47.81)	-1.1 (-1.17, -1.04)
20-24 years	81590.86 (55659.26, 108985.05)	61.81 (42.17, 82.56)	35644.22 (24285.85, 47363.52)	48.71 (33.19, 64.73)	-0.9 (-0.94, -0.86)
25-29 years	86960.98 (59668.72, 116893.6)	79.13 (54.3, 106.37)	55053.81 (37328.93, 74490.29)	63.66 (43.16, 86.13)	-0.77 (-0.79, -0.75)
30-34 years	89227.4 (60986.85, 117704.6)	101.11 (69.11, 133.38)	102484.32 (70473.74, 136128.19)	84.59 (58.17, 112.36)	-0.69 (-0.73, -0.66)
35-39 years	118824.85 (81952.75, 155114.7)	130.09 (89.72, 169.82)	118720.28 (80777.27, 157045.53)	112.04 (76.23, 148.21)	-0.62 (-0.65, -0.58)
40-44 years	115223.56 (79292.55, 152807.63)	171.73 (118.18, 227.75)	138806.78 (95948.72, 182447.45)	151.65 (104.82, 199.32)	-0.54 (-0.57, -0.5)
45-49 years	126178.3 (89437.82, 165584.36)	244.44 (173.27, 320.78)	238670.36 (168329.76, 313572.17)	216.34 (152.58, 284.23)	-0.48 (-0.53, -0.44)
50-54 years	168629.33 (119250.58, 219310.74)	353.44 (249.94, 459.67)	385959.51 (273671.06, 508494.59)	319.35 (226.44, 420.73)	-0.35 (-0.4, -0.31)
55-59 years	204592.83 (147421.41, 267156.94)	471.75 (339.92, 616.01)	514686.13 (368283.67, 676399.5)	468.14 (334.98, 615.23)	0 (-0.04, 0.04)
60-64 years	213426.98 (155401.24, 280846.21)	603.97 (439.76, 794.75)	489270.39 (351915.81, 641955.1)	670.19 (482.04, 879.33)	0.37 (0.32, 0.43)
65-69 years	228298.11 (161770.11, 302329.23)	836.81 (592.96, 1108.17)	750625.62 (533174.18, 999488.99)	978.61 (695.11, 1303.06)	0.51 (0.46, 0.56)
70-74 years	216903.22 (154192.81, 289368.97)	1152.66 (819.41, 1537.75)	750713.01 (529939.5, 1000494.18)	1408.56 (994.32, 1877.23)	0.65 (0.6, 0.71)
75-79 years	169401.33 (119615.76, 225514.7)	1488.5 (1051.04, 1981.55)	653553.49 (457549.26, 865314.96)	1973.35 (1381.53, 2612.75)	1.02 (0.95, 1.09)
80-84 years	88962.65 (61602.73, 118494.05)	1679.45 (1162.95, 2236.95)	482237.98 (337331.77, 629137.79)	2436.55 (1704.4, 3178.77)	1.35 (1.27, 1.43)
85-89 years	25959.08 (17502.39, 35610.04)	1538.9 (1037.57, 2111.03)	227023.97 (154806.92, 306465.78)	2383.27 (1625.14, 3217.23)	1.53 (1.45, 1.6)
90-94 years	4248.88 (2776.17, 5856.05)	1384.8 (904.81, 1908.62)	64917.62 (43160.35, 89046.5)	2214.12 (1472.05, 3037.07)	1.55 (1.46, 1.64)
95 plus years	545.3 (356.34, 748.74)	1346.69 (880.03, 1849.1)	13907.43 (9232.58, 19426.24)	2176.1 (1444.63, 3039.63)	1.48 (1.4, 1.55)

Notes: ASR: age-standardized rates; YLDs: years lived with disability; EAPCs: estimated annual percentage changes.

Table S48. Prevalence and YLDs of Alzheimer's disease and other dementias and its temporal trends from 1990, 2021 by gender, and age in China.

	1990		2021		EAPC 95%CI
	Number	ASR, per 100,000 persons	Number	ASR, per 100,000 persons	
**Prevalence**					
**Gender**					
Female	2512933.8 (2165052.11, 2892723.8)	785.19 (681.22, 900.41)	10828629.6 (9315735.2, 12515957.37)	1025.11 (879.04, 1186.81)	0.46 (0.36, 0.55)
Male	1511602.03 (1280687.86, 1737519.77)	574.55 (493.64, 666.55)	6162197.72 (5142286.06, 7141800.17)	731.21 (618.54, 851.63)	0.51 (0.44, 0.57)
**Age**					
40-44 years	13650 (5941.83, 23015.5)	20.34 (8.86, 34.3)	17470.74 (7433.09, 30197.43)	19.09 (8.12, 32.99)	-0.17 (-0.24, -0.11)
45-49 years	68521.12 (42119.25, 102374.46)	132.74 (81.6, 198.33)	142474.66 (90180.61, 214492.55)	129.14 (81.74, 194.42)	-0.08 (-0.15, -0.02)
50-54 years	157374.68 (111654.54, 214043.44)	329.85 (234.02, 448.63)	413233.97 (292799.24, 561458.58)	341.91 (242.27, 464.56)	0.09 (0.03, 0.14)
55-59 years	282530.84 (218529.25, 357822.71)	651.46 (503.88, 825.06)	800221.32 (621533.45, 1025915.86)	727.86 (565.33, 933.14)	0.26 (0.22, 0.3)
60-64 years	418251.13 (322289.51, 531990.8)	1183.59 (912.03, 1505.46)	1037431.29 (791767.3, 1326183.85)	1421.04 (1084.54, 1816.56)	0.4 (0.34, 0.45)
65-69 years	540451.55 (417071.23, 680141.27)	1981 (1528.75, 2493.02)	1930259.23 (1460803.83, 2410758.95)	2516.52 (1904.48, 3142.96)	0.44 (0.36, 0.51)
70-74 years	645211.58 (491544.59, 832036.57)	3428.76 (2612.15, 4421.58)	2419809.55 (1826008.62, 3132783.44)	4540.28 (3426.14, 5878.03)	0.48 (0.39, 0.57)
75-79 years	764815.86 (600994.06, 949281.2)	6720.29 (5280.82, 8341.15)	3013326.61 (2369680.63, 3726924.53)	9098.5 (7155.06, 11253.15)	0.53 (0.43, 0.63)
80-84 years	685639.06 (537472.48, 863440.15)	12943.61 (10146.49, 16300.16)	3379626.8 (2671389.13, 4255464.67)	17075.85 (13497.42, 21501.09)	0.47 (0.37, 0.57)
85-89 years	349464.22 (271603.45, 440245.99)	20716.9 (16101.17, 26098.62)	2531542.55 (1986434.31, 3160640.77)	26575.78 (20853.31, 33179.96)	0.4 (0.3, 0.5)
90-94 years	84742.73 (66694.21, 107308.55)	27619.5 (21737.1, 34974.2)	1031789.29 (813062.88, 1289058.31)	35190.78 (27730.78, 43965.34)	0.45 (0.35, 0.54)
95 plus years	13883.06 (10638.6, 17714.41)	34285.85 (26273.27, 43747.84)	273641.33 (211022.68, 340429.8)	42816.77 (33018.81, 53267.19)	0.52 (0.46, 0.58)
**YLDs**					
**Gender**					
Female	521765.74 (354161.61, 698504.78)	166.61 (114.06, 222.28)	2275549.94 (1555762.98, 3049867.28)	216.38 (147.99, 288.88)	0.44 (0.35, 0.53)
Male	286690.3 (194753.18, 376877.59)	113.18 (77.45, 149.5)	1184773.79 (818129.27, 1591260.42)	143.7 (99.59, 192.13)	0.5 (0.43, 0.56)
**Age**					
40-44 years	2550.22 (1032.71, 4700.7)	3.8 (1.54, 7.01)	3272.77 (1319.96, 6137.44)	3.58 (1.44, 6.71)	-0.17 (-0.23, -0.1)

45-49 years	12425.87 (6808.07, 20820.48)	24.07 (13.19, 40.33)	25924.52 (14013.75, 43538.66)	23.5 (12.7, 39.47)	-0.07 (-0.13, 0)
50-54 years	29076.39 (17441.46, 43874.65)	60.94 (36.56, 91.96)	76594.02 (46018.95, 115544.99)	63.37 (38.08, 95.6)	0.1 (0.04, 0.15)
55-59 years	53770.09 (33446.85, 75734.26)	123.98 (77.12, 174.63)	152756.67 (94763.88, 217869.12)	138.94 (86.19, 198.17)	0.28 (0.24, 0.31)
60-64 years	81619.37 (51376.32, 119505.06)	230.97 (145.39, 338.18)	202246.02 (126502.32, 293722.64)	277.03 (173.28, 402.33)	0.4 (0.35, 0.45)
65-69 years	107910.82 (70193.25, 157627.8)	395.54 (257.29, 577.78)	384981.01 (248028.88, 562474.84)	501.91 (323.36, 733.31)	0.43 (0.36, 0.51)
70-74 years	131662.72 (85021.53, 190339.99)	699.68 (451.82, 1011.5)	493541.43 (314834.98, 712549.18)	926.03 (590.72, 1336.95)	0.47 (0.38, 0.57)
75-79 years	148912.51 (99651.97, 209973.75)	1308.47 (875.62, 1845)	584639.14 (394385.59, 821858.32)	1765.27 (1190.82, 2481.54)	0.51 (0.4, 0.61)
80-84 years	141413.68 (90581.59, 197560.6)	2669.63 (1710.01, 3729.58)	692122.18 (445109.9, 958400.82)	3497 (2248.95, 4842.4)	0.44 (0.34, 0.54)
85-89 years	76271.16 (50900.08, 106561.25)	4521.5 (3017.45, 6317.15)	545955.56 (367576.79, 756439.62)	5731.36 (3858.77, 7941)	0.36 (0.26, 0.47)
90-94 years	19514.18 (12917, 26957.94)	6360.1 (4209.93, 8786.18)	233955.49 (157855.76, 321471.35)	7979.42 (5383.92, 10964.28)	0.41 (0.32, 0.5)
95 plus years	3329.02 (2290.14, 4617.65)	8221.41 (5655.78, 11403.82)	64334.93 (44118.14, 88428.3)	10066.51 (6903.18, 13836.41)	0.47 (0.42, 0.53)

Notes: ASR: age-standardized rates; YLDs: years lived with disability; EAPCs: estimated annual percentage changes.

Table S49. Prevalence and YLDs of Parkinson's disease and its temporal trends from 1990, 2021 by gender, and age in China.

	1990		2021		EAPC 95%CI
	Number	ASR, per 100,000 persons	Number	ASR, per 100,000 persons	
**Prevalence**					
**Gender**					
Female	319809.55 (267552.12, 383625.93)	82.99 (69.08, 99.36)	2150595.21 (1836903.68, 2542494.35)	195.7 (166.56, 231.35)	2.75 (2.64, 2.86)
Male	332023.12 (278514.31, 401955.72)	106.1 (88.02, 126.7)	2926461.66 (2452280.19, 3485039.82)	306.73 (259.07, 359.55)	3.45 (3.31, 3.58)
**Age**					
20-24 years	252.27 (57.96, 493.52)	0.19 (0.04, 0.37)	151.52 (28.26, 306.43)	0.21 (0.04, 0.42)	0.46 (0.37, 0.54)
25-29 years	1439.1 (331.58, 2814)	1.31 (0.3, 2.56)	1225.71 (231.06, 2488.27)	1.42 (0.27, 2.88)	0.45 (0.37, 0.53)
30-34 years	3321.79 (1346.88, 5864.41)	3.76 (1.53, 6.65)	5313.39 (2277.86, 9543.14)	4.39 (1.88, 7.88)	0.64 (0.57, 0.7)
35-39 years	7623.05 (4493.48, 11548.44)	8.35 (4.92, 12.64)	11861.09 (6924.67, 18059.7)	11.19 (6.53, 17.04)	0.98 (0.95, 1.01)
40-44 years	11404.42 (7418.69, 17501.32)	17 (11.06, 26.08)	29298.09 (20707.52, 41409.77)	32.01 (22.62, 45.24)	1.75 (1.56, 1.94)
45-49 years	18819.93 (13624.28, 25162.32)	36.46 (26.39, 48.75)	114798.86 (82435.94, 157200.49)	104.06 (74.72, 142.49)	2.57 (2.15, 3)
50-54 years	33256.51 (22978.1, 45551.76)	69.7 (48.16, 95.47)	285609.12 (206173.09, 382810.25)	236.32 (170.59, 316.74)	2.93 (2.43, 3.43)
55-59 years	53756.57 (39501.74, 70116.82)	123.95 (91.08, 161.67)	468713.09 (363298.87, 606417.22)	426.33 (330.44, 551.58)	3.11 (2.68, 3.54)
60-64 years	76269.1 (57308.42, 102451.55)	215.83 (162.17, 289.92)	504272.49 (383612.37, 666296.41)	690.74 (525.46, 912.67)	3.18 (2.87, 3.49)
65-69 years	108746.05 (84614.03, 141440.12)	398.6 (310.15, 518.44)	828473.94 (657240.67, 1056922.03)	1080.1 (856.86, 1377.93)	3.14 (3, 3.29)
70-74 years	125931.05 (93670.38, 167631.4)	669.22 (497.78, 890.82)	864029.12 (662318.88, 1102714.98)	1621.18 (1242.71, 2069.02)	3.13 (3.04, 3.23)
75-79 years	112747.15 (85290.13, 144982.75)	990.69 (749.43, 1273.94)	782641.03 (610446.68, 981418.22)	2363.12 (1843.2, 2963.31)	3.18 (3.06, 3.3)
80-84 years	68007.98 (48276.92, 90114.24)	1283.87 (911.38, 1701.19)	635240.86 (484107.4, 808952.02)	3209.61 (2445.99, 4087.3)	3.32 (3.2, 3.43)
85-89 years	24795.44 (18511.49, 32583.07)	1469.92 (1097.4, 1931.59)	379133.9 (294281.05, 477008.48)	3980.09 (3089.32, 5007.57)	3.48 (3.37, 3.58)
90-94 years	4803.71 (3339.86, 6736.49)	1565.63 (1088.53, 2195.57)	133763.42 (101586.51, 177867.02)	4562.21 (3464.77, 6066.43)	3.61 (3.49, 3.73)
95 plus years	658.53 (429.34, 989.37)	1626.33 (1060.3, 2443.37)	32531.23 (23959.2, 44334.21)	5090.17 (3748.91, 6936.99)	3.64 (3.47, 3.8)
**YLDs**					
**Gender**					

Female	45986.2 (31851.44, 62740.01)	11.77 (8.16, 16.11)	303966.48 (213912.85, 408793.98)	27.56 (19.46, 36.95)	2.72 (2.6, 2.84)
Male	48931.8 (33286.49, 66599.13)	15.23 (10.53, 20.46)	424512.37 (289946.76, 569918.8)	43.97 (30.49, 58.6)	3.42 (3.28, 3.56)
**Age**					
20-24 years	42.36 (8.93, 86.93)	0.03 (0.01, 0.07)	25.45 (4.76, 52.56)	0.03 (0.01, 0.07)	0.46 (0.38, 0.54)
25-29 years	241.65 (51, 494.15)	0.22 (0.05, 0.45)	205.91 (38.27, 425.28)	0.24 (0.04, 0.49)	0.45 (0.37, 0.54)
30-34 years	557.36 (212.14, 1008.52)	0.63 (0.24, 1.14)	891.53 (350.95, 1607.46)	0.74 (0.29, 1.33)	0.64 (0.57, 0.7)
35-39 years	1277.51 (648.49, 2079.29)	1.4 (0.71, 2.28)	1986.36 (1025.94, 3472.89)	1.87 (0.97, 3.28)	0.98 (0.94, 1.01)
40-44 years	1909.38 (1093.98, 3093.18)	2.85 (1.63, 4.61)	4825.8 (2900.08, 7700.27)	5.27 (3.17, 8.41)	1.67 (1.48, 1.86)
45-49 years	3037.95 (1948.15, 4584.26)	5.89 (3.77, 8.88)	17888.35 (10801.54, 26965.81)	16.21 (9.79, 24.44)	2.48 (2.06, 2.9)
50-54 years	5325.99 (3191.36, 8076.19)	11.16 (6.69, 16.93)	44023.48 (27352.5, 66373.6)	36.43 (22.63, 54.92)	2.9 (2.42, 3.39)
55-59 years	8127.79 (5073.01, 11693.52)	18.74 (11.7, 26.96)	71139.45 (46433.43, 102624.27)	64.71 (42.23, 93.34)	3.11 (2.67, 3.55)
60-64 years	11383.36 (7292, 16756.13)	32.21 (20.64, 47.42)	75343.26 (48788.2, 107814.59)	103.2 (66.83, 147.68)	3.18 (2.87, 3.5)
65-69 years	15915.92 (10510.65, 21898.68)	58.34 (38.53, 80.27)	121347.28 (79898.33, 166829.15)	158.2 (104.17, 217.5)	3.15 (3, 3.3)
70-74 years	18000.83 (11874.27, 25945.2)	95.66 (63.1, 137.88)	123664.04 (80220.76, 175456.98)	232.03 (150.52, 329.21)	3.14 (3.04, 3.24)
75-79 years	15787.59 (10546.81, 22628.17)	138.72 (92.67, 198.83)	109449.54 (73180.55, 156076.58)	330.47 (220.96, 471.26)	3.17 (3.06, 3.29)
80-84 years	9289.58 (6030.62, 13799.92)	175.37 (113.85, 260.52)	86399.1 (57437.73, 121024.37)	436.54 (290.21, 611.49)	3.3 (3.19, 3.42)
85-89 years	3310.51 (2211.22, 4738.1)	196.25 (131.09, 280.88)	50074.28 (34580.12, 68898.83)	525.67 (363.02, 723.29)	3.45 (3.34, 3.56)
90-94 years	626.62 (395.66, 913.44)	204.23 (128.95, 297.71)	17183.62 (11720.69, 24612.99)	586.07 (399.75, 839.46)	3.58 (3.45, 3.7)
95 plus years	83.58 (49.51, 132.1)	206.4 (122.26, 326.23)	4031.42 (2692.7, 5817.14)	630.8 (421.33, 910.21)	3.59 (3.42, 3.76)

Notes: ASR: age-standardized rates; YLDs: years lived with disability; EAPCs: estimated annual percentage changes.

Table S50. Prevalence and YLDs of upper digestive system diseases and its temporal trends from 1990, 2021 by gender, and age in China.

	1990		2021		EAPC 95%CI
	Number	ASR, per 100,000 persons	Number	ASR, per 100,000 persons	
**Prevalence**					
**Gender**					
Female	32067441.7 (28328040.89, 35886711.6)	6017.66 (5342.35, 6692.64)	50997572.23 (44809040.07, 56739539.91)	5625.6 (4953.78, 6282.07)	-0.3 (-0.42, -0.18)
Male	29597519.82 (26218480.51, 33274037.12)	5295.04 (4719.15, 5869.73)	43897936.99 (38513149.65, 49207292.28)	4861.27 (4320.79, 5450.21)	-0.38 (-0.51, -0.25)
**Age**					
<5 years	406152.42 (278206.28, 578136.59)	363.27 (248.83, 517.09)	181390.92 (126312.38, 254443.37)	233.55 (162.63, 327.6)	-1.84 (-2.01, -1.66)
5-9 years	452451.94 (256573.82, 769613.56)	433.89 (246.05, 738.04)	223923.27 (142029.94, 347830.62)	233.81 (148.3, 363.19)	-2.51 (-2.81, -2.2)
10-14 years	744530.41 (531253.21, 1004032.79)	727.85 (519.35, 981.53)	497384.92 (353918.51, 668157.18)	577.06 (410.61, 775.19)	-1.01 (-1.18, -0.83)
15-19 years	2831151.04 (2059554.45, 3735912.28)	2235.15 (1625.98, 2949.44)	1545055.53 (1115797.33, 2074809.18)	2069.12 (1494.26, 2778.56)	-0.39 (-0.54, -0.24)
20-24 years	5800032.59 (4194080.03, 7580197.44)	4393.93 (3177.31, 5742.53)	3045558.43 (2189829.26, 4047448.29)	4162.07 (2992.63, 5531.25)	-0.3 (-0.45, -0.14)
25-29 years	6414435.12 (4785377.62, 8402070.63)	5837.17 (4354.72, 7645.93)	4812823.4 (3573659.39, 6346058.32)	5565.11 (4132.25, 7338)	-0.25 (-0.41, -0.1)
30-34 years	6035112.5 (4433370.57, 8145144.94)	6839.09 (5023.97, 9230.22)	7944709.67 (5753006.42, 10847649.6)	6557.57 (4748.54, 8953.66)	-0.23 (-0.37, -0.09)
35-39 years	6991588.22 (5139151.99, 9568500.9)	7654.54 (5626.46, 10475.8)	7744613.66 (5629437.44, 10736558.26)	7308.77 (5312.63, 10132.34)	-0.21 (-0.35, -0.08)
40-44 years	5569649.81 (4075966.68, 7422700.1)	8301.22 (6074.98, 11063.08)	7153409.67 (5140920.96, 9641726.82)	7815.06 (5616.43, 10533.53)	-0.26 (-0.4, -0.13)
45-49 years	4579213.68 (3415893.41, 6026466.92)	8871.19 (6617.52, 11674.91)	9078470.07 (6664418.65, 12205268.15)	8229.1 (6040.9, 11063.36)	-0.34 (-0.47, -0.21)
50-54 years	4645312.18 (3439079.81, 6010090.98)	9736.38 (7208.17, 12596.9)	10804751.35 (7886389.03, 14227847.39)	8939.96 (6525.28, 11772.27)	-0.39 (-0.52, -0.27)
55-59 years	4817482.81 (3597956.95, 6198989.31)	11108.1 (8296.13, 14293.56)	11094107.15 (8119544.44, 14538263.83)	10090.84 (7385.27, 13223.53)	-0.43 (-0.56, -0.3)
60-64 years	4109958.21 (3180564.57, 5304955.08)	11630.6 (9000.55, 15012.27)	7706567.14 (5883937.78, 10045324.7)	10556.19 (8059.62, 13759.74)	-0.39 (-0.51, -0.27)
65-69 years	3379855.57 (2564271.79, 4425989.6)	12388.68 (9399.2, 16223.22)	8730626.53 (6542646.46, 11442298.78)	11382.3 (8529.79, 14917.57)	-0.32 (-0.44, -0.2)
70-74 years	2460329.69 (1911700.49, 3146329.53)	13074.58 (10159.08, 16720.1)	6418371.06 (4928927.51, 8235276.21)	12042.78 (9248.14, 15451.83)	-0.31 (-0.42, -0.2)
75-79 years	1514851.99 (1164430.94, 1983996.25)	13310.71 (10231.63, 17432.99)	4085870.46 (3135789.23, 5378761.48)	12336.96 (9468.27, 16240.74)	-0.29 (-0.39, -0.19)
80-84 years	677161.26 (510693.78, 876158.17)	12783.56 (9640.96, 16540.26)	2366051.66 (1762242.17, 3109401.65)	11954.67 (8903.88, 15710.51)	-0.27 (-0.35, -0.19)
85-89 years	197459.28 (147898.73, 265033.45)	11705.76 (8767.72, 15711.69)	1065674.1 (806196.79, 1453356.51)	11187.3 (8463.34, 15257.13)	-0.18 (-0.23, -0.13)

90-94 years	33356.5 (24832.55, 44213.86)	10871.61 (8093.47, 14410.26)	318228.71 (236447.77, 424829.83)	10853.69 (8064.42, 14489.48)	0.02 (-0.01, 0.04)
95 plus years	4876.3 (3466.64, 6657.09)	12042.59 (8561.27, 16440.47)	77921.54 (55736.59, 105980.9)	12192.41 (8721.13, 16582.87)	0.1 (0.08, 0.12)
**YLDs**					
**Gender**					
Female	480506.1 (302797.68, 741123.25)	93.21 (58.29, 142.23)	679715.13 (429822.02, 1050442.22)	72.58 (45.82, 112.54)	-0.91 (-1.02, -0.79)
Male	442459.7 (281046.29, 675519.53)	82.26 (51.82, 124.63)	559095.53 (355792.32, 872943.45)	60.9 (38.68, 93.43)	-1.14 (-1.28, -1.01)
**Age**					
<5 years	19182.94 (10440.49, 31086.03)	17.16 (9.34, 27.8)	7857.47 (4341, 12467.17)	10.12 (5.59, 16.05)	-2.13 (-2.31, -1.95)
5-9 years	20271.46 (9827.75, 38050.55)	19.44 (9.42, 36.49)	8900.1 (4427.68, 15807.13)	9.29 (4.62, 16.51)	-2.92 (-3.24, -2.59)
10-14 years	17907.36 (9985.37, 30213.09)	17.51 (9.76, 29.54)	8907.51 (4938.79, 13852.23)	10.33 (5.73, 16.07)	-2.16 (-2.45, -1.88)
15-19 years	35789.96 (20139.32, 56956.24)	28.26 (15.9, 44.97)	16054.13 (8310.69, 26000.07)	21.5 (11.13, 34.82)	-1.18 (-1.4, -0.96)
20-24 years	63765.53 (35049.05, 108092.91)	48.31 (26.55, 81.89)	28930.38 (14996.08, 51160.19)	39.54 (20.49, 69.92)	-0.9 (-1.1, -0.69)
25-29 years	71167.67 (39370.21, 120216.87)	64.76 (35.83, 109.4)	46540.01 (24711.68, 80710.4)	53.81 (28.57, 93.33)	-0.83 (-1.02, -0.65)
30-34 years	70038 (38796.57, 119284.08)	79.37 (43.96, 135.17)	80427.6 (44413.15, 139285.82)	66.38 (36.66, 114.97)	-0.77 (-0.92, -0.62)
35-39 years	87513.23 (51219.41, 144699.35)	95.81 (56.08, 158.42)	83996.14 (47853.41, 143464.29)	79.27 (45.16, 135.39)	-0.73 (-0.86, -0.61)
40-44 years	78729.99 (45166.09, 123667.06)	117.34 (67.32, 184.32)	84659.13 (47543.27, 138958.29)	92.49 (51.94, 151.81)	-0.87 (-0.98, -0.75)
45-49 years	72137.11 (43040.75, 113265.15)	139.75 (83.38, 219.43)	118136.91 (68455.42, 190147.39)	107.08 (62.05, 172.36)	-0.96 (-1.07, -0.85)
50-54 years	79448.89 (46357.73, 135112.65)	166.52 (97.16, 283.19)	151109.22 (88430.5, 267017.5)	125.03 (73.17, 220.93)	-1.04 (-1.15, -0.93)
55-59 years	87248.94 (50491.41, 145712.73)	201.18 (116.42, 335.98)	162206.61 (95035.63, 267422.42)	147.54 (86.44, 243.24)	-1.13 (-1.24, -1.02)
60-64 years	75578.64 (45742.59, 119203.56)	213.88 (129.45, 337.33)	112397.44 (68967.14, 179086)	153.96 (94.47, 245.31)	-1.13 (-1.25, -1.02)
65-69 years	59466.35 (36774.55, 94595.46)	217.97 (134.8, 346.73)	124574.33 (77539.14, 199456.71)	162.41 (101.09, 260.04)	-0.98 (-1.09, -0.87)
70-74 years	43082.21 (26701.84, 66873.2)	228.95 (141.9, 355.37)	92174.14 (56768.35, 142887.05)	172.95 (106.51, 268.1)	-0.95 (-1.05, -0.84)
75-79 years	26325.57 (16028.52, 42012.51)	231.32 (140.84, 369.16)	58430.57 (35099.17, 90252.78)	176.43 (105.98, 272.51)	-0.93 (-1.02, -0.83)
80-84 years	11660.05 (6985.28, 18160.45)	220.12 (131.87, 342.84)	33834.43 (20231.88, 54094.41)	170.95 (102.22, 273.32)	-0.89 (-1, -0.78)
85-89 years	3111.24 (1795.72, 4964.79)	184.44 (106.45, 294.32)	14469.17 (8706.78, 22832.86)	151.9 (91.4, 239.7)	-0.69 (-0.82, -0.56)
90-94 years	480.65 (294.97, 779.24)	156.66 (96.14, 253.97)	4234.25 (2667.35, 6677.5)	144.42 (90.97, 227.75)	-0.24 (-0.38, -0.09)
95 plus years	59.99 (35.65, 94.94)	148.14 (88.04, 234.46)	971.12 (576.5, 1523.98)	151.95 (90.21, 238.46)	0.19 (0.04, 0.34)

Notes: ASR: age-standardized rates; YLDs: years lived with disability; EAPCs: estimated annual percentage changes.

Table S51. Prevalence and YLDs of inflammatory bowel disease and its temporal trends from 1990, 2021 by gender, and age in China.

	1990		2021		EAPC 95%CI
	Number	ASR, per 100,000 persons	Number	ASR, per 100,000 persons	
**Prevalence**					
**Gender**					
Female	31950.19 (27037.72, 38468.97)	5.9 (4.99, 7.03)	86014.59 (72898.29, 102824.12)	9.47 (8.05, 11.39)	2.53 (1.89, 3.18)
Male	30147.75 (25384.99, 36335.01)	5.3 (4.46, 6.35)	82062.08 (69529.91, 98924.64)	8.87 (7.55, 10.66)	2.54 (1.93, 3.16)
**Age**					
<5 years	13.46 (7.81, 20.37)	0.01 (0.01, 0.02)	12.71 (7.7, 18.44)	0.02 (0.01, 0.02)	1.17 (0.74, 1.6)
5-9 years	244.99 (168.76, 337.62)	0.23 (0.16, 0.32)	281.89 (196.87, 372.71)	0.29 (0.21, 0.39)	1.39 (0.78, 2.01)
10-14 years	948.07 (701.2, 1238.38)	0.93 (0.69, 1.21)	1062.01 (806.06, 1375.16)	1.23 (0.94, 1.6)	1.72 (0.96, 2.48)
15-19 years	2821.8 (2162.12, 3699.11)	2.23 (1.71, 2.92)	2307.19 (1763.92, 2962.47)	3.09 (2.36, 3.97)	2.01 (1.14, 2.89)
20-24 years	5436.86 (4213.29, 7085.24)	4.12 (3.19, 5.37)	4283.11 (3326.86, 5568.99)	5.85 (4.55, 7.61)	2.21 (1.31, 3.12)
25-29 years	6872.21 (5235.83, 8941.28)	6.25 (4.76, 8.14)	7760.1 (5887.39, 10246.22)	8.97 (6.81, 11.85)	2.29 (1.41, 3.17)
30-34 years	6952.05 (5198.05, 9175.39)	7.88 (5.89, 10.4)	14029.33 (10656.1, 18583.18)	11.58 (8.8, 15.34)	2.36 (1.56, 3.16)
35-39 years	7835.84 (5839.61, 10430.69)	8.58 (6.39, 11.42)	14750.9 (11080.81, 19689.77)	13.92 (10.46, 18.58)	2.59 (1.88, 3.29)
40-44 years	6129.59 (4619.02, 8097.49)	9.14 (6.88, 12.07)	14519.66 (11127.26, 18823.66)	15.86 (12.16, 20.56)	2.76 (2.11, 3.42)
45-49 years	4985.65 (3840.25, 6435.68)	9.66 (7.44, 12.47)	19093.16 (14720.17, 24328.19)	17.31 (13.34, 22.05)	2.85 (2.23, 3.48)
50-54 years	4886.9 (3768.36, 6231.35)	10.24 (7.9, 13.06)	22149.19 (17220.17, 28134.49)	18.33 (14.25, 23.28)	2.85 (2.27, 3.43)
55-59 years	4601.9 (3534.58, 5857.01)	10.61 (8.15, 13.51)	20713.45 (16218.43, 26625.89)	18.84 (14.75, 24.22)	2.77 (2.26, 3.29)
60-64 years	3773.72 (2912.03, 4786.81)	10.68 (8.24, 13.55)	13828.63 (10783.86, 17346.1)	18.94 (14.77, 23.76)	2.67 (2.22, 3.11)
65-69 years	2880.93 (2189.69, 3716.85)	10.56 (8.03, 13.62)	14003.38 (10796.89, 17838.57)	18.26 (14.08, 23.26)	2.51 (2.12, 2.9)
70-74 years	1913.52 (1437.53, 2454.04)	10.17 (7.64, 13.04)	9182.56 (7020.34, 11665.2)	17.23 (13.17, 21.89)	2.37 (2.03, 2.7)
75-79 years	1116.95 (840.84, 1466.35)	9.81 (7.39, 12.88)	5303.17 (4035.82, 6908.73)	16.01 (12.19, 20.86)	2.19 (1.91, 2.47)
80-84 years	502.46 (385.99, 657.13)	9.49 (7.29, 12.41)	2979.33 (2327.86, 3872.99)	15.05 (11.76, 19.57)	2.03 (1.82, 2.24)
85-89 years	150.62 (115.47, 200.56)	8.93 (6.85, 11.89)	1320.98 (1024.2, 1736.04)	13.87 (10.75, 18.22)	1.83 (1.67, 2)

90-94 years	27.46 (21.08, 36.67)	8.95 (6.87, 11.95)	412.03 (316.77, 543.98)	14.05 (10.8, 18.55)	1.83 (1.7, 1.96)
95 plus years	2.96 (2.12, 4.04)	7.31 (5.25, 9.97)	83.9 (61.11, 114.46)	13.13 (9.56, 17.91)	2.21 (2.09, 2.34)
**YLDs**					
**Gender**					
Female	5337.82 (3515.73, 7616.34)	0.98 (0.65, 1.4)	13993.66 (9318.47, 19985.81)	1.54 (1.02, 2.21)	2.44 (1.8, 3.08)
Male	4811.99 (3141.85, 6977.4)	0.85 (0.56, 1.22)	12936.49 (8585.24, 18420.18)	1.4 (0.93, 1.99)	2.48 (1.87, 3.08)
**Age**					
<5 years	2.22 (1.18, 3.79)	0 (0, 0)	2.05 (1.12, 3.46)	0 (0, 0)	1.08 (0.66, 1.5)
5-9 years	40.37 (24.12, 63.56)	0.04 (0.02, 0.06)	45.29 (27.47, 70.91)	0.05 (0.03, 0.07)	1.3 (0.69, 1.91)
10-14 years	154.36 (97.78, 230.8)	0.15 (0.1, 0.23)	169.79 (107.99, 251.49)	0.2 (0.13, 0.29)	1.65 (0.9, 2.41)
15-19 years	455.36 (284.99, 660.32)	0.36 (0.22, 0.52)	367.89 (231, 535.6)	0.49 (0.31, 0.72)	1.97 (1.1, 2.84)
20-24 years	893.08 (565.43, 1287.9)	0.68 (0.43, 0.98)	688.93 (426.33, 1007.1)	0.94 (0.58, 1.38)	2.14 (1.24, 3.04)
25-29 years	1129.31 (708.62, 1678.35)	1.03 (0.64, 1.53)	1249.86 (777.63, 1856.91)	1.45 (0.9, 2.15)	2.22 (1.35, 3.1)
30-34 years	1141.72 (715.47, 1697.59)	1.29 (0.81, 1.92)	2258.53 (1367.07, 3465.85)	1.86 (1.13, 2.86)	2.29 (1.5, 3.09)
35-39 years	1286.53 (769.72, 1961.82)	1.41 (0.84, 2.15)	2372.93 (1415.04, 3623.12)	2.24 (1.34, 3.42)	2.51 (1.81, 3.21)
40-44 years	1007.23 (613.32, 1514.3)	1.5 (0.91, 2.26)	2334.48 (1404.48, 3518.51)	2.55 (1.53, 3.84)	2.68 (2.03, 3.33)
45-49 years	812.54 (501.6, 1255.38)	1.57 (0.97, 2.43)	3064.04 (1894.39, 4661.27)	2.78 (1.72, 4.23)	2.78 (2.16, 3.4)
50-54 years	788.99 (495.7, 1179.32)	1.65 (1.04, 2.47)	3513.91 (2239.26, 5186.74)	2.91 (1.85, 4.29)	2.77 (2.19, 3.34)
55-59 years	742.47 (467.77, 1106.97)	1.71 (1.08, 2.55)	3295.64 (2115.75, 4863.93)	3 (1.92, 4.42)	2.7 (2.19, 3.2)
60-64 years	612.69 (387.79, 900.81)	1.73 (1.1, 2.55)	2202.94 (1376.21, 3184.38)	3.02 (1.89, 4.36)	2.58 (2.14, 3.02)
65-69 years	469.51 (288.44, 700.52)	1.72 (1.06, 2.57)	2235.77 (1368.79, 3293.72)	2.91 (1.78, 4.29)	2.43 (2.04, 2.81)
70-74 years	313.88 (185.59, 467.44)	1.67 (0.99, 2.48)	1474.17 (860.37, 2178.1)	2.77 (1.61, 4.09)	2.28 (1.95, 2.61)
75-79 years	184.67 (109.91, 275.79)	1.62 (0.97, 2.42)	857.76 (512.54, 1273.3)	2.59 (1.55, 3.84)	2.1 (1.83, 2.37)
80-84 years	83.95 (52.4, 126.22)	1.58 (0.99, 2.38)	487.23 (305.56, 732.33)	2.46 (1.54, 3.7)	1.94 (1.74, 2.14)
85-89 years	25.37 (16.18, 37.91)	1.5 (0.96, 2.25)	218.78 (136.18, 322.63)	2.3 (1.43, 3.39)	1.76 (1.61, 1.92)
90-94 years	5.03 (3.08, 7.43)	1.64 (1, 2.42)	74.48 (45.37, 107.34)	2.54 (1.55, 3.66)	1.77 (1.64, 1.89)
95 plus years	0.56 (0.33, 0.85)	1.37 (0.81, 2.09)	15.68 (9.38, 23.43)	2.45 (1.47, 3.67)	2.19 (2.07, 2.31)

Notes: ASR: age-standardized rates; YLDs: years lived with disability; EAPCs: estimated annual percentage changes.

Table S52. Prevalence and YLDs of pancreatitis and its temporal trends from 1990, 2021 by gender, and age in China.

	1990		2021		EAPC 95%CI
	Number	ASR, per 100,000 persons	Number	ASR, per 100,000 persons	
**Prevalence**					
**Gender**					
Female	141104.44 (93749.6, 206033.99)	28.77 (19.08, 42.07)	195765.49 (138700.99, 276839.17)	19.13 (13.8, 26.34)	-1.45 (-1.95, -0.94)
Male	210905.59 (143636.88, 294776.97)	41.82 (28.19, 58.93)	292362.67 (204020.97, 413712.92)	29.21 (20.8, 40.36)	-1.29 (-1.76, -0.81)
**Age**					
<5 years	1532.24 (694.22, 3031.35)	1.37 (0.62, 2.71)	803.22 (392.68, 1543.75)	1.03 (0.51, 1.99)	-1.4 (-1.98, -0.81)
5-9 years	4337.37 (1997.41, 8062.94)	4.16 (1.92, 7.73)	2281.8 (1112.77, 4035.76)	2.38 (1.16, 4.21)	-2 (-2.65, -1.33)
10-14 years	6746.65 (3463.28, 12331.37)	6.6 (3.39, 12.06)	2901.34 (1440.91, 5245.84)	3.37 (1.67, 6.09)	-2.39 (-3.14, -1.63)
15-19 years	12064.45 (5691.33, 21416.14)	9.52 (4.49, 16.91)	3353.88 (1857.17, 6005.84)	4.49 (2.49, 8.04)	-2.68 (-3.5, -1.84)
20-24 years	17204.21 (8507.62, 30046.55)	13.03 (6.45, 22.76)	4359.6 (2436.88, 7200.51)	5.96 (3.33, 9.84)	-2.81 (-3.66, -1.95)
25-29 years	19379.14 (9921.11, 34725.39)	17.64 (9.03, 31.6)	7245.27 (4124.36, 12010.77)	8.38 (4.77, 13.89)	-2.67 (-3.46, -1.87)
30-34 years	20773.56 (11057.72, 37439.98)	23.54 (12.53, 42.43)	14749.36 (8531.66, 25505.23)	12.17 (7.04, 21.05)	-2.35 (-3.05, -1.65)
35-39 years	28342.65 (15256, 51147.65)	31.03 (16.7, 56)	18697.78 (10882.93, 31637.35)	17.65 (10.27, 29.86)	-1.99 (-2.61, -1.36)
40-44 years	27950.78 (16228.91, 48585.5)	41.66 (24.19, 72.41)	23650 (13882.61, 38570.13)	25.84 (15.17, 42.14)	-1.66 (-2.22, -1.11)
45-49 years	28328.83 (15673.9, 46859.53)	54.88 (30.36, 90.78)	40425.91 (22575.06, 66006.56)	36.64 (20.46, 59.83)	-1.43 (-1.92, -0.93)
50-54 years	34369.93 (19433.1, 59610.3)	72.04 (40.73, 124.94)	61181.5 (35934.7, 101323.94)	50.62 (29.73, 83.84)	-1.26 (-1.71, -0.81)
55-59 years	38996.73 (22146.29, 67254.98)	89.92 (51.06, 155.08)	72052.14 (43052.91, 117481.25)	65.54 (39.16, 106.86)	-1.14 (-1.56, -0.72)
60-64 years	36423.49 (20445.73, 60385.88)	103.07 (57.86, 170.88)	56605.84 (33936.4, 89477.51)	77.54 (46.48, 122.56)	-1.03 (-1.43, -0.63)
65-69 years	30594.83 (17917.26, 49700.17)	112.14 (65.67, 182.17)	67064.76 (39707.86, 106282.78)	87.43 (51.77, 138.56)	-0.92 (-1.3, -0.54)
70-74 years	22339.08 (13255.62, 36377.21)	118.71 (70.44, 193.31)	50239.29 (30608.98, 76825.53)	94.26 (57.43, 144.15)	-0.83 (-1.19, -0.47)
75-79 years	13892.47 (8504.36, 21492.14)	122.07 (74.73, 188.85)	32199.26 (20327.13, 47992.86)	97.22 (61.38, 144.91)	-0.8 (-1.17, -0.44)
80-84 years	6369.72 (4139.69, 9700.28)	120.25 (78.15, 183.12)	18740.04 (12584.19, 28124.12)	94.69 (63.58, 142.1)	-0.86 (-1.24, -0.47)
85-89 years	1971.61 (1239.99, 3012.09)	116.88 (73.51, 178.56)	8556.1 (5623.91, 13087.73)	89.82 (59.04, 137.39)	-0.94 (-1.34, -0.53)

90-94 years	347.63 (207.72, 547.8)	113.3 (67.7, 178.54)	2497.44 (1547.53, 3907.81)	85.18 (52.78, 133.28)	-1.02 (-1.46, -0.59)
95 plus years	44.66 (26.17, 76.98)	110.29 (64.63, 190.1)	523.63 (311.16, 875.97)	81.93 (48.69, 137.06)	-1.07 (-1.49, -0.65)
**YLDs**					
**Gender**					
Female	11293.74 (6263.54, 18217.41)	2.28 (1.27, 3.62)	15241.6 (8148.75, 25024.2)	1.52 (0.83, 2.46)	-1.45 (-1.88, -1.02)
Male	16172.36 (8625.43, 26531.68)	3.13 (1.67, 5.12)	21264.42 (11331.21, 35864.17)	2.18 (1.19, 3.62)	-1.28 (-1.71, -0.86)
**Age**					
<5 years	146.31 (71.45, 273.27)	0.13 (0.06, 0.24)	78.43 (39.14, 140.81)	0.1 (0.05, 0.18)	-1.32 (-1.81, -0.84)
5-9 years	363.86 (160.49, 715.83)	0.35 (0.15, 0.69)	194 (91.3, 364.93)	0.2 (0.1, 0.38)	-1.95 (-2.52, -1.37)
10-14 years	536.86 (263.31, 986.97)	0.52 (0.26, 0.96)	240.22 (120.04, 445.99)	0.28 (0.14, 0.52)	-2.23 (-2.89, -1.57)
15-19 years	951.89 (434, 1745.84)	0.75 (0.34, 1.38)	286.94 (145.93, 505)	0.38 (0.2, 0.68)	-2.36 (-3.07, -1.66)
20-24 years	1393.54 (669.36, 2481.85)	1.06 (0.51, 1.88)	397.78 (210.37, 677.22)	0.54 (0.29, 0.93)	-2.39 (-3.09, -1.68)
25-29 years	1666.32 (794.04, 3046.99)	1.52 (0.72, 2.77)	713.47 (395.73, 1220.18)	0.82 (0.46, 1.41)	-2.22 (-2.84, -1.59)
30-34 years	1907.3 (937.41, 3518.02)	2.16 (1.06, 3.99)	1521.56 (785.19, 2646.01)	1.26 (0.65, 2.18)	-1.95 (-2.48, -1.41)
35-39 years	2600.93 (1334.89, 4584.39)	2.85 (1.46, 5.02)	1843.9 (1020.4, 3111.68)	1.74 (0.96, 2.94)	-1.74 (-2.24, -1.23)
40-44 years	2417.34 (1207.32, 4411.99)	3.6 (1.8, 6.58)	2098.8 (1088.59, 3769.09)	2.29 (1.19, 4.12)	-1.57 (-2.06, -1.09)
45-49 years	2307.21 (1138.38, 4301.44)	4.47 (2.21, 8.33)	3305.97 (1629.29, 6058.66)	3 (1.48, 5.49)	-1.42 (-1.87, -0.96)
50-54 years	2614.04 (1207.01, 4921.87)	5.48 (2.53, 10.32)	4643.87 (2187.62, 8677.03)	3.84 (1.81, 7.18)	-1.27 (-1.68, -0.86)
55-59 years	2774.02 (1211.53, 5417.91)	6.4 (2.79, 12.49)	5067.57 (2245.72, 9738.37)	4.61 (2.04, 8.86)	-1.18 (-1.55, -0.81)
60-64 years	2518.47 (1213.41, 4572.06)	7.13 (3.43, 12.94)	3807.69 (1763.23, 6995.96)	5.22 (2.42, 9.58)	-1.11 (-1.48, -0.74)
65-69 years	2108.31 (1072.78, 3808.21)	7.73 (3.93, 13.96)	4514.93 (2184.35, 7951.6)	5.89 (2.85, 10.37)	-0.99 (-1.33, -0.64)
70-74 years	1546.24 (767.5, 2685.47)	8.22 (4.08, 14.27)	3371.9 (1697.45, 5846.2)	6.33 (3.18, 10.97)	-0.93 (-1.24, -0.62)
75-79 years	983.89 (511.47, 1625.07)	8.65 (4.49, 14.28)	2212.45 (1146.34, 3785.62)	6.68 (3.46, 11.43)	-0.91 (-1.2, -0.62)
80-84 years	458.42 (247.1, 773.65)	8.65 (4.66, 14.61)	1341.89 (736.79, 2275.38)	6.78 (3.72, 11.5)	-0.87 (-1.14, -0.6)
85-89 years	142.88 (76.54, 254.65)	8.47 (4.54, 15.1)	631.9 (338.05, 1085.93)	6.63 (3.55, 11.4)	-0.83 (-1.1, -0.57)
90-94 years	25.09 (12.77, 43.26)	8.18 (4.16, 14.1)	191.05 (99.6, 330.39)	6.52 (3.4, 11.27)	-0.81 (-1.09, -0.52)
95 plus years	3.18 (1.62, 5.54)	7.85 (4, 13.69)	41.71 (21.31, 71.77)	6.53 (3.33, 11.23)	-0.69 (-0.9, -0.47)

Notes: ASR: age-standardized rates; YLDs: years lived with disability; EAPCs: estimated annual percentage changes.

Table S53. Prevalence and YLDs of male infertility and its temporal trends from 1990, 2021 by gender, and age in China.

	1990		2021		EAPC 95%CI
	Number	ASR, per 100,000 persons	Number	ASR, per 100,000 persons	
**Prevalence**					
**Age**					
15-19 years	129450.41 (26009.67, 324151.43)	102.2 (20.53, 255.91)	84367.32 (16824.59, 223373.64)	112.98 (22.53, 299.14)	0.29 (0.21, 0.36)
20-24 years	1206002.21 (428178.59, 2705587.43)	913.63 (324.38, 2049.67)	768008.18 (283950.21, 1667694.06)	1049.56 (388.05, 2279.08)	0.22 (0.15, 0.29)
25-29 years	1734105.14 (628704.66, 4026647.28)	1578.05 (572.12, 3664.27)	1539391.78 (563776.18, 3568106.64)	1780.01 (651.9, 4125.83)	0.08 (-0.01, 0.17)
30-34 years	2279808.42 (684899.87, 4887027.5)	2583.52 (776.14, 5538.06)	3244303.64 (1033776.32, 7003941.97)	2677.85 (853.28, 5781.06)	-0.07 (-0.12, -0.03)
35-39 years	3153901.29 (1123822.42, 6554037.22)	3452.96 (1230.39, 7175.5)	3689843.1 (1383314.57, 7752950.85)	3482.19 (1305.47, 7316.64)	-0.1 (-0.14, -0.06)
40-44 years	1638066.11 (604763.21, 3379136.44)	2441.44 (901.36, 5036.4)	2260638.95 (854371.86, 4624878.31)	2469.73 (933.4, 5052.65)	-0.13 (-0.21, -0.05)
45-49 years	115293.11 (23581.7, 318730.7)	223.35 (45.68, 617.47)	259251.51 (55768.37, 721656.74)	235 (50.55, 654.14)	-0.22 (-0.44, 0)
**YLDs**					
**Age**					
15-19 years	792.03 (102.54, 2843.68)	0.63 (0.08, 2.25)	525.67 (68.13, 1883.39)	0.7 (0.09, 2.52)	0.3 (0.21, 0.38)
20-24 years	7139.28 (1774.98, 20555.55)	5.41 (1.34, 15.57)	4681.52 (1177.63, 12515.71)	6.4 (1.61, 17.1)	0.26 (0.18, 0.35)
25-29 years	9564.25 (2106.71, 25138.35)	8.7 (1.92, 22.88)	8732.21 (1978.21, 23132.05)	10.1 (2.29, 26.75)	0.12 (0.01, 0.22)
30-34 years	11874.09 (2769.14, 33583.68)	13.46 (3.14, 38.06)	17159.81 (4222.13, 48204.56)	14.16 (3.48, 39.79)	-0.05 (-0.11, 0)
35-39 years	16488.65 (4646.32, 45417.57)	18.05 (5.09, 49.72)	19400.9 (5674.1, 54441.11)	18.31 (5.35, 51.38)	-0.09 (-0.12, -0.05)
40-44 years	8678.56 (2283.47, 23544.71)	12.93 (3.4, 35.09)	12041.17 (3321.01, 32303.15)	13.15 (3.63, 35.29)	-0.13 (-0.21, -0.05)
45-49 years	617.77 (106.65, 2120.03)	1.2 (0.21, 4.11)	1389.57 (237.92, 4568.96)	1.26 (0.22, 4.14)	-0.23 (-0.45, -0.02)

Notes: ASR: age-standardized rates; YLDs: years lived with disability; EAPCs: estimated annual percentage changes.

Table S54. Prevalence and YLDs of female infertility and its temporal trends from 1990, 2021 by gender, and age in China.

	1990		2021		
	Number	ASR, per 100,000 persons	Number	ASR, per 100,000 persons	EAPC 95%CI
**Prevalence**					
**Age**					
15-19 years	100061.8 (3313.75, 449691.86)	79 (2.62, 355.02)	27740.41 (1964.75, 183098.63)	37.15 (2.63, 245.2)	-2.48 (-2.66, -2.31)
20-24 years	2336561.95 (633877.56, 5160700.24)	1770.11 (480.21, 3909.59)	1261671.94 (292798.39, 2883446.08)	1724.2 (400.14, 3940.53)	-0.12 (-0.24, -0.01)
25-29 years	3940727.35 (1152628.46, 10371245.02)	3586.08 (1048.9, 9437.89)	3194502.89 (911981.76, 8277048.28)	3693.83 (1054.53, 9570.83)	0.1 (0.01, 0.19)
30-34 years	5679346.22 (1367834.15, 12819073.55)	6435.93 (1550.05, 14526.8)	8253470.85 (1817283.42, 19559823.49)	6812.42 (1499.99, 16144.69)	0.09 (0.04, 0.13)
35-39 years	8505478.79 (2779548.68, 18005358.08)	9311.98 (3043.11, 19712.66)	10289599.87 (3156880.11, 22330459.76)	9710.54 (2979.22, 21073.78)	0.08 (0.06, 0.1)
40-44 years	4186760.32 (1171160.49, 9128813.82)	6240.11 (1745.54, 13605.94)	6168397.14 (1806951.01, 13408928.1)	6738.94 (1974.08, 14649.17)	0.08 (-0.02, 0.18)
45-49 years	47344.74 (3815.65, 381955.25)	91.72 (7.39, 739.95)	121616.99 (9990.54, 970979.69)	110.24 (9.06, 880.14)	-0.42 (-1.03, 0.2)
**YLDs**					
**Age**					
15-19 years	578.63 (11.4, 2708.66)	0.46 (0.01, 2.14)	158.91 (7.13, 1134.49)	0.21 (0.01, 1.52)	-2.54 (-2.72, -2.36)
20-24 years	13195.36 (2619.28, 39522.23)	10 (1.98, 29.94)	7289.52 (1176.13, 21736.67)	9.96 (1.61, 29.71)	-0.1 (-0.22, 0.03)
25-29 years	21081.05 (4211.48, 63479.89)	19.18 (3.83, 57.77)	17404.4 (3346.82, 54371.16)	20.12 (3.87, 62.87)	0.12 (0.02, 0.22)
30-34 years	29032.99 (5906.68, 80976.26)	32.9 (6.69, 91.76)	42612.01 (8141.26, 122173.47)	35.17 (6.72, 100.84)	0.1 (0.05, 0.16)
35-39 years	43634.74 (11094.61, 121150.87)	47.77 (12.15, 132.64)	53026.51 (12550.86, 150704.92)	50.04 (11.84, 142.22)	0.09 (0.07, 0.11)
40-44 years	21703.67 (4523.03, 66370.56)	32.35 (6.74, 98.92)	32114.88 (6881.01, 98310.36)	35.09 (7.52, 107.4)	0.09 (-0.01, 0.19)
45-49 years	249.8 (16.58, 2054.83)	0.48 (0.03, 3.98)	645.41 (42.46, 5001.6)	0.59 (0.04, 4.53)	-0.42 (-1.03, 0.2)

Notes: ASR: age-standardized rates; YLDs: years lived with disability; EAPCs: estimated annual percentage changes.

Table S55. Prevalence and YLDs of depressive disorders and its temporal trends from 1990, 2021 by gender, and age in China.

	1990		2021		EAPC 95%CI
	Number	ASR, per 100,000 persons	Number	ASR, per 100,000 persons	
**Prevalence**					
**Gender**					
Female	21543975.83 (19447809.3, 24046533.98)	3900.99 (3528.46, 4325.69)	32504067.24 (29097247.62, 36150365.44)	3543.44 (3194.8, 3960.41)	-0.45 (-0.5, -0.39)
Male	12935413.72 (11591162.85, 14451662.38)	2277.12 (2037.51, 2538.67)	20610588.88 (18371774.89, 23115571.73)	2222.58 (1992.07, 2475.33)	-0.45 (-0.54, -0.36)
**Age**					
<5 years	909.72 (462.7, 1467.57)	0.81 (0.41, 1.31)	755.82 (392.57, 1219.58)	0.97 (0.51, 1.57)	0.37 (0.26, 0.49)
5-9 years	62145.75 (40408.97, 89952.08)	59.6 (38.75, 86.26)	58110.34 (37261.61, 84103.2)	60.68 (38.91, 87.82)	0.39 (0.23, 0.56)
10-14 years	600824.79 (414139.65, 808328.16)	587.36 (404.86, 790.21)	435050.33 (301632.02, 583100.31)	504.74 (349.95, 676.51)	-0.14 (-0.34, 0.06)
15-19 years	2379531.15 (1800822.15, 3018541.98)	1878.6 (1421.72, 2383.09)	1021620.34 (779072.84, 1315933.74)	1368.14 (1043.32, 1762.28)	-1.02 (-1.12, -0.93)
20-24 years	3987014.62 (3058973.34, 5176974.63)	3020.44 (2317.39, 3921.92)	1504822.61 (1155554.5, 1953959.77)	2056.49 (1579.18, 2670.29)	-1.55 (-1.74, -1.35)
25-29 years	3728785.81 (3008603.24, 4505636.9)	3393.22 (2737.85, 4100.15)	2145435.06 (1731682.25, 2598606.01)	2480.79 (2002.36, 3004.79)	-1.27 (-1.47, -1.07)
30-34 years	3326597.62 (2729270.31, 4010901.6)	3769.76 (3092.86, 4545.22)	3612180.65 (2996018.78, 4310275.47)	2981.5 (2472.92, 3557.7)	-1.03 (-1.21, -0.85)
35-39 years	3919211.83 (3305194.04, 4593105.93)	4290.84 (3618.6, 5028.63)	3784124.09 (3144191.74, 4520004.28)	3571.17 (2967.25, 4265.63)	-0.89 (-1.03, -0.75)
40-44 years	3193061.5 (2597987.98, 3872445.17)	4759.06 (3872.14, 5771.64)	3889437.57 (3139892.98, 4754474.27)	4249.19 (3430.31, 5194.23)	-0.7 (-0.81, -0.59)
45-49 years	2610966.77 (2210813.42, 3097360.36)	5058.15 (4282.95, 6000.43)	5476283.73 (4572545.58, 6499622.57)	4963.93 (4144.74, 5891.53)	-0.4 (-0.48, -0.32)
50-54 years	2519868.04 (2191211.76, 2944299.2)	5281.54 (4592.69, 6171.13)	6742638.2 (5769735.86, 7882550.14)	5578.93 (4773.94, 6522.1)	-0.14 (-0.22, -0.06)
55-59 years	2376880.93 (2018200.23, 2765411.55)	5480.59 (4653.54, 6376.46)	6761254.05 (5741570.75, 7912529.34)	6149.82 (5222.35, 7196.98)	0.06 (-0.05, 0.18)
60-64 years	2011154.15 (1663771.32, 2433113.02)	5691.28 (4708.24, 6885.36)	4781509.97 (4016449.07, 5740800.92)	6549.55 (5501.6, 7863.55)	0.2 (0.06, 0.34)
65-69 years	1593417.74 (1341694.79, 1920441.91)	5840.59 (4917.91, 7039.28)	5121424.87 (4370123.59, 6102941.38)	6676.91 (5697.42, 7956.53)	0.25 (0.11, 0.39)
70-74 years	1094751.46 (921720.29, 1295445.69)	5817.68 (4898.17, 6884.21)	3533419.83 (2963282.27, 4161795.1)	6629.75 (5560, 7808.77)	0.29 (0.16, 0.42)
75-79 years	650738.42 (538823.41, 775683.47)	5717.91 (4734.54, 6815.78)	2148913.44 (1782922.46, 2554742.65)	6488.47 (5383.39, 7713.84)	0.34 (0.22, 0.47)
80-84 years	301384.73 (248141.33, 359380.93)	5689.59 (4684.45, 6784.45)	1257114.29 (1023614.35, 1519050.81)	6351.67 (5171.9, 7675.13)	0.38 (0.27, 0.48)
85-89 years	99972.66 (85123.08, 118811.34)	5926.57 (5046.26, 7043.36)	606235.69 (516369.12, 713407.4)	6364.18 (5420.77, 7489.25)	0.29 (0.2, 0.38)

90-94 years	19426.52 (15977.21, 23342.02)	6331.53 (5207.32, 7607.67)	191012.9 (155684.85, 229265.11)	6514.79 (5309.87, 7819.44)	0.18 (0.1, 0.26)
95 plus years	2745.34 (2046.92, 3544.78)	6779.95 (5055.11, 8754.26)	43312.34 (31547.51, 55767.7)	6777.1 (4936.25, 8726)	0.1 (0.02, 0.17)
**YLDs**					
**Gender**					
Female	3432316.88 (2382222.68, 4634669.96)	608.67 (425.43, 818.52)	4837434.48 (3418220.33, 6553138.02)	533.04 (378.22, 723.93)	-0.53 (-0.63, -0.43)
Male	1994351.78 (1381739.15, 2689934.74)	343.83 (242.01, 468.06)	3028508.04 (2136876.48, 4116003.85)	330.6 (233.39, 450.37)	-0.55 (-0.66, -0.44)
**Age**					
<5 years	160.16 (68.07, 297.82)	0.14 (0.06, 0.27)	133.07 (55.16, 248.77)	0.17 (0.07, 0.32)	0.36 (0.24, 0.48)
5-9 years	11053.2 (5696.51, 19483)	10.6 (5.46, 18.68)	10220.95 (5119.21, 17713.52)	10.67 (5.35, 18.5)	0.37 (0.18, 0.56)
10-14 years	111251.16 (68087.3, 170882.54)	108.76 (66.56, 167.05)	78053.32 (45356.3, 119348.5)	90.56 (52.62, 138.47)	-0.19 (-0.43, 0.05)
15-19 years	442393.55 (283121.44, 654302.4)	349.26 (223.52, 516.56)	180085.94 (113026.29, 266792.36)	241.17 (151.36, 357.28)	-1.19 (-1.3, -1.08)
20-24 years	721855.43 (466855.02, 1068110.52)	546.86 (353.68, 809.17)	253125.28 (162121.14, 374928.64)	345.92 (221.56, 512.38)	-1.84 (-2.05, -1.63)
25-29 years	635724.51 (413331.22, 936376.85)	578.51 (376.13, 852.11)	337214.27 (222054.77, 488393.11)	389.92 (256.76, 564.73)	-1.58 (-1.8, -1.34)
30-34 years	537662.5 (343587.83, 796705.06)	609.29 (389.36, 902.84)	539244.66 (347744.67, 785128.08)	445.09 (287.03, 648.05)	-1.31 (-1.53, -1.09)
35-39 years	607552.97 (405600.05, 843022.37)	665.16 (444.06, 922.96)	546081.44 (355655.86, 762796.63)	515.35 (335.64, 719.87)	-1.12 (-1.31, -0.94)
40-44 years	477282.64 (307287.79, 671449.74)	711.36 (457.99, 1000.75)	553253.64 (357449.39, 784146.85)	604.43 (390.51, 856.68)	-0.85 (-0.99, -0.7)
45-49 years	380019.79 (258816.41, 526387.82)	736.2 (501.4, 1019.76)	787628.37 (541852.1, 1076194.34)	713.94 (491.16, 975.51)	-0.43 (-0.54, -0.32)
50-54 years	359459.19 (249751.33, 488830.64)	753.41 (523.47, 1024.57)	981900.73 (683774.27, 1347950.52)	812.43 (565.76, 1115.31)	-0.08 (-0.18, 0.01)
55-59 years	332740.82 (226565.23, 451082.94)	767.23 (522.41, 1040.1)	991146.67 (678227.45, 1358838.38)	901.51 (616.89, 1235.96)	0.19 (0.08, 0.3)
60-64 years	278191.31 (191137.07, 387190.11)	787.24 (540.89, 1095.69)	700977.9 (481567.29, 969039.56)	960.18 (659.63, 1327.36)	0.36 (0.23, 0.49)
65-69 years	222011.86 (153824.99, 302767.26)	813.77 (563.84, 1109.78)	752421.52 (529075.43, 1030624.99)	980.95 (689.77, 1343.65)	0.42 (0.3, 0.53)
70-74 years	154429.03 (106922.05, 207632.23)	820.66 (568.2, 1103.39)	523222.2 (363316.99, 711483.71)	981.72 (681.69, 1334.96)	0.45 (0.35, 0.55)
75-79 years	93299.03 (63769.46, 127841.87)	819.8 (560.33, 1123.32)	320951.41 (218826.9, 448427.73)	969.09 (660.73, 1353.99)	0.49 (0.4, 0.59)
80-84 years	43813.13 (30299.88, 60614.33)	827.11 (572.01, 1144.29)	187798.83 (129913.64, 261602.46)	948.87 (656.4, 1321.77)	0.49 (0.41, 0.57)
85-89 years	14569.21 (10390.36, 19796.28)	863.69 (615.96, 1173.56)	89096.17 (63571.49, 122816.5)	935.32 (667.36, 1289.31)	0.35 (0.29, 0.41)
90-94 years	2810.82 (1929.62, 3975.07)	916.11 (628.91, 1295.56)	27383.72 (19172.99, 38566.5)	933.96 (653.92, 1315.37)	0.2 (0.14, 0.25)
95 plus years	388.37 (242.81, 579.26)	959.12 (599.64, 1430.54)	6002.42 (3637.66, 8908.09)	939.2 (569.19, 1393.85)	0.08 (0.02, 0.13)

Notes: ASR: age-standardized rates; YLDs: years lived with disability; EAPCs: estimated annual percentage changes.

Table S56. Prevalence and YLDs of anxiety disorders and its temporal trends from 1990, 2021 by gender, and age in China.

	1990		2021		EAPC 95%CI
	Number	ASR, per 100,000 persons	Number	ASR, per 100,000 persons	
**Prevalence**					
**Gender**					
Female	24630898.61 (21109854.35, 28536227.55)	4255.55 (3686.92, 4891.48)	32739246.4 (27899916.47, 37860364.69)	4380.91 (3734.06, 5095.45)	-0.44 (-0.6, -0.27)
Male	15865374.54 (13657782.48, 18434811.03)	2600.62 (2254.12, 2980.15)	20393140.67 (17503200.5, 23789424.14)	2632.79 (2243.57, 3076.79)	-0.29 (-0.39, -0.18)
**Age**					
<5 years	124765.65 (87538.09, 172900.43)	111.59 (78.29, 154.64)	102247.58 (72108.65, 140254.91)	131.65 (92.84, 180.58)	0.38 (0.17, 0.59)
5-9 years	1717816 (1231356.22, 2376031.37)	1647.34 (1180.84, 2278.56)	1641320.3 (1167433.37, 2218630.45)	1713.81 (1218.99, 2316.61)	0.25 (0.03, 0.48)
10-14 years	4176737.85 (3019882.18, 5602963.63)	4083.14 (2952.21, 5477.4)	3646123.57 (2655842.23, 4910638.66)	4230.19 (3081.28, 5697.26)	0.1 (-0.13, 0.32)
15-19 years	5927231.18 (4689093.14, 7473830.24)	4679.45 (3701.96, 5900.46)	3595383.78 (2769833.25, 4542364.41)	4814.89 (3709.33, 6083.08)	-0.21 (-0.37, -0.05)
20-24 years	5432651.78 (4144710.66, 7013781.1)	4115.62 (3139.91, 5313.43)	3026024.99 (2299209.6, 3979335.16)	4135.37 (3142.11, 5438.17)	-0.67 (-0.83, -0.51)
25-29 years	4097338.72 (3062016.66, 5432061.78)	3728.6 (2786.45, 4943.21)	3216337.17 (2392805.68, 4305845.05)	3719.08 (2766.82, 4978.89)	-0.85 (-1.08, -0.63)
30-34 years	3151653.2 (2429932.36, 4037664.15)	3571.51 (2753.64, 4575.55)	4438786.43 (3413098.61, 5719565.48)	3663.78 (2817.17, 4720.93)	-0.73 (-0.96, -0.5)
35-39 years	3285999.19 (2575294.45, 4148010.67)	3597.58 (2819.49, 4541.33)	3877302.55 (3028407.34, 4861301.83)	3659.1 (2857.98, 4587.72)	-0.52 (-0.7, -0.35)
40-44 years	2456528.55 (1821663.31, 3183817.34)	3661.31 (2715.08, 4745.28)	3400351.01 (2524198.53, 4459906.42)	3714.86 (2757.67, 4872.42)	-0.32 (-0.43, -0.2)
45-49 years	1944318.42 (1452006.43, 2494875.82)	3766.68 (2812.93, 4833.26)	4259821.03 (3189705.23, 5510750.75)	3861.28 (2891.28, 4995.17)	-0.27 (-0.4, -0.14)
50-54 years	1869042.17 (1482380.48, 2331102.34)	3917.43 (3107.01, 4885.89)	4874956.77 (3841296.62, 6094558.18)	4033.59 (3178.33, 5042.7)	-0.33 (-0.48, -0.19)
55-59 years	1777942.16 (1435867.45, 2212108.12)	4099.56 (3310.81, 5100.65)	4651226.95 (3724434.36, 5776496.23)	4230.6 (3387.62, 5254.11)	-0.47 (-0.66, -0.29)
60-64 years	1520040.09 (1165757.01, 2000763.32)	4301.5 (3298.93, 5661.88)	3190927.99 (2424361.01, 4157936.64)	4370.82 (3320.81, 5695.4)	-0.6 (-0.8, -0.39)
65-69 years	1230066.91 (935149, 1643747.62)	4508.74 (3427.74, 6025.07)	3499659.65 (2640012.32, 4699094.98)	4562.58 (3441.84, 6126.31)	-0.62 (-0.83, -0.41)
70-74 years	879209.02 (682719.47, 1149008.73)	4672.26 (3628.08, 6106.02)	2511852.99 (1952444.06, 3314638.48)	4712.98 (3663.37, 6219.25)	-0.6 (-0.79, -0.4)
75-79 years	548480.13 (435779.6, 690934.75)	4819.39 (3829.11, 6071.11)	1604826.74 (1280083.87, 2041153.69)	4845.65 (3865.11, 6163.1)	-0.53 (-0.7, -0.35)
80-84 years	261158.63 (209453.19, 324643.85)	4930.2 (3954.09, 6128.68)	980868.82 (777647.41, 1227932.33)	4955.92 (3929.13, 6204.23)	-0.41 (-0.58, -0.24)
85-89 years	80539.6 (62188.54, 100886.38)	4774.54 (3686.65, 5980.74)	459398.47 (353469.26, 580599.91)	4822.7 (3710.67, 6095.06)	-0.3 (-0.48, -0.11)

90-94 years	13207.06 (10039.33, 17270.58)	4304.47 (3272.04, 5628.86)	130390.42 (99403.25, 168359.78)	4447.17 (3390.3, 5742.17)	-0.18 (-0.4, 0.04)
95 plus years	1546.84 (1104.01, 2070.81)	3820.09 (2726.49, 5114.1)	24579.86 (17281.2, 33546.79)	3846.02 (2704, 5249.08)	-0.12 (-0.31, 0.07)
**YLDs**					
**Gender**					
Female	2961763.45 (2055326.5, 4084621.45)	508.11 (355.66, 697.99)	3869328.81 (2705314.11, 5246191.67)	525.58 (367.22, 715.9)	-0.41 (-0.57, -0.25)
Male	1934179.49 (1343229.31, 2678401.44)	314.48 (219.39, 429.27)	2445120.84 (1698436.91, 3323374.15)	318.79 (218.97, 438.6)	-0.28 (-0.38, -0.17)
**Age**					
<5 years	15671.52 (9491.81, 23689.37)	14.02 (8.49, 21.19)	12933.81 (7660.61, 19590.93)	16.65 (9.86, 25.22)	0.41 (0.2, 0.62)
5-9 years	216132.54 (130722.13, 317101.4)	207.27 (125.36, 304.09)	207606.19 (127762.26, 308541.58)	216.77 (133.4, 322.17)	0.27 (0.04, 0.5)
10-14 years	521535.47 (319503.39, 758736.84)	509.85 (312.34, 741.73)	457718.8 (276527.98, 677669.59)	531.04 (320.82, 786.22)	0.11 (-0.11, 0.34)
15-19 years	736571.82 (477156.99, 1082609.17)	581.51 (376.71, 854.7)	447601.17 (285308.92, 665601.95)	599.42 (382.08, 891.37)	-0.2 (-0.36, -0.04)
20-24 years	668060.9 (429522.81, 973149.34)	506.1 (325.39, 737.23)	373935.27 (234047.37, 552320.29)	511.02 (319.85, 754.8)	-0.65 (-0.81, -0.49)
25-29 years	500588.97 (313730.92, 733546.65)	455.54 (285.5, 667.53)	394863.11 (244403.35, 588571.96)	456.58 (282.61, 680.57)	-0.83 (-1.06, -0.61)
30-34 years	382480.68 (246764.95, 552670.46)	433.43 (279.64, 626.3)	541596.61 (356485.46, 792562.58)	447.03 (294.24, 654.18)	-0.71 (-0.93, -0.48)
35-39 years	395737.28 (256621.71, 563589.31)	433.26 (280.96, 617.03)	470265.34 (304111.43, 681106.7)	443.8 (287, 642.78)	-0.5 (-0.67, -0.33)
40-44 years	293882.11 (179827.06, 432765.89)	438.01 (268.02, 645.01)	408757.75 (246852.58, 610683.85)	446.57 (269.68, 667.17)	-0.29 (-0.41, -0.18)
45-49 years	230974.62 (144251.72, 334832.31)	447.46 (279.45, 648.66)	507958.25 (318217.9, 753517.25)	460.43 (288.45, 683.02)	-0.26 (-0.38, -0.13)
50-54 years	220375.93 (143498.03, 312439.49)	461.9 (300.77, 654.86)	574986.03 (375961.23, 820446.05)	475.75 (311.07, 678.85)	-0.33 (-0.47, -0.18)
55-59 years	207448.4 (139484.58, 291428.35)	478.33 (321.62, 671.97)	541989.74 (361782.74, 773571.81)	492.98 (329.07, 703.62)	-0.47 (-0.65, -0.29)
60-64 years	174737.6 (113549.53, 255969.22)	494.48 (321.33, 724.36)	366697.3 (240406.07, 541937.37)	502.29 (329.3, 742.33)	-0.6 (-0.8, -0.39)
65-69 years	138710.91 (89439.01, 204659.54)	508.44 (327.83, 750.17)	394541.23 (257326.44, 577559.77)	514.37 (335.48, 752.98)	-0.62 (-0.83, -0.41)
70-74 years	96845.27 (64923.27, 138024.33)	514.65 (345.01, 733.48)	276604.96 (186638.27, 391068)	518.99 (350.19, 733.76)	-0.6 (-0.79, -0.4)
75-79 years	59048.58 (41165.49, 82706.42)	518.85 (361.71, 726.73)	172385.72 (116915.35, 240307.48)	520.51 (353.02, 725.59)	-0.53 (-0.71, -0.36)
80-84 years	27417.69 (18579.46, 38572.76)	517.6 (350.75, 728.18)	102402.02 (70598.79, 143531.26)	517.39 (356.71, 725.2)	-0.42 (-0.59, -0.25)
85-89 years	8253.17 (5420.87, 11713.36)	489.26 (321.36, 694.39)	46486.84 (31174, 65240.61)	488.01 (327.26, 684.89)	-0.33 (-0.51, -0.14)
90-94 years	1319.53 (859.87, 1860.49)	430.06 (280.25, 606.37)	12795.24 (8425.97, 17924.27)	436.4 (287.38, 611.34)	-0.22 (-0.44, 0)
95 plus years	149.96 (97.5, 217)	370.34 (240.79, 535.92)	2324.27 (1540.88, 3342.9)	363.68 (241.1, 523.06)	-0.18 (-0.37, 0.01)

Notes: ASR: age-standardized rates; YLDs: years lived with disability; EAPCs: estimated annual percentage changes.

Table S57. Prevalence and YLDs of opioid use disorders and its temporal trends from 1990, 2021 by gender, and age in China.

	1990		2021		EAPC 95%CI
	Number	ASR, per 100,000 persons	Number	ASR, per 100,000 persons	
**Prevalence**					
**Gender**					
Female	1329304.68 (1106812.23, 1568985.75)	218.94 (186.15, 254.5)	790717.34 (646483.56, 942570.3)	102.29 (82.22, 124)	-3.26 (-3.61, -2.92)
Male	1068453.75 (897710.38, 1254084.99)	166.96 (143.03, 193.44)	681550.32 (579199.51, 793300.4)	86.63 (72.3, 102.07)	-3.36 (-3.81, -2.9)
**Age**					
<5 years	101964.35 (69615.18, 142367.97)	80.5 (54.96, 112.4)	29651.87 (18872.13, 43457.03)	39.71 (25.27, 58.2)	-3.35 (-3.79, -2.91)
5-9 years	473171.17 (334452.34, 646429.73)	358.46 (253.37, 489.72)	131263.08 (88365.55, 189784.55)	179.38 (120.76, 259.36)	-3.38 (-3.83, -2.92)
10-14 years	488336.46 (372744.85, 622367.57)	444.39 (339.2, 566.36)	195860.76 (143416.01, 257959.7)	226.48 (165.83, 298.28)	-3.35 (-3.8, -2.89)
15-19 years	340419.64 (257572.28, 439823.59)	385.77 (291.89, 498.42)	242518.15 (176699.51, 324744.16)	200.17 (145.85, 268.04)	-3.26 (-3.7, -2.82)
20-24 years	297430.02 (231203.07, 387824.3)	325.63 (253.13, 424.6)	175808.65 (132546.49, 231031.56)	165.91 (125.09, 218.03)	-3.25 (-3.66, -2.84)
25-29 years	185907.49 (136499.61, 243034.45)	277.08 (203.44, 362.23)	124435.27 (87730.72, 168434.59)	135.94 (95.85, 184.01)	-3.31 (-3.68, -2.93)
30-34 years	130745.82 (99006.45, 170416.58)	253.29 (191.8, 330.14)	131586.38 (96017.26, 174911.44)	119.28 (87.03, 158.55)	-3.33 (-3.66, -3)
35-39 years	111991.17 (82844.13, 149305.64)	234.73 (173.64, 312.94)	132111.37 (90257.19, 180629.62)	109.31 (74.68, 149.45)	-3.28 (-3.57, -3)
40-44 years	88399.34 (66141.9, 115519.34)	203.83 (152.51, 266.36)	107010.53 (76470.42, 143947.27)	97.33 (69.56, 130.93)	-3.06 (-3.29, -2.83)
45-49 years	62043.99 (45208.33, 85351.74)	175.58 (127.93, 241.53)	62001.3 (42622.59, 89457.69)	84.93 (58.38, 122.54)	-2.84 (-3.01, -2.68)
50-54 years	47768.74 (36710.93, 60870.66)	175.09 (134.56, 223.12)	59242.45 (42277.04, 78591.23)	77.24 (55.12, 102.46)	-3.16 (-3.33, -2.99)
55-59 years	35703.75 (25858.51, 47936.81)	189.74 (137.42, 254.74)	38601.95 (27825.78, 52623.31)	72.43 (52.21, 98.74)	-3.74 (-3.95, -3.53)
60-64 years	21571.27 (16279.88, 28373.28)	189.54 (143.05, 249.31)	22464.1 (16777.48, 29350.02)	67.83 (50.66, 88.62)	-4.05 (-4.29, -3.81)
65-69 years	9268.25 (6675.84, 12306.02)	174.97 (126.03, 232.32)	12479.51 (8983.87, 16557.55)	63.05 (45.39, 83.66)	-4.13 (-4.39, -3.86)
70-74 years	2594.4 (1966.98, 3371.54)	153.8 (116.61, 199.87)	5449.67 (4164.08, 6967.31)	57.21 (43.71, 73.14)	-4.07 (-4.37, -3.78)
75-79 years	396.6 (297.9, 518.79)	129.26 (97.09, 169.09)	1485.06 (1131.46, 1928.13)	50.65 (38.59, 65.76)	-3.9 (-4.19, -3.61)

80-84 years	45.97 (31.8, 62.5)	113.52 (78.53, 154.36)	297.56 (210.99, 396.13)	46.56 (33.01, 61.98)	-3.55 (-3.76, -3.34)
85-89 years					
90-94 years					
95 plus years	553614.68 (369028.19, 732751.71)	90.52 (61.34, 118.95)	325016.12 (222018.29, 419138.67)	42.49 (28.53, 56.03)	-3.25 (-3.6, -2.91)
**YLDs**	453557.94 (301255.41, 596860.7)	70.34 (47.79, 91.53)	286719.62 (196447.38, 370077.97)	36.69 (24.86, 48.34)	-3.34 (-3.8, -2.88)
**Gender**					
Female	44218.22 (25017.97, 65527.57)	34.91 (19.75, 51.73)	12919.84 (6771.84, 20465.9)	17.3 (9.07, 27.41)	-3.33 (-3.76, -2.89)
Male	203675.48 (122173.25, 294239.58)	154.3 (92.55, 222.91)	56752.57 (31962.85, 88678.65)	77.56 (43.68, 121.19)	-3.36 (-3.81, -2.9)
**Age**	208554.38 (132916.67, 291547.05)	189.79 (120.95, 265.31)	83988.84 (53651.1, 118920.2)	97.12 (62.04, 137.51)	-3.33 (-3.78, -2.87)
<5 years	143969.67 (93680.96, 204883.28)	163.15 (106.16, 232.18)	103274.62 (66368.5, 148778.14)	85.24 (54.78, 122.8)	-3.25 (-3.69, -2.8)
5-9 years	125032.95 (82137.1, 178791.32)	136.89 (89.93, 195.74)	74144.96 (48053.44, 109126.59)	69.97 (45.35, 102.99)	-3.23 (-3.64, -2.82)
10-14 years	77423.28 (49945.41, 109197.8)	115.39 (74.44, 162.75)	51941.69 (33008.66, 76551.56)	56.75 (36.06, 83.63)	-3.29 (-3.66, -2.92)
15-19 years	53999.24 (36358.76, 76541)	104.61 (70.44, 148.28)	54515.16 (35079.48, 78663)	49.41 (31.8, 71.3)	-3.32 (-3.65, -3)
20-24 years	45788.94 (28831.57, 65793.93)	95.97 (60.43, 137.9)	54039.8 (32096, 80549.7)	44.71 (26.56, 66.65)	-3.28 (-3.56, -3)
25-29 years	35638.08 (22061.33, 50225.86)	82.17 (50.87, 115.81)	43130.39 (26369.72, 61529.64)	39.23 (23.99, 55.97)	-3.06 (-3.29, -2.83)
30-34 years	24660.18 (15298.87, 36205.16)	69.78 (43.29, 102.46)	24522.13 (14473.11, 37872.07)	33.59 (19.82, 51.88)	-2.86 (-3.02, -2.69)
35-39 years	18463.45 (11627.63, 26429.77)	67.68 (42.62, 96.88)	22859.87 (14307.25, 33111.06)	29.8 (18.65, 43.17)	-3.16 (-3.33, -2.99)
40-44 years	13475.05 (8570.21, 20237.66)	71.61 (45.54, 107.55)	14542.49 (9005.41, 21878.33)	27.29 (16.9, 41.05)	-3.75 (-3.96, -3.54)
45-49 years	7907.36 (5271.98, 11300.4)	69.48 (46.32, 99.29)	8213.65 (5241.44, 11595.28)	24.8 (15.83, 35.01)	-4.07 (-4.31, -3.83)
50-54 years	3315.57 (2153.28, 4781.46)	62.59 (40.65, 90.27)	4424.95 (2882.06, 6272.91)	22.36 (14.56, 31.69)	-4.16 (-4.43, -3.89)
55-59 years	900.98 (590.74, 1271.71)	53.41 (35.02, 75.39)	1876.29 (1230.54, 2659.57)	19.7 (12.92, 27.92)	-4.11 (-4.4, -3.82)
60-64 years	134.7 (89.52, 190.7)	43.9 (29.18, 62.15)	493.89 (327.04, 708.23)	16.84 (11.15, 24.16)	-3.96 (-4.24, -3.67)
65-69 years	15.08 (9.47, 22.32)	37.24 (23.38, 55.13)	94.62 (59.47, 136.56)	14.81 (9.3, 21.37)	-3.63 (-3.84, -3.42)
70-74 years	1329304.68 (1106812.23, 1568985.75)	218.94 (186.15, 254.5)	790717.34 (646483.56, 942570.3)	102.29 (82.22, 124)	-3.26 (-3.61, -2.92)
75-79 years	1068453.75 (897710.38, 1254084.99)	166.96 (143.03, 193.44)	681550.32 (579199.51, 793300.4)	86.63 (72.3, 102.07)	-3.36 (-3.81, -2.9)
80-84 years					

85-89 years	101964.35 (69615.18, 142367.97)	80.5 (54.96, 112.4)	29651.87 (18872.13, 43457.03)	39.71 (25.27, 58.2)	-3.35 (-3.79, -2.91)
90-94 years	473171.17 (334452.34, 646429.73)	358.46 (253.37, 489.72)	131263.08 (88365.55, 189784.55)	179.38 (120.76, 259.36)	-3.38 (-3.83, -2.92)
95 plus years	488336.46 (372744.85, 622367.57)	444.39 (339.2, 566.36)	195860.76 (143416.01, 257959.7)	226.48 (165.83, 298.28)	-3.35 (-3.8, -2.89)

Notes: ASR: age-standardized rates; YLDs: years lived with disability; EAPCs: estimated annual percentage changes.

Table S58. Prevalence and YLDs of varicella and herpes zoster and its temporal trends from 1990, 2021 by gender, and age in China.

	1990		2021		EAPC 95%CI
	Number	ASR, per 100,000 persons	Number	ASR, per 100,000 persons	
**Prevalence**					
**Gender**					
Female	386097.62 (322866.41, 461117.32)	75.9 (63.93, 89.89)	591998.44 (476190.04, 723166.65)	75.67 (63.79, 89.64)	-0.07 (-0.16, 0.01)
Male	368644.33 (312810.8, 436274.61)	68.6 (58.12, 80.69)	524221.04 (422353.9, 641319.14)	68.35 (57.9, 80.36)	-0.08 (-0.15, -0.01)
**Age**					
<5 years	195586.36 (173694.01, 213252.18)	174.93 (155.35, 190.73)	123405.64 (108776.58, 140018.77)	158.89 (140.05, 180.28)	-0.1 (-0.18, -0.02)
5-9 years	28288.01 (16057.81, 43845.02)	27.13 (15.4, 42.05)	26905.46 (15380.96, 41158.1)	28.09 (16.06, 42.98)	0.13 (0.11, 0.14)
10-14 years	23691.64 (15019.15, 34333.46)	23.16 (14.68, 33.56)	20000.7 (12728.78, 29106.39)	23.2 (14.77, 33.77)	0.01 (0, 0.02)
15-19 years	33916.77 (22136.01, 47710.19)	26.78 (17.48, 37.67)	19917.38 (13058.34, 27963.56)	26.67 (17.49, 37.45)	-0.01 (-0.02, -0.01)
20-24 years	41898.71 (23062.62, 64760.38)	31.74 (17.47, 49.06)	23045.62 (12742.88, 35640.88)	31.49 (17.41, 48.71)	-0.02 (-0.03, -0.01)
25-29 years	40692.8 (24340.69, 62577.23)	37.03 (22.15, 56.95)	31733.44 (18873.64, 49141.13)	36.69 (21.82, 56.82)	-0.02 (-0.03, -0.02)
30-34 years	37155.7 (26126.44, 52259.83)	42.11 (29.61, 59.22)	50772.29 (35580.27, 71449.85)	41.91 (29.37, 58.97)	-0.04 (-0.05, -0.02)
35-39 years	43347.57 (30168.26, 61051.97)	47.46 (33.03, 66.84)	49978.96 (34796.43, 70527.53)	47.17 (32.84, 66.56)	-0.05 (-0.08, -0.02)
40-44 years	35530.91 (21504.64, 54528.52)	52.96 (32.05, 81.27)	48270.81 (29200.97, 73973.73)	52.74 (31.9, 80.82)	-0.05 (-0.09, -0.01)
45-49 years	33440.01 (21523.2, 50330.86)	64.78 (41.7, 97.5)	71379.5 (45882.95, 107303.24)	64.7 (41.59, 97.26)	-0.09 (-0.17, -0.01)
50-54 years	40454.81 (28353.73, 55930.61)	84.79 (59.43, 117.23)	102582.9 (71916.26, 141753.76)	84.88 (59.5, 117.29)	-0.13 (-0.27, 0.01)
55-59 years	45857.86 (30028.69, 64885.97)	105.74 (69.24, 149.61)	116513.01 (76338.49, 164940.41)	105.98 (69.43, 150.02)	-0.16 (-0.34, 0.01)
60-64 years	45207.81 (27546.64, 67421.24)	127.93 (77.95, 190.79)	93460.31 (56908, 139250.69)	128.02 (77.95, 190.74)	-0.18 (-0.37, 0.01)
65-69 years	40839.59 (26651.7, 58755.96)	149.7 (97.69, 215.37)	114779.4 (74912.28, 165118.88)	149.64 (97.66, 215.27)	-0.18 (-0.38, 0.01)
70-74 years	31665.43 (22737.13, 42409.35)	168.28 (120.83, 225.37)	89443.57 (64120.25, 119821.84)	167.82 (120.31, 224.82)	-0.17 (-0.36, 0.02)
75-79 years	21353.32 (14942.52, 29269.37)	187.63 (131.3, 257.18)	61889.96 (43282.2, 84768.17)	186.87 (130.69, 255.95)	-0.16 (-0.35, 0.02)

80-84 years	11036 (6828.83, 16182.08)	208.34 (128.92, 305.49)	41015.67 (25409.4, 60490.47)	207.24 (128.38, 305.63)	-0.15 (-0.34, 0.03)
85-89 years	3886.82 (2432.11, 5717.21)	230.42 (144.18, 338.93)	21884.24 (13703.51, 32240.42)	229.74 (143.86, 338.46)	0 (-0.18, 0.18)
90-94 years	777.56 (498.78, 1235.17)	253.42 (162.56, 402.57)	7444.16 (4777.7, 11813.87)	253.89 (162.95, 402.93)	0.26 (0.02, 0.51)
95 plus years	114.26 (57.18, 211.71)	282.17 (141.21, 522.84)	1796.48 (893.88, 3341.54)	281.1 (139.87, 522.85)	0.43 (0.11, 0.76)
**YLDs**					
**Gender**					
Female	16224.24 (9404.55, 25178.23)	3.18 (1.86, 4.91)	28693.08 (16285.3, 44211.89)	3.18 (1.84, 4.91)	-0.08 (-0.19, 0.03)
Male	14855.01 (8533.22, 23447.01)	2.82 (1.63, 4.34)	24907.33 (14341.69, 39162.22)	2.81 (1.62, 4.34)	-0.1 (-0.19, -0.01)
**Age**					
<5 years	1349.61 (630.64, 2601.13)	1.21 (0.56, 2.33)	880.49 (411.19, 1680.12)	1.13 (0.53, 2.16)	-0.07 (-0.12, -0.02)
5-9 years	866.33 (427.74, 1514.29)	0.83 (0.41, 1.45)	798.56 (400.6, 1393.69)	0.83 (0.42, 1.46)	0.03 (0, 0.05)
10-14 years	1245.86 (597.87, 2226.06)	1.22 (0.58, 2.18)	1043.99 (530.85, 1852.02)	1.21 (0.62, 2.15)	0.01 (-0.01, 0.03)
15-19 years	1881.89 (936.09, 3188.68)	1.49 (0.74, 2.52)	1109.2 (545.67, 1963.81)	1.49 (0.73, 2.63)	0 (-0.01, 0.01)
20-24 years	2332.87 (1096.62, 4256.86)	1.77 (0.83, 3.22)	1286.87 (597.33, 2302.69)	1.76 (0.82, 3.15)	-0.01 (-0.03, 0.02)
25-29 years	2276.48 (1117.9, 4041.46)	2.07 (1.02, 3.68)	1779.54 (895.69, 3248.43)	2.06 (1.04, 3.76)	-0.02 (-0.03, -0.01)
30-34 years	2074.34 (1083.65, 3506)	2.35 (1.23, 3.97)	2843.18 (1454.14, 4926.91)	2.35 (1.2, 4.07)	-0.03 (-0.05, 0)
35-39 years	2401.75 (1301.23, 4002.21)	2.63 (1.42, 4.38)	2778.7 (1458.62, 4713.15)	2.62 (1.38, 4.45)	-0.03 (-0.07, 0)
40-44 years	1955.3 (983.4, 3462.28)	2.91 (1.47, 5.16)	2659.23 (1293.14, 4846.51)	2.91 (1.41, 5.29)	-0.05 (-0.09, -0.01)
45-49 years	1834.91 (915.99, 3081.02)	3.55 (1.77, 5.97)	3922.69 (2005.69, 6610.16)	3.56 (1.82, 5.99)	-0.08 (-0.16, 0)
50-54 years	2215.7 (1147.98, 3527.97)	4.64 (2.41, 7.39)	5604.07 (2933.99, 8793.44)	4.64 (2.43, 7.28)	-0.13 (-0.27, 0)
55-59 years	2484.03 (1265.47, 4010.42)	5.73 (2.92, 9.25)	6325.52 (3241.96, 10206.55)	5.75 (2.95, 9.28)	-0.16 (-0.33, 0.01)
60-64 years	2438.4 (1090.48, 3989.92)	6.9 (3.09, 11.29)	5029.7 (2340.96, 8305.93)	6.89 (3.21, 11.38)	-0.18 (-0.37, 0.01)
65-69 years	2165.9 (997.01, 3556.9)	7.94 (3.65, 13.04)	6085.23 (2942.36, 9975.42)	7.93 (3.84, 13.01)	-0.19 (-0.38, 0.01)
70-74 years	1657.54 (856.58, 2707.17)	8.81 (4.55, 14.39)	4674.56 (2414.1, 7742.44)	8.77 (4.53, 14.53)	-0.17 (-0.36, 0.02)
75-79 years	1102.22 (571.22, 1923.07)	9.68 (5.02, 16.9)	3184.63 (1663.75, 5482.55)	9.62 (5.02, 16.55)	-0.18 (-0.36, 0.01)
80-84 years	559.61 (281.35, 1017.15)	10.56 (5.31, 19.2)	2072.29 (1050.07, 3792.79)	10.47 (5.31, 19.16)	-0.17 (-0.35, 0.02)

85-89 years	193.1 (99.8, 345.17)	11.45 (5.92, 20.46)	1079.18 (565.58, 1948.1)	11.33 (5.94, 20.45)	-0.02 (-0.2, 0.16)
90-94 years	37.95 (19.77, 70.16)	12.37 (6.44, 22.87)	358.86 (180.01, 667.29)	12.24 (6.14, 22.76)	0.23 (-0.02, 0.48)
95 plus years	5.46 (2.27, 11.76)	13.48 (5.6, 29.04)	83.93 (36.32, 174.16)	13.13 (5.68, 27.25)	0.39 (0.05, 0.72)

Notes: ASR: age-standardized rates; YLDs: years lived with disability; EAPCs: estimated annual percentage changes.

Table S59. Acupuncture demands based on prevalence and YLDs in the worldwide of future forecasts using bayesian age-period-cohort model.

Year	Sex	Number	ASR
**Prevalence**			
2022	Both	6683087508.54 (6561099374.72, 6805075642.35)	78830.5 (77391.18, 80269.81)
2023	Both	6806995974.46 (6655169650.12, 6958822298.81)	79205.12 (77438.05, 80972.2)
2024	Both	6933187996.75 (6733936821.44, 7132439172.05)	79592.99 (77305.13, 81880.85)
2025	Both	7061165937.12 (6799299679.31, 7323032194.94)	79994.52 (77027.39, 82961.64)
2026	Both	7188366219.45 (6850856680.22, 7525875758.67)	80385.16 (76610.3, 84160.01)
2027	Both	7314894699.05 (6889942901.56, 7739846496.55)	80768.01 (76075.03, 85460.99)
2028	Both	7441583202.72 (6918436177.97, 7964730227.47)	81159.24 (75452.5, 86865.98)
2029	Both	7570218054.68 (6938556254.6, 8201879854.75)	81566.97 (74759.28, 88374.65)
2030	Both	7700414728.54 (6950154441.16, 8450675015.92)	81991.1 (74000.22, 89981.98)
2031	Both	7829515016.99 (6950808062.09, 8708221971.89)	82405.3 (73153.61, 91656.99)
2032	Both	7957753742.73 (6940597510.07, 8974909975.39)	82813.31 (72223.48, 93403.14)
2033	Both	8086452095.06 (6920855216.51, 9252048973.61)	83232.91 (71229.11, 95236.7)
2034	Both	8217027303.62 (6892803931.05, 9541250676.2)	83672.54 (70179.48, 97165.61)
2035	Both	8349126452.98 (6856006469.86, 9842246436.09)	84131.7 (69074.37, 99189.04)
2036	Both	8479930674.26 (6807907745.86, 10151953602.66)	84583.92 (67891.03, 101276.81)
2037	Both	8609757783.1 (6748495533.24, 10471020032.96)	85033.38 (66631.39, 103435.37)
2038	Both	8740288282.5 (6679095854.3, 10801480710.7)	85499.05 (65311.49, 105686.61)
2039	Both	8872961972.79 (6600667617.86, 11145256327.73)	85989.5 (63937.5, 108041.5)
2040	Both	9007414275.2 (6512665378.47, 11502163171.93)	86503.79 (62507.11, 110500.46)
2041	Both	9140622226.79 (6412560572.26, 11868683881.32)	87015.41 (60998.74, 113032.07)
2042	Both	9272879408.23 (6300225585.93, 12245533230.54)	87528.67 (59412.93, 115644.42)
2043	Both	9406067387.1 (6176838506.19, 12635296268.01)	88062.84 (57762.27, 118363.4)

2044	Both	9541622004.76 (6043081134.56, 13040162874.97)	88626.53 (56050.43, 121202.63)
2045	Both	9679287132.14 (5898391691.75, 13460182572.52)	89218.73 (54273.59, 124163.87)
2022	Female	3847457444.88 (3774309154.61, 3920605735.15)	89811.22 (88966.81, 90655.63)
2023	Female	3921760222.36 (3831289817.64, 4012230627.09)	90301.26 (88851.5, 91751.02)
2024	Female	3997408541.16 (3879334996.27, 4115482086.05)	90805.45 (88586.22, 93024.67)
2025	Female	4074158345.4 (3919505201.84, 4228811488.96)	91323.4 (88206.27, 94440.52)
2026	Female	4150610790.14 (3951640233.21, 4349581347.07)	91826.78 (87699.29, 95954.28)
2027	Female	4226825267.1 (3976524767.63, 4477125766.57)	92318.24 (87075.17, 97561.32)
2028	Female	4303274855.58 (3995231621.59, 4611318089.58)	92815.51 (86359.8, 99271.23)
2029	Female	4380928911.49 (4008979014.39, 4752878808.58)	93327.82 (85567.5, 101088.14)
2030	Female	4459612468.55 (4017717371.61, 4901507565.49)	93854.07 (84700.61, 103007.53)
2031	Female	4537851270.01 (4020096681.19, 5055605858.83)	94363.31 (83732.78, 104993.84)
2032	Female	4615773759.68 (4016152522.9, 5215394996.46)	94858.47 (82667.3, 107049.65)
2033	Female	4694099326.21 (4006596720.74, 5381601931.68)	95359.24 (81525.17, 109193.31)
2034	Female	4773607155.02 (3992083511.53, 5555130798.51)	95875.2 (80316.83, 111433.58)
2035	Female	4854125708.65 (3972371140.45, 5735880276.84)	96404.72 (79041.83, 113767.61)
2036	Female	4934069494.68 (3946006588.9, 5922132400.45)	96916.4 (77674.55, 116158.25)
2037	Female	5013610730.36 (3912961225.02, 6114260235.7)	97413.51 (76217.48, 118609.54)
2038	Female	5093691210.11 (3873953867.09, 6313428553.13)	97917.02 (74689.49, 121144.54)
2039	Female	5175124348.84 (3829500709, 6520747988.68)	98436.62 (73098.96, 123774.27)
2040	Female	5257727799.7 (3779286421.9, 6736169177.49)	98970.12 (71444.24, 126496)
2041	Female	5339777304.17 (3721851063.76, 6957703544.58)	99485.89 (69702.03, 129269.74)
2042	Female	5421426051.1 (3657090756.77, 7185761345.44)	99987.17 (67874.37, 132099.97)
2043	Female	5503749143.07 (3585647908.11, 7421850378.03)	100495.1 (65977.37, 135012.83)
2044	Female	5587577598.64 (3507887060.91, 7667268136.38)	101019.16 (64017.6, 138020.72)
2045	Female	5672793937.94 (3423474835.12, 7922113040.75)	101556.87 (61992.89, 141120.84)

2022	Male	2835630063.65 (2786790220.11, 2884469907.2)	67704.17 (67113.82, 68294.52)
2023	Male	2885235752.1 (2823879832.48, 2946591671.72)	67963.24 (66931.79, 68994.7)
2024	Male	2935779455.59 (2854601825.18, 3016957086)	68232.36 (66643.71, 69821)
2025	Male	2987007591.72 (2879794477.46, 3094220705.98)	68511.13 (66274.9, 70747.36)
2026	Male	3037755429.3 (2899216447.01, 3176294411.6)	68777.1 (65814.4, 71739.81)
2027	Male	3088069431.96 (2913418133.93, 3262720729.98)	69032.21 (65269.21, 72795.22)
2028	Male	3138308347.13 (2923204556.38, 3353412137.89)	69290.01 (64659.31, 73920.71)
2029	Male	3189289143.19 (2929577240.21, 3449001046.17)	69556.86 (63995.25, 75118.48)
2030	Male	3240802259.99 (2932437069.55, 3549167450.42)	69831.96 (63279.12, 76384.8)
2031	Male	3291663746.98 (2930711380.9, 3652616113.06)	70091.83 (62491.1, 77692.57)
2032	Male	3341979983.05 (2924444987.17, 3759514978.93)	70339.13 (61634, 79044.26)
2033	Male	3392352768.85 (2914258495.77, 3870447041.93)	70588.66 (60724.11, 80453.21)
2034	Male	3443420148.61 (2900720419.52, 3986119877.69)	70846.81 (59768.94, 81924.67)
2035	Male	3495000744.33 (2883635329.41, 4106366159.25)	71112.27 (58768.53, 83456.01)
2036	Male	3545861179.58 (2861901156.95, 4229821202.21)	71361.61 (57703.91, 85019.32)
2037	Male	3596147052.74 (2835534308.22, 4356759797.26)	71597.75 (56577.43, 86618.08)
2038	Male	3646597072.39 (2805141987.21, 4488052157.57)	71836.16 (55403.54, 88268.78)
2039	Male	3697837623.95 (2771166908.86, 4624508339.05)	72083.02 (54188.19, 89977.84)
2040	Male	3749686475.51 (2733378956.57, 4765993994.44)	72336.59 (52930.53, 91742.65)
2041	Male	3800844922.62 (2690709508.51, 4910980336.74)	72573.76 (51613.72, 93533.79)
2042	Male	3851453357.13 (2643134829.15, 5059771885.1)	72797.53 (50239.88, 95355.17)
2043	Male	3902318244.03 (2591190598.09, 5213445889.98)	73023.2 (48821.33, 97225.07)
2044	Male	3954044406.12 (2535194073.65, 5372894738.59)	73256.71 (47362.76, 99150.66)
2045	Male	4006493194.2 (2474916856.63, 5538069531.77)	73496.26 (45863.13, 101129.39)
**YLDs**			
2022	Both	126789588.89 (122994312.04, 130584865.74)	3739.25 (3626.05, 3852.45)

2023	Both	130721681.35 (126037713.47, 135405649.22)	3801 (3662.19, 3939.8)
2024	Both	134801642.15 (128692555.05, 140910729.26)	3864.47 (3684.65, 4044.29)
2025	Both	139014923.26 (130984796.23, 147045050.29)	3929.88 (3695.59, 4164.17)
2026	Both	143301530.36 (132902892.51, 153700168.21)	3995.96 (3695.71, 4296.21)
2027	Both	147657453.97 (134469477.8, 160845430.14)	4063.02 (3686.48, 4439.56)
2028	Both	152123944.73 (135738436.19, 168509453.27)	4131.96 (3669.5, 4594.42)
2029	Both	156758575.55 (136762255.82, 176754895.27)	4203.46 (3645.74, 4761.19)
2030	Both	161546744.37 (137518435.26, 185575053.49)	4277.69 (3615.38, 4940)
2031	Both	166414101.64 (137928958.04, 194899245.23)	4352.97 (3576.89, 5129.05)
2032	Both	171358421.2 (137975998.42, 204740843.98)	4429.58 (3530.31, 5328.85)
2033	Both	176430355.85 (137687202.92, 215173508.77)	4508.84 (3476.56, 5541.12)
2034	Both	181695973.58 (137093781.52, 226298165.64)	4591.61 (3415.98, 5767.24)
2035	Both	187144362.64 (136160172.86, 238128552.42)	4678.04 (3348.22, 6007.86)
2036	Both	192687088.21 (134792711.9, 250581464.51)	4766.13 (3271.29, 6260.96)
2037	Both	198324906.38 (132964087.23, 263685725.53)	4856.19 (3184.89, 6527.49)
2038	Both	204127196.44 (130695286.67, 277559106.21)	4949.88 (3089.61, 6810.16)
2039	Both	210166594.48 (127998342.77, 292334846.19)	5048.29 (2985.43, 7111.15)
2040	Both	216434976.1 (124825273.09, 308044679.1)	5151.54 (2871.55, 7431.54)
2041	Both	222830351.13 (121071825.71, 324588876.54)	5257.38 (2745.74, 7769.03)
2042	Both	229356327.9 (116697051.33, 342015604.38)	5366.16 (2607.28, 8125.05)
2043	Both	236104335.15 (111704790.98, 360503879.31)	5479.9 (2456.21, 8503.6)
2044	Both	243152830.11 (106076613.13, 380229047.1)	5599.9 (2291.88, 8907.92)
2045	Both	250494815.86 (99742225.73, 401247405.99)	5726.34 (2112.9, 9339.78)
2022	Female	193320953.29 (187428009.79, 199213896.78)	4436.41 (4370.04, 4502.78)
2023	Female	199771370.97 (192389224.86, 207153517.08)	4514.1 (4398.39, 4629.8)
2024	Female	206470252.76 (196702535.47, 216237970.05)	4593.86 (4414.31, 4773.4)

2025	Female	213406275.6 (200429537.43, 226383013.8)	4675.81 (4420.32, 4931.31)
2026	Female	220496464.39 (203564129.75, 237428799.02)	4758.26 (4415.7, 5100.82)
2027	Female	227745034.04 (206147337.56, 249342730.53)	4841.44 (4401.05, 5281.83)
2028	Female	235212028.68 (208252151.37, 262171905.98)	4926.41 (4377.63, 5475.18)
2029	Female	242978881.84 (209945074.09, 276012689.58)	5013.94 (4346.22, 5681.66)
2030	Female	251035047.4 (211194068.82, 290876025.98)	5104.06 (4306.74, 5901.38)
2031	Female	259274403.98 (211878214, 306670593.99)	5194.55 (4257.17, 6131.93)
2032	Female	267702753.65 (211967732.31, 323437774.98)	5285.6 (4197.53, 6373.68)
2033	Female	276396506.04 (211491372.63, 341301639.46)	5378.69 (4128.91, 6628.47)
2034	Female	285449843.58 (210477563.42, 360422123.75)	5474.76 (4051.85, 6897.68)
2035	Female	294854260.15 (208864802.82, 380843717.49)	5573.8 (3966.01, 7181.59)
2036	Female	304478560.81 (206500463.2, 402456658.42)	5673.13 (3869.18, 7477.09)
2037	Female	314330665.74 (203331472.69, 425329858.79)	5772.94 (3761.25, 7784.64)
2038	Female	324519045.99 (199371053.51, 449667038.46)	5875.05 (3643.25, 8106.85)
2039	Female	335151360.77 (194612780.22, 475689941.31)	5980.56 (3515.52, 8445.6)
2040	Female	346225136.87 (188967520.84, 503482752.89)	6089.38 (3377.55, 8801.2)
2041	Female	357588132.73 (182258273.41, 532917992.05)	6198.49 (3227.27, 9169.7)
2042	Female	369254948.51 (174400659.43, 564109237.58)	6308.03 (3064.49, 9551.56)
2043	Female	381368548.83 (165367412.46, 597369685.2)	6420.12 (2890, 9950.24)
2044	Female	394052273.5 (155093711.34, 633010835.67)	6536.01 (2703.92, 10368.1)
2045	Female	407313674.19 (143441719.43, 671185628.96)	6655.54 (2505.63, 10805.44)
2022	Male	320110542.18 (310422321.84, 329798762.52)	3027.52 (2984.04, 3071)
2023	Male	330493052.32 (318426938.34, 342559166.3)	3072.88 (2998.17, 3147.58)
2024	Male	341271894.91 (325395090.52, 357148699.31)	3119.28 (3004.3, 3234.27)
2025	Male	352421198.85 (331414333.66, 373428064.05)	3166.81 (3004.09, 3329.53)
2026	Male	363797994.75 (336467022.26, 391128967.23)	3214.42 (2997.21, 3431.64)

2027	Male	375402488.02 (340616815.37, 410188160.67)	3262.25 (2984.04, 3540.45)
2028	Male	387335973.41 (343990587.56, 430681359.26)	3310.89 (2965.41, 3656.37)
2029	Male	399737457.38 (346707329.91, 452767584.85)	3360.79 (2941.82, 3779.77)
2030	Male	412581791.77 (348712504.08, 476451079.47)	3411.99 (2913.3, 3910.68)
2031	Male	425688505.62 (349807172.01, 501569839.22)	3463.07 (2878.61, 4047.52)
2032	Male	439061174.85 (349943730.73, 528178618.96)	3514.14 (2837.8, 4190.49)
2033	Male	452826861.89 (349178575.55, 556475148.23)	3566.09 (2791.61, 4340.57)
2034	Male	467145817.16 (347571344.94, 586720289.39)	3619.48 (2740.42, 4498.54)
2035	Male	481998622.8 (345024975.69, 618972269.91)	3674.27 (2684.08, 4664.46)
2036	Male	497165649.01 (341293175.1, 653038122.92)	3728.78 (2621.24, 4836.32)
2037	Male	512655572.11 (336295559.91, 689015584.31)	3783.11 (2551.89, 5014.33)
2038	Male	528646242.43 (330066340.18, 727226144.67)	3838.37 (2476.76, 5199.98)
2039	Male	545317955.25 (322611122.99, 768024787.51)	3895.22 (2396.15, 5394.29)
2040	Male	562660112.96 (313792793.93, 811527431.99)	3953.59 (2309.83, 5597.34)
2041	Male	580418483.86 (303330099.12, 857506868.59)	4011.55 (2216.52, 5806.57)
2042	Male	598611276.36 (291097710.75, 906124841.96)	4069.18 (2116.17, 6022.18)
2043	Male	617472883.98 (277072203.45, 957873564.51)	4127.8 (2009.45, 6246.15)
2044	Male	637205103.62 (261170324.47, 1013239882.76)	4188.16 (1896.53, 6479.79)
2045	Male	657808490.05 (243183945.16, 1072433034.95)	4250.13 (1777.14, 6723.12)

Notes: ASR: age-standardized rates; YLDs: years lived with disability.

Table S60. Prevalence and YLDs of musculoskeletal disorders in the worldwide of future forecasts using bayesian age-period-cohort model.

Year	Sex	Number	ASR
**Prevalence**			
2022	Both	1300631581.65 (1273805201.48, 1327457961.82)	14805.24 (14499.75, 15110.72)
2023	Both	1324780742.85 (1292203830.57, 1357357655.13)	14794.31 (14430.35, 15158.28)
2024	Both	1349446207.46 (1307699357.56, 1391193057.35)	14784.14 (14326.56, 15241.72)
2025	Both	1374424790.86 (1320465701.69, 1428383880.02)	14775.03 (14194.7, 15355.35)
2026	Both	1399307040.49 (1330492781.31, 1468121299.68)	14765.42 (14038.95, 15491.89)
2027	Both	1423854835.41 (1337812187.16, 1509897483.67)	14755.06 (13862.97, 15647.16)
2028	Both	1448375336.15 (1342945995.36, 1553804676.94)	14744.95 (13671.04, 15818.85)
2029	Both	1473262563.77 (1346382847.89, 1600142279.65)	14736.03 (13466.17, 16005.9)
2030	Both	1498322249.43 (1347994537.57, 1648649961.29)	14728.61 (13249.88, 16207.34)
2031	Both	1523205339.33 (1347471290.58, 1698939388.09)	14721.68 (13021.95, 16421.41)
2032	Both	1547680581.35 (1344596429.67, 1750764733.03)	14714.82 (12782.36, 16647.29)
2033	Both	1572014974.28 (1339652735.48, 1804377213.09)	14708.81 (12532.67, 16884.95)
2034	Both	1596584610.8 (1332976116.6, 1860193104.99)	14704.31 (12274.04, 17134.59)
2035	Both	1621229206.14 (1324414845.36, 1918043566.92)	14701.64 (12007.02, 17396.26)
2036	Both	1645663016.13 (1313698126.16, 1977627906.11)	14700.48 (11731.38, 17669.57)
2037	Both	1669662747.45 (1300605874.75, 2038719620.15)	14700.37 (11446.59, 17954.14)
2038	Both	1693510585.17 (1285378225.39, 2101642944.94)	14701.77 (11153.35, 18250.19)
2039	Both	1717534741.49 (1268256619.79, 2166812863.19)	14705.06 (10852.15, 18557.97)
2040	Both	1741599281.82 (1249105011.82, 2234093551.81)	14710.53 (10543.2, 18877.86)
2041	Both	1765509180.18 (1227731291.74, 2303287068.62)	14718.55 (10226.53, 19210.57)
2042	Both	1789062300.56 (1203936502.26, 2374188098.86)	14728.7 (9901.43, 19555.97)
2043	Both	1812476056.16 (1177867378.97, 2447084733.35)	14741.1 (9568.01, 19914.19)

2044	Both	1836018335.58 (1149674189.48, 2522362481.68)	14755.79 (9226.23, 20285.36)
2045	Both	1859599700.02 (1119250434.18, 2599948965.86)	14773.08 (8876.07, 20670.09)
2022	Female	805242539.89 (788250408.57, 822234671.22)	17797.85 (17616.21, 17979.49)
2023	Female	820450909.52 (799897743.15, 841004075.88)	17786.29 (17486.3, 18086.28)
2024	Female	835987423.37 (809750437.25, 862224409.49)	17775.6 (17325.79, 18225.4)
2025	Female	851731845.19 (817909195.27, 885554495.12)	17765.72 (17142.56, 18388.88)
2026	Female	867424969.95 (824358948.96, 910490990.94)	17754.29 (16937.83, 18570.75)
2027	Female	882926749.29 (829127216.28, 936726282.3)	17740.66 (16712.87, 18768.46)
2028	Female	898438878.59 (832548484.61, 964329272.58)	17726.43 (16471.35, 18981.52)
2029	Female	914191501.27 (834912145.66, 993470856.88)	17712.79 (16215.87, 19209.72)
2030	Female	930060461.78 (836134794.22, 1023986129.33)	17699.66 (15947.35, 19451.98)
2031	Female	945813587.4 (836009814.77, 1055617360.03)	17685.45 (15664.92, 19705.99)
2032	Female	961308088.14 (834403812.99, 1088212363.3)	17669.37 (15368.2, 19970.55)
2033	Female	976727581.68 (831507450.46, 1121947712.9)	17652.74 (15059.33, 20246.14)
2034	Female	992293921.68 (827518133.92, 1157069709.43)	17636.34 (14739.75, 20532.94)
2035	Female	1007894012.44 (822329769.24, 1193458255.64)	17620.12 (14409.88, 20830.36)
2036	Female	1023327034 (815755568.19, 1230898499.81)	17603.34 (14069.39, 21137.29)
2037	Female	1038452073.81 (807658914.21, 1269245233.42)	17585.17 (13717.67, 21452.67)
2038	Female	1053469802.45 (798209958.67, 1308729646.24)	17566.6 (13356.16, 21777.04)
2039	Female	1068580564.22 (787556001.42, 1349605127.01)	17548.01 (12985.66, 22110.36)
2040	Female	1083686102.96 (775603028.39, 1391769177.53)	17529.3 (12606.44, 22452.17)
2041	Female	1098640546.59 (762215909.34, 1435065183.85)	17510.5 (12218.65, 22802.35)
2042	Female	1113316349.33 (747273198.36, 1479359500.31)	17490.82 (11821.72, 23159.93)
2043	Female	1127881608 (730890988.12, 1524872227.88)	17470.88 (11416.55, 23525.22)
2044	Female	1142499725.35 (713161694.76, 1571837755.94)	17450.67 (11003.52, 23897.82)
2045	Female	1157104996.41 (694014546.05, 1620195446.77)	17430.04 (10582.81, 24277.26)

2022	Male	495389041.76 (485554792.91, 505223290.6)	11647.39 (11533.65, 11761.14)
2023	Male	504329833.33 (492306087.41, 516353579.25)	11637 (11446.58, 11827.43)
2024	Male	513458784.09 (497948920.32, 528968647.86)	11626.9 (11339.67, 11914.12)
2025	Male	522692945.67 (502556506.43, 542829384.91)	11617.3 (11218.21, 12016.38)
2026	Male	531882070.54 (506133832.34, 557630308.74)	11607.12 (11083.38, 12130.86)
2027	Male	540928086.12 (508684970.88, 573171201.37)	11596.08 (10936.05, 12256.1)
2028	Male	549936457.56 (510397510.76, 589475404.37)	11584.53 (10777.98, 12391.09)
2029	Male	559071062.5 (511470702.23, 606671422.77)	11573.2 (10610.78, 12535.62)
2030	Male	568261787.65 (511859743.35, 624663831.96)	11562.37 (10435.39, 12689.35)
2031	Male	577391751.93 (511461475.81, 643322028.06)	11551.42 (10251.6, 12851.24)
2032	Male	586372493.21 (510192616.69, 662552369.73)	11539.96 (10059.22, 13020.69)
2033	Male	595287392.6 (508145285.02, 682429500.19)	11528.08 (9858.97, 13197.18)
2034	Male	604290689.12 (505457982.68, 703123395.56)	11516.27 (9651.76, 13380.78)
2035	Male	613335193.7 (502085076.12, 724585311.28)	11504.88 (9438.2, 13571.56)
2036	Male	622335982.13 (497942557.97, 746729406.3)	11493.83 (9218.37, 13769.28)
2037	Male	631210673.64 (492946960.54, 769474386.74)	11482.67 (8991.91, 13973.42)
2038	Male	640040782.71 (487168266.72, 792913298.7)	11471.17 (8759.03, 14183.31)
2039	Male	648954177.27 (480700618.37, 817207736.18)	11459.57 (8520.25, 14398.9)
2040	Male	657913178.86 (473501983.43, 842324374.29)	11448.27 (8276.09, 14620.45)
2041	Male	666868633.59 (465515382.39, 868221884.78)	11437.7 (8026.91, 14848.49)
2042	Male	675745951.23 (456663303.91, 894828598.55)	11427.47 (7772.37, 15082.58)
2043	Male	684594448.16 (446976390.86, 922212505.47)	11417.03 (7512.35, 15321.71)
2044	Male	693518610.23 (436512494.71, 950524725.74)	11406.32 (7247.09, 15565.56)
2045	Male	702494703.61 (425235888.13, 979753519.09)	11395.77 (6977, 15814.53)
**YLDs**			
2022	Both	108804723.71 (106750143.34, 110859304.07)	1247.88 (1224.3, 1271.45)

2023	Both	110442751.74 (107907053.91, 112978449.58)	1244.88 (1216.28, 1273.48)
2024	Both	112103272.56 (108805420.9, 115401124.21)	1241.96 (1205.41, 1278.52)
2025	Both	113773724.95 (109472843.34, 118074606.57)	1239.16 (1192.29, 1286.03)
2026	Both	115431698.93 (109922617.12, 120940780.73)	1236.41 (1177.36, 1295.45)
2027	Both	117060323.15 (110160188.14, 123960458.15)	1233.65 (1160.89, 1306.42)
2028	Both	118676273.26 (110221438.89, 127131107.63)	1230.97 (1143.21, 1318.73)
2029	Both	120303527.07 (110139875.8, 130467178.34)	1228.41 (1124.55, 1332.27)
2030	Both	121930098.78 (109909970.13, 133950227.43)	1225.98 (1105.01, 1346.94)
2031	Both	123539817.09 (109518479.1, 137561155.07)	1223.66 (1084.64, 1362.68)
2032	Both	125117812.1 (108951414.7, 141284209.5)	1221.41 (1063.42, 1379.4)
2033	Both	126677181.67 (108225355.13, 145129008.21)	1219.28 (1041.46, 1397.09)
2034	Both	128239246.89 (107361230.2, 149117263.58)	1217.28 (1018.84, 1415.73)
2035	Both	129794366.68 (106351244.89, 153237488.47)	1215.43 (995.58, 1435.28)
2036	Both	131332352.78 (105185239.85, 157479465.71)	1213.76 (971.72, 1455.8)
2037	Both	132840042.08 (103849536.36, 161830547.81)	1212.23 (947.2, 1477.25)
2038	Both	134331799.62 (102357817.49, 166305781.75)	1210.86 (922.08, 1499.64)
2039	Both	135823770.76 (100723138.63, 170924402.89)	1209.66 (896.38, 1522.94)
2040	Both	137307781.87 (98938325.87, 175677237.87)	1208.61 (870.08, 1547.14)
2041	Both	138780711.18 (96998202.99, 180563219.36)	1207.82 (843.25, 1572.38)
2042	Both	140231681.16 (94890753.61, 185572608.72)	1207.24 (815.82, 1598.65)
2043	Both	141670009.87 (92622956.49, 190717063.26)	1206.87 (787.8, 1625.95)
2044	Both	143106897.66 (90201524.63, 196012270.69)	1206.7 (759.15, 1654.24)
2045	Both	144537114.69 (87620914.07, 201453315.3)	1206.71 (729.88, 1683.53)
2022	Female	67349984.92 (66050850.63, 68649119.22)	1510.19 (1495.76, 1524.63)
2023	Female	68383025.45 (66787246.96, 69978803.95)	1506.65 (1482.27, 1531.04)
2024	Female	69430573.42 (67364006.92, 71497139.91)	1503.22 (1466.35, 1540.09)

2025	Female	70484414.69 (67796742.66, 73172086.71)	1499.86 (1448.61, 1551.11)
2026	Female	71529872.84 (68092690.69, 74967054.99)	1496.47 (1429.24, 1563.7)
2027	Female	72556619.24 (68255452.31, 76857786.18)	1492.97 (1408.3, 1577.64)
2028	Female	73577577.33 (68309859.08, 78845295.59)	1489.49 (1386.1, 1592.87)
2029	Female	74606279.22 (68275462.62, 80937095.81)	1486.07 (1362.83, 1609.31)
2030	Female	75634140.47 (68147778.33, 83120502.61)	1482.69 (1338.53, 1626.85)
2031	Female	76649896.63 (67917480.32, 85382312.95)	1479.31 (1313.22, 1645.39)
2032	Female	77643937.34 (67575608.01, 87712266.67)	1475.83 (1286.85, 1664.81)
2033	Female	78627363.52 (67135128.29, 90119598.74)	1472.38 (1259.6, 1685.16)
2034	Female	79612249.81 (66608002.65, 92616496.97)	1468.95 (1231.54, 1706.36)
2035	Female	80590761.22 (65987861.34, 95193661.11)	1465.52 (1202.69, 1728.35)
2036	Female	81555107.52 (65267285.09, 97842929.95)	1462.12 (1173.1, 1751.13)
2037	Female	82496493.36 (64437452.83, 100555533.9)	1458.66 (1142.71, 1774.61)
2038	Female	83427025.48 (63509560.2, 103344490.76)	1455.23 (1111.62, 1798.83)
2039	Female	84356181.58 (62491362.59, 106221000.56)	1451.79 (1079.87, 1823.71)
2040	Female	85277180.19 (61377260.99, 109177099.4)	1448.32 (1047.47, 1849.17)
2041	Female	86186936.21 (60163422.57, 112210449.84)	1444.9 (1014.48, 1875.32)
2042	Female	87078134.87 (58842271.28, 115313998.46)	1441.46 (980.86, 1902.07)
2043	Female	87959918.22 (57420714.21, 118499122.23)	1438.05 (946.66, 1929.43)
2044	Female	88838516.41 (55902589.29, 121774443.53)	1434.61 (911.91, 1957.32)
2045	Female	89709386.52 (54283853.13, 125134919.91)	1431.11 (876.58, 1985.64)
2022	Male	41454738.78 (40699292.72, 42210184.85)	974.5 (965.53, 983.48)
2023	Male	42059726.29 (41119806.95, 42999645.63)	972.08 (956.7, 987.46)
2024	Male	42672699.14 (41441413.98, 43903984.31)	969.7 (946.31, 993.09)
2025	Male	43289310.27 (41676100.68, 44902519.86)	967.39 (934.79, 999.99)
2026	Male	43901826.09 (41829926.43, 45973725.75)	965.12 (922.28, 1007.95)

2027	Male	44503703.9 (41904735.83, 47102671.98)	962.84 (908.83, 1016.85)
2028	Male	45098695.93 (41911579.82, 48285812.04)	960.57 (894.57, 1026.56)
2029	Male	45697247.86 (41864413.19, 49530082.53)	958.32 (879.61, 1037.04)
2030	Male	46295958.31 (41762191.8, 50829724.83)	956.14 (864.02, 1048.27)
2031	Male	46889920.45 (41600998.78, 52178842.12)	954.03 (847.85, 1060.2)
2032	Male	47473874.76 (41375806.69, 53571942.83)	951.93 (831.06, 1072.8)
2033	Male	48049818.16 (41090226.84, 55009409.47)	949.84 (813.7, 1085.97)
2034	Male	48626997.08 (40753227.55, 56500766.61)	947.76 (795.81, 1099.72)
2035	Male	49203605.46 (40363383.55, 58043827.36)	945.73 (777.45, 1114.02)
2036	Male	49777245.26 (39917954.76, 59636535.76)	943.78 (758.65, 1128.91)
2037	Male	50343548.72 (39412083.53, 61275013.91)	941.88 (739.4, 1144.36)
2038	Male	50904774.14 (38848257.29, 62961291)	939.98 (719.68, 1160.28)
2039	Male	51467589.18 (38231776.04, 64703402.33)	938.08 (699.52, 1176.64)
2040	Male	52030601.67 (37561064.88, 66500138.46)	936.21 (678.96, 1193.46)
2041	Male	52593774.97 (36834780.43, 68352769.51)	934.43 (658.05, 1210.8)
2042	Male	53153546.29 (36048482.33, 70258610.26)	932.71 (636.77, 1228.65)
2043	Male	53710091.65 (35202242.27, 72217941.03)	931.01 (615.1, 1246.91)
2044	Male	54268381.25 (34298935.34, 74237827.16)	929.29 (593.04, 1265.53)
2045	Male	54827728.16 (33337060.94, 76318395.39)	927.58 (570.63, 1284.53)

Notes: ASR: age-standardized rates; YLDs: years lived with disability.

Table S61. Prevalence and YLDs of neurological disorders in the worldwide of future forecasts using bayesian age-period-cohort model.

Year	Sex	Number	ASR
**Prevalence**			
2022	Both	3426755117.43 (3323976085.43, 3529534149.43)	41082.24 (39849.45, 42315.03)
2023	Both	3472036247.85 (3355009009.59, 3589063486.11)	41140.16 (39752.8, 42527.52)
2024	Both	3516874715.28 (3376990259.97, 3656759170.59)	41191.75 (39552.54, 42830.96)
2025	Both	3561102331.39 (3390128423.78, 3732076239.01)	41237.22 (39256.44, 43218.01)
2026	Both	3604458872.63 (3395040859.61, 3813876885.64)	41275.22 (38876.02, 43674.41)
2027	Both	3647181108.41 (3392774049.86, 3901588166.96)	41307.35 (38424.55, 44190.16)
2028	Both	3688897593.82 (3383784645.41, 3994010542.24)	41336.37 (37915.53, 44757.22)
2029	Both	3730239384.73 (3369173632.07, 4091305137.4)	41363.12 (37356.97, 45369.28)
2030	Both	3771021956.67 (3349068955.73, 4192974957.61)	41386.98 (36752.84, 46021.12)
2031	Both	3810932389.4 (3323340454.32, 4298524324.49)	41405.31 (36103.47, 46707.15)
2032	Both	3850107840.52 (3292193645.39, 4408022035.65)	41419.1 (35411.57, 47426.63)
2033	Both	3888627153.29 (3255904968.96, 4521349337.61)	41432.05 (34683.38, 48180.73)
2034	Both	3926795021.9 (3214853380.35, 4638736663.45)	41445.26 (33921.78, 48968.75)
2035	Both	3964416112.56 (3168918656.51, 4759913568.62)	41457.82 (33127.07, 49788.58)
2036	Both	4001160909.3 (3117818082.44, 4884503736.16)	41466.93 (32297.33, 50636.54)
2037	Both	4037142087.4 (3061603644.95, 5012680529.86)	41473.46 (31433.33, 51513.6)
2038	Both	4072633248.72 (3000566907.14, 5144699590.3)	41481.74 (30539.59, 52423.89)
2039	Both	4107886285.14 (2934920002.87, 5280852567.41)	41492.96 (29617.56, 53368.36)
2040	Both	4142743942.29 (2864519363.19, 5420968521.38)	41505.99 (28666.43, 54345.54)
2041	Both	4176897793.64 (2789076955.79, 5564718631.49)	41517.94 (27683.74, 55352.15)
2042	Both	4210475230.12 (2708589507.98, 5712360952.26)	41529.67 (26669.4, 56389.94)
2043	Both	4243796132.13 (2623272921.02, 5864319343.24)	41546.15 (25626.89, 57465.41)

2044	Both	4277109495.72 (2533237667.84, 6020981323.61)	41568.75 (24556.67, 58580.83)
2045	Both	4310286528.69 (2438311628.81, 6182261428.56)	41596.17 (23457.14, 59735.21)
2022	Female	1909058109.8 (1858795207.05, 1959321012.55)	45638.23 (45092.71, 46183.75)
2023	Female	1934303693.72 (1876145869.04, 1992461518.41)	45707.31 (44850.83, 46563.79)
2024	Female	1959374220.17 (1888517354.45, 2030231085.88)	45770.74 (44514.79, 47026.69)
2025	Female	1984205220.33 (1896173442.14, 2072236998.53)	45828.78 (44107.15, 47550.41)
2026	Female	2008673565.2 (1899535929.09, 2117811201.32)	45879.76 (43636.56, 48122.96)
2027	Female	2032913496.3 (1899187919.58, 2166639073.01)	45925.17 (43110.01, 48740.33)
2028	Female	2056715531.51 (1895357336.87, 2218073726.15)	45966.93 (42535.14, 49398.71)
2029	Female	2080399309.2 (1888603867.02, 2272194751.38)	46005.74 (41916.53, 50094.95)
2030	Female	2103890568.28 (1879003472.39, 2328777664.17)	46040.83 (41256.05, 50825.6)
2031	Female	2127035251.15 (1866480704.99, 2387589797.31)	46069.08 (40552.45, 51585.71)
2032	Female	2149909972.09 (1851129602.21, 2448690341.97)	46091.18 (39807.36, 52375)
2033	Female	2172524800.71 (1833058220.31, 2511991381.11)	46110.14 (39025.96, 53194.33)
2034	Female	2195022840.31 (1812444476.57, 2577601204.05)	46126.85 (38210.88, 54042.81)
2035	Female	2217317677.77 (1789230375.08, 2645404980.45)	46140.16 (37362.48, 54917.85)
2036	Female	2239245636.63 (1763263175.84, 2715228097.42)	46146.77 (36478.77, 55814.76)
2037	Female	2260867907.96 (1734557283.64, 2787178532.29)	46147.14 (35560.6, 56733.68)
2038	Female	2282290800.62 (1703230344.48, 2861351256.76)	46144.98 (34612.57, 57677.4)
2039	Female	2303628810.07 (1669376329.48, 2937881290.65)	46141.25 (33636.63, 58645.86)
2040	Female	2324824804.81 (1632932183.87, 3016717425.75)	46134.52 (32632.61, 59636.44)
2041	Female	2345737654.67 (1593750169.05, 3097725140.28)	46121.43 (31598.57, 60644.3)
2042	Female	2366445862.19 (1551822816.51, 3181068907.86)	46102.38 (30535.11, 61669.64)
2043	Female	2387087749.5 (1507237319.45, 3266938179.54)	46081.71 (29446.46, 62716.96)
2044	Female	2407770382.22 (1460032474.15, 3355508290.29)	46060.49 (28334.16, 63786.81)
2045	Female	2428454318.17 (1410128098.51, 3446780537.82)	46037.09 (27197.79, 64876.4)

2022	Male	1517697007.63 (1465180878.38, 1570213136.88)	36535.43 (35995.28, 37075.58)
2023	Male	1537732554.13 (1478863140.55, 1596601967.71)	36583.54 (35779.79, 37387.28)
2024	Male	1557500495.11 (1488472905.51, 1626528084.71)	36622.75 (35477.49, 37768.01)
2025	Male	1576897111.06 (1493954981.64, 1659839240.49)	36652.42 (35107.58, 38197.27)
2026	Male	1595785307.43 (1495504930.52, 1696065684.33)	36670.67 (34678.11, 38663.23)
2027	Male	1614267612.11 (1493586130.28, 1734949093.95)	36678.45 (34195.71, 39161.19)
2028	Male	1632182062.31 (1488427308.54, 1775936816.09)	36678.52 (33668.47, 39688.57)
2029	Male	1649840075.53 (1480569765.04, 1819110386.01)	36671.34 (33100.53, 40242.14)
2030	Male	1667131388.39 (1470065483.34, 1864197293.44)	36655.6 (32493.27, 40817.92)
2031	Male	1683897138.25 (1456859749.33, 1910934527.17)	36628.29 (31845.73, 41410.84)
2032	Male	1700197868.43 (1441064043.18, 1959331693.69)	36589.82 (31159.76, 42019.88)
2033	Male	1716102352.58 (1422846748.65, 2009357956.51)	36543.93 (30441.18, 42646.67)
2034	Male	1731772181.59 (1402408903.78, 2061135459.4)	36491.31 (29692.56, 43290.05)
2035	Male	1747098434.79 (1379688281.43, 2114508588.16)	36430.45 (28914.15, 43946.74)
2036	Male	1761915272.67 (1354554906.6, 2169275638.74)	36358.2 (28104.53, 44611.87)
2037	Male	1776274179.44 (1327046361.31, 2225501997.58)	36274.89 (27265.01, 45284.77)
2038	Male	1790342448.1 (1297336562.66, 2283348333.54)	36184.84 (26400.84, 45968.83)
2039	Male	1804257475.07 (1265543673.39, 2342971276.76)	36088.82 (25514.12, 46663.52)
2040	Male	1817919137.48 (1231587179.32, 2404251095.63)	35985.14 (24604.82, 47365.46)
2041	Male	1831160138.97 (1195326786.74, 2466993491.21)	35870.6 (23671.7, 48069.51)
2042	Male	1844029367.93 (1156766691.46, 2531292044.4)	35745.53 (22715.94, 48775.11)
2043	Male	1856708382.63 (1116035601.56, 2597381163.7)	35614.78 (21742.38, 49487.19)
2044	Male	1869339113.51 (1073205193.69, 2665473033.32)	35479.31 (20752.83, 50205.79)
2045	Male	1881832210.52 (1028183530.29, 2735480890.74)	35337.32 (19747.25, 50927.4)
**YLDs**			
2022	Both	79222928.74 (76627799.08, 81818058.4)	934.21 (903.61, 964.82)

2023	Both	80774916.21 (77828199.24, 83721633.18)	936.69 (902.51, 970.86)
2024	Both	82381525.74 (78873977.93, 85889073.55)	939.03 (899.04, 979.01)
2025	Both	84016990.61 (79741036.51, 88292944.7)	941.27 (893.36, 989.18)
2026	Both	85659752.21 (80423271.31, 90896233.12)	943.43 (885.75, 1001.11)
2027	Both	87294899.67 (80922502.34, 93667297.01)	945.52 (876.48, 1014.55)
2028	Both	88949039.43 (81279218.35, 96618860.51)	947.54 (865.82, 1029.26)
2029	Both	90657916.32 (81534695.63, 99781137.01)	949.5 (853.92, 1045.07)
2030	Both	92397961.23 (81670693.14, 103125229.33)	951.41 (840.92, 1061.9)
2031	Both	94148016.73 (81668674.86, 106627358.6)	953.28 (826.88, 1079.69)
2032	Both	95891129.43 (81513386.96, 110268871.9)	955.12 (811.85, 1098.4)
2033	Both	97651859.15 (81227867.68, 114075850.62)	956.95 (795.91, 1117.99)
2034	Both	99461976.43 (80837366.06, 118086586.81)	958.76 (779.11, 1138.42)
2035	Both	101299220.03 (80319891.48, 122278548.59)	960.58 (761.48, 1159.68)
2036	Both	103141870.76 (79653721.32, 126630020.21)	962.4 (743.03, 1181.77)
2037	Both	104972473.92 (78821388.25, 131123559.59)	964.23 (723.76, 1204.7)
2038	Both	106817187.55 (77841680.43, 135792694.67)	966.09 (703.7, 1228.48)
2039	Both	108705988.61 (76732730.29, 140679246.93)	968 (682.88, 1253.11)
2040	Both	110620032.13 (75475161.52, 145764902.74)	969.95 (661.29, 1278.6)
2041	Both	112540448.94 (74049712.2, 151031185.67)	971.95 (638.93, 1304.97)
2042	Both	114452467.74 (72440765.42, 156464170.05)	974.02 (615.77, 1332.27)
2043	Both	116383244.49 (70662445.23, 162104043.75)	976.18 (591.83, 1360.52)
2044	Both	118360951.53 (68725762.31, 167996140.75)	978.43 (567.11, 1389.75)
2045	Both	120366622.86 (66611242.63, 174122003.1)	980.8 (541.59, 1420.01)
2022	Female	47295585.95 (45880666.17, 48710505.74)	1085.19 (1071.35, 1099.02)
2023	Female	48209668.22 (46585921.49, 49833414.96)	1088.31 (1066.59, 1110.02)
2024	Female	49155845 (47197607.61, 51114082.4)	1091.29 (1059.52, 1123.06)

2025	Female	50120424.58 (47705278.39, 52535570.76)	1094.16 (1050.68, 1137.63)
2026	Female	51091894.52 (48107994.22, 54075794.83)	1096.91 (1040.32, 1153.5)
2027	Female	52062165.71 (48407867.87, 55716463.56)	1099.55 (1028.56, 1170.53)
2028	Female	53045826.57 (48627689.6, 57463963.54)	1102.06 (1015.55, 1188.58)
2029	Female	54063075.51 (48790789.51, 59335361.51)	1104.47 (1001.36, 1207.57)
2030	Female	55101677.9 (48887596.08, 61315759.72)	1106.77 (986.09, 1227.45)
2031	Female	56150337.8 (48907835.16, 63392840.45)	1108.95 (969.74, 1248.17)
2032	Female	57199430.48 (48842352.15, 65556508.81)	1111.02 (952.34, 1269.7)
2033	Female	58262400.81 (48703563.51, 67821238.11)	1112.97 (933.96, 1291.99)
2034	Female	59356544.84 (48505000.64, 70208089.03)	1114.81 (914.64, 1314.99)
2035	Female	60469218.11 (48233879.13, 72704557.1)	1116.55 (894.41, 1338.68)
2036	Female	61587980.02 (47877420.07, 75298539.97)	1118.16 (873.29, 1363.03)
2037	Female	62702159.21 (47424698.79, 77979619.62)	1119.65 (851.28, 1388.03)
2038	Female	63825884.99 (46885713.7, 80766056.27)	1121.03 (828.43, 1413.64)
2039	Female	64975743.83 (46270409.31, 83681078.34)	1122.29 (804.76, 1439.83)
2040	Female	66140860.85 (45567483.94, 86714237.76)	1123.44 (780.32, 1466.57)
2041	Female	67310522.07 (44765766.51, 89855277.63)	1124.47 (755.09, 1493.85)
2042	Female	68475844.33 (43855761.45, 93095927.21)	1125.39 (729.1, 1521.67)
2043	Female	69651473.66 (42845044.2, 96457903.12)	1126.19 (702.37, 1550)
2044	Female	70853340.35 (41739964.15, 99966716.55)	1126.88 (674.95, 1578.81)
2045	Female	72071349.09 (40529762.59, 103612935.59)	1127.46 (646.83, 1608.09)
2022	Male	31927342.79 (30747132.91, 33107552.67)	780.09 (768.46, 791.71)
2023	Male	32565247.99 (31242277.75, 33888218.22)	781.99 (764.47, 799.5)
2024	Male	33225680.73 (31676370.32, 34774991.15)	783.73 (758.68, 808.78)
2025	Male	33896566.03 (32035758.12, 35757373.94)	785.32 (751.48, 819.17)
2026	Male	34567857.69 (32315277.09, 36820438.29)	786.77 (743.08, 830.46)

2027	Male	35232733.96 (32514634.47, 37950833.45)	788.07 (733.59, 842.55)
2028	Male	35903212.86 (32651528.75, 39154896.97)	789.23 (723.13, 855.33)
2029	Male	36594840.81 (32743906.11, 40445775.5)	790.24 (711.76, 868.73)
2030	Male	37296283.33 (32783097.06, 41809469.61)	791.12 (699.53, 882.7)
2031	Male	37997678.92 (32760839.7, 43234518.15)	791.83 (686.46, 897.19)
2032	Male	38691698.95 (32671034.82, 44712363.09)	792.38 (672.59, 912.17)
2033	Male	39389458.34 (32524304.17, 46254612.51)	792.79 (657.98, 927.61)
2034	Male	40105431.59 (32332365.41, 47878497.77)	793.07 (642.66, 943.48)
2035	Male	40830001.92 (32086012.35, 49573991.49)	793.2 (626.66, 959.74)
2036	Male	41553890.74 (31776301.25, 51331480.24)	793.16 (609.99, 976.34)
2037	Male	42270314.72 (31396689.46, 53143939.97)	792.97 (592.66, 993.28)
2038	Male	42991302.56 (30955966.73, 55026638.4)	792.63 (574.72, 1010.54)
2039	Male	43730244.78 (30462320.98, 56998168.59)	792.16 (556.22, 1028.1)
2040	Male	44479171.28 (29907677.58, 59050664.99)	791.55 (537.17, 1045.92)
2041	Male	45229926.87 (29283945.69, 61175908.04)	790.77 (517.57, 1063.98)
2042	Male	45976623.41 (28585003.97, 63368242.85)	789.84 (497.44, 1082.25)
2043	Male	46731770.83 (27817401.03, 65646140.63)	788.78 (476.83, 1100.73)
2044	Male	47507611.17 (26985798.15, 68029424.19)	787.59 (455.76, 1119.42)
2045	Male	48295273.77 (26081480.03, 70509067.51)	786.27 (434.27, 1138.28)

Notes: ASR: age-standardized rates; YLDs: years lived with disability.

Table S62. Prevalence and YLDs of digestive disorders in the worldwide of future forecasts using bayesian age-period-cohort model.

Year	Sex	Number	ASR
**Prevalence**			
2022	Both	9997122.51 (9614410.72, 10379834.31)	113.59 (109.24, 117.94)
2023	Both	10095030.19 (9653014.78, 10537045.6)	112.64 (107.71, 117.58)
2024	Both	10197469.89 (9661655.32, 10733284.47)	111.76 (105.88, 117.64)
2025	Both	10305019.57 (9643625.42, 10966413.73)	110.97 (103.84, 118.1)
2026	Both	10409405.57 (9593584.14, 11225227.01)	110.19 (101.54, 118.83)
2027	Both	10507930.98 (9510608.24, 11505253.73)	109.4 (99.01, 119.8)
2028	Both	10602010.35 (9401954.42, 11802066.28)	108.61 (96.3, 120.92)
2029	Both	10700929.42 (9278514.82, 12123344.03)	107.89 (93.53, 122.25)
2030	Both	10806122.82 (9142410.89, 12469834.75)	107.27 (90.73, 123.81)
2031	Both	10908822.43 (8985316.1, 12832328.76)	106.67 (87.83, 125.51)
2032	Both	11006717.84 (8803807.5, 13209628.18)	106.08 (84.81, 127.35)
2033	Both	11099994.09 (8600910.51, 13599077.68)	105.48 (81.69, 129.28)
2034	Both	11198834.33 (8385545.59, 14012123.07)	104.96 (78.53, 131.39)
2035	Both	11305528.03 (8159050.63, 14452005.43)	104.55 (75.38, 133.73)
2036	Both	11410524.17 (7912881.89, 14908166.45)	104.17 (72.14, 136.2)
2037	Both	11511780.15 (7643531.6, 15380028.7)	103.81 (68.81, 138.8)
2038	Both	11608509.19 (7352367.18, 15864651.21)	103.43 (65.37, 141.5)
2039	Both	11711893.84 (7047505.48, 16376820.21)	103.15 (61.9, 144.4)
2040	Both	11825273.03 (6730134.3, 16922244.64)	102.98 (58.41, 147.57)
2041	Both	11938576.74 (6391637.22, 17488762.95)	102.84 (54.83, 150.91)
2042	Both	12050244.95 (6028547.08, 18076743.15)	102.73 (51.13, 154.42)
2043	Both	12158398.43 (5642828.46, 18682357.28)	102.63 (47.33, 158.07)

2044	Both	12274768.29 (5240434.52, 19323750.79)	102.62 (43.49, 161.98)
2045	Both	12403446.93 (4819235.62, 20009255.97)	102.73 (39.56, 166.24)
2022	Female	4748890.02 (4579628.51, 4918151.52)	104.88 (103.1, 106.66)
2023	Female	4796670.08 (4596389.24, 4996950.91)	103.94 (101.09, 106.79)
2024	Female	4847698.86 (4598326.22, 5097071.5)	103.08 (98.89, 107.27)
2025	Female	4902371.42 (4587862.31, 5216880.54)	102.32 (96.61, 108.03)
2026	Female	4954504.9 (4560739.2, 5348270.6)	101.53 (94.14, 108.93)
2027	Female	5002788.22 (4516485.95, 5489090.49)	100.71 (91.48, 109.94)
2028	Female	5049087.69 (4459759.42, 5638415.96)	99.88 (88.7, 111.05)
2029	Female	5098914.68 (4396652.4, 5801176.96)	99.12 (85.89, 112.34)
2030	Female	5152891.47 (4328064.78, 5977718.16)	98.46 (83.09, 113.82)
2031	Female	5204521.2 (4248043.45, 6160998.95)	97.78 (80.19, 115.36)
2032	Female	5252537.94 (4154725.27, 6350350.61)	97.06 (77.16, 116.95)
2033	Female	5298615.17 (4050890.8, 6546339.53)	96.32 (74.05, 118.59)
2034	Female	5348647.85 (3941494.24, 6755801.47)	95.65 (70.94, 120.37)
2035	Female	5403466.1 (3826887.27, 6980044.92)	95.09 (67.86, 122.32)
2036	Female	5456124.1 (3701294.33, 7210953.86)	94.5 (64.69, 124.3)
2037	Female	5505389.77 (3562833.94, 7447945.6)	93.87 (61.44, 126.3)
2038	Female	5552882.28 (3413573.28, 7692191.28)	93.21 (58.12, 128.31)
2039	Female	5604990.66 (3258147.81, 7952371.53)	92.63 (54.81, 130.46)
2040	Female	5662944.12 (3096211.44, 8230866.02)	92.14 (51.52, 132.77)
2041	Female	5719428.26 (2922521.44, 8518238.12)	91.63 (48.17, 135.09)
2042	Female	5773364.43 (2735235.63, 8814184.34)	91.07 (44.74, 137.4)
2043	Female	5826211.71 (2537548.28, 9120312.37)	90.49 (41.28, 139.71)
2044	Female	5884645.93 (2330629.38, 9447251.53)	89.98 (37.81, 142.15)
2045	Female	5950128.93 (2113843.38, 9798489.97)	89.55 (34.35, 144.75)

2022	Male	5248232.5 (5034782.21, 5461682.79)	122.21 (120, 124.41)
2023	Male	5298360.11 (5056625.54, 5540094.69)	121.25 (117.94, 124.55)
2024	Male	5349771.03 (5063329.1, 5636212.97)	120.33 (115.63, 125.02)
2025	Male	5402648.15 (5055763.12, 5749533.19)	119.47 (113.17, 125.76)
2026	Male	5454900.67 (5032844.94, 5876956.4)	118.64 (110.57, 126.7)
2027	Male	5505142.77 (4994122.3, 6016163.24)	117.81 (107.8, 127.82)
2028	Male	5552922.66 (4942195, 6163650.33)	116.96 (104.88, 129.03)
2029	Male	5602014.75 (4881862.43, 6322167.07)	116.14 (101.9, 130.38)
2030	Male	5653231.36 (4814346.12, 6492116.6)	115.39 (98.9, 131.89)
2031	Male	5704301.23 (4737272.65, 6671329.81)	114.68 (95.83, 133.53)
2032	Male	5754179.9 (4649082.23, 6859277.57)	113.97 (92.66, 135.28)
2033	Male	5801378.93 (4550019.71, 7052738.15)	113.23 (89.38, 137.07)
2034	Male	5850186.47 (4444051.35, 7256321.59)	112.51 (86.08, 138.95)
2035	Male	5902061.93 (4332163.36, 7471960.5)	111.87 (82.77, 140.98)
2036	Male	5954400.07 (4211587.56, 7697212.59)	111.26 (79.41, 143.11)
2037	Male	6006390.38 (4080697.66, 7932083.1)	110.66 (75.99, 145.33)
2038	Male	6055626.91 (3938793.9, 8172459.93)	110.02 (72.49, 147.55)
2039	Male	6106903.18 (3789357.68, 8424448.68)	109.4 (68.96, 149.85)
2040	Male	6162328.91 (3633922.86, 8691378.62)	108.86 (65.43, 152.29)
2041	Male	6219148.48 (3469115.79, 8970524.83)	108.34 (61.87, 154.81)
2042	Male	6276880.52 (3293311.45, 9262558.81)	107.84 (58.25, 157.42)
2043	Male	6332186.73 (3105280.18, 9562044.91)	107.29 (54.57, 160)
2044	Male	6390122.36 (2909805.14, 9876499.25)	106.75 (50.86, 162.65)
2045	Male	6453318 (2705392.25, 10210766)	106.29 (47.14, 165.44)
**YLDs**			
2022	Both	970463.6 (935803.57, 1005123.64)	11.06 (10.66, 11.46)

2023	Both	980835.12 (938680.39, 1022989.86)	10.99 (10.51, 11.46)
2024	Both	991456.73 (937612.48, 1045300.97)	10.92 (10.32, 11.51)
2025	Both	1002247.26 (933130.19, 1071364.33)	10.85 (10.1, 11.6)
2026	Both	1012832.18 (925348.71, 1100315.65)	10.79 (9.86, 11.73)
2027	Both	1023040.99 (914355.87, 1131726.11)	10.73 (9.59, 11.87)
2028	Both	1033053.63 (900788.77, 1165318.49)	10.67 (9.3, 12.04)
2029	Both	1043375.63 (885317.64, 1201433.62)	10.61 (9, 12.22)
2030	Both	1053995.22 (868013.6, 1239976.85)	10.56 (8.7, 12.43)
2031	Both	1064572.94 (848545.5, 1280600.38)	10.52 (8.38, 12.65)
2032	Both	1074933.36 (826650.81, 1323215.91)	10.47 (8.05, 12.89)
2033	Both	1085134.16 (802545.13, 1367723.18)	10.43 (7.71, 13.15)
2034	Both	1095749.72 (776712.13, 1414787.31)	10.39 (7.36, 13.42)
2035	Both	1106844.04 (749150.6, 1464537.48)	10.36 (7.01, 13.72)
2036	Both	1118086.85 (719491.42, 1516682.28)	10.34 (6.65, 14.03)
2037	Both	1129296.7 (687415.94, 1571177.46)	10.32 (6.27, 14.36)
2038	Both	1140429.85 (652961.58, 1627898.12)	10.3 (5.89, 14.71)
2039	Both	1152132.98 (616480.27, 1687785.69)	10.29 (5.49, 15.08)
2040	Both	1164551.37 (577924.92, 1751186.26)	10.29 (5.09, 15.48)
2041	Both	1177370.9 (536950.47, 1817863.31)	10.29 (4.68, 15.9)
2042	Both	1190420.1 (493159.27, 1887845.3)	10.3 (4.25, 16.35)
2043	Both	1203543.75 (446420.06, 1960936.94)	10.31 (3.81, 16.82)
2044	Both	1217439.73 (397041.24, 2038394.13)	10.33 (3.35, 17.33)
2045	Both	1232331.2 (344812.52, 2120847.54)	10.37 (2.88, 17.87)
2022	Female	483275.69 (467082.44, 499468.95)	10.76 (10.58, 10.95)
2023	Female	488469.01 (468231.35, 508706.67)	10.69 (10.37, 11)
2024	Female	493862.58 (467350.45, 520374.71)	10.61 (10.14, 11.09)

2025	Female	499385.71 (464753.81, 534017.6)	10.55 (9.88, 11.21)
2026	Female	504729.44 (460412.4, 549046.48)	10.48 (9.61, 11.34)
2027	Female	509812.61 (454369.27, 565255.96)	10.41 (9.32, 11.49)
2028	Female	514824.92 (447033.36, 582616.48)	10.34 (9.01, 11.66)
2029	Female	520076.91 (438782.89, 601370.93)	10.27 (8.7, 11.84)
2030	Female	525521.99 (429613.27, 621430.72)	10.21 (8.38, 12.04)
2031	Female	530870.14 (419259.3, 642480.98)	10.14 (8.05, 12.24)
2032	Female	536026.78 (407579.91, 664473.66)	10.08 (7.7, 12.46)
2033	Female	541136.67 (394773.86, 687499.47)	10.02 (7.35, 12.68)
2034	Female	546542.24 (381114.72, 711969.76)	9.95 (6.99, 12.92)
2035	Female	552223.11 (366557.57, 737888.64)	9.9 (6.63, 13.17)
2036	Female	557899.62 (350840.99, 764958.25)	9.84 (6.26, 13.43)
2037	Female	563467.45 (333795.63, 793139.27)	9.79 (5.88, 13.69)
2038	Female	569037.46 (315523.07, 822551.84)	9.73 (5.49, 13.96)
2039	Female	574988.43 (296214.99, 853761.86)	9.67 (5.11, 14.24)
2040	Female	581333.29 (275806.46, 886868.57)	9.62 (4.71, 14.53)
2041	Female	587804.67 (254071.46, 921589.73)	9.57 (4.32, 14.83)
2042	Female	594295.86 (230780.89, 957909.61)	9.52 (3.92, 15.13)
2043	Female	600874.47 (205943.36, 995960.3)	9.47 (3.51, 15.43)
2044	Female	607945.79 (179762.05, 1036488.78)	9.42 (3.1, 15.75)
2045	Female	615556.57 (151967.68, 1079726.82)	9.38 (2.68, 16.07)
2022	Male	487187.91 (468721.13, 505654.69)	11.35 (11.15, 11.56)
2023	Male	492366.12 (470449.04, 514283.19)	11.28 (10.95, 11.61)
2024	Male	497594.14 (470262.03, 524926.26)	11.21 (10.73, 11.7)
2025	Male	502861.56 (468376.38, 537346.73)	11.15 (10.48, 11.81)
2026	Male	508102.74 (464936.31, 551269.17)	11.08 (10.22, 11.95)

2027	Male	513228.38 (459986.6, 566470.15)	11.02 (9.94, 12.1)
2028	Male	518228.71 (453755.41, 582702.01)	10.95 (9.65, 12.26)
2029	Male	523298.72 (446534.75, 600062.69)	10.89 (9.34, 12.44)
2030	Male	528473.23 (438400.33, 618546.13)	10.83 (9.03, 12.63)
2031	Male	533702.8 (429286.2, 638119.4)	10.77 (8.71, 12.83)
2032	Male	538906.58 (419070.9, 658742.25)	10.72 (8.38, 13.05)
2033	Male	543997.49 (407771.27, 680223.71)	10.66 (8.04, 13.28)
2034	Male	549207.48 (395597.4, 702817.56)	10.6 (7.69, 13.51)
2035	Male	554620.94 (382593.03, 726648.85)	10.55 (7.34, 13.76)
2036	Male	560187.23 (368650.44, 751724.03)	10.5 (6.98, 14.01)
2037	Male	565829.25 (353620.31, 778038.2)	10.45 (6.61, 14.28)
2038	Male	571392.39 (337438.51, 805346.27)	10.4 (6.24, 14.55)
2039	Male	577144.55 (320265.28, 834023.83)	10.35 (5.86, 14.83)
2040	Male	583218.07 (302118.46, 864317.69)	10.3 (5.48, 15.12)
2041	Male	589566.23 (282879.02, 896273.58)	10.26 (5.09, 15.42)
2042	Male	596124.24 (262378.39, 929935.69)	10.22 (4.7, 15.73)
2043	Male	602669.28 (240476.7, 964976.64)	10.17 (4.3, 16.04)
2044	Male	609493.94 (217279.19, 1001905.35)	10.13 (3.9, 16.36)
2045	Male	616774.63 (192844.85, 1041120.72)	10.09 (3.49, 16.69)

Notes: ASR: age-standardized rates; YLDs: years lived with disability.

Table S63. Prevalence and YLDs of genecological disorders in the worldwide of future forecasts using bayesian age-period-cohort model.

Year	Sex	Number	ASR
**Prevalence**			
2022	Both	171915793.12 (151409911.84, 192421674.4)	2079.7 (1831.7, 2327.7)
2023	Both	175602941.74 (153276059, 197929824.49)	2108.02 (1840.05, 2375.99)
2024	Both	179243088.45 (154412103.02, 204074073.89)	2135.96 (1840.1, 2431.82)
2025	Both	182792463.25 (154730774.08, 210854152.42)	2163.31 (1831.21, 2495.41)
2026	Both	186232308.25 (154196049.92, 218268566.57)	2190.2 (1813.38, 2567.02)
2027	Both	189561535.93 (152815061.22, 226308010.64)	2217.04 (1787.13, 2646.96)
2028	Both	192723733.36 (150596190.69, 234851276.04)	2244.02 (1753.24, 2734.8)
2029	Both	195701149.81 (147564571.73, 243837727.89)	2270.86 (1711.91, 2829.82)
2030	Both	198560630.58 (143786748.09, 253334513.07)	2297.46 (1663.15, 2931.77)
2031	Both	201399902.21 (139327143.92, 263472660.5)	2323.99 (1607.01, 3040.98)
2032	Both	204359570.36 (134263739.26, 274455401.46)	2351.08 (1543.75, 3158.41)
2033	Both	207424919.42 (128582343.98, 286267494.85)	2379.35 (1473.81, 3284.89)
2034	Both	210531001.46 (122216611.13, 298845391.78)	2408.77 (1396.91, 3420.63)
2035	Both	213744487.55 (115161580.16, 312327394.94)	2439.29 (1312.52, 3566.06)
2036	Both	217143770.75 (107390216.87, 326897324.63)	2470.95 (1219.92, 3721.97)
2037	Both	220883384.23 (98913994.35, 342879894.6)	2504.31 (1118.91, 3890.05)
2038	Both	224944807.62 (89719271.74, 360330018.91)	2540.25 (1010.05, 4072.45)
2039	Both	229337307.95 (79600320.14, 379383575.23)	2578.96 (891.21, 4270.59)
2040	Both	234044017.65 (68724844.67, 400157896.22)	2620.51 (764.71, 4486.05)
2041	Both	239039679.52 (57896714.03, 422781194.52)	2664.97 (640.41, 4720.69)
2042	Both	244358843.21 (45841897.73, 447504990.95)	2712.85 (503.7, 4977.46)
2043	Both	250033612.92 (34599861.44, 474608733.96)	2765.07 (378.05, 5260.35)

2044	Both	256151690.59 (23590623.33, 504497867.63)	2822.24 (256.69, 5573.37)
2045	Both	262728661.51 (17153789.67, 537517537.32)	2884.84 (185.85, 5920.9)
2022	Female	114476161.5 (97820860.14, 131131462.86)	2780.45 (2560.13, 3000.76)
2023	Female	116928915.04 (98959369.73, 134898460.34)	2817.74 (2557.57, 3077.91)
2024	Female	119340102.96 (99601125.73, 139079080.19)	2854.18 (2541.15, 3167.21)
2025	Female	121677829.75 (99671899.83, 143683759.68)	2889.33 (2511.67, 3267)
2026	Female	123928575.61 (99132030.55, 148725120.67)	2923.24 (2470.23, 3376.24)
2027	Female	126092328.02 (97977246.39, 154207409.66)	2956.33 (2418.14, 3494.52)
2028	Female	128131554 (96213042.41, 160050065.59)	2988.87 (2356.81, 3620.93)
2029	Female	130034956.43 (93857111.51, 166212801.35)	3020.35 (2286.5, 3754.21)
2030	Female	131853765.65 (90956701.15, 172750830.14)	3050.4 (2207.21, 3893.59)
2031	Female	133662729.42 (87557334.44, 179768124.39)	3079.03 (2119.03, 4039.02)
2032	Female	135567759.41 (83713132.61, 187422386.21)	3106.87 (2022.45, 4191.29)
2033	Female	137563542.35 (79415766.68, 195711318.01)	3134.78 (1918.35, 4351.21)
2034	Female	139598350.33 (74611290.73, 204585409.93)	3162.5 (1806.62, 4518.39)
2035	Female	141721467.72 (69289152.59, 214153782.86)	3189.52 (1686.75, 4692.28)
2036	Female	143992486.03 (63421177.9, 224563794.15)	3215.41 (1558.23, 4872.59)
2037	Female	146524551.92 (57008847.24, 236067377.08)	3240.51 (1420.98, 5060.05)
2038	Female	149318772.3 (50061668.46, 248735551.54)	3265.7 (1275.4, 5256.01)
2039	Female	152370487.68 (42383675.41, 262666579.41)	3290.94 (1121.22, 5460.66)
2040	Female	155666515.47 (34165591.82, 277962144.71)	3315.81 (957.87, 5673.75)
2041	Female	159190661.33 (26239667.98, 294740204.18)	3339.87 (784.77, 5894.98)
2042	Female	162971427.2 (17355051.85, 313217004.8)	3363.27 (601.57, 6124.97)
2043	Female	167045938.63 (9580978.46, 333652268.36)	3386.69 (408.39, 6365)
2044	Female	171485918.33 (2358974.34, 356397972.1)	3410.41 (205.02, 6615.81)
2045	Female	176308252.93 (0, 381766614.78)	3434.49 (-8.83, 6877.82)

2022	Male	57439631.62 (53589051.7, 61290211.54)	1380.19 (1327.71, 1432.67)
2023	Male	58674026.71 (54316689.27, 63031364.15)	1398.74 (1330, 1467.47)
2024	Male	59902985.49 (54810977.29, 64994993.7)	1416.9 (1326.62, 1507.19)
2025	Male	61114633.5 (55058874.25, 67170392.74)	1434.55 (1318.31, 1550.78)
2026	Male	62303732.64 (55064019.37, 69543445.9)	1451.71 (1305.66, 1597.75)
2027	Male	63469207.91 (54837814.84, 72100600.98)	1468.54 (1289.14, 1647.94)
2028	Male	64592179.36 (54383148.27, 74801210.45)	1485.02 (1269.07, 1700.96)
2029	Male	65666193.38 (53707460.22, 77624926.54)	1500.91 (1245.46, 1756.36)
2030	Male	66706864.94 (52830046.95, 80583682.93)	1516.1 (1218.31, 1813.89)
2031	Male	67737172.79 (51769809.47, 83704536.11)	1530.58 (1187.66, 1873.51)
2032	Male	68791810.95 (50550606.65, 87033015.25)	1544.59 (1153.72, 1935.45)
2033	Male	69861377.07 (49166577.3, 90556176.84)	1558.34 (1116.78, 1999.9)
2034	Male	70932651.13 (47605320.4, 94259981.86)	1571.81 (1076.88, 2066.75)
2035	Male	72023019.83 (45872427.57, 98173612.08)	1584.94 (1033.96, 2135.91)
2036	Male	73151284.72 (43969038.97, 102333530.48)	1597.64 (987.96, 2207.33)
2037	Male	74358832.31 (41905147.11, 106812517.52)	1610.1 (938.93, 2281.28)
2038	Male	75626035.32 (39657603.28, 111594467.36)	1622.66 (887.12, 2358.21)
2039	Male	76966820.27 (37216644.73, 116716995.81)	1635.4 (832.53, 2438.28)
2040	Male	78377502.18 (34559252.84, 122195751.51)	1648.28 (775.06, 2521.49)
2041	Male	79849018.19 (31657046.05, 128040990.34)	1661.17 (714.55, 2607.8)
2042	Male	81387416.01 (28486845.88, 134287986.15)	1674.16 (650.95, 2697.38)
2043	Male	82987674.29 (25018882.98, 140956465.61)	1687.45 (584.3, 2790.61)
2044	Male	84665772.26 (21231648.99, 148099895.53)	1701.06 (514.53, 2887.59)
2045	Male	86420408.58 (17153789.67, 155750922.55)	1714.94 (441.55, 2988.34)
**YLDs**			
2022	Both	957408.15 (851087.81, 1063728.49)	11.6 (10.31, 12.89)

2023	Both	977719.3 (861364.03, 1094074.56)	11.75 (10.35, 13.15)
2024	Both	997619.94 (867374.85, 1127865.04)	11.9 (10.35, 13.46)
2025	Both	1016854.74 (868689.27, 1165020.2)	12.05 (10.29, 13.81)
2026	Both	1035288.42 (865146.68, 1205430.17)	12.19 (10.19, 14.19)
2027	Both	1052882.78 (856812.16, 1248953.4)	12.33 (10.03, 14.62)
2028	Both	1069320.25 (843758.03, 1294882.47)	12.46 (9.83, 15.09)
2029	Both	1084504.22 (826154.77, 1342853.68)	12.59 (9.59, 15.59)
2030	Both	1098776.66 (804390.01, 1393163.31)	12.72 (9.31, 16.13)
2031	Both	1112627.47 (778835.95, 1446418.99)	12.84 (8.98, 16.7)
2032	Both	1126785.31 (749954.55, 1503616.07)	12.96 (8.62, 17.3)
2033	Both	1141174.89 (717727.68, 1564622.09)	13.09 (8.23, 17.95)
2034	Both	1155481.88 (681880.91, 1629082.85)	13.22 (7.79, 18.64)
2035	Both	1170091.16 (642479.42, 1697702.89)	13.35 (7.32, 19.38)
2036	Both	1185430.8 (599470.56, 1771391.04)	13.49 (6.81, 20.16)
2037	Both	1202349.89 (552886.71, 1851813.07)	13.63 (6.25, 21.01)
2038	Both	1220738.52 (502565.39, 1939149.47)	13.79 (5.66, 21.91)
2039	Both	1240690.35 (448279.24, 2034080.26)	13.95 (5.02, 22.9)
2040	Both	1262128.68 (388938.28, 2137124.22)	14.13 (4.33, 23.96)
2041	Both	1284912.38 (327465.27, 2248800.76)	14.33 (3.62, 25.11)
2042	Both	1309198.55 (264536.74, 2370212.22)	14.54 (2.91, 26.37)
2043	Both	1335157.56 (198205.28, 2502617.73)	14.77 (2.16, 27.75)
2044	Both	1363182.73 (134264.81, 2647760.33)	15.03 (1.46, 29.26)
2045	Both	1393302.78 (78206.6, 2807025.82)	15.31 (0.84, 30.93)
2022	Female	626317.85 (540709.84, 711925.85)	15.24 (14.1, 16.37)
2023	Female	639814.17 (547272, 732356.34)	15.44 (14.1, 16.79)
2024	Female	652968.36 (551064.64, 754872.09)	15.64 (14.01, 17.27)

2025	Female	665595.41 (551691.03, 779499.8)	15.83 (13.86, 17.8)
2026	Female	677591.61 (548937.18, 806246.04)	16.01 (13.64, 18.38)
2027	Female	688921.32 (542780.3, 835062.34)	16.17 (13.36, 18.99)
2028	Female	699366.57 (533255.04, 865478.1)	16.33 (13.02, 19.65)
2029	Female	708869.43 (520483.28, 897255.58)	16.48 (12.64, 20.33)
2030	Female	717693.61 (504747.44, 930639.79)	16.62 (12.21, 21.03)
2031	Female	726211.49 (486320.37, 966102.6)	16.75 (11.73, 21.76)
2032	Female	734960.92 (465532.14, 1004389.7)	16.86 (11.21, 22.52)
2033	Female	743910.43 (442384.9, 1045435.97)	16.98 (10.64, 23.31)
2034	Female	752810.36 (416662.15, 1088958.57)	17.08 (10.04, 24.13)
2035	Female	761950.95 (388397.28, 1135504.62)	17.18 (9.4, 24.97)
2036	Female	771662.33 (357534.7, 1185789.97)	17.28 (8.71, 25.84)
2037	Female	782554.88 (324049.11, 1241060.65)	17.36 (7.98, 26.74)
2038	Female	794628.12 (287903.43, 1301590.63)	17.45 (7.22, 27.68)
2039	Female	807871.92 (248913.56, 1367809.09)	17.53 (6.41, 28.65)
2040	Female	822224.31 (206118.69, 1440135.06)	17.62 (5.57, 29.66)
2041	Female	837594.83 (162604.37, 1519026.55)	17.69 (4.68, 30.71)
2042	Female	854105.38 (119183.44, 1605379.17)	17.77 (3.74, 31.79)
2043	Female	871929.35 (74076.56, 1700290.02)	17.84 (2.77, 32.92)
2044	Female	891378.93 (33177.25, 1805202.51)	17.92 (1.74, 34.1)
2045	Female	912490.19 (1588.39, 1921305.19)	18 (0.68, 35.32)
2022	Male	331090.3 (310377.96, 351802.64)	7.97 (7.68, 8.25)
2023	Male	337905.13 (314092.03, 361718.22)	8.07 (7.68, 8.45)
2024	Male	344651.58 (316310.21, 372992.95)	8.16 (7.65, 8.68)
2025	Male	351259.32 (316998.24, 385520.41)	8.26 (7.58, 8.93)
2026	Male	357696.81 (316209.5, 399184.13)	8.35 (7.49, 9.2)

2027	Male	363961.46 (314031.86, 413891.06)	8.43 (7.38, 9.48)
2028	Male	369953.68 (310502.99, 429404.37)	8.51 (7.24, 9.79)
2029	Male	375634.8 (305671.49, 445598.1)	8.59 (7.08, 10.1)
2030	Male	381083.05 (299642.57, 462523.52)	8.66 (6.9, 10.42)
2031	Male	386415.98 (292515.58, 480316.39)	8.73 (6.7, 10.76)
2032	Male	391824.39 (284422.41, 499226.37)	8.8 (6.48, 11.11)
2033	Male	397264.45 (275342.78, 519186.12)	8.86 (6.25, 11.47)
2034	Male	402671.52 (265218.76, 540124.28)	8.92 (5.99, 11.84)
2035	Male	408140.21 (254082.14, 562198.27)	8.97 (5.72, 12.22)
2036	Male	413768.47 (241935.86, 585601.07)	9.02 (5.43, 12.62)
2037	Male	419795.01 (228837.61, 610752.41)	9.07 (5.13, 13.02)
2038	Male	426110.4 (214661.96, 637558.84)	9.12 (4.81, 13.44)
2039	Male	432818.43 (199365.69, 666271.17)	9.18 (4.47, 13.88)
2040	Male	439904.37 (182819.58, 696989.16)	9.23 (4.12, 14.33)
2041	Male	447317.55 (164860.9, 729774.21)	9.28 (3.75, 14.8)
2042	Male	455093.17 (145353.3, 764833.05)	9.33 (3.37, 15.29)
2043	Male	463228.21 (124128.72, 802327.71)	9.38 (2.97, 15.79)
2044	Male	471803.81 (101087.56, 842557.82)	9.44 (2.55, 16.32)
2045	Male	480812.58 (76618.21, 885720.63)	9.49 (2.12, 16.86)

Notes: ASR: age-standardized rates; YLDs: years lived with disability.

Table S64. Prevalence and YLDs of mental disorders in the worldwide of future forecasts using bayesian age-period-cohort model.

Year	Sex	Number	ASR
**Prevalence**			
2022	Both	715955893.52 (678670089.19, 753241697.84)	8494.67 (8052.2, 8937.14)
2023	Both	750631321.61 (689903745.97, 811358897.26)	8797.29 (8085.55, 9509.03)
2024	Both	787598453.66 (692120926.14, 883075981.19)	9120.02 (8014.51, 10225.53)
2025	Both	827151925.26 (686795202.1, 967508648.42)	9466.11 (7860.07, 11072.15)
2026	Both	869201080.91 (673827114.77, 1064575047.06)	9834.1 (7624.1, 12044.11)
2027	Both	914187217.26 (652903418.44, 1175471016.08)	10228.42 (7305.77, 13151.07)
2028	Both	962840101.63 (623447502.97, 1302232700.28)	10658.04 (6902.29, 14413.79)
2029	Both	1016024211.13 (584435566.8, 1447612855.46)	11130.86 (6404.3, 15857.42)
2030	Both	1074358853.49 (534112329.12, 1614605377.86)	11653.15 (5795.61, 17510.69)
2031	Both	1137949891.25 (469875159.32, 1806024623.17)	12225.03 (5051.05, 19399.02)
2032	Both	1207683034.2 (388807603.93, 2026558464.46)	12854.91 (4142.81, 21567.01)
2033	Both	1284954139.23 (287143993.49, 2282764284.97)	13558.36 (3035.43, 24081.29)
2034	Both	1371379093.08 (159803520.56, 2583062340.64)	14350.51 (1680.06, 27022.58)
2035	Both	1468404906.62 (122781632.22, 2937469074.47)	15245.32 (1281.1, 30487.9)
2036	Both	1576878357.83 (78257732.01, 3357153113.57)	16249.85 (810.15, 34583.25)
2037	Both	1698856069.12 (23260261.27, 3858776653.78)	17383.54 (237.96, 39468.82)
2038	Both	1837490661.14 (0, 4466026062.06)	18677.99 (0, 45376.07)
2039	Both	1996339142.99 (0, 5210244156.4)	20167.53 (0, 52608.15)
2040	Both	2179228410.25 (0, 6132738305.35)	21888.8 (0, 61563.69)
2041	Both	2389600470.23 (0, 7286508683.72)	23873.96 (0, 72751.58)
2042	Both	2633196569.47 (0, 8749100956.04)	26177.81 (0, 86917.89)
2043	Both	2918394582.23 (0, 10633729738.27)	28881.03 (0, 105152.95)

2044	Both	3255192721.45 (0, 13100092954.01)	32080.42 (0, 128996.05)
2045	Both	3655664058.06 (0, 16375606919.62)	35891.86 (0, 160634.44)
2022	Female	441711198.59 (417996153.58, 465426243.59)	10434.78 (10035.48, 10834.08)
2023	Female	462964141.09 (422658915.17, 503269367.02)	10798.38 (9949.73, 11647.03)
2024	Female	485672724.17 (421072721.01, 550272727.33)	11179.74 (9749.26, 12610.21)
2025	Female	510054019.65 (414233782.6, 605874256.69)	11579.58 (9440.98, 13718.17)
2026	Female	536108047.2 (402045035.49, 670171058.9)	11992.6 (9019.65, 14965.55)
2027	Female	564152596.47 (384212984.34, 744092208.61)	12419.92 (8482.9, 16356.94)
2028	Female	594688010.24 (360225253.74, 829150766.74)	12867.56 (7829.99, 17905.13)
2029	Female	628304583.88 (329224913.94, 927384253.81)	13339.49 (7056.17, 19622.81)
2030	Female	665469529.27 (289837269.26, 1041101789.27)	13836.72 (6153.14, 21520.31)
2031	Female	706368113.36 (240088171.17, 1172648055.56)	14352.2 (5108.59, 23595.8)
2032	Female	751664605.75 (177626875.57, 1325702335.92)	14887.15 (3914.07, 25860.24)
2033	Female	802357811 (99343599.86, 1505372022.15)	15449.46 (2561.56, 28337.36)
2034	Female	859617972.59 (1054030.57, 1718289589.64)	16044.31 (1039.24, 31049.38)
2035	Female	924573003.58 (0, 1972586900.61)	16672.98 (-667.37, 34013.33)
2036	Female	998053971.8 (0, 2277762073.52)	17326.86 (-2572.44, 37226.16)
2037	Female	1081685754.73 (0, 2647693379.88)	18007.32 (-4689.75, 40704.4)
2038	Female	1177862221.83 (0, 3102078996.86)	18723.93 (-7036.94, 44484.81)
2039	Female	1289358383.78 (0, 3667408463.57)	19483.01 (-9635.07, 48601.08)
2040	Female	1419299693.14 (0, 4379291437.54)	20286.18 (-12506, 53078.37)
2041	Female	1570785290.98 (0, 5284626585.46)	21123.8 (-15664.73, 57912.33)
2042	Female	1748603515.92 (0, 6451913289.93)	21997.57 (-19132.08, 63127.21)
2043	Female	1959577966.29 (0, 7981523412.85)	22918.68 (-22939.54, 68776.9)
2044	Female	2212072476.57 (0, 10016883842.72)	23894.67 (-27120.95, 74910.3)
2045	Female	2516456988.37 (0, 12764727975.39)	24927.74 (-31708.27, 81563.74)

2022	Male	274244694.93 (260673935.61, 287815454.25)	6540.07 (6340.76, 6739.39)
2023	Male	287667180.52 (267244830.8, 308089530.24)	6773.86 (6372.45, 7175.27)
2024	Male	301925729.49 (271048205.13, 332803253.85)	7019.14 (6353.79, 7684.49)
2025	Male	317097905.61 (272561419.49, 361634391.73)	7276.4 (6289.94, 8262.85)
2026	Male	333093033.71 (271782079.28, 394403988.15)	7541.94 (6177.49, 8906.4)
2027	Male	350034620.79 (268690434.1, 431378807.47)	7816.59 (6015.55, 9617.63)
2028	Male	368152091.39 (263222249.23, 473081933.54)	8104.36 (5804.96, 10403.75)
2029	Male	387719627.25 (255210652.85, 520228601.64)	8407.9 (5544.28, 11271.53)
2030	Male	408889324.23 (244275059.86, 573503588.6)	8727.86 (5229.79, 12225.93)
2031	Male	431581777.88 (229786988.15, 633376567.61)	9059.26 (4854.29, 13264.23)
2032	Male	456018428.45 (211180728.37, 700856128.54)	9403.01 (4414.15, 14391.88)
2033	Male	482596328.23 (187800393.63, 777392262.83)	9764.43 (3907.27, 15621.59)
2034	Male	511761120.49 (158749489.99, 864772751)	10147.03 (3329.2, 16964.87)
2035	Male	543831903.04 (122781632.22, 964882173.86)	10551.63 (2673.47, 18429.8)
2036	Male	578824386.03 (78257732.01, 1079391040.05)	10972.1 (1931.85, 20012.35)
2037	Male	617170314.39 (23260261.27, 1211083273.89)	11409.44 (1098.31, 21720.58)
2038	Male	659628439.31 (0, 1363947065.2)	11870.2 (166.57, 23573.83)
2039	Male	706980759.21 (0, 1542835692.83)	12358.72 (-871.93, 25589.36)
2040	Male	759928717.11 (0, 1753446867.81)	12876.04 (-2027.04, 27779.11)
2041	Male	818815179.25 (0, 2001882098.27)	13415.15 (-3307.3, 30137.6)
2042	Male	884593053.55 (0, 2297187666.11)	13977.27 (-4721.97, 32676.51)
2043	Male	958816615.93 (0, 2652206325.42)	14570.15 (-6283.58, 35423.88)
2044	Male	1043120244.88 (0, 3083209111.29)	15199.01 (-8006.59, 38404.62)
2045	Male	1139207069.68 (0, 3610878944.23)	15865.29 (-9905.44, 41636.01)
**YLDs**			
2022	Both	101832572.47 (96111766.01, 107553378.94)	1209.06 (1141.12, 1276.99)

2023	Both	106571459.24 (97370984.74, 115771933.75)	1250.1 (1142.18, 1358.03)
2024	Both	111624499.82 (97267892.87, 125981106.76)	1293.95 (1127.54, 1460.36)
2025	Both	117034620.89 (96030719.37, 138038522.4)	1341.07 (1100.43, 1581.71)
2026	Both	122792378.43 (93655916.25, 151928840.61)	1391.3 (1061.24, 1721.36)
2027	Both	128963581 (90100156.16, 167827005.85)	1445.29 (1009.86, 1880.71)
2028	Both	135651937.58 (85272873.75, 186031001.42)	1504.32 (945.81, 2062.83)
2029	Both	142981893.46 (79013790.14, 206949996.78)	1569.54 (867.6, 2271.49)
2030	Both	151044595.95 (71051720.24, 231037471.67)	1641.88 (772.69, 2511.06)
2031	Both	159863479.89 (60988380.31, 258738579.48)	1721.43 (657.21, 2785.66)
2032	Both	169571799.76 (48361680.78, 290781918.73)	1809.47 (516.69, 3102.25)
2033	Both	180368223.77 (32562051.48, 328174396.06)	1908.23 (345.33, 3471.13)
2034	Both	192491178.43 (19768431.31, 372234581.53)	2019.95 (208.41, 3905.03)
2035	Both	206158578.81 (13868911.92, 424560267.42)	2146.72 (145.08, 4419.5)
2036	Both	221514143.45 (6545983.1, 486998731.53)	2289.81 (67.89, 5032.27)
2037	Both	238874111.92 (0, 562290777.9)	2452.19 (0, 5769.84)
2038	Both	258707181.24 (0, 654332473.33)	2638.59 (0, 6670.47)
2039	Both	281555761.85 (0, 768383178.8)	2854.26 (0, 7785.32)
2040	Both	308018142.08 (0, 911524045.79)	3104.95 (0, 9183.1)
2041	Both	338668750.93 (0, 1093120047.26)	3396.08 (0, 10954.34)
2042	Both	374428052.28 (0, 1326963003)	3736.49 (0, 13232.45)
2043	Both	416621714.52 (0, 1633387428.24)	4138.93 (0, 16214.11)
2044	Both	466863667.26 (0, 2041634740.5)	4619.12 (0, 20182.57)
2045	Both	527152474.28 (0, 2594203881.21)	5196.31 (0, 25548.57)
2022	Female	62350628.49 (58733964.93, 65967292.06)	1474.89 (1414.73, 1535.05)
2023	Female	65155798.24 (59103017.84, 71208578.65)	1521.92 (1395.04, 1648.8)
2024	Female	68152975.51 (58536791.11, 77769159.92)	1571.15 (1358.32, 1783.98)

2025	Female	71373049.56 (57191322.04, 85554777.08)	1622.66 (1305.79, 1939.52)
2026	Female	74818070.37 (55061688.65, 94574452.1)	1675.7 (1236.86, 2114.53)
2027	Female	78534561.23 (52108452.96, 104960669.49)	1730.42 (1151.32, 2309.53)
2028	Female	82592527.55 (48254406.38, 116930648.72)	1787.62 (1049.13, 2526.12)
2029	Female	87075357.51 (43369003.71, 130781711.3)	1847.8 (929.71, 2765.9)
2030	Female	92050053.64 (37243356.38, 146856750.9)	1911.07 (791.99, 3030.14)
2031	Female	97547880.02 (29578555.14, 165517204.9)	1976.48 (634.45, 3318.5)
2032	Female	103666583 (20007764.78, 187325401.21)	2044.17 (456.03, 3632.31)
2033	Female	110546586.72 (8036785.07, 213056388.37)	2115.17 (255.69, 3974.64)
2034	Female	118357034.55 (0, 243734725.09)	2190.11 (31.9, 4348.32)
2035	Female	127264015.13 (0, 280640051.97)	2269.13 (-217.2, 4755.46)
2036	Female	137400671.74 (0, 325317771.22)	2351.09 (-493.32, 5195.51)
2037	Female	149012211.97 (0, 380025883.08)	2436.17 (-798.19, 5670.52)
2038	Female	162450958.44 (0, 447981536.24)	2525.52 (-1134.1, 6185.14)
2039	Female	178133716.35 (0, 533595727.43)	2619.94 (-1503.73, 6743.61)
2040	Female	196541157.62 (0, 642926786.3)	2719.58 (-1909.83, 7348.98)
2041	Female	218176289.19 (0, 784188588.68)	2823.19 (-2354.13, 8000.51)
2042	Female	243796922.07 (0, 969493845.44)	2930.96 (-2839.21, 8701.13)
2043	Female	274474546.85 (0, 1216801624.02)	3044.23 (-3369.04, 9457.51)
2044	Female	311547652.23 (0, 1552274699.21)	3163.9 (-3947.86, 10275.66)
2045	Female	356715675.26 (0, 2014398438.88)	3290.18 (-4579.57, 11159.93)
2022	Male	39481943.98 (37377801.08, 41586086.88)	941.49 (910.79, 972.2)
2023	Male	41415661 (38267966.9, 44563355.1)	975.32 (913.71, 1036.92)
2024	Male	43471524.3 (38731101.76, 48211946.84)	1010.79 (908.82, 1112.76)
2025	Male	45661571.32 (38839397.33, 52483745.32)	1047.99 (896.93, 1199.05)
2026	Male	47974308.05 (38594227.6, 57354388.51)	1086.38 (877.55, 1295.21)

2027	Male	50429019.78 (37991703.2, 62866336.36)	1126.08 (850.55, 1401.62)
2028	Male	53059410.03 (37018467.36, 69100352.7)	1167.65 (815.99, 1519.32)
2029	Male	55906535.96 (35644786.43, 76168285.48)	1211.47 (773.62, 1649.32)
2030	Male	58994542.31 (33808363.86, 84180720.77)	1257.62 (722.88, 1792.35)
2031	Male	62315599.88 (31409825.17, 93221374.58)	1305.41 (662.74, 1948.07)
2032	Male	65905216.76 (28353916, 103456517.52)	1354.96 (592.61, 2117.31)
2033	Male	69821637.05 (24525266.41, 115118007.69)	1407.01 (512.14, 2301.88)
2034	Male	74134143.87 (19768431.31, 128499856.44)	1462.06 (420.62, 2503.51)
2035	Male	78894563.68 (13868911.92, 143920215.45)	1520.23 (317.07, 2723.38)
2036	Male	84113471.71 (6545983.1, 161680960.32)	1580.65 (200.3, 2961)
2037	Male	89861899.95 (0, 182264894.82)	1643.47 (69.4, 3217.55)
2038	Male	96256222.81 (0, 206350937.09)	1709.59 (-76.65, 3495.82)
2039	Male	103422045.5 (0, 234787451.37)	1779.6 (-239.14, 3798.34)
2040	Male	111476984.45 (0, 268597259.49)	1853.65 (-419.57, 4126.88)
2041	Male	120492461.75 (0, 308931458.58)	1930.79 (-619.16, 4480.73)
2042	Male	130631130.21 (0, 357469157.56)	2011.16 (-839.3, 4861.61)
2043	Male	142147167.67 (0, 416585804.22)	2095.81 (-1081.9, 5273.53)
2044	Male	155316015.03 (0, 489360041.29)	2185.47 (-1349.15, 5720.08)
2045	Male	170436799.02 (0, 579805442.33)	2280.32 (-1643.19, 6203.83)

Notes: ASR: age-standardized rates; YLDs: years lived with disability.

Table S65. Prevalence and YLDs of substance use disorders in the worldwide of future forecasts using bayesian age-period-cohort model.

Year	Sex	Number	ASR
**Prevalence**			
2022	Both	16189508.58 (14900009.74, 17479007.42)	271.64 (250.01, 293.27)
2023	Both	16523590.36 (15088145.33, 17959035.38)	274.92 (251.04, 298.81)
2024	Both	16868122.73 (15212644.05, 18523601.4)	278.39 (251.05, 305.72)
2025	Both	17224282.24 (15272721.92, 19175842.55)	282.02 (250.04, 313.99)
2026	Both	17593122.31 (15271101.73, 19915142.89)	285.8 (248.04, 323.57)
2027	Both	17978878.74 (15213893.63, 20743863.84)	289.78 (245.15, 334.41)
2028	Both	18383823.2 (15105935.53, 21661710.87)	294 (241.48, 346.51)
2029	Both	18806399.81 (14946904.93, 22665894.69)	298.44 (237.07, 359.82)
2030	Both	19246590.19 (14736123.22, 23757057.16)	303.11 (231.91, 374.31)
2031	Both	19704695.69 (14471735.34, 24937656.04)	308.01 (225.99, 390.02)
2032	Both	20184427.89 (14153364.38, 26215491.4)	313.17 (219.31, 407.03)
2033	Both	20685742.87 (13778304.59, 27593181.16)	318.65 (211.88, 425.41)
2034	Both	21210729.04 (13343705.18, 29077752.89)	324.43 (203.65, 445.22)
2035	Both	21758765.94 (12843938.35, 30673593.52)	330.54 (194.54, 466.53)
2036	Both	22329284.07 (12272510.68, 32386057.46)	336.98 (184.51, 489.46)
2037	Both	22927298.17 (11625039.07, 34229557.27)	343.81 (173.46, 514.15)
2038	Both	23544002.77 (10890393.8, 36197611.74)	351.06 (161.34, 540.78)
2039	Both	24188930.48 (10065204.55, 38312656.4)	358.76 (148.01, 569.51)
2040	Both	24863316.32 (9140179.06, 40586453.59)	366.93 (133.35, 600.5)
2041	Both	25568362.09 (8104458.56, 43032265.62)	375.6 (117.21, 634)
2042	Both	26308969.35 (6946919.04, 45671019.66)	384.84 (99.41, 670.27)
2043	Both	27084157.08 (5666631.77, 48515993.38)	394.7 (80.01, 709.64)

2044	Both	27897287.67 (4257358.75, 51588101.53)	405.23 (58.93, 752.44)
2045	Both	28751803.76 (2874137.89, 54912051.24)	416.46 (38.78, 799.02)
2022	Female	7952459.95 (7159442.86, 8745477.04)	266.05 (254.58, 277.51)
2023	Female	8173151.18 (7311223.02, 9035079.34)	271.05 (256.41, 285.69)
2024	Female	8399854.27 (7438815.43, 9360893.11)	276.22 (257.2, 295.23)
2025	Female	8633771.72 (7539397.69, 9728145.74)	281.54 (257.09, 305.99)
2026	Female	8876039.81 (7612017.85, 10140061.78)	287.01 (256.19, 317.84)
2027	Female	9128775.21 (7657846.11, 10599704.31)	292.65 (254.55, 330.74)
2028	Female	9392932.79 (7677897.79, 11107967.8)	298.47 (252.25, 344.68)
2029	Female	9667823.67 (7671431.5, 11664215.84)	304.46 (249.29, 359.64)
2030	Female	9953922.94 (7638134.76, 12269711.12)	310.63 (245.64, 375.61)
2031	Female	10251709.12 (7577113.35, 12926304.88)	316.95 (241.29, 392.62)
2032	Female	10562905.47 (7487934.72, 13637876.22)	323.45 (236.21, 410.69)
2033	Female	10887768.03 (7369113.13, 14406422.93)	330.14 (230.4, 429.89)
2034	Female	11227458.92 (7218957.07, 15235960.77)	337.02 (223.83, 450.22)
2035	Female	11581848.92 (7034407.21, 16129290.62)	344.08 (216.45, 471.7)
2036	Female	11950654.15 (6811868.98, 17089439.32)	351.31 (208.23, 494.39)
2037	Female	12336004.09 (6548698.05, 18123310.14)	358.73 (199.13, 518.33)
2038	Female	12734287.89 (6239239.76, 19229336.02)	366.35 (189.12, 543.57)
2039	Female	13150352.65 (5881465.24, 20419240.06)	374.17 (178.17, 570.17)
2040	Female	13584944.07 (5470302.23, 21699585.91)	382.18 (166.22, 598.15)
2041	Female	14038644.91 (4999843.36, 23077446.45)	390.39 (153.22, 627.57)
2042	Female	14513660.37 (4464113.37, 24563207.37)	398.81 (139.14, 658.49)
2043	Female	15009838.23 (3855158.02, 26164518.43)	407.46 (123.93, 690.98)
2044	Female	15529216.71 (3167234.94, 27892875.68)	416.32 (107.54, 725.1)
2045	Female	16073831.97 (2422680.93, 29761643.12)	425.4 (89.91, 760.9)

2022	Male	8237048.63 (7740566.89, 8733530.37)	275.76 (267.39, 284.13)
2023	Male	8350439.18 (7776922.31, 8923956.04)	277.22 (265.01, 289.43)
2024	Male	8468268.46 (7773828.62, 9162708.3)	278.81 (261.48, 296.13)
2025	Male	8590510.52 (7733324.23, 9447696.81)	280.49 (257.07, 303.92)
2026	Male	8717082.5 (7659083.88, 9775081.11)	282.23 (251.87, 312.6)
2027	Male	8850103.52 (7556047.52, 10144159.53)	284.07 (245.99, 322.15)
2028	Male	8990890.41 (7428037.74, 10553743.08)	286.03 (239.52, 332.54)
2029	Male	9138576.14 (7275473.43, 11001678.85)	288.09 (232.47, 343.72)
2030	Male	9292667.25 (7097988.46, 11487346.04)	290.23 (224.81, 355.65)
2031	Male	9452986.57 (6894621.99, 12011351.16)	292.4 (216.52, 368.28)
2032	Male	9621522.42 (6665429.66, 12577615.17)	294.66 (207.64, 381.68)
2033	Male	9797974.84 (6409191.46, 13186758.23)	297.02 (198.17, 395.86)
2034	Male	9983270.12 (6124748.12, 13841792.12)	299.45 (188.11, 410.8)
2035	Male	10176917.02 (5809531.14, 14544302.9)	301.94 (177.42, 426.46)
2036	Male	10378629.92 (5460641.7, 15296618.14)	304.47 (166.09, 442.86)
2037	Male	10591294.07 (5076341.02, 16106247.13)	307.06 (154.11, 460.02)
2038	Male	10809714.88 (4651154.04, 16968275.71)	309.74 (141.5, 477.99)
2039	Male	11038577.83 (4183739.31, 17893416.34)	312.49 (128.22, 496.75)
2040	Male	11278372.25 (3669876.83, 18886867.68)	315.28 (114.27, 516.29)
2041	Male	11529717.18 (3104615.2, 19954819.17)	318.12 (99.62, 536.61)
2042	Male	11795308.98 (2482805.68, 21107812.29)	321.02 (84.27, 557.76)
2043	Male	12074318.86 (1811473.74, 22351474.95)	323.99 (68.21, 579.77)
2044	Male	12368070.96 (1090123.81, 23695225.85)	327.03 (51.41, 602.64)
2045	Male	12677971.78 (451456.96, 25150408.12)	330.12 (33.87, 626.36)
**YLDs**			
2022	Both	6638776.51 (6127382.52, 7150170.5)	111.55 (102.95, 120.14)

2023	Both	6763967.79 (6192228.25, 7335707.33)	112.72 (103.19, 122.25)
2024	Both	6893048.83 (6230160.38, 7555937.29)	113.97 (103.01, 124.94)
2025	Both	7026454.84 (6241392.79, 7811516.89)	115.28 (102.4, 128.17)
2026	Both	7164622.89 (6227422.11, 8101823.68)	116.66 (101.38, 131.93)
2027	Both	7309317.98 (6190982.51, 8427653.44)	118.11 (100.01, 136.2)
2028	Both	7461490.25 (6134220.68, 8788759.82)	119.65 (98.33, 140.97)
2029	Both	7620361.74 (6056992.86, 9183730.61)	121.28 (96.35, 146.21)
2030	Both	7785849.68 (5959057.61, 9612641.74)	123 (94.07, 151.92)
2031	Both	7958080.1 (5839748.03, 10076412.16)	124.81 (91.5, 158.11)
2032	Both	8138576.67 (5698999.51, 10578153.83)	126.72 (88.62, 164.81)
2033	Both	8327264.57 (5535779.1, 11118750.04)	128.75 (85.45, 172.04)
2034	Both	8524893.6 (5348977.86, 11700809.34)	130.9 (81.96, 179.84)
2035	Both	8731146.59 (5136400.26, 12325892.92)	133.18 (78.13, 188.22)
2036	Both	8945802.76 (4895556.15, 12996049.38)	135.58 (73.93, 197.23)
2037	Both	9170907.34 (4624810.42, 13717004.26)	138.13 (69.33, 206.94)
2038	Both	9402758.88 (4319745.01, 14485772.76)	140.85 (64.31, 217.4)
2039	Both	9645138.64 (3979134.83, 15311142.45)	143.75 (58.82, 228.68)
2040	Both	9898497.83 (3599400.24, 16197595.43)	146.82 (52.8, 240.84)
2041	Both	10163340.61 (3176373.09, 17150308.12)	150.09 (46.2, 253.97)
2042	Both	10441618.25 (2705768.1, 18177468.4)	153.57 (38.95, 268.2)
2043	Both	10732893.32 (2187201.33, 19284128.78)	157.3 (31.08, 283.62)
2044	Both	11038325.18 (1620062.32, 20478109.75)	161.28 (22.57, 300.39)
2045	Both	11359230.35 (1066021.94, 21768928.74)	165.54 (14.47, 318.63)
2022	Female	3213261.03 (2897454.45, 3529067.61)	107.74 (103.12, 112.37)
2023	Female	3296586.85 (2953154.5, 3640019.2)	109.6 (103.69, 115.52)
2024	Female	3381996.3 (2998581.07, 3765411.53)	111.53 (103.82, 119.23)

2025	Female	3469992.89 (3032721.63, 3907264.15)	113.5 (103.6, 123.41)
2026	Female	3561084.92 (3055372.92, 4066796.92)	115.53 (103.04, 128.02)
2027	Female	3656143.73 (3067149.22, 4245138.24)	117.63 (102.2, 133.05)
2028	Female	3755487.79 (3068509.75, 4442465.83)	119.79 (101.09, 138.48)
2029	Female	3858771.89 (3059183.49, 4658360.3)	122.01 (99.71, 144.31)
2030	Female	3966178.54 (3039111.55, 4893245.54)	124.29 (98.05, 150.53)
2031	Female	4077926.74 (3008020.72, 5147832.77)	126.63 (96.12, 157.15)
2032	Female	4194710.4 (2965797.23, 5423623.57)	129.04 (93.9, 164.17)
2033	Female	4316542.93 (2911843.92, 5721241.94)	131.51 (91.39, 171.63)
2034	Female	4443842.38 (2845517.74, 6042167.02)	134.05 (88.58, 179.52)
2035	Female	4576543.2 (2765659.52, 6387426.88)	136.65 (85.46, 187.85)
2036	Female	4714553.21 (2670922.61, 6758183.81)	139.32 (82, 196.63)
2037	Female	4858724.66 (2560326.47, 7157122.86)	142.05 (78.2, 205.89)
2038	Female	5007501.21 (2431638.7, 7583363.71)	144.85 (74.05, 215.65)
2039	Female	5162772.84 (2284120.8, 8041424.89)	147.72 (69.54, 225.91)
2040	Female	5324827.62 (2115852.49, 8533802.76)	150.66 (64.64, 236.69)
2041	Female	5493922.3 (1924602.54, 9063242.06)	153.67 (59.33, 248.01)
2042	Female	5670919.13 (1708098.06, 9633740.21)	156.75 (53.61, 259.89)
2043	Female	5855689.99 (1463272.41, 10248107.58)	159.91 (47.46, 272.36)
2044	Female	6048938.79 (1188742.4, 10910593.73)	163.14 (40.85, 285.43)
2045	Female	6251451.48 (892418.71, 11626311.09)	166.45 (33.78, 299.13)
2022	Male	3425515.48 (3229928.07, 3621102.9)	114.77 (111.4, 118.15)
2023	Male	3467380.94 (3239073.75, 3695688.13)	115.22 (110.23, 120.21)
2024	Male	3511052.53 (3231579.31, 3790525.75)	115.73 (108.6, 122.86)
2025	Male	3556461.95 (3208671.17, 3904252.74)	116.27 (106.61, 125.94)
2026	Male	3603537.97 (3172049.19, 4035026.76)	116.84 (104.3, 129.38)

2027	Male	3653174.24 (3123833.29, 4182515.2)	117.44 (101.72, 133.17)
2028	Male	3706002.46 (3065710.93, 4346293.99)	118.1 (98.9, 137.3)
2029	Male	3761589.84 (2997809.38, 4525370.31)	118.8 (95.85, 141.75)
2030	Male	3819671.13 (2919946.06, 4719396.2)	119.53 (92.56, 146.5)
2031	Male	3880153.35 (2831727.31, 4928579.39)	120.27 (89.02, 151.52)
2032	Male	3943866.27 (2733202.28, 5154530.26)	121.04 (85.24, 156.85)
2033	Male	4010721.64 (2623935.19, 5397508.1)	121.86 (81.23, 162.49)
2034	Male	4081051.22 (2503460.12, 5658642.32)	122.71 (76.98, 168.43)
2035	Male	4154603.39 (2370740.74, 5938466.05)	123.57 (72.49, 174.65)
2036	Male	4231249.55 (2224633.54, 6237865.56)	124.45 (67.75, 181.15)
2037	Male	4312182.67 (2064483.95, 6559881.4)	125.35 (62.75, 187.96)
2038	Male	4395257.68 (1888106.3, 6902409.05)	126.29 (57.5, 195.08)
2039	Male	4482365.79 (1695014.03, 7269717.56)	127.25 (52, 202.51)
2040	Male	4573670.21 (1483547.75, 7663792.67)	128.23 (46.23, 210.23)
2041	Male	4669418.31 (1251770.55, 8087066.06)	129.23 (40.2, 218.26)
2042	Male	4770699.12 (997670.04, 8543728.19)	130.25 (33.89, 226.6)
2043	Male	4877203.33 (723928.93, 9036021.2)	131.29 (27.3, 235.28)
2044	Male	4989386.39 (431319.93, 9567516.02)	132.36 (20.44, 244.28)
2045	Male	5107778.87 (173603.23, 10142617.65)	133.45 (13.29, 253.61)

Notes: ASR: age-standardized rates; YLDs: years lived with disability.

Table S66. Prevalence and YLDs of infectious disorders in the worldwide of future forecasts using bayesian age-period-cohort model.

Year	Sex	Number	ASR
**Prevalence**			
2022	Both	5416863.79 (5299127.79, 5534599.78)	66.88 (65.41, 68.35)
2023	Both	5487430.14 (5340067.8, 5634792.48)	66.85 (65.03, 68.67)
2024	Both	5558457.05 (5365951.41, 5750962.7)	66.82 (64.47, 69.17)
2025	Both	5629225.95 (5378484.28, 5879967.61)	66.79 (63.76, 69.81)
2026	Both	5699582.78 (5379515.41, 6019650.16)	66.76 (62.95, 70.57)
2027	Both	5769049.14 (5369825.98, 6168272.31)	66.74 (62.04, 71.43)
2028	Both	5838678.42 (5351405.33, 6325951.51)	66.71 (61.05, 72.38)
2029	Both	5909270.58 (5325552.3, 6492988.85)	66.7 (59.99, 73.4)
2030	Both	5980427.88 (5292190.69, 6668665.07)	66.68 (58.87, 74.5)
2031	Both	6052034.94 (5251306.37, 6852763.51)	66.68 (57.69, 75.66)
2032	Both	6123509.22 (5202363.29, 7044655.15)	66.68 (56.46, 76.9)
2033	Both	6195332.33 (5145935.06, 7244729.59)	66.69 (55.17, 78.2)
2034	Both	6268310.92 (5082738.02, 7453883.82)	66.7 (53.83, 79.57)
2035	Both	6342022.59 (5012359.17, 7671686.01)	66.72 (52.45, 81)
2036	Both	6416291.19 (4934506.57, 7898075.81)	66.76 (51.02, 82.5)
2037	Both	6490530.16 (4848511.3, 8132549.03)	66.8 (49.54, 84.06)
2038	Both	6565084.94 (4754623.01, 8375546.86)	66.86 (48.02, 85.7)
2039	Both	6640671.62 (4653279.01, 8628064.22)	66.93 (46.45, 87.4)
2040	Both	6716829.95 (4543975.09, 8889684.82)	67.01 (44.84, 89.17)
2041	Both	6793436.68 (4426383.68, 9160489.69)	67.1 (43.18, 91.02)
2042	Both	6869936.65 (4299837.43, 9440035.87)	67.21 (41.47, 92.95)
2043	Both	6946606.18 (4164403.52, 9728808.85)	67.33 (39.71, 94.96)

2044	Both	7024142.12 (4020329.15, 10027955.08)	67.47 (37.9, 97.04)
2045	Both	7102119.95 (3867102.45, 10337137.46)	67.63 (36.04, 99.21)
2022	Female	2866675.22 (2801729.62, 2931620.81)	69.56 (68.76, 70.36)
2023	Female	2906029.27 (2824656.37, 2987402.18)	69.53 (68.18, 70.87)
2024	Female	2945707.67 (2839200.76, 3052214.58)	69.49 (67.46, 71.52)
2025	Female	2985342.82 (2846345.53, 3124340.12)	69.46 (66.63, 72.28)
2026	Female	3024882.61 (2847152.5, 3202612.72)	69.42 (65.71, 73.13)
2027	Female	3064077.27 (2842066.27, 3286088.27)	69.38 (64.71, 74.06)
2028	Female	3103420.85 (2832091.39, 3374750.31)	69.35 (63.62, 75.07)
2029	Female	3143345.74 (2817924.46, 3468767.03)	69.3 (62.47, 76.14)
2030	Female	3183642.13 (2799522.37, 3567761.9)	69.26 (61.25, 77.27)
2031	Female	3224269.12 (2776886.79, 3671651.46)	69.22 (59.98, 78.46)
2032	Female	3264910.08 (2749716.03, 3780104.13)	69.18 (58.64, 79.72)
2033	Female	3305787.73 (2718274.28, 3893301.18)	69.14 (57.25, 81.02)
2034	Female	3347324.11 (2682920.98, 4011727.23)	69.09 (55.8, 82.38)
2035	Female	3389287.87 (2643418.71, 4135157.04)	69.04 (54.3, 83.78)
2036	Female	3431592 (2599602.19, 4263581.81)	69 (52.75, 85.24)
2037	Female	3473915.45 (2551094.29, 4396736.61)	68.95 (51.16, 86.74)
2038	Female	3516439.25 (2498008.59, 4534869.92)	68.91 (49.52, 88.29)
2039	Female	3559558.73 (2440564.93, 4678552.52)	68.86 (47.83, 89.88)
2040	Female	3603020.51 (2378471.18, 4827569.83)	68.81 (46.1, 91.51)
2041	Female	3646752.89 (2311526.31, 4981979.48)	68.76 (44.34, 93.18)
2042	Female	3690447.53 (2239347.28, 5141547.79)	68.71 (42.52, 94.89)
2043	Female	3734260.15 (2161951.71, 5306568.59)	68.66 (40.67, 96.65)
2044	Female	3778595.96 (2079458.7, 5477733.23)	68.61 (38.78, 98.43)
2045	Female	3823241.29 (1991575.41, 5654907.17)	68.56 (36.85, 100.26)

2022	Male	2550188.57 (2497398.16, 2602978.98)	64.05 (63.38, 64.73)
2023	Male	2581400.86 (2515411.42, 2647390.3)	64.02 (62.9, 65.14)
2024	Male	2612749.38 (2526750.65, 2698748.12)	63.98 (62.3, 65.66)
2025	Male	2643883.12 (2532138.75, 2755627.5)	63.94 (61.61, 66.28)
2026	Male	2674700.17 (2532362.9, 2817037.44)	63.9 (60.84, 66.96)
2027	Male	2704971.88 (2527759.71, 2882184.04)	63.86 (60.01, 67.72)
2028	Male	2735257.57 (2519313.94, 2951201.2)	63.82 (59.11, 68.54)
2029	Male	2765924.83 (2507627.84, 3024221.82)	63.78 (58.15, 69.41)
2030	Male	2796785.75 (2492668.33, 3100903.17)	63.73 (57.14, 70.32)
2031	Male	2827765.81 (2474419.58, 3181112.04)	63.69 (56.08, 71.29)
2032	Male	2858599.14 (2452647.26, 3264551.02)	63.64 (54.98, 72.31)
2033	Male	2889544.6 (2427660.79, 3351428.41)	63.6 (53.82, 73.37)
2034	Male	2920986.82 (2399817.04, 3442156.59)	63.55 (52.62, 74.47)
2035	Male	2952734.72 (2368940.46, 3536528.97)	63.49 (51.38, 75.61)
2036	Male	2984699.19 (2334904.38, 3634494)	63.44 (50.1, 76.78)
2037	Male	3016614.71 (2297417, 3735812.41)	63.39 (48.78, 78)
2038	Male	3048645.68 (2256614.43, 3840676.94)	63.34 (47.42, 79.25)
2039	Male	3081112.89 (2212714.08, 3949511.69)	63.28 (46.03, 80.54)
2040	Male	3113809.44 (2165503.91, 4062114.98)	63.22 (44.59, 81.85)
2041	Male	3146683.79 (2114857.37, 4178510.21)	63.17 (43.13, 83.2)
2042	Male	3179489.12 (2060490.16, 4298488.09)	63.11 (41.63, 84.59)
2043	Male	3212346.04 (2002451.81, 4422240.26)	63.05 (40.1, 86)
2044	Male	3245546.15 (1940870.45, 4550221.86)	62.99 (38.54, 87.44)
2045	Male	3278878.66 (1875527.04, 4682230.28)	62.93 (36.95, 88.91)
**YLDs**			
2022	Both	230815.52 (222804.1, 238826.95)	2.71 (2.61, 2.8)

2023	Both	234426.21 (225242.15, 243610.28)	2.7 (2.59, 2.81)
2024	Both	238096.19 (227042.91, 249149.48)	2.7 (2.57, 2.83)
2025	Both	241790.48 (228207.72, 255373.23)	2.7 (2.54, 2.85)
2026	Both	245478.09 (228773.74, 262182.44)	2.69 (2.51, 2.88)
2027	Both	249135.54 (228778.36, 269492.73)	2.69 (2.47, 2.91)
2028	Both	252800.55 (228310.51, 277290.58)	2.69 (2.43, 2.95)
2029	Both	256520.07 (227445.38, 285594.76)	2.68 (2.38, 2.99)
2030	Both	260264.19 (226172.6, 294355.79)	2.68 (2.33, 3.04)
2031	Both	264007.11 (224475.68, 303538.53)	2.68 (2.28, 3.08)
2032	Both	267726.68 (222337.66, 313115.7)	2.68 (2.22, 3.14)
2033	Both	271457.39 (219796.61, 323118.17)	2.68 (2.17, 3.19)
2034	Both	275242.71 (216889.26, 333596.16)	2.68 (2.11, 3.25)
2035	Both	279055.8 (213588.85, 344522.75)	2.68 (2.05, 3.31)
2036	Both	282869.68 (209865.41, 355873.94)	2.68 (1.98, 3.37)
2037	Both	286662.48 (205692.79, 367632.18)	2.68 (1.92, 3.44)
2038	Both	290469.29 (201094.84, 379843.74)	2.68 (1.85, 3.51)
2039	Both	294335.09 (196095.26, 392574.93)	2.68 (1.78, 3.58)
2040	Both	298235.38 (190664.04, 405806.71)	2.68 (1.71, 3.66)
2041	Both	302145.05 (184768.71, 419521.39)	2.69 (1.64, 3.74)
2042	Both	306044.83 (178380.57, 433709.1)	2.69 (1.56, 3.82)
2043	Both	309967.35 (171509.54, 448425.16)	2.69 (1.48, 3.91)
2044	Both	313955.73 (164164.32, 463747.14)	2.7 (1.4, 4)
2045	Both	317990.42 (156313, 479667.84)	2.71 (1.32, 4.09)
2022	Female	123900.77 (119680.4, 128121.15)	2.83 (2.79, 2.87)
2023	Female	125833.94 (120958.23, 130709.64)	2.83 (2.76, 2.89)
2024	Female	127800.19 (121878.1, 133722.27)	2.82 (2.73, 2.92)

2025	Female	129780.51 (122445.81, 137115.21)	2.82 (2.69, 2.95)
2026	Female	131758.26 (122685.06, 140831.46)	2.81 (2.64, 2.98)
2027	Female	133722.76 (122619.22, 144826.3)	2.81 (2.59, 3.02)
2028	Female	135694.47 (122296.73, 149092.21)	2.8 (2.54, 3.06)
2029	Female	137697.56 (121756.84, 153638.28)	2.8 (2.49, 3.11)
2030	Female	139715.4 (120993.24, 158437.57)	2.79 (2.43, 3.15)
2031	Female	141733.44 (119996.07, 163470.82)	2.79 (2.37, 3.2)
2032	Female	143741.42 (118757.17, 168725.67)	2.78 (2.31, 3.25)
2033	Female	145757.85 (117296.55, 174219.15)	2.78 (2.25, 3.31)
2034	Female	147804.89 (115632.43, 179977.34)	2.77 (2.18, 3.36)
2035	Female	149866.78 (113749.04, 185984.53)	2.77 (2.12, 3.42)
2036	Female	151927.75 (111628.98, 192226.52)	2.76 (2.05, 3.48)
2037	Female	153977.9 (109259.19, 198696.62)	2.76 (1.98, 3.54)
2038	Female	156037.05 (106652.65, 205421.45)	2.75 (1.9, 3.6)
2039	Female	158128.81 (103820.96, 212436.66)	2.75 (1.83, 3.67)
2040	Female	160238.19 (100746.3, 219730.09)	2.74 (1.75, 3.73)
2041	Female	162349.49 (97409.39, 227289.59)	2.74 (1.67, 3.8)
2042	Female	164454.18 (93795.54, 235112.82)	2.73 (1.59, 3.87)
2043	Female	166571.38 (89910.3, 243232.46)	2.73 (1.51, 3.94)
2044	Female	168724.58 (85757.43, 251691.74)	2.72 (1.43, 4.02)
2045	Female	170902.14 (81318.4, 260485.87)	2.72 (1.35, 4.09)
2022	Male	106914.75 (103123.69, 110705.81)	2.57 (2.54, 2.61)
2023	Male	108592.28 (104283.92, 112900.64)	2.57 (2.51, 2.63)
2024	Male	110296.01 (105164.81, 115427.21)	2.57 (2.48, 2.65)
2025	Male	112009.96 (105761.91, 118258.02)	2.57 (2.45, 2.68)
2026	Male	113719.83 (106088.69, 121350.98)	2.56 (2.42, 2.71)

2027	Male	115412.78 (106159.14, 124666.43)	2.56 (2.38, 2.75)
2028	Male	117106.08 (106013.78, 128198.37)	2.56 (2.34, 2.78)
2029	Male	118822.51 (105688.54, 131956.48)	2.56 (2.29, 2.82)
2030	Male	120548.79 (105179.36, 135918.22)	2.56 (2.24, 2.87)
2031	Male	122273.66 (104479.61, 140067.72)	2.55 (2.2, 2.91)
2032	Male	123985.26 (103580.49, 144390.03)	2.55 (2.14, 2.96)
2033	Male	125699.54 (102500.06, 148899.02)	2.55 (2.09, 3)
2034	Male	127437.83 (101256.83, 153618.82)	2.55 (2.04, 3.05)
2035	Male	129189.02 (99839.81, 158538.23)	2.54 (1.98, 3.11)
2036	Male	130941.93 (98236.44, 163647.42)	2.54 (1.92, 3.16)
2037	Male	132684.58 (96433.6, 168935.56)	2.54 (1.86, 3.22)
2038	Male	134432.24 (94442.19, 174422.29)	2.54 (1.8, 3.27)
2039	Male	136206.29 (92274.31, 180138.27)	2.53 (1.74, 3.33)
2040	Male	137997.18 (89917.74, 186076.62)	2.53 (1.67, 3.39)
2041	Male	139795.56 (87359.32, 192231.81)	2.53 (1.6, 3.45)
2042	Male	141590.65 (84585.02, 198596.28)	2.53 (1.54, 3.51)
2043	Male	143395.97 (81599.24, 205192.7)	2.52 (1.47, 3.58)
2044	Male	145231.15 (78406.89, 212055.4)	2.52 (1.4, 3.64)
2045	Male	147088.29 (74994.6, 219181.97)	2.52 (1.32, 3.71)

Notes: ASR: age-standardized rates; YLDs: years lived with disability.

Table S67. Prevalence and YLDs of neoplasms in the worldwide of future forecasts using bayesian age-period-cohort model.

Year	Sex	Number	ASR
**Prevalence**			
2022	Both	158180979.3 (153057187.45, 163304771.15)	1810.43 (1751.73, 1869.12)
2023	Both	160401872.81 (154261390.09, 166542355.54)	1800.18 (1731.18, 1869.17)
2024	Both	162670595.8 (154905632.7, 170435558.9)	1790.07 (1704.52, 1875.62)
2025	Both	164952156.4 (155023331.06, 174880981.74)	1779.99 (1672.73, 1887.25)
2026	Both	167195684.77 (154638143.63, 179753225.91)	1769.72 (1636.67, 1902.78)
2027	Both	169352278.52 (153755664.16, 184948892.87)	1759.16 (1597, 1921.33)
2028	Both	171496939.1 (152495388.25, 190498489.86)	1748.81 (1554.87, 1942.74)
2029	Both	173683101.74 (150931635.04, 196434568.45)	1738.79 (1510.83, 1966.74)
2030	Both	175877380.98 (149047057.88, 202707704.08)	1728.97 (1465.02, 1992.93)
2031	Both	178032396.2 (146803732.78, 209261059.62)	1719.14 (1417.37, 2020.91)
2032	Both	180108163.86 (144167633.47, 216048694.25)	1709.23 (1367.93, 2050.54)
2033	Both	182179348.86 (141211935.69, 223146762.03)	1699.74 (1317.26, 2082.22)
2034	Both	184299441.7 (137980216.95, 230618666.45)	1690.78 (1265.57, 2115.99)
2035	Both	186433348.47 (134441279.27, 238425417.68)	1682.19 (1212.77, 2151.62)
2036	Both	188529734.15 (130549892.9, 246509575.4)	1673.78 (1158.7, 2188.85)
2037	Both	190556314.04 (126274454.45, 254838173.63)	1665.53 (1103.32, 2227.74)
2038	Both	192589601.75 (121669437.2, 263509766.31)	1657.9 (1046.97, 2268.82)
2039	Both	194682233.4 (116761158.21, 272603308.6)	1650.94 (989.69, 2312.19)
2040	Both	196798283.37 (111515228.69, 282081338.05)	1644.48 (931.32, 2357.64)
2041	Both	198888869.76 (105889587.36, 291888152.16)	1638.33 (871.66, 2405)
2042	Both	200937425.31 (99859153.28, 302015697.34)	1632.58 (810.66, 2454.51)
2043	Both	203016259.84 (93453614.8, 312578904.89)	1627.63 (748.46, 2506.81)

2044	Both	205171065.58 (86676563.48, 323665567.67)	1623.49 (684.96, 2562.02)
2045	Both	207367369.07 (79490623.98, 335244114.17)	1619.94 (619.94, 2619.94)
2022	Female	95361896.02 (92415325.83, 98308466.2)	2141.25 (2110.31, 2172.18)
2023	Female	96514603.07 (93004992.28, 100024213.86)	2128.6 (2077.87, 2179.33)
2024	Female	97684024.47 (93274453.08, 102093595.85)	2116.12 (2040.59, 2191.66)
2025	Female	98848856.16 (93241214.92, 104456497.4)	2103.5 (1999.55, 2207.46)
2026	Female	99985197.55 (92923790.74, 107046604.37)	2090.46 (1955.11, 2225.81)
2027	Female	101075590.66 (92335407.29, 109815774.03)	2076.95 (1907.57, 2246.32)
2028	Female	102164368.02 (91547222.25, 112781513.79)	2063.64 (1858, 2269.28)
2029	Female	103268437.43 (90591033.39, 115945841.47)	2050.52 (1806.68, 2294.36)
2030	Female	104366672.22 (89456589.18, 119276755.27)	2037.28 (1753.54, 2321.03)
2031	Female	105436090.21 (88126679.8, 122745500.62)	2023.67 (1698.48, 2348.86)
2032	Female	106461951.96 (86589005.5, 126334898.42)	2009.65 (1641.55, 2377.75)
2033	Female	107485631.02 (84886111.92, 130085150.12)	1995.83 (1583.47, 2408.2)
2034	Female	108525112.9 (83035257.4, 134014968.34)	1982.21 (1524.39, 2440.03)
2035	Female	109559414.52 (81019860.3, 138098968.73)	1968.5 (1464.2, 2472.8)
2036	Female	110565350.62 (78819852.24, 142310849)	1954.49 (1402.82, 2506.17)
2037	Female	111530462.88 (76422202.27, 146638723.49)	1940.22 (1340.28, 2540.16)
2038	Female	112492656.78 (73857078.48, 151128235.08)	1926.14 (1277.07, 2575.2)
2039	Female	113469868.74 (71135523.36, 155804214.12)	1912.19 (1213.25, 2611.12)
2040	Female	114443585.51 (68242244.2, 160644926.82)	1898.12 (1148.75, 2647.5)
2041	Female	115395925.35 (65161138.98, 165630711.72)	1883.83 (1083.53, 2684.13)
2042	Female	116321491.15 (61882058.6, 170760923.7)	1869.39 (1017.67, 2721.11)
2043	Female	117252114.75 (58421925.03, 176082304.47)	1855.12 (951.45, 2758.79)
2044	Female	118202254.65 (54783260.97, 181621248.33)	1840.93 (884.9, 2796.95)
2045	Female	119156836.86 (50951665.31, 187362008.42)	1826.58 (817.98, 2835.18)

2022	Male	62819083.28 (60641861.61, 64996304.94)	1501.66 (1476.41, 1526.9)
2023	Male	63887269.75 (61256397.81, 66518141.68)	1493.58 (1452.24, 1534.92)
2024	Male	64986571.33 (61631179.62, 68341963.05)	1485.42 (1423.76, 1547.08)
2025	Male	66103300.24 (61782116.14, 70424484.34)	1477.24 (1392.21, 1562.27)
2026	Male	67210487.22 (61714352.89, 72706621.54)	1468.86 (1357.97, 1579.74)
2027	Male	68276687.85 (61420256.87, 75133118.83)	1460.03 (1321.11, 1598.94)
2028	Male	69332571.03 (60948166, 77716976.07)	1450.96 (1282.18, 1619.74)
2029	Male	70414664.31 (60340601.64, 80488726.98)	1441.89 (1241.64, 1642.14)
2030	Male	71510708.75 (59590468.69, 83430948.82)	1432.84 (1199.68, 1666.01)
2031	Male	72596305.99 (58677052.98, 86515559.01)	1423.6 (1156.22, 1690.98)
2032	Male	73646211.9 (57578627.97, 89713795.83)	1413.99 (1111.2, 1716.78)
2033	Male	74693717.84 (56325823.76, 93061611.91)	1404.3 (1065.02, 1743.57)
2034	Male	75774328.83 (54944959.55, 96603698.11)	1394.73 (1017.98, 1771.48)
2035	Male	76873933.96 (53421418.96, 100326448.95)	1385.24 (970.12, 1800.37)
2036	Male	77964383.53 (51730040.66, 104198726.4)	1375.58 (921.31, 1829.84)
2037	Male	79025851.16 (49852252.18, 108199450.14)	1365.66 (871.56, 1859.77)
2038	Male	80096944.98 (47812358.72, 112381531.23)	1355.83 (821.18, 1890.47)
2039	Male	81212364.66 (45625634.85, 116799094.48)	1346.22 (770.35, 1922.09)
2040	Male	82354697.86 (43272984.49, 121436411.22)	1336.72 (719.05, 1954.38)
2041	Male	83492944.41 (40728448.38, 126257440.44)	1327.02 (667.15, 1986.89)
2042	Male	84615934.16 (37977094.68, 131254773.64)	1317.18 (614.7, 2019.66)
2043	Male	85764145.09 (35031689.76, 136496600.42)	1307.56 (561.96, 2053.17)
2044	Male	86968810.93 (31893302.52, 142044319.34)	1298.25 (509, 2087.5)
2045	Male	88210532.21 (28538958.67, 147882105.75)	1289.02 (455.77, 2122.26)
**YLDs**			
2022	Both	8271723.1 (7906236.12, 8637210.07)	93.04 (88.92, 97.15)

2023	Both	8415631.75 (8002449.33, 8828814.17)	92.4 (87.86, 96.95)
2024	Both	8565238.66 (8076521.81, 9053955.52)	91.79 (86.54, 97.03)
2025	Both	8717423.54 (8125575.08, 9309271.99)	91.18 (84.98, 97.38)
2026	Both	8868732.76 (8148132.64, 9589332.88)	90.58 (83.21, 97.95)
2027	Both	9015804.69 (8143243.83, 9888365.54)	89.97 (81.25, 98.69)
2028	Both	9163439.39 (8117986.71, 10208892.07)	89.38 (79.17, 99.59)
2029	Both	9315676.88 (8077393.1, 10553960.66)	88.82 (77, 100.65)
2030	Both	9469512.34 (8019401.13, 10919623.54)	88.28 (74.74, 101.82)
2031	Both	9621760.45 (7941344.92, 11302175.99)	87.74 (72.39, 103.09)
2032	Both	9769761.78 (7841032.62, 11698490.95)	87.2 (69.96, 104.44)
2033	Both	9918757.7 (7723477.19, 12114038.22)	86.7 (67.48, 105.92)
2034	Both	10072689.08 (7591776.18, 12553601.99)	86.23 (64.96, 107.5)
2035	Both	10228065.29 (7442810.95, 13013319.63)	85.79 (62.39, 109.19)
2036	Both	10381061.4 (7273176.13, 13488946.67)	85.35 (59.75, 110.94)
2037	Both	10529482.75 (7080861.24, 13978104.27)	84.92 (57.06, 112.78)
2038	Both	10679476.92 (6870296.53, 14488657.3)	84.54 (54.33, 114.74)
2039	Both	10835126.28 (6643616.82, 15026635.74)	84.2 (51.57, 116.83)
2040	Both	10992443.31 (6397410.96, 15587475.65)	83.89 (48.76, 119.02)
2041	Both	11147239.95 (6128281.89, 16166198)	83.59 (45.89, 121.3)
2042	Both	11298257.06 (5834751.11, 16761763.01)	83.31 (42.95, 123.66)
2043	Both	11451877.57 (5519971.04, 17383784.1)	83.07 (39.96, 126.18)
2044	Both	11611925.19 (5184762.52, 18039087.86)	82.89 (36.93, 128.86)
2045	Both	11774233.32 (4825750.67, 18722715.98)	82.74 (33.82, 131.66)
2022	Female	4138116.88 (3972376.44, 4303857.33)	89.05 (87.45, 90.65)
2023	Female	4211228.98 (4021530.54, 4400927.41)	88.58 (86.18, 90.99)
2024	Female	4286967.91 (4059234.61, 4514701.21)	88.13 (84.69, 91.57)

2025	Female	4363672.29 (4084245.21, 4643099.37)	87.68 (83.05, 92.32)
2026	Female	4439875.47 (4096202.84, 4783548.1)	87.22 (81.26, 93.18)
2027	Female	4514488.02 (4095153.06, 4933822.98)	86.74 (79.33, 94.15)
2028	Female	4590186.37 (4084904.06, 5095468.69)	86.29 (77.33, 95.24)
2029	Female	4668318.6 (4067345.6, 5269291.6)	85.85 (75.27, 96.43)
2030	Female	4747051.29 (4041126.3, 5452976.27)	85.41 (73.12, 97.7)
2031	Female	4824677.85 (4004761.13, 5644594.58)	84.95 (70.89, 99.01)
2032	Female	4900222.77 (3957370.64, 5843074.91)	84.46 (68.56, 100.36)
2033	Female	4976871.74 (3901935.69, 6051807.8)	84 (66.2, 101.8)
2034	Female	5056074.06 (3839592.77, 6272555.36)	83.56 (63.8, 103.33)
2035	Female	5135628.73 (3768438.94, 6502818.51)	83.12 (61.34, 104.9)
2036	Female	5213313.59 (3686542.55, 6740084.63)	82.65 (58.82, 106.49)
2037	Female	5288185.27 (3592995.85, 6983374.69)	82.16 (56.24, 108.08)
2038	Female	5364109.75 (3490628.94, 7237590.55)	81.69 (53.62, 109.75)
2039	Female	5442779.18 (3380319.02, 7505239.35)	81.25 (51, 111.5)
2040	Female	5521779.39 (3260129.91, 7783428.86)	80.8 (48.33, 113.27)
2041	Female	5598671.85 (3128194.54, 8069149.17)	80.32 (45.61, 115.03)
2042	Female	5672835.9 (2983819.98, 8361851.82)	79.81 (42.85, 116.78)
2043	Female	5748446.36 (2829227.26, 8667665.46)	79.33 (40.07, 118.58)
2044	Female	5827179.84 (2664776.87, 8989582.82)	78.87 (37.28, 120.45)
2045	Female	5906563.26 (2488650.96, 9324475.57)	78.4 (34.47, 122.34)
2022	Male	4133606.21 (3933859.67, 4333352.75)	99.5 (97.35, 101.65)
2023	Male	4204402.77 (3980918.78, 4427886.75)	98.64 (95.61, 101.67)
2024	Male	4278270.76 (4017287.2, 4539254.31)	97.8 (93.61, 101.98)
2025	Male	4353751.24 (4041329.87, 4666172.62)	96.96 (91.43, 102.48)
2026	Male	4428857.3 (4051929.81, 4805784.79)	96.12 (89.11, 103.14)

2027	Male	4501316.67 (4048090.77, 4954542.57)	95.28 (86.65, 103.91)
2028	Male	4573253.02 (4033082.65, 5113423.38)	94.44 (84.1, 104.79)
2029	Male	4647358.28 (4010047.5, 5284669.06)	93.61 (81.47, 105.75)
2030	Male	4722461.05 (3978274.83, 5466647.27)	92.79 (78.78, 106.81)
2031	Male	4797082.6 (3936583.79, 5657581.41)	91.98 (76.03, 107.93)
2032	Male	4869539.01 (3883661.98, 5855416.04)	91.17 (73.21, 109.13)
2033	Male	4941885.96 (3821541.5, 6062230.42)	90.37 (70.35, 110.38)
2034	Male	5016615.02 (3752183.41, 6281046.63)	89.58 (67.46, 111.7)
2035	Male	5092436.56 (3674372.01, 6510501.12)	88.81 (64.54, 113.08)
2036	Male	5167747.81 (3586633.58, 6748862.04)	88.04 (61.59, 114.5)
2037	Male	5241297.48 (3487865.39, 6994729.58)	87.29 (58.6, 115.97)
2038	Male	5315367.17 (3379667.59, 7251066.75)	86.54 (55.6, 117.49)
2039	Male	5392347.1 (3263297.8, 7521396.39)	85.83 (52.58, 119.07)
2040	Male	5470663.92 (3137281.05, 7804046.79)	85.12 (49.55, 120.69)
2041	Male	5548568.09 (3000087.36, 8097048.83)	84.42 (46.5, 122.33)
2042	Male	5625421.16 (2850931.13, 8399911.19)	83.72 (43.44, 124.01)
2043	Male	5703431.22 (2690743.79, 8716118.64)	83.05 (40.36, 125.74)
2044	Male	5784745.35 (2519985.65, 9049505.04)	82.41 (37.29, 127.53)
2045	Male	5867670.06 (2337099.71, 9398240.41)	81.78 (34.21, 129.34)

Notes: ASR: age-standardized rates; YLDs: years lived with disability.

Table S68. Prevalence and YLDs of neck pain in the worldwide of future forecasts using bayesian age-period-cohort model.

Year	Sex	Number	ASR
**Prevalence**			
2022	Both	211105614.81 (206516506.6, 215694723.03)	2434.11 (2381.17, 2487.05)
2023	Both	214334666.99 (208493722.35, 220175611.63)	2433.82 (2367.46, 2500.18)
2024	Both	217588872.17 (209778486.71, 225399257.62)	2433.67 (2346.27, 2521.07)
2025	Both	220848409.51 (210473747.5, 231223071.53)	2433.63 (2319.26, 2548)
2026	Both	224091421.77 (210652357.52, 237530486.02)	2433.69 (2287.68, 2579.71)
2027	Both	227307323.12 (210357947.46, 244256698.78)	2433.92 (2252.36, 2615.49)
2028	Both	230513578.78 (209647525.78, 251379631.77)	2434.45 (2213.98, 2654.92)
2029	Both	233734680.25 (208565948.97, 258903411.53)	2435.26 (2172.9, 2697.63)
2030	Both	236954808.6 (207110510.14, 266799107.06)	2436.35 (2129.32, 2743.37)
2031	Both	240159456.05 (205272227.11, 275046684.99)	2437.72 (2083.39, 2792.05)
2032	Both	243345991.82 (203047244.49, 283644739.15)	2439.47 (2035.22, 2843.72)
2033	Both	246523622.89 (200448848.46, 292598397.33)	2441.67 (1985, 2898.34)
2034	Both	249716991.27 (197498594.47, 301935388.07)	2444.3 (1932.77, 2955.83)
2035	Both	252915813.46 (194185771.03, 311645855.89)	2447.35 (1878.56, 3016.14)
2036	Both	256110430.2 (190496732.72, 321724127.68)	2450.86 (1822.38, 3079.34)
2037	Both	259305630.83 (186425233.5, 332186028.16)	2454.92 (1764.24, 3145.61)
2038	Both	262501667.04 (181968488.19, 343034845.89)	2459.57 (1704.15, 3214.98)
2039	Both	265720929.63 (177136693.94, 354305165.33)	2464.74 (1642.07, 3287.42)
2040	Both	268958470.92 (171917797.65, 365999144.18)	2470.47 (1577.95, 3362.99)
2041	Both	272212237.64 (166298301.68, 378126173.6)	2476.83 (1511.76, 3441.9)
2042	Both	275494243.18 (160269257.13, 390719229.23)	2483.96 (1443.45, 3524.46)
2043	Both	278800637.39 (153817459.79, 403783814.99)	2491.81 (1372.91, 3610.71)

2044	Both	282145615.38 (146937852.56, 417353378.2)	2500.36 (1300.02, 3700.7)
2045	Both	285527854.64 (139613689.66, 431442019.61)	2509.65 (1224.68, 3794.62)
2022	Female	126484746.32 (123804458.81, 129165033.82)	2874.21 (2841.99, 2906.43)
2023	Female	128486845.42 (125067035.35, 131906655.49)	2875.07 (2818.8, 2931.34)
2024	Female	130506964.03 (125924295.38, 135089632.68)	2876.1 (2789.54, 2962.67)
2025	Female	132532859.12 (126436318.96, 138629399.28)	2877.21 (2755.6, 2998.83)
2026	Female	134543848.72 (126638065.97, 142449631.47)	2878.15 (2717.41, 3038.89)
2027	Female	136535726.67 (126556700.72, 146514752.63)	2878.98 (2675.39, 3082.56)
2028	Female	138525187.06 (126231576.66, 150818797.46)	2879.93 (2630.15, 3129.72)
2029	Female	140526815.38 (125688927.15, 155364703.61)	2881.04 (2581.95, 3180.12)
2030	Female	142529385.28 (124925110.99, 160133659.57)	2882.19 (2530.91, 3233.47)
2031	Female	144515762.94 (123927154.56, 165104371.32)	2883.23 (2477, 3289.46)
2032	Female	146485240.91 (122693593.79, 170276888.02)	2884.25 (2420.37, 3348.12)
2033	Female	148449204.12 (121237513.18, 175660895.07)	2885.39 (2361.31, 3409.48)
2034	Female	150422884.5 (119572250.31, 181273518.69)	2886.64 (2299.9, 3473.38)
2035	Female	152398132.67 (117689845.17, 187106420.16)	2887.92 (2236.19, 3539.65)
2036	Female	154361467.13 (115576503.19, 193146431.07)	2889.12 (2170.15, 3608.09)
2037	Female	156316511.41 (113229686.26, 199403336.56)	2890.32 (2101.85, 3678.8)
2038	Female	158267683.22 (110651520.68, 205883845.75)	2891.59 (2031.43, 3751.75)
2039	Female	160228553.98 (107848860.28, 212608247.68)	2892.86 (1958.93, 3826.79)
2040	Female	162196019.9 (104814982.48, 219577057.32)	2894.09 (1884.38, 3903.79)
2041	Female	164163831.2 (101539352.46, 226788309.94)	2895.23 (1807.78, 3982.67)
2042	Female	166141624.16 (98019117.43, 234264130.9)	2896.39 (1729.21, 4063.57)
2043	Female	168130042.54 (94249052.25, 242011032.83)	2897.55 (1648.7, 4146.39)
2044	Female	170136274.95 (90226397.06, 250046152.85)	2898.63 (1566.29, 4230.97)
2045	Female	172160397.52 (85942609.91, 258378185.13)	2899.62 (1482.02, 4317.23)

2022	Male	84620868.5 (82712047.79, 86529689.21)	1986.48 (1963.05, 2009.91)
2023	Male	85847821.57 (83426687, 88268956.14)	1984.9 (1944.07, 2025.73)
2024	Male	87081908.14 (83854191.33, 90309624.95)	1983.27 (1920.58, 2045.96)
2025	Male	88315550.39 (84037428.54, 92593672.25)	1981.57 (1893.65, 2069.49)
2026	Male	89547573.05 (84014291.55, 95080854.55)	1979.97 (1863.94, 2096)
2027	Male	90771596.45 (83801246.74, 97741946.15)	1978.47 (1831.71, 2125.22)
2028	Male	91988391.72 (83415949.13, 100560834.31)	1977 (1797.18, 2156.83)
2029	Male	93207864.87 (82877021.82, 103538707.91)	1975.51 (1760.47, 2190.54)
2030	Male	94425423.32 (82185399.15, 106665447.49)	1973.97 (1721.74, 2226.2)
2031	Male	95643693.11 (81345072.56, 109942313.66)	1972.55 (1681.2, 2263.89)
2032	Male	96860750.91 (80353650.69, 113367851.13)	1971.25 (1638.93, 2303.56)
2033	Male	98074418.77 (79211335.28, 116937502.26)	1970 (1594.97, 2345.03)
2034	Male	99294106.77 (77926344.16, 120661869.38)	1968.73 (1549.35, 2388.11)
2035	Male	100517680.79 (76495925.86, 124539435.73)	1967.43 (1502.13, 2432.73)
2036	Male	101748963.08 (74920229.54, 128577696.62)	1966.23 (1453.47, 2478.98)
2037	Male	102989119.42 (73195547.24, 132782691.61)	1965.15 (1403.39, 2526.91)
2038	Male	104233983.83 (71316967.51, 137151000.14)	1964.11 (1351.9, 2576.33)
2039	Male	105492375.65 (69287833.66, 141696917.65)	1963.03 (1298.99, 2627.07)
2040	Male	106762451.02 (67102815.17, 146422086.87)	1961.91 (1244.72, 2679.09)
2041	Male	108048406.44 (64758949.22, 151337863.66)	1960.88 (1189.2, 2732.55)
2042	Male	109352619.02 (62250139.7, 156455098.34)	1959.98 (1132.45, 2787.5)
2043	Male	110670594.86 (59568407.55, 161772782.17)	1959.1 (1074.45, 2843.75)
2044	Male	112009340.43 (56711455.5, 167307225.35)	1958.18 (1015.19, 2901.16)
2045	Male	113367457.11 (53671079.75, 173063834.48)	1957.22 (954.73, 2959.72)
**YLDs**			
2022	Both	20910126.74 (20464014.14, 21356239.33)	241.38 (236.22, 246.53)

2023	Both	21203798.9 (20627790.53, 21779807.27)	241.13 (234.58, 247.69)
2024	Both	21498758.03 (20720234.59, 22277281.46)	240.9 (232.17, 249.63)
2025	Both	21793351.82 (20753262.93, 22833440.71)	240.68 (229.19, 252.18)
2026	Both	22085651.01 (20734701.79, 23436600.22)	240.48 (225.76, 255.19)
2027	Both	22375071.73 (20669360.18, 24080783.28)	240.29 (221.96, 258.62)
2028	Both	22662882.09 (20562508.74, 24763255.44)	240.13 (217.87, 262.4)
2029	Both	22951221.98 (20418398.36, 25484045.59)	240.01 (213.5, 266.51)
2030	Both	23238771.32 (20237075.52, 26240467.12)	239.91 (208.9, 270.91)
2031	Both	23524319.4 (20017938.19, 27030700.61)	239.84 (204.06, 275.61)
2032	Both	23808002.49 (19760997.22, 27855007.76)	239.81 (199.01, 280.6)
2033	Both	24090223.46 (19467212.95, 28713233.96)	239.82 (193.77, 285.88)
2034	Both	24373161.12 (19138589.37, 29607732.87)	239.89 (188.32, 291.45)
2035	Both	24656055.42 (18774371.13, 30537739.71)	239.99 (182.69, 297.29)
2036	Both	24938265.62 (18373533.95, 31502997.28)	240.14 (176.86, 303.42)
2037	Both	25220646.53 (17935776.22, 32505516.84)	240.35 (170.85, 309.85)
2038	Both	25502634.97 (17460440.06, 33544829.88)	240.62 (164.65, 316.59)
2039	Both	25786186.53 (16948404.58, 34623968.48)	240.94 (158.26, 323.63)
2040	Both	26071127.27 (16398759.33, 35743495.21)	241.32 (151.67, 330.97)
2041	Both	26357659.23 (15810473.1, 36904845.36)	241.77 (144.88, 338.65)
2042	Both	26647324.31 (15182858.08, 38111790.54)	242.29 (137.88, 346.7)
2043	Both	26939121.55 (14514237.81, 39364005.3)	242.89 (130.67, 355.11)
2044	Both	27234158.98 (13803951.67, 40664366.29)	243.56 (123.23, 363.89)
2045	Both	27532634.31 (13050535.78, 42014732.84)	244.3 (115.55, 373.06)
2022	Female	12439375.16 (12183591.23, 12695159.1)	283.38 (280.21, 286.54)
2023	Female	12619153.89 (12287437.26, 12950870.52)	283.17 (277.57, 288.77)
2024	Female	12799938 (12349952.12, 13249923.89)	282.98 (274.33, 291.63)

2025	Female	12980662.78 (12378045.53, 13583280.03)	282.8 (270.63, 294.97)
2026	Female	13159476.84 (12375540.76, 13943412.91)	282.6 (266.52, 298.69)
2027	Female	13336249.92 (12345385.49, 14327114.36)	282.4 (262.03, 302.77)
2028	Female	13512308.61 (12291175.64, 14733441.58)	282.2 (257.22, 307.19)
2029	Female	13688935.89 (12215389.91, 15162481.87)	282.02 (252.13, 311.92)
2030	Female	13865138.75 (12117828.02, 15612449.47)	281.85 (246.76, 316.94)
2031	Female	14039429.37 (11997449.71, 16081409.04)	281.66 (241.11, 322.21)
2032	Female	14211982.7 (11854357.89, 16569607.5)	281.48 (235.21, 327.75)
2033	Female	14383556.23 (11689581.2, 17077531.27)	281.31 (229.08, 333.54)
2034	Female	14555508.04 (11504375.64, 17606640.43)	281.15 (222.72, 339.57)
2035	Female	14727161.92 (11298130.92, 18156192.93)	280.99 (216.15, 345.83)
2036	Female	14897457.05 (11069764.08, 18725150.01)	280.82 (209.36, 352.29)
2037	Female	15066986.33 (10819234.96, 19314737.7)	280.66 (202.36, 358.96)
2038	Female	15235759 (10546477.66, 19925040.34)	280.51 (195.16, 365.85)
2039	Female	15404965.73 (10252112.64, 20557818.81)	280.35 (187.77, 372.93)
2040	Female	15574465.46 (9935650.97, 21213279.95)	280.19 (180.19, 380.19)
2041	Female	15743975.38 (9596323.52, 21891627.24)	280.02 (172.42, 387.62)
2042	Female	15914662.84 (9233991.93, 22595333.75)	279.86 (164.48, 395.25)
2043	Female	16086126.93 (8847877.8, 23324376.06)	279.7 (156.36, 403.05)
2044	Female	16258905.73 (8437643.81, 24080167.66)	279.54 (148.06, 411.01)
2045	Female	16433182.87 (8002583.16, 24863782.59)	279.36 (139.6, 419.12)
2022	Male	8470751.57 (8280422.91, 8661080.23)	198.74 (196.35, 201.13)
2023	Male	8584645 (8340353.26, 8828936.75)	198.45 (194.25, 202.64)
2024	Male	8698820.02 (8370282.47, 9027357.58)	198.14 (191.69, 204.6)
2025	Male	8812689.04 (8375217.4, 9250160.69)	197.84 (188.78, 206.89)
2026	Male	8926174.17 (8359161.03, 9493187.31)	197.54 (185.59, 209.49)

2027	Male	9038821.81 (8323974.69, 9753668.92)	197.25 (182.15, 212.36)
2028	Male	9150573.48 (8271333.1, 10029813.86)	196.97 (178.47, 215.48)
2029	Male	9262286.09 (8203008.46, 10321563.72)	196.69 (174.57, 218.8)
2030	Male	9373632.57 (8119247.49, 10628017.65)	196.4 (170.47, 222.33)
2031	Male	9484890.02 (8020488.48, 10949291.57)	196.12 (166.19, 226.05)
2032	Male	9596019.79 (7906639.33, 11285400.25)	195.85 (161.73, 229.97)
2033	Male	9706667.22 (7777631.76, 11635702.69)	195.59 (157.11, 234.08)
2034	Male	9817653.08 (7634213.73, 12001092.44)	195.33 (152.32, 238.33)
2035	Male	9928893.5 (7476240.22, 12381546.78)	195.06 (147.38, 242.75)
2036	Male	10040808.57 (7303769.87, 12777847.26)	194.8 (142.29, 247.32)
2037	Male	10153660.2 (7116541.26, 13190779.14)	194.56 (137.06, 252.05)
2038	Male	10266875.97 (6913962.4, 13619789.53)	194.32 (131.7, 256.93)
2039	Male	10381220.8 (6696291.94, 14066149.67)	194.07 (126.2, 261.94)
2040	Male	10496661.81 (6463108.36, 14530215.27)	193.82 (120.57, 267.07)
2041	Male	10613683.85 (6214149.57, 15013218.13)	193.58 (114.82, 272.34)
2042	Male	10732661.47 (5948866.15, 15516456.79)	193.35 (108.94, 277.75)
2043	Male	10852994.62 (5666360.01, 16039629.24)	193.12 (102.95, 283.28)
2044	Male	10975253.25 (5366307.86, 16584198.63)	192.89 (96.84, 288.93)
2045	Male	11099451.44 (5047952.62, 17150950.26)	192.65 (90.62, 294.68)

Notes: ASR: age-standardized rates; YLDs: years lived with disability.

Table S69. Prevalence and YLDs of low back pain in the worldwide of future forecasts using bayesian age-period-cohort model.

Year	Sex	Number	ASR
**Prevalence**			
2022	Both	644268216.44 (631443262.51, 657093170.37)	7428.28 (7280.32, 7576.25)
2023	Both	653688014.04 (637949877.53, 669426150.54)	7409.7 (7231.17, 7588.23)
2024	Both	663296136.06 (642940023.2, 683652248.92)	7392.06 (7165.02, 7619.1)
2025	Both	673014387.79 (646569260.88, 699459514.71)	7375.57 (7085.53, 7665.62)
2026	Both	682670199.35 (648876215.28, 716464183.43)	7359.58 (6994.96, 7724.21)
2027	Both	692129062.41 (649858467.22, 734399657.61)	7343.45 (6894.55, 7792.36)
2028	Both	701520742.67 (649771105.91, 753270379.42)	7327.86 (6786.74, 7868.98)
2029	Both	711028209.27 (648856171.76, 773200246.78)	7313.32 (6673.11, 7953.53)
2030	Both	720580518.01 (647081514.88, 794079521.15)	7299.96 (6554.41, 8045.52)
2031	Both	730052079.64 (644336937.43, 815767221.85)	7287.53 (6430.67, 8144.4)
2032	Both	739322225.88 (640503560.28, 838140891.49)	7275.37 (6301.36, 8249.39)
2033	Both	748495933.92 (635708799.28, 861283068.55)	7264.01 (6167.46, 8360.57)
2034	Both	757729046.31 (630103355.22, 885354737.4)	7253.73 (6029.51, 8477.94)
2035	Both	766965338.51 (623640419.98, 910290257.04)	7244.62 (5887.77, 8601.47)
2036	Both	776124868.85 (616237996.73, 936011740.96)	7236.84 (5742.31, 8731.37)
2037	Both	785093900.54 (607778923.93, 962408877.14)	7229.79 (5592.47, 8867.12)
2038	Both	793999900.49 (598379650.36, 989620150.63)	7223.81 (5438.72, 9008.9)
2039	Both	802954964.82 (588129190.76, 1017780738.88)	7218.99 (5281.25, 9156.73)
2040	Both	811910359.19 (576981887.72, 1046838830.67)	7215.44 (5120.15, 9310.73)
2041	Both	820837064.82 (564892013.87, 1076782115.77)	7213.67 (4955.61, 9471.74)
2042	Both	829629468.15 (551750840.67, 1107508095.63)	7213.17 (4786.95, 9639.38)
2043	Both	838372864.06 (537620515.75, 1139125212.38)	7214.05 (4614.31, 9813.79)

2044	Both	847149794.58 (522549226.8, 1171750362.37)	7216.21 (4437.57, 9994.84)
2045	Both	855933147.51 (506502285.45, 1205364009.56)	7219.76 (4256.71, 10182.81)
2022	Female	406002512.91 (397717807.29, 414287218.52)	9153.11 (9061.97, 9244.24)
2023	Female	412158796.57 (402037149.99, 422280443.14)	9133.39 (8979.72, 9287.05)
2024	Female	418442881.04 (405405542.23, 431480219.84)	9114.76 (8882.71, 9346.82)
2025	Female	424800956.57 (407909370.84, 441692542.31)	9097.15 (8774.87, 9419.44)
2026	Female	431120756.67 (409567109.48, 452674403.85)	9079.61 (8656.97, 9502.25)
2027	Female	437309983.91 (410369831.39, 464250136.42)	9060.99 (8528.72, 9593.25)
2028	Female	443474912.78 (410502984.15, 476446841.42)	9042.55 (8392.6, 9692.5)
2029	Female	449722804.45 (410111667.02, 489333941.88)	9024.86 (8249.95, 9799.76)
2030	Female	455999914.02 (409166976.8, 502832851.24)	9007.78 (8101.21, 9914.34)
2031	Female	462222176 (407594167.06, 516850184.93)	8990.94 (7946.28, 10035.6)
2032	Female	468302925.84 (405308166.04, 531297685.64)	8973.23 (7784.2, 10162.26)
2033	Female	474334032.26 (402412415.66, 546255648.86)	8955.61 (7616.41, 10294.81)
2034	Female	480407018.58 (398995659.77, 561818377.39)	8938.35 (7443.59, 10433.11)
2035	Female	486472751.07 (395016578.92, 577928923.22)	8921.3 (7265.9, 10576.7)
2036	Female	492475644.9 (390418853.89, 594532435.9)	8904.67 (7083.58, 10725.75)
2037	Female	498331762.45 (385118648.8, 611544876.11)	8887.42 (6895.74, 10879.1)
2038	Female	504146390.43 (379209997.07, 629082783.78)	8870.25 (6703.32, 11037.17)
2039	Female	509988385.48 (372747613.69, 647229157.28)	8853.15 (6506.61, 11199.69)
2040	Female	515814246.22 (365693444.78, 665935047.65)	8836 (6305.73, 11366.28)
2041	Female	521603278.71 (358017172.44, 685189384.99)	8819.47 (6101.17, 11537.76)
2042	Female	527276705.34 (349642061.71, 704911348.98)	8802.65 (5892.21, 11713.1)
2043	Female	532913234.5 (340625560.42, 725200908.58)	8785.9 (5679.35, 11892.44)
2044	Female	538561820.18 (330996312.84, 746127327.51)	8768.98 (5462.68, 12075.27)
2045	Female	544195650.41 (320726982.07, 767664318.74)	8751.77 (5242.29, 12261.25)

2022	Male	238265703.53 (233725455.21, 242805951.85)	5628.43 (5574.99, 5681.88)
2023	Male	241529217.47 (235912727.54, 247145707.4)	5611.37 (5519.99, 5702.75)
2024	Male	244853255.03 (237534480.96, 252172029.09)	5594.9 (5456.11, 5733.7)
2025	Male	248213431.22 (238659890.04, 257766972.4)	5579.29 (5386.02, 5772.56)
2026	Male	251549442.68 (239309105.79, 263789779.58)	5564.04 (5310.26, 5817.81)
2027	Male	254819078.5 (239488635.83, 270149521.18)	5548.81 (5229.02, 5868.6)
2028	Male	258045829.88 (239268121.76, 276823538.01)	5533.63 (5143.05, 5924.22)
2029	Male	261305404.82 (238744504.74, 283866304.9)	5518.94 (5053.25, 5984.63)
2030	Male	264580603.99 (237914538.08, 291246669.91)	5504.97 (4960.18, 6049.76)
2031	Male	267829903.64 (236742770.37, 298917036.92)	5491.48 (4863.78, 6119.19)
2032	Male	271019300.04 (235195394.23, 306843205.84)	5478.15 (4763.77, 6192.52)
2033	Male	274161901.66 (233296383.62, 315027419.69)	5464.89 (4660.43, 6269.34)
2034	Male	277322027.73 (231107695.46, 323536360)	5451.96 (4554.22, 6349.7)
2035	Male	280492587.44 (228623841.05, 332361333.82)	5439.6 (4445.51, 6433.69)
2036	Male	283649223.95 (225819142.84, 341479305.06)	5427.78 (4334.32, 6521.24)
2037	Male	286762138.08 (222660275.13, 350864001.04)	5416.17 (4220.34, 6612.01)
2038	Male	289853510.07 (219169653.29, 360537366.84)	5404.64 (4103.67, 6705.62)
2039	Male	292966579.34 (215381577.07, 370551581.6)	5393.29 (3984.55, 6802.03)
2040	Male	296096112.98 (211288442.94, 380903783.02)	5382.32 (3863.26, 6901.39)
2041	Male	299233786.1 (206874841.44, 391592730.77)	5371.97 (3739.96, 7003.99)
2042	Male	302352762.8 (202108778.96, 402596746.65)	5361.95 (3614.37, 7109.53)
2043	Male	305459629.56 (196994955.32, 413924303.8)	5352.01 (3486.47, 7217.56)
2044	Male	308587974.41 (191552913.96, 425623034.86)	5342.1 (3356.33, 7327.86)
2045	Male	311737497.1 (185775303.38, 437699690.83)	5332.39 (3224.17, 7440.61)
**YLDs**			
2022	Both	71816566.66 (70384511.09, 73248622.22)	827.76 (811.24, 844.28)

2023	Both	72772274.28 (71013324, 74531224.55)	824.88 (804.92, 844.83)
2024	Both	73742289.96 (71465995.68, 76018584.24)	822.1 (796.7, 847.5)
2025	Both	74718872.79 (71762258.5, 77675487.07)	819.44 (786.99, 851.9)
2026	Both	75687052.13 (71911431.93, 79462672.32)	816.86 (776.08, 857.65)
2027	Both	76633375.85 (71915232.59, 81351519.12)	814.29 (764.11, 864.47)
2028	Both	77569369.28 (71799676.55, 83339062.01)	811.79 (751.35, 872.23)
2029	Both	78512138.73 (71589096.24, 85435181.21)	809.41 (737.96, 880.86)
2030	Both	79454478.37 (71281050.51, 87627906.22)	807.15 (724.02, 890.28)
2031	Both	80386980.89 (70867856.66, 89906105.12)	805.01 (709.55, 900.47)
2032	Both	81297917.35 (70338130.16, 92257704.54)	802.92 (694.51, 911.33)
2033	Both	82196212.8 (69703882.81, 94688542.79)	800.94 (679, 922.88)
2034	Both	83095950.48 (68979462.54, 97212438.42)	799.07 (663.07, 935.08)
2035	Both	83991264.25 (68160483.61, 99822044.88)	797.33 (646.73, 947.93)
2036	Both	84877939.89 (67242090.16, 102513789.61)	795.75 (630.02, 961.47)
2037	Both	85745133.64 (66212985.43, 105277281.85)	794.27 (612.88, 975.66)
2038	Both	86603669.74 (65083669.49, 108123669.98)	792.93 (595.34, 990.52)
2039	Both	87462887.35 (63862017.92, 111063756.78)	791.71 (577.42, 1006.01)
2040	Both	88317693.12 (62543719.84, 114091666.39)	790.63 (559.12, 1022.14)
2041	Both	89169502.29 (61127468.88, 117211535.71)	789.75 (540.48, 1039.03)
2042	Both	90008699.13 (59602949.11, 120414449.15)	789.04 (521.43, 1056.64)
2043	Both	90841783.41 (57975167.43, 123708399.4)	788.49 (501.99, 1074.99)
2044	Both	91674669.55 (56247687.99, 127101651.1)	788.08 (482.12, 1094.05)
2045	Both	92504287.5 (54417148.52, 130591426.48)	787.82 (461.83, 1113.82)
2022	Female	44916668.54 (44001385.87, 45831951.2)	1014.12 (1003.95, 1024.29)
2023	Female	45529907.53 (44409102.71, 46650712.35)	1010.73 (993.56, 1027.9)
2024	Female	46152514.46 (44706029.32, 47598999.6)	1007.45 (981.52, 1033.38)

2025	Female	46778992.76 (44903456.33, 48654529.19)	1004.27 (968.28, 1040.26)
2026	Female	47400066.41 (45007231.08, 49792901.74)	1001.12 (953.97, 1048.28)
2027	Female	48006582.55 (45017776.77, 50995388.32)	997.9 (938.56, 1057.23)
2028	Female	48608258.31 (44953929.47, 52262587.14)	994.71 (922.33, 1067.09)
2029	Female	49214550.52 (44829793.09, 53599307.94)	991.59 (905.39, 1077.79)
2030	Female	49819857.6 (44642859.6, 54996855.6)	988.52 (887.78, 1089.26)
2031	Female	50418399.72 (44388390.88, 56448408.57)	985.51 (869.55, 1101.47)
2032	Female	51001805.92 (44058309.12, 57945302.72)	982.43 (850.59, 1114.27)
2033	Female	51578250.69 (43662484.72, 59494016.66)	979.39 (831.06, 1127.72)
2034	Female	52155371.27 (43208872.92, 61101869.63)	976.37 (811, 1141.75)
2035	Female	52727925.05 (42693448.83, 62762401.27)	973.36 (790.42, 1156.3)
2036	Female	53293463.85 (42113361.15, 64473566.54)	970.42 (769.4, 1171.44)
2037	Female	53844007.53 (41460613.23, 66227401.83)	967.45 (747.84, 1187.06)
2038	Female	54388771.19 (40743816.81, 68033725.56)	964.51 (725.83, 1203.18)
2039	Female	54932907.03 (39967408.32, 69898405.75)	961.57 (703.39, 1219.74)
2040	Female	55471765.53 (39127712.62, 71815818.45)	958.6 (680.53, 1236.68)
2041	Female	56006796.29 (38224418.17, 73789174.4)	955.73 (657.32, 1254.14)
2042	Female	56530790.29 (37250323.4, 75811257.19)	952.85 (633.7, 1272.01)
2043	Female	57050173.58 (36210318.65, 77890028.5)	950.01 (609.71, 1290.3)
2044	Female	57567784.41 (35106149.31, 80029419.5)	947.14 (585.36, 1308.93)
2045	Female	58080588.16 (33935176.92, 82225999.39)	944.23 (560.64, 1327.82)
2022	Male	26899898.12 (26383125.22, 27416671.02)	633.88 (627.79, 639.96)
2023	Male	27242366.75 (26604221.29, 27880512.2)	631.56 (621.2, 641.92)
2024	Male	27589775.5 (26759966.37, 28419584.64)	629.31 (613.61, 645.01)
2025	Male	27939880.02 (26858802.17, 29020957.88)	627.16 (605.33, 648.99)
2026	Male	28286985.72 (26904200.85, 29669770.59)	625.06 (596.43, 653.69)

2027	Male	28626793.31 (26897455.81, 30356130.8)	622.97 (586.93, 659.02)
2028	Male	28961110.97 (26845747.08, 31076474.87)	620.9 (576.91, 664.89)
2029	Male	29297588.21 (26759303.15, 31835873.27)	618.89 (566.48, 671.29)
2030	Male	29634620.76 (26638190.91, 32631050.62)	616.95 (555.7, 678.21)
2031	Male	29968581.17 (26479465.78, 33457696.55)	615.08 (544.56, 685.61)
2032	Male	30296111.43 (26279821.03, 34312401.82)	613.24 (533.03, 693.45)
2033	Male	30617962.11 (26041398.09, 35194526.13)	611.41 (521.15, 701.68)
2034	Male	30940579.21 (25770589.62, 36110568.79)	609.63 (508.96, 710.3)
2035	Male	31263339.19 (25467034.78, 37059643.61)	607.9 (496.5, 719.31)
2036	Male	31584476.04 (25128729.01, 38040223.08)	606.25 (483.79, 728.71)
2037	Male	31901126.11 (24752372.2, 39049880.01)	604.63 (470.78, 738.48)
2038	Male	32214898.55 (24339852.68, 40089944.42)	603.03 (457.49, 748.56)
2039	Male	32529980.32 (23894609.61, 41165351.03)	601.45 (443.95, 758.95)
2040	Male	32845927.58 (23416007.22, 42275847.95)	599.92 (430.18, 769.66)
2041	Male	33162706 (22903050.7, 43422361.3)	598.45 (416.19, 780.71)
2042	Male	33477908.84 (22352625.71, 44603191.96)	597.04 (401.98, 792.1)
2043	Male	33791609.84 (21764848.77, 45818370.9)	595.64 (387.52, 803.76)
2044	Male	34106885.14 (21141538.67, 47072231.6)	594.25 (372.83, 815.67)
2045	Male	34423699.35 (20481971.6, 48365427.09)	592.89 (357.93, 827.85)

Notes: ASR: age-standardized rates; YLDs: years lived with disability.

Table S70. Prevalence and YLDs of hip osteoarthritis in the worldwide of future forecasts using bayesian age-period-cohort model.

Year	Sex	Number	ASR
**Prevalence**			
2022	Both	37163988.5 (36380868.77, 37947108.23)	415.94 (407.16, 424.71)
2023	Both	37960219.53 (36908869.08, 39011569.99)	414.33 (402.84, 425.82)
2024	Both	38781973.55 (37324657.24, 40239289.86)	412.69 (397.16, 428.21)
2025	Both	39609391.4 (37632870.39, 41585912.41)	410.99 (390.46, 431.52)
2026	Both	40444629.6 (37849017.93, 43040241.28)	409.42 (383.11, 435.73)
2027	Both	41283660.19 (37973981.79, 44593338.58)	408.03 (375.27, 440.79)
2028	Both	42136819.8 (38024361.49, 46249278.12)	406.73 (366.97, 446.49)
2029	Both	43010252.89 (38008366.69, 48012139.09)	405.43 (358.19, 452.66)
2030	Both	43886134.16 (37910594.05, 49861674.28)	404.1 (348.97, 459.23)
2031	Both	44769671.08 (37734001.6, 51805340.56)	402.9 (339.45, 466.36)
2032	Both	45656241.51 (37470521.33, 53841961.69)	401.88 (329.65, 474.11)
2033	Both	46555420.45 (37128619.58, 55982221.32)	400.97 (319.56, 482.38)
2034	Both	47473342.8 (36711914.74, 58234770.86)	400.1 (309.14, 491.06)
2035	Both	48393042.02 (36205092.05, 60580992)	399.27 (298.39, 500.14)
2036	Both	49319128.93 (35608577.56, 63029680.3)	398.58 (287.39, 509.76)
2037	Both	50245696.63 (34912633.37, 65578759.89)	398.06 (276.14, 519.99)
2038	Both	51185946.37 (34125088.6, 68246804.14)	397.7 (264.61, 530.78)
2039	Both	52147668.93 (33247875.94, 71047461.92)	397.44 (252.78, 542.1)
2040	Both	53114238.19 (32266400.83, 73962075.55)	397.26 (240.61, 553.91)
2041	Both	54088826.51 (31178268.56, 76999384.45)	397.25 (228.15, 566.35)
2042	Both	55065121.35 (29972948.24, 80157294.45)	397.4 (215.35, 579.45)
2043	Both	56059345.93 (28655762.3, 83462929.56)	397.74 (202.21, 593.27)

2044	Both	57080349.07 (27225368.78, 86935329.37)	398.25 (188.69, 607.81)
2045	Both	58112501.24 (25667696.51, 90557305.96)	398.89 (174.75, 623.04)
2022	Female	19542083.86 (19077380.32, 20006787.39)	411.57 (406.17, 416.97)
2023	Female	19922285.23 (19327310.11, 20517260.36)	409.04 (399.98, 418.1)
2024	Female	20317488.82 (19522002.72, 21112974.92)	406.52 (392.91, 420.13)
2025	Female	20716566.01 (19661296.09, 21771835.92)	403.97 (385.16, 422.79)
2026	Female	21116712.65 (19749770.62, 22483654.69)	401.48 (376.91, 426.04)
2027	Female	21517118.34 (19790005.33, 23244231.35)	399.11 (368.3, 429.93)
2028	Female	21925355.97 (19793420.11, 24057291.83)	396.83 (359.34, 434.32)
2029	Female	22345386.89 (19765425.75, 24925348.04)	394.55 (350.02, 439.08)
2030	Female	22767214.59 (19697513.73, 25836915.45)	392.24 (340.36, 444.13)
2031	Female	23189301.67 (19587646.84, 26790956.51)	389.97 (330.42, 449.53)
2032	Female	23610498.2 (19433164.7, 27787831.7)	387.79 (320.24, 455.33)
2033	Female	24038020.62 (19240773.45, 28835267.79)	385.67 (309.86, 461.48)
2034	Female	24475765.47 (19013203.03, 29938327.9)	383.58 (299.26, 467.9)
2035	Female	24913942.24 (18741811.63, 31086072.85)	381.47 (288.42, 474.52)
2036	Female	25350442.08 (18423591.65, 32277292.51)	379.39 (277.4, 481.39)
2037	Female	25783417.09 (18054949.52, 33511884.65)	377.36 (266.19, 488.54)
2038	Female	26222249.54 (17642308.05, 34802191.03)	375.41 (254.85, 495.98)
2039	Female	26671872.73 (17187673.97, 36156071.49)	373.51 (243.36, 503.66)
2040	Female	27122511.94 (16682919.98, 37562103.9)	371.61 (231.7, 511.52)
2041	Female	27571189.61 (16124317.91, 39018061.31)	369.72 (219.89, 519.54)
2042	Female	28015722.66 (15507855.6, 40523589.71)	367.83 (207.92, 527.74)
2043	Female	28467288.64 (14838672.97, 42095904.31)	366.02 (195.83, 536.2)
2044	Female	28931593.99 (14117294.3, 43745893.69)	364.27 (183.64, 544.89)
2045	Female	29399440.55 (13336276.92, 45462604.18)	362.53 (171.33, 553.74)

2022	Male	17621904.64 (17303488.45, 17940320.84)	420.26 (415.52, 425)
2023	Male	18037934.3 (17581558.97, 18494309.63)	419.7 (410.95, 428.45)
2024	Male	18464484.73 (17802654.51, 19126314.94)	419.01 (405.3, 432.71)
2025	Male	18892825.4 (17971574.3, 19814076.49)	418.19 (398.8, 437.57)
2026	Male	19327916.95 (18099247.31, 20556586.59)	417.49 (391.79, 443.2)
2027	Male	19766541.85 (18183976.46, 21349107.24)	416.98 (384.35, 449.61)
2028	Male	20211463.83 (18230941.38, 22191986.28)	416.47 (376.38, 456.56)
2029	Male	20664866 (18242940.95, 23086791.05)	415.83 (367.82, 463.84)
2030	Male	21118919.58 (18213080.32, 24024758.83)	415.07 (358.73, 471.41)
2031	Male	21580369.41 (18146354.77, 25014384.05)	414.43 (349.33, 479.53)
2032	Male	22045743.31 (18037356.63, 26054129.99)	413.93 (339.63, 488.22)
2033	Male	22517399.82 (17887846.12, 27146953.53)	413.43 (329.54, 497.31)
2034	Male	22997577.33 (17698711.71, 28296442.96)	412.83 (319.02, 506.64)
2035	Male	23479099.78 (17463280.42, 29494919.15)	412.13 (308.09, 516.17)
2036	Male	23968686.85 (17184985.91, 30752387.79)	411.54 (296.92, 526.16)
2037	Male	24462279.55 (16857683.85, 32066875.24)	411.05 (285.49, 536.61)
2038	Male	24963696.83 (16482780.55, 33444613.12)	410.58 (273.75, 547.41)
2039	Male	25475796.2 (16060201.97, 34891390.43)	410.04 (261.67, 558.41)
2040	Male	25991726.25 (15583480.86, 36399971.65)	409.43 (249.26, 569.6)
2041	Male	26517636.9 (15053950.66, 37981323.14)	408.91 (236.63, 581.18)
2042	Male	27049398.69 (14465092.64, 39633704.74)	408.45 (223.75, 593.15)
2043	Male	27592057.29 (13817089.33, 41367025.24)	408.02 (210.61, 605.43)
2044	Male	28148755.08 (13108074.48, 43189435.68)	407.55 (197.19, 617.92)
2045	Male	28713060.69 (12331419.59, 45094701.78)	407.04 (183.49, 630.59)
**YLDs**			
2022	Both	1176737.3 (1149067.68, 1204406.92)	13.16 (12.85, 13.47)

2023	Both	1199954.7 (1163379.36, 1236530.04)	13.09 (12.69, 13.49)
2024	Both	1223796.13 (1173757.08, 1273835.18)	13.02 (12.48, 13.55)
2025	Both	1247659.29 (1180404.17, 1314914.42)	12.95 (12.25, 13.64)
2026	Both	1271749.15 (1183975.54, 1359522.76)	12.88 (11.99, 13.77)
2027	Both	1295918.45 (1184509.72, 1407327.17)	12.82 (11.71, 13.92)
2028	Both	1320438.4 (1182524.8, 1458351.99)	12.76 (11.42, 14.1)
2029	Both	1345438.88 (1178250.45, 1512627.31)	12.7 (11.12, 14.28)
2030	Both	1370376.86 (1171235.83, 1569517.88)	12.64 (10.8, 14.48)
2031	Both	1395532.3 (1161671.99, 1629392.6)	12.59 (10.47, 14.7)
2032	Both	1420752.47 (1149301.67, 1692203.27)	12.54 (10.14, 14.94)
2033	Both	1446297.68 (1134374.34, 1758221.01)	12.5 (9.79, 15.2)
2034	Both	1472303.02 (1116969.79, 1827636.24)	12.45 (9.44, 15.47)
2035	Both	1498258.31 (1096636.78, 1899879.84)	12.41 (9.07, 15.75)
2036	Both	1524404.03 (1073450.9, 1975357.17)	12.38 (8.7, 16.05)
2037	Both	1550564.73 (1047112.99, 2054016.48)	12.35 (8.32, 16.37)
2038	Both	1577111.99 (1017837.79, 2136386.2)	12.32 (7.93, 16.71)
2039	Both	1604233.29 (985646.59, 2222819.99)	12.3 (7.53, 17.07)
2040	Both	1631436.96 (950107.83, 2312766.1)	12.28 (7.12, 17.44)
2041	Both	1658896.09 (911173.66, 2406618.52)	12.27 (6.71, 17.83)
2042	Both	1686435.48 (868521.9, 2504349.06)	12.26 (6.28, 18.24)
2043	Both	1714513.94 (822269.77, 2606758.11)	12.26 (5.84, 18.68)
2044	Both	1743355.15 (772328.98, 2714381.31)	12.26 (5.39, 19.14)
2045	Both	1772507.78 (718273.78, 2826741.79)	12.27 (4.92, 19.62)
2022	Female	613895.15 (599064.31, 628726)	12.95 (12.77, 13.12)
2023	Female	624851.41 (605651.37, 644051.45)	12.85 (12.55, 13.14)
2024	Female	636135.86 (610290.53, 661981.19)	12.75 (12.31, 13.2)

2025	Female	647415.83 (613039.03, 681792.64)	12.65 (12.04, 13.27)
2026	Female	658769.79 (614211.76, 703327.82)	12.56 (11.75, 13.36)
2027	Female	670159.49 (613875.51, 726443.46)	12.47 (11.46, 13.47)
2028	Female	681736.4 (612320.8, 751152)	12.38 (11.15, 13.6)
2029	Female	693547.31 (609657.16, 777437.47)	12.29 (10.83, 13.74)
2030	Female	705292.48 (605640.78, 804944.18)	12.2 (10.51, 13.89)
2031	Female	717081.27 (600347.34, 833815.21)	12.11 (10.17, 14.05)
2032	Female	728871.02 (593683.53, 864058.51)	12.02 (9.83, 14.22)
2033	Female	740812.04 (585802.47, 895821.61)	11.94 (9.48, 14.4)
2034	Female	752952.03 (576737.79, 929166.26)	11.86 (9.13, 14.59)
2035	Female	765000.26 (566243.16, 963757.36)	11.78 (8.76, 14.79)
2036	Female	777033.16 (554334.8, 999731.52)	11.69 (8.4, 14.99)
2037	Female	788994.92 (540897.83, 1037092.01)	11.61 (8.03, 15.2)
2038	Female	801109.07 (526082.66, 1076135.49)	11.54 (7.65, 15.42)
2039	Female	813458.71 (509907.32, 1117010.1)	11.46 (7.27, 15.65)
2040	Female	825756.68 (492141.1, 1159372.25)	11.38 (6.89, 15.88)
2041	Female	838029.69 (472743.5, 1203315.87)	11.31 (6.5, 16.11)
2042	Female	850218.6 (451590.52, 1248846.69)	11.23 (6.11, 16.35)
2043	Female	862607.18 (428796.92, 1296417.44)	11.16 (5.72, 16.6)
2044	Female	875308.72 (404342.45, 1346274.99)	11.09 (5.32, 16.86)
2045	Female	888057.01 (378014.2, 1398099.81)	11.02 (4.93, 17.11)
2022	Male	562842.15 (550003.38, 575680.92)	13.37 (13.19, 13.55)
2023	Male	575103.29 (557727.99, 592478.59)	13.33 (13.02, 13.65)
2024	Male	587660.27 (563466.54, 611853.99)	13.29 (12.81, 13.78)
2025	Male	600243.46 (567365.13, 633121.78)	13.25 (12.57, 13.93)
2026	Male	612979.36 (569763.78, 656194.94)	13.21 (12.32, 14.1)

2027	Male	625758.96 (570634.21, 680883.71)	13.17 (12.05, 14.3)
2028	Male	638702 (570204.01, 707199.99)	13.14 (11.76, 14.51)
2029	Male	651891.57 (568593.29, 735189.85)	13.1 (11.46, 14.74)
2030	Male	665084.37 (565595.06, 764573.69)	13.06 (11.14, 14.98)
2031	Male	678451.02 (561324.65, 795577.4)	13.02 (10.81, 15.23)
2032	Male	691881.45 (555618.13, 828144.77)	12.98 (10.46, 15.5)
2033	Male	705485.64 (548571.88, 862399.4)	12.95 (10.11, 15.79)
2034	Male	719350.99 (540232, 898469.99)	12.91 (9.75, 16.08)
2035	Male	733258.05 (530393.62, 936122.47)	12.87 (9.37, 16.38)
2036	Male	747370.88 (519116.1, 975625.65)	12.84 (8.98, 16.69)
2037	Male	761569.81 (506215.15, 1016924.47)	12.8 (8.59, 17.02)
2038	Male	776002.92 (491755.13, 1060250.71)	12.77 (8.19, 17.35)
2039	Male	790774.58 (475739.27, 1105809.89)	12.74 (7.78, 17.69)
2040	Male	805680.29 (457966.72, 1153393.85)	12.7 (7.35, 18.04)
2041	Male	820866.4 (438430.16, 1203302.65)	12.66 (6.93, 18.4)
2042	Male	836216.88 (416931.38, 1255502.38)	12.63 (6.49, 18.77)
2043	Male	851906.76 (393472.86, 1310340.67)	12.6 (6.05, 19.15)
2044	Male	868046.43 (367986.54, 1368106.32)	12.57 (5.6, 19.54)
2045	Male	884450.78 (340259.58, 1428641.97)	12.53 (5.14, 19.93)

Notes: ASR: age-standardized rates; YLDs: years lived with disability.

Table S71. Prevalence and YLDs of knee osteoarthritis in the worldwide of future forecasts using bayesian age-period-cohort model.

Year	Sex	Number	ASR
**Prevalence**			
2022	Both	389576834.14 (376608542.72, 402545125.56)	4316.58 (4172.86, 4460.3)
2023	Both	400266681.92 (385012700.5, 415520663.34)	4330.69 (4165.61, 4495.76)
2024	Both	411224870.65 (392410296.24, 430039445.06)	4344.59 (4145.77, 4543.41)
2025	Both	422366854.54 (398754601.66, 445979107.43)	4358.26 (4114.56, 4601.96)
2026	Both	433615066.9 (404056180.67, 463173953.13)	4371.88 (4073.8, 4669.97)
2027	Both	444900683.29 (408321886.99, 481479479.59)	4385.81 (4025.14, 4746.47)
2028	Both	456285269.69 (411684545.02, 500885994.36)	4399.6 (3969.45, 4829.75)
2029	Both	467871687.06 (414270935.86, 521472438.26)	4413.12 (3907.41, 4918.84)
2030	Both	479583227.59 (416015376.42, 543151078.76)	4426.48 (3839.58, 5013.38)
2031	Both	491352567.43 (416842557.35, 565862577.51)	4439.89 (3766.38, 5113.4)
2032	Both	503108827.23 (416669864.24, 589547790.21)	4453.65 (3688.17, 5219.13)
2033	Both	514885422.57 (415531994.54, 614238850.6)	4467.15 (3604.76, 5329.54)
2034	Both	526803969.09 (413516691.92, 640091246.26)	4480.41 (3516.4, 5444.42)
2035	Both	538807501.7 (410550270.88, 667064732.51)	4493.65 (3423.32, 5563.97)
2036	Both	550827962.1 (406541375.72, 695114548.47)	4507.11 (3325.65, 5688.57)
2037	Both	562788975.65 (401393442.55, 724184508.75)	4521 (3223.41, 5818.59)
2038	Both	574706100.66 (395109577.1, 754302624.22)	4534.63 (3116.22, 5953.03)
2039	Both	586722408.73 (387767067.49, 785677749.97)	4548.16 (3004.25, 6092.07)
2040	Both	598806730.17 (379304705.98, 818308754.35)	4561.97 (2887.68, 6236.25)
2041	Both	610903818.24 (369635505.75, 852172130.74)	4576.32 (2766.49, 6386.14)
2042	Both	622944694 (358663993.37, 887225394.64)	4591.38 (2640.53, 6542.24)
2043	Both	634920209.64 (346362333.34, 923478085.93)	4606.45 (2509.33, 6703.57)

2044	Both	646977349.94 (332776755.96, 961177943.93)	4621.8 (2372.98, 6870.62)
2045	Both	659114288.27 (317850211.47, 1000378365.07)	4637.85 (2231.48, 7044.22)
2022	Female	239096547.5 (230741098.58, 247451996.42)	5055.12 (4966.94, 5143.3)
2023	Female	245562650.38 (235750845.99, 255374454.78)	5068.25 (4936.83, 5199.66)
2024	Female	252183744.94 (240105034.98, 264262454.89)	5080.87 (4893.31, 5268.44)
2025	Female	258913022.24 (243779648.6, 274046395.89)	5092.82 (4839.4, 5346.25)
2026	Female	265709772.3 (246789218.13, 284630326.47)	5104.27 (4776.81, 5431.73)
2027	Female	272535367.37 (249143028.48, 295927706.27)	5115.55 (4706.69, 5524.41)
2028	Female	279418869.13 (250917227.55, 307920510.7)	5126.07 (4629.38, 5622.76)
2029	Female	286417365.52 (252185269.84, 320649461.2)	5135.65 (4545.29, 5726)
2030	Female	293488000.74 (252911167.67, 334064833.81)	5144.28 (4454.8, 5833.77)
2031	Female	300595871.31 (253053874.96, 348137867.67)	5152.23 (4358.31, 5946.14)
2032	Female	307699369.58 (252563550.82, 362835188.34)	5159.76 (4256.18, 6063.33)
2033	Female	314808881.9 (251453741.92, 378164021.89)	5166.05 (4148.13, 6183.96)
2034	Female	321993009.22 (249772971.49, 394213046.94)	5171.09 (4034.51, 6307.68)
2035	Female	329219167.76 (247477811.23, 410960524.29)	5175.07 (3915.66, 6434.49)
2036	Female	336451556.74 (244515898.6, 428387214.88)	5178.24 (3791.89, 6564.59)
2037	Female	343644819.03 (240827961.29, 446461676.77)	5180.77 (3663.36, 6698.19)
2038	Female	350797649.99 (236408380.4, 465186919.57)	5181.72 (3529.72, 6833.72)
2039	Female	357993991.11 (231300974.52, 484687007.7)	5181.27 (3391.4, 6971.15)
2040	Female	365217814.62 (225469841.67, 504965787.58)	5179.79 (3248.8, 7110.77)
2041	Female	372441743.08 (218864534.48, 526018951.68)	5177.54 (3102.2, 7252.88)
2042	Female	379625557.01 (211426099.97, 547825014.05)	5174.62 (2951.66, 7397.59)
2043	Female	386751461.2 (203130164.45, 570372757.94)	5170.11 (2796.92, 7543.31)
2044	Female	393907188.29 (194001752.9, 593812623.68)	5164.29 (2638.39, 7690.2)
2045	Female	401097069.3 (184007315.92, 618186822.67)	5157.56 (2476.44, 7838.68)

2022	Male	150480286.64 (145867444.14, 155093129.14)	3503.73 (3449.92, 3557.54)
2023	Male	154704031.54 (149261854.51, 160146208.57)	3518.55 (3437.7, 3599.4)
2024	Male	159041125.72 (152305261.26, 165776990.17)	3533.24 (3417.2, 3649.27)
2025	Male	163453832.3 (154974953.06, 171932711.54)	3547.7 (3390.27, 3705.14)
2026	Male	167905294.6 (157266962.54, 178543626.65)	3561.97 (3357.87, 3766.07)
2027	Male	172365315.91 (159178858.51, 185551773.32)	3576.2 (3320.64, 3831.76)
2028	Male	176866400.56 (160767317.46, 192965483.66)	3590.12 (3278.84, 3901.39)
2029	Male	181454321.54 (162085666.03, 200822977.06)	3603.63 (3232.71, 3974.54)
2030	Male	186095226.85 (163104208.75, 209086244.94)	3616.75 (3182.49, 4051.02)
2031	Male	190756696.12 (163788682.39, 217724709.84)	3629.52 (3128.29, 4130.76)
2032	Male	195409457.64 (164106313.42, 226712601.87)	3642.07 (3070.29, 4213.86)
2033	Male	200076540.66 (164078252.62, 236074828.7)	3653.98 (3008.35, 4299.61)
2034	Male	204810959.87 (163743720.43, 245878199.32)	3665.25 (2942.67, 4387.83)
2035	Male	209588333.93 (163072459.65, 256104208.22)	3676 (2873.43, 4478.57)
2036	Male	214376405.35 (162025477.11, 266727333.59)	3686.27 (2800.73, 4571.82)
2037	Male	219144156.62 (160565481.26, 277722831.99)	3696.15 (2724.64, 4667.67)
2038	Male	223908450.68 (158701196.7, 289115704.66)	3705.13 (2644.94, 4765.31)
2039	Male	228728417.63 (156466092.98, 300990742.28)	3713.3 (2561.87, 4864.74)
2040	Male	233588915.54 (153834864.31, 313342966.77)	3720.9 (2475.66, 4966.13)
2041	Male	238462075.17 (150770971.27, 326153179.06)	3727.98 (2386.41, 5069.56)
2042	Male	243319136.99 (147237893.39, 339400380.59)	3734.6 (2294.14, 5175.06)
2043	Male	248168748.44 (143232168.89, 353105327.99)	3740.22 (2198.67, 5281.77)
2044	Male	253070161.65 (138775003.06, 367365320.25)	3745.02 (2100.24, 5389.8)
2045	Male	258017218.98 (133842895.55, 382191542.4)	3749.25 (1999.07, 5499.43)
**YLDs**			
2022	Both	12491004.61 (12082002.56, 12900006.67)	138.3 (133.77, 142.83)

2023	Both	12818907.77 (12338181.37, 13299634.16)	138.62 (133.42, 143.82)
2024	Both	13153901.5 (12561708.65, 13746094.34)	138.94 (132.68, 145.2)
2025	Both	13493592.18 (12751530.51, 14235653.85)	139.24 (131.58, 146.9)
2026	Both	13835714.73 (12908232.87, 14763196.58)	139.55 (130.19, 148.9)
2027	Both	14178246.84 (13032236.34, 15324257.34)	139.86 (128.55, 151.17)
2028	Both	14522560.45 (13127360, 15917760.91)	140.16 (126.69, 153.63)
2029	Both	14871780.9 (13197642.5, 16545919.31)	140.45 (124.64, 156.27)
2030	Both	15223811.53 (13241421.43, 17206201.62)	140.74 (122.4, 159.07)
2031	Both	15576733.03 (13256600.28, 17896865.78)	141.02 (120.01, 162.04)
2032	Both	15928493.31 (13240823.3, 18616163.32)	141.32 (117.46, 165.18)
2033	Both	16279594 (13194877.59, 19364310.42)	141.61 (114.76, 168.46)
2034	Both	16633789.29 (13121650.84, 20145927.73)	141.88 (111.9, 171.86)
2035	Both	16989635.04 (13019251.02, 20960019.06)	142.16 (108.91, 175.41)
2036	Both	17345210.48 (12885095.78, 21805325.18)	142.45 (105.78, 179.11)
2037	Both	17698319.81 (12716409.67, 22680229.96)	142.75 (102.52, 182.97)
2038	Both	18048910.35 (12513061.95, 23584758.74)	143.03 (99.11, 186.96)
2039	Both	18401383.37 (12277629.77, 24525136.97)	143.32 (95.56, 191.09)
2040	Both	18755101.41 (12008597.79, 25501605.04)	143.62 (91.88, 195.36)
2041	Both	19108557.1 (11703536.14, 26513578.06)	143.94 (88.06, 199.81)
2042	Both	19459824.13 (11359731.77, 27559916.49)	144.28 (84.1, 204.45)
2043	Both	19808101.82 (10976175.64, 28640028)	144.62 (80, 209.24)
2044	Both	20157855.18 (10554490.67, 29761219.7)	144.97 (75.74, 214.2)
2045	Both	20509340.94 (10093281.99, 30925399.88)	145.35 (71.33, 219.36)
2022	Female	7627756.75 (7365289.6, 7890223.91)	161.44 (158.66, 164.21)
2023	Female	7824631.75 (7517210.4, 8132053.1)	161.7 (157.58, 165.82)
2024	Female	8025496.99 (7648228.36, 8402765.63)	161.94 (156.08, 167.8)

2025	Female	8229051.58 (7757780.62, 8700322.54)	162.15 (154.26, 170.05)
2026	Female	8434109.35 (7846425.24, 9021793.47)	162.35 (152.17, 172.53)
2027	Female	8639586.49 (7914609.41, 9364563.56)	162.54 (149.85, 175.24)
2028	Female	8846013.27 (7964469.57, 9727556.96)	162.71 (147.32, 178.1)
2029	Female	9055101.27 (7998341.26, 10111861.28)	162.84 (144.57, 181.12)
2030	Female	9265697.61 (8015353.64, 10516041.58)	162.95 (141.64, 184.26)
2031	Female	9476799.32 (8014377.75, 10939220.89)	163.03 (138.52, 187.54)
2032	Female	9687261.42 (7994065.52, 11380457.31)	163.1 (135.24, 190.96)
2033	Female	9897036.81 (7954637.15, 11839436.48)	163.12 (131.78, 194.47)
2034	Female	10108228.48 (7897713.84, 12318743.13)	163.11 (128.15, 198.08)
2035	Female	10320017.52 (7822205.35, 12817829.69)	163.07 (124.37, 201.77)
2036	Female	10531371.38 (7726638.72, 13336104.04)	163 (120.45, 205.55)
2037	Female	10741048.96 (7609383.77, 13872714.15)	162.92 (116.39, 209.44)
2038	Female	10948646.1 (7470128.3, 14427163.91)	162.78 (112.18, 213.38)
2039	Female	11156736.95 (7310374.95, 15003098.95)	162.61 (107.84, 217.37)
2040	Female	11365012.98 (7129270.64, 15600755.33)	162.4 (103.38, 221.43)
2041	Female	11572697.55 (6925421.72, 16219973.38)	162.18 (98.8, 225.55)
2042	Female	11778731.81 (6697232.58, 16860231.05)	161.94 (94.11, 229.76)
2043	Female	11982217.58 (6443885.16, 17520550.01)	161.65 (89.31, 233.99)
2044	Female	12185817.79 (6166334.32, 18205301.26)	161.32 (84.39, 238.25)
2045	Female	12389833.88 (5863814.38, 18915853.37)	160.97 (79.38, 242.56)
2022	Male	4863247.86 (4716712.96, 5009782.77)	112.92 (111.2, 114.64)
2023	Male	4994276.01 (4820970.97, 5167581.06)	113.3 (110.71, 115.9)
2024	Male	5128404.5 (4913480.3, 5343328.71)	113.68 (109.96, 117.4)
2025	Male	5264540.6 (4993749.9, 5535331.31)	114.05 (109, 119.09)
2026	Male	5401605.37 (5061807.63, 5741403.12)	114.41 (107.88, 120.94)

2027	Male	5538660.35 (5117626.92, 5959693.79)	114.77 (106.6, 122.94)
2028	Male	5676547.19 (5162890.43, 6190203.94)	115.12 (105.18, 125.05)
2029	Male	5816679.63 (5199301.23, 6434058.03)	115.45 (103.62, 127.27)
2030	Male	5958113.91 (5226067.79, 6690160.04)	115.77 (101.93, 129.6)
2031	Male	6099933.71 (5242222.53, 6957644.89)	116.07 (100.13, 132.02)
2032	Male	6241231.89 (5246757.77, 7235706.01)	116.37 (98.2, 134.55)
2033	Male	6382557.19 (5240240.44, 7524873.94)	116.65 (96.15, 137.16)
2034	Male	6525560.8 (5223937, 7827184.6)	116.91 (93.99, 139.84)
2035	Male	6669617.52 (5197045.67, 8142189.37)	117.16 (91.71, 142.6)
2036	Male	6813839.1 (5158457.05, 8469221.14)	117.39 (89.34, 145.43)
2037	Male	6957270.86 (5107025.91, 8807515.8)	117.61 (86.86, 148.35)
2038	Male	7100264.25 (5042933.65, 9157594.84)	117.8 (84.27, 151.32)
2039	Male	7244646.42 (4967254.82, 9522038.02)	117.96 (81.58, 154.35)
2040	Male	7390088.43 (4879327.15, 9900849.71)	118.12 (78.8, 157.43)
2041	Male	7535859.55 (4778114.41, 10293604.68)	118.26 (75.93, 160.59)
2042	Male	7681092.32 (4662499.2, 10699685.44)	118.39 (72.97, 163.8)
2043	Male	7825884.23 (4532290.48, 11119477.99)	118.49 (69.91, 167.06)
2044	Male	7972037.39 (4388156.34, 11555918.44)	118.56 (66.77, 170.36)
2045	Male	8119507.06 (4229467.61, 12009546.51)	118.63 (63.54, 173.71)

Notes: ASR: age-standardized rates; YLDs: years lived with disability.

Table S72. Prevalence and YLDs of rheumatoid arthritis in the worldwide of future forecasts using bayesian age-period-cohort model.

Year	Sex	Number	ASR
**Prevalence**			
2022	Both	18538938.25 (18204594.86, 18873281.64)	209.42 (205.64, 213.2)
2023	Both	18950981.14 (18526372.19, 19375590.1)	209.87 (205.17, 214.58)
2024	Both	19369443.79 (18803204.15, 19935683.42)	210.32 (204.16, 216.47)
2025	Both	19791759.8 (19039826.94, 20543692.65)	210.75 (202.74, 218.76)
2026	Both	20216676.82 (19240517.92, 21192835.73)	211.18 (200.98, 221.38)
2027	Both	20642720.33 (19406655.4, 21878785.27)	211.65 (198.97, 224.33)
2028	Both	21071491.14 (19542656.01, 22600326.26)	212.13 (196.74, 227.53)
2029	Both	21505196.1 (19651920.85, 23358471.35)	212.61 (194.28, 230.94)
2030	Both	21940959.48 (19732260.09, 24149658.87)	213.08 (191.63, 234.54)
2031	Both	22377749.4 (19782416.72, 24973082.07)	213.57 (188.79, 238.35)
2032	Both	22814515.97 (19800575.47, 25828456.46)	214.1 (185.8, 242.39)
2033	Both	23251462.21 (19787388.18, 26715536.23)	214.64 (182.65, 246.64)
2034	Both	23690957.67 (19744904.97, 27637010.37)	215.2 (179.34, 251.06)
2035	Both	24131043.25 (19671132.99, 28590953.52)	215.76 (175.86, 255.65)
2036	Both	24571355.1 (19564956.9, 29577753.3)	216.33 (172.23, 260.44)
2037	Both	25012188.92 (19425320.22, 30599057.61)	216.96 (168.47, 265.45)
2038	Both	25453007.93 (19251761.15, 31654254.72)	217.63 (164.57, 270.68)
2039	Both	25895781.39 (19045459.23, 32746103.55)	218.3 (160.51, 276.1)
2040	Both	26339029.5 (18804699.12, 33873359.88)	219 (156.3, 281.7)
2041	Both	26782944.6 (18528578.84, 35037310.36)	219.72 (151.94, 287.5)
2042	Both	27229212.86 (18216696.2, 36241729.51)	220.5 (147.44, 293.56)
2043	Both	27676857.72 (17867901.78, 37485813.66)	221.33 (142.8, 299.86)

2044	Both	28127155.77 (17482329.46, 38771982.08)	222.19 (137.99, 306.39)
2045	Both	28578515.93 (17058031.38, 40099000.48)	223.07 (133.02, 313.12)
2022	Female	13407221.76 (13165744.05, 13648699.47)	294 (291.22, 296.77)
2023	Female	13701455.09 (13394884.09, 14008026.09)	294.58 (289.79, 299.37)
2024	Female	14000505.05 (13591656.65, 14409353.46)	295.18 (287.85, 302.5)
2025	Female	14302398.55 (13759403.54, 14845393.56)	295.76 (285.5, 306.03)
2026	Female	14605319.33 (13900413.59, 15310225.06)	296.34 (282.79, 309.89)
2027	Female	14908459.49 (14016025.31, 15800893.67)	296.93 (279.77, 314.1)
2028	Female	15213812 (14110189.54, 16317434.47)	297.54 (276.47, 318.62)
2029	Female	15523055.65 (14185425.1, 16860686.19)	298.16 (272.91, 323.41)
2030	Female	15833969.08 (14240015.06, 17427923.11)	298.76 (269.08, 328.44)
2031	Female	16144762.17 (14272153.88, 18017370.46)	299.35 (264.99, 333.71)
2032	Female	16454892.39 (14280814.62, 18628970.16)	299.95 (260.67, 339.23)
2033	Female	16765436.05 (14267262.67, 19263609.42)	300.58 (256.14, 345.01)
2034	Female	17078226.29 (14233056.43, 19923396.15)	301.2 (251.38, 351.01)
2035	Female	17391628.24 (14176559.3, 20606697.17)	301.81 (246.41, 357.21)
2036	Female	17704214.22 (14096055.02, 21312373.42)	302.4 (241.21, 363.6)
2037	Female	18016393.77 (13991041.99, 22041745.56)	303.02 (235.82, 370.22)
2038	Female	18328743.38 (13861943, 22795543.75)	303.65 (230.23, 377.06)
2039	Female	18642932.23 (13709774.28, 23576090.19)	304.28 (224.45, 384.1)
2040	Female	18957712.66 (13533146.21, 24382279.11)	304.89 (218.47, 391.32)
2041	Female	19271986.89 (13330559.51, 25213414.27)	305.49 (212.28, 398.69)
2042	Female	19587166.95 (13101972.17, 26072361.72)	306.1 (205.92, 406.29)
2043	Female	19903448.42 (12847178.95, 26959717.89)	306.73 (199.37, 414.09)
2044	Female	20222119.91 (12566495.7, 27877744.12)	307.36 (192.65, 422.07)
2045	Female	20541991.45 (12258463.41, 28825519.49)	307.96 (185.73, 430.19)

2022	Male	5131716.49 (5038850.81, 5224582.17)	120.27 (119.13, 121.42)
2023	Male	5249526.05 (5131488.1, 5367564)	120.58 (118.61, 122.55)
2024	Male	5368938.73 (5211547.5, 5526329.96)	120.87 (117.86, 123.88)
2025	Male	5489361.25 (5280423.4, 5698299.09)	121.13 (116.92, 125.34)
2026	Male	5611357.5 (5340104.33, 5882610.67)	121.41 (115.85, 126.96)
2027	Male	5734260.84 (5390630.09, 6077891.6)	121.7 (114.66, 128.74)
2028	Male	5857679.13 (5432466.48, 6282891.79)	121.99 (113.35, 130.63)
2029	Male	5982140.45 (5466495.75, 6497785.16)	122.25 (111.9, 132.6)
2030	Male	6106990.4 (5492245.04, 6721735.77)	122.49 (110.33, 134.66)
2031	Male	6232987.23 (5510262.84, 6955711.61)	122.74 (108.67, 136.82)
2032	Male	6359623.57 (5519760.85, 7199486.3)	123.01 (106.92, 139.11)
2033	Male	6486026.16 (5520125.51, 7451926.81)	123.28 (105.07, 141.48)
2034	Male	6612731.38 (5511848.54, 7713614.23)	123.51 (103.11, 143.92)
2035	Male	6739415.02 (5494573.68, 7984256.35)	123.73 (101.05, 146.41)
2036	Male	6867140.88 (5468901.88, 8265379.88)	123.95 (98.9, 148.99)
2037	Male	6995795.14 (5434278.24, 8557312.04)	124.19 (96.69, 151.69)
2038	Male	7124264.56 (5389818.15, 8858710.97)	124.43 (94.39, 154.47)
2039	Male	7252849.16 (5335684.96, 9170013.36)	124.64 (92, 157.29)
2040	Male	7381316.84 (5271552.92, 9491080.76)	124.83 (89.51, 160.16)
2041	Male	7510957.71 (5198019.33, 9823896.1)	125.03 (86.95, 163.11)
2042	Male	7642045.91 (5114724.04, 10169367.79)	125.25 (84.33, 166.17)
2043	Male	7773409.3 (5020722.83, 10526095.78)	125.47 (81.64, 169.3)
2044	Male	7905035.86 (4915833.77, 10894237.96)	125.66 (78.85, 172.47)
2045	Male	8036524.48 (4799567.96, 11273480.99)	125.83 (75.99, 175.68)
**YLDs**			
2022	Both	2433524.81 (2386487.62, 2480561.99)	27.51 (26.98, 28.05)

2023	Both	2483925.7 (2424594.02, 2543257.37)	27.54 (26.88, 28.2)
2024	Both	2534858.2 (2456386.48, 2613329.91)	27.57 (26.72, 28.43)
2025	Both	2586051.82 (2482571.96, 2689531.67)	27.6 (26.49, 28.7)
2026	Both	2637519.58 (2503893.79, 2771145.37)	27.63 (26.23, 29.03)
2027	Both	2689100.03 (2520574.3, 2857625.75)	27.66 (25.93, 29.4)
2028	Both	2740829.77 (2533076.83, 2948582.7)	27.7 (25.59, 29.8)
2029	Both	2792891.19 (2541782.38, 3044000)	27.73 (25.23, 30.22)
2030	Both	2844989.34 (2546502.84, 3143475.85)	27.76 (24.85, 30.68)
2031	Both	2897191.17 (2547262.88, 3247119.46)	27.8 (24.44, 31.16)
2032	Both	2949397.34 (2543858.55, 3354936.13)	27.84 (24.01, 31.67)
2033	Both	3001447.67 (2536241.38, 3466653.96)	27.88 (23.56, 32.21)
2034	Both	3053538.46 (2524606.82, 3582470.11)	27.93 (23.09, 32.77)
2035	Both	3105502.86 (2508787.88, 3702217.83)	27.97 (22.59, 33.35)
2036	Both	3157518.3 (2488824.19, 3826212.41)	28.02 (22.08, 33.96)
2037	Both	3209657.86 (2464599.17, 3954716.54)	28.08 (21.55, 34.6)
2038	Both	3261646.78 (2435911.66, 4087381.9)	28.14 (21.01, 35.27)
2039	Both	3313621.94 (2402843.98, 4224399.9)	28.2 (20.44, 35.96)
2040	Both	3365486.5 (2365259.45, 4365713.55)	28.26 (19.85, 36.67)
2041	Both	3417508.76 (2323210.96, 4511806.56)	28.33 (19.25, 37.42)
2042	Both	3469928.31 (2276647.22, 4663209.4)	28.41 (18.62, 38.19)
2043	Both	3522399.59 (2225282.09, 4819517.08)	28.49 (17.98, 39)
2044	Both	3574965.71 (2169068.11, 4980863.3)	28.58 (17.32, 39.84)
2045	Both	3627525.06 (2107830.35, 5147219.77)	28.67 (16.63, 40.7)
2022	Female	1750660.28 (1718307.89, 1783012.67)	38.5 (38.13, 38.87)
2023	Female	1786489.55 (1745348.03, 1827631.06)	38.54 (37.89, 39.19)
2024	Female	1822693.88 (1767875.68, 1877512.08)	38.58 (37.59, 39.56)

2025	Female	1859075.38 (1786427.95, 1931722.81)	38.61 (37.23, 39.99)
2026	Female	1895586.11 (1801493.27, 1989678.95)	38.65 (36.83, 40.46)
2027	Female	1932129.74 (1813250.41, 2051009.06)	38.68 (36.39, 40.98)
2028	Female	1968798.81 (1822083.1, 2115514.51)	38.73 (35.91, 41.54)
2029	Female	2005704.3 (1828240.39, 2183168.21)	38.76 (35.4, 42.13)
2030	Female	2042633.87 (1831585.91, 2253681.83)	38.8 (34.85, 42.75)
2031	Female	2079574.93 (1832087.82, 2327062.03)	38.84 (34.27, 43.41)
2032	Female	2116470.77 (1829618.98, 2403322.56)	38.88 (33.66, 44.1)
2033	Female	2153269.97 (1824196.04, 2482343.9)	38.92 (33.02, 44.82)
2034	Female	2190091.51 (1815939.28, 2564243.73)	38.96 (32.36, 45.56)
2035	Female	2226812.24 (1804728.43, 2648896.06)	39 (31.66, 46.33)
2036	Female	2263497.04 (1790549.05, 2736445.02)	39.03 (30.94, 47.12)
2037	Female	2300213.34 (1773338.84, 2827087.84)	39.08 (30.2, 47.95)
2038	Female	2336817.48 (1752994.52, 2920640.43)	39.12 (29.43, 48.8)
2039	Female	2373393.56 (1729565.92, 3017221.2)	39.16 (28.64, 49.68)
2040	Female	2409877.96 (1702963.27, 3116792.64)	39.2 (27.82, 50.57)
2041	Female	2446403.73 (1673192, 3219615.46)	39.23 (26.98, 51.49)
2042	Female	2483162.55 (1640240.45, 3326084.65)	39.28 (26.12, 52.43)
2043	Female	2519939.83 (1603927.62, 3435952.04)	39.32 (25.23, 53.4)
2044	Female	2556758.54 (1564218.37, 3549298.71)	39.36 (24.32, 54.39)
2045	Female	2593569.76 (1521007.4, 3666132.11)	39.39 (23.39, 55.4)
2022	Male	682864.53 (668179.74, 697549.32)	15.97 (15.8, 16.14)
2023	Male	697436.15 (679245.99, 715626.31)	16 (15.71, 16.29)
2024	Male	712164.32 (688510.8, 735817.84)	16.02 (15.58, 16.46)
2025	Male	726976.44 (696144.02, 757808.86)	16.04 (15.43, 16.64)
2026	Male	741933.48 (702400.53, 781466.43)	16.06 (15.26, 16.85)

2027	Male	756970.29 (707323.89, 806616.7)	16.08 (15.07, 17.09)
2028	Male	772030.96 (710993.72, 833068.19)	16.1 (14.87, 17.33)
2029	Male	787186.89 (713541.99, 860831.79)	16.12 (14.64, 17.59)
2030	Male	802355.47 (714916.93, 889794.02)	16.13 (14.4, 17.86)
2031	Male	817616.24 (715175.06, 920057.43)	16.15 (14.15, 18.14)
2032	Male	832926.57 (714239.57, 951613.58)	16.16 (13.89, 18.44)
2033	Male	848177.7 (712045.34, 984310.05)	16.18 (13.61, 18.75)
2034	Male	863446.96 (708667.53, 1018226.38)	16.2 (13.32, 19.07)
2035	Male	878690.61 (704059.44, 1053321.78)	16.21 (13.02, 19.4)
2036	Male	894021.26 (698275.14, 1089767.38)	16.22 (12.7, 19.74)
2037	Male	909444.52 (691260.33, 1127628.7)	16.23 (12.38, 20.09)
2038	Male	924829.3 (682917.14, 1166741.47)	16.25 (12.04, 20.45)
2039	Male	940228.38 (673278.06, 1207178.7)	16.26 (11.69, 20.82)
2040	Male	955608.55 (662296.18, 1248920.91)	16.27 (11.33, 21.2)
2041	Male	971105.03 (650018.96, 1292191.1)	16.28 (10.97, 21.58)
2042	Male	986765.76 (636406.77, 1337124.75)	16.29 (10.59, 21.98)
2043	Male	1002459.76 (621354.47, 1383565.05)	16.3 (10.2, 22.39)
2044	Male	1018207.17 (604849.74, 1431564.59)	16.31 (9.8, 22.81)
2045	Male	1033955.3 (586822.95, 1481087.66)	16.31 (9.4, 23.23)

Notes: ASR: age-standardized rates; YLDs: years lived with disability.

Table S73. Prevalence and YLDs of tension-type headache in the worldwide of future forecasts using bayesian age-period-cohort model.

Year	Sex	Number	ASR
**Prevalence**			
2022	Both	2055016833.29 (2027419145.96, 2082614520.63)	24730.79 (24397.95, 25063.63)
2023	Both	2077480358.17 (2040992700.47, 2113968015.87)	24725.32 (24289.94, 25160.69)
2024	Both	2099704963.85 (2049616191.39, 2149793736.31)	24719.81 (24128.58, 25311.04)
2025	Both	2121577517.05 (2054181334.99, 2188973699.11)	24713.67 (23926.55, 25500.79)
2026	Both	2142909588.45 (2055122807.7, 2230696369.2)	24705.21 (23690.43, 25720)
2027	Both	2163923429.5 (2052930062.88, 2274916796.12)	24695.59 (23425.29, 25965.89)
2028	Both	2184295089.56 (2047626612.08, 2320963567.05)	24686.39 (23137.08, 26235.7)
2029	Both	2204334579.17 (2039711403.95, 2368957754.39)	24677.55 (22828.47, 26526.63)
2030	Both	2223959911.04 (2029232160.26, 2418687661.83)	24668.57 (22500.78, 26836.36)
2031	Both	2243020110.46 (2016095650.94, 2469944569.99)	24657.91 (22153.4, 27162.42)
2032	Both	2261686086.96 (2000425622.7, 2522946551.23)	24646.7 (21787.29, 27506.11)
2033	Both	2279979347.77 (1982332795.16, 2577625900.39)	24636.58 (21405.22, 27867.94)
2034	Both	2297930703.78 (1961919434.27, 2633941973.28)	24627.57 (21008.11, 28247.03)
2035	Both	2315448027.44 (1939155283.58, 2691740771.29)	24619.25 (20596.23, 28642.27)
2036	Both	2332391156.3 (1913929985.18, 2750852327.42)	24610.37 (20168.68, 29052.06)
2037	Both	2348907940.72 (1886320397.34, 2811495484.1)	24602.02 (19725.94, 29478.1)
2038	Both	2365080384.35 (1856442995.87, 2873717772.83)	24595.74 (19269.83, 29921.65)
2039	Both	2380925870.06 (1824353382.81, 2937498357.31)	24591.52 (18800.72, 30382.33)
2040	Both	2396400145.98 (1790042248.84, 3002758043.11)	24588.95 (18318.48, 30859.43)
2041	Both	2411407213.26 (1753430295.66, 3069384130.87)	24587 (17822.19, 31351.81)
2042	Both	2426102082.63 (1714579404.09, 3137624761.18)	24586.62 (17311.99, 31861.26)
2043	Both	2440533869.34 (1673548196.86, 3207519541.82)	24589.16 (16788.98, 32389.35)

2044	Both	2454704756.85 (1630356143.19, 3279053370.52)	24594.64 (16253.17, 32936.11)
2045	Both	2468619978.04 (1585010799.53, 3352229156.55)	24602.78 (15704.25, 33501.31)
2022	Female	1065683943.41 (1051514032, 1079853854.81)	25616.82 (25429.44, 25804.19)
2023	Female	1077328628.44 (1058564961.38, 1096092295.5)	25609.63 (25269.93, 25949.33)
2024	Female	1088818034.55 (1063025317.43, 1114610751.67)	25601.22 (25071.44, 26131)
2025	Female	1100113439.21 (1065378774.98, 1134848103.44)	25591.24 (24842.54, 26339.94)
2026	Female	1111161749.63 (1065895769.22, 1156427730.03)	25578.89 (24586.28, 26571.5)
2027	Female	1122068597.89 (1064824631.01, 1179312564.77)	25565.12 (24305.14, 26825.1)
2028	Female	1132644293.98 (1062152417.36, 1203136170.6)	25550.04 (24001.97, 27098.12)
2029	Female	1143021119.5 (1058108892.03, 1227933346.98)	25533.23 (23678.31, 27388.14)
2030	Female	1153175770.08 (1052736069.17, 1253615470.99)	25514.45 (23335.39, 27693.5)
2031	Female	1163074872.15 (1046027337.18, 1280122407.12)	25493.06 (22973.41, 28012.72)
2032	Female	1172800650.35 (1038043270.34, 1307558030.36)	25470.1 (22593.34, 28346.86)
2033	Female	1182313829.24 (1028794847.66, 1335832810.82)	25445.69 (22196.58, 28694.8)
2034	Female	1191617776.91 (1018323655.91, 1364911897.91)	25419.51 (21783.86, 29055.16)
2035	Female	1200681439.86 (1006628840.4, 1394734039.31)	25391.39 (21355.84, 29426.93)
2036	Female	1209475917.99 (993690726.4, 1425261109.58)	25360.91 (20912.58, 29809.25)
2037	Female	1218070393.44 (979544500.14, 1456596286.74)	25329.05 (20454.76, 30203.34)
2038	Female	1226444240.58 (964199086.02, 1488689395.15)	25295.86 (19983.25, 30608.47)
2039	Female	1234596681.66 (947677216.31, 1521516147.01)	25260.97 (19498.47, 31023.48)
2040	Female	1242529575.94 (929995360.79, 1555063791.09)	25224.21 (19000.83, 31447.6)
2041	Female	1250246247.17 (911153704.42, 1589338789.91)	25185.31 (18490.39, 31880.23)
2042	Female	1257829515 (891187123.67, 1624471906.34)	25145.08 (17967.65, 32322.52)
2043	Female	1265249053.35 (870085597.57, 1660412509.13)	25103.54 (17433.21, 32773.88)
2044	Female	1272488884.03 (847849190.41, 1697128577.65)	25060.33 (16887.36, 33233.3)
2045	Female	1279572911 (824498673.82, 1734647148.17)	25015.34 (16330.46, 33700.22)

2022	Male	989332889.89 (975905113.96, 1002760665.82)	23831.97 (23654.92, 24009.02)
2023	Male	1000151729.73 (982427739.09, 1017875720.37)	23828.13 (23508.29, 24147.98)
2024	Male	1010886929.3 (986590873.96, 1035182984.64)	23824.59 (23326.47, 24322.7)
2025	Male	1021464077.84 (988802560.01, 1054125595.67)	23820.16 (23116.7, 24523.63)
2026	Male	1031747838.82 (989227038.48, 1074268639.17)	23812.03 (22879.76, 24744.3)
2027	Male	1041854831.61 (988105431.87, 1095604231.35)	23801.16 (22617.92, 24984.41)
2028	Male	1051650795.58 (985474194.72, 1117827396.44)	23790.23 (22336.42, 25244.04)
2029	Male	1061313459.66 (981602511.92, 1141024407.41)	23779.26 (22037.13, 25521.4)
2030	Male	1070784140.96 (976496091.09, 1165072190.84)	23767.2 (21720.36, 25814.03)
2031	Male	1079945238.31 (970068313.76, 1189822162.87)	23751.28 (21384.28, 26118.29)
2032	Male	1088885436.61 (962382352.36, 1215388520.87)	23732.41 (21029.66, 26435.16)
2033	Male	1097665518.53 (953537947.5, 1241793089.56)	23713.31 (20660.2, 26766.41)
2034	Male	1106312926.87 (943595778.36, 1269030075.38)	23694.05 (20276.92, 27111.19)
2035	Male	1114766587.58 (932526443.18, 1297006731.98)	23673.67 (19879.65, 27467.69)
2036	Male	1122915238.31 (920239258.78, 1325591217.84)	23649.73 (19466.71, 27832.74)
2037	Male	1130837547.28 (906775897.2, 1354899197.35)	23623.07 (19038.63, 28207.51)
2038	Male	1138636143.77 (892243909.85, 1385028377.69)	23596.31 (18598.37, 28594.24)
2039	Male	1146329188.4 (876676166.5, 1415982210.3)	23569.48 (18146.58, 28992.39)
2040	Male	1153870570.04 (860046888.05, 1447694252.02)	23541.63 (17682.95, 29400.3)
2041	Male	1161160966.1 (842276591.24, 1480045340.95)	23510.58 (17206.06, 29815.09)
2042	Male	1168272567.63 (823392280.42, 1513152854.84)	23477.02 (16716.28, 30237.75)
2043	Male	1175284815.99 (803462599.29, 1547107032.69)	23443.21 (16215.85, 30670.57)
2044	Male	1182215872.82 (782506952.77, 1581924792.87)	23409.23 (15705.28, 31113.18)
2045	Male	1189047067.04 (760512125.71, 1617582008.38)	23374.27 (15184.34, 31564.21)
**YLDs**			
2022	Both	4713788.92 (4640571.02, 4787006.82)	55.7 (54.83, 56.57)

2023	Both	4771693.87 (4676895.29, 4866492.45)	55.7 (54.59, 56.81)
2024	Both	4829195.39 (4701422.26, 4956968.51)	55.7 (54.22, 57.18)
2025	Both	4886201.79 (4716279.08, 5056124.5)	55.7 (53.76, 57.64)
2026	Both	4942120.62 (4722280.63, 5161960.6)	55.7 (53.22, 58.19)
2027	Both	4997543.45 (4720576.3, 5274510.6)	55.7 (52.61, 58.8)
2028	Both	5051872.84 (4711548.2, 5392197.47)	55.71 (51.95, 59.47)
2029	Both	5105568.48 (4696162.46, 5514974.5)	55.72 (51.24, 60.2)
2030	Both	5158525.05 (4674608.05, 5642442.05)	55.73 (50.48, 60.97)
2031	Both	5210247.54 (4646457.73, 5774037.36)	55.73 (49.68, 61.79)
2032	Both	5261409.37 (4612108.1, 5910710.65)	55.75 (48.84, 62.65)
2033	Both	5311355.25 (4571289.01, 6051421.49)	55.77 (47.97, 63.57)
2034	Both	5360391.97 (4524488.81, 6196295.12)	55.79 (47.05, 64.53)
2035	Both	5408547.01 (4471841.58, 6345252.44)	55.81 (46.1, 65.53)
2036	Both	5455524.99 (4413040.34, 6498009.64)	55.84 (45.12, 66.57)
2037	Both	5502118.61 (4348482.61, 6655754.61)	55.87 (44.09, 67.66)
2038	Both	5547852.06 (4277946.88, 6817757.24)	55.91 (43.04, 68.79)
2039	Both	5592722.22 (4201559.16, 6983885.27)	55.96 (41.95, 69.97)
2040	Both	5636868.3 (4119475.67, 7154260.93)	56.01 (40.83, 71.19)
2041	Both	5680179.98 (4031518.53, 7328841.43)	56.06 (39.67, 72.46)
2042	Both	5723519.79 (3938039.95, 7508999.62)	56.12 (38.47, 73.77)
2043	Both	5766630.94 (3838914.62, 7694347.26)	56.19 (37.25, 75.14)
2044	Both	5809247.84 (3734000.45, 7884495.23)	56.27 (35.98, 76.56)
2045	Both	5851434.79 (3623320.8, 8079548.78)	56.35 (34.68, 78.02)
2022	Female	2634425.62 (2593871.76, 2674979.48)	62.01 (61.5, 62.52)
2023	Female	2666747.82 (2614163.84, 2719331.81)	62 (61.09, 62.91)
2024	Female	2698869.86 (2627927.73, 2769811.99)	62 (60.6, 63.4)

2025	Female	2730749.95 (2636364.53, 2825135.38)	62 (60.03, 63.97)
2026	Female	2761925.89 (2639778.12, 2884073.66)	61.99 (59.39, 64.59)
2027	Female	2792810.63 (2638840.37, 2946780.89)	61.98 (58.69, 65.27)
2028	Female	2823114.4 (2633843.87, 3012384.92)	61.98 (57.94, 66.01)
2029	Female	2853077.87 (2625325.49, 3080830.24)	61.97 (57.14, 66.8)
2030	Female	2882652.3 (2613405.59, 3151899)	61.96 (56.29, 67.63)
2031	Female	2911445.16 (2597725.87, 3225164.46)	61.95 (55.4, 68.5)
2032	Female	2939930.17 (2578555.41, 3301304.93)	61.94 (54.46, 69.42)
2033	Female	2967732.29 (2555758.37, 3379706.22)	61.93 (53.48, 70.37)
2034	Female	2995011.37 (2529609.9, 3460412.85)	61.91 (52.46, 71.37)
2035	Female	3021792.08 (2500200.02, 3543384.15)	61.9 (51.41, 72.39)
2036	Female	3047816.98 (2467271.64, 3628362.32)	61.88 (50.31, 73.45)
2037	Female	3073639.12 (2431105.2, 3716173.03)	61.86 (49.17, 74.54)
2038	Female	3098954.08 (2391563.35, 3806344.82)	61.84 (48, 75.67)
2039	Female	3123737.13 (2348711.18, 3898763.08)	61.81 (46.8, 76.82)
2040	Female	3148087.35 (2302660.5, 3993514.21)	61.78 (45.56, 78.01)
2041	Female	3171885.19 (2253262.96, 4090507.42)	61.75 (44.28, 79.21)
2042	Female	3195742.79 (2200782.6, 4190702.97)	61.71 (42.97, 80.45)
2043	Female	3219470.94 (2145129.99, 4293811.9)	61.68 (41.63, 81.72)
2044	Female	3242872.45 (2086203.32, 4399541.59)	61.64 (40.26, 83.01)
2045	Female	3265999.47 (2024033.96, 4507964.97)	61.59 (38.85, 84.33)
2022	Male	2079363.3 (2046699.26, 2112027.34)	49.35 (48.94, 49.76)
2023	Male	2104946.04 (2062731.45, 2147160.64)	49.36 (48.62, 50.09)
2024	Male	2130325.53 (2073494.53, 2187156.53)	49.36 (48.23, 50.49)
2025	Male	2155451.83 (2079914.55, 2230989.11)	49.36 (47.78, 50.95)
2026	Male	2180194.73 (2082502.51, 2277886.94)	49.36 (47.27, 51.46)

2027	Male	2204732.82 (2081735.94, 2327729.71)	49.36 (46.71, 52.02)
2028	Male	2228758.44 (2077704.33, 2379812.55)	49.36 (46.11, 52.62)
2029	Male	2252490.61 (2070836.96, 2434144.26)	49.36 (45.47, 53.25)
2030	Male	2275872.76 (2061202.46, 2490543.05)	49.36 (44.79, 53.93)
2031	Male	2298802.38 (2048731.86, 2548872.9)	49.35 (44.07, 54.63)
2032	Male	2321479.21 (2033552.69, 2609405.72)	49.34 (43.32, 55.37)
2033	Male	2343622.96 (2015530.64, 2671715.27)	49.34 (42.53, 56.14)
2034	Male	2365380.6 (1994878.92, 2735882.28)	49.33 (41.71, 56.94)
2035	Male	2386754.92 (1971641.55, 2801868.29)	49.32 (40.87, 57.76)
2036	Male	2407708.01 (1945768.7, 2869647.33)	49.3 (39.99, 58.61)
2037	Male	2428479.49 (1917377.4, 2939581.58)	49.28 (39.08, 59.49)
2038	Male	2448897.97 (1886383.53, 3011412.42)	49.27 (38.14, 60.4)
2039	Male	2468985.08 (1852847.98, 3085122.19)	49.25 (37.17, 61.33)
2040	Male	2488780.95 (1816815.17, 3160746.73)	49.23 (36.18, 62.28)
2041	Male	2508294.79 (1778255.57, 3238334.01)	49.2 (35.16, 63.25)
2042	Male	2527777 (1737257.35, 3318296.65)	49.17 (34.11, 64.24)
2043	Male	2547160 (1693784.63, 3400535.37)	49.15 (33.04, 65.25)
2044	Male	2566375.38 (1647797.12, 3484953.64)	49.11 (31.94, 66.29)
2045	Male	2585435.33 (1599286.84, 3571583.81)	49.08 (30.82, 67.34)

Notes: ASR: age-standardized rates; YLDs: years lived with disability.

Table S74. Prevalence and YLDs of migraine in the worldwide of future forecasts using bayesian age-period-cohort model.

Year	Sex	Number	ASR
**Prevalence**			
2022	Both	1184978656.18 (1169143597.79, 1200813714.57)	14244.55 (14053.92, 14435.18)
2023	Both	1197301424.07 (1176244390.63, 1218358457.5)	14247.32 (13996.36, 14498.28)
2024	Both	1209293587.95 (1180250066.59, 1238337109.31)	14248.33 (13905.61, 14591.04)
2025	Both	1220965700.2 (1181780494.73, 1260150905.67)	14247.89 (13789.96, 14705.82)
2026	Both	1232487782.2 (1181376096.57, 1283599467.84)	14248.16 (13656.42, 14839.9)
2027	Both	1243925336.61 (1179275685.72, 1308574987.49)	14249.04 (13507.34, 14990.75)
2028	Both	1254894146.65 (1175298470.83, 1334489822.47)	14249.47 (13344.14, 15154.81)
2029	Both	1265452501.91 (1169619592.56, 1361285411.25)	14248.52 (13167.49, 15329.54)
2030	Both	1275637428.84 (1162350277.41, 1388924580.27)	14246.56 (12978.79, 15514.33)
2031	Both	1285648350.94 (1153704599.19, 1417592102.69)	14245.65 (12780.39, 15710.91)
2032	Both	1295557646.34 (1143735763.35, 1447379529.33)	14245.79 (12572.29, 15919.3)
2033	Both	1305062458.95 (1132225583.1, 1477899334.8)	14245.82 (12354.11, 16137.52)
2034	Both	1314116097.98 (1119183688.37, 1509048507.6)	14244.92 (12125.69, 16364.15)
2035	Both	1322781383.45 (1104701435.54, 1540861331.36)	14243.53 (11887.8, 16599.26)
2036	Both	1331281086.61 (1088975836.47, 1573586336.76)	14243.54 (11642.11, 16844.97)
2037	Both	1339700346.26 (1072051877.19, 1607348815.33)	14244.99 (11388.4, 17101.57)
2038	Both	1347831131.05 (1053785328.13, 1641876933.98)	14246.74 (11126.05, 17367.43)
2039	Both	1355565839.98 (1034121126.83, 1677010553.13)	14248.18 (10854.78, 17641.57)
2040	Both	1362973779.24 (1013136583.66, 1712810974.83)	14249.76 (10575.12, 17924.41)
2041	Both	1370269103.26 (990993370.92, 1749544835.61)	14253.21 (10288.29, 18218.14)
2042	Both	1377528519.33 (967716793.64, 1787340245.02)	14258.6 (9993.98, 18523.23)
2043	Both	1384566711.02 (943183168.31, 1825950253.73)	14264.91 (9691.48, 18838.33)

2044	Both	1391285296.07 (917340131.3, 1865230460.84)	14271.66 (9380.51, 19162.82)
2045	Both	1397760100.82 (890250018.39, 1905270183.25)	14279.35 (9061.35, 19497.34)
2022	Female	741905567.67 (732012900.2, 751798235.14)	17895.08 (17761.04, 18029.11)
2023	Female	749315982.66 (736176546.39, 762455418.93)	17892.9 (17650.83, 18134.97)
2024	Female	756509343.16 (738402073.27, 774616613.04)	17888.36 (17511.05, 18265.68)
2025	Female	763493445.02 (739078825.4, 787908064.63)	17881.79 (17348.53, 18415.06)
2026	Female	770368517.2 (738540107.03, 802196927.36)	17875.58 (17168.42, 18582.74)
2027	Female	777169977.67 (736931339.16, 817408616.18)	17869.39 (16971.59, 18767.18)
2028	Female	783678420.29 (734160655.51, 833196185.07)	17861.71 (16758.5, 18964.92)
2029	Female	789925353.82 (730333741.13, 849516966.51)	17851.54 (16529.6, 19173.48)
2030	Female	795934962.46 (725522033.5, 866347891.42)	17839.22 (16286.24, 19392.19)
2031	Female	801825213.77 (719854237.41, 883796190.14)	17827.03 (16031.11, 19622.95)
2032	Female	807637089.95 (713360925.68, 901913254.22)	17814.74 (15764.03, 19865.45)
2033	Female	813193696.54 (705918135.98, 920469257.11)	17800.8 (15484.48, 20117.12)
2034	Female	818463241.86 (697530442.65, 939396041.06)	17784.3 (15192.41, 20376.19)
2035	Female	823486353.94 (688257031.52, 958715676.36)	17765.63 (14888.77, 20642.49)
2036	Female	828397557.77 (678218030.57, 978577084.98)	17746.85 (14575.58, 20918.11)
2037	Female	833249470.72 (667442081.81, 999056859.62)	17727.8 (14252.65, 21202.95)
2038	Female	837915356.18 (655843105.77, 1019987606.58)	17707 (13919.31, 21494.69)
2039	Female	842328994.89 (643388535.77, 1041269454.02)	17683.72 (13575.52, 21791.92)
2040	Female	846534742.83 (630128507.15, 1062940978.51)	17658.39 (13222.07, 22094.72)
2041	Female	850662488.13 (616162130.56, 1085162845.71)	17632.86 (12860.54, 22405.19)
2042	Female	854761223.06 (601507049.44, 1108015396.68)	17607.03 (12490.76, 22723.3)
2043	Female	858716123.09 (586088122.12, 1131344124.06)	17579.55 (12112.17, 23046.93)
2044	Female	862466916.39 (569874698.4, 1155059134.38)	17549.85 (11724.78, 23374.91)
2045	Female	866061277.01 (552907144.62, 1179215409.4)	17518.36 (11329.25, 23707.47)

2022	Male	443073088.51 (437130697.6, 449015479.43)	10626.35 (10545.93, 10706.77)
2023	Male	447985441.41 (440067844.24, 455903038.57)	10635.64 (10490.25, 10781.02)
2024	Male	452784244.79 (441847993.32, 463720496.27)	10643.41 (10416.59, 10870.22)
2025	Male	457472255.18 (442701669.32, 472242841.04)	10649.78 (10328.93, 10970.64)
2026	Male	462119265.01 (442835989.54, 481402540.48)	10656.44 (10230.55, 11082.33)
2027	Male	466755358.94 (442344346.56, 491166371.31)	10663.31 (10122.1, 11204.52)
2028	Male	471215726.36 (441137815.33, 501293637.4)	10669.43 (10003.74, 11335.12)
2029	Male	475527148.08 (439285851.43, 511768444.73)	10673.92 (9875.47, 11472.38)
2030	Male	479702466.38 (436828243.91, 522576688.85)	10676.98 (9738.08, 11615.89)
2031	Male	483823137.16 (433850361.78, 533795912.55)	10680.21 (9593.36, 11767.07)
2032	Male	487920556.39 (430374837.67, 545466275.11)	10683.56 (9441.27, 11925.85)
2033	Male	491868762.41 (426307447.12, 557430077.7)	10686.02 (9281.41, 12090.64)
2034	Male	495652856.13 (421653245.72, 569652466.53)	10686.85 (9113.55, 12260.16)
2035	Male	499295029.51 (416444404.01, 582145655)	10686.28 (8938.24, 12434.32)
2036	Male	502883528.84 (410757805.9, 595009251.78)	10685.75 (8756.86, 12614.64)
2037	Male	506450875.54 (404609795.37, 608291955.71)	10685.22 (8569.3, 12801.13)
2038	Male	509915774.88 (397942222.35, 621889327.4)	10683.72 (8375.09, 12992.34)
2039	Male	513236845.09 (390732591.06, 635741099.12)	10680.64 (8174.06, 13187.23)
2040	Male	516439036.41 (383008076.51, 649869996.32)	10676.28 (7966.66, 13385.89)
2041	Male	519606615.13 (374831240.36, 664381989.9)	10671.91 (7754, 13589.82)
2042	Male	522767296.27 (366209744.2, 679324848.34)	10667.49 (7535.93, 13799.05)
2043	Male	525850587.93 (357095046.19, 694606129.67)	10662.13 (7312.06, 14012.2)
2044	Male	528818379.67 (347465432.89, 710171326.46)	10655.36 (7082.28, 14228.44)
2045	Male	531698823.81 (337342873.78, 726054773.85)	10647.47 (6847.01, 14447.94)
**YLDs**			
2022	Both	44368795.86 (43751425.03, 44986166.68)	532.46 (525.04, 539.88)

2023	Both	44788509.78 (43971083.2, 45605936.36)	532 (522.28, 541.73)
2024	Both	45194928.44 (44072969.68, 46316887.2)	531.48 (518.27, 544.69)
2025	Both	45587925.46 (44080101.26, 47095749.65)	530.89 (513.31, 548.48)
2026	Both	45975092.62 (44014036.28, 47936148.97)	530.35 (507.69, 553)
2027	Both	46359926.43 (43884697.5, 48835155.37)	529.85 (501.52, 558.18)
2028	Both	46727353.12 (43685219.83, 49769486.41)	529.35 (494.83, 563.87)
2029	Both	47078576.61 (43421579.42, 50735573.8)	528.79 (487.64, 569.94)
2030	Both	47414338.02 (43097551.65, 51731124.4)	528.19 (480.01, 576.38)
2031	Both	47743435.97 (42722355.27, 52764516.66)	527.65 (472.03, 583.26)
2032	Both	48069633.9 (42298757.66, 53840510.13)	527.16 (463.72, 590.61)
2033	Both	48380295.82 (41817744.99, 54942846.65)	526.69 (455.05, 598.33)
2034	Both	48672940.92 (41279291.4, 56066590.44)	526.18 (446.01, 606.35)
2035	Both	48949553.14 (40686675.76, 57212430.52)	525.65 (436.62, 614.67)
2036	Both	49220179.61 (40048685.22, 58391673.99)	525.18 (426.97, 623.39)
2037	Both	49489218.36 (39367820.57, 59610616.15)	524.79 (417.04, 632.53)
2038	Both	49747860.56 (38638041.9, 60857679.21)	524.42 (406.81, 642.03)
2039	Both	49990803.91 (37856475.82, 62125131.99)	524.04 (396.26, 651.82)
2040	Both	50220384.86 (37025953.94, 63414815.78)	523.66 (385.4, 661.91)
2041	Both	50446521.51 (36153903.19, 64739139.82)	523.35 (374.29, 672.42)
2042	Both	50673284.8 (35241945.34, 66104624.26)	523.15 (362.93, 683.36)
2043	Both	50893130.87 (34285107.12, 67501154.62)	522.98 (351.29, 694.68)
2044	Both	51100840.89 (33280474.07, 68921207.71)	522.83 (339.34, 706.33)
2045	Both	51298740.19 (32230107.2, 70367373.19)	522.71 (327.09, 718.34)
2022	Female	27486686.12 (27107809.73, 27865562.51)	662.01 (656.88, 667.15)
2023	Female	27728074.98 (27226813.36, 28229336.6)	661.07 (651.86, 670.28)
2024	Female	27960727.02 (27273111.72, 28648342.32)	660.04 (645.76, 674.33)

2025	Female	28184424.42 (27260841.92, 29108006.92)	658.93 (638.81, 679.04)
2026	Female	28403680.46 (27203190.14, 29604170.78)	657.83 (631.23, 684.43)
2027	Female	28620547.21 (27106198.02, 30134896.41)	656.77 (623.06, 690.47)
2028	Female	28826633.41 (26966583.8, 30686683.03)	655.66 (614.33, 697)
2029	Female	29022351.12 (26787784.65, 31256917.59)	654.47 (605.03, 703.91)
2030	Female	29208044.25 (26572092.6, 31843995.89)	653.18 (595.2, 711.16)
2031	Female	29388994.04 (26325120.64, 32452867.44)	651.91 (584.96, 718.85)
2032	Female	29567438.48 (26048535.89, 33086341.07)	650.66 (574.33, 726.99)
2033	Female	29736250.83 (25737446.58, 33735055.09)	649.37 (563.28, 735.47)
2034	Female	29893574.63 (25391609.39, 34395539.87)	647.99 (551.78, 744.19)
2035	Female	30040543.99 (25013039, 35068048.99)	646.51 (539.87, 753.14)
2036	Female	30183300.61 (24607177.02, 35759424.2)	645.03 (527.65, 762.42)
2037	Female	30324586.93 (24175635.34, 36473538.52)	643.58 (515.11, 772.05)
2038	Female	30459255.12 (23714994.54, 37203515.69)	642.08 (502.22, 781.93)
2039	Female	30583871.18 (23223416.03, 37944326.32)	640.48 (488.98, 791.98)
2040	Female	30699789.89 (22702655.31, 38696924.47)	638.8 (475.4, 802.19)
2041	Female	30813120.97 (22157367.48, 39468874.46)	637.12 (461.56, 812.67)
2042	Female	30926451.62 (21588666.24, 40264237)	635.45 (447.46, 823.44)
2043	Female	31035277.4 (20993668.84, 41076885.95)	633.73 (433.08, 834.39)
2044	Female	31136243.54 (20370556.78, 41901930.3)	631.93 (418.39, 845.47)
2045	Female	31230688.68 (19720626.04, 42740751.32)	630.05 (403.44, 856.67)
2022	Male	16882109.73 (16643615.29, 17120604.17)	404.1 (400.88, 407.33)
2023	Male	17060434.8 (16744269.84, 17376599.75)	404.18 (398.4, 409.96)
2024	Male	17234201.42 (16799857.96, 17668544.88)	404.2 (395.23, 413.17)
2025	Male	17403501.04 (16819259.34, 17987742.73)	404.16 (391.52, 416.81)
2026	Male	17571412.17 (16810846.14, 18331978.2)	404.15 (387.41, 420.89)

2027	Male	17739379.22 (16778499.47, 18700258.96)	404.16 (382.92, 425.39)
2028	Male	17900719.7 (16718636.03, 19082803.37)	404.14 (378.06, 430.23)
2029	Male	18056225.49 (16633794.77, 19478656.2)	404.07 (372.83, 435.31)
2030	Male	18206293.78 (16525459.05, 19887128.5)	403.94 (367.24, 440.63)
2031	Male	18354441.93 (16397234.63, 20311649.22)	403.82 (361.39, 446.25)
2032	Male	18502195.42 (16250221.78, 20754169.07)	403.73 (355.28, 452.18)
2033	Male	18644044.99 (16080298.41, 21207791.56)	403.61 (348.87, 458.34)
2034	Male	18779366.29 (15887682.01, 21671050.58)	403.42 (342.16, 464.69)
2035	Male	18909009.15 (15673636.76, 22144381.53)	403.19 (335.17, 471.2)
2036	Male	19036878.99 (15441508.2, 22632249.79)	402.96 (327.96, 477.95)
2037	Male	19164631.43 (15192185.23, 23137077.63)	402.74 (320.53, 484.95)
2038	Male	19288605.44 (14923047.36, 23654163.51)	402.5 (312.86, 492.14)
2039	Male	19406932.73 (14633059.79, 24180805.67)	402.2 (304.93, 499.46)
2040	Male	19520594.97 (14323298.63, 24717891.31)	401.84 (296.76, 506.92)
2041	Male	19633400.54 (13996535.72, 25270265.35)	401.49 (288.4, 514.58)
2042	Male	19746833.18 (13653279.1, 25840387.26)	401.15 (279.84, 522.46)
2043	Male	19857853.48 (13291438.28, 26424268.67)	400.78 (271.08, 530.49)
2044	Male	19964597.35 (12909917.29, 27019277.41)	400.36 (262.09, 538.63)
2045	Male	20068051.51 (12509481.16, 27626621.86)	399.89 (252.9, 546.88)

Notes: ASR: age-standardized rates; YLDs: years lived with disability.

Table S75. Prevalence and YLDs of stroke in the worldwide of future forecasts using bayesian age-period-cohort model.

Year	Sex	Number	ASR
**Prevalence**			
2022	Both	97952331.78 (95944061.05, 99960602.51)	1109.68 (1086.92, 1132.44)
2023	Both	100849363.41 (98331164, 103367562.81)	1116.49 (1088.6, 1144.38)
2024	Both	103853415.85 (100527139.19, 107179692.51)	1123.21 (1087.22, 1159.2)
2025	Both	106937447.33 (102534245.18, 111340649.47)	1129.92 (1083.37, 1176.47)
2026	Both	110089355.16 (104365791.05, 115812919.26)	1136.81 (1077.67, 1195.95)
2027	Both	113273514.72 (106000780.47, 120546248.97)	1143.82 (1070.34, 1217.31)
2028	Both	116518034.22 (107476436.93, 125559631.51)	1150.81 (1061.45, 1240.18)
2029	Both	119862289.09 (108831597.11, 130892981.07)	1157.78 (1051.16, 1264.4)
2030	Both	123280078.08 (110039324.75, 136520831.41)	1164.81 (1039.61, 1290)
2031	Both	126761893.58 (111084274.43, 142439512.72)	1172.08 (1027.01, 1317.15)
2032	Both	130271335.39 (111925594.07, 148617076.72)	1179.55 (1013.31, 1345.8)
2033	Both	133827832.37 (112576840.7, 155078824.04)	1187.06 (998.4, 1375.71)
2034	Both	137472258.99 (113066300.59, 161878217.39)	1194.62 (982.35, 1406.88)
2035	Both	141183901.14 (113367791.27, 169000011.02)	1202.31 (965.22, 1439.41)
2036	Both	144953383.97 (113462480.13, 176444287.81)	1210.32 (947.13, 1473.51)
2037	Both	148744853.35 (113309757.53, 184179949.16)	1218.58 (927.99, 1509.17)
2038	Both	152576204.57 (112917136.13, 192235273.01)	1226.94 (907.68, 1546.19)
2039	Both	156491299.04 (112308322.34, 200674275.75)	1235.43 (886.24, 1584.62)
2040	Both	160472845.52 (111458874.03, 209486817.01)	1244.15 (863.7, 1624.6)
2041	Both	164510778.25 (110347453.66, 218674102.85)	1253.24 (840.12, 1666.35)
2042	Both	168569733.87 (108934771.38, 228204696.36)	1262.63 (815.37, 1709.89)
2043	Both	172664589.22 (107221801.1, 238107377.33)	1272.22 (789.37, 1755.07)

2044	Both	176840971.05 (105225430.59, 248456511.51)	1282.05 (762.11, 1801.99)
2045	Both	181086374.94 (102922694.32, 259250055.55)	1292.22 (733.6, 1850.84)
2022	Female	47917074.46 (46958324.59, 48875824.32)	1036.03 (1025.33, 1046.73)
2023	Female	49488669.63 (48252563.7, 50724775.56)	1045.9 (1027.05, 1064.76)
2024	Female	51121503.66 (49449059.08, 52793948.24)	1055.7 (1026.45, 1084.95)
2025	Female	52803856.09 (50553639.64, 55054072.54)	1065.49 (1024.1, 1106.89)
2026	Female	54534258.21 (51577326.28, 57491190.13)	1075.52 (1020.41, 1130.63)
2027	Female	56293271.34 (52506594.92, 60079947.76)	1085.66 (1015.33, 1155.99)
2028	Female	58094449.83 (53357903.55, 62830996.1)	1095.77 (1008.82, 1182.72)
2029	Female	59955422.28 (54147192.19, 65763652.37)	1105.8 (1000.89, 1210.7)
2030	Female	61865517.48 (54861889.08, 68869145.88)	1115.83 (991.67, 1239.99)
2031	Female	63826816.14 (55498879.38, 72154752.9)	1126.14 (981.41, 1270.87)
2032	Female	65819812.83 (56034686.1, 75604939.56)	1136.6 (969.97, 1303.23)
2033	Female	67852608.8 (56472723.3, 79232494.29)	1147.04 (957.24, 1336.84)
2034	Female	69942317.15 (56822374.03, 83062260.27)	1157.41 (943.2, 1371.61)
2035	Female	72079151.16 (57069454.25, 87088848.07)	1167.8 (927.95, 1407.65)
2036	Female	74265838.33 (57208615.22, 91323061.44)	1178.47 (911.69, 1445.26)
2037	Female	76482418.99 (57215637.92, 95749200.06)	1189.34 (894.3, 1484.39)
2038	Female	78734166.87 (57089051.05, 100379282.68)	1200.18 (875.63, 1524.74)
2039	Female	81040100 (56836089.36, 105244110.63)	1210.96 (855.68, 1566.25)
2040	Female	83392597.76 (56443358.74, 110341836.77)	1221.78 (834.52, 1609.05)
2041	Female	85796300.21 (55904516.75, 115688083.67)	1232.9 (812.31, 1653.49)
2042	Female	88232439.64 (55196158.19, 121268721.09)	1244.23 (788.94, 1699.51)
2043	Female	90702003.89 (54311394.99, 127092612.79)	1255.53 (764.28, 1746.79)
2044	Female	93225206.17 (53253784.26, 133196628.09)	1266.8 (738.32, 1795.29)
2045	Female	95799542.12 (52011446.55, 139587637.7)	1278.16 (711.14, 1845.17)

2022	Male	50035257.32 (48985736.46, 51084778.19)	1198.01 (1185.64, 1210.38)
2023	Male	51360693.77 (50078600.3, 52642787.25)	1201.38 (1181, 1221.76)
2024	Male	52731912.19 (51078080.11, 54385744.28)	1204.56 (1173.93, 1235.2)
2025	Male	54133591.24 (51980605.54, 56286576.93)	1207.63 (1165.05, 1250.22)
2026	Male	55555096.95 (52788464.77, 58321729.13)	1210.76 (1154.77, 1266.74)
2027	Male	56980243.38 (53494185.55, 60466301.21)	1213.94 (1143.2, 1284.67)
2028	Male	58423584.39 (54118533.38, 62728635.4)	1216.97 (1130.29, 1303.65)
2029	Male	59906866.81 (54684404.92, 65129328.71)	1219.85 (1116.13, 1323.57)
2030	Male	61414560.6 (55177435.66, 67651685.53)	1222.65 (1100.84, 1344.45)
2031	Male	62935077.44 (55585395.05, 70284759.82)	1225.46 (1084.56, 1366.36)
2032	Male	64451522.56 (55890907.97, 73012137.16)	1228.27 (1067.27, 1389.27)
2033	Male	65975223.57 (56104117.4, 75846329.75)	1230.91 (1048.89, 1412.93)
2034	Male	67529941.84 (56243926.56, 78815957.12)	1233.4 (1029.49, 1437.32)
2035	Male	69104749.99 (56298337.02, 81911162.95)	1235.82 (1009.16, 1462.48)
2036	Male	70687545.64 (56253864.91, 85121226.36)	1238.21 (987.96, 1488.45)
2037	Male	72262434.35 (56094119.61, 88430749.1)	1240.52 (965.85, 1515.19)
2038	Male	73842037.71 (55828085.08, 91855990.34)	1242.65 (942.79, 1542.5)
2039	Male	75451199.04 (55472232.97, 95430165.12)	1244.64 (918.85, 1570.43)
2040	Male	77080247.76 (55015515.29, 99144980.24)	1246.57 (894.11, 1599.03)
2041	Male	78714478.04 (54442936.9, 102986019.18)	1248.42 (868.57, 1628.27)
2042	Male	80337294.23 (53738613.2, 106935975.27)	1250.13 (842.19, 1658.07)
2043	Male	81962585.32 (52910406.11, 111014764.54)	1251.66 (814.98, 1688.35)
2044	Male	83615764.88 (51971646.33, 115259883.43)	1253.09 (787.02, 1719.17)
2045	Male	85286832.81 (50911247.78, 119662417.85)	1254.48 (758.36, 1750.6)
**YLDs**			
2022	Both	15871886.83 (15518852.66, 16224921.01)	180.09 (176.08, 184.1)

2023	Both	16341425.6 (15905718.22, 16777132.98)	181.06 (176.23, 185.9)
2024	Both	16830319.79 (16263537.49, 17397102.1)	182.01 (175.88, 188.15)
2025	Both	17333144.11 (16590870.59, 18075417.63)	182.95 (175.11, 190.79)
2026	Both	17847402.34 (16889096.28, 18805708.4)	183.91 (174.03, 193.79)
2027	Both	18365704.66 (17153273.59, 19578135.74)	184.88 (172.67, 197.1)
2028	Both	18893575.83 (17390639.02, 20396512.64)	185.84 (171.05, 200.64)
2029	Both	19438918.8 (17609015.95, 21268821.65)	186.78 (169.18, 204.38)
2030	Both	19996737.08 (17803364.71, 22190109.45)	187.72 (167.11, 208.33)
2031	Both	20565336.81 (17970966.37, 23159707.25)	188.69 (164.86, 212.52)
2032	Both	21137244.27 (18103728.05, 24170760.49)	189.69 (162.44, 216.94)
2033	Both	21715771.88 (18204170.81, 25227372.96)	190.68 (159.81, 221.55)
2034	Both	22308800.24 (18278013.4, 26339587.08)	191.66 (156.99, 226.33)
2035	Both	22912573.58 (18320680.61, 27504466.56)	192.65 (153.99, 231.3)
2036	Both	23525768.67 (18329175.55, 28722361.78)	193.68 (150.84, 236.51)
2037	Both	24141198.59 (18295740.28, 29986656.89)	194.74 (147.53, 241.96)
2038	Both	24761464.94 (18221551.9, 31301377.98)	195.8 (144.02, 247.59)
2039	Both	25394837.03 (18111419.2, 32678254.86)	196.86 (140.32, 253.41)
2040	Both	26038188.26 (17961185.29, 34115191.22)	197.95 (136.45, 259.44)
2041	Both	26690185.36 (17767528.95, 35612841.77)	199.08 (132.42, 265.74)
2042	Both	27343733.96 (17523104.25, 37164363.66)	200.26 (128.21, 272.3)
2043	Both	28000547.87 (17227865.4, 38773230.33)	201.45 (123.81, 279.09)
2044	Both	28669210.68 (16885470.29, 40452951.07)	202.66 (119.2, 286.11)
2045	Both	29347124.86 (16491975.73, 42202273.99)	203.9 (114.41, 293.4)
2022	Female	8202864.53 (8022448.86, 8383280.2)	176.68 (174.74, 178.62)
2023	Female	8465828.44 (8238850.02, 8692806.86)	178.13 (174.8, 181.46)
2024	Female	8739519.52 (8439078.02, 9039961.02)	179.55 (174.44, 184.65)

2025	Female	9021592.94 (8623272, 9419913.87)	180.95 (173.78, 188.12)
2026	Female	9311834.83 (8793243.6, 9830426.07)	182.39 (172.88, 191.89)
2027	Female	9606092.38 (8945947.93, 10266236.84)	183.83 (171.75, 195.92)
2028	Female	9906926.83 (9084652.91, 10729200.76)	185.26 (170.37, 200.15)
2029	Female	10217902.63 (9212726.79, 11223078.47)	186.65 (168.74, 204.57)
2030	Female	10537033.25 (9327942.53, 11746123.98)	188.03 (166.88, 209.18)
2031	Female	10865014.96 (9430063.5, 12299966.41)	189.45 (164.86, 214.04)
2032	Female	11197745.33 (9514382.9, 12881107.76)	190.89 (162.64, 219.14)
2033	Female	11536437.26 (9581479.57, 13491394.94)	192.31 (160.2, 224.41)
2034	Female	11884363.64 (9633349.37, 14135377.91)	193.69 (157.54, 229.84)
2035	Female	12239704.19 (9667510.4, 14811897.97)	195.06 (154.68, 235.44)
2036	Female	12603396.06 (9683418.9, 15523373.21)	196.46 (151.65, 241.28)
2037	Female	12971261.24 (9676321.16, 16266201.31)	197.9 (148.45, 247.35)
2038	Female	13343647.6 (9645713.67, 17041581.54)	199.3 (145.03, 253.57)
2039	Female	13724084.99 (9593188.81, 17854981.18)	200.67 (141.39, 259.94)
2040	Female	14111198.75 (9516527.77, 18705869.72)	202.02 (137.57, 266.47)
2041	Female	14506448.16 (9415146.07, 19597750.24)	203.41 (133.58, 273.25)
2042	Female	14905938.44 (9284610.52, 20527266.36)	204.83 (129.42, 280.25)
2043	Female	15309015.54 (9123446.72, 21494584.37)	206.23 (125.05, 287.41)
2044	Female	15719378.95 (8932640.04, 22506117.86)	207.6 (120.48, 294.71)
2045	Female	16136476.11 (8710293.74, 23562658.47)	208.95 (115.72, 302.18)
2022	Male	7669022.3 (7496403.8, 7841640.8)	185.44 (183.41, 187.47)
2023	Male	7875597.16 (7666868.21, 8084326.12)	185.87 (182.58, 189.16)
2024	Male	8090800.27 (7824459.47, 8357141.07)	186.27 (181.37, 191.18)
2025	Male	8311551.17 (7967598.58, 8655503.76)	186.65 (179.87, 193.43)
2026	Male	8535567.51 (8095852.68, 8975282.33)	187.02 (178.14, 195.91)

2027	Male	8759612.28 (8207325.66, 9311898.9)	187.4 (176.2, 198.6)
2028	Male	8986649 (8305986.11, 9667311.89)	187.75 (174.05, 201.45)
2029	Male	9221016.17 (8396289.16, 10045743.17)	188.07 (171.71, 204.44)
2030	Male	9459703.82 (8475422.18, 10443985.47)	188.37 (169.18, 207.56)
2031	Male	9700321.85 (8540902.87, 10859740.83)	188.66 (166.5, 210.83)
2032	Male	9939498.94 (8589345.14, 11289652.73)	188.95 (163.65, 214.24)
2033	Male	10179334.63 (8622691.24, 11735978.02)	189.19 (160.63, 217.76)
2034	Male	10424436.6 (8644664.03, 12204209.17)	189.41 (157.45, 221.36)
2035	Male	10672869.4 (8653170.21, 12692568.59)	189.59 (154.12, 225.07)
2036	Male	10922372.61 (8645756.65, 13198988.56)	189.77 (150.65, 228.88)
2037	Male	11169937.35 (8619419.12, 13720455.58)	189.92 (147.04, 232.79)
2038	Male	11417817.34 (8575838.23, 14259796.44)	190.03 (143.28, 236.77)
2039	Male	11670752.04 (8518230.39, 14823273.68)	190.1 (139.38, 240.82)
2040	Male	11926989.51 (8444657.52, 15409321.5)	190.16 (135.37, 244.94)
2041	Male	12183737.21 (8352382.88, 16015091.53)	190.19 (131.23, 249.15)
2042	Male	12437795.52 (8238493.73, 16637097.3)	190.19 (126.97, 253.42)
2043	Male	12691532.32 (8104418.68, 17278645.96)	190.16 (122.58, 257.74)
2044	Male	12949831.73 (7952830.25, 17946833.21)	190.1 (118.09, 262.11)
2045	Male	13210648.76 (7781681.99, 18639615.52)	190.02 (113.49, 266.55)

Notes: ASR: age-standardized rates; YLDs: years lived with disability.

Table S76. Prevalence and YLDs of Alzheimer's disease and other dementias in the worldwide of future forecasts using bayesian age-period-cohort model.

Year	Sex	Number	ASR
**Prevalence**			
2022	Both	59704324.88 (58489078.35, 60919571.42)	696.74 (682.56, 710.92)
2023	Both	62125545.81 (60534671.71, 63716419.9)	703.12 (685.12, 721.13)
2024	Both	64803278.59 (62631315.8, 66975241.38)	709.62 (685.83, 733.4)
2025	Both	67656345.42 (64713958.45, 70598732.39)	716.31 (685.16, 747.46)
2026	Both	70598260.06 (66706895.09, 74489625.03)	723.03 (683.18, 762.89)
2027	Both	73556457.17 (68541924.39, 78570989.95)	729.63 (679.89, 779.38)
2028	Both	76681315.11 (70361580.38, 83001049.84)	736.26 (675.57, 796.94)
2029	Both	80091146.85 (72265827.99, 87916465.71)	743.05 (670.44, 815.66)
2030	Both	83708159.31 (74169179.18, 93247139.44)	750.06 (664.58, 835.55)
2031	Both	87433854.64 (75968517.83, 98899191.45)	757.09 (657.79, 856.38)
2032	Both	91172598.63 (77566747.85, 104778449.41)	763.93 (649.91, 877.96)
2033	Both	95064239.79 (79076149.86, 111052329.71)	770.79 (641.13, 900.45)
2034	Both	99232929.16 (80583932.26, 117881926.05)	777.82 (631.6, 924.04)
2035	Both	103602817.63 (82006496.68, 125199138.58)	785.09 (621.38, 948.8)
2036	Both	108065612.9 (83237484.74, 132893741.06)	792.39 (610.26, 974.51)
2037	Both	112512789.47 (84176339.46, 140849239.48)	799.53 (598.07, 1000.99)
2038	Both	117085150.48 (84920160.58, 149250140.38)	806.71 (584.97, 1028.45)
2039	Both	121915241.01 (85546118.55, 158284363.46)	814.07 (571.07, 1057.08)
2040	Both	126935911.16 (85980937.48, 167890884.84)	821.69 (556.39, 1087)
2041	Both	132044628.84 (86130401.81, 177958855.87)	829.44 (540.79, 1118.1)
2042	Both	137135862.08 (85904747.83, 188366976.32)	837.17 (524.12, 1150.22)
2043	Both	142356395.27 (85384359.6, 199328430.93)	845.04 (506.49, 1183.6)

2044	Both	147835785.8 (84622835.12, 211048736.48)	853.15 (487.91, 1218.4)
2045	Both	153501911.15 (83546590.3, 223457232.01)	861.57 (468.39, 1254.75)
2022	Female	37714053.48 (36938457.48, 38489649.48)	772.77 (763.42, 782.12)
2023	Female	39225253.65 (38211945.03, 40238562.26)	780.15 (764.82, 795.48)
2024	Female	40895016.26 (39514157.02, 42275875.51)	787.7 (764.66, 810.74)
2025	Female	42677485.02 (40809329.58, 44545640.47)	795.45 (763.35, 827.54)
2026	Female	44521179.13 (42052713.5, 46989644.75)	803.13 (760.8, 845.46)
2027	Female	46383444.07 (43204148.89, 49562739.25)	810.54 (756.85, 864.22)
2028	Female	48355479.56 (44349785.73, 52361173.39)	817.92 (751.84, 884)
2029	Female	50508810.24 (45549597.6, 55468022.89)	825.46 (745.99, 904.94)
2030	Female	52798709.83 (46753428.22, 58843991.43)	833.17 (739.33, 927.02)
2031	Female	55164919.31 (47897597.26, 62432241.35)	840.76 (731.61, 949.92)
2032	Female	57549188.59 (48922233.46, 66176143.72)	847.99 (722.61, 973.38)
2033	Female	60037562.08 (49895616.62, 70179507.54)	855.13 (712.6, 997.66)
2034	Female	62705408.27 (50869706.24, 74541110.29)	862.36 (701.79, 1022.94)
2035	Female	65506187.12 (51792420.87, 79219953.38)	869.74 (690.2, 1049.28)
2036	Female	68370757.26 (52595612.61, 84145901.91)	876.99 (677.62, 1076.36)
2037	Female	71230161.12 (53215088.44, 89245233.81)	883.9 (663.87, 1103.94)
2038	Female	74170260.39 (53709337.87, 94631182.91)	890.69 (649.14, 1132.23)
2039	Female	77273075.42 (54125477.67, 100420673.17)	897.53 (633.6, 1161.45)
2040	Female	80496663.35 (54417418.04, 106575908.65)	904.47 (617.29, 1191.65)
2041	Female	83775255.76 (54524910.75, 113025600.77)	911.36 (600.07, 1222.65)
2042	Female	87041527.02 (54390819.35, 119692234.69)	918.03 (581.82, 1254.23)
2043	Female	90383209.03 (54062655.28, 126703762.78)	924.61 (562.67, 1286.56)
2044	Female	93882125.23 (53574382.65, 134189867.81)	931.22 (542.68, 1319.76)
2045	Female	97497088.46 (52882316.93, 142111859.99)	937.91 (521.91, 1353.92)

2022	Male	21990271.4 (21550620.87, 22429921.94)	593.5 (586.51, 600.49)
2023	Male	22900292.16 (22322726.68, 23477857.64)	598.8 (587.26, 610.34)
2024	Male	23908262.33 (23117158.78, 24699365.87)	604.18 (586.81, 621.56)
2025	Male	24978860.4 (23904628.87, 26053091.92)	609.7 (585.48, 633.92)
2026	Male	26077080.93 (24654181.59, 27499980.28)	615.2 (583.25, 647.16)
2027	Male	27173013.1 (25337775.5, 29008250.7)	620.56 (580.02, 661.09)
2028	Male	28325835.55 (26011794.65, 30639876.45)	625.87 (575.97, 675.77)
2029	Male	29582336.61 (26716230.39, 32448442.82)	631.26 (571.26, 691.27)
2030	Male	30909449.48 (27415750.96, 34403148.01)	636.78 (565.94, 707.62)
2031	Male	32268935.33 (28070920.57, 36466950.1)	642.26 (559.88, 724.63)
2032	Male	33623410.04 (28644514.39, 38602305.69)	647.51 (552.89, 742.12)
2033	Male	35026677.71 (29180533.24, 40872822.17)	652.68 (545.15, 760.2)
2034	Male	36527520.89 (29714226.02, 43340815.76)	657.89 (536.78, 779)
2035	Male	38096630.51 (30214075.81, 45979185.2)	663.19 (527.82, 798.55)
2036	Male	39694855.64 (30641872.14, 48747839.15)	668.41 (518.14, 818.69)
2037	Male	41282628.35 (30961251.03, 51604005.67)	673.39 (507.59, 839.18)
2038	Male	42914890.09 (31210822.71, 54618957.47)	678.26 (496.32, 860.21)
2039	Male	44642165.59 (31420640.89, 57863690.29)	683.16 (484.42, 881.89)
2040	Male	46439247.81 (31563519.44, 61314976.19)	688.11 (471.95, 904.28)
2041	Male	48269373.08 (31605491.06, 64933255.1)	693.01 (458.8, 927.22)
2042	Male	50094335.05 (31513928.47, 68674741.63)	697.69 (444.86, 950.53)
2043	Male	51973186.24 (31321704.32, 72624668.15)	702.33 (430.26, 974.41)
2044	Male	53953660.57 (31048452.46, 76858868.67)	707 (415.06, 998.94)
2045	Male	56004822.69 (30664273.36, 81345372.01)	711.7 (399.27, 1024.14)
**YLDs**			
2022	Both	12156638.06 (11910924.4, 12402351.72)	142.37 (139.49, 145.25)

2023	Both	12641987.07 (12318372.55, 12965601.59)	143.57 (139.9, 147.25)
2024	Both	13180525.26 (12736890.89, 13624159.64)	144.79 (139.92, 149.67)
2025	Both	13753983.9 (13151785.96, 14356181.84)	146.05 (139.66, 152.45)
2026	Both	14344781.75 (13547715.88, 15141847.63)	147.32 (139.14, 155.51)
2027	Both	14937680.65 (13910333.59, 15965027.72)	148.58 (138.36, 158.8)
2028	Both	15564808.2 (14270110.21, 16859506.19)	149.84 (137.37, 162.3)
2029	Both	16251026.56 (14648059.88, 17853993.23)	151.12 (136.21, 166.03)
2030	Both	16979862.31 (15026154.64, 18933569.98)	152.45 (134.91, 170)
2031	Both	17732246.65 (15384215.24, 20080278.05)	153.8 (133.43, 174.17)
2032	Both	18488486.36 (15702171.5, 21274801.23)	155.11 (131.73, 178.49)
2033	Both	19278802.28 (16004243.92, 22553360.63)	156.43 (129.85, 183)
2034	Both	20128775.68 (16308199.48, 23949351.89)	157.78 (127.82, 187.73)
2035	Both	21020967.16 (16595222.65, 25446711.66)	159.17 (125.64, 192.69)
2036	Both	21932847.68 (16843187.3, 27022508.05)	160.57 (123.29, 197.85)
2037	Both	22840514.05 (17030182.46, 28650845.65)	161.95 (120.73, 203.17)
2038	Both	23773172.84 (17176348.11, 30369997.56)	163.33 (117.98, 208.68)
2039	Both	24758879.1 (17298054.96, 32219703.24)	164.75 (115.07, 214.43)
2040	Both	25783304.54 (17379869.71, 34186739.37)	166.21 (111.99, 220.43)
2041	Both	26826058.01 (17402916.7, 36249199.32)	167.7 (108.74, 226.67)
2042	Both	27864848.94 (17348365.71, 38381332.17)	169.19 (105.27, 233.12)
2043	Both	28930184.29 (17232825.51, 40627543.06)	170.71 (101.61, 239.81)
2044	Both	30049157.36 (17067404.16, 43030910.56)	172.27 (97.75, 246.79)
2045	Both	31206809.37 (16836852.3, 45576766.44)	173.89 (93.7, 254.08)
2022	Female	7935712.48 (7774369.85, 8097055.11)	162.41 (160.45, 164.37)
2023	Female	8246712.5 (8034664.2, 8458760.8)	163.82 (160.59, 167.05)
2024	Female	8591479.59 (8301337.83, 8881621.35)	165.27 (160.41, 170.13)

2025	Female	8959333.26 (8566039.11, 9352627.41)	166.75 (159.99, 173.52)
2026	Female	9339309.96 (8819269.18, 9859350.75)	168.23 (159.31, 177.15)
2027	Female	9722119.11 (9052283.09, 10391955.13)	169.65 (158.35, 180.96)
2028	Female	10127719.09 (9283979.81, 10971458.36)	171.07 (157.16, 184.98)
2029	Female	10571745.12 (9527524.07, 11615966.17)	172.51 (155.8, 189.23)
2030	Female	11044512.97 (9772113.97, 12316911.96)	173.99 (154.27, 193.72)
2031	Female	11533881.63 (10004815.71, 13062947.56)	175.46 (152.53, 198.38)
2032	Female	12027497.36 (10212906.68, 13842088.03)	176.85 (150.53, 203.16)
2033	Female	12544396.88 (10411510.31, 14677283.45)	178.22 (148.32, 208.11)
2034	Female	13100741.18 (10611713.6, 15589768.76)	179.6 (145.94, 213.27)
2035	Female	13685678.57 (10801623, 16569734.14)	181.02 (143.41, 218.63)
2036	Female	14284302.24 (10966582.14, 17602022.34)	182.41 (140.67, 224.15)
2037	Female	14881037.76 (11092260.1, 18669815.43)	183.73 (137.7, 229.77)
2038	Female	15494257.65 (11191218.45, 19797296.85)	185.03 (134.52, 235.53)
2039	Female	16141949.86 (11273880, 21010019.71)	186.33 (131.18, 241.48)
2040	Female	16814910.06 (11330276.44, 22299543.68)	187.65 (127.68, 247.62)
2041	Female	17499310.85 (11347755.09, 23650866.61)	188.96 (123.99, 253.92)
2042	Female	18180401.2 (11313941.72, 25046860.67)	190.23 (120.1, 260.35)
2043	Female	18877038.7 (11239082.07, 26514995.33)	191.47 (116.03, 266.91)
2044	Female	19606986.52 (11130596.43, 28083376.62)	192.72 (111.78, 273.65)
2045	Female	20361465.07 (10979212.49, 29743717.65)	193.98 (107.37, 280.58)
2022	Male	4220925.58 (4136554.55, 4305296.61)	115.11 (113.75, 116.48)
2023	Male	4395274.58 (4283708.35, 4506840.8)	116.11 (113.84, 118.37)
2024	Male	4589045.67 (4435553.06, 4742538.29)	117.11 (113.7, 120.53)
2025	Male	4794650.65 (4585746.86, 5003554.44)	118.14 (113.37, 122.91)
2026	Male	5005471.79 (4728446.7, 5282496.88)	119.17 (112.89, 125.46)

2027	Male	5215561.54 (4858050.5, 5573072.59)	120.18 (112.2, 128.15)
2028	Male	5437089.12 (4986130.4, 5888047.83)	121.18 (111.36, 130.99)
2029	Male	5679281.44 (5120535.82, 6238027.06)	122.19 (110.39, 133.98)
2030	Male	5935349.35 (5254040.67, 6616658.02)	123.22 (109.3, 137.14)
2031	Male	6198365.01 (5379399.53, 7017330.49)	124.25 (108.07, 140.43)
2032	Male	6460989.01 (5489264.81, 7432713.2)	125.24 (106.66, 143.83)
2033	Male	6734405.4 (5592733.61, 7876077.18)	126.22 (105.11, 147.34)
2034	Male	7028034.5 (5696485.88, 8359583.12)	127.21 (103.43, 150.98)
2035	Male	7335288.58 (5793599.64, 8876977.52)	128.21 (101.64, 154.77)
2036	Male	7648545.44 (5876605.17, 9420485.7)	129.19 (99.71, 158.68)
2037	Male	7959476.29 (5937922.36, 9981030.22)	130.14 (97.61, 162.67)
2038	Male	8278915.19 (5985129.66, 10572700.72)	131.06 (95.37, 166.75)
2039	Male	8616929.24 (6024174.96, 11209683.53)	131.98 (93.01, 170.96)
2040	Male	8968394.48 (6049593.27, 11887195.69)	132.91 (90.53, 175.29)
2041	Male	9326747.16 (6055161.61, 12598332.71)	133.83 (87.92, 179.74)
2042	Male	9684447.74 (6034423.99, 13334471.5)	134.71 (85.16, 184.27)
2043	Male	10053145.59 (5993743.44, 14112547.74)	135.59 (82.27, 188.9)
2044	Male	10442170.84 (5936807.73, 14947533.94)	136.46 (79.27, 193.65)
2045	Male	10845344.3 (5857639.81, 15833048.79)	137.33 (76.14, 198.53)

Notes: ASR: age-standardized rates; YLDs: years lived with disability.

Table S77. Prevalence and YLDs of Parkinson's disease in the worldwide of future forecasts using bayesian age-period-cohort model.

Year	Sex	Number	ASR
**Prevalence**			
2022	Both	12599319.27 (11996258.07, 13202380.47)	226.22 (215.4, 237.05)
2023	Both	13135809.46 (12448609.44, 13823009.47)	229.05 (217.07, 241.03)
2024	Both	13707585.89 (12892982.96, 14522188.82)	231.87 (218.09, 245.65)
2025	Both	14306865.27 (13317848.1, 15295882.44)	234.68 (218.46, 250.9)
2026	Both	14925573.39 (13714209.98, 16136936.81)	237.49 (218.22, 256.77)
2027	Both	15556088.44 (14074849.25, 17037327.63)	240.31 (217.43, 263.19)
2028	Both	16210030.72 (14411109.77, 18008951.66)	243.1 (216.12, 270.08)
2029	Both	16900320.27 (14733667.38, 19066973.16)	245.89 (214.36, 277.42)
2030	Both	17617369.64 (15032017.1, 20202722.18)	248.66 (212.17, 285.15)
2031	Both	18348065.45 (15292520.66, 21403610.25)	251.37 (209.51, 293.24)
2032	Both	19082615.75 (15504938.6, 22660292.9)	254.04 (206.41, 301.68)
2033	Both	19832254.58 (15677434.02, 23987075.14)	256.67 (202.89, 310.46)
2034	Both	20612364.69 (15819693.21, 25405036.17)	259.28 (198.98, 319.59)
2035	Both	21414873.85 (15921881.43, 26907866.28)	261.87 (194.68, 329.06)
2036	Both	22224334.56 (15968997.24, 28479671.89)	264.38 (189.94, 338.81)
2037	Both	23031012.12 (15951323.23, 30110701.01)	266.81 (184.76, 348.86)
2038	Both	23848402.62 (15876724.2, 31820081.03)	269.22 (179.19, 359.25)
2039	Both	24693559.13 (15753333.26, 33633785.01)	271.62 (173.23, 370)
2040	Both	25559550.53 (15572145, 35546956.06)	274.02 (166.89, 381.15)
2041	Both	26430619.02 (15319144.98, 37542093.05)	276.38 (160.12, 392.64)
2042	Both	27296717.4 (14985020.9, 39608413.89)	278.7 (152.91, 404.49)
2043	Both	28173561.33 (14576133.34, 41770989.33)	281.04 (145.3, 416.77)

2044	Both	29077504.49 (14096567.9, 44058441.09)	283.41 (137.28, 429.54)
2045	Both	29999777.3 (13536232.8, 46463321.79)	285.82 (128.83, 442.81)
2022	Female	5662267.22 (5430240.85, 5894293.59)	185.68 (181.98, 189.39)
2023	Female	5898945.37 (5631290.48, 6166600.26)	187.85 (182.63, 193.08)
2024	Female	6151104.76 (5828796.08, 6473413.44)	190.01 (182.75, 197.27)
2025	Female	6415875.18 (6018621.74, 6813128.61)	192.15 (182.46, 201.85)
2026	Female	6690852.5 (6198171.31, 7183533.69)	194.31 (181.82, 206.79)
2027	Female	6973086.12 (6364700.35, 7581471.88)	196.48 (180.87, 212.08)
2028	Female	7266796.04 (6522472.62, 8011119.46)	198.62 (179.6, 217.65)
2029	Female	7577013.86 (6675645.96, 8478381.76)	200.74 (178.02, 223.46)
2030	Female	7900005.52 (6819976.1, 8980034.95)	202.83 (176.14, 229.52)
2031	Female	8230894.57 (6950078.65, 9511710.5)	204.87 (173.95, 235.78)
2032	Female	8565696.86 (7061503.59, 10069890.13)	206.87 (171.45, 242.28)
2033	Female	8908377.3 (7157029.27, 10659725.33)	208.81 (168.65, 248.97)
2034	Female	9265028.39 (7240455.74, 11289601.04)	210.7 (165.55, 255.85)
2035	Female	9632260.19 (7307546.59, 11956973.79)	212.54 (162.17, 262.91)
2036	Female	10003650.49 (7351838.67, 12655462.32)	214.29 (158.48, 270.1)
2037	Female	10375079.19 (7368995.73, 13381162.65)	215.95 (154.47, 277.44)
2038	Female	10751516.86 (7361913.55, 14141120.18)	217.55 (150.18, 284.91)
2039	Female	11140082.44 (7334143.31, 14946021.57)	219.09 (145.63, 292.54)
2040	Female	11538075.31 (7281992.62, 15794158.01)	220.58 (140.82, 300.33)
2041	Female	11938828.13 (7199346.73, 16678309.54)	221.98 (135.73, 308.23)
2042	Female	12338161.14 (7082114.54, 17594207.74)	223.3 (130.37, 316.24)
2043	Female	12742120.61 (6932879.92, 18551361.3)	224.57 (124.76, 324.38)
2044	Female	13157681.54 (6753711.14, 19561651.94)	225.8 (118.93, 332.67)
2045	Female	13581753.74 (6540631.11, 20622876.37)	226.98 (112.87, 341.1)

2022	Male	6937052.05 (6566017.21, 7308086.88)	275.61 (268.67, 282.55)
2023	Male	7236864.09 (6817318.96, 7656409.22)	279.19 (269.8, 288.57)
2024	Male	7556481.13 (7064186.88, 8048775.38)	282.73 (270.05, 295.41)
2025	Male	7890990.09 (7299226.36, 8482753.83)	286.25 (269.58, 302.91)
2026	Male	8234720.9 (7516038.67, 8953403.12)	289.73 (268.47, 310.98)
2027	Male	8583002.33 (7710148.9, 9455855.75)	293.18 (266.79, 319.57)
2028	Male	8943234.68 (7888637.15, 9997832.2)	296.57 (264.55, 328.6)
2029	Male	9323306.41 (8058021.42, 10588591.4)	299.91 (261.78, 338.03)
2030	Male	9717364.11 (8212041, 11222687.22)	303.16 (258.49, 347.84)
2031	Male	10117170.88 (8342442, 11891899.75)	306.28 (254.63, 357.94)
2032	Male	10516918.89 (8443435.01, 12590402.77)	309.28 (250.22, 368.33)
2033	Male	10923877.27 (8520404.74, 13327349.8)	312.14 (245.28, 379)
2034	Male	11347336.3 (8579237.47, 14115435.13)	314.89 (239.85, 389.94)
2035	Male	11782613.66 (8614334.83, 14950892.49)	317.53 (233.93, 401.13)
2036	Male	12220684.07 (8617158.57, 15824209.57)	319.96 (227.45, 412.46)
2037	Male	12655932.93 (8582327.5, 16729538.36)	322.19 (220.46, 423.93)
2038	Male	13096885.75 (8514810.65, 17678960.85)	324.27 (212.98, 435.55)
2039	Male	13553476.69 (8419189.95, 18687763.44)	326.22 (205.07, 447.36)
2040	Male	14021475.22 (8290152.38, 19752798.05)	328.05 (196.74, 459.35)
2041	Male	14491790.88 (8119798.24, 20863783.52)	329.68 (187.95, 471.41)
2042	Male	14958556.26 (7902906.36, 22014206.15)	331.14 (178.73, 483.54)
2043	Male	15431440.72 (7643253.42, 23219628.02)	332.46 (169.13, 495.8)
2044	Male	15919822.95 (7342856.75, 24496789.15)	333.68 (159.17, 508.19)
2045	Male	16418023.56 (6995601.69, 25840445.42)	334.78 (148.87, 520.7)
**YLDs**			
2022	Both	1781962.54 (1701479.56, 1862445.53)	20.12 (19.21, 21.03)

2023	Both	1855368.64 (1762510.3, 1948226.98)	20.35 (19.33, 21.37)
2024	Both	1933284.65 (1821562.38, 2045006.92)	20.58 (19.39, 21.77)
2025	Both	2014720.44 (1877263.71, 2152177.17)	20.8 (19.38, 22.22)
2026	Both	2098728.55 (1928650.84, 2268806.26)	21.03 (19.33, 22.74)
2027	Both	2184245.42 (1974775.58, 2393715.26)	21.26 (19.22, 23.3)
2028	Both	2272633.49 (2017054.91, 2528212.08)	21.48 (19.07, 23.9)
2029	Both	2365552.87 (2056881.68, 2674224.06)	21.7 (18.87, 24.54)
2030	Both	2461802.92 (2092925.8, 2830680.04)	21.92 (18.64, 25.21)
2031	Both	2559875.54 (2123560.39, 2996190.7)	22.14 (18.36, 25.92)
2032	Both	2658402.05 (2147339.16, 3169464.94)	22.35 (18.05, 26.65)
2033	Both	2758632.88 (2165168.66, 3352097.1)	22.56 (17.71, 27.42)
2034	Both	2862515.69 (2178263.18, 3546768.19)	22.77 (17.32, 28.21)
2035	Both	2969116.55 (2185428.04, 3752805.06)	22.97 (16.9, 29.04)
2036	Both	3076770.48 (2184945.5, 3968595.46)	23.17 (16.45, 29.89)
2037	Both	3184111.9 (2175432.69, 4192791.11)	23.36 (15.96, 30.77)
2038	Both	3292658.74 (2157737.21, 4427580.26)	23.56 (15.43, 31.68)
2039	Both	3404518.81 (2132818.43, 4676219.18)	23.75 (14.87, 32.62)
2040	Both	3518926.73 (2099582.07, 4938271.4)	23.94 (14.27, 33.6)
2041	Both	3634277.79 (2056444.83, 5212110.76)	24.13 (13.64, 34.61)
2042	Both	3749152.46 (2002060.75, 5496244.17)	24.31 (12.97, 35.66)
2043	Both	3865341.84 (1937063.78, 5793619.91)	24.5 (12.27, 36.74)
2044	Both	3984819.81 (1861844.53, 6107795.09)	24.7 (11.52, 37.87)
2045	Both	4106566.1 (1775125.62, 6438006.58)	24.89 (10.74, 39.05)
2022	Female	791306.5 (761164.69, 821448.3)	16.38 (16.07, 16.69)
2023	Female	823126.17 (787822.42, 858429.92)	16.55 (16.1, 17)
2024	Female	856872.96 (813624.15, 900121.78)	16.71 (16.08, 17.35)

2025	Female	892189.5 (838115.87, 946263.13)	16.88 (16.02, 17.73)
2026	Female	928863.3 (861104.67, 996621.93)	17.05 (15.94, 18.15)
2027	Female	966472.43 (882213.68, 1050731.18)	17.21 (15.83, 18.6)
2028	Female	1005453.79 (901931.8, 1108975.77)	17.38 (15.69, 19.07)
2029	Female	1046424.34 (920779.94, 1172068.73)	17.54 (15.52, 19.56)
2030	Female	1088931.6 (938235.67, 1239627.53)	17.7 (15.33, 20.07)
2031	Female	1132502.67 (953727.38, 1311277.95)	17.85 (15.11, 20.6)
2032	Female	1176563.58 (966609.62, 1386517.54)	18 (14.86, 21.14)
2033	Female	1221476.82 (977135.81, 1465817.83)	18.15 (14.59, 21.71)
2034	Female	1267975.54 (985764.38, 1550186.7)	18.29 (14.29, 22.29)
2035	Female	1315694.04 (992010.29, 1639377.79)	18.42 (13.97, 22.88)
2036	Female	1364033.89 (995204.51, 1732863.27)	18.55 (13.62, 23.49)
2037	Female	1412386.35 (994707.52, 1830065.18)	18.67 (13.24, 24.11)
2038	Female	1461230.58 (990789.69, 1931671.48)	18.79 (12.85, 24.74)
2039	Female	1511408.53 (983865.08, 2038951.98)	18.9 (12.42, 25.38)
2040	Female	1562670.82 (973529.65, 2151811.99)	19.01 (11.98, 26.03)
2041	Female	1614432.99 (959156.66, 2269709.32)	19.11 (11.52, 26.69)
2042	Female	1666057.66 (940129.51, 2391985.82)	19.2 (11.03, 27.37)
2043	Female	1718158.54 (916686.37, 2519630.7)	19.29 (10.52, 28.06)
2044	Female	1771545.25 (889045.18, 2654045.32)	19.37 (10, 28.75)
2045	Female	1825922.12 (856750.15, 2795094.1)	19.46 (9.45, 29.46)
2022	Male	990656.05 (940314.86, 1040997.23)	24.65 (24.05, 25.25)
2023	Male	1032242.47 (974687.88, 1089797.06)	24.95 (24.12, 25.78)
2024	Male	1076411.69 (1007938.23, 1144885.14)	25.25 (24.1, 26.39)
2025	Male	1122530.94 (1039147.83, 1205914.05)	25.54 (24.02, 27.05)
2026	Male	1169865.25 (1067546.17, 1272184.33)	25.82 (23.88, 27.77)

2027	Male	1217772.99 (1092561.89, 1342984.08)	26.11 (23.69, 28.53)
2028	Male	1267179.71 (1115123.1, 1419236.31)	26.39 (23.45, 29.32)
2029	Male	1319128.53 (1136101.74, 1502155.33)	26.66 (23.16, 30.16)
2030	Male	1372871.32 (1154690.13, 1591052.51)	26.92 (22.82, 31.02)
2031	Male	1427372.88 (1169833.01, 1684912.74)	27.18 (22.43, 31.92)
2032	Male	1481838.47 (1180729.55, 1782947.4)	27.42 (22, 32.84)
2033	Male	1537156.06 (1188032.85, 1886279.27)	27.65 (21.51, 33.78)
2034	Male	1594540.15 (1192498.8, 1996581.5)	27.86 (20.98, 34.74)
2035	Male	1653422.51 (1193417.75, 2113427.26)	28.07 (20.41, 35.73)
2036	Male	1712736.59 (1189740.99, 2235732.19)	28.26 (19.79, 36.73)
2037	Male	1771725.55 (1180725.17, 2362725.93)	28.43 (19.12, 37.75)
2038	Male	1831428.16 (1166947.53, 2495908.78)	28.59 (18.41, 38.77)
2039	Male	1893110.28 (1148953.35, 2637267.21)	28.74 (17.67, 39.82)
2040	Male	1956255.91 (1126052.42, 2786459.41)	28.88 (16.88, 40.88)
2041	Male	2019844.8 (1097288.17, 2942401.44)	29 (16.06, 41.94)
2042	Male	2083094.8 (1061931.24, 3104258.36)	29.11 (15.2, 43.02)
2043	Male	2147183.31 (1020377.41, 3273989.21)	29.21 (14.31, 44.11)
2044	Male	2213274.56 (972799.35, 3453749.77)	29.3 (13.38, 45.21)
2045	Male	2280643.98 (918375.47, 3642912.48)	29.37 (12.43, 46.32)

Notes: ASR: age-standardized rates; YLDs: years lived with disability.

Table S78. Prevalence and YLDs of upper digestive system diseases in the worldwide of future forecasts using bayesian age-period-cohort model.

Year	Sex	Number	ASR
**Prevalence**			
2022	Both	895368156.53 (856739703.41, 933996609.65)	10364.1 (9916.93, 10811.28)
2023	Both	910579764.9 (867892439.1, 953267090.71)	10385.78 (9898.84, 10872.71)
2024	Both	925760313.18 (876700946.22, 974819680.14)	10406.25 (9854.71, 10957.79)
2025	Both	940875923.28 (883048623.98, 998703222.57)	10425.71 (9784.83, 11066.58)
2026	Both	955827114.58 (886950388.8, 1024703840.36)	10443.37 (9690.71, 11196.04)
2027	Both	970600104.79 (888574324.35, 1052625885.22)	10459.33 (9575.27, 11343.4)
2028	Both	985270715.64 (888205992.63, 1082335438.65)	10474.22 (9442.16, 11506.27)
2029	Both	999918377.84 (886066174.65, 1113770581.03)	10488.54 (9294.08, 11683)
2030	Both	1014502697.64 (882204790.11, 1146800605.17)	10502.36 (9132.52, 11872.19)
2031	Both	1028914082.5 (876570275.64, 1181257889.35)	10514.71 (8957.56, 12071.86)
2032	Both	1043166105.19 (869204519.3, 1217127691.09)	10525.76 (8770.08, 12281.44)
2033	Both	1057321291.4 (860220829.19, 1254421753.61)	10536.75 (8572.09, 12501.41)
2034	Both	1071509961.71 (849751886.8, 1293268036.63)	10548.32 (8364.71, 12731.93)
2035	Both	1085692771.37 (837761395.41, 1333624147.32)	10560.36 (8148.11, 12972.61)
2036	Both	1099732033.95 (824119206.23, 1375344861.67)	10571.7 (7921.44, 13221.95)
2037	Both	1113658533.96 (808825080.33, 1418491987.59)	10582.5 (7684.86, 13480.15)
2038	Both	1127530393.79 (791934324.97, 1463126462.62)	10594.42 (7439.92, 13748.92)
2039	Both	1141523525.76 (773558727.34, 1509488324.18)	10608.12 (7187.18, 14029.06)
2040	Both	1155579441.87 (753619161.86, 1557539721.88)	10623.34 (6926.29, 14320.38)
2041	Both	1169520767 (731947549.94, 1607093984.06)	10638.68 (6656.06, 14621.31)
2042	Both	1183362959.29 (708504953.01, 1658220965.56)	10654.33 (6376.31, 14932.36)
2043	Both	1197197124.92 (683331027.83, 1711063222.01)	10672.1 (6088.11, 15256.09)

2044	Both	1211190806.98 (656482164.37, 1765899449.6)	10692.71 (5791.62, 15593.79)
2045	Both	1225292067.91 (627863908.25, 1822720227.56)	10715.82 (5486.17, 15945.47)
2022	Female	473910150.91 (453016170.58, 494804131.24)	10812.02 (10614.45, 11009.6)
2023	Female	482220689.43 (459162658.48, 505278720.37)	10837.88 (10557.18, 11118.57)
2024	Female	490505451.02 (464059764.22, 516951137.82)	10862.01 (10472.25, 11251.76)
2025	Female	498754067.95 (467646359.17, 529861776.72)	10884.51 (10366.06, 11402.96)
2026	Female	506925837.17 (469936199.87, 543915474.47)	10904.56 (10241.19, 11567.93)
2027	Female	515016591.62 (471018067.19, 559015116.04)	10922.1 (10099.54, 11744.66)
2028	Female	523052608.71 (471028140.19, 575077077.23)	10937.51 (9943.29, 11931.73)
2029	Female	531065361.24 (470073133.42, 592057589.06)	10951.14 (9773.94, 12128.34)
2030	Female	539039904.21 (468185418.55, 609894389.87)	10962.96 (9592.22, 12333.69)
2031	Female	546929820.27 (465348185.14, 628511455.41)	10971.94 (9397.74, 12546.15)
2032	Female	554743206.25 (461584343.26, 647902069.24)	10978.14 (9190.96, 12765.31)
2033	Female	562500180.43 (456942110.87, 668058249.99)	10982.54 (8973.55, 12991.52)
2034	Female	570255163.98 (451479760.38, 689030567.58)	10985.6 (8746.47, 13224.72)
2035	Female	577992469.28 (445182844.8, 710802093.77)	10987.12 (8509.94, 13464.29)
2036	Female	585651843.85 (437992874.98, 733310812.73)	10985.87 (8263.25, 13708.49)
2037	Female	593250827.55 (429910762.57, 756590892.53)	10981.91 (8006.72, 13957.1)
2038	Female	600811143.75 (420958555.96, 780663731.55)	10976.61 (7741.96, 14211.25)
2039	Female	608410630.1 (411184777.27, 805636482.92)	10970.47 (7469.75, 14471.19)
2040	Female	616024479.37 (400552828.34, 831496130.41)	10963.14 (7190.11, 14736.17)
2041	Female	623572515.83 (388983679.42, 858161352.25)	10953.19 (6902.28, 15004.11)
2042	Female	631064943.38 (376458213.57, 885671673.19)	10940.67 (6606.51, 15274.83)
2043	Female	638540804.27 (362991409.63, 914090198.91)	10927.12 (6304.25, 15550)
2044	Female	646075887.9 (348606014.68, 943545761.12)	10913.1 (5996.14, 15830.06)
2045	Female	653647726.49 (333256076.44, 974039376.54)	10898.16 (5682.13, 16114.19)

2022	Male	421458005.62 (403723532.82, 439192478.41)	9903.28 (9728.96, 10077.6)
2023	Male	428359075.48 (408729780.62, 447988370.34)	9919.79 (9670.06, 10169.53)
2024	Male	435254862.16 (412641181.99, 457868542.32)	9934.93 (9586.5, 10283.35)
2025	Male	442121855.33 (415402264.8, 468841445.85)	9948.72 (9484.1, 10413.35)
2026	Male	448901277.41 (417014188.93, 480788365.89)	9960.2 (9364.91, 10555.48)
2027	Male	455583513.17 (417556257.16, 493610769.18)	9969.32 (9230.67, 10707.98)
2028	Male	462218106.93 (417177852.44, 507258361.41)	9976.75 (9083.65, 10869.85)
2029	Male	468853016.6 (415993041.23, 521712991.97)	9982.95 (8925.36, 11040.54)
2030	Male	475462793.43 (414019371.56, 536906215.3)	9987.82 (8756.4, 11219.25)
2031	Male	481984262.22 (411222090.5, 552746433.94)	9990.21 (8576.18, 11404.25)
2032	Male	488422898.94 (407620176.03, 569225621.85)	9990.15 (8385.12, 11595.19)
2033	Male	494821110.97 (403278718.32, 586363503.61)	9988.92 (8185.05, 11792.79)
2034	Male	501254797.74 (398272126.42, 604237469.06)	9987.12 (7976.99, 11997.24)
2035	Male	507700302.08 (392578550.61, 622822053.55)	9984.49 (7761.05, 12207.93)
2036	Male	514080190.09 (386126331.25, 642034048.94)	9979.66 (7536.35, 12422.97)
2037	Male	520407706.41 (378914317.76, 661901095.06)	9972.69 (7303.15, 12642.22)
2038	Male	526719250.04 (370975769.02, 682462731.07)	9965.2 (7063.19, 12867.21)
2039	Male	533112895.66 (362373950.07, 703851841.25)	9957.85 (6817.26, 13098.43)
2040	Male	539554962.49 (353066333.52, 726043591.46)	9950.19 (6565.24, 13335.14)
2041	Male	545948251.16 (342963870.53, 748932631.8)	9940.65 (6306.2, 13575.11)
2042	Male	552298015.91 (332046739.44, 772549292.38)	9929.26 (6040.29, 13818.24)
2043	Male	558656320.65 (320339618.2, 796973023.1)	9917.8 (5769.03, 14066.56)
2044	Male	565114919.08 (307876149.69, 822353688.48)	9906.89 (5493.01, 14320.77)
2045	Male	571644341.42 (294607831.81, 848680851.02)	9896.06 (5212.05, 14580.06)
**YLDs**			
2022	Both	8684521.27 (8329886.5, 9039156.04)	100.62 (96.51, 104.73)

2023	Both	8821237.53 (8427268.36, 9215206.7)	100.7 (96.2, 105.2)
2024	Both	8957423.11 (8501479.04, 9413367.17)	100.77 (95.63, 105.9)
2025	Both	9092829.66 (8551908.07, 9633751.25)	100.83 (94.83, 106.83)
2026	Both	9226681.39 (8579212.68, 9874150.09)	100.88 (93.79, 107.96)
2027	Both	9358927.88 (8585241.12, 10132614.63)	100.92 (92.57, 109.27)
2028	Both	9489954.58 (8572444.04, 10407465.12)	100.95 (91.18, 110.72)
2029	Both	9620453.62 (8542901.22, 10698006.02)	100.98 (89.66, 112.3)
2030	Both	9750131.95 (8497202.98, 11003060.91)	101.01 (88.02, 114)
2031	Both	9878131.69 (8435050.39, 11321212.98)	101.02 (86.25, 115.79)
2032	Both	10004673.85 (8356932.88, 11652414.82)	101.03 (84.38, 117.69)
2033	Both	10130006.9 (8263621.48, 11996392.31)	101.04 (82.41, 119.68)
2034	Both	10255248.4 (8156309.01, 12354187.78)	101.06 (80.35, 121.76)
2035	Both	10380129.08 (8034785.56, 12725472.61)	101.08 (78.22, 123.94)
2036	Both	10503519.55 (7898006.64, 13109032.47)	101.1 (75.99, 126.2)
2037	Both	10625806.17 (7746083.3, 13505529.05)	101.11 (73.68, 128.55)
2038	Both	10747206.57 (7579304.82, 13915108.32)	101.14 (71.29, 130.99)
2039	Both	10869229.01 (7398673.99, 14339784.02)	101.18 (68.83, 133.53)
2040	Both	10991443.45 (7203599.16, 14779287.75)	101.23 (66.29, 136.17)
2041	Both	11112352.18 (6992681.13, 15232023.22)	101.29 (63.68, 138.9)
2042	Both	11232221.76 (6765694.36, 15698749.17)	101.35 (60.98, 141.72)
2043	Both	11351502.58 (6522802.39, 16180202.76)	101.43 (58.21, 144.65)
2044	Both	11471635.99 (6264543.87, 16678728.11)	101.53 (55.36, 147.71)
2045	Both	11592286.65 (5990212.61, 17194360.69)	101.66 (52.43, 150.89)
2022	Female	4691859.36 (4486602.31, 4897116.4)	107.22 (105.3, 109.15)
2023	Female	4767334.17 (4540931.09, 4993737.24)	107.32 (104.57, 110.07)
2024	Female	4842428.75 (4582868.38, 5101989.12)	107.4 (103.57, 111.22)

2025	Female	4917054.82 (4611914.67, 5222194.97)	107.47 (102.38, 112.55)
2026	Female	4990907.96 (4628372.15, 5353443.77)	107.51 (101.01, 114.02)
2027	Female	5063995 (4633203.94, 5494786.06)	107.54 (99.49, 115.6)
2028	Female	5136422.64 (4627609.21, 5645236.07)	107.56 (97.83, 117.28)
2029	Female	5208446.28 (4612612.54, 5804280.01)	107.56 (96.06, 119.06)
2030	Female	5279938.03 (4588582.26, 5971293.79)	107.54 (94.16, 120.91)
2031	Female	5350554.13 (4555489.02, 6145619.25)	107.5 (92.16, 122.84)
2032	Female	5420412.92 (4513620.2, 6327205.63)	107.44 (90.04, 124.83)
2033	Female	5489539.47 (4463301.46, 6515777.48)	107.36 (87.83, 126.89)
2034	Female	5558383.81 (4405058.07, 6711709.55)	107.27 (85.52, 129.02)
2035	Female	5626827.19 (4338813.99, 6914840.4)	107.16 (83.13, 131.2)
2036	Female	5694409.44 (4264141.2, 7124677.69)	107.03 (80.65, 133.42)
2037	Female	5761339.88 (4181115.73, 7341564.02)	106.88 (78.08, 135.68)
2038	Female	5827651.41 (4089829.68, 7565473.15)	106.72 (75.44, 138)
2039	Female	5893988.42 (3990708.23, 7797268.61)	106.54 (72.73, 140.36)
2040	Female	5960169.59 (3883484.47, 8036854.71)	106.36 (69.95, 142.76)
2041	Female	6025570.33 (3767544.93, 8283595.72)	106.15 (67.1, 145.19)
2042	Female	6090349.98 (3642790.86, 8537909.1)	105.92 (64.18, 147.65)
2043	Female	6154658.78 (3509251.32, 8800066.25)	105.67 (61.21, 150.14)
2044	Female	6219110.14 (3367126.21, 9071094.07)	105.42 (58.18, 152.66)
2045	Female	6283570.81 (3216080.02, 9351061.6)	105.16 (55.1, 155.22)
2022	Male	3992661.91 (3843284.19, 4142039.64)	93.86 (92.36, 95.36)
2023	Male	4053903.36 (3886337.27, 4221469.45)	93.91 (91.69, 96.13)
2024	Male	4114994.36 (3918610.66, 4311378.05)	93.95 (90.81, 97.1)
2025	Male	4175774.84 (3939993.4, 4411556.28)	93.99 (89.75, 98.22)
2026	Male	4235773.43 (3950840.53, 4520706.33)	94 (88.55, 99.45)

2027	Male	4294932.87 (3952037.18, 4637828.57)	94 (87.23, 100.78)
2028	Male	4353531.94 (3944834.83, 4762229.05)	93.99 (85.78, 102.2)
2029	Male	4412007.34 (3930288.68, 4893726.01)	93.97 (84.24, 103.7)
2030	Male	4470193.92 (3908620.72, 5031767.12)	93.94 (82.61, 105.27)
2031	Male	4527577.55 (3879561.38, 5175593.73)	93.89 (80.87, 106.9)
2032	Male	4584260.94 (3843312.68, 5325209.19)	93.82 (79.04, 108.59)
2033	Male	4640467.43 (3800320.02, 5480614.84)	93.74 (77.13, 110.35)
2034	Male	4696864.59 (3751250.94, 5642478.23)	93.66 (75.16, 112.16)
2035	Male	4753301.89 (3695971.57, 5810632.21)	93.57 (73.11, 114.03)
2036	Male	4809110.11 (3633865.44, 5984354.78)	93.46 (70.98, 115.94)
2037	Male	4864466.3 (3564967.57, 6163965.03)	93.34 (68.78, 117.9)
2038	Male	4919555.16 (3489475.14, 6349635.17)	93.21 (66.53, 119.9)
2039	Male	4975240.58 (3407965.76, 6542515.41)	93.09 (64.22, 121.96)
2040	Male	5031273.86 (3320114.68, 6742433.04)	92.96 (61.85, 124.07)
2041	Male	5086781.85 (3225136.21, 6948427.49)	92.81 (59.43, 126.2)
2042	Male	5141871.78 (3122903.51, 7160840.06)	92.65 (56.94, 128.37)
2043	Male	5196843.79 (3013551.07, 7380136.52)	92.49 (54.41, 130.58)
2044	Male	5252525.85 (2897417.66, 7607634.04)	92.34 (51.83, 132.84)
2045	Male	5308715.84 (2774132.59, 7843299.09)	92.18 (49.22, 135.15)

Notes: ASR: age-standardized rates; YLDs: years lived with disability.

Table S79. Prevalence and YLDs of inflammatory bowel disease in the worldwide of future forecasts using bayesian age-period-cohort model.

Year	Sex	Number	ASR
**Prevalence**			
2022	Both	3919666.1 (3663169.25, 4176162.94)	44.61 (41.69, 47.53)
2023	Both	3966755.61 (3672930.27, 4260580.96)	44.37 (41.09, 47.66)
2024	Both	4014048.91 (3660355.74, 4367742.09)	44.14 (40.25, 48.03)
2025	Both	4061060.57 (3626256.03, 4495865.11)	43.91 (39.21, 48.62)
2026	Both	4107781.9 (3573010.8, 4642553)	43.7 (38.01, 49.39)
2027	Both	4154164.92 (3502672.43, 4805657.4)	43.49 (36.67, 50.31)
2028	Both	4200864.2 (3417635.52, 4984092.89)	43.29 (35.22, 51.37)
2029	Both	4248515.26 (3319456.09, 5177574.43)	43.11 (33.68, 52.54)
2030	Both	4296832.5 (3208362.46, 5385302.53)	42.94 (32.06, 53.82)
2031	Both	4346009.52 (3084520.53, 5607498.51)	42.79 (30.37, 55.21)
2032	Both	4396025.46 (2947691.69, 5844359.22)	42.67 (28.61, 56.72)
2033	Both	4447256.67 (2798093.21, 6096420.14)	42.56 (26.77, 58.34)
2034	Both	4500547.36 (2635892.61, 6365202.11)	42.47 (24.87, 60.07)
2035	Both	4555880.26 (2460374.28, 6651386.25)	42.42 (22.9, 61.93)
2036	Both	4613601.87 (2270726.37, 6956477.38)	42.4 (20.86, 63.93)
2037	Both	4673752.84 (2065743.47, 7281762.21)	42.41 (18.74, 66.08)
2038	Both	4736473.76 (1844437.86, 7628509.67)	42.45 (16.52, 68.38)
2039	Both	4802830 (1605854.96, 7999806.16)	42.53 (14.21, 70.85)
2040	Both	4873070.06 (1348266.72, 8397884.2)	42.65 (11.79, 73.51)
2041	Both	4947723.87 (1069575.45, 8825907.17)	42.82 (9.24, 76.39)
2042	Both	5026962.05 (767337.3, 9286834.56)	43.04 (6.55, 79.52)
2043	Both	5110716 (439316.76, 9783183.19)	43.3 (3.71, 82.9)

2044	Both	5200297.2 (158679.61, 10320594.07)	43.61 (1.33, 86.58)
2045	Both	5296314.47 (0, 10904176.42)	43.99 (0, 90.59)
2022	Female	2041429.86 (1910561.76, 2172297.95)	45.45 (44.1, 46.8)
2023	Female	2064549.47 (1913205.43, 2215893.51)	45.16 (43.07, 47.24)
2024	Female	2087822.99 (1903557.69, 2272088.28)	44.87 (41.85, 47.89)
2025	Female	2110981.4 (1882287.4, 2339675.4)	44.58 (40.48, 48.67)
2026	Female	2133887.91 (1850713.88, 2417061.95)	44.28 (38.99, 49.57)
2027	Female	2156530.83 (1810011.77, 2503049.89)	43.99 (37.41, 50.57)
2028	Female	2179374.87 (1761555.52, 2597194.21)	43.69 (35.74, 51.65)
2029	Female	2202798.21 (1706188.43, 2699407.99)	43.4 (34, 52.8)
2030	Female	2226642.49 (1644011.24, 2809273.73)	43.1 (32.19, 54.01)
2031	Female	2250875.5 (1575003.18, 2926747.82)	42.81 (30.33, 55.29)
2032	Female	2275492.16 (1499037.32, 3051947)	42.51 (28.41, 56.62)
2033	Female	2300818.3 (1416286.94, 3185349.66)	42.22 (26.44, 58)
2034	Female	2327326.75 (1326817.52, 3327835.98)	41.92 (24.42, 59.42)
2035	Female	2354993 (1230207.3, 3479778.7)	41.63 (22.37, 60.89)
2036	Female	2383871.23 (1125926.11, 3641816.35)	41.35 (20.29, 62.4)
2037	Female	2413991.96 (1013314.31, 3814669.61)	41.07 (18.17, 63.96)
2038	Female	2445579.96 (891861.44, 3999298.48)	40.78 (16.02, 65.55)
2039	Female	2479221.42 (760983.16, 4197460.81)	40.51 (13.85, 67.17)
2040	Female	2515021.46 (619667.85, 4410381.03)	40.24 (11.65, 68.83)
2041	Female	2553132.44 (466685.84, 4639603.32)	39.97 (9.42, 70.52)
2042	Female	2593649.87 (300663.79, 4886793.37)	39.71 (7.18, 72.25)
2043	Female	2636710.86 (120513.91, 5153730.04)	39.46 (4.91, 74.01)
2044	Female	2683036.99 (0, 5443652.42)	39.21 (2.62, 75.79)
2045	Female	2732927.51 (0, 5759437.25)	38.96 (0.32, 77.61)

2022	Male	1878236.24 (1752607.49, 2003864.98)	43.75 (42.44, 45.06)
2023	Male	1902206.14 (1759724.84, 2044687.45)	43.56 (41.6, 45.51)
2024	Male	1926225.93 (1756798.05, 2095653.8)	43.36 (40.58, 46.14)
2025	Male	1950079.17 (1743968.64, 2156189.71)	43.16 (39.41, 46.9)
2026	Male	1973893.99 (1722296.92, 2225491.05)	42.95 (38.14, 47.76)
2027	Male	1997634.09 (1692660.66, 2302607.51)	42.75 (36.78, 48.72)
2028	Male	2021489.34 (1656080, 2386898.67)	42.54 (35.33, 49.75)
2029	Male	2045717.05 (1613267.66, 2478166.44)	42.33 (33.82, 50.85)
2030	Male	2070190.01 (1564351.22, 2576028.8)	42.12 (32.23, 52)
2031	Male	2095134.02 (1509517.35, 2680750.69)	41.9 (30.59, 53.22)
2032	Male	2120533.3 (1448654.38, 2792412.23)	41.69 (28.9, 54.49)
2033	Male	2146438.37 (1381806.27, 2911070.48)	41.48 (27.15, 55.81)
2034	Male	2173220.61 (1309075.1, 3037366.13)	41.26 (25.36, 57.16)
2035	Male	2200887.27 (1230166.97, 3171607.56)	41.04 (23.53, 58.56)
2036	Male	2229730.64 (1144800.25, 3314661.03)	40.83 (21.66, 60.01)
2037	Male	2259760.88 (1052429.17, 3467092.6)	40.63 (19.76, 61.5)
2038	Male	2290893.8 (952576.42, 3629211.19)	40.42 (17.81, 63.02)
2039	Male	2323608.58 (844871.8, 3802345.35)	40.21 (15.84, 64.58)
2040	Male	2358048.59 (728598.87, 3987503.17)	40.01 (13.84, 66.17)
2041	Male	2394591.43 (602889.62, 4186303.85)	39.81 (11.82, 67.8)
2042	Male	2433312.18 (466673.5, 4400041.2)	39.62 (9.76, 69.48)
2043	Male	2474005.14 (318802.85, 4629453.15)	39.43 (7.68, 71.18)
2044	Male	2517260.21 (158679.61, 4876941.64)	39.24 (5.58, 72.91)
2045	Male	2563386.97 (0, 5144739.18)	39.06 (3.45, 74.67)
**YLDs**			
2022	Both	593776.8 (562718.9, 624834.7)	6.77 (6.41, 7.12)

2023	Both	600656.69 (563347.19, 637966.19)	6.73 (6.31, 7.15)
2024	Both	607547.38 (560367.7, 654727.06)	6.69 (6.17, 7.21)
2025	Both	614381.41 (554176.23, 674586.6)	6.66 (6, 7.31)
2026	Both	621149.38 (545223.03, 697075.73)	6.62 (5.81, 7.43)
2027	Both	627838.88 (533805.71, 721872.05)	6.59 (5.6, 7.58)
2028	Both	634548.62 (520273.03, 748824.22)	6.56 (5.38, 7.74)
2029	Both	641389.99 (504855.17, 777924.81)	6.53 (5.14, 7.92)
2030	Both	648326.98 (487576.65, 809077.31)	6.5 (4.89, 8.12)
2031	Both	655370.71 (468435.07, 842306.36)	6.48 (4.63, 8.33)
2032	Both	662507.86 (447371.58, 877644.14)	6.46 (4.36, 8.56)
2033	Both	669797.54 (424426.09, 915168.98)	6.44 (4.08, 8.8)
2034	Both	677387.74 (399644.27, 955131.2)	6.42 (3.79, 9.06)
2035	Both	685282.8 (372928.03, 997637.57)	6.41 (3.49, 9.34)
2036	Both	693506.9 (344143.52, 1042870.93)	6.41 (3.18, 9.64)
2037	Both	702052.34 (313103.47, 1091002.97)	6.41 (2.85, 9.96)
2038	Both	710948.43 (279679.64, 1142220.21)	6.41 (2.52, 10.3)
2039	Both	720380.52 (243756.32, 1197009.04)	6.42 (2.17, 10.67)
2040	Both	730388.62 (205089.4, 1255693.65)	6.43 (1.8, 11.06)
2041	Both	741010.76 (163388.94, 1318662.41)	6.45 (1.42, 11.49)
2042	Both	752251.58 (118282.73, 1386303.91)	6.48 (1.01, 11.95)
2043	Both	764113.42 (69549.98, 1458984.43)	6.52 (0.59, 12.45)
2044	Both	776824.49 (17447.83, 1537572.38)	6.56 (0.15, 12.99)
2045	Both	790476.79 (0, 1622798.05)	6.61 (0, 13.58)
2022	Female	314964.55 (299048.05, 330881.04)	7.04 (6.86, 7.21)
2023	Female	318382.26 (299039.01, 337725.51)	6.99 (6.7, 7.29)
2024	Female	321815.24 (297069.38, 346561.11)	6.95 (6.51, 7.38)

2025	Female	325219.65 (293380.11, 357059.19)	6.9 (6.3, 7.5)
2026	Female	328566.06 (288205.71, 368926.4)	6.86 (6.07, 7.64)
2027	Female	331851.89 (281712.96, 381990.82)	6.81 (5.83, 7.79)
2028	Female	335151.59 (274106.55, 396196.63)	6.76 (5.57, 7.95)
2029	Female	338533.32 (265514.8, 411551.83)	6.71 (5.31, 8.12)
2030	Female	341970.8 (255943.98, 427997.61)	6.67 (5.03, 8.31)
2031	Female	345446.12 (245375.65, 445516.59)	6.62 (4.74, 8.5)
2032	Female	348954.58 (233781.75, 464127.41)	6.57 (4.45, 8.7)
2033	Female	352549.22 (221198.25, 483900.2)	6.53 (4.15, 8.91)
2034	Female	356315.08 (207651.49, 504978.66)	6.48 (3.84, 9.12)
2035	Female	360246.83 (193083.36, 527410.3)	6.43 (3.53, 9.34)
2036	Female	364335.79 (177411.29, 551260.73)	6.39 (3.21, 9.57)
2037	Female	368580.83 (160538.36, 576624.28)	6.34 (2.88, 9.8)
2038	Female	373020.89 (142406.07, 603637.27)	6.3 (2.56, 10.03)
2039	Female	377759.37 (122949.98, 632570.96)	6.25 (2.22, 10.28)
2040	Female	382808.15 (102032.84, 663586.35)	6.2 (1.89, 10.52)
2041	Female	388166.55 (79494.21, 696856.14)	6.16 (1.55, 10.77)
2042	Female	393838.47 (55134.9, 732590.02)	6.12 (1.21, 11.03)
2043	Female	399850.21 (28843.19, 771032.36)	6.07 (0.86, 11.29)
2044	Female	406327.12 (934.18, 812662.92)	6.03 (0.51, 11.55)
2045	Female	413308.25 (0, 857860.76)	5.99 (0.16, 11.81)
2022	Male	278812.25 (263670.85, 293953.66)	6.49 (6.32, 6.66)
2023	Male	282274.43 (264308.18, 300240.68)	6.46 (6.19, 6.73)
2024	Male	285732.14 (263298.32, 308165.95)	6.43 (6.03, 6.83)
2025	Male	289161.76 (260796.12, 317527.4)	6.4 (5.86, 6.95)
2026	Male	292583.32 (257017.32, 328149.33)	6.37 (5.66, 7.08)

2027	Male	295986.99 (252092.75, 339881.23)	6.34 (5.45, 7.23)
2028	Male	299397.03 (246166.48, 352627.59)	6.31 (5.23, 7.38)
2029	Male	302856.68 (239340.37, 366372.98)	6.27 (5, 7.55)
2030	Male	306356.18 (231632.67, 381079.69)	6.24 (4.76, 7.72)
2031	Male	309924.59 (223059.42, 396789.77)	6.21 (4.51, 7.9)
2032	Male	313553.29 (213589.84, 413516.73)	6.18 (4.25, 8.1)
2033	Male	317248.31 (203227.85, 431268.78)	6.14 (3.99, 8.29)
2034	Male	321072.66 (191992.78, 450152.54)	6.11 (3.72, 8.5)
2035	Male	325035.97 (179844.68, 470227.27)	6.07 (3.44, 8.71)
2036	Male	329171.11 (166732.23, 491610.19)	6.04 (3.15, 8.92)
2037	Male	333471.51 (152565.11, 514378.7)	6.01 (2.86, 9.15)
2038	Male	337927.54 (137273.57, 538582.94)	5.97 (2.57, 9.37)
2039	Male	342621.14 (120806.34, 564438.08)	5.94 (2.27, 9.61)
2040	Male	347580.47 (103056.56, 592107.3)	5.9 (1.96, 9.84)
2041	Male	352844.21 (83894.73, 621806.26)	5.87 (1.66, 10.08)
2042	Male	358413.11 (63147.83, 653713.89)	5.84 (1.35, 10.33)
2043	Male	364263.21 (40706.79, 687952.07)	5.8 (1.03, 10.58)
2044	Male	370497.37 (16513.65, 724909.46)	5.77 (0.71, 10.83)
2045	Male	377168.54 (0, 764937.29)	5.74 (0.39, 11.09)

Notes: ASR: age-standardized rates; YLDs: years lived with disability.

Table S80. Prevalence and YLDs of pancreatitis in the worldwide of future forecasts using bayesian age-period-cohort model.

Year	Sex	Number	ASR
**Prevalence**			
2022	Both	6055816.79 (5803678.76, 6307954.83)	68.75 (65.89, 71.62)
2023	Both	6105401.36 (5821472.24, 6389330.47)	68.04 (64.87, 71.21)
2024	Both	6159218.54 (5825463.36, 6492973.72)	67.38 (63.72, 71.04)
2025	Both	6219745.96 (5818679.71, 6620812.21)	66.82 (62.5, 71.14)
2026	Both	6278541.31 (5793379.41, 6763703.21)	66.27 (61.14, 71.4)
2027	Both	6331949.8 (5746236.66, 6917662.94)	65.71 (59.62, 71.81)
2028	Both	6380901.55 (5682848.55, 7078954.55)	65.14 (57.99, 72.29)
2029	Both	6434093.88 (5612696.4, 7255491.37)	64.63 (56.35, 72.91)
2030	Both	6494659.26 (5538983.56, 7450334.96)	64.22 (54.73, 73.71)
2031	Both	6553359.33 (5452772.98, 7653945.68)	63.82 (53.05, 74.59)
2032	Both	6607596.89 (5350450.64, 7864743.14)	63.41 (51.28, 75.55)
2033	Both	6657614.09 (5234900.17, 8080328.01)	63 (49.45, 76.55)
2034	Both	6712053.51 (5113675.19, 8310431.83)	62.65 (47.62, 77.67)
2035	Both	6774538.19 (4989237, 8559864.92)	62.39 (45.81, 78.96)
2036	Both	6835130.39 (4853489.57, 8817533.12)	62.14 (43.96, 80.33)
2037	Both	6892239.11 (4703511.97, 9083123.35)	61.9 (42.06, 81.78)
2038	Both	6945564.83 (4540658.15, 9354364.16)	61.66 (40.09, 83.29)
2039	Both	7003670.94 (4370803.03, 9642384.23)	61.47 (38.1, 84.93)
2040	Both	7070724.4 (4197095.69, 9953686.82)	61.37 (36.13, 86.76)
2041	Both	7136632.46 (4012448.4, 10275927.81)	61.29 (34.12, 88.69)
2042	Both	7200980.99 (3814679.48, 10610624.15)	61.23 (32.07, 90.75)
2043	Both	7262913.25 (3603081.84, 10955453.32)	61.17 (29.95, 92.91)

2044	Both	7330405.27 (3383171.25, 11322573.08)	61.17 (27.8, 95.26)
2045	Both	7407655.9 (3157366.03, 11719918.37)	61.26 (25.65, 97.85)
2022	Female	2695649.08 (2583995.28, 2807302.89)	59.16 (58.08, 60.25)
2023	Female	2719036.75 (2592248.07, 2845825.44)	58.49 (56.86, 60.13)
2024	Female	2744934.81 (2594376.2, 2895493.41)	57.88 (55.57, 60.2)
2025	Female	2774856.76 (2592258.16, 2957455.36)	57.37 (54.27, 60.47)
2026	Female	2803768.67 (2581299.98, 3026237.36)	56.86 (52.9, 60.82)
2027	Female	2829530.03 (2559493.81, 3099566.24)	56.32 (51.41, 61.22)
2028	Female	2853129.35 (2530021.86, 3176236.83)	55.76 (49.85, 61.66)
2029	Female	2879412.37 (2497959.25, 3260865.48)	55.26 (48.3, 62.21)
2030	Female	2910055.5 (2464939.8, 3355171.2)	54.86 (46.81, 62.9)
2031	Female	2939362.05 (2425583.99, 3453140.11)	54.44 (45.26, 63.63)
2032	Female	2965552.52 (2377691.27, 3553413.76)	54 (43.63, 64.36)
2033	Female	2989892.92 (2323602.66, 3656183.18)	53.54 (41.97, 65.12)
2034	Female	3017136.45 (2267329.31, 3766943.58)	53.14 (40.32, 65.96)
2035	Female	3049037.61 (2210002.2, 3888098.54)	52.83 (38.71, 66.94)
2036	Female	3079249.13 (2146955.7, 4012304.48)	52.49 (37.07, 67.92)
2037	Female	3106393.17 (2075777.55, 4138589.76)	52.13 (35.35, 68.9)
2038	Female	3132108.74 (1998562.73, 4268156.77)	51.75 (33.61, 69.89)
2039	Female	3161072.16 (1918441.64, 4407251.68)	51.43 (31.89, 70.97)
2040	Female	3195206.69 (1837414.08, 4559020.28)	51.18 (30.19, 72.16)
2041	Female	3227770.46 (1750563.04, 4714830.93)	50.91 (28.46, 73.35)
2042	Female	3257912.87 (1655610.58, 4874432.14)	50.6 (26.68, 74.52)
2043	Female	3287602.24 (1554299.76, 5040146.96)	50.29 (24.87, 75.71)
2044	Female	3321141.54 (1450062.19, 5218768.59)	50.02 (23.07, 76.97)
2045	Female	3360297.78 (1343784.64, 5413956.18)	49.82 (21.29, 78.35)

2022	Male	3360167.71 (3219683.47, 3500651.94)	78.23 (76.81, 79.65)
2023	Male	3386364.6 (3229224.17, 3543505.03)	77.47 (75.39, 79.55)
2024	Male	3414283.73 (3231087.16, 3597480.3)	76.76 (73.85, 79.67)
2025	Male	3444889.2 (3226421.55, 3663356.85)	76.13 (72.27, 79.99)
2026	Male	3474772.64 (3212079.43, 3737465.85)	75.52 (70.6, 80.44)
2027	Male	3502419.77 (3186742.85, 3818096.7)	74.9 (68.82, 80.99)
2028	Male	3527772.2 (3152826.69, 3902717.71)	74.26 (66.95, 81.58)
2029	Male	3554681.52 (3114737.14, 3994625.89)	73.66 (65.06, 82.27)
2030	Male	3584603.76 (3074043.76, 4095163.76)	73.15 (63.2, 83.09)
2031	Male	3613997.28 (3027188.99, 4200805.57)	72.65 (61.3, 83.99)
2032	Male	3642044.37 (2972759.36, 4311329.37)	72.15 (59.34, 84.96)
2033	Male	3667721.17 (2911297.51, 4424144.83)	71.62 (57.31, 85.93)
2034	Male	3694917.06 (2846345.88, 4543488.25)	71.13 (55.28, 86.98)
2035	Male	3725500.59 (2779234.8, 4671766.38)	70.71 (53.27, 88.14)
2036	Male	3755881.26 (2706533.87, 4805228.64)	70.3 (51.24, 89.36)
2037	Male	3785845.94 (2627734.42, 4944533.59)	69.9 (49.16, 90.64)
2038	Male	3813456.09 (2542095.41, 5086207.39)	69.47 (47.03, 91.91)
2039	Male	3842598.78 (2452361.39, 5235132.55)	69.06 (44.89, 93.23)
2040	Male	3875517.71 (2359681.61, 5394666.54)	68.71 (42.76, 94.66)
2041	Male	3908862 (2261885.36, 5561096.88)	68.38 (40.62, 96.13)
2042	Male	3943068.12 (2159068.89, 5736192.01)	68.06 (38.44, 97.68)
2043	Male	3975311.02 (2048782.08, 5915306.36)	67.71 (36.22, 99.2)
2044	Male	4009263.73 (1933109.06, 6103804.49)	67.37 (33.98, 100.76)
2045	Male	4047358.12 (1813581.39, 6305962.19)	67.08 (31.75, 102.41)
**YLDs**			
2022	Both	375469.01 (362818.98, 388119.04)	4.28 (4.14, 4.43)

2023	Both	378797.14 (363903.93, 393690.35)	4.24 (4.08, 4.41)
2024	Both	382396.44 (364065.44, 400727.44)	4.21 (4.01, 4.41)
2025	Both	386411.73 (363572.88, 409250.58)	4.18 (3.93, 4.43)
2026	Both	390292.28 (361958.96, 418625.6)	4.15 (3.85, 4.46)
2027	Both	393855.81 (359017.31, 428694.31)	4.12 (3.76, 4.49)
2028	Both	397203.95 (355159.11, 439248.78)	4.09 (3.66, 4.53)
2029	Both	400795.27 (350888.78, 450701.76)	4.07 (3.56, 4.58)
2030	Both	404796.98 (346373.51, 463220.45)	4.05 (3.46, 4.64)
2031	Both	408696.89 (341104.23, 476289.55)	4.03 (3.36, 4.7)
2032	Both	412382.34 (334857.48, 489907.2)	4.01 (3.25, 4.77)
2033	Both	415873.37 (327829.13, 503917.61)	3.99 (3.14, 4.84)
2034	Both	419594.49 (320399.71, 518789.27)	3.97 (3.02, 4.92)
2035	Both	423722.82 (312690.75, 534757.52)	3.96 (2.91, 5.01)
2036	Both	427786.17 (304314.19, 551317.31)	3.95 (2.8, 5.1)
2037	Both	431733.38 (295099.23, 568542.85)	3.94 (2.68, 5.2)
2038	Both	435515.15 (285086.54, 586249.47)	3.93 (2.55, 5.31)
2039	Both	439523.91 (274559.34, 604940.62)	3.92 (2.43, 5.42)
2040	Both	443953.1 (263700.65, 624921.71)	3.92 (2.31, 5.55)
2041	Both	448396.23 (252236.9, 645734.36)	3.92 (2.18, 5.68)
2042	Both	452871.82 (239958.34, 667565.79)	3.92 (2.05, 5.82)
2043	Both	457268.02 (226806.32, 690192.97)	3.93 (1.92, 5.97)
2044	Both	461921.19 (213071.97, 714134.95)	3.93 (1.78, 6.14)
2045	Both	467015.25 (198938.1, 739756.35)	3.94 (1.64, 6.31)
2022	Female	167423.36 (161618.11, 173228.62)	3.7 (3.65, 3.76)
2023	Female	169010.78 (162163.98, 175857.57)	3.67 (3.58, 3.76)
2024	Female	170753.66 (162306.31, 179201.02)	3.64 (3.5, 3.78)

2025	Female	172716.92 (162167.43, 183266.4)	3.61 (3.42, 3.8)
2026	Female	174618.85 (161504.56, 187733.13)	3.58 (3.34, 3.83)
2027	Female	176324.4 (160172.91, 192475.89)	3.56 (3.25, 3.86)
2028	Female	177949.86 (158428.76, 197470.96)	3.53 (3.16, 3.89)
2029	Female	179732.7 (156525.08, 202940.32)	3.5 (3.07, 3.93)
2030	Female	181734.56 (154522.68, 208946.44)	3.48 (2.98, 3.98)
2031	Female	183675.77 (152148.69, 215202.84)	3.46 (2.89, 4.03)
2032	Female	185442.47 (149247.41, 221637.53)	3.44 (2.79, 4.09)
2033	Female	187152.02 (146000.54, 228303.51)	3.41 (2.69, 4.14)
2034	Female	189023.31 (142596.38, 235450.24)	3.39 (2.59, 4.2)
2035	Female	191108.71 (139065.21, 243154.83)	3.38 (2.49, 4.27)
2036	Female	193132.87 (135213.29, 251111.61)	3.36 (2.38, 4.34)
2037	Female	195004.58 (130863.73, 259267.43)	3.34 (2.28, 4.41)
2038	Female	196844.55 (126160.54, 267721.07)	3.32 (2.17, 4.48)
2039	Female	198854.43 (121239.92, 276741.3)	3.31 (2.06, 4.55)
2040	Female	201086.24 (116208.4, 286425.79)	3.3 (1.96, 4.63)
2041	Female	203283.78 (110848.34, 296467.2)	3.28 (1.85, 4.72)
2042	Female	205387.06 (104992.7, 306854.15)	3.27 (1.74, 4.8)
2043	Female	207516.1 (98745.62, 317731.35)	3.25 (1.62, 4.88)
2044	Female	209840.68 (92300.92, 329375.04)	3.24 (1.51, 4.97)
2045	Female	212397.99 (85674.15, 341894.18)	3.23 (1.39, 5.07)
2022	Male	208045.65 (201200.88, 214890.43)	4.85 (4.78, 4.92)
2023	Male	209786.37 (201739.95, 217832.79)	4.81 (4.7, 4.93)
2024	Male	211642.77 (201759.13, 221526.42)	4.77 (4.6, 4.95)
2025	Male	213694.81 (201405.44, 225984.17)	4.74 (4.51, 4.97)
2026	Male	215673.44 (200454.4, 230892.47)	4.71 (4.41, 5.01)

2027	Male	217531.41 (198844.4, 236218.42)	4.68 (4.31, 5.05)
2028	Male	219254.09 (196730.35, 241777.82)	4.64 (4.19, 5.1)
2029	Male	221062.57 (194363.7, 247761.44)	4.61 (4.08, 5.15)
2030	Male	223062.42 (191850.83, 254274)	4.59 (3.97, 5.21)
2031	Male	225021.12 (188955.53, 261086.72)	4.56 (3.85, 5.27)
2032	Male	226939.87 (185610.07, 268269.67)	4.54 (3.73, 5.34)
2033	Male	228721.35 (181828.58, 275614.11)	4.51 (3.61, 5.41)
2034	Male	230571.18 (177803.33, 283339.03)	4.48 (3.49, 5.48)
2035	Male	232614.11 (173625.53, 291602.69)	4.46 (3.36, 5.56)
2036	Male	234653.3 (169100.9, 300205.7)	4.44 (3.24, 5.64)
2037	Male	236728.8 (164235.5, 309275.41)	4.42 (3.11, 5.73)
2038	Male	238670.61 (158926.01, 318528.4)	4.4 (2.98, 5.82)
2039	Male	240669.48 (153319.42, 328199.32)	4.38 (2.84, 5.92)
2040	Male	242866.87 (147492.25, 338495.92)	4.36 (2.71, 6.01)
2041	Male	245112.45 (141388.56, 349267.16)	4.34 (2.57, 6.11)
2042	Male	247484.76 (134965.64, 360711.64)	4.33 (2.44, 6.22)
2043	Male	249751.93 (128060.7, 372461.61)	4.31 (2.3, 6.33)
2044	Male	252080.51 (120771.05, 384759.91)	4.3 (2.16, 6.43)
2045	Male	254617.26 (113263.95, 397862.17)	4.28 (2.01, 6.55)

Notes: ASR: age-standardized rates; YLDs: years lived with disability.

Table S81. Prevalence and YLDs of male infertility in the worldwide of future forecasts using bayesian age-period-cohort model.

Year	Sex	Number	ASR
**Prevalence**			
2022	Male	57439752.67 (53579015.39, 61300489.94)	1380.19 (1327.58, 1432.8)
2023	Male	58674669.24 (54304606.03, 63044732.45)	1398.75 (1329.81, 1467.69)
2024	Male	59903999.87 (54795632.45, 65012367.28)	1416.92 (1326.33, 1507.51)
2025	Male	61115781.65 (55038883.05, 67192680.25)	1434.56 (1317.9, 1551.22)
2026	Male	62305078.68 (55038067.39, 69572089.97)	1451.72 (1305.11, 1598.33)
2027	Male	63471462.15 (54805158.71, 72137765.58)	1468.56 (1288.43, 1648.7)
2028	Male	64595966.05 (54343075.37, 74848856.72)	1485.06 (1268.19, 1701.93)
2029	Male	65671719.96 (53658977.5, 77684462.42)	1500.97 (1244.39, 1757.55)
2030	Male	66714140.94 (52771810.85, 80656471.02)	1516.18 (1217.04, 1815.32)
2031	Male	67746276.22 (51700178.06, 83792374.38)	1530.68 (1186.16, 1875.2)
2032	Male	68803352.52 (50467957.24, 87138747.8)	1544.7 (1151.97, 1937.43)
2033	Male	69876414.03 (49069601.45, 90683226.6)	1558.49 (1114.77, 2002.21)
2034	Male	70952061.47 (47492322.32, 94411800.62)	1572.01 (1074.59, 2069.43)
2035	Male	72047435.53 (45741089.77, 98353781.29)	1585.18 (1031.37, 2138.99)
2036	Male	73181103.37 (43816287.36, 102545919.38)	1597.93 (985.02, 2210.84)
2037	Male	74394830.15 (41727408.73, 107062251.57)	1610.44 (935.62, 2285.25)
2038	Male	75669709.86 (39451373.98, 111888045.74)	1623.06 (883.41, 2362.71)
2039	Male	77019805.05 (36977694.44, 117061915.66)	1635.87 (828.39, 2443.35)
2040	Male	78441336.33 (34282348.63, 122600324.04)	1648.84 (770.46, 2527.21)
2041	Male	79924982.2 (31335685.63, 128514278.77)	1661.82 (709.45, 2614.2)
2042	Male	81477061.1 (28113320.39, 134840801.81)	1674.91 (645.28, 2704.53)
2043	Male	83093252.3 (24584293.74, 141602210.85)	1688.3 (578.02, 2798.58)
2044	Male	84789747.85 (20725535.33, 148853960.37)	1702.04 (507.6, 2896.48)

2045	Male	86565308.31 (16593472.9, 156630853.66)	1716.05 (433.89, 2998.2)
**YLDs**			
2022	Male	331122.15 (310477.65, 351766.66)	7.97 (7.68, 8.25)
2023	Male	337954.84 (314265.88, 361643.8)	8.07 (7.69, 8.45)
2024	Male	344718.44 (316588.21, 372848.68)	8.17 (7.65, 8.68)
2025	Male	351342.2 (317404.38, 385280.02)	8.26 (7.59, 8.93)
2026	Male	357794.64 (316763.09, 398826.2)	8.35 (7.51, 9.19)
2027	Male	364073.13 (314749.42, 413396.84)	8.43 (7.39, 9.47)
2028	Male	370076.98 (311397.56, 428756.4)	8.52 (7.26, 9.77)
2029	Male	375766.76 (306754.63, 444778.88)	8.6 (7.11, 10.08)
2030	Male	381220.18 (300925.12, 461515.24)	8.67 (6.93, 10.4)
2031	Male	386554.58 (294008.52, 479100.65)	8.74 (6.74, 10.74)
2032	Male	391960.52 (286137.74, 497783.3)	8.8 (6.52, 11.08)
2033	Male	397393.44 (277292.68, 517494.19)	8.86 (6.29, 11.43)
2034	Male	402787.85 (267415.3, 538160.4)	8.92 (6.04, 11.8)
2035	Male	408237.79 (256538.44, 559937.13)	8.98 (5.78, 12.18)
2036	Male	413840.77 (244666.75, 583014.79)	9.03 (5.49, 12.57)
2037	Male	419835.04 (231861.19, 607808.89)	9.08 (5.19, 12.97)
2038	Male	426110.28 (217997.71, 634222.84)	9.13 (4.88, 13.39)
2039	Male	432769.3 (203035.96, 662502.65)	9.18 (4.55, 13.82)
2040	Male	439796.44 (186849.24, 692743.63)	9.24 (4.2, 14.27)
2041	Male	447140.14 (169277.4, 725002.88)	9.29 (3.84, 14.73)
2042	Male	454834.74 (150188.11, 759481.37)	9.34 (3.47, 15.21)
2043	Male	462876.13 (129417.29, 796334.97)	9.39 (3.07, 15.71)
2044	Male	471344.22 (106833.36, 835855.07)	9.45 (2.66, 16.23)
2045	Male	480230.37 (82827.38, 878231)	9.5 (2.24, 16.77)

Notes: ASR: age-standardized rates; YLDs: years lived with disability.

Table S82. Prevalence and YLDs of female infertility in the worldwide of future forecasts using bayesian age-period-cohort model.

Year	Sex	Number	ASR
**Prevalence**			
2022	Female	114518735.57 (97657769.72, 131379701.41)	2781.3 (2558.6, 3004)
2023	Female	116983157.4 (98832316.39, 135133998.41)	2818.89 (2557.09, 3080.68)
2024	Female	119406584.73 (99526748.95, 139286420.51)	2855.64 (2542.18, 3169.11)
2025	Female	121758022.37 (99667279.8, 143848764.94)	2891.16 (2514.61, 3267.72)
2026	Female	124022914.11 (99212679.31, 148833148.9)	2925.48 (2475.45, 3375.52)
2027	Female	126198549.54 (98155090.39, 154242008.7)	2958.98 (2425.89, 3492.07)
2028	Female	128245697.41 (96494992.64, 159996402.17)	2991.87 (2367.24, 3616.5)
2029	Female	130154908.06 (94249882.25, 166059933.87)	3023.72 (2299.77, 3747.66)
2030	Female	131978984.55 (91468713.33, 172489255.78)	3054.17 (2223.5, 3884.84)
2031	Female	133792554.63 (88199245.8, 179385863.46)	3083.26 (2138.53, 4028)
2032	Female	135698705.28 (84496978.13, 186900432.43)	3111.58 (2045.31, 4177.85)
2033	Female	137688029.37 (80352620.89, 195023437.85)	3139.89 (1944.68, 4335.11)
2034	Female	139709714.56 (75716193.11, 203703236.01)	3167.97 (1836.49, 4499.45)
2035	Female	141815169.02 (70583805.44, 213046532.61)	3195.37 (1720.33, 4670.41)
2036	Female	144065369.34 (64935149.47, 223195589.21)	3221.72 (1595.7, 4847.74)
2037	Female	146571186.23 (58752640.19, 234389732.26)	3247.32 (1462.52, 5032.12)
2038	Female	149328449.03 (52079065.94, 246683697.42)	3272.96 (1321.15, 5224.77)
2039	Female	152331563.72 (44744467.83, 260162910.3)	3298.6 (1171.32, 5425.88)
2040	Female	155567998.69 (36619748.29, 274916315.82)	3323.88 (1012.51, 5635.26)
2041	Female	159022350.95 (28907479.44, 291046387.33)	3348.42 (844.15, 5852.68)
2042	Female	162720504.39 (20492451.64, 308743822.89)	3372.34 (665.94, 6078.74)
2043	Female	166693674.99 (12443300.1, 328231923.38)	3396.27 (477.96, 6314.57)
2044	Female	171009591.66 (4614915.78, 349823557.82)	3420.47 (280.04, 6560.9)

2045	Female	175681785.68 (0, 373785683.48)	3445 (71.89, 6818.11)
**YLDs**			
2022	Female	626328.85 (540713.24, 711944.46)	15.24 (14.11, 16.37)
2023	Female	639831.65 (547290.16, 732373.15)	15.45 (14.1, 16.79)
2024	Female	652993.02 (551102.51, 754883.52)	15.64 (14.02, 17.27)
2025	Female	665628.35 (551754.36, 779502.35)	15.83 (13.86, 17.8)
2026	Female	677633.46 (549031.62, 806235.3)	16.01 (13.64, 18.38)
2027	Female	688971.53 (542910.4, 835032.66)	16.18 (13.36, 18.99)
2028	Female	699424.77 (533425.87, 865423.66)	16.34 (13.03, 19.64)
2029	Female	708935.3 (520696.89, 897173.7)	16.49 (12.65, 20.33)
2030	Female	717767.79 (505007.1, 930528.48)	16.62 (12.22, 21.03)
2031	Female	726294.45 (486629.27, 965959.64)	16.75 (11.74, 21.76)
2032	Female	735051.87 (465892.42, 1004211.33)	16.87 (11.21, 22.52)
2033	Female	744006.65 (442795.82, 1045217.47)	16.98 (10.65, 23.31)
2034	Female	752909.43 (417122.34, 1088696.52)	17.09 (10.05, 24.12)
2035	Female	762051.46 (388905.81, 1135197.1)	17.19 (9.41, 24.97)
2036	Female	771763.67 (358091.03, 1185436.31)	17.28 (8.72, 25.84)
2037	Female	782655.74 (324651.4, 1240660.09)	17.37 (8, 26.74)
2038	Female	794725 (288528.16, 1301142.56)	17.45 (7.23, 27.67)
2039	Female	807961.14 (249568.35, 1367315.36)	17.54 (6.43, 28.65)
2040	Female	822302.87 (206792.2, 1439601.49)	17.62 (5.58, 29.66)
2041	Female	837660.48 (163141.55, 1518464.04)	17.7 (4.7, 30.7)
2042	Female	854155.33 (119710.58, 1604803.97)	17.77 (3.76, 31.78)
2043	Female	871958.81 (74443.19, 1699725.03)	17.85 (2.79, 32.91)
2044	Female	891382.38 (33497.01, 1804681.79)	17.93 (1.77, 34.08)
2045	Female	912461.8 (1664.63, 1920878.35)	18 (0.7, 35.31)

Notes: ASR: age-standardized rates; YLDs: years lived with disability.

Table S83. Prevalence and YLDs of depressive disorders in the worldwide of future forecasts using bayesian age-period-cohort model.

Year	Sex	Number	ASR
**Prevalence**			
2022	Both	345415513.17 (326654096.58, 364176929.76)	4047.26 (3827.41, 4267.12)
2023	Both	360222000.74 (333680927.65, 386763073.83)	4164.97 (3858.08, 4471.87)
2024	Both	375845920.98 (337357582.06, 414334259.9)	4289.17 (3849.94, 4728.4)
2025	Both	392358922.87 (338253901.63, 446463944.1)	4420.73 (3811.16, 5030.29)
2026	Both	409669981.42 (336478472.51, 482861490.33)	4558.56 (3744.23, 5372.9)
2027	Both	427898309.82 (332085260.27, 523711359.37)	4703.94 (3650.82, 5757.06)
2028	Both	447275048.61 (325068779.18, 569481318.04)	4859.5 (3532.03, 6186.97)
2029	Both	468062331.83 (315300955.46, 620823708.21)	5027.48 (3387.06, 6667.91)
2030	Both	490393924.37 (302432432.62, 678355416.12)	5209.31 (3213.21, 7205.42)
2031	Both	514176204.21 (285864623.5, 742487784.92)	5403.75 (3005.06, 7802.43)
2032	Both	539628785.17 (265077338.07, 814180232.28)	5612.81 (2758.13, 8467.48)
2033	Both	567090907.61 (239451955.38, 894729859.83)	5840.69 (2467.5, 9213.87)
2034	Both	597012453.91 (208182754.06, 985842153.76)	6091.06 (2125.65, 10056.48)
2035	Both	629671807.67 (170109997.33, 1089233618.01)	6366.54 (1722.05, 11011.04)
2036	Both	665040660.19 (123694640.72, 1206386679.66)	6666.39 (1242.53, 12090.25)
2037	Both	703525386.83 (77828954.87, 1339873809.88)	6994.2 (776.99, 13317.34)
2038	Both	745773776.32 (60713302.57, 1493226292.63)	7356.78 (601.41, 14726.16)
2039	Both	792517640.6 (40076172.3, 1670857160.89)	7760.61 (393.9, 16356.74)
2040	Both	844352299.26 (15194536.57, 1877809567.62)	8211.13 (147.84, 18255.3)
2041	Both	901508881.88 (0, 2119400503.74)	8710 (0, 20469.44)
2042	Both	964827396.03 (0, 2403634106.75)	9264.78 (0, 23072.03)
2043	Both	1035646712.78 (0, 2741684893.53)	9887.94 (0, 26165.59)

2044	Both	1115378408.53 (0, 3147673005.28)	10592.57 (0, 29879.56)
2045	Both	1205436011.65 (0, 3639353084.98)	11391.59 (0, 34376.13)
2022	Female	209087753.68 (197261239.06, 220914268.3)	4858.98 (4693.3, 5024.67)
2023	Female	217678419.17 (200436735.35, 234920102.99)	4990.65 (4665.48, 5315.83)
2024	Female	226743655.86 (201292651.98, 252194659.73)	5127.79 (4597.8, 5657.77)
2025	Female	236334409.05 (200257296.23, 272411521.86)	5270.59 (4495.54, 6045.64)
2026	Female	246411302.91 (197421181.15, 295401424.67)	5416.95 (4357.71, 6476.19)
2027	Female	257057616.9 (192811082.41, 321304151.38)	5567.37 (4184.46, 6950.29)
2028	Female	268416321.85 (186395983.06, 350436660.63)	5723.81 (3976.49, 7471.12)
2029	Female	280650506.34 (178059073.48, 383241939.21)	5887.59 (3733.33, 8041.86)
2030	Female	293856532.46 (167546392.1, 420166672.83)	6058.97 (3453.26, 8664.67)
2031	Female	308006691.96 (154451921.02, 461561462.89)	6235.08 (3132.78, 9337.39)
2032	Female	323253905.77 (138381020.18, 508126791.36)	6416.52 (2770.32, 10062.73)
2033	Female	339817142.76 (118842361.82, 560791923.71)	6605.95 (2364.91, 10847)
2034	Female	357982983.7 (95190614.11, 620775353.29)	6805.14 (1914.39, 11695.9)
2035	Female	377954155.66 (66535019.5, 689373291.82)	7014.36 (1415.58, 12613.13)
2036	Female	399771192.79 (31719654.8, 767822730.78)	7230.02 (864.62, 13595.41)
2037	Female	423727364.14 (0, 858106719.36)	7452.7 (258.72, 14646.68)
2038	Female	450261126.02 (0, 962914294.61)	7685.64 (-405.18, 15776.46)
2039	Female	479869031.8 (0, 1085636115.58)	7930.93 (-1131.3, 16993.17)
2040	Female	513000570.56 (0, 1230300385.46)	8188.87 (-1924.31, 18302.06)
2041	Female	549924442.64 (0, 1401409067.76)	8455.38 (-2787.7, 19698.47)
2042	Female	591280191.18 (0, 1605513617.19)	8731.07 (-3725.66, 21187.8)
2043	Female	638022613.08 (0, 1851681447.8)	9019.61 (-4744.18, 22783.39)
2044	Female	691195296.02 (0, 2151579175.08)	9323.41 (-5850.1, 24496.92)
2045	Female	751913697.39 (0, 2520213040.28)	9642.86 (-7049.9, 26335.61)

2022	Male	136327759.49 (129392857.52, 143262661.46)	3225.18 (3137.08, 3313.28)
2023	Male	142543581.57 (133244192.29, 151842970.84)	3326.65 (3163.76, 3489.54)
2024	Male	149102265.12 (136064930.08, 162139600.17)	3432.58 (3171.11, 3694.06)
2025	Male	156024513.82 (137996605.4, 174052422.24)	3543.15 (3162.13, 3924.17)
2026	Male	163258678.51 (139057291.37, 187460065.66)	3656.8 (3135.97, 4177.63)
2027	Male	170840692.92 (139274177.85, 202407207.99)	3773.87 (3092.68, 4455.06)
2028	Male	178858726.76 (138672796.12, 219044657.41)	3895.85 (3032.98, 4758.71)
2029	Male	187411825.49 (137241881.98, 237581769)	4023.82 (2956.82, 5090.82)
2030	Male	196537391.91 (134886040.52, 258188743.29)	4157.99 (2863.21, 5452.77)
2031	Male	206169512.25 (131412702.47, 280926322.02)	4296.15 (2749.41, 5842.89)
2032	Male	216374879.41 (126696317.89, 306053440.92)	4438.73 (2614.55, 6262.91)
2033	Male	227273764.84 (120609593.56, 333937936.12)	4587.88 (2458.61, 6717.15)
2034	Male	239029470.21 (112992139.95, 365066800.46)	4745.08 (2280.71, 7209.46)
2035	Male	251717652.01 (103574977.82, 399860326.2)	4910.56 (2079, 7742.12)
2036	Male	265269467.4 (91974985.92, 438563948.88)	5081.44 (1850.37, 8312.52)
2037	Male	279798022.69 (77828954.87, 481767090.52)	5258.19 (1593.24, 8923.15)
2038	Male	295512650.3 (60713302.57, 530311998.03)	5443.52 (1306.53, 9580.51)
2039	Male	312648608.8 (40076172.3, 585221045.31)	5639.25 (988.21, 10290.3)
2040	Male	331351728.7 (15194536.57, 647509182.16)	5845.65 (635.54, 11055.76)
2041	Male	351584439.24 (0, 717991435.98)	6059.29 (245.42, 11873.16)
2042	Male	373547204.86 (0, 798120489.56)	6280.69 (-184.55, 12745.92)
2043	Male	397624099.7 (0, 890003445.73)	6513.04 (-657.14, 13683.23)
2044	Male	424183112.51 (0, 996093830.2)	6758.52 (-1175.97, 14693)
2045	Male	453522314.25 (0, 1119140044.7)	7017.44 (-1744.93, 15779.81)
**YLDs**			
2022	Both	58278234.05 (54667422.35, 61889045.74)	685.14 (642.68, 727.59)

2023	Both	60865674.37 (55680578.63, 66050770.11)	706.41 (646.23, 766.6)
2024	Both	63610736.25 (56025142.26, 71196330.24)	729.01 (642.08, 815.95)
2025	Both	66530318.97 (55813978.9, 77246659.04)	753.13 (631.83, 874.43)
2026	Both	69614258.07 (55066843.43, 84161672.7)	778.64 (615.95, 941.33)
2027	Both	72890096.32 (53785488.1, 91994704.55)	805.82 (594.65, 1016.99)
2028	Both	76404001.7 (51952314.62, 100855688.79)	835.2 (567.97, 1102.42)
2029	Both	80209769.09 (49521339.34, 110898198.84)	867.25 (535.53, 1198.97)
2030	Both	84340455.83 (46400137.31, 122280774.36)	902.32 (496.54, 1308.09)
2031	Both	88792294.61 (42445215.19, 135139374.03)	940.3 (449.67, 1430.94)
2032	Both	93617584.31 (37508781.14, 149726387.48)	981.69 (393.55, 1569.83)
2033	Both	98888326.76 (31402480.59, 166374172.92)	1027.35 (326.53, 1728.16)
2034	Both	104702415.99 (23877555.29, 185527276.68)	1078.11 (246.24, 1909.97)
2035	Both	111132678.65 (14589322.09, 207676072.18)	1134.65 (149.44, 2119.87)
2036	Both	118205751.75 (10379492, 233326900.22)	1197.16 (105.56, 2362.47)
2037	Both	126028008.04 (6701209.99, 263262321.84)	1266.59 (67.6, 2645.05)
2038	Both	134752974.26 (2185287.93, 298547513.59)	1344.53 (21.81, 2977.86)
2039	Both	144561256.91 (0, 340568649.47)	1432.61 (0, 3373.85)
2040	Both	155626039.78 (0, 391048103.04)	1532.46 (0, 3849.16)
2041	Both	168076327 (0, 452055224.31)	1645.21 (0, 4423.01)
2042	Both	182165512.05 (0, 526583744.33)	1773.19 (0, 5123.32)
2043	Both	198264265.24 (0, 618851437.61)	1919.85 (0, 5989.46)
2044	Both	216791627.77 (0, 734510337.33)	2089.16 (0, 7074.36)
2045	Both	238222354.27 (0, 881189754.51)	2285.53 (0, 8449.23)
2022	Female	35262855.97 (32983491.93, 37542220.01)	823.24 (790.65, 855.82)
2023	Female	36734857.05 (33368201.3, 40101512.79)	846.33 (781.86, 910.81)
2024	Female	38296787.71 (33294260.51, 43299314.9)	870.45 (765.07, 975.82)

2025	Female	39960322.76 (32845459.16, 47075186.35)	895.62 (741.29, 1049.95)
2026	Female	41722521.99 (32038132.56, 51406911.43)	921.49 (710.33, 1132.65)
2027	Female	43602191.33 (30871548.27, 56332834.39)	948.16 (672.18, 1224.14)
2028	Female	45628084 (29328836.65, 61927331.34)	975.96 (626.88, 1325.05)
2029	Female	47833839.96 (27372932.6, 68294747.32)	1005.13 (574.25, 1436.01)
2030	Female	50242584.75 (24936805.27, 75548364.23)	1035.71 (513.92, 1557.5)
2031	Female	52858195.06 (21920804.69, 83795585.43)	1067.23 (445.22, 1689.24)
2032	Female	55716647.97 (18213550.83, 93219745.11)	1099.79 (367.78, 1831.8)
2033	Female	58865229.43 (13669430.62, 104061028.25)	1133.84 (281.28, 1986.4)
2034	Female	62367110.59 (8093863.52, 116640357.66)	1169.66 (185.17, 2154.15)
2035	Female	66274498.61 (1221578.74, 131327455.45)	1207.31 (78.78, 2335.84)
2036	Female	70617165.39 (0, 148529219.5)	1246.22 (-38.63, 2531.08)
2037	Female	75471709.62 (0, 168850934.98)	1286.5 (-167.68, 2740.68)
2038	Female	80944761.18 (0, 193116326.66)	1328.65 (-309.15, 2966.45)
2039	Female	87161931.49 (0, 222408174.78)	1373.02 (-463.99, 3210.02)
2040	Female	94253248.39 (0, 258111356.37)	1419.66 (-633.2, 3472.53)
2041	Female	102332972.17 (0, 301952656.94)	1467.97 (-817.47, 3753.42)
2042	Female	111594948.31 (0, 356405227.84)	1518.04 (-1017.74, 4053.82)
2043	Female	122312466.97 (0, 424958288.01)	1570.45 (-1235.35, 4376.25)
2044	Female	134804384.87 (0, 512362643.69)	1625.59 (-1471.8, 4722.98)
2045	Female	149447211.01 (0, 625139217.63)	1683.54 (-1728.48, 5095.55)
2022	Male	23015378.08 (21683930.42, 24346825.73)	545.41 (528.1, 562.71)
2023	Male	24130817.32 (22312377.32, 25949257.32)	564.31 (531.83, 596.79)
2024	Male	25313948.54 (22730881.75, 27897015.34)	584.12 (531.6, 636.65)
2025	Male	26569996.22 (22968519.74, 30171472.69)	604.87 (527.97, 681.78)
2026	Male	27891736.07 (23028710.88, 32754761.27)	626.3 (520.77, 731.83)

2027	Male	29287905 (22913939.83, 35661870.16)	648.47 (509.95, 786.98)
2028	Male	30775917.71 (22623477.97, 38928357.45)	671.65 (495.59, 847.7)
2029	Male	32375929.13 (22148406.74, 42603451.53)	696.04 (477.6, 914.49)
2030	Male	34097871.08 (21463332.03, 46732410.13)	721.7 (455.72, 987.69)
2031	Male	35934099.55 (20524410.5, 51343788.6)	748.25 (429.41, 1067.08)
2032	Male	37900936.34 (19295230.31, 56506642.37)	775.76 (398.43, 1153.08)
2033	Male	40023097.32 (17733049.97, 62313144.67)	804.62 (362.66, 1246.57)
2034	Male	42335305.4 (15783691.77, 68886919.03)	835.09 (321.8, 1348.37)
2035	Male	44858180.04 (13367743.34, 76348616.73)	867.22 (275.41, 1459.03)
2036	Male	47588586.36 (10379492, 84797680.72)	900.55 (222.88, 1578.22)
2037	Male	50556298.42 (6701209.99, 94411386.86)	935.16 (163.79, 1706.53)
2038	Male	53808213.09 (2185287.93, 105431186.93)	971.52 (97.76, 1845.28)
2039	Male	57399325.42 (0, 118160474.68)	1009.94 (24.21, 1995.66)
2040	Male	61372791.39 (0, 132936746.68)	1050.49 (-57.5, 2158.47)
2041	Male	65743354.82 (0, 150102567.37)	1092.63 (-148, 2333.25)
2042	Male	70570563.74 (0, 170178516.48)	1136.46 (-247.91, 2520.82)
2043	Male	75951798.27 (0, 193893149.6)	1182.52 (-358.01, 2723.05)
2044	Male	81987242.91 (0, 222147693.65)	1231.19 (-479.24, 2941.63)
2045	Male	88775143.27 (0, 256050536.87)	1282.56 (-612.54, 3177.67)

Notes: ASR: age-standardized rates; YLDs: years lived with disability.

Table S84. Prevalence and YLDs of anxiety disorders in the worldwide of future forecasts using bayesian age-period-cohort model.

Year	Sex	Number	ASR
**Prevalence**			
2022	Both	374211161.24 (353738263.39, 394684059.09)	4495.83 (4249.82, 4741.85)
2023	Both	395798691.66 (359403233.68, 432194149.64)	4702.19 (4269.78, 5134.6)
2024	Both	419079865.91 (359314686.02, 478845045.79)	4924.58 (4222.31, 5626.85)
2025	Both	444325882.45 (354178781.5, 534472983.41)	5165.92 (4117.92, 6213.92)
2026	Both	471626217.34 (343691107.12, 599561327.57)	5426.89 (3954.94, 6898.84)
2027	Both	501371663.27 (327328698.27, 675414628.28)	5711.37 (3729.03, 7693.7)
2028	Both	534098473.88 (304267470.66, 763929477.09)	6026.11 (3433.4, 8618.82)
2029	Both	570501678.73 (273292734.59, 867710622.87)	6377.77 (3055.81, 9699.72)
2030	Both	611204890.86 (232561746.94, 989848034.79)	6772.73 (2577.86, 10967.6)
2031	Both	656621870.81 (179472643.04, 1133771098.58)	7214.74 (1973.16, 12456.32)
2032	Both	707643639.31 (110729788.27, 1304557490.34)	7712.63 (1208.44, 14216.83)
2033	Both	765547578.03 (30038777.72, 1509245277.57)	8280.17 (327.15, 16321.88)
2034	Both	831833581.4 (0, 1757111289.4)	8932.44 (0, 18865.45)
2035	Both	908135902.59 (0, 2060171559.86)	9685.83 (0, 21969.18)
2036	Both	995983696.84 (0, 2433518014.98)	10555.2 (0, 25784.73)
2037	Both	1097826777.42 (0, 2899022108.52)	11564.95 (0, 30532.59)
2038	Both	1217145144.75 (0, 3488217123.56)	12750.18 (0, 36531.47)
2039	Both	1358109502.79 (0, 4245077039.04)	14153.16 (0, 44226.2)
2040	Both	1525810647.34 (0, 5231804105.69)	15824.73 (0, 54243.49)
2041	Both	1726004701.38 (0, 6535605758.27)	17822.23 (0, 67460.56)
2042	Both	1966999022.65 (0, 8287070349.29)	20228.66 (0, 85190.43)
2043	Both	2260409652.94 (0, 10683616620.95)	23160.23 (0, 109416.66)

2044	Both	2621239835.64 (0, 14021486762.75)	26767.95 (0, 143118.22)
2045	Both	3069198238.37 (0, 18750472300.1)	31249.07 (0, 190810.06)
2022	Female	235614809.78 (222498575.65, 248731043.91)	5654.69 (5417.43, 5891.96)
2023	Female	250090318.91 (226363061.81, 273817576.02)	5931.56 (5411.9, 6451.22)
2024	Female	265752043.13 (226442344.2, 305061742.06)	6225.01 (5331.6, 7118.43)
2025	Female	282795549.27 (223148172.35, 342442926.2)	6536.07 (5177.32, 7894.81)
2026	Female	301306135.12 (216230451.1, 386381819.13)	6862.29 (4943.06, 8781.52)
2027	Female	321561841.28 (205278166.19, 437845516.37)	7204.74 (4623.97, 9785.5)
2028	Female	343944024.17 (189663636.33, 498224412)	7567.29 (4215.58, 10918.99)
2029	Female	368942152.4 (168469221.79, 569415083.01)	7952.86 (3710.82, 12194.91)
2030	Female	397016340.79 (140326517.06, 653706164.51)	8362.85 (3100.18, 13625.51)
2031	Female	428504703.61 (103310739.47, 753698667.74)	8793.95 (2371.66, 15216.24)
2032	Female	464066805.08 (54940356.53, 873193253.64)	9247.55 (1514.64, 16980.45)
2033	Female	504631358.86 (0, 1017451616.94)	9728.74 (517.73, 18939.74)
2034	Female	551307502.21 (0, 1193476968.28)	10241.4 (-633.24, 21116.04)
2035	Female	605331786.03 (0, 1410450519.72)	10787.36 (-1954.56, 23529.28)
2036	Female	667915242.77 (0, 1680096110.78)	11362.74 (-3462.31, 26187.78)
2037	Female	740933758.22 (0, 2019414696.29)	11969.38 (-5173.85, 29112.62)
2038	Female	827016499.98 (0, 2452991970.31)	12613.73 (-7110.56, 32338.02)
2039	Female	929376193.14 (0, 3015516463.44)	13300.74 (-9296.79, 35898.27)
2040	Female	1051985533.11 (0, 3756566411.89)	14032.89 (-11758.33, 39824.12)
2041	Female	1199443387.01 (0, 4746523113.71)	14806.02 (-14516.6, 44128.65)
2042	Female	1378330648.72 (0, 6091446079.47)	15622.71 (-17599.13, 48844.55)
2043	Female	1597823067.98 (0, 7952745642.26)	16491 (-21042.49, 54024.49)
2044	Female	1869928846.2 (0, 10575026949.9)	17417.19 (-24886.42, 59720.81)
2045	Female	2210588620.26 (0, 14333136806.37)	18404.72 (-29170.92, 65980.35)

2022	Male	138596351.47 (131239687.75, 145953015.19)	3333.01 (3205.39, 3460.62)
2023	Male	145708372.75 (133040171.88, 158376573.62)	3463.05 (3189.92, 3736.18)
2024	Male	153327822.77 (132872341.81, 173783303.73)	3599.88 (3137.06, 4062.71)
2025	Male	161530333.18 (131030609.15, 192030057.21)	3743.81 (3048.53, 4439.09)
2026	Male	170320082.23 (127460656.02, 213179508.44)	3893.19 (2922.2, 4864.19)
2027	Male	179809821.99 (122050532.07, 237569111.91)	4048.44 (2756.82, 5340.05)
2028	Male	190154449.71 (114603834.33, 265705065.09)	4211.61 (2551.67, 5871.55)
2029	Male	201559526.33 (104823512.81, 298295539.85)	4384.06 (2304.64, 6463.48)
2030	Male	214188550.07 (92235229.87, 336141870.27)	4566.18 (2012.45, 7119.91)
2031	Male	228117167.21 (76161903.58, 380072430.84)	4755.81 (1670.47, 7841.15)
2032	Male	243576834.22 (55789431.74, 431364236.7)	4953.38 (1275.25, 8631.51)
2033	Male	260916219.18 (30038777.72, 491793660.64)	5161.45 (823.4, 9499.5)
2034	Male	280526079.19 (0, 563634321.12)	5381.72 (310.27, 10453.17)
2035	Male	302804116.55 (0, 649721040.14)	5614.69 (-269.68, 11499.07)
2036	Male	328068454.07 (0, 753421904.21)	5857.86 (-921.96, 12637.68)
2037	Male	356893019.2 (0, 879607412.23)	6111.72 (-1652.08, 13875.52)
2038	Male	390128644.77 (0, 1035225153.25)	6379.34 (-2466.83, 15225.5)
2039	Male	428733309.65 (0, 1229560575.6)	6662.76 (-3374.16, 16699.68)
2040	Male	473825114.22 (0, 1475237693.79)	6962.66 (-4382.4, 18307.72)
2041	Male	526561314.37 (0, 1789082644.57)	7276.33 (-5497.92, 20050.58)
2042	Male	588668373.93 (0, 2195624269.82)	7604.45 (-6728.97, 21937.87)
2043	Male	662586584.96 (0, 2730870978.68)	7950.61 (-8087.3, 23988.52)
2044	Male	751310989.43 (0, 3446459812.85)	8317.28 (-9585.5, 26220.05)
2045	Male	858609618.1 (0, 4417335493.73)	8705.39 (-11235.8, 28646.57)
**YLDs**			
2022	Both	44271291.88 (41861895.37, 46680688.39)	533.17 (504.15, 562.2)

2023	Both	46759930.07 (42390649.32, 51129210.82)	557.07 (505.01, 609.13)
2024	Both	49444186.28 (42224465.36, 56663907.2)	582.85 (497.74, 667.96)
2025	Both	52356963.83 (41446884.49, 63267043.17)	610.88 (483.58, 738.17)
2026	Both	55509372.41 (40019821.98, 70998922.85)	641.24 (462.3, 820.17)
2027	Both	58948949.8 (37879283.55, 80018616.05)	674.4 (433.35, 915.45)
2028	Both	62739534.99 (34923471.31, 90555598.67)	711.19 (395.87, 1026.5)
2029	Both	66964561.9 (31002501.3, 102926622.5)	752.42 (348.34, 1156.5)
2030	Both	71699650.31 (25889474.93, 117509825.68)	798.87 (288.45, 1309.29)
2031	Both	76995735.7 (19264101.85, 134727369.54)	851.01 (212.91, 1489.11)
2032	Both	82961164.42 (10717510.57, 155204818.28)	909.92 (117.53, 1702.3)
2033	Both	89749994.65 (447439.27, 179809476.69)	977.28 (4.87, 1957.96)
2034	Both	97543375.38 (0, 209688028.53)	1054.96 (0, 2267.87)
2035	Both	106540768.47 (0, 246334654.99)	1144.97 (0, 2647.37)
2036	Both	116931160.18 (0, 291637390.44)	1249.19 (0, 3115.68)
2037	Both	129015042.35 (0, 348336263.88)	1370.63 (0, 3700.8)
2038	Both	143217847.11 (0, 420389588.72)	1513.67 (0, 4443.3)
2039	Both	160050811.6 (0, 513336245.69)	1683.56 (0, 5400.05)
2040	Both	180142617.92 (0, 635047364.13)	1886.68 (0, 6651.51)
2041	Both	204210346.09 (0, 796609623.52)	2130.3 (0, 8310.89)
2042	Both	233285960.18 (0, 1014659055.36)	2424.88 (0, 10547.97)
2043	Both	268811785.11 (0, 1314402536.5)	2785.08 (0, 13619.93)
2044	Both	312656722.12 (0, 1733765375.55)	3230.04 (0, 17914.33)
2045	Both	367287915.99 (0, 2330494571.7)	3784.87 (0, 24020.25)
2022	Female	27631088.67 (26104638.91, 29157538.43)	665.76 (637.92, 693.6)
2023	Female	29296741.39 (26529841.78, 32063641)	697.86 (636.91, 758.82)
2024	Female	31097616.03 (26513998.7, 35681233.37)	731.88 (627.15, 836.62)

2025	Female	33056237.3 (26106142.7, 40006331.9)	767.94 (608.76, 927.13)
2026	Female	35181898.55 (25278061.18, 45085735.91)	805.75 (581.03, 1030.46)
2027	Female	37506736.28 (23983520.9, 51029951.66)	845.41 (543.43, 1147.39)
2028	Female	40073980.41 (22150971.15, 57996989.67)	887.4 (495.45, 1279.35)
2029	Female	42939726.17 (19676095.97, 66203356.38)	932.06 (436.28, 1427.83)
2030	Female	46156541.34 (16403019.14, 75910063.55)	979.53 (364.83, 1594.24)
2031	Female	49762411.06 (12112251.09, 87412571.02)	1029.44 (279.71, 1779.17)
2032	Female	53832743.64 (6521419.82, 101144067.45)	1081.94 (179.7, 1984.17)
2033	Female	58473082.63 (0, 117702820.73)	1137.61 (63.5, 2211.73)
2034	Female	63809107.43 (0, 137883733.65)	1196.93 (-70.53, 2464.38)
2035	Female	69981165.73 (0, 162728566.51)	1260.08 (-224.24, 2744.4)
2036	Female	77126070.18 (0, 193564887.87)	1326.61 (-399.5, 3052.73)
2037	Female	85456640.66 (0, 232317576.37)	1396.75 (-598.3, 3391.8)
2038	Female	95270431.97 (0, 281765291.36)	1471.22 (-823.09, 3765.54)
2039	Female	106929588.62 (0, 345819166.83)	1550.61 (-1076.67, 4177.89)
2040	Female	120882432.35 (0, 430063223.47)	1635.19 (-1361.99, 4632.36)
2041	Female	137647100.39 (0, 542410908.92)	1724.48 (-1681.51, 5130.46)
2042	Female	157965149.35 (0, 694770792.53)	1818.77 (-2038.39, 5675.94)
2043	Female	182868387.93 (0, 905231478.62)	1918.99 (-2436.83, 6274.81)
2044	Female	213703585.37 (0, 1201146129.28)	2025.86 (-2881.35, 6933.08)
2045	Female	252257992.21 (0, 1624365358.28)	2139.76 (-3376.54, 7656.07)
2022	Male	16640203.21 (15757256.45, 17523149.96)	400.53 (384.22, 416.84)
2023	Male	17463188.68 (15860807.54, 19065569.82)	415.5 (380.06, 450.95)
2024	Male	18346570.24 (15710466.66, 20982673.83)	431.25 (370.94, 491.56)
2025	Male	19300726.53 (15340741.79, 23260711.27)	447.81 (357.07, 538.54)
2026	Male	20327473.87 (14741760.8, 25913186.94)	464.96 (338.2, 591.71)

2027	Male	21442213.51 (13895762.65, 28988664.38)	482.74 (314.18, 651.31)
2028	Male	22665554.58 (12772500.16, 32558609)	501.43 (284.93, 717.92)
2029	Male	24024835.73 (11326405.33, 36723266.13)	521.16 (250.17, 792.14)
2030	Male	25543108.96 (9486455.79, 41599762.14)	541.98 (209.5, 874.47)
2031	Male	27233324.64 (7151850.76, 47314798.52)	563.63 (162.36, 964.9)
2032	Male	29128420.79 (4196090.75, 54060750.83)	586.14 (108.33, 1063.96)
2033	Male	31276912.02 (447439.27, 62106655.96)	609.83 (46.97, 1172.69)
2034	Male	33734267.95 (0, 71804294.88)	634.88 (-22.31, 1292.08)
2035	Male	36559602.74 (0, 83606088.49)	661.37 (-100.2, 1422.94)
2036	Male	39805090 (0, 98072502.57)	688.96 (-187.35, 1565.27)
2037	Male	43558401.69 (0, 116018687.51)	717.72 (-284.41, 1719.85)
2038	Male	47947415.15 (0, 138624297.36)	748 (-392.28, 1888.27)
2039	Male	53121222.98 (0, 167517078.86)	780.04 (-511.94, 2072.01)
2040	Male	59260185.57 (0, 204984140.66)	813.91 (-644.42, 2272.25)
2041	Male	66563245.7 (0, 254198714.6)	849.29 (-790.45, 2489.02)
2042	Male	75320810.82 (0, 319888262.83)	886.22 (-951.02, 2723.45)
2043	Male	85943397.18 (0, 409171057.88)	925.13 (-1127.6, 2977.86)
2044	Male	98953136.75 (0, 532619246.26)	966.31 (-1321.79, 3254.4)
2045	Male	115029923.78 (0, 706129213.42)	1009.85 (-1535.06, 3554.76)

Notes: ASR: age-standardized rates; YLDs: years lived with disability.

Table S85. Prevalence and YLDs of opioid use disorders in the worldwide of future forecasts using bayesian age-period-cohort model.

Year	Sex	Number	ASR
**Prevalence**			
2022	Both	16189508.58 (14900009.74, 17479007.42)	271.64 (250.01, 293.27)
2023	Both	16523590.36 (15088145.33, 17959035.38)	274.92 (251.04, 298.81)
2024	Both	16868122.73 (15212644.05, 18523601.4)	278.39 (251.05, 305.72)
2025	Both	17224282.24 (15272721.92, 19175842.55)	282.02 (250.04, 313.99)
2026	Both	17593122.31 (15271101.73, 19915142.89)	285.8 (248.04, 323.57)
2027	Both	17978878.74 (15213893.63, 20743863.84)	289.78 (245.15, 334.41)
2028	Both	18383823.2 (15105935.53, 21661710.87)	294 (241.48, 346.51)
2029	Both	18806399.81 (14946904.93, 22665894.69)	298.44 (237.07, 359.82)
2030	Both	19246590.19 (14736123.22, 23757057.16)	303.11 (231.91, 374.31)
2031	Both	19704695.69 (14471735.34, 24937656.04)	308.01 (225.99, 390.02)
2032	Both	20184427.89 (14153364.38, 26215491.4)	313.17 (219.31, 407.03)
2033	Both	20685742.87 (13778304.59, 27593181.16)	318.65 (211.88, 425.41)
2034	Both	21210729.04 (13343705.18, 29077752.89)	324.43 (203.65, 445.22)
2035	Both	21758765.94 (12843938.35, 30673593.52)	330.54 (194.54, 466.53)
2036	Both	22329284.07 (12272510.68, 32386057.46)	336.98 (184.51, 489.46)
2037	Both	22927298.17 (11625039.07, 34229557.27)	343.81 (173.46, 514.15)
2038	Both	23544002.77 (10890393.8, 36197611.74)	351.06 (161.34, 540.78)
2039	Both	24188930.48 (10065204.55, 38312656.4)	358.76 (148.01, 569.51)
2040	Both	24863316.32 (9140179.06, 40586453.59)	366.93 (133.35, 600.5)
2041	Both	25568362.09 (8104458.56, 43032265.62)	375.6 (117.21, 634)
2042	Both	26308969.35 (6946919.04, 45671019.66)	384.84 (99.41, 670.27)
2043	Both	27084157.08 (5666631.77, 48515993.38)	394.7 (80.01, 709.64)

2044	Both	27897287.67 (4257358.75, 51588101.53)	405.23 (58.93, 752.44)
2045	Both	28751803.76 (2874137.89, 54912051.24)	416.46 (38.78, 799.02)
2022	Female	7952459.95 (7159442.86, 8745477.04)	266.05 (254.58, 277.51)
2023	Female	8173151.18 (7311223.02, 9035079.34)	271.05 (256.41, 285.69)
2024	Female	8399854.27 (7438815.43, 9360893.11)	276.22 (257.2, 295.23)
2025	Female	8633771.72 (7539397.69, 9728145.74)	281.54 (257.09, 305.99)
2026	Female	8876039.81 (7612017.85, 10140061.78)	287.01 (256.19, 317.84)
2027	Female	9128775.21 (7657846.11, 10599704.31)	292.65 (254.55, 330.74)
2028	Female	9392932.79 (7677897.79, 11107967.8)	298.47 (252.25, 344.68)
2029	Female	9667823.67 (7671431.5, 11664215.84)	304.46 (249.29, 359.64)
2030	Female	9953922.94 (7638134.76, 12269711.12)	310.63 (245.64, 375.61)
2031	Female	10251709.12 (7577113.35, 12926304.88)	316.95 (241.29, 392.62)
2032	Female	10562905.47 (7487934.72, 13637876.22)	323.45 (236.21, 410.69)
2033	Female	10887768.03 (7369113.13, 14406422.93)	330.14 (230.4, 429.89)
2034	Female	11227458.92 (7218957.07, 15235960.77)	337.02 (223.83, 450.22)
2035	Female	11581848.92 (7034407.21, 16129290.62)	344.08 (216.45, 471.7)
2036	Female	11950654.15 (6811868.98, 17089439.32)	351.31 (208.23, 494.39)
2037	Female	12336004.09 (6548698.05, 18123310.14)	358.73 (199.13, 518.33)
2038	Female	12734287.89 (6239239.76, 19229336.02)	366.35 (189.12, 543.57)
2039	Female	13150352.65 (5881465.24, 20419240.06)	374.17 (178.17, 570.17)
2040	Female	13584944.07 (5470302.23, 21699585.91)	382.18 (166.22, 598.15)
2041	Female	14038644.91 (4999843.36, 23077446.45)	390.39 (153.22, 627.57)
2042	Female	14513660.37 (4464113.37, 24563207.37)	398.81 (139.14, 658.49)
2043	Female	15009838.23 (3855158.02, 26164518.43)	407.46 (123.93, 690.98)
2044	Female	15529216.71 (3167234.94, 27892875.68)	416.32 (107.54, 725.1)
2045	Female	16073831.97 (2422680.93, 29761643.12)	425.4 (89.91, 760.9)

2022	Male	8237048.63 (7740566.89, 8733530.37)	275.76 (267.39, 284.13)
2023	Male	8350439.18 (7776922.31, 8923956.04)	277.22 (265.01, 289.43)
2024	Male	8468268.46 (7773828.62, 9162708.3)	278.81 (261.48, 296.13)
2025	Male	8590510.52 (7733324.23, 9447696.81)	280.49 (257.07, 303.92)
2026	Male	8717082.5 (7659083.88, 9775081.11)	282.23 (251.87, 312.6)
2027	Male	8850103.52 (7556047.52, 10144159.53)	284.07 (245.99, 322.15)
2028	Male	8990890.41 (7428037.74, 10553743.08)	286.03 (239.52, 332.54)
2029	Male	9138576.14 (7275473.43, 11001678.85)	288.09 (232.47, 343.72)
2030	Male	9292667.25 (7097988.46, 11487346.04)	290.23 (224.81, 355.65)
2031	Male	9452986.57 (6894621.99, 12011351.16)	292.4 (216.52, 368.28)
2032	Male	9621522.42 (6665429.66, 12577615.17)	294.66 (207.64, 381.68)
2033	Male	9797974.84 (6409191.46, 13186758.23)	297.02 (198.17, 395.86)
2034	Male	9983270.12 (6124748.12, 13841792.12)	299.45 (188.11, 410.8)
2035	Male	10176917.02 (5809531.14, 14544302.9)	301.94 (177.42, 426.46)
2036	Male	10378629.92 (5460641.7, 15296618.14)	304.47 (166.09, 442.86)
2037	Male	10591294.07 (5076341.02, 16106247.13)	307.06 (154.11, 460.02)
2038	Male	10809714.88 (4651154.04, 16968275.71)	309.74 (141.5, 477.99)
2039	Male	11038577.83 (4183739.31, 17893416.34)	312.49 (128.22, 496.75)
2040	Male	11278372.25 (3669876.83, 18886867.68)	315.28 (114.27, 516.29)
2041	Male	11529717.18 (3104615.2, 19954819.17)	318.12 (99.62, 536.61)
2042	Male	11795308.98 (2482805.68, 21107812.29)	321.02 (84.27, 557.76)
2043	Male	12074318.86 (1811473.74, 22351474.95)	323.99 (68.21, 579.77)
2044	Male	12368070.96 (1090123.81, 23695225.85)	327.03 (51.41, 602.64)
2045	Male	12677971.78 (451456.96, 25150408.12)	330.12 (33.87, 626.36)
**YLDs**			
2022	Both	6638776.51 (6127382.52, 7150170.5)	111.55 (102.95, 120.14)

2023	Both	6763967.79 (6192228.25, 7335707.33)	112.72 (103.19, 122.25)
2024	Both	6893048.83 (6230160.38, 7555937.29)	113.97 (103.01, 124.94)
2025	Both	7026454.84 (6241392.79, 7811516.89)	115.28 (102.4, 128.17)
2026	Both	7164622.89 (6227422.11, 8101823.68)	116.66 (101.38, 131.93)
2027	Both	7309317.98 (6190982.51, 8427653.44)	118.11 (100.01, 136.2)
2028	Both	7461490.25 (6134220.68, 8788759.82)	119.65 (98.33, 140.97)
2029	Both	7620361.74 (6056992.86, 9183730.61)	121.28 (96.35, 146.21)
2030	Both	7785849.68 (5959057.61, 9612641.74)	123 (94.07, 151.92)
2031	Both	7958080.1 (5839748.03, 10076412.16)	124.81 (91.5, 158.11)
2032	Both	8138576.67 (5698999.51, 10578153.83)	126.72 (88.62, 164.81)
2033	Both	8327264.57 (5535779.1, 11118750.04)	128.75 (85.45, 172.04)
2034	Both	8524893.6 (5348977.86, 11700809.34)	130.9 (81.96, 179.84)
2035	Both	8731146.59 (5136400.26, 12325892.92)	133.18 (78.13, 188.22)
2036	Both	8945802.76 (4895556.15, 12996049.38)	135.58 (73.93, 197.23)
2037	Both	9170907.34 (4624810.42, 13717004.26)	138.13 (69.33, 206.94)
2038	Both	9402758.88 (4319745.01, 14485772.76)	140.85 (64.31, 217.4)
2039	Both	9645138.64 (3979134.83, 15311142.45)	143.75 (58.82, 228.68)
2040	Both	9898497.83 (3599400.24, 16197595.43)	146.82 (52.8, 240.84)
2041	Both	10163340.61 (3176373.09, 17150308.12)	150.09 (46.2, 253.97)
2042	Both	10441618.25 (2705768.1, 18177468.4)	153.57 (38.95, 268.2)
2043	Both	10732893.32 (2187201.33, 19284128.78)	157.3 (31.08, 283.62)
2044	Both	11038325.18 (1620062.32, 20478109.75)	161.28 (22.57, 300.39)
2045	Both	11359230.35 (1066021.94, 21768928.74)	165.54 (14.47, 318.63)
2022	Female	3213261.03 (2897454.45, 3529067.61)	107.74 (103.12, 112.37)
2023	Female	3296586.85 (2953154.5, 3640019.2)	109.6 (103.69, 115.52)
2024	Female	3381996.3 (2998581.07, 3765411.53)	111.53 (103.82, 119.23)

2025	Female	3469992.89 (3032721.63, 3907264.15)	113.5 (103.6, 123.41)
2026	Female	3561084.92 (3055372.92, 4066796.92)	115.53 (103.04, 128.02)
2027	Female	3656143.73 (3067149.22, 4245138.24)	117.63 (102.2, 133.05)
2028	Female	3755487.79 (3068509.75, 4442465.83)	119.79 (101.09, 138.48)
2029	Female	3858771.89 (3059183.49, 4658360.3)	122.01 (99.71, 144.31)
2030	Female	3966178.54 (3039111.55, 4893245.54)	124.29 (98.05, 150.53)
2031	Female	4077926.74 (3008020.72, 5147832.77)	126.63 (96.12, 157.15)
2032	Female	4194710.4 (2965797.23, 5423623.57)	129.04 (93.9, 164.17)
2033	Female	4316542.93 (2911843.92, 5721241.94)	131.51 (91.39, 171.63)
2034	Female	4443842.38 (2845517.74, 6042167.02)	134.05 (88.58, 179.52)
2035	Female	4576543.2 (2765659.52, 6387426.88)	136.65 (85.46, 187.85)
2036	Female	4714553.21 (2670922.61, 6758183.81)	139.32 (82, 196.63)
2037	Female	4858724.66 (2560326.47, 7157122.86)	142.05 (78.2, 205.89)
2038	Female	5007501.21 (2431638.7, 7583363.71)	144.85 (74.05, 215.65)
2039	Female	5162772.84 (2284120.8, 8041424.89)	147.72 (69.54, 225.91)
2040	Female	5324827.62 (2115852.49, 8533802.76)	150.66 (64.64, 236.69)
2041	Female	5493922.3 (1924602.54, 9063242.06)	153.67 (59.33, 248.01)
2042	Female	5670919.13 (1708098.06, 9633740.21)	156.75 (53.61, 259.89)
2043	Female	5855689.99 (1463272.41, 10248107.58)	159.91 (47.46, 272.36)
2044	Female	6048938.79 (1188742.4, 10910593.73)	163.14 (40.85, 285.43)
2045	Female	6251451.48 (892418.71, 11626311.09)	166.45 (33.78, 299.13)
2022	Male	3425515.48 (3229928.07, 3621102.9)	114.77 (111.4, 118.15)
2023	Male	3467380.94 (3239073.75, 3695688.13)	115.22 (110.23, 120.21)
2024	Male	3511052.53 (3231579.31, 3790525.75)	115.73 (108.6, 122.86)
2025	Male	3556461.95 (3208671.17, 3904252.74)	116.27 (106.61, 125.94)
2026	Male	3603537.97 (3172049.19, 4035026.76)	116.84 (104.3, 129.38)

2027	Male	3653174.24 (3123833.29, 4182515.2)	117.44 (101.72, 133.17)
2028	Male	3706002.46 (3065710.93, 4346293.99)	118.1 (98.9, 137.3)
2029	Male	3761589.84 (2997809.38, 4525370.31)	118.8 (95.85, 141.75)
2030	Male	3819671.13 (2919946.06, 4719396.2)	119.53 (92.56, 146.5)
2031	Male	3880153.35 (2831727.31, 4928579.39)	120.27 (89.02, 151.52)
2032	Male	3943866.27 (2733202.28, 5154530.26)	121.04 (85.24, 156.85)
2033	Male	4010721.64 (2623935.19, 5397508.1)	121.86 (81.23, 162.49)
2034	Male	4081051.22 (2503460.12, 5658642.32)	122.71 (76.98, 168.43)
2035	Male	4154603.39 (2370740.74, 5938466.05)	123.57 (72.49, 174.65)
2036	Male	4231249.55 (2224633.54, 6237865.56)	124.45 (67.75, 181.15)
2037	Male	4312182.67 (2064483.95, 6559881.4)	125.35 (62.75, 187.96)
2038	Male	4395257.68 (1888106.3, 6902409.05)	126.29 (57.5, 195.08)
2039	Male	4482365.79 (1695014.03, 7269717.56)	127.25 (52, 202.51)
2040	Male	4573670.21 (1483547.75, 7663792.67)	128.23 (46.23, 210.23)
2041	Male	4669418.31 (1251770.55, 8087066.06)	129.23 (40.2, 218.26)
2042	Male	4770699.12 (997670.04, 8543728.19)	130.25 (33.89, 226.6)
2043	Male	4877203.33 (723928.93, 9036021.2)	131.29 (27.3, 235.28)
2044	Male	4989386.39 (431319.93, 9567516.02)	132.36 (20.44, 244.28)
2045	Male	5107778.87 (173603.23, 10142617.65)	133.45 (13.29, 253.61)

Notes: ASR: age-standardized rates; YLDs: years lived with disability.

Table S86. Prevalence and YLDs of varicella and herpes zoster in the worldwide of future forecasts using bayesian age-period-cohort model.

Year	Sex	Number	ASR
**Prevalence**			
2022	Both	5416863.79 (5299127.79, 5534599.78)	66.88 (65.41, 68.35)
2023	Both	5487430.14 (5340067.8, 5634792.48)	66.85 (65.03, 68.67)
2024	Both	5558457.05 (5365951.41, 5750962.7)	66.82 (64.47, 69.17)
2025	Both	5629225.95 (5378484.28, 5879967.61)	66.79 (63.76, 69.81)
2026	Both	5699582.78 (5379515.41, 6019650.16)	66.76 (62.95, 70.57)
2027	Both	5769049.14 (5369825.98, 6168272.31)	66.74 (62.04, 71.43)
2028	Both	5838678.42 (5351405.33, 6325951.51)	66.71 (61.05, 72.38)
2029	Both	5909270.58 (5325552.3, 6492988.85)	66.7 (59.99, 73.4)
2030	Both	5980427.88 (5292190.69, 6668665.07)	66.68 (58.87, 74.5)
2031	Both	6052034.94 (5251306.37, 6852763.51)	66.68 (57.69, 75.66)
2032	Both	6123509.22 (5202363.29, 7044655.15)	66.68 (56.46, 76.9)
2033	Both	6195332.33 (5145935.06, 7244729.59)	66.69 (55.17, 78.2)
2034	Both	6268310.92 (5082738.02, 7453883.82)	66.7 (53.83, 79.57)
2035	Both	6342022.59 (5012359.17, 7671686.01)	66.72 (52.45, 81)
2036	Both	6416291.19 (4934506.57, 7898075.81)	66.76 (51.02, 82.5)
2037	Both	6490530.16 (4848511.3, 8132549.03)	66.8 (49.54, 84.06)
2038	Both	6565084.94 (4754623.01, 8375546.86)	66.86 (48.02, 85.7)
2039	Both	6640671.62 (4653279.01, 8628064.22)	66.93 (46.45, 87.4)
2040	Both	6716829.95 (4543975.09, 8889684.82)	67.01 (44.84, 89.17)
2041	Both	6793436.68 (4426383.68, 9160489.69)	67.1 (43.18, 91.02)
2042	Both	6869936.65 (4299837.43, 9440035.87)	67.21 (41.47, 92.95)
2043	Both	6946606.18 (4164403.52, 9728808.85)	67.33 (39.71, 94.96)

2044	Both	7024142.12 (4020329.15, 10027955.08)	67.47 (37.9, 97.04)
2045	Both	7102119.95 (3867102.45, 10337137.46)	67.63 (36.04, 99.21)
2022	Female	2866675.22 (2801729.62, 2931620.81)	69.56 (68.76, 70.36)
2023	Female	2906029.27 (2824656.37, 2987402.18)	69.53 (68.18, 70.87)
2024	Female	2945707.67 (2839200.76, 3052214.58)	69.49 (67.46, 71.52)
2025	Female	2985342.82 (2846345.53, 3124340.12)	69.46 (66.63, 72.28)
2026	Female	3024882.61 (2847152.5, 3202612.72)	69.42 (65.71, 73.13)
2027	Female	3064077.27 (2842066.27, 3286088.27)	69.38 (64.71, 74.06)
2028	Female	3103420.85 (2832091.39, 3374750.31)	69.35 (63.62, 75.07)
2029	Female	3143345.74 (2817924.46, 3468767.03)	69.3 (62.47, 76.14)
2030	Female	3183642.13 (2799522.37, 3567761.9)	69.26 (61.25, 77.27)
2031	Female	3224269.12 (2776886.79, 3671651.46)	69.22 (59.98, 78.46)
2032	Female	3264910.08 (2749716.03, 3780104.13)	69.18 (58.64, 79.72)
2033	Female	3305787.73 (2718274.28, 3893301.18)	69.14 (57.25, 81.02)
2034	Female	3347324.11 (2682920.98, 4011727.23)	69.09 (55.8, 82.38)
2035	Female	3389287.87 (2643418.71, 4135157.04)	69.04 (54.3, 83.78)
2036	Female	3431592 (2599602.19, 4263581.81)	69 (52.75, 85.24)
2037	Female	3473915.45 (2551094.29, 4396736.61)	68.95 (51.16, 86.74)
2038	Female	3516439.25 (2498008.59, 4534869.92)	68.91 (49.52, 88.29)
2039	Female	3559558.73 (2440564.93, 4678552.52)	68.86 (47.83, 89.88)
2040	Female	3603020.51 (2378471.18, 4827569.83)	68.81 (46.1, 91.51)
2041	Female	3646752.89 (2311526.31, 4981979.48)	68.76 (44.34, 93.18)
2042	Female	3690447.53 (2239347.28, 5141547.79)	68.71 (42.52, 94.89)
2043	Female	3734260.15 (2161951.71, 5306568.59)	68.66 (40.67, 96.65)
2044	Female	3778595.96 (2079458.7, 5477733.23)	68.61 (38.78, 98.43)
2045	Female	3823241.29 (1991575.41, 5654907.17)	68.56 (36.85, 100.26)

2022	Male	2550188.57 (2497398.16, 2602978.98)	64.05 (63.38, 64.73)
2023	Male	2581400.86 (2515411.42, 2647390.3)	64.02 (62.9, 65.14)
2024	Male	2612749.38 (2526750.65, 2698748.12)	63.98 (62.3, 65.66)
2025	Male	2643883.12 (2532138.75, 2755627.5)	63.94 (61.61, 66.28)
2026	Male	2674700.17 (2532362.9, 2817037.44)	63.9 (60.84, 66.96)
2027	Male	2704971.88 (2527759.71, 2882184.04)	63.86 (60.01, 67.72)
2028	Male	2735257.57 (2519313.94, 2951201.2)	63.82 (59.11, 68.54)
2029	Male	2765924.83 (2507627.84, 3024221.82)	63.78 (58.15, 69.41)
2030	Male	2796785.75 (2492668.33, 3100903.17)	63.73 (57.14, 70.32)
2031	Male	2827765.81 (2474419.58, 3181112.04)	63.69 (56.08, 71.29)
2032	Male	2858599.14 (2452647.26, 3264551.02)	63.64 (54.98, 72.31)
2033	Male	2889544.6 (2427660.79, 3351428.41)	63.6 (53.82, 73.37)
2034	Male	2920986.82 (2399817.04, 3442156.59)	63.55 (52.62, 74.47)
2035	Male	2952734.72 (2368940.46, 3536528.97)	63.49 (51.38, 75.61)
2036	Male	2984699.19 (2334904.38, 3634494)	63.44 (50.1, 76.78)
2037	Male	3016614.71 (2297417, 3735812.41)	63.39 (48.78, 78)
2038	Male	3048645.68 (2256614.43, 3840676.94)	63.34 (47.42, 79.25)
2039	Male	3081112.89 (2212714.08, 3949511.69)	63.28 (46.03, 80.54)
2040	Male	3113809.44 (2165503.91, 4062114.98)	63.22 (44.59, 81.85)
2041	Male	3146683.79 (2114857.37, 4178510.21)	63.17 (43.13, 83.2)
2042	Male	3179489.12 (2060490.16, 4298488.09)	63.11 (41.63, 84.59)
2043	Male	3212346.04 (2002451.81, 4422240.26)	63.05 (40.1, 86)
2044	Male	3245546.15 (1940870.45, 4550221.86)	62.99 (38.54, 87.44)
2045	Male	3278878.66 (1875527.04, 4682230.28)	62.93 (36.95, 88.91)
**YLDs**			
2022	Both	230815.52 (222804.1, 238826.95)	2.71 (2.61, 2.8)

2023	Both	234426.21 (225242.15, 243610.28)	2.7 (2.59, 2.81)
2024	Both	238096.19 (227042.91, 249149.48)	2.7 (2.57, 2.83)
2025	Both	241790.48 (228207.72, 255373.23)	2.7 (2.54, 2.85)
2026	Both	245478.09 (228773.74, 262182.44)	2.69 (2.51, 2.88)
2027	Both	249135.54 (228778.36, 269492.73)	2.69 (2.47, 2.91)
2028	Both	252800.55 (228310.51, 277290.58)	2.69 (2.43, 2.95)
2029	Both	256520.07 (227445.38, 285594.76)	2.68 (2.38, 2.99)
2030	Both	260264.19 (226172.6, 294355.79)	2.68 (2.33, 3.04)
2031	Both	264007.11 (224475.68, 303538.53)	2.68 (2.28, 3.08)
2032	Both	267726.68 (222337.66, 313115.7)	2.68 (2.22, 3.14)
2033	Both	271457.39 (219796.61, 323118.17)	2.68 (2.17, 3.19)
2034	Both	275242.71 (216889.26, 333596.16)	2.68 (2.11, 3.25)
2035	Both	279055.8 (213588.85, 344522.75)	2.68 (2.05, 3.31)
2036	Both	282869.68 (209865.41, 355873.94)	2.68 (1.98, 3.37)
2037	Both	286662.48 (205692.79, 367632.18)	2.68 (1.92, 3.44)
2038	Both	290469.29 (201094.84, 379843.74)	2.68 (1.85, 3.51)
2039	Both	294335.09 (196095.26, 392574.93)	2.68 (1.78, 3.58)
2040	Both	298235.38 (190664.04, 405806.71)	2.68 (1.71, 3.66)
2041	Both	302145.05 (184768.71, 419521.39)	2.69 (1.64, 3.74)
2042	Both	306044.83 (178380.57, 433709.1)	2.69 (1.56, 3.82)
2043	Both	309967.35 (171509.54, 448425.16)	2.69 (1.48, 3.91)
2044	Both	313955.73 (164164.32, 463747.14)	2.7 (1.4, 4)
2045	Both	317990.42 (156313, 479667.84)	2.71 (1.32, 4.09)
2022	Female	123900.77 (119680.4, 128121.15)	2.83 (2.79, 2.87)
2023	Female	125833.94 (120958.23, 130709.64)	2.83 (2.76, 2.89)
2024	Female	127800.19 (121878.1, 133722.27)	2.82 (2.73, 2.92)

2025	Female	129780.51 (122445.81, 137115.21)	2.82 (2.69, 2.95)
2026	Female	131758.26 (122685.06, 140831.46)	2.81 (2.64, 2.98)
2027	Female	133722.76 (122619.22, 144826.3)	2.81 (2.59, 3.02)
2028	Female	135694.47 (122296.73, 149092.21)	2.8 (2.54, 3.06)
2029	Female	137697.56 (121756.84, 153638.28)	2.8 (2.49, 3.11)
2030	Female	139715.4 (120993.24, 158437.57)	2.79 (2.43, 3.15)
2031	Female	141733.44 (119996.07, 163470.82)	2.79 (2.37, 3.2)
2032	Female	143741.42 (118757.17, 168725.67)	2.78 (2.31, 3.25)
2033	Female	145757.85 (117296.55, 174219.15)	2.78 (2.25, 3.31)
2034	Female	147804.89 (115632.43, 179977.34)	2.77 (2.18, 3.36)
2035	Female	149866.78 (113749.04, 185984.53)	2.77 (2.12, 3.42)
2036	Female	151927.75 (111628.98, 192226.52)	2.76 (2.05, 3.48)
2037	Female	153977.9 (109259.19, 198696.62)	2.76 (1.98, 3.54)
2038	Female	156037.05 (106652.65, 205421.45)	2.75 (1.9, 3.6)
2039	Female	158128.81 (103820.96, 212436.66)	2.75 (1.83, 3.67)
2040	Female	160238.19 (100746.3, 219730.09)	2.74 (1.75, 3.73)
2041	Female	162349.49 (97409.39, 227289.59)	2.74 (1.67, 3.8)
2042	Female	164454.18 (93795.54, 235112.82)	2.73 (1.59, 3.87)
2043	Female	166571.38 (89910.3, 243232.46)	2.73 (1.51, 3.94)
2044	Female	168724.58 (85757.43, 251691.74)	2.72 (1.43, 4.02)
2045	Female	170902.14 (81318.4, 260485.87)	2.72 (1.35, 4.09)
2022	Male	106914.75 (103123.69, 110705.81)	2.57 (2.54, 2.61)
2023	Male	108592.28 (104283.92, 112900.64)	2.57 (2.51, 2.63)
2024	Male	110296.01 (105164.81, 115427.21)	2.57 (2.48, 2.65)
2025	Male	112009.96 (105761.91, 118258.02)	2.57 (2.45, 2.68)
2026	Male	113719.83 (106088.69, 121350.98)	2.56 (2.42, 2.71)

2027	Male	115412.78 (106159.14, 124666.43)	2.56 (2.38, 2.75)
2028	Male	117106.08 (106013.78, 128198.37)	2.56 (2.34, 2.78)
2029	Male	118822.51 (105688.54, 131956.48)	2.56 (2.29, 2.82)
2030	Male	120548.79 (105179.36, 135918.22)	2.56 (2.24, 2.87)
2031	Male	122273.66 (104479.61, 140067.72)	2.55 (2.2, 2.91)
2032	Male	123985.26 (103580.49, 144390.03)	2.55 (2.14, 2.96)
2033	Male	125699.54 (102500.06, 148899.02)	2.55 (2.09, 3)
2034	Male	127437.83 (101256.83, 153618.82)	2.55 (2.04, 3.05)
2035	Male	129189.02 (99839.81, 158538.23)	2.54 (1.98, 3.11)
2036	Male	130941.93 (98236.44, 163647.42)	2.54 (1.92, 3.16)
2037	Male	132684.58 (96433.6, 168935.56)	2.54 (1.86, 3.22)
2038	Male	134432.24 (94442.19, 174422.29)	2.54 (1.8, 3.27)
2039	Male	136206.29 (92274.31, 180138.27)	2.53 (1.74, 3.33)
2040	Male	137997.18 (89917.74, 186076.62)	2.53 (1.67, 3.39)
2041	Male	139795.56 (87359.32, 192231.81)	2.53 (1.6, 3.45)
2042	Male	141590.65 (84585.02, 198596.28)	2.53 (1.54, 3.51)
2043	Male	143395.97 (81599.24, 205192.7)	2.52 (1.47, 3.58)
2044	Male	145231.15 (78406.89, 212055.4)	2.52 (1.4, 3.64)
2045	Male	147088.29 (74994.6, 219181.97)	2.52 (1.32, 3.71)

Notes: ASR: age-standardized rates; YLDs: years lived with disability.

Table S87. Acupuncture demands based on prevalence and YLDs in China of future forecasts using bayesian age-period-cohort model.

Year	Sex	Number	ASR
**Prevalence**			
2022	Both	1101642654.84 (1076603040.31, 1126682269.37)	62825.33 (61395.87, 64254.79)
2023	Both	1116953469.3 (1086537733.36, 1147369205.23)	63189.45 (61466.87, 64912.04)
2024	Both	1132124911.42 (1093137211.29, 1171112611.54)	63550.92 (61360.09, 65741.75)
2025	Both	1147161974.12 (1096831925.37, 1197492022.86)	63910.78 (61103.88, 66717.68)
2026	Both	1162016553.67 (1097991682.79, 1226041424.55)	64267.47 (60722.59, 67812.34)
2027	Both	1176800251.11 (1096995318.23, 1256605183.99)	64618.27 (60230.95, 69005.58)
2028	Both	1190345902.56 (1092999309.03, 1287692496.09)	64962.61 (59643.08, 70282.14)
2029	Both	1203215540.26 (1086698058.04, 1319733022.47)	65298.08 (58965.86, 71630.29)
2030	Both	1215569642.73 (1078340008.03, 1352799277.42)	65626.19 (58206.14, 73046.23)
2031	Both	1227551768.42 (1068104236.67, 1386999300.18)	65950.96 (57370.14, 74531.78)
2032	Both	1239387454.23 (1056204330.75, 1422570577.71)	66271.69 (56458.3, 76085.07)
2033	Both	1250473821.57 (1042192995.64, 1458754647.51)	66587.37 (55473.37, 77701.37)
2034	Both	1261059002.64 (1026342386.91, 1495775618.36)	66894.8 (54415.16, 79374.44)
2035	Both	1271250278.16 (1008776377.13, 1533724179.18)	67195.73 (53286.52, 81104.93)
2036	Both	1281158947.22 (989591281.01, 1572726613.42)	67496.43 (52092.39, 82900.47)
2037	Both	1290932108.84 (968884568.74, 1612979648.95)	67796.66 (50831.42, 84761.91)
2038	Both	1300122998.98 (946353622.13, 1653892375.82)	68095.63 (49504.2, 86687.05)
2039	Both	1308964204.88 (922205311.19, 1695723098.57)	68389.9 (48108.91, 88670.89)
2040	Both	1317477788.99 (896469855.13, 1738485722.85)	68681.02 (46646.69, 90715.35)
2041	Both	1325702453.1 (869167772.51, 1782237133.68)	68976.43 (45121.42, 92831.44)
2042	Both	1333684622.43 (840303788.14, 1827065456.72)	69275.1 (43530.06, 95020.15)
2043	Both	1340836910.29 (809536538.05, 1872137282.53)	69575.5 (41871.34, 97279.66)

2044	Both	1347467186.17 (777097459.54, 1917836912.8)	69873.65 (40142.25, 99605.04)
2045	Both	1353690852.34 (743070603.53, 1964311101.15)	70170.78 (38342.46, 101999.09)
2022	Female	652572316.07 (637351077.31, 667793554.82)	74443.41 (73619.64, 75267.18)
2023	Female	662335342.4 (644035558.65, 680635126.16)	74901.35 (73522.52, 76280.18)
2024	Female	672004599.37 (648784494.87, 695224703.87)	75352.45 (73265.19, 77439.71)
2025	Female	681571566.35 (651804987.09, 711338145.61)	75798.64 (72884.33, 78712.96)
2026	Female	691016894.03 (653310175.36, 728723612.7)	76241.57 (72396.32, 80086.82)
2027	Female	700372025.02 (653489597.32, 747254452.71)	76674.32 (71801.76, 81546.88)
2028	Female	708978086.86 (651872226.82, 766083946.91)	77093 (71105.46, 83080.55)
2029	Female	717142318.9 (648845794.57, 785438843.23)	77495.1 (70311.14, 84679.05)
2030	Female	724986682.2 (644582786.57, 805390577.83)	77882.82 (69424.98, 86340.67)
2031	Female	732639628.09 (639227535.28, 826051720.91)	78263.17 (68455.41, 88070.93)
2032	Female	740234864.43 (632905643.3, 847564085.56)	78630.43 (67398.32, 89862.54)
2033	Female	747388472.97 (625325530.53, 869451415.41)	78980.05 (66253.84, 91706.26)
2034	Female	754219922.71 (616624588.73, 891815256.69)	79308.77 (65022.79, 93594.74)
2035	Female	760814492.04 (606893886.7, 914735097.38)	79618.84 (63709.8, 95527.89)
2036	Female	767279336.04 (596221783.84, 938336888.24)	79919.92 (62323.91, 97515.93)
2037	Female	773697154.85 (584660400.94, 962733908.76)	80206.47 (60861.1, 99551.85)
2038	Female	779769034.95 (572000591.6, 987537478.3)	80473.9 (59320.98, 101626.82)
2039	Female	785615839.12 (558352368.43, 1012879309.82)	80718.54 (57703.87, 103733.21)
2040	Female	791252323.31 (543734994.57, 1038769652.05)	80942.61 (56013.91, 105871.31)
2041	Female	796719506.11 (528172787.48, 1065266224.75)	81157.48 (54260.25, 108054.71)
2042	Female	802012597.51 (511647980.18, 1092377214.83)	81357.32 (52439.23, 110275.42)
2043	Female	806750035 (493936683.37, 1119563386.64)	81536.88 (50550.27, 112523.49)
2044	Female	811120704.41 (475185241.66, 1147056167.17)	81691.4 (48593.36, 114789.44)
2045	Female	815209491.51 (455457077.89, 1174961905.13)	81822.91 (46572.38, 117073.44)

2022	Male	449070338.77 (439251962.99, 458888714.55)	51457.54 (50900.29, 52014.79)
2023	Male	454618126.89 (442502174.72, 466734079.07)	51731.53 (50766.89, 52696.18)
2024	Male	460120312.05 (444352716.42, 475887907.68)	52005.22 (50524.61, 53485.83)
2025	Male	465590407.77 (445026938.29, 486153877.26)	52278.53 (50196.94, 54360.13)
2026	Male	470999659.64 (444681507.43, 497317811.85)	52545.66 (49788.29, 55303.02)
2027	Male	476428226.1 (443505720.91, 509350731.28)	52804.24 (49301.48, 56307)
2028	Male	481367815.7 (441127082.21, 521608549.18)	53055.14 (48743.71, 57366.57)
2029	Male	486073221.36 (437852263.47, 534294179.24)	53296.7 (48117.99, 58475.42)
2030	Male	490582960.53 (433757221.46, 547408699.59)	53529.16 (47427.53, 59630.79)
2031	Male	494912140.33 (428876701.39, 560947579.26)	53751.8 (46673.4, 60830.2)
2032	Male	499152589.8 (423298687.45, 575006492.15)	53964.24 (45856.07, 62072.42)
2033	Male	503085348.6 (416867465.11, 589303232.09)	54167.12 (44979.22, 63355.03)
2034	Male	506839079.92 (409717798.18, 603960361.67)	54358.27 (44043.66, 64672.87)
2035	Male	510435786.12 (401882490.43, 618989081.8)	54537.84 (43051.49, 66024.2)
2036	Male	513879611.18 (393369497.17, 634389725.19)	54706.52 (42004.31, 67408.72)
2037	Male	517234954 (384224167.8, 650245740.19)	54864.26 (40902.5, 68826.01)
2038	Male	520353964.03 (374353030.53, 666354897.52)	55011.77 (39748.95, 70274.59)
2039	Male	523348365.75 (363852942.76, 682843788.75)	55146.69 (38544.09, 71749.29)
2040	Male	526225465.68 (352734860.55, 699716070.8)	55269.12 (37289.73, 73248.52)
2041	Male	528982946.98 (340994985.04, 716970908.93)	55380.83 (35988.07, 74773.58)
2042	Male	531672024.92 (328655807.96, 734688241.89)	55481.58 (34639.37, 76323.8)
2043	Male	534086875.29 (315599854.69, 752573895.89)	55571.22 (33245.61, 77896.83)
2044	Male	536346481.75 (301912217.88, 770780745.63)	55646.52 (31806.9, 79486.15)
2045	Male	538481360.83 (287613525.65, 789349196.02)	55707.54 (30325.08, 81090)
**YLDs**			
2022	Both	57106173.44 (54474937.1, 59737409.78)	3070.75 (2929.12, 3212.37)

2023	Both	58182662 (55269517.54, 61095806.45)	3088.08 (2933.3, 3242.85)
2024	Both	59283556.75 (55928363.61, 62638749.89)	3105.34 (2929.41, 3281.28)
2025	Both	60399779.9 (56435971.73, 64363588.08)	3122.65 (2917.52, 3327.78)
2026	Both	61517861.63 (56785979.95, 66249743.32)	3139.96 (2898.21, 3381.72)
2027	Both	62630618.23 (56982759.45, 68278477.02)	3157.13 (2872.16, 3442.11)
2028	Both	63720555.92 (57023096.66, 70418015.17)	3174.3 (2840.34, 3508.26)
2029	Both	64822462.96 (56947266.04, 72697659.89)	3191.51 (2803.4, 3579.62)
2030	Both	65927274.14 (56751964.71, 75102583.56)	3208.85 (2761.81, 3655.89)
2031	Both	67021400.5 (56427888.59, 77614912.41)	3226.41 (2715.88, 3736.93)
2032	Both	68096533.83 (55969830.12, 80223237.55)	3244.05 (2665.67, 3822.43)
2033	Both	69143460.57 (55373654.39, 82913266.74)	3261.89 (2611.47, 3912.32)
2034	Both	70196221.97 (54668342.71, 85724101.23)	3279.95 (2553.4, 4006.51)
2035	Both	71245250.46 (53845455.91, 88645045.02)	3298.31 (2491.56, 4105.06)
2036	Both	72275668.92 (52892273.18, 91659064.67)	3317.15 (2426.06, 4208.24)
2037	Both	73274597.6 (51798553.89, 94750641.32)	3336.36 (2356.73, 4315.98)
2038	Both	74246206.93 (50568604.45, 97923809.41)	3356.02 (2283.65, 4428.4)
2039	Both	75221534.37 (49223211.42, 101219857.31)	3376.15 (2206.75, 4545.54)
2040	Both	76191967.22 (47753952.04, 104629982.39)	3396.83 (2126.01, 4667.64)
2041	Both	77143925.4 (46149303, 108138547.79)	3418.3 (2041.41, 4795.19)
2042	Both	78063696.79 (44398824.59, 111728568.98)	3440.46 (1952.69, 4928.24)
2043	Both	78960617.14 (42507788.61, 115413445.66)	3463.39 (1859.75, 5067.04)
2044	Both	79861822.05 (40488732.67, 119234911.43)	3487.05 (1762.39, 5211.71)
2045	Both	80756808.01 (38331858.08, 123181757.93)	3511.54 (1660.46, 5362.62)
2022	Female	33823722.76 (32209195.54, 35438249.98)	3620.69 (3552.5, 3688.87)
2023	Female	34522832.98 (32734271.64, 36311394.32)	3644.54 (3545, 3744.08)
2024	Female	35237250.58 (33177300.68, 37297200.48)	3668.21 (3527.82, 3808.61)

2025	Female	35962542.71 (33529251.14, 38395834.28)	3691.77 (3503.17, 3880.38)
2026	Female	36690508.92 (33785562.19, 39595455.65)	3715.08 (3472.04, 3958.13)
2027	Female	37417781.03 (33949229.71, 40886332.35)	3737.84 (3434.77, 4040.91)
2028	Female	38133026.64 (34017175.51, 42248877.78)	3760.15 (3391.99, 4128.32)
2029	Female	38856531.8 (34012672.91, 43700390.69)	3782.1 (3344.1, 4220.11)
2030	Female	39584460.42 (33934867.33, 45234053.52)	3803.78 (3291.38, 4316.18)
2031	Female	40308999.53 (33778133.77, 46839865.29)	3825.22 (3233.98, 4416.46)
2032	Female	41026232.75 (33539857.8, 48512607.7)	3846.16 (3171.76, 4520.56)
2033	Female	41730616.2 (33217095.24, 50244137.16)	3866.62 (3104.93, 4628.31)
2034	Female	42441777.35 (32826026.29, 52057528.42)	3886.65 (3033.67, 4739.62)
2035	Female	43153306.82 (32360627.24, 53945986.4)	3906.32 (2958.14, 4854.5)
2036	Female	43854582.99 (31811603.43, 55897562.55)	3925.83 (2878.52, 4973.15)
2037	Female	44536910.05 (31171823.27, 57901996.83)	3944.92 (2794.63, 5095.21)
2038	Female	45203147.71 (30443823.49, 59962471.94)	3963.56 (2706.6, 5220.52)
2039	Female	45872349.05 (29639897.51, 62104800.58)	3981.74 (2614.51, 5348.97)
2040	Female	46538892.31 (28754345.51, 64323439.12)	3999.56 (2518.5, 5480.62)
2041	Female	47192941.67 (27778907.79, 66606975.56)	4017.29 (2418.75, 5615.82)
2042	Female	47825223.66 (26706542.28, 68943905.03)	4034.66 (2315.11, 5754.21)
2043	Female	48441737.41 (25540444.82, 71343029.99)	4051.59 (2207.63, 5895.56)
2044	Female	49059590.56 (24288181.13, 73830999.99)	4068.02 (2096.36, 6039.67)
2045	Female	49673644.15 (22944179, 76403109.3)	4084.03 (1981.41, 6186.65)
2022	Male	23282450.68 (22265741.57, 24299159.8)	2527.34 (2483.42, 2571.27)
2023	Male	23659829.02 (22535245.9, 24784412.13)	2537.99 (2473.44, 2602.54)
2024	Male	24046306.17 (22751062.93, 25341549.41)	2548.51 (2457.26, 2639.75)
2025	Male	24437237.19 (22906720.59, 25967753.8)	2558.94 (2436.35, 2681.53)
2026	Male	24827352.72 (23000417.76, 26654287.67)	2569.26 (2411.39, 2727.13)

2027	Male	25212837.2 (23033529.73, 27392144.68)	2579.29 (2382.62, 2775.96)
2028	Male	25587529.27 (23005921.15, 28169137.39)	2589.1 (2350.45, 2827.74)
2029	Male	25965931.17 (22934593.13, 28997269.2)	2598.71 (2315.14, 2882.27)
2030	Male	26342813.71 (22817097.38, 29868530.05)	2608.16 (2276.87, 2939.44)
2031	Male	26712400.97 (22649754.83, 30775047.12)	2617.5 (2235.77, 2999.22)
2032	Male	27070301.08 (22429972.32, 31710629.84)	2626.54 (2191.76, 3061.33)
2033	Male	27412844.36 (22156559.15, 32669129.58)	2635.36 (2145.01, 3125.7)
2034	Male	27754444.61 (21842316.42, 33666572.8)	2643.92 (2095.63, 3192.22)
2035	Male	28091943.65 (21484828.67, 34699058.62)	2652.29 (2043.71, 3260.87)
2036	Male	28421085.93 (21080669.75, 35761502.12)	2660.54 (1989.36, 3331.71)
2037	Male	28737687.55 (20626730.62, 36848644.49)	2668.5 (1932.48, 3404.53)
2038	Male	29043059.22 (20124780.96, 37961337.47)	2676.24 (1873.2, 3479.28)
2039	Male	29349185.32 (19583313.91, 39115056.73)	2683.73 (1811.58, 3555.88)
2040	Male	29653074.9 (18999606.53, 40306543.28)	2691 (1747.69, 3634.31)
2041	Male	29950983.72 (18370395.21, 41531572.24)	2698.18 (1681.62, 3714.73)
2042	Male	30238473.13 (17692282.31, 42784663.95)	2705.1 (1613.3, 3796.9)
2043	Male	30518879.73 (16967343.79, 44070415.67)	2711.82 (1542.82, 3880.81)
2044	Male	30802231.48 (16200551.53, 45403911.43)	2718.27 (1470.21, 3966.33)
2045	Male	31083163.86 (15387679.09, 46778648.63)	2724.51 (1395.54, 4053.49)

Notes: ASR: age-standardized rates; YLDs: years lived with disability.

Table S88. Prevalence and YLDs of musculoskeletal disorders in China of future forecasts using bayesian age-period-cohort model.

Year	Sex	Number	ASR
**Prevalence**			
2022	Both	280129339.05 (266014955.88, 294243722.21)	13500.53 (12819.59, 14181.48)
2023	Both	284936745.69 (269198455.21, 300675036.17)	13502.67 (12756, 14249.33)
2024	Both	289743239.57 (271438176.16, 308048302.97)	13504.42 (12650.28, 14358.57)
2025	Both	294508176.06 (272682891.87, 316333460.25)	13506.41 (12504.39, 14508.42)
2026	Both	299148236.97 (272919114.15, 325377359.79)	13508.34 (12322.73, 14693.95)
2027	Both	303605645.27 (272178146.14, 335033144.4)	13510.38 (12110.52, 14910.25)
2028	Both	307886927.44 (270550935.66, 345222919.21)	13512.92 (11872.76, 15153.09)
2029	Both	312116956.45 (268208138.41, 356025774.48)	13516.54 (11613.31, 15419.76)
2030	Both	316260696.3 (265156727.33, 367364665.27)	13521.81 (11334.9, 15708.72)
2031	Both	320246994.7 (261361879.58, 379132109.83)	13529.04 (11039.15, 16018.92)
2032	Both	324022005.58 (256801702.22, 391242308.95)	13538.19 (10727, 16349.38)
2033	Both	327587941.12 (251505659.01, 403670223.24)	13549.45 (10399.57, 16699.34)
2034	Both	331079424.04 (245595733.67, 416563114.4)	13563.07 (10057.64, 17068.51)
2035	Both	334470836.24 (239058581.9, 429883090.59)	13579.54 (9701.77, 17457.31)
2036	Both	337703292.19 (231854862.47, 443551721.91)	13599.64 (9332.37, 17866.9)
2037	Both	340710325.66 (223945162.8, 457475488.52)	13623.26 (8949.05, 18297.48)
2038	Both	343511902.54 (215357558.95, 471666246.13)	13650.43 (8551.67, 18749.2)
2039	Both	346231556.52 (206177478.71, 486285634.33)	13681.05 (8139.84, 19222.26)
2040	Both	348851753.35 (196390837.98, 501312668.72)	13715.48 (7713.21, 19717.75)
2041	Both	351333527.38 (185969808.21, 516697246.56)	13754.89 (7271.57, 20238.21)
2042	Both	353613444.32 (174878254.55, 532348634.09)	13799.22 (6813.81, 20784.63)
2043	Both	355678225.38 (163116691.91, 548239758.85)	13848.46 (6339.01, 21357.9)

2044	Both	357628586.26 (150734191.5, 564522981.03)	13902.17 (5845.91, 21958.43)
2045	Both	359475000.56 (137726122.51, 581223878.6)	13960.66 (5333.35, 22587.97)
2022	Female	171687445.66 (163261471.66, 180113419.66)	16211.77 (15866.69, 16556.84)
2023	Female	174808422.38 (165361567.81, 184255276.94)	16196.76 (15695.23, 16698.29)
2024	Female	177926974.29 (166862661.06, 188991287.52)	16180.81 (15475.48, 16886.14)
2025	Female	181020693.8 (167738666.19, 194302721.42)	16164.29 (15220.14, 17108.43)
2026	Female	184033699.37 (167980295.27, 200087103.48)	16146.06 (14934.61, 17357.52)
2027	Female	186928978.81 (167607059.07, 206250898.55)	16125.61 (14622.18, 17629.05)
2028	Female	189717846.77 (166681473.27, 212754220.26)	16103.5 (14286.44, 17920.56)
2029	Female	192478000.63 (165308595.55, 219647405.71)	16080.62 (13930.31, 18230.93)
2030	Female	195190352.04 (163493770.59, 226886933.49)	16057.46 (13555.69, 18559.24)
2031	Female	197807438.32 (161211251.52, 234403625.13)	16033.95 (13163.53, 18904.37)
2032	Female	200292655.6 (158443736.8, 242141574.39)	16009.38 (12754.1, 19264.65)
2033	Female	202657677.35 (155216641.08, 250098713.61)	15983.83 (12328.64, 19639.01)
2034	Female	204987061.06 (151604705.69, 258369416.44)	15957.64 (11888.36, 20026.93)
2035	Female	207256117.27 (147591738.93, 266920495.61)	15931.3 (11434.3, 20428.3)
2036	Female	209416070.69 (143144012.15, 275688129.23)	15905.57 (10967.48, 20843.67)
2037	Female	211412038.69 (138228245.07, 284595832.32)	15879.66 (10487.67, 21271.65)
2038	Female	213267481.73 (132870334.97, 293664628.5)	15853.25 (9995.38, 21711.12)
2039	Female	215068785.15 (127129401.14, 303008169.16)	15826.14 (9491.1, 22161.18)
2040	Female	216795576.89 (120991042.13, 312600111.65)	15798.7 (8975.53, 22621.88)
2041	Female	218410800.21 (114431772.34, 322389828.08)	15772.24 (8449.65, 23094.82)
2042	Female	219857325.03 (107422426.59, 332292223.47)	15745.99 (7913.22, 23578.75)
2043	Female	221142084.8 (99974591.11, 342309578.5)	15719.44 (7366.47, 24072.41)
2044	Female	222335229.5 (92124293.85, 352546165.14)	15691.98 (6809.57, 24574.38)
2045	Female	223442677.46 (83870294.12, 363015060.81)	15663.81 (6243, 25084.63)

2022	Male	108441893.38 (102753484.22, 114130302.55)	10700.59 (10465.34, 10935.85)
2023	Male	110128323.31 (103836887.4, 116419759.23)	10712.81 (10376.54, 11049.07)
2024	Male	111816265.28 (104575515.11, 119057015.46)	10724.2 (10255.85, 11192.56)
2025	Male	113487482.26 (104944225.68, 122030738.83)	10735.21 (10111.43, 11358.99)
2026	Male	115114537.6 (104938818.88, 125290256.32)	10745.87 (9947.47, 11544.27)
2027	Male	116676666.46 (104571087.07, 128782245.85)	10756.07 (9766.27, 11745.87)
2028	Male	118169080.67 (103869462.39, 132468698.95)	10765.41 (9569.36, 11961.47)
2029	Male	119638955.82 (102899542.86, 136378368.77)	10774.12 (9358.23, 12190.01)
2030	Male	121070344.26 (101662956.73, 140477731.78)	10782.64 (9134.14, 12431.14)
2031	Male	122439556.38 (100150628.06, 144728484.71)	10791.25 (8897.89, 12684.61)
2032	Male	123729349.99 (98357965.42, 149100734.55)	10799.75 (8649.75, 12949.76)
2033	Male	124930263.78 (96289017.92, 153571509.63)	10807.7 (8390.03, 13225.38)
2034	Male	126092362.97 (93991027.98, 158193697.96)	10815.07 (8119.26, 13510.88)
2035	Male	127214718.98 (91466842.98, 162962594.97)	10822.25 (7838.11, 13806.38)
2036	Male	128287221.5 (88710850.32, 167863592.68)	10829.69 (7547.15, 14112.24)
2037	Male	129298286.97 (85716917.73, 172879656.2)	10837.18 (7246.37, 14428)
2038	Male	130244420.81 (82487223.98, 178001617.63)	10844.24 (6935.86, 14752.62)
2039	Male	131162771.37 (79048077.57, 183277465.17)	10850.58 (6615.8, 15085.35)
2040	Male	132056176.46 (75399795.86, 188712557.07)	10856.43 (6286.6, 15426.26)
2041	Male	132922727.18 (71538035.87, 194307418.48)	10862.44 (5948.77, 15776.11)
2042	Male	133756119.29 (67455827.96, 200056410.62)	10868.37 (5602.26, 16134.48)
2043	Male	134536140.58 (63142100.81, 205930180.35)	10873.85 (5247.18, 16500.52)
2044	Male	135293356.76 (58609897.64, 211976815.89)	10878.31 (4883.55, 16873.07)
2045	Male	136032323.1 (53855828.4, 218208817.8)	10881.86 (4511.66, 17252.05)
**YLDs**			
2022	Both	21263628.77 (20238128.76, 22289128.78)	1062.48 (1011.12, 1113.84)

2023	Both	21544147.05 (20391557.81, 22696736.28)	1061.79 (1004.86, 1118.72)
2024	Both	21822527.54 (20467181.35, 23177873.73)	1061.13 (995.1, 1127.16)
2025	Both	22096875.65 (20465020.37, 23728730.94)	1060.54 (982.1, 1138.97)
2026	Both	22363072.35 (20388341.66, 24337803.04)	1060.04 (966.33, 1153.75)
2027	Both	22618229.42 (20242054.24, 24994404.6)	1059.64 (948.22, 1171.05)
2028	Both	22859127.16 (20030292.94, 25687961.38)	1059.36 (928.18, 1190.54)
2029	Both	23094012.81 (19765326.9, 26422698.72)	1059.22 (906.48, 1211.95)
2030	Both	23321555.82 (19449158.8, 27193952.83)	1059.25 (883.32, 1235.18)
2031	Both	23538888.14 (19081500.14, 27996276.14)	1059.51 (858.85, 1260.17)
2032	Both	23743858.54 (18662306.8, 28825410.29)	1060.01 (833.14, 1286.88)
2033	Both	23931711.43 (18189935.45, 29673487.41)	1060.74 (806.25, 1315.23)
2034	Both	24111197.66 (17672778.36, 30549616.96)	1061.7 (778.22, 1345.18)
2035	Both	24282327.3 (17111644.6, 31453010.01)	1062.91 (749.08, 1376.74)
2036	Both	24444008.03 (16506174.2, 32381841.87)	1064.46 (718.87, 1410.04)
2037	Both	24594106.23 (15855328.05, 33332884.41)	1066.35 (687.56, 1445.14)
2038	Both	24729680.35 (15158100.55, 34301260.14)	1068.57 (655.11, 1482.03)
2039	Both	24857472.37 (14419375.62, 35295569.12)	1071.09 (621.48, 1520.71)
2040	Both	24978295.07 (13639641.57, 36316948.58)	1073.93 (586.61, 1561.25)
2041	Both	25092836.24 (12818883.11, 37366789.38)	1077.2 (550.5, 1603.9)
2042	Both	25199678.89 (11955893.96, 38443463.82)	1080.9 (513.05, 1648.75)
2043	Both	25293853.01 (11048242.26, 39539463.75)	1085.03 (474.16, 1695.9)
2044	Both	25380144.04 (10098082.7, 40662205.38)	1089.54 (433.72, 1745.36)
2045	Both	25460977.56 (9105680.6, 41816274.53)	1094.44 (391.62, 1797.27)
2022	Female	12805135.95 (12214047.11, 13396224.79)	1257.82 (1232.06, 1283.58)
2023	Female	12984849.17 (12311901.43, 13657796.92)	1255.45 (1216.39, 1294.52)
2024	Female	13162588.07 (12358450.24, 13966725.91)	1253.06 (1196.89, 1309.24)

2025	Female	13337525.24 (12354948.42, 14320102.05)	1250.68 (1174.61, 1326.76)
2026	Female	13506980.99 (12304077.31, 14709884.67)	1248.28 (1150.02, 1346.54)
2027	Female	13669140.28 (12209197.6, 15129082.95)	1245.81 (1123.37, 1368.25)
2028	Female	13822594.71 (12073434.11, 15571755.3)	1243.29 (1094.9, 1391.68)
2029	Female	13972347.8 (11904225.74, 16040469.86)	1240.74 (1064.82, 1416.67)
2030	Female	14118024.91 (11703095.35, 16532954.47)	1238.21 (1033.26, 1443.15)
2031	Female	14257944.85 (11469831.65, 17046058.04)	1235.73 (1000.36, 1471.09)
2032	Female	14390716.6 (11204234.5, 17577198.69)	1233.24 (966.12, 1500.36)
2033	Female	14514356.7 (10905926.35, 18122787.05)	1230.75 (930.64, 1530.85)
2034	Female	14634211.41 (10579818.22, 18688604.61)	1228.22 (893.97, 1562.46)
2035	Female	14749553.08 (10225705.33, 19273400.83)	1225.68 (856.19, 1595.18)
2036	Female	14858824.01 (9842641.49, 19875006.54)	1223.25 (817.41, 1629.09)
2037	Female	14959485.6 (9429136.36, 20489834.84)	1220.86 (777.62, 1664.11)
2038	Female	15050715.83 (8985274.48, 21116157.18)	1218.48 (736.85, 1700.11)
2039	Female	15137478.89 (8514449.47, 21760508.3)	1216.05 (695.11, 1736.99)
2040	Female	15219375.95 (8016338.19, 22422413.71)	1213.59 (652.45, 1774.72)
2041	Female	15295824.25 (7490357.59, 23101290.92)	1211.24 (608.98, 1813.5)
2042	Female	15364255.01 (6935003.33, 23793506.69)	1208.94 (564.67, 1853.22)
2043	Female	15422988.77 (6349728.81, 24496248.73)	1206.65 (519.53, 1893.77)
2044	Female	15475448.68 (5736104.62, 25214792.73)	1204.29 (473.58, 1935.01)
2045	Female	15523234.29 (5094352.54, 25952116.04)	1201.87 (426.84, 1976.91)
2022	Male	8458492.82 (8024081.65, 8892903.99)	863.3 (844.86, 881.74)
2023	Male	8559297.87 (8079656.38, 9038939.36)	863.87 (837.32, 890.42)
2024	Male	8659939.47 (8108731.12, 9211147.82)	864.41 (827.31, 901.52)
2025	Male	8759350.41 (8110071.94, 9408628.88)	864.96 (815.46, 914.47)
2026	Male	8856091.36 (8084264.35, 9627918.37)	865.55 (802.13, 928.98)

2027	Male	8949089.14 (8032856.63, 9865321.65)	866.17 (787.5, 944.85)
2028	Male	9036532.45 (7956858.83, 10116206.07)	866.78 (771.68, 961.88)
2029	Male	9121665.01 (7861101.16, 10382228.85)	867.36 (754.76, 979.96)
2030	Male	9203530.91 (7746063.45, 10660998.36)	867.94 (736.84, 999.05)
2031	Male	9280943.29 (7611668.49, 10950218.1)	868.57 (717.98, 1019.15)
2032	Male	9353141.95 (7458072.29, 11248211.6)	869.23 (698.22, 1040.23)
2033	Male	9417354.73 (7284009.11, 11550700.36)	869.88 (677.57, 1062.18)
2034	Male	9476986.25 (7092960.15, 11861012.35)	870.48 (656.04, 1084.92)
2035	Male	9532774.22 (6885939.26, 12179609.18)	871.06 (633.68, 1108.44)
2036	Male	9585184.02 (6663532.72, 12506835.33)	871.66 (610.53, 1132.78)
2037	Male	9634620.63 (6426191.69, 12843049.57)	872.26 (586.61, 1157.91)
2038	Male	9678964.52 (6172826.07, 13185102.97)	872.85 (561.93, 1183.77)
2039	Male	9719993.48 (5904926.15, 13535060.82)	873.36 (536.47, 1210.24)
2040	Male	9758919.12 (5623303.37, 13894534.87)	873.79 (510.26, 1237.31)
2041	Male	9797011.99 (5328525.52, 14265498.46)	874.2 (483.35, 1265.05)
2042	Male	9835423.88 (5020890.63, 14649957.12)	874.58 (455.74, 1293.42)
2043	Male	9870864.24 (4698513.46, 15043215.02)	874.92 (427.45, 1322.4)
2044	Male	9904695.36 (4361978.08, 15447412.64)	875.16 (398.47, 1351.84)
2045	Male	9937743.27 (4011328.06, 15864158.49)	875.28 (368.83, 1381.73)

Notes: ASR: age-standardized rates; YLDs: years lived with disability.

Table S89. Prevalence and YLDs of neurological disorders in China of future forecasts using bayesian age-period-cohort model.

Year	Sex	Number	ASR
**Prevalence**			
2022	Both	545535891.19 (506100698.74, 584971083.63)	33298.68 (30888.22, 35709.13)
2023	Both	553196439.61 (510826052.3, 595566826.93)	33567.35 (30992.48, 36142.21)
2024	Both	560669635.68 (513814185.65, 607525085.7)	33824.42 (30993.3, 36655.54)
2025	Both	568058324.36 (515023243.43, 621093405.3)	34069.99 (30884.16, 37255.82)
2026	Both	575459724.63 (514542716.99, 636376732.28)	34302.73 (30665.79, 37939.67)
2027	Both	583119238.78 (512670404.2, 653568073.37)	34524.69 (30346.95, 38702.43)
2028	Both	590261654.34 (508848283.79, 671675024.89)	34737.66 (29938.5, 39536.82)
2029	Both	597195516.86 (503467597.32, 690923436.39)	34942.07 (29448.63, 40435.51)
2030	Both	603995715.28 (496676772.02, 711314658.54)	35137.65 (28883, 41392.3)
2031	Both	610714666.61 (488575110.46, 732854222.76)	35322.58 (28244.46, 42400.71)
2032	Both	617518535.65 (479332133.87, 755704937.43)	35498.76 (27537.99, 43459.53)
2033	Both	624063725.2 (468719123.39, 779408327.02)	35668.71 (26769.16, 44568.27)
2034	Both	630532543.66 (456906086.32, 804159001.01)	35832.97 (25940.6, 45725.34)
2035	Both	636929053.46 (443901702.82, 829956404.11)	35991.07 (25053.15, 46928.99)
2036	Both	643231274.77 (429683328.32, 856779221.22)	36141.08 (24105.93, 48176.22)
2037	Both	649516473.83 (414290382.32, 884742565.34)	36284.87 (23100.49, 49469.26)
2038	Both	655513636.84 (397550791.56, 913476482.12)	36425.59 (22039.54, 50811.63)
2039	Both	661400217.63 (379574083.1, 943226352.16)	36563.93 (20923.33, 52204.53)
2040	Both	667229588.23 (360367493.98, 974091682.49)	36699.38 (19750.62, 53648.14)
2041	Both	673022206.62 (339903564.93, 1006140848.31)	36830 (18518.97, 55141.02)
2042	Both	678908825.11 (318199860.48, 1039617789.74)	36957.76 (17227.86, 56687.66)
2043	Both	684485965.63 (295033228.44, 1073938702.81)	37086.72 (15877.94, 58295.51)

2044	Both	689946439.45 (270465488.56, 1109427390.34)	37218.06 (14467.47, 59968.66)
2045	Both	695402427.32 (244488324.7, 1146316529.94)	37351.23 (12993.23, 61709.24)
2022	Female	312511635.24 (290744952.19, 334278318.29)	38408.72 (37376.04, 39441.4)
2023	Female	317021641.88 (293591929.13, 340451354.64)	38731.17 (37327.15, 40135.2)
2024	Female	321429007.37 (295455374.37, 347402640.37)	39040.14 (37143.34, 40936.95)
2025	Female	325793548.27 (296315444.03, 355271652.51)	39335.37 (36849.87, 41820.88)
2026	Female	330175260.85 (296229922.44, 364120599.25)	39615.06 (36460.39, 42769.74)
2027	Female	334712504.4 (295368445.75, 374056563.04)	39879.94 (35985.05, 43774.83)
2028	Female	338980279.25 (293424984.23, 384535574.26)	40130.51 (35431.86, 44829.16)
2029	Female	343136413.31 (290605158.21, 395667668.42)	40366.82 (34805.86, 45927.78)
2030	Female	347225856.72 (286993774.56, 407457938.88)	40588.15 (34109.9, 47066.4)
2031	Female	351283426.9 (282649357.94, 419917495.87)	40792.05 (33344.52, 48239.58)
2032	Female	355401310 (277665223.84, 433137396.15)	40979.03 (32512.58, 49445.49)
2033	Female	359378813.87 (271904022.3, 446853605.45)	41150.37 (31618.36, 50682.38)
2034	Female	363307463.03 (265452467.4, 461162458.67)	41306.22 (30664.62, 51947.82)
2035	Female	367193509.64 (258318038.56, 476068980.73)	41445.61 (29652.79, 53238.44)
2036	Female	371031936.59 (250492490.13, 491571383.04)	41565.96 (28582.92, 54548.99)
2037	Female	374867110.9 (241997888.32, 507736333.49)	41667.79 (27457.45, 55878.13)
2038	Female	378533723.33 (232727239.88, 524340206.78)	41753.11 (26280.58, 57225.63)
2039	Female	382118180.35 (222735369.87, 541500990.83)	41822.29 (25055.02, 58589.57)
2040	Female	385658190.98 (212032597.41, 559283784.55)	41874.32 (23782.27, 59966.37)
2041	Female	389175803.44 (200610239.64, 577741367.25)	41906.66 (22462.92, 61350.39)
2042	Female	392750585.97 (188482110.85, 597019061.08)	41920.08 (21099.6, 62740.56)
2043	Female	396146760.64 (175515850.32, 616777670.95)	41917.63 (19696.72, 64138.54)
2044	Female	399457973.63 (161742286.74, 637173660.52)	41900.06 (18257.04, 65543.09)
2045	Female	402759005.9 (147164452.83, 658353558.97)	41866.35 (16782.28, 66950.43)

2022	Male	233024255.94 (215355746.54, 250692765.34)	28347.33 (27529.09, 29165.58)
2023	Male	236174797.73 (217234123.17, 255115472.29)	28565.9 (27460.95, 29670.85)
2024	Male	239240628.31 (218358811.28, 260122445.34)	28771.51 (27286.97, 30256.06)
2025	Male	242264776.09 (218707799.4, 265821752.79)	28963.55 (27026.24, 30900.85)
2026	Male	245284463.78 (218312794.54, 272256133.03)	29139.63 (26688.53, 31590.74)
2027	Male	248406734.39 (217301958.44, 279511510.33)	29300.68 (26282.29, 32319.06)
2028	Male	251281375.09 (215423299.56, 287139450.62)	29448.37 (25815.26, 33081.47)
2029	Male	254059103.54 (212862439.11, 295255767.97)	29583.06 (25291.97, 33874.15)
2030	Male	256769858.56 (209682997.46, 303856719.66)	29703.85 (24714.63, 34693.06)
2031	Male	259431239.71 (205925752.52, 312936726.89)	29807.88 (24083.25, 35532.51)
2032	Male	262117225.65 (201666910.03, 322567541.28)	29895.89 (23400.72, 36391.06)
2033	Male	264684911.33 (196815101.09, 332554721.57)	29970.12 (22671.76, 37268.48)
2034	Male	267225080.63 (191453618.92, 342996542.34)	30031.01 (21899.1, 38162.93)
2035	Male	269735543.82 (185583664.26, 353887423.38)	30077.43 (21083.87, 39070.98)
2036	Male	272199338.18 (179190838.18, 365207838.18)	30106.39 (20225.86, 39986.93)
2037	Male	274649362.93 (172292494, 377006231.85)	30118.57 (19327.54, 40909.61)
2038	Male	276979913.51 (164823551.68, 389136275.34)	30116.64 (18393.29, 41839.98)
2039	Male	279282037.28 (156838713.23, 401725361.34)	30101.12 (17425.7, 42776.54)
2040	Male	281571397.25 (148334896.57, 414807897.94)	30070.84 (16426.05, 43715.63)
2041	Male	283846403.18 (139293325.29, 428399481.06)	30022.95 (15394.76, 44651.14)
2042	Male	286158239.14 (129717749.62, 442598728.66)	29958.27 (14334.42, 45582.13)
2043	Male	288339204.99 (119517378.11, 457161031.87)	29880.23 (13249.32, 46511.13)
2044	Male	290488465.82 (108723201.83, 472253729.82)	29789.66 (12142.07, 47437.24)
2045	Male	292643421.42 (97323871.86, 487962970.98)	29685.46 (11014.21, 48356.71)
**YLDs**			
2022	Both	17900432.52 (16876091.21, 18924773.83)	974.96 (919.14, 1030.78)

2023	Both	18424468.89 (17288130.99, 19560806.79)	985.96 (925.11, 1046.82)
2024	Both	18975786.42 (17668155.33, 20283417.52)	996.77 (928.03, 1065.52)
2025	Both	19547813.47 (18004646.08, 21090980.86)	1007.46 (927.85, 1087.07)
2026	Both	20134436.07 (18291070.6, 21977801.53)	1018.03 (924.73, 1111.34)
2027	Both	20731183.39 (18524628.1, 22937738.68)	1028.51 (918.91, 1138.1)
2028	Both	21341698.36 (18710871.63, 23972525.09)	1038.86 (910.65, 1167.07)
2029	Both	21982309.98 (18864925.02, 25099694.94)	1049.1 (900.14, 1198.05)
2030	Both	22644930.15 (18978915.57, 26310944.72)	1059.25 (887.55, 1230.95)
2031	Both	23321283.4 (19044333.83, 27598232.97)	1069.33 (872.97, 1265.7)
2032	Both	24004606.32 (19054052.8, 28955159.85)	1079.37 (856.46, 1302.28)
2033	Both	24700551.45 (19011537.98, 30389564.91)	1089.39 (838.11, 1340.66)
2034	Both	25427817.03 (18928782.66, 31926851.39)	1099.38 (817.97, 1380.8)
2035	Both	26176777.41 (18794766.13, 33558788.7)	1109.39 (796.04, 1422.74)
2036	Both	26936675.03 (18597989.08, 35275360.97)	1119.41 (772.3, 1466.52)
2037	Both	27697668.97 (18328452.45, 37066885.49)	1129.48 (746.75, 1512.22)
2038	Both	28467622.16 (17989123.95, 38946120.36)	1139.65 (719.39, 1559.91)
2039	Both	29267476.27 (17589398.55, 40945554)	1149.92 (690.2, 1609.63)
2040	Both	30089353.58 (17118970.14, 43059737.02)	1160.31 (659.13, 1661.49)
2041	Both	30923851.51 (16566978.59, 45280724.43)	1170.84 (626.11, 1715.57)
2042	Both	31762407.8 (15923808.94, 47601006.65)	1181.57 (591.06, 1772.08)
2043	Both	32617794.57 (15191577.66, 50044011.48)	1192.55 (553.94, 1831.16)
2044	Both	33511193.68 (14373552.69, 52648834.67)	1203.8 (514.65, 1892.94)
2045	Both	34428684.42 (13455169.08, 55402199.76)	1215.33 (473.08, 1957.59)
2022	Female	10232914.03 (9672275.71, 10793552.35)	1107 (1083.31, 1130.69)
2023	Female	10555187.41 (9926123.2, 11184251.63)	1121.62 (1086.47, 1156.78)
2024	Female	10894612.13 (10160237.23, 11628987.03)	1136.06 (1085.92, 1186.2)

2025	Female	11248044.06 (10368791.15, 12127296.98)	1150.32 (1082.39, 1218.25)
2026	Female	11612486.16 (10548821.48, 12676150.84)	1164.38 (1076.24, 1252.53)
2027	Female	11985769.91 (10699177.05, 13272362.77)	1178.21 (1067.62, 1288.8)
2028	Female	12369164.33 (10822164.17, 13916164.48)	1191.79 (1056.69, 1326.89)
2029	Female	12772114.86 (10926246.31, 14617983.42)	1205.14 (1043.56, 1366.72)
2030	Female	13190973.26 (11007409.44, 15374537.08)	1218.26 (1028.3, 1408.23)
2031	Female	13621773.64 (11060993, 16182554.28)	1231.14 (1010.91, 1451.37)
2032	Female	14061036.11 (11082704.91, 17039367.31)	1243.75 (991.39, 1496.1)
2033	Female	14512127.75 (11074021.77, 17950233.72)	1256.09 (969.82, 1542.36)
2034	Female	14985612.41 (11040992.79, 18930232.02)	1268.18 (946.24, 1590.12)
2035	Female	15475676.09 (10976465.24, 19974886.95)	1280.01 (920.68, 1639.35)
2036	Female	15975747.84 (10872957.54, 21078538.15)	1291.57 (893.13, 1690.01)
2037	Female	16479457.2 (10723635.96, 22235278.45)	1302.85 (863.62, 1742.09)
2038	Female	16991328.33 (10529531.88, 23453124.77)	1313.86 (832.18, 1795.54)
2039	Female	17523807.48 (10295456.73, 24752158.22)	1324.6 (798.87, 1850.33)
2040	Female	18072456.34 (10014826.5, 26130086.18)	1335.06 (763.69, 1906.43)
2041	Female	18631806.69 (9680621.7, 27582991.68)	1345.22 (726.66, 1963.79)
2042	Female	19196748.14 (9286419.33, 29107076.95)	1355.1 (687.79, 2022.41)
2043	Female	19774709.27 (8832438.65, 30716979.9)	1364.71 (647.14, 2082.28)
2044	Female	20378871.8 (8319741.09, 32438002.51)	1374.04 (604.74, 2143.33)
2045	Female	21002280.86 (7739332.59, 34265229.13)	1383.09 (560.63, 2205.55)
2022	Male	7667518.49 (7203815.5, 8131221.48)	843.12 (823.95, 862.29)
2023	Male	7869281.48 (7362007.79, 8376555.16)	850.51 (823.58, 877.45)
2024	Male	8081174.3 (7507918.11, 8654430.49)	857.66 (820.6, 894.73)
2025	Male	8299769.41 (7635854.93, 8963683.89)	864.59 (815.51, 913.66)
2026	Male	8521949.91 (7742249.12, 9301650.69)	871.27 (808.59, 933.95)

2027	Male	8745413.48 (7825451.05, 9665375.91)	877.71 (799.98, 955.43)
2028	Male	8972534.04 (7888707.46, 10056360.61)	883.88 (789.81, 977.94)
2029	Male	9210195.12 (7938678.71, 10481711.53)	889.77 (778.16, 1001.39)
2030	Male	9453956.89 (7971506.13, 10936407.65)	895.4 (765.11, 1025.7)
2031	Male	9699509.76 (7983340.83, 11415678.69)	900.75 (750.67, 1050.83)
2032	Male	9943570.21 (7971347.89, 11915792.54)	905.81 (734.89, 1076.73)
2033	Male	10188423.7 (7937516.21, 12439331.19)	910.59 (717.82, 1103.36)
2034	Male	10442204.62 (7887789.87, 12996619.37)	915.09 (699.52, 1130.65)
2035	Male	10701101.32 (7818300.88, 13583901.75)	919.3 (680.02, 1158.58)
2036	Male	10960927.18 (7725031.54, 14196822.82)	923.21 (659.34, 1187.09)
2037	Male	11218211.77 (7604816.5, 14831607.05)	926.82 (637.5, 1216.15)
2038	Male	11476293.83 (7459592.07, 15492995.59)	930.16 (614.57, 1245.75)
2039	Male	11743668.8 (7293941.82, 16193395.78)	933.22 (590.6, 1275.85)
2040	Male	12016897.24 (7104143.65, 16929650.84)	936 (565.61, 1306.4)
2041	Male	12292044.82 (6886356.88, 17697732.76)	938.47 (539.61, 1337.34)
2042	Male	12565659.66 (6637389.62, 18493929.7)	940.65 (512.64, 1368.66)
2043	Male	12843085.3 (6359139.01, 19327031.58)	942.58 (484.78, 1400.38)
2044	Male	13132321.88 (6053811.59, 20210832.16)	944.25 (456.05, 1432.45)
2045	Male	13426403.56 (5715836.49, 21136970.64)	945.67 (426.5, 1464.84)

Notes: ASR: age-standardized rates; YLDs: years lived with disability.

Table S90. Prevalence and YLDs of digestive disorders in China of future forecasts using bayesian age-period-cohort model.

Year	Sex	Number	ASR
**Prevalence**			
2022	Both	686320.05 (616425.46, 756214.64)	33.83 (30.36, 37.3)
2023	Both	701491.85 (601911.92, 801071.78)	34.04 (29.19, 38.89)
2024	Both	717719.3 (572856.91, 862581.7)	34.29 (27.36, 41.22)
2025	Both	735321.34 (532118.13, 938524.54)	34.61 (25.04, 44.19)
2026	Both	754587.57 (480772.62, 1028402.53)	35.02 (22.3, 47.73)
2027	Both	775882.84 (418677.77, 1133087.9)	35.53 (19.16, 51.89)
2028	Both	799800.17 (344887.53, 1254712.81)	36.16 (15.59, 56.74)
2029	Both	827235.21 (257600.87, 1396869.56)	36.95 (11.5, 62.41)
2030	Both	858894.28 (153770.78, 1564017.78)	37.93 (6.78, 69.08)
2031	Both	895523.2 (28987.98, 1762067.08)	39.13 (1.26, 77.01)
2032	Both	938076.29 (0, 1999146.47)	40.59 (0, 86.51)
2033	Both	987802.86 (0, 2286515.87)	42.36 (0, 98.06)
2034	Both	1046559.91 (0, 2640530.46)	44.5 (0, 112.29)
2035	Both	1116301.18 (0, 3083280.08)	47.1 (0, 130.1)
2036	Both	1199323.53 (0, 3645633.22)	50.25 (0, 152.74)
2037	Both	1298601.05 (0, 4372093.78)	54.07 (0, 182.02)
2038	Both	1418021.97 (0, 5327731.88)	58.71 (0, 220.57)
2039	Both	1563210.69 (0, 6611259.46)	64.4 (0, 272.33)
2040	Both	1740992.7 (0, 8369080.32)	71.41 (0, 343.22)
2041	Both	1960168.75 (0, 10822752.01)	80.12 (0, 442.22)
2042	Both	2232629.28 (0, 14313657.13)	91.01 (0, 583.22)
2043	Both	2574517.19 (0, 19372963.03)	104.76 (0, 787.89)

2044	Both	3009454.56 (0, 26848977.95)	122.33 (0, 1090.68)
2045	Both	3569585.91 (0, 38090828.67)	145.06 (0, 1546.77)
2022	Female	295699.38 (262588.97, 328809.8)	29.1 (27.22, 30.98)
2023	Female	302721.13 (257062.43, 348379.83)	29.24 (25.74, 32.74)
2024	Female	310252 (245099.67, 375404.32)	29.38 (23.85, 34.91)
2025	Female	318431.74 (227851.52, 409011.95)	29.52 (21.63, 37.41)
2026	Female	327395.2 (205794.97, 448995.44)	29.66 (19.12, 40.2)
2027	Female	337312.94 (178860.11, 495765.78)	29.8 (16.33, 43.26)
2028	Female	348455.32 (146595.4, 550315.23)	29.93 (13.3, 46.56)
2029	Female	361228 (108144.43, 614311.58)	30.06 (10.03, 50.1)
2030	Female	375955.38 (62069.84, 689840.93)	30.2 (6.53, 53.87)
2031	Female	392991.87 (6295.55, 779696.84)	30.34 (2.81, 57.86)
2032	Female	412788.88 (0, 887730.64)	30.47 (-1.12, 62.06)
2033	Female	435933.44 (0, 1019288.78)	30.6 (-5.26, 66.47)
2034	Female	463286.99 (0, 1182137.01)	30.73 (-9.6, 71.07)
2035	Female	495760.47 (0, 1386819.56)	30.87 (-14.14, 75.87)
2036	Female	534449.4 (0, 1648206.6)	31 (-18.87, 80.87)
2037	Female	580779.1 (0, 1987865.14)	31.13 (-23.8, 86.06)
2038	Female	636622.24 (0, 2437526.66)	31.26 (-28.91, 91.42)
2039	Female	704672.32 (0, 3045514.74)	31.39 (-34.2, 96.98)
2040	Female	788207.02 (0, 3883876.8)	31.52 (-39.68, 102.72)
2041	Female	891497.74 (0, 5062357.85)	31.65 (-45.34, 108.64)
2042	Female	1020345.28 (0, 6750990.73)	31.78 (-51.18, 114.74)
2043	Female	1182711.08 (0, 9216116.98)	31.9 (-57.2, 121)
2044	Female	1390220.55 (0, 12884933.58)	32.03 (-63.39, 127.45)
2045	Female	1658760.46 (0, 18440150.18)	32.16 (-69.76, 134.08)

2022	Male	390620.66 (353836.49, 427404.83)	38.56 (36.28, 40.84)
2023	Male	398770.72 (344849.49, 452691.95)	38.76 (34.36, 43.15)
2024	Male	407467.31 (327757.24, 487177.37)	38.95 (31.91, 45.99)
2025	Male	416889.6 (304266.61, 529512.59)	39.14 (29.03, 49.25)
2026	Male	427192.37 (274977.65, 579407.09)	39.33 (25.77, 52.9)
2027	Male	438569.89 (239817.67, 637322.11)	39.52 (22.15, 56.88)
2028	Male	451344.85 (198292.13, 704397.58)	39.7 (18.21, 61.19)
2029	Male	466007.21 (149456.44, 782557.98)	39.87 (13.95, 65.79)
2030	Male	482938.89 (91700.93, 874176.85)	40.04 (9.39, 70.7)
2031	Male	502531.33 (22692.42, 982370.23)	40.21 (4.55, 75.88)
2032	Male	525287.4 (0, 1111415.83)	40.38 (-0.57, 81.32)
2033	Male	551869.42 (0, 1267227.09)	40.53 (-5.96, 87.02)
2034	Male	583272.92 (0, 1458393.45)	40.68 (-11.6, 92.97)
2035	Male	620540.71 (0, 1696460.51)	40.83 (-17.49, 99.16)
2036	Male	664874.13 (0, 1997426.62)	40.98 (-23.63, 105.6)
2037	Male	717821.95 (0, 2384228.64)	41.12 (-30.01, 112.26)
2038	Male	781399.73 (0, 2890205.22)	41.26 (-36.61, 119.13)
2039	Male	858538.36 (0, 3565744.71)	41.39 (-43.44, 126.23)
2040	Male	952785.67 (0, 4485203.52)	41.52 (-50.5, 133.55)
2041	Male	1068671.01 (0, 5760394.16)	41.65 (-57.78, 141.09)
2042	Male	1212284 (0, 7562666.4)	41.78 (-65.27, 148.83)
2043	Male	1391806.12 (0, 10156846.05)	41.9 (-72.96, 156.76)
2044	Male	1619234.01 (0, 13964044.37)	42.02 (-80.87, 164.91)
2045	Male	1910825.45 (0, 19650678.49)	42.14 (-88.99, 173.27)
**YLDs**			
2022	Both	66002.06 (59578.8, 72425.32)	3.36 (3.02, 3.7)

2023	Both	67031.03 (58424.1, 75637.97)	3.37 (2.93, 3.81)
2024	Both	68105.7 (56162.5, 80048.89)	3.38 (2.78, 3.98)
2025	Both	69244.49 (53011.57, 85477.42)	3.4 (2.59, 4.2)
2026	Both	70464.66 (49089.72, 91839.59)	3.42 (2.38, 4.46)
2027	Both	71788.75 (44436.26, 99141.24)	3.45 (2.13, 4.77)
2028	Both	73242.87 (39036.9, 107448.83)	3.48 (1.85, 5.11)
2029	Both	74876.54 (32839.65, 116913.43)	3.53 (1.54, 5.51)
2030	Both	76724.04 (25730.73, 127717.35)	3.58 (1.2, 5.96)
2031	Both	78820.42 (17539.7, 140101.13)	3.65 (0.81, 6.49)
2032	Both	81211.28 (8032.93, 154392.45)	3.73 (0.37, 7.09)
2033	Both	83938.67 (456.12, 170996.49)	3.83 (0.02, 7.8)
2034	Both	87086.9 (0, 190518.66)	3.94 (0, 8.63)
2035	Both	90732.63 (0, 213714.84)	4.08 (0, 9.62)
2036	Both	94960.47 (0, 241577.39)	4.25 (0, 10.81)
2037	Both	99878.32 (0, 275456.39)	4.45 (0, 12.25)
2038	Both	105590.55 (0, 317102.89)	4.68 (0, 14.04)
2039	Both	112289.53 (0, 369112.47)	4.95 (0, 16.27)
2040	Both	120180.32 (0, 435000.07)	5.28 (0, 19.1)
2041	Both	129509.36 (0, 519667.57)	5.67 (0, 22.73)
2042	Both	140595.99 (0, 630098.07)	6.14 (0, 27.47)
2043	Both	153789.59 (0, 775995.21)	6.71 (0, 33.75)
2044	Both	169654.37 (0, 971905.27)	7.39 (0, 42.19)
2045	Both	188881.65 (0, 1238859.84)	8.22 (0, 53.7)
2022	Female	30587.8 (27146.7, 34028.9)	3.11 (2.92, 3.29)
2023	Female	31099.23 (26654.38, 35544.09)	3.11 (2.78, 3.44)
2024	Female	31637.36 (25625.63, 37649.09)	3.12 (2.61, 3.62)

2025	Female	32210.93 (24151.71, 40270.16)	3.12 (2.4, 3.83)
2026	Female	32828.68 (22289.29, 43368.07)	3.12 (2.18, 4.07)
2027	Female	33502.43 (20057.22, 46947.64)	3.13 (1.93, 4.32)
2028	Female	34246.36 (17446.05, 51046.67)	3.13 (1.66, 4.59)
2029	Female	35085.37 (14424.4, 55746.33)	3.13 (1.38, 4.88)
2030	Female	36036.58 (10927.77, 61145.4)	3.13 (1.07, 5.19)
2031	Female	37118.21 (6860.67, 67375.74)	3.13 (0.75, 5.51)
2032	Female	38354.23 (2093.37, 74617.92)	3.13 (0.42, 5.84)
2033	Female	39768.09 (0, 83099.74)	3.13 (0.07, 6.19)
2034	Female	41403.7 (0, 93157.93)	3.13 (-0.3, 6.55)
2035	Female	43301.7 (0, 105218.48)	3.13 (-0.67, 6.93)
2036	Female	45508.49 (0, 119849.05)	3.13 (-1.06, 7.31)
2037	Female	48084.03 (0, 137830.79)	3.12 (-1.46, 7.71)
2038	Female	51089.77 (0, 160197.89)	3.12 (-1.87, 8.11)
2039	Female	54631.39 (0, 188478.98)	3.12 (-2.29, 8.52)
2040	Female	58823.57 (0, 224771.26)	3.11 (-2.72, 8.95)
2041	Female	63806.69 (0, 272035.42)	3.11 (-3.16, 9.38)
2042	Female	69764.56 (0, 334533.85)	3.1 (-3.61, 9.81)
2043	Female	76911.54 (0, 418307.42)	3.09 (-4.07, 10.26)
2044	Female	85576.86 (0, 532428.21)	3.09 (-4.53, 10.71)
2045	Female	96168.01 (0, 690139.35)	3.08 (-5.01, 11.16)
2022	Male	35414.26 (32432.1, 38396.42)	3.61 (3.43, 3.79)
2023	Male	35931.8 (31769.71, 40093.89)	3.62 (3.28, 3.95)
2024	Male	36468.33 (30536.87, 42399.8)	3.63 (3.09, 4.16)
2025	Male	37033.56 (28859.86, 45207.26)	3.63 (2.88, 4.39)
2026	Male	37635.98 (26800.44, 48471.53)	3.64 (2.63, 4.65)

2027	Male	38286.32 (24379.04, 52193.61)	3.65 (2.37, 4.93)
2028	Male	38996.51 (21590.85, 56402.16)	3.66 (2.07, 5.24)
2029	Male	39791.17 (18415.24, 61167.09)	3.66 (1.76, 5.56)
2030	Male	40687.46 (14802.96, 66571.95)	3.67 (1.43, 5.9)
2031	Male	41702.21 (10679.03, 72725.39)	3.67 (1.08, 6.26)
2032	Male	42857.05 (5939.56, 79774.54)	3.67 (0.71, 6.64)
2033	Male	44170.58 (456.12, 87896.75)	3.68 (0.33, 7.03)
2034	Male	45683.2 (0, 97360.73)	3.68 (-0.07, 7.43)
2035	Male	47430.93 (0, 108496.36)	3.68 (-0.49, 7.85)
2036	Male	49451.98 (0, 121728.34)	3.68 (-0.92, 8.28)
2037	Male	51794.28 (0, 137625.6)	3.68 (-1.36, 8.73)
2038	Male	54500.78 (0, 156905)	3.68 (-1.82, 9.18)
2039	Male	57658.14 (0, 180633.5)	3.68 (-2.29, 9.65)
2040	Male	61356.76 (0, 210228.81)	3.68 (-2.77, 10.13)
2041	Male	65702.67 (0, 247632.15)	3.68 (-3.27, 10.62)
2042	Male	70831.44 (0, 295564.22)	3.67 (-3.77, 11.11)
2043	Male	76878.05 (0, 357687.8)	3.67 (-4.28, 11.62)
2044	Male	84077.5 (0, 439477.05)	3.66 (-4.81, 12.13)
2045	Male	92713.64 (0, 548720.49)	3.66 (-5.34, 12.65)

Notes: ASR: age-standardized rates; YLDs: years lived with disability.

Table S91. Prevalence and YLDs of genecological disorders in China of future forecasts using bayesian age-period-cohort model.

Year	Sex	Number	ASR
**Prevalence**			
2022	Both	42691995.17 (37114661.65, 48269328.7)	2744.06 (2387.45, 3100.67)
2023	Both	42149418.84 (36287035.71, 48011801.97)	2716.55 (2340.87, 3092.22)
2024	Both	41611476.64 (35315238.62, 47907714.66)	2687.87 (2283.62, 3092.12)
2025	Both	41001602.68 (34142815.36, 47860390.01)	2657.54 (2215.71, 3099.38)
2026	Both	40244616.03 (32723359.28, 47765872.78)	2624.89 (2137.25, 3112.53)
2027	Both	39272783.59 (31028451.92, 47517115.26)	2589.79 (2049.11, 3130.47)
2028	Both	38042086.29 (29067724.88, 47016447.71)	2553.43 (1953.93, 3152.93)
2029	Both	36604701.88 (26921386.03, 46288017.74)	2515.82 (1852.93, 3178.7)
2030	Both	35048272.69 (24684991.05, 45411554.33)	2476.27 (1746.41, 3206.13)
2031	Both	33460331.17 (22440547.43, 44480114.91)	2434.11 (1634.44, 3233.79)
2032	Both	31935983.72 (20263100.74, 43608866.69)	2389.68 (1517.79, 3261.56)
2033	Both	30411148.71 (18126181.76, 42696115.66)	2344.25 (1398.28, 3290.22)
2034	Both	28821370.65 (16007072.66, 41635668.64)	2297.84 (1276.53, 3319.15)
2035	Both	27256530.81 (13967880.54, 40545758.19)	2250.14 (1152.73, 3347.62)
2036	Both	25808184.85 (12053052.44, 39565579.48)	2200.82 (1026.87, 3375.01)
2037	Both	24589341.4 (10295089.32, 38887363.75)	2150.82 (899.31, 3402.77)
2038	Both	23537321.73 (8654976.94, 38427930.88)	2101.9 (771.34, 3433.38)
2039	Both	22603963.09 (7146738.84, 38127018.09)	2054.54 (647.94, 3467.73)
2040	Both	21779027.3 (5698813.73, 37982756.01)	2008.89 (523.76, 3506.43)
2041	Both	21050847.52 (4352149.75, 37992099.77)	1964.79 (404.67, 3549.86)
2042	Both	20427202.85 (3082699.89, 38187221.94)	1922.93 (289.04, 3599.98)
2043	Both	19850744.31 (2111437.44, 38482463.82)	1884.39 (200.79, 3659.61)

2044	Both	19358027 (1652424.57, 38959723.91)	1849.73 (157.29, 3730.95)
2045	Both	18945974.66 (1180012.4, 39632533.66)	1819.1 (111.77, 3815.78)
2022	Female	30567278.87 (25536190.77, 35598366.97)	3949.32 (3580.2, 4318.44)
2023	Female	30157870.03 (24941961.06, 35373779)	3901.72 (3484.31, 4319.12)
2024	Female	29747280.75 (24246599.91, 35247961.58)	3850.86 (3369.81, 4331.91)
2025	Female	29275116.42 (23398929.52, 35151303.31)	3796.21 (3238.9, 4353.51)
2026	Female	28682259.94 (22358351.79, 35006168.1)	3736.74 (3093.31, 4380.17)
2027	Female	27914765.57 (21101137.83, 34728393.32)	3672.05 (2935.27, 4408.84)
2028	Female	26938230.56 (19635620.62, 34240840.49)	3603.87 (2769.18, 4438.56)
2029	Female	25793781.59 (18024783.72, 33562779.45)	3532.03 (2596.96, 4467.1)
2030	Female	24548491.69 (16341885.46, 32755097.92)	3455.19 (2419.02, 4491.35)
2031	Female	23269912.03 (14650413.23, 31889410.83)	3371.87 (2235.56, 4508.18)
2032	Female	22030884.53 (13008101.39, 31053667.68)	3282.01 (2047.88, 4516.14)
2033	Female	20784686.4 (11399878.87, 30169493.92)	3188.07 (1859.38, 4516.76)
2034	Female	19479107.84 (9810191.85, 29148023.83)	3090.48 (1671.6, 4509.37)
2035	Female	18183461.81 (8286502.53, 28080998.21)	2988.41 (1485.02, 4491.79)
2036	Female	16969321.21 (6862684.79, 27078219.85)	2880.96 (1300.07, 4461.85)
2037	Female	15922415.76 (5560482.27, 26288119.52)	2769.14 (1118.13, 4420.15)
2038	Female	15016591.86 (4364489.53, 25676958.56)	2656.28 (941.7, 4370.85)
2039	Female	14203664.34 (3291985.85, 25181173.57)	2543.86 (771.88, 4315.85)
2040	Female	13474606.1 (2275041.77, 24797685.57)	2432.12 (608.91, 4255.32)
2041	Female	12820075.27 (1359096.46, 24523608.55)	2320.87 (452.81, 4188.93)
2042	Female	12243837.54 (522466.03, 24380725.18)	2211.14 (303.85, 4118.43)
2043	Female	11715618.24 (0, 24323649.13)	2104.91 (162.6, 4047.23)
2044	Female	11257029.23 (0, 24407804.96)	2003.2 (29.04, 3977.36)
2045	Female	10864682.84 (0, 24643188.93)	1906.21 (-97.12, 3909.54)

2022	Male	12124716.3 (11578470.87, 12670961.73)	1561.7 (1517.35, 1606.06)
2023	Male	11991548.81 (11345074.65, 12638022.97)	1553 (1489.66, 1616.34)
2024	Male	11864195.89 (11068638.71, 12659753.07)	1544.61 (1456.85, 1632.37)
2025	Male	11726486.27 (10743885.84, 12709086.69)	1536.29 (1420.17, 1652.41)
2026	Male	11562356.09 (10365007.49, 12759704.68)	1527.66 (1379.97, 1675.34)
2027	Male	11358018.02 (9927314.1, 12788721.94)	1518.52 (1336.51, 1700.54)
2028	Male	11103855.74 (9432104.25, 12775607.22)	1509.47 (1290.84, 1728.09)
2029	Male	10810920.3 (8896602.31, 12725238.28)	1500.68 (1243.47, 1757.89)
2030	Male	10499781 (8343105.59, 12656456.42)	1491.97 (1194.39, 1789.54)
2031	Male	10190419.14 (7790134.2, 12590704.08)	1482.94 (1143.36, 1822.53)
2032	Male	9905099.18 (7254999.35, 12555199.01)	1473.52 (1090.36, 1856.68)
2033	Male	9626462.32 (6726302.89, 12526621.74)	1464.12 (1035.97, 1892.28)
2034	Male	9342262.81 (6196880.81, 12487644.81)	1454.89 (980.43, 1929.35)
2035	Male	9073069 (5681378.01, 12464759.98)	1445.6 (923.66, 1967.54)
2036	Male	8838863.64 (5190367.65, 12487359.63)	1435.82 (865.4, 2006.25)
2037	Male	8666925.64 (4734607.04, 12599244.23)	1425.61 (805.7, 2045.51)
2038	Male	8520729.87 (4290487.42, 12750972.33)	1415.29 (744.92, 2085.67)
2039	Male	8400298.75 (3854752.99, 12945844.51)	1405.07 (683.24, 2126.9)
2040	Male	8304421.2 (3423771.95, 13185070.44)	1394.82 (620.63, 2169.02)
2041	Male	8230772.26 (2993053.29, 13468491.23)	1384.25 (556.94, 2211.55)
2042	Male	8183365.31 (2560233.86, 13806496.75)	1373.44 (492.22, 2254.67)
2043	Male	8135126.07 (2111437.44, 14158814.7)	1362.6 (426.64, 2298.57)
2044	Male	8100997.77 (1652424.57, 14551918.95)	1351.86 (360.29, 2343.42)
2045	Male	8081291.82 (1180012.4, 14989344.73)	1341.21 (293.2, 2389.22)
**YLDs**			
2022	Both	227599.72 (204622.67, 250576.78)	14.72 (13.24, 16.2)

2023	Both	225091.04 (200378, 249804.08)	14.6 (13, 16.2)
2024	Both	222594.52 (195318.23, 249870.81)	14.48 (12.71, 16.25)
2025	Both	219709.72 (189160, 250259.45)	14.34 (12.35, 16.33)
2026	Both	216043.12 (181670.6, 250415.63)	14.19 (11.94, 16.45)
2027	Both	211227.03 (172696.38, 249757.68)	14.03 (11.47, 16.58)
2028	Both	205004.23 (162271.2, 247737.27)	13.85 (10.97, 16.74)
2029	Both	197639.14 (150821.87, 244456.42)	13.67 (10.43, 16.91)
2030	Both	189598.14 (138868.41, 240327.87)	13.47 (9.87, 17.08)
2031	Both	181355.33 (126862.92, 235847.73)	13.26 (9.28, 17.25)
2032	Both	173414.43 (115214.09, 231614.78)	13.04 (8.66, 17.41)
2033	Both	165416.7 (103755.18, 227078.22)	12.8 (8.03, 17.58)
2034	Both	157016.49 (92352.52, 221680.46)	12.56 (7.38, 17.74)
2035	Both	148703.54 (81360.94, 216046.14)	12.31 (6.73, 17.89)
2036	Both	140985.45 (71056.84, 210920.48)	12.05 (6.06, 18.03)
2037	Both	134476.87 (61653, 207314.4)	11.78 (5.39, 18.17)
2038	Both	128800.83 (52897.82, 204724.4)	11.51 (4.72, 18.31)
2039	Both	123713.27 (44726.52, 202848.12)	11.25 (4.06, 18.46)
2040	Both	119165.05 (37083.17, 201670.94)	11 (3.41, 18.62)
2041	Both	115105.81 (29724.88, 201180.25)	10.75 (2.76, 18.8)
2042	Both	111574.07 (23121.56, 201521.54)	10.51 (2.17, 19)
2043	Both	108275.9 (16984.41, 202255.23)	10.29 (1.61, 19.24)
2044	Both	105408.2 (12643.92, 203794.21)	10.08 (1.21, 19.53)
2045	Both	102956.6 (10205.32, 206192.43)	9.9 (0.98, 19.87)
2022	Female	161526.55 (141230.29, 181822.81)	21.02 (19.49, 22.54)
2023	Female	159589.17 (138110.33, 181068.01)	20.8 (19, 22.6)
2024	Female	157634.88 (134391.29, 180878.48)	20.56 (18.41, 22.72)

2025	Female	155346.24 (129816.79, 180875.68)	20.3 (17.73, 22.88)
2026	Female	152409.59 (124185.12, 180634.06)	20.02 (16.97, 23.06)
2027	Female	148534.61 (117376.05, 179693.16)	19.69 (16.14, 23.25)
2028	Female	143533.17 (109439.31, 177627.03)	19.35 (15.27, 23.43)
2029	Female	137613.77 (100715.58, 174511.97)	18.98 (14.36, 23.6)
2030	Female	131126.57 (91600.96, 170652.18)	18.58 (13.42, 23.75)
2031	Female	124428.05 (82438.64, 166417.47)	18.15 (12.44, 23.85)
2032	Female	117901.53 (73540.97, 162262.09)	17.67 (11.44, 23.9)
2033	Female	111301.3 (64820.17, 157782.43)	17.17 (10.43, 23.9)
2034	Female	104346.46 (56181.98, 152510.95)	16.64 (9.42, 23.86)
2035	Female	97401.94 (47889.43, 146914.45)	16.08 (8.41, 23.75)
2036	Female	90848.85 (40145.41, 141558.7)	15.49 (7.41, 23.58)
2037	Female	85144.46 (33092.24, 137210.36)	14.87 (6.42, 23.32)
2038	Female	80153.66 (26636.03, 133691.85)	14.24 (5.46, 23.02)
2039	Female	75622.81 (20722.95, 130670.78)	13.61 (4.53, 22.68)
2040	Female	71504.99 (15312.15, 128121.84)	12.97 (3.64, 22.3)
2041	Female	67754.25 (10178.58, 126023.44)	12.34 (2.8, 21.89)
2042	Female	64390.61 (5801.27, 124474.92)	11.71 (1.99, 21.43)
2043	Female	61269.95 (1972.98, 123254.77)	11.1 (1.23, 20.97)
2044	Female	58511.61 (0, 122644.96)	10.52 (0.52, 20.51)
2045	Female	56102.34 (0, 122689.22)	9.96 (-0.14, 20.06)
2022	Male	66073.18 (63392.38, 68753.97)	8.58 (8.37, 8.79)
2023	Male	65501.87 (62267.67, 68736.07)	8.55 (8.24, 8.87)
2024	Male	64959.63 (60926.94, 68992.33)	8.53 (8.09, 8.97)
2025	Male	64363.49 (59343.2, 69383.77)	8.5 (7.91, 9.1)
2026	Male	63633.53 (57485.49, 69781.57)	8.48 (7.72, 9.24)

2027	Male	62692.42 (55320.33, 70064.52)	8.45 (7.51, 9.39)
2028	Male	61471.06 (52831.88, 70110.24)	8.43 (7.29, 9.56)
2029	Male	60025.37 (50106.29, 69944.45)	8.4 (7.06, 9.74)
2030	Male	58471.57 (47267.45, 69675.69)	8.37 (6.82, 9.92)
2031	Male	56927.27 (44424.28, 69430.26)	8.35 (6.57, 10.12)
2032	Male	55512.91 (41673.12, 69352.69)	8.32 (6.31, 10.33)
2033	Male	54115.4 (38935.01, 69295.8)	8.29 (6.03, 10.54)
2034	Male	52670.03 (36170.54, 69169.51)	8.26 (5.75, 10.76)
2035	Male	51301.6 (33471.51, 69131.69)	8.23 (5.47, 10.99)
2036	Male	50136.6 (30911.43, 69361.78)	8.2 (5.17, 11.23)
2037	Male	49332.41 (28560.77, 70104.05)	8.17 (4.86, 11.47)
2038	Male	48647.17 (26261.79, 71032.55)	8.13 (4.55, 11.72)
2039	Male	48090.46 (24003.58, 72177.34)	8.1 (4.23, 11.98)
2040	Male	47660.06 (21771.02, 73549.1)	8.07 (3.9, 12.24)
2041	Male	47351.55 (19546.3, 75156.8)	8.03 (3.56, 12.51)
2042	Male	47183.45 (17320.29, 77046.62)	8 (3.22, 12.78)
2043	Male	47005.94 (15011.43, 79000.46)	7.97 (2.87, 13.06)
2044	Male	46896.58 (12643.92, 81149.25)	7.93 (2.52, 13.35)
2045	Male	46854.26 (10205.32, 83503.21)	7.9 (2.15, 13.64)

Notes: ASR: age-standardized rates; YLDs: years lived with disability.

Table S92. Prevalence and YLDs of mental disorders in China of future forecasts using bayesian age-period-cohort model.

Year	Sex	Number	ASR
**Prevalence**			
2022	Both	105946215.05 (97347051.66, 114545378.44)	6267.01 (5757.65, 6776.36)
2023	Both	108527754.37 (98962661.17, 118092847.56)	6377.72 (5814.85, 6940.59)
2024	Both	111169601.6 (100114650.93, 122224552.27)	6491.74 (5845.39, 7138.1)
2025	Both	113884041.29 (100785493.1, 126982589.49)	6609.67 (5848.66, 7370.67)
2026	Both	116670861.61 (100984087.31, 132357635.91)	6731.72 (5825.87, 7637.56)
2027	Both	119553609.59 (100749853.37, 138357365.81)	6857.83 (5778.5, 7937.15)
2028	Both	122398713.23 (99989917.88, 144807508.58)	6988.76 (5708.58, 8268.95)
2029	Both	125291877.16 (98789335.83, 151794418.49)	7125.24 (5617.39, 8633.09)
2030	Both	128237912.77 (97152566.31, 159323259.24)	7267.94 (5505.48, 9030.4)
2031	Both	131227267.88 (95064449.35, 167390086.4)	7417.36 (5372.58, 9462.13)
2032	Both	134264217.13 (92514602.74, 176013831.53)	7573.59 (5217.67, 9929.51)
2033	Both	137307079.24 (89459663.51, 185154494.98)	7737.58 (5040.11, 10435.05)
2034	Both	140411887.71 (85917439.4, 194906336.02)	7910.13 (4838.67, 10981.58)
2035	Both	143581373.6 (81860516.92, 205302230.28)	8092.11 (4611.63, 11572.58)
2036	Both	146803505.43 (77247115.68, 216359895.18)	8284.35 (4356.65, 12212.05)
2037	Both	150067245.31 (72032996.7, 228101493.92)	8487.23 (4070.61, 12903.86)
2038	Both	153431744.11 (66203767.57, 240659720.66)	8702.02 (3750.49, 13653.55)
2039	Both	156919773.65 (59713404.05, 254126143.24)	8929.83 (3392.54, 14467.11)
2040	Both	160541864.62 (52498595.61, 268585133.62)	9171.98 (2992.26, 15351.69)
2041	Both	164295135.14 (44480292.62, 284109977.67)	9429.84 (2544.14, 16315.55)
2042	Both	168177554.57 (35569917.53, 300785191.61)	9704.3 (2041.47, 17367.13)
2043	Both	172251899.81 (25686214.71, 318822267.28)	9997.11 (1477.8, 18517.39)

2044	Both	176544228.87 (16121245.14, 338391539.85)	10310.01 (952.59, 19778.56)
2045	Both	181074376.57 (13372802.51, 359675240.53)	10645.04 (789.24, 21165.22)
2022	Female	65340303.47 (60156269.94, 70524337)	7820.69 (7566.62, 8074.75)
2023	Female	67134178.05 (61265923.98, 73002432.12)	7981.61 (7596.9, 8366.33)
2024	Female	68978767.31 (62043835.77, 75913698.85)	8146.62 (7588.03, 8705.22)
2025	Female	70881974.58 (62481954.75, 79281994.42)	8316.25 (7548.25, 9084.25)
2026	Female	72842379.21 (62590269.93, 83094488.48)	8490.46 (7481.14, 9499.78)
2027	Female	74875010.73 (62396649.61, 87353371.85)	8668.52 (7387.47, 9949.57)
2028	Female	76899451.38 (61846462.12, 91952440.65)	8850.85 (7268.23, 10433.48)
2029	Female	78970601.18 (60991216.31, 96949986.04)	9038.02 (7123.83, 10952.21)
2030	Female	81092672.12 (59830653.37, 102354690.88)	9230.57 (6954.21, 11506.93)
2031	Female	83259188.51 (58349963.64, 108168413.38)	9428.78 (6758.77, 12098.79)
2032	Female	85473431.94 (56537128.77, 114409735.12)	9631.96 (6536.05, 12727.87)
2033	Female	87709701.51 (54359694.39, 121059708.62)	9840.45 (6285.47, 13395.43)
2034	Female	90005739.58 (51822447.34, 128189031.82)	10054.69 (6006.35, 14103.04)
2035	Female	92364505.25 (48900685.47, 135828325.02)	10275.21 (5697.82, 14852.6)
2036	Female	94778100.07 (45559014.4, 143997185.74)	10502.41 (5358.75, 15646.08)
2037	Female	97239861.07 (41759798.33, 152719923.81)	10735.59 (4987.28, 16483.9)
2038	Female	99791128.89 (37482560.15, 162099697.63)	10975.05 (4582.29, 17367.81)
2039	Female	102449240.05 (32685012.17, 172213467.94)	11221.21 (4142.51, 18299.91)
2040	Female	105223533.09 (27311852.28, 183135213.9)	11474.67 (3666.6, 19282.74)
2041	Female	108112910.91 (21294986.61, 194930835.22)	11736.03 (3152.92, 20319.13)
2042	Female	111117378.67 (14557727.82, 207677029.53)	12004.54 (2599.37, 21409.71)
2043	Female	114282801.89 (7024206.63, 221546079.54)	12280.4 (2004.27, 22556.53)
2044	Female	117630585.77 (0, 236685498.78)	12563.97 (1365.83, 23762.1)
2045	Female	121178224.77 (0, 253255739.45)	12855.93 (682.14, 25029.72)

2022	Male	40605911.58 (37190781.72, 44021041.44)	4766.3 (4616.62, 4915.97)
2023	Male	41393576.31 (37696737.19, 45090415.44)	4828.59 (4622.55, 5034.62)
2024	Male	42190834.29 (38070815.15, 46310853.42)	4891.94 (4610.85, 5173.04)
2025	Male	43002066.71 (38303538.34, 47700595.07)	4956.61 (4585.19, 5328.03)
2026	Male	43828482.4 (38393817.38, 49263147.42)	5022.54 (4547.61, 5497.48)
2027	Male	44678598.86 (38353203.76, 51003993.97)	5089.43 (4498.98, 5679.88)
2028	Male	45499261.84 (38143455.76, 52855067.93)	5157.57 (4440.34, 5874.8)
2029	Male	46321275.99 (37798119.52, 54844432.46)	5227.26 (4372.31, 6082.21)
2030	Male	47145240.65 (37321912.94, 56968568.36)	5298.67 (4295.13, 6302.21)
2031	Male	47968079.37 (36714485.71, 59221673.02)	5371.75 (4208.7, 6534.79)
2032	Male	48790785.19 (35977473.97, 61604096.41)	5446.16 (4112.64, 6779.68)
2033	Male	49597377.73 (35099969.12, 64094786.35)	5522.2 (4007.13, 7037.28)
2034	Male	50406148.13 (34094992.06, 66717304.2)	5600.09 (3892.19, 7308)
2035	Male	51216868.35 (32959831.45, 69473905.26)	5679.96 (3767.67, 7592.25)
2036	Male	52025405.35 (31688101.28, 72362709.43)	5761.73 (3633.25, 7890.22)
2037	Male	52827384.24 (30273198.37, 75381570.11)	5845.07 (3488.39, 8201.75)
2038	Male	53640615.22 (28721207.42, 78560023.03)	5930.25 (3333.07, 8527.44)
2039	Male	54470533.59 (27028391.89, 81912675.3)	6017.5 (3167.12, 8867.89)
2040	Male	55318331.53 (25186743.33, 85449919.72)	6106.96 (2990.29, 9223.63)
2041	Male	56182224.23 (23185306.01, 89179142.45)	6198.6 (2802.14, 9595.06)
2042	Male	57060175.9 (21012189.71, 93108162.08)	6292.09 (2602.13, 9982.06)
2043	Male	57969097.92 (18662008.09, 97276187.74)	6387.7 (2390.03, 10385.36)
2044	Male	58913643.1 (16121245.14, 101706041.07)	6485.61 (2165.54, 10805.68)
2045	Male	59896151.8 (13372802.51, 106419501.08)	6586 (1928.28, 11243.73)
**YLDs**			
2022	Both	14067121.9 (12833117.56, 15301126.23)	833.63 (760.4, 906.86)

2023	Both	14291397.57 (12964822.96, 15617972.18)	841.61 (763.37, 919.86)
2024	Both	14518143.96 (13051188.75, 15985099.16)	849.8 (763.78, 935.82)
2025	Both	14748557.54 (13088631.27, 16408483.81)	858.25 (761.47, 955.02)
2026	Both	14982254.73 (13076145.66, 16888363.8)	866.94 (756.43, 977.46)
2027	Both	15222550.74 (13018307.19, 17426794.3)	875.89 (748.78, 1002.99)
2028	Both	15451869.27 (12903004.64, 18000733.91)	885.15 (738.8, 1031.5)
2029	Both	15680631.14 (12742014.65, 18619247.63)	894.8 (726.7, 1062.91)
2030	Both	15909109.81 (12537451.11, 19280768.5)	904.89 (712.6, 1097.19)
2031	Both	16135821.69 (12289312.38, 19982330.99)	915.44 (696.57, 1134.31)
2032	Both	16361964.66 (11999269.14, 20724660.18)	926.43 (678.61, 1174.26)
2033	Both	16580128.43 (11662597.81, 21497659.06)	937.96 (658.77, 1217.15)
2034	Both	16797344.03 (11284763.06, 22309924.99)	950.09 (637.06, 1263.12)
2035	Both	17013274.06 (10865113.89, 23161434.22)	962.86 (613.41, 1312.32)
2036	Both	17225810.72 (10401547.4, 24050074.03)	976.32 (587.72, 1364.93)
2037	Both	17433864.49 (9892628, 24975100.99)	990.48 (559.84, 1421.13)
2038	Both	17643002 (9340662.05, 25945341.95)	1005.43 (529.67, 1481.2)
2039	Both	17855411.47 (8745145.5, 26965677.45)	1021.25 (497.04, 1545.46)
2040	Both	18070935.61 (8103696.63, 28038174.59)	1038 (461.75, 1614.25)
2041	Both	18287850.24 (7412861.34, 29162839.13)	1055.75 (423.55, 1687.96)
2042	Both	18505197.89 (6669442.74, 30340953.05)	1074.54 (382.12, 1766.97)
2043	Both	18730184.75 (5872995.64, 31587373.85)	1094.47 (337.16, 1851.79)
2044	Both	18963966.76 (5019632.37, 32908301.15)	1115.64 (288.3, 1942.98)
2045	Both	19206808.07 (4104382.69, 34309233.45)	1138.15 (235.1, 2041.2)
2022	Female	8650400.74 (7897760.91, 9403040.58)	1036.41 (1002.7, 1070.11)
2023	Female	8793957.1 (7983944.26, 9603969.94)	1046.73 (1000.8, 1092.66)
2024	Female	8939557.45 (8042506.27, 9836608.64)	1057.24 (995.08, 1119.4)

2025	Female	9087786.52 (8071043.99, 10104529.05)	1067.99 (986.38, 1149.6)
2026	Female	9238050.11 (8068587.03, 10407513.2)	1078.94 (975.11, 1182.76)
2027	Female	9392373.53 (8037923.81, 10746823.26)	1090.01 (961.48, 1218.54)
2028	Female	9540211.88 (7971811.03, 11108612.73)	1101.28 (945.72, 1256.84)
2029	Female	9687996.63 (7877474.18, 11498519.08)	1112.79 (927.97, 1297.61)
2030	Female	9835827.16 (7756106.41, 11915547.91)	1124.59 (908.31, 1340.87)
2031	Female	9982514.46 (7607439.51, 12357589.41)	1136.67 (886.75, 1386.59)
2032	Female	10128791.12 (7432467.79, 12825114.45)	1148.97 (863.24, 1434.7)
2033	Female	10270125.56 (7228283.01, 13311968.11)	1161.53 (837.81, 1485.25)
2034	Female	10410873.25 (6998228.77, 13823517.73)	1174.4 (810.5, 1538.3)
2035	Female	10550708.79 (6741795.08, 14359622.49)	1187.6 (781.28, 1593.91)
2036	Female	10688051.79 (6457487.07, 14918616.5)	1201.14 (750.13, 1652.15)
2037	Female	10822152.13 (6144379.39, 15499924.86)	1214.97 (716.96, 1712.97)
2038	Female	10956806.71 (5804113.49, 16109499.94)	1229.1 (681.76, 1776.45)
2039	Female	11093301.5 (5436327.69, 16750275.3)	1243.59 (644.5, 1842.67)
2040	Female	11231476.44 (5039507.69, 17423445.18)	1258.45 (605.16, 1911.74)
2041	Female	11370008.11 (4611402.04, 18128614.19)	1273.72 (563.66, 1983.77)
2042	Female	11508229.39 (4150033.99, 18866424.79)	1289.32 (519.93, 2058.72)
2043	Female	11650839.98 (3655254.4, 19646425.56)	1305.29 (473.9, 2136.68)
2044	Female	11798567.77 (3124674.32, 20472461.22)	1321.64 (425.53, 2217.74)
2045	Female	11951556.99 (2555221.44, 21347892.54)	1338.41 (374.76, 2302.05)
2022	Male	5416721.15 (4935356.66, 5898085.65)	637.61 (616.55, 658.68)
2023	Male	5497440.47 (4980878.69, 6014002.24)	643.38 (614.81, 671.94)
2024	Male	5578586.5 (5008682.48, 6148490.52)	649.22 (610.71, 687.72)
2025	Male	5660771.02 (5017587.28, 6303954.76)	655.17 (604.77, 705.56)
2026	Male	5744204.61 (5007558.63, 6480850.6)	661.21 (597.25, 725.17)

2027	Male	5830177.21 (4980383.37, 6679971.05)	667.32 (588.3, 746.33)
2028	Male	5911657.4 (4931193.61, 6892121.18)	673.52 (578.06, 768.98)
2029	Male	5992634.51 (4864540.47, 7120728.55)	679.86 (566.64, 793.09)
2030	Male	6073282.65 (4781344.7, 7365220.59)	686.36 (554.07, 818.65)
2031	Male	6153307.22 (4681872.87, 7624741.58)	693 (540.37, 845.64)
2032	Male	6233173.55 (4566801.35, 7899545.74)	699.76 (525.5, 874.01)
2033	Male	6310002.87 (4434314.8, 8185690.95)	706.66 (509.51, 903.8)
2034	Male	6386470.78 (4286534.29, 8486407.27)	713.73 (492.41, 935.04)
2035	Male	6462565.27 (4123318.81, 8801811.74)	720.98 (474.2, 967.77)
2036	Male	6537758.93 (3944060.33, 9131457.52)	728.42 (454.85, 1001.99)
2037	Male	6611712.37 (3748248.61, 9475176.12)	735.99 (434.31, 1037.67)
2038	Male	6686195.29 (3536548.56, 9835842.01)	743.73 (412.6, 1074.87)
2039	Male	6762109.97 (3308817.81, 10215402.14)	751.67 (389.7, 1113.64)
2040	Male	6839459.17 (3064188.94, 10614729.41)	759.83 (365.6, 1154.05)
2041	Male	6917842.12 (2801459.3, 11034224.95)	768.18 (340.25, 1196.1)
2042	Male	6996968.5 (2519408.74, 11474528.27)	776.7 (313.61, 1239.79)
2043	Male	7079344.77 (2217741.24, 11940948.3)	785.42 (285.66, 1285.18)
2044	Male	7165398.99 (1894958.05, 12435839.93)	794.36 (256.38, 1332.34)
2045	Male	7255251.08 (1549161.26, 12961340.9)	803.54 (225.73, 1381.34)

Notes: ASR: age-standardized rates; YLDs: years lived with disability.

Table S93. Prevalence and YLDs of substance use disorders in China of future forecasts using bayesian age-period-cohort model.

Year	Sex	Number	ASR
**Prevalence**			
2022	Both	1517937.35 (1374500.77, 1661373.93)	129.8 (117.56, 142.03)
2023	Both	1534144.88 (1355311.28, 1712978.48)	131.93 (116.55, 147.31)
2024	Both	1550713.21 (1317948.44, 1783477.99)	134.25 (114.06, 154.44)
2025	Both	1568535.2 (1266056, 1871014.4)	136.78 (110.3, 163.25)
2026	Both	1588312.83 (1202089.58, 1974536.08)	139.54 (105.43, 173.65)
2027	Both	1611155.45 (1127691.92, 2094618.98)	142.56 (99.51, 185.61)
2028	Both	1637074.53 (1042870.2, 2231278.85)	145.91 (92.56, 199.26)
2029	Both	1666118.76 (947040.87, 2385196.66)	149.64 (84.52, 214.76)
2030	Both	1699479.21 (839665.12, 2559293.31)	153.81 (75.28, 232.34)
2031	Both	1738238.75 (719298.42, 2757179.08)	158.45 (64.65, 252.26)
2032	Both	1784244.08 (583831.7, 2984656.45)	163.63 (52.37, 274.9)
2033	Both	1835704.14 (429216.7, 3242191.57)	169.45 (38.13, 300.76)
2034	Both	1894161.45 (256379.05, 3536698.53)	175.99 (22.08, 330.48)
2035	Both	1961481.41 (167820.84, 3877269.61)	183.38 (14.37, 364.83)
2036	Both	2039584.59 (65615.85, 4275224.46)	191.71 (5.41, 404.76)
2037	Both	2132214.29 (0, 4748642.09)	201.12 (0, 451.53)
2038	Both	2231593.07 (0, 5292128.7)	211.8 (0, 506.8)
2039	Both	2343085.59 (0, 5932180.76)	223.98 (0, 572.74)
2040	Both	2469016.29 (0, 6694072.36)	237.89 (0, 652.17)
2041	Both	2611740.28 (0, 7610103.04)	253.83 (0, 748.79)
2042	Both	2774921.07 (0, 8725811.43)	272.15 (0, 867.62)
2043	Both	2958830.03 (0, 10089121.18)	293.33 (0, 1015.56)

2044	Both	3168984.63 (0, 11780903.33)	317.94 (0, 1202.08)
2045	Both	3410046.64 (0, 13904740.8)	346.68 (0, 1440.27)
2022	Female	807336.65 (715252.67, 899420.63)	136.78 (129.23, 144.33)
2023	Female	819919.96 (713679.37, 926160.55)	139.36 (127.93, 150.79)
2024	Female	832680.25 (703909.39, 961451.12)	142.07 (125.44, 158.71)
2025	Female	846026.4 (686655.13, 1005397.67)	144.93 (122, 167.86)
2026	Female	860273.23 (662881.23, 1057665.23)	147.9 (117.68, 178.12)
2027	Female	875951.3 (633479.13, 1118423.48)	150.99 (112.51, 189.46)
2028	Female	893048.92 (598606.7, 1187491.15)	154.21 (106.53, 201.9)
2029	Female	911553.36 (558128.86, 1264977.87)	157.6 (99.73, 215.48)
2030	Female	932035.28 (511996.14, 1352074.42)	161.15 (92.08, 230.22)
2031	Female	955003.28 (459764.07, 1450242.5)	164.83 (83.54, 246.13)
2032	Female	981356.07 (400786.05, 1561926.1)	168.65 (74.06, 263.24)
2033	Female	1010227.92 (333430.64, 1687025.2)	172.62 (63.62, 281.62)
2034	Female	1042281.59 (256379.05, 1828184.13)	176.77 (52.17, 301.36)
2035	Female	1078307.36 (167820.84, 1988793.88)	181.08 (39.67, 322.5)
2036	Female	1119075.89 (65615.85, 2172895.7)	185.55 (26.04, 345.07)
2037	Female	1166233.5 (0, 2387139.23)	190.17 (11.23, 369.11)
2038	Female	1215951.49 (0, 2627599.78)	194.95 (-4.82, 394.73)
2039	Female	1270629.01 (0, 2903617.5)	199.93 (-22.17, 422.02)
2040	Female	1331083.55 (0, 3222767.72)	205.08 (-40.9, 451.06)
2041	Female	1398033.9 (0, 3594054.65)	210.41 (-61.07, 481.89)
2042	Female	1472743.24 (0, 4029957.47)	215.91 (-82.76, 514.58)
2043	Female	1554806.65 (0, 4541407.82)	221.6 (-106.05, 549.26)
2044	Female	1646230.64 (0, 5148865)	227.5 (-131.04, 586.04)
2045	Female	1748261.69 (0, 5875504.58)	233.61 (-157.81, 625.03)

2022	Male	710600.7 (659248.1, 761953.3)	122.25 (116.6, 127.9)
2023	Male	714224.92 (641631.92, 786817.93)	123.77 (113.26, 134.27)
2024	Male	718032.96 (614039.05, 822026.87)	125.36 (108.65, 142.08)
2025	Male	722508.8 (579400.88, 865616.73)	127.04 (102.99, 151.1)
2026	Male	728039.6 (539208.35, 916870.84)	128.79 (96.35, 161.22)
2027	Male	735204.15 (494212.79, 976195.5)	130.58 (88.77, 172.39)
2028	Male	744025.61 (444263.5, 1043787.71)	132.45 (80.28, 184.62)
2029	Male	754565.4 (388912.01, 1120218.79)	134.4 (70.89, 197.91)
2030	Male	767443.93 (327668.98, 1207218.89)	136.43 (60.59, 212.28)
2031	Male	783235.46 (259534.35, 1306936.58)	138.53 (49.37, 227.7)
2032	Male	802888 (183045.65, 1422730.35)	140.69 (37.2, 244.19)
2033	Male	825476.22 (95786.07, 1555166.37)	142.92 (24.06, 261.78)
2034	Male	851879.86 (0, 1708514.41)	145.24 (9.95, 280.53)
2035	Male	883174.05 (0, 1888475.74)	147.64 (-5.18, 300.46)
2036	Male	920508.7 (0, 2102328.75)	150.11 (-21.36, 321.57)
2037	Male	965980.79 (0, 2361502.86)	152.64 (-38.62, 343.9)
2038	Male	1015641.58 (0, 2664528.92)	155.25 (-56.99, 367.49)
2039	Male	1072456.57 (0, 3028563.26)	157.94 (-76.52, 392.4)
2040	Male	1137932.74 (0, 3471304.64)	160.71 (-97.25, 418.68)
2041	Male	1213706.38 (0, 4016048.39)	163.56 (-119.2, 446.33)
2042	Male	1302177.84 (0, 4695853.97)	166.48 (-142.44, 475.4)
2043	Male	1404023.38 (0, 5547713.36)	169.48 (-166.99, 505.95)
2044	Male	1522753.99 (0, 6632038.33)	172.56 (-192.93, 538.05)
2045	Male	1661784.95 (0, 8029236.21)	175.73 (-220.29, 571.74)
**YLDs**			
2022	Both	630450.96 (570721.99, 690179.93)	54.39 (49.25, 59.53)

2023	Both	636361.73 (561636.13, 711087.32)	55.26 (48.77, 61.75)
2024	Both	642399.67 (544898.52, 739900.82)	56.21 (47.67, 64.76)
2025	Both	648950.35 (522098.24, 775802.46)	57.26 (46.03, 68.48)
2026	Both	656317.59 (494300.08, 818335.09)	58.4 (43.92, 72.88)
2027	Both	664983.29 (462214.36, 867752.22)	59.65 (41.36, 77.94)
2028	Both	674943.88 (425838.38, 924049.37)	61.04 (38.37, 83.71)
2029	Both	686202.7 (384915.62, 987489.79)	62.59 (34.91, 90.27)
2030	Both	699269.76 (339220.99, 1059318.52)	64.33 (30.94, 97.71)
2031	Both	714609.72 (288142.54, 1141076.91)	66.27 (26.38, 106.15)
2032	Both	733019.1 (230780.72, 1235257.48)	68.44 (21.12, 115.76)
2033	Both	753700.5 (165409.81, 1341991.18)	70.87 (15.01, 126.74)
2034	Both	777299.15 (92016, 1464199.65)	73.62 (8.09, 139.37)
2035	Both	804615.06 (53819.88, 1605766.56)	76.73 (4.69, 153.99)
2036	Both	836475.28 (10271.42, 1771540.22)	80.25 (0.82, 171.01)
2037	Both	874493.66 (0, 1969301.07)	84.22 (0, 190.98)
2038	Both	915307.52 (0, 2196587.36)	88.74 (0, 214.61)
2039	Both	961188.42 (0, 2464697.4)	93.9 (0, 242.84)
2040	Both	1013123.08 (0, 2784414.09)	99.8 (0, 276.9)
2041	Both	1072115.11 (0, 3169533.11)	106.57 (0, 318.4)
2042	Both	1139724.82 (0, 3639521.81)	114.36 (0, 369.53)
2043	Both	1216096.94 (0, 4214948.63)	123.39 (0, 433.28)
2044	Both	1303536.42 (0, 4930271.2)	133.88 (0, 513.77)
2045	Both	1404007.73 (0, 5829666.32)	146.16 (0, 616.7)
2022	Female	332034.92 (293845.53, 370224.31)	56.85 (53.63, 60.06)
2023	Female	336682.23 (292443.08, 380921.37)	57.89 (53, 62.78)
2024	Female	341380.63 (287526.44, 395234.81)	58.99 (51.86, 66.12)

2025	Female	346309.69 (279452.45, 413166.93)	60.15 (50.31, 69.98)
2026	Female	351610.47 (268662.48, 434558.45)	61.35 (48.38, 74.31)
2027	Female	357517.52 (255548.22, 459486.82)	62.6 (46.1, 79.1)
2028	Female	364020.4 (240169.8, 487871)	63.9 (43.46, 84.35)
2029	Female	371104.41 (222459.34, 519749.49)	65.28 (40.47, 90.08)
2030	Female	379016.44 (202389.62, 555643.25)	66.71 (37.11, 96.31)
2031	Female	387979.34 (179761.73, 596196.96)	68.2 (33.38, 103.03)
2032	Female	398384.77 (154280.91, 642488.64)	69.75 (29.25, 110.25)
2033	Female	409850.45 (125231.03, 694469.87)	71.36 (24.7, 118.01)
2034	Female	422653.05 (92016, 753290.09)	73.03 (19.73, 126.33)
2035	Female	437137.97 (53819.88, 820456.07)	74.78 (14.31, 135.25)
2036	Female	453647.34 (10271.42, 897782.45)	76.58 (8.41, 144.76)
2037	Female	472901.33 (0, 988251.88)	78.45 (2.01, 154.89)
2038	Female	493265.25 (0, 1090232.94)	80.38 (-4.92, 165.69)
2039	Female	515757.89 (0, 1207883.61)	82.39 (-12.4, 177.18)
2040	Female	540743.87 (0, 1344688.81)	84.47 (-20.46, 189.4)
2041	Female	568554.69 (0, 1504848.94)	86.62 (-29.12, 202.37)
2042	Female	599759.52 (0, 1694201.19)	88.84 (-38.43, 216.11)
2043	Female	634224.38 (0, 1918055.56)	91.13 (-48.42, 230.68)
2044	Female	672819.96 (0, 2186027.03)	93.51 (-59.12, 246.13)
2045	Female	716122.77 (0, 2509264.63)	95.97 (-70.56, 262.49)
2022	Male	298416.04 (276876.46, 319955.62)	51.69 (49.29, 54.09)
2023	Male	299679.5 (269193.05, 330165.95)	52.33 (47.87, 56.78)
2024	Male	301019.04 (257372.07, 344666.01)	52.99 (45.91, 60.07)
2025	Male	302640.66 (242645.78, 362635.53)	53.69 (43.52, 63.87)
2026	Male	304707.12 (225637.6, 383776.64)	54.42 (40.71, 68.14)

2027	Male	307465.77 (206666.13, 408265.4)	55.17 (37.5, 72.85)
2028	Male	310923.47 (185668.58, 436178.37)	55.95 (33.91, 78)
2029	Male	315098.29 (162456.28, 467740.3)	56.77 (29.95, 83.6)
2030	Male	320253.32 (136831.37, 503675.27)	57.62 (25.6, 89.65)
2031	Male	326630.38 (108380.81, 544879.95)	58.5 (20.86, 96.15)
2032	Male	334634.33 (76499.81, 592768.84)	59.41 (15.73, 103.09)
2033	Male	343850.04 (40178.78, 647521.31)	60.35 (10.19, 110.5)
2034	Male	354646.11 (0, 710909.55)	61.32 (4.24, 118.4)
2035	Male	367477.09 (0, 785310.5)	62.33 (-2.14, 126.79)
2036	Male	382827.94 (0, 873757.76)	63.36 (-8.96, 135.68)
2037	Male	401592.33 (0, 981049.19)	64.43 (-16.23, 145.09)
2038	Male	422042.26 (0, 1106354.42)	65.52 (-23.97, 155.02)
2039	Male	445430.53 (0, 1256813.8)	66.65 (-32.2, 165.5)
2040	Male	472379.21 (0, 1439725.27)	67.82 (-40.93, 176.57)
2041	Male	503560.41 (0, 1664684.17)	69.01 (-50.18, 188.21)
2042	Male	539965.3 (0, 1945320.61)	70.24 (-59.96, 200.45)
2043	Male	581872.57 (0, 2296893.07)	71.5 (-70.3, 213.3)
2044	Male	630716.46 (0, 2744244.17)	72.79 (-81.22, 226.81)
2045	Male	687884.96 (0, 3320401.69)	74.12 (-92.74, 240.99)

Notes: ASR: age-standardized rates; YLDs: years lived with disability.

Table S94. Prevalence and YLDs of infectious disorders in China of future forecasts using bayesian age-period-cohort model.

Year	Sex	Number	ASR
**Prevalence**			
2022	Both	1123499.94 (1062206.59, 1184793.29)	70.25 (66.32, 74.18)
2023	Both	1132135.72 (1058399.66, 1205871.78)	69.99 (65.3, 74.67)
2024	Both	1141545.03 (1048067.67, 1235022.38)	69.73 (63.87, 75.6)
2025	Both	1151262.66 (1031775.85, 1270749.47)	69.49 (62.1, 76.88)
2026	Both	1160987.14 (1010263.21, 1311711.07)	69.27 (60.08, 78.46)
2027	Both	1170533.97 (984066.15, 1357001.79)	69.07 (57.84, 80.29)
2028	Both	1180401.18 (954064.86, 1406737.5)	68.89 (55.44, 82.34)
2029	Both	1190924.6 (920755.24, 1461093.95)	68.74 (52.88, 84.6)
2030	Both	1201964.51 (884098.47, 1519830.55)	68.62 (50.18, 87.06)
2031	Both	1213400.63 (843989.6, 1582811.66)	68.54 (47.36, 89.72)
2032	Both	1225211.51 (800338.24, 1650084.78)	68.49 (44.4, 92.58)
2033	Both	1237571.46 (753161.5, 1721981.42)	68.49 (41.32, 95.66)
2034	Both	1250917.62 (702540.75, 1799294.5)	68.53 (38.1, 98.96)
2035	Both	1265129.88 (648130.44, 1882129.32)	68.62 (34.74, 102.5)
2036	Both	1280080.6 (589540.92, 1970620.28)	68.76 (31.23, 106.3)
2037	Both	1295763.89 (526396.33, 2065131.46)	68.97 (27.55, 110.38)
2038	Both	1312289.31 (458340.3, 2166238.33)	69.23 (23.69, 114.77)
2039	Both	1330128.81 (385036.63, 2275220.99)	69.56 (19.62, 119.5)
2040	Both	1349121.88 (305810.41, 2392433.35)	69.95 (15.31, 124.6)
2041	Both	1369067.81 (219929.89, 2518210.07)	70.43 (10.74, 130.11)
2042	Both	1389870.77 (129525.7, 2653136.45)	70.98 (6.46, 136.1)
2043	Both	1411567.65 (91015.93, 2798114.58)	71.62 (4.33, 142.6)

2044	Both	1434758.85 (52311.08, 2955336.37)	72.35 (2.29, 149.7)
2045	Both	1459379.23 (12689.44, 3125905.93)	73.19 (0.53, 157.46)
2022	Female	597011.29 (562269.89, 631752.69)	73.77 (71.61, 75.92)
2023	Female	602717.79 (560652.29, 644783.3)	73.48 (69.95, 77.01)
2024	Female	608845.08 (555129.01, 662561.15)	73.19 (67.92, 78.46)
2025	Female	615170.21 (546086.43, 684254)	72.91 (65.63, 80.19)
2026	Female	621543.67 (533976.19, 709111.15)	72.62 (63.11, 82.13)
2027	Female	627879.53 (519115.68, 736643.39)	72.33 (60.4, 84.26)
2028	Female	634417.52 (501949.36, 766885.68)	72.04 (57.52, 86.56)
2029	Female	641357.94 (482745.66, 799970.22)	71.75 (54.49, 89.01)
2030	Female	648636.23 (461474.38, 835798.07)	71.46 (51.32, 91.6)
2031	Female	656190.36 (438058.01, 874322.7)	71.17 (48.02, 94.32)
2032	Female	664011.28 (412420.51, 915602.04)	70.88 (44.6, 97.16)
2033	Female	672202.98 (384537.79, 959868.18)	70.58 (41.06, 100.1)
2034	Female	681011.25 (354405.87, 1007616.63)	70.29 (37.42, 103.16)
2035	Female	690375.56 (321789.23, 1058961.89)	69.99 (33.69, 106.3)
2036	Female	700226.83 (286419.98, 1114033.69)	69.7 (29.86, 109.54)
2037	Female	710564.12 (248027.81, 1173100.42)	69.4 (25.94, 112.85)
2038	Female	721476.01 (206347.25, 1236604.78)	69.1 (21.94, 116.25)
2039	Female	733243.54 (161088.75, 1305398.32)	68.79 (17.87, 119.72)
2040	Female	745792.76 (111776.26, 1379809.26)	68.49 (13.72, 123.25)
2041	Female	759019.21 (57887.18, 1460155.57)	68.18 (9.51, 126.84)
2042	Female	772881.35 (1756.82, 1546926.49)	67.86 (5.24, 130.49)
2043	Female	787445.47 (0, 1640886.17)	67.55 (0.9, 134.19)
2044	Female	803075.59 (0, 1743560.2)	67.23 (-3.48, 137.94)
2045	Female	819756.44 (0, 1855861.33)	66.91 (-7.92, 141.74)

2022	Male	526488.65 (499936.7, 553040.6)	66.67 (64.99, 68.35)
2023	Male	529417.93 (497747.37, 561088.48)	66.4 (63.72, 69.08)
2024	Male	532699.95 (492938.66, 572461.23)	66.13 (62.18, 70.09)
2025	Male	536092.44 (485689.42, 586495.47)	65.86 (60.43, 71.29)
2026	Male	539443.47 (476287.02, 602599.92)	65.59 (58.53, 72.66)
2027	Male	542654.44 (464950.47, 620358.41)	65.32 (56.48, 74.16)
2028	Male	545983.67 (452115.5, 639851.83)	65.05 (54.31, 75.79)
2029	Male	549566.66 (438009.58, 661123.73)	64.77 (52.03, 77.52)
2030	Male	553328.28 (422624.09, 684032.48)	64.5 (49.64, 79.36)
2031	Male	557210.27 (405931.59, 708488.96)	64.22 (47.16, 81.28)
2032	Male	561200.23 (387917.72, 734482.73)	63.95 (44.6, 83.3)
2033	Male	565368.48 (368623.72, 762113.24)	63.67 (41.95, 85.39)
2034	Male	569906.37 (348134.88, 791677.87)	63.39 (39.23, 87.56)
2035	Male	574754.33 (326341.22, 823167.43)	63.11 (36.43, 89.79)
2036	Male	579853.76 (303120.95, 856586.58)	62.83 (33.57, 92.09)
2037	Male	585199.78 (278368.51, 892031.04)	62.55 (30.65, 94.44)
2038	Male	590813.3 (251993.05, 929633.55)	62.26 (27.67, 96.85)
2039	Male	596885.28 (223947.88, 969822.67)	61.98 (24.64, 99.32)
2040	Male	603329.12 (194034.15, 1012624.1)	61.69 (21.55, 101.82)
2041	Male	610048.6 (162042.71, 1058054.5)	61.39 (18.41, 104.37)
2042	Male	616989.42 (127768.88, 1106209.96)	61.1 (15.24, 106.96)
2043	Male	624122.17 (91015.93, 1157228.41)	60.8 (12.01, 109.59)
2044	Male	631683.26 (52311.08, 1211776.17)	60.5 (8.76, 112.24)
2045	Male	639622.79 (12689.44, 1270044.6)	60.2 (5.46, 114.93)
**YLDs**			
2022	Both	55090.45 (51466.46, 58714.45)	3 (2.79, 3.22)

2023	Both	55867.29 (51534.2, 60200.37)	3.01 (2.76, 3.26)
2024	Both	56659.55 (51204.81, 62114.29)	3.01 (2.7, 3.31)
2025	Both	57458.53 (50520.18, 64396.87)	3.01 (2.63, 3.39)
2026	Both	58257.61 (49523.22, 66992)	3.01 (2.55, 3.48)
2027	Both	59055.23 (48245.56, 69864.89)	3.02 (2.45, 3.58)
2028	Both	59851.26 (46708.48, 72994.03)	3.02 (2.35, 3.7)
2029	Both	60670.51 (44942.45, 76398.57)	3.03 (2.24, 3.83)
2030	Both	61509.36 (42946.66, 80072.06)	3.04 (2.11, 3.97)
2031	Both	62364.95 (40715.51, 84014.38)	3.05 (1.98, 4.12)
2032	Both	63239.28 (38242.61, 88235.94)	3.06 (1.85, 4.28)
2033	Both	64138.19 (35521.49, 92754.9)	3.08 (1.7, 4.46)
2034	Both	65085.72 (32550.15, 97621.29)	3.1 (1.54, 4.66)
2035	Both	66080.09 (29305.75, 102854.43)	3.12 (1.38, 4.86)
2036	Both	67118.68 (25760.87, 108476.5)	3.15 (1.2, 5.09)
2037	Both	68203.12 (21885.25, 114521)	3.17 (1.01, 5.34)
2038	Both	69340.44 (17646.27, 121034.62)	3.21 (0.81, 5.6)
2039	Both	70559.66 (13007.92, 128111.4)	3.24 (0.59, 5.89)
2040	Both	71859.43 (7934.69, 135803.52)	3.28 (0.36, 6.21)
2041	Both	73237.04 (4722.12, 144168.9)	3.33 (0.22, 6.56)
2042	Both	74694.15 (2457.05, 153283.81)	3.38 (0.11, 6.94)
2043	Both	76235.83 (37.98, 163242.64)	3.44 (0, 7.37)
2044	Both	77896.84 (0, 174221.36)	3.5 (0, 7.84)
2045	Both	79683.36 (0, 186359.55)	3.58 (0, 8.36)
2022	Female	29514.62 (27583.49, 31445.75)	3.18 (3.09, 3.28)
2023	Female	29955.52 (27599.86, 32311.18)	3.18 (3.02, 3.35)
2024	Female	30406.34 (27380.6, 33432.07)	3.18 (2.93, 3.43)

2025	Female	30862.99 (26955.27, 34770.72)	3.18 (2.82, 3.53)
2026	Female	31322.3 (26350.45, 36294.15)	3.18 (2.71, 3.64)
2027	Female	31783.86 (25584.18, 37983.55)	3.18 (2.59, 3.76)
2028	Female	32248.17 (24667.91, 39828.44)	3.17 (2.46, 3.89)
2029	Female	32728.7 (23616.76, 41840.63)	3.17 (2.31, 4.03)
2030	Female	33224.25 (22429.3, 44019.19)	3.17 (2.17, 4.18)
2031	Female	33733.72 (21100.94, 46366.49)	3.17 (2.01, 4.33)
2032	Female	34258.67 (19626.2, 48891.13)	3.17 (1.85, 4.49)
2033	Female	34802.66 (17999.01, 51606.31)	3.17 (1.68, 4.66)
2034	Female	35378.93 (16214.68, 54543.19)	3.16 (1.5, 4.83)
2035	Female	35987.13 (14257.28, 57716.97)	3.16 (1.32, 5.01)
2036	Female	36626.27 (12107.68, 61144.86)	3.16 (1.13, 5.19)
2037	Female	37298.07 (9744.38, 64851.77)	3.16 (0.93, 5.38)
2038	Female	38007.53 (7143.43, 68871.63)	3.16 (0.73, 5.58)
2039	Female	38771.39 (4276.66, 73266.13)	3.15 (0.53, 5.78)
2040	Female	39589.95 (1124.39, 78074.87)	3.15 (0.32, 5.99)
2041	Female	40462.29 (0, 83341.52)	3.15 (0.1, 6.2)
2042	Female	41390.52 (0, 89123.43)	3.15 (-0.12, 6.41)
2043	Female	42379.68 (0, 95493.02)	3.14 (-0.34, 6.63)
2044	Female	43450.96 (0, 102573.06)	3.14 (-0.57, 6.86)
2045	Female	44609.56 (0, 110468.88)	3.14 (-0.81, 7.08)
2022	Male	25575.83 (23882.97, 27268.7)	2.83 (2.75, 2.9)
2023	Male	25911.77 (23934.34, 27889.19)	2.83 (2.7, 2.96)
2024	Male	26253.21 (23824.21, 28682.22)	2.83 (2.63, 3.03)
2025	Male	26595.53 (23564.91, 29626.16)	2.83 (2.56, 3.1)
2026	Male	26935.31 (23172.77, 30697.85)	2.83 (2.48, 3.19)

2027	Male	27271.36 (22661.39, 31881.34)	2.84 (2.39, 3.28)
2028	Male	27603.08 (22040.57, 33165.59)	2.84 (2.29, 3.38)
2029	Male	27941.81 (21325.68, 34557.94)	2.84 (2.19, 3.48)
2030	Male	28285.11 (20517.36, 36052.87)	2.84 (2.08, 3.6)
2031	Male	28631.23 (19614.57, 37647.88)	2.84 (1.97, 3.71)
2032	Male	28980.61 (18616.41, 39344.81)	2.84 (1.85, 3.83)
2033	Male	29335.53 (17522.48, 41148.59)	2.84 (1.72, 3.96)
2034	Male	29706.79 (16335.47, 43078.1)	2.84 (1.59, 4.09)
2035	Male	30092.97 (15048.47, 45137.46)	2.84 (1.46, 4.23)
2036	Male	30492.42 (13653.19, 47331.64)	2.85 (1.32, 4.37)
2037	Male	30905.05 (12140.87, 49669.23)	2.85 (1.18, 4.52)
2038	Male	31332.91 (10502.84, 52162.99)	2.85 (1.03, 4.67)
2039	Male	31788.26 (8731.25, 54845.27)	2.85 (0.87, 4.82)
2040	Male	32269.47 (6810.3, 57728.65)	2.85 (0.72, 4.98)
2041	Male	32774.75 (4722.12, 60827.38)	2.85 (0.56, 5.14)
2042	Male	33303.62 (2457.05, 64160.38)	2.85 (0.39, 5.3)
2043	Male	33856.15 (37.98, 67749.62)	2.85 (0.22, 5.47)
2044	Male	34445.88 (0, 71648.3)	2.85 (0.05, 5.64)
2045	Male	35073.8 (0, 75890.68)	2.85 (-0.13, 5.82)

Notes: ASR: age-standardized rates; YLDs: years lived with disability.

Table S95. Prevalence and YLDs of neoplasms in China of future forecasts using bayesian age-period-cohort model.

Year	Sex	Number	ASR
**Prevalence**			
2022	Both	33790102.44 (31307595.07, 36272609.81)	1815.72 (1682.34, 1949.09)
2023	Both	34426089.26 (31759073.91, 37093104.62)	1828.23 (1686.46, 1970.01)
2024	Both	35061320.55 (32120733.49, 38001907.61)	1840.58 (1685.86, 1995.3)
2025	Both	35689737.63 (32377127.34, 39002347.91)	1852.68 (1680.11, 2025.24)
2026	Both	36307916.42 (32522708.04, 40093124.79)	1864.6 (1669.32, 2059.88)
2027	Both	36912785.19 (32557734.47, 41267835.92)	1876.47 (1653.86, 2099.09)
2028	Both	37497548.53 (32482901.3, 42512195.75)	1888.42 (1634.3, 2142.54)
2029	Both	38068848.65 (32310263.93, 43827433.38)	1900.36 (1610.94, 2189.79)
2030	Both	38627371.78 (32044914.09, 45209829.47)	1912.26 (1584.01, 2240.5)
2031	Both	39177280.04 (31693265.23, 46661294.86)	1924.22 (1553.8, 2294.63)
2032	Both	39724101.07 (31261970.51, 48186231.64)	1936.38 (1520.56, 2352.2)
2033	Both	40255487.15 (30744110.16, 49766864.15)	1948.81 (1484.49, 2413.12)
2034	Both	40777956.22 (30146677.08, 51409235.37)	1961.42 (1445.62, 2477.23)
2035	Both	41290815.06 (29469704.55, 53111925.58)	1974.21 (1403.95, 2544.46)
2036	Both	41797224.68 (28715072.35, 54879377.01)	1987.32 (1359.57, 2615.08)
2037	Both	42301283.87 (27884854.64, 56717713.1)	2000.94 (1312.53, 2689.35)
2038	Both	42789073.8 (26970158.5, 58607989.11)	2015.1 (1262.84, 2767.36)
2039	Both	43267686.66 (25975611.23, 60559762.09)	2029.73 (1210.37, 2849.08)
2040	Both	43733401.42 (24897980.16, 62568822.68)	2044.81 (1155.02, 2934.61)
2041	Both	44187534.74 (23736549.88, 64638519.6)	2060.59 (1096.74, 3024.44)
2042	Both	44631647.37 (22490565.04, 66772729.7)	2077.21 (1035.42, 3118.99)
2043	Both	45047536.44 (21150602.41, 68944470.47)	2094.7 (970.93, 3218.47)

2044	Both	45441718.19 (19719892.5, 71163543.88)	2112.99 (903.01, 3322.97)
2045	Both	45819817.31 (18199678.05, 73439956.57)	2132.08 (831.41, 3432.75)
2022	Female	20888338.79 (19445354.39, 22331323.18)	2354.06 (2292.09, 2416.04)
2023	Female	21184261.21 (19635654.46, 22732867.97)	2368.96 (2285.44, 2452.49)
2024	Female	21473224.8 (19764440.22, 23182009.38)	2383.51 (2271.43, 2495.59)
2025	Female	21753313.89 (19825103.89, 23681523.88)	2397.64 (2251.48, 2543.8)
2026	Female	22025731.68 (19818708.88, 24232754.47)	2411.47 (2226.58, 2596.37)
2027	Female	22291831.22 (19749549.15, 24834113.28)	2425.05 (2197.29, 2652.8)
2028	Female	22542885.63 (19615027.37, 25470743.89)	2438.34 (2164.04, 2712.64)
2029	Female	22780016.58 (19420893.17, 26139139.99)	2451.28 (2127.04, 2775.53)
2030	Female	23005842.89 (19172902.69, 26838783.1)	2463.81 (2086.41, 2841.22)
2031	Female	23226506.33 (18878547.75, 27574464.91)	2476.07 (2042.38, 2909.76)
2032	Female	23448881.99 (18545004.5, 28352759.48)	2488.08 (1995.05, 2981.11)
2033	Female	23656525.51 (18161335.26, 29151715.76)	2499.79 (1944.54, 3055.04)
2034	Female	23851134.21 (17730845.8, 29971422.62)	2511.1 (1890.89, 3131.32)
2035	Female	24036472.38 (17257464.64, 30815480.12)	2521.99 (1834.14, 3209.84)
2036	Female	24220042.65 (16746815.98, 31693269.32)	2532.63 (1774.47, 3290.78)
2037	Female	24410001.54 (16204101.42, 32615901.67)	2543.04 (1711.92, 3374.16)
2038	Female	24588354.01 (15617588.69, 33559119.32)	2553.15 (1646.54, 3459.75)
2039	Female	24756458.92 (14988868.38, 34524049.47)	2562.85 (1578.37, 3547.34)
2040	Female	24916292.26 (14319217.76, 35513366.77)	2572.17 (1507.44, 3636.91)
2041	Female	25073761.4 (13611435.64, 36536087.16)	2581.38 (1433.96, 3728.8)
2042	Female	25234574.19 (12867465.88, 37601682.5)	2590.5 (1357.92, 3823.08)
2043	Female	25380865.69 (12077823.2, 38683908.17)	2599.4 (1279.34, 3919.45)
2044	Female	25513874.67 (11243501.5, 39784247.84)	2607.94 (1198.22, 4017.66)
2045	Female	25638369.21 (10366326.75, 40910411.67)	2616.16 (1114.6, 4117.71)

2022	Male	12901763.65 (11862240.68, 13941286.62)	1315.08 (1276.14, 1354.02)
2023	Male	13241828.05 (12123419.45, 14360236.64)	1326.69 (1275.49, 1377.89)
2024	Male	13588095.75 (12356293.27, 14819898.24)	1338.18 (1270.67, 1405.69)
2025	Male	13936423.74 (12552023.45, 15320824.03)	1349.51 (1262.41, 1436.6)
2026	Male	14282184.74 (12703999.16, 15860370.32)	1360.64 (1251.17, 1470.1)
2027	Male	14620953.98 (12808185.31, 16433722.64)	1371.59 (1237.25, 1505.93)
2028	Male	14954662.89 (12867873.93, 17041451.85)	1382.46 (1221, 1543.93)
2029	Male	15288832.08 (12889370.76, 17688293.39)	1393.24 (1202.54, 1583.94)
2030	Male	15621528.89 (12872011.4, 18371046.37)	1403.86 (1181.9, 1625.82)
2031	Male	15950773.71 (12814717.48, 19086829.94)	1414.31 (1159.13, 1669.48)
2032	Male	16275219.09 (12716966.01, 19833472.16)	1424.62 (1134.31, 1714.93)
2033	Male	16598961.64 (12582774.9, 20615148.38)	1434.89 (1107.57, 1762.2)
2034	Male	16926822.01 (12415831.28, 21437812.74)	1445.08 (1078.95, 1811.21)
2035	Male	17254342.68 (12212239.91, 22296445.46)	1455.12 (1048.41, 1861.83)
2036	Male	17577182.03 (11968256.37, 23186107.68)	1465.02 (1015.96, 1914.07)
2037	Male	17891282.33 (11680753.22, 24101811.43)	1474.8 (981.65, 1967.96)
2038	Male	18200719.79 (11352569.8, 25048869.78)	1484.58 (945.59, 2023.58)
2039	Male	18511227.74 (10986742.86, 26035712.62)	1494.3 (907.75, 2080.84)
2040	Male	18817109.16 (10578762.4, 27055455.92)	1503.86 (868.11, 2139.6)
2041	Male	19113773.33 (10125114.24, 28102432.43)	1513.3 (826.68, 2199.92)
2042	Male	19397073.18 (9623099.16, 29171047.2)	1522.69 (783.51, 2261.88)
2043	Male	19666670.75 (9072779.21, 30260562.3)	1532.13 (738.66, 2325.59)
2044	Male	19927843.52 (8476391, 31379296.04)	1541.53 (692.12, 2390.94)
2045	Male	20181448.1 (7833351.31, 32529544.9)	1550.79 (643.84, 2457.75)
**YLDs**			
2022	Both	1958496.04 (1749394.22, 2167597.86)	92.85 (82.89, 102.82)

2023	Both	2024969.1 (1797929.01, 2252009.2)	93.87 (83.28, 104.45)
2024	Both	2093929.02 (1841401.19, 2346456.84)	94.91 (83.39, 106.43)
2025	Both	2164332.67 (1877876.35, 2450788.99)	95.97 (83.17, 108.77)
2026	Both	2235208.91 (1905975.61, 2564442.2)	97.05 (82.64, 111.45)
2027	Both	2305857.63 (1924935.46, 2686779.8)	98.15 (81.8, 114.5)
2028	Both	2377107.72 (1935585.25, 2818630.18)	99.32 (80.71, 117.92)
2029	Both	2449935.06 (1938786.1, 2961084.01)	100.54 (79.38, 121.69)
2030	Both	2523778.26 (1934000.06, 3113556.45)	101.79 (77.79, 125.79)
2031	Both	2598256.24 (1920748.13, 3275764.34)	103.08 (75.96, 130.2)
2032	Both	2673395.42 (1898833.28, 3447957.57)	104.44 (73.91, 134.96)
2033	Both	2750468.35 (1869010.48, 3631926.22)	105.87 (71.64, 140.1)
2034	Both	2830218.19 (1831420.12, 3829016.26)	107.38 (69.15, 145.62)
2035	Both	2911618.01 (1784859.01, 4038377.01)	108.96 (66.41, 151.5)
2036	Both	2993918.61 (1728275.32, 4259561.9)	110.59 (63.42, 157.75)
2037	Both	3076867.57 (1660922.93, 4492812.2)	112.3 (60.16, 164.44)
2038	Both	3162139.9 (1583178.87, 4741100.92)	114.13 (56.64, 171.63)
2039	Both	3250671.64 (1494699.26, 5006644.01)	116.07 (52.81, 179.33)
2040	Both	3340701.47 (1393654.22, 5287748.71)	118.09 (48.65, 187.53)
2041	Both	3430931.89 (1278638.16, 5583361.53)	120.2 (44.15, 196.27)
2042	Both	3520727.9 (1148332.19, 5893418.14)	122.42 (39.25, 205.65)
2043	Both	3610550.53 (1002055.79, 6219536.23)	124.8 (33.93, 215.76)
2044	Both	3701657.72 (839169.14, 6564960.97)	127.31 (28.14, 226.64)
2045	Both	3793713.81 (658336.27, 6930514.17)	129.94 (21.86, 238.3)
2022	Female	895967.78 (816743.57, 975191.98)	83.9 (80.88, 86.92)
2023	Female	928258.86 (839923.58, 1016594.13)	85.02 (80.8, 89.24)
2024	Female	961626.47 (859743.06, 1063509.87)	86.17 (80.33, 92)

2025	Female	995616.12 (875349.49, 1115882.76)	87.31 (79.53, 95.09)
2026	Female	1029884.9 (886314.13, 1173455.67)	88.44 (78.44, 98.45)
2027	Female	1064340.38 (892603.95, 1236076.81)	89.59 (77.09, 102.08)
2028	Female	1099295.68 (894657.36, 1303933.99)	90.78 (75.54, 106.01)
2029	Female	1134942.24 (892712.64, 1377171.84)	91.99 (73.78, 110.19)
2030	Female	1170961.04 (886488.88, 1455433.21)	93.19 (71.79, 114.6)
2031	Female	1207233.01 (875780.37, 1538685.65)	94.38 (69.55, 119.21)
2032	Female	1243975.6 (860588.26, 1627362.94)	95.59 (67.1, 124.07)
2033	Female	1281702.01 (841151.46, 1722252.56)	96.83 (64.46, 129.21)
2034	Female	1320510.8 (817305.26, 1823716.35)	98.1 (61.6, 134.6)
2035	Female	1359905.9 (788433.14, 1931378.67)	99.35 (58.51, 140.2)
2036	Female	1399679.3 (754063.4, 2045295.21)	100.59 (55.18, 145.99)
2037	Female	1440018.81 (713919.13, 2166118.5)	101.82 (51.62, 152.03)
2038	Female	1481593.25 (667973.84, 2295212.65)	103.11 (47.86, 158.36)
2039	Female	1524581.13 (615796.57, 2433365.69)	104.41 (43.87, 164.94)
2040	Female	1568225.94 (556478.16, 2579973.71)	105.68 (39.64, 171.73)
2041	Female	1612146.98 (489401.52, 2735028.34)	106.93 (35.17, 178.7)
2042	Female	1656419.1 (413833.18, 2899299.55)	108.18 (30.46, 185.91)
2043	Female	1701280.23 (329211.93, 3073839.48)	109.48 (25.52, 193.44)
2044	Female	1746994.41 (234761.8, 3259934.69)	110.78 (20.35, 201.22)
2045	Female	1793196.17 (129495.15, 3457881.61)	112.05 (14.93, 209.17)
2022	Male	1062528.26 (932650.65, 1192405.88)	103.55 (98.84, 108.27)
2023	Male	1096710.25 (958005.43, 1235415.07)	104.47 (98.68, 110.25)
2024	Male	1132302.55 (981658.13, 1282946.97)	105.39 (98.15, 112.63)
2025	Male	1168716.54 (1002526.86, 1334906.23)	106.32 (97.31, 115.34)
2026	Male	1205324 (1019661.48, 1390986.53)	107.27 (96.19, 118.34)

2027	Male	1241517.25 (1032331.51, 1450702.99)	108.23 (94.85, 121.61)
2028	Male	1277812.04 (1040927.89, 1514696.19)	109.21 (93.3, 125.13)
2029	Male	1314992.82 (1046073.47, 1583912.17)	110.22 (91.56, 128.88)
2030	Male	1352817.21 (1047511.19, 1658123.24)	111.25 (89.64, 132.86)
2031	Male	1391023.23 (1044967.76, 1737078.69)	112.29 (87.54, 137.05)
2032	Male	1429419.82 (1038245.01, 1820594.62)	113.37 (85.26, 141.48)
2033	Male	1468766.34 (1027859.02, 1909673.66)	114.49 (82.83, 146.14)
2034	Male	1509707.39 (1014114.86, 2005299.92)	115.64 (80.23, 151.04)
2035	Male	1551712.11 (996425.87, 2106998.34)	116.81 (77.46, 156.16)
2036	Male	1594239.31 (974211.92, 2214266.69)	118.01 (74.51, 161.51)
2037	Male	1636848.75 (947003.81, 2326693.7)	119.24 (71.39, 167.09)
2038	Male	1680546.65 (915205.04, 2445888.26)	120.51 (68.09, 172.93)
2039	Male	1726090.51 (878902.69, 2573278.32)	121.82 (64.63, 179.01)
2040	Male	1772475.53 (837176.06, 2707774.99)	123.15 (60.97, 185.33)
2041	Male	1818784.92 (789236.64, 2848333.19)	124.5 (57.12, 191.88)
2042	Male	1864308.8 (734499.01, 2994118.59)	125.88 (53.07, 198.68)
2043	Male	1909270.3 (672843.86, 3145696.75)	127.3 (48.84, 205.77)
2044	Male	1954663.31 (604407.33, 3305026.29)	128.76 (44.41, 213.12)
2045	Male	2000517.64 (528841.12, 3472632.57)	130.23 (39.76, 220.7)

Notes: ASR: age-standardized rates; YLDs: years lived with disability.

Table S96. Prevalence and YLDs of neck pain in China of future forecasts using bayesian age-period-cohort model.

Year	Sex	Number	ASR
**Prevalence**			
2022	Both	49751584.43 (47551049.11, 51952119.75)	2535.29 (2422.83, 2647.74)
2023	Both	50217856.51 (47776459.96, 52659253.06)	2531.97 (2408.52, 2655.43)
2024	Both	50669040.51 (47841759.72, 53496321.3)	2528.63 (2387.11, 2670.14)
2025	Both	51099465.6 (47743705.55, 54455225.65)	2525.24 (2358.91, 2691.57)
2026	Both	51503295.03 (47490557.21, 55516032.85)	2521.83 (2324.75, 2718.9)
2027	Both	51876948.63 (47094936.27, 56658960.98)	2518.41 (2285.55, 2751.27)
2028	Both	52211567.97 (46564493.11, 57858642.82)	2515.18 (2242.28, 2788.07)
2029	Both	52519304.46 (45921921.55, 59116687.37)	2512.17 (2195.57, 2828.78)
2030	Both	52801223.91 (45176311.49, 60426136.34)	2509.4 (2145.78, 2873.01)
2031	Both	53057641.03 (44333538.78, 61781743.28)	2506.85 (2093.18, 2920.53)
2032	Both	53291864.32 (43400745.97, 63182982.68)	2504.54 (2037.92, 2971.15)
2033	Both	53494658.62 (42375492.96, 64613824.28)	2502.63 (1980.35, 3024.91)
2034	Both	53679918.42 (41272907.68, 66086929.15)	2501.2 (1920.63, 3081.76)
2035	Both	53846213.85 (40094250.44, 67598177.27)	2500.22 (1858.8, 3141.65)
2036	Both	53990873.93 (38839059.15, 69142688.72)	2499.7 (1794.83, 3204.58)
2037	Both	54114289.62 (37508980.14, 70719599.11)	2499.64 (1728.71, 3270.57)
2038	Both	54195781.74 (36092654.95, 72298908.52)	2500.2 (1660.57, 3339.83)
2039	Both	54255410.29 (34606695.25, 73904125.33)	2501.44 (1590.42, 3412.47)
2040	Both	54295715.58 (33054070.14, 75537361.03)	2503.36 (1518.16, 3488.57)
2041	Both	54317997.09 (31436059.53, 77199934.65)	2505.99 (1443.69, 3568.28)
2042	Both	54325833.88 (29754867.91, 78896799.86)	2509.33 (1366.9, 3651.76)
2043	Both	54296603.78 (27999572.9, 80593634.66)	2513.55 (1287.77, 3739.34)

2044	Both	54245119.62 (26180081.87, 82310157.38)	2518.69 (1206.16, 3831.23)
2045	Both	54175060.66 (24298909.95, 84051211.38)	2524.77 (1121.9, 3927.65)
2022	Female	29109114.74 (27934604.34, 30283625.13)	2925.64 (2874.68, 2976.6)
2023	Female	29406630.04 (28098304.41, 30714955.67)	2920.43 (2845.96, 2994.9)
2024	Female	29693069.55 (28170690.44, 31215448.67)	2915.14 (2810.2, 3020.07)
2025	Female	29965908.57 (28150863.76, 31780953.39)	2909.74 (2769.23, 3050.25)
2026	Female	30221588.27 (28043212.35, 32399964.19)	2904.13 (2723.87, 3084.39)
2027	Female	30458302.4 (27854847.88, 33061756.93)	2898.21 (2674.58, 3121.83)
2028	Female	30672585.25 (27591276.91, 33753893.59)	2892.22 (2622.06, 3162.38)
2029	Female	30870448.61 (27264366.39, 34476530.83)	2886.27 (2566.72, 3205.82)
2030	Female	31052382.93 (26878964.24, 35225801.62)	2880.33 (2508.76, 3251.91)
2031	Female	31217542.43 (26437384.84, 35997700.02)	2874.28 (2448.2, 3300.36)
2032	Female	31367218.51 (25943173.16, 36791263.85)	2868.02 (2385.09, 3350.95)
2033	Female	31498739.27 (25397037.98, 37600440.57)	2861.77 (2319.8, 3403.74)
2034	Female	31619792.35 (24807482.67, 38432102.02)	2855.64 (2252.56, 3458.72)
2035	Female	31727715.05 (24173830.76, 39281599.35)	2849.6 (2183.43, 3515.77)
2036	Female	31818397.74 (23494060.49, 40142734.99)	2843.53 (2112.39, 3574.68)
2037	Female	31889933.83 (22767994.76, 41011872.89)	2837.34 (2039.4, 3635.27)
2038	Female	31932289.25 (21990704.58, 41873873.92)	2831.22 (1964.75, 3697.7)
2039	Female	31957888.44 (21173034.12, 42742742.75)	2825.28 (1888.57, 3761.98)
2040	Female	31967692.94 (20316889.18, 43618496.69)	2819.46 (1810.89, 3828.03)
2041	Female	31961145.69 (19422817.36, 44499474.01)	2813.69 (1731.67, 3895.7)
2042	Female	31939238.73 (18492279.67, 45386197.79)	2807.87 (1650.9, 3964.84)
2043	Female	31890260.45 (17520162.44, 46260358.45)	2802.18 (1568.74, 4035.62)
2044	Female	31822398.94 (16513083.55, 47131714.33)	2796.68 (1485.3, 4108.07)
2045	Female	31738786.86 (15473918.83, 48003654.89)	2791.34 (1400.57, 4182.12)

2022	Male	20642469.69 (19616444.77, 21668494.62)	2143.81 (2098.88, 2188.73)
2023	Male	20811226.47 (19678155.55, 21944297.39)	2141.59 (2076.43, 2206.76)
2024	Male	20975970.95 (19671069.28, 22280872.63)	2139.24 (2047.85, 2230.62)
2025	Male	21133557.03 (19592841.79, 22674272.26)	2136.74 (2014.7, 2258.78)
2026	Male	21281706.76 (19447344.86, 23116068.67)	2134.17 (1977.85, 2290.49)
2027	Male	21418646.22 (19240088.4, 23597204.05)	2131.51 (1937.75, 2325.27)
2028	Male	21538982.71 (18973216.2, 24104749.22)	2128.76 (1894.79, 2362.73)
2029	Male	21648855.85 (18657555.17, 24640156.54)	2125.92 (1849.23, 2402.61)
2030	Male	21748840.98 (18297347.25, 25200334.71)	2123 (1801.26, 2444.74)
2031	Male	21840098.6 (17896153.93, 25784043.26)	2120.05 (1751.06, 2489.03)
2032	Male	21924645.82 (17457572.82, 26391718.82)	2117.04 (1698.71, 2535.37)
2033	Male	21995919.35 (16978454.98, 27013383.71)	2114 (1644.37, 2583.62)
2034	Male	22060126.07 (16465425.02, 27654827.12)	2110.91 (1588.15, 2633.67)
2035	Male	22118498.8 (15920419.68, 28316577.92)	2107.79 (1530.13, 2685.44)
2036	Male	22172476.19 (15344998.65, 28999953.73)	2104.68 (1470.42, 2738.93)
2037	Male	22224355.79 (14740985.37, 29707726.21)	2101.55 (1409.04, 2794.07)
2038	Male	22263492.49 (14101950.37, 30425034.61)	2098.42 (1346.07, 2850.77)
2039	Male	22297521.85 (13433661.12, 31161382.58)	2095.29 (1281.61, 2908.98)
2040	Male	22328022.65 (12737180.96, 31918864.34)	2092.17 (1215.69, 2968.66)
2041	Male	22356851.4 (12013242.16, 32700460.64)	2089.09 (1148.37, 3029.82)
2042	Male	22386595.15 (11262588.23, 33510602.07)	2086.04 (1079.66, 3092.41)
2043	Male	22406343.33 (10479410.45, 34333276.21)	2083.01 (1009.63, 3156.39)
2044	Male	22422720.69 (9666998.32, 35178443.05)	2080.02 (938.32, 3221.72)
2045	Male	22436273.8 (8824991.12, 36047556.48)	2077.07 (865.77, 3288.37)
**YLDs**			
2022	Both	4938726.49 (4717805.27, 5159647.7)	253.04 (241.67, 264.42)

2023	Both	4978321.22 (4732793.73, 5223848.71)	252.58 (240.06, 265.1)
2024	Both	5016066.78 (4731231.21, 5300902.35)	252.11 (237.73, 266.49)
2025	Both	5051568.03 (4713129.12, 5390006.94)	251.65 (234.72, 268.57)
2026	Both	5084403.06 (4679589, 5489217.11)	251.18 (231.11, 271.25)
2027	Both	5114408.45 (4632119.49, 5596697.41)	250.72 (226.99, 274.45)
2028	Both	5140570.55 (4571417.01, 5709724.09)	250.28 (222.47, 278.09)
2029	Both	5163975.93 (4499666.67, 5828285.19)	249.87 (217.61, 282.12)
2030	Both	5184819.98 (4417874.49, 5951765.46)	249.48 (212.45, 286.52)
2031	Both	5203214.7 (4326712.97, 6079716.43)	249.12 (207.01, 291.24)
2032	Both	5219603.78 (4226993.68, 6212213.89)	248.79 (201.3, 296.28)
2033	Both	5232877.41 (4118336.59, 6347418.23)	248.5 (195.37, 301.64)
2034	Both	5244341.23 (4002201.79, 6486480.67)	248.27 (189.22, 307.31)
2035	Both	5253968.64 (3878822.3, 6629114.98)	248.08 (182.87, 313.29)
2036	Both	5261601.52 (3748243.58, 6774959.46)	247.94 (176.3, 319.58)
2037	Both	5267426.59 (3610736.31, 6924116.87)	247.86 (169.52, 326.19)
2038	Both	5269168.41 (3465031.62, 7073305.2)	247.83 (162.54, 333.12)
2039	Both	5268734.49 (3312722.86, 7224746.12)	247.88 (155.36, 340.4)
2040	Both	5266499.56 (3154191.03, 7378808.08)	248 (147.97, 348.04)
2041	Both	5262702.27 (2989629.25, 7535775.28)	248.2 (140.36, 356.04)
2042	Both	5257845.82 (2819327.16, 7696364.49)	248.47 (132.51, 364.42)
2043	Both	5249500.11 (2642103.05, 7856897.18)	248.83 (124.43, 373.23)
2044	Both	5239030.93 (2458873.89, 8019187.98)	249.28 (116.09, 382.48)
2045	Both	5226939 (2269944.82, 8183933.19)	249.84 (107.48, 392.19)
2022	Female	2873069 (2754685.69, 2991452.31)	290.63 (285.4, 295.87)
2023	Female	2898093.85 (2765876.98, 3030310.73)	289.93 (282.24, 297.62)
2024	Female	2921807.05 (2767513.31, 3076100.79)	289.22 (278.37, 300.06)

2025	Female	2944046.66 (2759711.22, 3128382.1)	288.5 (273.98, 303.02)
2026	Female	2964564.17 (2743105.65, 3186022.69)	287.77 (269.15, 306.38)
2027	Female	2983302.69 (2718577.27, 3248028.11)	287 (263.92, 310.08)
2028	Female	2999855.05 (2686657.91, 3313052.19)	286.24 (258.37, 314.1)
2029	Female	3014741.52 (2648482.26, 3381000.78)	285.48 (252.54, 318.41)
2030	Female	3028050.13 (2604587.54, 3451512.72)	284.72 (246.45, 323)
2031	Female	3039753.21 (2555266.61, 3524239.8)	283.96 (240.1, 327.82)
2032	Female	3050051.75 (2500936.95, 3599166.54)	283.18 (233.5, 332.85)
2033	Female	3058580.17 (2441599.15, 3675561.19)	282.4 (226.69, 338.11)
2034	Female	3066053.61 (2378065.63, 3754041.6)	281.64 (219.68, 343.59)
2035	Female	3072264.32 (2310325.45, 3834203.19)	280.88 (212.49, 349.28)
2036	Female	3076880.61 (2238244.37, 3915516.86)	280.13 (205.11, 355.15)
2037	Female	3079809.27 (2161876.18, 3997742.35)	279.36 (197.54, 361.19)
2038	Female	3079938.38 (2080659.54, 4079217.22)	278.61 (189.81, 367.41)
2039	Female	3078441.16 (1995621.53, 4161260.8)	277.87 (181.92, 373.81)
2040	Female	3075472.67 (1906992.67, 4243952.68)	277.14 (173.9, 380.39)
2041	Female	3071056.22 (1814874.49, 4327237.95)	276.43 (165.72, 387.13)
2042	Female	3065387.36 (1719457.66, 4411317.06)	275.7 (157.4, 394)
2043	Female	3057206.2 (1620183.85, 4494228.56)	274.99 (148.94, 401.04)
2044	Female	3047254.75 (1517653.68, 4576855.81)	274.3 (140.36, 408.25)
2045	Female	3035912.91 (1412174.78, 4659651.04)	273.63 (131.66, 415.6)
2022	Male	2065657.49 (1963119.58, 2168195.4)	215.42 (210.87, 219.97)
2023	Male	2080227.37 (1966916.75, 2193537.99)	215.12 (208.52, 221.72)
2024	Male	2094259.73 (1963717.9, 2224801.55)	214.81 (205.56, 224.05)
2025	Male	2107521.37 (1953417.9, 2261624.84)	214.48 (202.15, 226.81)
2026	Male	2119838.89 (1936483.35, 2303194.43)	214.15 (198.37, 229.92)

2027	Male	2131105.76 (1913542.22, 2348669.3)	213.81 (194.28, 233.34)
2028	Male	2140715.5 (1884759.1, 2396671.9)	213.46 (189.9, 237.03)
2029	Male	2149234.41 (1851184.41, 2447284.41)	213.11 (185.26, 240.96)
2030	Male	2156769.85 (1813286.96, 2500252.74)	212.75 (180.39, 245.11)
2031	Male	2163461.5 (1771446.36, 2555476.63)	212.39 (175.3, 249.48)
2032	Male	2169552.03 (1726056.73, 2613047.34)	212.03 (169.99, 254.06)
2033	Male	2174297.24 (1676737.45, 2671857.04)	211.66 (164.5, 258.82)
2034	Male	2178287.62 (1624136.16, 2732439.07)	211.29 (158.82, 263.77)
2035	Male	2181704.32 (1568496.85, 2794911.79)	210.92 (152.96, 268.88)
2036	Male	2184720.9 (1509999.21, 2859442.6)	210.55 (146.94, 274.17)
2037	Male	2187617.32 (1448860.12, 2926374.51)	210.19 (140.75, 279.62)
2038	Male	2189230.03 (1384372.08, 2994087.98)	209.82 (134.41, 285.22)
2039	Male	2190293.33 (1317101.34, 3063485.32)	209.45 (127.93, 290.98)
2040	Male	2191026.89 (1247198.36, 3134855.41)	209.09 (121.3, 296.88)
2041	Male	2191646.05 (1174754.76, 3208537.33)	208.73 (114.54, 302.92)
2042	Male	2192458.46 (1099869.5, 3285047.43)	208.38 (107.64, 309.11)
2043	Male	2192293.91 (1021919.2, 3362668.62)	208.02 (100.61, 315.44)
2044	Male	2191776.19 (941220.21, 3442332.17)	207.67 (93.46, 321.89)
2045	Male	2191026.09 (857770.04, 3524282.15)	207.33 (86.18, 328.48)

Notes: ASR: age-standardized rates; YLDs: years lived with disability.

Table S97. Prevalence and YLDs of low back pain in China of future forecasts using bayesian age-period-cohort model.

Year	Sex	Number	ASR
**Prevalence**			
2022	Both	103945602.96 (97291419.33, 110599786.58)	5345.19 (5002.1, 5688.29)
2023	Both	105328726.19 (97859291.17, 112798161.22)	5344.48 (4964.48, 5724.47)
2024	Both	106731327.92 (97959848.54, 115502807.3)	5344.45 (4904.26, 5784.63)
2025	Both	108142363.01 (97594538.13, 118690187.88)	5345.47 (4823.25, 5867.69)
2026	Both	109540120.05 (96787055.58, 122293184.52)	5347.73 (4724.48, 5970.97)
2027	Both	110912137.15 (95571094.67, 126253179.63)	5351.38 (4610.8, 6091.95)
2028	Both	112234732.25 (93965789.54, 130503674.97)	5356.51 (4484.54, 6228.49)
2029	Both	113561191.17 (92043238.49, 135079143.84)	5363.25 (4347.31, 6379.2)
2030	Both	114880882.24 (89809928.04, 139951836.45)	5371.91 (4200.31, 6543.5)
2031	Both	116175671.82 (87260521.32, 145090822.32)	5382.84 (4044.34, 6721.33)
2032	Both	117434795.43 (84392818.1, 150476772.75)	5396.15 (3879.69, 6912.6)
2033	Both	118621392.7 (81186993.13, 156055792.27)	5411.85 (3706.5, 7117.19)
2034	Both	119790004.86 (77685323.4, 161894686.31)	5429.92 (3524.72, 7335.12)
2035	Both	120944504.57 (73888202.18, 168000806.96)	5450.62 (3334.25, 7566.98)
2036	Both	122083481.11 (69789472.88, 174377489.34)	5474.45 (3134.93, 7813.97)
2037	Both	123202011.12 (65379739.32, 181024282.92)	5501.5 (2926.13, 8076.87)
2038	Both	124297586.87 (60650381.17, 187944792.57)	5531.8 (2707.21, 8356.38)
2039	Both	125399264.05 (55605497.73, 195193030.38)	5565.24 (2477.33, 8653.15)
2040	Both	126512546.79 (50232728.18, 202792365.41)	5602.01 (2235.61, 8968.41)
2041	Both	127643054.77 (44515648.69, 210770460.85)	5642.77 (1981.09, 9304.45)
2042	Both	128787649.08 (38432332.87, 219142965.3)	5687.68 (1712.38, 9662.97)
2043	Both	129933639.57 (31955163.61, 227912115.53)	5736.82 (1427.98, 10045.65)

2044	Both	131103726.81 (25063710.74, 237143742.89)	5790.08 (1126.17, 10453.99)
2045	Both	132313553.8 (17731214.34, 246899433.4)	5847.71 (805.75, 10890.38)
2022	Female	62723103.16 (59019923.59, 66426282.72)	6338.12 (6172.18, 6504.06)
2023	Female	63607393.19 (59356337.96, 67858448.42)	6327.06 (6069.64, 6584.49)
2024	Female	64499170.56 (59366217.86, 69632123.25)	6316.23 (5941.92, 6690.55)
2025	Female	65393862.97 (59062855.04, 71724870.89)	6305.8 (5795.82, 6815.78)
2026	Female	66277256.73 (58469505.83, 74085007.63)	6295.57 (5634.44, 6956.7)
2027	Female	67141631.19 (57611678.25, 76671584.13)	6285.31 (5459.46, 7111.17)
2028	Female	67975290.06 (56505073.82, 79445506.3)	6275.1 (5272.4, 7277.79)
2029	Female	68811630.84 (55194289.78, 82428971.9)	6265.03 (5074.42, 7455.63)
2030	Female	69648334.13 (53685815.88, 85610852.37)	6255.29 (4866.43, 7644.14)
2031	Female	70476009.01 (51976448.9, 88975569.12)	6246.08 (4649.15, 7843)
2032	Female	71289370.14 (50064179.28, 92514561)	6237.16 (4422.82, 8051.5)
2033	Female	72071208.39 (47939222.08, 96203194.7)	6228.39 (4187.86, 8268.92)
2034	Female	72856318.98 (45625364.37, 100087273.59)	6219.66 (3944.65, 8494.67)
2035	Female	73643151.43 (43117535.35, 104168767.51)	6211.16 (3693.67, 8728.65)
2036	Female	74425385.68 (40406143.09, 108444628.27)	6203.42 (3435.47, 8971.36)
2037	Female	75192555.26 (37479077.13, 112906033.38)	6196.2 (3170.07, 9222.34)
2038	Female	75949144.6 (34332675.62, 117565613.58)	6189.2 (2897.59, 9480.82)
2039	Female	76720829.25 (30969964.08, 122471694.42)	6182.12 (2618.14, 9746.1)
2040	Female	77504284.26 (27376511.81, 127632056.71)	6175.09 (2332.03, 10018.16)
2041	Female	78295118.89 (23535672.78, 133054564.99)	6168.84 (2039.66, 10298.03)
2042	Female	79078705.75 (19426992.35, 138730419.15)	6163.16 (1741, 10585.33)
2043	Female	79857129.76 (15035479.1, 144678780.43)	6157.62 (1436.09, 10879.16)
2044	Female	80649492.44 (10345959.42, 150953025.45)	6151.79 (1125.03, 11178.56)
2045	Female	81466088.96 (5338453.41, 157597264.64)	6145.76 (808.04, 11483.49)

2022	Male	41222499.8 (38271495.74, 44173503.86)	4328.45 (4204.69, 4452.22)
2023	Male	41721333.01 (38502953.21, 44939712.8)	4335.8 (4161.37, 4510.24)
2024	Male	42232157.36 (38593630.69, 45870684.04)	4343.37 (4102.72, 4584.01)
2025	Male	42748500.04 (38531683.09, 46965316.99)	4351.34 (4032.66, 4670.01)
2026	Male	43262863.32 (38317549.75, 48208176.89)	4359.9 (3953.42, 4766.37)
2027	Male	43770505.96 (37959416.42, 49581595.5)	4369.01 (3866.15, 4871.87)
2028	Male	44259442.19 (37460715.72, 51058168.67)	4378.43 (3771.5, 4985.36)
2029	Male	44749560.33 (36848948.71, 52650171.94)	4388.06 (3669.95, 5106.16)
2030	Male	45232548.11 (36124112.15, 54340984.08)	4398.04 (3562, 5234.07)
2031	Male	45699662.81 (35284072.42, 56115253.2)	4408.55 (3448.04, 5369.07)
2032	Male	46145425.29 (34328638.83, 57962211.76)	4419.54 (3328.15, 5510.92)
2033	Male	46550184.31 (33247771.05, 59852597.57)	4430.7 (3202.38, 5659.02)
2034	Male	46933685.88 (32059959.03, 61807412.73)	4441.79 (3070.75, 5812.84)
2035	Male	47301353.14 (30770666.83, 63832039.45)	4452.83 (2933.41, 5972.25)
2036	Male	47658095.43 (29383329.79, 65932861.07)	4464.01 (2790.59, 6137.43)
2037	Male	48009455.87 (27900662.19, 68118249.54)	4475.25 (2642.31, 6308.2)
2038	Male	48348442.27 (26317705.55, 70379178.99)	4486.34 (2488.58, 6484.09)
2039	Male	48678434.8 (24635533.65, 72721335.96)	4496.91 (2329.38, 6664.44)
2040	Male	49008262.53 (22856216.38, 75160308.69)	4506.9 (2164.8, 6849.01)
2041	Male	49347935.88 (20979975.91, 77715895.85)	4516.53 (1995.04, 7038.01)
2042	Male	49708943.33 (19005340.52, 80412546.15)	4525.76 (1820.18, 7231.34)
2043	Male	50076509.81 (16919684.51, 83233335.1)	4534.53 (1640.35, 7428.71)
2044	Male	50454234.38 (14717751.32, 86190717.44)	4542.49 (1455.57, 7629.41)
2045	Male	50847464.84 (12392760.93, 89302168.75)	4549.57 (1265.99, 7833.14)
**YLDs**			
2022	Both	11726348.58 (10972317.66, 12480379.5)	603.01 (564.1, 641.91)

2023	Both	11868069.54 (11021684.08, 12714454.99)	602.66 (559.54, 645.77)
2024	Both	12010652.15 (11016845.28, 13004459.02)	602.39 (552.42, 652.36)
2025	Both	12153227.05 (10958809.75, 13347644.36)	602.25 (542.95, 661.55)
2026	Both	12293851.4 (10851039.21, 13736663.59)	602.26 (531.5, 673.02)
2027	Both	12431425.98 (10697800.71, 14165051.25)	602.43 (518.38, 686.49)
2028	Both	12562824.38 (10500966.93, 14624681.83)	602.79 (503.86, 701.71)
2029	Both	12693438.22 (10268287.97, 15118588.47)	603.33 (488.12, 718.54)
2030	Both	12822534.57 (10001039.4, 15644029.73)	604.09 (471.29, 736.9)
2031	Both	12948730.93 (9699262.13, 16198199.73)	605.12 (453.46, 756.78)
2032	Both	13071278.91 (9363125.62, 16779432.2)	606.43 (434.67, 778.19)
2033	Both	13185474.34 (8990058.64, 17380890.03)	608.02 (414.93, 801.11)
2034	Both	13296797.69 (8584460.9, 18009134.47)	609.88 (394.23, 825.53)
2035	Both	13406028.54 (8146740.51, 18665316.56)	612.04 (372.55, 851.52)
2036	Both	13513600.65 (7676664.12, 19350537.19)	614.56 (349.89, 879.23)
2037	Both	13619417.56 (7173477.04, 20065358.08)	617.45 (326.16, 908.74)
2038	Both	13722135.38 (6635674.42, 20808596.34)	620.71 (301.3, 940.13)
2039	Both	13824511.61 (6063585.55, 21585437.67)	624.34 (275.19, 973.48)
2040	Both	13927647.8 (5456205.65, 22399089.96)	628.33 (247.75, 1008.92)
2041	Both	14032912.82 (4812079.52, 23253746.11)	632.79 (218.85, 1046.73)
2042	Both	14140616.65 (4128991.72, 24152241.58)	637.71 (188.33, 1087.09)
2043	Both	14248409.93 (3403512.9, 25093306.96)	643.11 (156.02, 1130.2)
2044	Both	14358181.84 (2633248.03, 26083115.65)	648.98 (121.72, 1176.25)
2045	Both	14472027.46 (1816199.11, 27129083.38)	655.35 (85.44, 1225.49)
2022	Female	7016534.85 (6604438.95, 7428630.76)	710.19 (691.49, 728.9)
2023	Female	7104801.73 (6630407.77, 7579195.7)	708.44 (679.31, 737.58)
2024	Female	7192964.66 (6618256.96, 7767672.36)	706.73 (664.28, 749.17)

2025	Female	7280754.25 (6570217.25, 7991291.24)	705.05 (647.18, 762.92)
2026	Female	7366950.87 (6489612.75, 8244288.99)	703.42 (628.38, 778.45)
2027	Female	7450898 (6379693.28, 8522102.72)	701.79 (608.07, 795.5)
2028	Female	7530965.3 (6242067.53, 8819863.08)	700.17 (586.43, 813.92)
2029	Female	7610500.34 (6081510.53, 9139490.14)	698.58 (563.57, 833.59)
2030	Female	7689567.11 (5899134, 9480000.21)	697.03 (539.6, 854.45)
2031	Female	7767605.28 (5695050.83, 9840159.73)	695.54 (514.62, 876.46)
2032	Female	7844347.26 (5469320.51, 10219374.01)	694.1 (488.64, 899.55)
2033	Female	7917531.64 (5220650.2, 10614413.07)	692.68 (461.73, 923.62)
2034	Female	7990602.47 (4951448.36, 11029756.57)	691.27 (433.92, 948.61)
2035	Female	8063592.64 (4661367.79, 11465817.48)	689.88 (405.26, 974.5)
2036	Female	8136195.48 (4349634.13, 11922756.83)	688.59 (375.82, 1001.35)
2037	Female	8207516.71 (4015064.44, 12399968.98)	687.36 (345.61, 1029.1)
2038	Female	8277410.7 (3656938.95, 12897882.45)	686.15 (314.64, 1057.67)
2039	Female	8348413.97 (3275501.37, 13421326.57)	684.95 (282.91, 1086.98)
2040	Female	8420437.72 (2869355.56, 13971519.88)	683.74 (250.48, 1117.01)
2041	Female	8493459.97 (2436862.32, 14550057.62)	682.63 (217.37, 1147.88)
2042	Female	8566237.82 (1975853.91, 15156621.73)	681.57 (183.6, 1179.55)
2043	Female	8638473.48 (1484486.86, 15792460.1)	680.54 (149.17, 1211.92)
2044	Female	8711862.73 (960993.05, 16462732.41)	679.48 (114.08, 1244.87)
2045	Female	8787761.65 (404047.02, 17172703.86)	678.39 (78.37, 1278.41)
2022	Male	4709813.73 (4367878.71, 5051748.74)	493.67 (479.33, 508.01)
2023	Male	4763267.8 (4391276.31, 5135259.29)	494.48 (474.41, 514.54)
2024	Male	4817687.49 (4398588.32, 5236786.67)	495.31 (467.76, 522.85)
2025	Male	4872472.8 (4388592.49, 5356353.12)	496.19 (459.82, 532.57)
2026	Male	4926900.53 (4361426.45, 5492374.6)	497.15 (450.84, 543.46)

2027	Male	4980527.98 (4318107.43, 5642948.53)	498.17 (440.95, 555.39)
2028	Male	5031859.08 (4258899.41, 5804818.75)	499.24 (430.23, 568.24)
2029	Male	5082937.89 (4186777.44, 5979098.33)	500.33 (418.74, 581.92)
2030	Male	5132967.46 (4101905.4, 6164029.52)	501.47 (406.53, 596.4)
2031	Male	5181125.65 (4004211.3, 6358040)	502.67 (393.64, 611.69)
2032	Male	5226931.65 (3893805.11, 6560058.19)	503.92 (380.09, 627.75)
2033	Male	5267942.7 (3769408.45, 6766476.96)	505.2 (365.87, 644.53)
2034	Male	5306195.22 (3633012.54, 6979377.9)	506.48 (350.99, 661.96)
2035	Male	5342435.9 (3485372.72, 7199499.08)	507.74 (335.47, 680.02)
2036	Male	5377405.17 (3327029.99, 7427780.36)	509.03 (319.33, 698.73)
2037	Male	5411900.85 (3158412.61, 7665389.1)	510.32 (302.57, 718.07)
2038	Male	5444724.68 (2978735.47, 7910713.89)	511.6 (285.19, 738)
2039	Male	5476097.64 (2788084.18, 8164111.1)	512.81 (267.19, 758.43)
2040	Male	5507210.09 (2586850.09, 8427570.08)	513.96 (248.59, 779.34)
2041	Male	5539452.85 (2375217.2, 8703688.5)	515.07 (229.39, 800.74)
2042	Male	5574378.83 (2153137.81, 8995619.84)	516.13 (209.62, 822.63)
2043	Male	5609936.45 (1919026.03, 9300846.86)	517.14 (189.29, 844.99)
2044	Male	5646319.11 (1672254.98, 9620383.24)	518.05 (168.39, 867.71)
2045	Male	5684265.8 (1412152.09, 9956379.52)	518.86 (146.95, 890.77)

Notes: ASR: age-standardized rates; YLDs: years lived with disability.

Table S98. Prevalence and YLDs of hip osteoarthritis in China of future forecasts using bayesian age-period-cohort model.

Year	Sex	Number	ASR
**Prevalence**			
2022	Both	5683235.64 (5527437.41, 5839033.88)	261.41 (254.21, 268.61)
2023	Both	5851232.36 (5644229.88, 6058234.84)	262.51 (253.18, 271.84)
2024	Both	6024468.16 (5738960.08, 6309976.25)	263.57 (251.04, 276.11)
2025	Both	6199407.99 (5812068.66, 6586747.31)	264.6 (248.01, 281.19)
2026	Both	6374042.45 (5864140.25, 6883944.64)	265.64 (244.32, 286.96)
2027	Both	6545972.22 (5894178.87, 7197765.57)	266.7 (240.06, 293.34)
2028	Both	6719026.11 (5906597.86, 7531454.36)	267.79 (235.31, 300.27)
2029	Both	6896130.44 (5904187.65, 7888073.23)	268.88 (230.09, 307.67)
2030	Both	7073852.51 (5883769.23, 8263935.8)	269.97 (224.43, 315.51)
2031	Both	7250263.51 (5843378.04, 8657148.97)	271.1 (218.36, 323.85)
2032	Both	7423012.02 (5780720.59, 9065303.46)	272.29 (211.9, 332.68)
2033	Both	7596342.5 (5699033.73, 9493651.28)	273.55 (205.07, 342.03)
2034	Both	7773416.72 (5600165.17, 9946668.28)	274.85 (197.85, 351.85)
2035	Both	7950919.6 (5480943.39, 10420895.81)	276.19 (190.23, 362.16)
2036	Both	8126744.89 (5339182.42, 10914307.35)	277.62 (182.23, 373)
2037	Both	8298109.98 (5172386.67, 11423833.28)	279.13 (173.83, 384.43)
2038	Both	8469582.66 (4983029.07, 11956136.25)	280.76 (165.03, 396.48)
2039	Both	8644705.21 (4772241.8, 12517168.62)	282.48 (155.81, 409.15)
2040	Both	8820115.99 (4536936.83, 13103295.16)	284.29 (146.12, 422.46)
2041	Both	8993617.04 (4274865.16, 13712368.92)	286.23 (135.97, 436.49)
2042	Both	9162323.48 (3983609.61, 14341037.36)	288.3 (125.3, 451.3)
2043	Both	9330752.67 (3664285.84, 14997219.49)	290.54 (114.09, 466.99)

2044	Both	9502544.94 (3316742.84, 15688347.04)	292.93 (102.29, 483.57)
2045	Both	9674917.99 (2938064.45, 16411771.53)	295.48 (89.84, 501.12)
2022	Female	2618272.52 (2539750.03, 2696795.01)	233.65 (229.52, 237.79)
2023	Female	2695987.71 (2588434.02, 2803541.4)	234.18 (226.79, 241.58)
2024	Female	2776147.7 (2624175.91, 2928119.48)	234.65 (223.17, 246.13)
2025	Female	2857251.68 (2647940.49, 3066562.88)	235.06 (218.85, 251.26)
2026	Female	2938605.59 (2660482.29, 3216728.89)	235.45 (213.97, 256.93)
2027	Female	3019026.3 (2661354.67, 3376697.94)	235.84 (208.57, 263.11)
2028	Female	3100181.3 (2652438.45, 3547924.15)	236.2 (202.68, 269.72)
2029	Female	3183440.2 (2634928.93, 3731951.48)	236.51 (196.32, 276.7)
2030	Female	3267371.98 (2607440.26, 3927303.71)	236.75 (189.5, 284)
2031	Female	3351331.67 (2569209.39, 4133453.95)	236.98 (182.29, 291.68)
2032	Female	3434135.7 (2519034.22, 4349237.17)	237.2 (174.7, 299.7)
2033	Female	3517672.93 (2458120.63, 4577225.23)	237.4 (166.75, 308.06)
2034	Female	3603431.51 (2387080.71, 4819782.31)	237.56 (158.43, 316.7)
2035	Female	3689915.25 (2304360.12, 5075470.39)	237.67 (149.76, 325.58)
2036	Female	3776302.81 (2208927.44, 5343678.19)	237.76 (140.77, 334.75)
2037	Female	3861197.38 (2099448.37, 5622946.38)	237.83 (131.46, 344.19)
2038	Female	3946723.72 (1976661.68, 5916785.75)	237.89 (121.87, 353.91)
2039	Female	4034666.74 (1840712.34, 6228621.14)	237.92 (111.98, 363.87)
2040	Female	4123487.18 (1689969.78, 6557004.58)	237.92 (101.8, 374.03)
2041	Female	4212229.58 (1523184.82, 6901274.35)	237.89 (91.36, 384.42)
2042	Female	4299453.18 (1338975.08, 7259931.28)	237.85 (80.65, 395.04)
2043	Female	4387410.99 (1137231.57, 7637590.41)	237.8 (69.7, 405.9)
2044	Female	4478058.98 (917193.26, 8038924.7)	237.74 (58.5, 416.99)
2045	Female	4570092.6 (677044.17, 8463141.04)	237.66 (47.07, 428.25)

2022	Male	3064963.12 (2987687.38, 3142238.87)	291.43 (287.41, 295.45)
2023	Male	3155244.65 (3055795.86, 3254693.45)	293.18 (286.37, 299.99)
2024	Male	3248320.47 (3114784.17, 3381856.77)	294.88 (284.54, 305.22)
2025	Male	3342156.3 (3164128.18, 3520184.43)	296.52 (282.08, 310.96)
2026	Male	3435436.85 (3203657.95, 3667215.75)	298.14 (279.09, 317.18)
2027	Male	3526945.91 (3232824.2, 3821067.62)	299.75 (275.63, 323.87)
2028	Male	3618844.81 (3254159.41, 3983530.21)	301.35 (271.72, 330.98)
2029	Male	3712690.24 (3269258.73, 4156121.75)	302.9 (267.37, 338.44)
2030	Male	3806480.53 (3276328.97, 4336632.09)	304.39 (262.56, 346.21)
2031	Male	3898931.84 (3274168.65, 4523695.03)	305.84 (257.36, 354.33)
2032	Male	3988876.33 (3261686.37, 4716066.28)	307.29 (251.77, 362.8)
2033	Male	4078669.58 (3240913.09, 4916426.06)	308.72 (245.82, 371.63)
2034	Male	4169985.21 (3213084.46, 5126885.97)	310.11 (239.48, 380.74)
2035	Male	4261004.35 (3176583.28, 5345425.42)	311.43 (232.76, 390.11)
2036	Male	4350442.07 (3130254.98, 5570629.16)	312.73 (225.69, 399.77)
2037	Male	4436912.6 (3072938.3, 5800886.9)	314 (218.27, 409.73)
2038	Male	4522858.94 (3006367.39, 6039350.5)	315.26 (210.52, 420.01)
2039	Male	4610038.47 (2931529.47, 6288547.48)	316.49 (202.43, 430.54)
2040	Male	4696628.81 (2846967.04, 6546290.58)	317.66 (194.01, 441.3)
2041	Male	4781387.46 (2751680.34, 6811094.58)	318.8 (185.27, 452.33)
2042	Male	4862870.3 (2644634.53, 7081106.08)	319.91 (176.2, 463.63)
2043	Male	4943341.68 (2527054.27, 7359629.08)	321.03 (166.84, 475.21)
2044	Male	5024485.96 (2399549.58, 7649422.34)	322.1 (157.17, 487.04)
2045	Male	5104825.39 (2261020.28, 7948630.49)	323.14 (147.2, 499.08)
**YLDs**			
2022	Both	183464.93 (176512.65, 190417.21)	8.42 (8.09, 8.75)

2023	Both	188875.77 (180203.1, 197548.44)	8.46 (8.06, 8.86)
2024	Both	194444.78 (183130.67, 205758.88)	8.5 (7.99, 9)
2025	Both	200068.19 (185289.65, 214846.73)	8.54 (7.9, 9.18)
2026	Both	205681.13 (186696.33, 224665.93)	8.58 (7.78, 9.38)
2027	Both	211214.13 (187333.44, 235094.81)	8.61 (7.63, 9.6)
2028	Both	216775.24 (187337.88, 246212.59)	8.66 (7.47, 9.84)
2029	Both	222460.74 (186807.01, 258114.47)	8.7 (7.29, 10.1)
2030	Both	228170.32 (185650.69, 270689.94)	8.74 (7.1, 10.38)
2031	Both	233842.79 (183806.67, 283878.9)	8.78 (6.89, 10.67)
2032	Both	239413.37 (181210.11, 297616.62)	8.83 (6.67, 10.98)
2033	Both	245000.57 (177949.01, 312052.12)	8.88 (6.43, 11.32)
2034	Both	250708.33 (174081.53, 327335.13)	8.93 (6.18, 11.67)
2035	Both	256441.77 (169511.96, 343371.58)	8.98 (5.92, 12.04)
2036	Both	262136.81 (164167.94, 360105.68)	9.03 (5.64, 12.42)
2037	Both	267717.91 (157974.04, 377461.79)	9.09 (5.35, 12.84)
2038	Both	273310.38 (150986.21, 395634.55)	9.16 (5.04, 13.27)
2039	Both	279029.28 (143231.18, 414827.37)	9.22 (4.72, 13.73)
2040	Both	284779.57 (134609.2, 434949.95)	9.3 (4.38, 14.21)
2041	Both	290497.37 (125040.49, 455954.26)	9.37 (4.02, 14.73)
2042	Both	296106.08 (114443.65, 477768.51)	9.45 (3.64, 15.27)
2043	Both	301723.27 (102821.47, 500625.07)	9.54 (3.23, 15.85)
2044	Both	307465.46 (90147.33, 524783.59)	9.64 (2.81, 16.47)
2045	Both	313261.06 (76352.94, 550207.83)	9.74 (2.36, 17.12)
2022	Female	83717.06 (80361.94, 87072.18)	7.47 (7.31, 7.62)
2023	Female	86109.47 (81806.02, 90412.91)	7.48 (7.21, 7.75)
2024	Female	88569.55 (82806.75, 94332.36)	7.49 (7.08, 7.91)

2025	Female	91054.62 (83386.72, 98722.51)	7.5 (6.93, 8.08)
2026	Female	93539.97 (83569.59, 103510.36)	7.51 (6.76, 8.27)
2027	Female	95992.25 (83350.68, 108633.82)	7.52 (6.57, 8.47)
2028	Female	98461.06 (82792.31, 114129.81)	7.53 (6.36, 8.69)
2029	Female	100988.38 (81934.7, 120042.06)	7.53 (6.14, 8.93)
2030	Female	103534.74 (80741, 126328.48)	7.54 (5.9, 9.17)
2031	Female	106077.71 (79185.75, 132969.67)	7.54 (5.66, 9.43)
2032	Female	108585.32 (77235.49, 139935.14)	7.55 (5.39, 9.7)
2033	Female	111112.66 (74924.31, 147301.01)	7.55 (5.12, 9.97)
2034	Female	113704.87 (72268.88, 155140.85)	7.55 (4.84, 10.26)
2035	Female	116322.21 (69224.19, 163420.22)	7.55 (4.54, 10.56)
2036	Female	118938.55 (65754.83, 172122.27)	7.55 (4.23, 10.86)
2037	Female	121516.45 (61820.07, 181212.83)	7.55 (3.92, 11.18)
2038	Female	124116.08 (57434.04, 190798.11)	7.55 (3.59, 11.5)
2039	Female	126791.14 (52593.89, 200988.4)	7.54 (3.25, 11.83)
2040	Female	129502.48 (47247.87, 211757.09)	7.54 (2.91, 12.17)
2041	Female	132222.42 (41350.33, 223094.51)	7.53 (2.56, 12.51)
2042	Female	134912.88 (34852.66, 234973.1)	7.53 (2.19, 12.87)
2043	Female	137633.75 (27734.77, 247532.73)	7.53 (1.82, 13.23)
2044	Female	140442.82 (19957.24, 260928.39)	7.52 (1.45, 13.59)
2045	Female	143311.63 (11491.29, 275170.62)	7.51 (1.06, 13.97)
2022	Male	99747.87 (96150.7, 103345.03)	9.45 (9.29, 9.61)
2023	Male	102766.3 (98397.08, 107135.53)	9.52 (9.25, 9.78)
2024	Male	105875.22 (100323.93, 111426.52)	9.58 (9.19, 9.98)
2025	Male	109013.57 (101902.92, 116124.23)	9.65 (9.1, 10.2)
2026	Male	112141.15 (103126.74, 121155.57)	9.71 (9, 10.43)

2027	Male	115221.87 (103982.75, 126460.99)	9.78 (8.88, 10.68)
2028	Male	118314.18 (104545.57, 132082.78)	9.85 (8.75, 10.94)
2029	Male	121472.36 (104872.31, 138072.42)	9.91 (8.59, 11.22)
2030	Male	124635.57 (104909.69, 144361.46)	9.97 (8.43, 11.51)
2031	Male	127765.08 (104620.92, 150909.23)	10.03 (8.25, 11.82)
2032	Male	130828.05 (103974.62, 157681.48)	10.09 (8.05, 12.14)
2033	Male	133887.91 (103024.71, 164751.12)	10.15 (7.84, 12.47)
2034	Male	137003.46 (101812.65, 172194.28)	10.21 (7.62, 12.81)
2035	Male	140119.57 (100287.78, 179951.36)	10.27 (7.38, 13.16)
2036	Male	143198.26 (98413.11, 187983.41)	10.33 (7.13, 13.52)
2037	Male	146201.47 (96153.97, 196248.96)	10.38 (6.86, 13.9)
2038	Male	149194.3 (93552.17, 204836.44)	10.44 (6.59, 14.29)
2039	Male	152238.13 (90637.29, 213838.98)	10.49 (6.3, 14.69)
2040	Male	155277.1 (87361.33, 223192.86)	10.55 (6, 15.1)
2041	Male	158274.95 (83690.16, 232859.75)	10.6 (5.68, 15.51)
2042	Male	161193.2 (79590.99, 242795.41)	10.65 (5.35, 15.94)
2043	Male	164089.52 (75086.7, 253092.34)	10.7 (5.02, 16.39)
2044	Male	167022.64 (70190.09, 263855.2)	10.75 (4.66, 16.84)
2045	Male	169949.43 (64861.65, 275037.21)	10.8 (4.3, 17.3)

Notes: ASR: age-standardized rates; YLDs: years lived with disability.

Table S99. Prevalence and YLDs of knee osteoarthritis in China of future forecasts using bayesian age-period-cohort model.

Year	Sex	Number	ASR
**Prevalence**			
2022	Both	113129948.72 (106382215.4, 119877682.05)	5012.16 (4713.15, 5311.18)
2023	Both	115642564.66 (107871305.72, 123413823.61)	5014.9 (4677.81, 5351.99)
2024	Both	118128725.54 (108770970.59, 127486480.49)	5017.29 (4619.72, 5414.86)
2025	Both	120570730.95 (109079953.27, 132061508.64)	5019.33 (4540.8, 5497.86)
2026	Both	122950509.03 (108828150.32, 137072867.74)	5021.17 (4444.17, 5598.17)
2027	Both	125246344.93 (108041732.39, 142450957.47)	5022.95 (4332.58, 5713.32)
2028	Both	127477421.67 (106777523.17, 148177320.18)	5024.64 (4208.17, 5841.12)
2029	Both	129671337.58 (105083771.66, 154258903.51)	5026.29 (4072.41, 5980.17)
2030	Both	131811009.8 (102959793.66, 160662225.94)	5027.94 (3926.24, 6129.64)
2031	Both	133878651.01 (100398268.49, 167359033.53)	5029.69 (3770.25, 6289.14)
2032	Both	135856204.31 (97389575.46, 174322833.15)	5031.65 (3604.81, 6458.49)
2033	Both	137780066 (93966623.54, 181593508.45)	5033.83 (3430.27, 6637.38)
2034	Both	139686897.86 (90153203.14, 189220592.57)	5036.47 (3246.89, 6826.04)
2035	Both	141551005.81 (85924804.84, 197177206.78)	5039.74 (3054.64, 7024.83)
2036	Both	143340712.55 (81252899.71, 205428525.39)	5043.72 (2853.26, 7234.18)
2037	Both	145022394.32 (76110479.59, 213934309.05)	5048.5 (2642.4, 7454.61)
2038	Both	146640087.12 (70517541.8, 222762632.45)	5054.17 (2421.77, 7686.57)
2039	Both	148250875.09 (64489250.01, 232012500.18)	5061.15 (2191.04, 7931.27)
2040	Both	149828996.94 (57992840.82, 241665153.05)	5069.75 (1949.55, 8189.95)
2041	Both	151338373.46 (50991452.35, 251685294.56)	5080.04 (1696.32, 8463.77)
2042	Both	152740449.92 (43450883.63, 262030016.21)	5092.16 (1430.28, 8754.04)
2043	Both	154068395.58 (35365269.01, 272772284.27)	5106.27 (1150.44, 9062.18)

2044	Both	155383518.44 (26752398.47, 284047454.86)	5122.92 (858.29, 9390.59)
2045	Both	156677094.4 (20870041.81, 295880411.45)	5142.5 (663.45, 9741.81)
2022	Female	72437870.59 (68289674.23, 76586066.95)	6291.23 (6116.87, 6465.58)
2023	Female	74125023.86 (69383220.68, 78866827.03)	6290.33 (6042.23, 6538.43)
2024	Female	75796106.51 (70140220.94, 81451992.07)	6288.36 (5943.7, 6633.02)
2025	Female	77439070.48 (70552033.46, 84326107.5)	6285.02 (5827.02, 6743.02)
2026	Female	79042774.7 (70630983.42, 87454565.99)	6280.35 (5695.52, 6865.17)
2027	Female	80592527.26 (70388567.77, 90796486.76)	6274.22 (5551.01, 6997.44)
2028	Female	82099443.4 (69858129.43, 94340757.36)	6266.42 (5394.81, 7138.02)
2029	Female	83579775.86 (69068738.02, 98090813.7)	6256.89 (5228, 7285.78)
2030	Female	85020934.75 (68018340.51, 102023528.99)	6245.52 (5051.25, 7439.79)
2031	Female	86410798.32 (66701815.21, 106119781.42)	6232.26 (4865.12, 7599.4)
2032	Female	87735726.4 (65112133.57, 110359319.23)	6216.96 (4670.03, 7763.88)
2033	Female	89017769.97 (63271769.76, 114763770.18)	6199.43 (4466.59, 7932.27)
2034	Female	90279258.01 (61198165.02, 119360351)	6179.92 (4255.6, 8104.25)
2035	Female	91501202.87 (58876387.26, 124126018.49)	6158.5 (4037.62, 8279.38)
2036	Female	92662009.88 (56289982.73, 129034037.02)	6135.05 (3812.99, 8457.1)
2037	Female	93738462.91 (53423723.97, 134053201.86)	6109.39 (3582.01, 8636.77)
2038	Female	94757956.49 (50296105.27, 139219807.7)	6081.43 (3345.24, 8817.61)
2039	Female	95756778.86 (46924456.7, 144589101.03)	6051.63 (3103.48, 8999.78)
2040	Female	96716543.34 (43293048.98, 150140037.7)	6020.19 (2857.25, 9183.14)
2041	Female	97612522.54 (39384030.25, 155841014.83)	5986.97 (2606.84, 9367.11)
2042	Female	98418661.86 (35182125.58, 161655198.14)	5951.78 (2352.52, 9551.04)
2043	Female	99156566.44 (30694758.22, 167618374.66)	5914.58 (2094.82, 9734.35)
2044	Female	99865424.87 (25928714.76, 173802134.99)	5875.93 (1834.39, 9917.47)
2045	Female	100536669.07 (20870041.81, 180203296.32)	5836.07 (1571.68, 10100.46)

2022	Male	40692078.13 (38092541.17, 43291615.1)	3668.92 (3551.11, 3786.73)
2023	Male	41517540.81 (38488085.04, 44546996.58)	3670.84 (3496.64, 3845.03)
2024	Male	42332619.03 (38630749.65, 46034488.41)	3672.38 (3424.77, 3919.98)
2025	Male	43131660.47 (38527919.81, 47735401.14)	3673.44 (3339.85, 4007.02)
2026	Male	43907734.33 (38197166.9, 49618301.75)	3673.91 (3244.07, 4103.74)
2027	Male	44653817.66 (37653164.62, 51654470.71)	3673.65 (3138.63, 4208.67)
2028	Male	45377978.28 (36919393.74, 53836562.82)	3672.53 (3024.45, 4320.61)
2029	Male	46091561.72 (36015033.64, 56168089.81)	3670.6 (2902.33, 4438.87)
2030	Male	46790075.05 (34941453.15, 58638696.95)	3667.85 (2772.83, 4562.88)
2031	Male	47467852.7 (33696453.28, 61239252.12)	3664.18 (2636.31, 4692.06)
2032	Male	48120477.91 (32277441.89, 63963513.93)	3659.42 (2493.05, 4825.79)
2033	Male	48762296.03 (30694853.78, 66829738.27)	3653.39 (2343.42, 4963.36)
2034	Male	49407639.85 (28955038.13, 69860241.57)	3646.26 (2187.98, 5104.54)
2035	Male	50049802.94 (27048417.59, 73051188.29)	3638.13 (2027.16, 5249.1)
2036	Male	50678702.67 (24962916.97, 76394488.37)	3628.92 (1861.23, 5396.61)
2037	Male	51283931.4 (22686755.62, 79881107.19)	3618.45 (1690.4, 5546.49)
2038	Male	51882130.64 (20221436.53, 83542824.75)	3606.56 (1515.03, 5698.09)
2039	Male	52494096.23 (17564793.31, 87423399.15)	3593.5 (1335.61, 5851.4)
2040	Male	53112453.6 (14699791.84, 91525115.35)	3579.47 (1152.55, 6006.4)
2041	Male	53725850.92 (11607422.11, 95844279.73)	3564.42 (966.11, 6162.74)
2042	Male	54321788.06 (8268758.05, 100374818.07)	3548.19 (776.53, 6319.85)
2043	Male	54911829.14 (4670510.79, 105153909.61)	3530.65 (584.15, 6477.14)
2044	Male	55518093.57 (823683.71, 110245319.88)	3512.08 (389.4, 6634.75)
2045	Male	56140425.33 (0, 115677115.12)	3492.69 (192.59, 6792.8)
**YLDs**			
2022	Both	3668698.7 (3450071.16, 3887326.25)	162.3 (152.62, 171.98)

2023	Both	3745335.31 (3492028.46, 3998642.15)	162.25 (151.26, 173.24)
2024	Both	3820657.11 (3513663.79, 4127650.43)	162.19 (149.15, 175.23)
2025	Both	3894219.82 (3515394.29, 4273045.34)	162.12 (146.33, 177.9)
2026	Both	3965542.91 (3498517.97, 4432567.85)	162.04 (142.94, 181.14)
2027	Both	4034025.01 (3464093.81, 4603956.22)	161.96 (139.06, 184.86)
2028	Both	4100142.16 (3413910.6, 4786373.73)	161.87 (134.75, 188.99)
2029	Both	4164713.65 (3349517.04, 4979910.25)	161.79 (130.08, 193.49)
2030	Both	4227348.28 (3271060.49, 5183636.07)	161.71 (125.08, 198.34)
2031	Both	4287600.17 (3178424.62, 5396775.71)	161.64 (119.75, 203.52)
2032	Both	4345001.84 (3071388.03, 5618615.64)	161.57 (114.12, 209.02)
2033	Both	4400575.78 (2950891.36, 5850260.2)	161.52 (108.2, 214.84)
2034	Both	4455427.45 (2817640.57, 6093214.34)	161.49 (101.99, 221)
2035	Both	4508898.94 (2670949.25, 6346848.63)	161.49 (95.48, 227.5)
2036	Both	4560110.92 (2509954.44, 6610267.4)	161.53 (88.68, 234.37)
2037	Both	4608105.19 (2333807.24, 6882403.14)	161.59 (81.56, 241.63)
2038	Both	4654142.34 (2142976.08, 7165308.61)	161.7 (74.11, 249.28)
2039	Both	4699963.33 (1937792.48, 7462134.18)	161.86 (66.32, 257.39)
2040	Both	4744898.44 (1717182, 7772614.88)	162.08 (58.16, 266)
2041	Both	4787929.47 (1479892.66, 8095966.29)	162.38 (49.59, 275.16)
2042	Both	4827943.02 (1224735.21, 8431172.88)	162.74 (40.57, 284.92)
2043	Both	4865802.08 (952109.17, 8780460.37)	163.19 (31.15, 295.33)
2044	Both	4903371.15 (767268.96, 9148270.36)	163.74 (24.9, 306.48)
2045	Both	4940585.66 (605136.77, 9536076.02)	164.39 (19.44, 318.49)
2022	Female	2337861.52 (2204885.47, 2470837.58)	203.01 (197.36, 208.66)
2023	Female	2388714.41 (2236336.67, 2541092.16)	202.77 (194.7, 210.84)
2024	Female	2438716.93 (2256447.32, 2620986.54)	202.49 (191.26, 213.72)

2025	Female	2487548.14 (2265130.76, 2709965.52)	202.17 (187.25, 217.09)
2026	Female	2534902.06 (2262936.32, 2806867.79)	201.8 (182.76, 220.85)
2027	Female	2580379.65 (2250366.54, 2910392.75)	201.38 (177.85, 224.92)
2028	Female	2624256.39 (2228473.31, 3020039.47)	200.91 (172.56, 229.25)
2029	Female	2666994.15 (2198183.08, 3135805.23)	200.38 (166.95, 233.81)
2030	Female	2708274.39 (2159541.46, 3257007.33)	199.79 (161.02, 238.56)
2031	Female	2747772.06 (2112469.36, 3383074.76)	199.14 (154.8, 243.48)
2032	Female	2785130.66 (2056830.93, 3513430.38)	198.42 (148.31, 248.54)
2033	Female	2820970.46 (1993304.87, 3648636.06)	197.64 (141.55, 253.72)
2034	Female	2855945.77 (1922440.93, 3789450.62)	196.79 (134.57, 259.01)
2035	Female	2889538.76 (1843858.46, 3935219.05)	195.89 (127.38, 264.39)
2036	Female	2921126.19 (1757103.65, 4085148.73)	194.92 (120, 269.84)
2037	Female	2950047.04 (1661761.07, 4238333)	193.89 (112.42, 275.35)
2038	Female	2977085.81 (1558384.23, 4395787.38)	192.78 (104.68, 280.88)
2039	Female	3003330.72 (1447510.03, 4559151.4)	191.62 (96.8, 286.45)
2040	Female	3028276.92 (1328709.01, 4727844.83)	190.42 (88.79, 292.05)
2041	Female	3051201.25 (1201467.3, 4900935.19)	189.16 (80.66, 297.67)
2042	Female	3071348.21 (1065346.68, 5077349.74)	187.85 (72.43, 303.27)
2043	Female	3089277.77 (920544.39, 5258011.15)	186.48 (64.11, 308.85)
2044	Female	3106161.9 (767268.96, 5445054.84)	185.07 (55.72, 314.41)
2045	Female	3121847.73 (605136.77, 5638558.69)	183.62 (47.27, 319.97)
2022	Male	1330837.18 (1245185.69, 1416488.67)	119.7 (115.74, 123.67)
2023	Male	1356620.89 (1255691.79, 1457549.99)	119.7 (113.76, 125.65)
2024	Male	1381940.18 (1257216.47, 1506663.88)	119.69 (111.19, 128.19)
2025	Male	1406671.67 (1250263.53, 1563079.82)	119.66 (108.18, 131.14)
2026	Male	1430640.85 (1235581.65, 1625700.06)	119.61 (104.79, 134.42)

2027	Male	1453645.37 (1213727.27, 1693563.47)	119.53 (101.08, 137.98)
2028	Male	1475885.77 (1185437.29, 1766334.26)	119.42 (97.07, 141.77)
2029	Male	1497719.49 (1151333.96, 1844105.02)	119.28 (92.79, 145.78)
2030	Male	1519073.89 (1111519.03, 1926628.74)	119.12 (88.27, 149.98)
2031	Male	1539828.11 (1065955.26, 2013700.95)	118.93 (83.5, 154.35)
2032	Male	1559871.18 (1014557.1, 2105185.26)	118.7 (78.52, 158.88)
2033	Male	1579605.32 (957586.49, 2201624.15)	118.43 (73.32, 163.54)
2034	Male	1599481.68 (895199.64, 2303763.72)	118.12 (67.93, 168.32)
2035	Male	1619360.18 (827090.78, 2411629.58)	117.79 (62.37, 173.21)
2036	Male	1638984.73 (752850.79, 2525118.67)	117.42 (56.63, 178.21)
2037	Male	1658058.15 (672046.17, 2644070.14)	117.02 (50.75, 183.29)
2038	Male	1677056.54 (584591.84, 2769521.23)	116.57 (44.71, 188.42)
2039	Male	1696632.61 (490282.44, 2902982.78)	116.08 (38.54, 193.62)
2040	Male	1716621.52 (388472.99, 3044770.06)	115.57 (32.26, 198.88)
2041	Male	1736728.23 (278425.35, 3195031.11)	115.04 (25.88, 204.2)
2042	Male	1756594.8 (159388.53, 3353823.14)	114.46 (19.39, 209.54)
2043	Male	1776524.31 (31564.78, 3522449.22)	113.85 (12.81, 214.89)
2044	Male	1797209.25 (0, 3703215.51)	113.21 (6.16, 220.26)
2045	Male	1818737.93 (0, 3897517.33)	112.55 (-0.55, 225.66)

Notes: ASR: age-standardized rates; YLDs: years lived with disability.

Table S100. Prevalence and YLDs of rheumatoid arthritis in China of future forecasts using bayesian age-period-cohort model.

Year	Sex	Number	ASR
**Prevalence**			
2022	Both	4953675.65 (4826079.49, 5081271.81)	242.64 (236.34, 248.95)
2023	Both	5049285.34 (4895252.76, 5203317.92)	243.82 (236.33, 251.31)
2024	Both	5144815.42 (4949147.6, 5340483.24)	245.01 (235.64, 254.37)
2025	Both	5239343.7 (4988463.53, 5490223.87)	246.2 (234.37, 258.04)
2026	Both	5332781.51 (5014821.08, 5650741.95)	247.45 (232.65, 262.25)
2027	Both	5424615.72 (5028924.18, 5820307.26)	248.77 (230.58, 266.96)
2028	Both	5514785.62 (5031786.83, 5997784.4)	250.13 (228.18, 272.08)
2029	Both	5603963.78 (5024660.46, 6183267.09)	251.51 (225.46, 277.55)
2030	Both	5691131.71 (5006984.47, 6375278.96)	252.91 (222.46, 283.36)
2031	Both	5776281.1 (4978917.46, 6573644.73)	254.38 (219.21, 289.55)
2032	Both	5859027.25 (4940189.09, 6777865.4)	255.94 (215.74, 296.14)
2033	Both	5937947.16 (4889928.93, 6985965.39)	257.56 (212.03, 303.08)
2034	Both	6014086.17 (4829275.68, 7198896.66)	259.2 (208.06, 310.35)
2035	Both	6087436.63 (4758370.88, 7416502.38)	260.89 (203.84, 317.94)
2036	Both	6158970.84 (4677997.04, 7639944.64)	262.66 (199.4, 325.92)
2037	Both	6229171.62 (4588430.68, 7869912.56)	264.54 (194.74, 334.33)
2038	Both	6295295.9 (4487804.5, 8102787.29)	266.49 (189.84, 343.14)
2039	Both	6358286.75 (4377020.8, 8339552.7)	268.48 (184.66, 352.31)
2040	Both	6418781.25 (4256624.85, 8580937.64)	270.54 (179.22, 361.85)
2041	Both	6478373.31 (4127623.25, 8829123.36)	272.69 (173.52, 371.86)
2042	Both	6538132.08 (3990477.76, 9085786.4)	274.97 (167.57, 382.36)
2043	Both	6594505.22 (3842990.61, 9346019.82)	277.33 (161.33, 393.34)

2044	Both	6648196.52 (3685711.15, 9610681.89)	279.77 (154.77, 404.77)
2045	Both	6699720.86 (3518953.24, 9880488.48)	282.28 (147.88, 416.68)
2022	Female	3325674.81 (3234858.22, 3416491.4)	323.12 (318.86, 327.37)
2023	Female	3389555.56 (3281491.72, 3497619.39)	324.61 (317.79, 331.43)
2024	Female	3453180.46 (3317809.04, 3588551.88)	326.13 (316.02, 336.23)
2025	Female	3515898.98 (3344058.14, 3687739.83)	327.67 (313.73, 341.62)
2026	Female	3577553.75 (3361205.1, 3793902.39)	329.29 (311.03, 347.55)
2027	Female	3637836.04 (3369802.22, 3905869.87)	330.98 (307.95, 354.01)
2028	Female	3696853.86 (3370697.06, 4023010.66)	332.71 (304.5, 360.92)
2029	Female	3754985.87 (3364691.26, 4145280.48)	334.47 (300.69, 368.24)
2030	Female	3811552.14 (3351440.41, 4271663.88)	336.25 (296.54, 375.96)
2031	Female	3866484.72 (3331027.31, 4401942.12)	338.11 (292.09, 384.13)
2032	Female	3919549.76 (3303329.95, 4535769.58)	340.05 (287.36, 392.75)
2033	Female	3969883.43 (3267859.31, 4671907.54)	342.04 (282.3, 401.77)
2034	Female	4018089.24 (3225314.74, 4810863.73)	344.04 (276.93, 411.15)
2035	Female	4064171.79 (3175820.47, 4952523.12)	346.07 (271.23, 420.9)
2036	Female	4108763.81 (3119912.46, 5097615.16)	348.18 (265.27, 431.09)
2037	Female	4152257.41 (3057862.71, 5246652.1)	350.37 (259.02, 441.73)
2038	Female	4192846.3 (2988462.99, 5397229.6)	352.6 (252.46, 452.75)
2039	Female	4231082.12 (2912279.88, 5549884.37)	354.85 (245.58, 464.12)
2040	Female	4267447.75 (2829739.92, 5705155.57)	357.12 (238.38, 475.86)
2041	Female	4303023.51 (2741549.21, 5864497.81)	359.47 (230.89, 488.04)
2042	Female	4338624.55 (2648110.01, 6029139.09)	361.89 (223.11, 500.68)
2043	Female	4371823.21 (2547938.31, 6195708.11)	364.35 (215.01, 513.69)
2044	Female	4403072.25 (2441411.34, 6364733.16)	366.81 (206.56, 527.06)
2045	Female	4432836.95 (2328823.04, 6536850.86)	369.3 (197.79, 540.81)

2022	Male	1628000.84 (1591221.27, 1664780.41)	162.26 (160.39, 164.14)
2023	Male	1659729.78 (1613761.03, 1705698.53)	163.05 (159.84, 166.26)
2024	Male	1691634.96 (1631338.56, 1751931.36)	163.82 (158.94, 168.71)
2025	Male	1723444.72 (1644405.39, 1802484.05)	164.6 (157.77, 171.43)
2026	Male	1755227.77 (1653615.97, 1856839.56)	165.43 (156.41, 174.44)
2027	Male	1786779.68 (1659121.97, 1914437.39)	166.3 (154.87, 177.72)
2028	Male	1817931.76 (1661089.77, 1974773.74)	167.18 (153.14, 181.22)
2029	Male	1848977.9 (1659969.2, 2037986.61)	168.05 (151.2, 184.9)
2030	Male	1879579.57 (1655544.05, 2103615.09)	168.92 (149.07, 188.77)
2031	Male	1909796.38 (1647890.15, 2171702.62)	169.82 (146.79, 192.85)
2032	Male	1939477.48 (1636859.14, 2242095.82)	170.78 (144.38, 197.18)
2033	Male	1968063.73 (1622069.61, 2314057.85)	171.74 (141.79, 201.69)
2034	Male	1995996.94 (1603960.94, 2388032.93)	172.68 (139.01, 206.35)
2035	Male	2023264.83 (1582550.41, 2463979.26)	173.62 (136.07, 211.17)
2036	Male	2050207.03 (1558084.58, 2542329.48)	174.58 (132.97, 216.19)
2037	Male	2076914.21 (1530567.97, 2623260.45)	175.59 (129.74, 221.43)
2038	Male	2102449.6 (1499341.51, 2705557.69)	176.59 (126.34, 226.84)
2039	Male	2127204.62 (1464740.92, 2789668.33)	177.57 (122.77, 232.38)
2040	Male	2151333.5 (1426884.93, 2875782.07)	178.54 (119.02, 238.07)
2041	Male	2175349.8 (1386074.04, 2964625.55)	179.53 (115.12, 243.93)
2042	Male	2199507.53 (1342367.75, 3056647.32)	180.54 (111.07, 250)
2043	Male	2222682.01 (1295052.31, 3150311.7)	181.54 (106.86, 256.23)
2044	Male	2245124.27 (1244299.81, 3245948.73)	182.52 (102.47, 262.57)
2045	Male	2266883.91 (1190130.2, 3343637.62)	183.47 (97.92, 269.03)
**YLDs**			
2022	Both	658269.51 (639705.14, 676833.88)	32.4 (31.45, 33.35)

2023	Both	670276.86 (648012.51, 692541.21)	32.55 (31.44, 33.66)
2024	Both	682231.24 (654189.85, 710272.62)	32.7 (31.33, 34.08)
2025	Both	694016.28 (658343.18, 729689.38)	32.86 (31.14, 34.57)
2026	Both	705616.69 (660688.88, 750544.5)	33.02 (30.89, 35.15)
2027	Both	716984.18 (661344.6, 772623.76)	33.19 (30.59, 35.79)
2028	Both	728096.7 (660445.54, 795747.86)	33.36 (30.24, 36.49)
2029	Both	739041.5 (658164.59, 819918.42)	33.54 (29.85, 37.24)
2030	Both	749695.67 (654444.21, 844947.13)	33.73 (29.42, 38.03)
2031	Both	760054.35 (649304.52, 870804.17)	33.92 (28.95, 38.88)
2032	Both	770098.62 (642739.43, 897457.82)	34.12 (28.45, 39.79)
2033	Both	779623.56 (634629.07, 924618.06)	34.33 (27.92, 40.74)
2034	Both	788767.53 (625127.91, 952407.16)	34.55 (27.35, 41.74)
2035	Both	797540.41 (614265.74, 980815.09)	34.77 (26.75, 42.79)
2036	Both	806059.71 (602135.99, 1009983.43)	35 (26.11, 43.89)
2037	Both	814426.74 (588801.99, 1040051.49)	35.25 (25.45, 45.06)
2038	Both	822265.8 (574013.46, 1070518.14)	35.51 (24.74, 46.27)
2039	Both	829705.67 (557893.02, 1101518.33)	35.78 (24.01, 47.54)
2040	Both	836832.91 (540512.46, 1133153.36)	36.05 (23.23, 48.87)
2041	Both	843832.49 (521982.47, 1165682.5)	36.34 (22.42, 50.26)
2042	Both	850879.26 (502380.49, 1199378.03)	36.64 (21.56, 51.72)
2043	Both	857501.13 (481419.08, 1233583.18)	36.96 (20.67, 53.25)
2044	Both	863799.6 (459173.31, 1268425.88)	37.29 (19.73, 54.84)
2045	Both	869846.25 (435681.29, 1304011.22)	37.63 (18.75, 56.51)
2022	Female	441072.8 (428797.09, 453348.51)	43.12 (42.54, 43.7)
2023	Female	449145.03 (434408.05, 463882.01)	43.32 (42.37, 44.26)
2024	Female	457152.7 (438564.85, 475740.54)	43.52 (42.11, 44.92)

2025	Female	465013.18 (441334.26, 488692.11)	43.72 (41.77, 45.67)
2026	Female	472712.25 (442860.45, 502564.05)	43.93 (41.38, 46.48)
2027	Female	480222.09 (443229.48, 517214.69)	44.15 (40.94, 47.37)
2028	Female	487540.39 (442543.61, 532537.18)	44.38 (40.44, 48.32)
2029	Female	494713.69 (440908.84, 548518.54)	44.61 (39.9, 49.33)
2030	Female	501660.49 (438289.17, 565031.81)	44.85 (39.31, 50.39)
2031	Female	508378.81 (434701.82, 582055.8)	45.09 (38.67, 51.51)
2032	Female	514860.66 (430149.36, 599571.96)	45.35 (38, 52.7)
2033	Female	520972.71 (424555.82, 617389.61)	45.61 (37.28, 53.94)
2034	Female	526792.13 (418013.72, 635570.54)	45.87 (36.51, 55.23)
2035	Female	532326.72 (410546.9, 654106.54)	46.14 (35.71, 56.57)
2036	Female	537660.82 (402225.76, 673095.88)	46.41 (34.86, 57.97)
2037	Female	542872.76 (393103.75, 692641.78)	46.7 (33.97, 59.43)
2038	Female	547706.74 (383012.01, 712401.47)	46.99 (33.04, 60.94)
2039	Female	552236 (372026.23, 732445.76)	47.28 (32.06, 62.5)
2040	Female	556525.9 (360203.12, 752848.68)	47.58 (31.05, 64.12)
2041	Female	560709.62 (347626.35, 773792.88)	47.89 (29.99, 65.79)
2042	Female	564919.72 (334360.33, 795479.1)	48.21 (28.89, 67.53)
2043	Female	568826.67 (320206.28, 817447.06)	48.53 (27.74, 69.31)
2044	Female	572492.03 (305213.74, 839770.32)	48.85 (26.55, 71.15)
2045	Female	575979.67 (289419.73, 862539.61)	49.18 (25.31, 73.04)
2022	Male	217196.71 (210908.05, 223485.37)	21.72 (21.43, 22.01)
2023	Male	221131.83 (213604.46, 228659.2)	21.81 (21.33, 22.3)
2024	Male	225078.54 (215625, 234532.09)	21.91 (21.19, 22.63)
2025	Male	229003.1 (217008.92, 240997.27)	22.01 (21.01, 23)
2026	Male	232904.44 (217828.43, 247980.45)	22.11 (20.8, 23.41)

2027	Male	236762.09 (218115.12, 255409.06)	22.21 (20.57, 23.86)
2028	Male	240556.31 (217901.94, 263210.67)	22.32 (20.3, 24.34)
2029	Male	244327.81 (217255.75, 271399.88)	22.43 (20.02, 24.84)
2030	Male	248035.18 (216155.04, 279915.32)	22.54 (19.7, 25.37)
2031	Male	251675.54 (214602.7, 288748.37)	22.65 (19.37, 25.93)
2032	Male	255237.96 (212590.07, 297885.85)	22.77 (19.02, 26.52)
2033	Male	258650.85 (210073.25, 307228.45)	22.88 (18.64, 27.13)
2034	Male	261975.4 (207114.19, 316836.61)	23 (18.23, 27.77)
2035	Male	265213.69 (203718.84, 326708.54)	23.12 (17.81, 28.43)
2036	Male	268398.89 (199910.23, 336887.55)	23.24 (17.36, 29.11)
2037	Male	271553.98 (195698.24, 347409.71)	23.36 (16.9, 29.82)
2038	Male	274559.06 (191001.45, 358116.67)	23.48 (16.41, 30.56)
2039	Male	277469.68 (185866.78, 369072.57)	23.6 (15.89, 31.32)
2040	Male	280307.01 (180309.34, 380304.68)	23.72 (15.36, 32.09)
2041	Male	283122.87 (174356.12, 391889.62)	23.85 (14.8, 32.89)
2042	Male	285959.54 (168020.15, 403898.92)	23.97 (14.22, 33.72)
2043	Male	288674.46 (161212.8, 416136.12)	24.09 (13.62, 34.56)
2044	Male	291307.57 (153959.58, 428655.56)	24.21 (13, 35.43)
2045	Male	293866.59 (146261.56, 441471.61)	24.33 (12.36, 36.31)

Notes: ASR: age-standardized rates; YLDs: years lived with disability.

Table S101. Prevalence and YLDs of tension-type headache in China of future forecasts using bayesian age-period-cohort model.

Year	Sex	Number	ASR
**Prevalence**			
2022	Both	293928071.71 (280914450.39, 306941693.03)	18717.03 (17883.4, 19550.65)
2023	Both	296826166.85 (282245740.04, 311406593.66)	18855.42 (17922.85, 19787.99)
2024	Both	299596265.17 (282510892.66, 316681637.69)	18994.67 (17903.37, 20085.96)
2025	Both	302331853.16 (281825143.56, 322838562.75)	19134.88 (17826.86, 20442.89)
2026	Both	305105099.22 (280347768.25, 329862430.19)	19274.25 (17697.56, 20850.94)
2027	Both	308127688.33 (278363688.46, 337891688.2)	19414.03 (17522.78, 21305.29)
2028	Both	310776399.17 (275389868.29, 346162930.04)	19555.29 (17309.01, 21801.56)
2029	Both	313279214.89 (271704245.56, 354854184.21)	19698.81 (17060.7, 22336.93)
2030	Both	315676398.19 (267387832.53, 363964963.85)	19844.8 (16780.2, 22909.39)
2031	Both	317977619.32 (262478722.21, 373476516.43)	19991.69 (16467.41, 23515.98)
2032	Both	320273984.64 (257073617.4, 383474351.89)	20140.79 (16124.08, 24157.5)
2033	Both	322501012.78 (251128541.5, 393873484.06)	20293.3 (15751.99, 24834.62)
2034	Both	324701483.68 (244693662.25, 404709305.11)	20450.11 (15351.99, 25548.22)
2035	Both	326840756.21 (237748974.39, 415932538.02)	20611.49 (14923.98, 26299.01)
2036	Both	328859632.89 (230255359.45, 427463906.34)	20776.21 (14466.32, 27086.09)
2037	Both	330767201.43 (222225368.93, 439309033.93)	20945.52 (13979.05, 27911.99)
2038	Both	332551653.62 (213651930.97, 451451376.26)	21120.72 (13462.3, 28779.13)
2039	Both	334247959.31 (204565011.21, 463930907.41)	21302.74 (12915.54, 29689.95)
2040	Both	335882711.95 (194979620.22, 476785803.68)	21492.03 (12337.47, 30646.6)
2041	Both	337457326.89 (184889304.8, 490025348.97)	21687.64 (11725.65, 31649.64)
2042	Both	339051081.76 (174326741.25, 503775422.26)	21890.84 (11078.73, 32702.94)
2043	Both	340497899.36 (163192035.54, 517803763.18)	22102.93 (10395.36, 33810.49)

2044	Both	341842387.65 (151506648.48, 532178126.82)	22324.98 (9673.53, 34976.44)
2045	Both	343186352.78 (139304680.8, 547068024.76)	22557.6 (8910.51, 36204.7)
2022	Female	160162625.95 (153599685.13, 166725566.76)	20514.23 (20153.16, 20875.3)
2023	Female	161657485.25 (154308889.67, 169006080.83)	20647.96 (20108.2, 21187.72)
2024	Female	163077132.06 (154471582.68, 171682681.44)	20781.56 (20008.19, 21554.94)
2025	Female	164469867.69 (154148098.74, 174791636.64)	20914.98 (19865.54, 21964.42)
2026	Female	165873547.91 (153421088.01, 178326007.8)	21046.19 (19684.44, 22407.93)
2027	Female	167397555.37 (152438388.21, 182356722.54)	21175.88 (19468.93, 22882.84)
2028	Female	168737243.08 (150964096.34, 186510389.82)	21304.4 (19222.09, 23386.71)
2029	Female	170001023.18 (149133760.42, 190868285.95)	21432.26 (18946.21, 23918.31)
2030	Female	171207987.89 (146987773.86, 195428201.92)	21559.46 (18642.42, 24476.51)
2031	Female	172362081.1 (144544991.18, 200179171.01)	21684.22 (18309.85, 25058.58)
2032	Female	173509215.68 (141855343.99, 205163087.37)	21807.22 (17949.62, 25664.82)
2033	Female	174607770.03 (138890611.7, 210324928.37)	21928.91 (17563.02, 26294.8)
2034	Female	175681178.98 (135679662.03, 215682695.93)	22049.8 (17151.15, 26948.46)
2035	Female	176712916.89 (132214471.62, 221211362.17)	22169.92 (16714.44, 27625.4)
2036	Female	177674260.25 (128476912.07, 226871608.43)	22287.7 (16251.92, 28323.49)
2037	Female	178572062.78 (124476325.08, 232667800.48)	22403.79 (15764.22, 29043.35)
2038	Female	179379171.09 (120197279.23, 238561062.95)	22518.61 (15252.21, 29785)
2039	Female	180114992.66 (115660233.51, 244569751.81)	22632.63 (14716.6, 30548.66)
2040	Female	180798975.9 (110880586.52, 250717365.28)	22745.88 (14157.61, 31334.15)
2041	Female	181437542.38 (105861773.58, 257013311.18)	22857.08 (13574.49, 32139.67)
2042	Female	182078290.11 (100629074.28, 263527505.94)	22966.78 (12967.64, 32965.93)
2043	Female	182617028.88 (95122133.72, 270111924.04)	23075.36 (12337.62, 33813.09)
2044	Female	183073632.35 (89356731.39, 276790533.32)	23183.2 (11684.91, 34681.48)
2045	Female	183508025.52 (83362062.43, 283653988.6)	23290.32 (11009.62, 35571.03)

2022	Male	133765445.77 (127314765.26, 140216126.27)	16971.35 (16619.01, 17323.69)
2023	Male	135168681.6 (127936850.37, 142400512.83)	17114.15 (16586.94, 17641.37)
2024	Male	136519133.11 (128039309.97, 144998956.24)	17257.19 (16500.8, 18013.57)
2025	Male	137861985.47 (127677044.82, 148046926.11)	17400.2 (16372.32, 18428.07)
2026	Male	139231551.31 (126926680.24, 151536422.39)	17540.88 (16205.16, 18876.6)
2027	Male	140730132.95 (125925300.25, 155534965.66)	17679.9 (16003.1, 19356.7)
2028	Male	142039156.09 (124425771.95, 159652540.22)	17818.18 (15769.48, 19866.87)
2029	Male	143278191.7 (122570485.14, 163985898.27)	17956.32 (15506.43, 20406.21)
2030	Male	144468410.3 (120400058.67, 168536761.93)	18094.07 (15214.7, 20973.44)
2031	Male	145615538.22 (117933731.02, 173297345.42)	18229.26 (14893.08, 21565.44)
2032	Male	146764768.97 (115218273.41, 178311264.52)	18362.54 (14542.61, 22182.46)
2033	Male	147893242.75 (112237929.8, 183548555.69)	18494.91 (14164.89, 22824.94)
2034	Male	149020304.7 (109014000.22, 189026609.18)	18627.01 (13760.92, 23493.09)
2035	Male	150127839.32 (105534502.78, 194721175.86)	18758.58 (13330.84, 24186.33)
2036	Male	151185372.64 (101778447.38, 200592297.91)	18887.63 (12873.38, 24901.87)
2037	Male	152195138.65 (97749043.85, 206641233.45)	19014.68 (12389.11, 25640.24)
2038	Male	153172482.53 (93454651.74, 212890313.31)	19140.74 (11879.15, 26402.33)
2039	Male	154132966.65 (88904777.7, 219361155.6)	19266.38 (11344.13, 27188.63)
2040	Male	155083736.05 (84099033.7, 226068438.4)	19391.42 (10784.08, 27998.75)
2041	Male	156019784.51 (79027531.22, 233012037.79)	19514.09 (10198.06, 28830.12)
2042	Male	156972791.64 (73697666.97, 240247916.32)	19634.88 (9586.42, 29683.33)
2043	Male	157880870.48 (68069901.81, 247691839.14)	19754.67 (8949.92, 30559.42)
2044	Male	158768755.3 (62149917.09, 255387593.5)	19873.99 (8288.99, 31459)
2045	Male	159678327.26 (55942618.36, 263414036.16)	19992.68 (7603.59, 32381.77)
**YLDs**			
2022	Both	744567.73 (724944.55, 764190.91)	43.97 (42.79, 45.16)

2023	Both	754044.79 (727583.87, 780505.72)	44.41 (42.82, 45.99)
2024	Both	763385.57 (726658.5, 800112.65)	44.85 (42.67, 47.02)
2025	Both	772721.35 (723037.67, 822405.04)	45.3 (42.38, 48.23)
2026	Both	781906.89 (717033.93, 846779.86)	45.76 (41.95, 49.57)
2027	Both	791311.23 (709211.97, 873410.49)	46.22 (41.41, 51.03)
2028	Both	800114.34 (698997.24, 901231.45)	46.69 (40.78, 52.61)
2029	Both	808718 (686864.59, 930571.41)	47.18 (40.06, 54.3)
2030	Both	817244.37 (672980.63, 961508.12)	47.69 (39.26, 56.12)
2031	Both	825519.71 (657231.8, 993807.62)	48.2 (38.37, 58.04)
2032	Both	833887.16 (639892.07, 1027882.26)	48.73 (37.38, 60.07)
2033	Both	841673.76 (620453.19, 1062894.32)	49.27 (36.32, 62.23)
2034	Both	849226.46 (599210.74, 1099242.18)	49.84 (35.16, 64.52)
2035	Both	856681.52 (576260.38, 1137102.66)	50.43 (33.92, 66.95)
2036	Both	863875.61 (551471.32, 1176279.91)	51.04 (32.58, 69.5)
2037	Both	871127.82 (525008.81, 1217246.83)	51.67 (31.14, 72.21)
2038	Both	878042.83 (496589.75, 1259495.91)	52.33 (29.59, 75.07)
2039	Both	884815.96 (466308.77, 1303323.15)	53.02 (27.94, 78.1)
2040	Both	891646.12 (434215.07, 1349077.17)	53.75 (26.17, 81.32)
2041	Both	898441.48 (400175.94, 1396707.02)	54.5 (24.27, 84.73)
2042	Both	905573.37 (364241.36, 1446905.38)	55.29 (22.24, 88.35)
2043	Both	912780.37 (326167.54, 1499393.2)	56.12 (20.05, 92.19)
2044	Both	920149.68 (285865.02, 1554434.33)	56.99 (17.7, 96.28)
2045	Both	927928.84 (243240.25, 1612617.43)	57.92 (15.17, 100.66)
2022	Female	384671.65 (374104.21, 395239.1)	46.05 (45.31, 46.78)
2023	Female	390314.64 (375571.19, 405058.09)	46.57 (45.16, 47.98)
2024	Female	395914.71 (374971.11, 416858.32)	47.09 (44.84, 49.34)

2025	Female	401547.4 (372832.72, 430262.08)	47.63 (44.4, 50.86)
2026	Female	407115.37 (369318.12, 444912.62)	48.16 (43.82, 52.51)
2027	Female	412813.88 (364723.75, 460904.02)	48.7 (43.12, 54.27)
2028	Female	418246.25 (358777.87, 477714.63)	49.23 (42.31, 56.16)
2029	Female	423630.4 (351725.85, 495534.94)	49.78 (41.39, 58.16)
2030	Female	429033.06 (343647.84, 514418.28)	50.33 (40.36, 60.3)
2031	Female	434334.19 (334454.78, 534213.59)	50.87 (39.22, 62.53)
2032	Female	439708.31 (324272.38, 555144.24)	51.42 (37.96, 64.88)
2033	Female	444828.49 (312853.89, 576803.09)	51.97 (36.6, 67.33)
2034	Female	449886.37 (300342.93, 599429.82)	52.52 (35.14, 69.91)
2035	Female	454953.76 (286772.07, 623135.45)	53.09 (33.57, 72.6)
2036	Female	459912.5 (272038.59, 647786.41)	53.64 (31.89, 75.39)
2037	Female	464922.76 (256200.46, 673645.06)	54.2 (30.1, 78.3)
2038	Female	469804.48 (239117.96, 700491)	54.76 (28.2, 81.31)
2039	Female	474665.25 (220816.01, 728514.49)	55.32 (26.2, 84.44)
2040	Female	479620.84 (201293.83, 757947.85)	55.89 (24.09, 87.68)
2041	Female	484600.26 (180442.95, 788757.57)	56.45 (21.87, 91.03)
2042	Female	489806.97 (158245.34, 821368.59)	57.01 (19.53, 94.49)
2043	Female	495112.27 (134548.45, 855676.09)	57.58 (17.09, 98.06)
2044	Female	500565.37 (109259.37, 891871.37)	58.14 (14.54, 101.75)
2045	Female	506325.38 (82274.51, 930376.25)	58.71 (11.87, 105.56)
2022	Male	359896.07 (350840.34, 368951.81)	41.99 (41.43, 42.55)
2023	Male	363730.16 (352012.69, 375447.63)	42.34 (41.33, 43.35)
2024	Male	367470.86 (351687.4, 383254.32)	42.69 (41.11, 44.27)
2025	Male	371173.95 (350204.95, 392142.96)	43.05 (40.81, 45.29)
2026	Male	374791.52 (347715.8, 401867.24)	43.4 (40.42, 46.38)

2027	Male	378497.35 (344488.22, 412506.48)	43.75 (39.95, 47.56)
2028	Male	381868.09 (340219.37, 423516.82)	44.1 (39.4, 48.8)
2029	Male	385087.6 (335138.73, 435036.47)	44.46 (38.79, 50.13)
2030	Male	388211.32 (329332.79, 447089.84)	44.82 (38.11, 51.53)
2031	Male	391185.52 (322777.02, 459594.03)	45.17 (37.36, 52.99)
2032	Male	394178.85 (315619.69, 472738.02)	45.52 (36.54, 54.51)
2033	Male	396845.27 (307599.31, 486091.23)	45.88 (35.65, 56.1)
2034	Male	399340.08 (298867.81, 499812.35)	46.23 (34.71, 57.75)
2035	Male	401727.76 (289488.31, 513967.21)	46.59 (33.7, 59.48)
2036	Male	403963.12 (279432.73, 528493.5)	46.94 (32.62, 61.26)
2037	Male	406205.06 (268808.36, 543601.77)	47.29 (31.47, 63.1)
2038	Male	408238.35 (257471.78, 559004.92)	47.64 (30.26, 65.01)
2039	Male	410150.72 (245492.77, 574808.66)	47.98 (29, 66.97)
2040	Male	412025.28 (232921.24, 591129.32)	48.34 (27.67, 69.01)
2041	Male	413841.22 (219732.99, 607949.45)	48.68 (26.27, 71.1)
2042	Male	415766.4 (205996.02, 625536.79)	49.02 (24.81, 73.24)
2043	Male	417668.1 (191619.09, 643717.12)	49.36 (23.28, 75.44)
2044	Male	419584.31 (176605.66, 662562.96)	49.71 (21.7, 77.71)
2045	Male	421603.46 (160965.74, 682241.19)	50.05 (20.05, 80.04)

Notes: ASR: age-standardized rates; YLDs: years lived with disability.

Table S102. Prevalence and YLDs of migraine in China of future forecasts using bayesian age-period-cohort model.

Year	Sex	Number	ASR
**Prevalence**			
2022	Both	188919306.9 (184829024.63, 193009589.17)	11704.01 (11449.93, 11958.08)
2023	Both	188794816.63 (183378879.44, 194210753.81)	11705.08 (11368.58, 12041.58)
2024	Both	188581247.89 (181149358.22, 196013137.56)	11706.62 (11244.57, 12168.67)
2025	Both	188316006.3 (178360923.53, 198271089.07)	11708.29 (11088.65, 12327.92)
2026	Both	188048545.04 (175177281.3, 200919808.78)	11710.42 (10908.15, 12512.69)
2027	Both	187877039.23 (171752392.45, 204001686)	11713.86 (10707.65, 12720.07)
2028	Both	187498014.19 (167855249.55, 207140778.84)	11719.48 (10490.7, 12948.27)
2029	Both	186993683.13 (163600562.19, 210386804.07)	11726.52 (10258.29, 13194.75)
2030	Both	186410002.51 (159056817.96, 213763187.05)	11734.68 (10011.26, 13458.09)
2031	Both	185801261.26 (154288913.31, 217313609.2)	11744.4 (9750.62, 13738.18)
2032	Both	185260018.83 (149382618.42, 221137419.24)	11756.49 (9477.23, 14035.74)
2033	Both	184563248.6 (144168663.28, 224957833.91)	11771.75 (9192.16, 14351.33)
2034	Both	183760121.36 (138706209.18, 228814033.54)	11789.43 (8894.99, 14683.86)
2035	Both	182889617.14 (133039627.17, 232739607.1)	11809.31 (8585.62, 15033)
2036	Both	182000677.57 (127213753.58, 236787601.55)	11831.93 (8264.26, 15399.59)
2037	Both	181167361.04 (121282138.63, 241052583.45)	11858.08 (7931.05, 15785.1)
2038	Both	180282266.2 (115173752.3, 245390780.1)	11888.52 (7586.17, 16190.87)
2039	Both	179352943.03 (108901810.72, 249804075.33)	11922.58 (7228.74, 16616.43)
2040	Both	178402162.17 (102485748.58, 254318575.77)	11960.13 (6858.15, 17062.11)
2041	Both	177463244.31 (95946181.02, 258980307.61)	12001.71 (6474.03, 17529.4)
2042	Both	176589774.36 (89307186.27, 263872362.46)	12048.11 (6075.83, 18020.39)
2043	Both	175712859.08 (82527517.12, 268898201.03)	12100.02 (5662.95, 18537.08)

2044	Both	174833841.06 (75606946.84, 274060735.29)	12156.95 (5234.08, 19079.82)
2045	Both	173967239.05 (68548911.84, 279385566.26)	12218.83 (4788, 19649.67)
2022	Female	117860936.88 (115205859.23, 120516014.52)	14816.19 (14626.72, 15005.65)
2023	Female	117791669.98 (114282320.7, 121301019.26)	14815.24 (14471.09, 15159.4)
2024	Female	117666726.98 (112855559.38, 122477894.57)	14814.65 (14276.48, 15352.83)
2025	Female	117507236.95 (111064991.08, 123949482.81)	14813.83 (14051.51, 15576.14)
2026	Female	117340446.28 (109012506.67, 125668385.88)	14812.43 (13800.12, 15824.73)
2027	Female	117223842.62 (106792725.61, 127654959.63)	14810.49 (13524.72, 16096.26)
2028	Female	116977869.94 (104272376.23, 129683363.65)	14809.43 (13228.81, 16390.05)
2029	Female	116653867.74 (101523733.51, 131784001.98)	14808.69 (12913.5, 16703.87)
2030	Female	116278942.35 (98588669.8, 133969214.9)	14807.67 (12579.55, 17035.8)
2031	Female	115883603.39 (95505261.61, 136261945.18)	14806.1 (12227.62, 17384.57)
2032	Female	115521686.03 (92324161.19, 138719210.87)	14804.02 (11858.36, 17749.68)
2033	Female	115067230.62 (88951962.74, 141182498.5)	14802.74 (11473.7, 18131.79)
2034	Female	114548060.86 (85423145.01, 143672976.72)	14801.71 (11073.92, 18529.51)
2035	Female	113987623.6 (81764624.1, 146210623.11)	14800.35 (10659.19, 18941.51)
2036	Female	113413740.02 (78002212.24, 148825267.79)	14798.42 (10229.83, 19367.02)
2037	Female	112869860.61 (74167125.2, 151572596.02)	14796.01 (9786.15, 19805.87)
2038	Female	112299469.37 (70223063.32, 154375875.42)	14794.28 (9329.46, 20259.1)
2039	Female	111704996.34 (66176256.46, 157233736.22)	14792.7 (8859.83, 20725.57)
2040	Female	111100113.21 (62037886.95, 160162339.48)	14790.76 (8377.35, 21204.18)
2041	Female	110502793.63 (57818439.58, 163187147.67)	14788.28 (7882.2, 21694.37)
2042	Female	109944461.12 (53530999.84, 166357922.4)	14785.36 (7374.59, 22196.12)
2043	Female	109386940.94 (49152848.85, 169621033.03)	14782.97 (6855.38, 22710.55)
2044	Female	108831397.64 (44683312.41, 172979482.86)	14780.64 (6324.63, 23236.66)
2045	Female	108286740.73 (40123472.05, 176450009.42)	14777.92 (5782.39, 23773.46)

2022	Male	71058370.02 (69623165.39, 72493574.66)	8737.52 (8636.13, 8838.91)
2023	Male	71003146.64 (69096558.74, 72909734.55)	8743.88 (8559.47, 8928.29)
2024	Male	70914520.92 (68293798.84, 73535242.99)	8749.92 (8461.52, 9038.31)
2025	Male	70808769.35 (67295932.45, 74321606.26)	8755.46 (8346.91, 9164.01)
2026	Male	70708098.76 (66164774.63, 75251422.9)	8761.06 (8218.33, 9303.79)
2027	Male	70653196.61 (64959666.84, 76346726.38)	8767.25 (8077.45, 9457.05)
2028	Male	70520144.25 (63582873.32, 77457415.19)	8773.94 (7925.35, 9622.54)
2029	Male	70339815.39 (62076828.68, 78602802.09)	8780.15 (7762, 9798.29)
2030	Male	70131060.15 (60468148.16, 79793972.15)	8785.7 (7587.96, 9983.44)
2031	Male	69917657.86 (58783651.71, 81051664.02)	8791.25 (7404.26, 10178.23)
2032	Male	69738332.8 (57058457.23, 82418208.37)	8797.31 (7211.55, 10383.07)
2033	Male	69496017.98 (55216700.54, 83775335.41)	8803.71 (7010.14, 10597.28)
2034	Male	69212060.49 (53283064.17, 85141056.82)	8809.49 (6799.67, 10819.31)
2035	Male	68901993.53 (51275003.07, 86528983.99)	8814.49 (6580.38, 11048.6)
2036	Male	68586937.55 (49211541.34, 87962333.76)	8819.41 (6353.04, 11285.77)
2037	Male	68297500.43 (47115013.43, 89479987.43)	8824.73 (6118.07, 11531.39)
2038	Male	67982796.83 (44950688.98, 91014904.68)	8830.21 (5875.55, 11784.88)
2039	Male	67647946.68 (42725554.26, 92570339.11)	8834.98 (5625.15, 12044.81)
2040	Male	67302048.96 (40447861.63, 94156236.3)	8838.93 (5367.06, 12310.79)
2041	Male	66960450.69 (38127741.44, 95793159.93)	8842.72 (5101.86, 12583.57)
2042	Male	66645313.24 (35776186.43, 97514440.05)	8846.81 (4829.83, 12863.8)
2043	Male	66325918.14 (33374668.27, 99277168)	8850.92 (4550.98, 13150.87)
2044	Male	66002443.42 (30923634.43, 101081252.42)	8854.29 (4265.11, 13443.48)
2045	Male	65680498.32 (28425439.79, 102935556.84)	8856.85 (3972.39, 13741.32)
**YLDs**			
2022	Both	7172567.8 (7030242.05, 7314893.55)	340.79 (336.95, 344.63)

2023	Both	7172070.27 (6978813.35, 7365327.19)	341.21 (334.15, 348.27)
2024	Both	7168225.48 (6899018.56, 7437432.4)	341.61 (330.55, 352.68)
2025	Both	7162397.51 (6799088.84, 7525706.18)	342 (326.31, 357.69)
2026	Both	7156164.64 (6684594.77, 7627734.51)	342.4 (321.55, 363.25)
2027	Both	7153415.06 (6561260.81, 7745569.32)	342.85 (316.33, 369.36)
2028	Both	7142794.33 (6420327.18, 7865261.48)	343.31 (310.68, 375.95)
2029	Both	7127279.21 (6265942.32, 7988616.09)	343.76 (304.58, 382.93)
2030	Both	7108587.04 (6100633.6, 8116540.47)	344.18 (298.07, 390.29)
2031	Both	7088567.27 (5926647.45, 8250487.1)	344.62 (291.2, 398.04)
2032	Both	7071017.27 (5747451.69, 8394582.86)	345.09 (283.98, 406.21)
2033	Both	7046784.87 (5556026.93, 8537542.81)	345.59 (276.42, 414.76)
2034	Both	7017845.21 (5354762.08, 8680928.34)	346.06 (268.5, 423.62)
2035	Both	6985834.19 (5145511.13, 8826157.24)	346.5 (260.23, 432.77)
2036	Both	6952619.69 (4930026.96, 8975212.43)	346.95 (251.65, 442.25)
2037	Both	6921400.01 (4710656.12, 9132143.9)	347.44 (242.77, 452.11)
2038	Both	6887889.04 (4484545.93, 9291232.15)	347.94 (233.59, 462.29)
2039	Both	6852114.15 (4252087.16, 9452141.14)	348.41 (224.09, 472.73)
2040	Both	6815117.33 (4014204.02, 9616030.65)	348.85 (214.27, 483.43)
2041	Both	6778257.95 (3771800.89, 9784715.02)	349.3 (204.17, 494.42)
2042	Both	6743958.93 (3526066.69, 9961851.17)	349.77 (193.8, 505.75)
2043	Both	6709696.04 (3275464.29, 10143927.8)	350.26 (183.15, 517.37)
2044	Both	6675115.64 (3019823.7, 10330407.58)	350.71 (172.2, 529.23)
2045	Both	6640895.74 (2759403.1, 10522388.39)	351.14 (160.96, 541.31)
2022	Female	4375998.95 (4287231.23, 4464766.67)	549.62 (543.03, 556.22)
2023	Female	4375866.37 (4255076.21, 4496656.53)	549.94 (537.75, 562.13)
2024	Female	4373695.85 (4205125.73, 4542265.97)	550.27 (531.11, 569.42)

2025	Female	4370227.79 (4142467.26, 4597988.31)	550.57 (523.39, 577.76)
2026	Female	4366190.07 (4070367.27, 4662012.87)	550.82 (514.68, 586.97)
2027	Female	4363889.63 (3992315.19, 4735464.07)	551.05 (505.09, 597)
2028	Female	4356723.1 (3903313.46, 4810132.74)	551.3 (494.76, 607.84)
2029	Female	4346560.4 (3805960.38, 4887160.43)	551.56 (483.72, 619.39)
2030	Female	4334377.11 (3701750.56, 4967003.65)	551.78 (471.98, 631.59)
2031	Female	4321060.24 (3591868.57, 5050251.9)	551.96 (459.56, 644.36)
2032	Female	4308828.54 (3478372.84, 5139284.24)	552.11 (446.5, 657.72)
2033	Female	4292699.91 (3357525.76, 5227874.07)	552.29 (432.87, 671.7)
2034	Female	4273771.64 (3230692.96, 5316850.32)	552.46 (418.68, 686.23)
2035	Female	4252978.66 (3098955.9, 5407001.43)	552.6 (403.93, 701.27)
2036	Female	4231229.13 (2963216.11, 5499242.15)	552.69 (388.62, 716.75)
2037	Female	4210401.6 (2824860.72, 5595942.48)	552.75 (372.78, 732.72)
2038	Female	4188393.13 (2682540.68, 5694245.57)	552.83 (356.46, 749.21)
2039	Female	4165143.09 (2536429.25, 5793856.92)	552.9 (339.65, 766.16)
2040	Female	4141237.42 (2387042.22, 5895432.62)	552.94 (322.36, 783.52)
2041	Female	4117272.02 (2234778.75, 5999765.29)	552.93 (304.6, 801.27)
2042	Female	4094681.38 (2080330.37, 6109032.4)	552.9 (286.37, 819.43)
2043	Female	4072139.26 (1922904.66, 6221373.87)	552.88 (267.72, 838.04)
2044	Female	4049477.47 (1762415.17, 6336539.76)	552.84 (248.64, 857.04)
2045	Female	4027097.11 (1598992.24, 6455201.97)	552.77 (229.14, 876.41)
2022	Male	2796568.85 (2743010.82, 2850126.87)	442.55 (433.71, 451.38)
2023	Male	2796203.9 (2723737.14, 2868670.66)	442.86 (430.87, 454.84)
2024	Male	2794529.64 (2693892.83, 2895166.44)	443.18 (426.49, 459.87)
2025	Male	2792169.73 (2656621.58, 2927717.87)	443.5 (420.96, 466.05)
2026	Male	2789974.57 (2614227.5, 2965721.64)	443.84 (414.54, 473.13)

2027	Male	2789525.43 (2568945.61, 3010105.25)	444.22 (407.39, 481.05)
2028	Male	2786071.23 (2517013.72, 3055128.74)	444.69 (399.65, 489.73)
2029	Male	2780718.8 (2459981.94, 3101455.66)	445.2 (391.32, 499.08)
2030	Male	2774209.93 (2398883.03, 3149536.82)	445.74 (382.45, 509.04)
2031	Male	2767507.04 (2334778.87, 3200235.2)	446.33 (373.06, 519.61)
2032	Male	2762188.73 (2269078.84, 3255298.62)	447.02 (363.2, 530.84)
2033	Male	2754084.95 (2198501.17, 3309668.74)	447.82 (352.91, 542.73)
2034	Male	2744073.56 (2124069.11, 3364078.02)	448.7 (342.15, 555.24)
2035	Male	2732855.52 (2046555.23, 3419155.81)	449.65 (330.93, 568.36)
2036	Male	2721390.56 (1966810.85, 3475970.28)	450.68 (319.26, 582.1)
2037	Male	2710998.41 (1885795.4, 3536201.42)	451.86 (307.16, 596.56)
2038	Male	2699495.91 (1802005.25, 3596986.58)	453.19 (294.62, 611.76)
2039	Male	2686971.06 (1715657.9, 3658284.22)	454.64 (281.6, 627.67)
2040	Male	2673879.91 (1627161.8, 3720598.03)	456.2 (268.1, 644.31)
2041	Male	2660985.94 (1537022.14, 3784949.73)	457.9 (254.09, 661.72)
2042	Male	2649277.54 (1445736.32, 3852818.77)	459.79 (239.58, 680)
2043	Male	2637556.78 (1352559.63, 3922553.93)	461.87 (224.52, 699.21)
2044	Male	2625638.17 (1257408.52, 3993867.81)	464.12 (208.89, 719.35)
2045	Male	2613798.64 (1160410.86, 4067186.42)	466.54 (192.62, 740.45)

Notes: ASR: age-standardized rates; YLDs: years lived with disability.

Table S103. Prevalence and YLDs of stroke in China of future forecasts using bayesian age-period-cohort model.

Year	Sex	Number	ASR
**Prevalence**			
2022	Both	27376706.68 (25719228.16, 29034185.2)	1308.63 (1229.44, 1387.81)
2023	Both	28393050.18 (26547748.35, 30238352)	1322.3 (1236.38, 1408.21)
2024	Both	29455068.2 (27328780.96, 31581355.43)	1335.72 (1239.29, 1432.16)
2025	Both	30551415.27 (28041787.44, 33061043.1)	1349.02 (1238.15, 1459.9)
2026	Both	31670943.19 (28672951.66, 34668934.72)	1362.33 (1233.25, 1491.4)
2027	Both	32797563.54 (29207575.11, 36387551.97)	1375.56 (1224.81, 1526.3)
2028	Both	33949802.68 (29663985.77, 38235619.59)	1388.66 (1213.11, 1564.21)
2029	Both	35147263.78 (30059076.09, 40235451.48)	1401.63 (1198.4, 1604.87)
2030	Both	36371937.1 (30375119.78, 42368754.41)	1414.57 (1180.94, 1648.2)
2031	Both	37608249.05 (30596170.19, 44620327.92)	1427.61 (1160.95, 1694.28)
2032	Both	38835993.63 (30703249.33, 46968737.93)	1440.68 (1138.41, 1742.95)
2033	Both	40075887.71 (30711372.12, 49440403.29)	1453.75 (1113.39, 1794.11)
2034	Both	41352690.3 (30635699.63, 52069680.97)	1466.83 (1085.93, 1847.72)
2035	Both	42649325.6 (30457856.61, 54840794.58)	1479.99 (1056.09, 1903.89)
2036	Both	43950346.47 (30161203.35, 57739489.58)	1493.38 (1023.92, 1962.85)
2037	Both	45233406.37 (29725932.29, 60740880.45)	1506.95 (989.3, 2024.59)
2038	Both	46521166.67 (29164216.58, 63878116.76)	1520.64 (952.17, 2089.11)
2039	Both	47841911.55 (28488088.55, 67195734.55)	1534.5 (912.51, 2156.49)
2040	Both	49176738.75 (27678352.59, 70675124.91)	1548.64 (870.29, 2226.99)
2041	Both	50508365.61 (26717720.02, 74299011.21)	1563.23 (825.45, 2301.01)
2042	Both	51811237.47 (25586728.12, 78035746.83)	1578.25 (777.82, 2378.69)
2043	Both	53107158.91 (24292700.18, 81921617.63)	1593.68 (727.24, 2460.11)

2044	Both	54423824.49 (22841204.86, 86006444.11)	1609.53 (673.58, 2545.48)
2045	Both	55737811.15 (21212358.47, 90263263.83)	1625.93 (616.67, 2635.19)
2022	Female	13089158.06 (12384820.75, 13793495.38)	1226.74 (1198.96, 1254.53)
2023	Female	13677406.88 (12868012.39, 14486801.38)	1247.2 (1206.36, 1288.05)
2024	Female	14294091.18 (13325263.46, 15262918.91)	1267.42 (1209.09, 1325.75)
2025	Female	14934132.77 (13747294.67, 16120970.87)	1287.46 (1208.14, 1366.78)
2026	Female	15594132.8 (14129822.89, 17058442.72)	1307.55 (1204.23, 1410.86)
2027	Female	16265929.43 (14465381.59, 18066477.27)	1327.52 (1197.44, 1457.6)
2028	Female	16957175.15 (14761172.55, 19153177.75)	1347.26 (1187.85, 1506.68)
2029	Female	17677537.29 (15024559.16, 20330515.42)	1366.72 (1175.5, 1557.95)
2030	Female	18420299.12 (15247454.81, 21593143.44)	1385.99 (1160.52, 1611.45)
2031	Female	19181596.5 (15423690.95, 22939502.04)	1405.32 (1143.14, 1667.5)
2032	Female	19951701.15 (15542341.93, 24361060.37)	1424.59 (1123.24, 1725.94)
2033	Female	20740731.78 (15608833.85, 25872629.72)	1443.65 (1100.73, 1786.56)
2034	Female	21560188.65 (15628009.79, 27492367.51)	1462.42 (1075.59, 1849.24)
2035	Female	22400443.61 (15588057.98, 29212829.24)	1481.01 (1047.92, 1914.09)
2036	Female	23255188.74 (15479770.13, 31030607.36)	1499.72 (1017.92, 1981.52)
2037	Female	24110688.22 (15289540.2, 32931836.25)	1518.44 (985.46, 2051.43)
2038	Female	24976910.3 (15020363.55, 34933457.04)	1536.99 (950.45, 2123.54)
2039	Female	25868753.79 (14676298.23, 37061209.36)	1555.3 (912.86, 2197.73)
2040	Female	26776387.18 (14245522.02, 39307252.33)	1573.46 (872.77, 2274.15)
2041	Female	27693716.19 (13718617.97, 41668814.41)	1591.81 (830.33, 2353.29)
2042	Female	28606010.81 (13082493.92, 44129527.7)	1610.25 (785.44, 2435.06)
2043	Female	29520472.43 (12336083.16, 46704861.71)	1628.57 (738, 2519.13)
2044	Female	30452308.35 (11479675.41, 49424941.29)	1646.69 (688, 2605.39)
2045	Female	31393851.5 (10502566.31, 52285136.69)	1664.74 (635.48, 2694)

2022	Male	14287548.62 (13334407.41, 15240689.82)	1397.49 (1359.96, 1435.01)
2023	Male	14715643.29 (13679735.96, 15751550.62)	1404.29 (1355.33, 1453.25)
2024	Male	15160977.01 (14003517.5, 16318436.52)	1410.76 (1346.44, 1475.08)
2025	Male	15617282.5 (14294492.77, 16940072.23)	1416.91 (1334.13, 1499.7)
2026	Male	16076810.38 (14543128.77, 17610492)	1422.68 (1318.87, 1526.48)
2027	Male	16531634.11 (14742193.52, 18321074.71)	1427.99 (1300.93, 1555.04)
2028	Male	16992627.53 (14902813.23, 19082441.84)	1432.89 (1280.64, 1585.14)
2029	Male	17469726.49 (15034516.93, 19904936.06)	1437.41 (1258.19, 1616.62)
2030	Male	17951637.97 (15127664.97, 20775610.97)	1441.53 (1233.71, 1649.35)
2031	Male	18426652.56 (15172479.24, 21680825.87)	1445.17 (1207.21, 1683.13)
2032	Male	18884292.48 (15160907.39, 22607677.56)	1448.25 (1178.72, 1717.79)
2033	Male	19335155.92 (15102538.27, 23567773.57)	1450.84 (1148.39, 1753.29)
2034	Male	19792501.65 (15007689.85, 24577313.46)	1452.97 (1116.36, 1789.58)
2035	Male	20248881.99 (14869798.63, 25627965.34)	1454.64 (1082.69, 1826.58)
2036	Male	20695157.73 (14681433.23, 26708882.22)	1455.75 (1047.37, 1864.12)
2037	Male	21122718.15 (14436392.1, 27809044.2)	1456.23 (1010.42, 1902.04)
2038	Male	21544256.37 (14143853.03, 28944659.72)	1456.21 (972.02, 1940.4)
2039	Male	21973157.76 (13811790.32, 30134525.2)	1455.73 (932.28, 1979.19)
2040	Male	22400351.57 (13432830.57, 31367872.58)	1454.81 (891.27, 2018.34)
2041	Male	22814649.43 (12999102.05, 32630196.8)	1453.34 (848.99, 2057.69)
2042	Male	23205226.66 (12504234.19, 33906219.14)	1451.27 (805.46, 2097.08)
2043	Male	23586686.48 (11956617.03, 35216755.92)	1448.75 (760.87, 2136.63)
2044	Male	23971516.14 (11361529.45, 36581502.83)	1445.85 (715.32, 2176.37)
2045	Male	24343959.65 (10709792.16, 37978127.15)	1442.57 (668.88, 2216.26)
**YLDs**			
2022	Both	5317458.66 (5040741.01, 5594176.3)	255.15 (241.82, 268.47)

2023	Both	5517432.61 (5204813.21, 5830052.01)	257.77 (243.12, 272.43)
2024	Both	5726921.98 (5359740.69, 6094103.27)	260.34 (243.6, 277.09)
2025	Both	5943543.5 (5501676.39, 6385410.6)	262.89 (243.28, 282.49)
2026	Both	6165054.28 (5628205.79, 6701902.78)	265.43 (242.25, 288.62)
2027	Both	6388105.94 (5736486.25, 7039725.64)	267.97 (240.55, 295.39)
2028	Both	6616538.59 (5830449.73, 7402627.45)	270.47 (238.24, 302.71)
2029	Both	6854420.72 (5913649.5, 7795191.94)	272.95 (235.37, 310.53)
2030	Both	7098008.44 (5982352.65, 8213664.23)	275.42 (232, 318.84)
2031	Both	7344128.97 (6033241.89, 8655016.06)	277.91 (228.15, 327.67)
2032	Both	7588534.55 (6062263.38, 9114805.72)	280.4 (223.82, 336.97)
2033	Both	7835420.4 (6072478.82, 9598361.97)	282.88 (219.04, 346.73)
2034	Both	8089881.29 (6067070.8, 10112691.78)	285.37 (213.79, 356.95)
2035	Both	8348421.5 (6042275.66, 10654567.34)	287.86 (208.09, 367.63)
2036	Both	8607937.35 (5994727.21, 11221147.5)	290.4 (201.96, 378.83)
2037	Both	8863810.82 (5920321.62, 11807300.02)	292.96 (195.37, 390.55)
2038	Both	9120543.13 (5821563.35, 12419522.91)	295.54 (188.31, 402.77)
2039	Both	9383908.66 (5701055.9, 13066761.42)	298.14 (180.77, 415.52)
2040	Both	9650229.71 (5555028.8, 13745430.62)	300.8 (172.75, 428.84)
2041	Both	9916173.93 (5380076.67, 14452271.19)	303.53 (164.24, 442.82)
2042	Both	10176624.06 (5172242.87, 15181005.25)	306.34 (155.21, 457.47)
2043	Both	10436369.72 (4933324.78, 15939414.66)	309.22 (145.63, 472.8)
2044	Both	10701060.45 (4664595.26, 16737525.65)	312.17 (135.48, 488.85)
2045	Both	10965593.65 (4361797.96, 17569389.33)	315.21 (124.73, 505.7)
2022	Female	2659714.81 (2521439.13, 2797990.49)	249.8 (244.29, 255.31)
2023	Female	2776858.89 (2617533.2, 2936184.58)	253.71 (245.56, 261.87)
2024	Female	2899690.58 (2708301.57, 3091079.6)	257.57 (245.89, 269.25)

2025	Female	3027119.25 (2791902.79, 3262335.71)	261.39 (245.5, 277.28)
2026	Female	3158419.47 (2867516.44, 3449322.5)	265.21 (244.51, 285.91)
2027	Female	3291933.22 (2933674.7, 3650191.73)	269.01 (242.95, 295.06)
2028	Female	3429274.38 (2991925.94, 3866622.82)	272.75 (240.84, 304.67)
2029	Female	3572449.7 (3043828.67, 4101070.74)	276.43 (238.18, 314.69)
2030	Female	3720062.73 (3087731.05, 4352394.41)	280.07 (235, 325.15)
2031	Female	3871300.89 (3122380.13, 4620221.66)	283.72 (231.34, 336.1)
2032	Female	4024197.7 (3145586.89, 4902808.51)	287.35 (227.19, 347.51)
2033	Female	4180880.64 (3158538.96, 5203222.31)	290.93 (222.53, 359.34)
2034	Female	4343711.54 (3162271.27, 5525151.81)	294.45 (217.35, 371.55)
2035	Female	4510649.26 (3154346.98, 5866951.54)	297.93 (211.67, 384.18)
2036	Female	4680314.82 (3132864.03, 6227765.6)	301.43 (205.54, 397.31)
2037	Female	4849864.92 (3095063.1, 6604666.74)	304.92 (198.94, 410.9)
2038	Female	5021252.03 (3041568.59, 7000935.47)	308.37 (191.83, 424.91)
2039	Female	5197539.07 (2973306.53, 7421771.62)	311.77 (184.23, 439.31)
2040	Female	5376827.61 (2887992.31, 7865662.91)	315.13 (176.14, 454.11)
2041	Female	5557956.73 (2783813.59, 8332099.87)	318.52 (167.6, 469.44)
2042	Female	5738059.75 (2658215.68, 8817903.81)	321.92 (158.59, 485.25)
2043	Female	5918715.59 (2511084.58, 9326346.59)	325.29 (149.08, 501.49)
2044	Female	6103006.26 (2342537.66, 9863474.85)	328.61 (139.09, 518.13)
2045	Female	6289309.48 (2150417.6, 10428201.35)	331.91 (128.62, 535.19)
2022	Male	2657743.85 (2519301.89, 2796185.81)	262.02 (256.25, 267.79)
2023	Male	2740573.72 (2587280.01, 2893867.43)	263.33 (255.46, 271.21)
2024	Male	2827231.4 (2651439.12, 3003023.67)	264.58 (253.92, 275.25)
2025	Male	2916424.25 (2709773.59, 3123074.9)	265.78 (251.78, 279.77)
2026	Male	3006634.82 (2760689.35, 3252580.28)	266.91 (249.14, 284.69)

2027	Male	3096172.73 (2802811.54, 3389533.92)	267.98 (246.04, 289.91)
2028	Male	3187264.21 (2838523.79, 3536004.63)	268.96 (242.51, 295.41)
2029	Male	3281971.02 (2869820.83, 3694121.2)	269.87 (238.6, 301.15)
2030	Male	3377945.71 (2894621.61, 3861269.82)	270.72 (234.32, 307.11)
2031	Male	3472828.08 (2910861.76, 4034794.4)	271.48 (229.69, 313.27)
2032	Male	3564336.85 (2916676.49, 4211997.21)	272.14 (224.69, 319.58)
2033	Male	3654539.76 (2913939.86, 4395139.66)	272.7 (219.35, 326.05)
2034	Male	3746169.75 (2904799.53, 4587539.97)	273.18 (213.7, 332.65)
2035	Male	3837772.24 (2887928.68, 4787615.8)	273.57 (207.75, 339.38)
2036	Male	3927622.54 (2861863.18, 4993381.89)	273.86 (201.5, 346.22)
2037	Male	4013945.9 (2825258.52, 5202633.29)	274.03 (194.94, 353.12)
2038	Male	4099291.1 (2779994.76, 5418587.44)	274.1 (188.11, 360.09)
2039	Male	4186369.59 (2727749.38, 5644989.8)	274.08 (181.03, 367.14)
2040	Male	4273402.1 (2667036.49, 5879767.71)	273.99 (173.71, 374.26)
2041	Male	4358217.2 (2596263.08, 6120171.32)	273.79 (166.16, 381.41)
2042	Male	4438564.31 (2514027.19, 6363101.44)	273.47 (158.37, 388.57)
2043	Male	4517654.13 (2422240.2, 6613068.07)	273.07 (150.38, 395.76)
2044	Male	4598054.2 (2322057.6, 6874050.8)	272.6 (142.21, 402.98)
2045	Male	4676284.17 (2211380.36, 7141187.98)	272.06 (133.87, 410.24)

Notes: ASR: age-standardized rates; YLDs: years lived with disability.

Table S104. Prevalence and YLDs of Alzheimer's disease and other dementias in China of future forecasts using bayesian age-period-cohort model.

Year	Sex	Number	ASR
**Prevalence**			
2022	Both	17858226.49 (17088499.97, 18627953.02)	904.77 (865.75, 943.79)
2023	Both	19071080.9 (18011547.87, 20130613.93)	931.72 (879.9, 983.54)
2024	Both	20420827.82 (18919008.52, 21922647.12)	959.55 (888.9, 1030.21)
2025	Both	21888361.43 (19792082.31, 23984640.56)	988.54 (893.75, 1083.33)
2026	Both	23449339.15 (20601982.9, 26296695.4)	1018.49 (894.67, 1142.32)
2027	Both	25079905.46 (21316196.65, 28843614.27)	1049.17 (891.53, 1206.81)
2028	Both	26845722.7 (21976508.72, 31714936.67)	1080.84 (884.57, 1277.11)
2029	Both	28803283.96 (22605950.26, 35000617.66)	1113.82 (873.92, 1353.73)
2030	Both	30927530.99 (23155113.14, 38699948.83)	1148.38 (859.51, 1437.25)
2031	Both	33182834.22 (23566675.96, 42798992.47)	1184.35 (840.85, 1527.85)
2032	Both	35528340.51 (23781201.42, 47275479.61)	1221.45 (817.3, 1625.61)
2033	Both	38039011.61 (23818228.08, 52259795.15)	1260.06 (788.71, 1731.42)
2034	Both	40789670.96 (23680945.69, 57898396.23)	1300.57 (754.81, 1846.33)
2035	Both	43754885.29 (23300648.56, 64209122.03)	1343.31 (715.15, 1971.47)
2036	Both	46895463.92 (22600702.31, 71190225.53)	1388.29 (668.95, 2107.62)
2037	Both	50160949.28 (21500381.54, 78821517.02)	1435.28 (615.19, 2255.38)
2038	Both	53640554.09 (19979533.88, 87301574.3)	1484.72 (553.12, 2416.32)
2039	Both	57432363.11 (17993662.28, 96871063.94)	1537.03 (481.82, 2592.23)
2040	Both	61519113.48 (15436070.88, 107602156.08)	1592.64 (400.1, 2785.18)
2041	Both	65871132.62 (12189148.89, 119553116.35)	1651.82 (306.41, 2997.22)
2042	Both	70441053.73 (8125500.68, 132756988.83)	1714.56 (198.86, 3230.3)
2043	Both	75359840.77 (4613858.91, 147591976.62)	1781.37 (110.71, 3487.38)

2044	Both	80752791.39 (3441300.89, 164481800.57)	1852.7 (80.14, 3771.81)
2045	Both	86582456.25 (2043830.41, 183569921.63)	1929.12 (46.04, 4087.66)
2022	Female	11142650.89 (10653421.85, 11631879.93)	1034.39 (1006.98, 1061.8)
2023	Female	11959031.36 (11269339.33, 12648723.39)	1068.35 (1020.42, 1116.27)
2024	Female	12869696.34 (11874107.95, 13865284.74)	1103.48 (1028.63, 1178.33)
2025	Female	13865726.46 (12458242.18, 15273210.75)	1139.86 (1032.86, 1246.86)
2026	Female	14933723.38 (13003637.86, 16863808.9)	1177.11 (1032.96, 1321.27)
2027	Female	16059835.23 (13488469.92, 18631200.55)	1214.81 (1028.43, 1401.2)
2028	Female	17285952.49 (13936132.86, 20635772.12)	1253.39 (1019.56, 1487.22)
2029	Female	18649186.37 (14358341.56, 22940031.19)	1293.2 (1006.48, 1579.93)
2030	Female	20136984.12 (14722538.37, 25551429.88)	1334.39 (989.07, 1679.71)
2031	Female	21728663.4 (14989566.7, 28467760.1)	1376.59 (966.82, 1786.36)
2032	Female	23398863.13 (15117440.68, 31680285.57)	1419.28 (939.08, 1899.49)
2033	Female	25197710.63 (15113258.22, 35282163.03)	1462.89 (905.95, 2019.82)
2034	Female	27176272.87 (14972684.58, 39379861.16)	1507.78 (867.45, 2148.12)
2035	Female	29320969.37 (14645417.59, 43996521.16)	1554.16 (823.39, 2284.94)
2036	Female	31607735.65 (14074379.24, 49141092.05)	1601.83 (773.32, 2430.34)
2037	Female	34003404.97 (13198206.43, 54808603.51)	1650.26 (716.65, 2583.87)
2038	Female	36568654.09 (11991336.61, 61145971.57)	1699.75 (653.26, 2746.25)
2039	Female	39373291.99 (10410228.58, 68336355.39)	1750.6 (582.97, 2918.24)
2040	Female	42410797.87 (8370573.15, 76451022.59)	1803.04 (505.51, 3100.58)
2041	Female	45664797.46 (5777888.57, 85551706.35)	1857.08 (420.47, 3293.7)
2042	Female	49105463.87 (2528522.57, 95682787.22)	1912.32 (327.31, 3497.33)
2043	Female	52825717.6 (0, 107137589.19)	1968.85 (225.71, 3712)
2044	Female	56918749.54 (0, 120255017.76)	2026.81 (115.33, 3938.28)
2045	Female	61366945.37 (0, 135182375.13)	2086.38 (-4.22, 4176.98)

2022	Male	6715575.61 (6435078.12, 6996073.09)	741.62 (723.96, 759.27)
2023	Male	7112049.54 (6742208.54, 7481890.53)	759.14 (730.12, 788.17)
2024	Male	7551131.48 (7044900.58, 8057362.39)	777.05 (733.08, 821.02)
2025	Male	8022634.97 (7333840.13, 8711429.81)	795.4 (733.7, 857.1)
2026	Male	8515615.77 (7598345.04, 9432886.5)	813.97 (732, 895.93)
2027	Male	9020070.22 (7827726.73, 10212413.71)	832.53 (727.82, 937.23)
2028	Male	9559770.21 (8040375.86, 11079164.56)	851.25 (721.36, 981.14)
2029	Male	10154097.59 (8247608.7, 12060586.48)	870.33 (712.76, 1027.89)
2030	Male	10790546.86 (8432574.77, 13148518.96)	889.84 (702.05, 1077.63)
2031	Male	11454170.82 (8577109.26, 14331232.37)	909.58 (689.01, 1130.15)
2032	Male	12129477.39 (8663760.73, 15595194.04)	929.27 (673.34, 1185.2)
2033	Male	12841300.99 (8704969.86, 16977632.12)	949.09 (655.17, 1243)
2034	Male	13613398.09 (8708261.11, 18518535.07)	969.23 (634.6, 1303.85)
2035	Male	14433915.92 (8655230.97, 20212600.87)	989.78 (611.63, 1367.92)
2036	Male	15287728.28 (8526323.07, 22049133.48)	1010.6 (586.07, 1435.12)
2037	Male	16157544.3 (8302175.1, 24012913.51)	1031.4 (557.68, 1505.12)
2038	Male	17071899.99 (7988197.27, 26155602.72)	1052.36 (526.51, 1578.2)
2039	Male	18059071.12 (7583433.7, 28534708.55)	1073.62 (492.62, 1654.63)
2040	Male	19108315.61 (7065497.72, 31151133.49)	1095.28 (455.94, 1734.61)
2041	Male	20206335.15 (6411260.31, 34001410)	1117.25 (416.37, 1818.13)
2042	Male	21335589.86 (5596978.11, 37074201.61)	1139.28 (373.69, 1904.87)
2043	Male	22534123.17 (4613858.91, 40454387.43)	1161.52 (327.92, 1995.12)
2044	Male	23834041.85 (3441300.89, 44226782.8)	1184.06 (279.03, 2089.1)
2045	Male	25215510.88 (2043830.41, 48387546.5)	1206.94 (226.94, 2186.94)
**YLDs**			
2022	Both	3661111.89 (3506127.35, 3816096.43)	187.33 (179.39, 195.27)

2023	Both	3927513.64 (3701811.58, 4153215.71)	193.75 (182.6, 204.9)
2024	Both	4225247.69 (3891976.96, 4558518.42)	200.41 (184.58, 216.24)
2025	Both	4550306.92 (4072722.98, 5027890.87)	207.37 (185.58, 229.17)
2026	Both	4898058.71 (4237690.28, 5558427.15)	214.63 (185.66, 243.61)
2027	Both	5263832.43 (4379296.54, 6148368.33)	222.14 (184.77, 259.51)
2028	Both	5662142.76 (4505372.38, 6818913.14)	229.95 (182.92, 276.98)
2029	Both	6105935.02 (4619623.36, 7592246.69)	238.13 (180.11, 296.16)
2030	Both	6591105.02 (4710566.88, 8471643.16)	246.76 (176.29, 317.23)
2031	Both	7111820.05 (4765151.02, 9458489.08)	255.83 (171.34, 340.31)
2032	Both	7660768.44 (4769204.57, 10552332.3)	265.28 (165.08, 365.49)
2033	Both	8256853.4 (4724592.72, 11789114.09)	275.22 (157.41, 393.03)
2034	Both	8917665.79 (4627542.46, 13207789.11)	285.73 (148.2, 423.25)
2035	Both	9637460.65 (4458097.55, 14816823.76)	296.9 (137.29, 456.52)
2036	Both	10407881.69 (4194506.51, 16621256.87)	308.79 (124.41, 493.16)
2037	Both	11217458.21 (3813445.55, 18621470.87)	321.35 (109.23, 533.46)
2038	Both	12087523.55 (3302109, 20872938.1)	334.71 (91.46, 577.96)
2039	Both	13043283.3 (2640098.31, 23446468.3)	348.98 (70.7, 627.25)
2040	Both	14083757.79 (1792818.46, 26374701.15)	364.3 (46.49, 682.1)
2041	Both	15205909.01 (932255.52, 29690747.92)	380.78 (23.59, 743.32)
2042	Both	16402731.03 (686824.55, 33424892.07)	398.48 (16.87, 811.72)
2043	Both	17709134.74 (389214.19, 37699157.88)	417.55 (9.25, 888.49)
2044	Both	19159406.3 (32895.98, 42654961.61)	438.15 (0.74, 974.95)
2045	Both	20750545.59 (0, 48371172.56)	460.5 (0, 1072.79)
2022	Female	2355892.66 (2253287.33, 2458497.99)	219.78 (213.69, 225.87)
2023	Female	2539180.27 (2386634.33, 2691726.21)	227.94 (216.77, 239.12)
2024	Female	2744446.38 (2515855.44, 2973037.33)	236.42 (218.63, 254.21)

2025	Female	2969880.46 (2638931.19, 3300829.74)	245.23 (219.51, 270.96)
2026	Female	3212922.58 (2751667.23, 3674177.93)	254.32 (219.35, 289.29)
2027	Female	3470856.09 (2848809.53, 4092902.64)	263.58 (218.01, 309.14)
2028	Female	3753179.64 (2934640.25, 4571719.02)	273.1 (215.54, 330.67)
2029	Female	4068672.08 (3010816.68, 5126527.49)	282.97 (211.94, 354.01)
2030	Female	4415528.44 (3069466.87, 5761590)	293.23 (207.16, 379.3)
2031	Female	4790435.33 (3101234.37, 6479636.29)	303.8 (201.07, 406.53)
2032	Female	5188883.78 (3095704.77, 7282062.79)	314.58 (193.52, 435.63)
2033	Female	5623865.63 (3052477.9, 8195253.37)	325.65 (184.49, 466.81)
2034	Female	6107884.55 (2967184.42, 9248584.68)	337.1 (173.95, 500.25)
2035	Female	6638101.27 (2824801.44, 10451401.1)	348.97 (161.82, 536.12)
2036	Female	7209452.46 (2608527.39, 11810377.54)	361.25 (147.99, 574.5)
2037	Female	7814446.1 (2300058.45, 13328833.76)	373.8 (132.29, 615.32)
2038	Female	8468186.9 (1886840.51, 15049533.28)	386.71 (114.65, 658.76)
2039	Female	9189236.16 (1350264.28, 17028208.05)	400.02 (94.99, 705.06)
2040	Female	9978432.28 (661854.68, 19295013.91)	413.82 (73.2, 754.43)
2041	Female	10834717.91 (0, 21880621.24)	428.1 (49.16, 807.05)
2042	Female	11754084.98 (0, 24814424.52)	442.78 (22.69, 862.87)
2043	Female	12762302.75 (0, 28194664.13)	457.88 (-6.32, 922.08)
2044	Female	13885952.08 (0, 32138443.79)	473.44 (-37.99, 984.87)
2045	Female	15125631.46 (0, 36720266.34)	489.5 (-72.49, 1051.49)
2022	Male	1305219.23 (1252840.01, 1357598.44)	146.49 (142.94, 150.04)
2023	Male	1388333.38 (1315177.25, 1461489.5)	150.56 (144.37, 156.76)
2024	Male	1480801.31 (1376121.53, 1585481.09)	154.74 (145.12, 164.36)
2025	Male	1580426.46 (1433791.78, 1727061.13)	159.03 (145.35, 172.71)
2026	Male	1685136.14 (1486023.05, 1884249.22)	163.4 (145.06, 181.74)

2027	Male	1792976.35 (1530487.01, 2055465.68)	167.81 (144.2, 191.43)
2028	Male	1908963.13 (1570732.14, 2247194.12)	172.3 (142.8, 201.79)
2029	Male	2037262.94 (1608806.68, 2465719.2)	176.88 (140.88, 212.88)
2030	Male	2175576.58 (1641100.01, 2710053.16)	181.59 (138.44, 224.74)
2031	Male	2321384.72 (1663916.65, 2978852.79)	186.39 (135.43, 237.35)
2032	Male	2471884.66 (1673499.8, 3270269.51)	191.22 (131.78, 250.67)
2033	Male	2632987.77 (1672114.82, 3593860.72)	196.12 (127.5, 264.75)
2034	Male	2809781.24 (1660358.04, 3959204.43)	201.13 (122.61, 279.65)
2035	Male	2999359.38 (1633296.11, 4365422.65)	206.25 (117.08, 295.41)
2036	Male	3198429.22 (1585979.12, 4810879.33)	211.47 (110.89, 312.06)
2037	Male	3403012.11 (1513387.1, 5292637.11)	216.74 (103.96, 329.53)
2038	Male	3619336.65 (1415268.49, 5823404.82)	222.09 (96.31, 347.88)
2039	Male	3854047.14 (1289834.03, 6418260.25)	227.54 (87.91, 367.18)
2040	Male	4105325.51 (1130963.78, 7079687.24)	233.11 (78.75, 387.46)
2041	Male	4371191.1 (932255.52, 7810126.68)	238.79 (68.8, 408.77)
2042	Male	4648646.05 (686824.55, 8610467.55)	244.52 (57.99, 431.05)
2043	Male	4946831.99 (389214.19, 9504493.75)	250.35 (46.33, 454.36)
2044	Male	5273454.21 (32895.98, 10516517.82)	256.27 (33.79, 478.76)
2045	Male	5624914.12 (0, 11650906.22)	262.31 (20.34, 504.28)

Notes: ASR: age-standardized rates; YLDs: years lived with disability.

Table S105. Prevalence and YLDs of Parkinson's disease in China of future forecasts using bayesian age-period-cohort model.

Year	Sex	Number	ASR
**Prevalence**			
2022	Both	5595714.88 (4973141.19, 6218288.56)	414.79 (368.69, 460.9)
2023	Both	5912245.18 (5218981.9, 6605508.46)	424.06 (374.38, 473.73)
2024	Both	6251861.83 (5459555.69, 7044167.97)	433.35 (378.48, 488.23)
2025	Both	6609124.25 (5684735.34, 7533513.17)	442.65 (380.77, 504.52)
2026	Both	6979167.97 (5886495.26, 8071840.67)	451.97 (381.23, 522.7)
2027	Both	7357196.43 (6058256.17, 8656136.68)	461.31 (379.89, 542.72)
2028	Both	7752549.82 (6205922.89, 9299176.75)	470.67 (376.79, 564.55)
2029	Both	8173798.48 (6333927.83, 10013669.13)	480.07 (372.03, 588.11)
2030	Both	8614022.91 (6433809.92, 10794235.89)	489.48 (365.61, 613.36)
2031	Both	9065838.07 (6497068.49, 11634607.65)	498.91 (357.55, 640.27)
2032	Both	9522684.89 (6516356.08, 12529013.7)	508.35 (347.86, 668.84)
2033	Both	9995160.89 (6496307.63, 13494014.15)	517.83 (336.55, 699.11)
2034	Both	10494203.48 (6439862.39, 14548544.56)	527.38 (323.61, 731.14)
2035	Both	11012510.09 (6337283.03, 15687737.15)	536.99 (308.99, 765)
2036	Both	11540821.23 (6178315.04, 16903327.43)	546.64 (292.6, 800.68)
2037	Both	12071236.51 (5954434.82, 18188038.2)	556.34 (274.37, 838.31)
2038	Both	12617214.02 (5667937.85, 19566490.2)	566.15 (254.25, 878.04)
2039	Both	13192341.95 (5318184.72, 21066499.18)	576.11 (232.15, 920.07)
2040	Both	13787544.26 (4893137.65, 22681950.87)	586.26 (207.94, 964.58)
2041	Both	14390934.02 (4380739.36, 24401128.68)	596.55 (181.45, 1011.65)
2042	Both	14992451.34 (3771032.69, 26213872.79)	607.01 (152.5, 1061.52)
2043	Both	15608016.07 (3060905.68, 28155139.41)	617.72 (120.92, 1114.53)

2044	Both	16253851.82 (2242724.17, 30265045.07)	628.78 (86.5, 1171.08)
2045	Both	16919095.1 (1301602.26, 32536857.28)	640.23 (48.96, 1231.54)
2022	Female	2294001.03 (2075627.62, 2512374.44)	321.93 (308.18, 335.68)
2023	Female	2427555.61 (2181625.82, 2673485.4)	328.79 (310.77, 346.82)
2024	Female	2571040.7 (2285618.95, 2856462.45)	335.62 (311.79, 359.45)
2025	Female	2722531.13 (2383751.64, 3061310.62)	342.4 (311.46, 373.34)
2026	Female	2880346.13 (2473099.51, 3287592.74)	349.17 (309.94, 388.39)
2027	Female	3042689.92 (2551170.61, 3534209.23)	355.91 (307.33, 404.49)
2028	Female	3213132.4 (2620256.91, 3806007.89)	362.59 (303.65, 421.54)
2029	Female	3394953.38 (2682009.81, 4107896.95)	369.18 (298.9, 439.47)
2030	Female	3585561.96 (2733056.68, 4438067.24)	375.66 (293.09, 458.23)
2031	Female	3782208.74 (2769956.91, 4794460.58)	382.03 (286.25, 477.81)
2032	Female	3982267.07 (2789544.46, 5174989.69)	388.28 (278.36, 498.19)
2033	Female	4189824.84 (2793461.97, 5586187.71)	394.39 (269.45, 519.34)
2034	Female	4409140.33 (2782718.34, 6035562.33)	400.36 (259.51, 541.21)
2035	Female	4637281.08 (2753197.4, 6521364.76)	406.16 (248.55, 563.78)
2036	Female	4870571.73 (2700566.72, 7040576.74)	411.77 (236.56, 586.97)
2037	Female	5105763.59 (2621113.84, 7590413.34)	417.17 (223.55, 610.79)
2038	Female	5348065.94 (2515619.37, 8180512.5)	422.4 (209.56, 635.23)
2039	Female	5602959.99 (2383834.11, 8822085.87)	427.44 (194.61, 660.27)
2040	Female	5866941.27 (2220771.53, 9513111.02)	432.29 (178.72, 685.87)
2041	Female	6135224.08 (2021310.38, 10249137.78)	436.91 (161.87, 711.94)
2042	Female	6403739.44 (1781161.25, 11026317.63)	441.29 (144.1, 738.48)
2043	Female	6679073.65 (1499145.8, 11859004.42)	445.48 (125.45, 765.5)
2044	Female	6967905.1 (1172258.74, 12763558.7)	449.49 (105.95, 793.02)
2045	Female	7265771.53 (794210.55, 13737376.19)	453.32 (85.63, 821.01)

2022	Male	3301713.85 (2897513.57, 3705914.12)	520.97 (493.32, 548.63)
2023	Male	3484689.57 (3037356.07, 3932023.07)	533.21 (498.34, 568.09)
2024	Male	3680821.13 (3173936.75, 4187705.52)	545.4 (500.68, 590.13)
2025	Male	3886593.12 (3300983.7, 4472202.54)	557.53 (500.61, 614.44)
2026	Male	4098821.84 (3413395.75, 4784247.93)	569.62 (498.41, 640.83)
2027	Male	4314506.51 (3507085.56, 5121927.45)	581.66 (494.19, 669.13)
2028	Male	4539417.42 (3585665.98, 5493168.86)	593.57 (488.01, 699.14)
2029	Male	4778845.09 (3651918.01, 5905772.17)	605.31 (479.9, 730.71)
2030	Male	5028460.95 (3700753.24, 6356168.65)	616.82 (469.87, 763.78)
2031	Male	5283629.33 (3727111.58, 6840147.08)	628.1 (457.94, 798.26)
2032	Male	5540417.82 (3726811.62, 7354024.02)	639.12 (444.11, 834.13)
2033	Male	5805336.05 (3702845.66, 7907826.44)	649.85 (428.41, 871.29)
2034	Male	6085063.14 (3657144.05, 8512982.23)	660.25 (410.84, 909.66)
2035	Male	6375229.02 (3584085.64, 9166372.4)	670.29 (391.42, 949.16)
2036	Male	6670249.51 (3477748.32, 9862750.69)	679.89 (370.12, 989.66)
2037	Male	6965472.92 (3333320.98, 10597624.86)	689.05 (346.99, 1031.11)
2038	Male	7269148.08 (3152318.47, 11385977.69)	697.78 (322.09, 1073.46)
2039	Male	7589381.96 (2934350.61, 12244413.31)	706.07 (295.47, 1116.66)
2040	Male	7920602.99 (2672366.13, 13168839.85)	713.9 (267.17, 1160.63)
2041	Male	8255709.94 (2359428.98, 14151990.9)	721.18 (237.21, 1205.14)
2042	Male	8588711.9 (1989871.44, 15187555.16)	727.91 (205.65, 1250.17)
2043	Male	8928942.42 (1561759.89, 16296134.99)	734.15 (172.6, 1295.71)
2044	Male	9285946.72 (1070465.42, 17501486.36)	739.93 (138.12, 1341.75)
2045	Male	9653323.58 (507391.71, 18799481.09)	745.25 (102.28, 1388.22)
**YLDs**			
2022	Both	793661.85 (711847.62, 875476.08)	36.84 (33.04, 40.64)

2023	Both	836059.51 (743873.98, 928245.04)	37.57 (33.42, 41.71)
2024	Both	881350.18 (774145.32, 988555.05)	38.3 (33.64, 42.96)
2025	Both	928813.27 (801302.07, 1056324.47)	39.02 (33.66, 44.38)
2026	Both	977805.14 (824357.69, 1131252.6)	39.75 (33.51, 45.99)
2027	Both	1027682.6 (842531.72, 1212833.48)	40.48 (33.18, 47.77)
2028	Both	1079670.53 (856673.88, 1302667.19)	41.2 (32.69, 49.72)
2029	Both	1134887.56 (867374.72, 1402400.4)	41.93 (32.05, 51.82)
2030	Both	1192420.91 (873518.89, 1511322.93)	42.66 (31.25, 54.08)
2031	Both	1251319.63 (873992.68, 1628646.58)	43.4 (30.31, 56.49)
2032	Both	1310719.22 (867811.13, 1753627.31)	44.13 (29.22, 59.05)
2033	Both	1371991.79 (855499.6, 1888483.98)	44.87 (27.98, 61.77)
2034	Both	1436570.71 (837333.11, 2035808.31)	45.62 (26.59, 64.66)
2035	Both	1503552.33 (811997.09, 2195107.58)	46.38 (25.04, 67.72)
2036	Both	1571827.51 (778134.23, 2365520.78)	47.15 (23.33, 70.97)
2037	Both	1640407.37 (734562.96, 2546251.78)	47.93 (21.45, 74.41)
2038	Both	1711036.58 (681344.32, 2740728.85)	48.72 (19.38, 78.06)
2039	Both	1785471.09 (618089.38, 2952854.05)	49.53 (17.13, 81.94)
2040	Both	1862602.34 (543061.58, 3182146.1)	50.37 (14.66, 86.08)
2041	Both	1941040.47 (454535.95, 3427550.8)	51.23 (11.96, 90.5)
2042	Both	2019535.69 (350985.71, 3688102.86)	52.12 (9.02, 95.22)
2043	Both	2100110.26 (231471.77, 3968820.25)	53.04 (5.8, 100.28)
2044	Both	2184851.39 (141559.72, 4275573.82)	54 (3.46, 105.73)
2045	Both	2272481.97 (89943.32, 4608253.53)	55.01 (2.14, 111.62)
2022	Female	321784.47 (293202.18, 350366.76)	28.36 (27.2, 29.51)
2023	Female	339552.39 (307159.98, 371944.81)	28.89 (27.35, 30.43)
2024	Female	358551.14 (320613.14, 396489.14)	29.42 (27.36, 31.49)

2025	Female	378525 (333065.73, 423984.27)	29.95 (27.25, 32.65)
2026	Female	399252.34 (344162.22, 454342.46)	30.47 (27.04, 33.9)
2027	Female	420489.49 (353597.71, 487381.28)	30.99 (26.73, 35.24)
2028	Female	442692.63 (361689.89, 523695.37)	31.5 (26.33, 36.66)
2029	Female	466278 (368662.59, 563893.41)	31.99 (25.84, 38.15)
2030	Female	490897.03 (374077.68, 607716.37)	32.48 (25.26, 39.7)
2031	Female	516193.28 (377497.86, 654888.71)	32.96 (24.59, 41.32)
2032	Female	541811.02 (378510.59, 705111.45)	33.42 (23.83, 43.01)
2033	Female	568261.4 (377328.18, 759194.62)	33.87 (22.99, 44.75)
2034	Female	596084.4 (374084.95, 818083.85)	34.31 (22.06, 46.55)
2035	Female	624907.51 (368265.43, 881549.59)	34.73 (21.05, 48.41)
2036	Female	654287.75 (359340.17, 949235.33)	35.13 (19.95, 50.31)
2037	Female	683805.96 (346840.44, 1020771.49)	35.52 (18.77, 52.27)
2038	Female	714111.02 (330863.9, 1097358.14)	35.89 (17.51, 54.27)
2039	Female	745883.88 (311375.25, 1180392.79)	36.24 (16.18, 56.31)
2040	Female	778686.38 (287753.95, 1269619.65)	36.58 (14.77, 58.4)
2041	Female	811954.39 (259373.97, 1364536.24)	36.9 (13.28, 60.52)
2042	Female	845165.85 (225694.18, 1464639.57)	37.2 (11.72, 62.68)
2043	Female	879114.06 (186551.42, 1571680.31)	37.49 (10.1, 64.88)
2044	Female	914613.99 (141559.72, 1687678.12)	37.76 (8.4, 67.12)
2045	Female	951126.45 (89943.32, 1812337.6)	38.01 (6.65, 69.38)
2022	Male	471877.38 (418645.44, 525109.32)	46.5 (44.13, 48.87)
2023	Male	496507.12 (436714, 556300.23)	47.47 (44.33, 50.61)
2024	Male	522799.04 (453532.18, 592065.91)	48.43 (44.26, 52.59)
2025	Male	550288.27 (468236.34, 632340.2)	49.38 (43.95, 54.8)
2026	Male	578552.81 (480195.47, 676910.14)	50.32 (43.44, 57.19)

2027	Male	607193.11 (488934.01, 725452.2)	51.24 (42.72, 59.77)
2028	Male	636977.9 (494983.99, 778971.82)	52.16 (41.82, 62.49)
2029	Male	668609.56 (498712.13, 838506.99)	53.05 (40.73, 65.37)
2030	Male	701523.88 (499441.21, 903606.56)	53.92 (39.46, 68.38)
2031	Male	735126.35 (496494.82, 973757.87)	54.76 (38.01, 71.52)
2032	Male	768908.19 (489300.53, 1048515.86)	55.58 (36.37, 74.78)
2033	Male	803730.39 (478171.42, 1129289.36)	56.37 (34.57, 78.17)
2034	Male	840486.31 (463248.16, 1217724.46)	57.12 (32.59, 81.66)
2035	Male	878644.82 (443731.66, 1313557.99)	57.85 (30.44, 85.26)
2036	Male	917539.76 (418794.06, 1416285.46)	58.53 (28.12, 88.95)
2037	Male	956601.41 (387722.53, 1525480.29)	59.18 (25.63, 92.72)
2038	Male	996925.56 (350480.42, 1643370.7)	59.79 (22.99, 96.58)
2039	Male	1039587.2 (306714.13, 1772461.25)	60.36 (20.2, 100.51)
2040	Male	1083915.96 (255307.63, 1912526.45)	60.89 (17.26, 104.51)
2041	Male	1129086.08 (195161.98, 2063014.56)	61.37 (14.18, 108.57)
2042	Male	1174369.83 (125291.54, 2223463.3)	61.81 (10.96, 112.67)
2043	Male	1220996.21 (44920.35, 2397139.94)	62.21 (7.61, 116.82)
2044	Male	1270237.4 (0, 2587895.7)	62.58 (4.15, 121.01)
2045	Male	1321355.52 (0, 2795915.93)	62.9 (0.57, 125.24)

Notes: ASR: age-standardized rates; YLDs: years lived with disability.

Table S106. Prevalence and YLDs of upper digestive system diseases in China of future forecasts using bayesian age-period-cohort model.

Year	Sex	Number	ASR
**Prevalence**			
2022	Both	99533866.32 (93174984.81, 105892747.83)	5274.12 (4937.08, 5611.17)
2023	Both	100794450.65 (93439447.86, 108149453.44)	5285.56 (4899.71, 5671.4)
2024	Both	102022616.7 (93065104.15, 110980129.26)	5295.98 (4830.79, 5761.17)
2025	Both	103221803.47 (92100960.58, 114342646.37)	5305.8 (4733.92, 5877.68)
2026	Both	104398429.35 (90626434.53, 118170424.18)	5315.48 (4614, 6016.97)
2027	Both	105553552.33 (88703359.41, 122403745.25)	5325.25 (4474.83, 6175.67)
2028	Both	106671079.68 (86364954.21, 126977205.15)	5335.3 (4319.32, 6351.28)
2029	Both	107767110.31 (83654321.43, 131879899.18)	5345.85 (4149.36, 6542.35)
2030	Both	108856760.28 (80599692.7, 137113827.86)	5357.36 (3966.31, 6748.4)
2031	Both	109957232.55 (77219589.99, 142694875.12)	5370.21 (3770.94, 6969.48)
2032	Both	111084819.67 (73523340.08, 148646299.26)	5384.64 (3563.5, 7205.79)
2033	Both	112188795.39 (69475653.73, 154901937.05)	5401.1 (3344.33, 7457.86)
2034	Both	113298757.22 (65091478.77, 161506035.67)	5419.9 (3113.34, 7726.46)
2035	Both	114438568.74 (60373035.8, 168504101.67)	5441.5 (2870.23, 8012.78)
2036	Both	115630939.34 (55312532.07, 175949346.62)	5466.24 (2614.29, 8318.2)
2037	Both	116897859.88 (49892916.26, 183902803.5)	5494.36 (2344.47, 8644.24)
2038	Both	118177972.07 (44060709.54, 192295234.59)	5526.46 (2059.81, 8993.11)
2039	Both	119509410.77 (37802656.41, 201216165.12)	5563 (1758.93, 9367.07)
2040	Both	120905060.52 (31084885.12, 210725235.92)	5604.41 (1440.04, 9768.77)
2041	Both	122374457.55 (23862833.16, 220886081.94)	5650.98 (1100.91, 10201.05)
2042	Both	123923481.05 (16084464.27, 231765160.59)	5703 (739.39, 10667.15)
2043	Both	125505675.82 (7692660.92, 243333124.41)	5761.3 (353.9, 11171.68)

2044	Both	127157691.11 (778179.78, 255722666.52)	5826.52 (36.45, 11719.43)
2045	Both	128897687.69 (0, 269048189.28)	5899.22 (0, 12315.83)
2022	Female	53840812.63 (50226251.15, 57455374.11)	5655.64 (5485.8, 5825.49)
2023	Female	54617797.86 (50454877.14, 58780718.59)	5667.88 (5402.75, 5933.02)
2024	Female	55378630.13 (50334814.26, 60422446)	5678.08 (5290.23, 6065.94)
2025	Female	56125423.95 (49888086.89, 62362761)	5686.46 (5155.48, 6217.43)
2026	Female	56861434.98 (49155039.74, 64567830.22)	5693.18 (5002.11, 6384.25)
2027	Female	57586677.65 (48167866.05, 67005489.26)	5698.15 (4832.04, 6564.27)
2028	Female	58293013.37 (46944954.62, 69641072.12)	5701.34 (4646.87, 6755.82)
2029	Female	58989564.6 (45509672.69, 72469456.52)	5702.78 (4447.86, 6957.71)
2030	Female	59684466.34 (43876703.47, 75492229.22)	5702.71 (4236.13, 7169.29)
2031	Female	60386380.78 (42054795.5, 78717966.07)	5701.23 (4012.48, 7389.97)
2032	Female	61102651.14 (40047112.01, 82158190.27)	5698.21 (3777.43, 7618.99)
2033	Female	61808184.46 (37835582.14, 85780786.79)	5693.9 (3531.9, 7855.91)
2034	Female	62518470.92 (35426780.39, 89610161.45)	5688.43 (3276.61, 8100.25)
2035	Female	63245669.01 (32820121.91, 93671216.12)	5682 (3012.22, 8351.77)
2036	Female	64001019.2 (30009327.46, 97992710.94)	5674.57 (2739.12, 8610.02)
2037	Female	64794460.88 (26982698.28, 102606223.49)	5665.98 (2457.6, 8874.37)
2038	Female	65596973.04 (23712020.39, 107481925.69)	5656.61 (2168.35, 9144.87)
2039	Female	66428093.97 (20187436.11, 112668751.83)	5646.63 (1871.89, 9421.36)
2040	Female	67295150.5 (16388316.21, 118201984.79)	5636.12 (1568.6, 9703.64)
2041	Female	68203044.07 (12287611.93, 124118476.21)	5624.92 (1258.7, 9991.14)
2042	Female	69153747.05 (7854963.19, 130455193.67)	5612.78 (942.41, 10283.15)
2043	Female	70125099.56 (3056862.36, 137204800.78)	5600.17 (620.23, 10580.11)
2044	Female	71136013.74 (0, 144441949.99)	5587.27 (292.49, 10882.05)
2045	Female	72197308.57 (0, 152235732.28)	5574.13 (-40.55, 11188.81)

2022	Male	45693053.69 (42948733.66, 48437373.72)	4886.48 (4751.92, 5021.03)
2023	Male	46176652.79 (42984570.72, 49368734.85)	4894.63 (4680.32, 5108.95)
2024	Male	46643986.57 (42730289.89, 50557683.26)	4901.27 (4584.67, 5217.86)
2025	Male	47096379.53 (42212873.68, 51979885.37)	4906.53 (4470.9, 5342.16)
2026	Male	47536994.37 (41471394.79, 53602593.96)	4910.6 (4341.96, 5479.25)
2027	Male	47966874.68 (40535493.37, 55398255.99)	4913.42 (4199.4, 5627.45)
2028	Male	48378066.31 (39419999.59, 57336133.03)	4914.89 (4044.45, 5785.33)
2029	Male	48777545.7 (38144648.75, 59410442.66)	4914.97 (3878.09, 5951.85)
2030	Male	49172293.94 (36722989.22, 61621598.65)	4913.83 (3701.22, 6126.44)
2031	Male	49570851.77 (35164794.49, 63976909.05)	4911.61 (3514.55, 6308.68)
2032	Male	49982168.53 (33476228.07, 66488108.99)	4908.25 (3318.53, 6497.98)
2033	Male	50380610.93 (31640071.6, 69121150.27)	4903.84 (3113.84, 6693.85)
2034	Male	50780286.3 (29664698.38, 71895874.22)	4898.46 (2901.04, 6895.87)
2035	Male	51192899.72 (27552913.9, 74832885.55)	4892.23 (2680.68, 7103.79)
2036	Male	51629920.14 (25303204.61, 77956635.68)	4885.22 (2453.12, 7317.32)
2037	Male	52103398.99 (22910217.98, 81296580.01)	4877.33 (2218.63, 7536.02)
2038	Male	52580999.03 (20348689.15, 84813308.9)	4868.74 (1977.73, 7759.76)
2039	Male	53081316.79 (17615220.3, 88547413.29)	4859.57 (1730.81, 7988.32)
2040	Male	53609910.02 (14696568.91, 92523251.13)	4849.88 (1478.23, 8221.54)
2041	Male	54171413.48 (11575221.23, 96767605.73)	4839.62 (1220.19, 8459.04)
2042	Male	54769734 (8229501.08, 101309966.92)	4828.62 (956.89, 8700.34)
2043	Male	55380576.25 (4635798.55, 106128323.63)	4817.14 (688.72, 8945.57)
2044	Male	56021677.38 (778179.78, 111280716.53)	4805.33 (415.98, 9194.68)
2045	Male	56700379.12 (0, 116812457)	4793.22 (138.89, 9447.55)
**YLDs**			
2022	Both	1294074.86 (1210628.21, 1377521.51)	66.93 (62.59, 71.27)

2023	Both	1307508.94 (1205569.04, 1409448.83)	66.76 (61.53, 71.99)
2024	Both	1320725.75 (1189429.56, 1452021.94)	66.59 (59.95, 73.24)
2025	Both	1333699.79 (1163851.88, 1503547.71)	66.44 (57.95, 74.92)
2026	Both	1346412.61 (1130351.84, 1562473.37)	66.31 (55.64, 76.97)
2027	Both	1358747.61 (1089824.41, 1627670.81)	66.2 (53.07, 79.32)
2028	Both	1370753.54 (1042967.04, 1698540.04)	66.11 (50.28, 81.94)
2029	Both	1382751.65 (990382.87, 1775120.43)	66.06 (47.29, 84.83)
2030	Both	1394945.17 (932349.01, 1857541.33)	66.06 (44.13, 87.98)
2031	Both	1407533.09 (868952.19, 1946113.99)	66.09 (40.78, 91.41)
2032	Both	1420657.42 (800090.57, 2041224.28)	66.18 (37.25, 95.12)
2033	Both	1434113.64 (725420.02, 2142807.26)	66.33 (33.52, 99.13)
2034	Both	1448305.44 (644821.99, 2251788.88)	66.54 (29.6, 103.48)
2035	Both	1463534.82 (557939.43, 2369130.21)	66.82 (25.45, 108.2)
2036	Both	1480057.15 (464196.02, 2495918.28)	67.18 (21.04, 113.33)
2037	Both	1498077.24 (362797.5, 2633356.97)	67.63 (16.35, 118.91)
2038	Both	1517318.48 (252719.74, 2781917.23)	68.16 (11.32, 125.01)
2039	Both	1538324.12 (133088.02, 2943707.79)	68.8 (5.94, 131.69)
2040	Both	1561298.1 (29055.55, 3120530.01)	69.56 (1.32, 139.06)
2041	Both	1586365.63 (0, 3314347.06)	70.43 (0, 147.18)
2042	Both	1613587.17 (0, 3527323.61)	71.43 (0, 156.19)
2043	Both	1642817.85 (0, 3761460.25)	72.58 (0, 166.22)
2044	Both	1674716.9 (0, 4021002.35)	73.89 (0, 177.45)
2045	Both	1709565.18 (0, 4310023.62)	75.38 (0, 190.08)
2022	Female	711956.78 (661267.55, 762646.01)	72.52 (70.03, 75.01)
2023	Female	720422.72 (659556.25, 781289.19)	72.28 (68.18, 76.39)
2024	Female	728767.59 (651613.92, 805921.27)	72.03 (65.88, 78.17)

2025	Female	736977.08 (638208.47, 835745.69)	71.77 (63.27, 80.26)
2026	Female	745032.4 (620151.1, 869913.69)	71.49 (60.4, 82.59)
2027	Female	752869.12 (597955.91, 907782.32)	71.21 (57.29, 85.12)
2028	Female	760545.32 (572049.25, 949041.4)	70.91 (53.98, 87.84)
2029	Female	768247.81 (542772.19, 993723.44)	70.6 (50.48, 90.71)
2030	Female	776078.8 (510262.65, 1041894.95)	70.28 (46.82, 93.74)
2031	Female	784129.77 (474542.28, 1093717.25)	69.95 (43, 96.9)
2032	Female	792459.78 (435527.02, 1149392.55)	69.61 (39.05, 100.17)
2033	Female	801013.31 (393039.58, 1208987.04)	69.26 (34.96, 103.56)
2034	Female	810025.05 (346982.28, 1273067.83)	68.91 (30.76, 107.05)
2035	Female	819632.67 (297099.08, 1342166.26)	68.55 (26.45, 110.64)
2036	Female	829946.66 (243011.22, 1416882.1)	68.18 (22.05, 114.31)
2037	Female	841042.14 (184217.81, 1497866.47)	67.8 (17.55, 118.05)
2038	Female	852843.07 (120118.55, 1585567.59)	67.42 (12.97, 121.87)
2039	Female	865666.36 (50208.8, 1681271.47)	67.03 (8.31, 125.76)
2040	Female	879616.11 (0, 1786063.41)	66.64 (3.58, 129.71)
2041	Female	894751.34 (0, 1901132.12)	66.25 (-1.21, 133.71)
2042	Female	911082.48 (0, 2027769.59)	65.85 (-6.06, 137.75)
2043	Female	928607.56 (0, 2167422.16)	65.44 (-10.96, 141.84)
2044	Female	947700.03 (0, 2322702.99)	65.03 (-15.9, 145.97)
2045	Female	968511.75 (0, 2496148.3)	64.63 (-20.89, 150.14)
2022	Male	582118.08 (549360.67, 614875.5)	61.23 (59.46, 63.01)
2023	Male	587086.22 (546012.79, 628159.65)	61.08 (57.99, 64.16)
2024	Male	591958.15 (537815.64, 646100.66)	60.91 (56.19, 65.64)
2025	Male	596722.72 (525643.41, 667802.02)	60.74 (54.14, 67.35)
2026	Male	601380.21 (510200.73, 692559.69)	60.57 (51.89, 69.26)

2027	Male	605878.49 (491868.5, 719888.49)	60.4 (49.44, 71.35)
2028	Male	610208.22 (470917.79, 749498.64)	60.21 (46.83, 73.59)
2029	Male	614503.84 (447610.69, 781396.99)	60.02 (44.07, 75.97)
2030	Male	618866.37 (422086.37, 815646.38)	59.82 (41.17, 78.48)
2031	Male	623403.32 (394409.91, 852396.73)	59.62 (38.14, 81.11)
2032	Male	628197.64 (364563.55, 891831.73)	59.42 (34.99, 83.84)
2033	Male	633100.33 (332380.44, 933820.22)	59.2 (31.73, 86.68)
2034	Male	638280.38 (297839.71, 978721.06)	58.98 (28.36, 89.61)
2035	Male	643902.15 (260840.36, 1026963.95)	58.76 (24.9, 92.62)
2036	Male	650110.49 (221184.81, 1079036.18)	58.53 (21.34, 95.72)
2037	Male	657035.09 (178579.69, 1135490.5)	58.3 (17.7, 98.89)
2038	Male	664475.41 (132601.19, 1196349.64)	58.06 (13.98, 102.13)
2039	Male	672657.77 (82879.22, 1262436.32)	57.81 (10.19, 105.43)
2040	Male	681681.99 (29055.55, 1334466.6)	57.56 (6.33, 108.79)
2041	Male	691614.3 (0, 1413214.94)	57.3 (2.4, 112.21)
2042	Male	702504.69 (0, 1499554.02)	57.04 (-1.58, 115.67)
2043	Male	714210.28 (0, 1594038.1)	56.77 (-5.62, 119.17)
2044	Male	727016.86 (0, 1698299.36)	56.5 (-9.71, 122.71)
2045	Male	741053.43 (0, 1813875.32)	56.22 (-13.85, 126.3)

Notes: ASR: age-standardized rates; YLDs: years lived with disability.

Table S107. Prevalence and YLDs of inflammatory bowel disease in China of future forecasts using bayesian age-period-cohort model.

Year	Sex	Number	ASR
**Prevalence**			
2022	Both	178660.17 (156625.46, 200694.89)	9.49 (8.31, 10.67)
2023	Both	180710.78 (151266.57, 210155)	9.53 (7.97, 11.09)
2024	Both	182912.54 (142047.68, 223777.4)	9.57 (7.43, 11.72)
2025	Both	185383.39 (129777.07, 240989.78)	9.64 (6.74, 12.54)
2026	Both	188256.35 (114875.8, 261637.21)	9.73 (5.93, 13.53)
2027	Both	191681.88 (97404.26, 285960.32)	9.85 (5, 14.69)
2028	Both	195734.25 (77101.8, 314368.11)	10 (3.94, 16.06)
2029	Both	200577.79 (53491.58, 347666.1)	10.2 (2.72, 17.68)
2030	Both	206405.21 (25789.88, 387023.46)	10.45 (1.31, 19.59)
2031	Both	213447.51 (3810.72, 434085.31)	10.76 (0.19, 21.87)
2032	Both	221992.65 (0, 491165.2)	11.14 (0, 24.65)
2033	Both	232227.66 (0, 561099.62)	11.62 (0, 28.06)
2034	Both	244558.29 (0, 648270.81)	12.2 (0, 32.32)
2035	Both	259484.23 (0, 758930.71)	12.91 (0, 37.74)
2036	Both	277643.23 (0, 902170.54)	13.78 (0, 44.75)
2037	Both	299878.06 (0, 1091480.67)	14.85 (0, 54.01)
2038	Both	326985.01 (0, 1345835.09)	16.17 (0, 66.49)
2039	Both	360365.48 (0, 1695228.19)	17.81 (0, 83.65)
2040	Both	401871.43 (0, 2185744.22)	19.85 (0, 107.77)
2041	Both	454027.18 (0, 2889355.63)	22.42 (0, 142.37)
2042	Both	520362.18 (0, 3920276.03)	25.69 (0, 193.03)
2043	Both	605205.06 (0, 5456203.12)	29.89 (0, 268.69)

2044	Both	715331.1 (0, 7788677.51)	35.37 (0, 383.79)
2045	Both	860449.6 (0, 11394835.27)	42.6 (0, 562.07)
2022	Female	92395.67 (80325.72, 104465.62)	9.86 (9.12, 10.59)
2023	Female	93538.67 (77434.39, 109642.94)	9.88 (8.56, 11.21)
2024	Female	94775.15 (72428.1, 117122.2)	9.91 (7.84, 11.97)
2025	Female	96172.87 (65736.27, 126609.55)	9.93 (7.01, 12.84)
2026	Female	97808.78 (57575.25, 138042.61)	9.94 (6.07, 13.82)
2027	Female	99770.1 (47955.91, 151584.83)	9.96 (5.04, 14.88)
2028	Female	102105.93 (36704.01, 167508.69)	9.97 (3.93, 16.01)
2029	Female	104912.6 (23507.34, 186319.06)	9.98 (2.74, 17.22)
2030	Female	108305.76 (7861.57, 208751.55)	9.98 (1.47, 18.5)
2031	Female	112425.37 (0, 235847.46)	9.98 (0.14, 19.83)
2032	Female	117446.51 (0, 269082.03)	9.98 (-1.26, 21.23)
2033	Female	123498.43 (0, 310323.68)	9.98 (-2.72, 22.68)
2034	Female	130831.55 (0, 362440.93)	9.97 (-4.23, 24.17)
2035	Female	139761.75 (0, 429591.65)	9.96 (-5.79, 25.72)
2036	Female	150698.23 (0, 517913.19)	9.95 (-7.4, 27.3)
2037	Female	164187.03 (0, 636644.56)	9.93 (-9.06, 28.93)
2038	Female	180783.6 (0, 799112.15)	9.92 (-10.76, 30.59)
2039	Female	201418.6 (0, 1026520.46)	9.89 (-12.5, 32.29)
2040	Female	227345.11 (0, 1351957.03)	9.87 (-14.27, 34.02)
2041	Female	260295.47 (0, 1827855.32)	9.84 (-16.08, 35.77)
2042	Female	302722.22 (0, 2538599.2)	9.82 (-17.92, 37.55)
2043	Female	357749.87 (0, 3617841.26)	9.78 (-19.79, 39.35)
2044	Female	430234.53 (0, 5287320.28)	9.75 (-21.68, 41.18)
2045	Female	527247.7 (0, 7914920.07)	9.71 (-23.59, 43.02)

2022	Male	86264.5 (76299.74, 96229.26)	9.1 (8.5, 9.7)
2023	Male	87172.11 (73832.17, 100512.05)	9.12 (8.03, 10.21)
2024	Male	88137.39 (69619.58, 106655.2)	9.14 (7.44, 10.84)
2025	Male	89210.52 (64040.8, 114380.23)	9.15 (6.75, 11.55)
2026	Male	90447.57 (57300.55, 123594.6)	9.16 (5.97, 12.35)
2027	Male	91911.78 (49448.35, 134375.48)	9.17 (5.12, 13.22)
2028	Male	93628.32 (40397.79, 146859.42)	9.18 (4.21, 14.15)
2029	Male	95665.18 (29984.23, 161347.04)	9.18 (3.23, 15.13)
2030	Male	98099.46 (17928.31, 178271.9)	9.18 (2.19, 16.17)
2031	Male	101022.14 (3810.72, 198237.84)	9.18 (1.09, 17.26)
2032	Male	104546.14 (0, 222083.17)	9.17 (-0.06, 18.4)
2033	Male	108729.23 (0, 250775.94)	9.16 (-1.25, 19.58)
2034	Male	113726.74 (0, 285829.89)	9.15 (-2.49, 20.79)
2035	Male	119722.49 (0, 329339.06)	9.14 (-3.77, 22.05)
2036	Male	126945.01 (0, 384257.34)	9.12 (-5.09, 23.33)
2037	Male	135691.02 (0, 454836.11)	9.1 (-6.44, 24.65)
2038	Male	146201.41 (0, 546722.94)	9.08 (-7.83, 26)
2039	Male	158946.88 (0, 668707.73)	9.06 (-9.25, 27.37)
2040	Male	174526.32 (0, 833787.19)	9.03 (-10.7, 28.77)
2041	Male	193731.71 (0, 1061500.31)	9 (-12.17, 30.18)
2042	Male	217639.96 (0, 1381676.83)	8.97 (-13.67, 31.62)
2043	Male	247455.19 (0, 1838361.86)	8.94 (-15.19, 33.08)
2044	Male	285096.57 (0, 2501357.23)	8.91 (-16.73, 34.55)
2045	Male	333201.9 (0, 3479915.2)	8.87 (-18.29, 36.03)
**YLDs**			
2022	Both	28137.13 (24910.04, 31366.66)	1.5 (1.32, 1.67)

2023	Both	28474.1 (23905.26, 33045.29)	1.5 (1.26, 1.75)
2024	Both	28842.37 (22277.03, 35409.89)	1.51 (1.16, 1.86)
2025	Both	29262.21 (20164.17, 38362.3)	1.52 (1.05, 2)
2026	Both	29756.22 (17620.88, 41893.63)	1.54 (0.91, 2.17)
2027	Both	30351.19 (14641.03, 46063.56)	1.56 (0.75, 2.37)
2028	Both	31064.19 (11165.22, 50965.64)	1.59 (0.57, 2.61)
2029	Both	31926.57 (7091.94, 56764.43)	1.62 (0.36, 2.89)
2030	Both	32973.49 (2268.91, 63693.34)	1.67 (0.11, 3.22)
2031	Both	34247.37 (0, 72082.04)	1.73 (0, 3.63)
2032	Both	35802.98 (0, 82402.33)	1.8 (0, 4.13)
2033	Both	37683.68 (0, 95263.32)	1.88 (0, 4.76)
2034	Both	39971.68 (0, 111606.2)	1.99 (0, 5.56)
2035	Both	42768.59 (0, 132802.23)	2.13 (0, 6.6)
2036	Both	46206.12 (0, 160896.41)	2.29 (0, 7.97)
2037	Both	50462.97 (0, 199012.53)	2.49 (0, 9.83)
2038	Both	55726.7 (0, 251745.06)	2.75 (0, 12.41)
2039	Both	62310.57 (0, 326508.74)	3.07 (0, 16.07)
2040	Both	70638.17 (0, 435066.55)	3.48 (0, 21.39)
2041	Both	81300.97 (0, 596404.73)	4 (0, 29.3)
2042	Both	95149.42 (0, 841711.95)	4.68 (0, 41.3)
2043	Both	113299.91 (0, 1221657.61)	5.57 (0, 59.93)
2044	Both	137498.69 (0, 1822150.95)	6.77 (0, 89.4)
2045	Both	170329.16 (0, 2789209.52)	8.39 (0, 136.92)
2022	Female	14743.65 (12970.2, 16518.09)	1.58 (1.46, 1.69)
2023	Female	14924.97 (12405.43, 17445.52)	1.58 (1.36, 1.8)
2024	Female	15125.63 (11494.14, 18758.12)	1.58 (1.24, 1.93)

2025	Female	15357.5 (10312.5, 20403.52)	1.59 (1.09, 2.08)
2026	Female	15633.97 (8888.08, 22380.9)	1.59 (0.93, 2.24)
2027	Female	15970.85 (7213.1, 24729.65)	1.59 (0.76, 2.42)
2028	Female	16379.29 (5248.15, 27511.51)	1.59 (0.57, 2.62)
2029	Female	16878.02 (2926.46, 30831.01)	1.59 (0.36, 2.82)
2030	Female	17488.47 (151.62, 34837.33)	1.59 (0.14, 3.04)
2031	Female	18236.76 (0, 39742.83)	1.59 (-0.08, 3.27)
2032	Female	19156.74 (0, 45853.99)	1.59 (-0.32, 3.51)
2033	Female	20277.82 (0, 53578.15)	1.59 (-0.57, 3.75)
2034	Female	21652.04 (0, 63545.13)	1.59 (-0.83, 4.01)
2035	Female	23345.24 (0, 76688.52)	1.59 (-1.1, 4.27)
2036	Female	25444.13 (0, 94423.31)	1.58 (-1.37, 4.54)
2037	Female	28067.46 (0, 118944.32)	1.58 (-1.65, 4.81)
2038	Female	31346.99 (0, 153555.28)	1.58 (-1.94, 5.1)
2039	Female	35496.35 (0, 203640.57)	1.57 (-2.24, 5.38)
2040	Female	40810.22 (0, 277883.21)	1.57 (-2.54, 5.68)
2041	Female	47706.38 (0, 390515.8)	1.56 (-2.85, 5.97)
2042	Female	56793.64 (0, 565266.99)	1.56 (-3.16, 6.28)
2043	Female	68896.8 (0, 841361.76)	1.55 (-3.47, 6.58)
2044	Female	85306.72 (0, 1286149.1)	1.55 (-3.79, 6.89)
2045	Female	107965.28 (0, 2015727.41)	1.54 (-4.12, 7.2)
2022	Male	13393.48 (11939.84, 14848.58)	1.41 (1.32, 1.51)
2023	Male	13549.13 (11499.83, 15599.77)	1.42 (1.25, 1.59)
2024	Male	13716.75 (10782.89, 16651.77)	1.42 (1.15, 1.7)
2025	Male	13904.71 (9851.67, 17958.78)	1.43 (1.04, 1.82)
2026	Male	14122.25 (8732.8, 19512.73)	1.43 (0.91, 1.95)

2027	Male	14380.34 (7427.93, 21333.91)	1.43 (0.77, 2.1)
2028	Male	14684.9 (5917.06, 23454.14)	1.43 (0.62, 2.25)
2029	Male	15048.55 (4165.47, 25933.42)	1.44 (0.46, 2.42)
2030	Male	15485.03 (2117.3, 28856.01)	1.44 (0.28, 2.59)
2031	Male	16010.61 (0, 32339.21)	1.44 (0.1, 2.77)
2032	Male	16646.25 (0, 36548.34)	1.44 (-0.09, 2.97)
2033	Male	17405.86 (0, 41685.17)	1.44 (-0.29, 3.16)
2034	Male	18319.65 (0, 48061.07)	1.44 (-0.49, 3.37)
2035	Male	19423.35 (0, 56113.71)	1.44 (-0.71, 3.58)
2036	Male	20761.99 (0, 66473.1)	1.43 (-0.93, 3.8)
2037	Male	22395.51 (0, 80068.21)	1.43 (-1.15, 4.02)
2038	Male	24379.71 (0, 98189.79)	1.43 (-1.39, 4.25)
2039	Male	26814.22 (0, 122868.17)	1.43 (-1.62, 4.48)
2040	Male	29827.96 (0, 157183.33)	1.42 (-1.87, 4.72)
2041	Male	33594.59 (0, 205888.93)	1.42 (-2.11, 4.95)
2042	Male	38355.78 (0, 276444.95)	1.42 (-2.37, 5.2)
2043	Male	44403.11 (0, 380295.85)	1.41 (-2.62, 5.44)
2044	Male	52191.98 (0, 536001.86)	1.41 (-2.88, 5.69)
2045	Male	62363.88 (0, 773482.12)	1.4 (-3.14, 5.94)

Notes: ASR: age-standardized rates; YLDs: years lived with disability.

Table S108. Prevalence and YLDs of pancreatitis in China of future forecasts using bayesian age-period-cohort model.

Year	Sex	Number	ASR
**Prevalence**			
2022	Both	509683.66 (448453.36, 570913.96)	24.34 (21.4, 27.28)
2023	Both	521863.7 (432253.44, 611473.95)	24.44 (20.23, 28.65)
2024	Both	535289.66 (402520.84, 668058.48)	24.58 (18.47, 30.69)
2025	Both	550355.02 (361733.71, 738976.32)	24.8 (16.29, 33.3)
2026	Both	567415.74 (310375.23, 824456.24)	25.1 (13.73, 36.48)
2027	Both	586907.57 (247543.14, 926272)	25.52 (10.76, 40.29)
2028	Both	609676.08 (171259.36, 1048092.8)	26.08 (7.32, 44.84)
2029	Both	636879.57 (78047.25, 1195711.9)	26.82 (3.29, 50.35)
2030	Both	669514.13 (0, 1376730.02)	27.77 (0, 57.09)
2031	Both	708695.24 (0, 1601753.16)	28.97 (0, 65.48)
2032	Both	755866.39 (0, 1886127.94)	30.49 (0, 76.08)
2033	Both	813252.54 (0, 2253321.36)	32.41 (0, 89.76)
2034	Both	883938.3 (0, 2739216.91)	34.81 (0, 107.82)
2035	Both	971553.59 (0, 3397513.26)	37.83 (0, 132.22)
2036	Both	1080762.97 (0, 4311223.85)	41.65 (0, 166.02)
2037	Both	1217926.05 (0, 5612210.6)	46.5 (0, 214.06)
2038	Both	1392455.83 (0, 7517066.35)	52.72 (0, 284.2)
2039	Both	1617963.38 (0, 10386614.94)	60.77 (0, 389.42)
2040	Both	1913166.27 (0, 14823833.32)	71.33 (0, 551.52)
2041	Both	2304899.19 (0, 21854682.29)	85.39 (0, 807.57)
2042	Both	2832802.11 (0, 33253570.07)	104.39 (0, 1221.76)
2043	Both	3557806.18 (0, 52151669.8)	130.56 (0, 1907.17)

2044	Both	4575192.22 (0, 84167996.97)	167.3 (0, 3065.95)
2045	Both	6032957.04 (0, 139469027.58)	220.01 (0, 5064.9)
2022	Female	203310.32 (177704.06, 228916.58)	19.14 (17.65, 20.64)
2023	Female	208716.99 (171430.78, 246003.19)	19.17 (16.33, 22.01)
2024	Female	214701.1 (159524.73, 269877.46)	19.19 (14.67, 23.72)
2025	Female	221426.34 (142937.43, 299915.25)	19.21 (12.74, 25.69)
2026	Female	229047.58 (121806.26, 336288.89)	19.23 (10.59, 27.87)
2027	Female	237757.08 (95673.03, 379841.12)	19.24 (8.22, 30.26)
2028	Female	247943.04 (63597.62, 432288.46)	19.24 (5.67, 32.82)
2029	Female	260130.45 (23939.98, 496320.92)	19.25 (2.94, 35.55)
2030	Female	274768.46 (0, 575475.73)	19.24 (0.06, 38.43)
2031	Female	292373.68 (0, 674744.51)	19.24 (-2.98, 41.47)
2032	Female	313616.33 (0, 801408.07)	19.23 (-6.17, 44.63)
2033	Female	339556.38 (0, 966771.09)	19.22 (-9.49, 47.92)
2034	Female	371655.79 (0, 1188283.91)	19.21 (-12.93, 51.34)
2035	Female	411637.74 (0, 1492355.68)	19.19 (-16.5, 54.88)
2036	Female	461757.26 (0, 1920422.14)	19.17 (-20.18, 58.53)
2037	Female	525116.64 (0, 2539191.85)	19.16 (-23.98, 62.29)
2038	Female	606395.56 (0, 3459992.42)	19.13 (-27.88, 66.15)
2039	Female	712401.74 (0, 4870849.85)	19.11 (-31.89, 70.12)
2040	Female	852583.15 (0, 7090343.26)	19.1 (-36, 74.19)
2041	Female	1040677.25 (0, 10668425.63)	19.08 (-40.21, 78.37)
2042	Female	1297224.74 (0, 16569656.48)	19.06 (-44.52, 82.64)
2043	Female	1654374.16 (0, 26522799.71)	19.04 (-48.92, 87.01)
2044	Female	2162980.02 (0, 43674962.43)	19.03 (-53.42, 91.48)
2045	Female	2903145.42 (0, 73801871.76)	19.02 (-58.02, 96.07)

2022	Male	306373.35 (270749.31, 341997.38)	29.52 (27.35, 31.69)
2023	Male	313146.71 (260822.66, 365470.76)	29.6 (25.45, 33.76)
2024	Male	320588.57 (242996.11, 398181.02)	29.68 (23.05, 36.31)
2025	Male	328928.68 (218796.29, 439061.08)	29.75 (20.25, 39.25)
2026	Male	338368.16 (188568.96, 488167.35)	29.81 (17.1, 42.52)
2027	Male	349150.5 (151870.11, 546430.88)	29.86 (13.64, 46.09)
2028	Male	361733.04 (107661.74, 615804.34)	29.91 (9.89, 49.92)
2029	Male	376749.12 (54107.27, 699390.98)	29.94 (5.87, 54.02)
2030	Male	394745.66 (0, 801254.28)	29.97 (1.6, 58.35)
2031	Male	416321.56 (0, 927008.65)	30 (-2.9, 62.9)
2032	Male	442250.06 (0, 1084719.87)	30.01 (-7.63, 67.65)
2033	Male	473696.15 (0, 1286550.27)	30.02 (-12.56, 72.59)
2034	Male	512282.51 (0, 1550933)	30.02 (-17.69, 77.73)
2035	Male	559915.85 (0, 1905157.58)	30.02 (-23.01, 83.04)
2036	Male	619005.71 (0, 2390801.71)	30.01 (-28.5, 88.53)
2037	Male	692809.41 (0, 3073018.75)	30 (-34.17, 94.16)
2038	Male	786060.27 (0, 4057073.94)	29.98 (-39.99, 99.95)
2039	Male	905561.64 (0, 5515765.09)	29.96 (-45.98, 105.89)
2040	Male	1060583.11 (0, 7733490.06)	29.94 (-52.11, 111.98)
2041	Male	1264221.94 (0, 11186256.66)	29.91 (-58.4, 118.22)
2042	Male	1535577.37 (0, 16683913.59)	29.89 (-64.82, 124.59)
2043	Male	1903432.02 (0, 25628870.09)	29.86 (-71.37, 131.09)
2044	Male	2412212.2 (0, 40493034.54)	29.83 (-78.07, 137.73)
2045	Male	3129811.62 (0, 65667155.83)	29.81 (-84.91, 144.52)
**YLDs**			
2022	Both	37727.72 (34317.52, 41137.93)	1.85 (1.67, 2.03)

2023	Both	38424.43 (33269.6, 43579.25)	1.85 (1.59, 2.11)
2024	Both	39174.28 (31479.57, 46868.99)	1.85 (1.48, 2.23)
2025	Both	39993.73 (29100.97, 50886.49)	1.86 (1.35, 2.37)
2026	Both	40894.88 (26179.15, 55610.6)	1.87 (1.19, 2.55)
2027	Both	41893.36 (22701.38, 61085.34)	1.89 (1.02, 2.76)
2028	Both	43025.89 (18621.83, 67429.95)	1.91 (0.82, 3)
2029	Both	44343.57 (13848.42, 74838.72)	1.94 (0.6, 3.29)
2030	Both	45880.72 (8220.58, 83540.86)	1.99 (0.35, 3.62)
2031	Both	47670.67 (1700.79, 93834.68)	2.04 (0.07, 4.01)
2032	Both	49756.82 (0, 106130.44)	2.1 (0, 4.49)
2033	Both	52212.25 (0, 121031.84)	2.18 (0, 5.06)
2034	Both	55136.88 (0, 139405.09)	2.28 (0, 5.77)
2035	Both	58626.97 (0, 162399.93)	2.4 (0, 6.65)
2036	Both	62792.87 (0, 191616.68)	2.55 (0, 7.77)
2037	Both	67778.17 (0, 229364.75)	2.73 (0, 9.22)
2038	Both	73794.26 (0, 279111.53)	2.95 (0, 11.14)
2039	Both	81135.75 (0, 346107.88)	3.22 (0, 13.71)
2040	Both	90148.42 (0, 438121.09)	3.55 (0, 17.23)
2041	Both	101276.29 (0, 566926.67)	3.97 (0, 22.15)
2042	Both	115121.72 (0, 750721.55)	4.49 (0, 29.18)
2043	Both	132533.9 (0, 1018135.73)	5.15 (0, 39.39)
2044	Both	154748.48 (0, 1415071.48)	6 (0, 54.52)
2045	Both	183432.62 (0, 2014715.2)	7.09 (0, 77.37)
2022	Female	15762.1 (14188.36, 17335.85)	1.51 (1.42, 1.61)
2023	Female	16115.06 (13798.13, 18431.98)	1.51 (1.33, 1.69)
2024	Female	16496.7 (13076.86, 19916.54)	1.51 (1.23, 1.8)

2025	Female	16913.72 (12086.93, 21740.5)	1.51 (1.1, 1.92)
2026	Female	17371.27 (10845.4, 23897.15)	1.51 (0.96, 2.06)
2027	Female	17876.27 (9342.67, 26409.86)	1.51 (0.81, 2.21)
2028	Female	18447.17 (7553.67, 29340.67)	1.51 (0.65, 2.37)
2029	Female	19109.07 (5429.71, 32788.43)	1.51 (0.48, 2.54)
2030	Female	19877.93 (2887.41, 36868.46)	1.51 (0.3, 2.72)
2031	Female	20769.95 (0, 41732.53)	1.5 (0.1, 2.91)
2032	Female	21806.08 (0, 47589.82)	1.5 (-0.1, 3.1)
2033	Female	23023.93 (0, 54754.54)	1.5 (-0.3, 3.3)
2034	Female	24474.48 (0, 63680.63)	1.5 (-0.52, 3.52)
2035	Female	26206.58 (0, 74975.86)	1.5 (-0.74, 3.73)
2036	Female	28277.75 (0, 89499.39)	1.49 (-0.97, 3.96)
2037	Female	30763.01 (0, 108504.91)	1.49 (-1.21, 4.19)
2038	Female	33776.37 (0, 133908.82)	1.49 (-1.45, 4.43)
2039	Female	37474.99 (0, 168641.74)	1.48 (-1.7, 4.67)
2040	Female	42044.86 (0, 217095.17)	1.48 (-1.96, 4.92)
2041	Female	47728.53 (0, 286018.54)	1.48 (-2.22, 5.18)
2042	Female	54857.12 (0, 385965)	1.48 (-2.48, 5.44)
2043	Female	63913.82 (0, 533830.17)	1.47 (-2.76, 5.7)
2044	Female	75597.04 (0, 756985.09)	1.47 (-3.03, 5.97)
2045	Female	90859.1 (0, 1099603.15)	1.47 (-3.31, 6.25)
2022	Male	21965.62 (20129.16, 23802.08)	2.18 (2.06, 2.29)
2023	Male	22309.37 (19471.47, 25147.28)	2.17 (1.94, 2.41)
2024	Male	22677.58 (18402.71, 26952.46)	2.17 (1.79, 2.56)
2025	Male	23080.02 (17014.04, 29145.99)	2.17 (1.62, 2.72)
2026	Male	23523.61 (15333.75, 31713.46)	2.17 (1.44, 2.91)

2027	Male	24017.09 (13358.71, 34675.47)	2.17 (1.23, 3.11)
2028	Male	24578.72 (11068.16, 38089.28)	2.17 (1.01, 3.32)
2029	Male	25234.5 (8418.71, 42050.29)	2.17 (0.78, 3.55)
2030	Male	26002.79 (5333.17, 46672.4)	2.16 (0.53, 3.79)
2031	Male	26900.72 (1700.79, 52102.15)	2.16 (0.27, 4.05)
2032	Male	27950.75 (0, 58540.62)	2.16 (0, 4.31)
2033	Male	29188.32 (0, 66277.3)	2.15 (-0.28, 4.59)
2034	Male	30662.4 (0, 75724.46)	2.15 (-0.57, 4.87)
2035	Male	32420.4 (0, 87424.07)	2.15 (-0.88, 5.17)
2036	Male	34515.11 (0, 102117.28)	2.14 (-1.19, 5.47)
2037	Male	37015.16 (0, 120859.84)	2.14 (-1.51, 5.78)
2038	Male	40017.89 (0, 145202.7)	2.13 (-1.84, 6.1)
2039	Male	43660.76 (0, 177466.13)	2.13 (-2.17, 6.43)
2040	Male	48103.56 (0, 221025.92)	2.12 (-2.52, 6.77)
2041	Male	53547.76 (0, 280908.14)	2.12 (-2.87, 7.11)
2042	Male	60264.6 (0, 364756.55)	2.11 (-3.23, 7.46)
2043	Male	68620.08 (0, 484305.56)	2.11 (-3.6, 7.82)
2044	Male	79151.45 (0, 658086.39)	2.11 (-3.97, 8.18)
2045	Male	92573.52 (0, 915112.05)	2.1 (-4.35, 8.55)

Notes: ASR: age-standardized rates; YLDs: years lived with disability.

Table S109. Prevalence and YLDs of male infertility in China of future forecasts using bayesian age-period-cohort model.

Year	Sex	Number	ASR
**Prevalence**			
2022	Male	12124868.54 (11579476.16, 12670260.93)	1561.72 (1517.45, 1606)
2023	Male	11991637.52 (11347108.09, 12636166.95)	1553.02 (1489.9, 1616.14)
2024	Male	11864186.58 (11072116.99, 12656256.18)	1544.62 (1457.27, 1631.96)
2025	Male	11726353.17 (10749053.55, 12703652.8)	1536.29 (1420.82, 1651.75)
2026	Male	11562087.83 (10372019.21, 12752156.45)	1527.65 (1380.89, 1674.4)
2027	Male	11357614.75 (9936284.33, 12778945.17)	1518.51 (1337.71, 1699.31)
2028	Male	11103275.13 (9443020.3, 12763529.96)	1509.45 (1292.34, 1726.55)
2029	Male	10810112.83 (8909408.9, 12710816.76)	1500.65 (1245.29, 1756.02)
2030	Male	10498709.72 (8357752.19, 12639667.24)	1491.93 (1196.52, 1787.33)
2031	Male	10189062.78 (7806602.73, 12571522.84)	1482.89 (1145.83, 1819.96)
2032	Male	9903435.63 (7273311.94, 12533559.31)	1473.47 (1093.18, 1853.76)
2033	Male	9624445.32 (6746418.93, 12502471.7)	1464.06 (1039.14, 1888.99)
2034	Male	9339841.23 (6218716.45, 12460966.01)	1454.82 (983.96, 1925.69)
2035	Male	9070198.2 (5704909.1, 12435487.31)	1445.52 (927.56, 1963.48)
2036	Male	8835507.83 (5215665.87, 12455349.8)	1435.73 (869.67, 2001.8)
2037	Male	8663027.27 (4761851.82, 12564202.72)	1425.51 (810.35, 2040.67)
2038	Male	8516222.86 (4319762.37, 12712683.35)	1415.19 (749.96, 2080.42)
2039	Male	8395103.47 (3886150.14, 12904056.8)	1404.95 (688.67, 2121.24)
2040	Male	8298459.31 (3457418.24, 13139500.39)	1394.7 (626.45, 2162.94)
2041	Male	8223974.36 (3029126.32, 13418822.4)	1384.11 (563.17, 2205.05)
2042	Male	8175646.38 (2598952.46, 13752340.3)	1373.3 (498.86, 2247.75)
2043	Male	8126408.85 (2152893.02, 14099924.67)	1362.45 (433.68, 2291.22)
2044	Male	8091182.44 (1696496.57, 14487925.77)	1351.69 (367.75, 2335.63)

2045	Male	8070273.14 (1227180.35, 14919838.03)	1341.03 (301.08, 2380.98)
**YLDs**			
2022	Male	66073.33 (63395.89, 68750.78)	8.58 (8.37, 8.78)
2023	Male	65501.29 (62279.2, 68723.38)	8.55 (8.24, 8.86)
2024	Male	64958.15 (60949.6, 68966.69)	8.53 (8.09, 8.97)
2025	Male	64360.95 (59378.78, 69343.11)	8.5 (7.92, 9.09)
2026	Male	63629.83 (57535.07, 69724.58)	8.48 (7.73, 9.23)
2027	Male	62687.44 (55384.58, 69990.31)	8.45 (7.52, 9.38)
2028	Male	61464.58 (52910.95, 70018.22)	8.43 (7.31, 9.55)
2029	Male	60017.16 (50200.04, 69834.27)	8.4 (7.08, 9.72)
2030	Male	58461.42 (47375.7, 69547.13)	8.37 (6.84, 9.91)
2031	Male	56914.98 (44546.95, 69283.01)	8.34 (6.59, 10.1)
2032	Male	55498.25 (41810.39, 69186.1)	8.32 (6.33, 10.3)
2033	Male	54098.1 (39086.81, 69109.4)	8.29 (6.06, 10.51)
2034	Male	52649.83 (36336.54, 68963.12)	8.26 (5.78, 10.73)
2035	Male	51278.22 (33651.76, 68904.69)	8.23 (5.49, 10.96)
2036	Male	50109.72 (31106.56, 69112.87)	8.2 (5.2, 11.19)
2037	Male	49301.53 (28772.33, 69830.73)	8.17 (4.9, 11.43)
2038	Male	48611.86 (26490.75, 70732.97)	8.13 (4.59, 11.68)
2039	Male	48050.22 (24251.11, 71849.33)	8.1 (4.27, 11.93)
2040	Male	47614.36 (22038.58, 73190.13)	8.07 (3.94, 12.19)
2041	Male	47299.8 (19835.61, 74763.99)	8.03 (3.61, 12.46)
2042	Male	47125 (17633.55, 76616.44)	8 (3.27, 12.73)
2043	Male	46940.23 (15350.1, 78530.35)	7.96 (2.93, 13)
2044	Male	46822.91 (13010.3, 80635.52)	7.93 (2.57, 13.28)
2045	Male	46771.89 (10602.08, 82941.7)	7.89 (2.22, 13.57)

Notes: ASR: age-standardized rates; YLDs: years lived with disability.

Table S110. Prevalence and YLDs of female infertility in China of future forecasts using bayesian age-period-cohort model.

Year	Sex	Number	ASR
**Prevalence**			
2022	Female	30563580.17 (25532813.45, 35594346.88)	3948.77 (3579.49, 4318.05)
2023	Female	30153484.4 (24937103.48, 35369865.33)	3901.08 (3483.23, 4318.93)
2024	Female	29742081.29 (24239268.76, 35244893.81)	3850.1 (3368.19, 4332.01)
2025	Female	29269136.04 (23388420.38, 35149851.7)	3795.33 (3236.68, 4353.97)
2026	Female	28675713.26 (22344396.09, 35007030.42)	3735.77 (3090.52, 4381.02)
2027	Female	27907943.95 (21083824.38, 34732063.53)	3671.04 (2931.96, 4410.12)
2028	Female	26931028.86 (19614567.4, 34247490.31)	3602.79 (2765.36, 4440.23)
2029	Female	25786258.23 (17999856.45, 33572660.01)	3530.88 (2592.61, 4469.15)
2030	Female	24540999.18 (16313356.43, 32768641.93)	3454 (2414.2, 4493.79)
2031	Female	23263020.76 (14618821.46, 31907220.06)	3370.73 (2230.36, 4511.1)
2032	Female	22025071.1 (12973795.03, 31076347.17)	3281.02 (2042.41, 4519.62)
2033	Female	20779960.68 (11362658.27, 30197263.09)	3187.24 (1853.65, 4520.82)
2034	Female	19475653.31 (9770055.96, 29181250.65)	3089.82 (1665.62, 4514.02)
2035	Female	18181792.48 (8243435.3, 28120534.71)	2987.98 (1478.84, 4497.12)
2036	Female	16970206.66 (6817057.55, 27125426.2)	2880.89 (1293.79, 4467.99)
2037	Female	15926470.53 (5511972.9, 26344554.27)	2769.53 (1111.83, 4427.24)
2038	Female	15023780.46 (4308892.66, 25743775.71)	2657.14 (935.38, 4378.91)
2039	Female	14213952.18 (3230983.75, 25259531.67)	2545.19 (765.54, 4324.85)
2040	Female	13488113.09 (2207522.01, 24889138.68)	2433.92 (602.58, 4265.26)
2041	Female	12837095.53 (1285289.56, 24630184.47)	2323.18 (446.51, 4199.86)
2042	Female	12264578.61 (442895.28, 24504725.4)	2213.99 (297.62, 4130.36)
2043	Female	11739792.06 (0, 24466817.25)	2108.27 (156.44, 4060.09)
2044	Female	11284327.96 (0, 24572575.25)	2007 (22.97, 3991.03)

2045	Female	10894870.22 (0, 24832791.71)	1910.39 (-103.12, 3923.91)
**YLDs**			
2022	Female	161541.38 (141303.84, 181778.91)	21.02 (19.5, 22.54)
2023	Female	159609.61 (138220.1, 180999.13)	20.8 (19.02, 22.59)
2024	Female	157659.86 (134551.99, 180767.73)	20.57 (18.43, 22.71)
2025	Female	155374.39 (130040.11, 180708.68)	20.31 (17.76, 22.86)
2026	Female	152439.34 (124478.38, 180400.29)	20.02 (17.01, 23.03)
2027	Female	148564.15 (117742.31, 179386)	19.7 (16.19, 23.21)
2028	Female	143560.63 (109877.24, 177244.03)	19.36 (15.33, 23.38)
2029	Female	137637.09 (101220.8, 174053.38)	18.99 (14.43, 23.55)
2030	Female	131143.66 (92167.5, 170119.82)	18.59 (13.5, 23.68)
2031	Female	124436.94 (83060.29, 165813.58)	18.15 (12.54, 23.77)
2032	Female	117900.42 (74212.92, 161587.93)	17.68 (11.54, 23.81)
2033	Female	111288.87 (65536.36, 157041.39)	17.17 (10.54, 23.81)
2034	Female	104321.49 (56933.51, 151709.46)	16.65 (9.54, 23.75)
2035	Female	97363.19 (48668.82, 146057.56)	16.09 (8.54, 23.64)
2036	Female	90795.07 (40947.83, 140648.4)	15.5 (7.55, 23.45)
2037	Female	85074.27 (33918.82, 136243.05)	14.88 (6.56, 23.2)
2038	Female	80066.31 (27489.31, 132663.49)	14.25 (5.61, 22.89)
2039	Female	75517.63 (21582.36, 129578.82)	13.61 (4.69, 22.54)
2040	Female	71381.24 (16199.57, 126962.87)	12.98 (3.8, 22.16)
2041	Female	67611.11 (11086.71, 124793.07)	12.35 (2.96, 21.74)
2042	Female	64227.25 (6678.83, 123166.81)	11.72 (2.15, 21.28)
2043	Female	61086.26 (2705.67, 121864.13)	11.11 (1.39, 20.82)
2044	Female	58307.16 (48.57, 121162.74)	10.52 (0.68, 20.36)
2045	Female	55876.53 (0, 121103.89)	9.96 (0.02, 19.9)

Notes: ASR: age-standardized rates; YLDs: years lived with disability.

Table S111. Prevalence and YLDs of depressive disorders in China of future forecasts using bayesian age-period-cohort model.

Year	Sex	Number	ASR
**Prevalence**			
2022	Both	52387546.06 (47613877.86, 57161214.25)	2759.39 (2507.88, 3010.9)
2023	Both	52639337.45 (47585379.21, 57693295.69)	2745.06 (2481.42, 3008.7)
2024	Both	52886272.77 (47391144.14, 58381401.4)	2731.29 (2447.38, 3015.2)
2025	Both	53124427.63 (47018109.7, 59230745.57)	2718.13 (2405.53, 3030.72)
2026	Both	53343768.42 (46461774.55, 60225762.28)	2705.39 (2356.13, 3054.65)
2027	Both	53545037.49 (45734773.13, 61355301.84)	2693.16 (2299.99, 3086.33)
2028	Both	53717079.48 (44843119.67, 62591039.3)	2681.8 (2238.3, 3125.31)
2029	Both	53881193.03 (43818663.28, 63943722.78)	2671.52 (2171.95, 3171.1)
2030	Both	54036763 (42671225.27, 65402300.74)	2662.37 (2101.51, 3223.24)
2031	Both	54176777.23 (41403236.34, 66950318.13)	2654.23 (2027.24, 3281.21)
2032	Both	54307598.07 (40026128.72, 68589067.43)	2647.16 (1949.49, 3344.83)
2033	Both	54398755.02 (38524042.62, 70273467.43)	2641.48 (1868.68, 3414.29)
2034	Both	54479591.57 (36923541.92, 72035641.22)	2637.37 (1785, 3489.73)
2035	Both	54551984.46 (35228200.4, 73875768.51)	2634.88 (1698.46, 3571.3)
2036	Both	54610473.7 (33435445.09, 75785502.31)	2633.94 (1608.85, 3659.04)
2037	Both	54659771.04 (31548851.23, 77770690.84)	2634.65 (1516.05, 3753.24)
2038	Both	54687384.32 (29562152.89, 79812615.76)	2637.26 (1420.03, 3854.5)
2039	Both	54713929.57 (27486517.89, 81941341.25)	2641.96 (1320.53, 3963.38)
2040	Both	54739986.98 (25319479.92, 84160494.04)	2648.81 (1217.2, 4080.42)
2041	Both	54759671.7 (23054547.14, 86464796.27)	2657.84 (1109.52, 4206.15)
2042	Both	54774727.2 (20688734.49, 88860719.92)	2669.14 (997, 4341.28)
2043	Both	54791465.49 (18220149.6, 91362781.38)	2682.98 (879.14, 4486.82)

2044	Both	54820773.14 (15645929.06, 93995618.99)	2699.52 (755.24, 4643.8)
2045	Both	54862470.59 (12957044.08, 96767920.48)	2718.91 (624.47, 4813.35)
2022	Female	32209646.14 (29012797.28, 35406495)	3393.68 (3272.02, 3515.35)
2023	Female	32319491.18 (28981428.05, 35657554.31)	3368.97 (3215.62, 3522.31)
2024	Female	32425722.3 (28869938.31, 35981506.29)	3344.85 (3149.77, 3539.93)
2025	Female	32525257.6 (28667765.31, 36382749.89)	3321.28 (3076.8, 3565.76)
2026	Female	32610722.3 (28366888.77, 36854555.83)	3297.88 (2998.01, 3597.75)
2027	Female	32683943.74 (27972505.07, 37395382.41)	3274.73 (2914.59, 3634.86)
2028	Female	32737645.71 (27485511.1, 37989780.31)	3252.18 (2827.7, 3676.67)
2029	Female	32784626.25 (26924204.27, 38645048.24)	3230.46 (2738.07, 3722.85)
2030	Female	32823904.73 (26293880.81, 39353928.66)	3209.54 (2646.05, 3773.02)
2031	Female	32849974.93 (25595404.47, 40104545.39)	3189.13 (2551.66, 3826.6)
2032	Female	32867738.82 (24837396.01, 40898081.64)	3169.3 (2455.16, 3883.43)
2033	Female	32858957.79 (24010924.28, 41706991.31)	3150.32 (2357, 3943.64)
2034	Female	32840590.6 (23132758.95, 42548422.26)	3132.35 (2257.44, 4007.25)
2035	Female	32812773.72 (22205545.61, 43420001.82)	3115.34 (2156.52, 4074.16)
2036	Female	32770785.32 (21228069.7, 44313500.95)	3099.1 (2054.16, 4144.04)
2037	Female	32718022.34 (20204524.94, 45231519.75)	3083.64 (1950.41, 4216.87)
2038	Female	32649139.05 (19133596.94, 46164681.16)	3069.17 (1845.5, 4292.85)
2039	Female	32575037.54 (18023302.19, 47126772.9)	3055.78 (1739.49, 4372.08)
2040	Female	32495296.6 (16873709.71, 48116883.49)	3043.45 (1632.36, 4454.54)
2041	Female	32405143.19 (15682394.48, 49127891.9)	3032.04 (1524.01, 4540.07)
2042	Female	32306245.19 (14450446.45, 50162043.93)	3021.56 (1414.4, 4628.72)
2043	Female	32203124.28 (13179962.22, 51226286.34)	3012.13 (1303.6, 4720.66)
2044	Female	32101239.3 (11872430.93, 52330047.67)	3003.8 (1191.59, 4816.01)
2045	Female	31999845.68 (10525838.15, 53473853.2)	2996.55 (1078.29, 4914.82)

2022	Male	20177899.91 (18601080.58, 21754719.25)	2127.48 (2060.47, 2194.48)
2023	Male	20319846.27 (18603951.16, 22035741.38)	2123.05 (2028.2, 2217.89)
2024	Male	20460550.47 (18521205.83, 22399895.11)	2118.79 (1987.76, 2249.83)
2025	Male	20599170.03 (18350344.39, 22847995.68)	2114.76 (1941.33, 2288.19)
2026	Male	20733046.12 (18094885.78, 23371206.46)	2110.83 (1889.99, 2331.66)
2027	Male	20861093.75 (17762268.06, 23959919.44)	2106.84 (1834.3, 2379.38)
2028	Male	20979433.77 (17357608.57, 24601258.98)	2103.01 (1774.96, 2431.06)
2029	Male	21096566.78 (16894459.01, 25298674.54)	2099.51 (1712.46, 2486.56)
2030	Male	21212858.27 (16377344.46, 26048372.08)	2096.4 (1647.09, 2545.72)
2031	Male	21326802.31 (15807831.87, 26845772.74)	2093.59 (1578.92, 2608.26)
2032	Male	21439859.25 (15188732.71, 27690985.79)	2090.94 (1507.98, 2673.9)
2033	Male	21539797.23 (14513118.34, 28566476.12)	2088.64 (1434.56, 2742.71)
2034	Male	21639000.97 (13790782.97, 29487218.96)	2086.82 (1358.85, 2814.79)
2035	Male	21739210.74 (13022654.79, 30455766.69)	2085.54 (1280.95, 2890.13)
2036	Male	21839688.38 (12207375.4, 31472001.36)	2084.73 (1200.83, 2968.63)
2037	Male	21941748.69 (11344326.29, 32539171.09)	2084.26 (1118.43, 3050.09)
2038	Male	22038245.28 (10428555.95, 33647934.6)	2084.3 (1033.9, 3134.69)
2039	Male	22138892.03 (9463215.7, 34814568.35)	2084.94 (947.29, 3222.58)
2040	Male	22244690.38 (8445770.21, 36043610.56)	2086.23 (858.61, 3313.84)
2041	Male	22354528.51 (7372152.66, 37336904.37)	2088.12 (767.79, 3408.44)
2042	Male	22468482.01 (6238288.03, 38698675.99)	2090.51 (674.75, 3506.27)
2043	Male	22588341.21 (5040187.38, 40136495.04)	2093.51 (579.51, 3607.51)
2044	Male	22719533.84 (3773498.14, 41665571.32)	2097.21 (482.05, 3712.37)
2045	Male	22862624.91 (2431205.93, 43294067.27)	2101.64 (382.3, 3820.98)
**YLDs**			
2022	Both	7768219.11 (6957140.82, 8579297.4)	415.04 (371.68, 458.4)

2023	Both	7764254.39 (6899311.87, 8629196.92)	410.8 (365, 456.59)
2024	Both	7759978.15 (6807731.66, 8712224.63)	406.67 (356.73, 456.62)
2025	Both	7755026.26 (6681776.35, 8828276.18)	402.67 (346.88, 458.45)
2026	Both	7748219.36 (6522890.01, 8973548.71)	398.77 (335.63, 461.91)
2027	Both	7740373.46 (6335218.93, 9145527.99)	395.01 (323.19, 466.83)
2028	Both	7729218.59 (6120466.74, 9337970.44)	391.43 (309.81, 473.05)
2029	Both	7718108.04 (5884176.93, 9552039.14)	388.07 (295.65, 480.48)
2030	Both	7707099.96 (5628378.7, 9785821.22)	384.93 (280.84, 489.01)
2031	Both	7695389.3 (5353853.53, 10036925.07)	382 (265.42, 498.57)
2032	Both	7684530.04 (5062646.4, 10306413.67)	379.3 (249.45, 509.14)
2033	Both	7669296.38 (4752119.77, 10586473)	376.88 (232.98, 520.77)
2034	Both	7654401.99 (4425868.71, 10882935.27)	374.75 (216.02, 533.48)
2035	Both	7640070.76 (4083949.19, 11196192.32)	372.94 (198.55, 547.34)
2036	Both	7625539.59 (3725568.84, 11525510.33)	371.44 (180.49, 562.38)
2037	Both	7611816.98 (3350679.93, 11872954.03)	370.26 (161.81, 578.7)
2038	Both	7597323.19 (2957888.67, 12236757.7)	369.45 (142.44, 596.46)
2039	Both	7585205.77 (2547439.85, 12622971.69)	369.04 (122.29, 615.79)
2040	Both	7575378.07 (2117660.63, 13033097.28)	369.04 (101.22, 636.86)
2041	Both	7566907.1 (1666364.07, 13467488.46)	369.46 (79.08, 659.85)
2042	Both	7560177.44 (1194832.93, 13928960.65)	370.33 (56.2, 684.97)
2043	Both	7557049.17 (953137.91, 14423568.58)	371.69 (44.69, 712.53)
2044	Both	7559034.16 (706254.16, 14957626.51)	373.58 (32.86, 742.84)
2045	Both	7566054.7 (450695.51, 15535227.18)	376.03 (20.55, 776.27)
2022	Female	4803803.51 (4261297.72, 5346309.3)	513.25 (491.79, 534.71)
2023	Female	4792625.26 (4223787.11, 5361463.42)	506.61 (478.82, 534.4)
2024	Female	4781175.83 (4170020.13, 5392331.54)	500.07 (464.02, 536.12)

2025	Female	4769094.94 (4098836.13, 5439353.75)	493.62 (447.93, 539.31)
2026	Female	4755440.23 (4009981.38, 5500899.07)	487.22 (430.87, 543.56)
2027	Female	4740889.13 (3905480.55, 5576297.7)	480.88 (413.1, 548.66)
2028	Female	4723961.15 (3785978.32, 5661943.97)	474.65 (394.82, 554.49)
2029	Female	4706645.84 (3654751.63, 5758540.06)	468.56 (376.17, 560.95)
2030	Female	4688860.71 (3513038.97, 5864682.44)	462.6 (357.23, 567.96)
2031	Female	4669904.88 (3361344.66, 5978465.11)	456.72 (338.02, 575.41)
2032	Female	4650834.59 (3201274.95, 6100394.24)	450.94 (318.61, 583.27)
2033	Female	4628559.72 (3031464.96, 6225654.49)	445.3 (299.07, 591.54)
2034	Female	4605749.99 (2854363.08, 6357136.91)	439.82 (279.44, 600.2)
2035	Female	4582362.38 (2670271.85, 6494452.92)	434.48 (259.74, 609.22)
2036	Female	4557702.11 (2479001.28, 6636402.95)	429.25 (239.98, 618.52)
2037	Female	4532406.58 (2281082.59, 6783730.57)	424.15 (220.17, 628.12)
2038	Female	4505915.51 (2076313.97, 6935517.05)	419.19 (200.36, 638.02)
2039	Female	4479795 (1865434.41, 7094155.6)	414.39 (180.55, 648.22)
2040	Female	4453837.25 (1648106.77, 7259567.73)	409.73 (160.76, 658.71)
2041	Female	4427270.08 (1423742.84, 7430797.32)	405.2 (140.97, 669.44)
2042	Female	4400356.56 (1192135.55, 7608578.84)	400.81 (121.2, 680.42)
2043	Female	4374257.66 (953137.91, 7795381.4)	396.56 (101.45, 691.68)
2044	Female	4349619.41 (706254.16, 7993034.8)	392.47 (81.73, 703.2)
2045	Female	4326224.78 (450695.51, 8202044.2)	388.51 (62.04, 714.99)
2022	Male	2964415.6 (2695843.1, 3232988.1)	317.25 (305.19, 329.3)
2023	Male	2971629.13 (2675524.76, 3267733.5)	315.25 (297.59, 332.92)
2024	Male	2978802.31 (2637711.54, 3319893.09)	313.28 (288.44, 338.13)
2025	Male	2985931.32 (2582940.21, 3388922.43)	311.34 (278.19, 344.49)
2026	Male	2992779.14 (2512908.63, 3472649.64)	309.4 (267.04, 351.76)

2027	Male	2999484.33 (2429738.38, 3569230.28)	307.45 (255.14, 359.77)
2028	Male	3005257.44 (2334488.42, 3676026.46)	305.53 (242.61, 368.45)
2029	Male	3011462.19 (2229425.3, 3793499.09)	303.64 (229.53, 377.74)
2030	Male	3018239.25 (2115339.73, 3921138.78)	301.79 (215.98, 387.6)
2031	Male	3025484.41 (1992508.86, 4058459.97)	299.97 (201.97, 397.98)
2032	Male	3033695.44 (1861371.45, 4206019.43)	298.17 (187.53, 408.8)
2033	Male	3040736.66 (1720654.81, 4360818.51)	296.4 (172.73, 420.08)
2034	Male	3048651.99 (1571505.63, 4525798.36)	294.7 (157.58, 431.81)
2035	Male	3057708.37 (1413677.34, 4701739.4)	293.04 (142.13, 443.96)
2036	Male	3067837.47 (1246567.56, 4889107.38)	291.44 (126.36, 456.51)
2037	Male	3079410.4 (1069597.34, 5089223.46)	289.86 (110.31, 469.42)
2038	Male	3091407.68 (881574.69, 5301240.66)	288.34 (93.98, 482.71)
2039	Male	3105410.77 (682005.44, 5528816.1)	286.89 (77.4, 496.38)
2040	Male	3121540.82 (469553.85, 5773529.55)	285.5 (60.57, 510.44)
2041	Male	3139637.01 (242621.23, 6036691.13)	284.17 (43.5, 524.85)
2042	Male	3159820.88 (2697.38, 6320381.81)	282.89 (26.18, 539.6)
2043	Male	3182791.51 (0, 6628187.18)	281.67 (8.63, 554.72)
2044	Male	3209414.74 (0, 6964591.72)	280.53 (-9.15, 570.2)
2045	Male	3239829.92 (0, 7333182.98)	279.45 (-27.17, 586.07)

Notes: ASR: age-standardized rates; YLDs: years lived with disability.

Table S112. Prevalence and YLDs of anxiety disorders in China of future forecasts using bayesian age-period-cohort model.

Year	Sex	Number	ASR
**Prevalence**			
2022	Both	55213295.86 (50180576.4, 60246015.32)	3655.74 (3321.62, 3989.87)
2023	Both	58784501.4 (52270865.89, 65298136.9)	3878.15 (3447.76, 4308.53)
2024	Both	62638835.65 (53777154.2, 71500517.11)	4118.11 (3535.29, 4700.93)
2025	Both	66828474.87 (54712467.5, 78944482.25)	4378.28 (3584.87, 5171.7)
2026	Both	71413013.52 (55059510.06, 87766516.97)	4661.92 (3595.43, 5728.41)
2027	Both	76479720.09 (54770403.84, 98189036.33)	4972.1 (3562.62, 6381.58)
2028	Both	81914919.35 (53610255.2, 110219583.49)	5312.3 (3479.52, 7145.07)
2029	Both	87854244.05 (51483186.53, 124225301.56)	5686.85 (3336.47, 8037.24)
2030	Both	94374667.69 (48185446.73, 140563888.65)	6101.34 (3120.49, 9082.2)
2031	Both	101565003.15 (43443557.79, 159686448.5)	6562.89 (2814.14, 10311.63)
2032	Both	109529182.46 (36894480.8, 182163884.11)	7079.03 (2393.37, 11764.69)
2033	Both	118369064.12 (28056446.89, 208681681.35)	7658.49 (1826.33, 13490.65)
2034	Both	128255682.58 (16316080.65, 240205107.46)	8311.89 (1072.32, 15553.21)
2035	Both	139357450.27 (14416984.86, 277917604.85)	9052.74 (953.48, 18036.52)
2036	Both	151877372.39 (13115606.49, 323361755.82)	9898.37 (870.04, 21053.3)
2037	Both	166035637.02 (11609884.99, 378508576.63)	10868.72 (772.81, 24751.07)
2038	Both	182313187.01 (9890919.08, 446474757.33)	11988.1 (659.86, 29326.48)
2039	Both	201085602.64 (7927178.58, 530972776)	13286.53 (528.95, 35045.32)
2040	Both	222862402.28 (5685210, 637158999.84)	14802.5 (377.33, 42274.31)
2041	Both	248281624.45 (3127430.32, 772137164.39)	16585.71 (202.04, 51526.2)
2042	Both	278115792.01 (299914.1, 945690088.91)	18697.33 (16.35, 63514.76)
2043	Both	313533656.52 (0, 1172221169.15)	21214.77 (0, 79246.61)

2044	Both	355836547.38 (0, 1471541615.65)	24237.35 (0, 100157.89)
2045	Both	406751013.27 (0, 1872225515.38)	27895.45 (0, 128327.37)
2022	Female	34781756.05 (31906777.19, 37656734.91)	4713.61 (4498.93, 4928.28)
2023	Female	37689809.54 (33582801.27, 41796817.81)	5090.79 (4667.57, 5514.01)
2024	Female	40860906.13 (34780578.92, 46941233.34)	5497.55 (4783.17, 6211.93)
2025	Female	44340053.1 (35518670.01, 53161436.19)	5936.73 (4847.25, 7026.21)
2026	Female	48180005.19 (35777123.4, 60582886.98)	6411.87 (4854.55, 7969.18)
2027	Female	52454227.3 (35497195.59, 69411259)	6925.3 (4795.67, 9054.92)
2028	Female	57102491.39 (34486954.73, 79718028.06)	7479.08 (4659.43, 10298.73)
2029	Female	62234800.06 (32630247.2, 91839352.92)	8076.02 (4433.51, 11718.53)
2030	Female	67926043.08 (29725332.71, 106126753.45)	8720.11 (4104.41, 13335.81)
2031	Female	74263901.56 (25503989.8, 123023813.33)	9416.68 (3656.9, 15176.46)
2032	Female	81349855 (19609590.63, 143090119.37)	10169.58 (3072.45, 17266.71)
2033	Female	89287176.08 (11570865.45, 167003486.71)	10982.03 (2330.29, 19633.78)
2034	Female	98239092.94 (781544.98, 195706463.85)	11858.16 (1407.6, 22308.72)
2035	Female	108374434.02 (0, 230368557.2)	12803.58 (279, 25328.17)
2036	Female	119897153.04 (0, 272516923.61)	13825.79 (-1084.41, 28735.98)
2037	Female	133031613.47 (0, 324110414.53)	14930.43 (-2715.52, 32576.39)
2038	Female	148220039.72 (0, 388179381.83)	16122.6 (-4650.46, 36895.67)
2039	Female	165842202.68 (0, 468413154.66)	17408.6 (-6929.29, 41746.5)
2040	Female	186403555.59 (0, 569926516.46)	18796.68 (-9597.18, 47190.55)
2041	Female	210537461.24 (0, 699774257.16)	20297.46 (-12706.05, 53300.97)
2042	Female	239013280.97 (0, 867686277.41)	21919.14 (-16312.69, 60150.97)
2043	Female	272970181.97 (0, 1087951721)	23669.28 (-20478.62, 67817.18)
2044	Female	313705836.26 (0, 1380305610.42)	25557.19 (-25272.5, 76386.88)
2045	Female	362936269.96 (0, 1773218721.14)	27594.93 (-30772.47, 85962.33)

2022	Male	20431539.81 (18273799.21, 22589280.41)	2657.76 (2545.44, 2770.09)
2023	Male	21094691.86 (18688064.62, 23501319.1)	2732.95 (2571.71, 2894.2)
2024	Male	21777929.52 (18996575.28, 24559283.76)	2810.22 (2582.3, 3038.14)
2025	Male	22488421.78 (19193797.49, 25783046.06)	2889.81 (2580.05, 3199.57)
2026	Male	23233008.33 (19282386.66, 27183630)	2971.9 (2566.45, 3377.35)
2027	Male	24025492.79 (19273208.25, 28777777.33)	3056.44 (2541.97, 3570.91)
2028	Male	24812427.95 (19123300.47, 30501555.44)	3143.49 (2506.75, 3780.22)
2029	Male	25619443.99 (18852939.34, 32385948.64)	3233.18 (2460.71, 4005.65)
2030	Male	26448624.61 (18460114.02, 34437135.21)	3325.72 (2403.57, 4247.88)
2031	Male	27301101.58 (17939568, 36662635.17)	3421.28 (2334.89, 4507.67)
2032	Male	28179327.46 (17284890.18, 39073764.74)	3519.78 (2254, 4785.57)
2033	Male	29081888.04 (16485581.44, 41678194.65)	3621.29 (2160.32, 5082.26)
2034	Male	30016589.64 (15534535.68, 44498643.61)	3725.89 (2053.25, 5398.54)
2035	Male	30983016.26 (14416984.86, 47549047.66)	3833.76 (1932.13, 5735.39)
2036	Male	31980219.35 (13115606.49, 50844832.21)	3945.03 (1796.22, 6093.83)
2037	Male	33004023.55 (11609884.99, 54398162.1)	4059.6 (1644.6, 6474.61)
2038	Male	34093147.29 (9890919.08, 58295375.5)	4177.58 (1476.49, 6878.66)
2039	Male	35243399.96 (7927178.58, 62559621.34)	4299.06 (1291.07, 7307.05)
2040	Male	36458846.69 (5685210, 67232483.38)	4424.25 (1087.44, 7761.07)
2041	Male	37744163.2 (3127430.32, 72362907.23)	4553.32 (864.62, 8242.02)
2042	Male	39102511.04 (299914.1, 78003811.5)	4686.17 (621.53, 8750.82)
2043	Male	40563474.55 (0, 84269448.16)	4822.91 (357.15, 9288.67)
2044	Male	42130711.12 (0, 91236005.23)	4963.66 (70.4, 9856.93)
2045	Male	43814743.31 (0, 99006794.23)	5108.67 (-239.87, 10457.21)
**YLDs**			
2022	Both	6577336.15 (5977779.3, 7176893.01)	441.58 (401.22, 481.95)

2023	Both	6997355.99 (6219951.79, 7774760.18)	468.47 (416.34, 520.6)
2024	Both	7449979.67 (6390933.76, 8509025.58)	497.47 (426.72, 568.22)
2025	Both	7941513.16 (6492670.67, 9390355.66)	528.91 (432.45, 625.37)
2026	Both	8479226.73 (6523528.47, 10434924.99)	563.18 (433.39, 692.97)
2027	Both	9073863.36 (6478265.76, 11669460.95)	600.66 (429.03, 772.29)
2028	Both	9710493.61 (6328131.15, 13092856.07)	641.76 (418.51, 865.01)
2029	Both	10405200.13 (6061963.71, 14748436.56)	687.02 (400.65, 973.39)
2030	Both	11167059.6 (5655759.4, 16678359.81)	737.11 (373.86, 1100.37)
2031	Both	12006617.36 (5077228.81, 18936005.91)	792.91 (336, 1249.82)
2032	Both	12936227.55 (4283368.19, 21589086.91)	855.32 (284.09, 1426.54)
2033	Both	13967605.83 (3216805.84, 24718405.82)	925.41 (214.22, 1636.59)
2034	Both	15120257.83 (1831893.65, 28437239.6)	1004.48 (123.55, 1887.81)
2035	Both	16413842.14 (1688966.1, 32885615.7)	1094.18 (114.28, 2190.55)
2036	Both	17872224.8 (1522734.33, 38246687.87)	1196.62 (103.42, 2558.73)
2037	Both	19521209.63 (1330541.54, 44754747.84)	1314.25 (90.74, 3010.59)
2038	Both	21418401.76 (1111011.95, 52783473.07)	1450.04 (75.97, 3570.51)
2039	Both	23606682.68 (859948.28, 62773233.41)	1607.69 (58.8, 4271.54)
2040	Both	26145935.21 (572915.22, 75340396.18)	1791.92 (38.85, 5159.38)
2041	Both	29111568.87 (245538.61, 91336925.96)	2008.85 (15.85, 6298.02)
2042	Both	32595109.89 (0, 111939266.01)	2266.02 (0, 7776.86)
2043	Both	36735392.2 (0, 138884069.68)	2573.01 (0, 9722.32)
2044	Both	41684965.47 (0, 174556844.06)	2942.12 (0, 12315.29)
2045	Both	47648804.61 (0, 222413737.58)	3389.55 (0, 15818.3)
2022	Female	4113569.99 (3771399.37, 4455740.6)	566.74 (540.61, 592.86)
2023	Female	4453115.37 (3964111.19, 4942119.55)	612.01 (560.61, 663.41)
2024	Female	4822898.56 (4099187.93, 5546609.19)	660.83 (574.15, 747.51)

2025	Female	5228261.9 (4179088.02, 6277435.77)	713.53 (581.4, 845.65)
2026	Female	5675537.44 (4201659.7, 7149415.19)	770.53 (581.75, 959.3)
2027	Female	6173658.16 (4160187.77, 8187128.54)	832.11 (574.05, 1090.18)
2028	Female	6714549.98 (4031415.33, 9397684.63)	898.54 (556.95, 1240.13)
2029	Female	7311137.05 (3801699.37, 10820574.74)	970.14 (528.97, 1411.31)
2030	Female	7972155.89 (3447252.72, 12497059.05)	1047.38 (488.46, 1606.3)
2031	Female	8707916.56 (2936417.3, 14479415.82)	1130.91 (433.59, 1828.23)
2032	Female	9530391.19 (2227001.97, 16833780.4)	1221.19 (362.12, 2080.26)
2033	Female	10451556.7 (1263072.06, 19640041.34)	1318.6 (271.52, 2365.68)
2034	Female	11490037.65 (0, 23008692.9)	1423.65 (159.06, 2688.25)
2035	Female	12665466.26 (0, 27077830.04)	1537 (21.64, 3052.36)
2036	Female	14001725.61 (0, 32028423.83)	1659.55 (-144.21, 3463.3)
2037	Female	15525067.2 (0, 38093004.51)	1791.96 (-342.48, 3926.41)
2038	Female	17288199.71 (0, 45634080.91)	1934.87 (-577.53, 4447.26)
2039	Female	19334660.16 (0, 55089136.66)	2089.01 (-854.2, 5032.22)
2040	Female	21723723.94 (0, 67068888.86)	2255.38 (-1177.96, 5688.72)
2041	Female	24530083.92 (0, 82418582.36)	2435.24 (-1555.06, 6425.53)
2042	Female	27844751.26 (0, 102307358.25)	2629.56 (-1992.38, 7251.5)
2043	Female	31802816.22 (0, 128456799.38)	2839.27 (-2497.33, 8175.87)
2044	Female	36556435.49 (0, 163242291.35)	3065.48 (-3078.23, 9209.19)
2045	Female	42309085.52 (0, 210105740.68)	3309.63 (-3744.49, 10363.75)
2022	Male	2463766.17 (2206379.93, 2721152.41)	324.07 (310.24, 337.89)
2023	Male	2544240.61 (2255840.6, 2832640.63)	333.53 (313.53, 353.53)
2024	Male	2627081.11 (2291745.83, 2962416.39)	343.26 (314.85, 371.67)
2025	Male	2713251.26 (2313582.64, 3112919.89)	353.29 (314.56, 392.02)
2026	Male	2803689.28 (2321868.76, 3285509.8)	363.64 (312.83, 414.45)

2027	Male	2900205.2 (2318077.99, 3482332.41)	374.31 (309.72, 438.9)
2028	Male	2995943.63 (2296715.82, 3695171.44)	385.3 (305.24, 465.36)
2029	Male	3094063.08 (2260264.34, 3927861.82)	396.63 (299.36, 493.89)
2030	Male	3194903.72 (2208506.68, 4181300.75)	408.32 (292.06, 524.59)
2031	Male	3298700.8 (2140811.52, 4456590.09)	420.41 (283.27, 557.55)
2032	Male	3405836.36 (2056366.21, 4755306.51)	432.87 (272.9, 592.85)
2033	Male	3516049.13 (1953733.77, 5078364.48)	445.72 (260.87, 630.57)
2034	Male	3630220.17 (1831893.65, 5428546.7)	458.97 (247.11, 670.83)
2035	Male	3748375.88 (1688966.1, 5807785.66)	472.64 (231.52, 713.76)
2036	Male	3870499.18 (1522734.33, 6218264.04)	486.75 (214.01, 759.48)
2037	Male	3996142.44 (1330541.54, 6661743.33)	501.29 (194.46, 808.11)
2038	Male	4130202.06 (1111011.95, 7149392.16)	516.26 (172.76, 859.76)
2039	Male	4272022.51 (859948.28, 7684096.74)	531.69 (148.8, 914.58)
2040	Male	4422211.27 (572915.22, 8271507.32)	547.6 (122.47, 972.74)
2041	Male	4581484.95 (245538.61, 8918343.6)	564.02 (93.63, 1034.41)
2042	Male	4750358.64 (0, 9631907.76)	580.92 (62.13, 1099.72)
2043	Male	4932575.98 (0, 10427270.3)	598.33 (27.85, 1168.82)
2044	Male	5128529.98 (0, 11314552.71)	616.27 (-9.36, 1241.89)
2045	Male	5339719.08 (0, 12307996.9)	634.75 (-49.66, 1319.16)

Notes: ASR: age-standardized rates; YLDs: years lived with disability.

Table S113. Prevalence and YLDs of opioid use disorders in China of future forecasts using bayesian age-period-cohort model.

Year	Sex	Number	ASR
**Prevalence**			
2022	Both	1517937.35 (1374500.77, 1661373.93)	129.8 (117.56, 142.03)
2023	Both	1534144.88 (1355311.28, 1712978.48)	131.93 (116.55, 147.31)
2024	Both	1550713.21 (1317948.44, 1783477.99)	134.25 (114.06, 154.44)
2025	Both	1568535.2 (1266056, 1871014.4)	136.78 (110.3, 163.25)
2026	Both	1588312.83 (1202089.58, 1974536.08)	139.54 (105.43, 173.65)
2027	Both	1611155.45 (1127691.92, 2094618.98)	142.56 (99.51, 185.61)
2028	Both	1637074.53 (1042870.2, 2231278.85)	145.91 (92.56, 199.26)
2029	Both	1666118.76 (947040.87, 2385196.66)	149.64 (84.52, 214.76)
2030	Both	1699479.21 (839665.12, 2559293.31)	153.81 (75.28, 232.34)
2031	Both	1738238.75 (719298.42, 2757179.08)	158.45 (64.65, 252.26)
2032	Both	1784244.08 (583831.7, 2984656.45)	163.63 (52.37, 274.9)
2033	Both	1835704.14 (429216.7, 3242191.57)	169.45 (38.13, 300.76)
2034	Both	1894161.45 (256379.05, 3536698.53)	175.99 (22.08, 330.48)
2035	Both	1961481.41 (167820.84, 3877269.61)	183.38 (14.37, 364.83)
2036	Both	2039584.59 (65615.85, 4275224.46)	191.71 (5.41, 404.76)
2037	Both	2132214.29 (0, 4748642.09)	201.12 (0, 451.53)
2038	Both	2231593.07 (0, 5292128.7)	211.8 (0, 506.8)
2039	Both	2343085.59 (0, 5932180.76)	223.98 (0, 572.74)
2040	Both	2469016.29 (0, 6694072.36)	237.89 (0, 652.17)
2041	Both	2611740.28 (0, 7610103.04)	253.83 (0, 748.79)
2042	Both	2774921.07 (0, 8725811.43)	272.15 (0, 867.62)
2043	Both	2958830.03 (0, 10089121.18)	293.33 (0, 1015.56)

2044	Both	3168984.63 (0, 11780903.33)	317.94 (0, 1202.08)
2045	Both	3410046.64 (0, 13904740.8)	346.68 (0, 1440.27)
2022	Female	807336.65 (715252.67, 899420.63)	136.78 (129.23, 144.33)
2023	Female	819919.96 (713679.37, 926160.55)	139.36 (127.93, 150.79)
2024	Female	832680.25 (703909.39, 961451.12)	142.07 (125.44, 158.71)
2025	Female	846026.4 (686655.13, 1005397.67)	144.93 (122, 167.86)
2026	Female	860273.23 (662881.23, 1057665.23)	147.9 (117.68, 178.12)
2027	Female	875951.3 (633479.13, 1118423.48)	150.99 (112.51, 189.46)
2028	Female	893048.92 (598606.7, 1187491.15)	154.21 (106.53, 201.9)
2029	Female	911553.36 (558128.86, 1264977.87)	157.6 (99.73, 215.48)
2030	Female	932035.28 (511996.14, 1352074.42)	161.15 (92.08, 230.22)
2031	Female	955003.28 (459764.07, 1450242.5)	164.83 (83.54, 246.13)
2032	Female	981356.07 (400786.05, 1561926.1)	168.65 (74.06, 263.24)
2033	Female	1010227.92 (333430.64, 1687025.2)	172.62 (63.62, 281.62)
2034	Female	1042281.59 (256379.05, 1828184.13)	176.77 (52.17, 301.36)
2035	Female	1078307.36 (167820.84, 1988793.88)	181.08 (39.67, 322.5)
2036	Female	1119075.89 (65615.85, 2172895.7)	185.55 (26.04, 345.07)
2037	Female	1166233.5 (0, 2387139.23)	190.17 (11.23, 369.11)
2038	Female	1215951.49 (0, 2627599.78)	194.95 (-4.82, 394.73)
2039	Female	1270629.01 (0, 2903617.5)	199.93 (-22.17, 422.02)
2040	Female	1331083.55 (0, 3222767.72)	205.08 (-40.9, 451.06)
2041	Female	1398033.9 (0, 3594054.65)	210.41 (-61.07, 481.89)
2042	Female	1472743.24 (0, 4029957.47)	215.91 (-82.76, 514.58)
2043	Female	1554806.65 (0, 4541407.82)	221.6 (-106.05, 549.26)
2044	Female	1646230.64 (0, 5148865)	227.5 (-131.04, 586.04)
2045	Female	1748261.69 (0, 5875504.58)	233.61 (-157.81, 625.03)

2022	Male	710600.7 (659248.1, 761953.3)	122.25 (116.6, 127.9)
2023	Male	714224.92 (641631.92, 786817.93)	123.77 (113.26, 134.27)
2024	Male	718032.96 (614039.05, 822026.87)	125.36 (108.65, 142.08)
2025	Male	722508.8 (579400.88, 865616.73)	127.04 (102.99, 151.1)
2026	Male	728039.6 (539208.35, 916870.84)	128.79 (96.35, 161.22)
2027	Male	735204.15 (494212.79, 976195.5)	130.58 (88.77, 172.39)
2028	Male	744025.61 (444263.5, 1043787.71)	132.45 (80.28, 184.62)
2029	Male	754565.4 (388912.01, 1120218.79)	134.4 (70.89, 197.91)
2030	Male	767443.93 (327668.98, 1207218.89)	136.43 (60.59, 212.28)
2031	Male	783235.46 (259534.35, 1306936.58)	138.53 (49.37, 227.7)
2032	Male	802888 (183045.65, 1422730.35)	140.69 (37.2, 244.19)
2033	Male	825476.22 (95786.07, 1555166.37)	142.92 (24.06, 261.78)
2034	Male	851879.86 (0, 1708514.41)	145.24 (9.95, 280.53)
2035	Male	883174.05 (0, 1888475.74)	147.64 (-5.18, 300.46)
2036	Male	920508.7 (0, 2102328.75)	150.11 (-21.36, 321.57)
2037	Male	965980.79 (0, 2361502.86)	152.64 (-38.62, 343.9)
2038	Male	1015641.58 (0, 2664528.92)	155.25 (-56.99, 367.49)
2039	Male	1072456.57 (0, 3028563.26)	157.94 (-76.52, 392.4)
2040	Male	1137932.74 (0, 3471304.64)	160.71 (-97.25, 418.68)
2041	Male	1213706.38 (0, 4016048.39)	163.56 (-119.2, 446.33)
2042	Male	1302177.84 (0, 4695853.97)	166.48 (-142.44, 475.4)
2043	Male	1404023.38 (0, 5547713.36)	169.48 (-166.99, 505.95)
2044	Male	1522753.99 (0, 6632038.33)	172.56 (-192.93, 538.05)
2045	Male	1661784.95 (0, 8029236.21)	175.73 (-220.29, 571.74)
**YLDs**			
2022	Both	630450.96 (570721.99, 690179.93)	54.39 (49.25, 59.53)

2023	Both	636361.73 (561636.13, 711087.32)	55.26 (48.77, 61.75)
2024	Both	642399.67 (544898.52, 739900.82)	56.21 (47.67, 64.76)
2025	Both	648950.35 (522098.24, 775802.46)	57.26 (46.03, 68.48)
2026	Both	656317.59 (494300.08, 818335.09)	58.4 (43.92, 72.88)
2027	Both	664983.29 (462214.36, 867752.22)	59.65 (41.36, 77.94)
2028	Both	674943.88 (425838.38, 924049.37)	61.04 (38.37, 83.71)
2029	Both	686202.7 (384915.62, 987489.79)	62.59 (34.91, 90.27)
2030	Both	699269.76 (339220.99, 1059318.52)	64.33 (30.94, 97.71)
2031	Both	714609.72 (288142.54, 1141076.91)	66.27 (26.38, 106.15)
2032	Both	733019.1 (230780.72, 1235257.48)	68.44 (21.12, 115.76)
2033	Both	753700.5 (165409.81, 1341991.18)	70.87 (15.01, 126.74)
2034	Both	777299.15 (92016, 1464199.65)	73.62 (8.09, 139.37)
2035	Both	804615.06 (53819.88, 1605766.56)	76.73 (4.69, 153.99)
2036	Both	836475.28 (10271.42, 1771540.22)	80.25 (0.82, 171.01)
2037	Both	874493.66 (0, 1969301.07)	84.22 (0, 190.98)
2038	Both	915307.52 (0, 2196587.36)	88.74 (0, 214.61)
2039	Both	961188.42 (0, 2464697.4)	93.9 (0, 242.84)
2040	Both	1013123.08 (0, 2784414.09)	99.8 (0, 276.9)
2041	Both	1072115.11 (0, 3169533.11)	106.57 (0, 318.4)
2042	Both	1139724.82 (0, 3639521.81)	114.36 (0, 369.53)
2043	Both	1216096.94 (0, 4214948.63)	123.39 (0, 433.28)
2044	Both	1303536.42 (0, 4930271.2)	133.88 (0, 513.77)
2045	Both	1404007.73 (0, 5829666.32)	146.16 (0, 616.7)
2022	Female	332034.92 (293845.53, 370224.31)	56.85 (53.63, 60.06)
2023	Female	336682.23 (292443.08, 380921.37)	57.89 (53, 62.78)
2024	Female	341380.63 (287526.44, 395234.81)	58.99 (51.86, 66.12)

2025	Female	346309.69 (279452.45, 413166.93)	60.15 (50.31, 69.98)
2026	Female	351610.47 (268662.48, 434558.45)	61.35 (48.38, 74.31)
2027	Female	357517.52 (255548.22, 459486.82)	62.6 (46.1, 79.1)
2028	Female	364020.4 (240169.8, 487871)	63.9 (43.46, 84.35)
2029	Female	371104.41 (222459.34, 519749.49)	65.28 (40.47, 90.08)
2030	Female	379016.44 (202389.62, 555643.25)	66.71 (37.11, 96.31)
2031	Female	387979.34 (179761.73, 596196.96)	68.2 (33.38, 103.03)
2032	Female	398384.77 (154280.91, 642488.64)	69.75 (29.25, 110.25)
2033	Female	409850.45 (125231.03, 694469.87)	71.36 (24.7, 118.01)
2034	Female	422653.05 (92016, 753290.09)	73.03 (19.73, 126.33)
2035	Female	437137.97 (53819.88, 820456.07)	74.78 (14.31, 135.25)
2036	Female	453647.34 (10271.42, 897782.45)	76.58 (8.41, 144.76)
2037	Female	472901.33 (0, 988251.88)	78.45 (2.01, 154.89)
2038	Female	493265.25 (0, 1090232.94)	80.38 (-4.92, 165.69)
2039	Female	515757.89 (0, 1207883.61)	82.39 (-12.4, 177.18)
2040	Female	540743.87 (0, 1344688.81)	84.47 (-20.46, 189.4)
2041	Female	568554.69 (0, 1504848.94)	86.62 (-29.12, 202.37)
2042	Female	599759.52 (0, 1694201.19)	88.84 (-38.43, 216.11)
2043	Female	634224.38 (0, 1918055.56)	91.13 (-48.42, 230.68)
2044	Female	672819.96 (0, 2186027.03)	93.51 (-59.12, 246.13)
2045	Female	716122.77 (0, 2509264.63)	95.97 (-70.56, 262.49)
2022	Male	298416.04 (276876.46, 319955.62)	51.69 (49.29, 54.09)
2023	Male	299679.5 (269193.05, 330165.95)	52.33 (47.87, 56.78)
2024	Male	301019.04 (257372.07, 344666.01)	52.99 (45.91, 60.07)
2025	Male	302640.66 (242645.78, 362635.53)	53.69 (43.52, 63.87)
2026	Male	304707.12 (225637.6, 383776.64)	54.42 (40.71, 68.14)

2027	Male	307465.77 (206666.13, 408265.4)	55.17 (37.5, 72.85)
2028	Male	310923.47 (185668.58, 436178.37)	55.95 (33.91, 78)
2029	Male	315098.29 (162456.28, 467740.3)	56.77 (29.95, 83.6)
2030	Male	320253.32 (136831.37, 503675.27)	57.62 (25.6, 89.65)
2031	Male	326630.38 (108380.81, 544879.95)	58.5 (20.86, 96.15)
2032	Male	334634.33 (76499.81, 592768.84)	59.41 (15.73, 103.09)
2033	Male	343850.04 (40178.78, 647521.31)	60.35 (10.19, 110.5)
2034	Male	354646.11 (0, 710909.55)	61.32 (4.24, 118.4)
2035	Male	367477.09 (0, 785310.5)	62.33 (-2.14, 126.79)
2036	Male	382827.94 (0, 873757.76)	63.36 (-8.96, 135.68)
2037	Male	401592.33 (0, 981049.19)	64.43 (-16.23, 145.09)
2038	Male	422042.26 (0, 1106354.42)	65.52 (-23.97, 155.02)
2039	Male	445430.53 (0, 1256813.8)	66.65 (-32.2, 165.5)
2040	Male	472379.21 (0, 1439725.27)	67.82 (-40.93, 176.57)
2041	Male	503560.41 (0, 1664684.17)	69.01 (-50.18, 188.21)
2042	Male	539965.3 (0, 1945320.61)	70.24 (-59.96, 200.45)
2043	Male	581872.57 (0, 2296893.07)	71.5 (-70.3, 213.3)
2044	Male	630716.46 (0, 2744244.17)	72.79 (-81.22, 226.81)
2045	Male	687884.96 (0, 3320401.69)	74.12 (-92.74, 240.99)

Notes: ASR: age-standardized rates; YLDs: years lived with disability.

Table S114. Prevalence and YLDs of varicella and herpes zoster in China of future forecasts using bayesian age-period-cohort model.

Year	Sex	Number	ASR
**Prevalence**			
2022	Both	1123499.94 (1062206.59, 1184793.29)	70.25 (66.32, 74.18)
2023	Both	1132135.72 (1058399.66, 1205871.78)	69.99 (65.3, 74.67)
2024	Both	1141545.03 (1048067.67, 1235022.38)	69.73 (63.87, 75.6)
2025	Both	1151262.66 (1031775.85, 1270749.47)	69.49 (62.1, 76.88)
2026	Both	1160987.14 (1010263.21, 1311711.07)	69.27 (60.08, 78.46)
2027	Both	1170533.97 (984066.15, 1357001.79)	69.07 (57.84, 80.29)
2028	Both	1180401.18 (954064.86, 1406737.5)	68.89 (55.44, 82.34)
2029	Both	1190924.6 (920755.24, 1461093.95)	68.74 (52.88, 84.6)
2030	Both	1201964.51 (884098.47, 1519830.55)	68.62 (50.18, 87.06)
2031	Both	1213400.63 (843989.6, 1582811.66)	68.54 (47.36, 89.72)
2032	Both	1225211.51 (800338.24, 1650084.78)	68.49 (44.4, 92.58)
2033	Both	1237571.46 (753161.5, 1721981.42)	68.49 (41.32, 95.66)
2034	Both	1250917.62 (702540.75, 1799294.5)	68.53 (38.1, 98.96)
2035	Both	1265129.88 (648130.44, 1882129.32)	68.62 (34.74, 102.5)
2036	Both	1280080.6 (589540.92, 1970620.28)	68.76 (31.23, 106.3)
2037	Both	1295763.89 (526396.33, 2065131.46)	68.97 (27.55, 110.38)
2038	Both	1312289.31 (458340.3, 2166238.33)	69.23 (23.69, 114.77)
2039	Both	1330128.81 (385036.63, 2275220.99)	69.56 (19.62, 119.5)
2040	Both	1349121.88 (305810.41, 2392433.35)	69.95 (15.31, 124.6)
2041	Both	1369067.81 (219929.89, 2518210.07)	70.43 (10.74, 130.11)
2042	Both	1389870.77 (129525.7, 2653136.45)	70.98 (6.46, 136.1)
2043	Both	1411567.65 (91015.93, 2798114.58)	71.62 (4.33, 142.6)

2044	Both	1434758.85 (52311.08, 2955336.37)	72.35 (2.29, 149.7)
2045	Both	1459379.23 (12689.44, 3125905.93)	73.19 (0.53, 157.46)
2022	Female	597011.29 (562269.89, 631752.69)	73.77 (71.61, 75.92)
2023	Female	602717.79 (560652.29, 644783.3)	73.48 (69.95, 77.01)
2024	Female	608845.08 (555129.01, 662561.15)	73.19 (67.92, 78.46)
2025	Female	615170.21 (546086.43, 684254)	72.91 (65.63, 80.19)
2026	Female	621543.67 (533976.19, 709111.15)	72.62 (63.11, 82.13)
2027	Female	627879.53 (519115.68, 736643.39)	72.33 (60.4, 84.26)
2028	Female	634417.52 (501949.36, 766885.68)	72.04 (57.52, 86.56)
2029	Female	641357.94 (482745.66, 799970.22)	71.75 (54.49, 89.01)
2030	Female	648636.23 (461474.38, 835798.07)	71.46 (51.32, 91.6)
2031	Female	656190.36 (438058.01, 874322.7)	71.17 (48.02, 94.32)
2032	Female	664011.28 (412420.51, 915602.04)	70.88 (44.6, 97.16)
2033	Female	672202.98 (384537.79, 959868.18)	70.58 (41.06, 100.1)
2034	Female	681011.25 (354405.87, 1007616.63)	70.29 (37.42, 103.16)
2035	Female	690375.56 (321789.23, 1058961.89)	69.99 (33.69, 106.3)
2036	Female	700226.83 (286419.98, 1114033.69)	69.7 (29.86, 109.54)
2037	Female	710564.12 (248027.81, 1173100.42)	69.4 (25.94, 112.85)
2038	Female	721476.01 (206347.25, 1236604.78)	69.1 (21.94, 116.25)
2039	Female	733243.54 (161088.75, 1305398.32)	68.79 (17.87, 119.72)
2040	Female	745792.76 (111776.26, 1379809.26)	68.49 (13.72, 123.25)
2041	Female	759019.21 (57887.18, 1460155.57)	68.18 (9.51, 126.84)
2042	Female	772881.35 (1756.82, 1546926.49)	67.86 (5.24, 130.49)
2043	Female	787445.47 (0, 1640886.17)	67.55 (0.9, 134.19)
2044	Female	803075.59 (0, 1743560.2)	67.23 (-3.48, 137.94)
2045	Female	819756.44 (0, 1855861.33)	66.91 (-7.92, 141.74)

2022	Male	526488.65 (499936.7, 553040.6)	66.67 (64.99, 68.35)
2023	Male	529417.93 (497747.37, 561088.48)	66.4 (63.72, 69.08)
2024	Male	532699.95 (492938.66, 572461.23)	66.13 (62.18, 70.09)
2025	Male	536092.44 (485689.42, 586495.47)	65.86 (60.43, 71.29)
2026	Male	539443.47 (476287.02, 602599.92)	65.59 (58.53, 72.66)
2027	Male	542654.44 (464950.47, 620358.41)	65.32 (56.48, 74.16)
2028	Male	545983.67 (452115.5, 639851.83)	65.05 (54.31, 75.79)
2029	Male	549566.66 (438009.58, 661123.73)	64.77 (52.03, 77.52)
2030	Male	553328.28 (422624.09, 684032.48)	64.5 (49.64, 79.36)
2031	Male	557210.27 (405931.59, 708488.96)	64.22 (47.16, 81.28)
2032	Male	561200.23 (387917.72, 734482.73)	63.95 (44.6, 83.3)
2033	Male	565368.48 (368623.72, 762113.24)	63.67 (41.95, 85.39)
2034	Male	569906.37 (348134.88, 791677.87)	63.39 (39.23, 87.56)
2035	Male	574754.33 (326341.22, 823167.43)	63.11 (36.43, 89.79)
2036	Male	579853.76 (303120.95, 856586.58)	62.83 (33.57, 92.09)
2037	Male	585199.78 (278368.51, 892031.04)	62.55 (30.65, 94.44)
2038	Male	590813.3 (251993.05, 929633.55)	62.26 (27.67, 96.85)
2039	Male	596885.28 (223947.88, 969822.67)	61.98 (24.64, 99.32)
2040	Male	603329.12 (194034.15, 1012624.1)	61.69 (21.55, 101.82)
2041	Male	610048.6 (162042.71, 1058054.5)	61.39 (18.41, 104.37)
2042	Male	616989.42 (127768.88, 1106209.96)	61.1 (15.24, 106.96)
2043	Male	624122.17 (91015.93, 1157228.41)	60.8 (12.01, 109.59)
2044	Male	631683.26 (52311.08, 1211776.17)	60.5 (8.76, 112.24)
2045	Male	639622.79 (12689.44, 1270044.6)	60.2 (5.46, 114.93)
**YLDs**			
2022	Both	55090.45 (51466.46, 58714.45)	3 (2.79, 3.22)

2023	Both	55867.29 (51534.2, 60200.37)	3.01 (2.76, 3.26)
2024	Both	56659.55 (51204.81, 62114.29)	3.01 (2.7, 3.31)
2025	Both	57458.53 (50520.18, 64396.87)	3.01 (2.63, 3.39)
2026	Both	58257.61 (49523.22, 66992)	3.01 (2.55, 3.48)
2027	Both	59055.23 (48245.56, 69864.89)	3.02 (2.45, 3.58)
2028	Both	59851.26 (46708.48, 72994.03)	3.02 (2.35, 3.7)
2029	Both	60670.51 (44942.45, 76398.57)	3.03 (2.24, 3.83)
2030	Both	61509.36 (42946.66, 80072.06)	3.04 (2.11, 3.97)
2031	Both	62364.95 (40715.51, 84014.38)	3.05 (1.98, 4.12)
2032	Both	63239.28 (38242.61, 88235.94)	3.06 (1.85, 4.28)
2033	Both	64138.19 (35521.49, 92754.9)	3.08 (1.7, 4.46)
2034	Both	65085.72 (32550.15, 97621.29)	3.1 (1.54, 4.66)
2035	Both	66080.09 (29305.75, 102854.43)	3.12 (1.38, 4.86)
2036	Both	67118.68 (25760.87, 108476.5)	3.15 (1.2, 5.09)
2037	Both	68203.12 (21885.25, 114521)	3.17 (1.01, 5.34)
2038	Both	69340.44 (17646.27, 121034.62)	3.21 (0.81, 5.6)
2039	Both	70559.66 (13007.92, 128111.4)	3.24 (0.59, 5.89)
2040	Both	71859.43 (7934.69, 135803.52)	3.28 (0.36, 6.21)
2041	Both	73237.04 (4722.12, 144168.9)	3.33 (0.22, 6.56)
2042	Both	74694.15 (2457.05, 153283.81)	3.38 (0.11, 6.94)
2043	Both	76235.83 (37.98, 163242.64)	3.44 (0, 7.37)
2044	Both	77896.84 (0, 174221.36)	3.5 (0, 7.84)
2045	Both	79683.36 (0, 186359.55)	3.58 (0, 8.36)
2022	Female	29514.62 (27583.49, 31445.75)	3.18 (3.09, 3.28)
2023	Female	29955.52 (27599.86, 32311.18)	3.18 (3.02, 3.35)
2024	Female	30406.34 (27380.6, 33432.07)	3.18 (2.93, 3.43)

2025	Female	30862.99 (26955.27, 34770.72)	3.18 (2.82, 3.53)
2026	Female	31322.3 (26350.45, 36294.15)	3.18 (2.71, 3.64)
2027	Female	31783.86 (25584.18, 37983.55)	3.18 (2.59, 3.76)
2028	Female	32248.17 (24667.91, 39828.44)	3.17 (2.46, 3.89)
2029	Female	32728.7 (23616.76, 41840.63)	3.17 (2.31, 4.03)
2030	Female	33224.25 (22429.3, 44019.19)	3.17 (2.17, 4.18)
2031	Female	33733.72 (21100.94, 46366.49)	3.17 (2.01, 4.33)
2032	Female	34258.67 (19626.2, 48891.13)	3.17 (1.85, 4.49)
2033	Female	34802.66 (17999.01, 51606.31)	3.17 (1.68, 4.66)
2034	Female	35378.93 (16214.68, 54543.19)	3.16 (1.5, 4.83)
2035	Female	35987.13 (14257.28, 57716.97)	3.16 (1.32, 5.01)
2036	Female	36626.27 (12107.68, 61144.86)	3.16 (1.13, 5.19)
2037	Female	37298.07 (9744.38, 64851.77)	3.16 (0.93, 5.38)
2038	Female	38007.53 (7143.43, 68871.63)	3.16 (0.73, 5.58)
2039	Female	38771.39 (4276.66, 73266.13)	3.15 (0.53, 5.78)
2040	Female	39589.95 (1124.39, 78074.87)	3.15 (0.32, 5.99)
2041	Female	40462.29 (0, 83341.52)	3.15 (0.1, 6.2)
2042	Female	41390.52 (0, 89123.43)	3.15 (-0.12, 6.41)
2043	Female	42379.68 (0, 95493.02)	3.14 (-0.34, 6.63)
2044	Female	43450.96 (0, 102573.06)	3.14 (-0.57, 6.86)
2045	Female	44609.56 (0, 110468.88)	3.14 (-0.81, 7.08)
2022	Male	25575.83 (23882.97, 27268.7)	2.83 (2.75, 2.9)
2023	Male	25911.77 (23934.34, 27889.19)	2.83 (2.7, 2.96)
2024	Male	26253.21 (23824.21, 28682.22)	2.83 (2.63, 3.03)
2025	Male	26595.53 (23564.91, 29626.16)	2.83 (2.56, 3.1)
2026	Male	26935.31 (23172.77, 30697.85)	2.83 (2.48, 3.19)

2027	Male	27271.36 (22661.39, 31881.34)	2.84 (2.39, 3.28)
2028	Male	27603.08 (22040.57, 33165.59)	2.84 (2.29, 3.38)
2029	Male	27941.81 (21325.68, 34557.94)	2.84 (2.19, 3.48)
2030	Male	28285.11 (20517.36, 36052.87)	2.84 (2.08, 3.6)
2031	Male	28631.23 (19614.57, 37647.88)	2.84 (1.97, 3.71)
2032	Male	28980.61 (18616.41, 39344.81)	2.84 (1.85, 3.83)
2033	Male	29335.53 (17522.48, 41148.59)	2.84 (1.72, 3.96)
2034	Male	29706.79 (16335.47, 43078.1)	2.84 (1.59, 4.09)
2035	Male	30092.97 (15048.47, 45137.46)	2.84 (1.46, 4.23)
2036	Male	30492.42 (13653.19, 47331.64)	2.85 (1.32, 4.37)
2037	Male	30905.05 (12140.87, 49669.23)	2.85 (1.18, 4.52)
2038	Male	31332.91 (10502.84, 52162.99)	2.85 (1.03, 4.67)
2039	Male	31788.26 (8731.25, 54845.27)	2.85 (0.87, 4.82)
2040	Male	32269.47 (6810.3, 57728.65)	2.85 (0.72, 4.98)
2041	Male	32774.75 (4722.12, 60827.38)	2.85 (0.56, 5.14)
2042	Male	33303.62 (2457.05, 64160.38)	2.85 (0.39, 5.3)
2043	Male	33856.15 (37.98, 67749.62)	2.85 (0.22, 5.47)
2044	Male	34445.88 (0, 71648.3)	2.85 (0.05, 5.64)
2045	Male	35073.8 (0, 75890.68)	2.85 (-0.13, 5.82)

Notes: ASR: age-standardized rates; YLDs: years lived with disability.

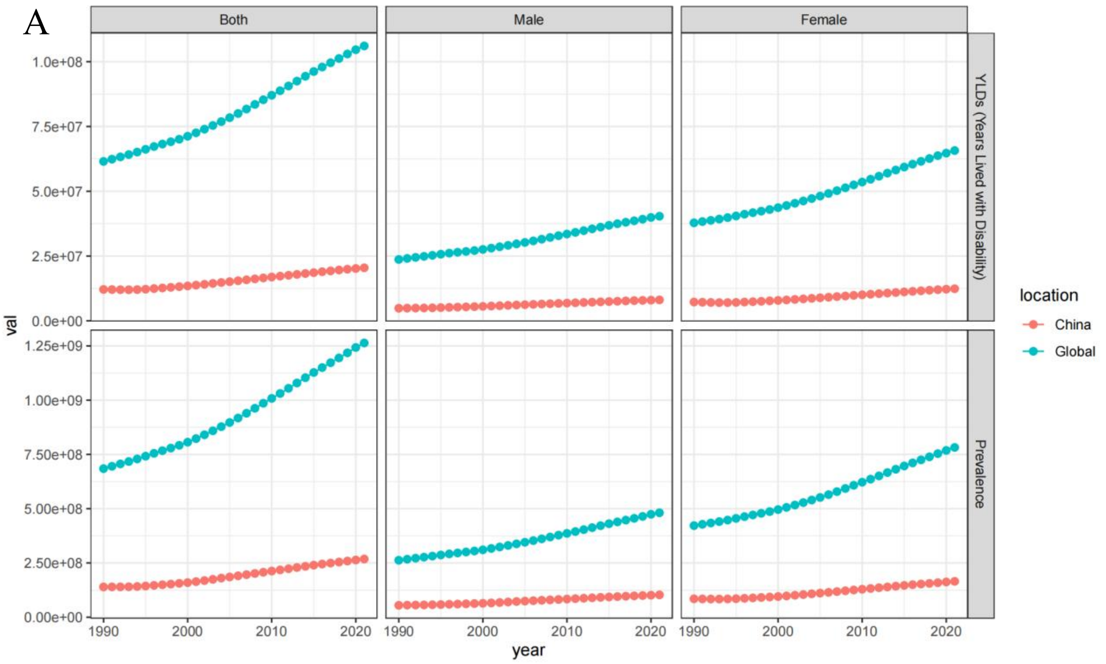

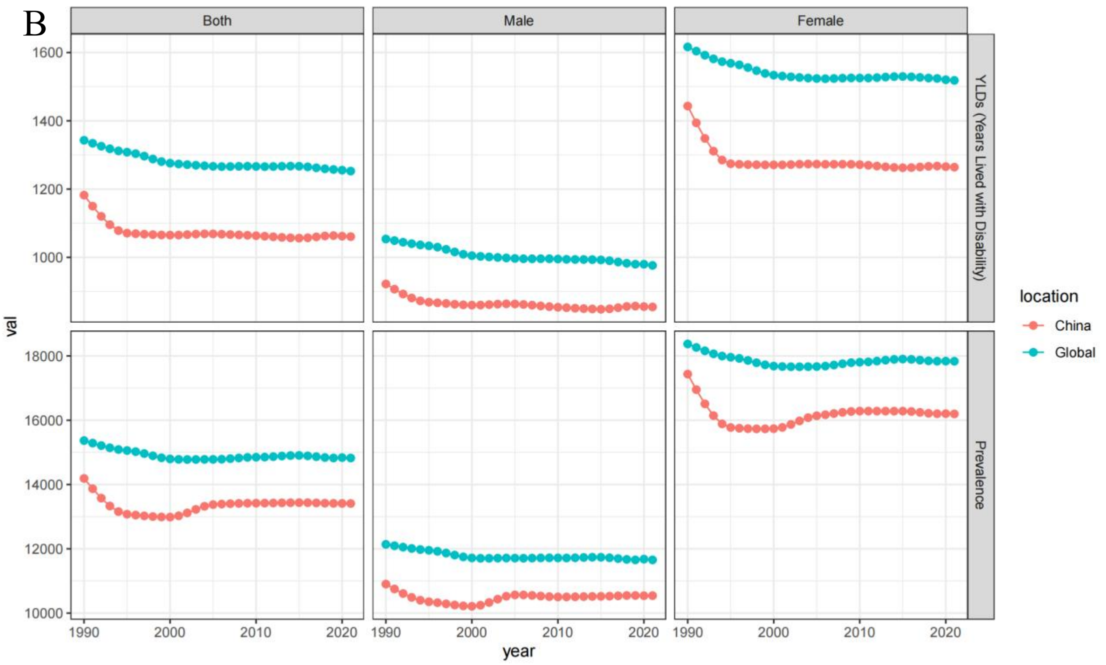

Fig. S1 (A) Trends in the number of prevalent cases and years lived with disability of musculoskeletal disorders from 1990 to 2021; (B) Trends in the age-standardized rates of prevalence and years lived with disability of musculoskeletal disorders from 1990 to 2021.

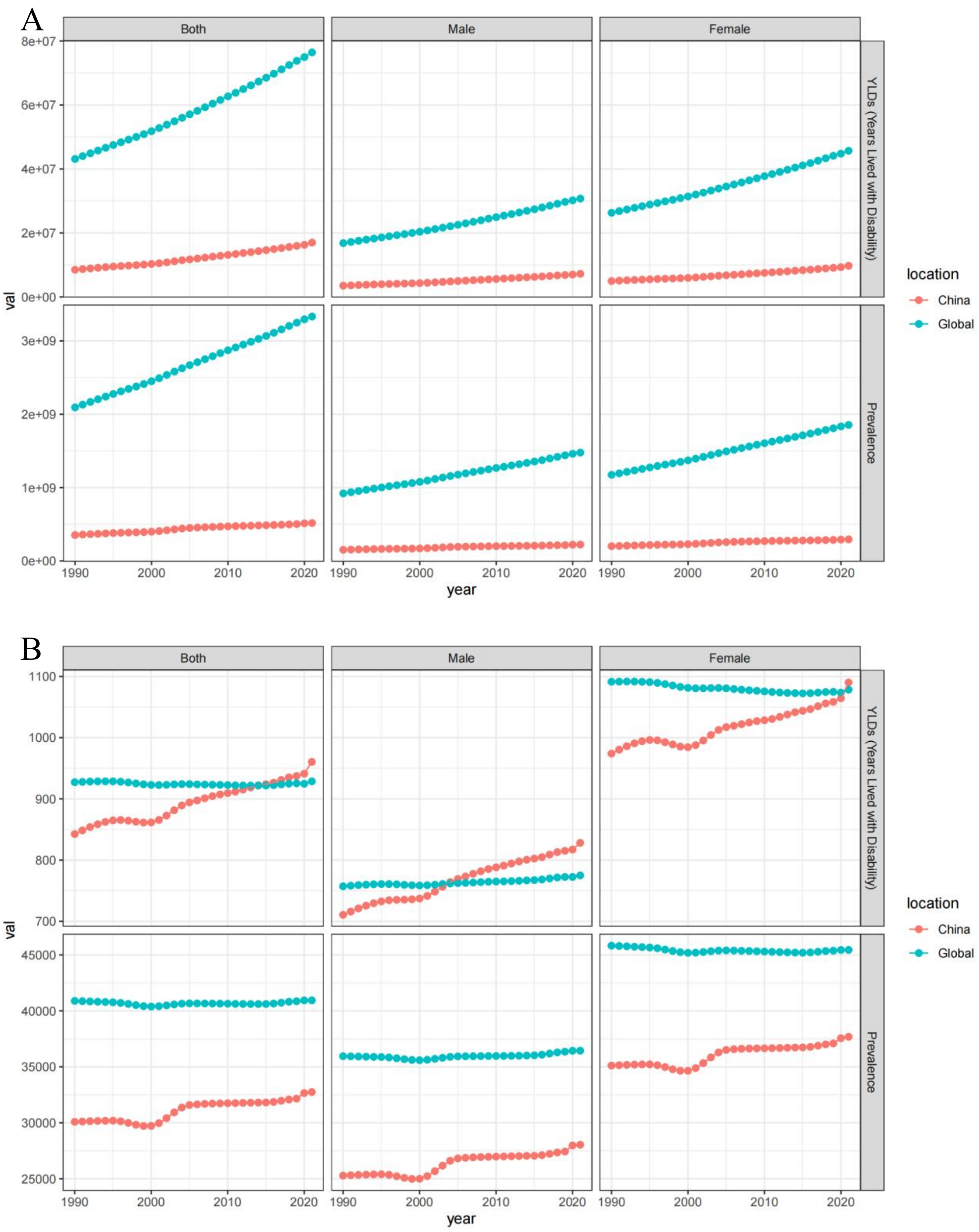

Fig. S2 (A) Trends in the number of prevalent cases and years lived with disability of neurological disorders from 1990 to 2021; (B) Trends in the age-standardized rates of prevalence and years lived with disability of neurological disorders from 1990 to 2021.

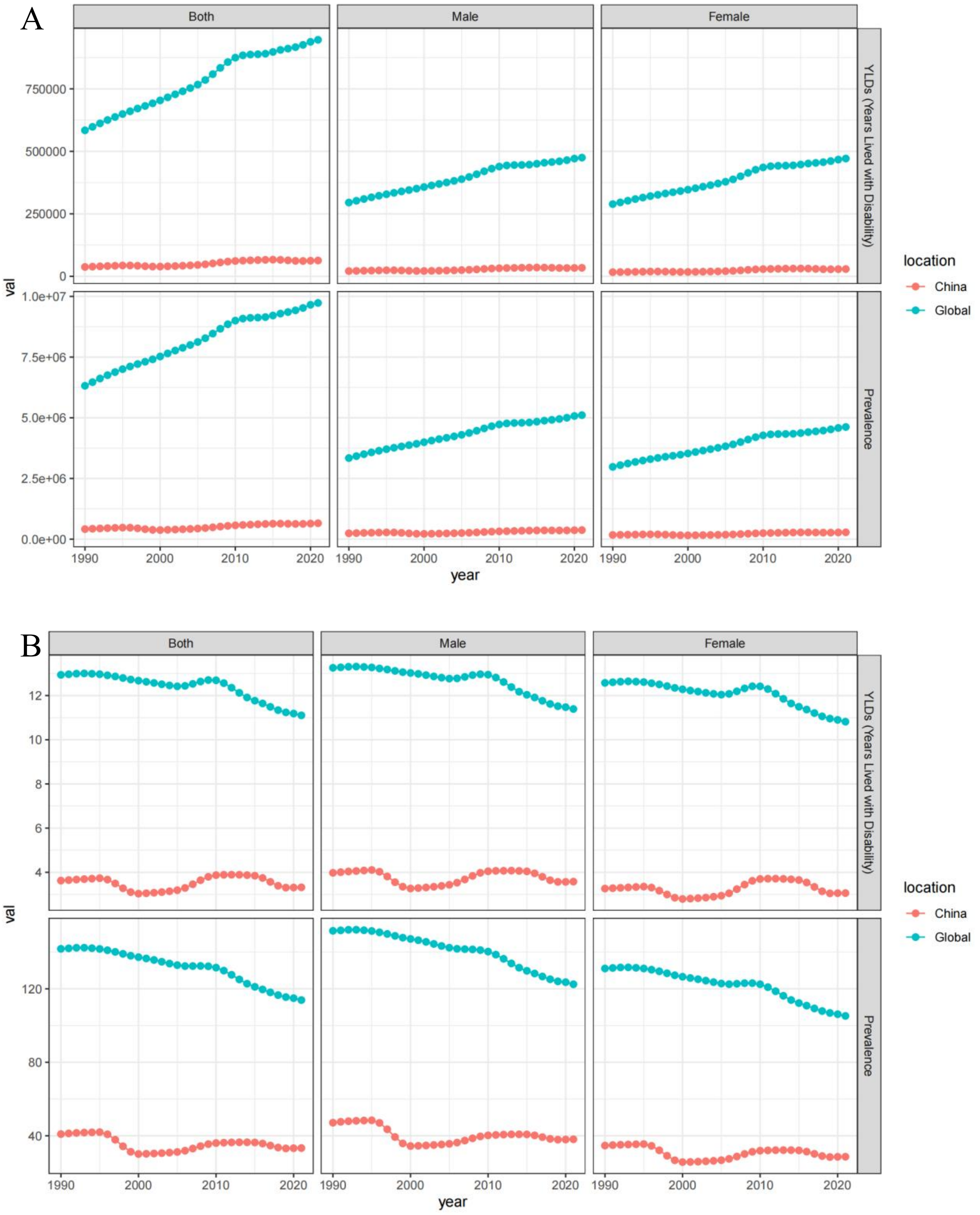

Fig. S3 (A) Trends in the number of prevalent cases and years lived with disability of digestive disorders from 1990 to 2021; (B) Trends in the age-standardized rates of prevalence and years lived with disability of digestive disorders from 1990 to 2021.

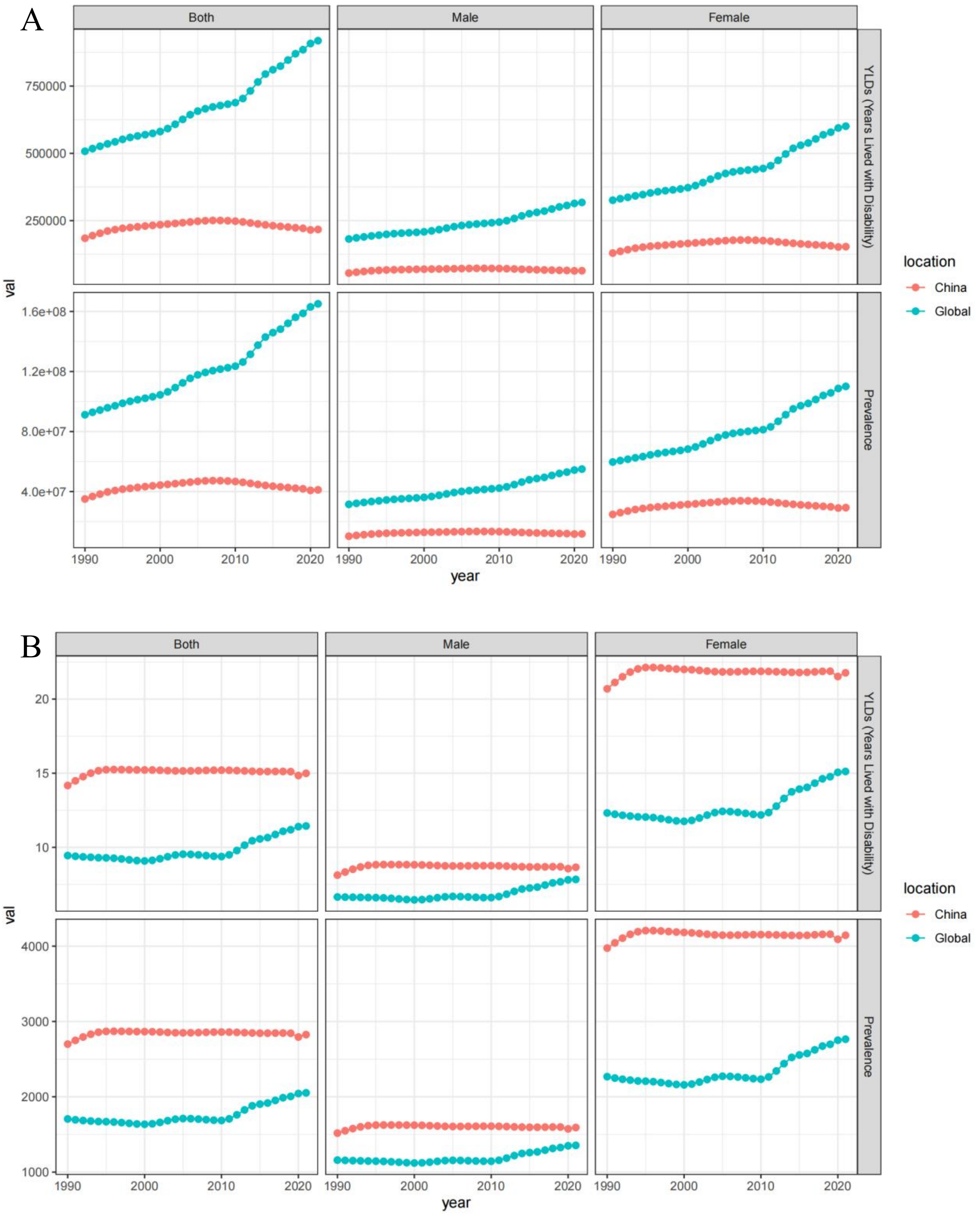

Fig. S4 (A) Trends in the number of prevalent cases and years lived with disability of genecological disorders from 1990 to 2021; (B) Trends in the age-standardized rates of prevalence and years lived with disability of genecological disorders from 1990 to 2021.

A

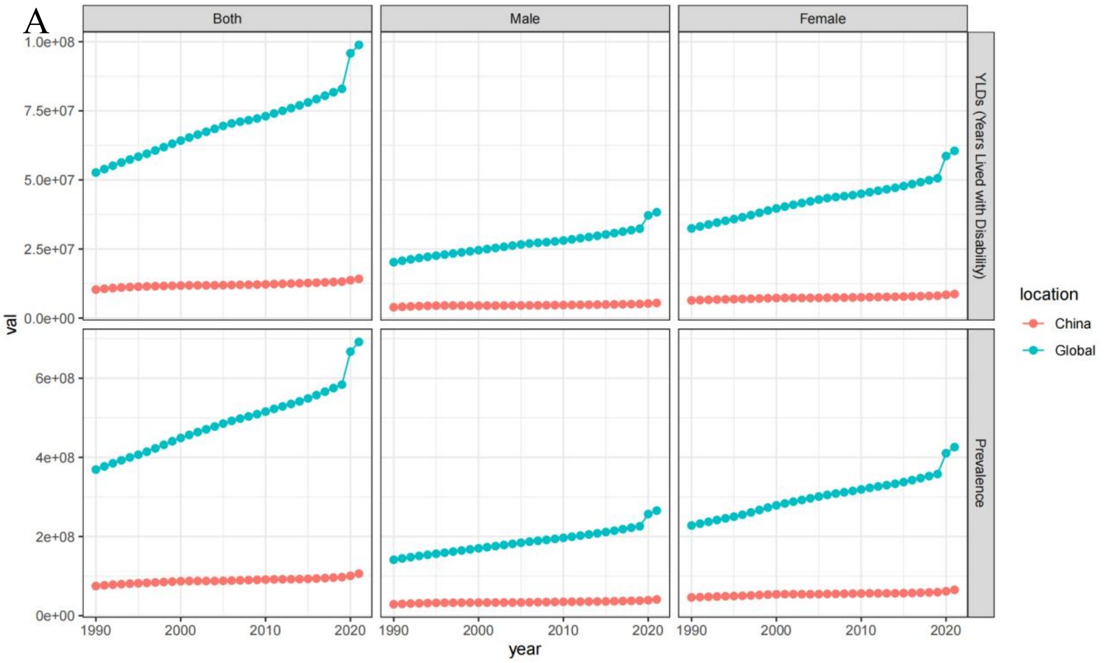

B

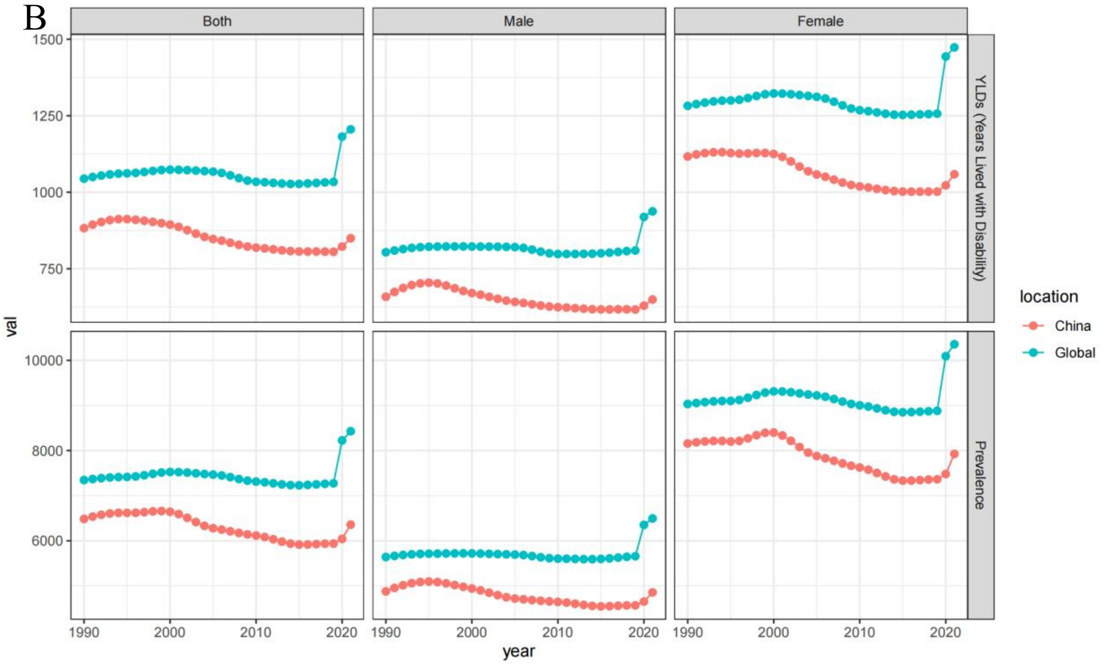

Fig. S5 (A) Trends in the number of prevalent cases and years lived with disability of mental disorders from 1990 to 2021; (B) Trends in the age-standardized rates of prevalence and years lived with disability of mental disorders from 1990 to 2021.

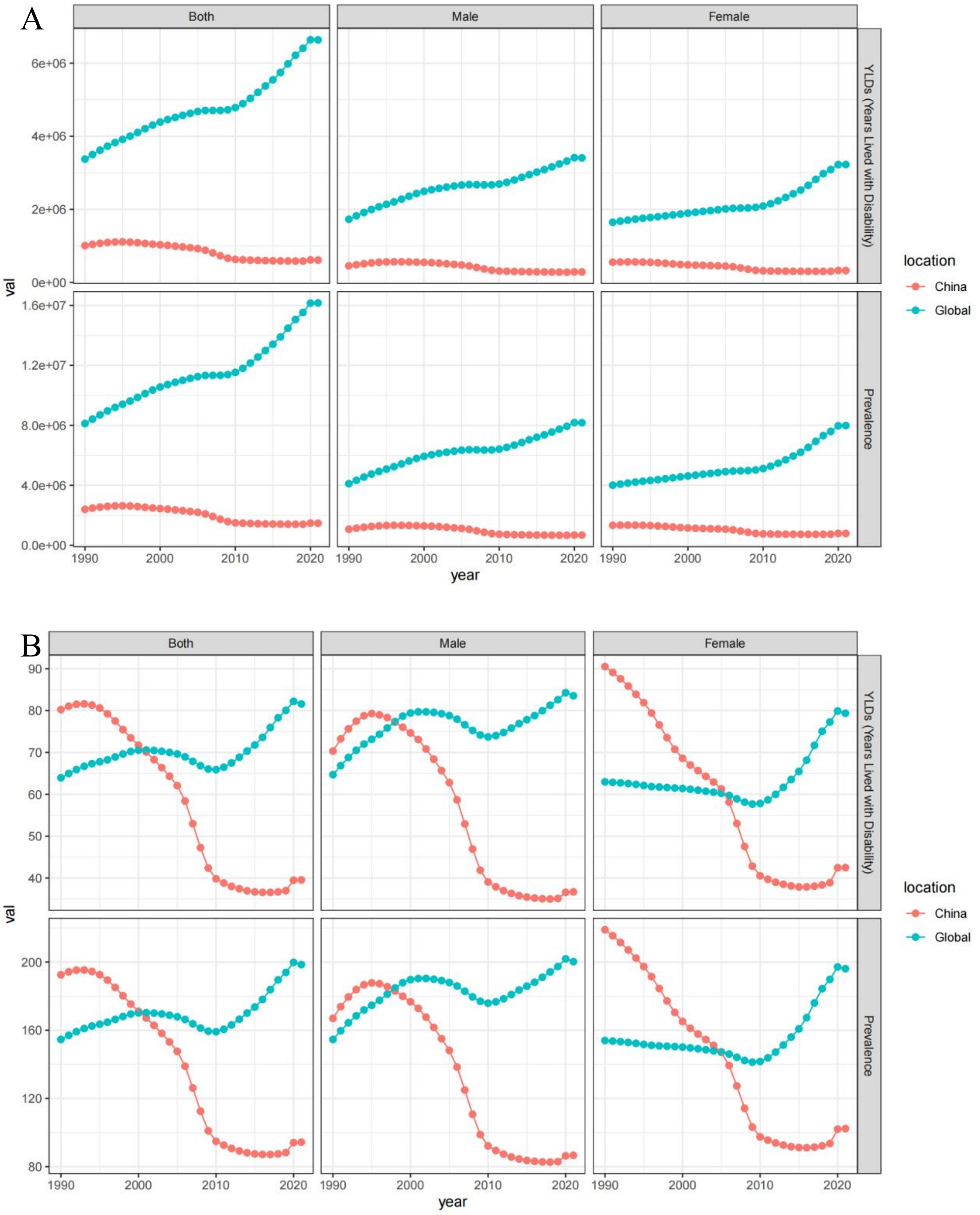

Fig. S6 (A) Trends in the number of prevalent cases and years lived with disability of substance use disorders from 1990 to 2021; (B) Trends in the age-standardized rates of prevalence and years lived with disability of substance use disorders from 1990 to 2021.

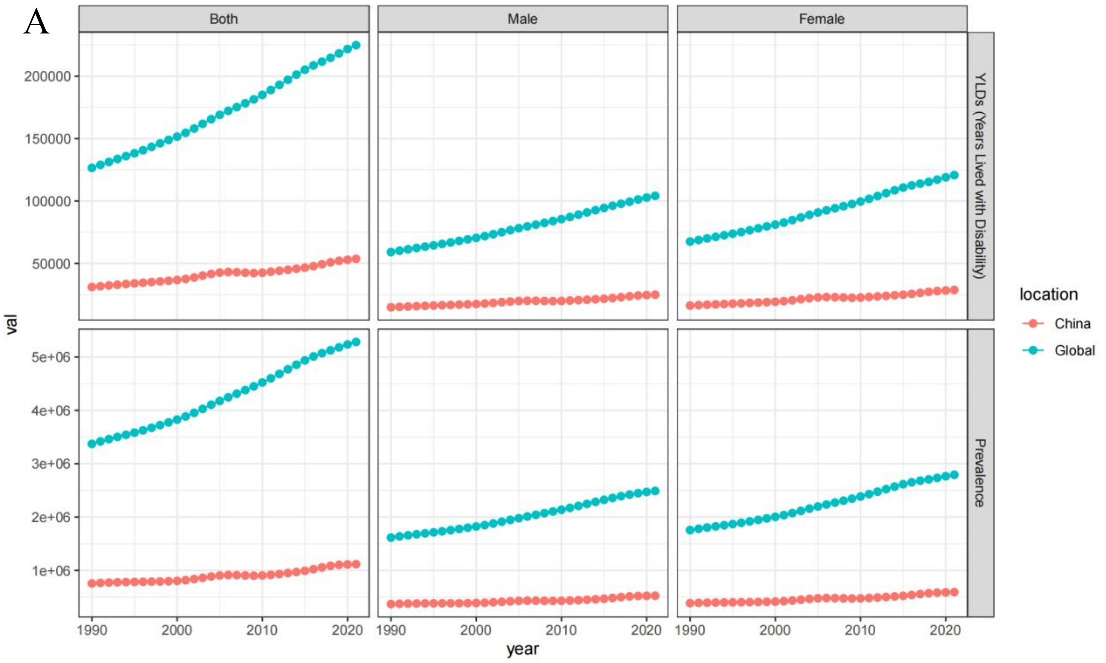

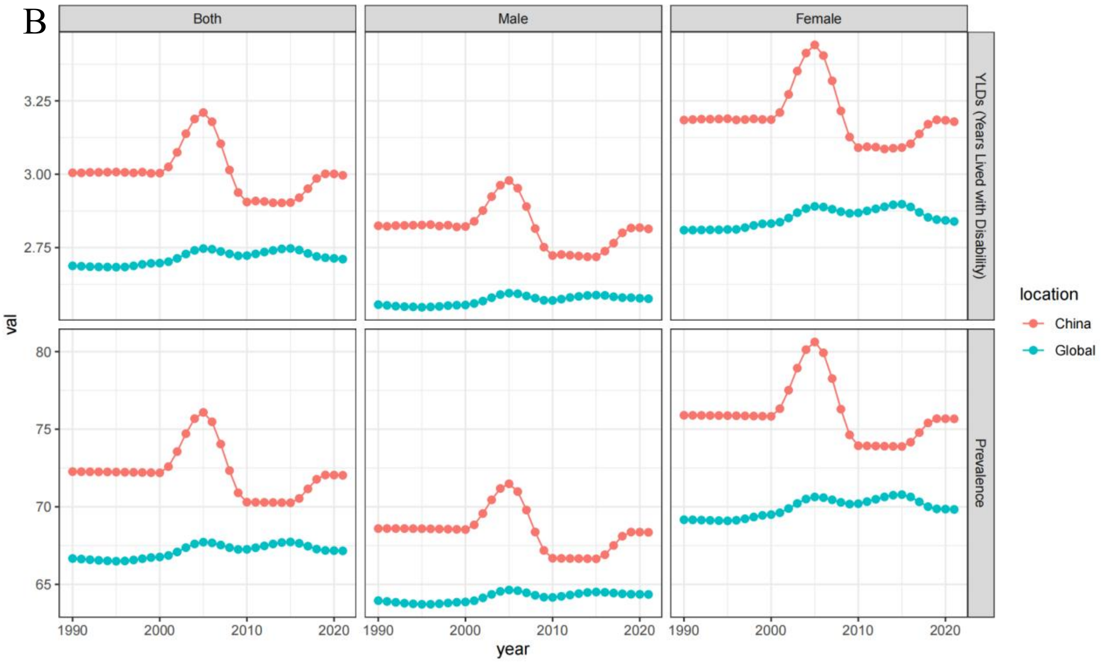

Fig. S7 (A) Trends in the number of prevalent cases and years lived with disability of infectious disorders from 1990 to 2021; (B) Trends in the age-standardized rates of prevalence and years lived with disability of infectious disorders from 1990 to 2021.

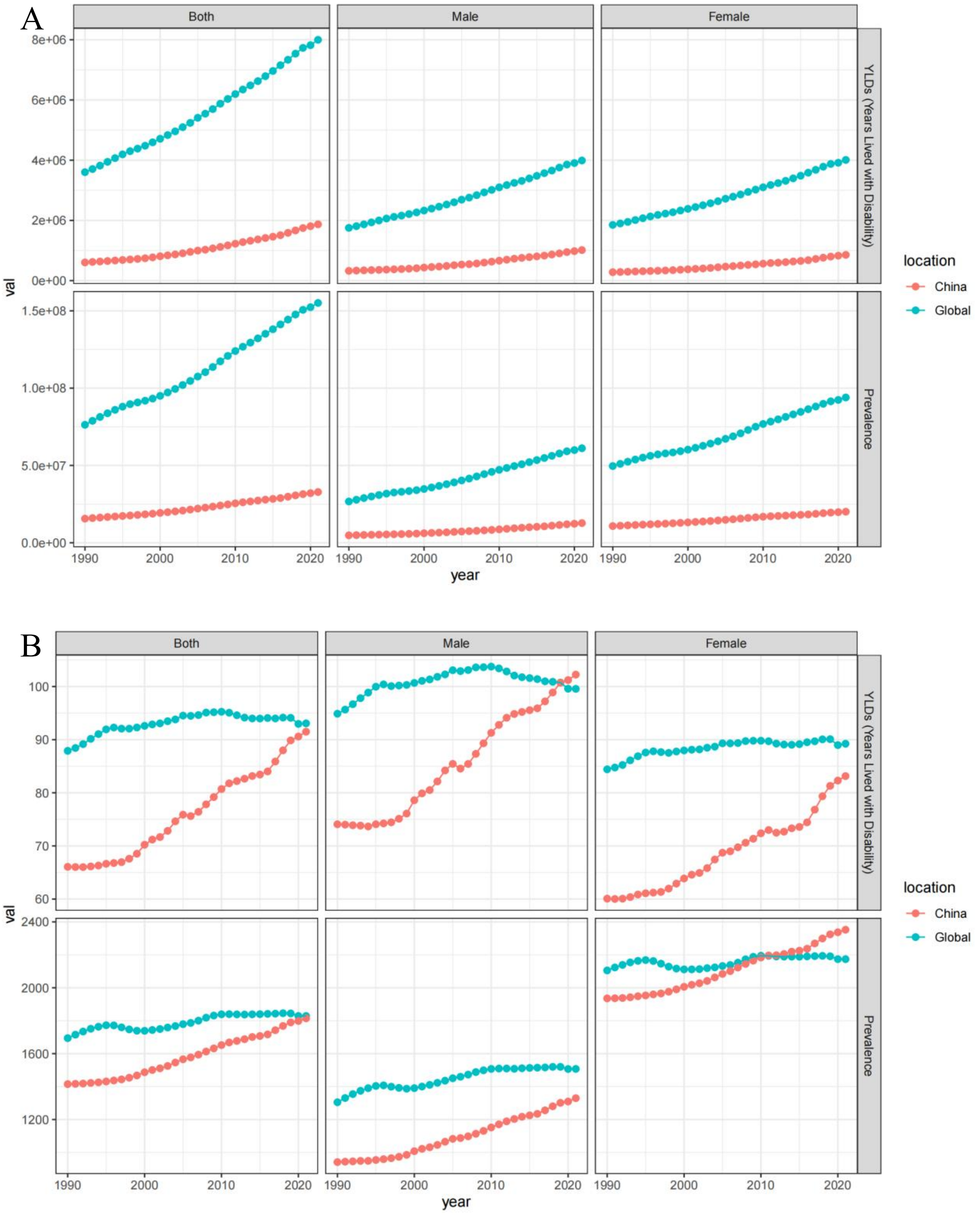

Fig. S8 (A) Trends in the number of prevalent cases and years lived with disability of neoplasms from 1990 to 2021; (B) Trends in the age-standardized rates of prevalence and years lived with disability of neoplasms from 1990 to 2021.

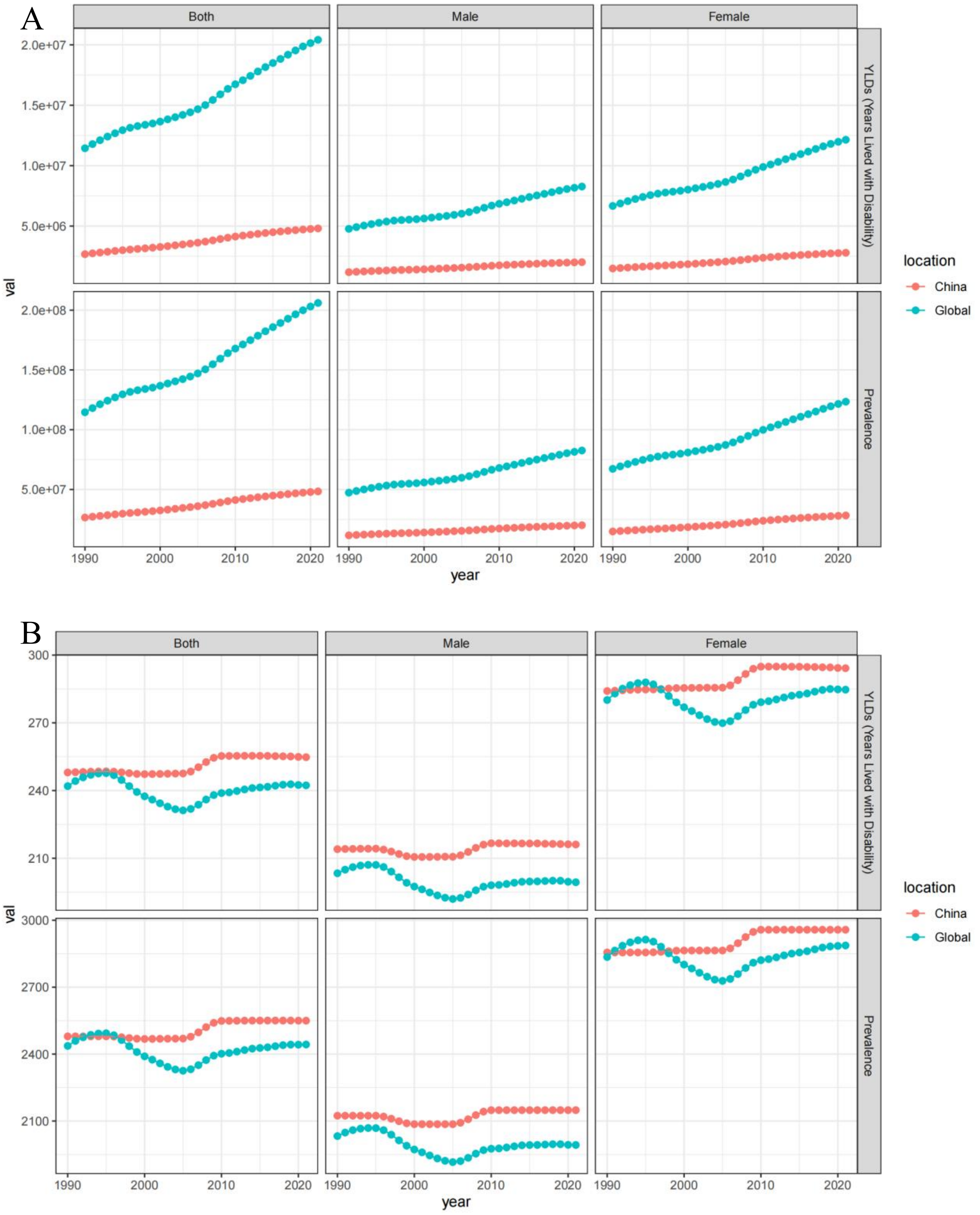

Fig. S9 (A) Trends in the number of prevalent cases and years lived with disability of neck pain from 1990 to 2021; (B) Trends in the age-standardized rates of prevalence and years lived with disability of neck pain from 1990 to 2021.

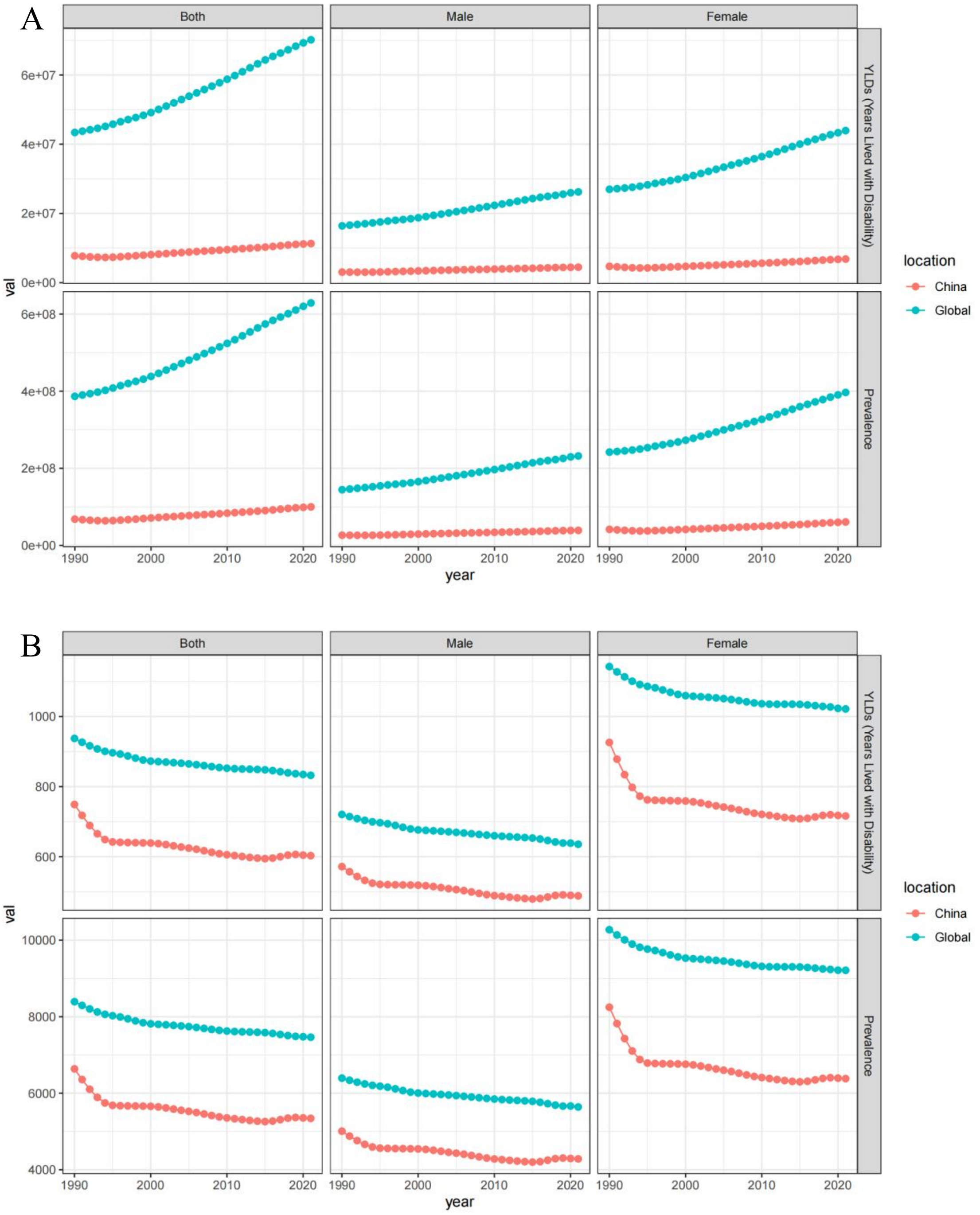

Fig. S10 (A) Trends in the number of prevalent cases and years lived with disability of low back pain from 1990 to 2021; (B) Trends in the age-standardized rates of prevalence and years lived with disability of low back pain from 1990 to 2021.

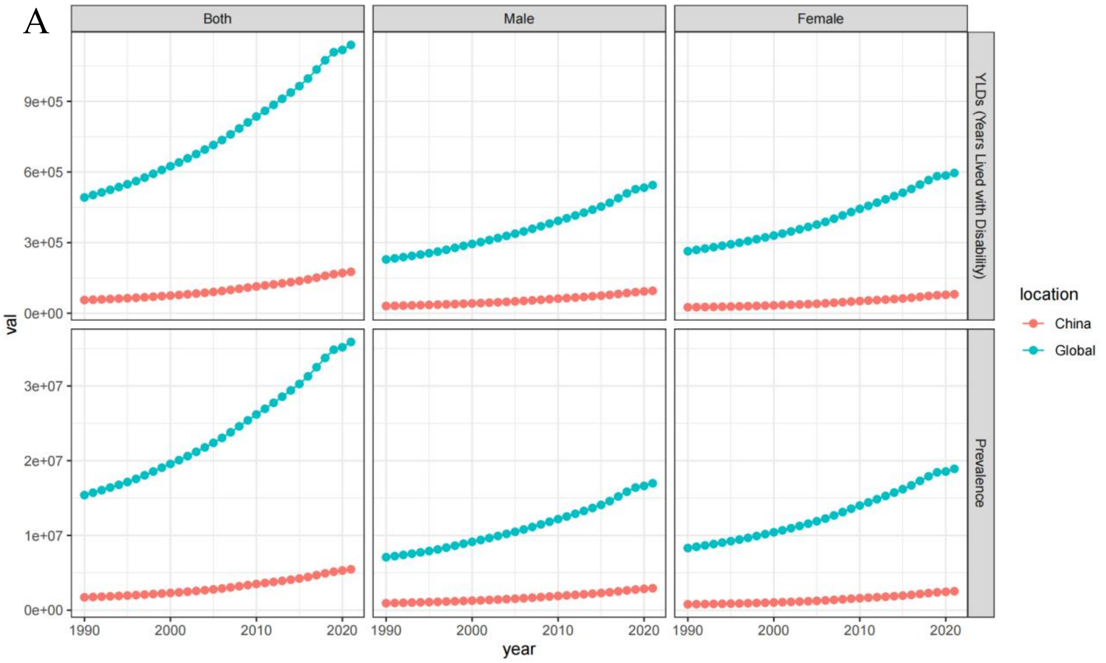

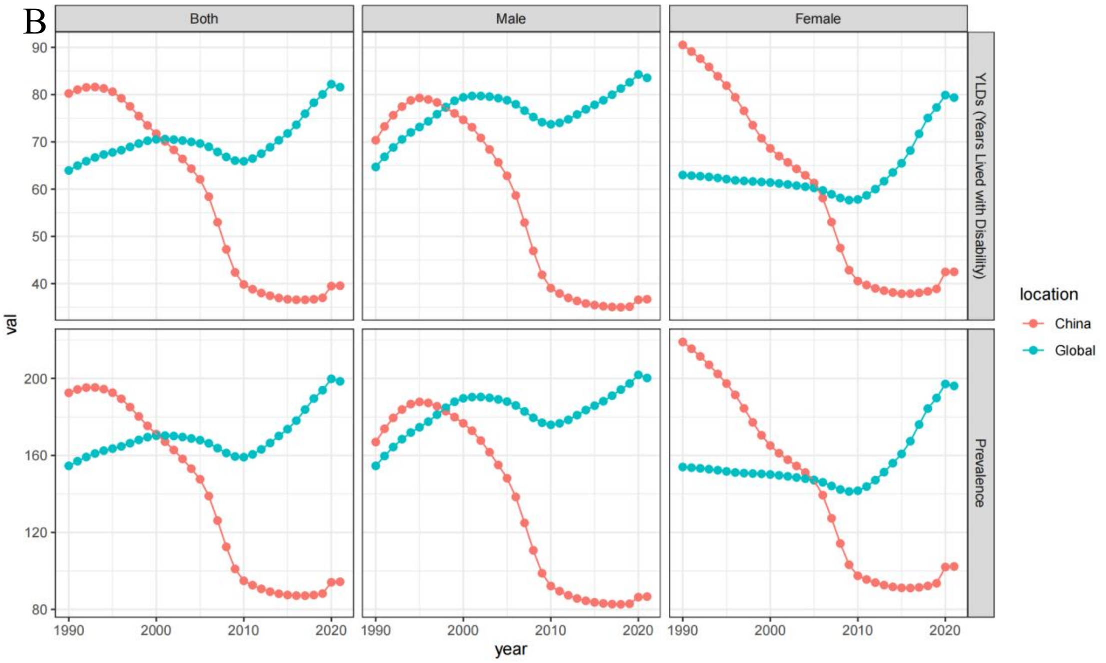

Fig. S11 (A) Trends in the number of prevalent cases and years lived with disability of hip osteoarthritis from 1990 to 2021; (B) Trends in the age-standardized rates of prevalence and years lived with disability of hip osteoarthritis from 1990 to 2021.

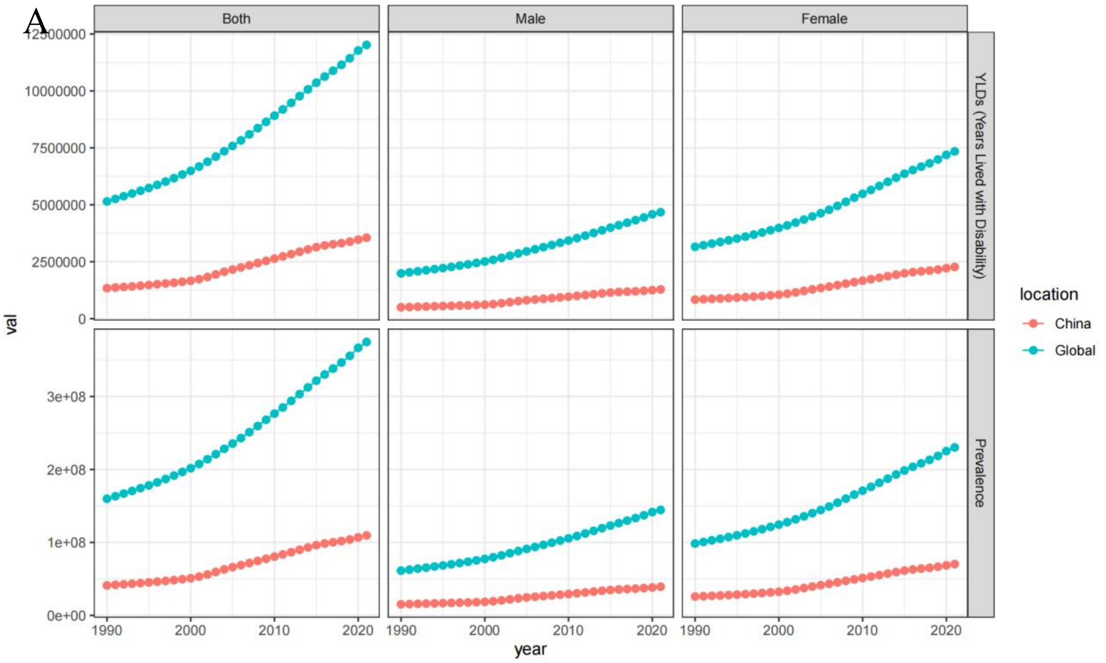

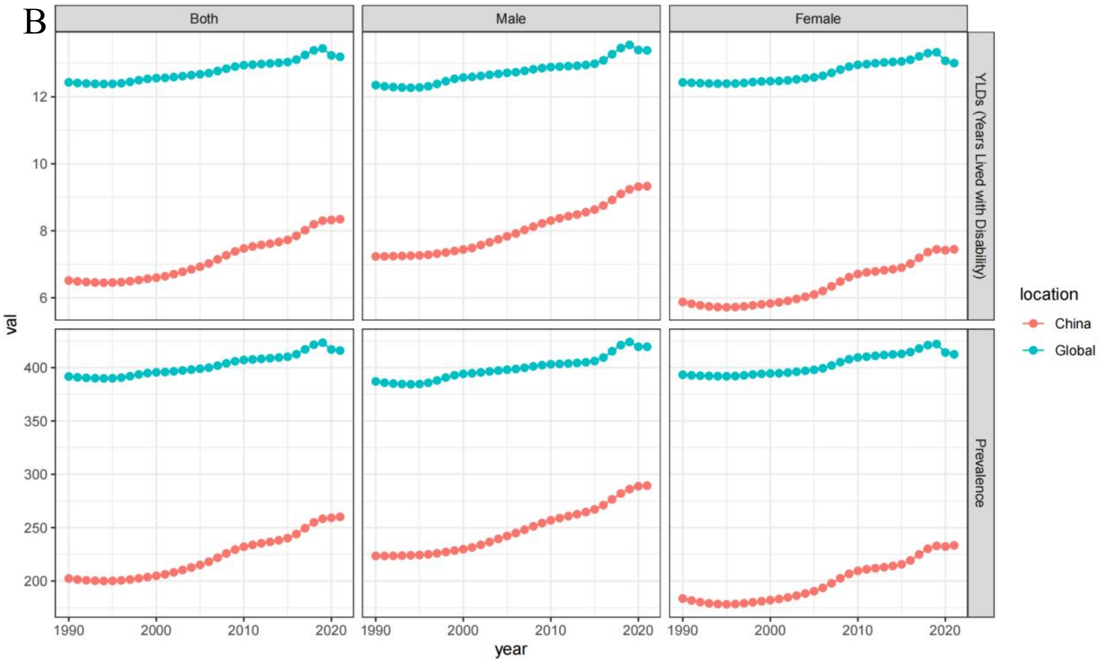

Fig. S12 (A) Trends in the number of prevalent cases and years lived with disability of knee osteoarthritis from 1990 to 2021; (B) Trends in the age-standardized rates of prevalence and years lived with disability of knee osteoarthritis from 1990 to 2021.

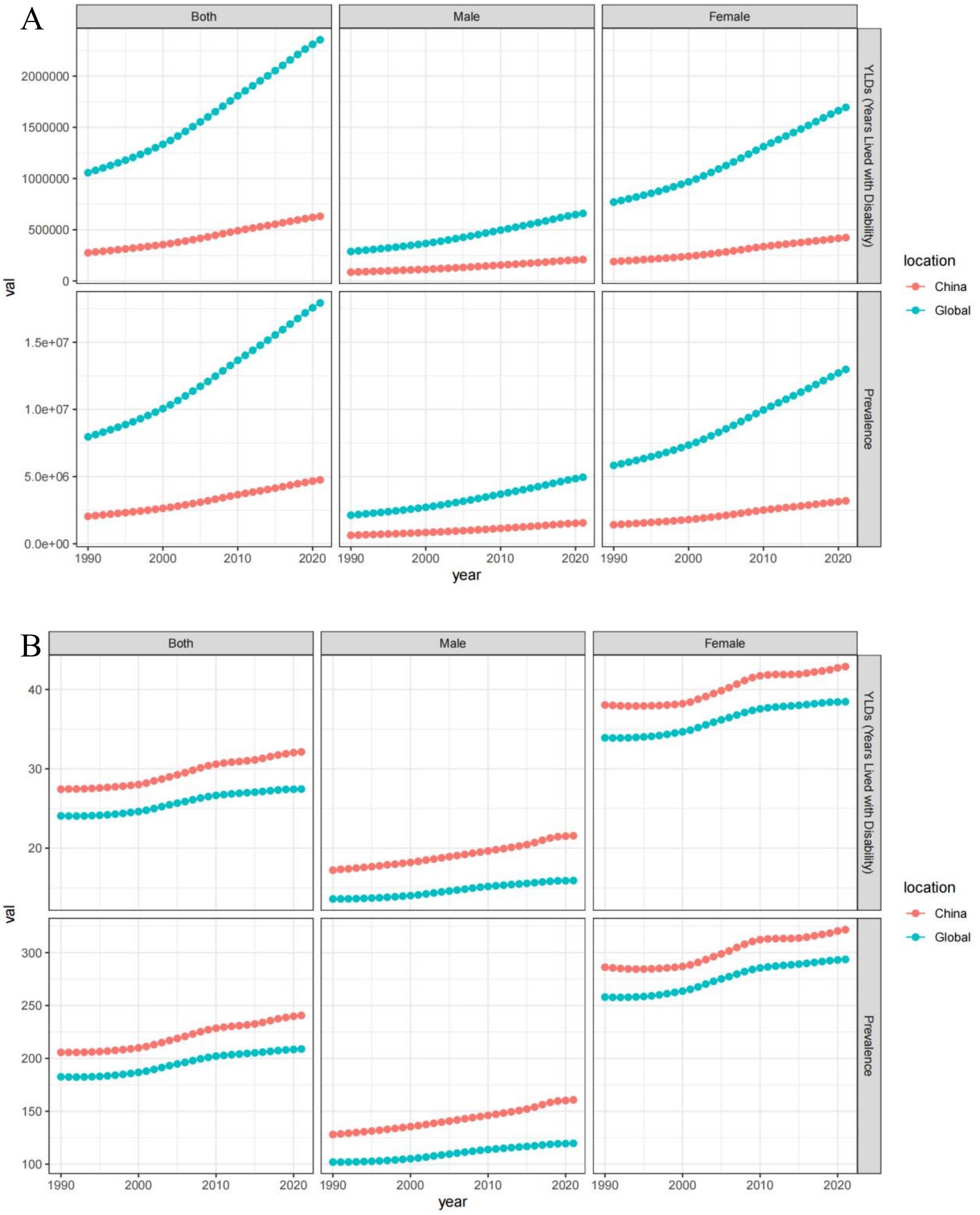

Fig. S13 (A) Trends in the number of prevalent cases and years lived with disability of rheumatoid arthritis from 1990 to 2021; (B) Trends in the age-standardized rates of prevalence and years lived with disability of rheumatoid arthritis from 1990 to 2021.

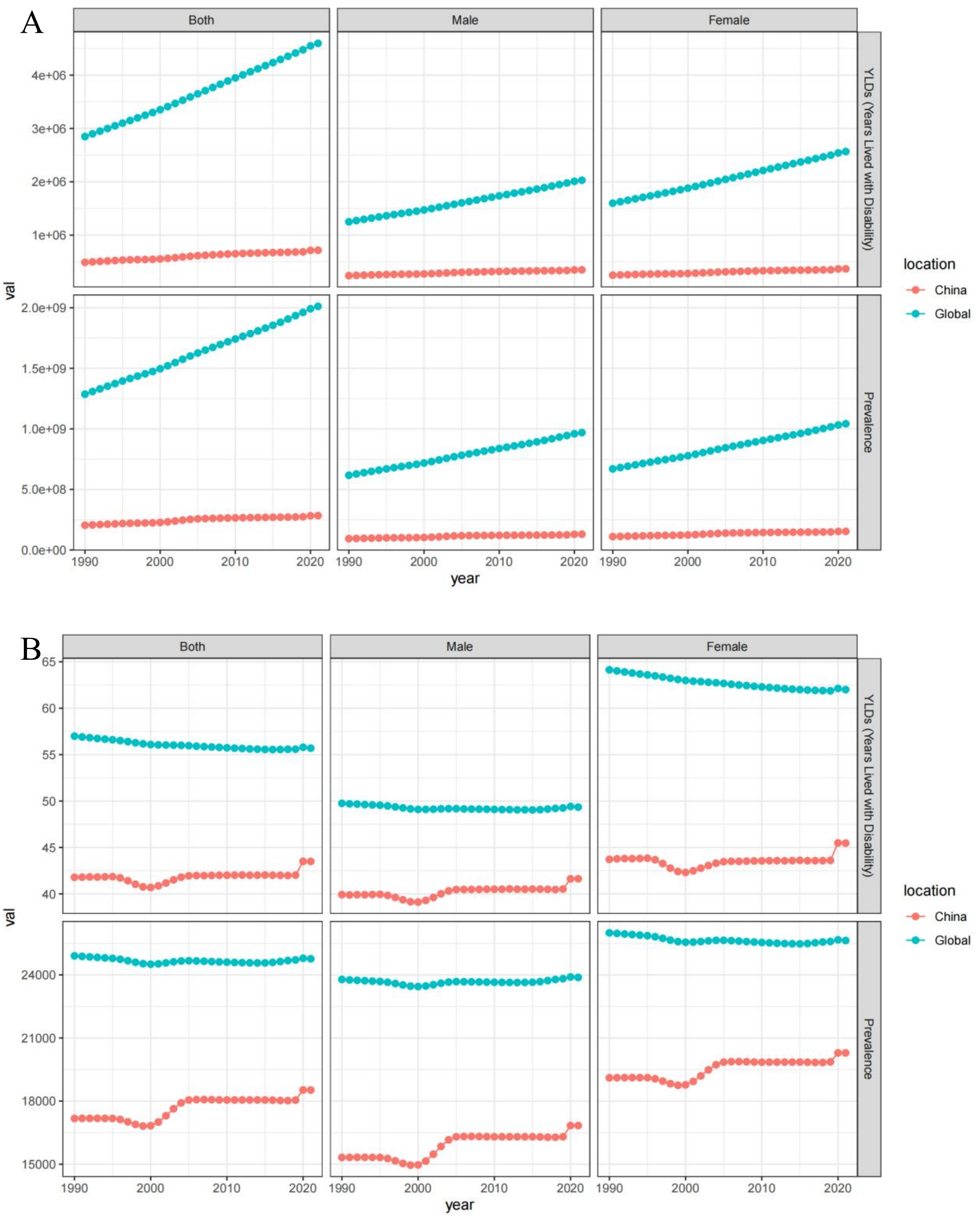

Fig. S14 (A) Trends in the number of prevalent cases and years lived with disability of tension-type headache from 1990 to 2021; (B) Trends in the age-standardized rates of prevalence and years lived with disability of tension-type headache from 1990 to 2021.

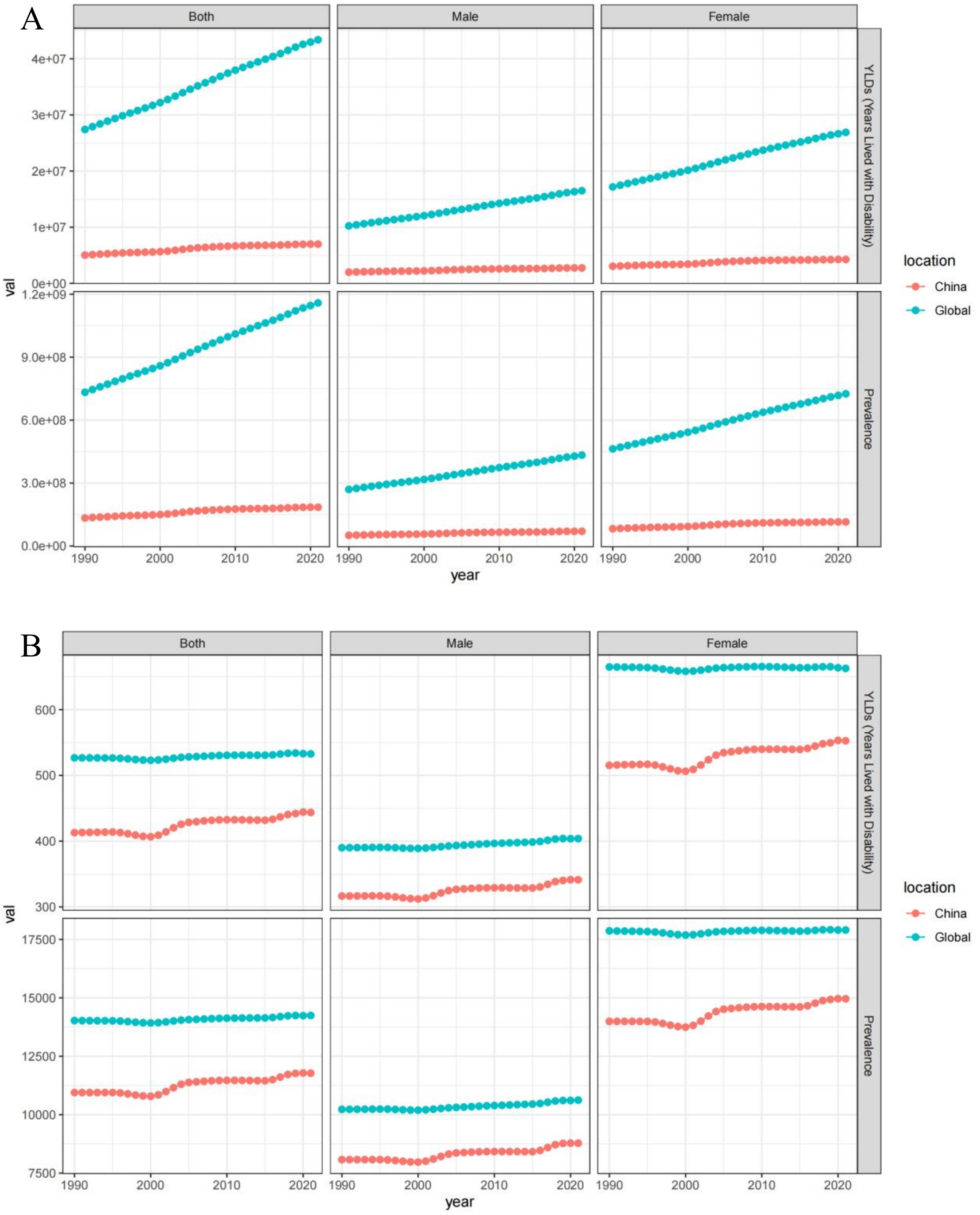

Fig. S15 (A) Trends in the number of prevalent cases and years lived with disability of migraine from 1990 to 2021; (B) Trends in the age-standardized rates of prevalence and years lived with disability of migraine from 1990 to 2021.

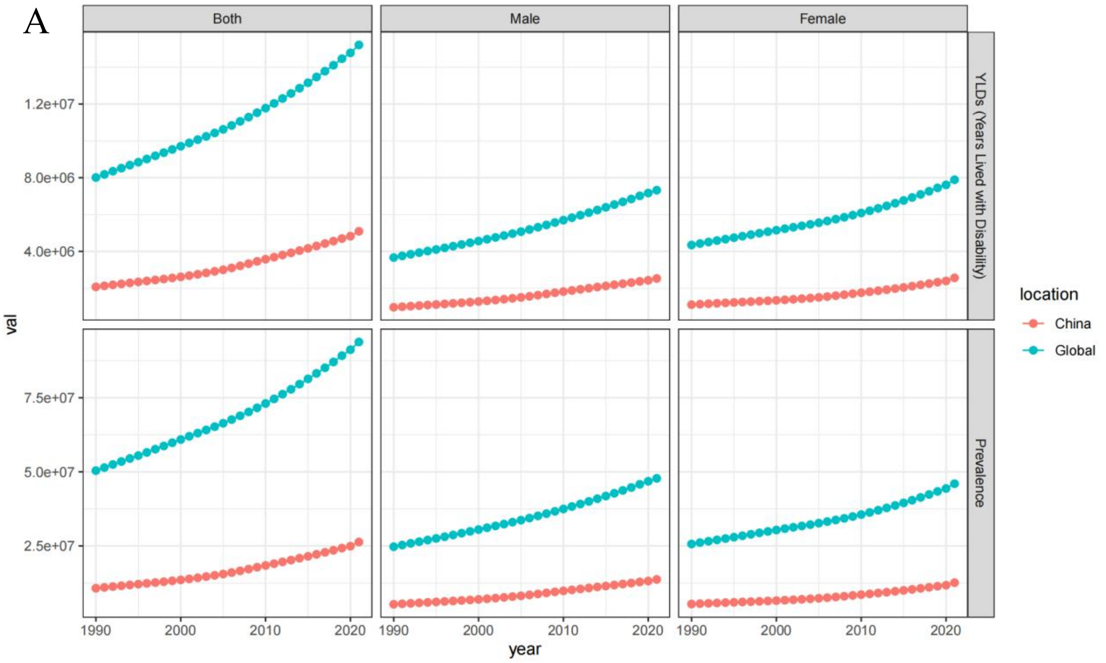

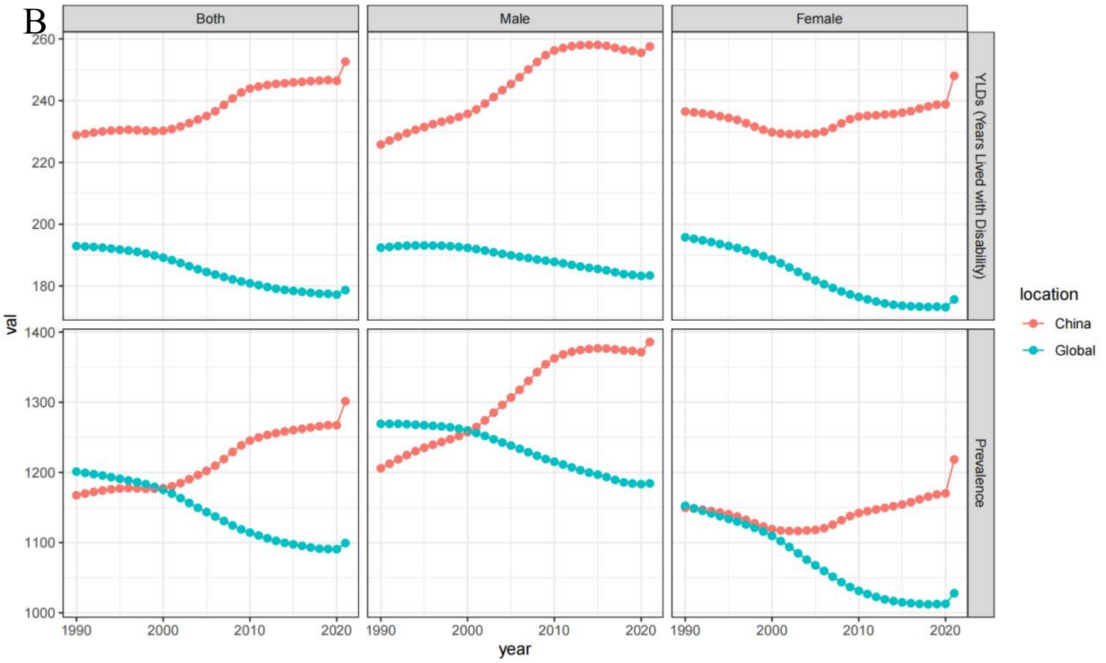

Fig. S16 (A) Trends in the number of prevalent cases and years lived with disability of stroke from 1990 to 2021; (B) Trends in the age-standardized rates of prevalence and years lived with disability of stroke from 1990 to 2021.

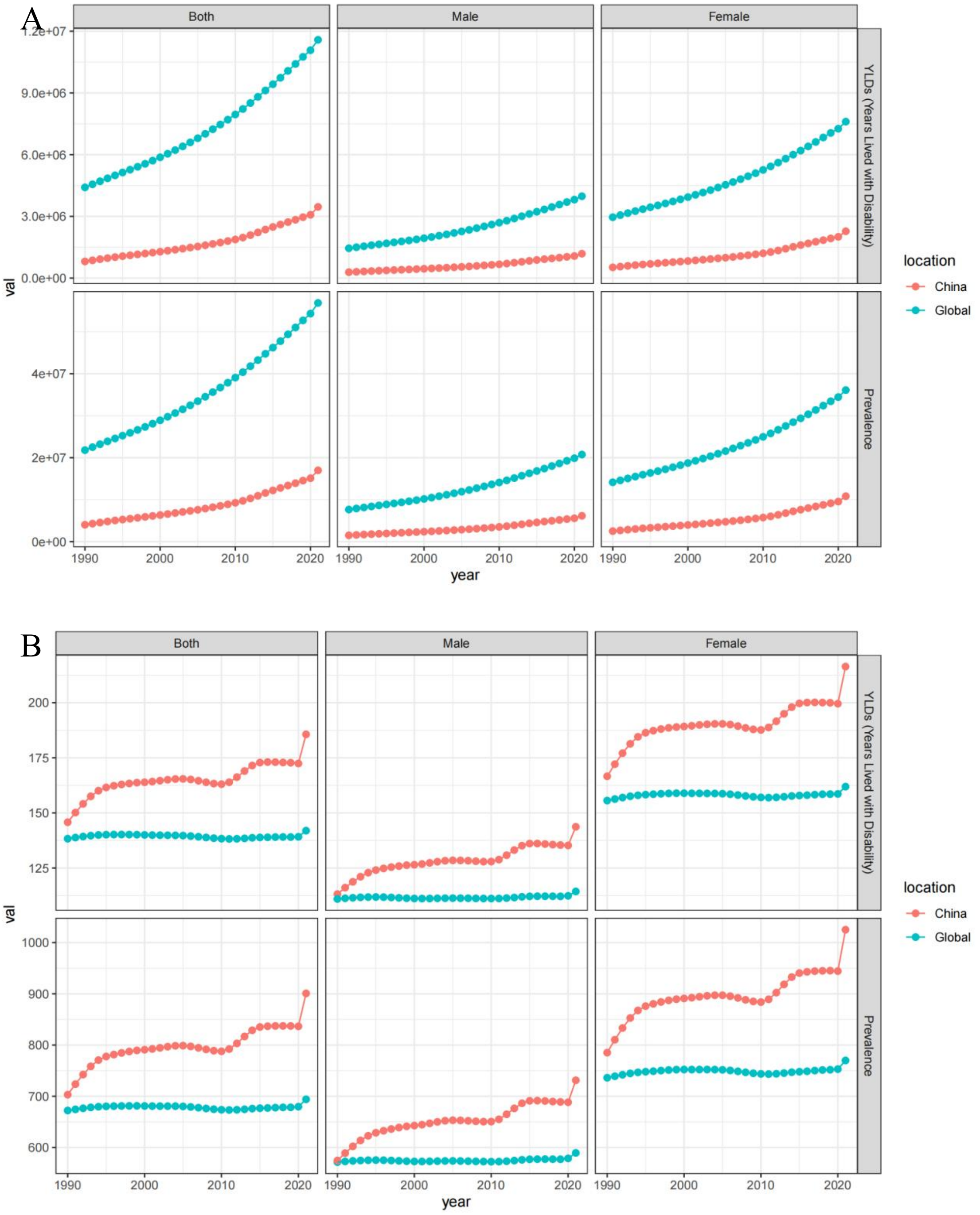

Fig. S17 (A) Trends in the number of prevalent cases and years lived with disability of Alzheimer's disease and other dementias from 1990 to 2021; (B) Trends in the age-standardized rates of prevalence and years lived with disability of Alzheimer's disease and other dementias from 1990 to 2021.

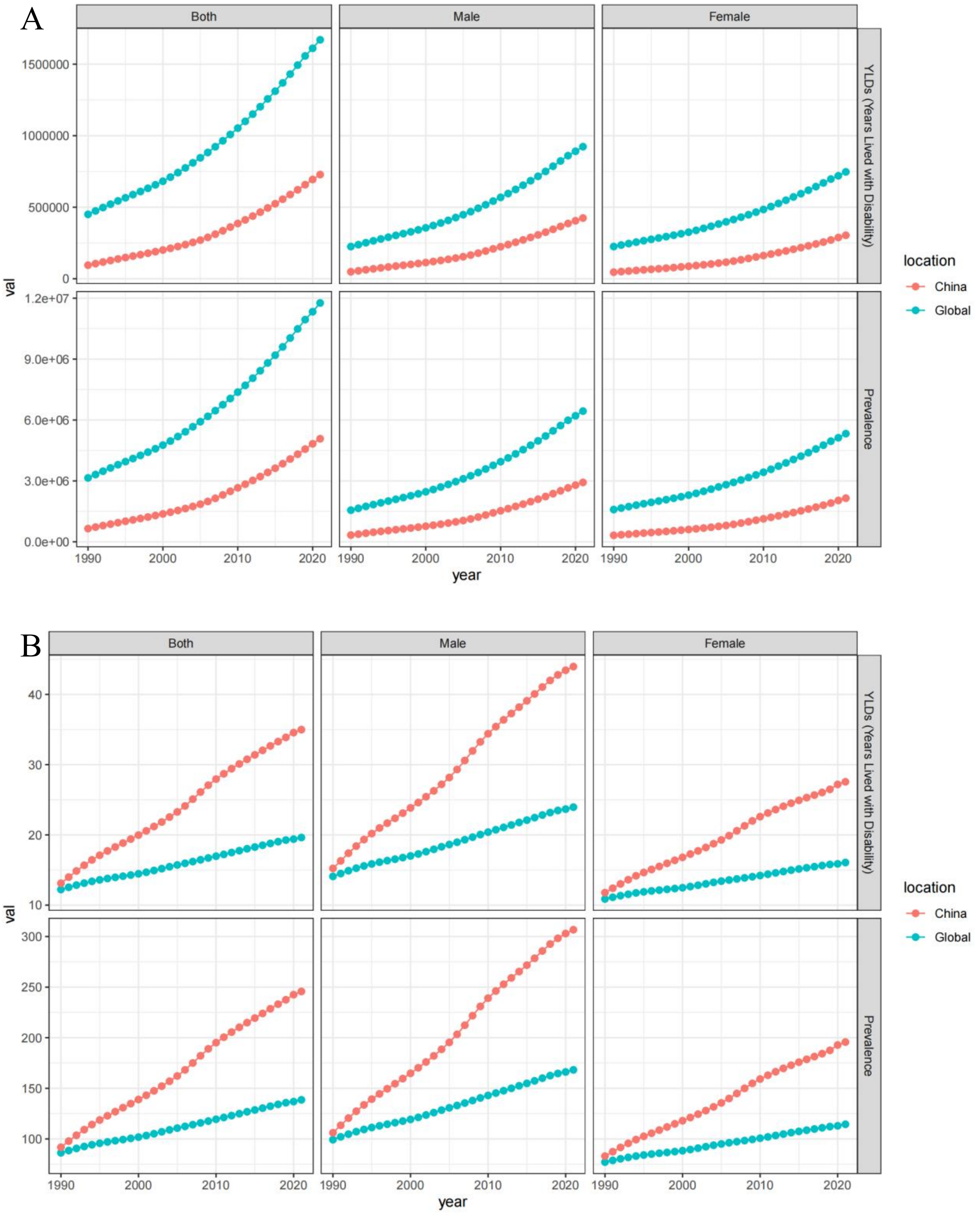

Fig. S18 (A) Trends in the number of prevalent cases and years lived with disability of Parkinson's disease from 1990 to 2021; (B) Trends in the age-standardized rates of prevalence and years lived with disability of Parkinson's disease from 1990 to 2021.

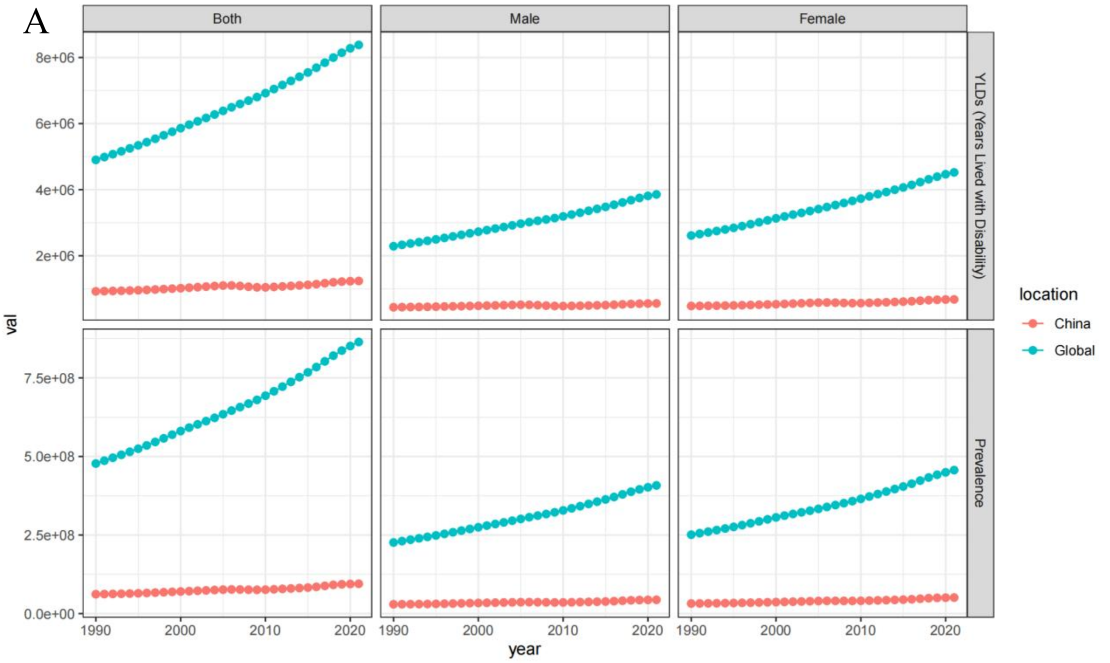

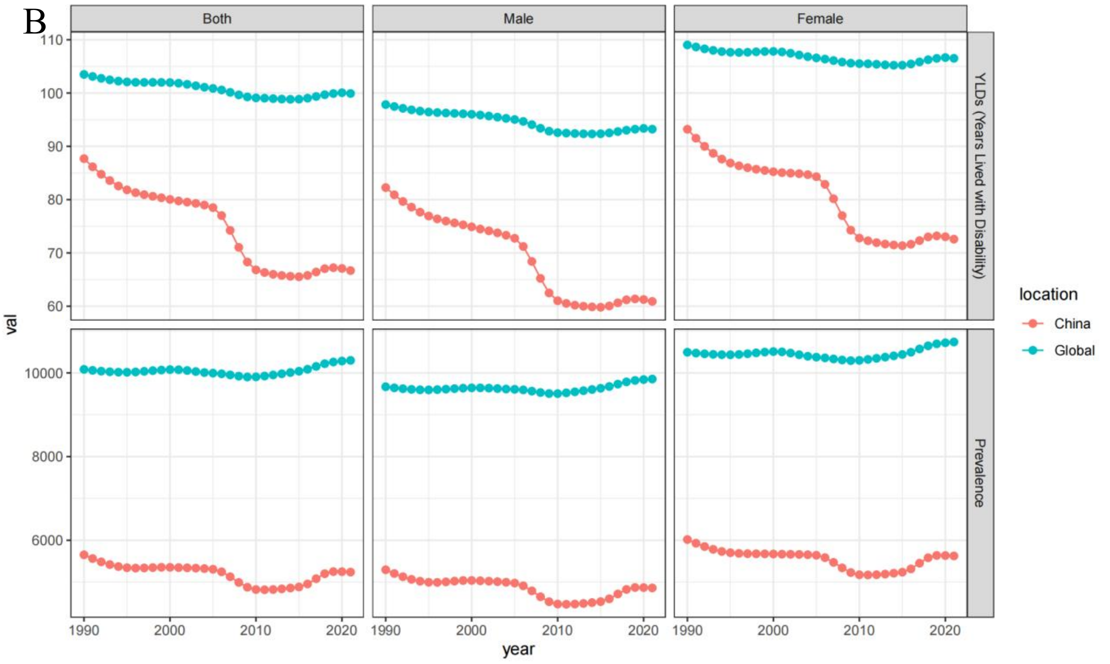

Fig. S19 (A) Trends in the number of prevalent cases and years lived with disability of upper digestive system diseases from 1990 to 2021; (B) Trends in the age-standardized rates of prevalence and years lived with disability of upper digestive system diseases from 1990 to 2021.

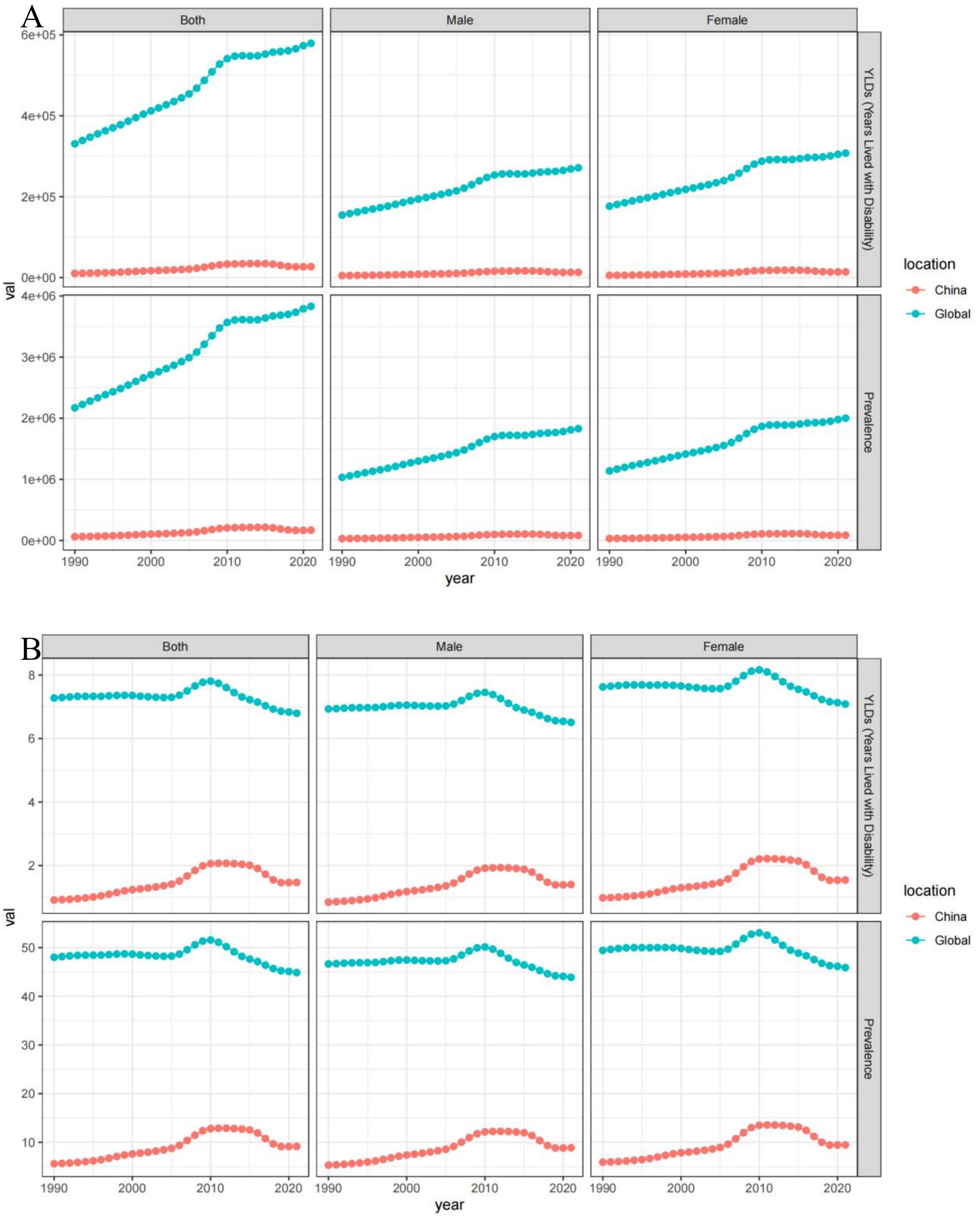

Fig. S20 (A) Trends in the number of prevalent cases and years lived with disability of inflammatory bowel disease from 1990 to 2021; (B) Trends in the age-standardized rates of prevalence and years lived with disability of inflammatory bowel disease from 1990 to 2021.

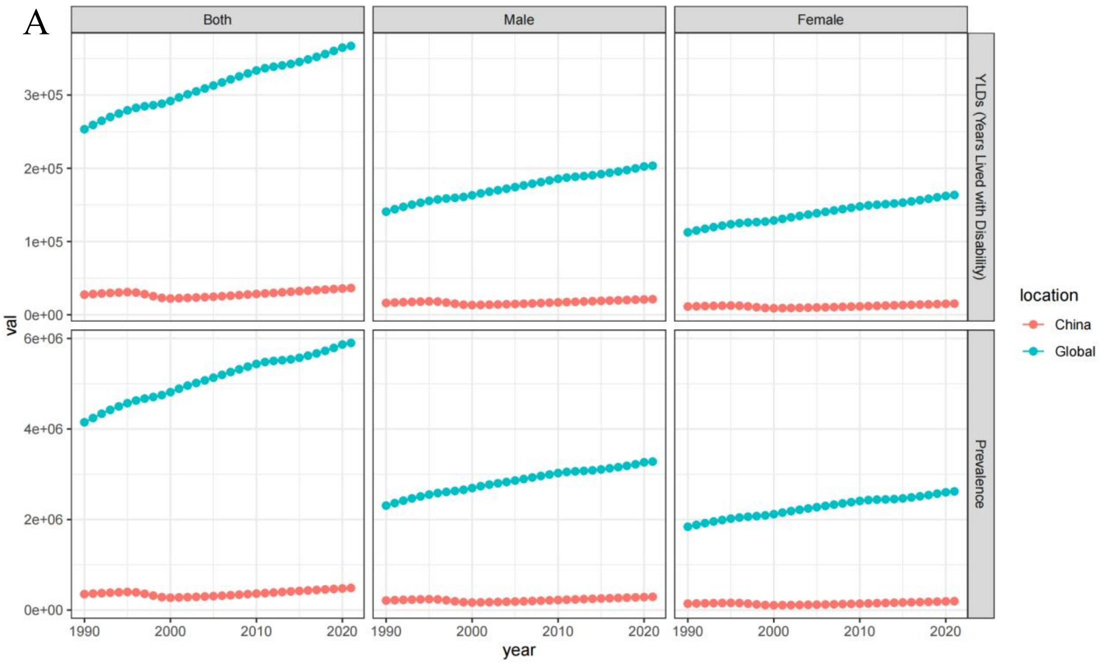

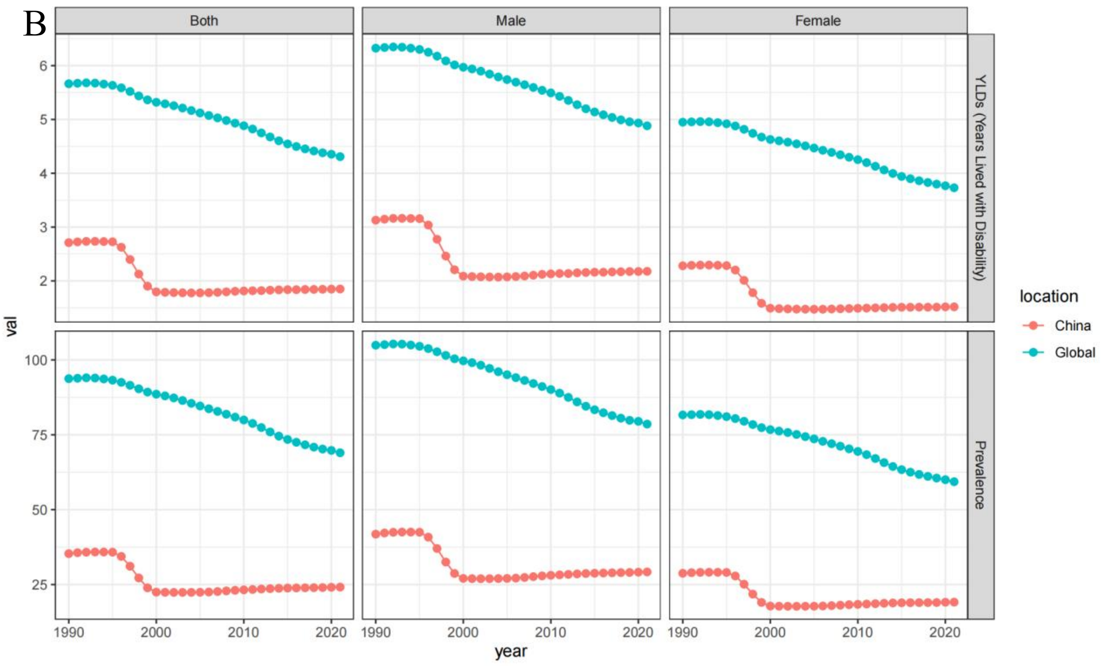

Fig. S21 (A) Trends in the number of prevalent cases and years lived with disability of pancreatitis from 1990 to 2021; (B) Trends in the age-standardized rates of prevalence and years lived with disability of pancreatitis from 1990 to 2021.

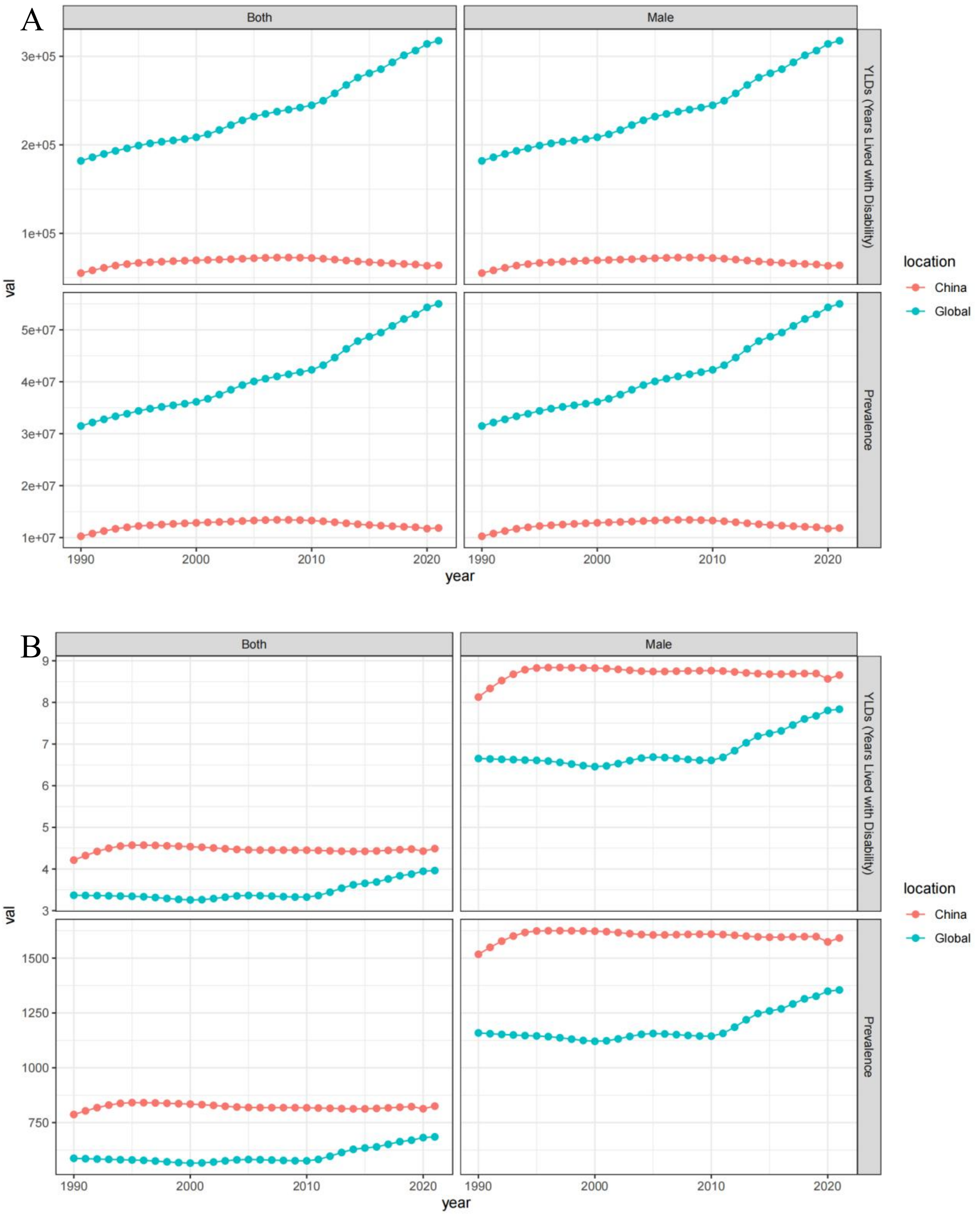

Fig. S22 (A) Trends in the number of prevalent cases and years lived with disability of male infertility from 1990 to 2021; (B) Trends in the age-standardized rates of prevalence and years lived with disability of male infertility from 1990 to 2021.

A
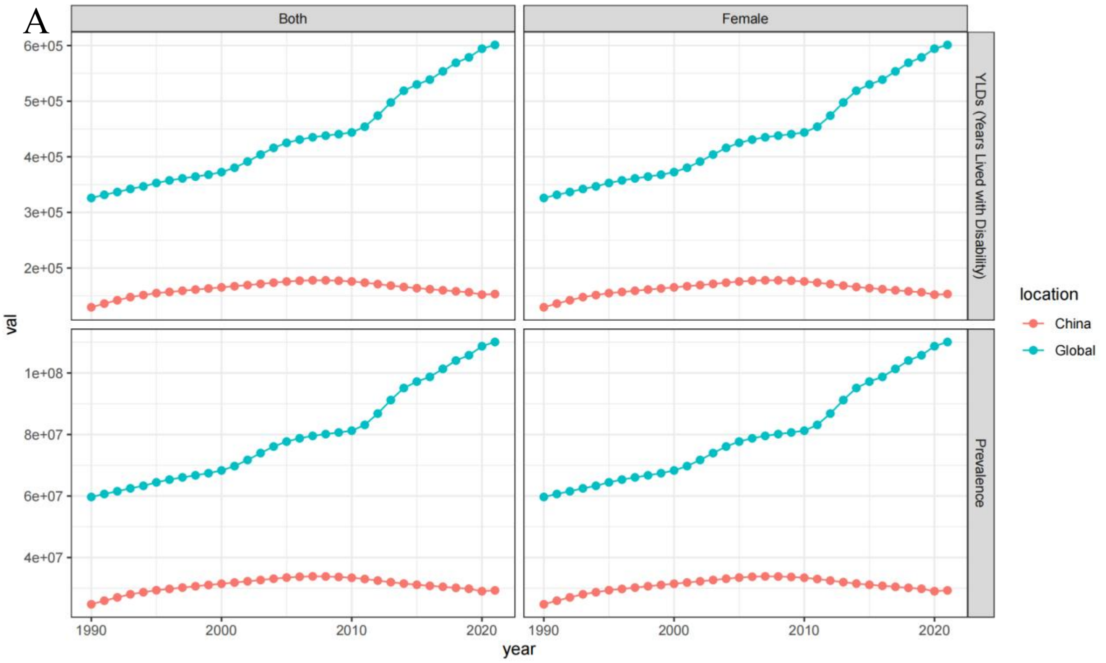

B
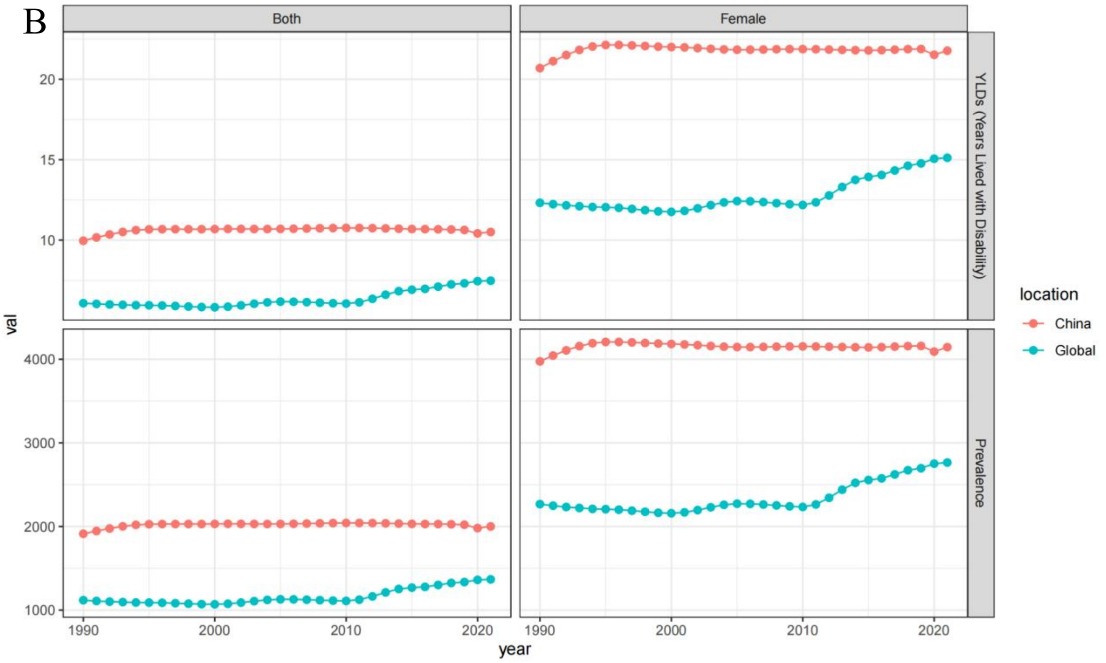

Fig. S23 (A) Trends in the number of prevalent cases and years lived with disability of female infertility from 1990 to 2021; (B) Trends in the age-standardized rates of prevalence and years lived with disability of female infertility from 1990 to 2021.

A

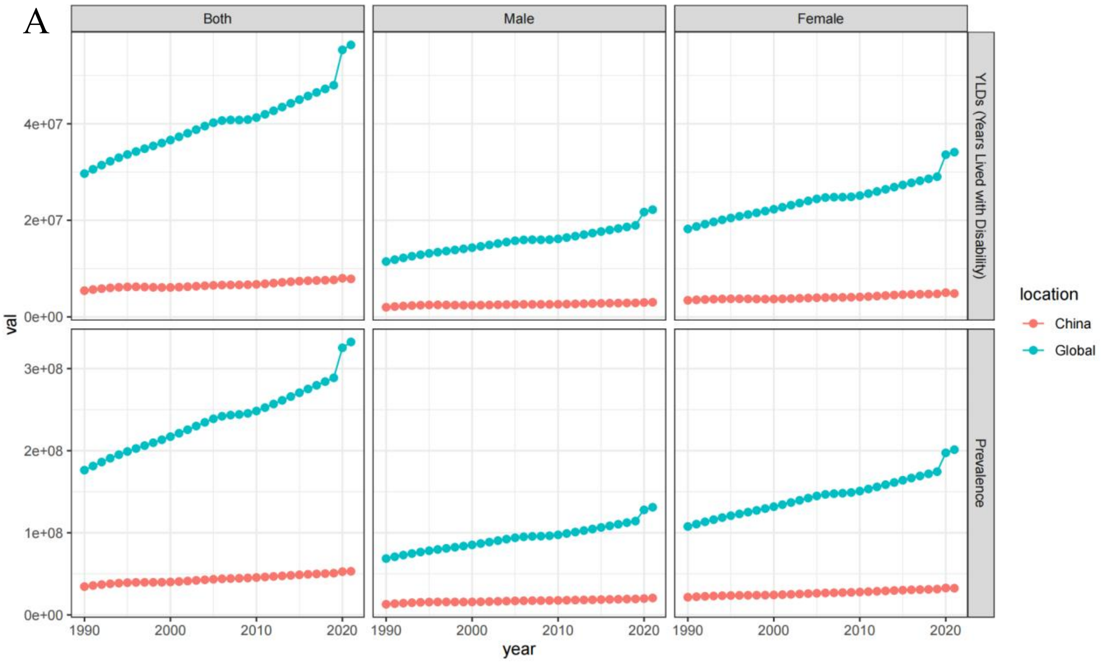

B

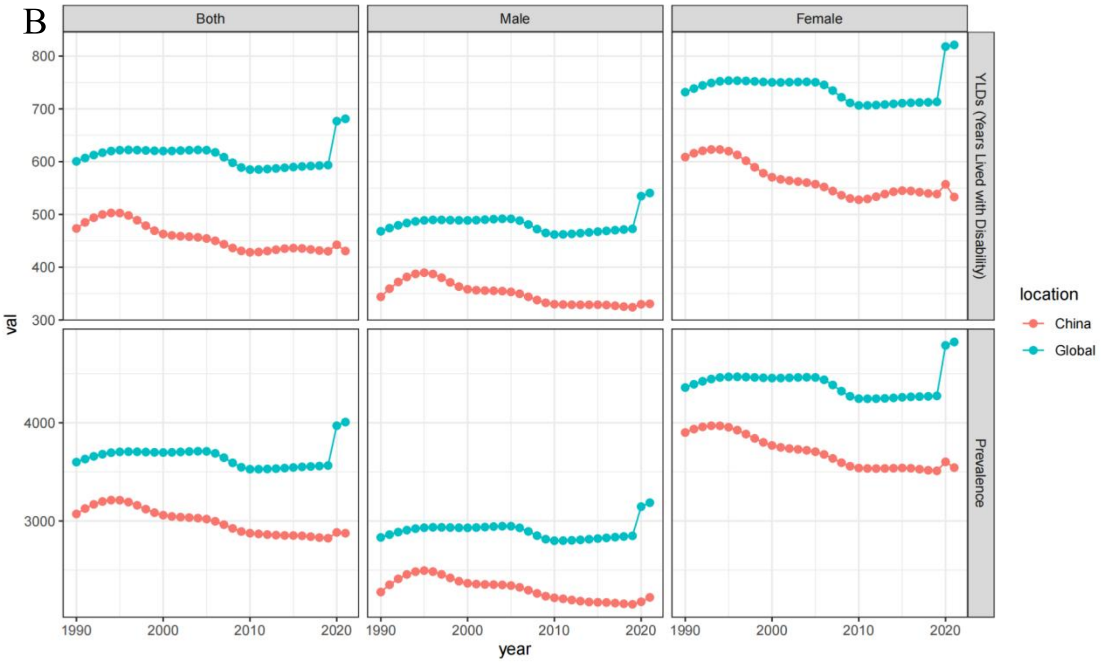

Fig. S24 (A) Trends in the number of prevalent cases and years lived with disability of depressive disorders from 1990 to 2021; (B) Trends in the age-standardized rates of prevalence and years lived with disability of depressive disorders from 1990 to 2021.

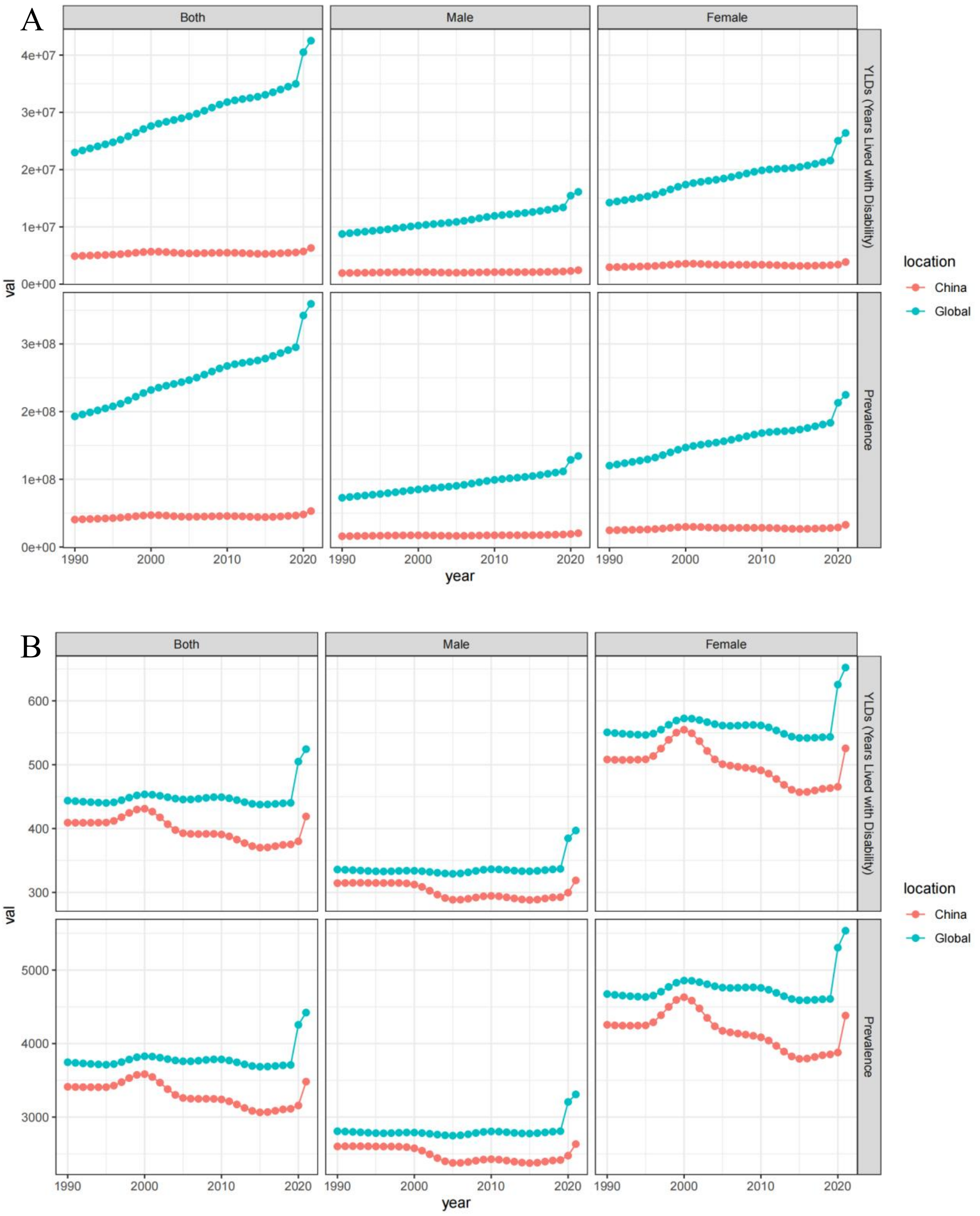

Fig. S25 (A) Trends in the number of prevalent cases and years lived with disability of anxiety disorders from 1990 to 2021; (B) Trends in the age-standardized rates of prevalence and years lived with disability of anxiety disorders from 1990 to 2021.

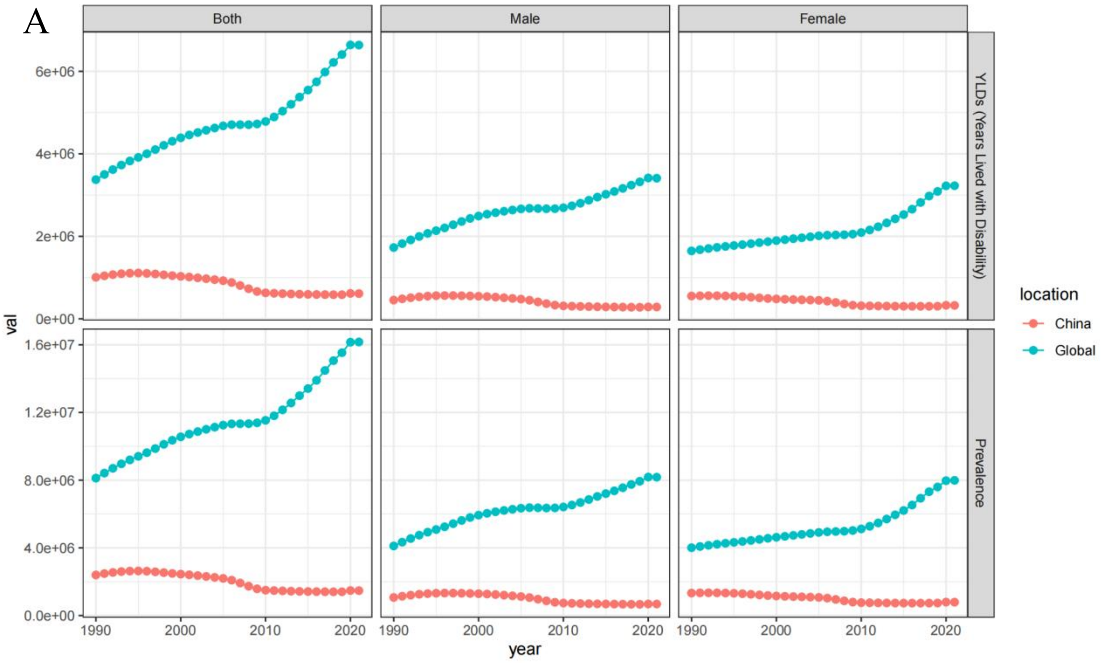

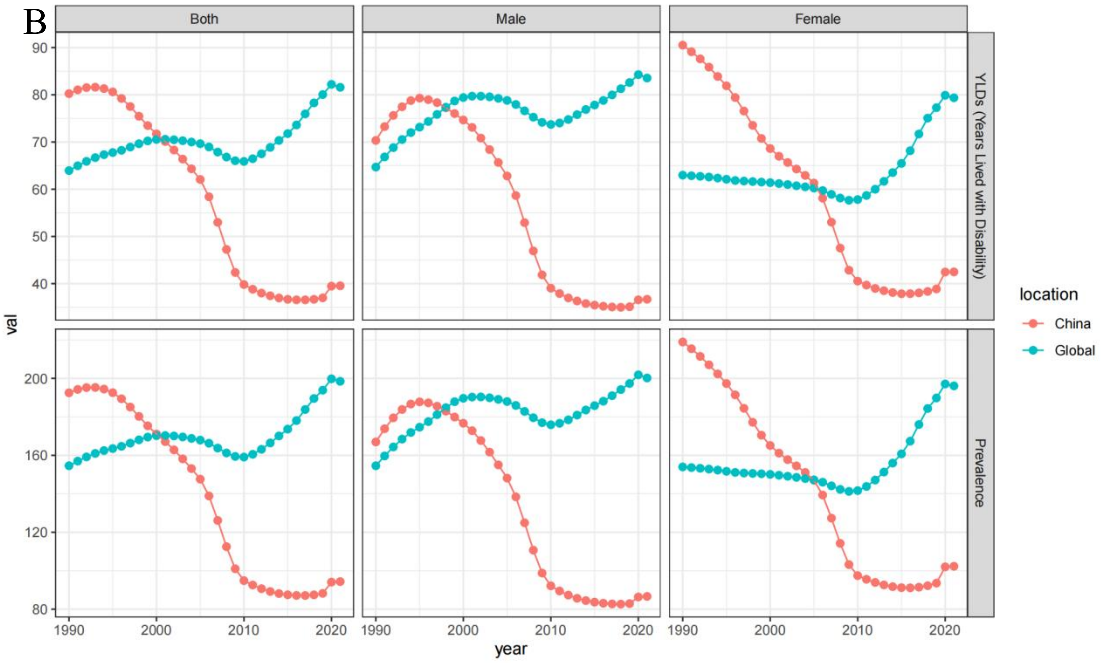

Fig. S26 (A) Trends in the number of prevalent cases and years lived with disability of opioid use disorders from 1990 to 2021; (B) Trends in the age-standardized rates of prevalence and years lived with disability of opioid use disorders from 1990 to 2021.

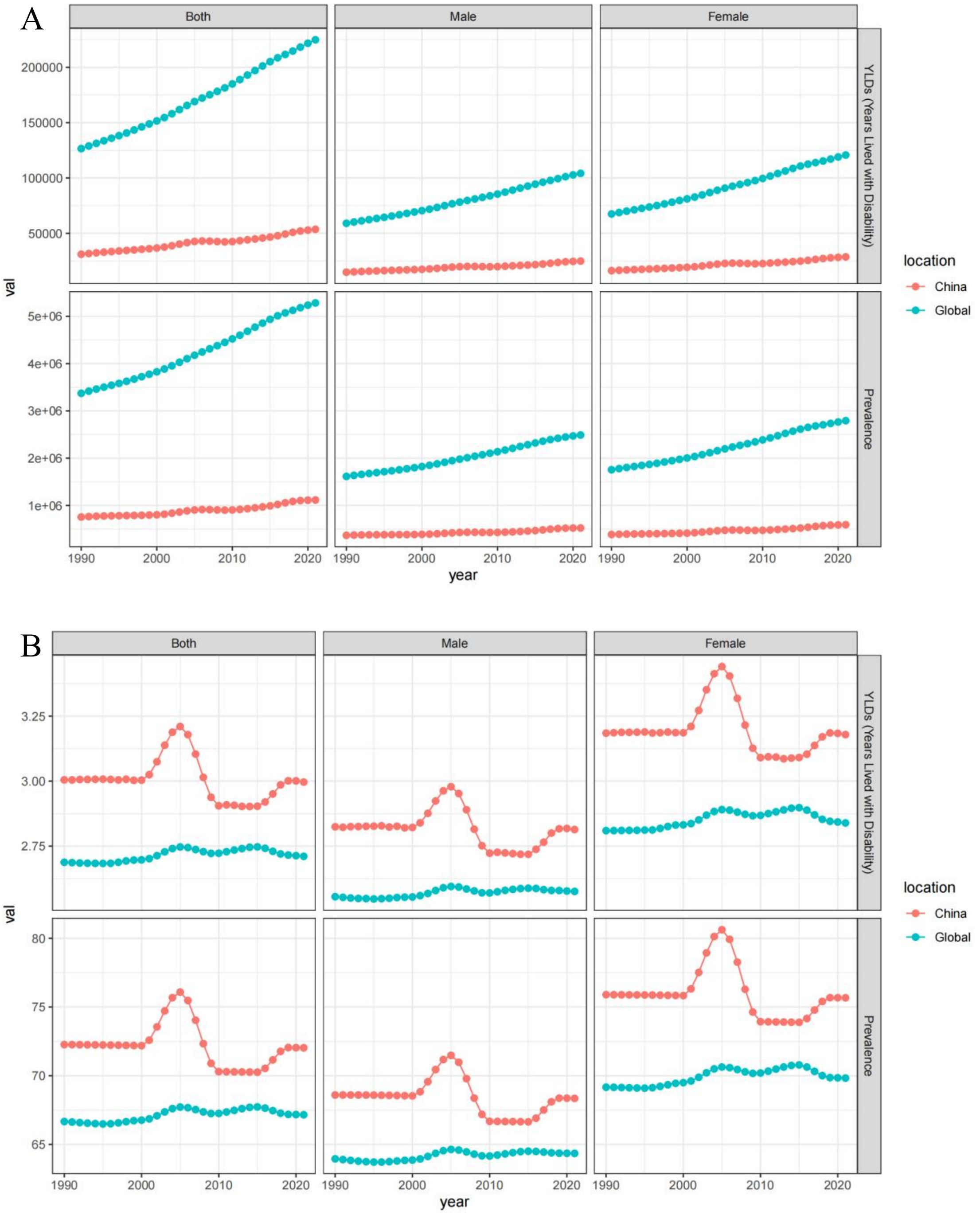

Fig. S27 (A) Trends in the number of prevalent cases and years lived with disability of varicella and herpes zoster from 1990 to 2021; (B) Trends in the age-standardized rates of prevalence and years lived with disability of varicella and herpes zoster from 1990 to 2021.

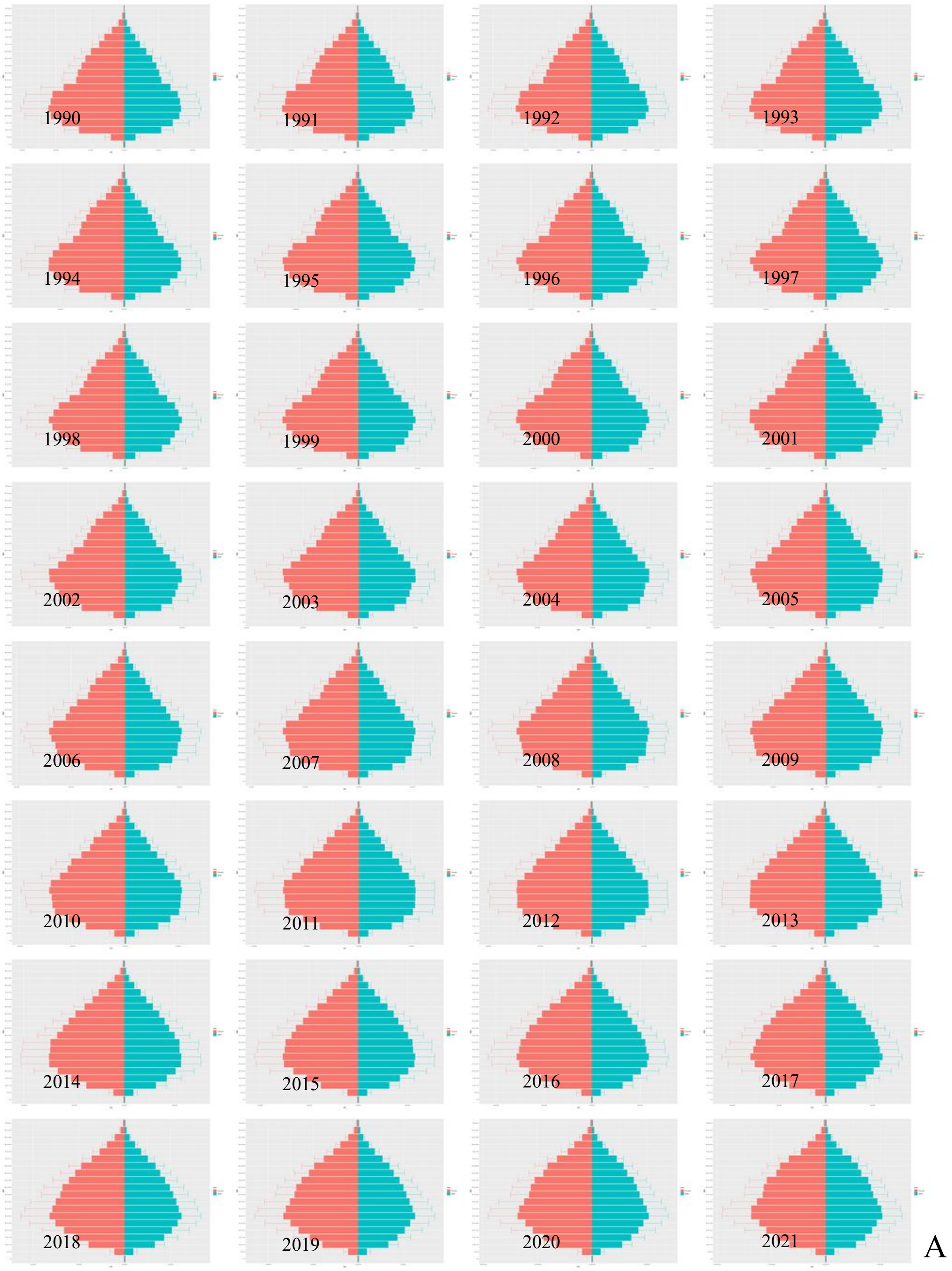
1990
1991
1992
1993
1994
1995
1996
1997
1998
1999
2000
2001
2002
2003
2004
2005
2006
2007
2008
2009
2010
2011
2012
2013
2014
2015
2016
2017
2018
2019
2020
2021
A

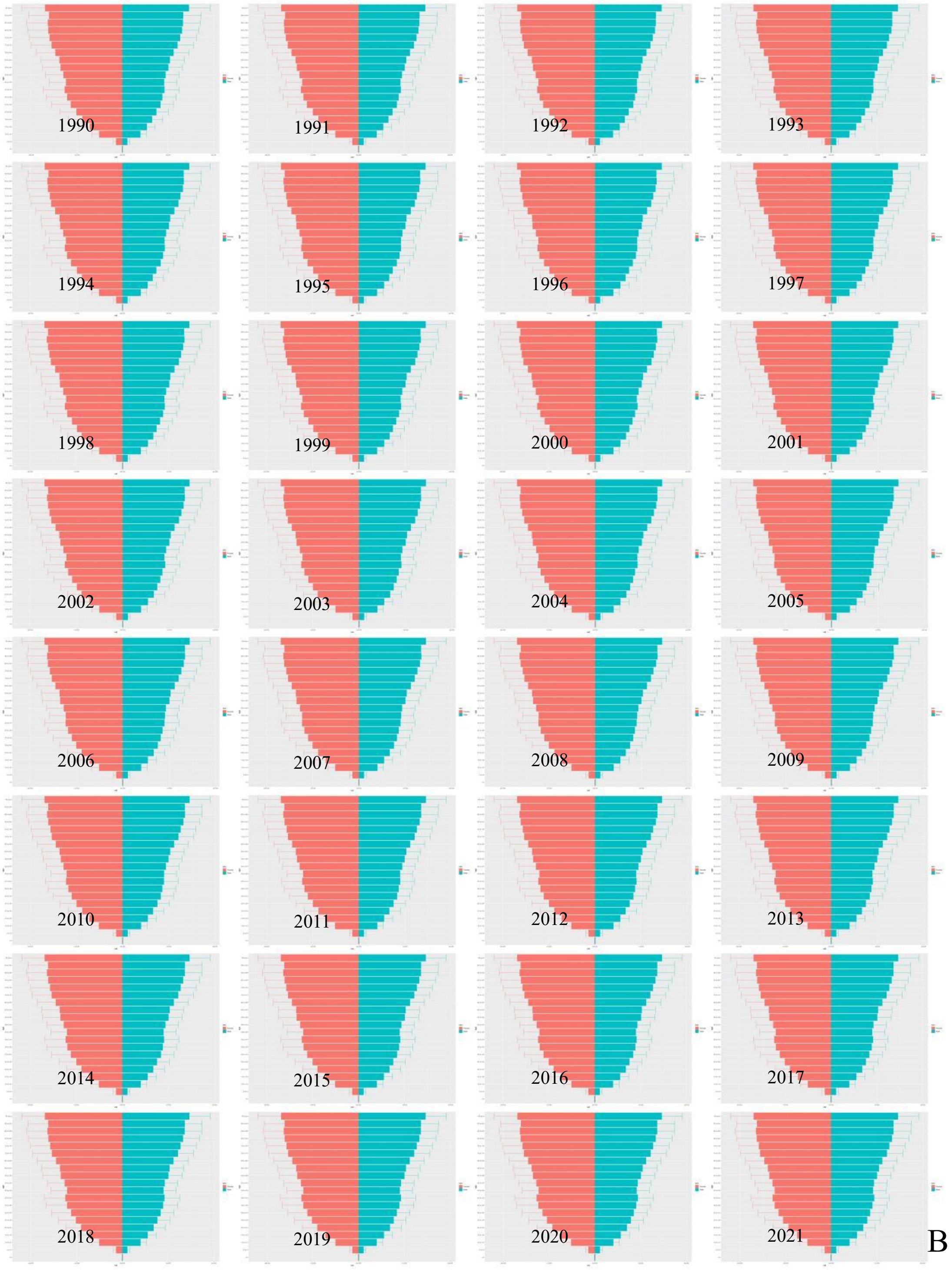
1990
1991
1992
1993
1994
1995
1996
1997
1998
1999
2000
2001
2002
2003
2004
2005
2006
2007
2008
2009
2010
2011
2012
2013
2014
2015
2016
2017
2018
2019
2020
2021
B

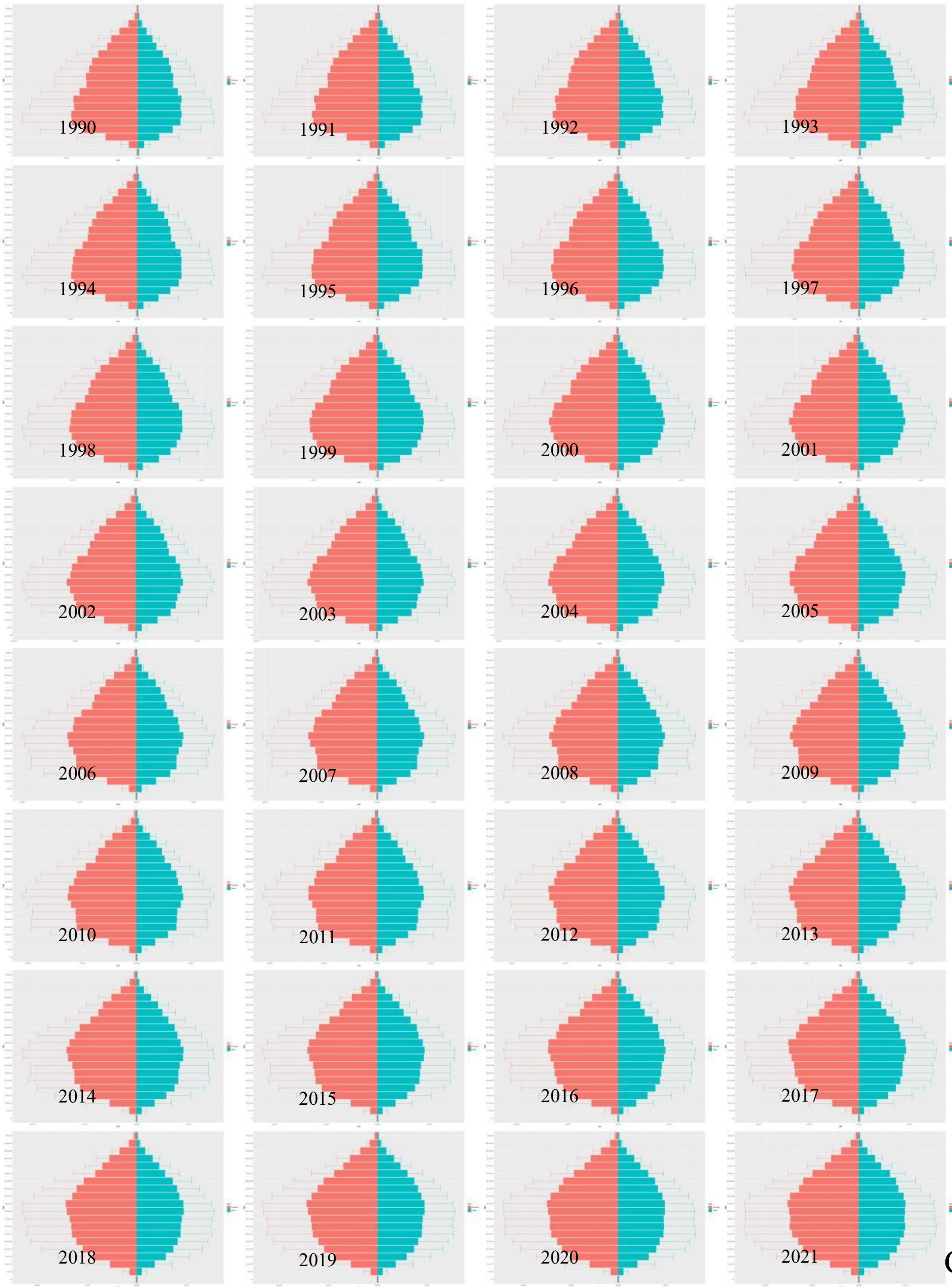
1990
1991
1992
1993
1994
1995
1996
1997
1998
1999
2000
2001
2002
2003
2004
2005
2006
2007
2008
2009
2010
2011
2012
2013
2014
2015
2016
2017
2018
2019
2020
2021

C

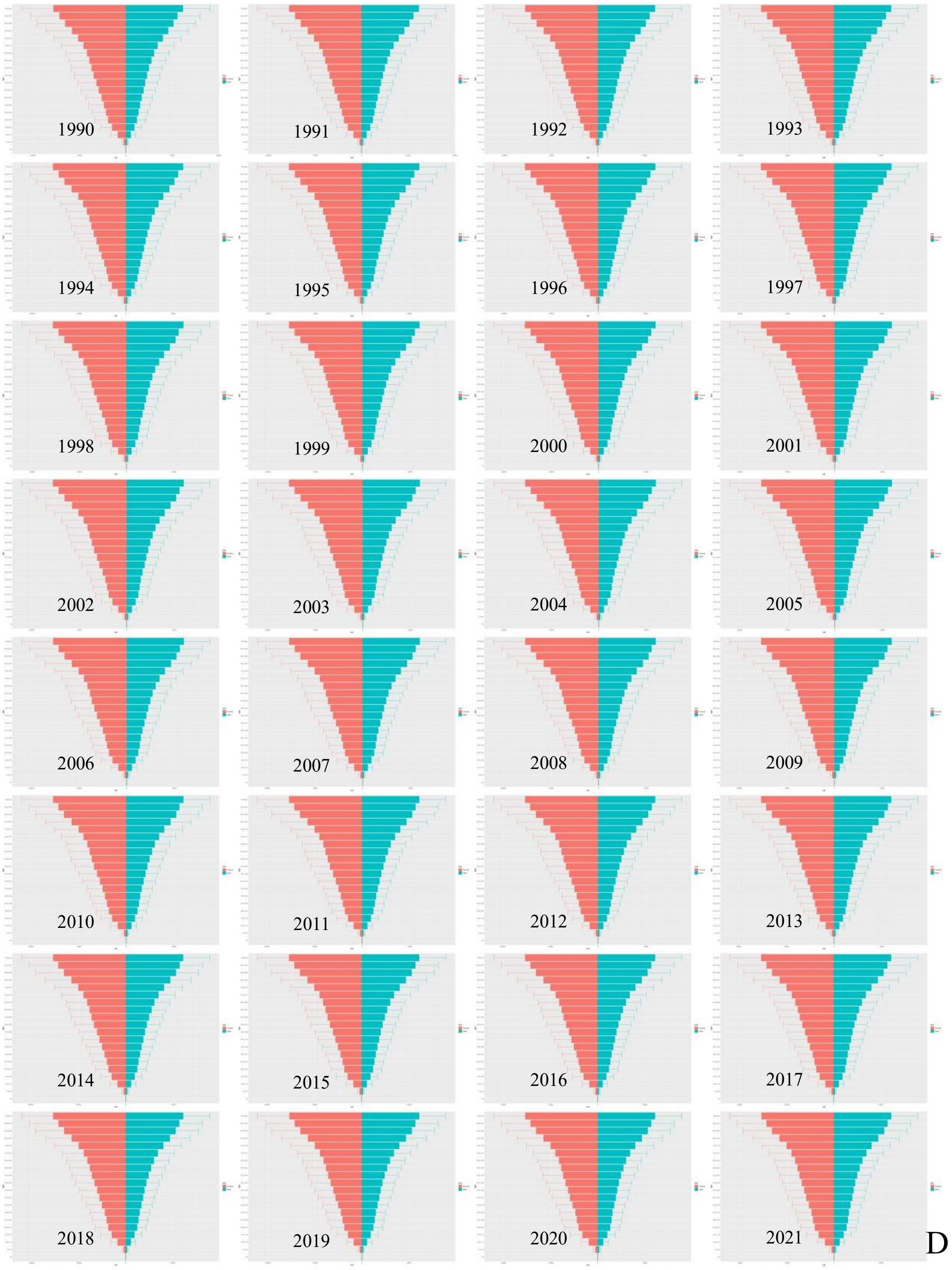

Fig. S28 (A) Acupuncture demands based on prevalence cases in different ages from 1990 to 2021 in the worldwide; (B) Acupuncture demands based on prevalence rates in different ages from 1990 to 2021 in the worldwide; (C) Acupuncture demands based on years lived with disability in different ages from 1990 to 2021 in the worldwide; (D) Acupuncture demands based on years lived with disability rates in different ages from 1990 to 2021 in the worldwide.

Notes: red for female, green for male; the ordinate from bottom to top is "<5", "5 to 9", "10 to 14", "15 to 19", "20 to 24", "25 to 29", "30 to 34", "35 to 39", "40 to 44", "45 to 49", "50 to 54", "55 to 59", "60 to 64", "65 to 69", "70 to 74", "75 to 79", "80 to 84", "85 to 89", "90 to 94", "95 plus".

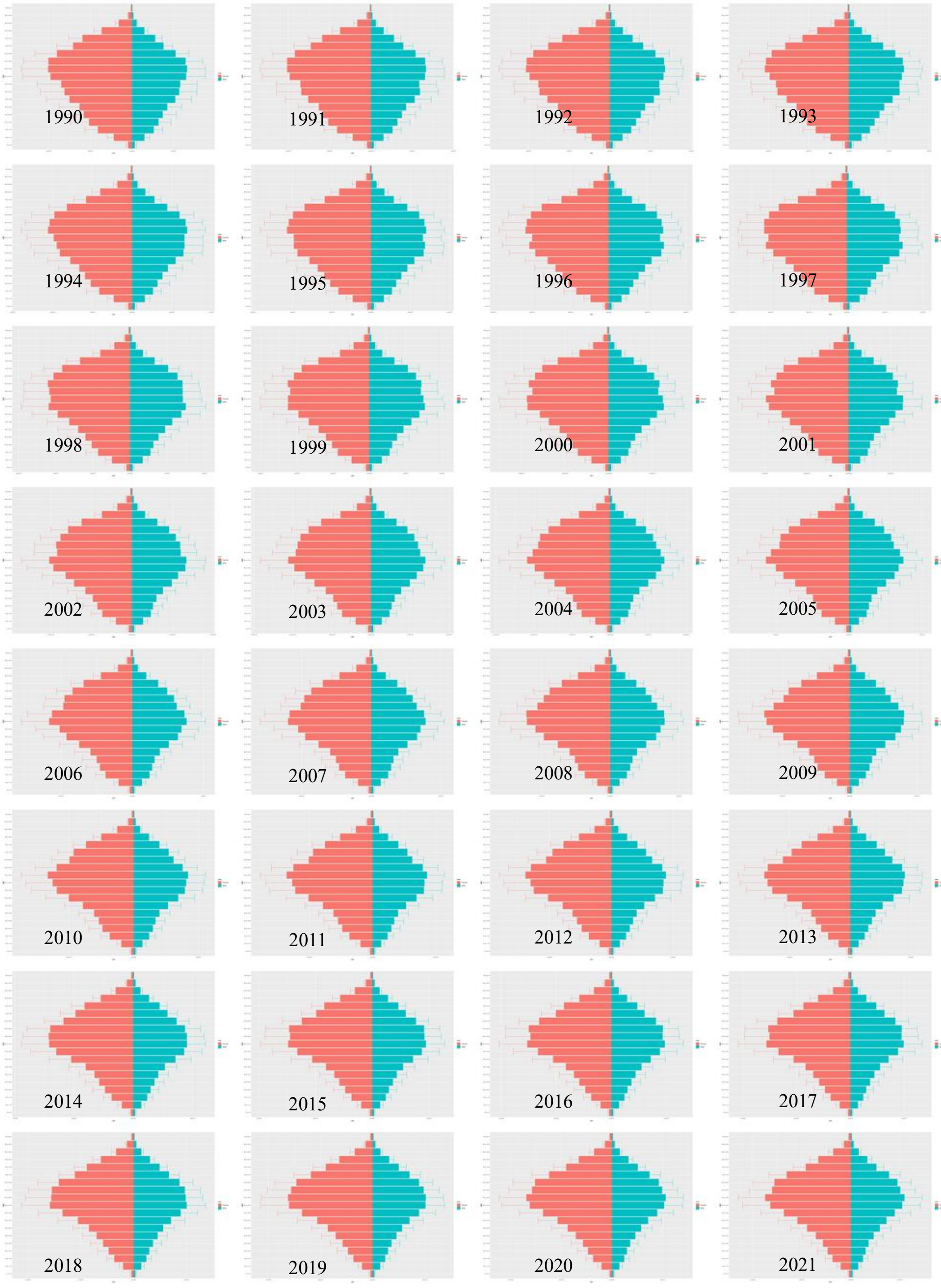
1990
1991
1992
1993
1994
1995
1996
1997
1998
1999
2000
2001
2002
2003
2004
2005
2006
2007
2008
2009
2010
2011
2012
2013
2014
2015
2016
2017
2018
2019
2020
2021

A

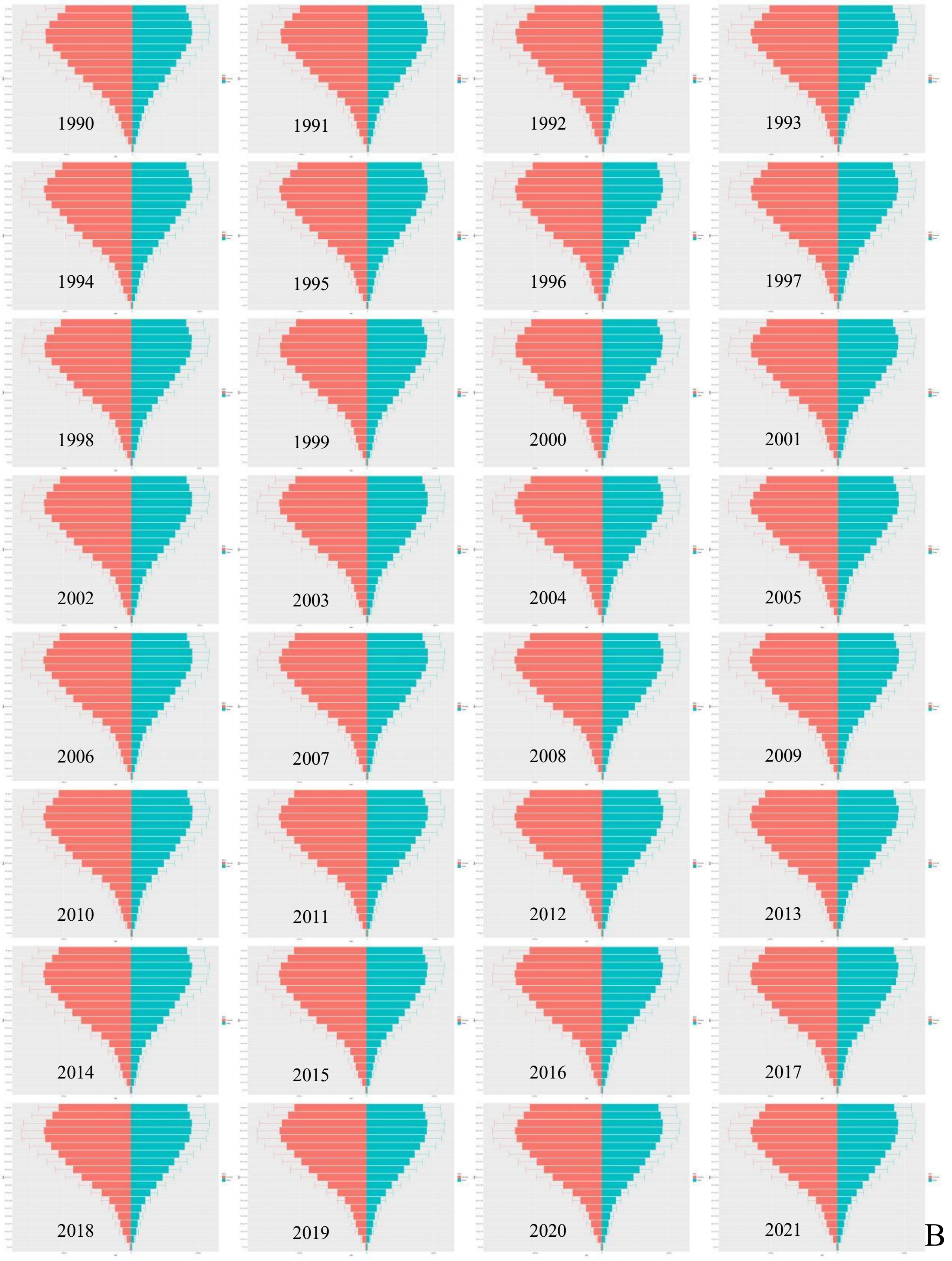
1990
1991
1992
1993
1994
1995
1996
1997
1998
1999
2000
2001
2002
2003
2004
2005
2006
2007
2008
2009
2010
2011
2012
2013
2014
2015
2016
2017
2018
2019
2020
2021
B

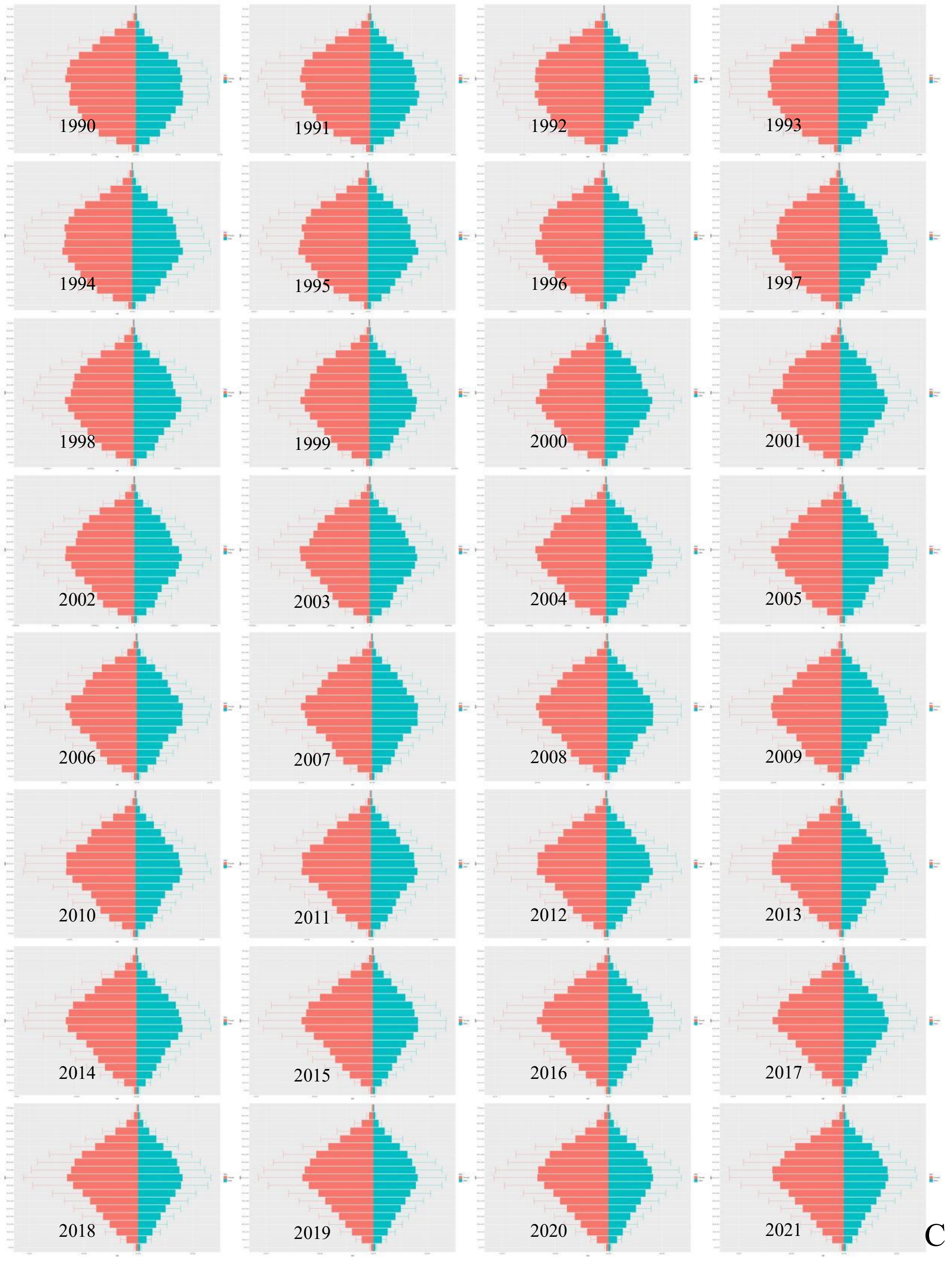
1990
1991
1992
1993
1994
1995
1996
1997
1998
1999
2000
2001
2002
2003
2004
2005
2006
2007
2008
2009
2010
2011
2012
2013
2014
2015
2016
2017
2018
2019
2020
2021
C

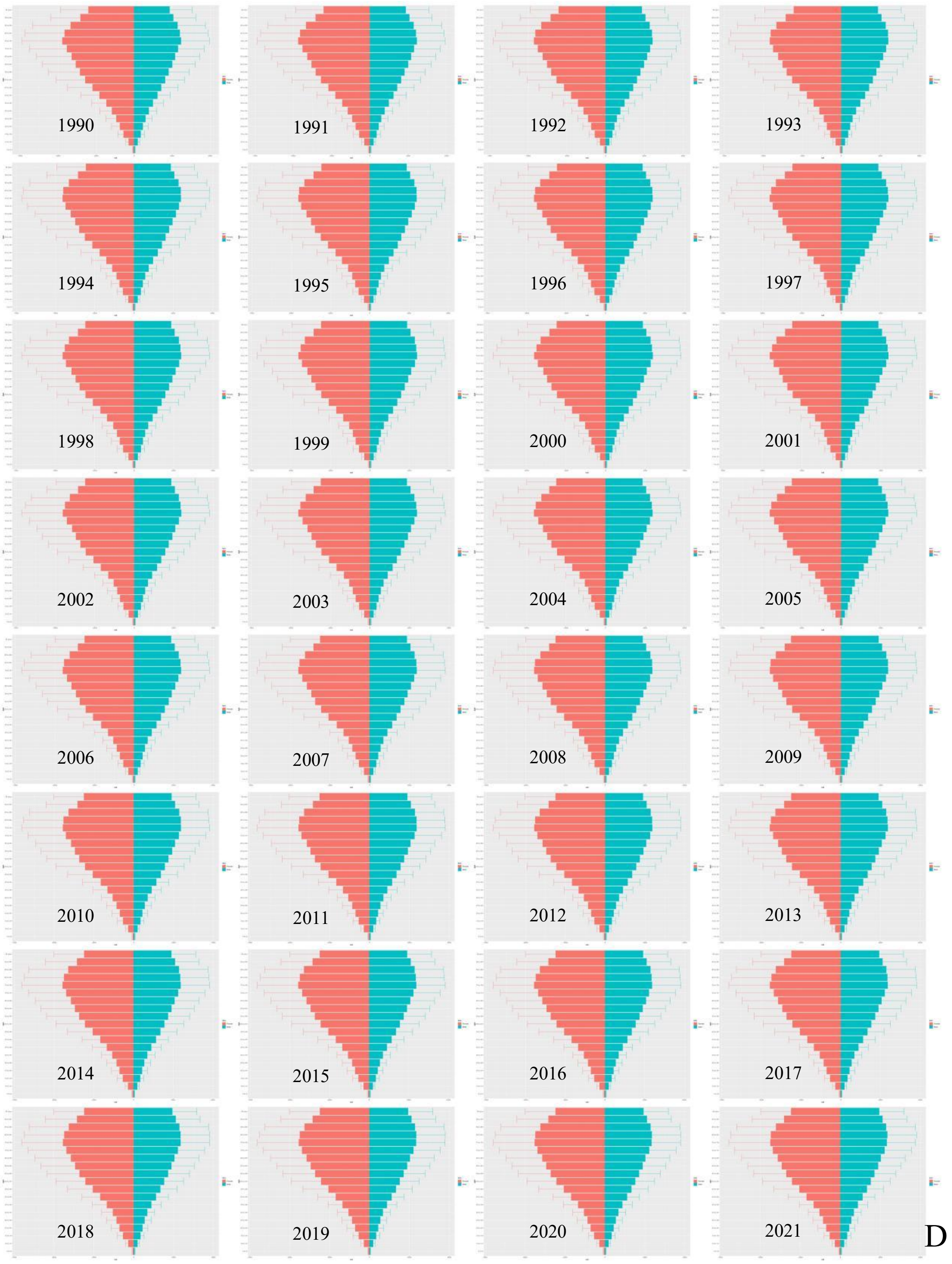

Fig. S29 (A) The prevalence cases of musculoskeletal disorders in different ages from 1990 to 2021 in the worldwide; (B) The prevalence rates of musculoskeletal disorders in different ages from 1990 to 2021 in the worldwide; (C) The years lived with disability of musculoskeletal disorders in different ages from 1990 to 2021 in the worldwide; (D) The years lived with disability rates of musculoskeletal disorders in different ages from 1990 to 2021 in the worldwide.

Notes: red for female, green for male; the ordinate from bottom to top is "5 to 9", "10 to 14", "15 to 19", "20 to 24", "25 to 29", "30 to 34", "35 to 39", "40 to 44", "45 to 49", "50 to 54", "55 to 59", "60 to 64", "65 to 69", "70 to 74", "75 to 79", "80 to 84", "85 to 89", "90 to 94", "95 plus".

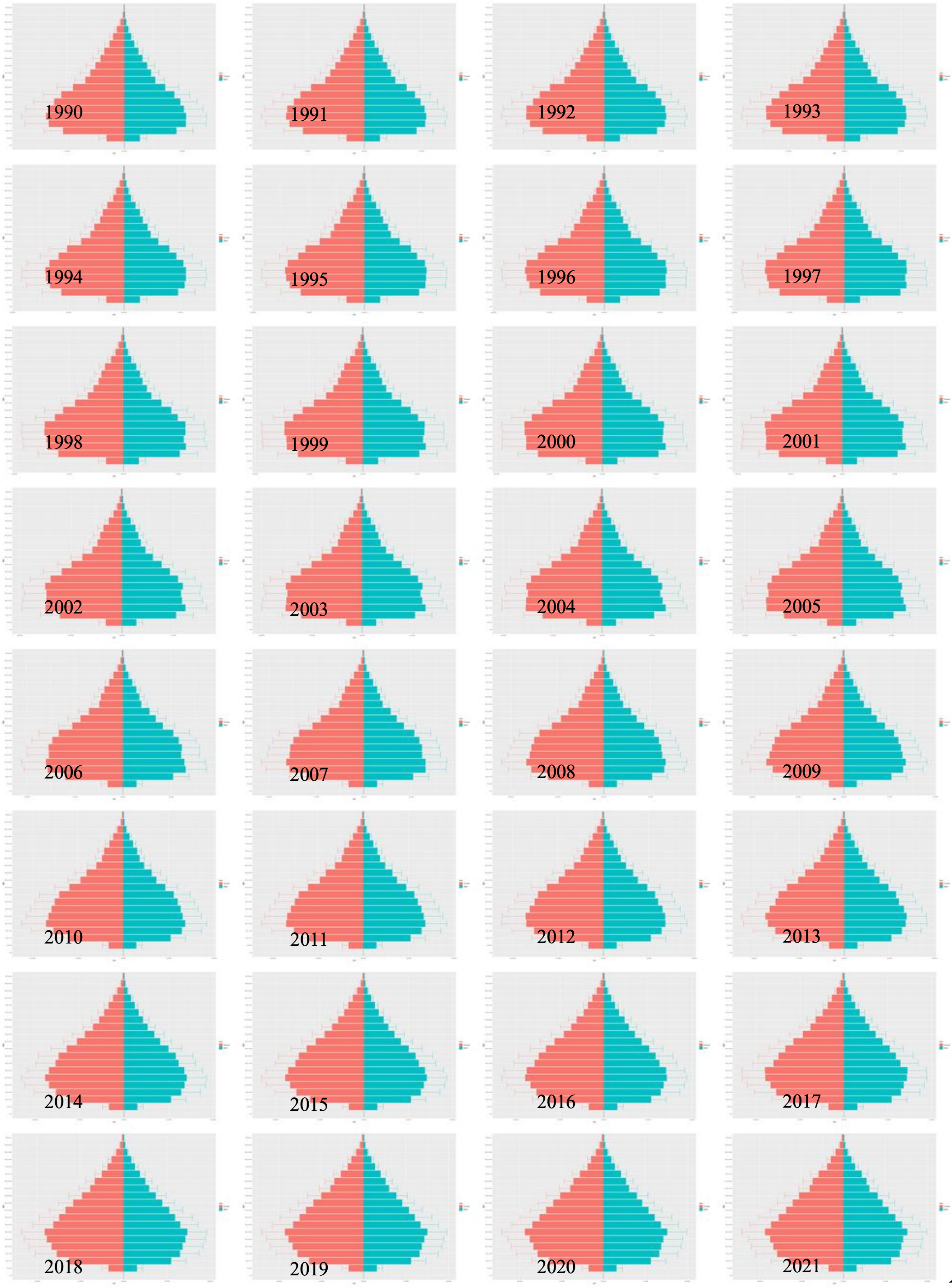
1990
1991
1992
1993
1994
1995
1996
1997
1998
1999
2000
2001
2002
2003
2004
2005
2006
2007
2008
2009
2010
2011
2012
2013
2014
2015
2016
2017
2018
2019
2020
2021

A

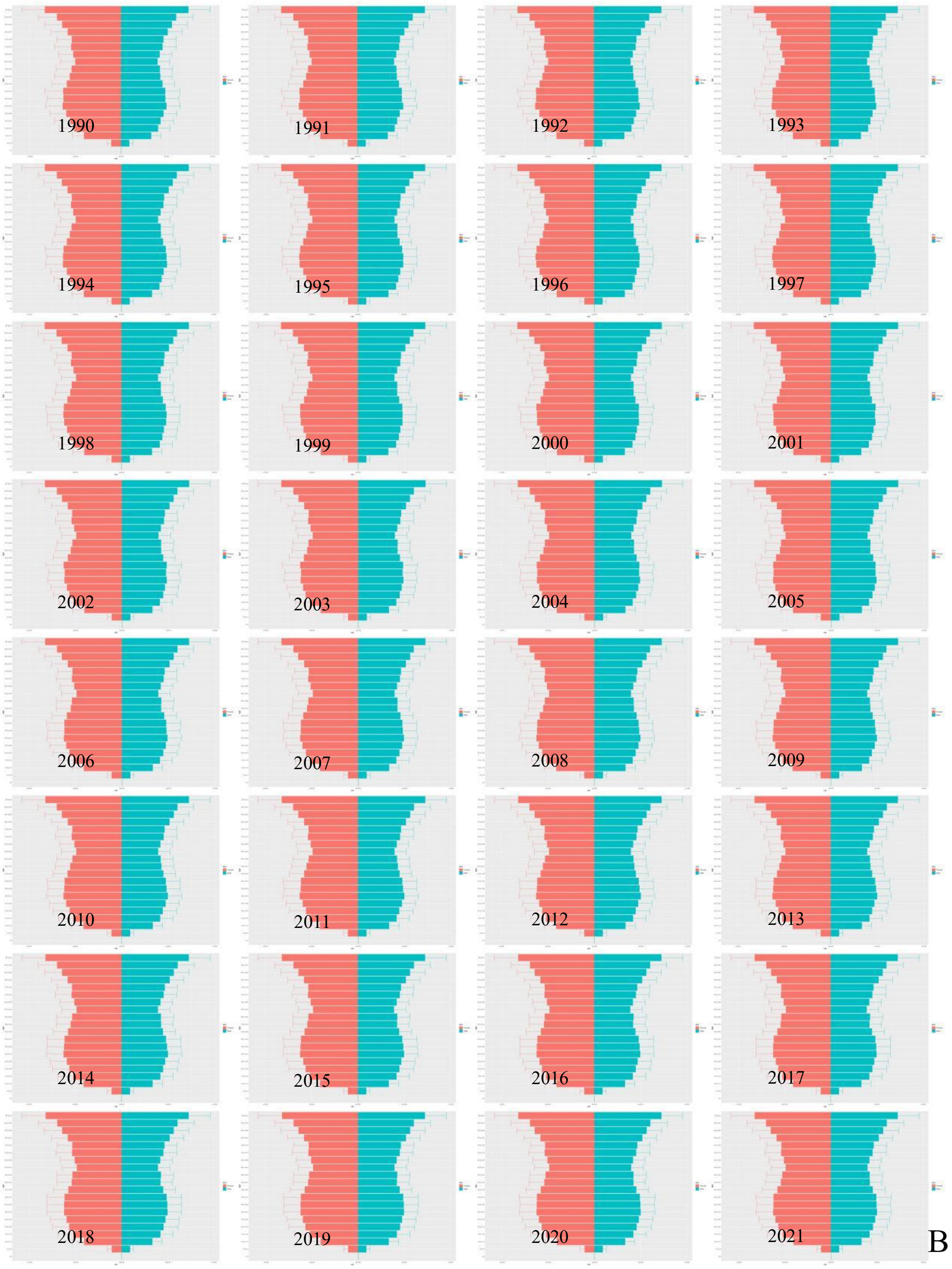
1990
1991
1992
1993
1994
1995
1996
1997
1998
1999
2000
2001
2002
2003
2004
2005
2006
2007
2008
2009
2010
2011
2012
2013
2014
2015
2016
2017
2018
2019
2020
2021
B

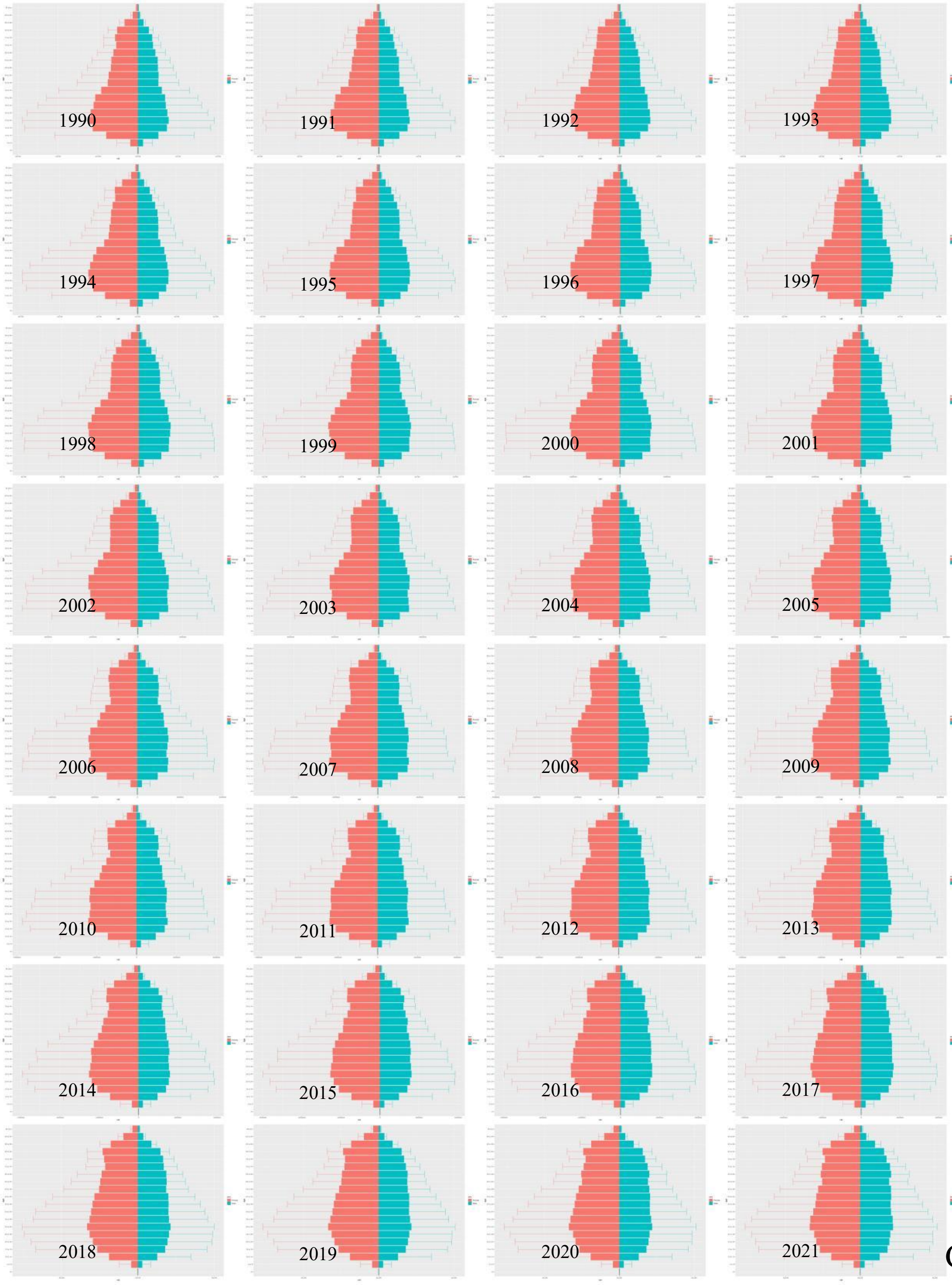
1990
1991
1992
1993
1994
1995
1996
1997
1998
1999
2000
2001
2002
2003
2004
2005
2006
2007
2008
2009
2010
2011
2012
2013
2014
2015
2016
2017
2018
2019
2020
2021
C

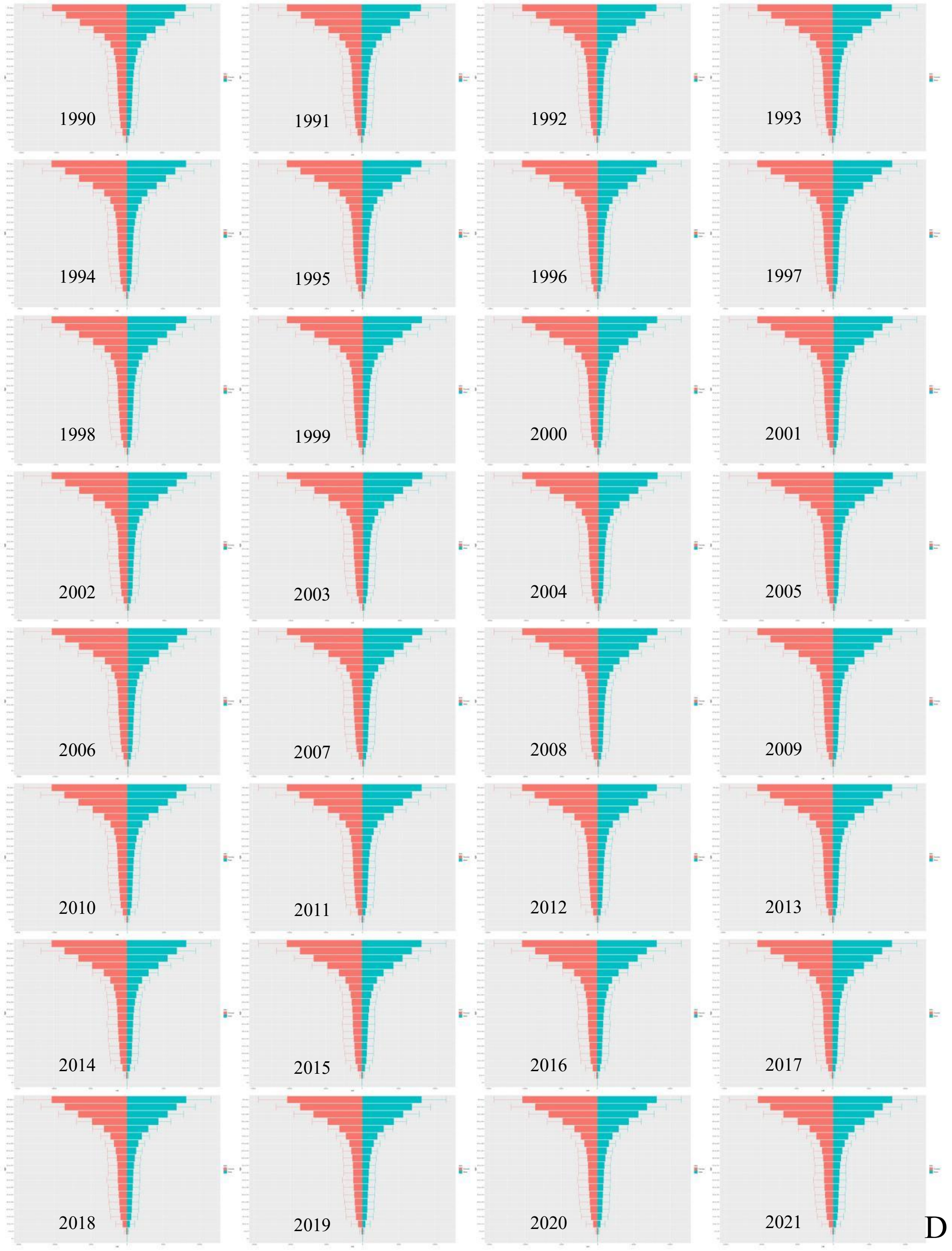

Fig. S30 (A) The prevalence cases of neurological disorders in different ages from 1990 to 2021 in the worldwide; (B) The prevalence rates of neurological disorders in different ages from 1990 to 2021 in the worldwide; (C) The years lived with disability of neurological disorders in different ages from 1990 to 2021 in the worldwide; (D) The years lived with disability rates of neurological disorders in different ages from 1990 to 2021 in the worldwide.

Notes: red for female, green for male; the ordinate from bottom to top is "<5", "5 to 9", "10 to 14", "15 to 19", "20 to 24", "25 to 29", "30 to 34", "35 to 39", "40 to 44", "45 to 49", "50 to 54", "55 to 59", "60 to 64", "65 to 69", "70 to 74", "75 to 79", "80 to 84", "85 to 89", "90 to 94", "95 plus".

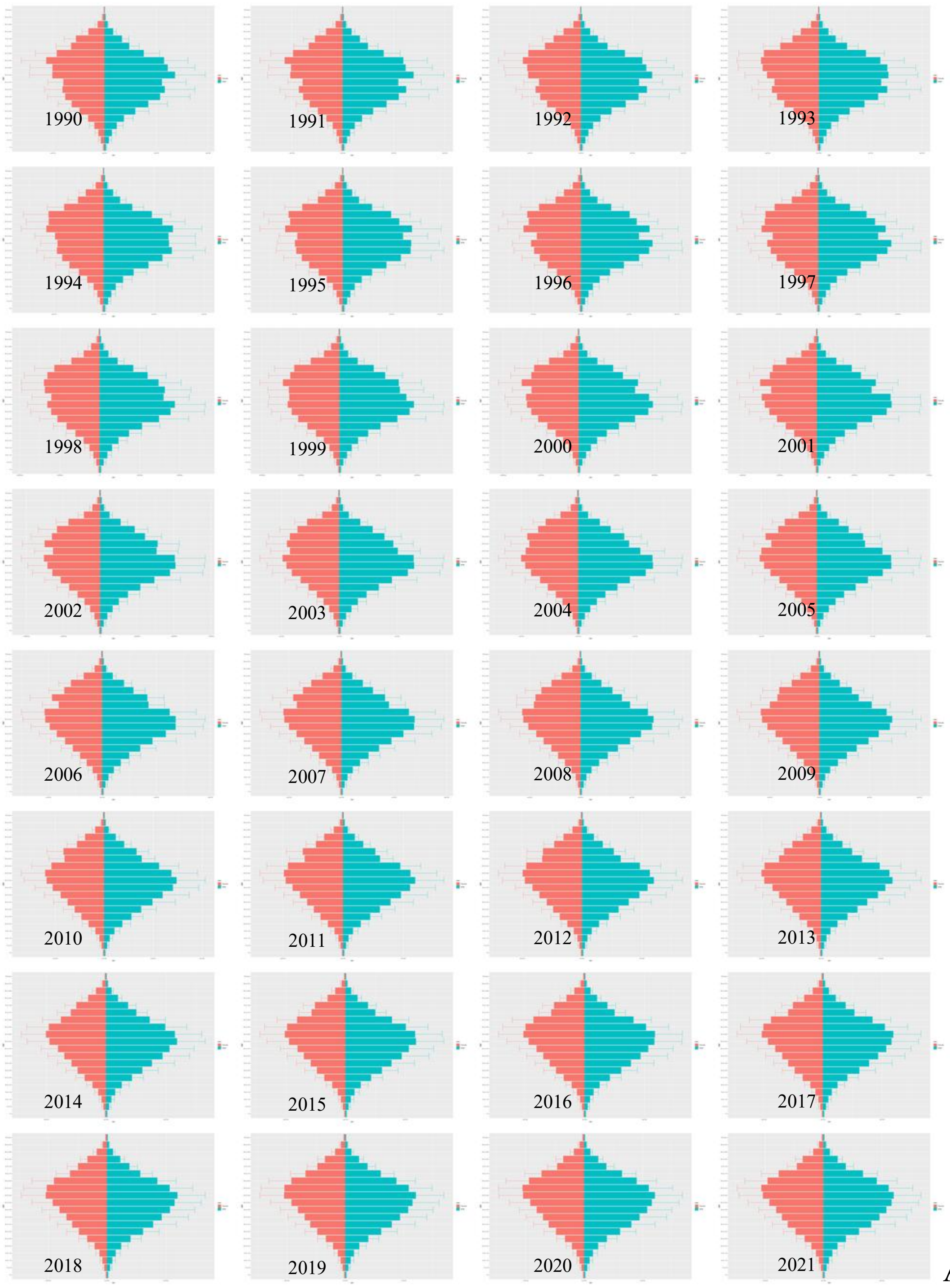
1990
1991
1992
1993
1994
1995
1996
1997
1998
1999
2000
2001
2002
2003
2004
2005
2006
2007
2008
2009
2010
2011
2012
2013
2014
2015
2016
2017
2018
2019
2020
2021

A

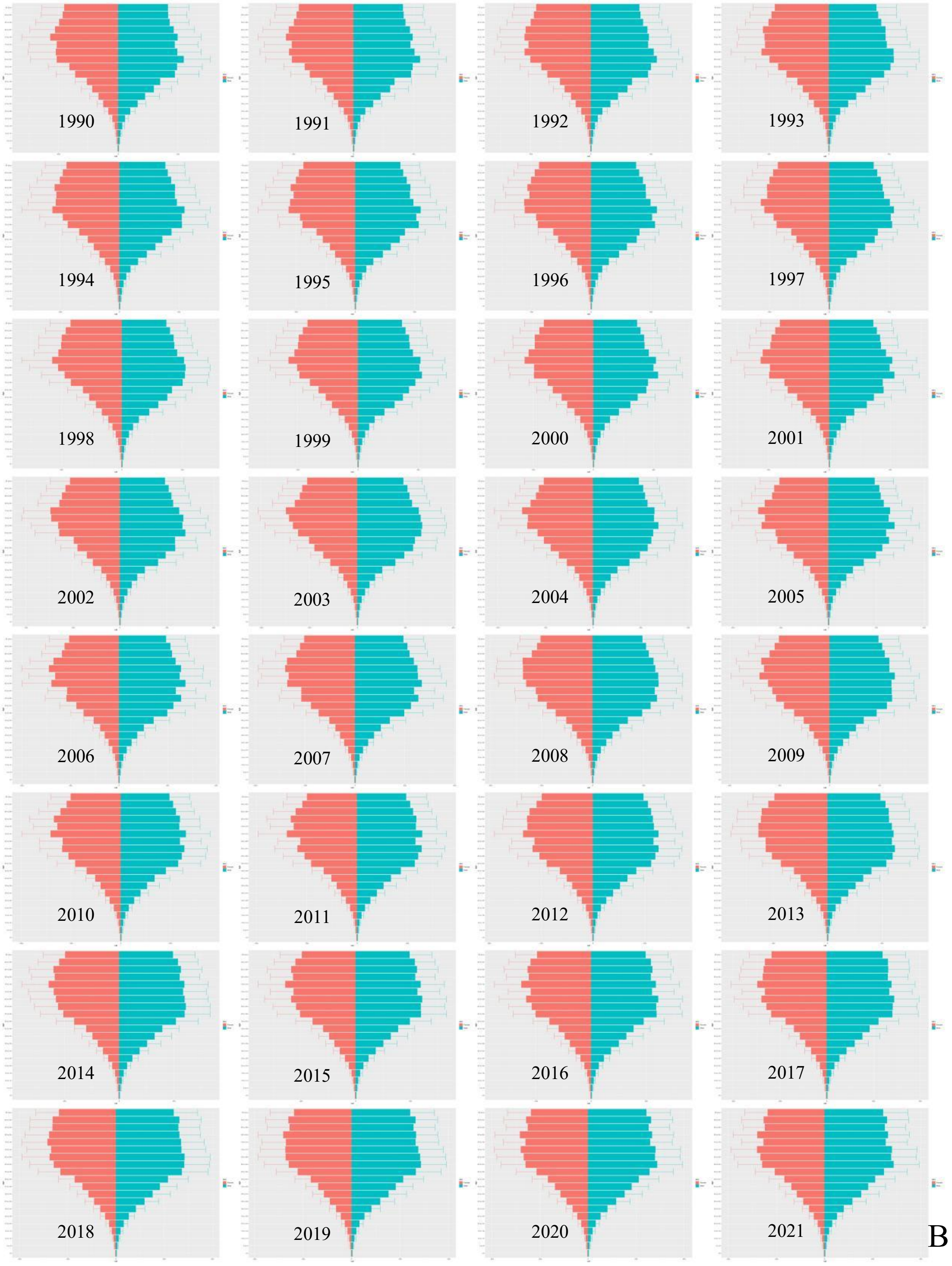
1990
1991
1992
1993
1994
1995
1996
1997
1998
1999
2000
2001
2002
2003
2004
2005
2006
2007
2008
2009
2010
2011
2012
2013
2014
2015
2016
2017
2018
2019
2020
2021
B

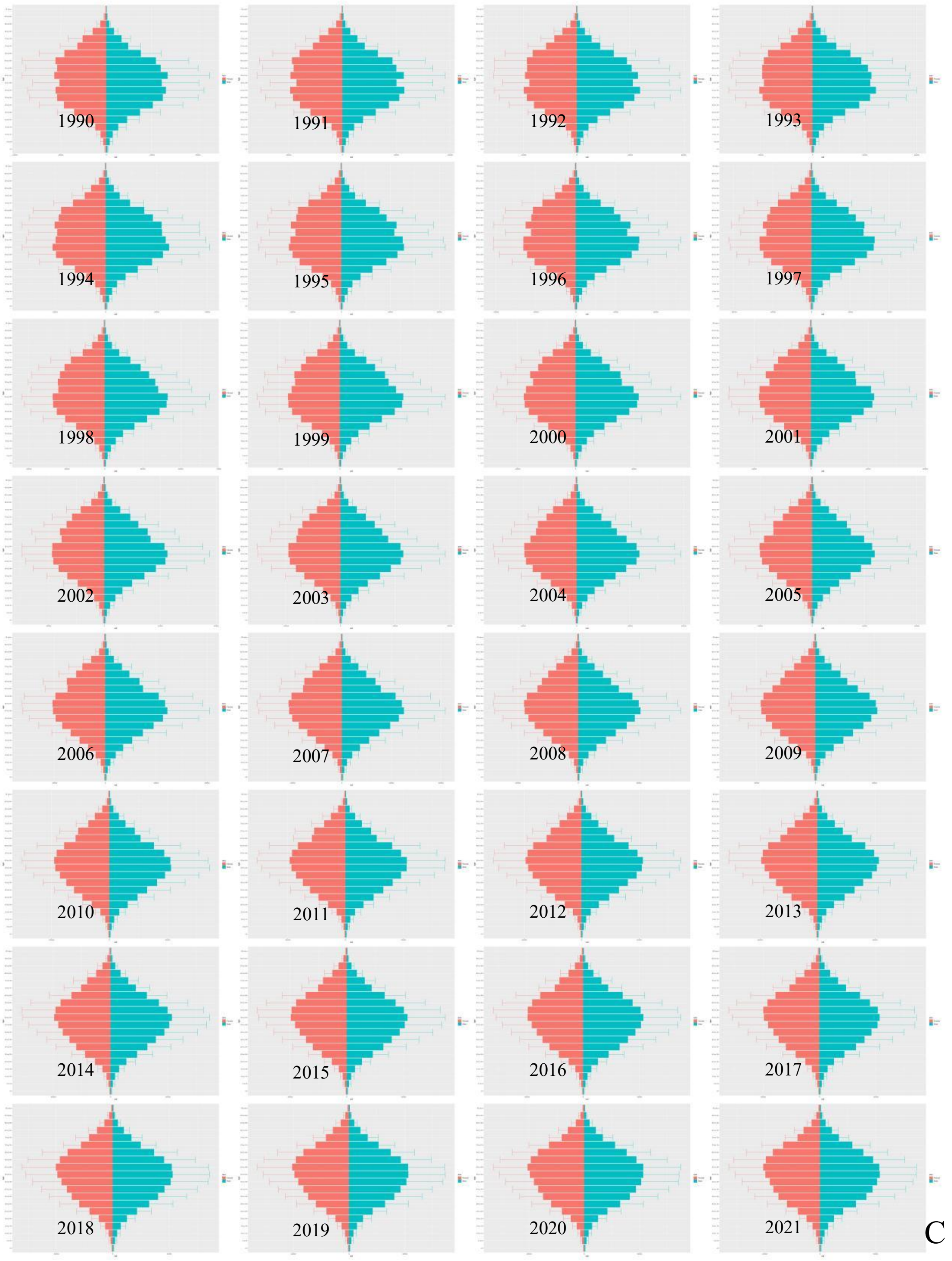
1990
1991
1992
1993
1994
1995
1996
1997
1998
1999
2000
2001
2002
2003
2004
2005
2006
2007
2008
2009
2010
2011
2012
2013
2014
2015
2016
2017
2018
2019
2020
2021
C

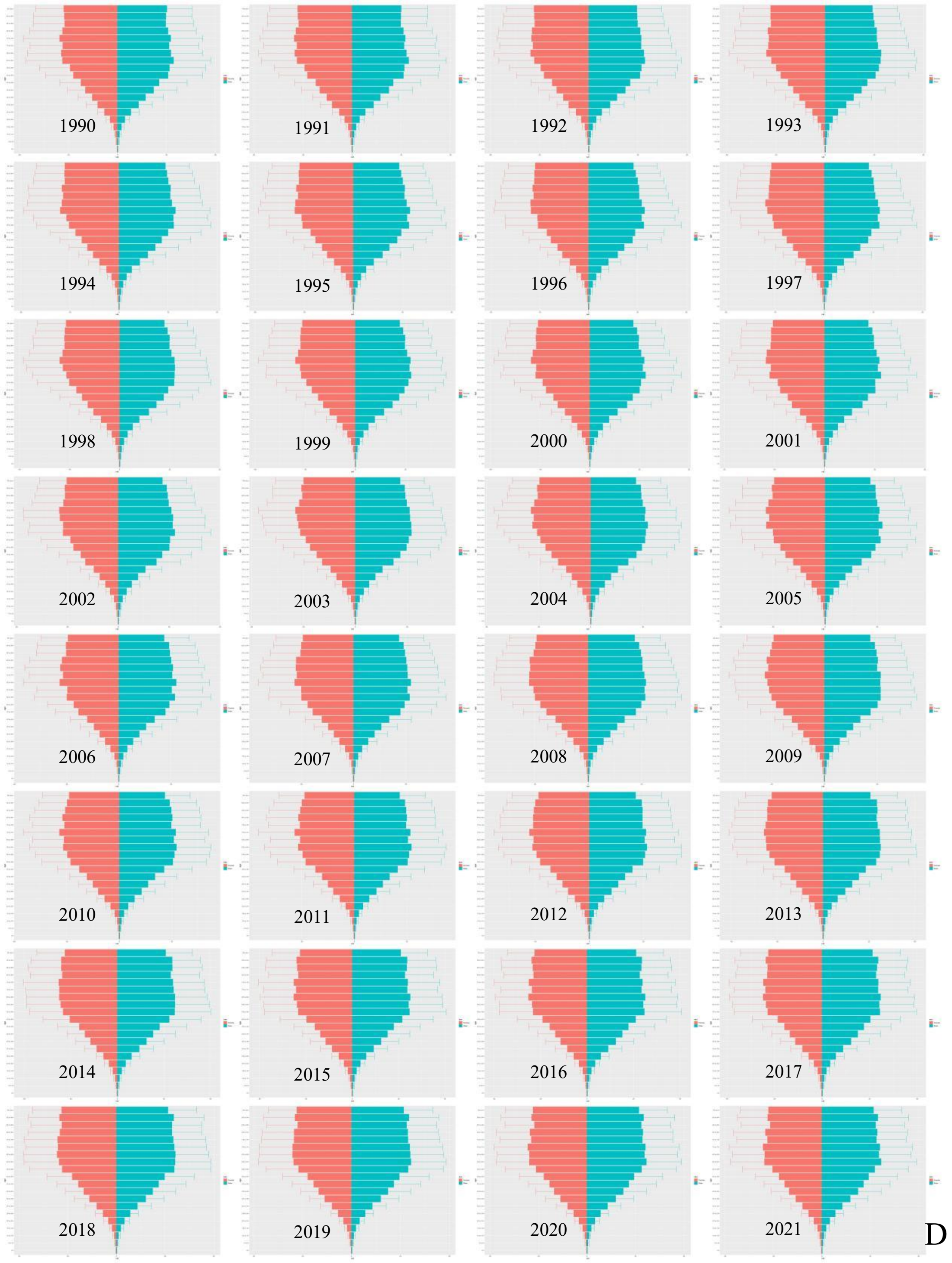

Fig. S31 (A) The prevalence cases of digestive disorders in different ages from 1990 to 2021 in the worldwide; (B) The prevalence rates of digestive disorders in different ages from 1990 to 2021 in the worldwide; (C) The years lived with disability of digestive disorders in different ages from 1990 to 2021 in the worldwide; (D) The years lived with disability rates of digestive disorders in different ages from 1990 to 2021 in the worldwide.

Notes: red for female, green for male; the ordinate from bottom to top is "<5", "5 to 9", "10 to 14", "15 to 19", "20 to 24", "25 to 29", "30 to 34", "35 to 39", "40 to 44", "45 to 49", "50 to 54", "55 to 59", "60 to 64", "65 to 69", "70 to 74", "75 to 79", "80 to 84", "85 to 89", "90 to 94", "95 plus".

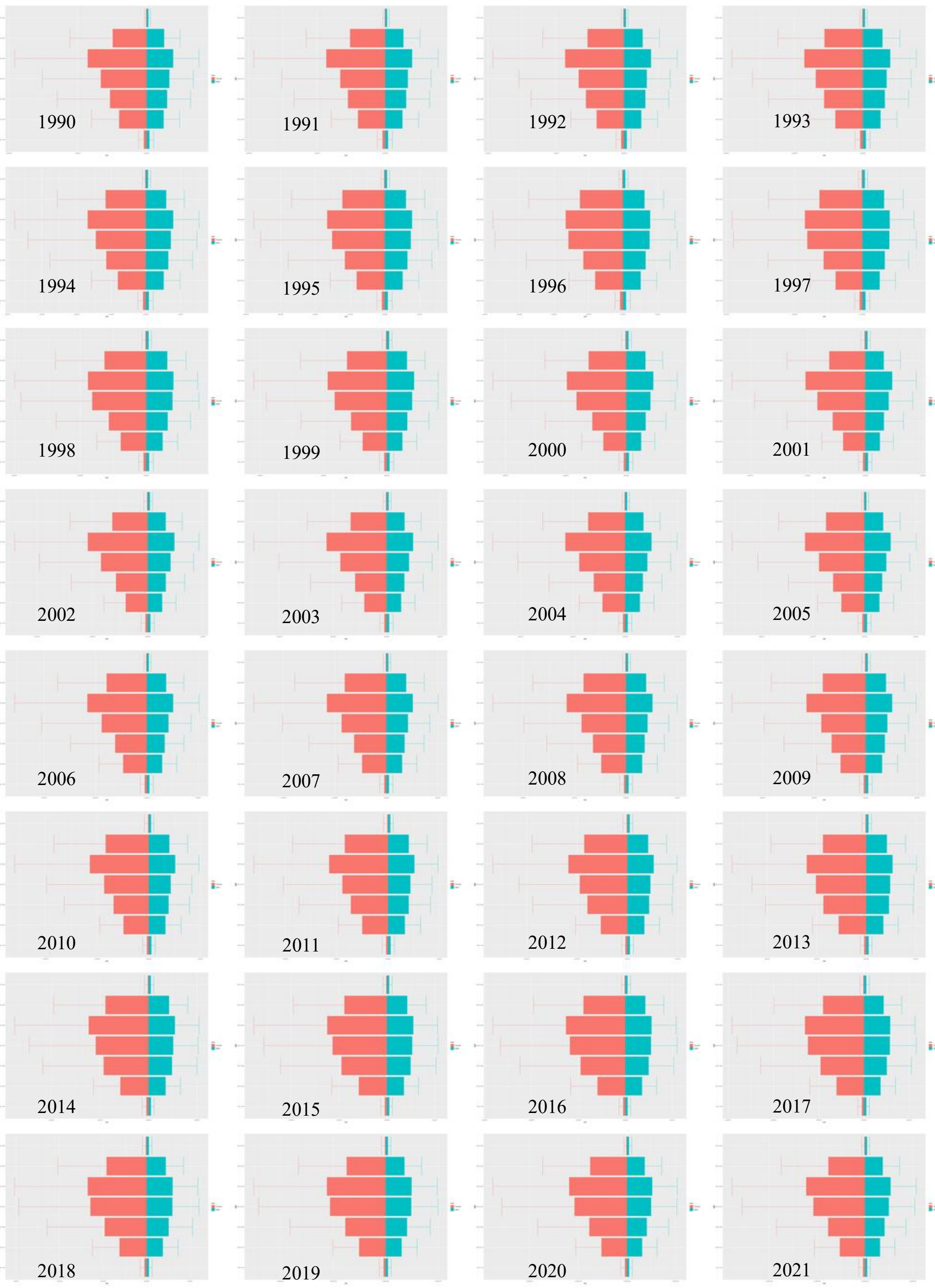
1990
1991
1992
1993
1994
1995
1996
1997
1998
1999
2000
2001
2002
2003
2004
2005
2006
2007
2008
2009
2010
2011
2012
2013
2014
2015
2016
2017
2018
2019
2020
2021

A

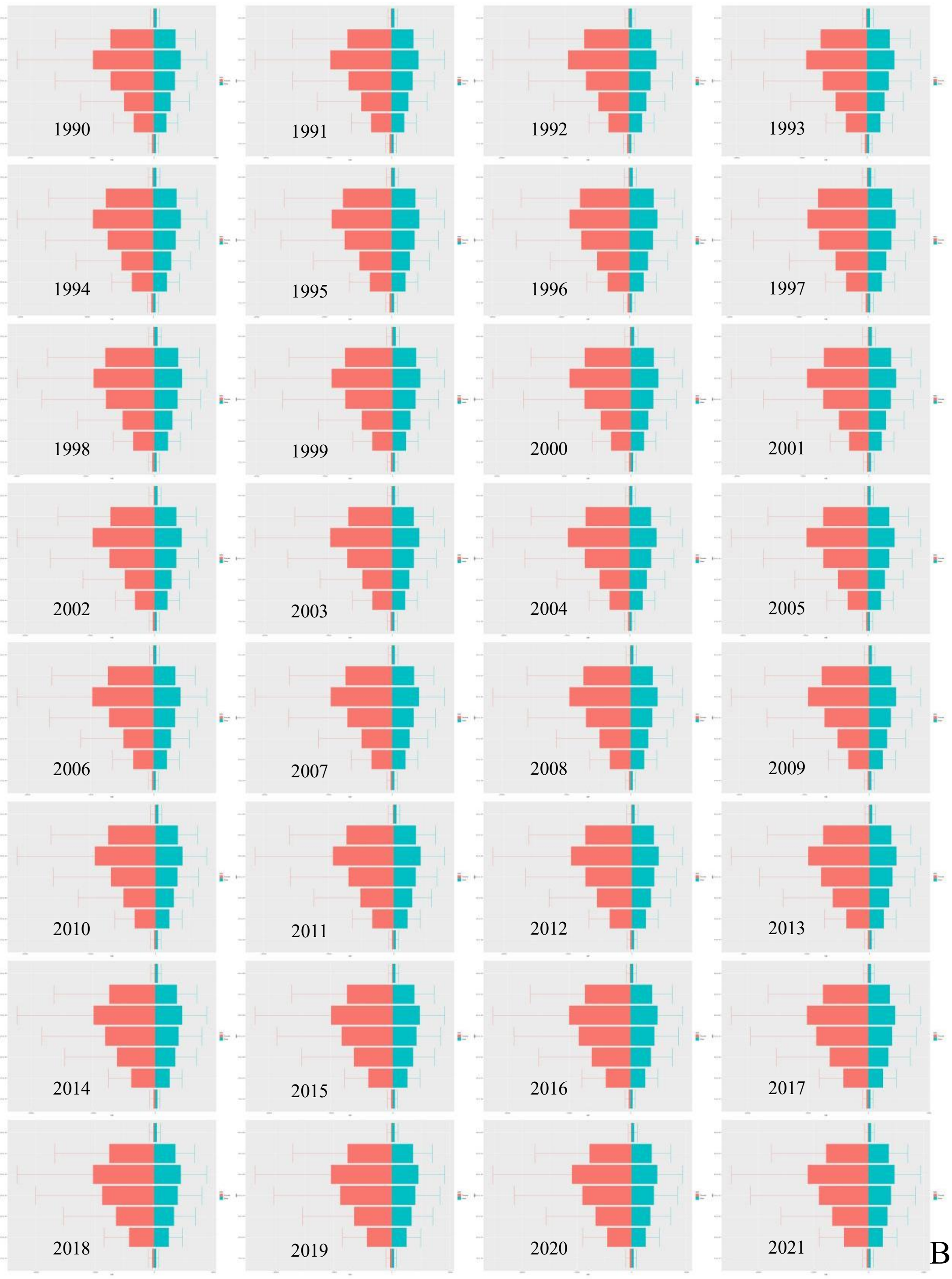
1990
1991
1992
1993
1994
1995
1996
1997
1998
1999
2000
2001
2002
2003
2004
2005
2006
2007
2008
2009
2010
2011
2012
2013
2014
2015
2016
2017
2018
2019
2020
2021
B

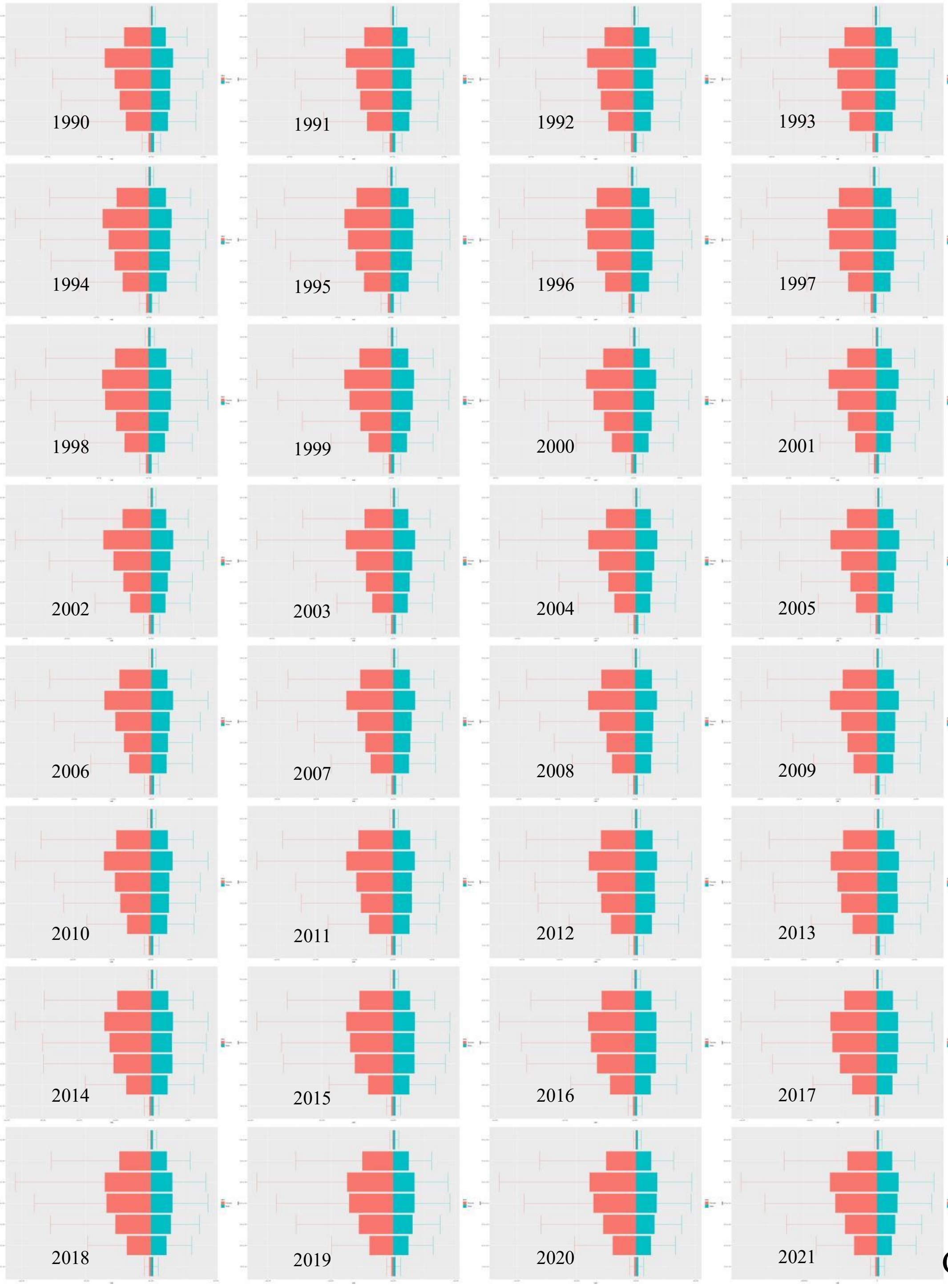
1990
1991
1992
1993
1994
1995
1996
1997
1998
1999
2000
2001
2002
2003
2004
2005
2006
2007
2008
2009
2010
2011
2012
2013
2014
2015
2016
2017
2018
2019
2020
2021

C

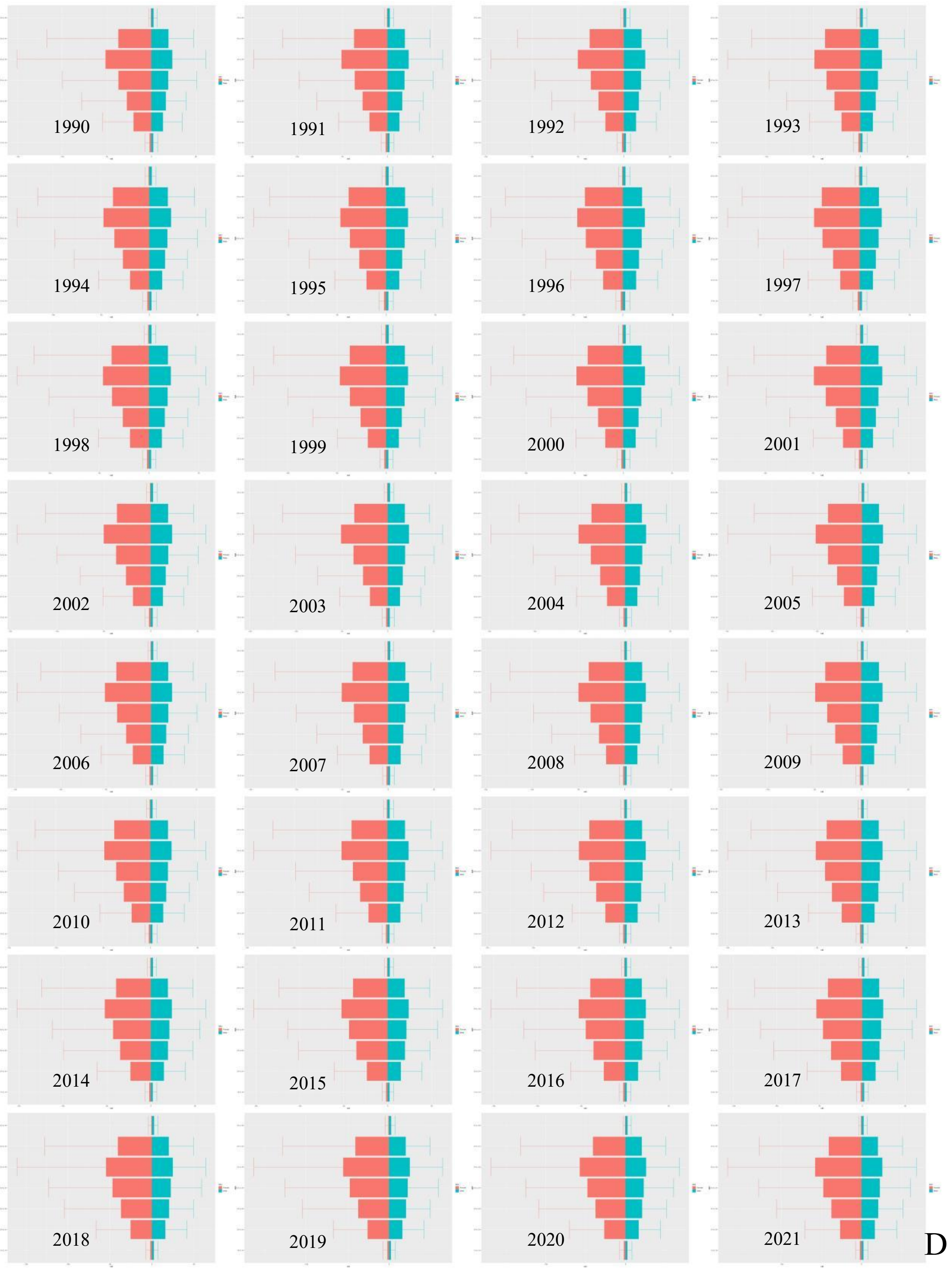

Fig. S32 (A) The prevalence cases of genecological disorders in different ages from 1990 to 2021 in the worldwide; (B) The prevalence rates of genecological disorders in different ages from 1990 to 2021 in the worldwide; (C) The years lived with disability of genecological disorders in different ages from 1990 to 2021 in the worldwide; (D) The years lived with disability rates of genecological disorders in different ages from 1990 to 2021 in the worldwide.

Notes: red for female, green for male; the ordinate from bottom to top is "15 to 19", "20 to 24", "25 to 29", "30 to 34", "35 to 39", "40 to 44", "45 to 49".

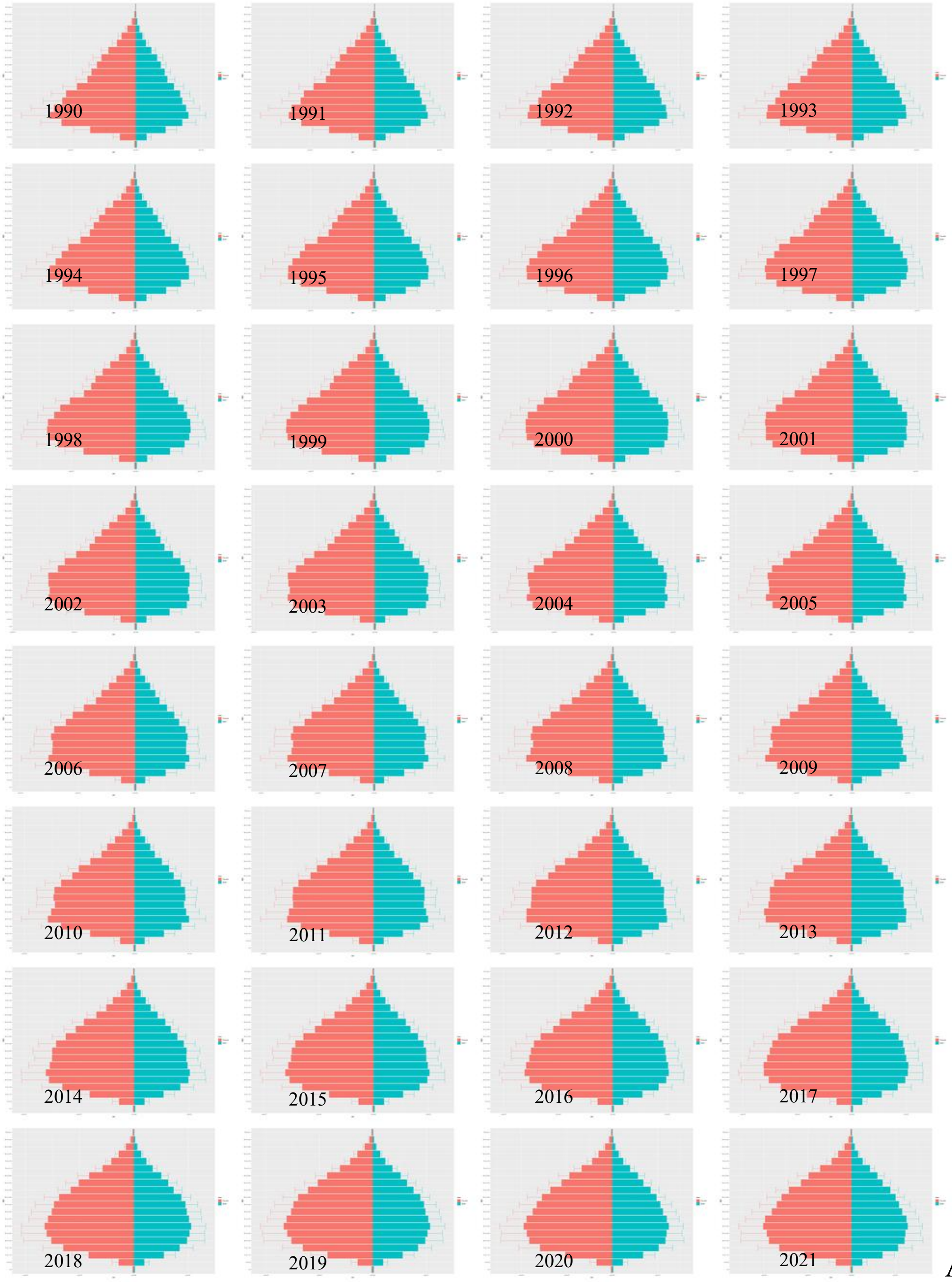
1990
1991
1992
1993
1994
1995
1996
1997
1998
1999
2000
2001
2002
2003
2004
2005
2006
2007
2008
2009
2010
2011
2012
2013
2014
2015
2016
2017
2018
2019
2020
2021

A

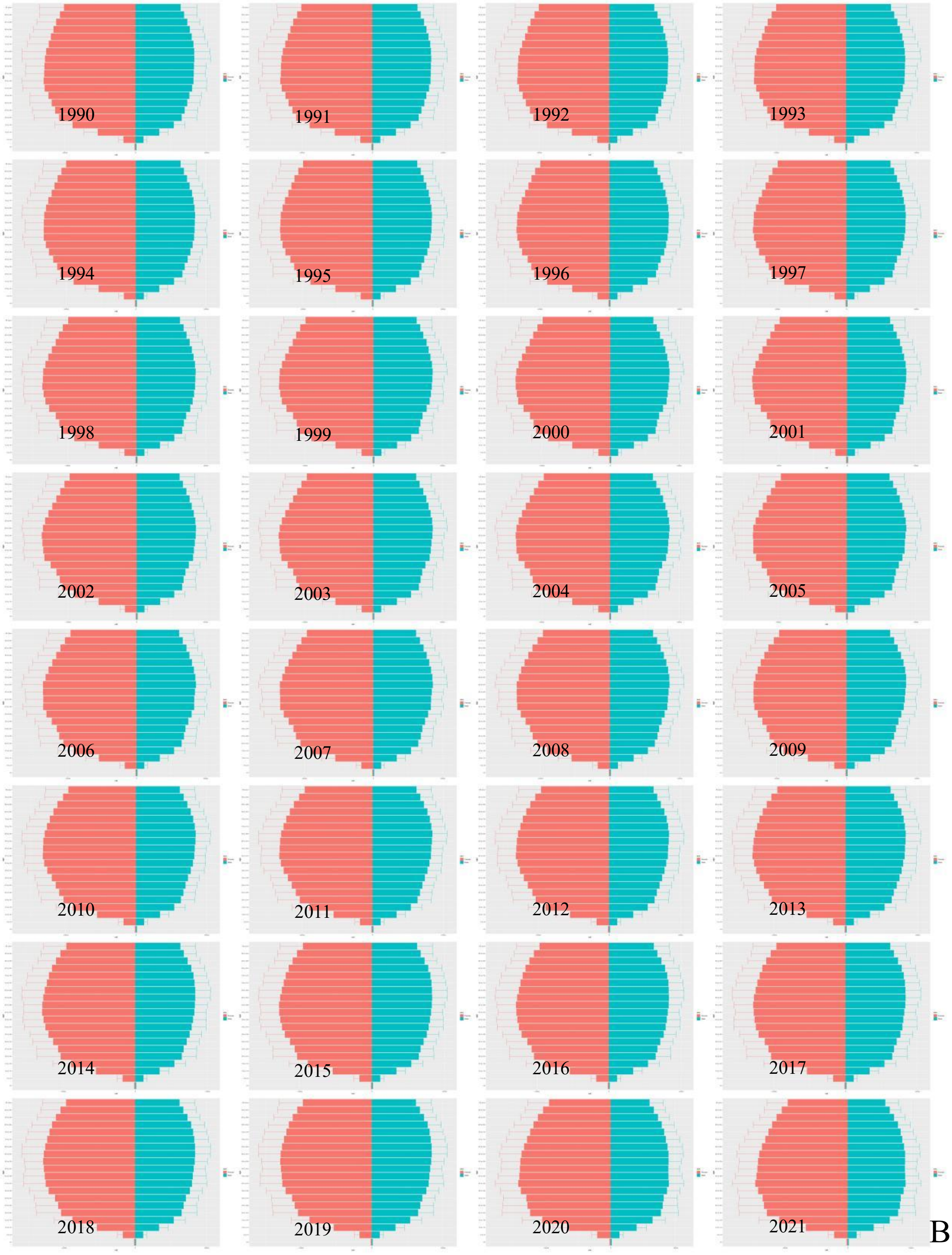
1990
1991
1992
1993
1994
1995
1996
1997
1998
1999
2000
2001
2002
2003
2004
2005
2006
2007
2008
2009
2010
2011
2012
2013
2014
2015
2016
2017
2018
2019
2020
2021
B

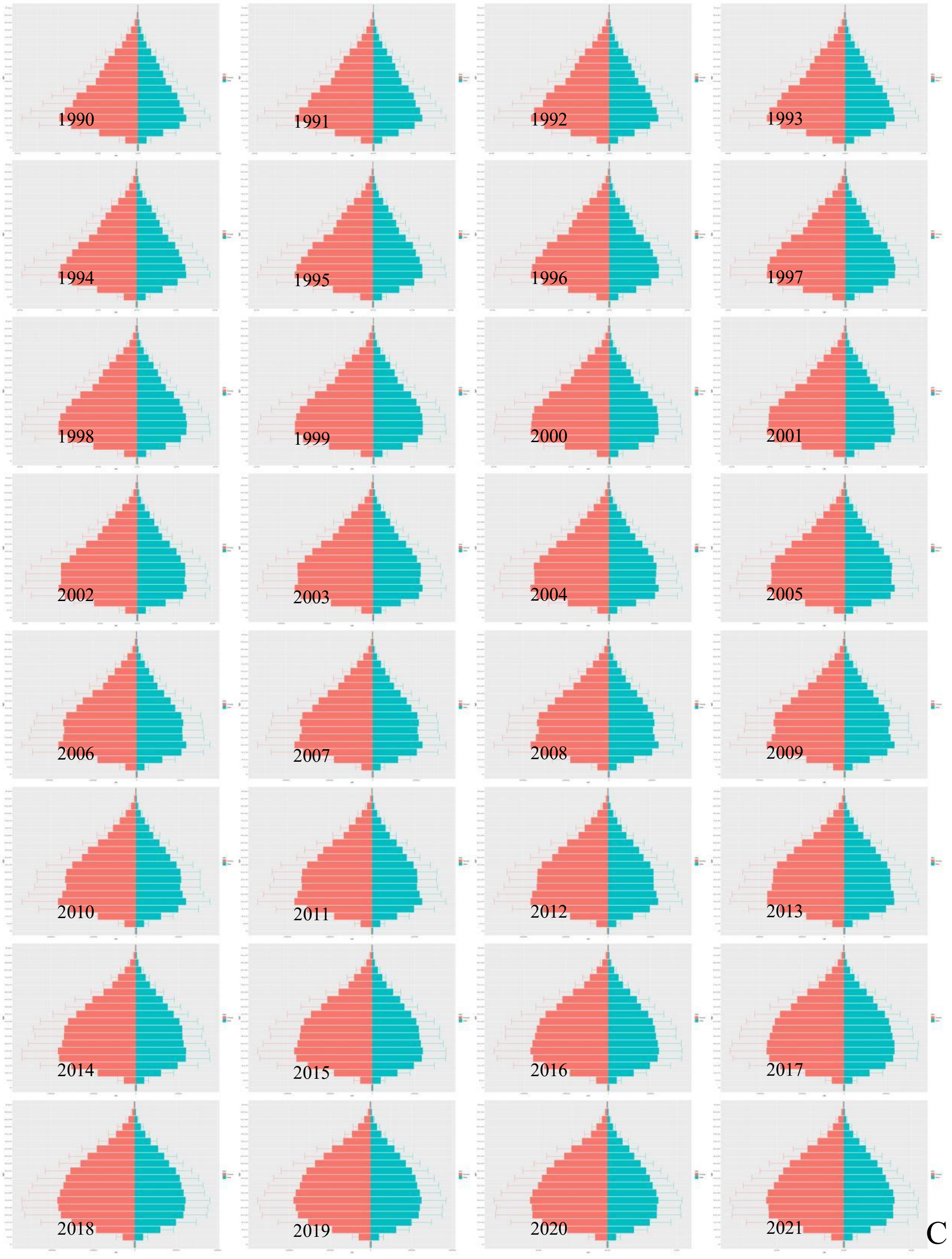
1990
1991
1992
1993
1994
1995
1996
1997
1998
1999
2000
2001
2002
2003
2004
2005
2006
2007
2008
2009
2010
2011
2012
2013
2014
2015
2016
2017
2018
2019
2020
2021
C

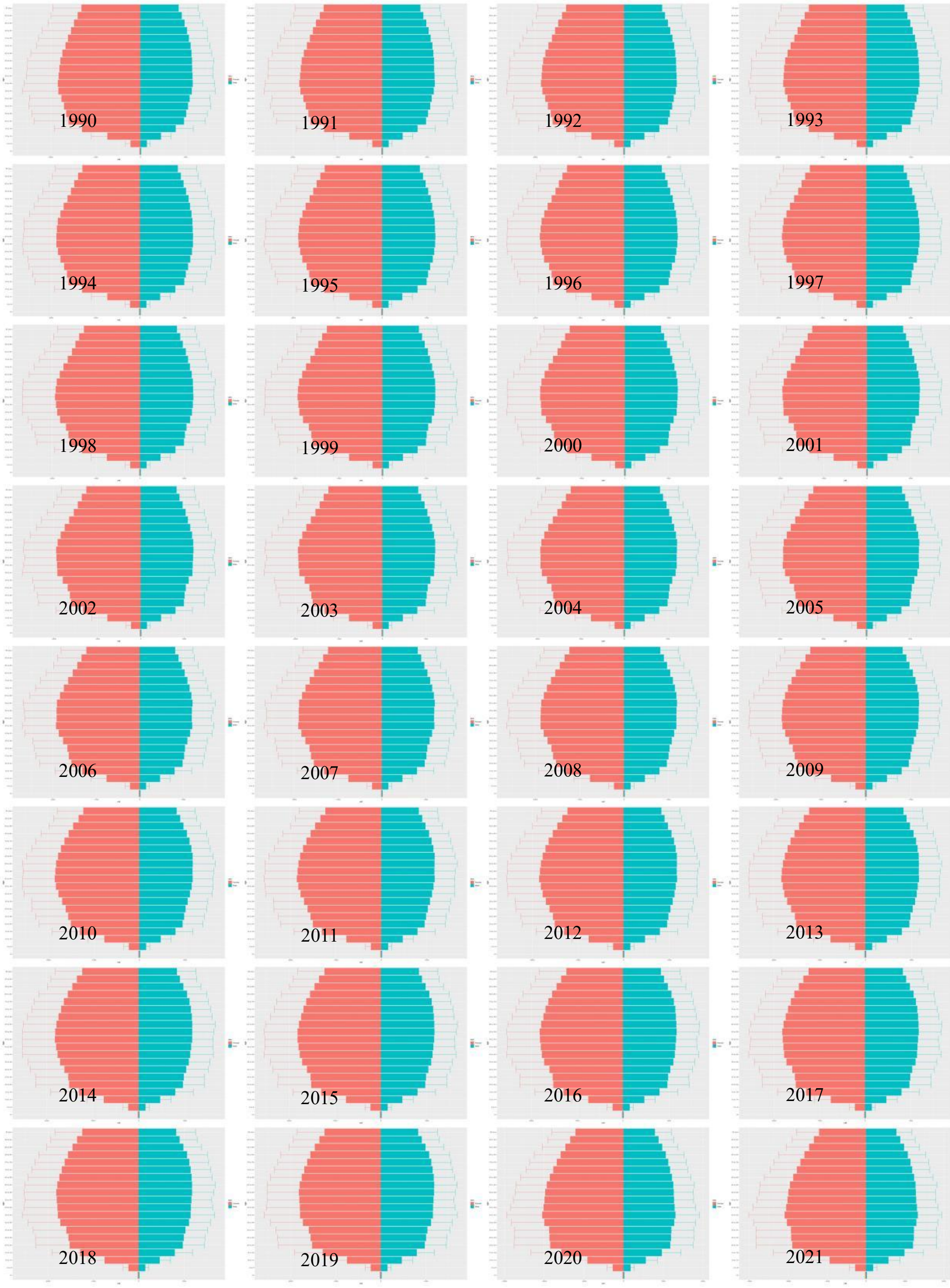

D

Fig. S33 (A) The prevalence cases of mental disorders in different ages from 1990 to 2021 in the worldwide; (B) The prevalence rates of mental disorders in different ages from 1990 to 2021 in the worldwide; (C) The years lived with disability of mental disorders in different ages from 1990 to 2021 in the worldwide; (D) The years lived with disability rates of mental disorders in different ages from 1990 to 2021 in the worldwide.

Notes: red for female, green for male; the ordinate from bottom to top is "<5", "5 to 9", "10 to 14", "15 to 19", "20 to 24", "25 to 29", "30 to 34", "35 to 39", "40 to 44", "45 to 49", "50 to 54", "55 to 59", "60 to 64", "65 to 69", "70 to 74", "75 to 79", "80 to 84", "85 to 89", "90 to 94", "95 plus".

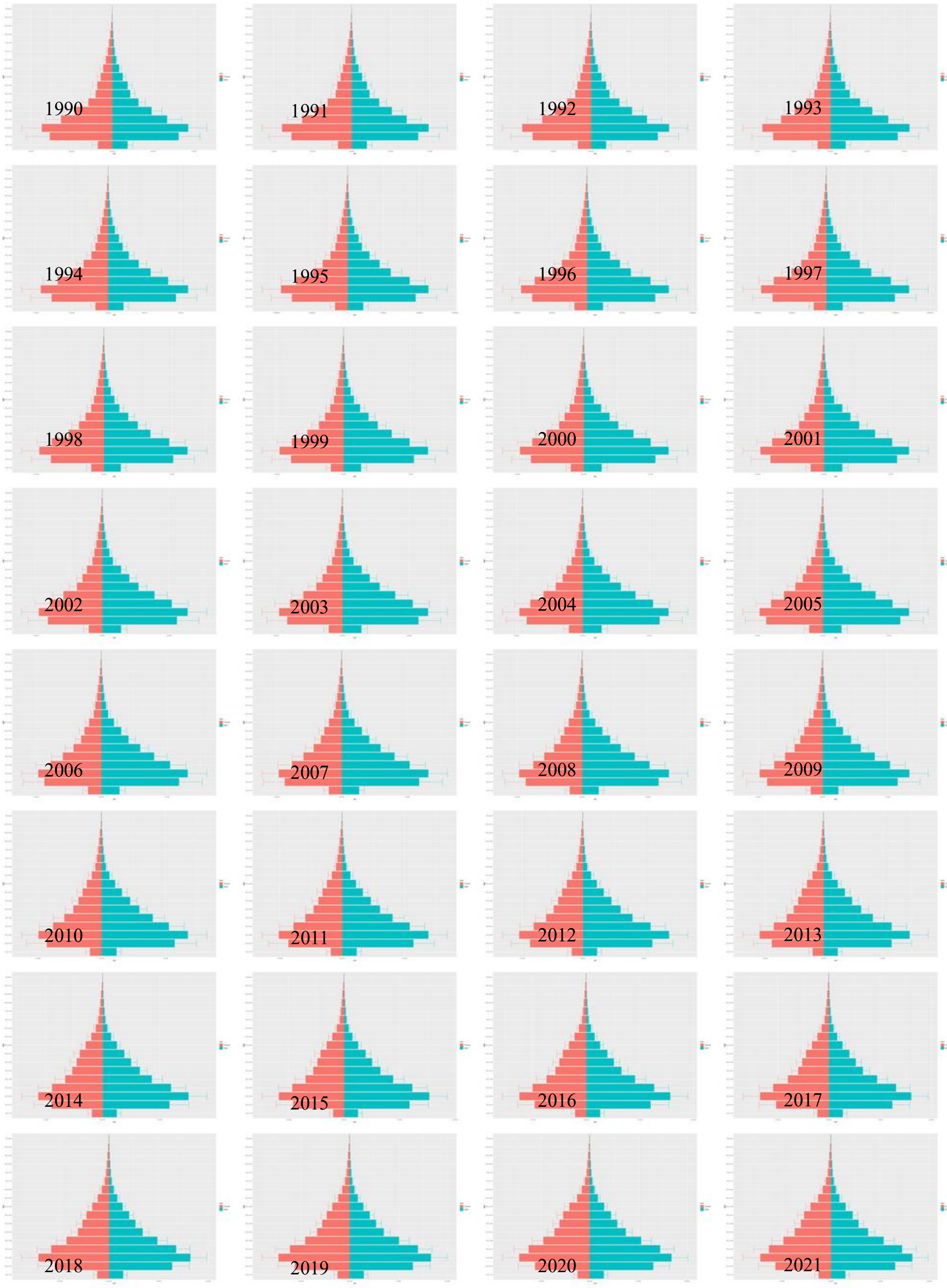
1990
1991
1992
1993
1994
1995
1996
1997
1998
1999
2000
2001
2002
2003
2004
2005
2006
2007
2008
2009
2010
2011
2012
2013
2014
2015
2016
2017
2018
2019
2020
2021

A

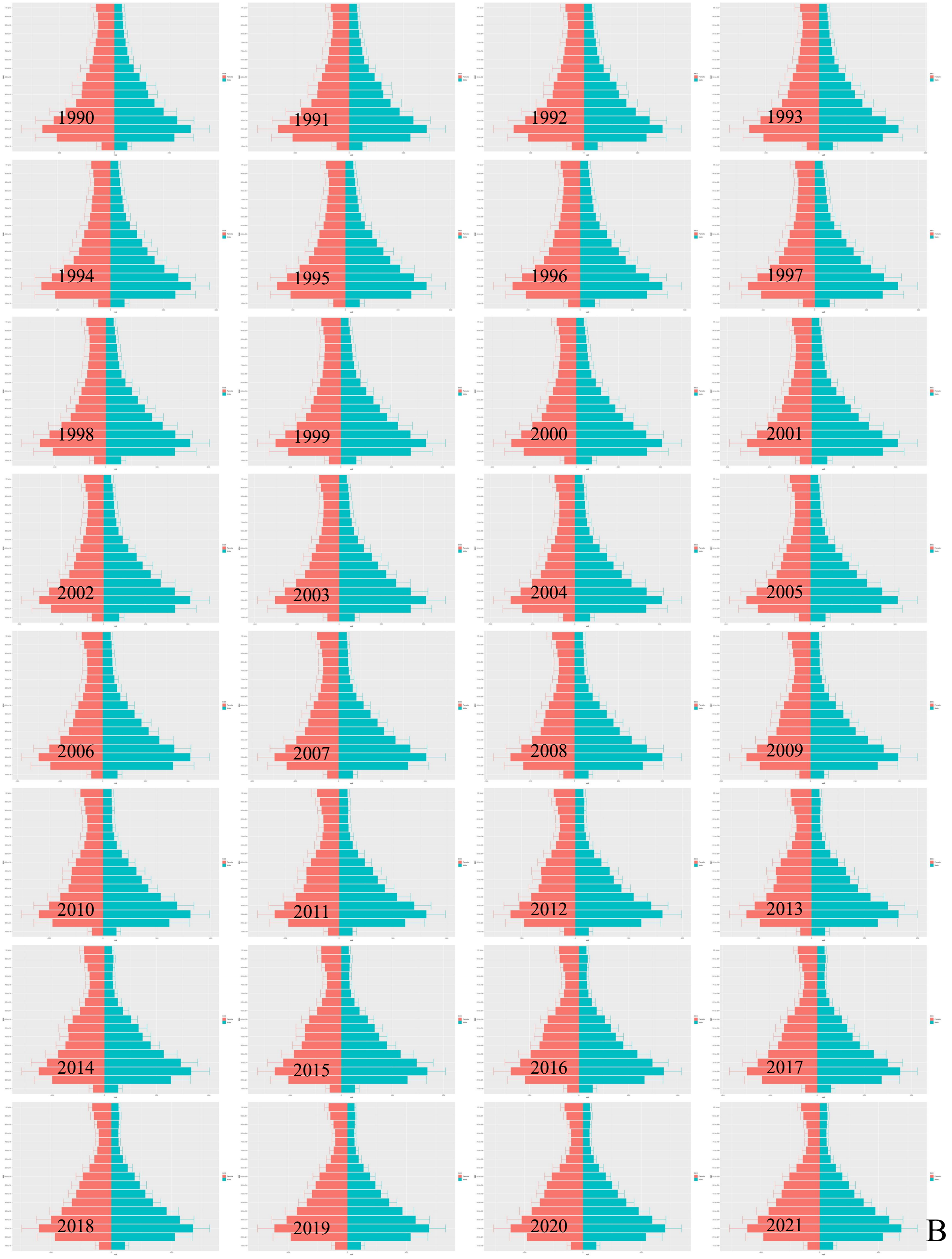
1990
1991
1992
1993
1994
1995
1996
1997
1998
1999
2000
2001
2002
2003
2004
2005
2006
2007
2008
2009
2010
2011
2012
2013
2014
2015
2016
2017
2018
2019
2020
2021
B

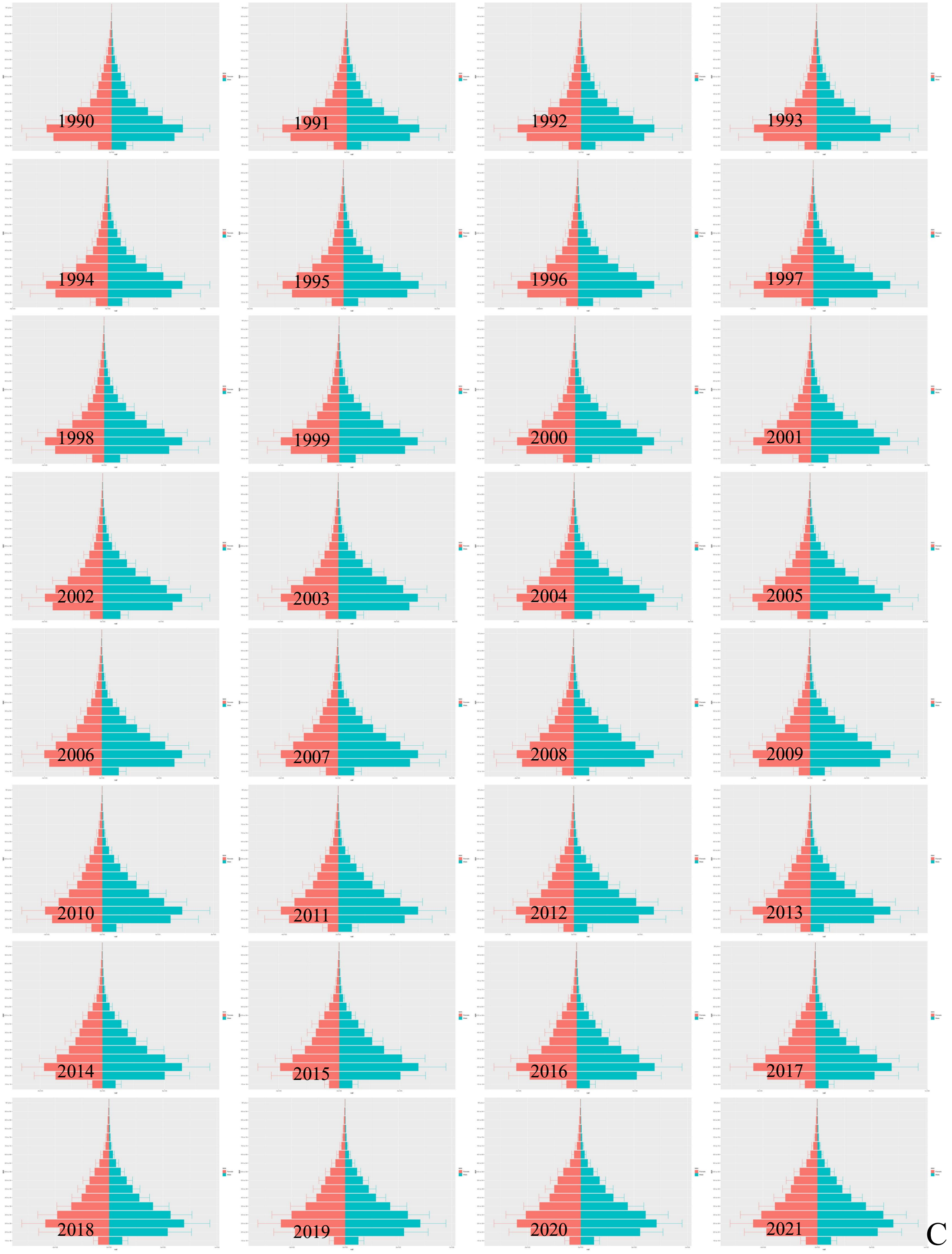
1990
1991
1992
1993
1994
1995
1996
1997
1998
1999
2000
2001
2002
2003
2004
2005
2006
2007
2008
2009
2010
2011
2012
2013
2014
2015
2016
2017
2018
2019
2020
2021
C

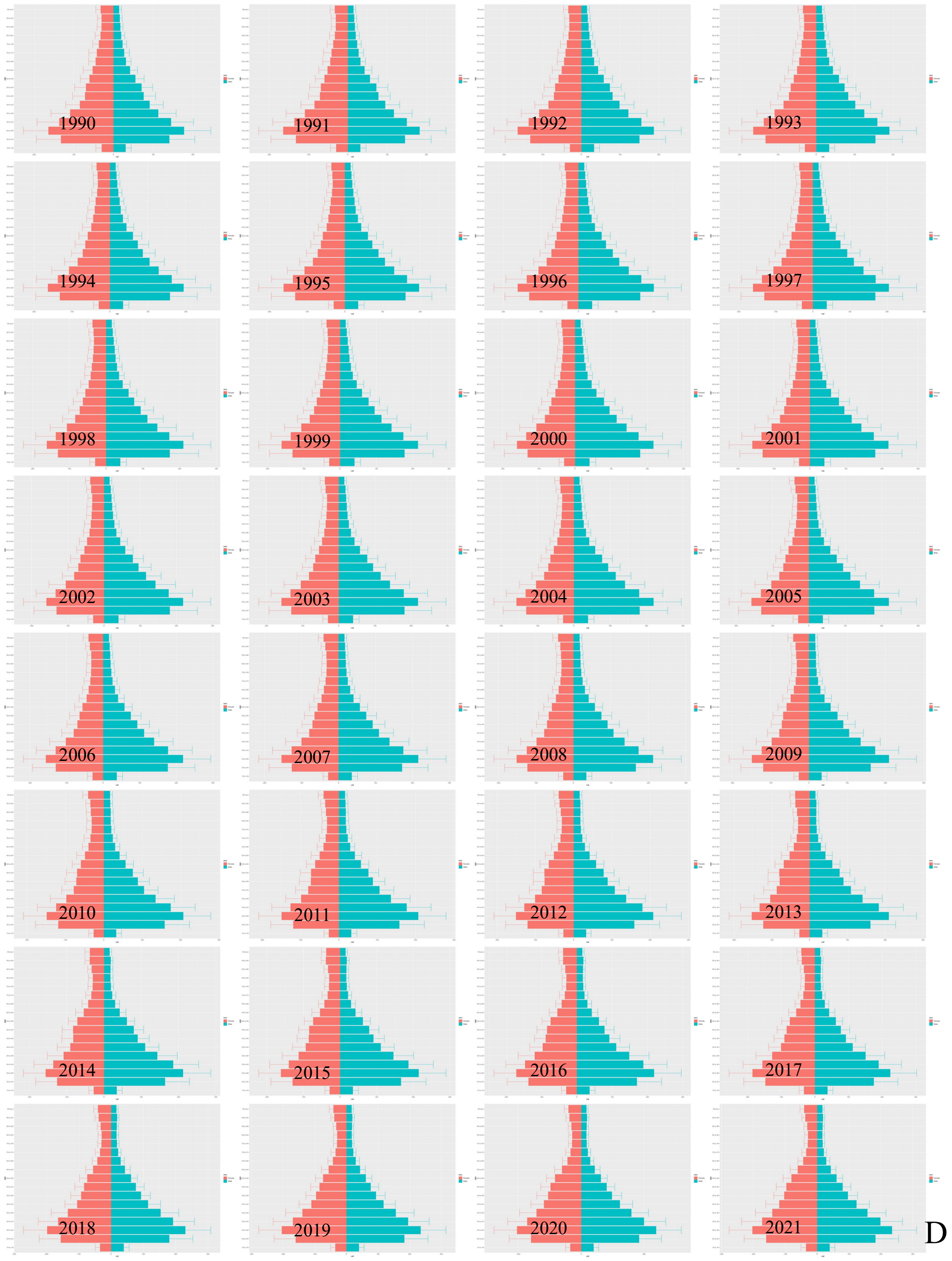

Fig. S34 (A) The prevalence cases of substance use disorders in different ages from 1990 to 2021 in the worldwide; (B) The prevalence rates of substance use disorders in different ages from 1990 to 2021 in the worldwide; (C) The years lived with disability of substance use disorders in different ages from 1990 to 2021 in the worldwide; (D) The years lived with disability rates of substance use disorders in different ages from 1990 to 2021 in the worldwide.

Notes: red for female, green for male; the ordinate from bottom to top is "15 to 19", "20 to 24", "25 to 29", "30 to 34", "35 to 39", "40 to 44", "45 to 49", "50 to 54", "55 to 59", "60 to 64", "65 to 69", "70 to 74", "75 to 79", "80 to 84", "85 to 89", "90 to 94", "95 plus".

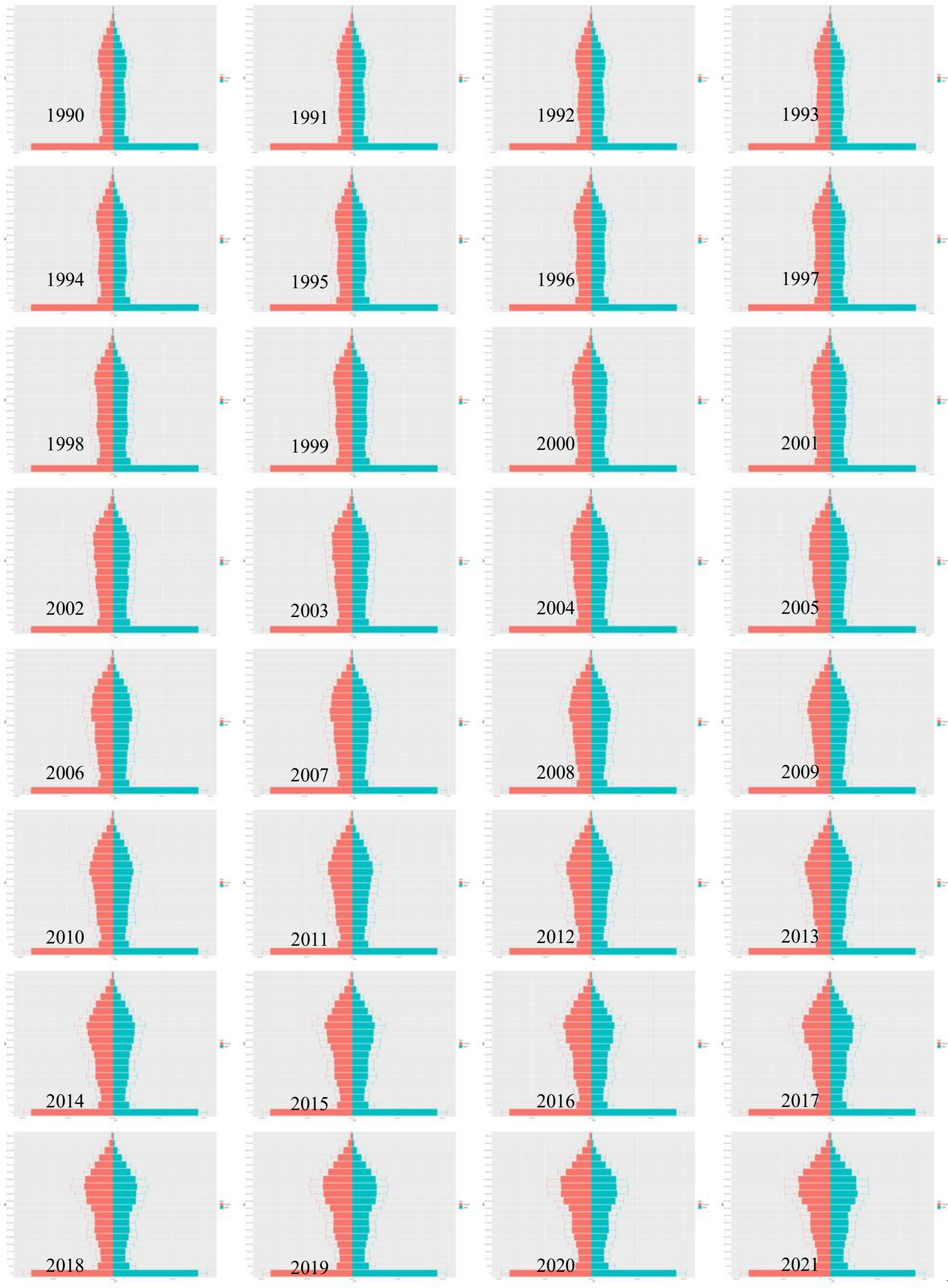
1990
1991
1992
1993
1994
1995
1996
1997
1998
1999
2000
2001
2002
2003
2004
2005
2006
2007
2008
2009
2010
2011
2012
2013
2014
2015
2016
2017
2018
2019
2020
2021

A

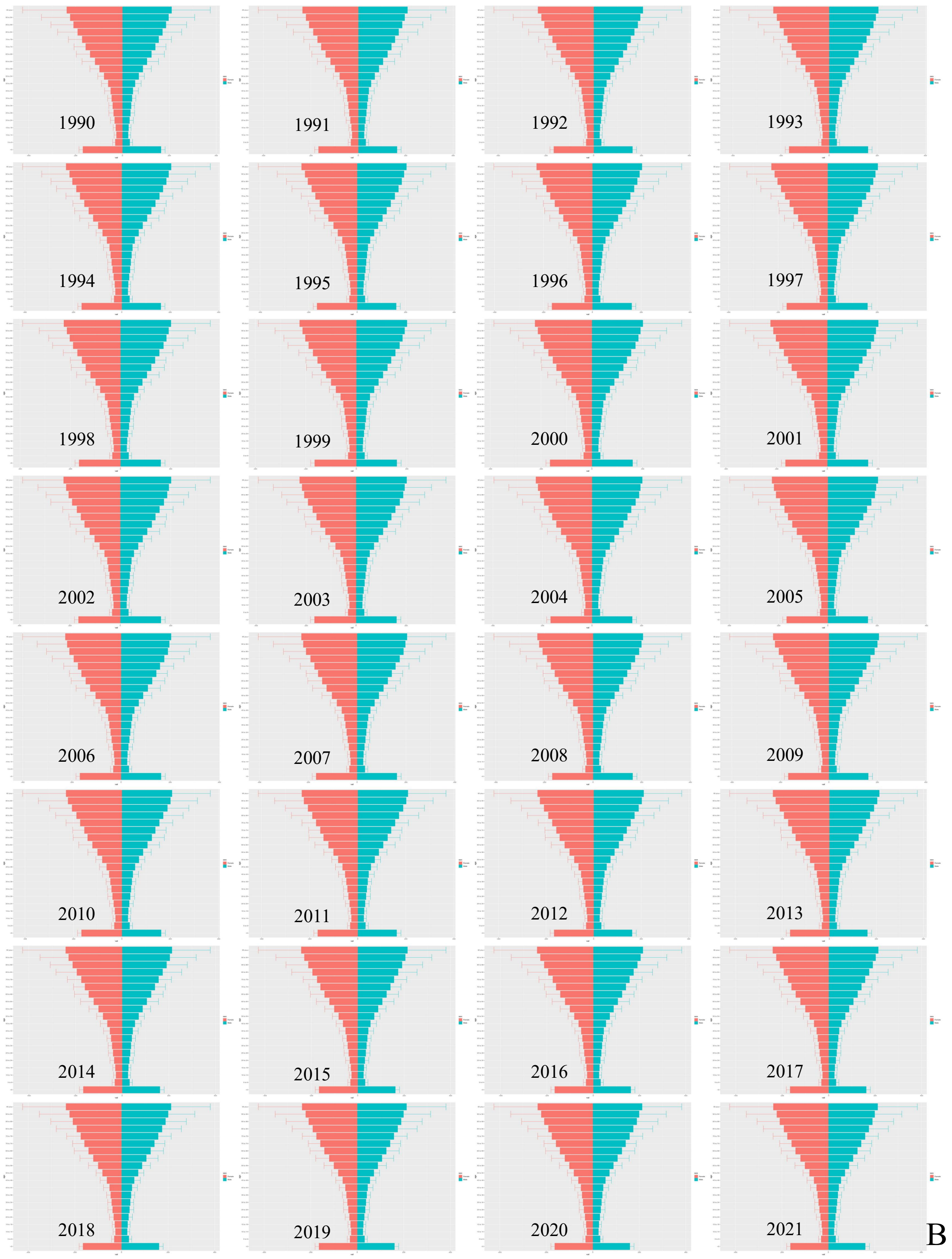
1990
1991
1992
1993
1994
1995
1996
1997
1998
1999
2000
2001
2002
2003
2004
2005
2006
2007
2008
2009
2010
2011
2012
2013
2014
2015
2016
2017
2018
2019
2020
2021
B

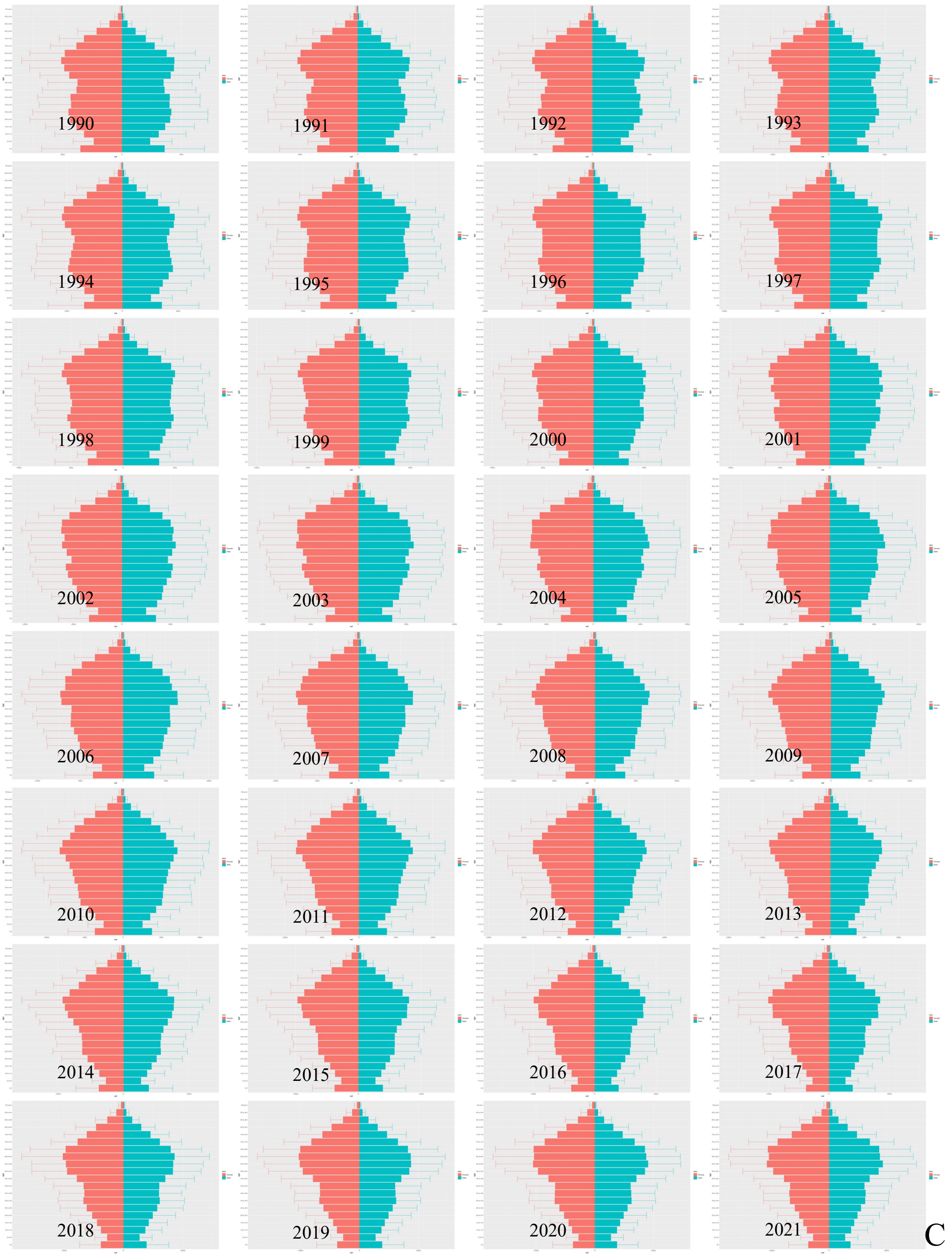
1990
1991
1992
1993
1994
1995
1996
1997
1998
1999
2000
2001
2002
2003
2004
2005
2006
2007
2008
2009
2010
2011
2012
2013
2014
2015
2016
2017
2018
2019
2020
2021
C

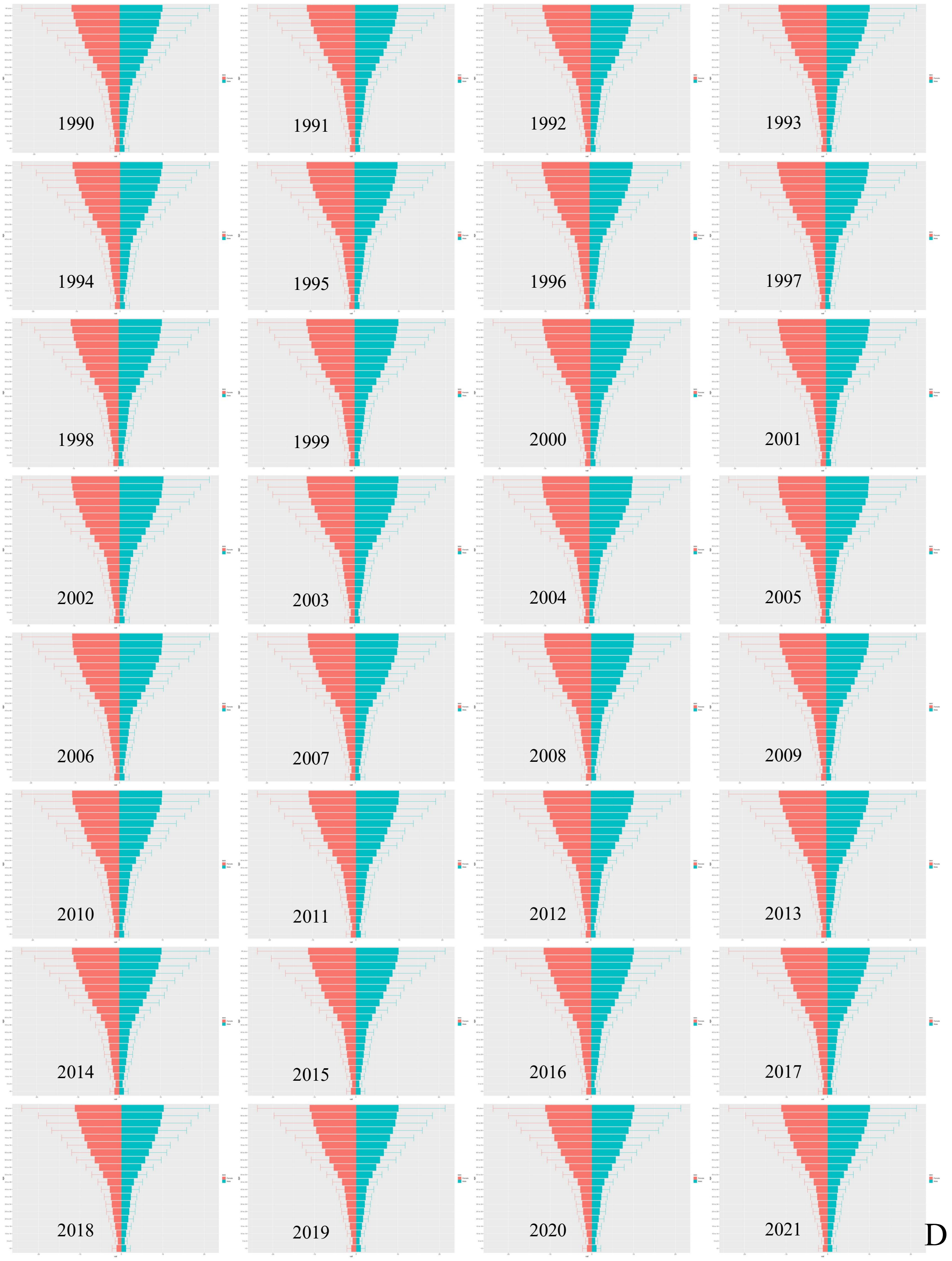

Fig. S35 (A) The prevalence cases of infectious disorders in different ages from 1990 to 2021 in the worldwide; (B) The prevalence rates of infectious disorders in different ages from 1990 to 2021 in the worldwide; (C) The years lived with disability of infectious disorders in different ages from 1990 to 2021 in the worldwide; (D) The years lived with disability rates of infectious disorders in different ages from 1990 to 2021 in the worldwide.

Notes: red for female, green for male; the ordinate from bottom to top is "<5", "5 to 9", "10 to 14", "15 to 19", "20 to 24", "25 to 29", "30 to 34", "35 to 39", "40 to 44", "45 to 49", "50 to 54", "55 to 59", "60 to 64", "65 to 69", "70 to 74", "75 to 79", "80 to 84", "85 to 89", "90 to 94", "95 plus".

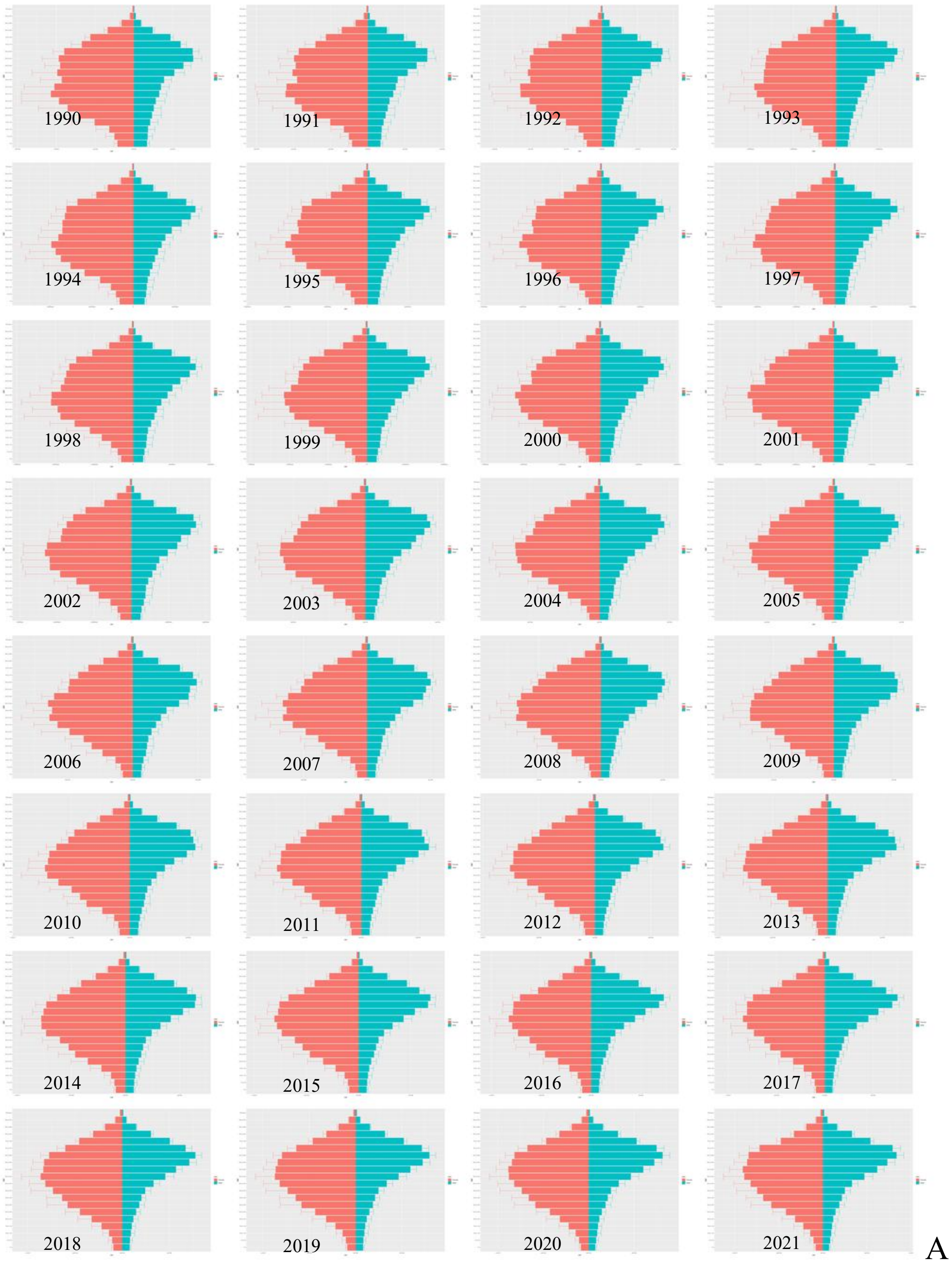
1990
1991
1992
1993
1994
1995
1996
1997
1998
1999
2000
2001
2002
2003
2004
2005
2006
2007
2008
2009
2010
2011
2012
2013
2014
2015
2016
2017
2018
2019
2020
2021

A

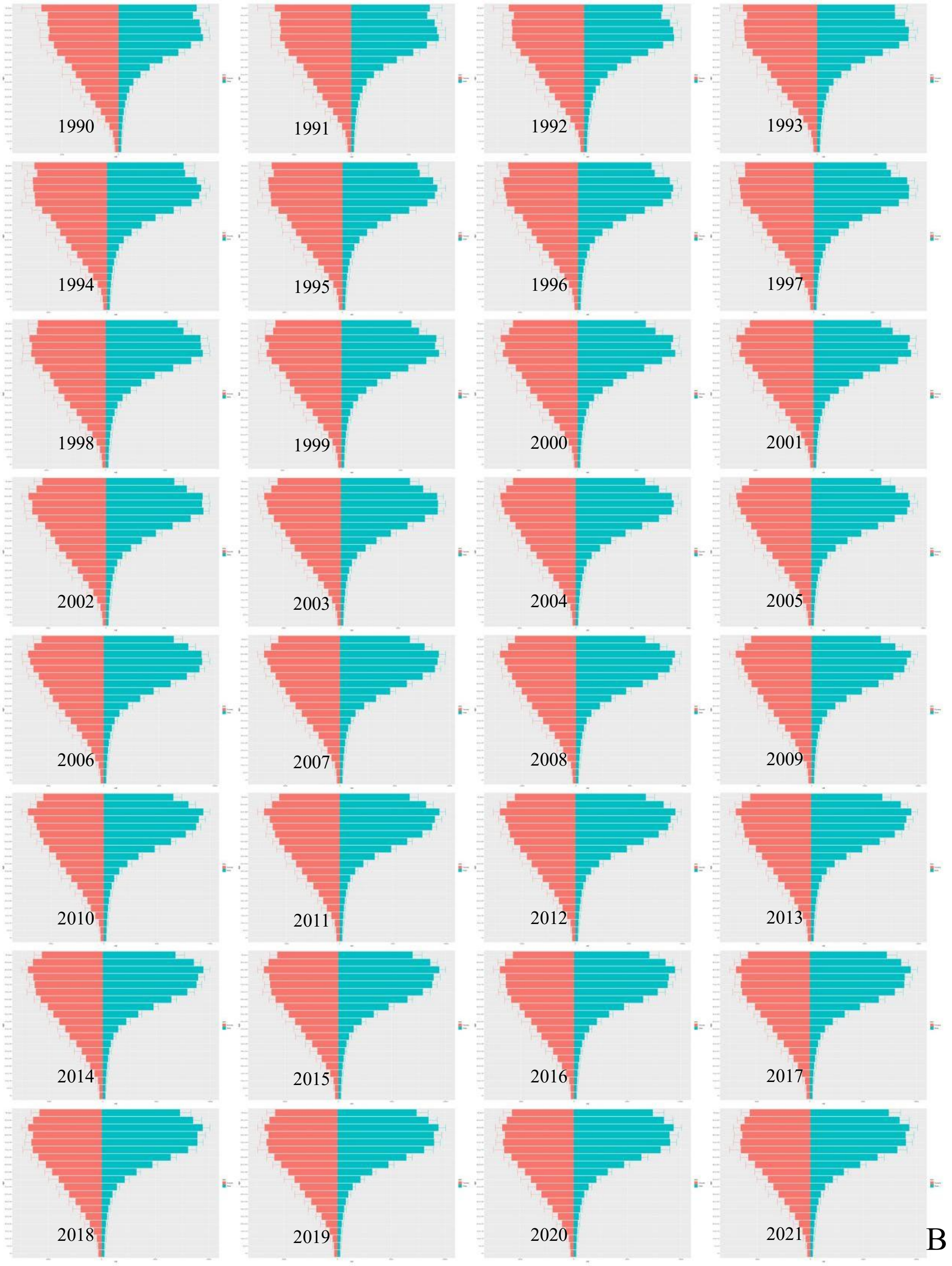
1990
1991
1992
1993
1994
1995
1996
1997
1998
1999
2000
2001
2002
2003
2004
2005
2006
2007
2008
2009
2010
2011
2012
2013
2014
2015
2016
2017
2018
2019
2020
2021
B

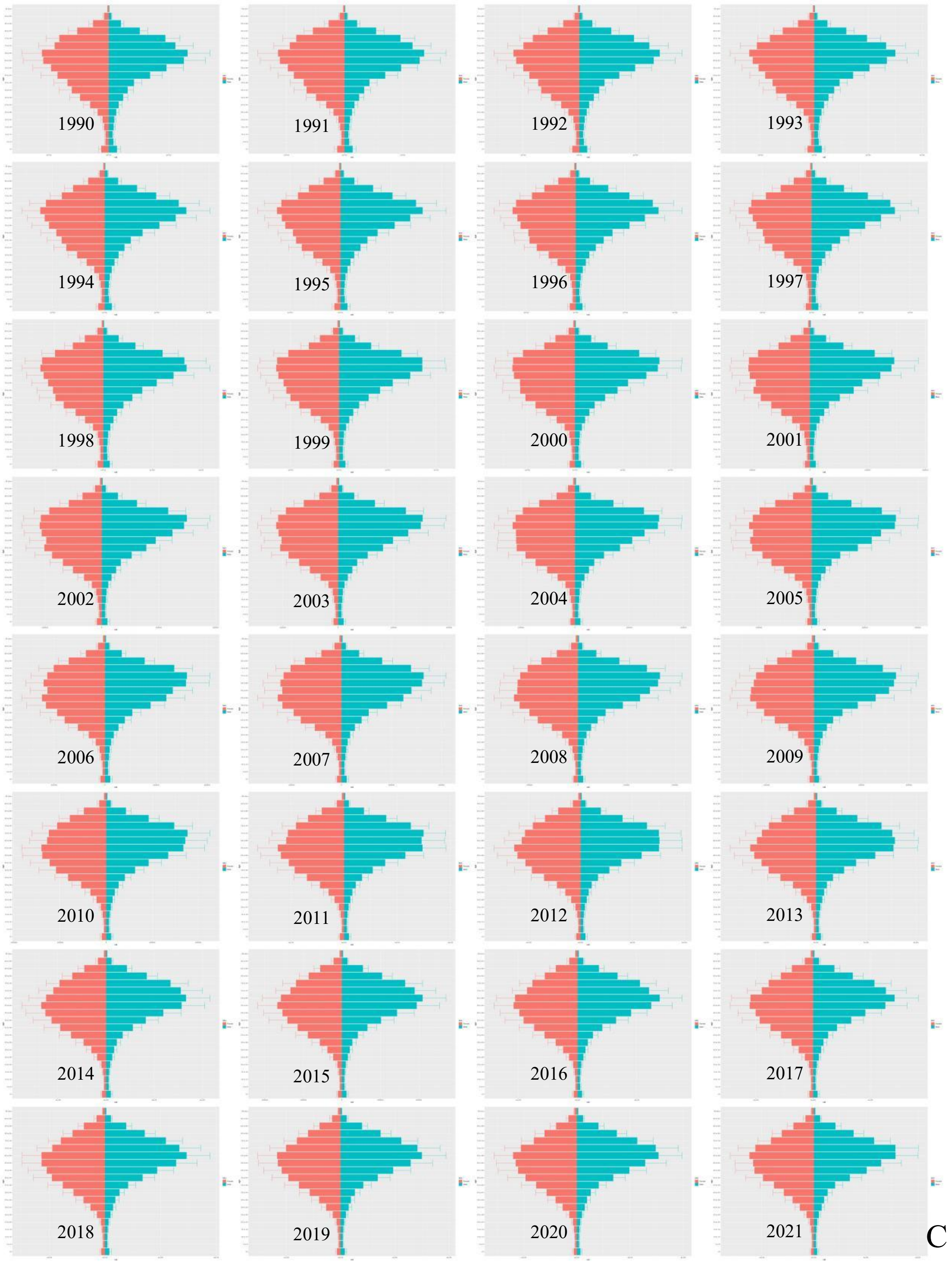
1990
1991
1992
1993
1994
1995
1996
1997
1998
1999
2000
2001
2002
2003
2004
2005
2006
2007
2008
2009
2010
2011
2012
2013
2014
2015
2016
2017
2018
2019
2020
2021
C

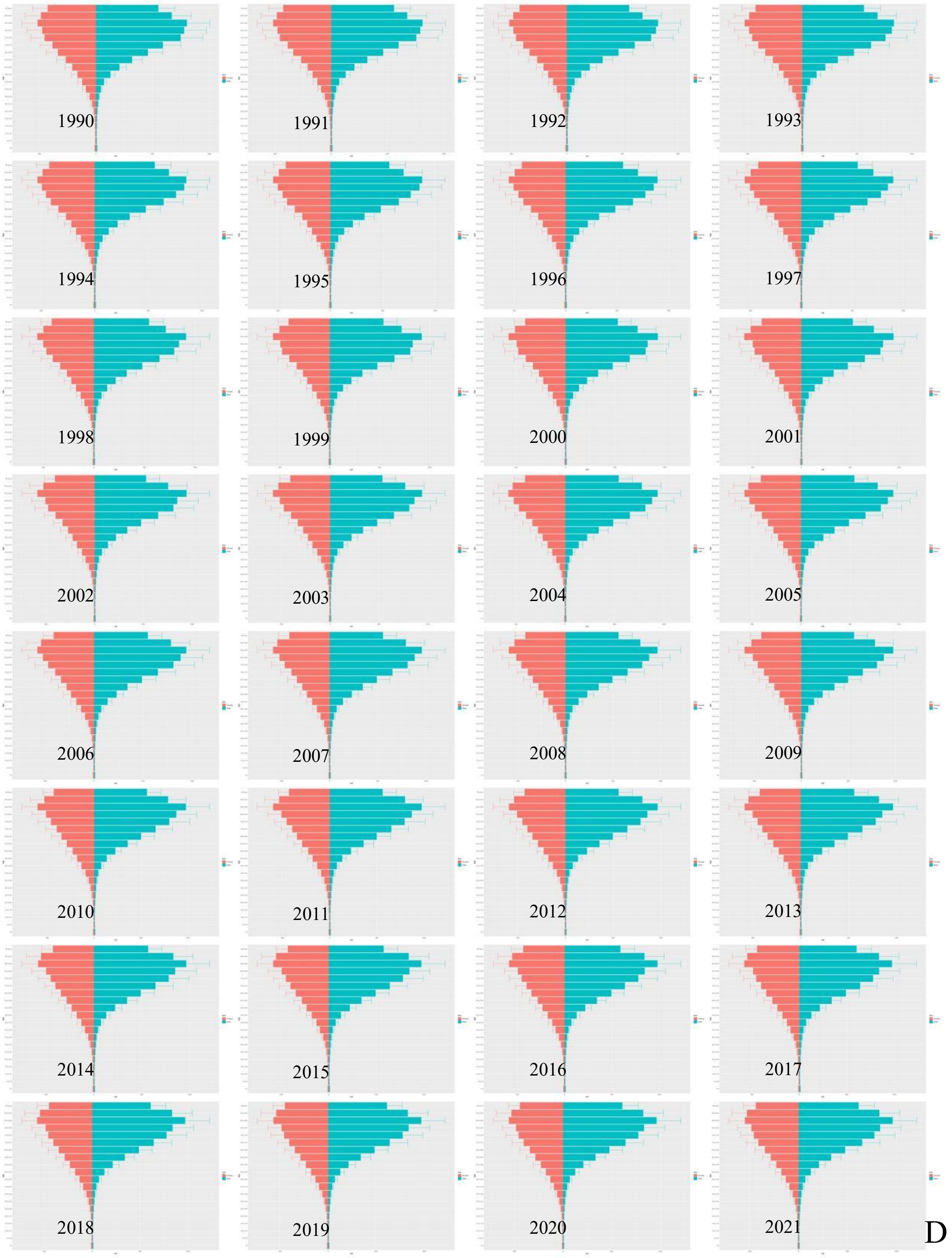

Fig. S36 (A) The prevalence cases of neoplasms in different ages from 1990 to 2021 in the worldwide; (B) The prevalence rates of neoplasms in different ages from 1990 to 2021 in the worldwide; (C) The years lived with disability of neoplasms in different ages from 1990 to 2021 in the worldwide; (D) The years lived with disability rates of neoplasms in different ages from 1990 to 2021 in the worldwide.

Notes: red for female, green for male; the ordinate from bottom to top is "<5", "5 to 9", "10 to 14", "15 to 19", "20 to 24", "25 to 29", "30 to 34", "35 to 39", "40 to 44", "45 to 49", "50 to 54", "55 to 59", "60 to 64", "65 to 69", "70 to 74", "75 to 79", "80 to 84", "85 to 89", "90 to 94", "95 plus".

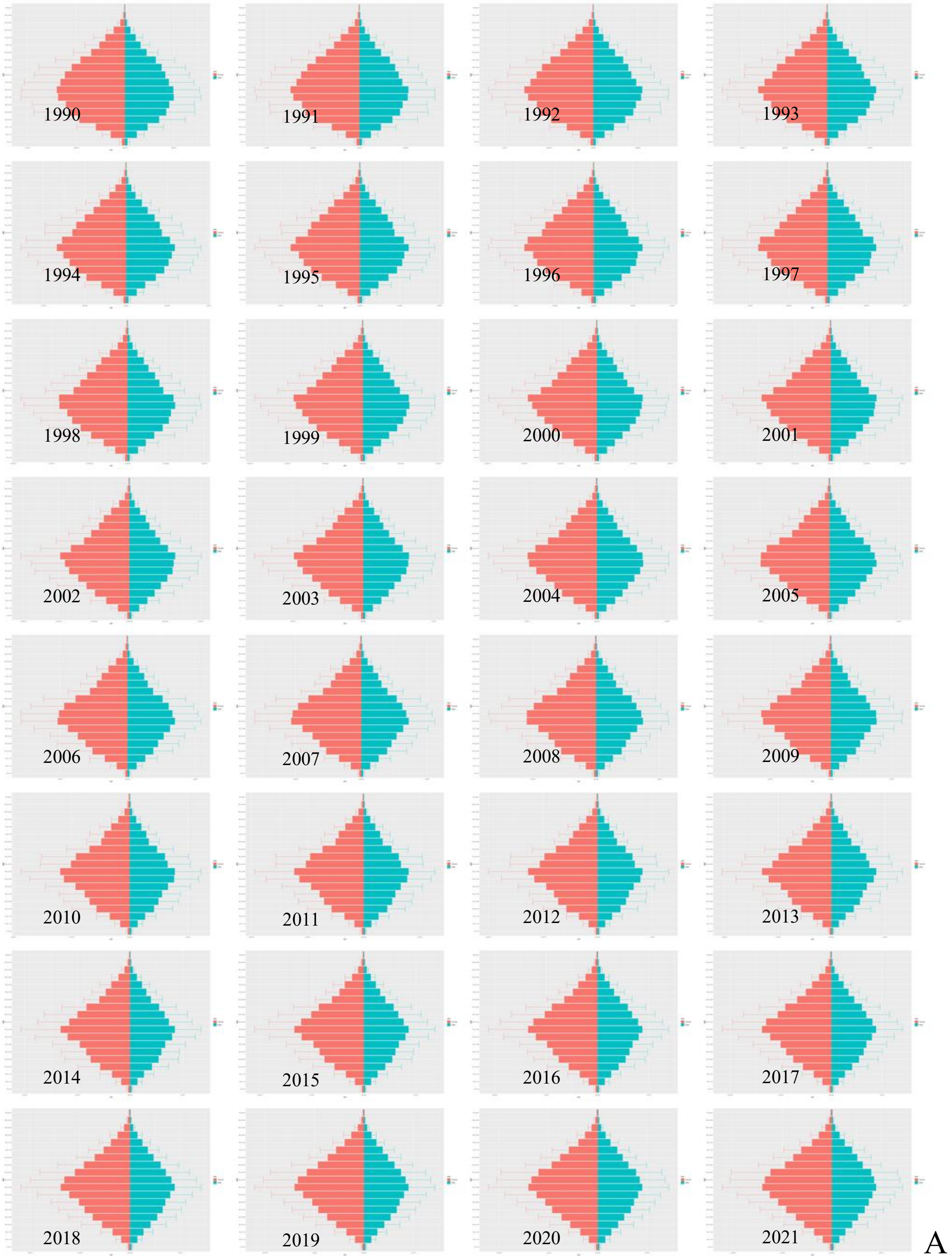
1990
1991
1992
1993
1994
1995
1996
1997
1998
1999
2000
2001
2002
2003
2004
2005
2006
2007
2008
2009
2010
2011
2012
2013
2014
2015
2016
2017
2018
2019
2020
2021
A

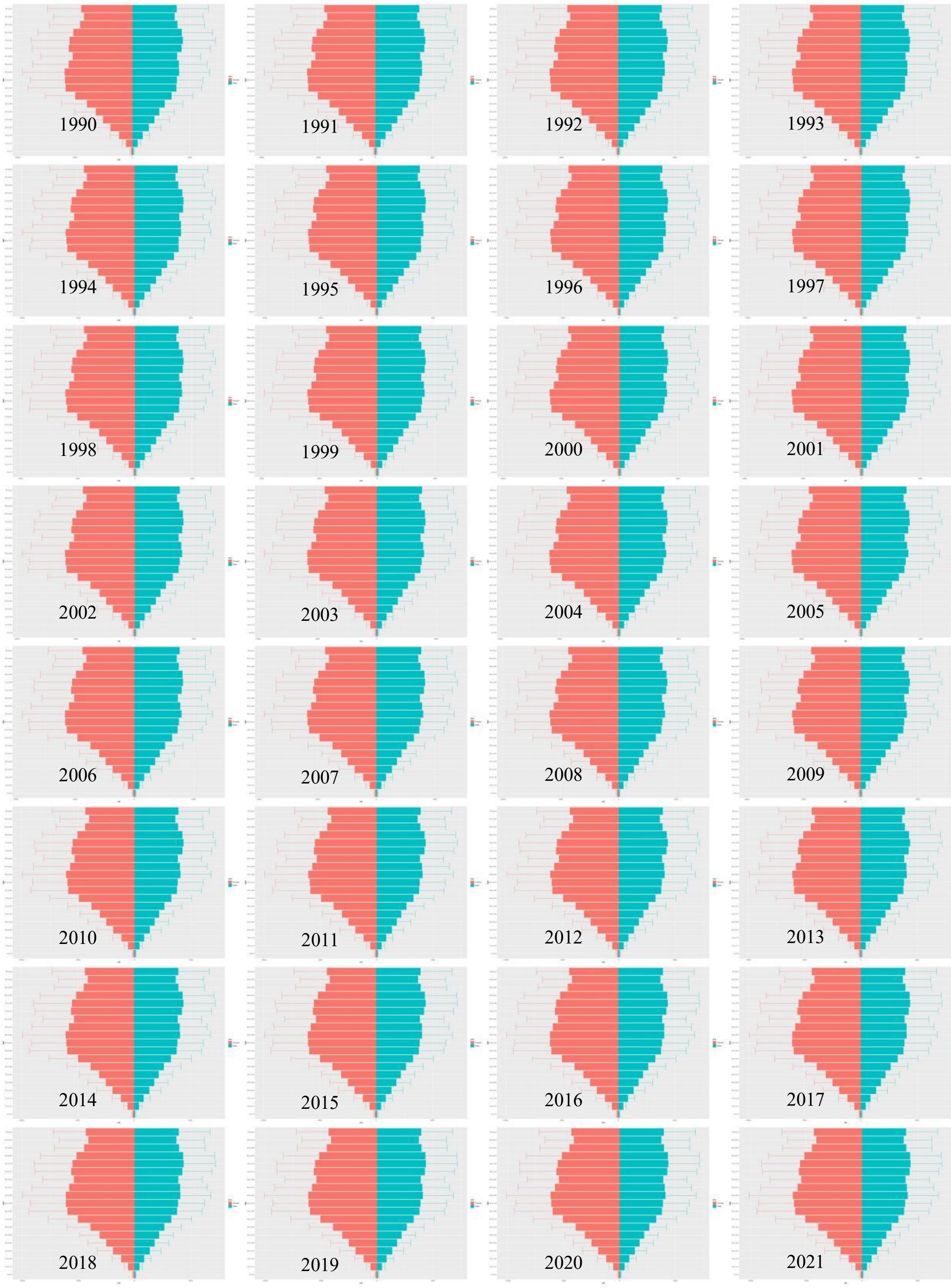
1990
1991
1992
1993
1994
1995
1996
1997
1998
1999
2000
2001
2002
2003
2004
2005
2006
2007
2008
2009
2010
2011
2012
2013
2014
2015
2016
2017
2018
2019
2020
2021

B

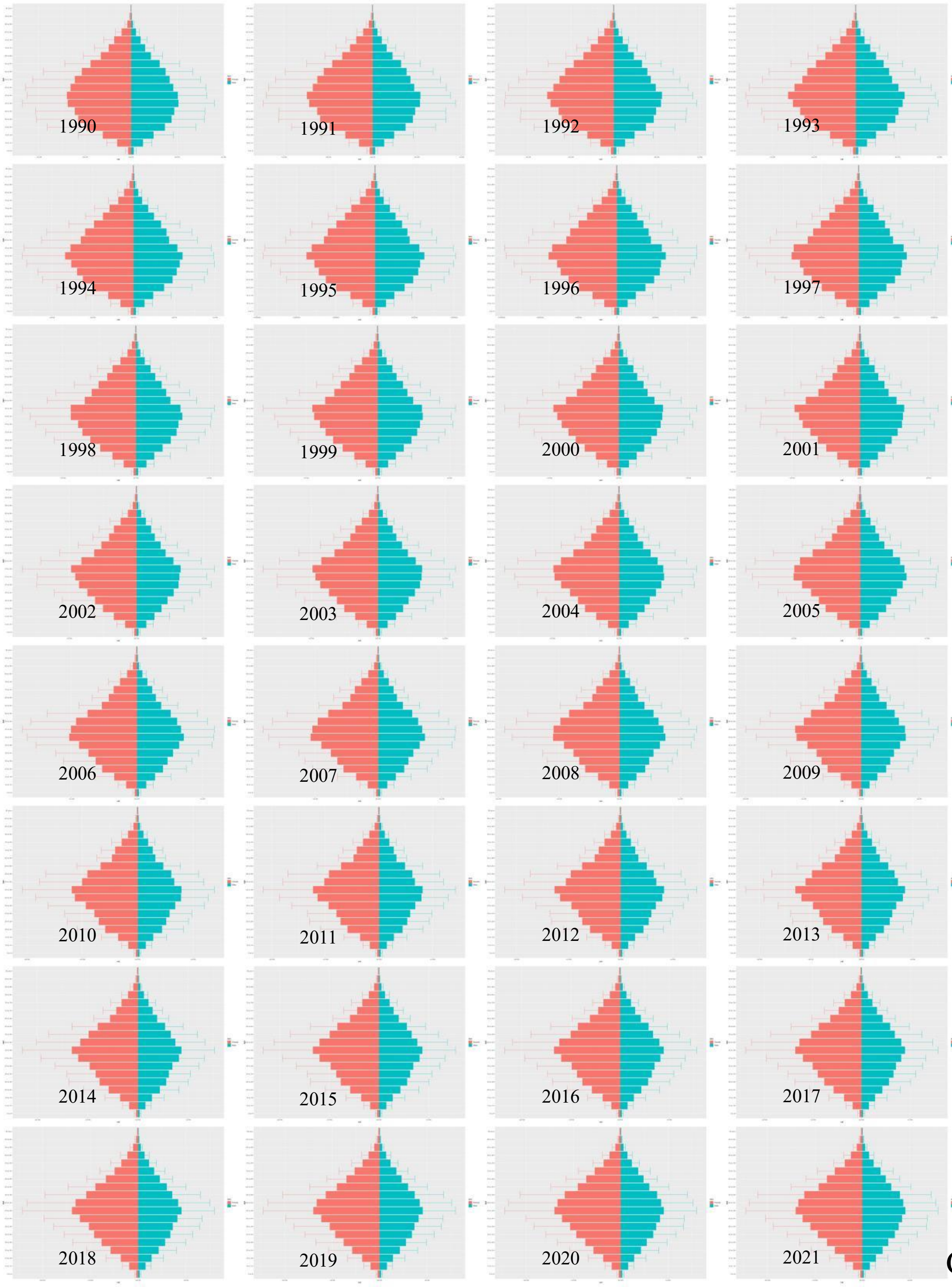
1990
1991
1992
1993
1994
1995
1996
1997
1998
1999
2000
2001
2002
2003
2004
2005
2006
2007
2008
2009
2010
2011
2012
2013
2014
2015
2016
2017
2018
2019
2020
2021

C

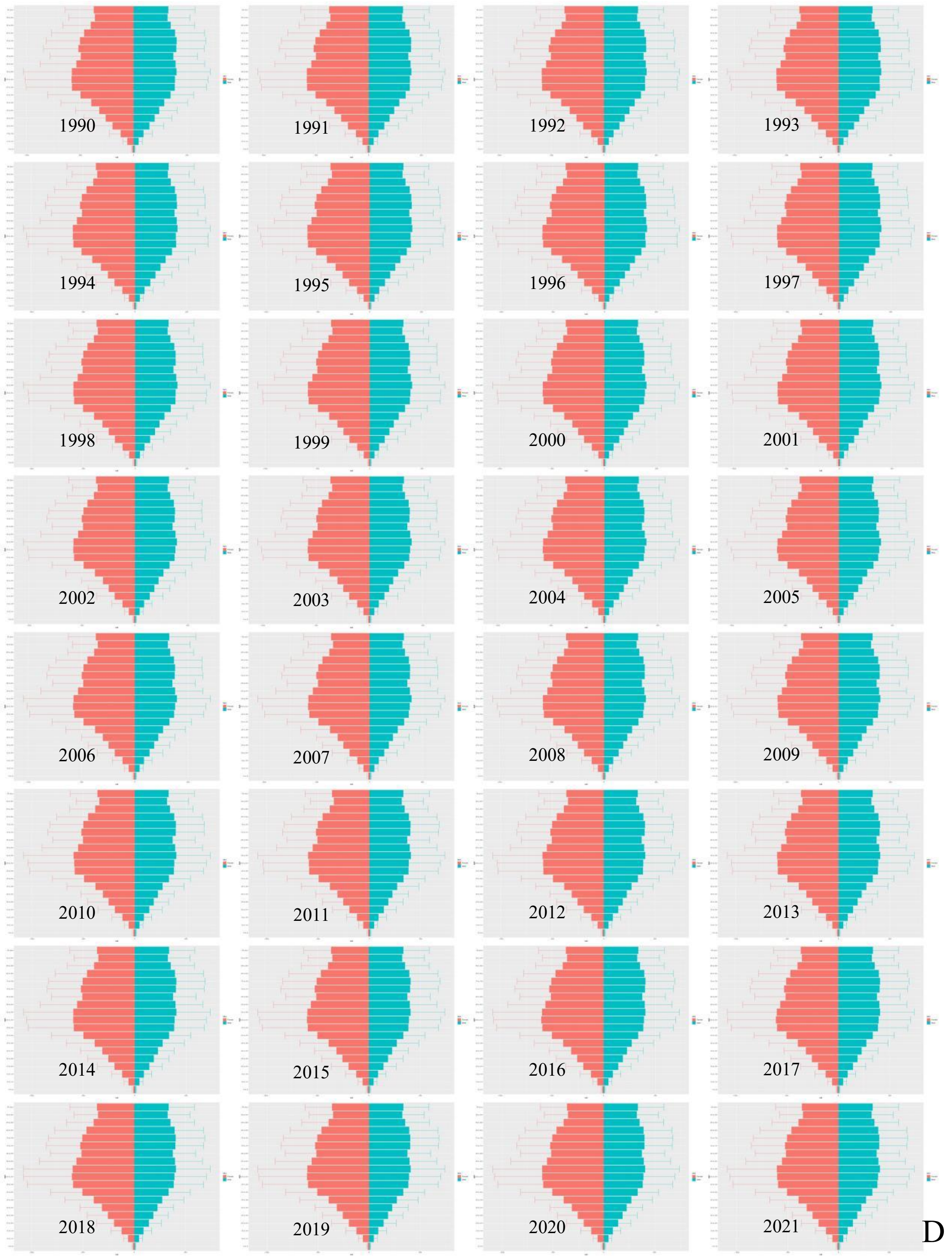

Fig. S37 (A) The prevalence cases of neck pain in different ages from 1990 to 2021 in the worldwide; (B) The prevalence rates of neck pain in different ages from 1990 to 2021 in the worldwide; (C) The years lived with disability of neck pain in different ages from 1990 to 2021 in the worldwide; (D) The years lived with disability rates of neck pain in different ages from 1990 to 2021 in the worldwide.

Notes: red for female, green for male; the ordinate from bottom to top is "5 to 9", "10 to 14", "15 to 19", "20 to 24", "25 to 29", "30 to 34", "35 to 39", "40 to 44", "45 to 49", "50 to 54", "55 to 59", "60 to 64", "65 to 69", "70 to 74", "75 to 79", "80 to 84", "85 to 89", "90 to 94", "95 plus".

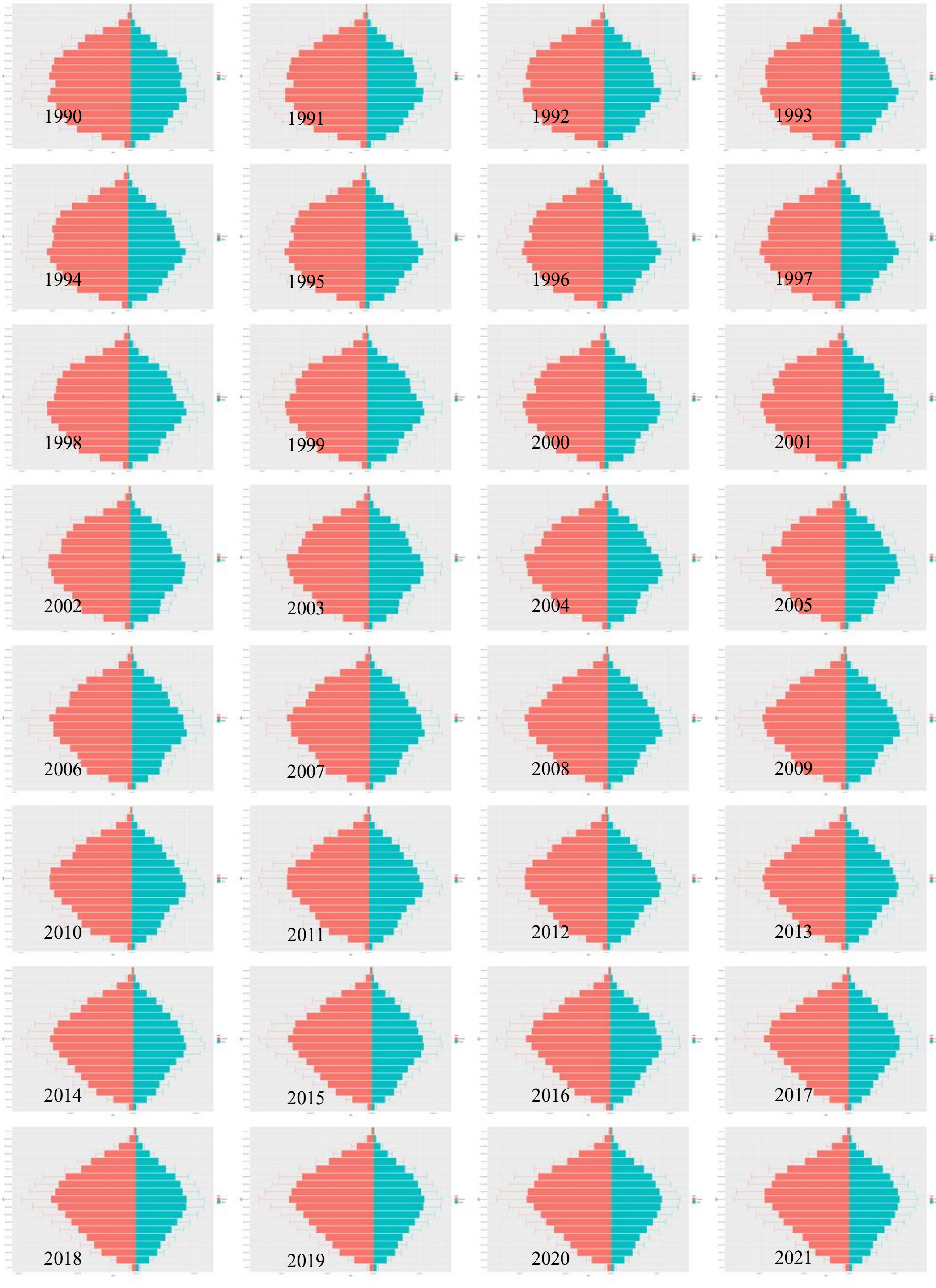
1990
1991
1992
1993
1994
1995
1996
1997
1998
1999
2000
2001
2002
2003
2004
2005
2006
2007
2008
2009
2010
2011
2012
2013
2014
2015
2016
2017
2018
2019
2020
2021

A

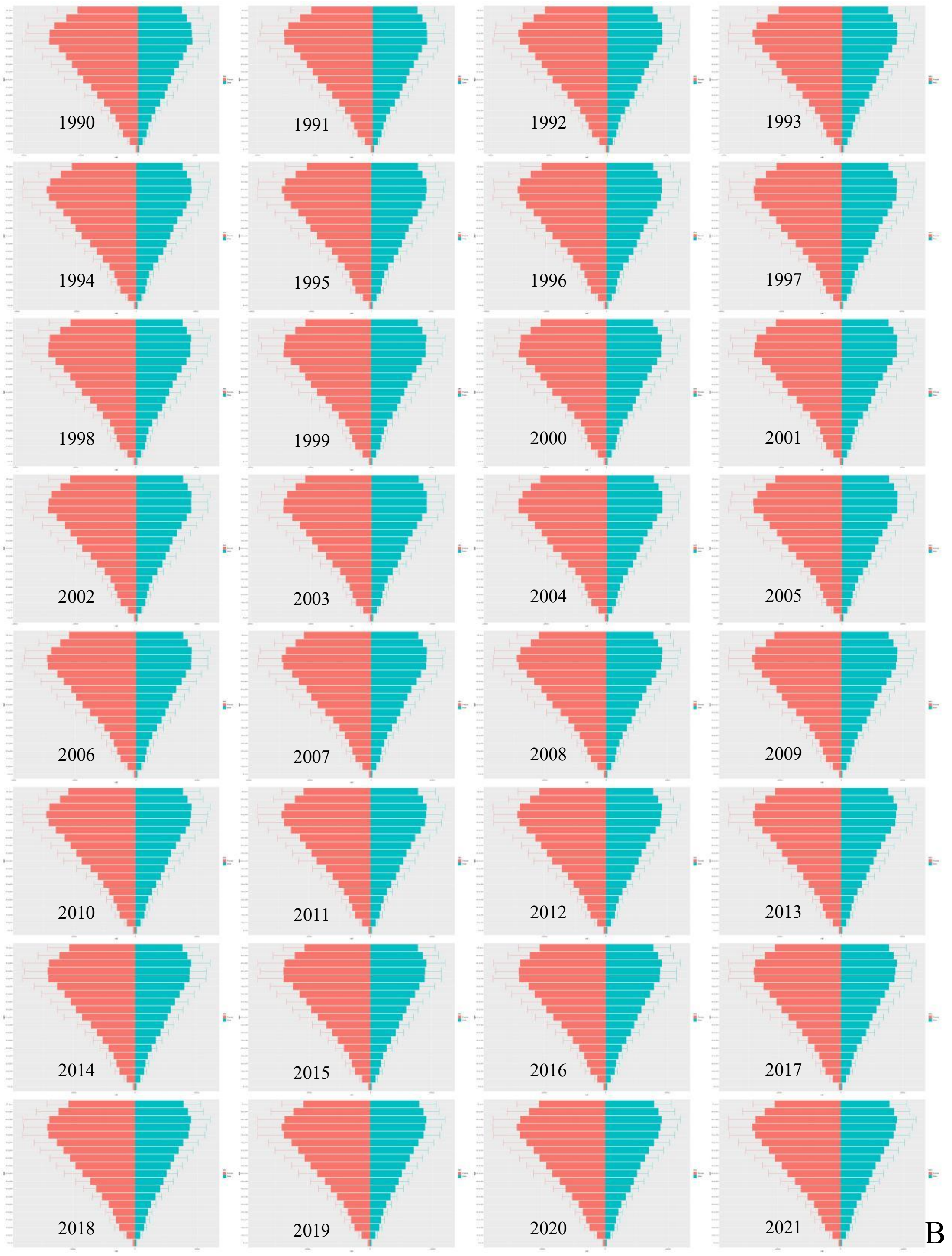
1990
1991
1992
1993
1994
1995
1996
1997
1998
1999
2000
2001
2002
2003
2004
2005
2006
2007
2008
2009
2010
2011
2012
2013
2014
2015
2016
2017
2018
2019
2020
2021
B

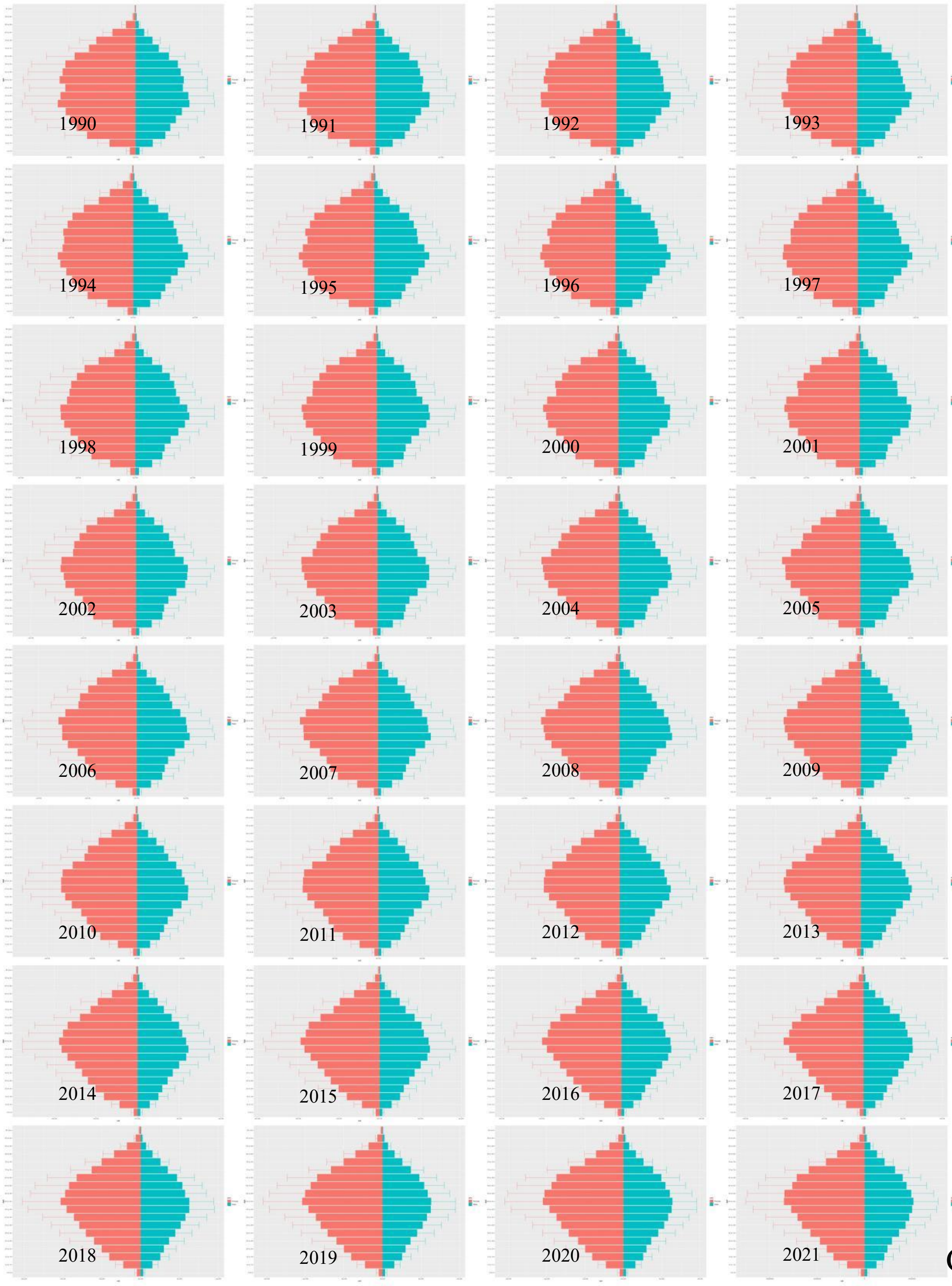
1990
1991
1992
1993
1994
1995
1996
1997
1998
1999
2000
2001
2002
2003
2004
2005
2006
2007
2008
2009
2010
2011
2012
2013
2014
2015
2016
2017
2018
2019
2020
2021

C

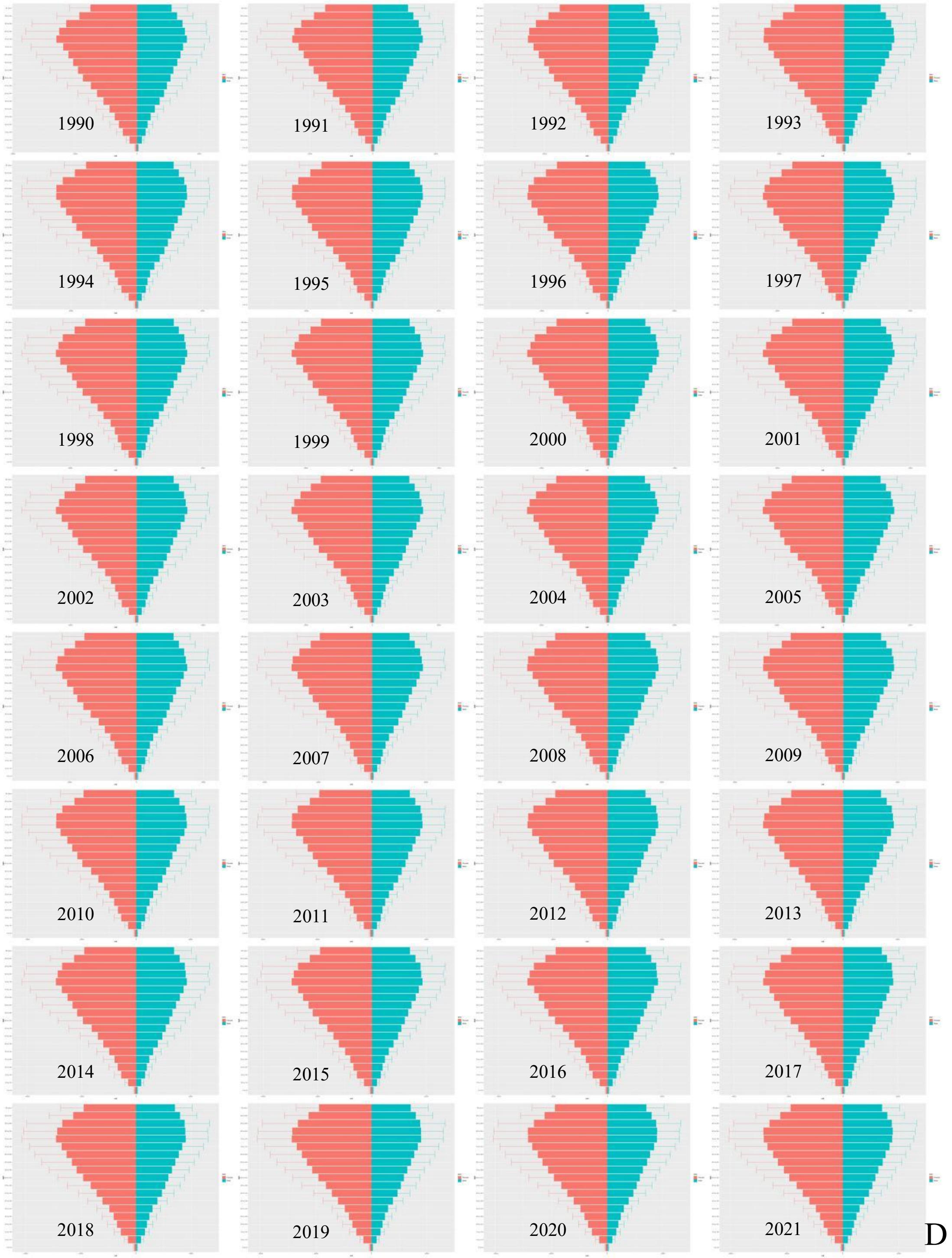

Fig. S38 (A) The prevalence cases of low back pain in different ages from 1990 to 2021 in the worldwide; (B) The prevalence rates of low back pain in different ages from 1990 to 2021 in the worldwide; (C) The years lived with disability of low back pain in different ages from 1990 to 2021 in the worldwide; (D) The years lived with disability rates of low back pain in different ages from 1990 to 2021 in the worldwide.

Notes: red for female, green for male; the ordinate from bottom to top is "5 to 9", "10 to 14", "15 to 19", "20 to 24", "25 to 29", "30 to 34", "35 to 39", "40 to 44", "45 to 49", "50 to 54", "55 to 59", "60 to 64", "65 to 69", "70 to 74", "75 to 79", "80 to 84", "85 to 89", "90 to 94", "95 plus".

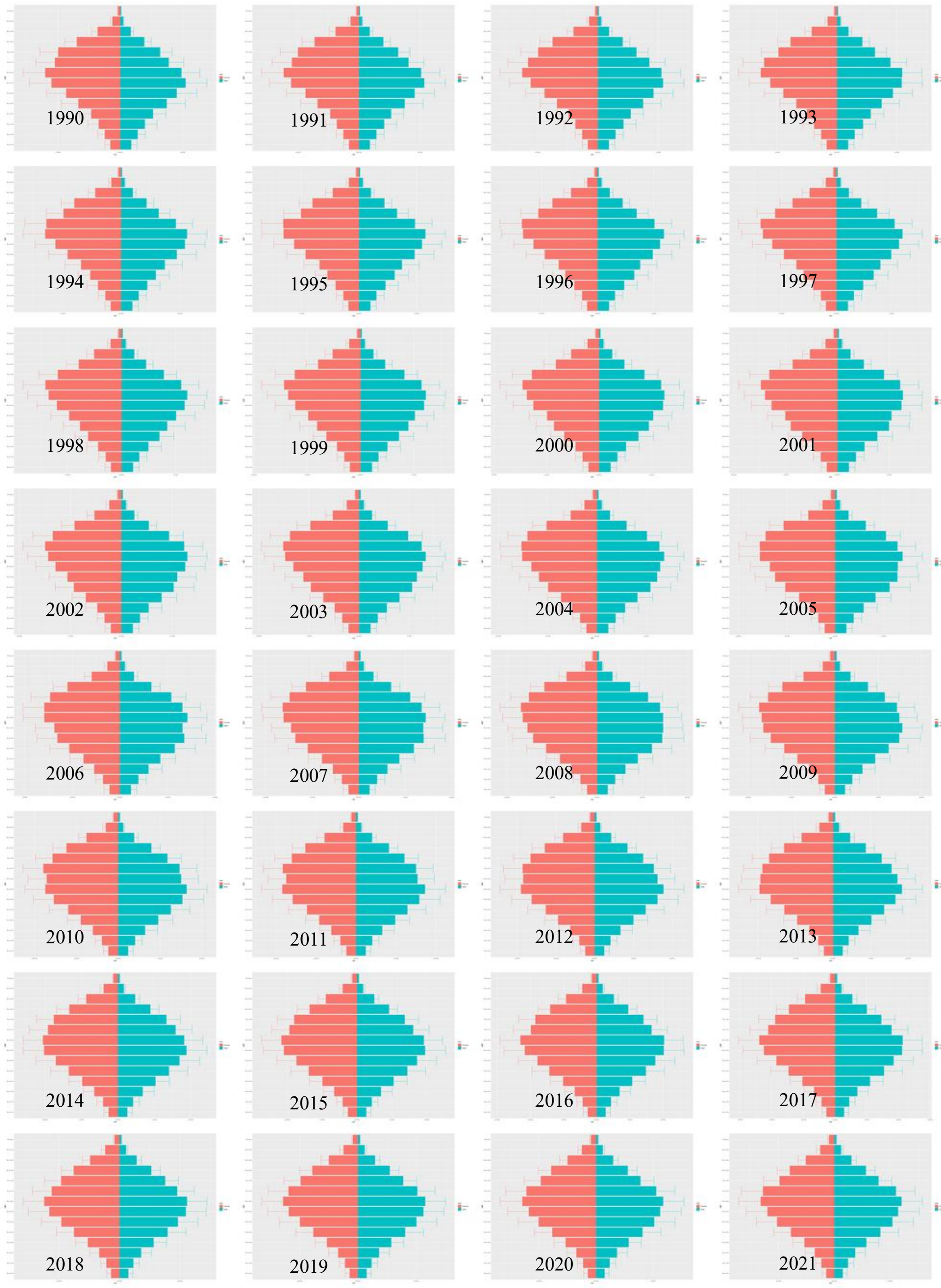
1990
1991
1992
1993
1994
1995
1996
1997
1998
1999
2000
2001
2002
2003
2004
2005
2006
2007
2008
2009
2010
2011
2012
2013
2014
2015
2016
2017
2018
2019
2020
2021

A

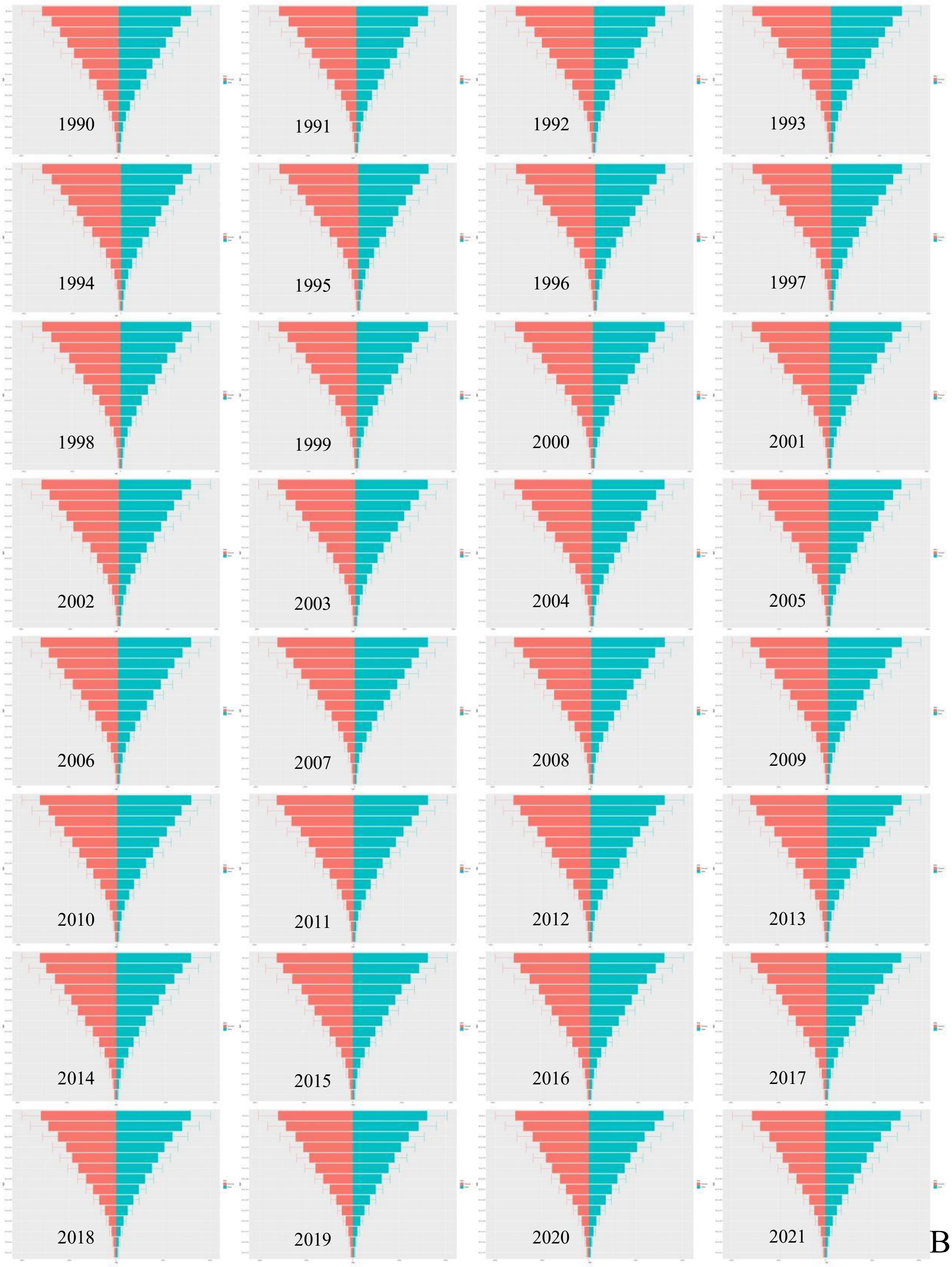
1990
1991
1992
1993
1994
1995
1996
1997
1998
1999
2000
2001
2002
2003
2004
2005
2006
2007
2008
2009
2010
2011
2012
2013
2014
2015
2016
2017
2018
2019
2020
2021

B

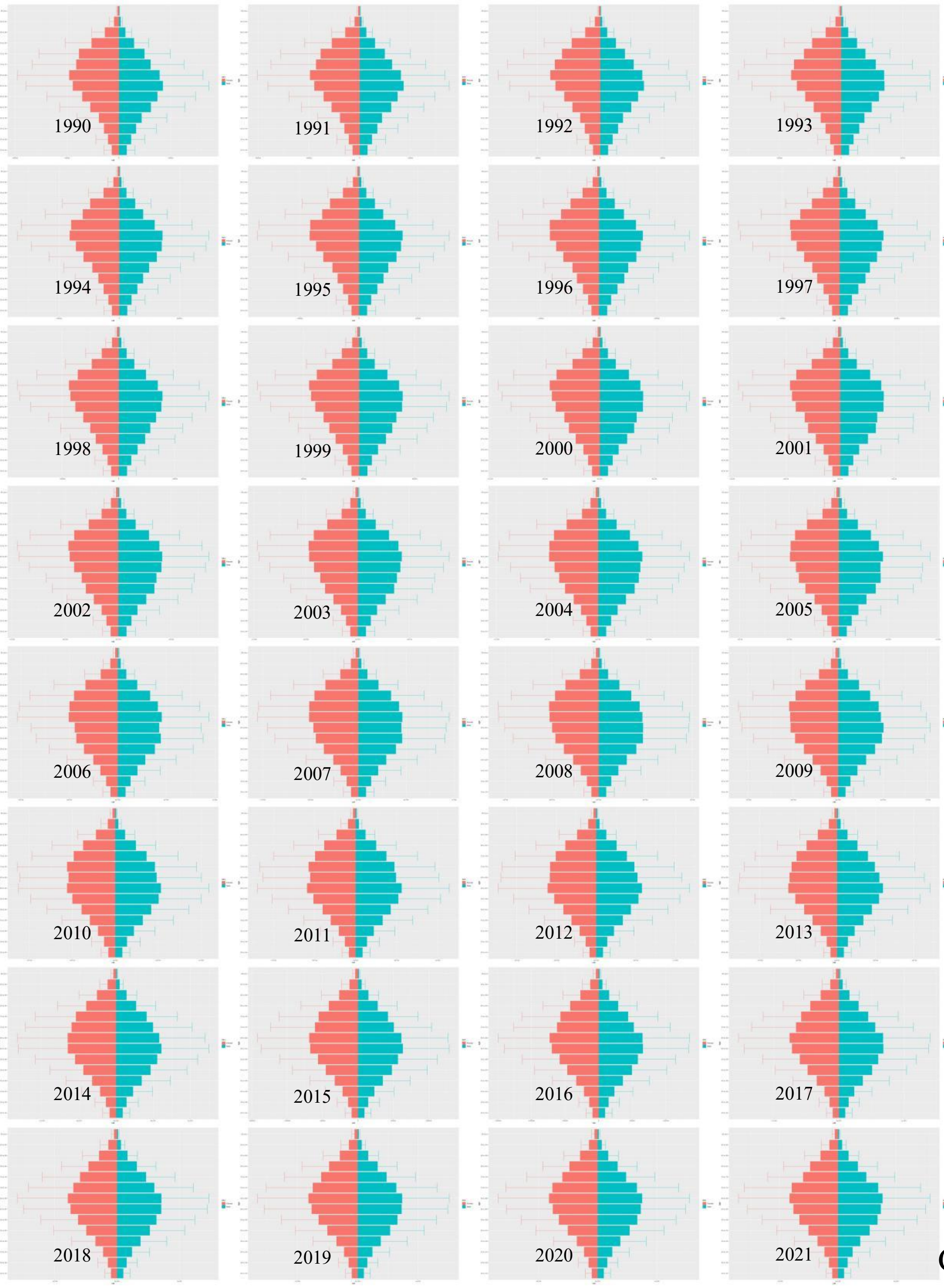
1990
1991
1992
1993
1994
1995
1996
1997
1998
1999
2000
2001
2002
2003
2004
2005
2006
2007
2008
2009
2010
2011
2012
2013
2014
2015
2016
2017
2018
2019
2020
2021

C

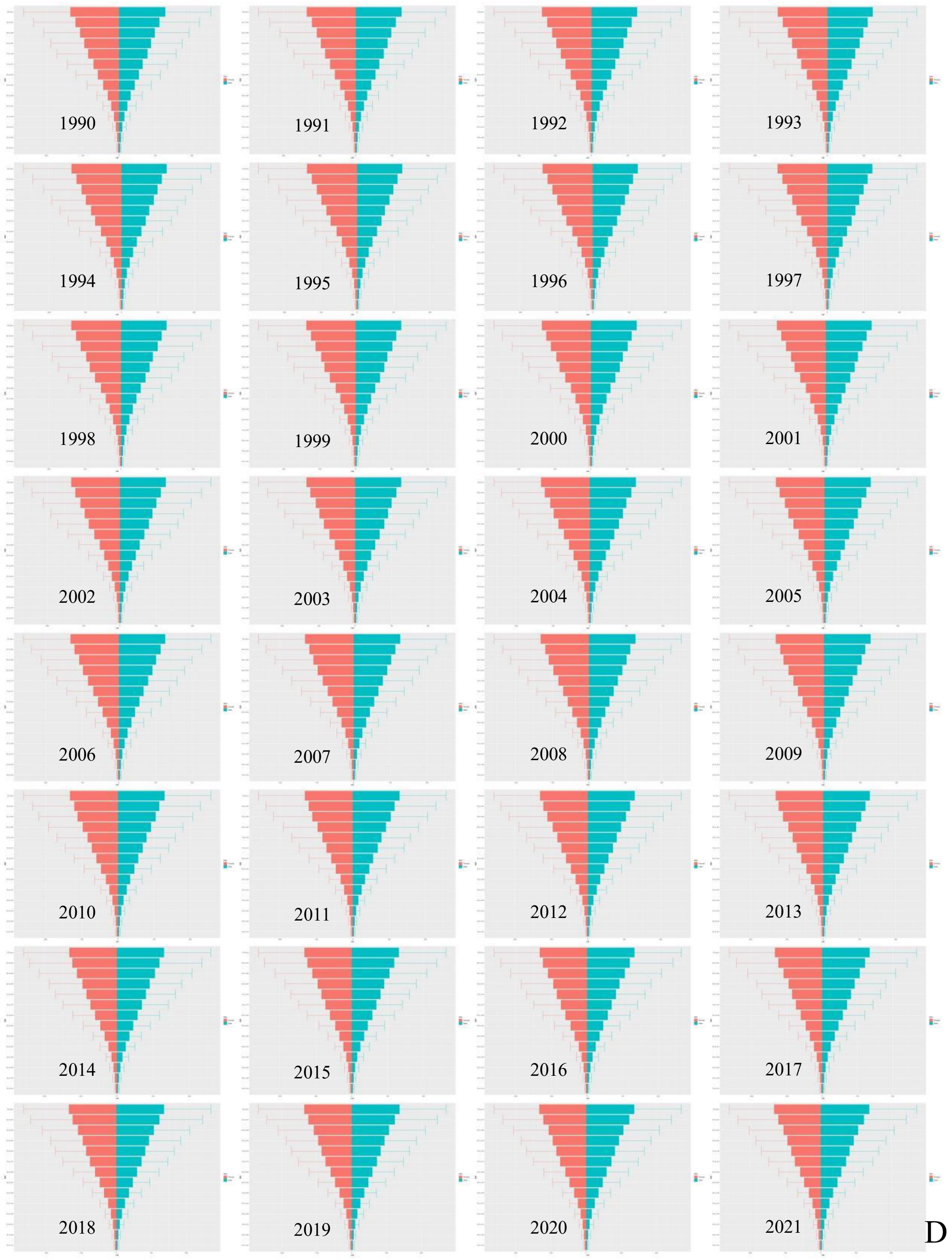

Fig. S39 (A) The prevalence cases of hip osteoarthritis in different ages from 1990 to 2021 in the worldwide; (B) The prevalence rates of hip osteoarthritis in different ages from 1990 to 2021 in the worldwide; (C) The years lived with disability of hip osteoarthritis in different ages from 1990 to 2021 in the worldwide; (D) The years lived with disability rates of hip osteoarthritis in different ages from 1990 to 2021 in the worldwide.

Notes: red for female, green for male; the ordinate from bottom to top is "30 to 34", "35 to 39", "40 to 44", "45 to 49", "50 to 54", "55 to 59", "60 to 64", "65 to 69", "70 to 74", "75 to 79", "80 to 84", "85 to 89", "90 to 94", "95 plus".

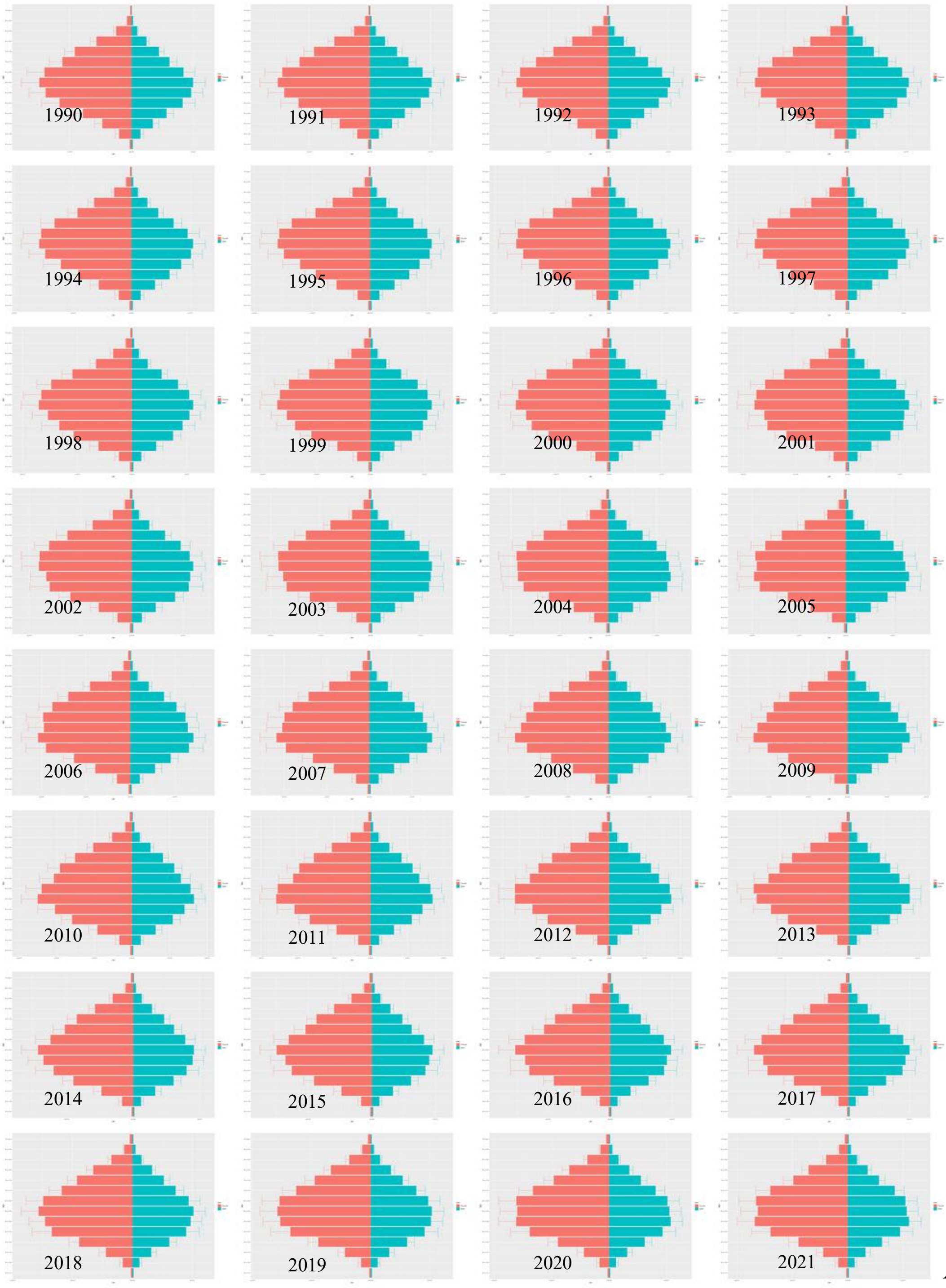
1990
1991
1992
1993
1994
1995
1996
1997
1998
1999
2000
2001
2002
2003
2004
2005
2006
2007
2008
2009
2010
2011
2012
2013
2014
2015
2016
2017
2018
2019
2020
2021

A

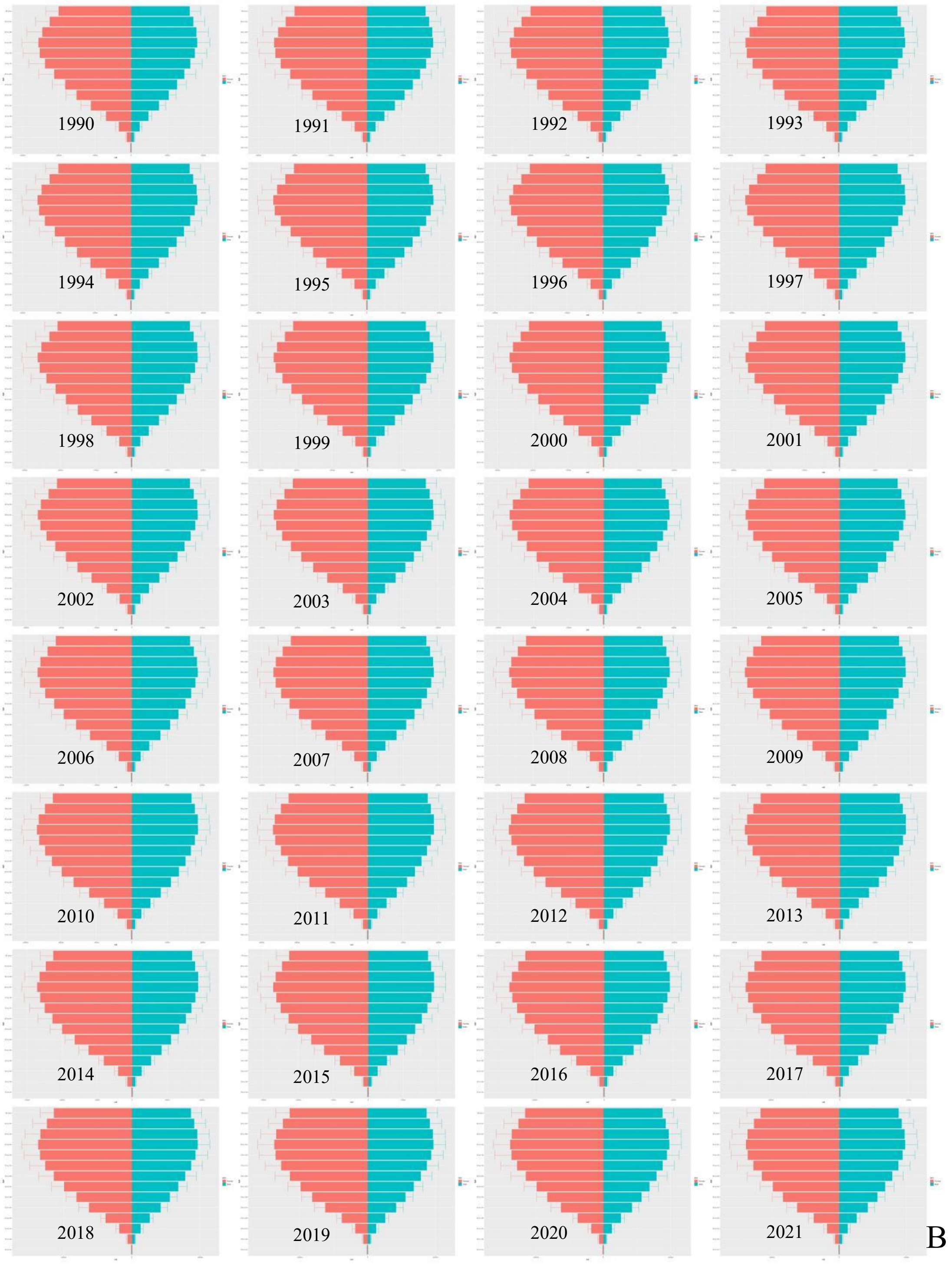
1990
1991
1992
1993
1994
1995
1996
1997
1998
1999
2000
2001
2002
2003
2004
2005
2006
2007
2008
2009
2010
2011
2012
2013
2014
2015
2016
2017
2018
2019
2020
2021
B

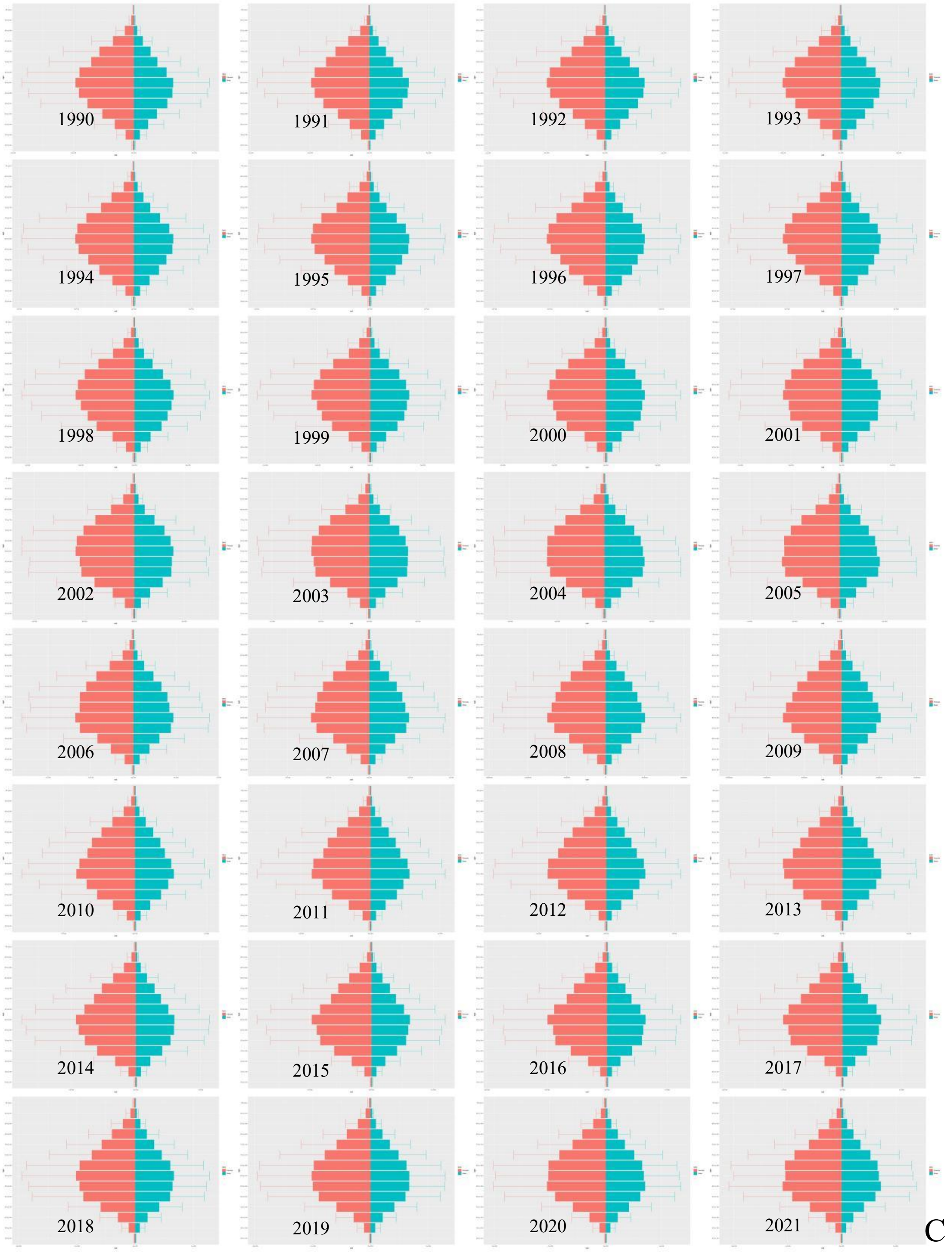
1990
1991
1992
1993
1994
1995
1996
1997
1998
1999
2000
2001
2002
2003
2004
2005
2006
2007
2008
2009
2010
2011
2012
2013
2014
2015
2016
2017
2018
2019
2020
2021
C

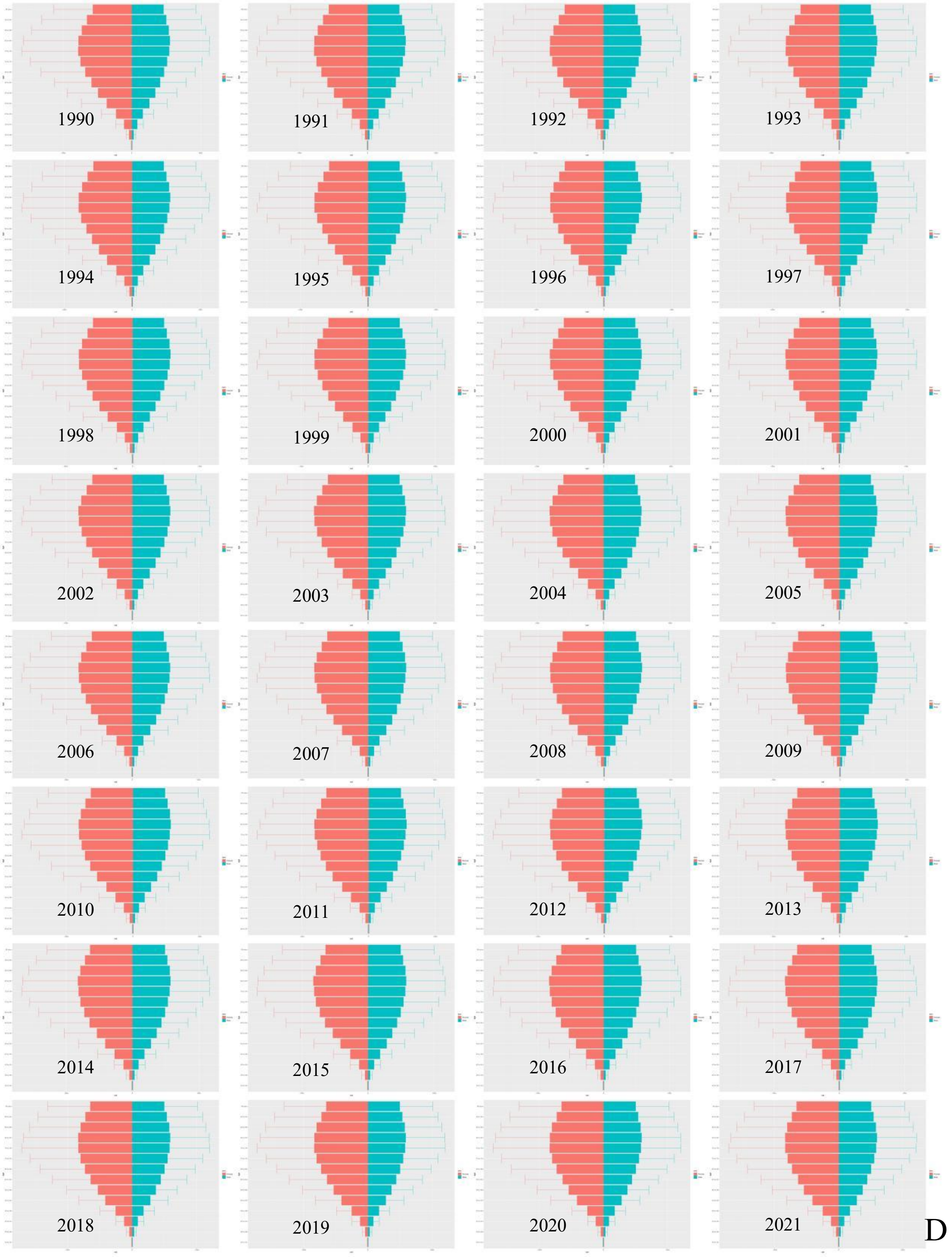

Fig. S40 (A) The prevalence cases of knee osteoarthritis in different ages from 1990 to 2021 in the worldwide; (B) The prevalence rates of knee osteoarthritis in different ages from 1990 to 2021 in the worldwide; (C) The years lived with disability of knee osteoarthritis in different ages from 1990 to 2021 in the worldwide; (D) The years lived with disability rates of knee osteoarthritis in different ages from 1990 to 2021 in the worldwide.

Notes: red for female, green for male; the ordinate from bottom to top is "35 to 39", "40 to 44", "45 to 49", "50 to 54", "55 to 59", "60 to 64", "65 to 69", "70 to 74", "75 to 79", "80 to 84", "85 to 89", "90 to 94", "95 plus".

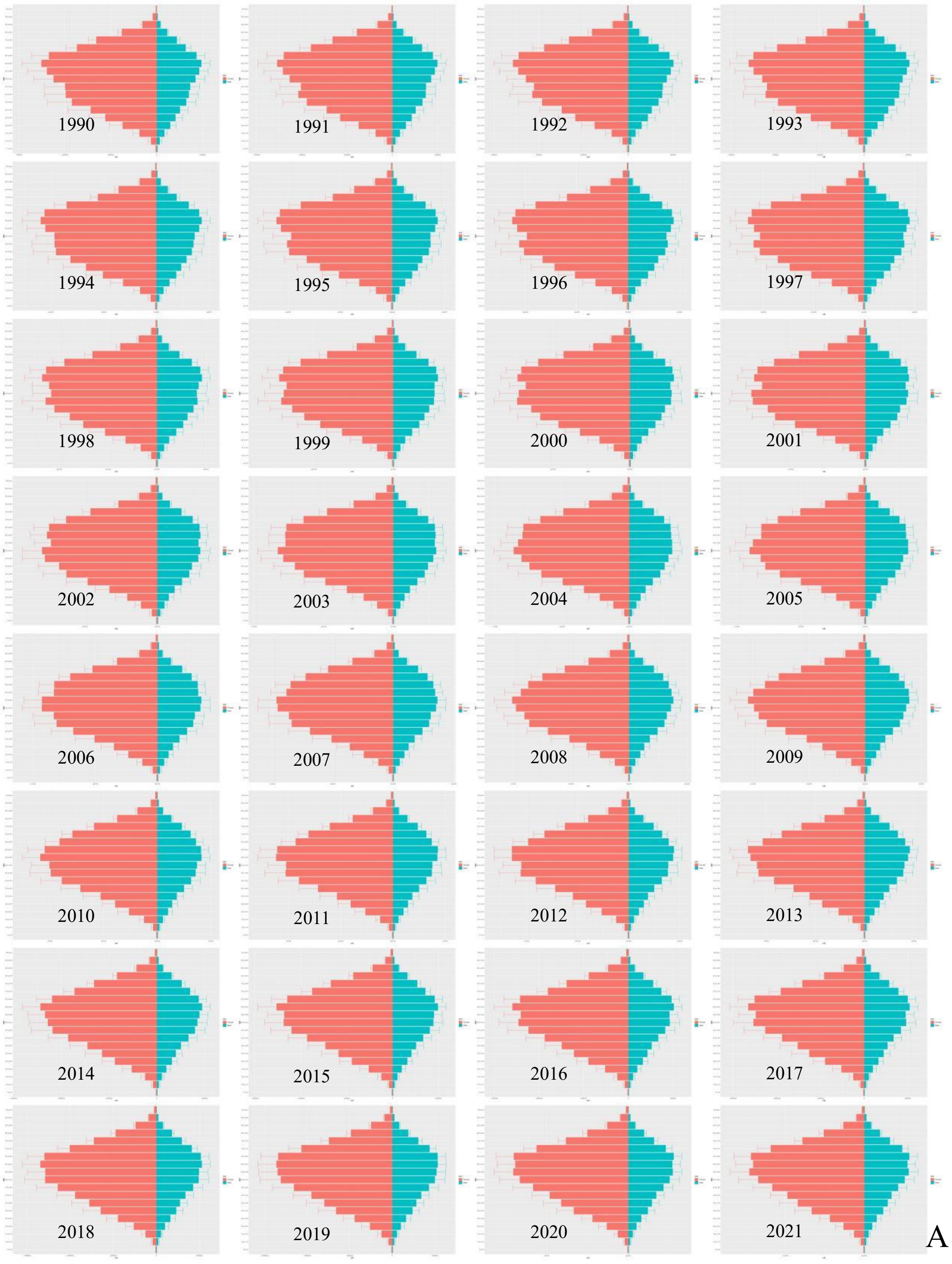
1990
1991
1992
1993
1994
1995
1996
1997
1998
1999
2000
2001
2002
2003
2004
2005
2006
2007
2008
2009
2010
2011
2012
2013
2014
2015
2016
2017
2018
2019
2020
2021

A

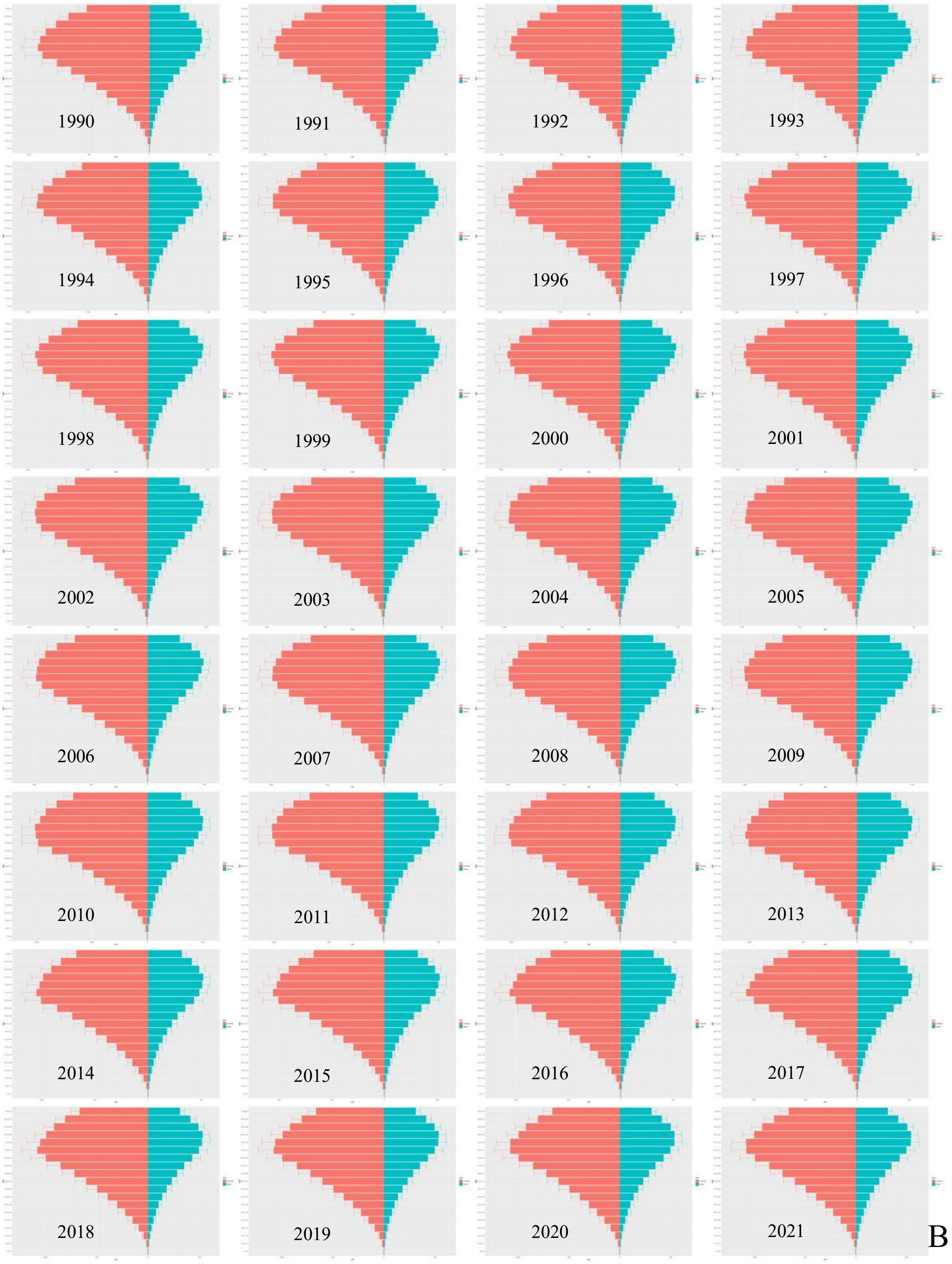
1990
1991
1992
1993
1994
1995
1996
1997
1998
1999
2000
2001
2002
2003
2004
2005
2006
2007
2008
2009
2010
2011
2012
2013
2014
2015
2016
2017
2018
2019
2020
2021
B

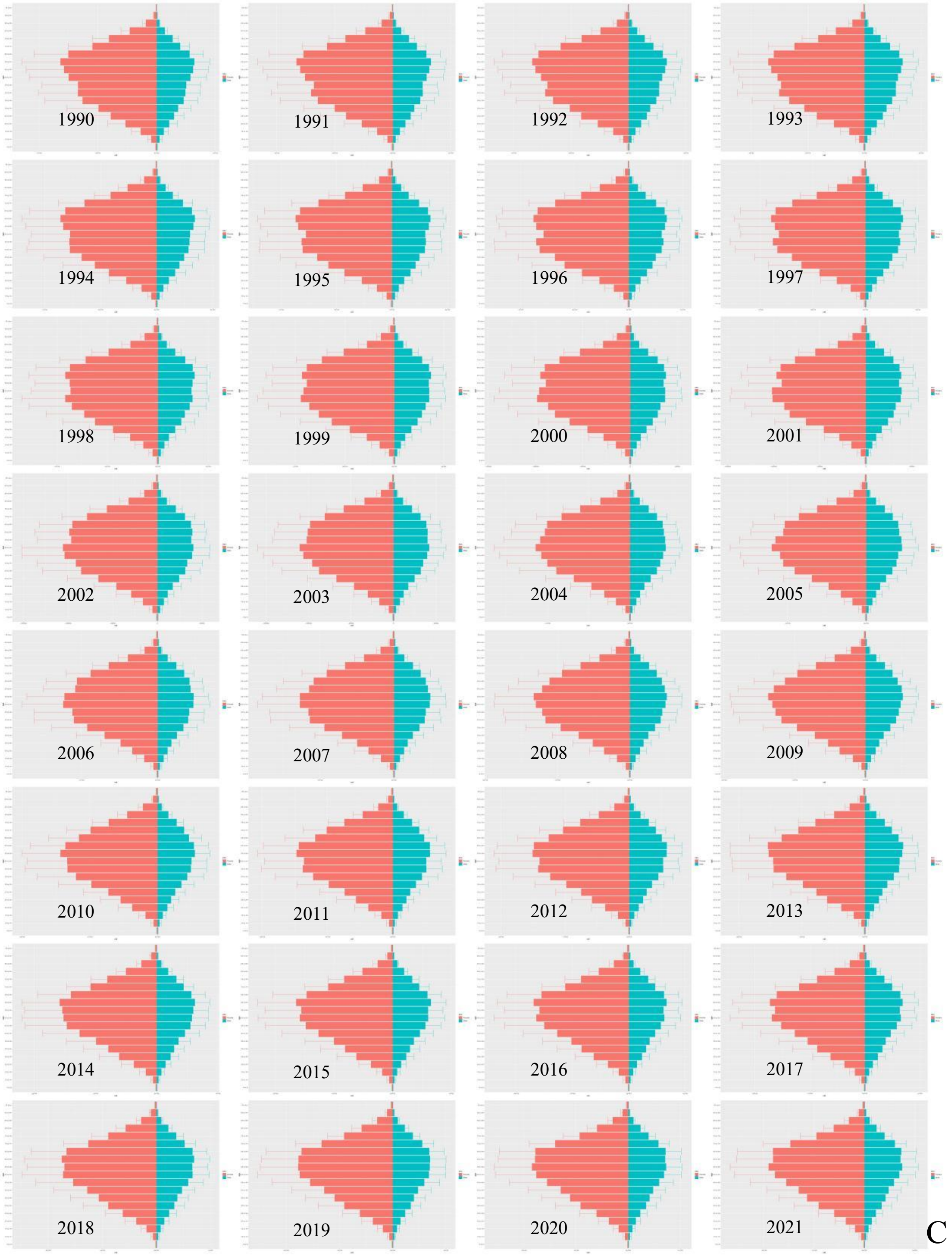
1990
1991
1992
1993
1994
1995
1996
1997
1998
1999
2000
2001
2002
2003
2004
2005
2006
2007
2008
2009
2010
2011
2012
2013
2014
2015
2016
2017
2018
2019
2020
2021

C

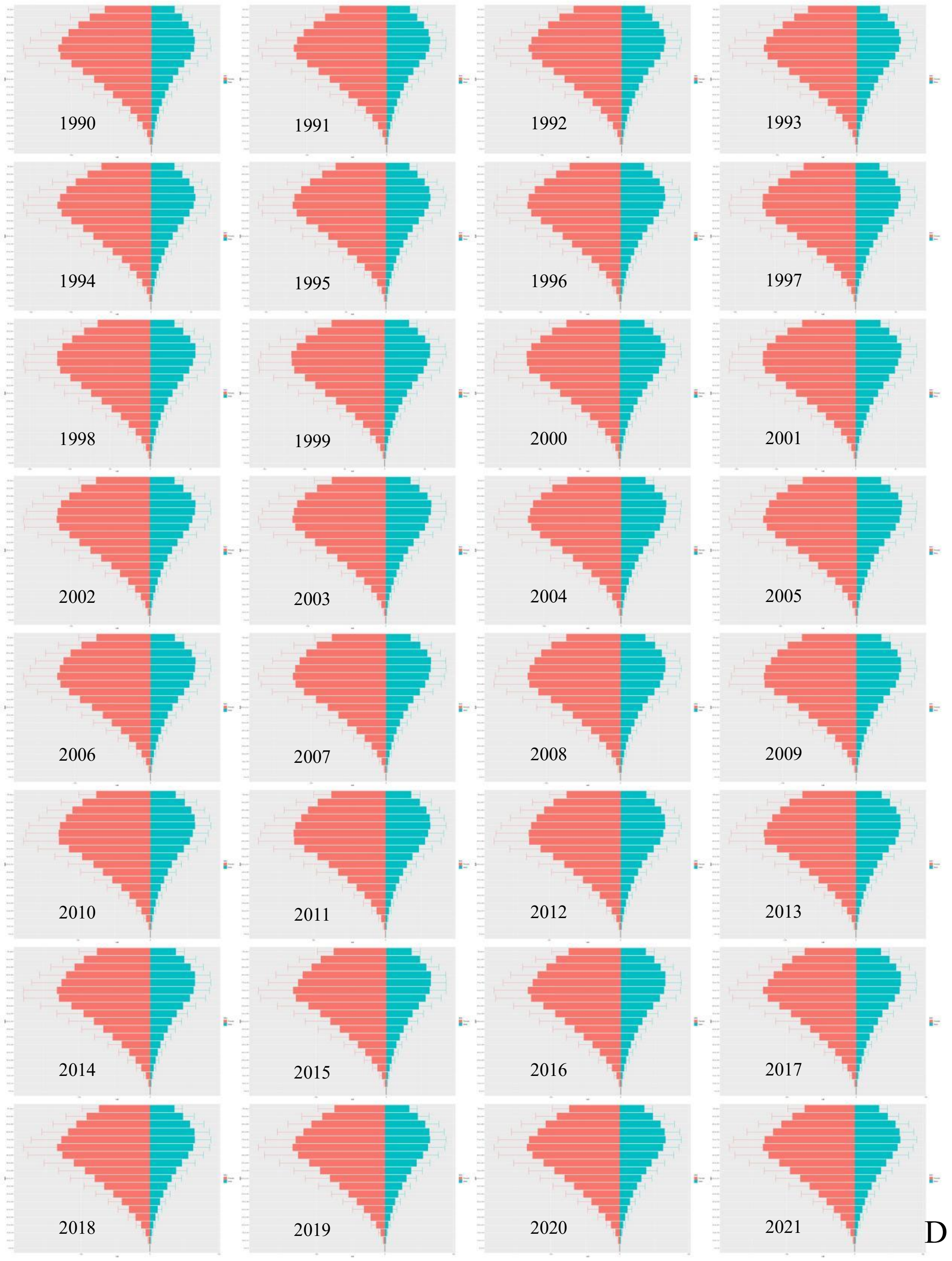

Fig. S41 (A) The prevalence cases of rheumatoid arthritis in different ages from 1990 to 2021 in the worldwide; (B) The prevalence rates of rheumatoid arthritis in different ages from 1990 to 2021 in the worldwide; (C) The years lived with disability of rheumatoid arthritis in different ages from 1990 to 2021 in the worldwide; (D) The years lived with disability rates of rheumatoid arthritis in different ages from 1990 to 2021 in the worldwide.

Notes: red for female, green for male; the ordinate from bottom to top is "5 to 9", "10 to 14", "15 to 19", "20 to 24", "25 to 29", "30 to 34", "35 to 39", "40 to 44", "45 to 49", "50 to 54", "55 to 59", "60 to 64", "65 to 69", "70 to 74", "75 to 79", "80 to 84", "85 to 89", "90 to 94", "95 plus".

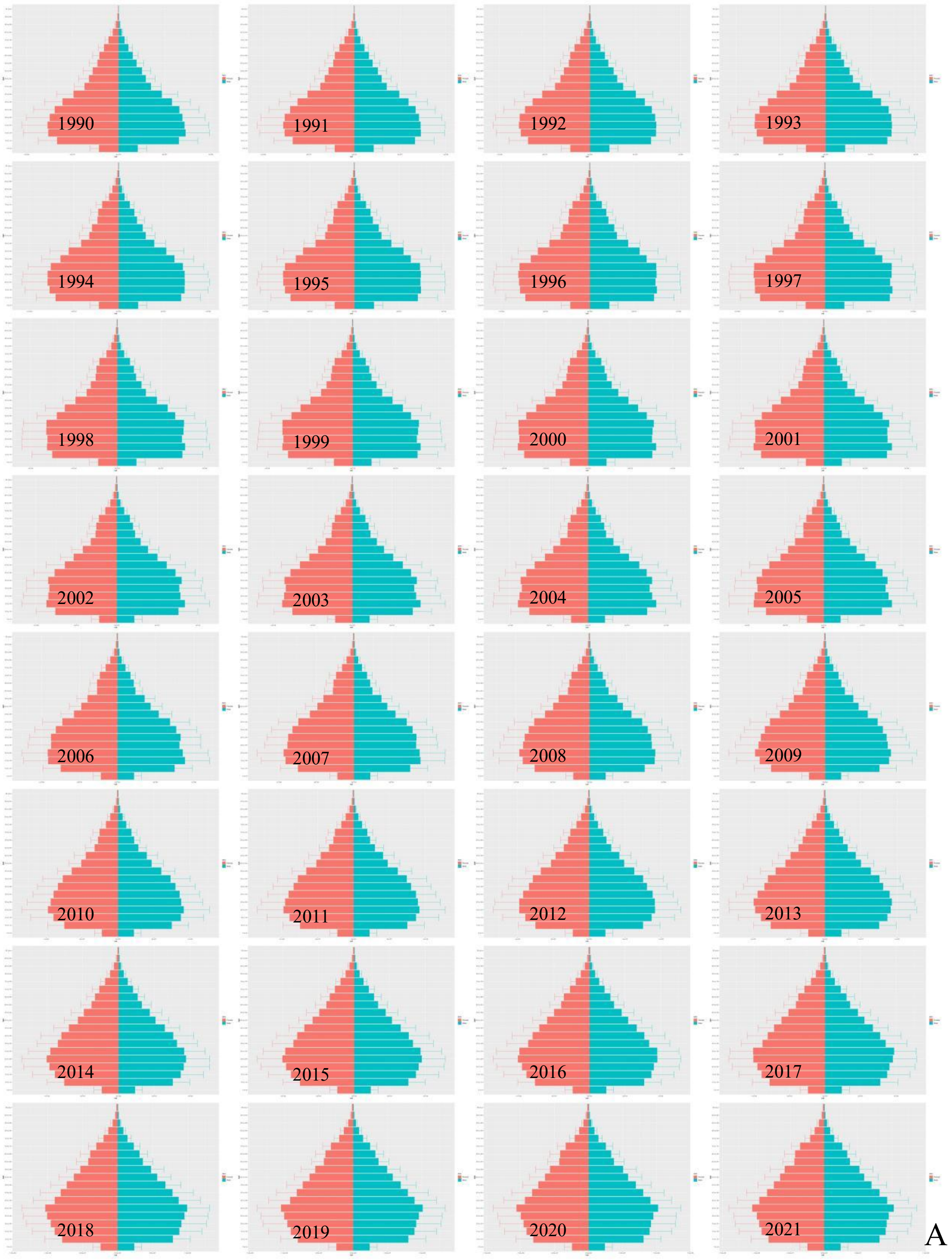
1990
1991
1992
1993
1994
1995
1996
1997
1998
1999
2000
2001
2002
2003
2004
2005
2006
2007
2008
2009
2010
2011
2012
2013
2014
2015
2016
2017
2018
2019
2020
2021
A

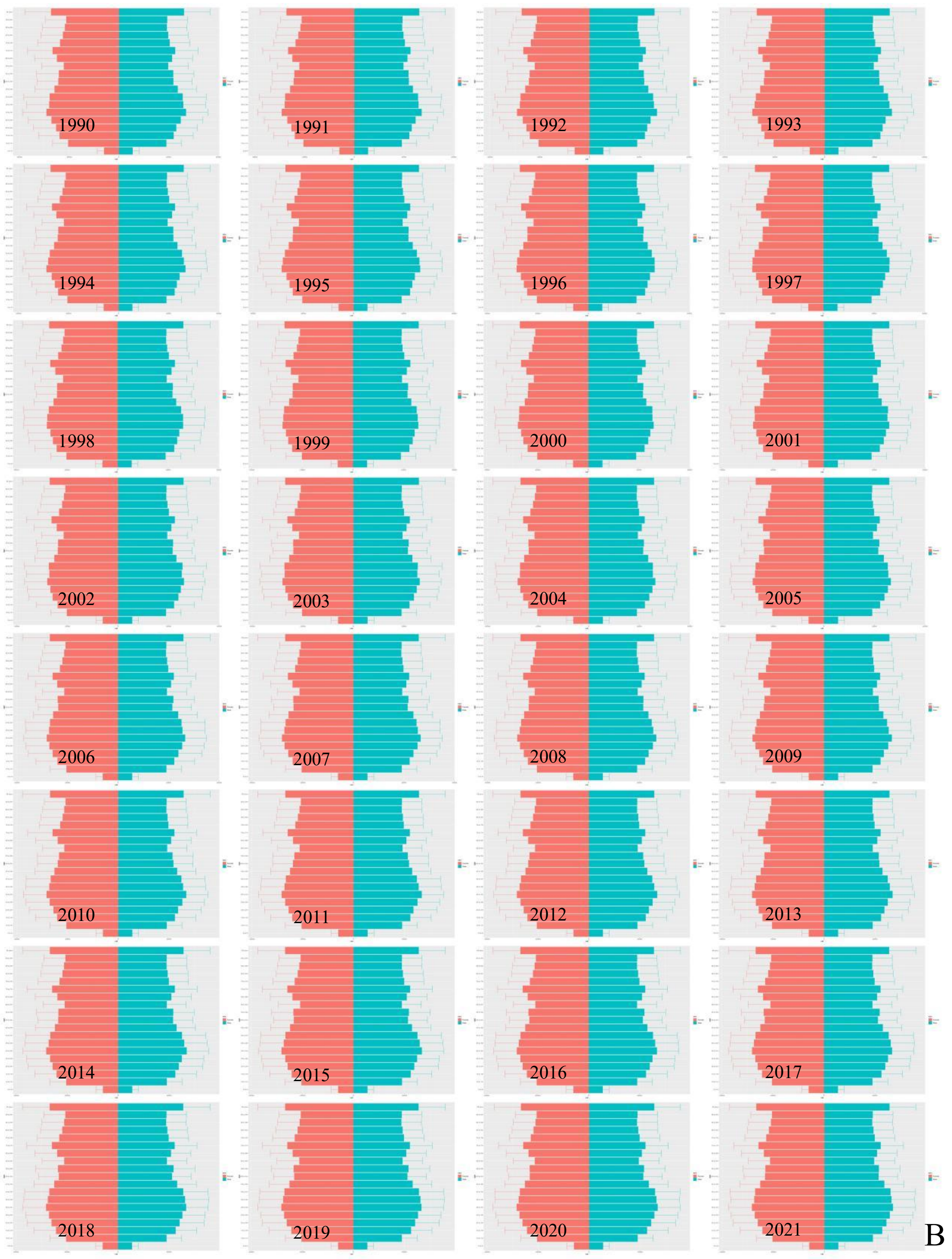
1990
1991
1992
1993
1994
1995
1996
1997
1998
1999
2000
2001
2002
2003
2004
2005
2006
2007
2008
2009
2010
2011
2012
2013
2014
2015
2016
2017
2018
2019
2020
2021
B

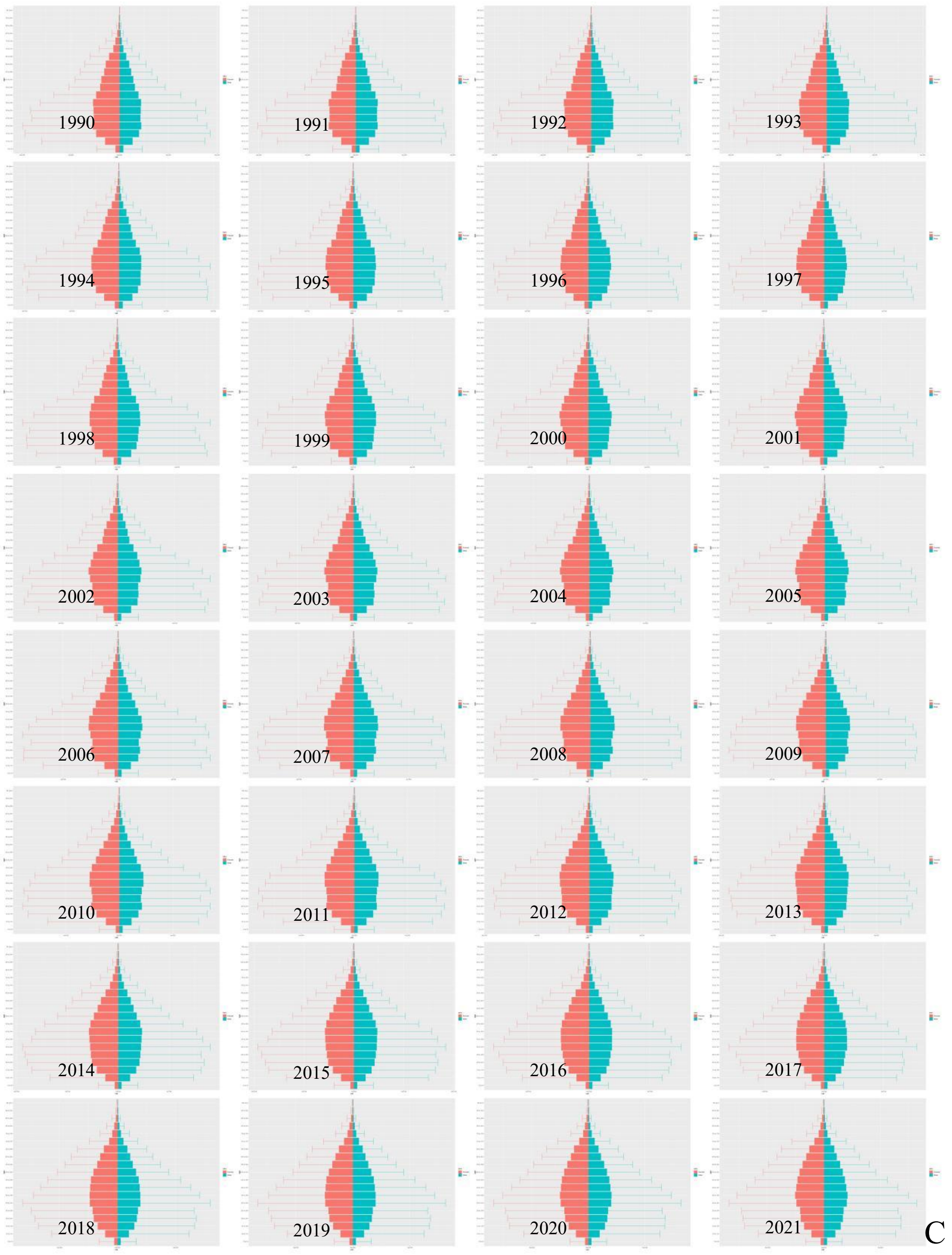
1990
1991
1992
1993
1994
1995
1996
1997
1998
1999
2000
2001
2002
2003
2004
2005
2006
2007
2008
2009
2010
2011
2012
2013
2014
2015
2016
2017
2018
2019
2020
2021
C

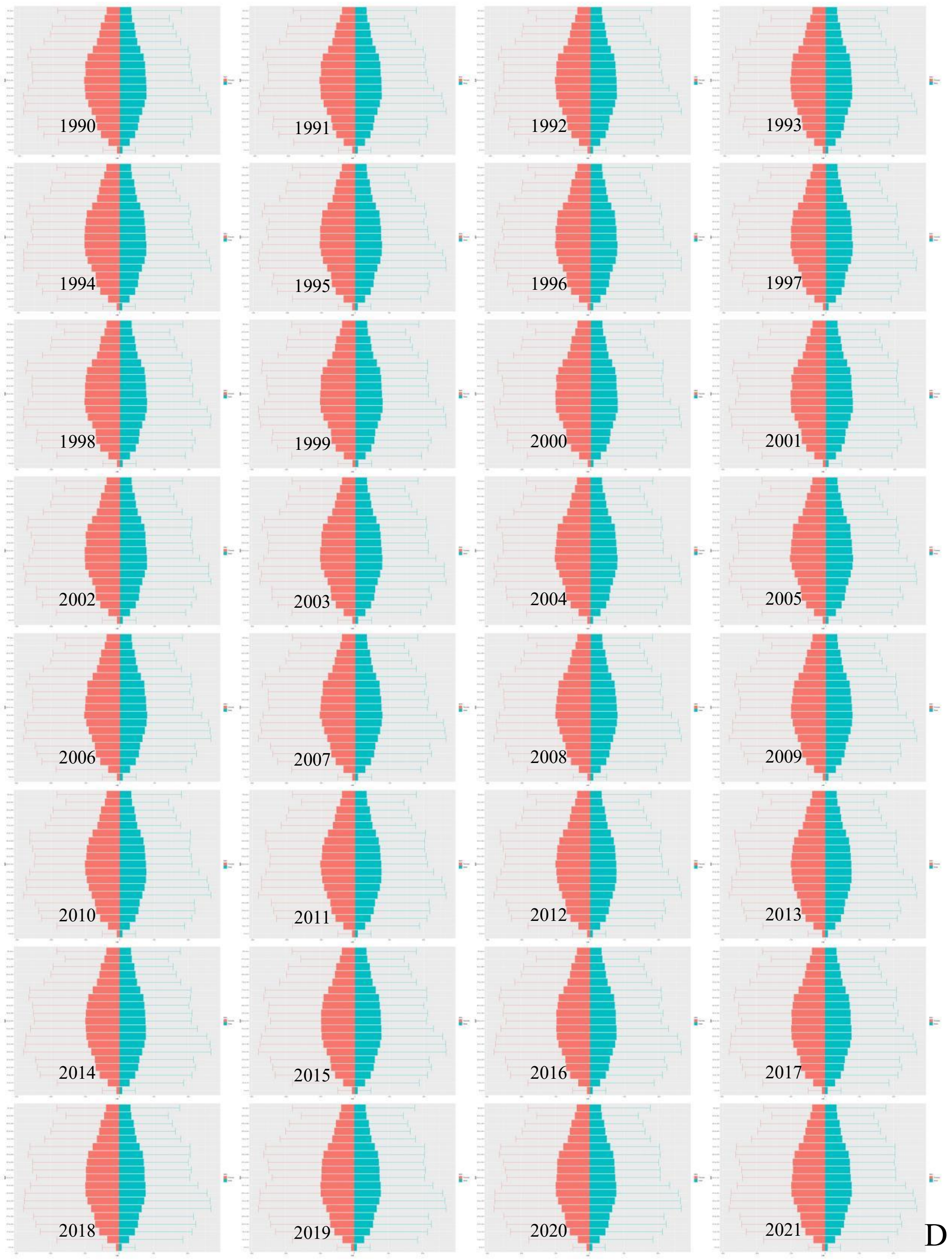

Fig. S42 (A) The prevalence cases of tension-type headache in different ages from 1990 to 2021 in the worldwide; (B) The prevalence rates of tension-type headache in different ages from 1990 to 2021 in the worldwide; (C) The years lived with disability of tension-type headache in different ages from 1990 to 2021 in the worldwide; (D) The years lived with disability rates of tension-type headache in different ages from 1990 to 2021 in the worldwide.

Notes: red for female, green for male; the ordinate from bottom to top is "5 to 9", "10 to 14", "15 to 19", "20 to 24", "25 to 29", "30 to 34", "35 to 39", "40 to 44", "45 to 49", "50 to 54", "55 to 59", "60 to 64", "65 to 69", "70 to 74", "75 to 79", "80 to 84", "85 to 89", "90 to 94", "95 plus".

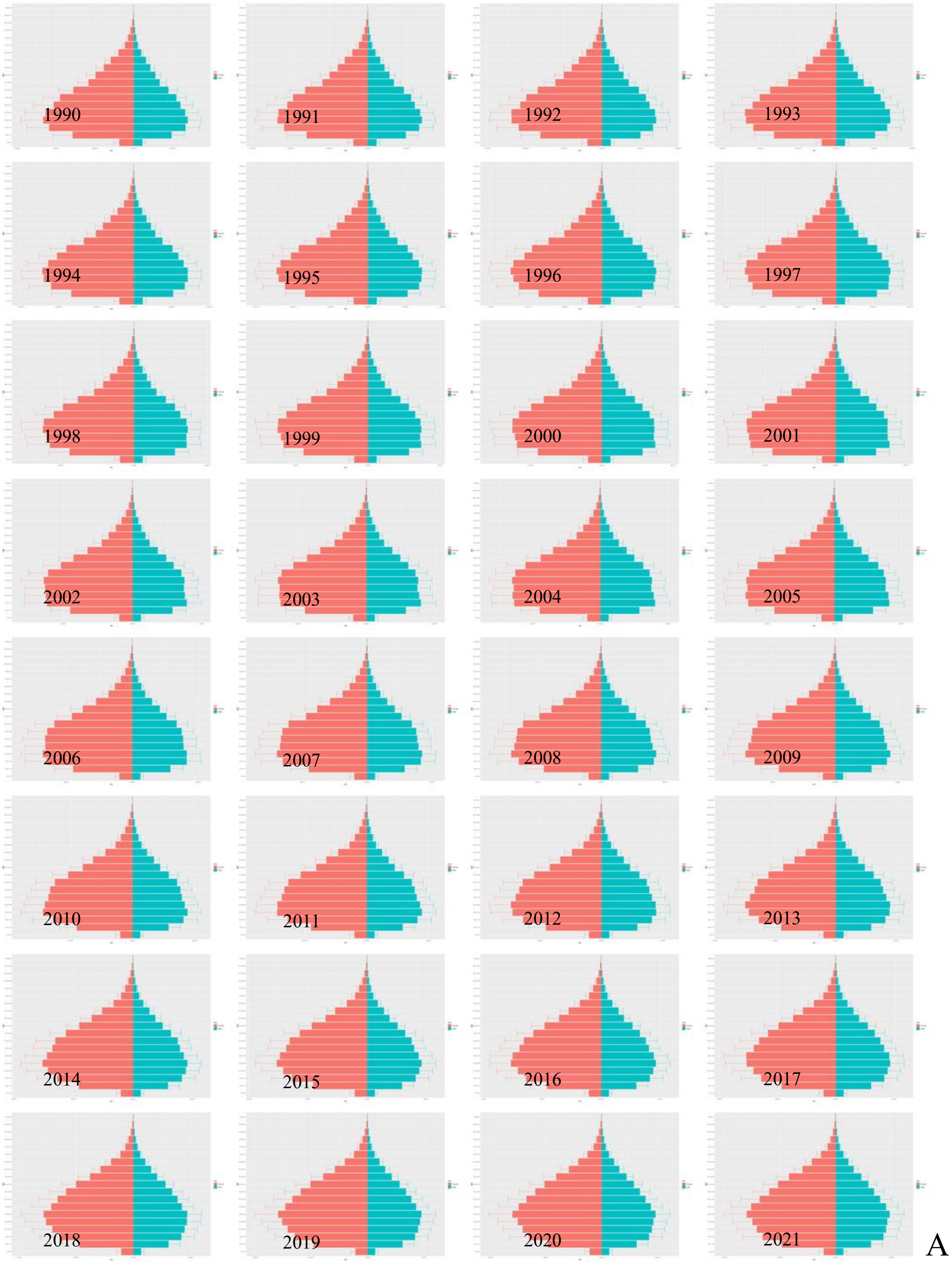
1990
1991
1992
1993
1994
1995
1996
1997
1998
1999
2000
2001
2002
2003
2004
2005
2006
2007
2008
2009
2010
2011
2012
2013
2014
2015
2016
2017
2018
2019
2020
2021
A

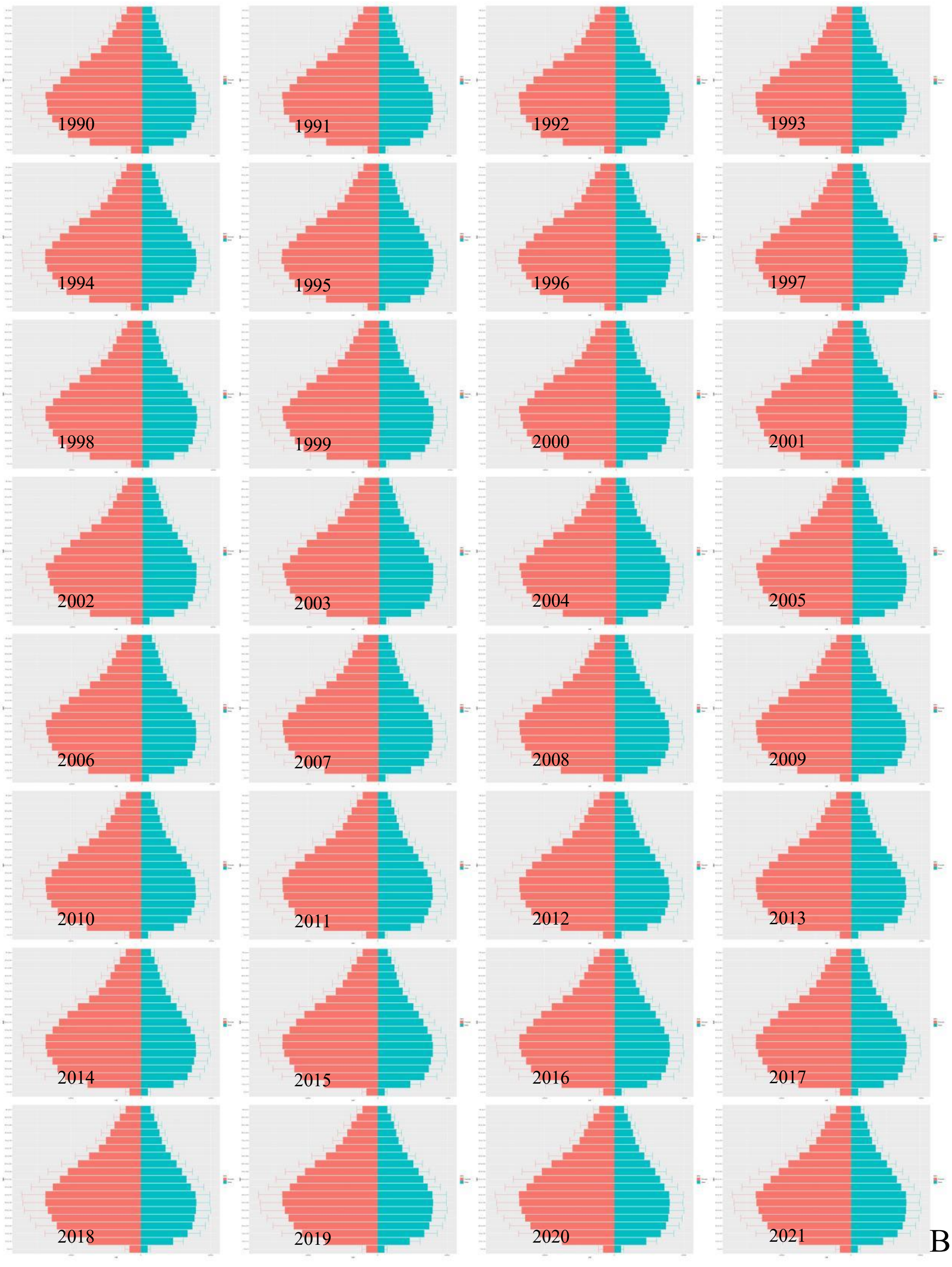
1990
1991
1992
1993
1994
1995
1996
1997
1998
1999
2000
2001
2002
2003
2004
2005
2006
2007
2008
2009
2010
2011
2012
2013
2014
2015
2016
2017
2018
2019
2020
2021

B

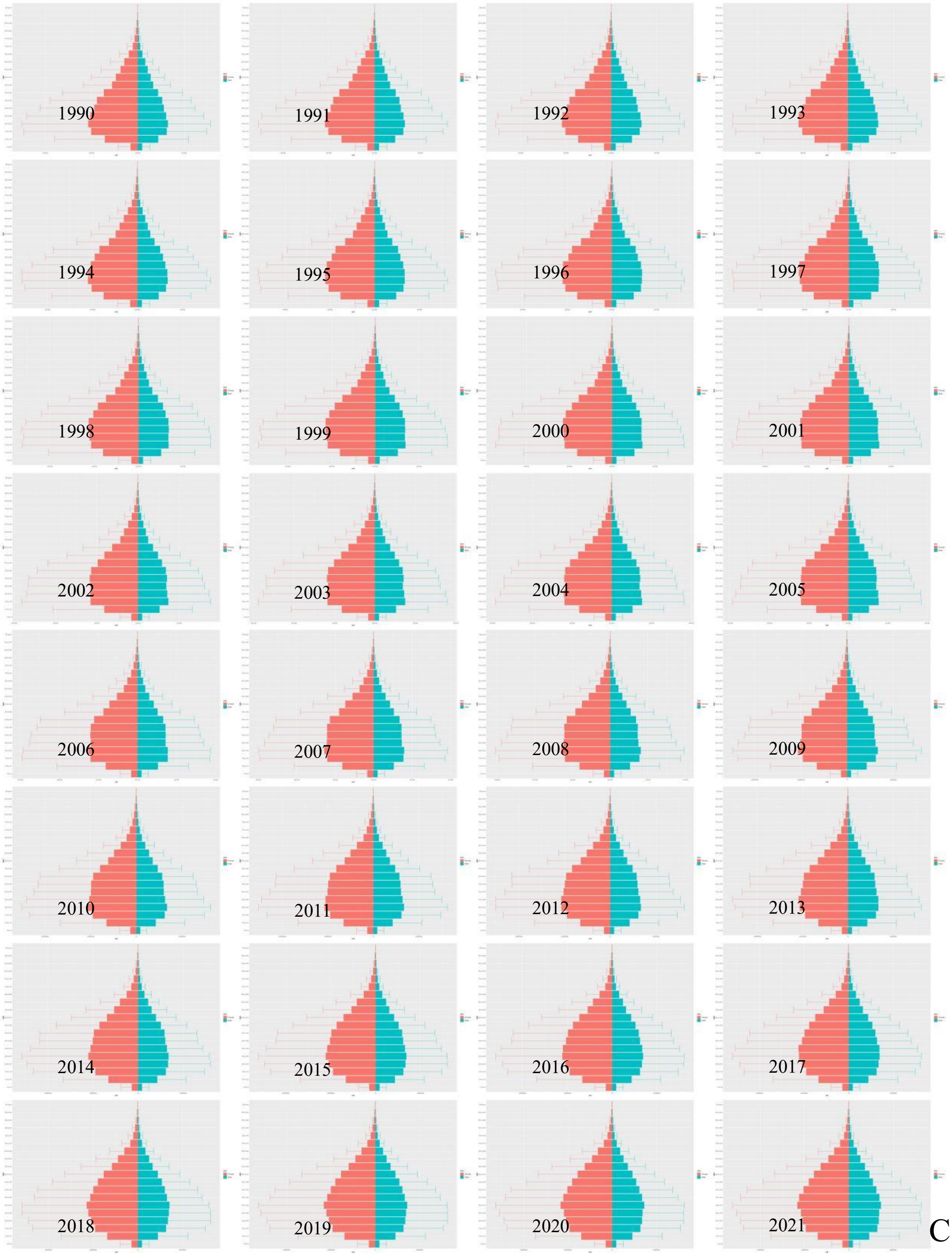
1990
1991
1992
1993
1994
1995
1996
1997
1998
1999
2000
2001
2002
2003
2004
2005
2006
2007
2008
2009
2010
2011
2012
2013
2014
2015
2016
2017
2018
2019
2020
2021
C

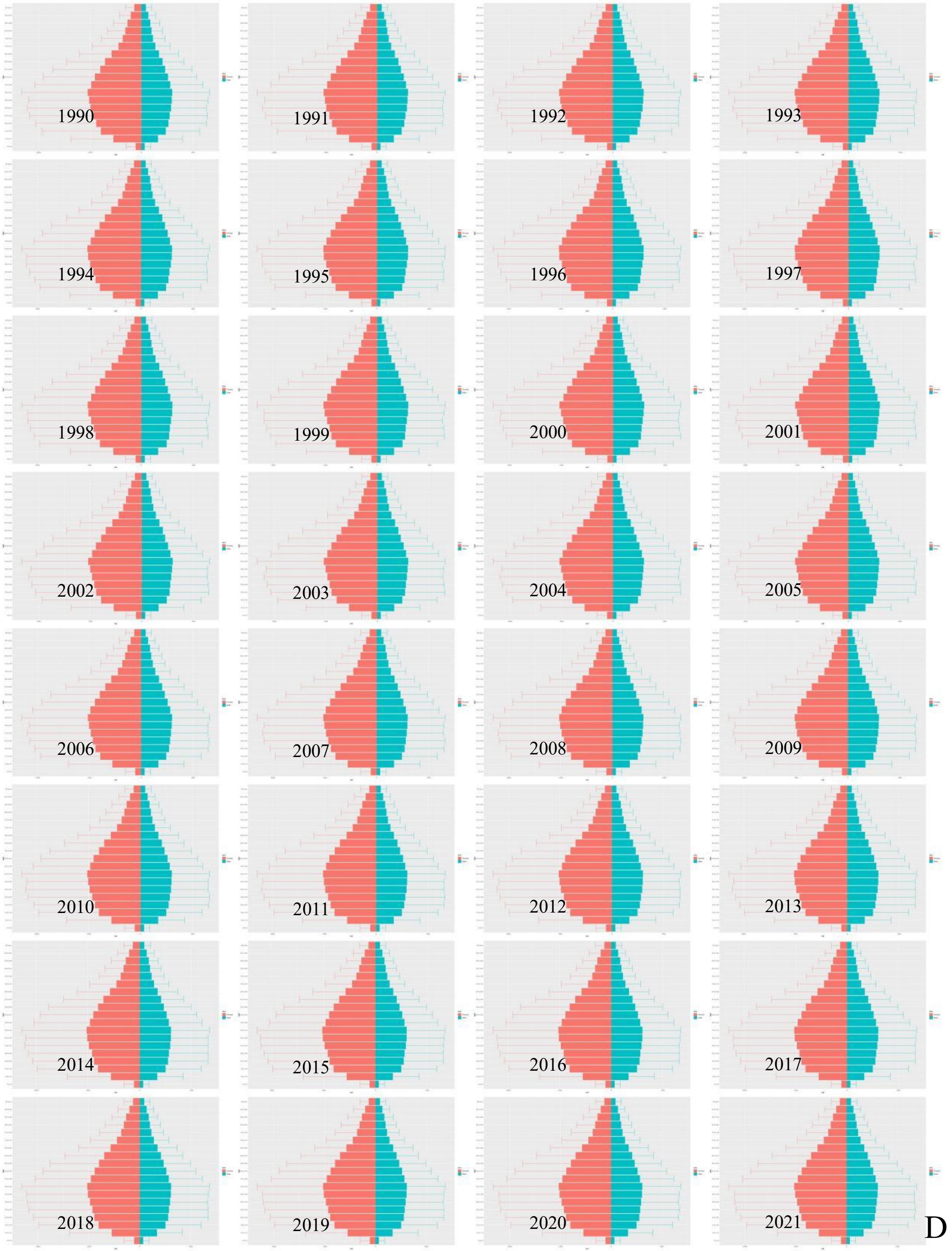

Fig. S43 (A) The prevalence cases of migraine in different ages from 1990 to 2021 in the worldwide; (B) The prevalence rates of migraine in different ages from 1990 to 2021 in the worldwide; (C) The years lived with disability of migraine in different ages from 1990 to 2021 in the worldwide; (D) The years lived with disability rates of migraine in different ages from 1990 to 2021 in the worldwide.

Notes: red for female, green for male; the ordinate from bottom to top is "5 to 9", "10 to 14", "15 to 19", "20 to 24", "25 to 29", "30 to 34", "35 to 39", "40 to 44", "45 to 49", "50 to 54", "55 to 59", "60 to 64", "65 to 69", "70 to 74", "75 to 79", "80 to 84", "85 to 89", "90 to 94", "95 plus".

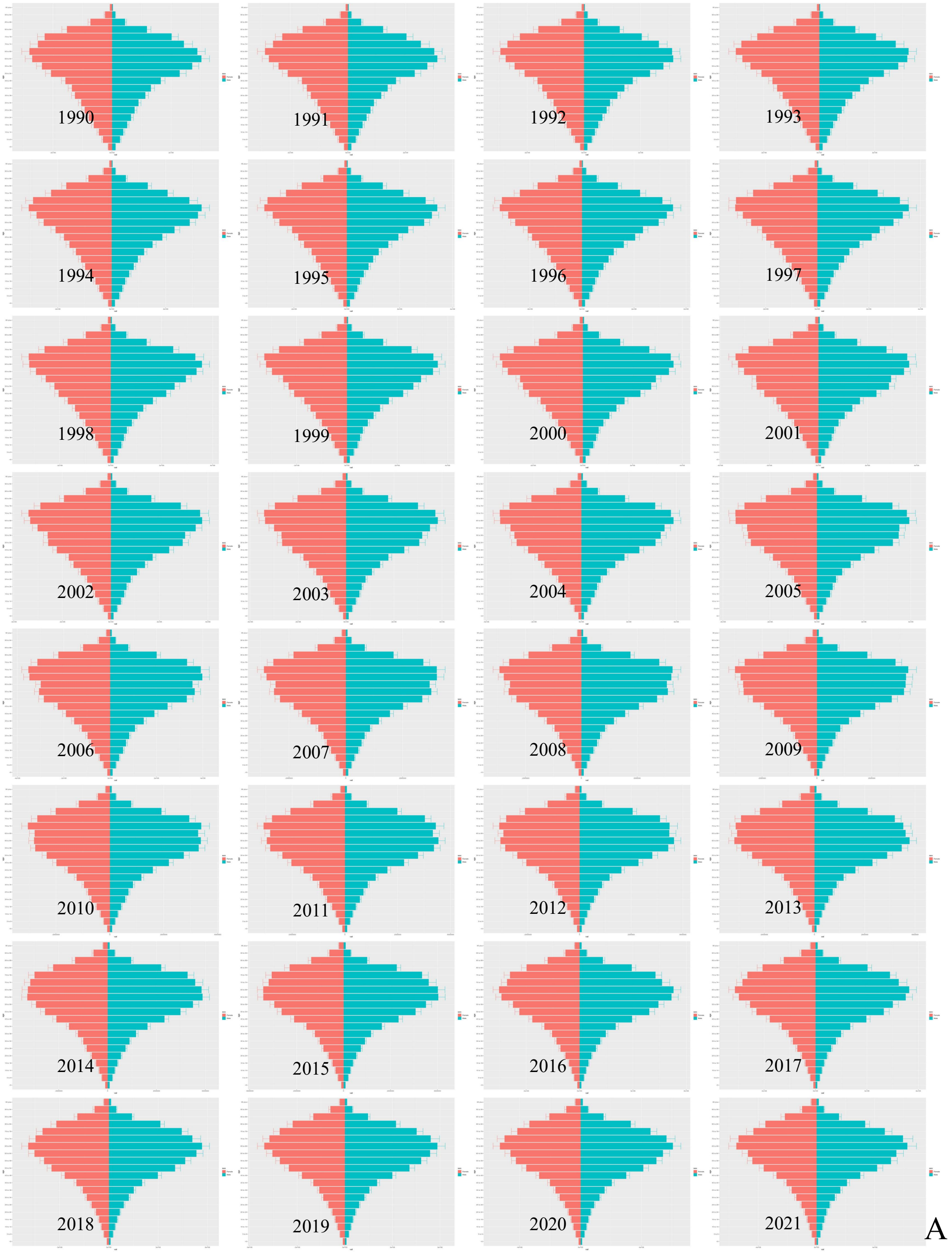
1990
1991
1992
1993
1994
1995
1996
1997
1998
1999
2000
2001
2002
2003
2004
2005
2006
2007
2008
2009
2010
2011
2012
2013
2014
2015
2016
2017
2018
2019
2020
2021
A

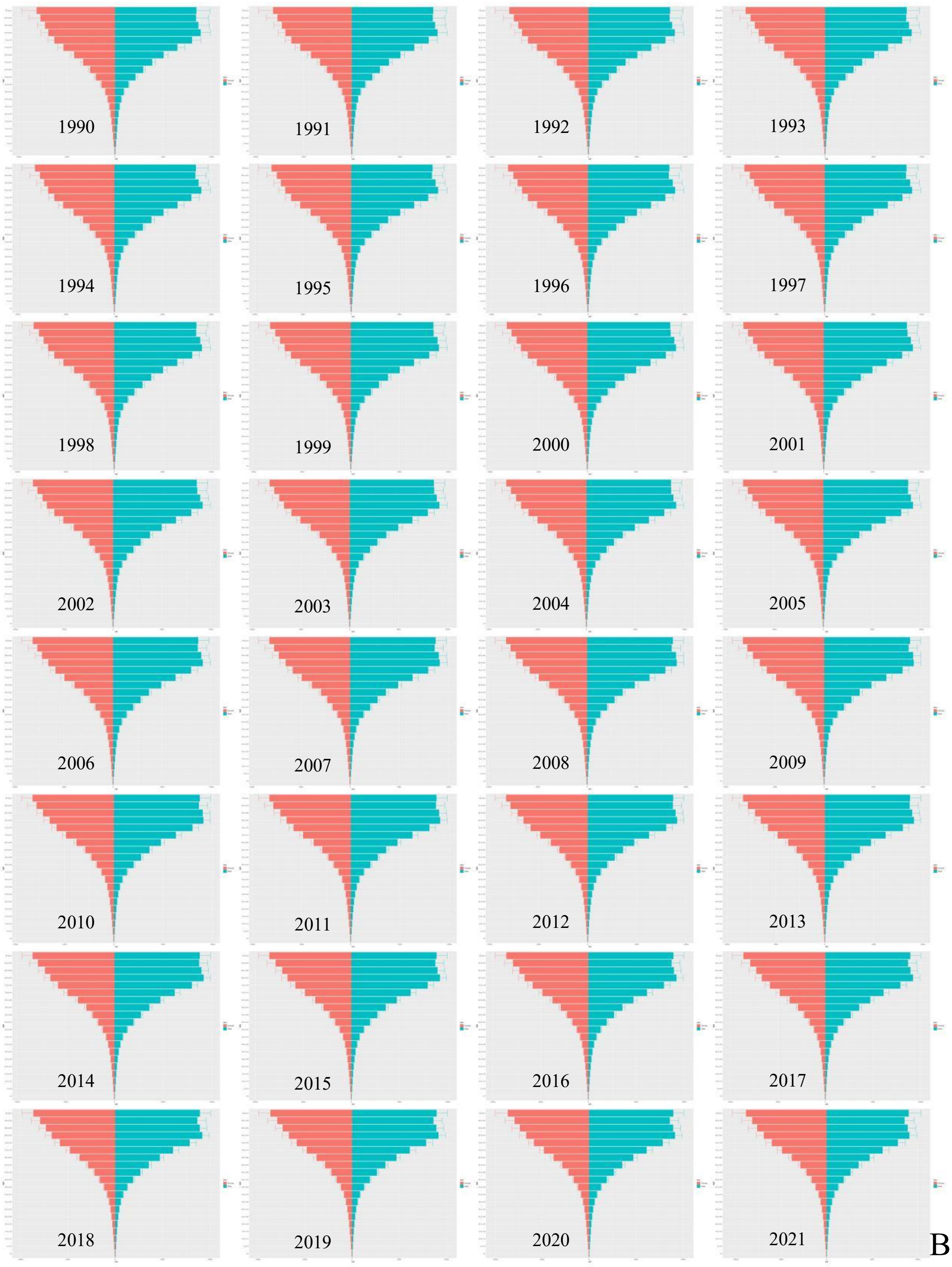
1990
1991
1992
1993
1994
1995
1996
1997
1998
1999
2000
2001
2002
2003
2004
2005
2006
2007
2008
2009
2010
2011
2012
2013
2014
2015
2016
2017
2018
2019
2020
2021

B

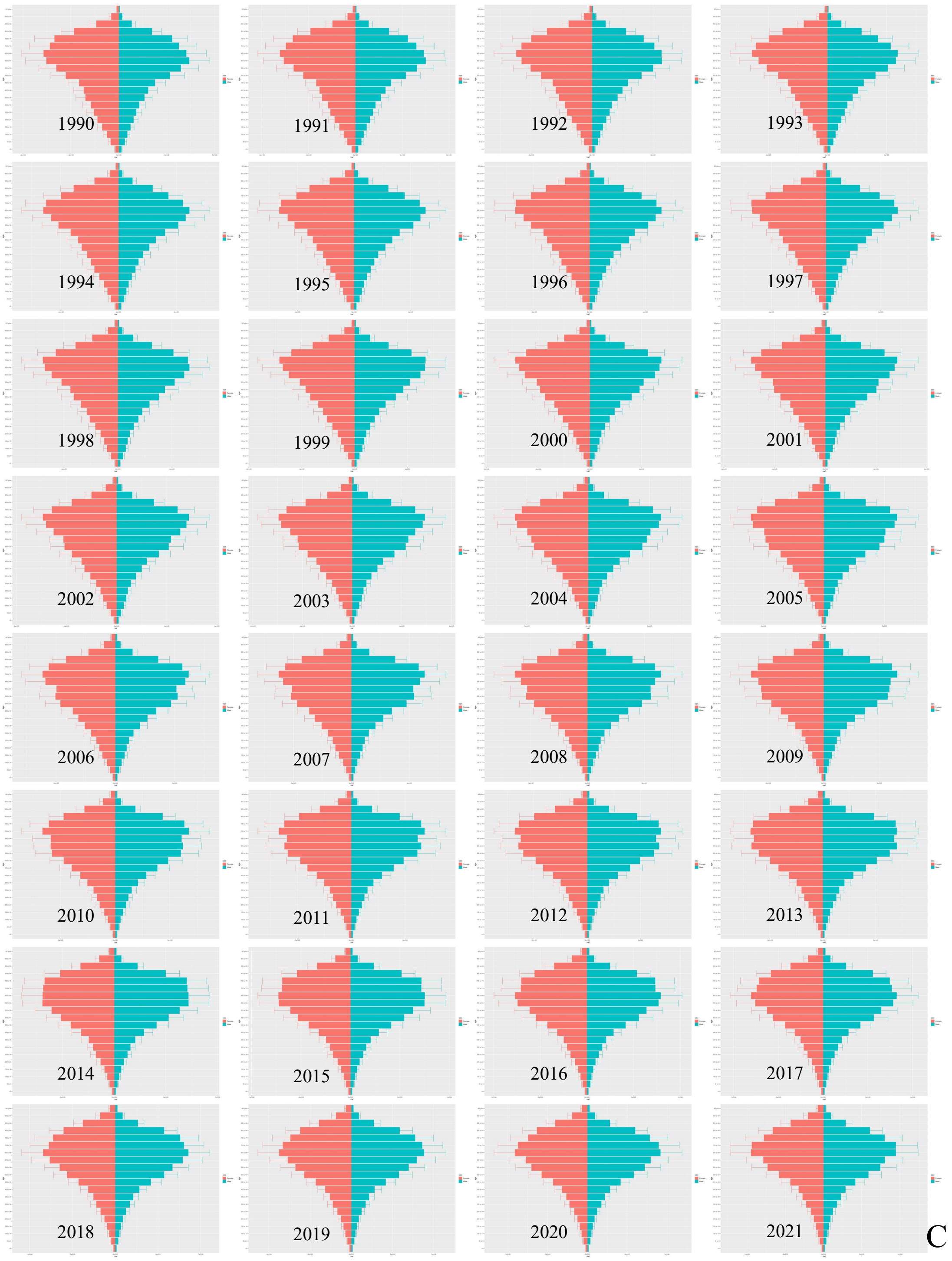
1990
1991
1992
1993
1994
1995
1996
1997
1998
1999
2000
2001
2002
2003
2004
2005
2006
2007
2008
2009
2010
2011
2012
2013
2014
2015
2016
2017
2018
2019
2020
2021
C

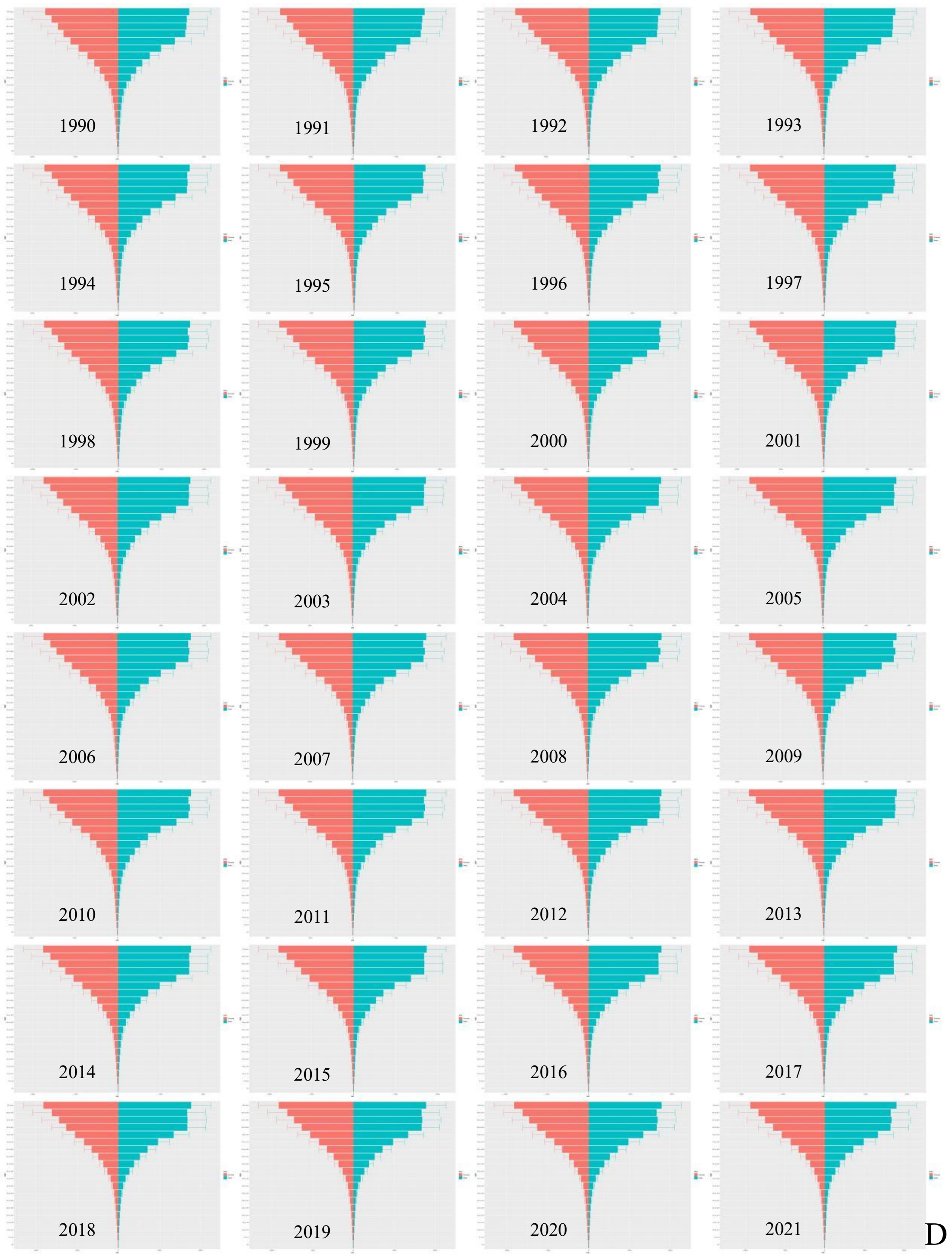

Fig. S44 (A) The prevalence cases of stroke in different ages from 1990 to 2021 in the worldwide; (B) The prevalence rates of stroke in different ages from 1990 to 2021 in the worldwide; (C) The years lived with disability of stroke in different ages from 1990 to 2021 in the worldwide; (D) The years lived with disability rates of stroke in different ages from 1990 to 2021 in the worldwide.

Notes: red for female, green for male; the ordinate from bottom to top is "<5", "5 to 9", "10 to 14", "15 to 19", "20 to 24", "25 to 29", "30 to 34", "35 to 39", "40 to 44", "45 to 49", "50 to 54", "55 to 59", "60 to 64", "65 to 69", "70 to 74", "75 to 79", "80 to 84", "85 to 89", "90 to 94", "95 plus".

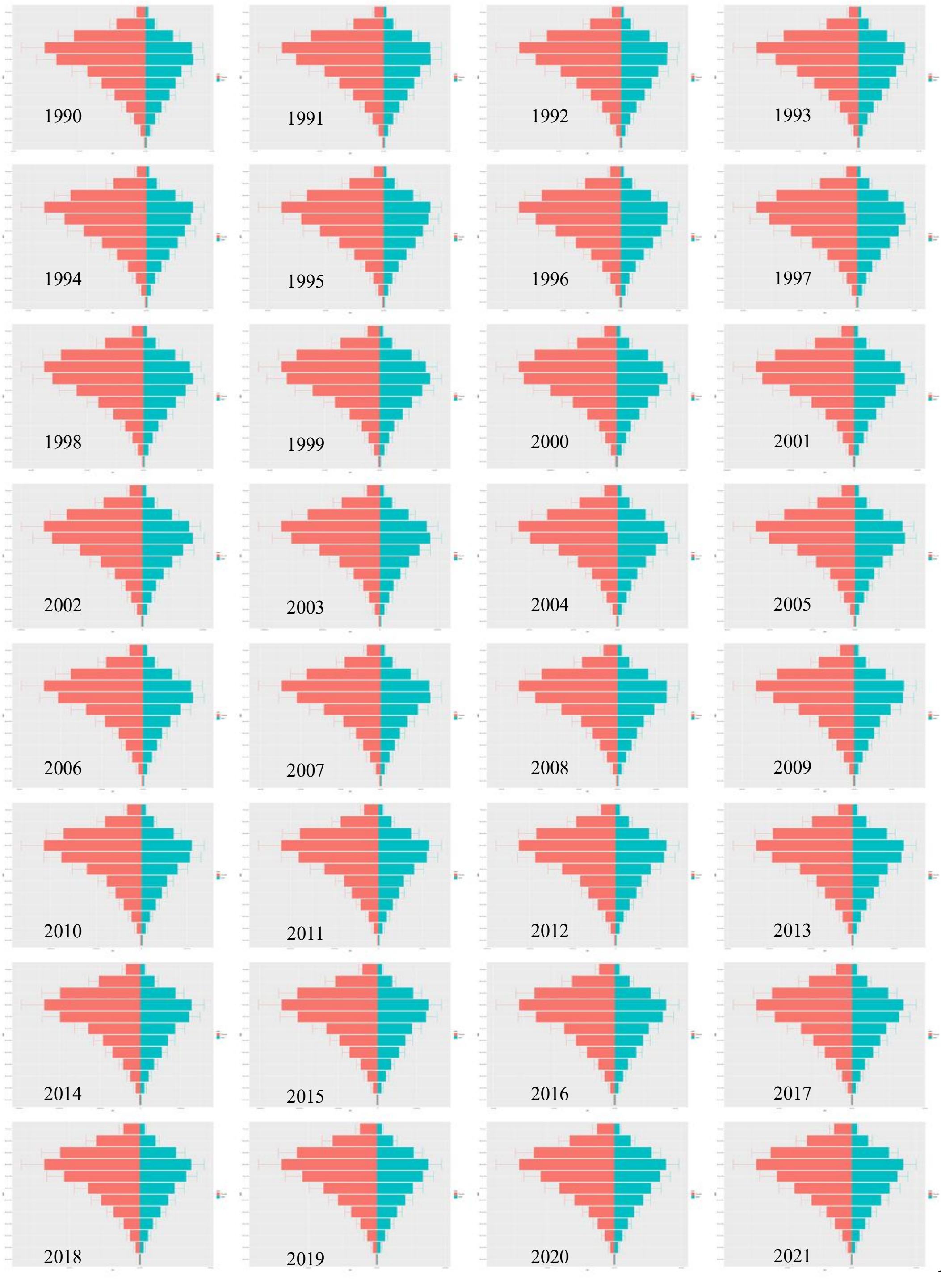
1990
1991
1992
1993
1994
1995
1996
1997
1998
1999
2000
2001
2002
2003
2004
2005
2006
2007
2008
2009
2010
2011
2012
2013
2014
2015
2016
2017
2018
2019
2020
2021
A

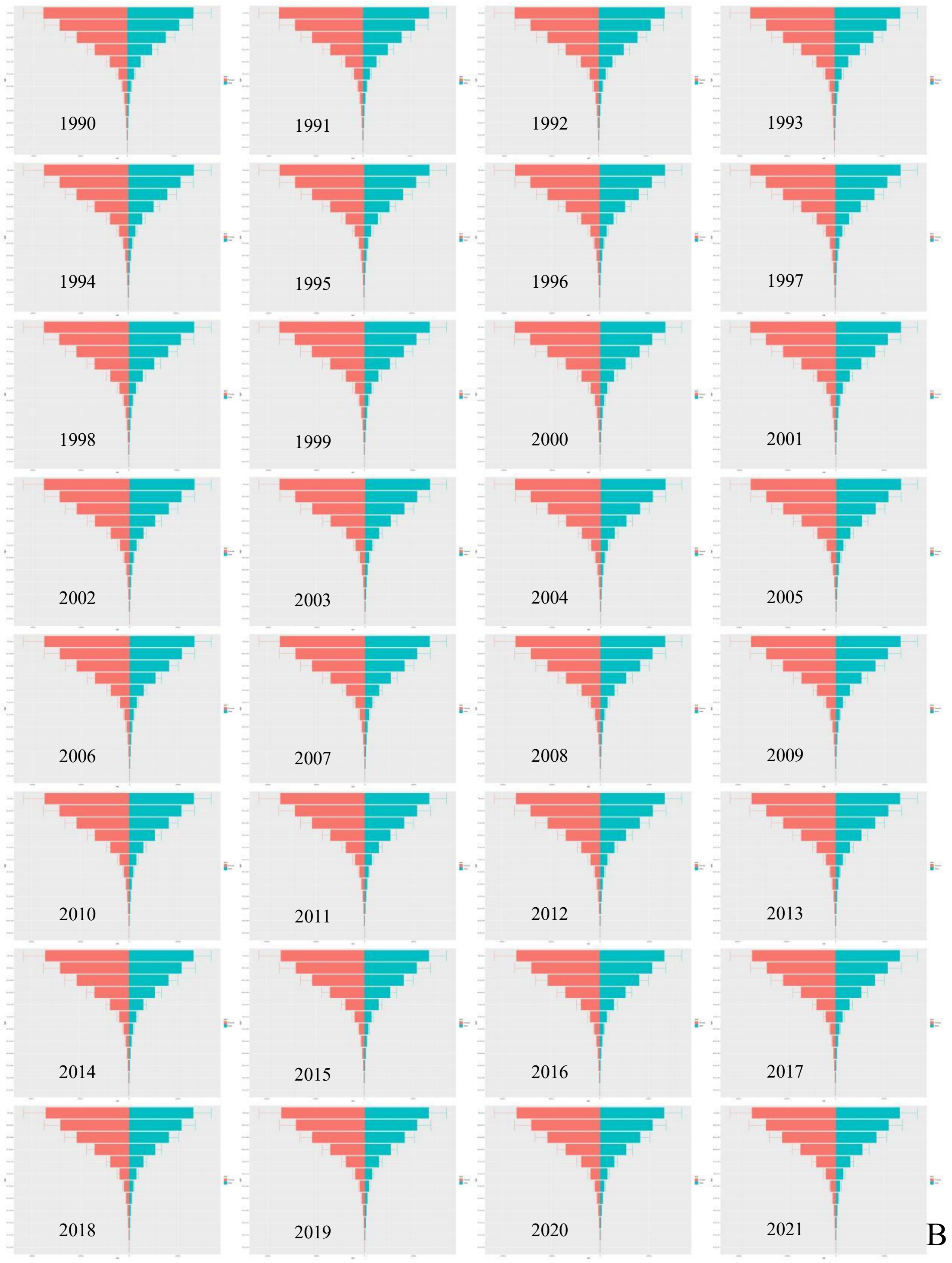
1990
1991
1992
1993
1994
1995
1996
1997
1998
1999
2000
2001
2002
2003
2004
2005
2006
2007
2008
2009
2010
2011
2012
2013
2014
2015
2016
2017
2018
2019
2020
2021
B

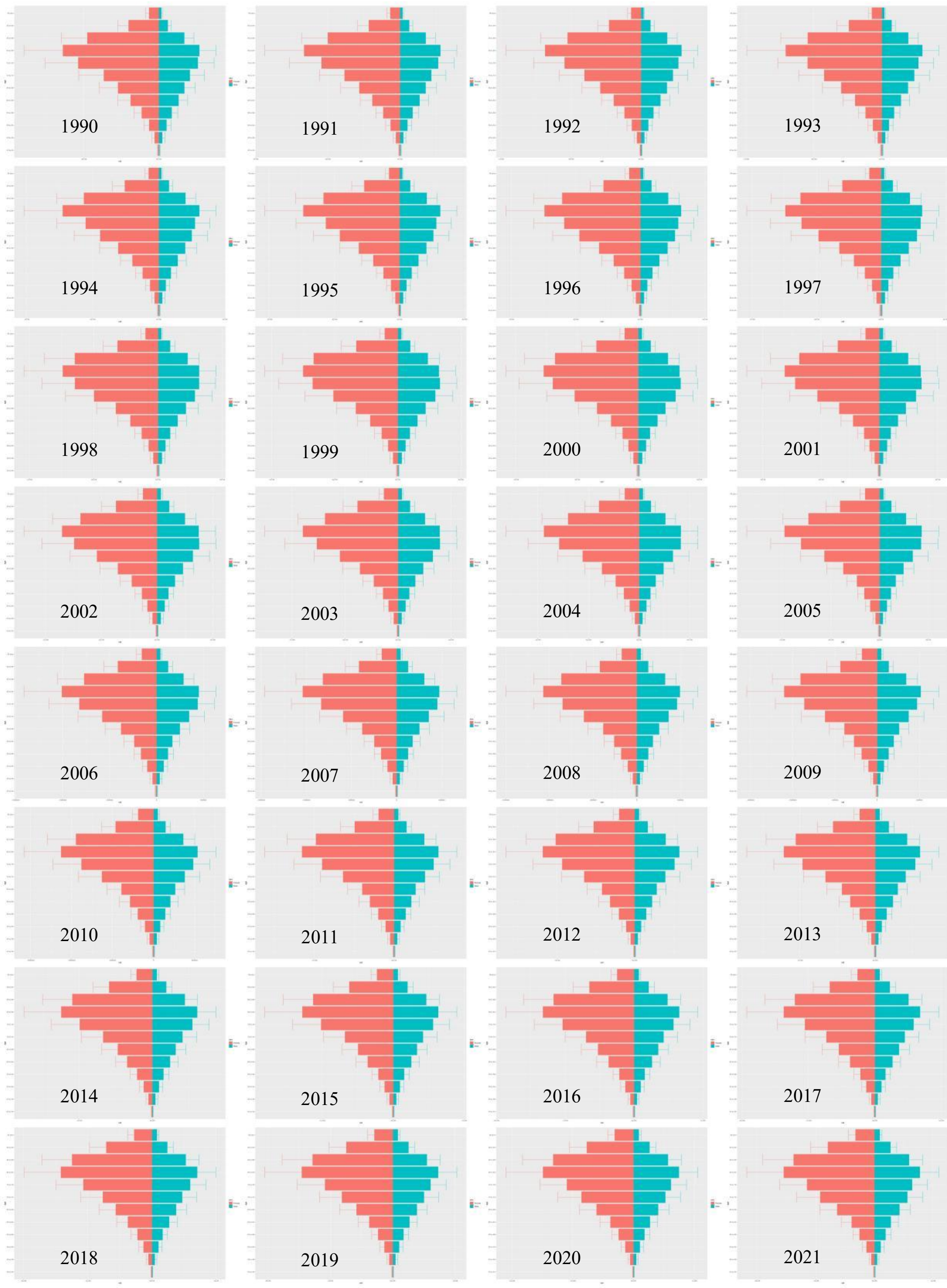
1990
1991
1992
1993
1994
1995
1996
1997
1998
1999
2000
2001
2002
2003
2004
2005
2006
2007
2008
2009
2010
2011
2012
2013
2014
2015
2016
2017
2018
2019
2020
2021

C

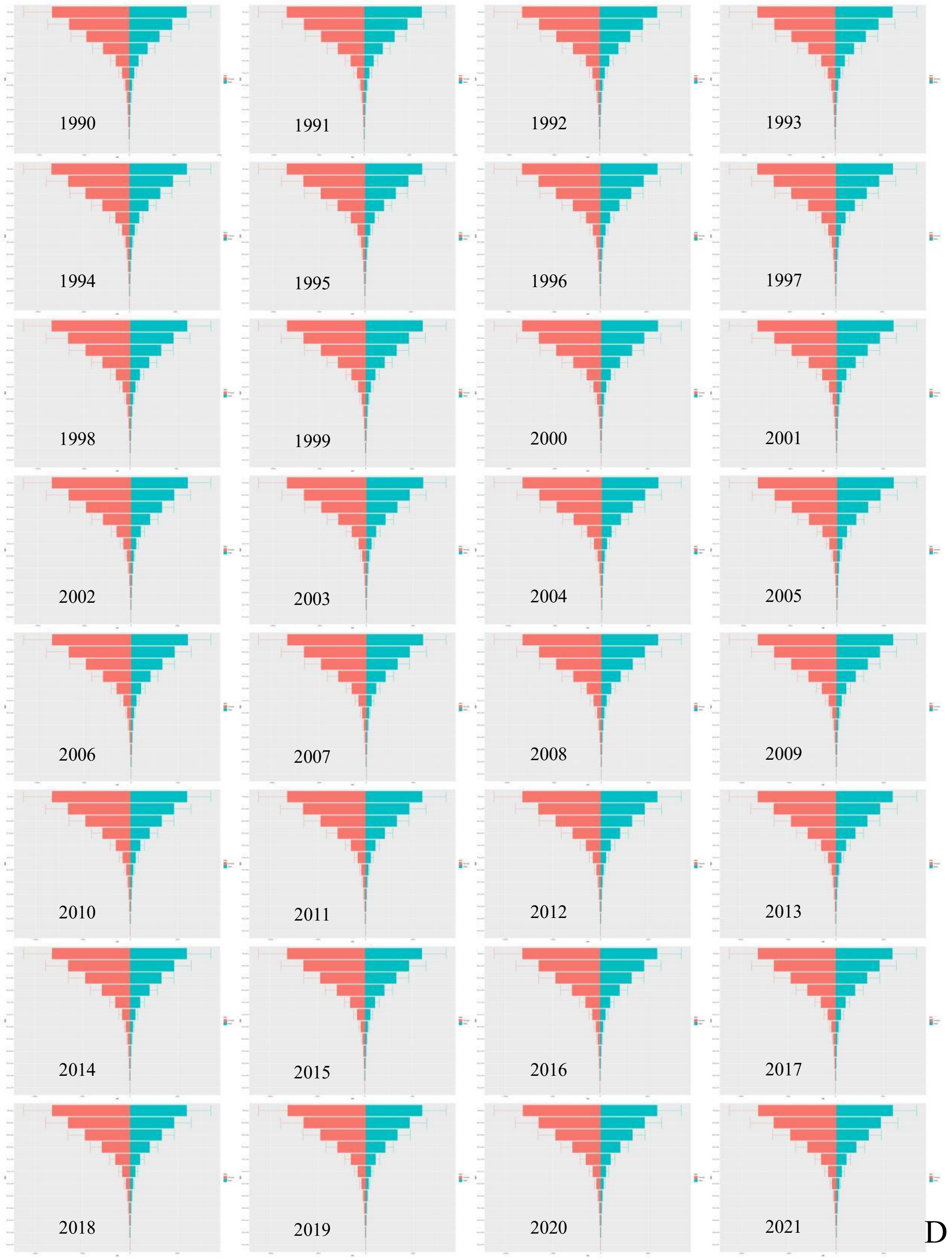

Fig. S45 (A) The prevalence cases of Alzheimer's disease and other dementias in different ages from 1990 to 2021 in the worldwide; (B) The prevalence rates of Alzheimer's disease and other dementias in different ages from 1990 to 2021 in the worldwide; (C) The years lived with disability of Alzheimer's disease and other dementias in different ages from 1990 to 2021 in the worldwide; (D) The years lived with disability rates of Alzheimer's disease and other dementias in different ages from 1990 to 2021 in the worldwide.

Notes: red for female, green for male; the ordinate from bottom to top is "40 to 44", "45 to 49", "50 to 54", "55 to 59", "60 to 64", "65 to 69", "70 to 74", "75 to 79", "80 to 84", "85 to 89", "90 to 94", "95 plus".

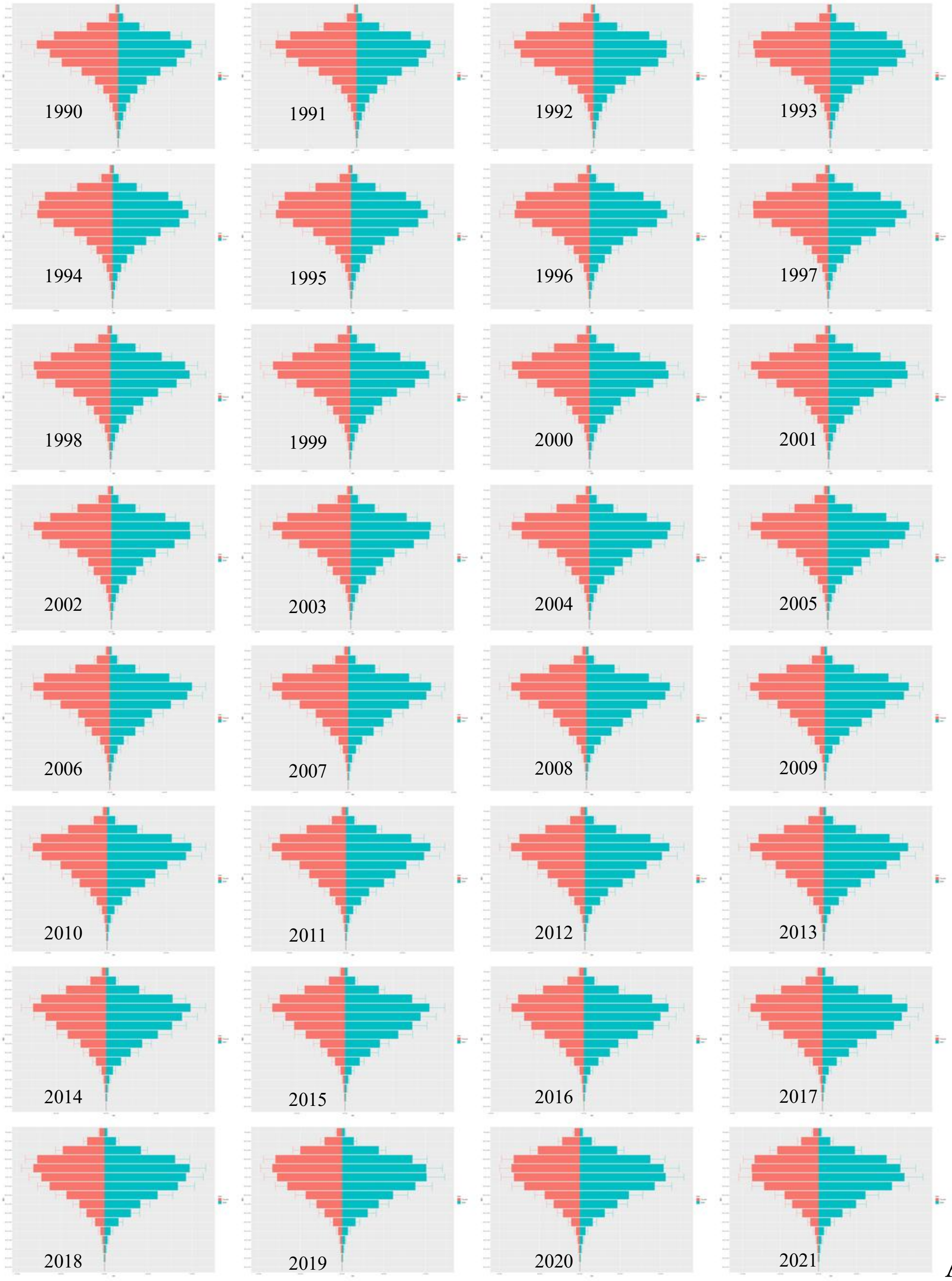
1990
1991
1992
1993
1994
1995
1996
1997
1998
1999
2000
2001
2002
2003
2004
2005
2006
2007
2008
2009
2010
2011
2012
2013
2014
2015
2016
2017
2018
2019
2020
2021
A

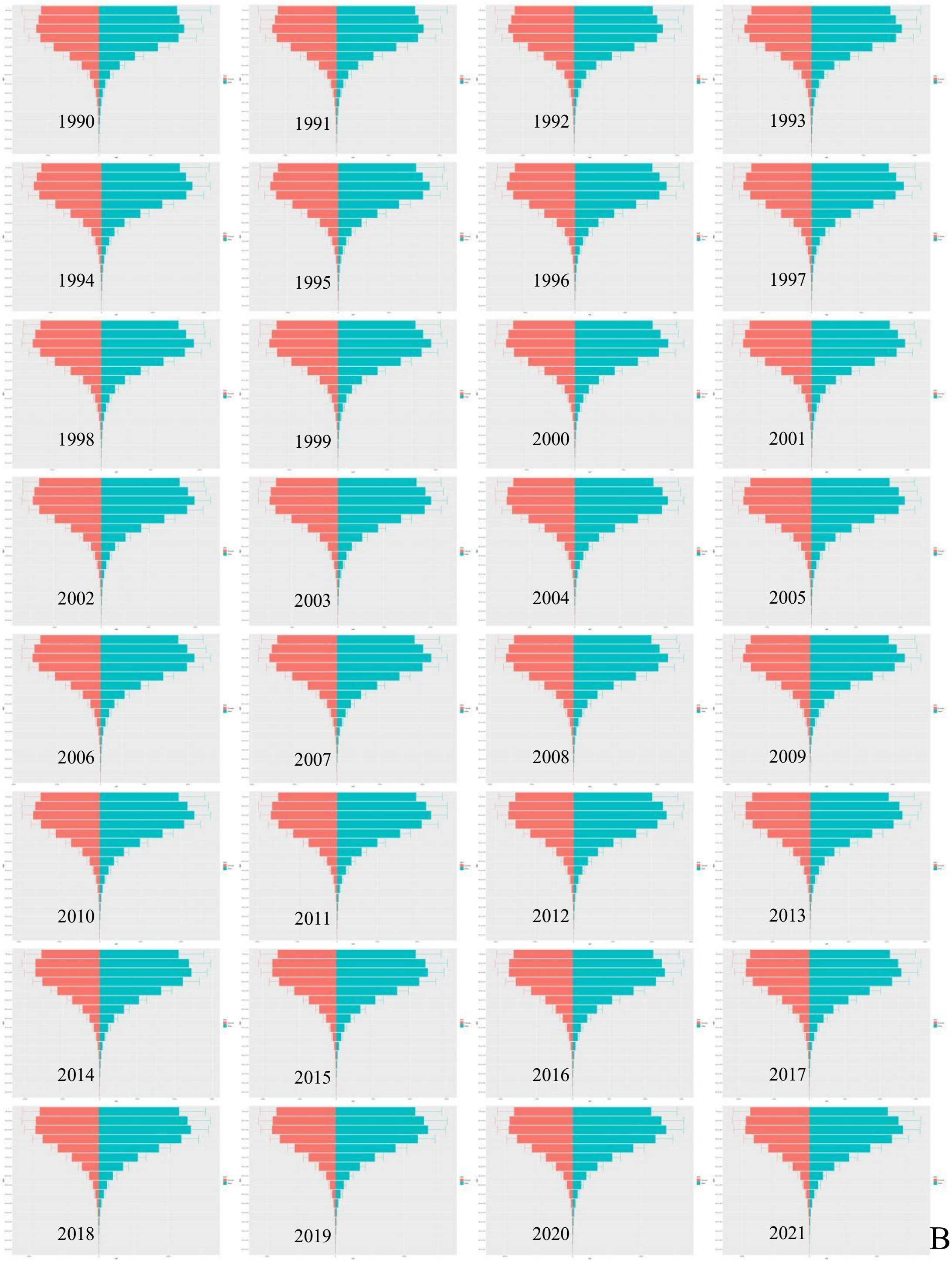
1990
1991
1992
1993
1994
1995
1996
1997
1998
1999
2000
2001
2002
2003
2004
2005
2006
2007
2008
2009
2010
2011
2012
2013
2014
2015
2016
2017
2018
2019
2020
2021
B

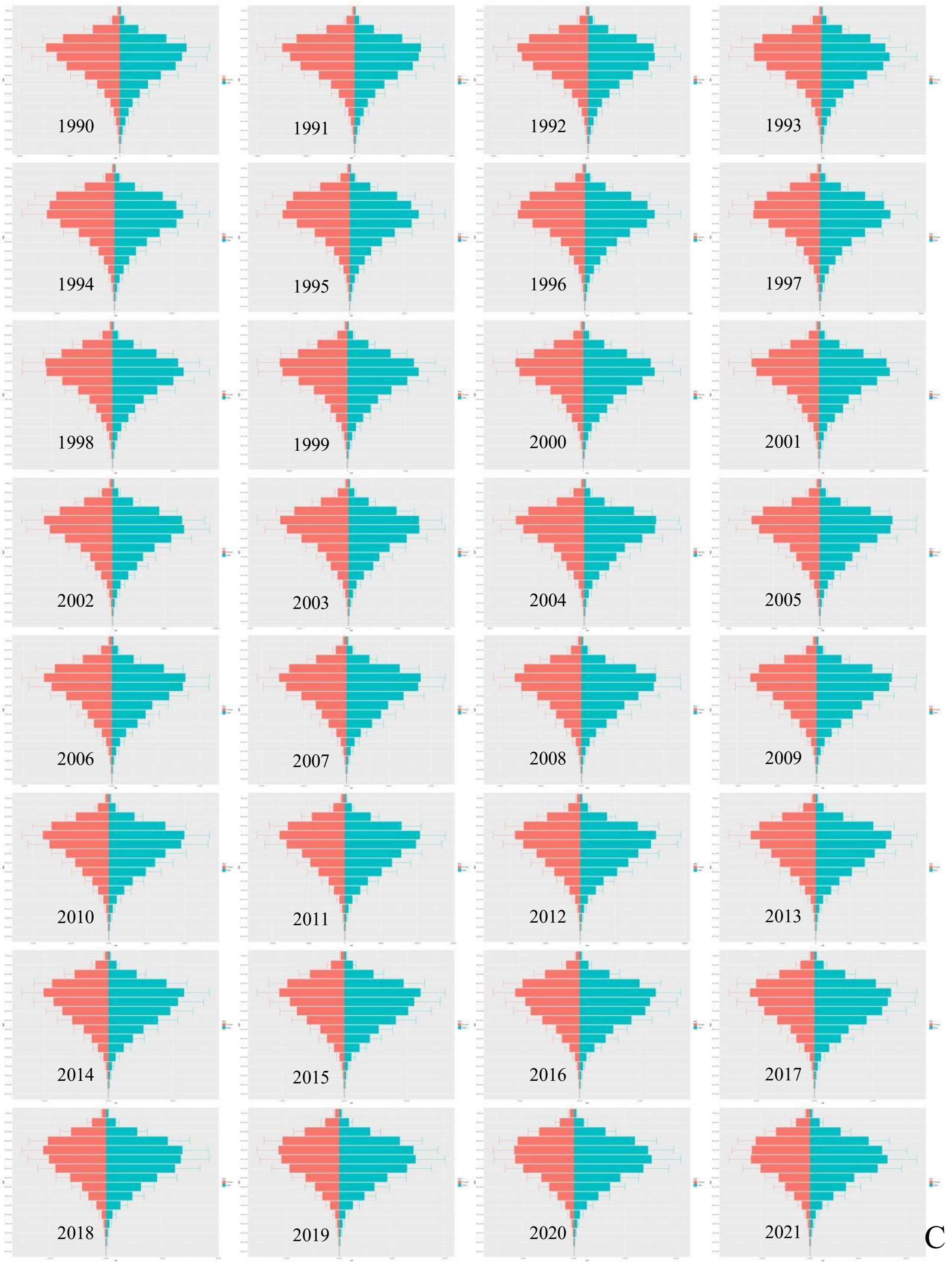
1990
1991
1992
1993
1994
1995
1996
1997
1998
1999
2000
2001
2002
2003
2004
2005
2006
2007
2008
2009
2010
2011
2012
2013
2014
2015
2016
2017
2018
2019
2020
2021
C

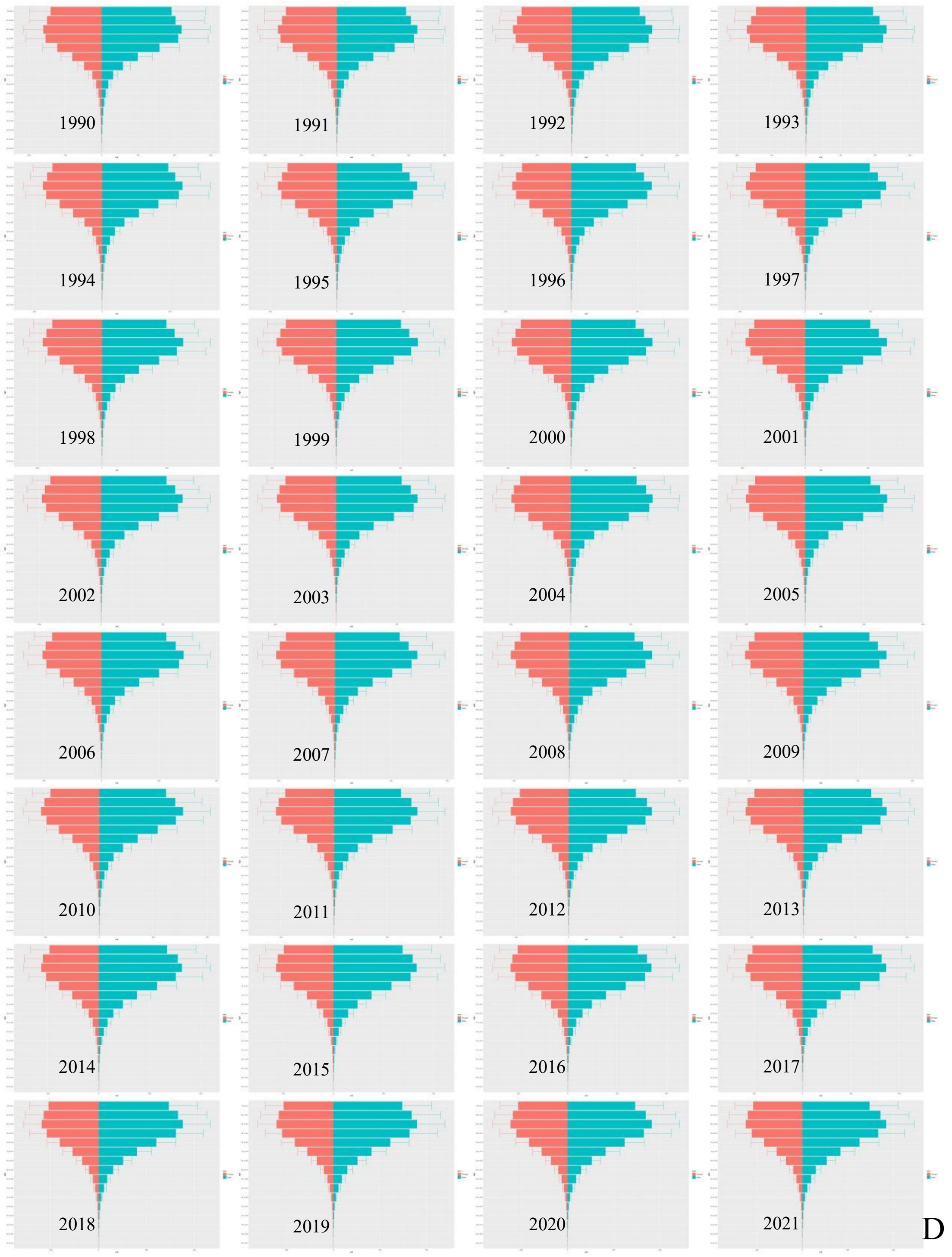

Fig. S46 (A) The prevalence cases of Parkinson's disease in different ages from 1990 to 2021 in the worldwide; (B) The prevalence rates of Parkinson's disease in different ages from 1990 to 2021 in the worldwide; (C) The years lived with disability of Parkinson's disease in different ages from 1990 to 2021 in the worldwide; (D) The years lived with disability rates of Parkinson's disease in different ages from 1990 to 2021 in the worldwide.

Notes: red for female, green for male; the ordinate from bottom to top is "20 to 24", "25 to 29", "30 to 34", "35 to 39", "40 to 44", "45 to 49", "50 to 54", "55 to 59", "60 to 64", "65 to 69", "70 to 74", "75 to 79", "80 to 84", "85 to 89", "90 to 94", "95 plus".

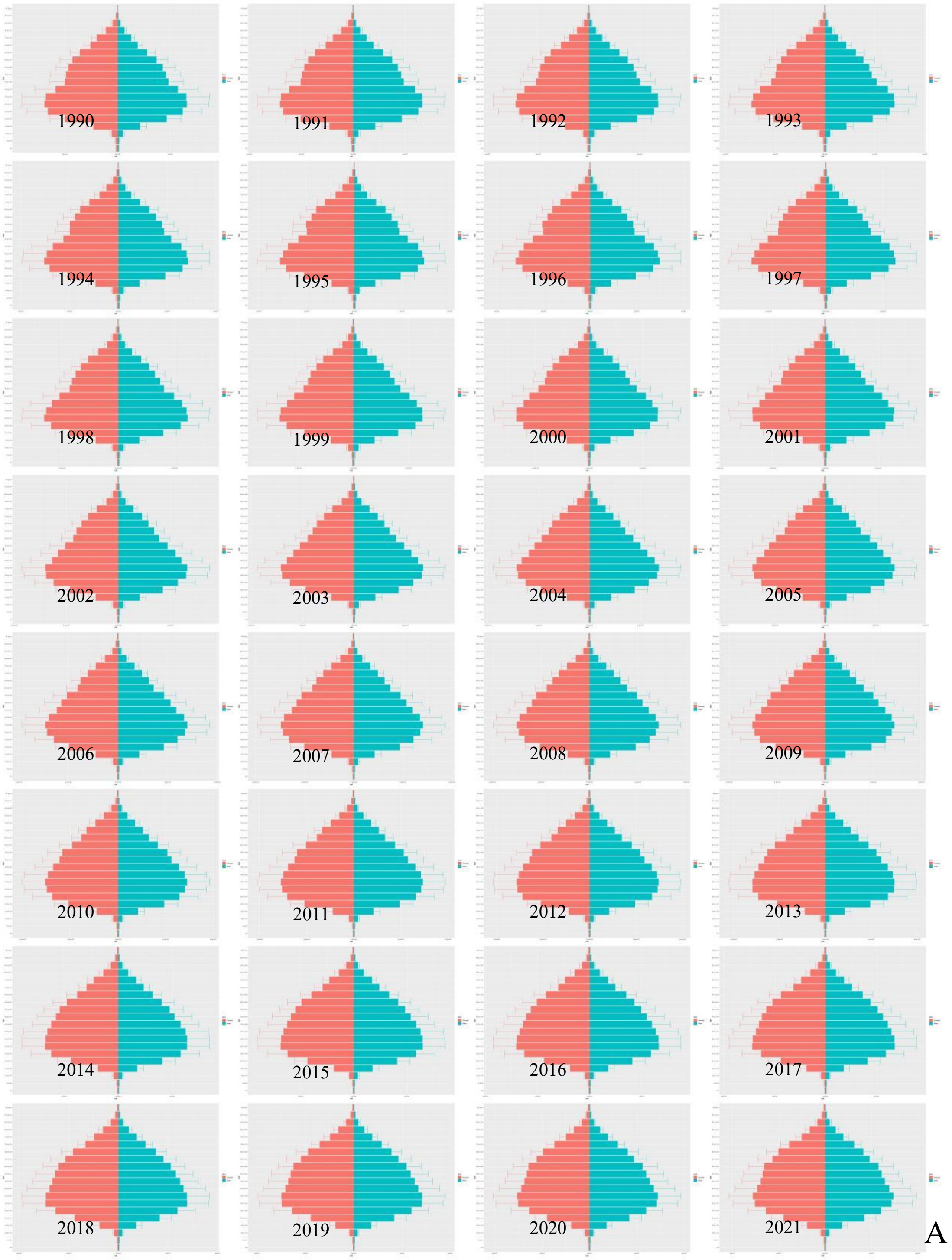
1990
1991
1992
1993
1994
1995
1996
1997
1998
1999
2000
2001
2002
2003
2004
2005
2006
2007
2008
2009
2010
2011
2012
2013
2014
2015
2016
2017
2018
2019
2020
2021
A

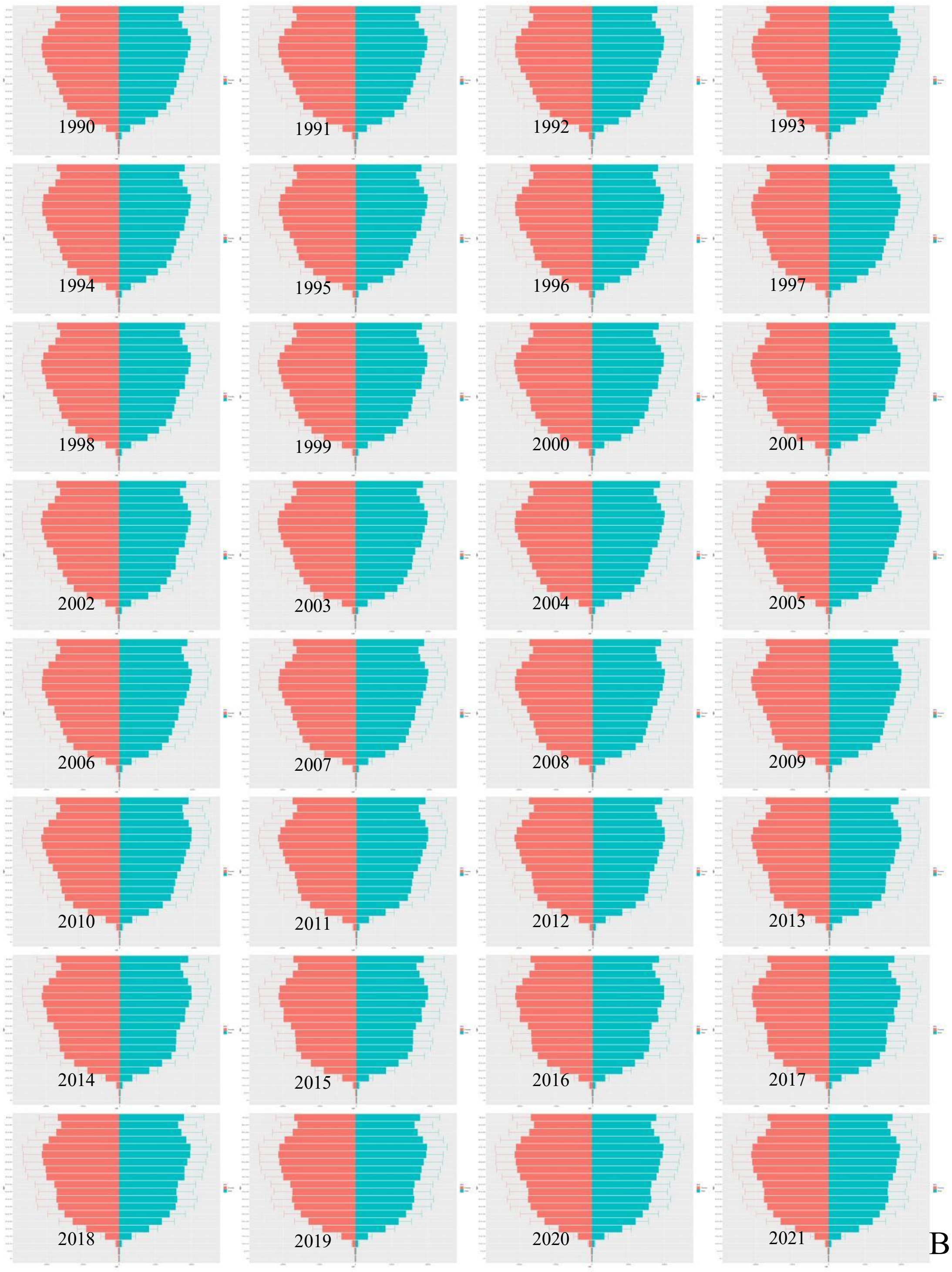
1990
1991
1992
1993
1994
1995
1996
1997
1998
1999
2000
2001
2002
2003
2004
2005
2006
2007
2008
2009
2010
2011
2012
2013
2014
2015
2016
2017
2018
2019
2020
2021

B

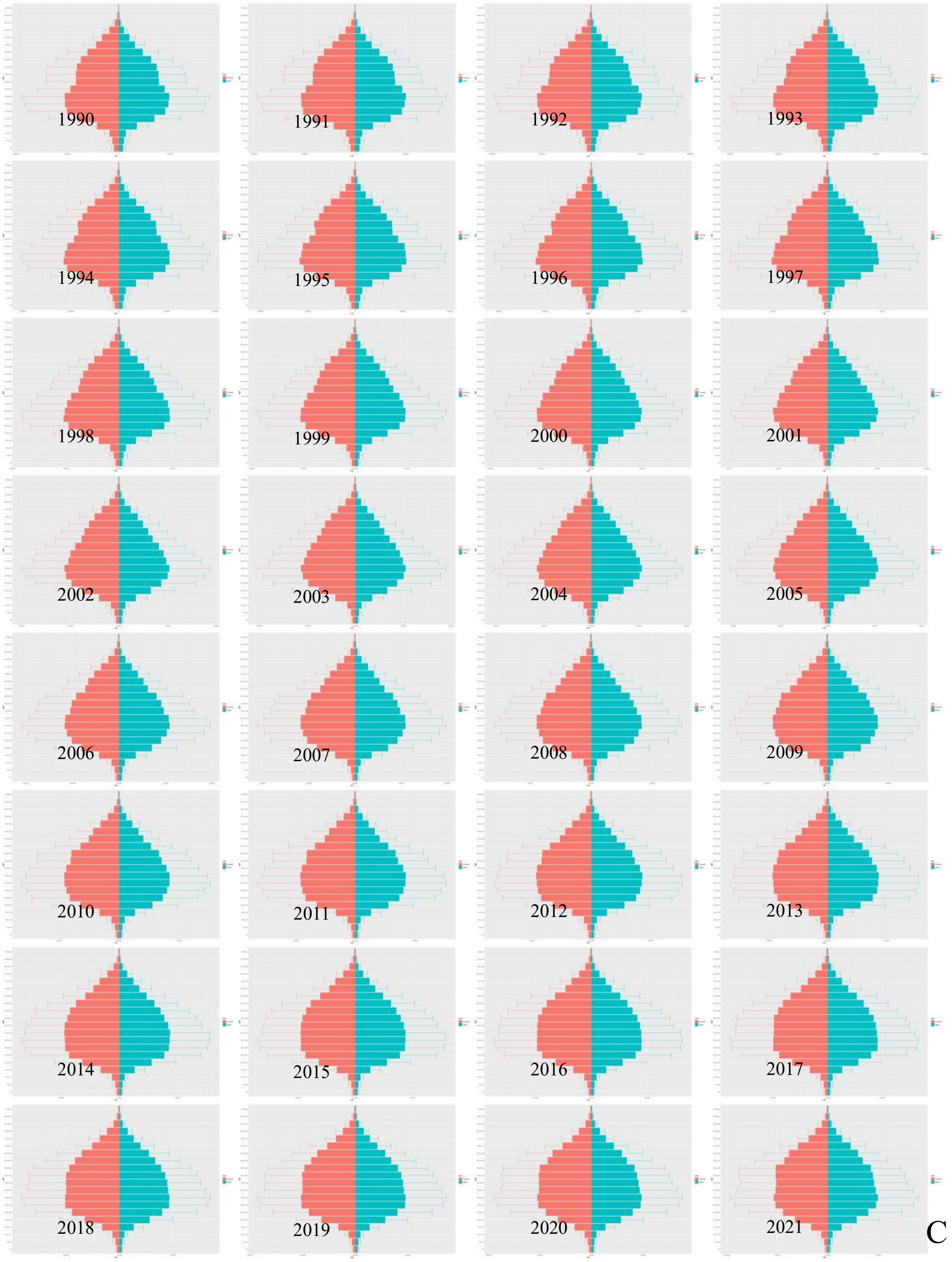
1990
1991
1992
1993
1994
1995
1996
1997
1998
1999
2000
2001
2002
2003
2004
2005
2006
2007
2008
2009
2010
2011
2012
2013
2014
2015
2016
2017
2018
2019
2020
2021
C

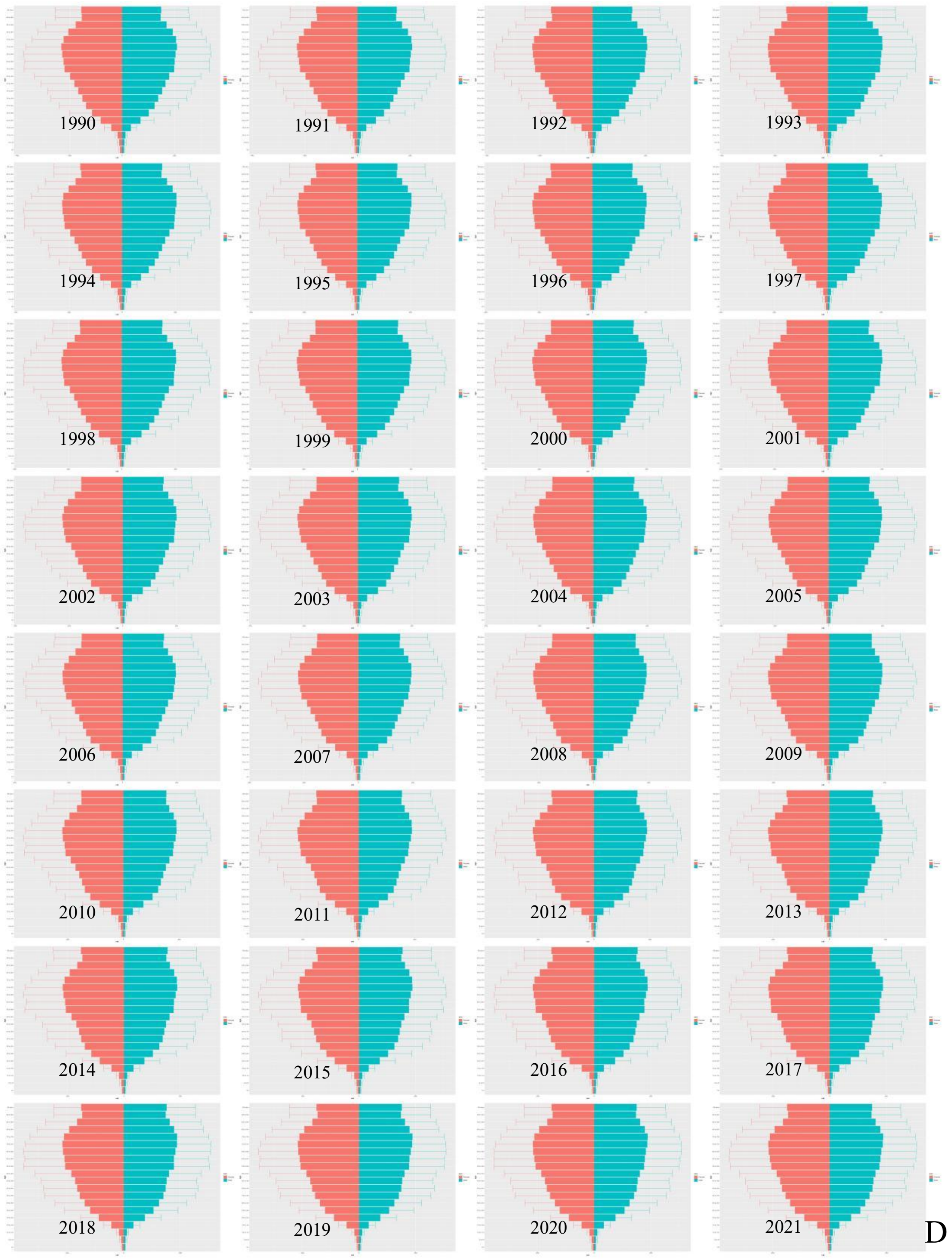

Fig. S47 (A) The prevalence cases of upper digestive system diseases in different ages from 1990 to 2021 in the worldwide; (B) The prevalence rates of upper digestive system diseases in different ages from 1990 to 2021 in the worldwide; (C) The years lived with disability of upper digestive system diseases in different ages from 1990 to 2021 in the worldwide; (D) The years lived with disability rates of upper digestive system diseases in different ages from 1990 to 2021 in the worldwide.

Notes: red for female, green for male; the ordinate from bottom to top is "<5", "5 to 9", "10 to 14", "15 to 19", "20 to 24", "25 to 29", "30 to 34", "35 to 39", "40 to 44", "45 to 49", "50 to 54", "55 to 59", "60 to 64", "65 to 69", "70 to 74", "75 to 79", "80 to 84", "85 to 89", "90 to 94", "95 plus".

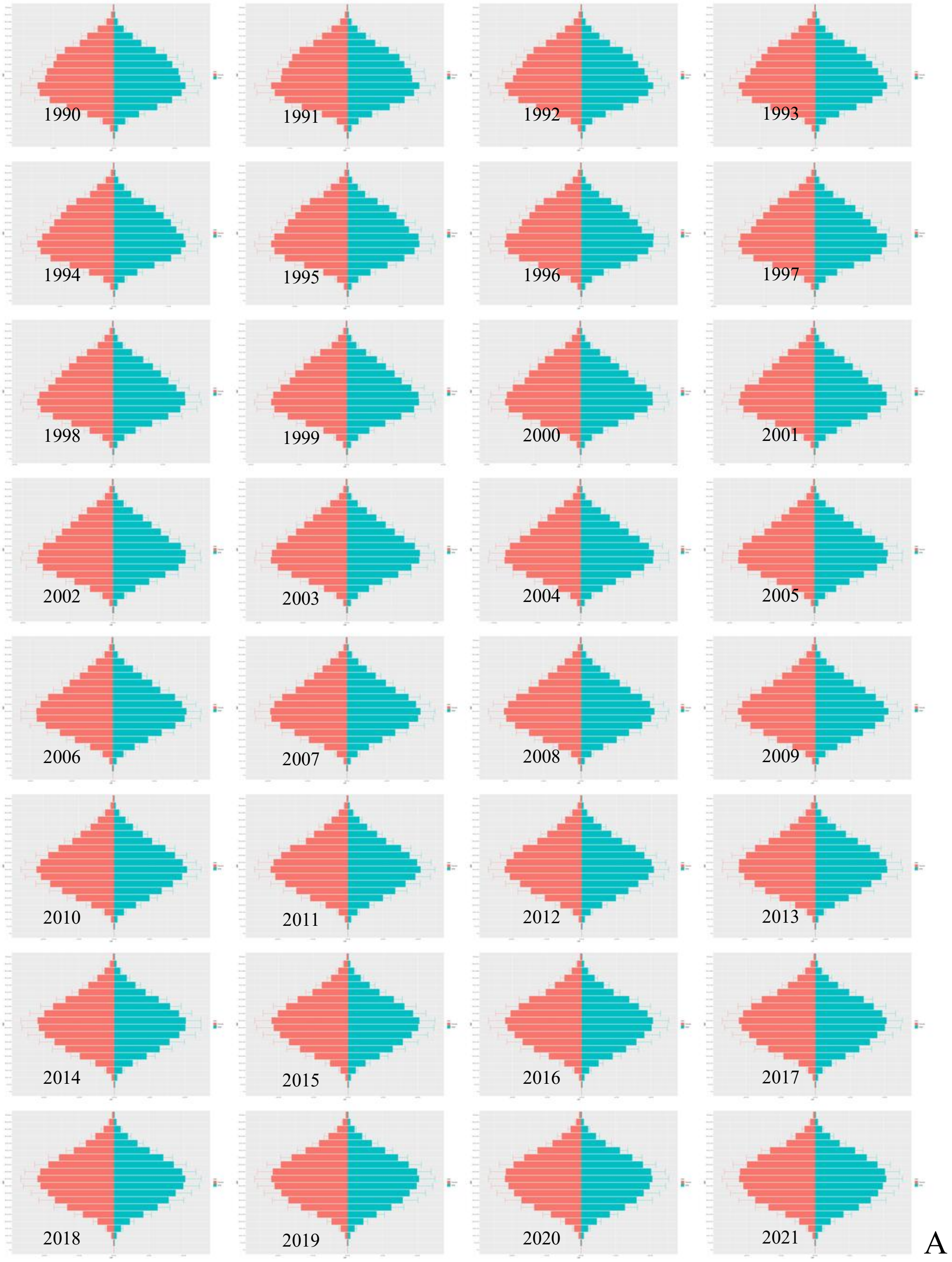
1990
1991
1992
1993
1994
1995
1996
1997
1998
1999
2000
2001
2002
2003
2004
2005
2006
2007
2008
2009
2010
2011
2012
2013
2014
2015
2016
2017
2018
2019
2020
2021
A

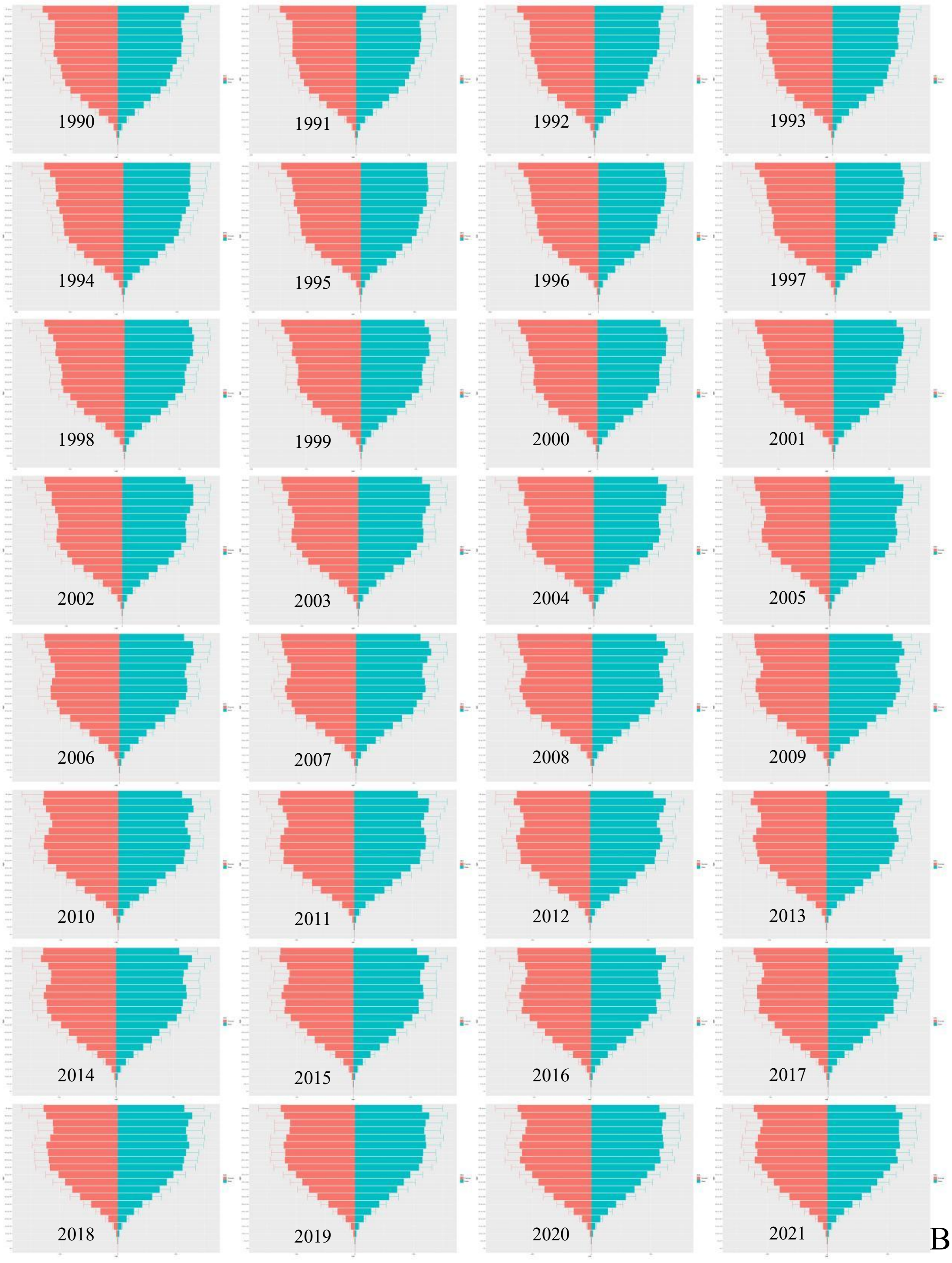
1990
1991
1992
1993
1994
1995
1996
1997
1998
1999
2000
2001
2002
2003
2004
2005
2006
2007
2008
2009
2010
2011
2012
2013
2014
2015
2016
2017
2018
2019
2020
2021
B

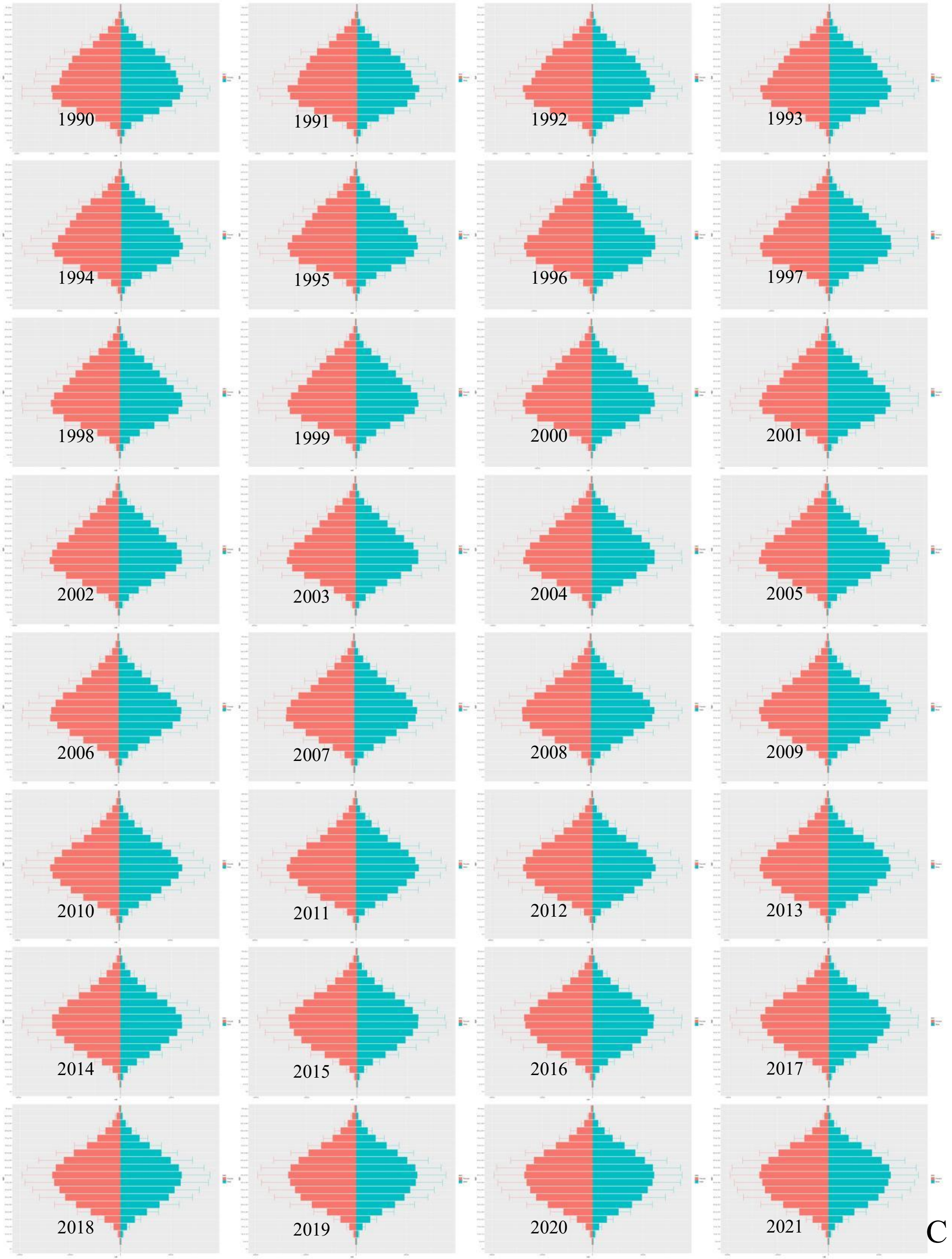

1990
1991
1992
1993
1994
1995
1996
1997
1998
1999
2000
2001
2002
2003
2004
2005
2006
2007
2008
2009
2010
2011
2012
2013
2014
2015
2016
2017
2018
2019
2020
2021
C

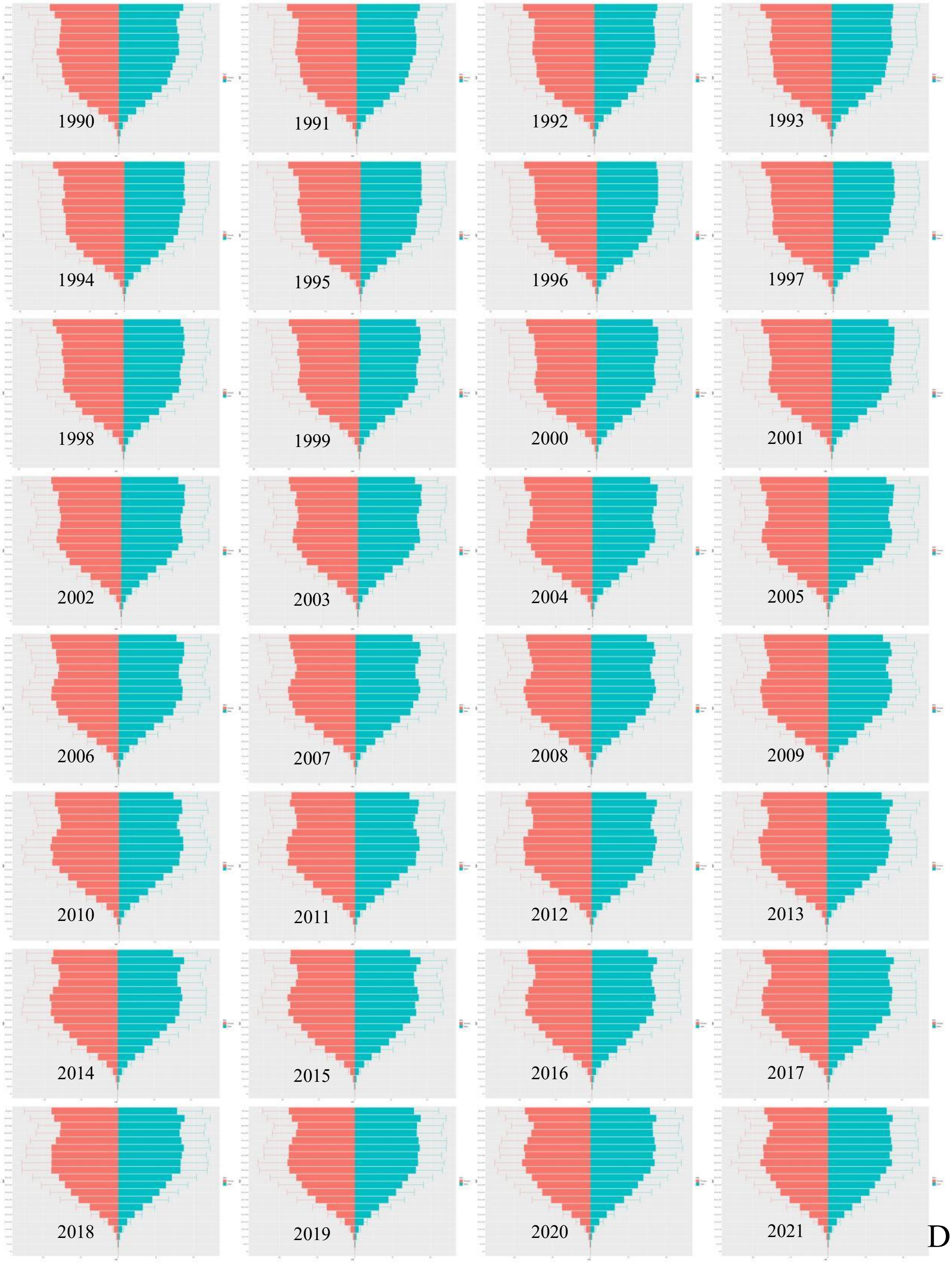

D

Fig. S48 (A) The prevalence cases of inflammatory bowel disease in different ages from 1990 to 2021 in the worldwide; (B) The prevalence rates of inflammatory bowel disease in different ages from 1990 to 2021 in the worldwide; (C) The years lived with disability of inflammatory bowel disease in different ages from 1990 to 2021 in the worldwide; (D) The years lived with disability rates of inflammatory bowel disease in different ages from 1990 to 2021 in the worldwide.

Notes: red for female, green for male; the ordinate from bottom to top is "<5", "5 to 9", "10 to 14", "15 to 19", "20 to 24", "25 to 29", "30 to 34", "35 to 39", "40 to 44", "45 to 49", "50 to 54", "55 to 59", "60 to 64", "65 to 69", "70 to 74", "75 to 79", "80 to 84", "85 to 89", "90 to 94", "95 plus".

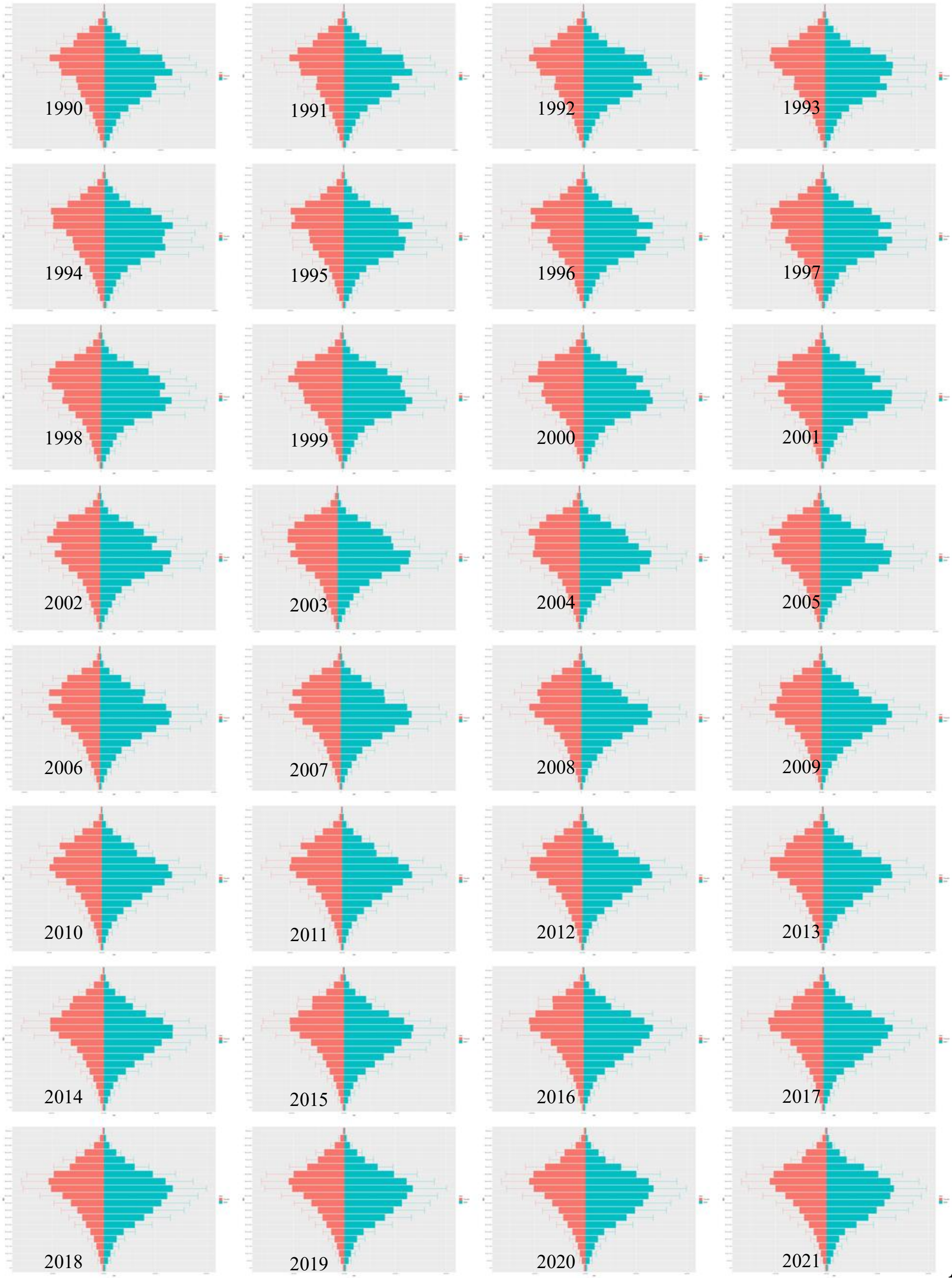
1990
1991
1992
1993
1994
1995
1996
1997
1998
1999
2000
2001
2002
2003
2004
2005
2006
2007
2008
2009
2010
2011
2012
2013
2014
2015
2016
2017
2018
2019
2020
2021

A

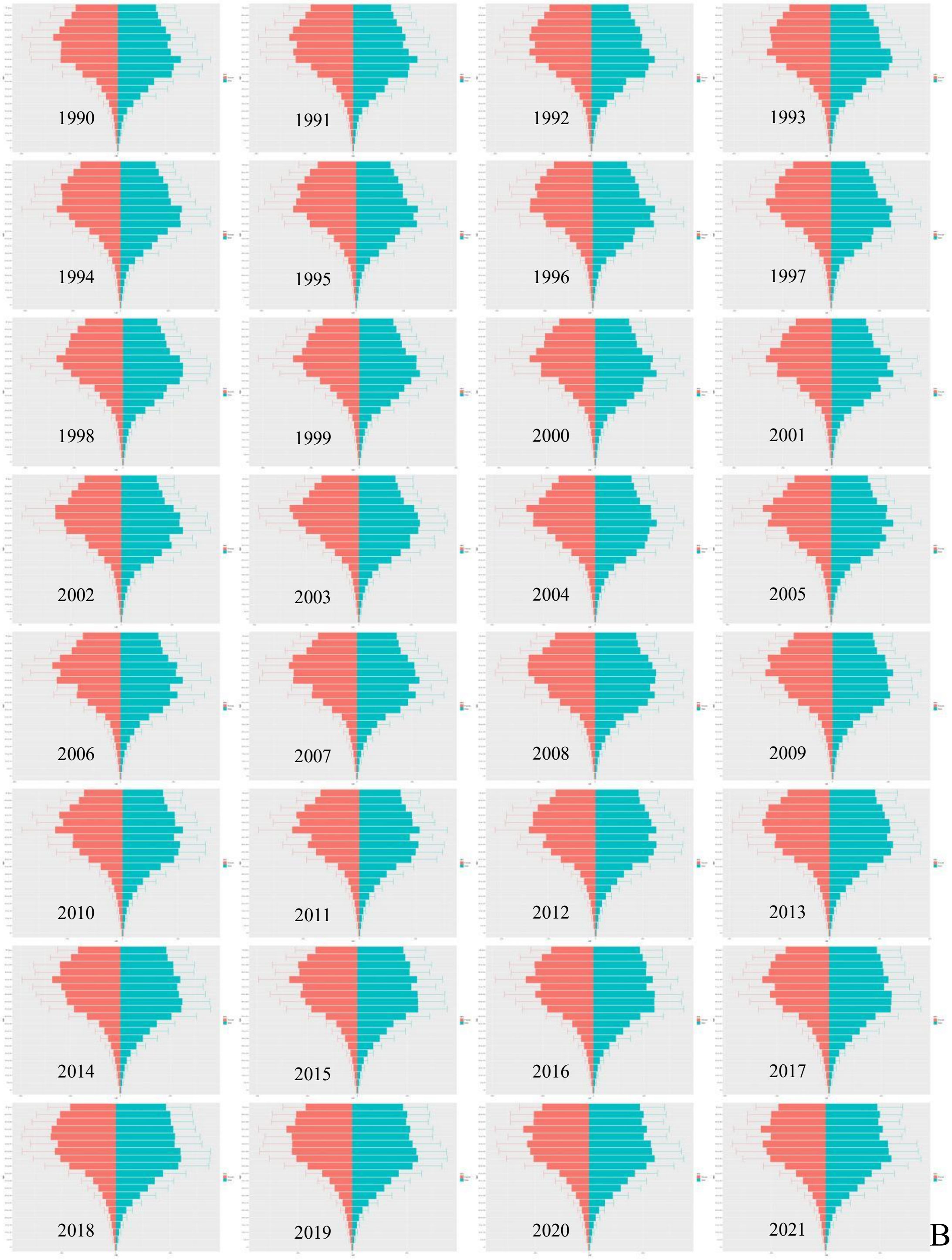
1990
1991
1992
1993
1994
1995
1996
1997
1998
1999
2000
2001
2002
2003
2004
2005
2006
2007
2008
2009
2010
2011
2012
2013
2014
2015
2016
2017
2018
2019
2020
2021

B

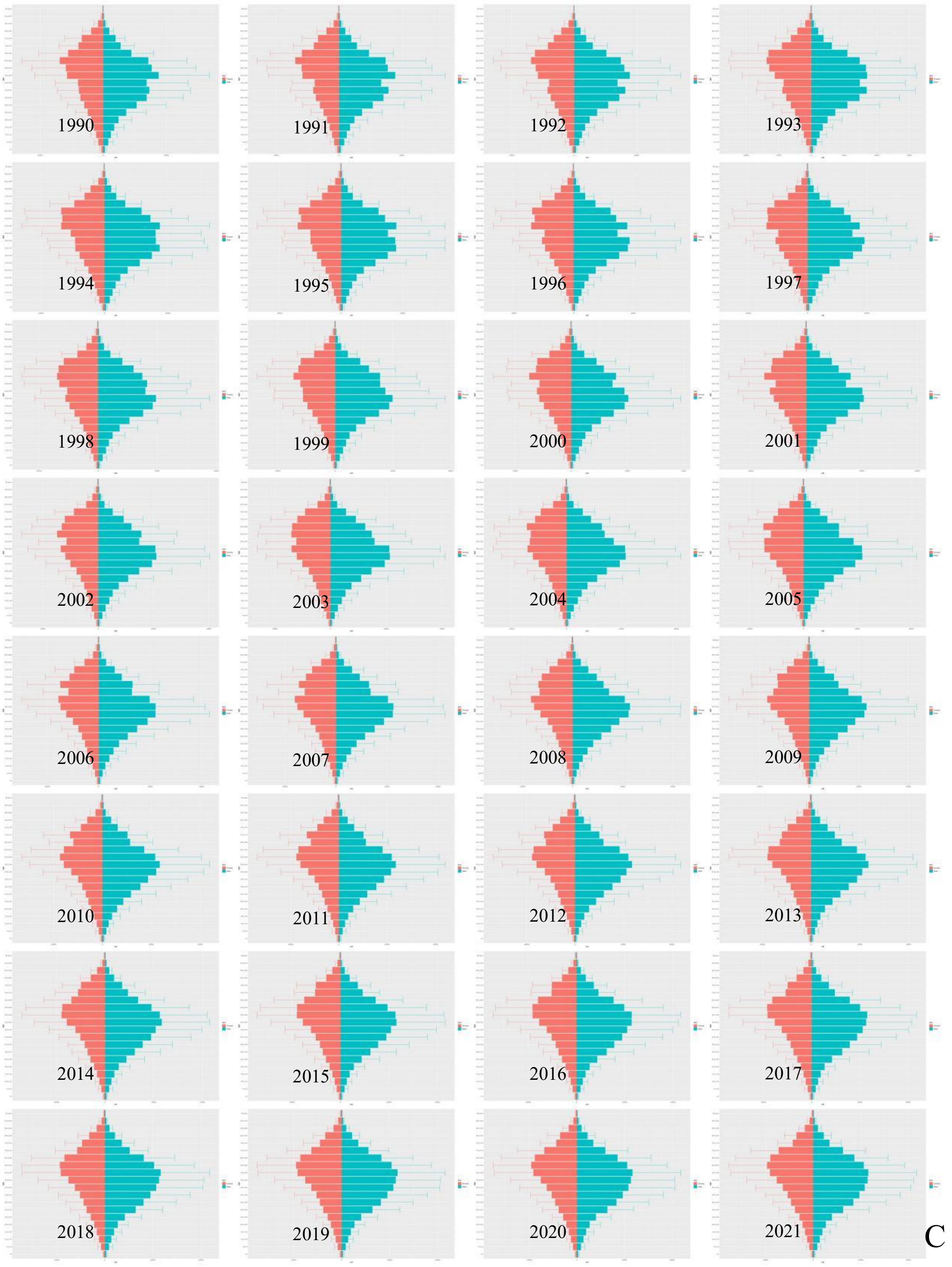
1990
1991
1992
1993
1994
1995
1996
1997
1998
1999
2000
2001
2002
2003
2004
2005
2006
2007
2008
2009
2010
2011
2012
2013
2014
2015
2016
2017
2018
2019
2020
2021
C

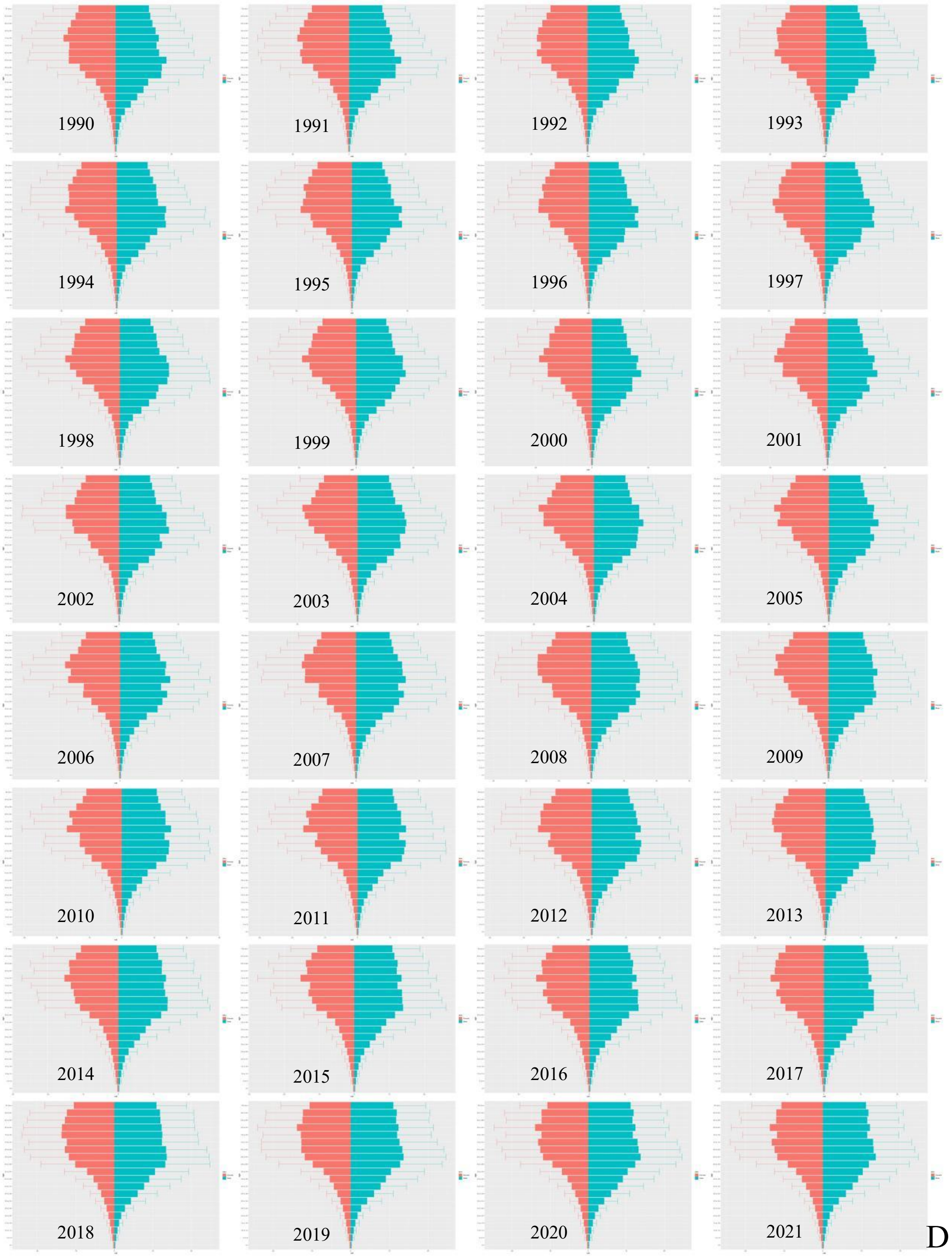

Fig. S49 (A) The prevalence cases of pancreatitis in different ages from 1990 to 2021 in the worldwide; (B) The prevalence rates of pancreatitis in different ages from 1990 to 2021 in the worldwide; (C) The years lived with disability of pancreatitis in different ages from 1990 to 2021 in the worldwide; (D) The years lived with disability rates of pancreatitis in different ages from 1990 to 2021 in the worldwide.

Notes: red for female, green for male; the ordinate from bottom to top is "<5", "5 to 9", "10 to 14", "15 to 19", "20 to 24", "25 to 29", "30 to 34", "35 to 39", "40 to 44", "45 to 49", "50 to 54", "55 to 59", "60 to 64", "65 to 69", "70 to 74", "75 to 79", "80 to 84", "85 to 89", "90 to 94", "95 plus".

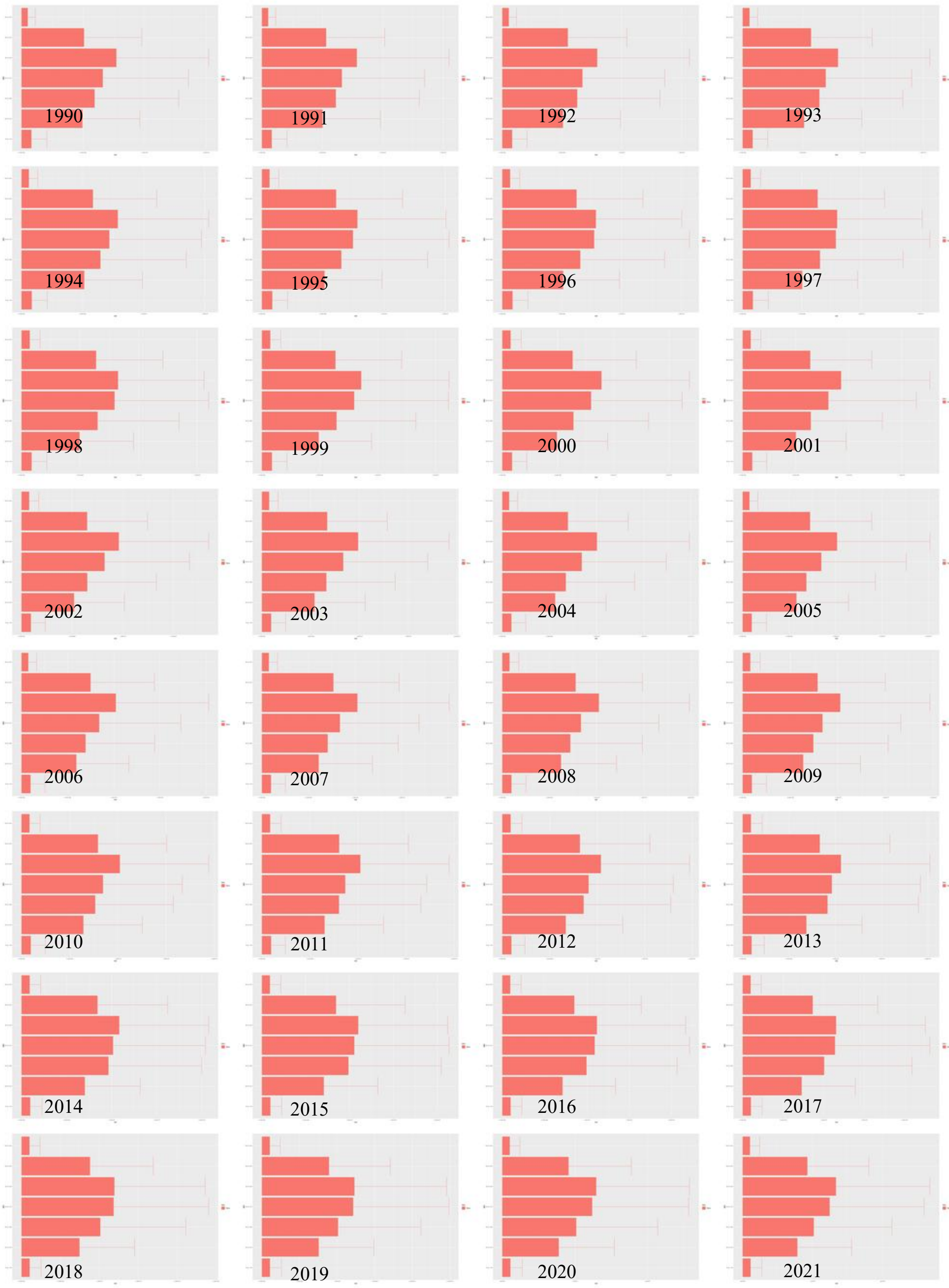
1990
1991
1992
1993
1994
1995
1996
1997
1998
1999
2000
2001
2002
2003
2004
2005
2006
2007
2008
2009
2010
2011
2012
2013
2014
2015
2016
2017
2018
2019
2020
2021

A

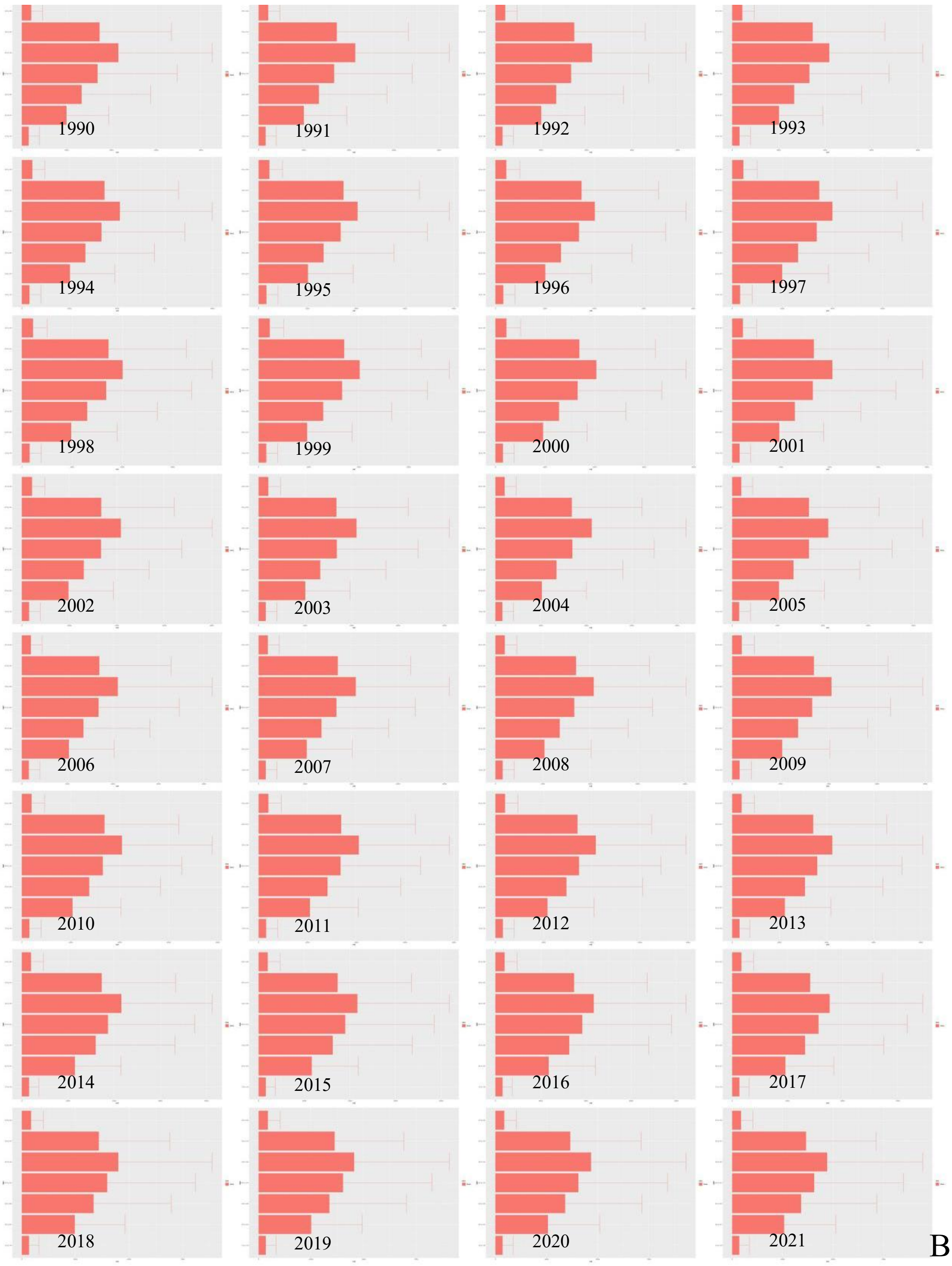
1990
1991
1992
1993
1994
1995
1996
1997
1998
1999
2000
2001
2002
2003
2004
2005
2006
2007
2008
2009
2010
2011
2012
2013
2014
2015
2016
2017
2018
2019
2020
2021
B

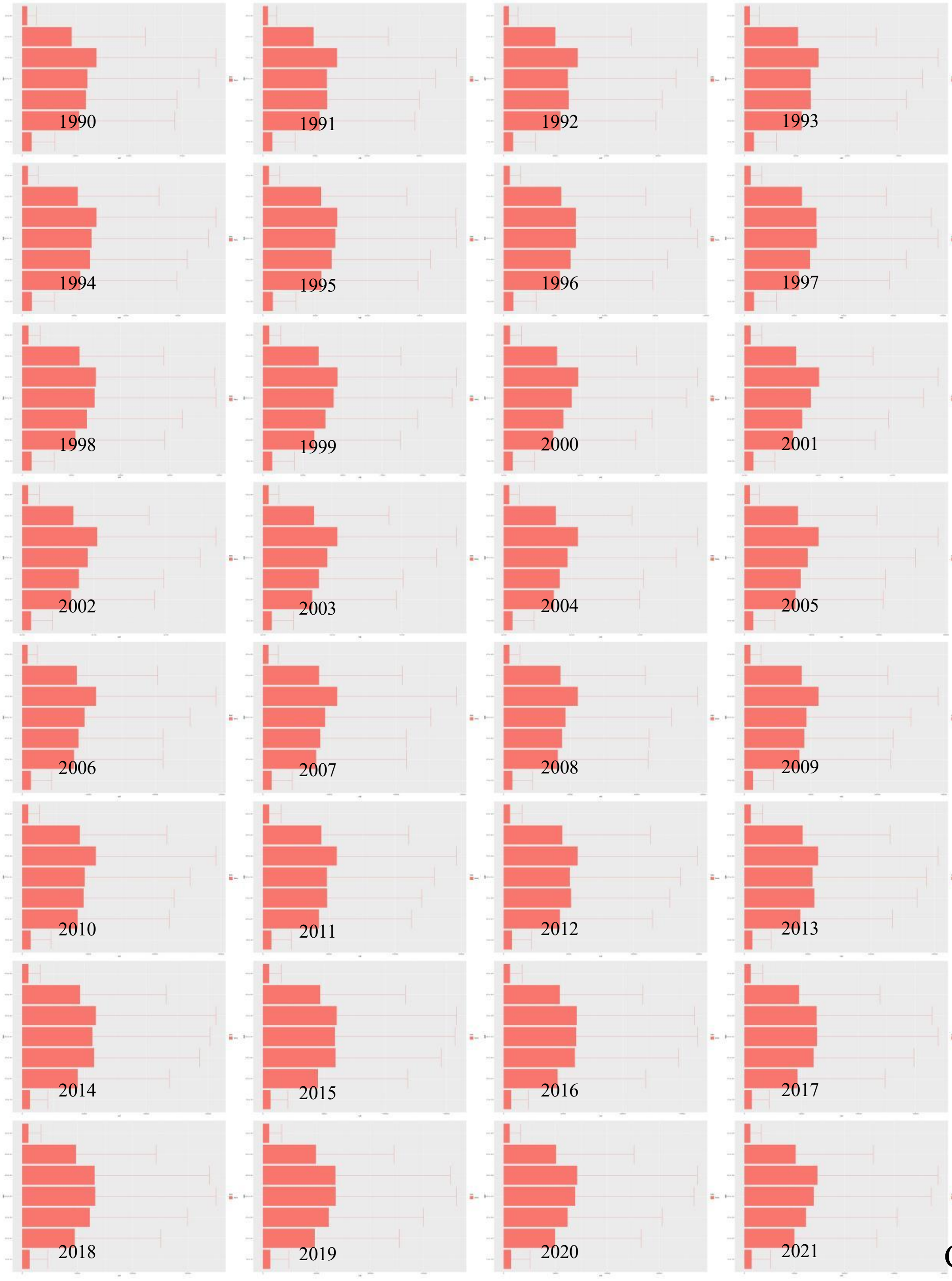
1990
1991
1992
1993
1994
1995
1996
1997
1998
1999
2000
2001
2002
2003
2004
2005
2006
2007
2008
2009
2010
2011
2012
2013
2014
2015
2016
2017
2018
2019
2020
2021

C

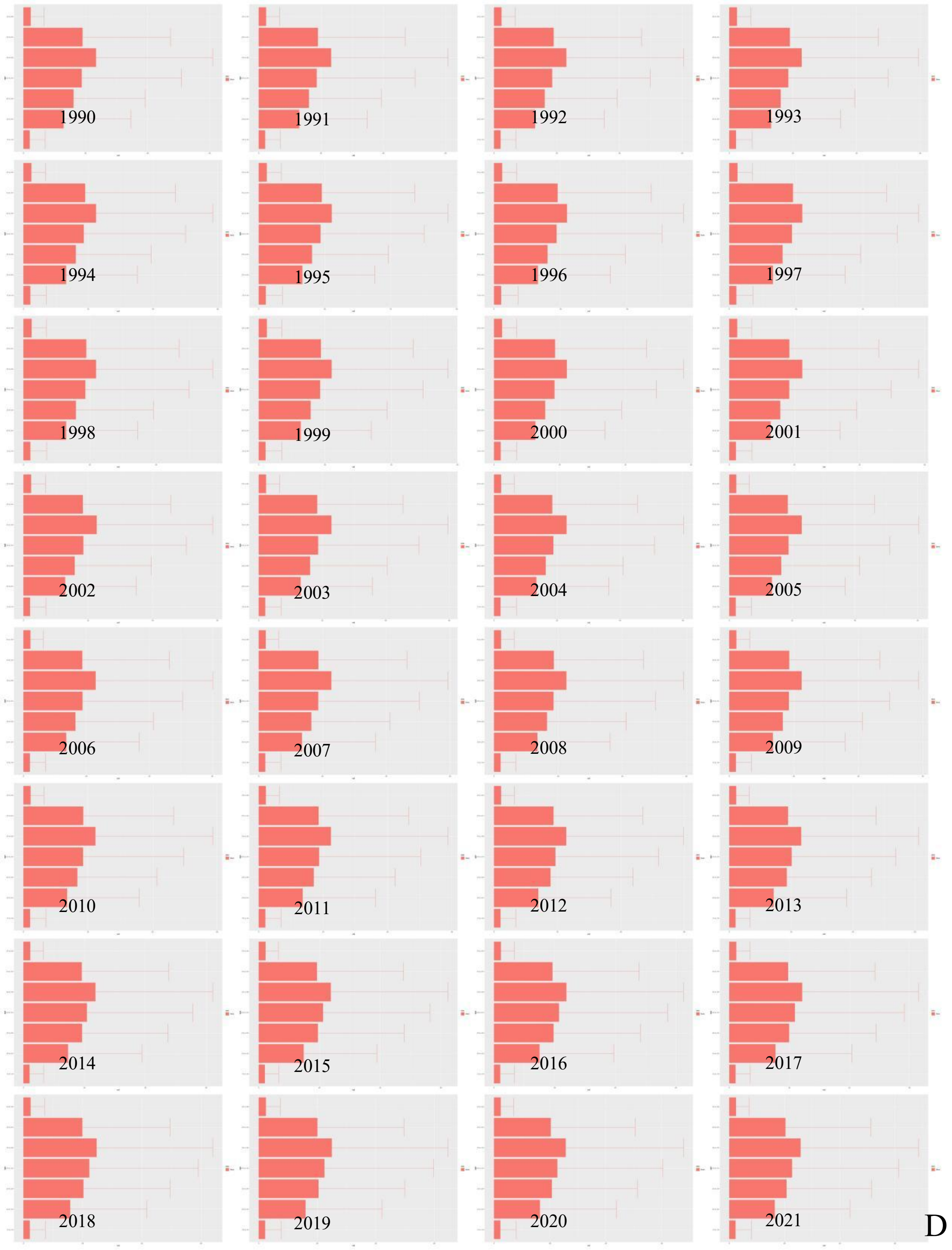

Fig. S50 (A) The prevalence cases of male infertility in different ages from 1990 to 2021 in the worldwide; (B) The prevalence rates of male infertility in different ages from 1990 to 2021 in the worldwide; (C) The years lived with disability of male infertility in different ages from 1990 to 2021 in the worldwide; (D) The years lived with disability rates of male infertility in different ages from 1990 to 2021 in the worldwide.

Notes: red for male; the ordinate from bottom to top is "15 to 19", "20 to 24", "25 to 29", "30 to 34", "35 to 39", "40 to 44", "45 to 49".

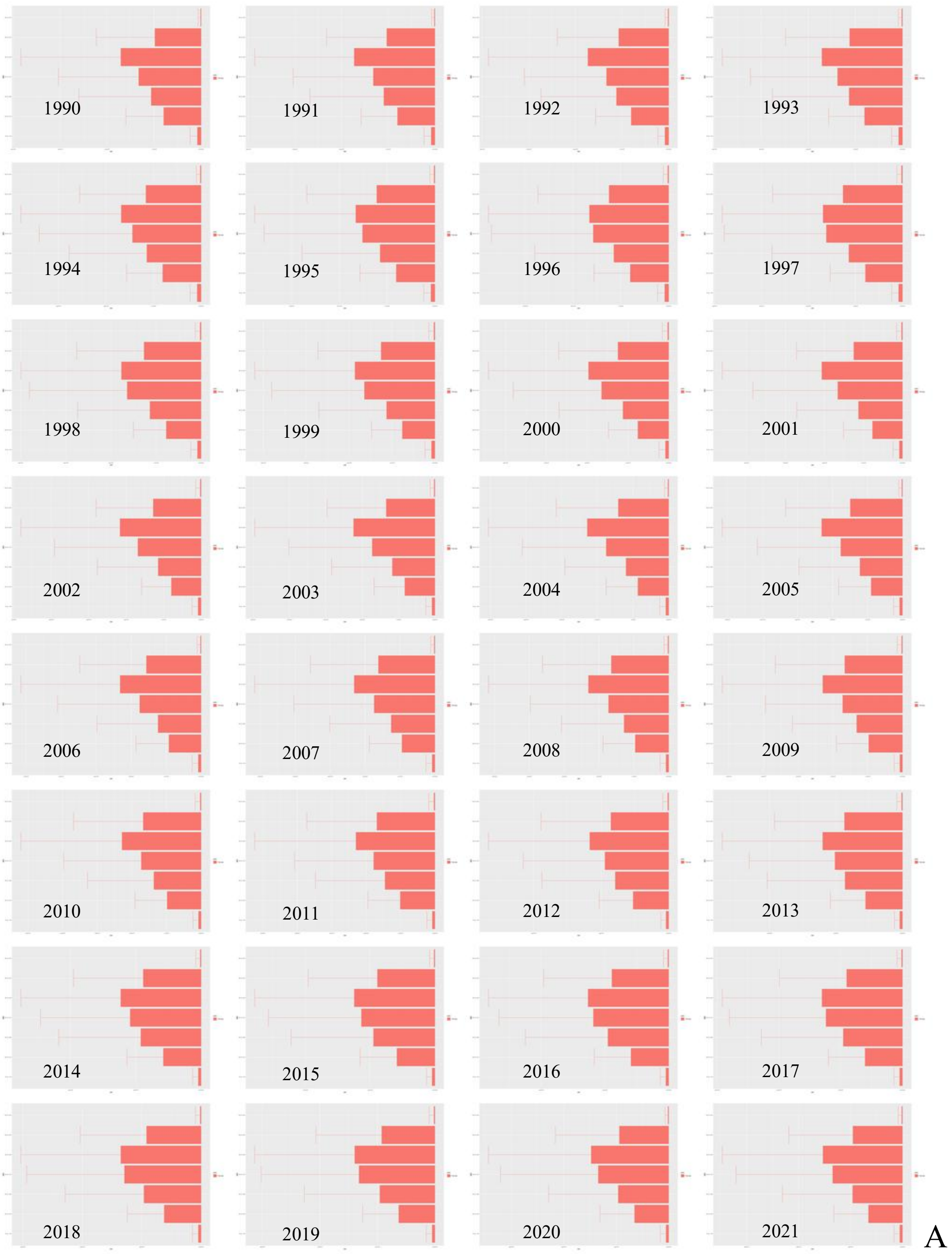
1990
1991
1992
1993
1994
1995
1996
1997
1998
1999
2000
2001
2002
2003
2004
2005
2006
2007
2008
2009
2010
2011
2012
2013
2014
2015
2016
2017
2018
2019
2020
2021
A

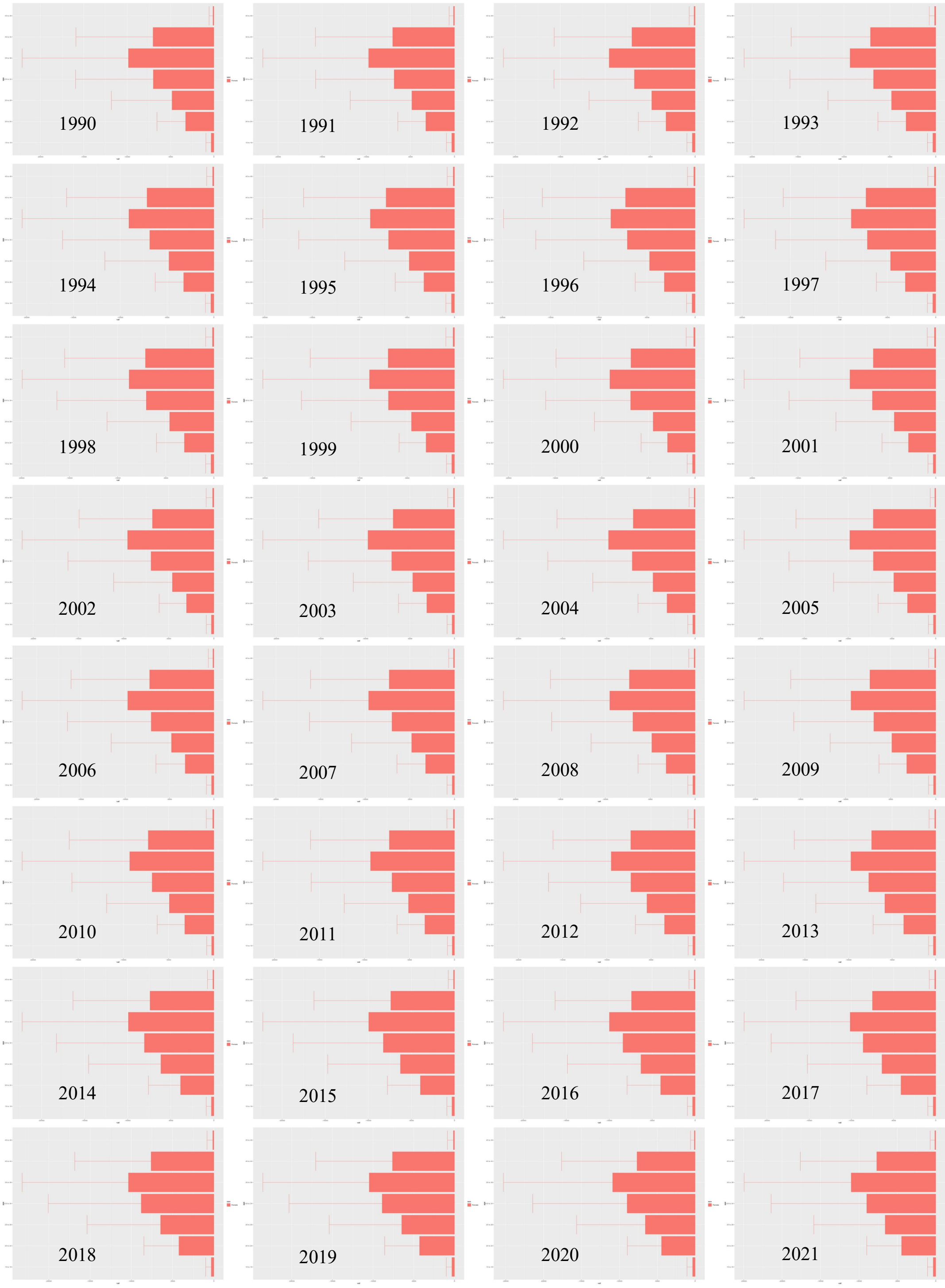
1990
1991
1992
1993
1994
1995
1996
1997
1998
1999
2000
2001
2002
2003
2004
2005
2006
2007
2008
2009
2010
2011
2012
2013
2014
2015
2016
2017
2018
2019
2020
2021

B

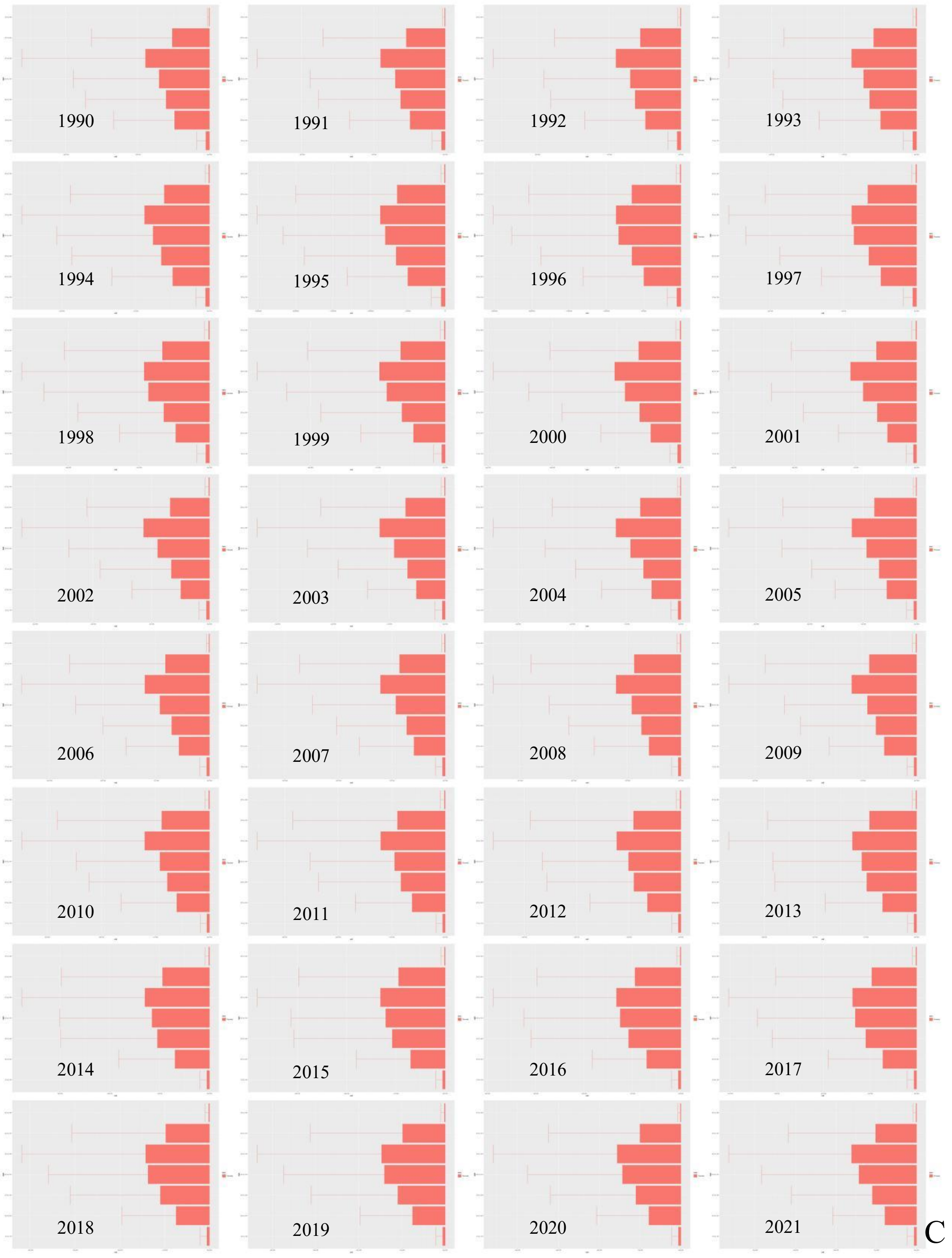
1990
1991
1992
1993
1994
1995
1996
1997
1998
1999
2000
2001
2002
2003
2004
2005
2006
2007
2008
2009
2010
2011
2012
2013
2014
2015
2016
2017
2018
2019
2020
2021
C

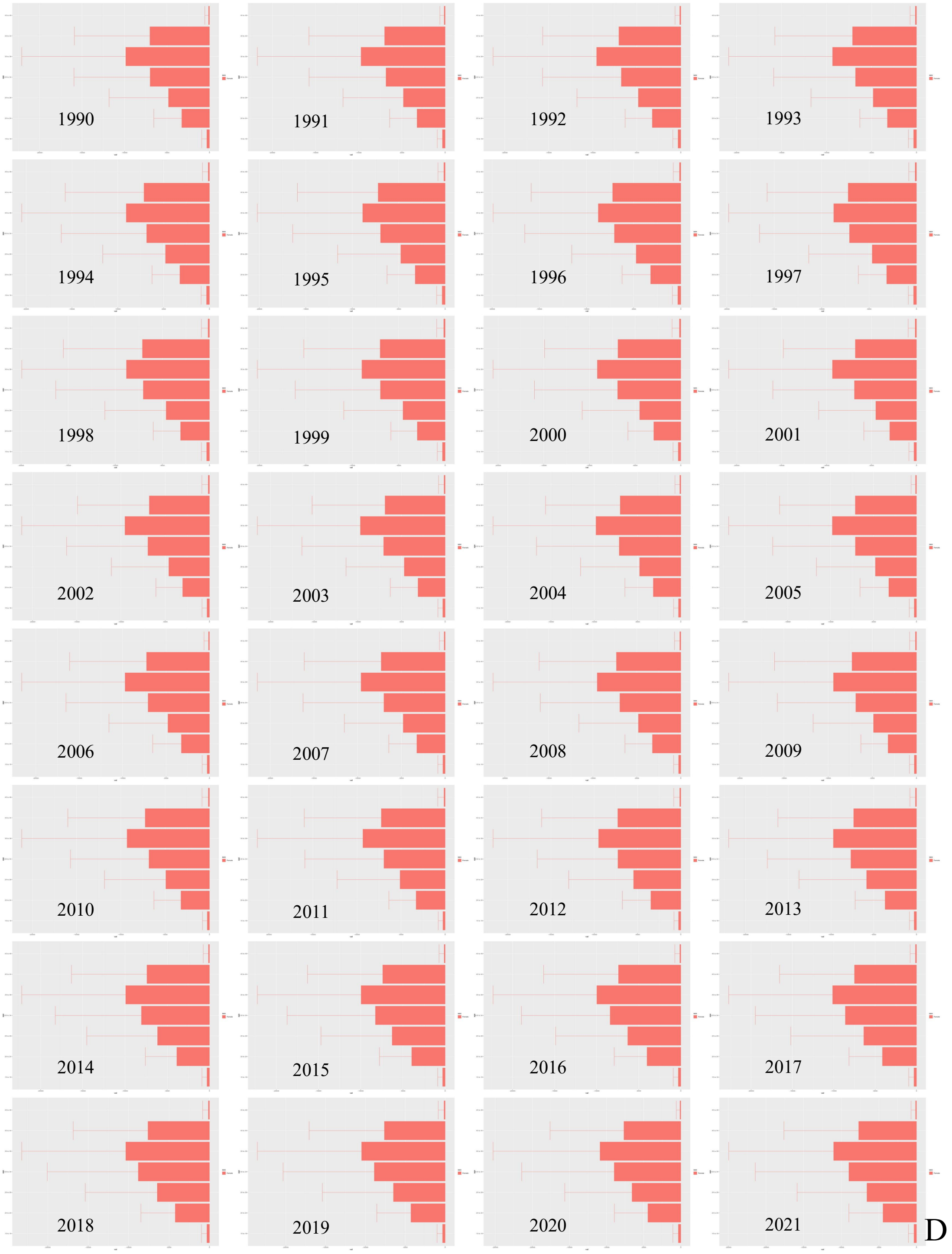

Fig. S51 (A) The prevalence cases of female infertility in different ages from 1990 to 2021 in the worldwide; (B) The prevalence rates of female infertility in different ages from 1990 to 2021 in the worldwide; (C) The years lived with disability of female infertility in different ages from 1990 to 2021 in the worldwide; (D) The years lived with disability rates of female infertility in different ages from 1990 to 2021 in the worldwide.

Notes: red for female; the ordinate from bottom to top is "<5", "5 to 9", "10 to 14", "15 to 19", "20 to 24", "25 to 29", "30 to 34", "35 to 39", "40 to 44", "45 to 49", "50 to 54", "55 to 59", "60 to 64", "65 to 69", "70 to 74", "75 to 79", "80 to 84", "85 to 89", "90 to 94", "95 plus".

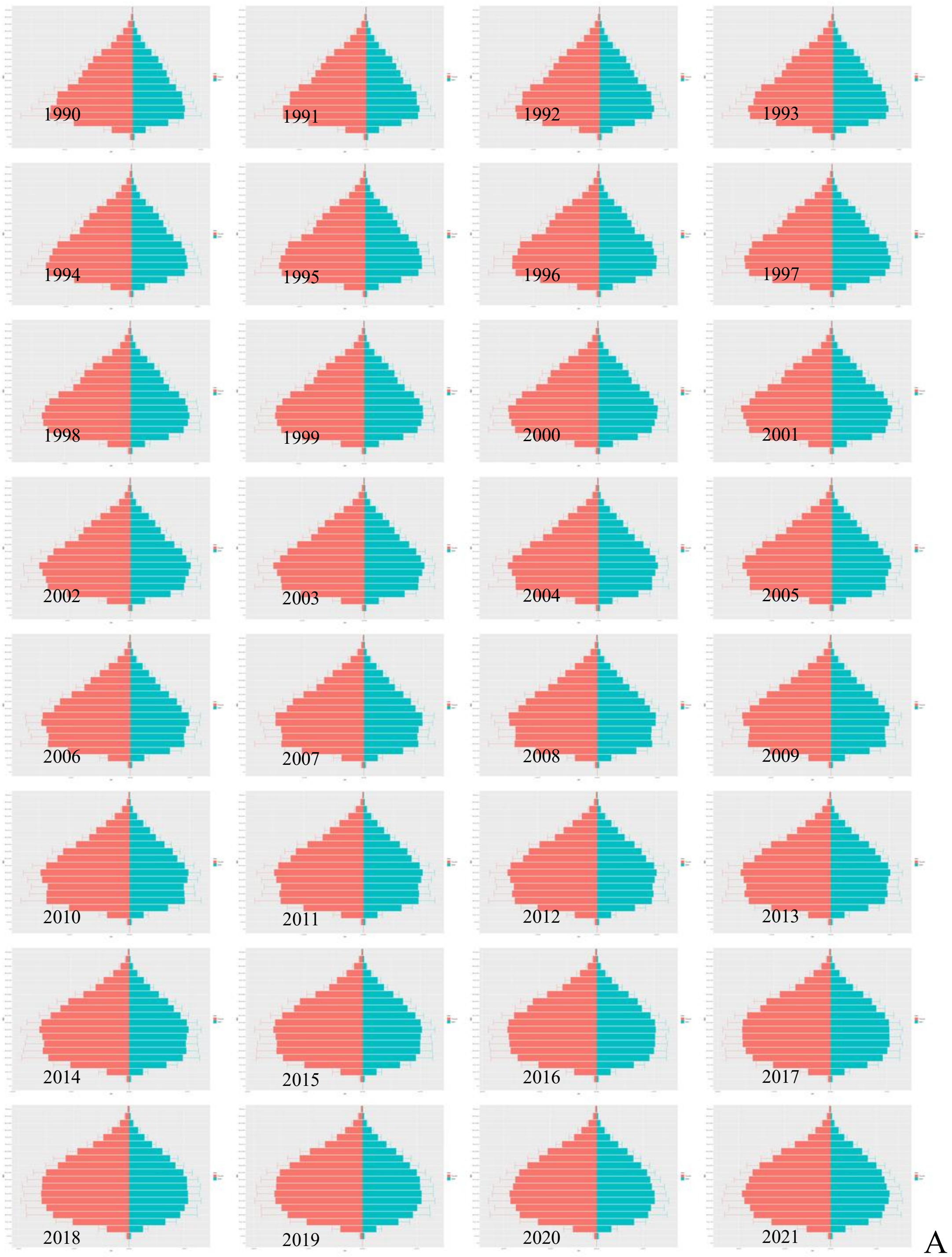
1990
1991
1992
1993
1994
1995
1996
1997
1998
1999
2000
2001
2002
2003
2004
2005
2006
2007
2008
2009
2010
2011
2012
2013
2014
2015
2016
2017
2018
2019
2020
2021
A

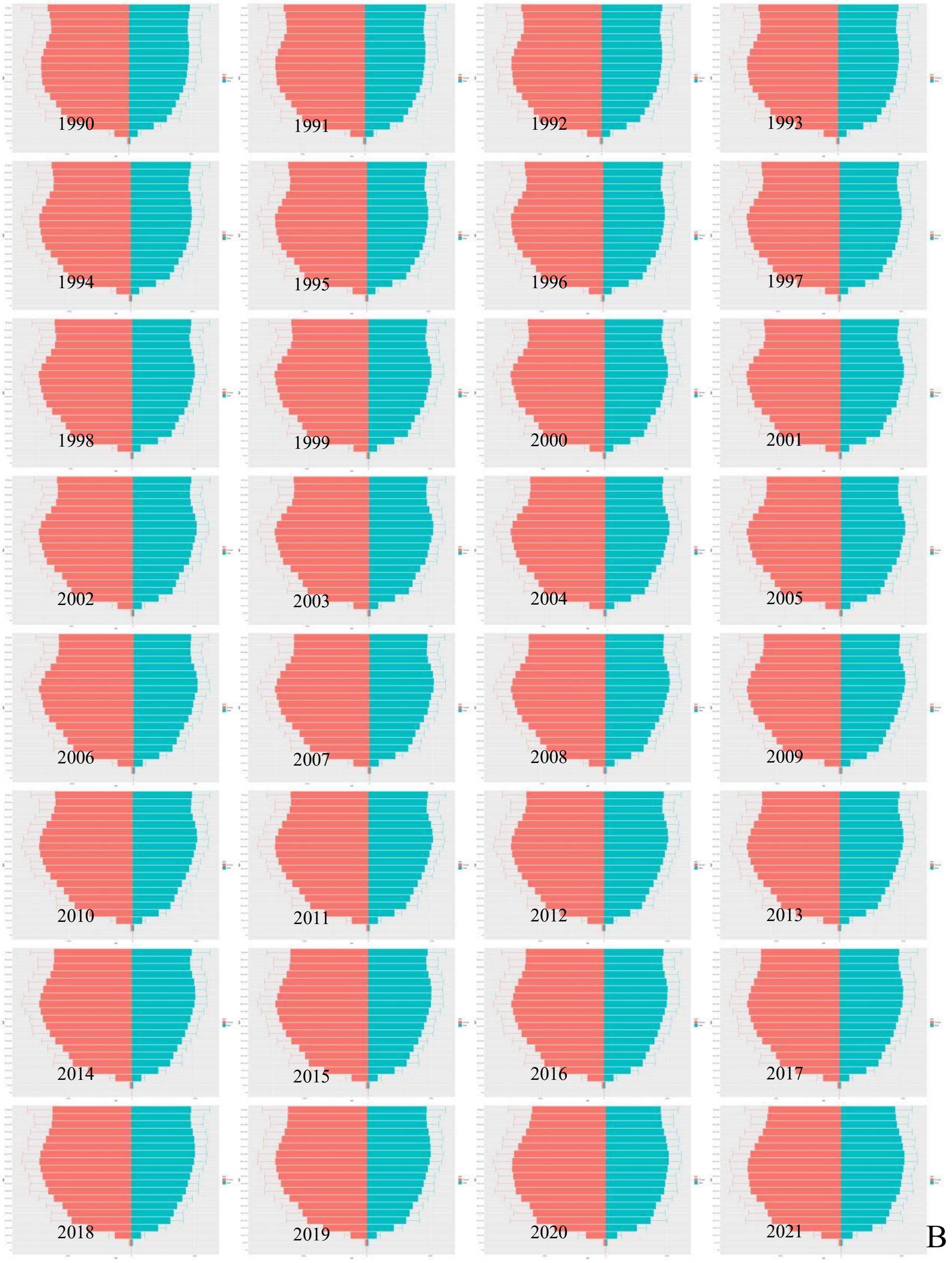
1990
1991
1992
1993
1994
1995
1996
1997
1998
1999
2000
2001
2002
2003
2004
2005
2006
2007
2008
2009
2010
2011
2012
2013
2014
2015
2016
2017
2018
2019
2020
2021
B

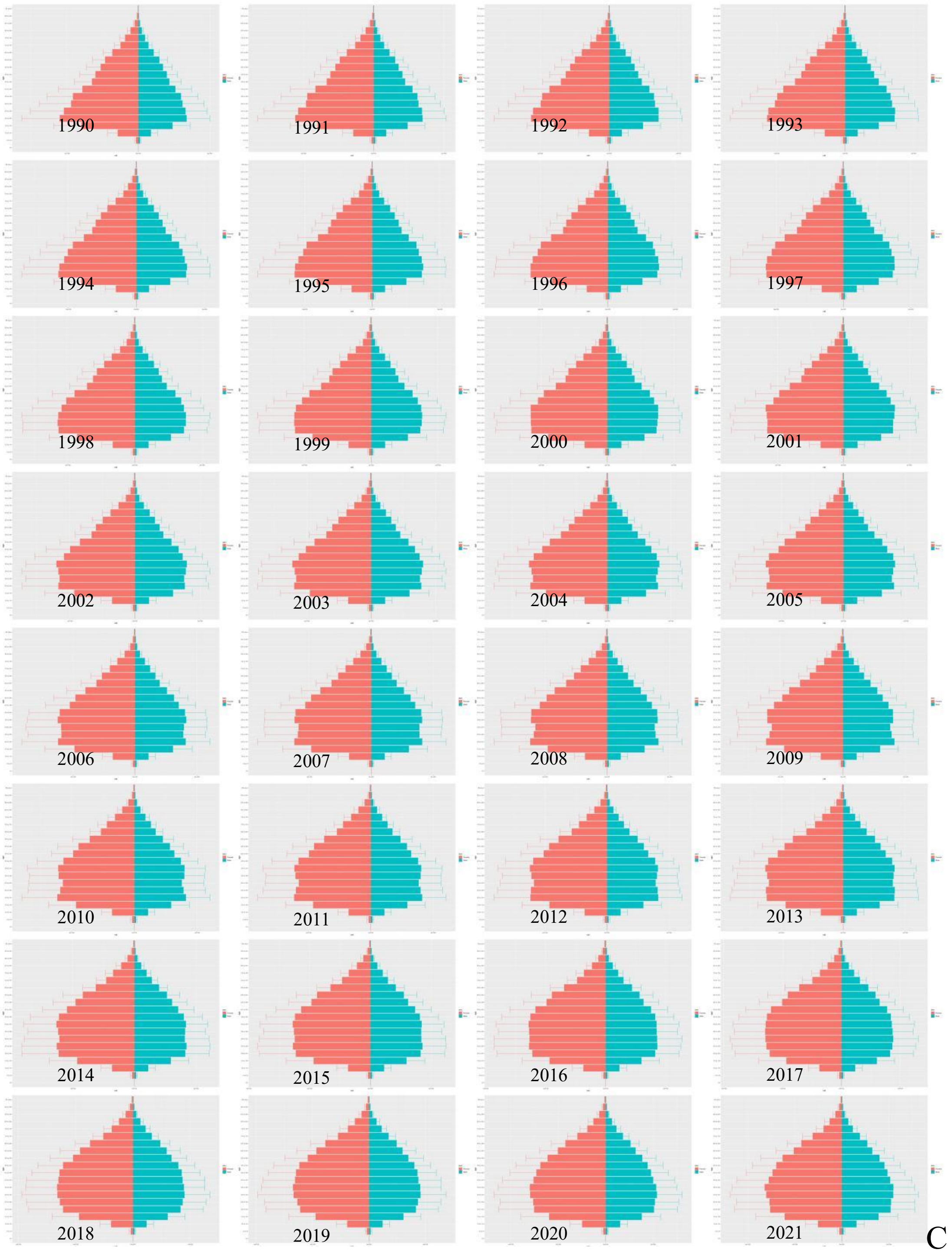
1990
1991
1992
1993
1994
1995
1996
1997
1998
1999
2000
2001
2002
2003
2004
2005
2006
2007
2008
2009
2010
2011
2012
2013
2014
2015
2016
2017
2018
2019
2020
2021
C

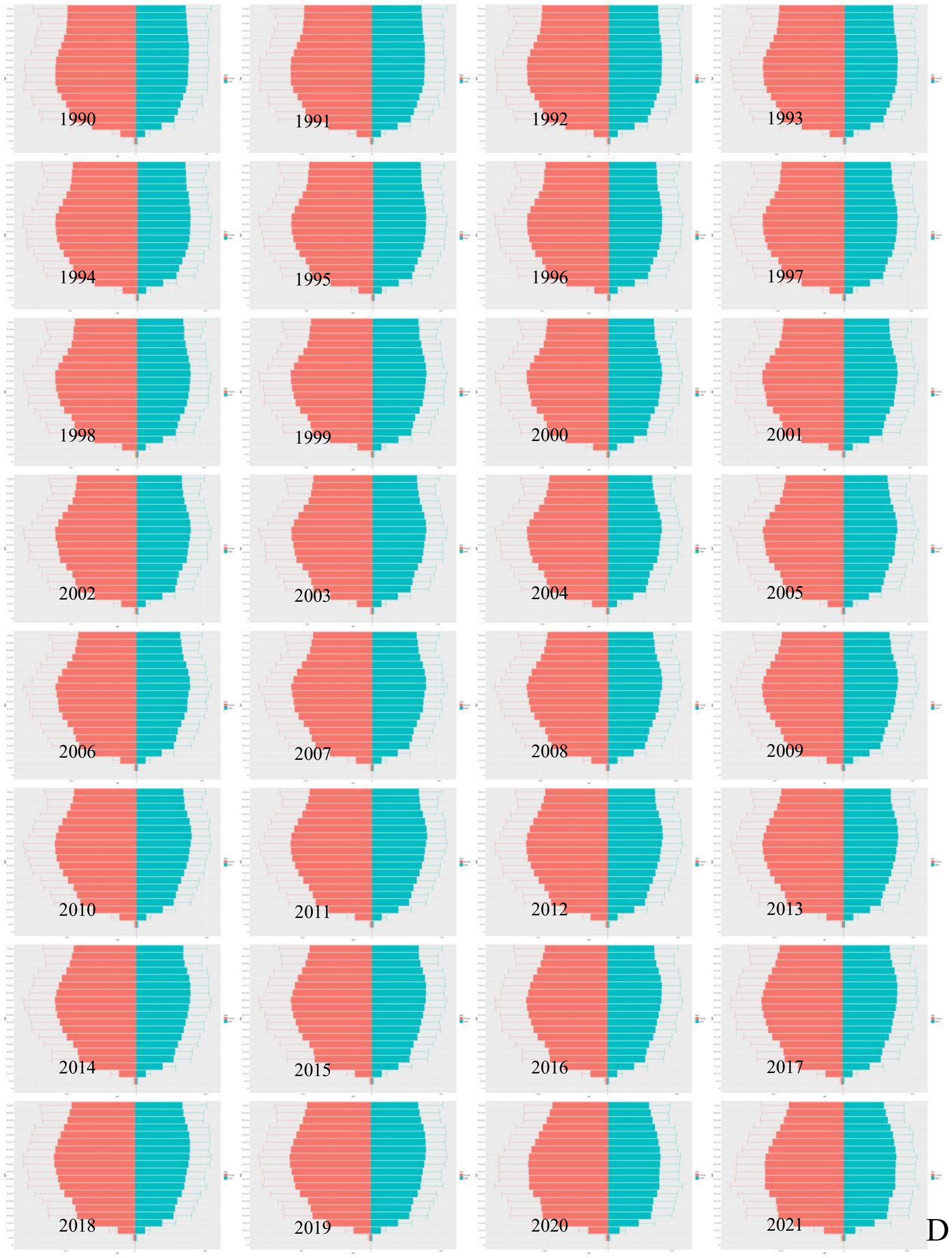

Fig. S52 (A) The prevalence cases of depressive disorders in different ages from 1990 to 2021 in the worldwide; (B) The prevalence rates of depressive disorders in different ages from 1990 to 2021 in the worldwide; (C) The years lived with disability of depressive disorders in different ages from 1990 to 2021 in the worldwide; (D) The years lived with disability rates of depressive disorders in different ages from 1990 to 2021 in the worldwide.

Notes: red for female, green for male; the ordinate from bottom to top is "<5", "5 to 9", "10 to 14", "15 to 19", "20 to 24", "25 to 29", "30 to 34", "35 to 39", "40 to 44", "45 to 49", "50 to 54", "55 to 59", "60 to 64", "65 to 69", "70 to 74", "75 to 79", "80 to 84", "85 to 89", "90 to 94", "95 plus".

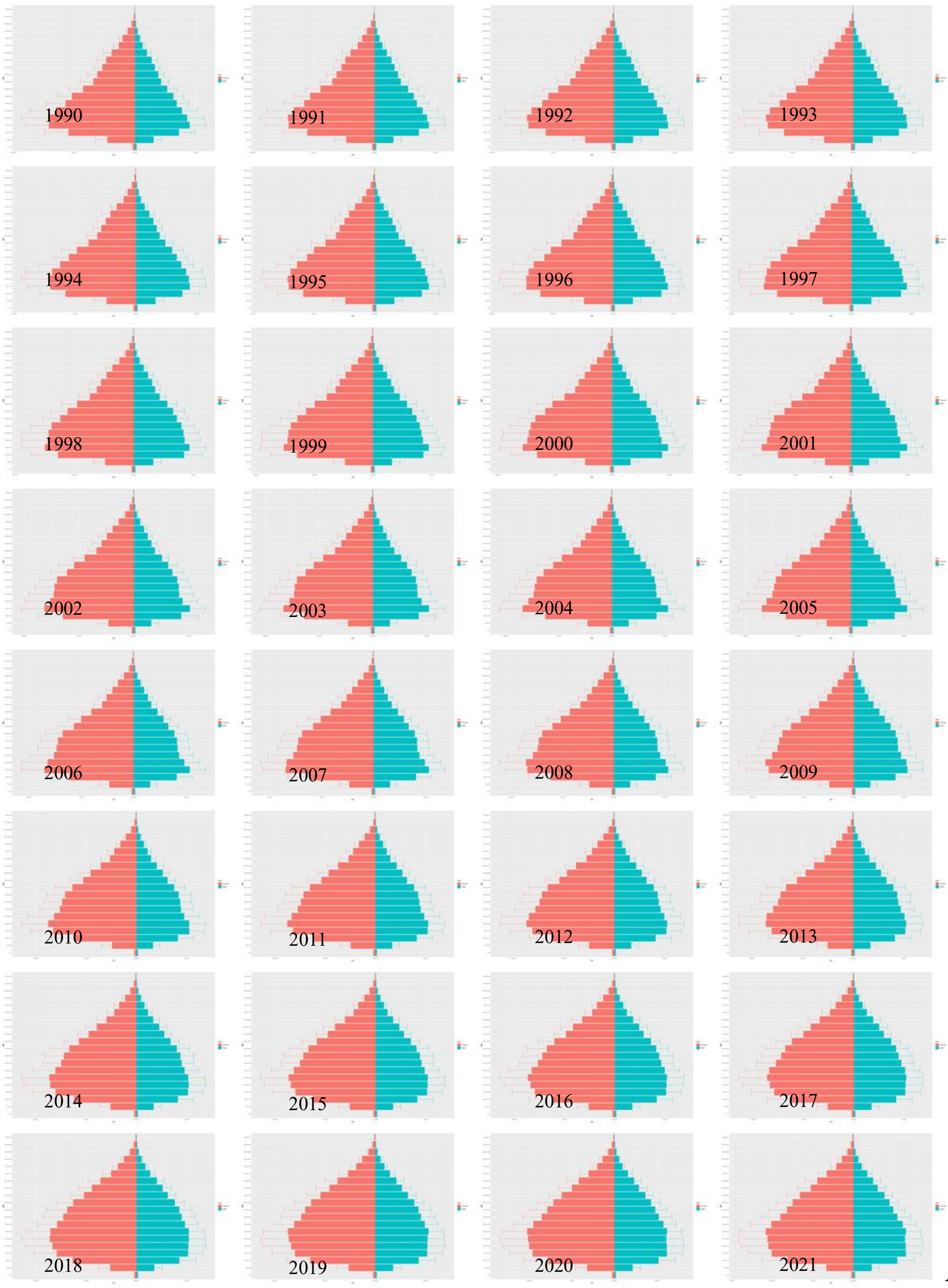
1990
1991
1992
1993
1994
1995
1996
1997
1998
1999
2000
2001
2002
2003
2004
2005
2006
2007
2008
2009
2010
2011
2012
2013
2014
2015
2016
2017
2018
2019
2020
2021
A

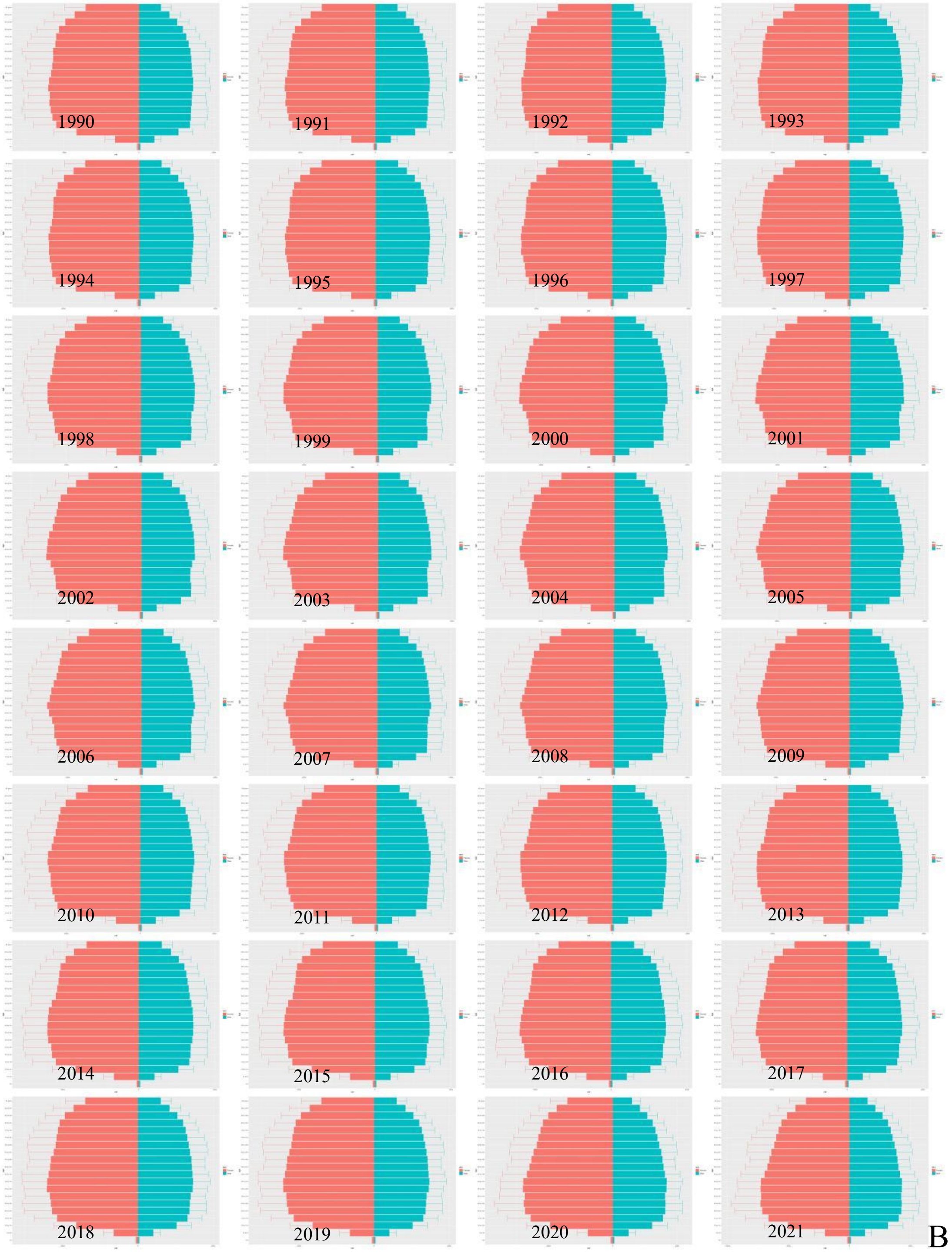
1990
1991
1992
1993
1994
1995
1996
1997
1998
1999
2000
2001
2002
2003
2004
2005
2006
2007
2008
2009
2010
2011
2012
2013
2014
2015
2016
2017
2018
2019
2020
2021
B

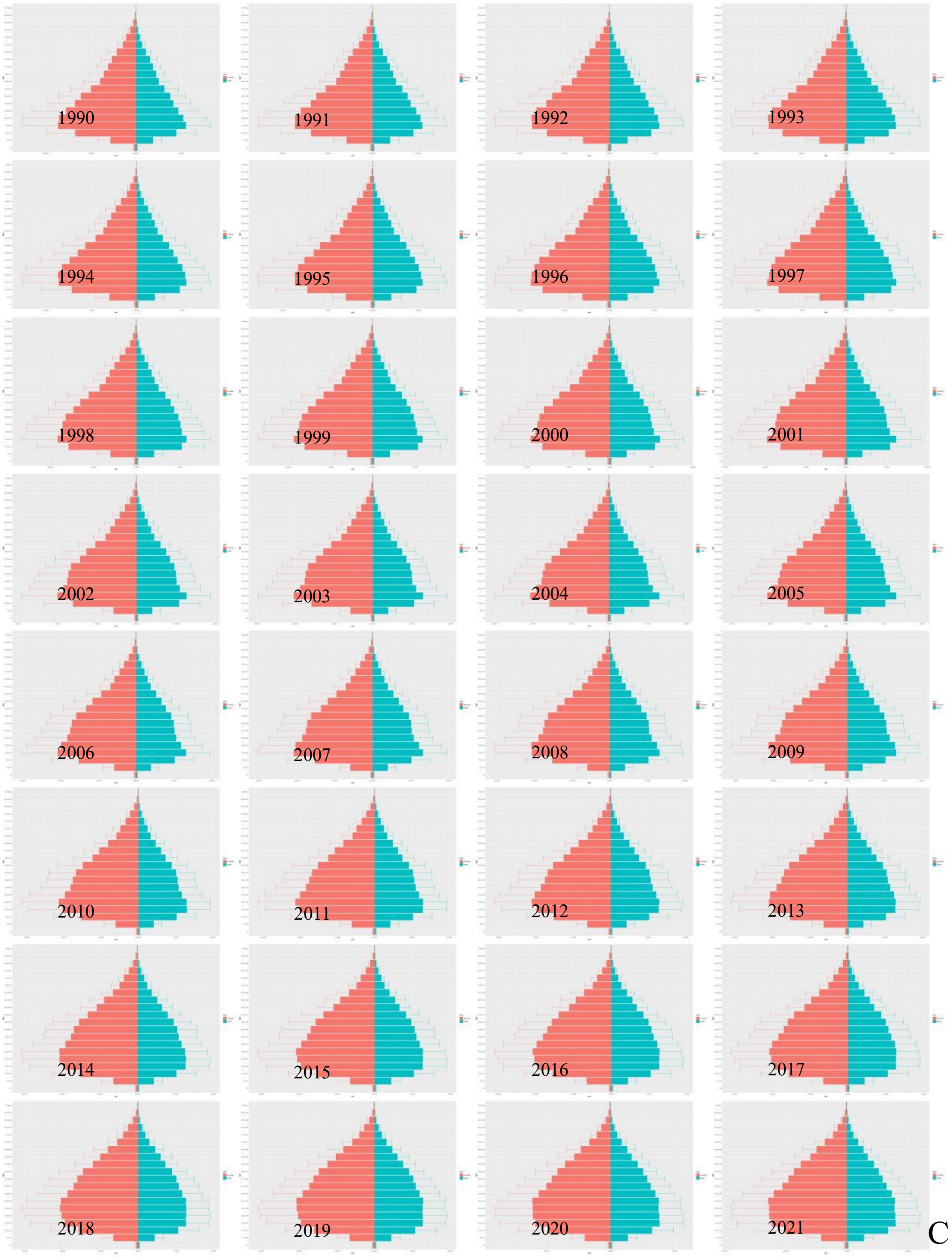
1990
1991
1992
1993
1994
1995
1996
1997
1998
1999
2000
2001
2002
2003
2004
2005
2006
2007
2008
2009
2010
2011
2012
2013
2014
2015
2016
2017
2018
2019
2020
2021
C

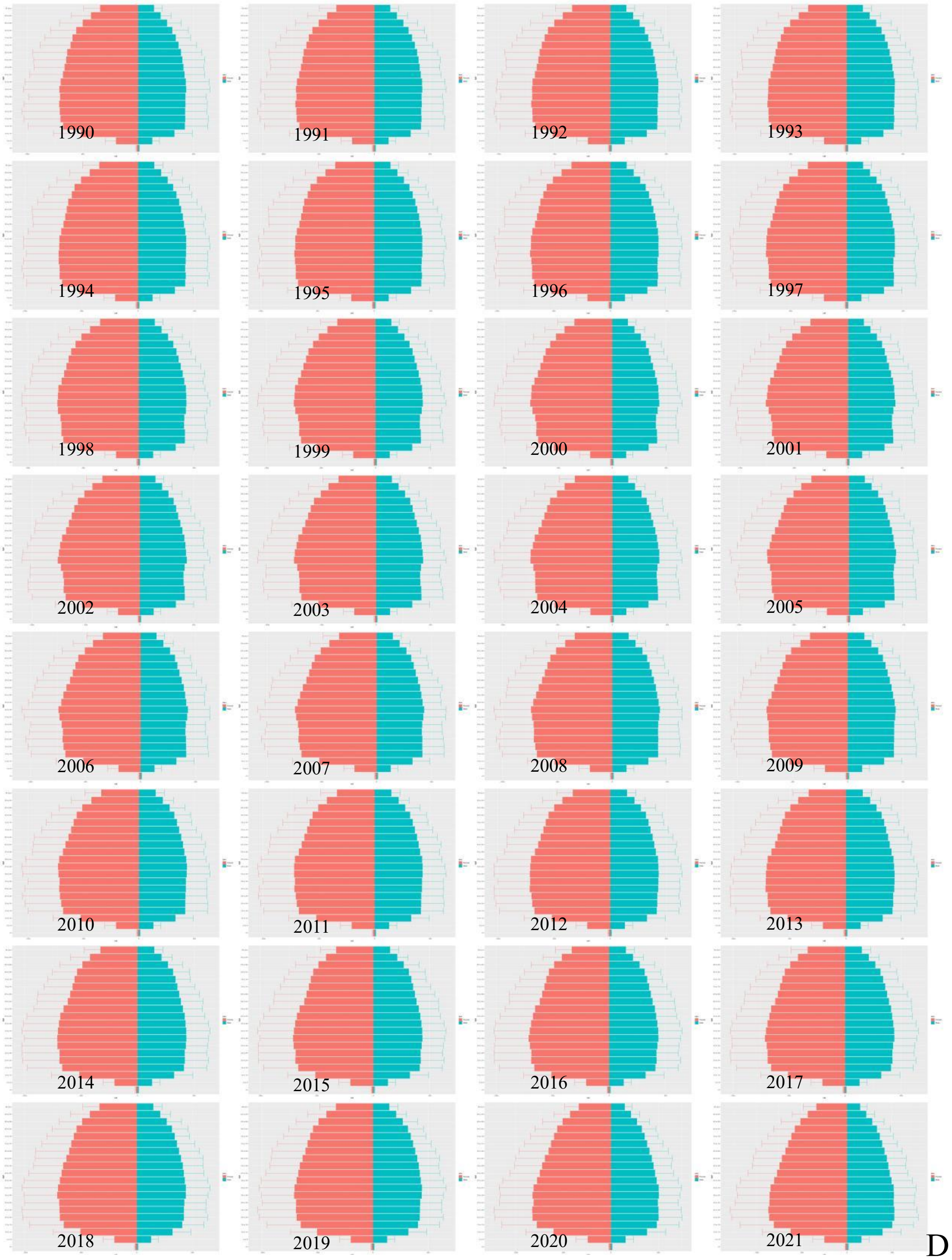

Fig. S53 (A) The prevalence cases of anxiety disorders in different ages from 1990 to 2021 in the worldwide; (B) The prevalence rates of anxiety disorders in different ages from 1990 to 2021 in the worldwide; (C) The years lived with disability of anxiety disorders in different ages from 1990 to 2021 in the worldwide; (D) The years lived with disability rates of anxiety disorders in different ages from 1990 to 2021 in the worldwide.

Notes: red for female, green for male; the ordinate from bottom to top is "<5", "5 to 9", "10 to 14", "15 to 19", "20 to 24", "25 to 29", "30 to 34", "35 to 39", "40 to 44", "45 to 49", "50 to 54", "55 to 59", "60 to 64", "65 to 69", "70 to 74", "75 to 79", "80 to 84", "85 to 89", "90 to 94", "95 plus".

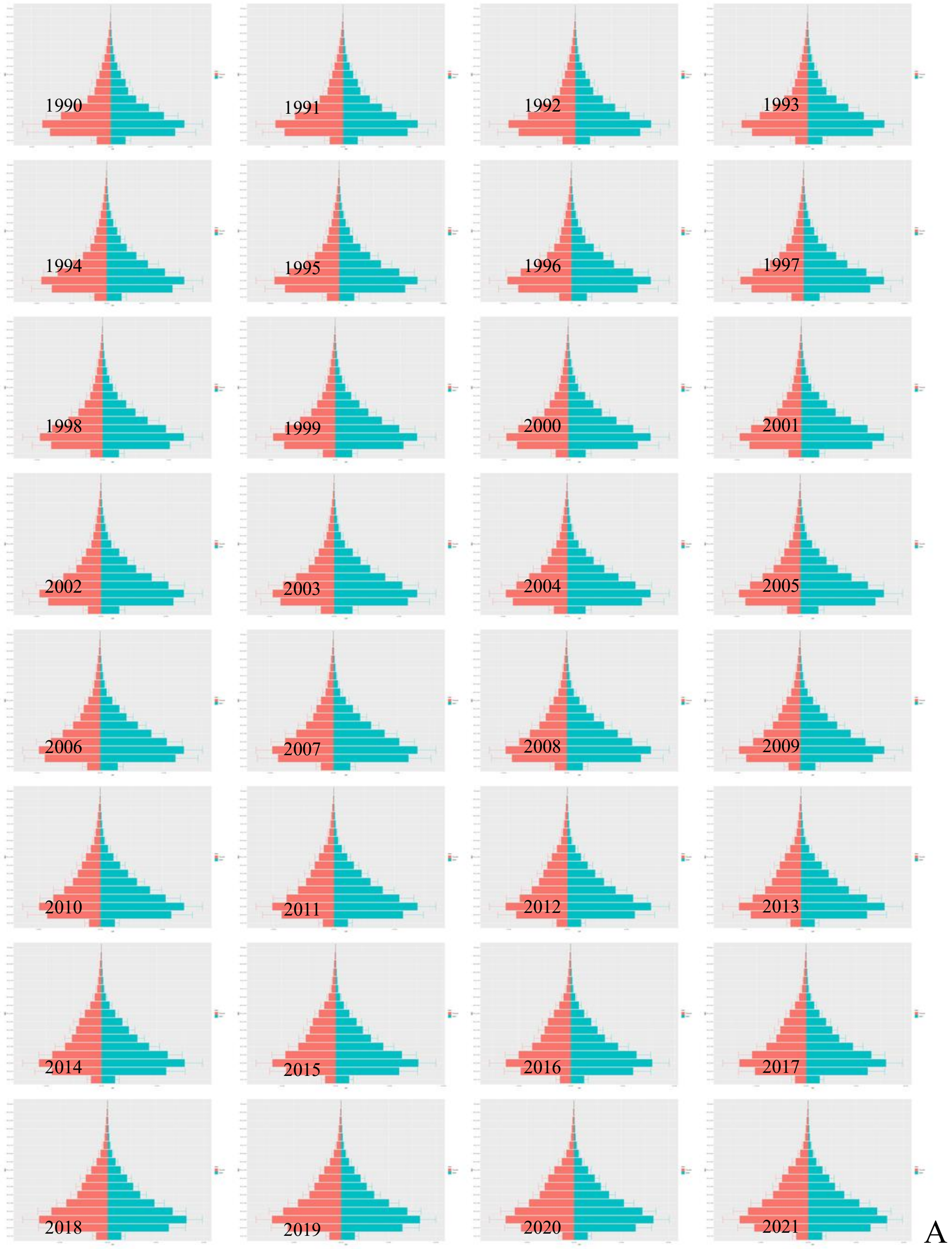
1990
1991
1992
1993
1994
1995
1996
1997
1998
1999
2000
2001
2002
2003
2004
2005
2006
2007
2008
2009
2010
2011
2012
2013
2014
2015
2016
2017
2018
2019
2020
2021
A

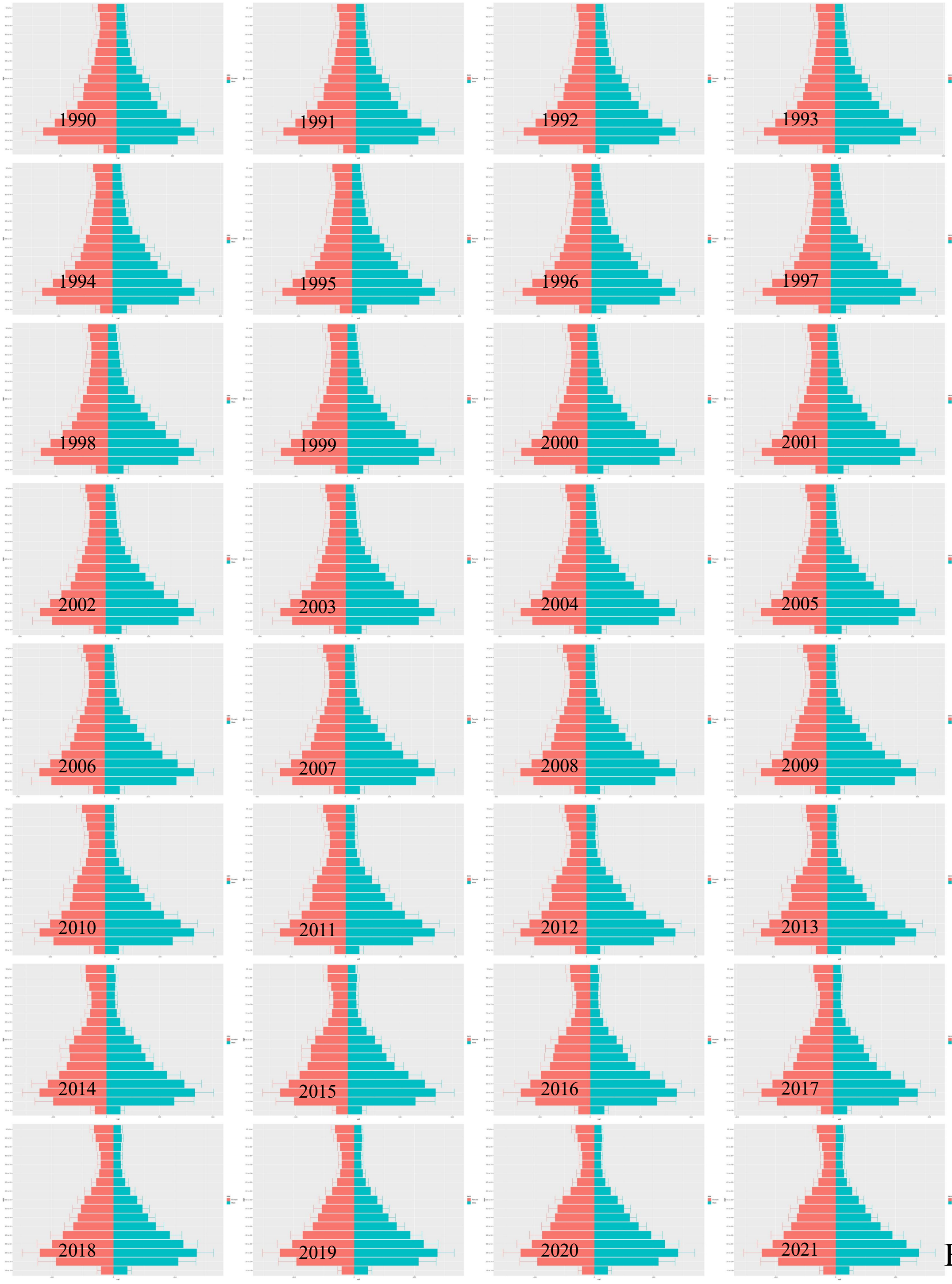
1990
1991
1992
1993
1994
1995
1996
1997
1998
1999
2000
2001
2002
2003
2004
2005
2006
2007
2008
2009
2010
2011
2012
2013
2014
2015
2016
2017
2018
2019
2020
2021

B

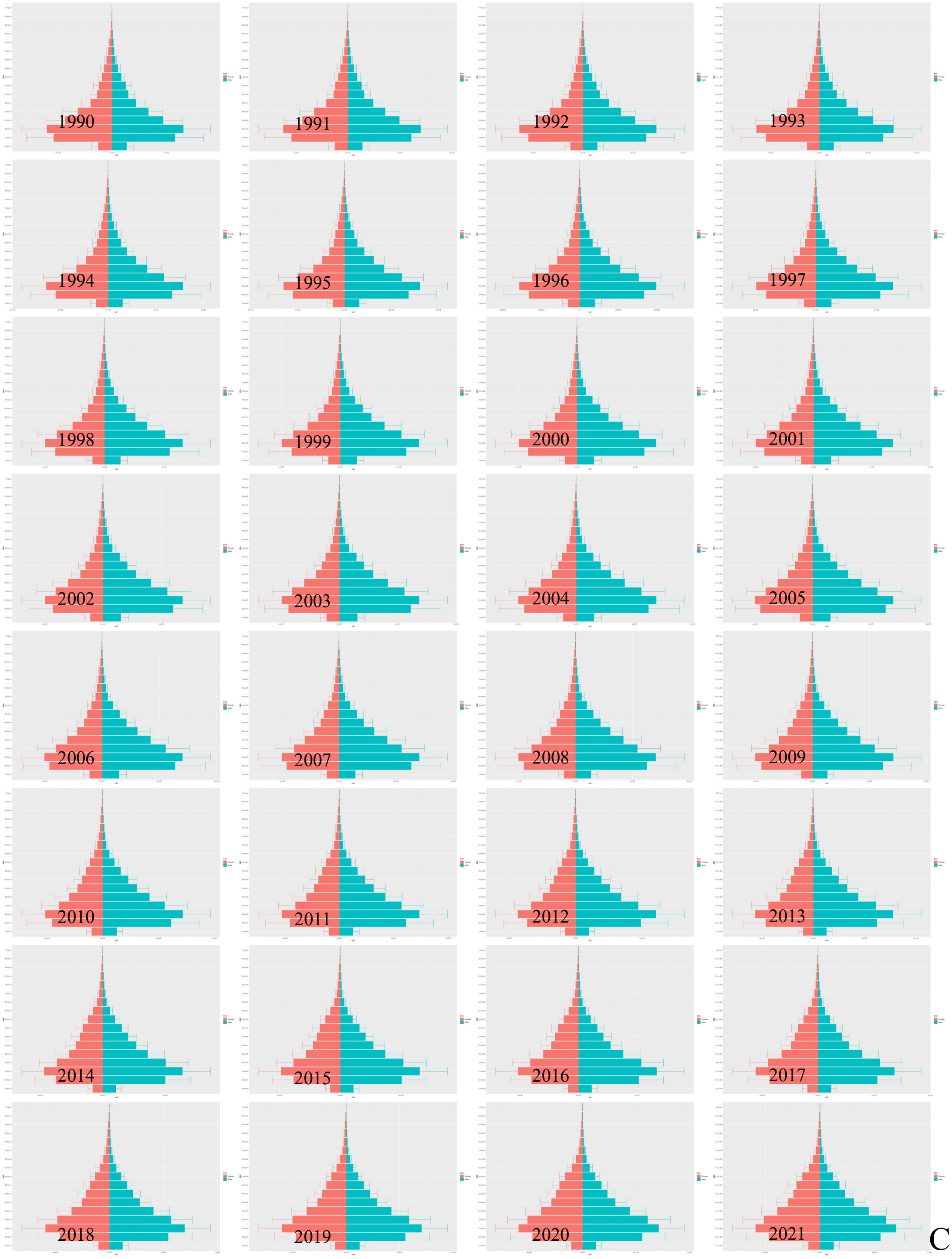
1990
1991
1992
1993
1994
1995
1996
1997
1998
1999
2000
2001
2002
2003
2004
2005
2006
2007
2008
2009
2010
2011
2012
2013
2014
2015
2016
2017
2018
2019
2020
2021

C

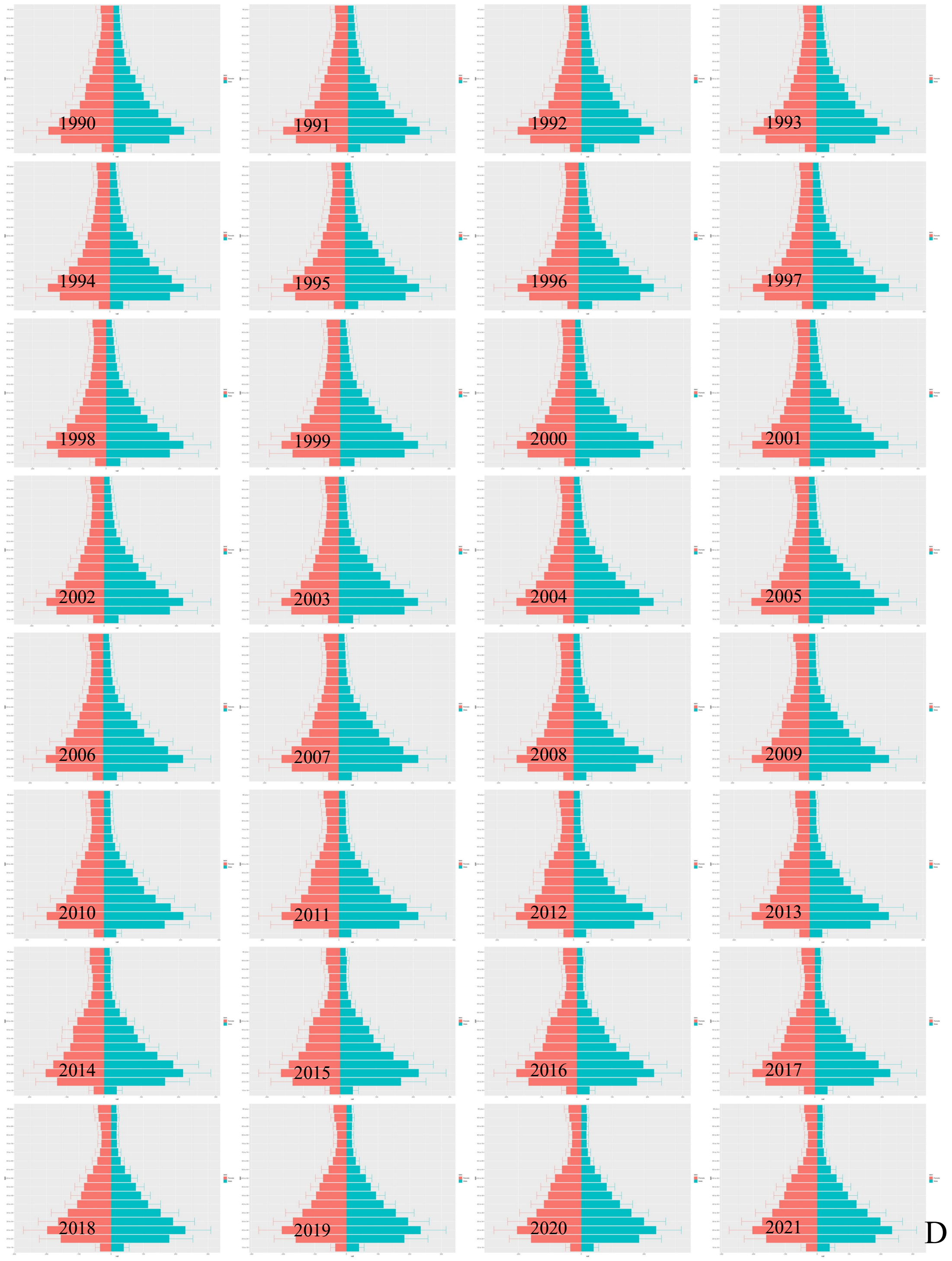

Fig. S54 (A) The prevalence cases of opioid use disorders in different ages from 1990 to 2021 in the worldwide; (B) The prevalence rates of opioid use disorders in different ages from 1990 to 2021 in the worldwide; (C) The years lived with disability of opioid use disorders in different ages from 1990 to 2021 in the worldwide; (D) The years lived with disability rates of opioid use disorders in different ages from 1990 to 2021 in the worldwide.

Notes: red for female, green for male; the ordinate from bottom to top is "<5", "5 to 9", "10 to 14", "15 to 19", "20 to 24", "25 to 29", "30 to 34", "35 to 39", "40 to 44", "45 to 49", "50 to 54", "55 to 59", "60 to 64", "65 to 69", "70 to 74", "75 to 79", "80 to 84", "85 to 89", "90 to 94", "95 plus".

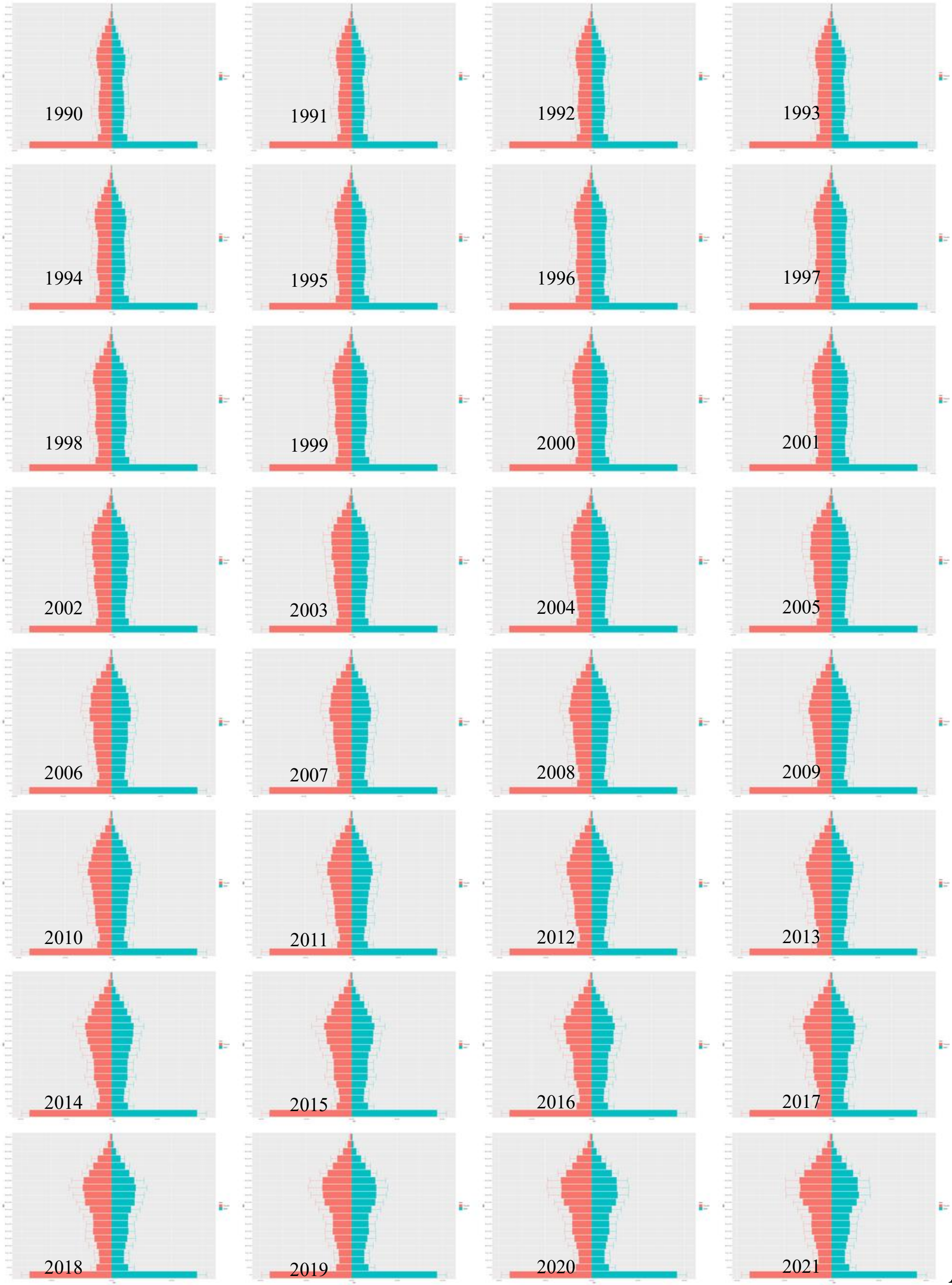
1990
1991
1992
1993
1994
1995
1996
1997
1998
1999
2000
2001
2002
2003
2004
2005
2006
2007
2008
2009
2010
2011
2012
2013
2014
2015
2016
2017
2018
2019
2020
2021
A

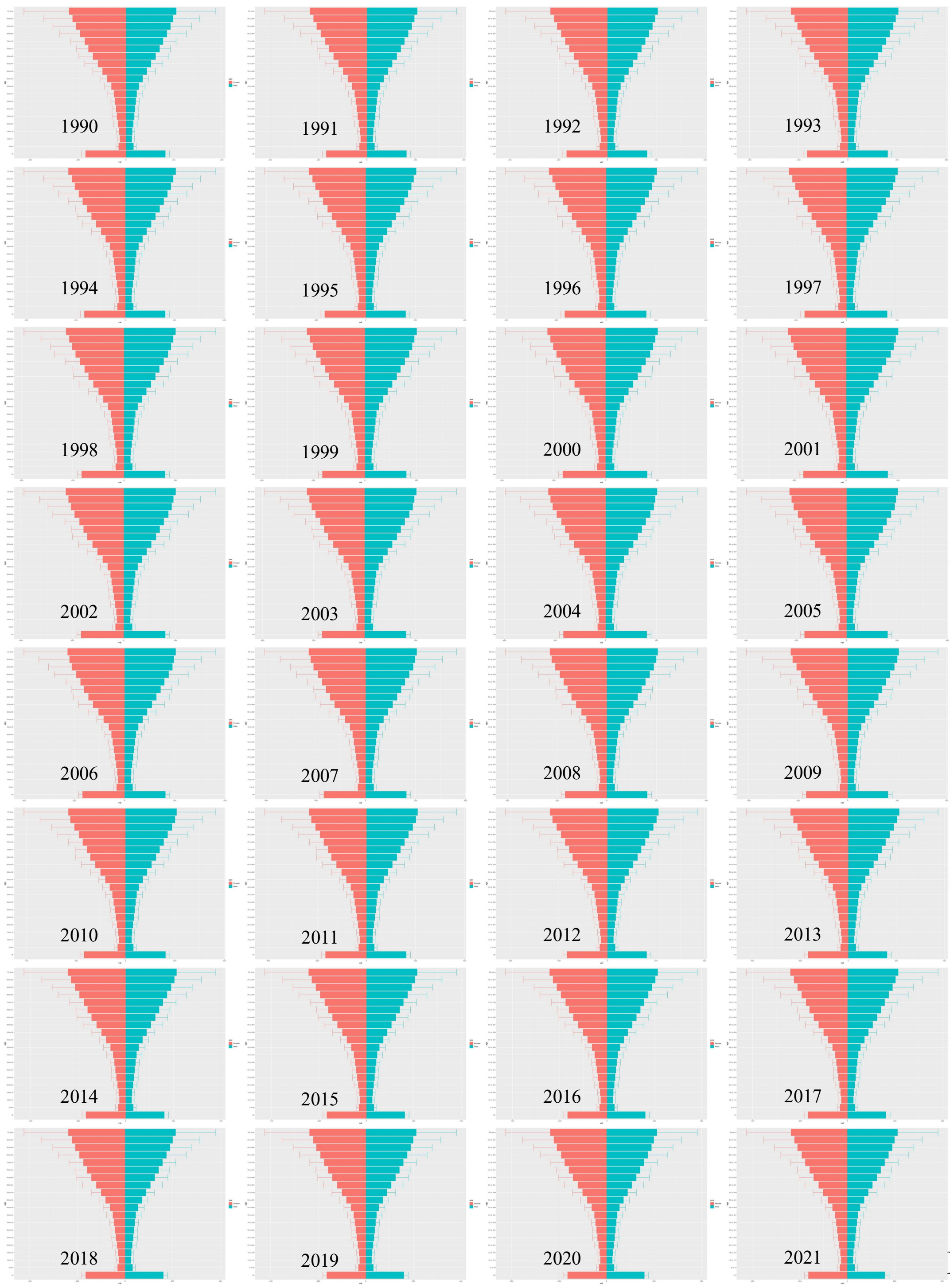
1990
1991
1992
1993
1994
1995
1996
1997
1998
1999
2000
2001
2002
2003
2004
2005
2006
2007
2008
2009
2010
2011
2012
2013
2014
2015
2016
2017
2018
2019
2020
2021

B

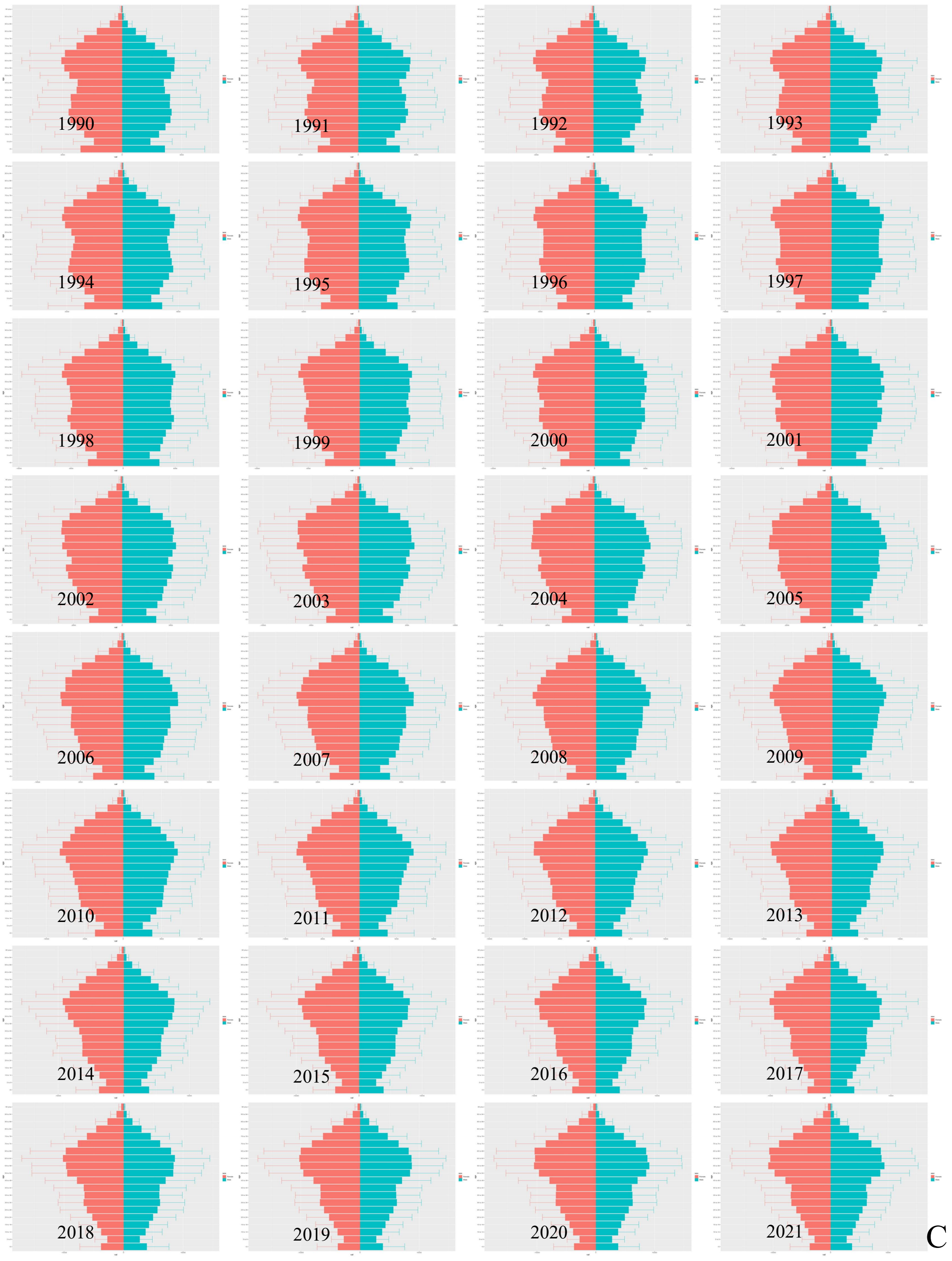
1990
1991
1992
1993
1994
1995
1996
1997
1998
1999
2000
2001
2002
2003
2004
2005
2006
2007
2008
2009
2010
2011
2012
2013
2014
2015
2016
2017
2018
2019
2020
2021
C

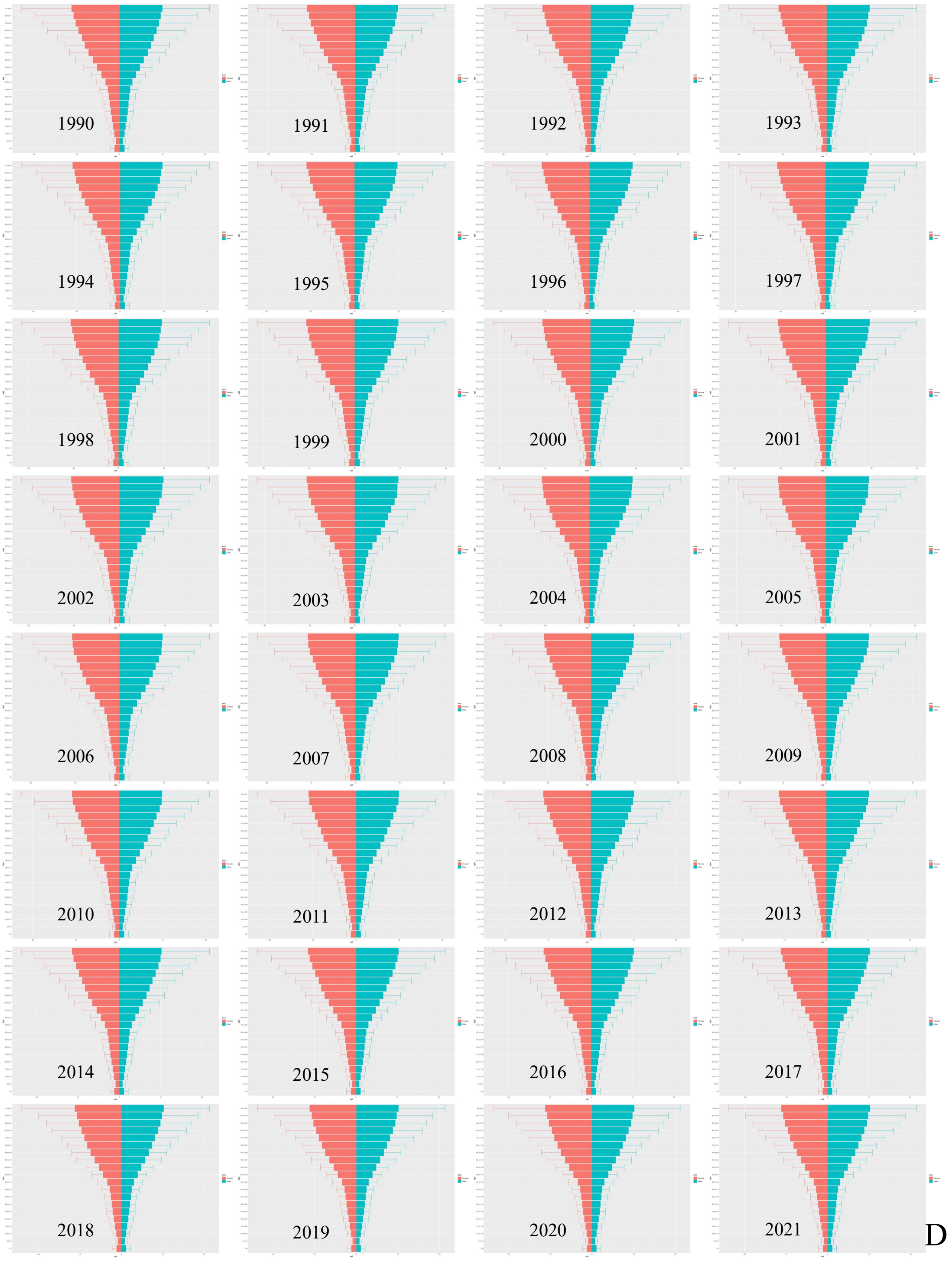

Fig. S55 (A) The prevalence cases of varicella and herpes zoster in different ages from 1990 to 2021 in the worldwide; (B) The prevalence rates of varicella and herpes zoster in different ages from 1990 to 2021 in the worldwide; (C) The years lived with disability of varicella and herpes zoster in different ages from 1990 to 2021 in the worldwide; (D) The years lived with disability rates of varicella and herpes zoster in different ages from 1990 to 2021 in the worldwide.

Notes: red for female, green for male; the ordinate from bottom to top is "<5", "5 to 9", "10 to 14", "15 to 19", "20 to 24", "25 to 29", "30 to 34", "35 to 39", "40 to 44", "45 to 49", "50 to 54", "55 to 59", "60 to 64", "65 to 69", "70 to 74", "75 to 79", "80 to 84", "85 to 89", "90 to 94", "95 plus".

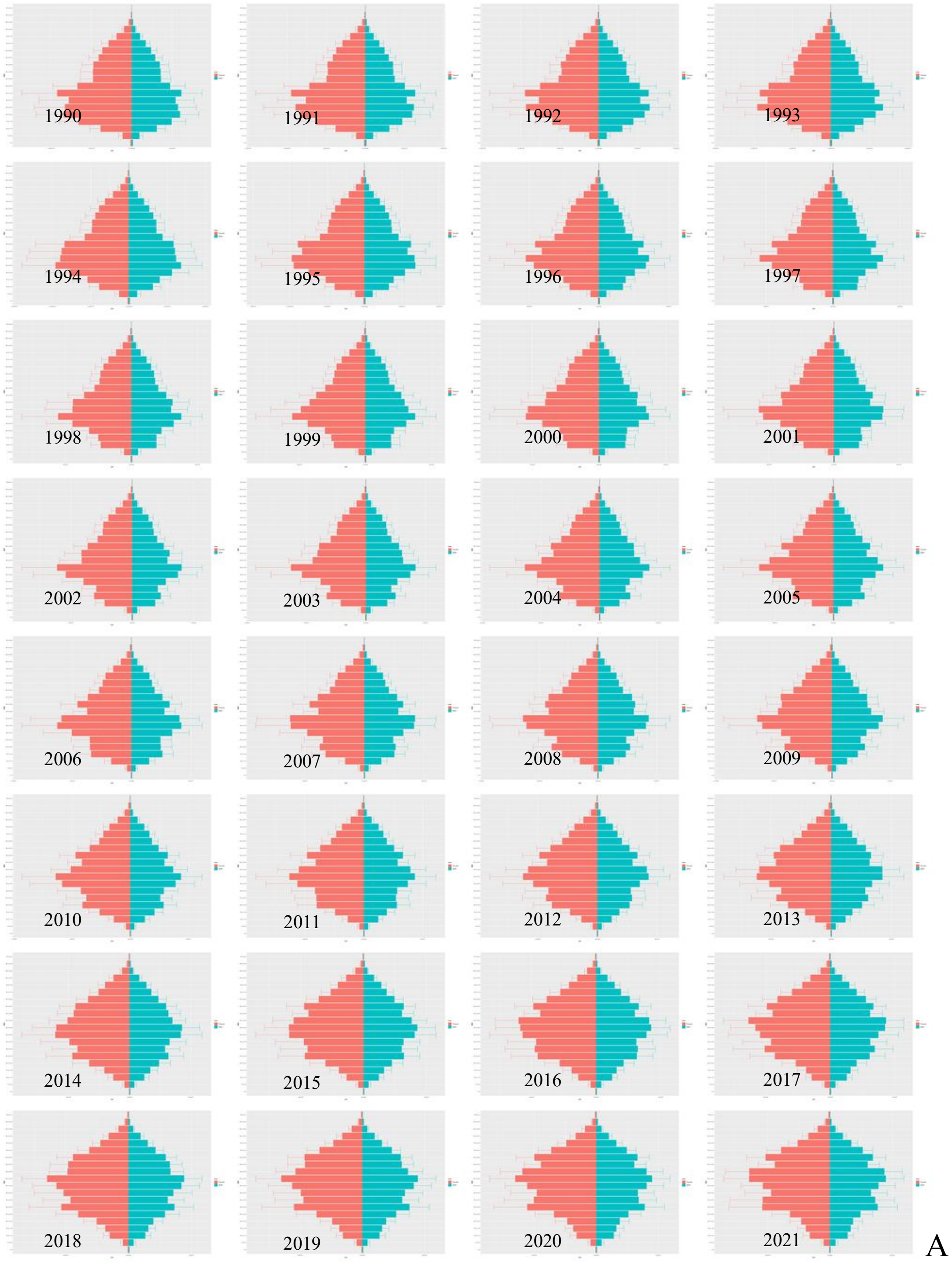
1990
1991
1992
1993
1994
1995
1996
1997
1998
1999
2000
2001
2002
2003
2004
2005
2006
2007
2008
2009
2010
2011
2012
2013
2014
2015
2016
2017
2018
2019
2020
2021
A

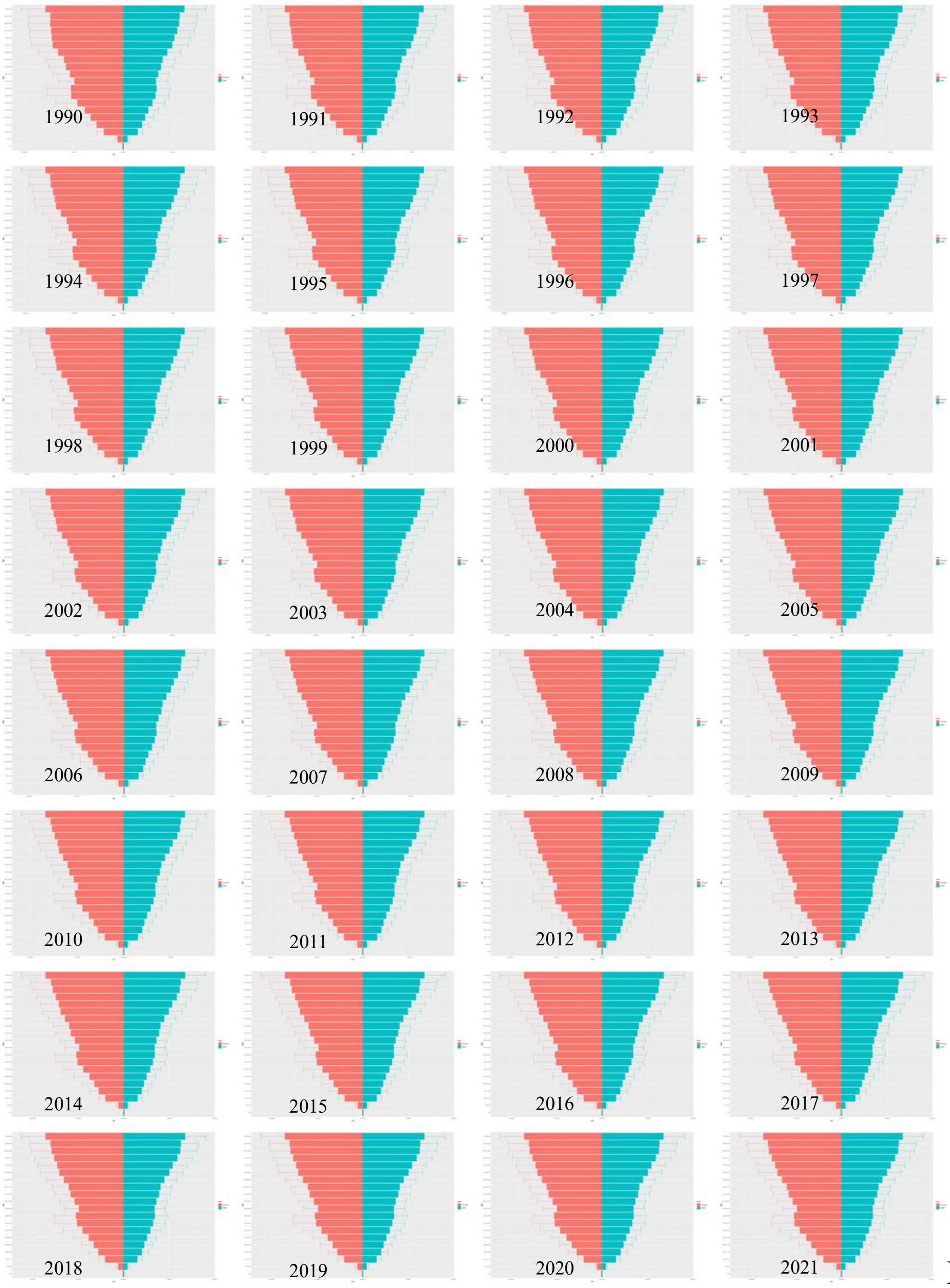
1990
1991
1992
1993
1994
1995
1996
1997
1998
1999
2000
2001
2002
2003
2004
2005
2006
2007
2008
2009
2010
2011
2012
2013
2014
2015
2016
2017
2018
2019
2020
2021

B

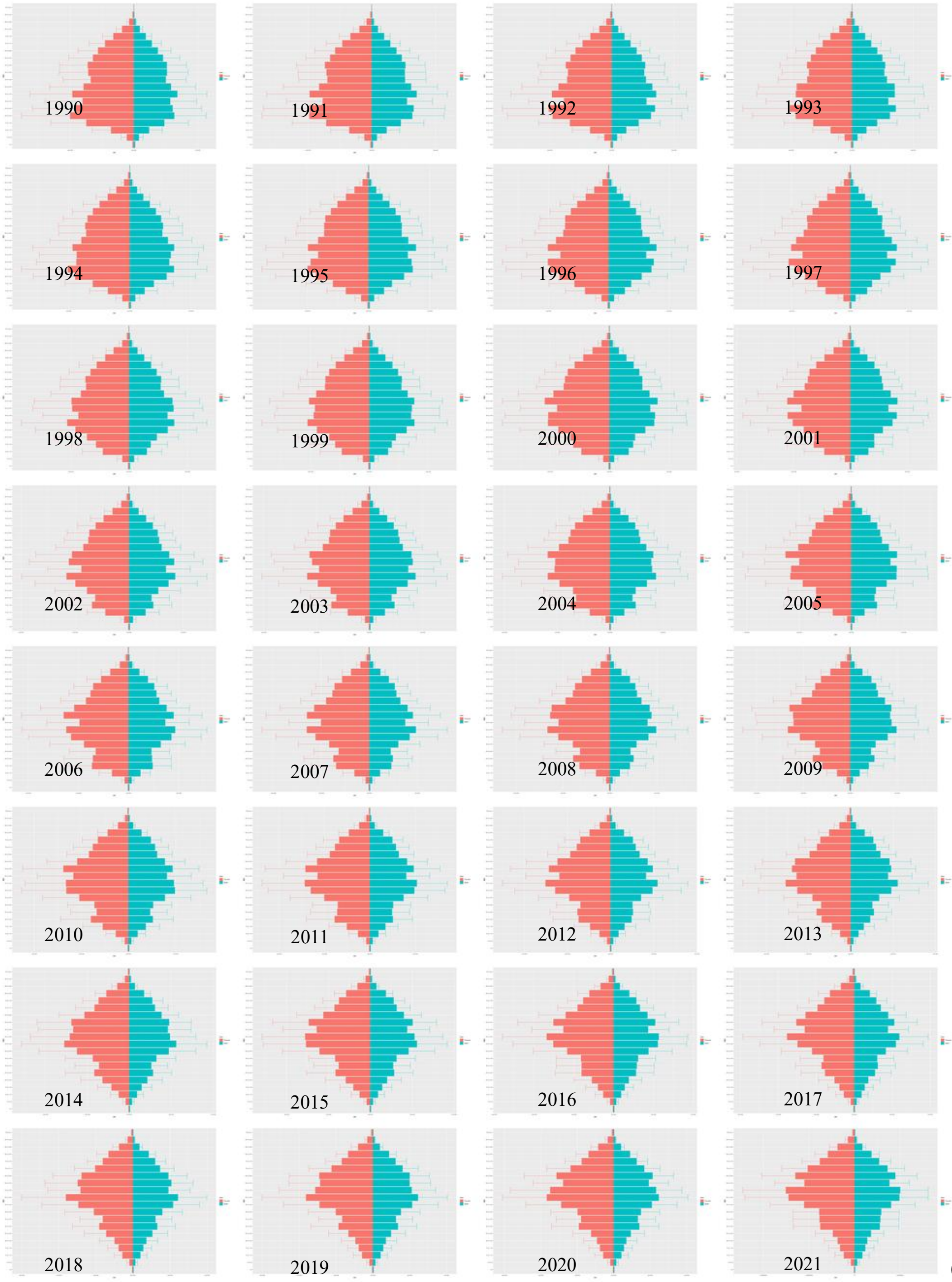
1990
1991
1992
1993
1994
1995
1996
1997
1998
1999
2000
2001
2002
2003
2004
2005
2006
2007
2008
2009
2010
2011
2012
2013
2014
2015
2016
2017
2018
2019
2020
2021

C

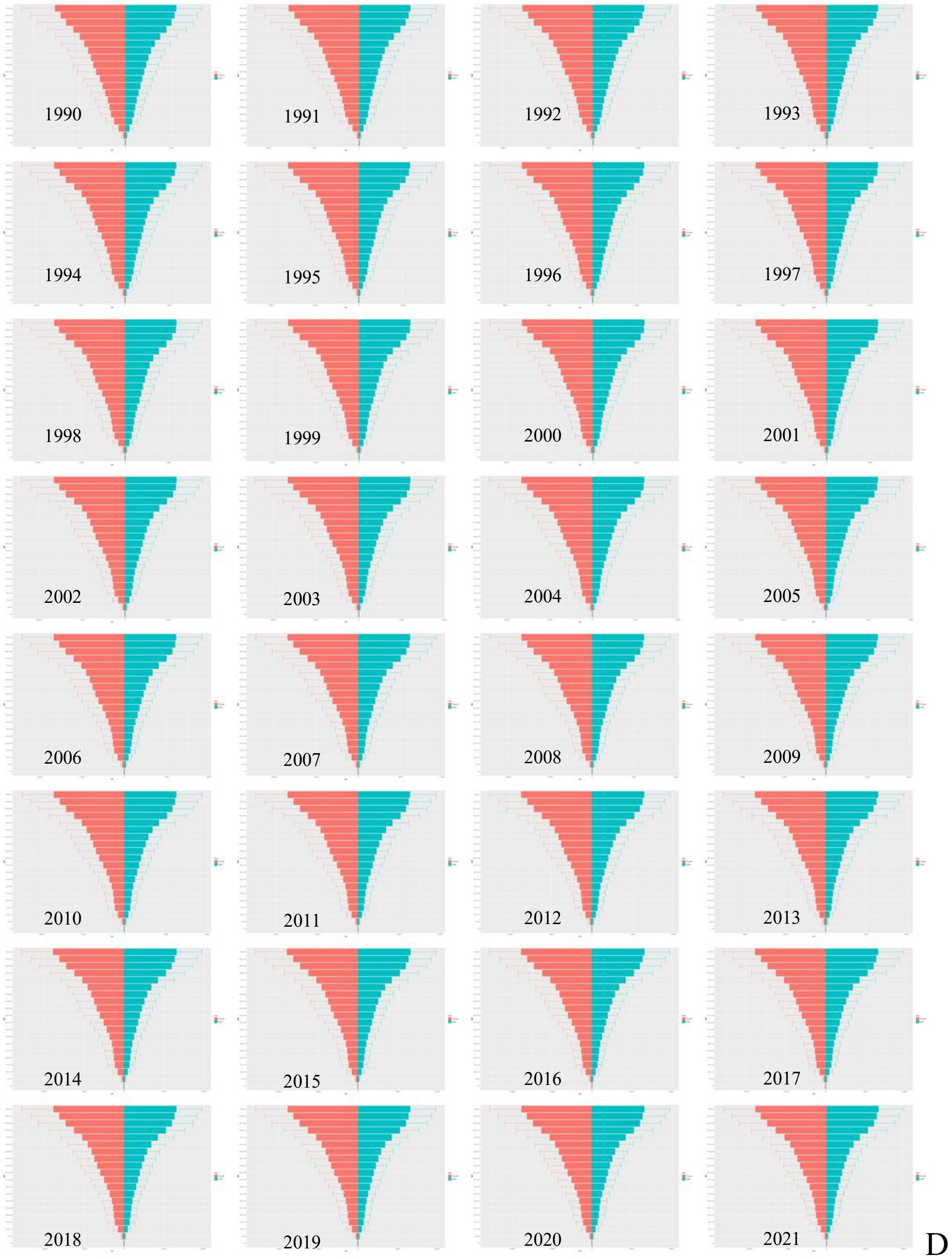

Fig. S56 (A) Acupuncture demands based on prevalence cases in different ages from 1990 to 2021 in China; (B) Acupuncture demands based on prevalence rates in different ages from 1990 to 2021 in China; (C) Acupuncture demands based on years lived with disability in different ages from 1990 to 2021 in China; (D) Acupuncture demands based on years lived with disability rates in different ages from 1990 to 2021 in China.

Notes: red for female, green for male; the ordinate from bottom to top is "<5", "5 to 9", "10 to 14", "15 to 19", "20 to 24", "25 to 29", "30 to 34", "35 to 39", "40 to 44", "45 to 49", "50 to 54", "55 to 59", "60 to 64", "65 to 69", "70 to 74", "75 to 79", "80 to 84", "85 to 89", "90 to 94", "95 plus".

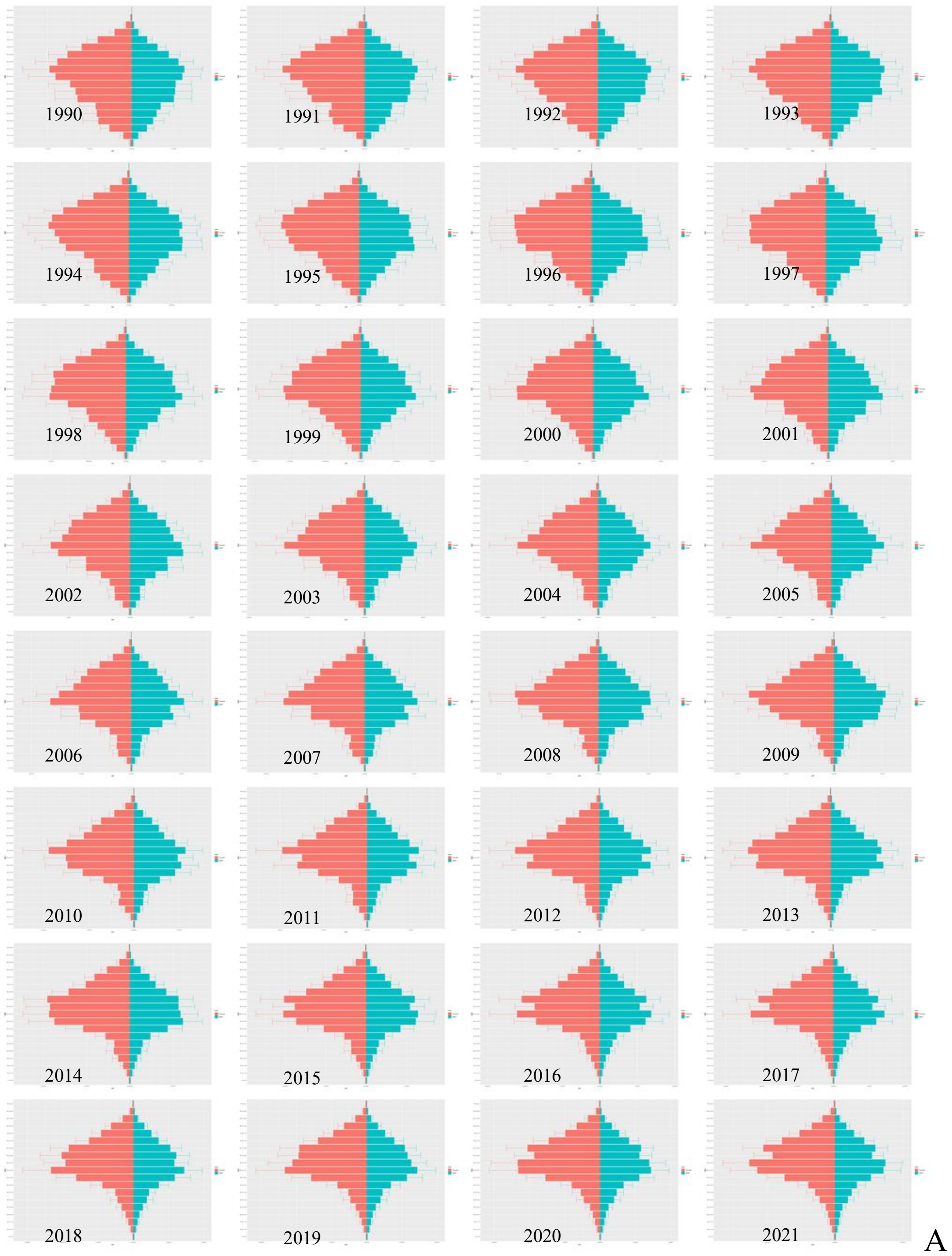
1990
1991
1992
1993
1994
1995
1996
1997
1998
1999
2000
2001
2002
2003
2004
2005
2006
2007
2008
2009
2010
2011
2012
2013
2014
2015
2016
2017
2018
2019
2020
2021
A

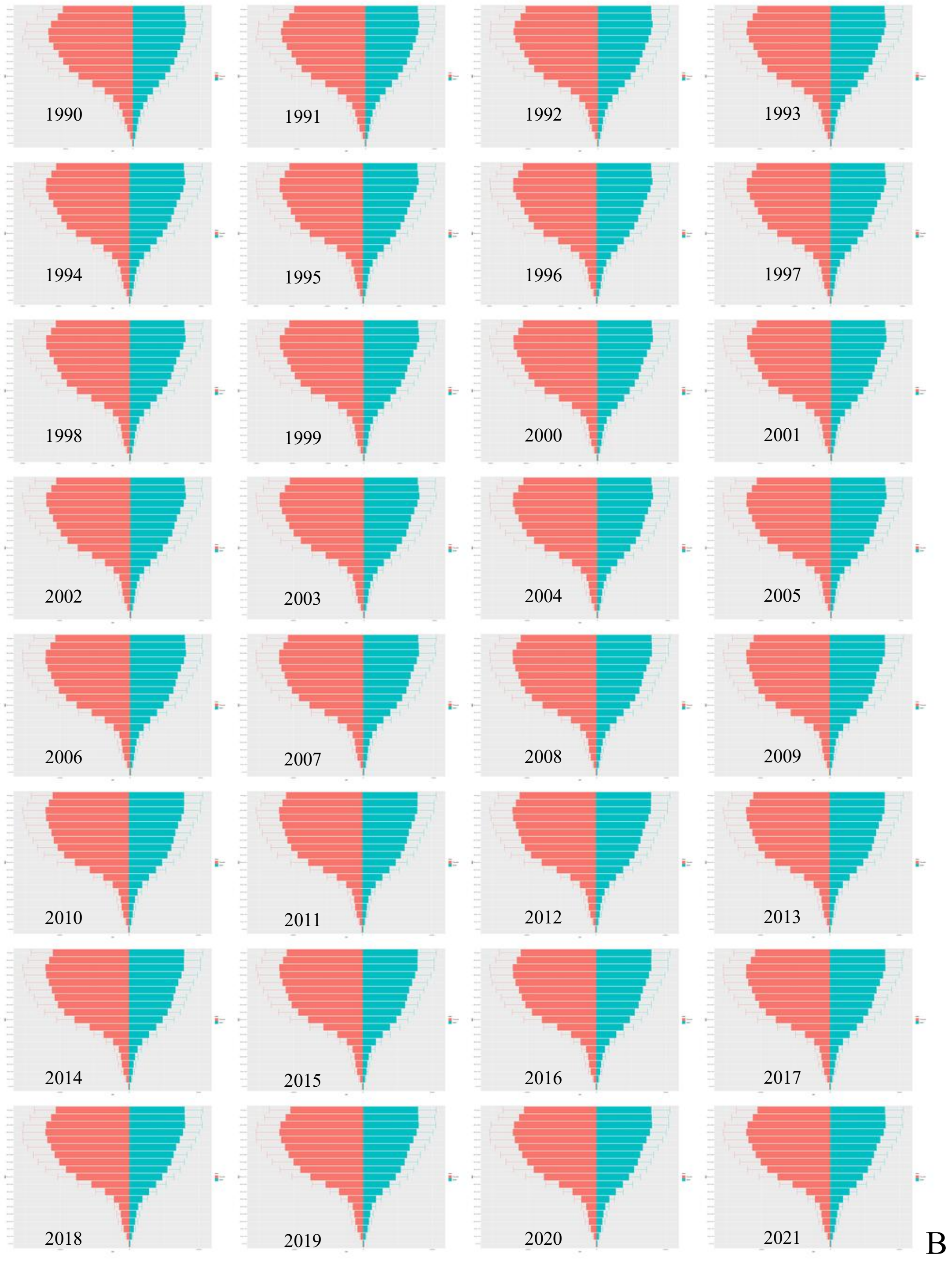
1990
1991
1992
1993
1994
1995
1996
1997
1998
1999
2000
2001
2002
2003
2004
2005
2006
2007
2008
2009
2010
2011
2012
2013
2014
2015
2016
2017
2018
2019
2020
2021
B

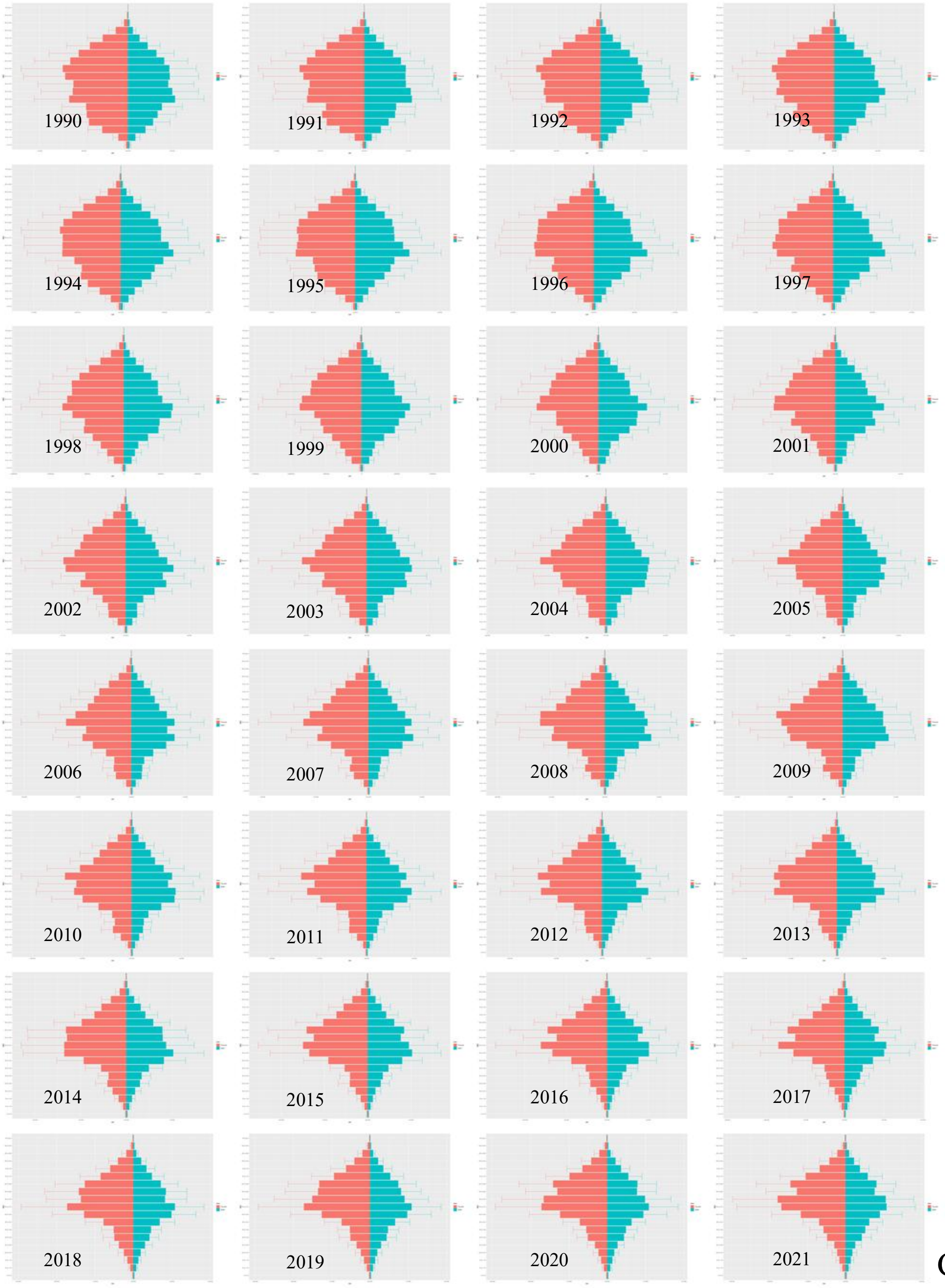
1990
1991
1992
1993
1994
1995
1996
1997
1998
1999
2000
2001
2002
2003
2004
2005
2006
2007
2008
2009
2010
2011
2012
2013
2014
2015
2016
2017
2018
2019
2020
2021

C

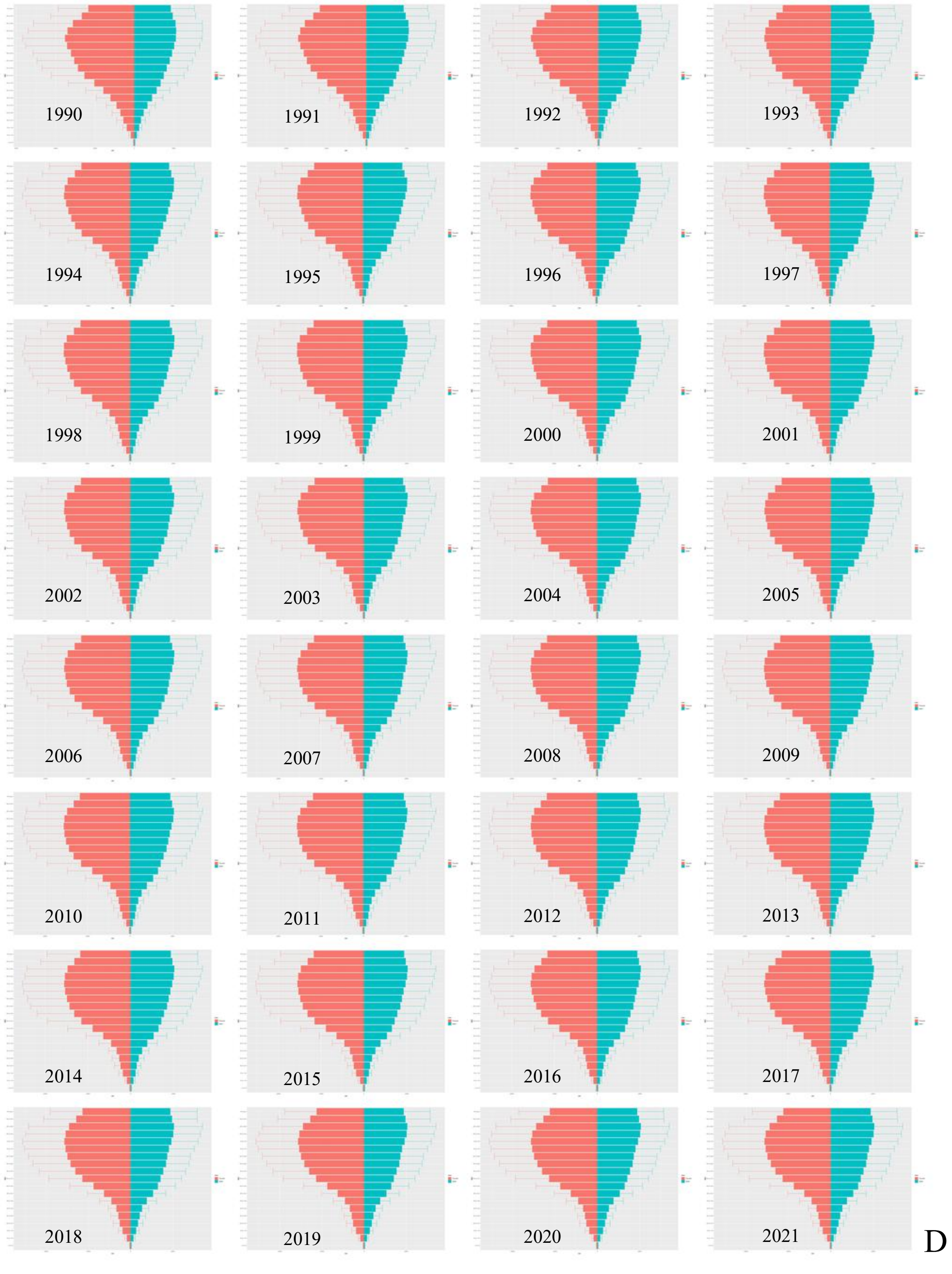

Fig. S57 (A) The prevalence cases of musculoskeletal disorders in different ages from 1990 to 2021 in China; (B) The prevalence rates of musculoskeletal disorders in different ages from 1990 to 2021 in China; (C) The years lived with disability of musculoskeletal disorders in different ages from 1990 to 2021 in China; (D) The years lived with disability rates of musculoskeletal disorders in different ages from 1990 to 2021 in China.

Notes: red for female, green for male; the ordinate from bottom to top is "5 to 9", "10 to 14", "15 to 19", "20 to 24", "25 to 29", "30 to 34", "35 to 39", "40 to 44", "45 to 49", "50 to 54", "55 to 59", "60 to 64", "65 to 69", "70 to 74", "75 to 79", "80 to 84", "85 to 89", "90 to 94", "95 plus".

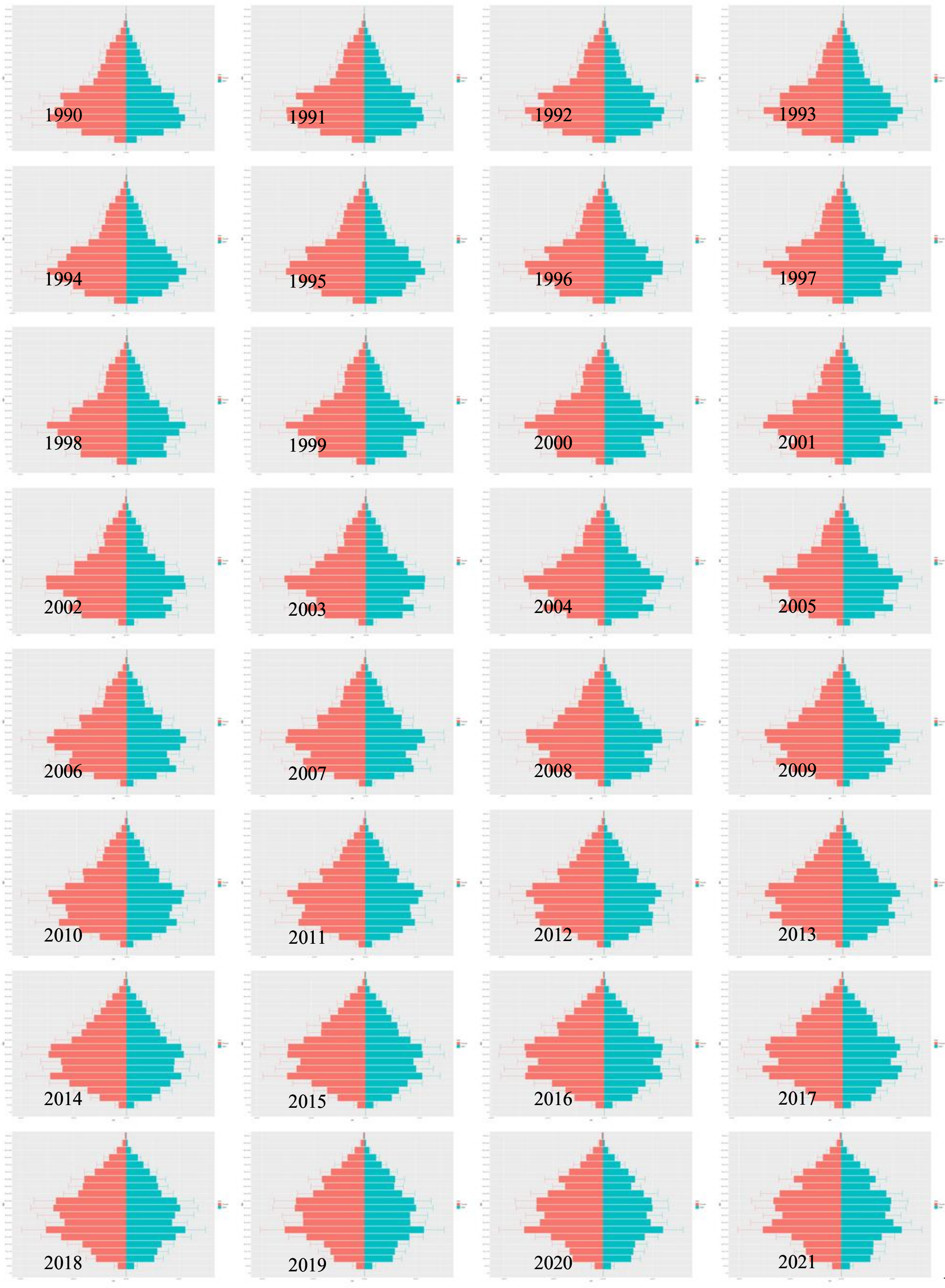
1990
1991
1992
1993
1994
1995
1996
1997
1998
1999
2000
2001
2002
2003
2004
2005
2006
2007
2008
2009
2010
2011
2012
2013
2014
2015
2016
2017
2018
2019
2020
2021

A

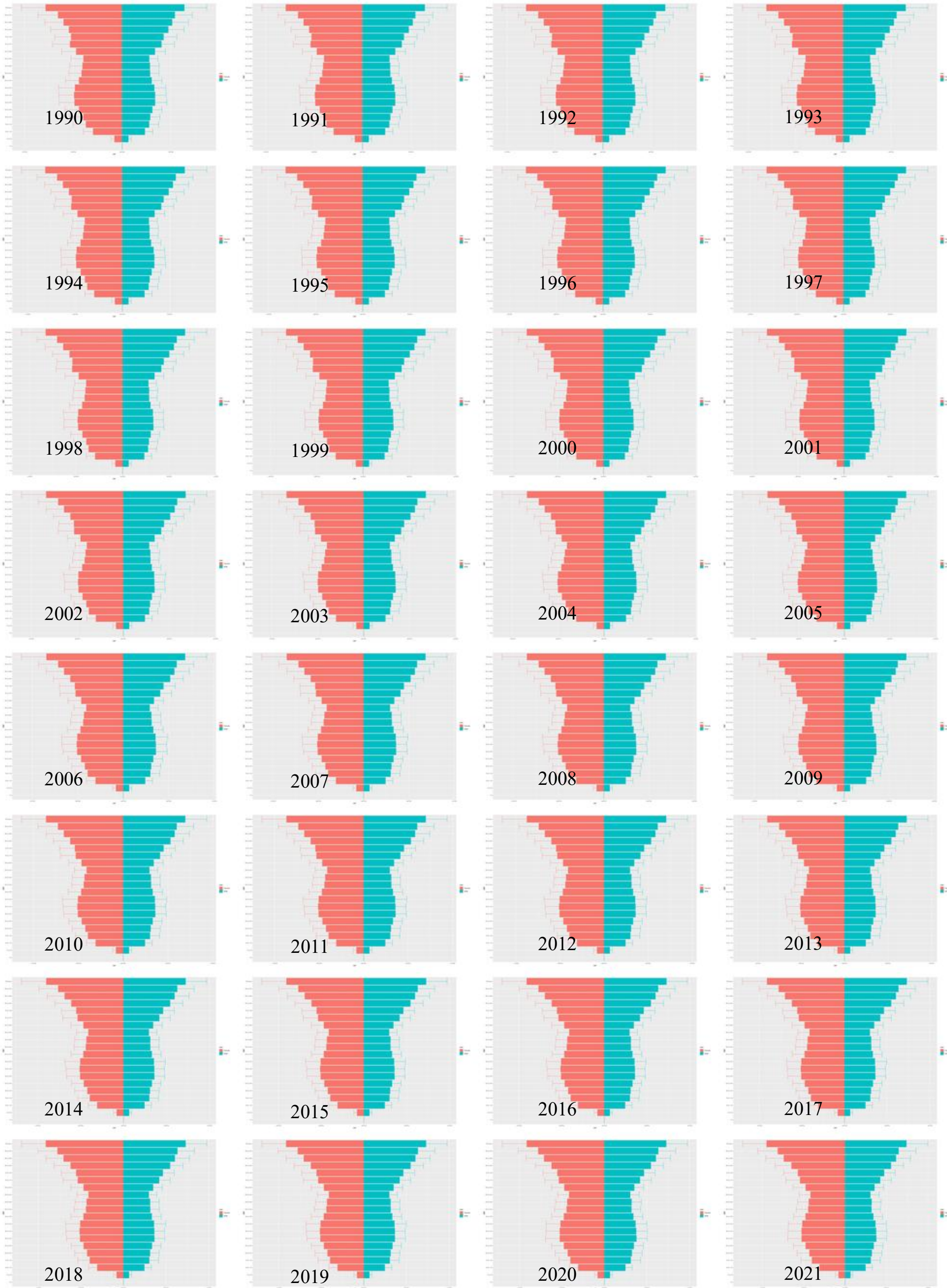
1990
1991
1992
1993
1994
1995
1996
1997
1998
1999
2000
2001
2002
2003
2004
2005
2006
2007
2008
2009
2010
2011
2012
2013
2014
2015
2016
2017
2018
2019
2020
2021

B

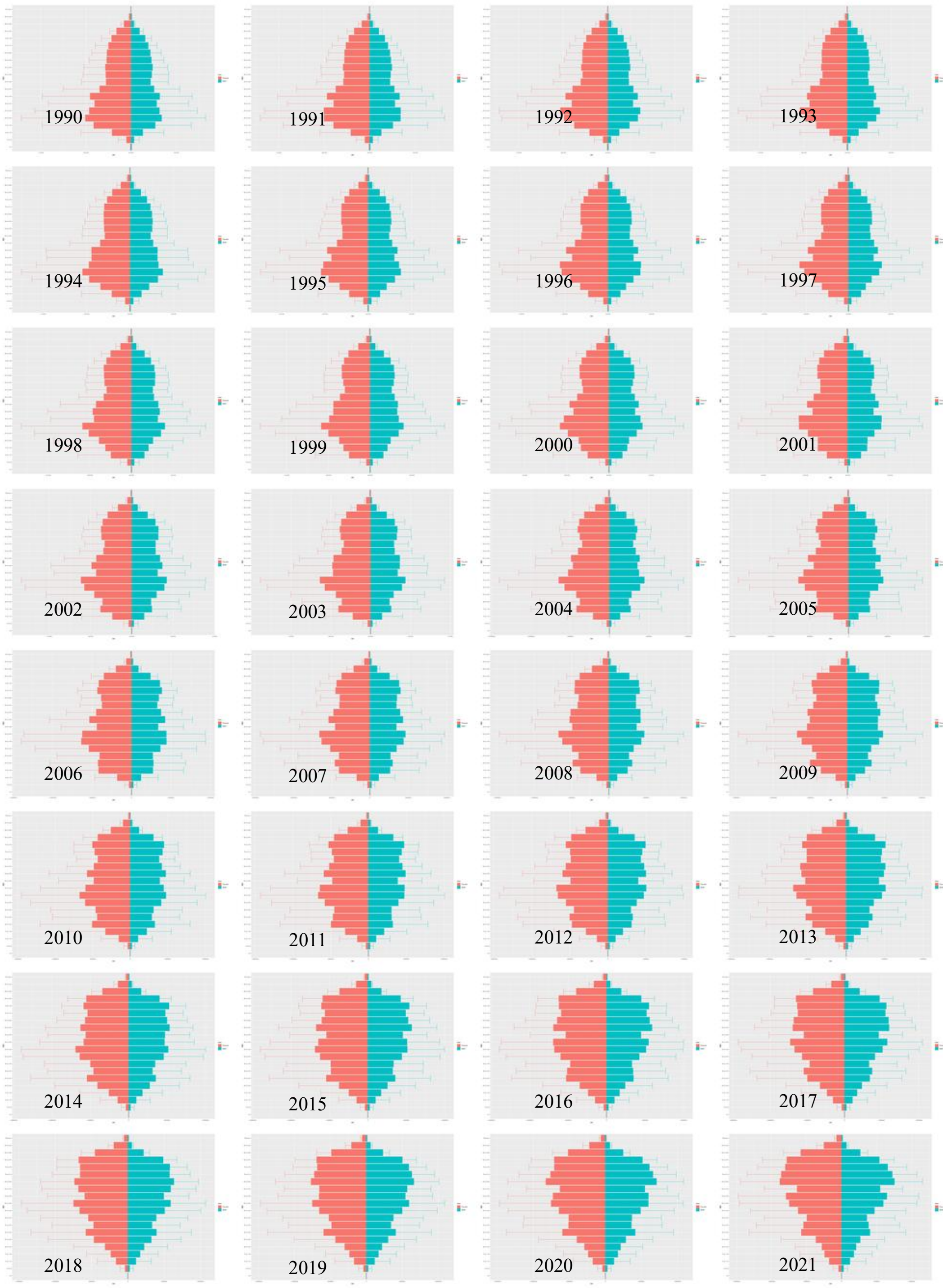
1990
1991
1992
1993
1994
1995
1996
1997
1998
1999
2000
2001
2002
2003
2004
2005
2006
2007
2008
2009
2010
2011
2012
2013
2014
2015
2016
2017
2018
2019
2020
2021

C

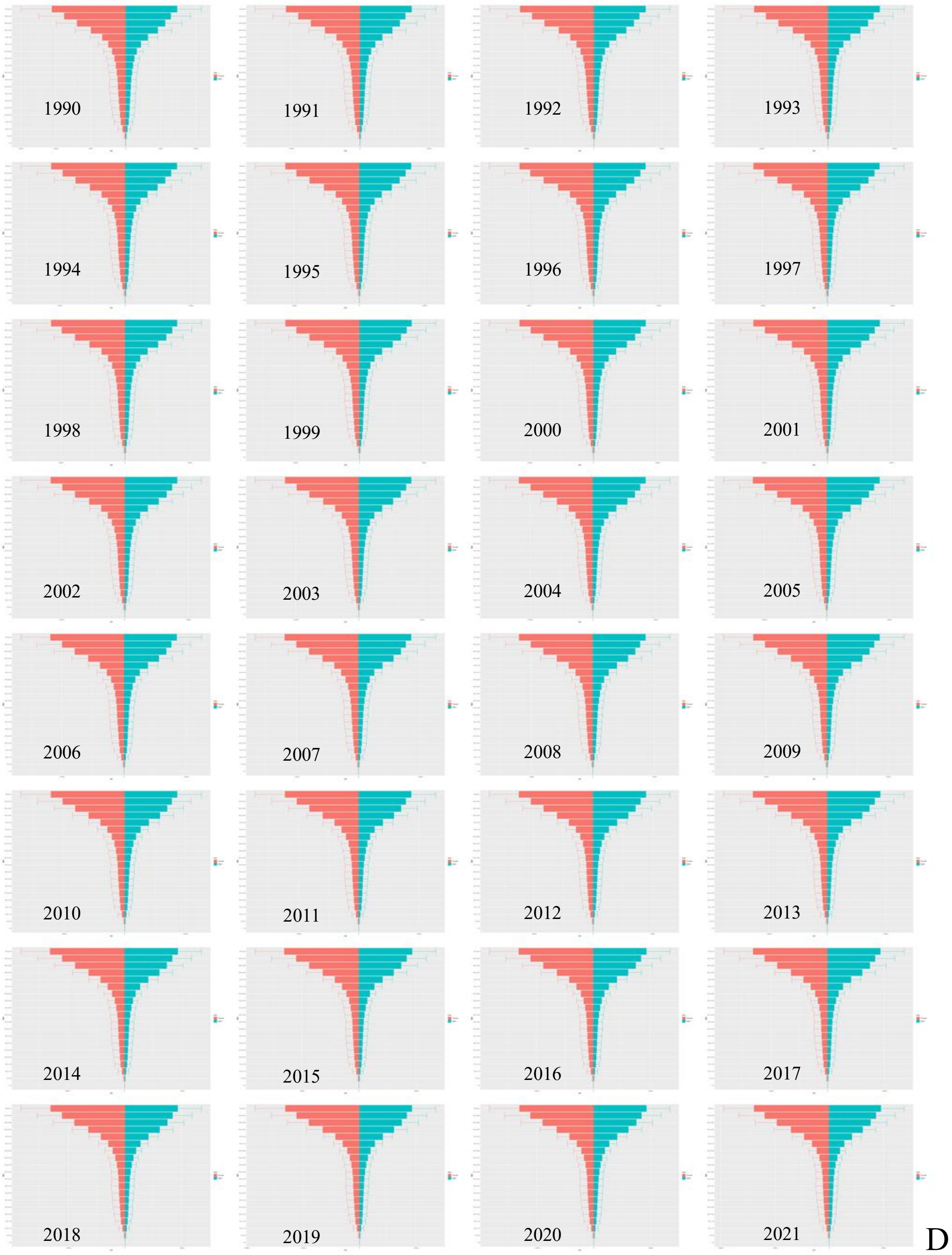

Fig. S58 (A) The prevalence cases of neurological disorders in different ages from 1990 to 2021 in China; (B) The prevalence rates of neurological disorders in different ages from 1990 to 2021 in China; (C) The years lived with disability of neurological disorders in different ages from 1990 to 2021 in China; (D) The years lived with disability rates of neurological disorders in different ages from 1990 to 2021 in China.

Notes: red for female, green for male; the ordinate from bottom to top is "<5", "5 to 9", "10 to 14", "15 to 19", "20 to 24", "25 to 29", "30 to 34", "35 to 39", "40 to 44", "45 to 49", "50 to 54", "55 to 59", "60 to 64", "65 to 69", "70 to 74", "75 to 79", "80 to 84", "85 to 89", "90 to 94", "95 plus".

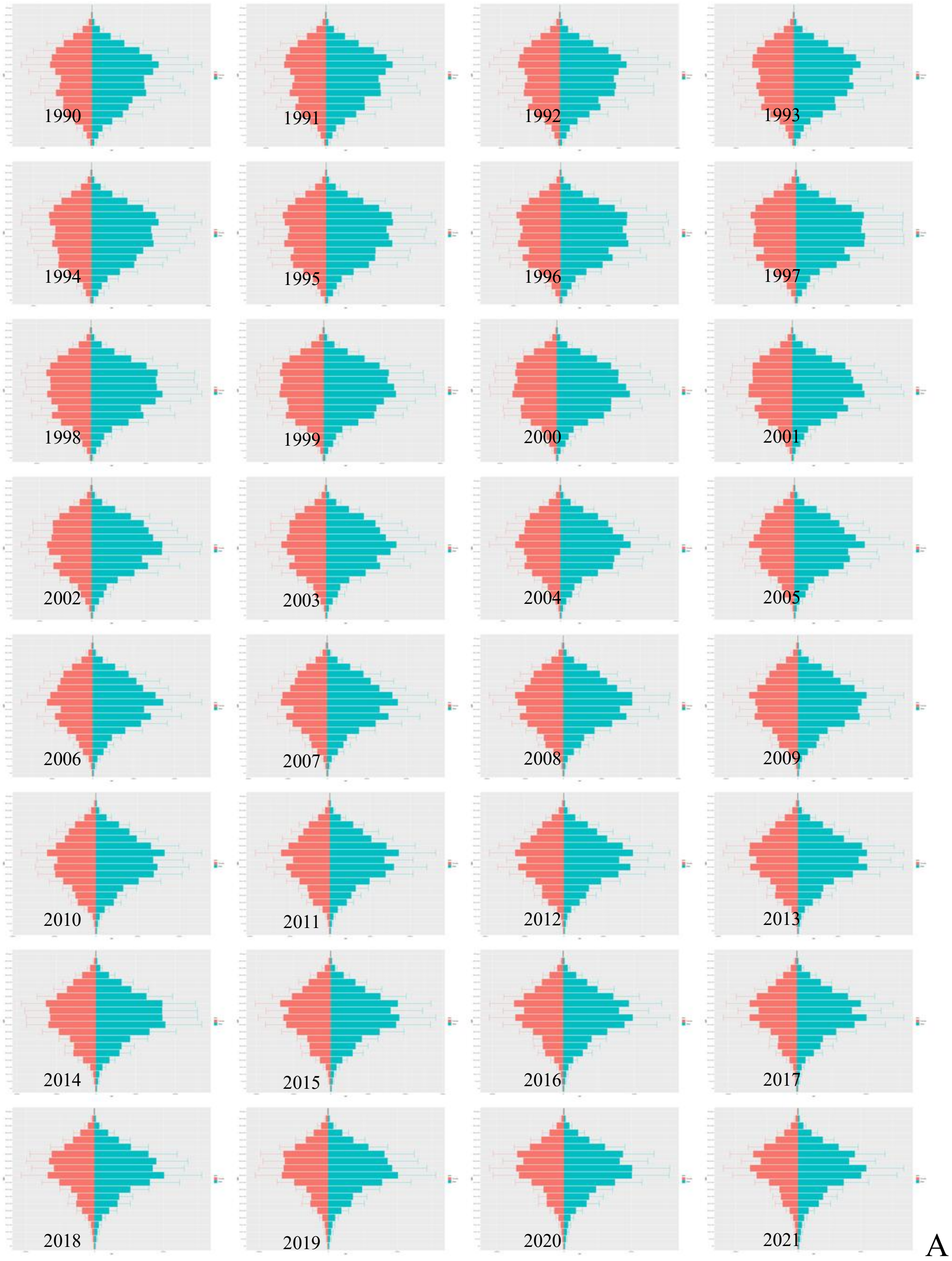
1990
1991
1992
1993
1994
1995
1996
1997
1998
1999
2000
2001
2002
2003
2004
2005
2006
2007
2008
2009
2010
2011
2012
2013
2014
2015
2016
2017
2018
2019
2020
2021

A

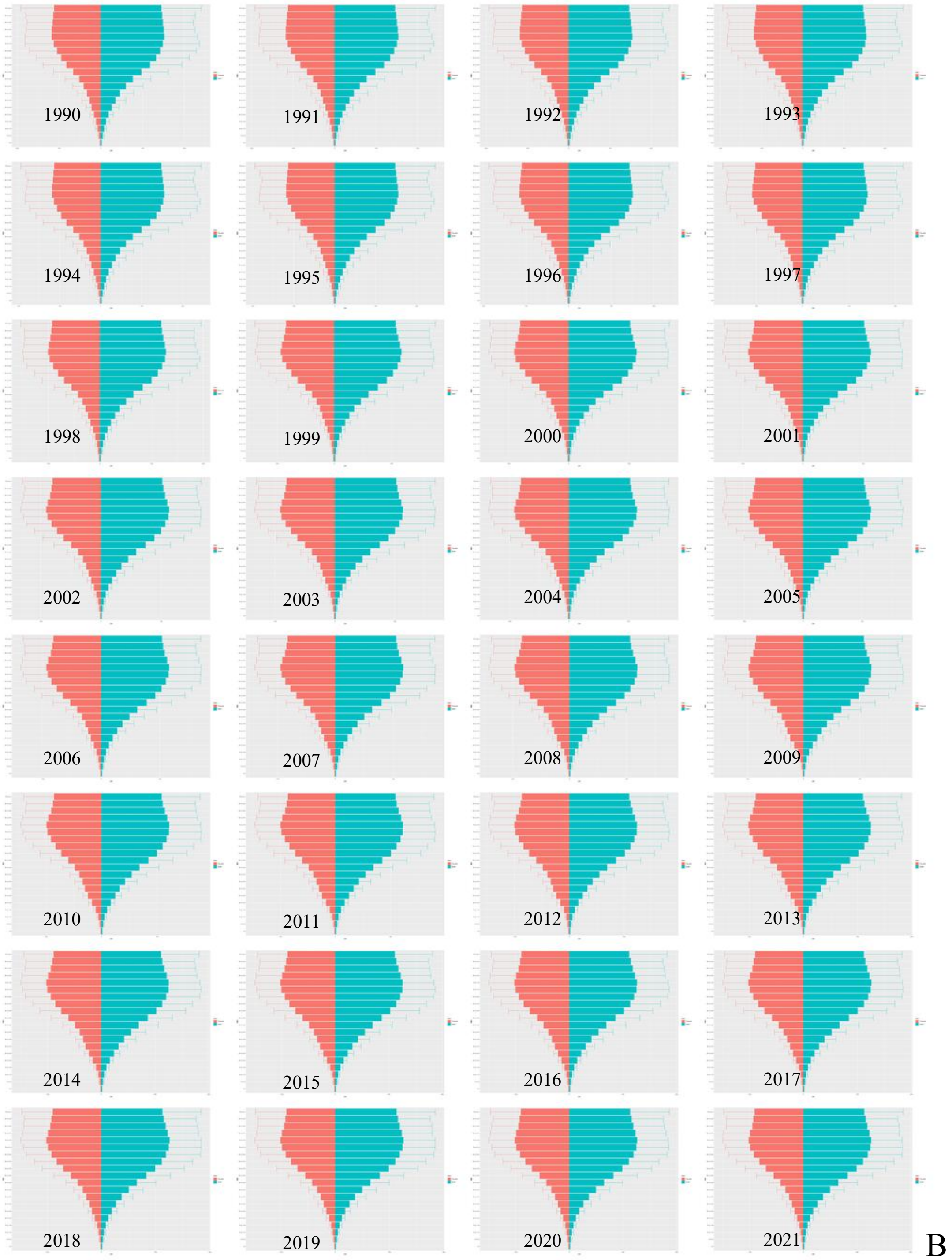
1990
1991
1992
1993
1994
1995
1996
1997
1998
1999
2000
2001
2002
2003
2004
2005
2006
2007
2008
2009
2010
2011
2012
2013
2014
2015
2016
2017
2018
2019
2020
2021
B

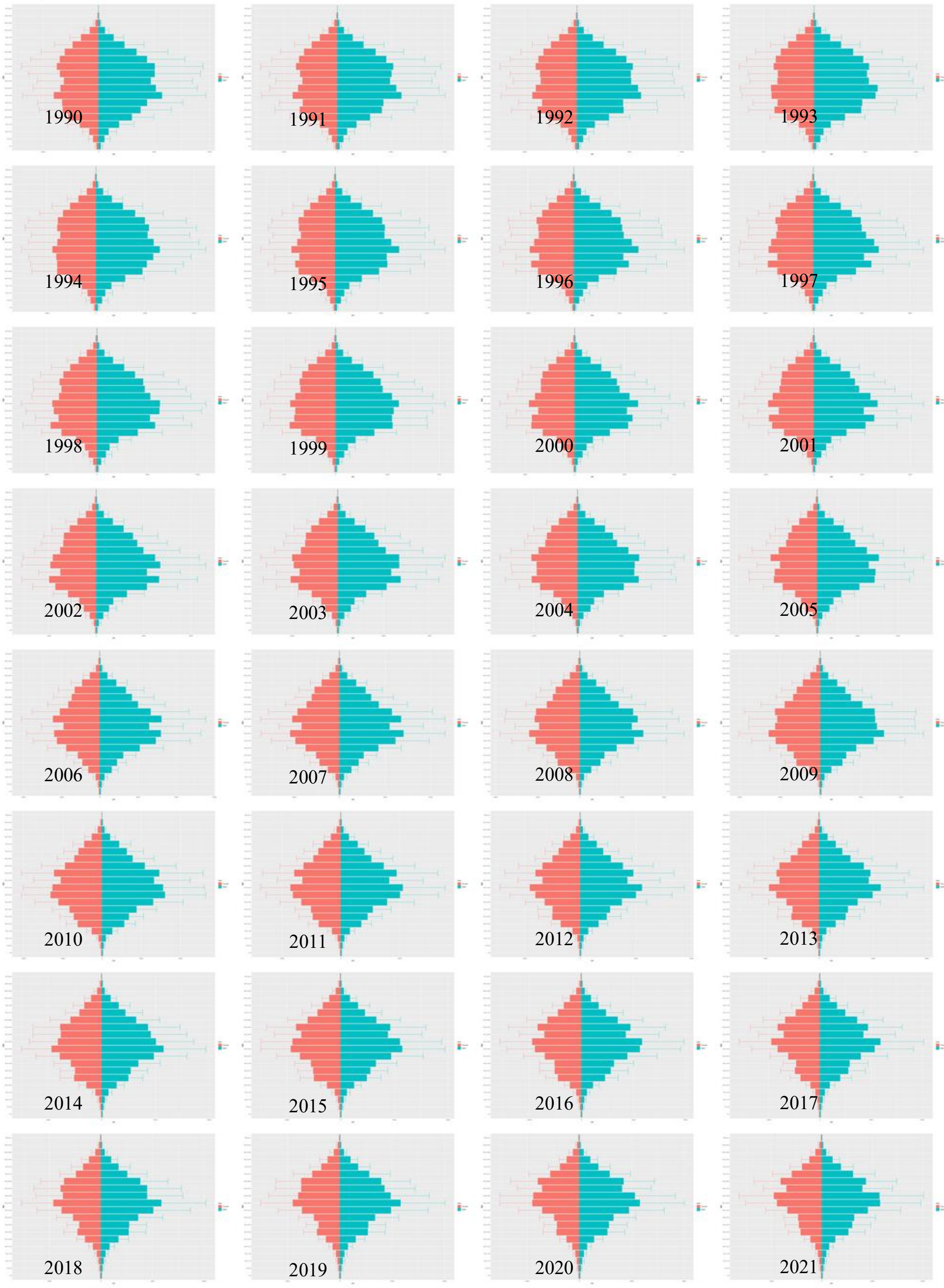
1990
1991
1992
1993
1994
1995
1996
1997
1998
1999
2000
2001
2002
2003
2004
2005
2006
2007
2008
2009
2010
2011
2012
2013
2014
2015
2016
2017
2018
2019
2020
2021

C

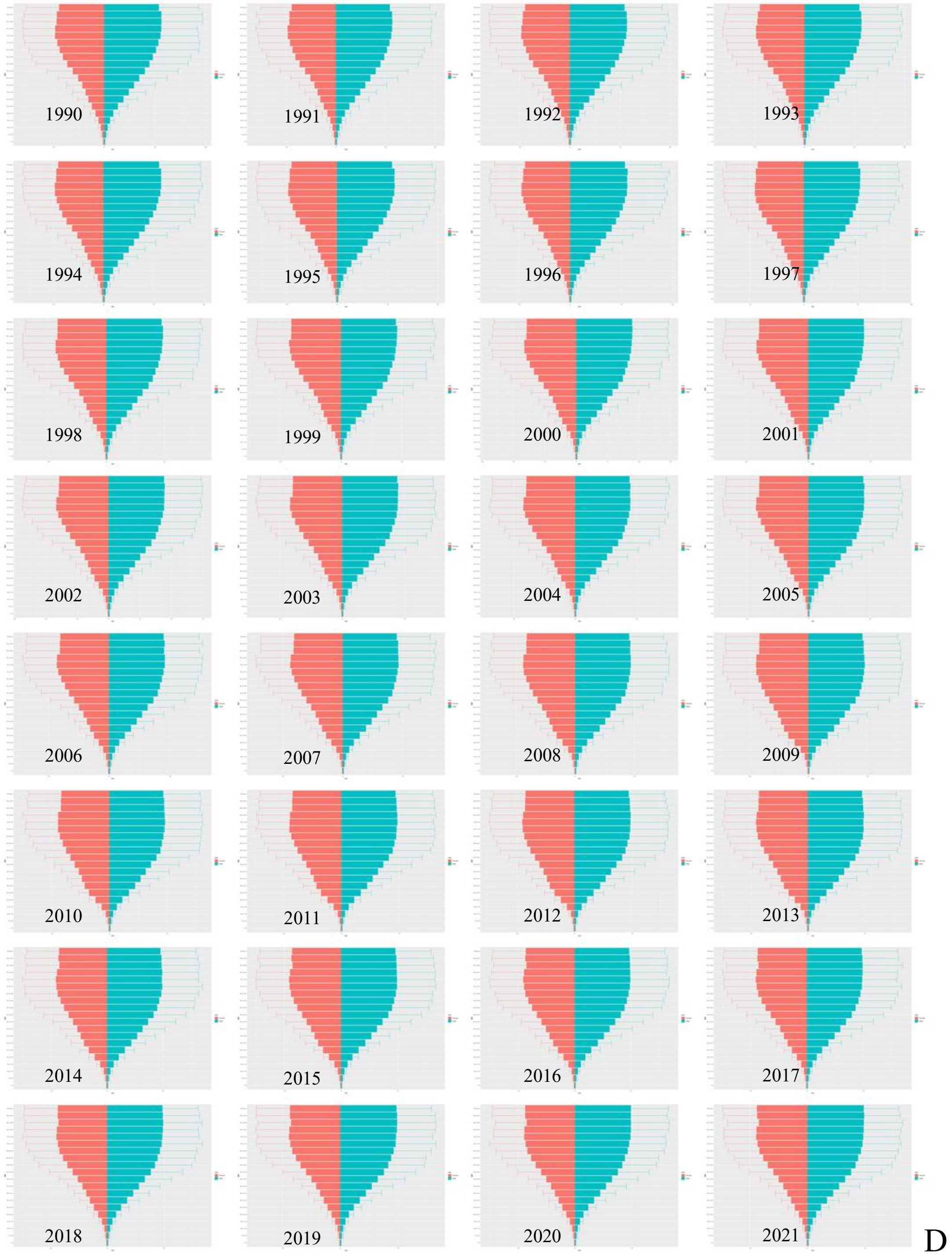

Fig. S59 (A) The prevalence cases of digestive disorders in different ages from 1990 to 2021 in China; (B) The prevalence rates of digestive disorders in different ages from 1990 to 2021 in China; (C) The years lived with disability of digestive disorders in different ages from 1990 to 2021 in China; (D) The years lived with disability rates of digestive disorders in different ages from 1990 to 2021 in China. Notes: red for female, green for male; the ordinate from bottom to top is "<5", "5 to 9", "10 to 14", "15 to 19", "20 to 24", "25 to 29", "30 to 34", "35 to 39", "40 to 44", "45 to 49", "50 to 54", "55 to 59", "60 to 64", "65 to 69", "70 to 74", "75 to 79", "80 to 84", "85 to 89", "90 to 94", "95 plus".

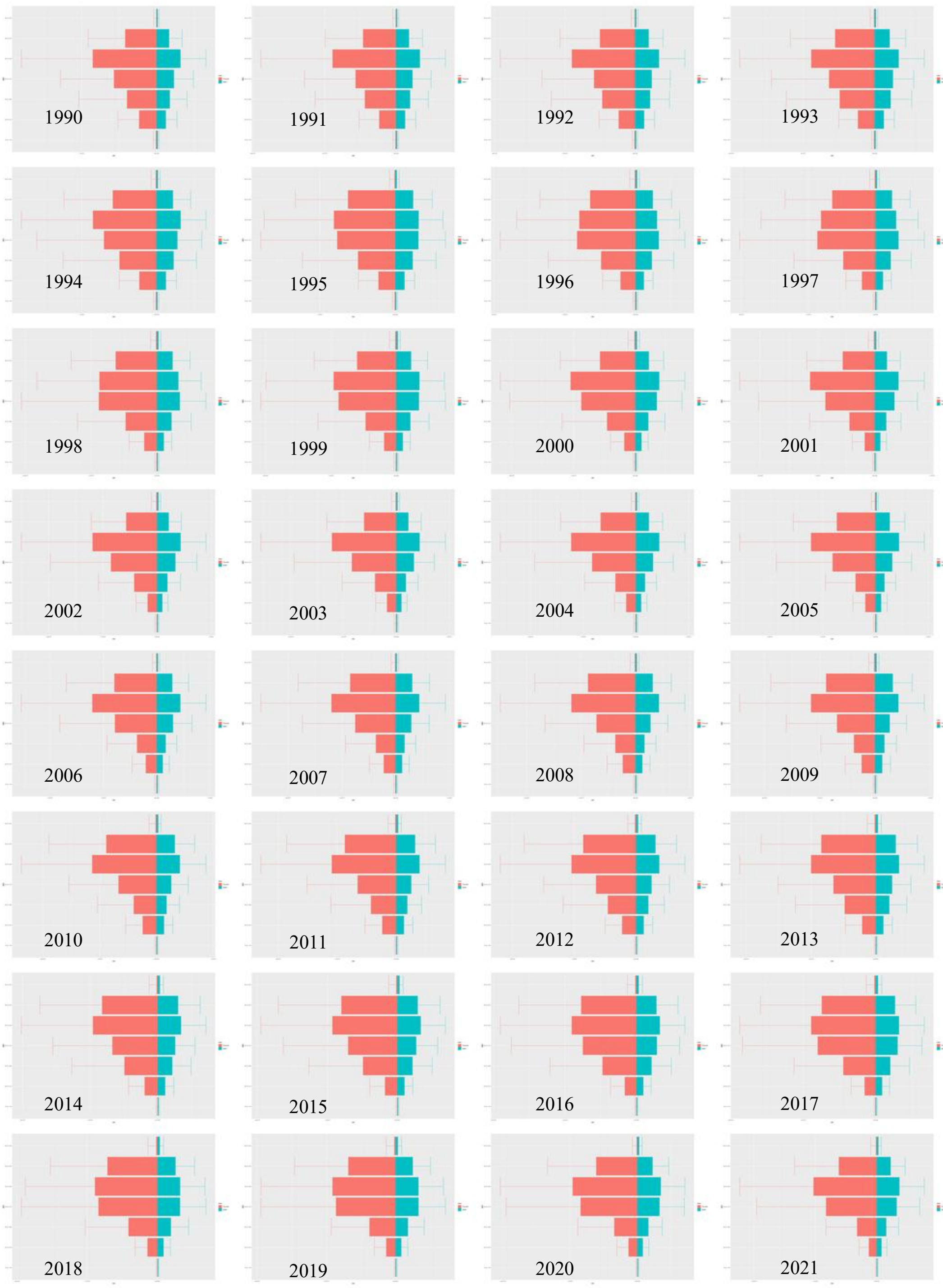
1990
1991
1992
1993
1994
1995
1996
1997
1998
1999
2000
2001
2002
2003
2004
2005
2006
2007
2008
2009
2010
2011
2012
2013
2014
2015
2016
2017
2018
2019
2020
2021

A

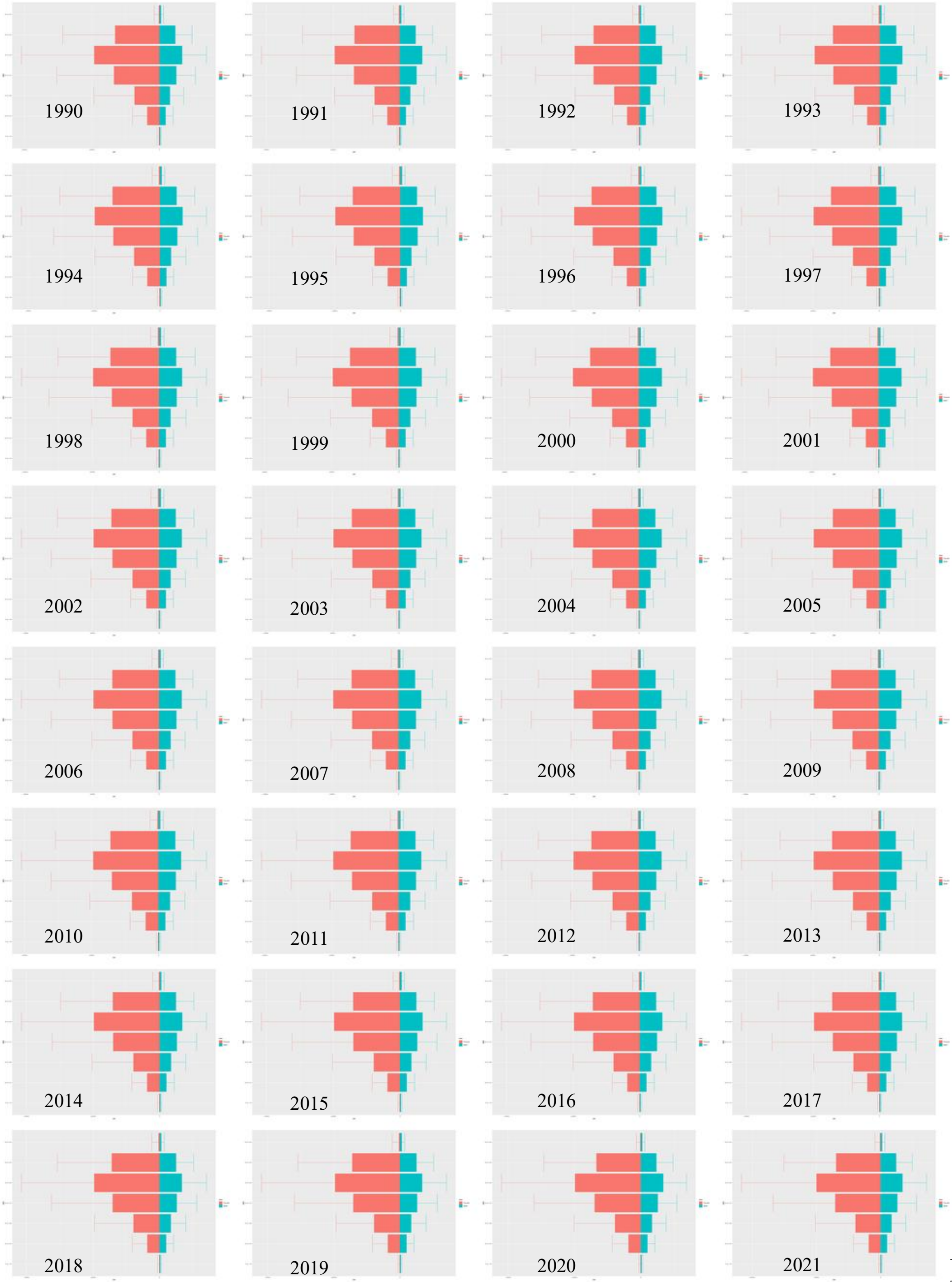
1990
1991
1992
1993
1994
1995
1996
1997
1998
1999
2000
2001
2002
2003
2004
2005
2006
2007
2008
2009
2010
2011
2012
2013
2014
2015
2016
2017
2018
2019
2020
2021

B

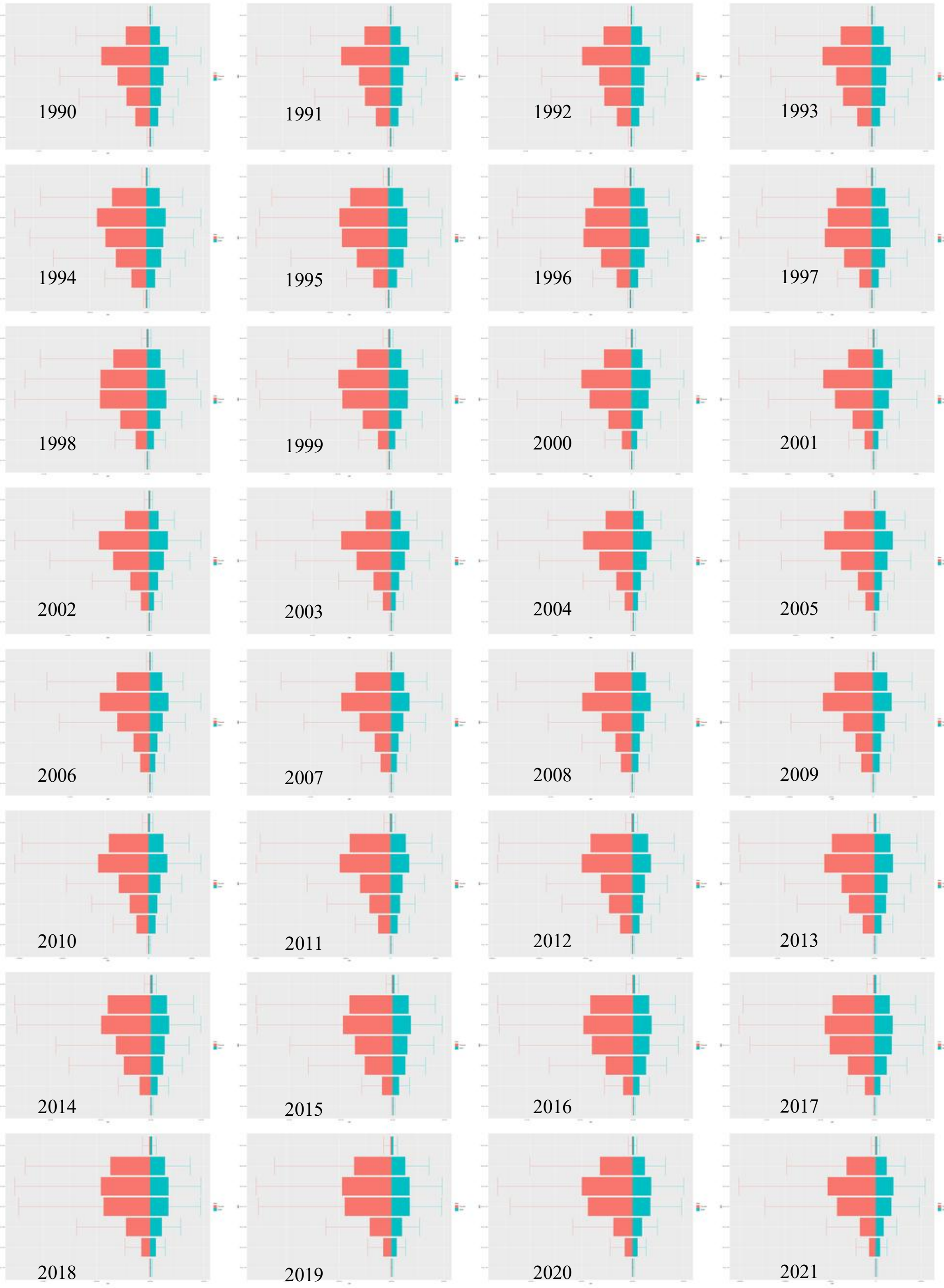
1990
1991
1992
1993
1994
1995
1996
1997
1998
1999
2000
2001
2002
2003
2004
2005
2006
2007
2008
2009
2010
2011
2012
2013
2014
2015
2016
2017
2018
2019
2020
2021

C

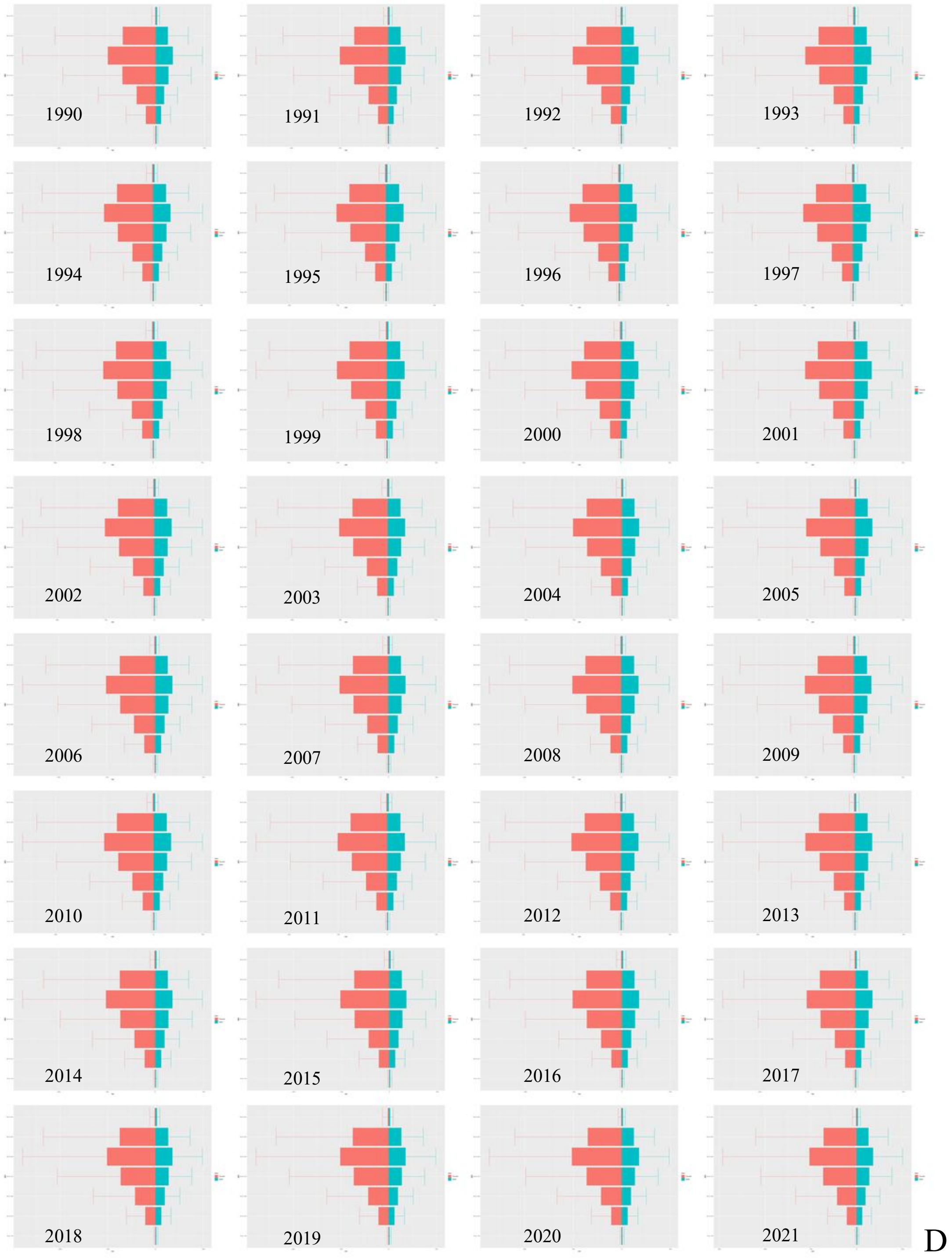

Fig. S60 (A) The prevalence cases of genecological disorders in different ages from 1990 to 2021 in China; (B) The prevalence rates of genecological disorders in different ages from 1990 to 2021 in China; (C) The years lived with disability of genecological disorders in different ages from 1990 to 2021 in China; (D) The years lived with disability rates of genecological disorders in different ages from 1990 to 2021 in China.

Notes: red for female, green for male; the ordinate from bottom to top is "15 to 19", "20 to 24", "25 to 29", "30 to 34", "35 to 39", "40 to 44", "45 to 49".

1999 2000 2001

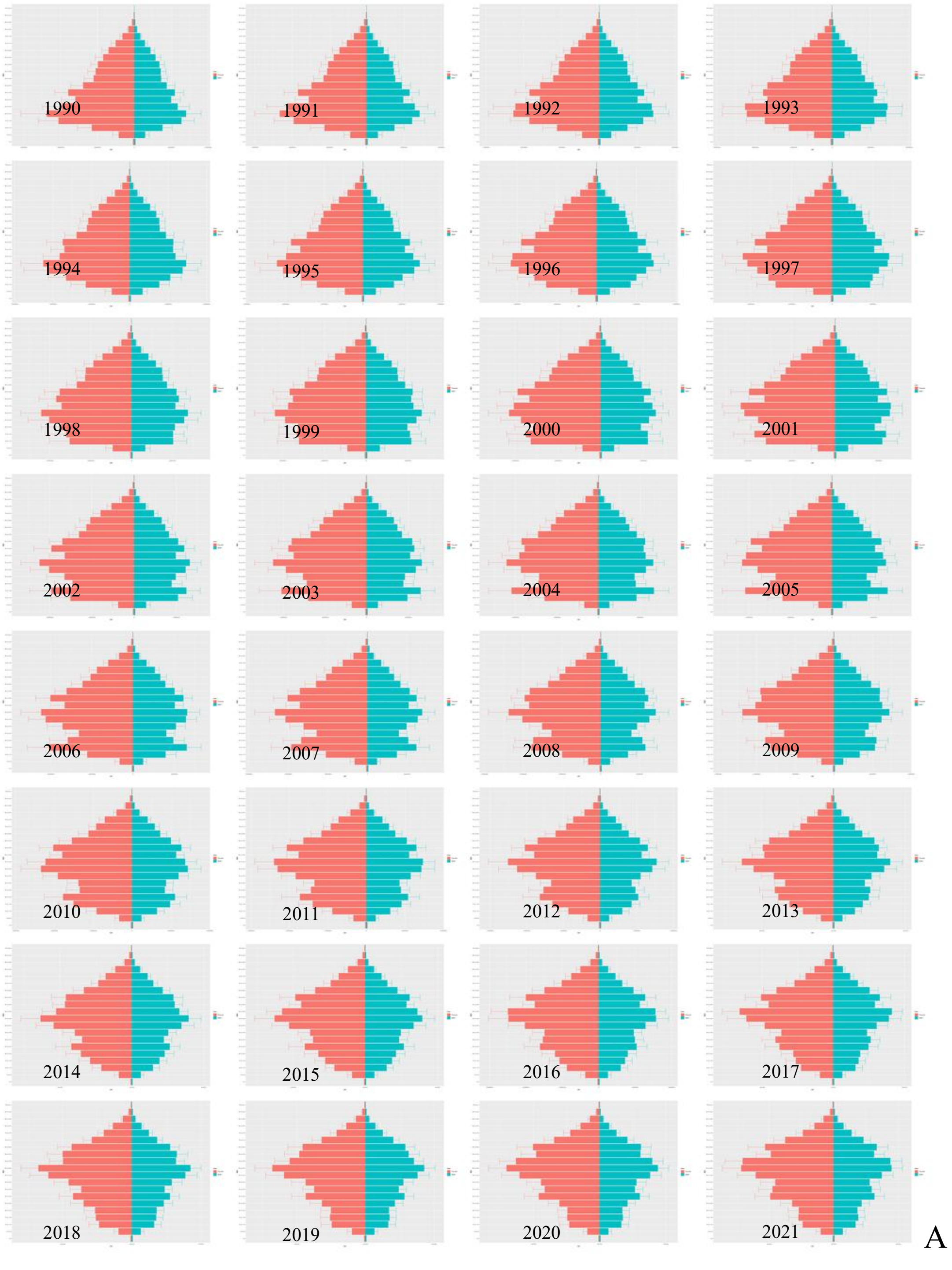
1990
1991
1992
1993
1994
1995
1996
1997
1998
1999
2000
2001
2002
2003
2004
2005
2006
2007
2008
2009
2010
2011
2012
2013
2014
2015
2016
2017
2018
2019
2020
2021
A

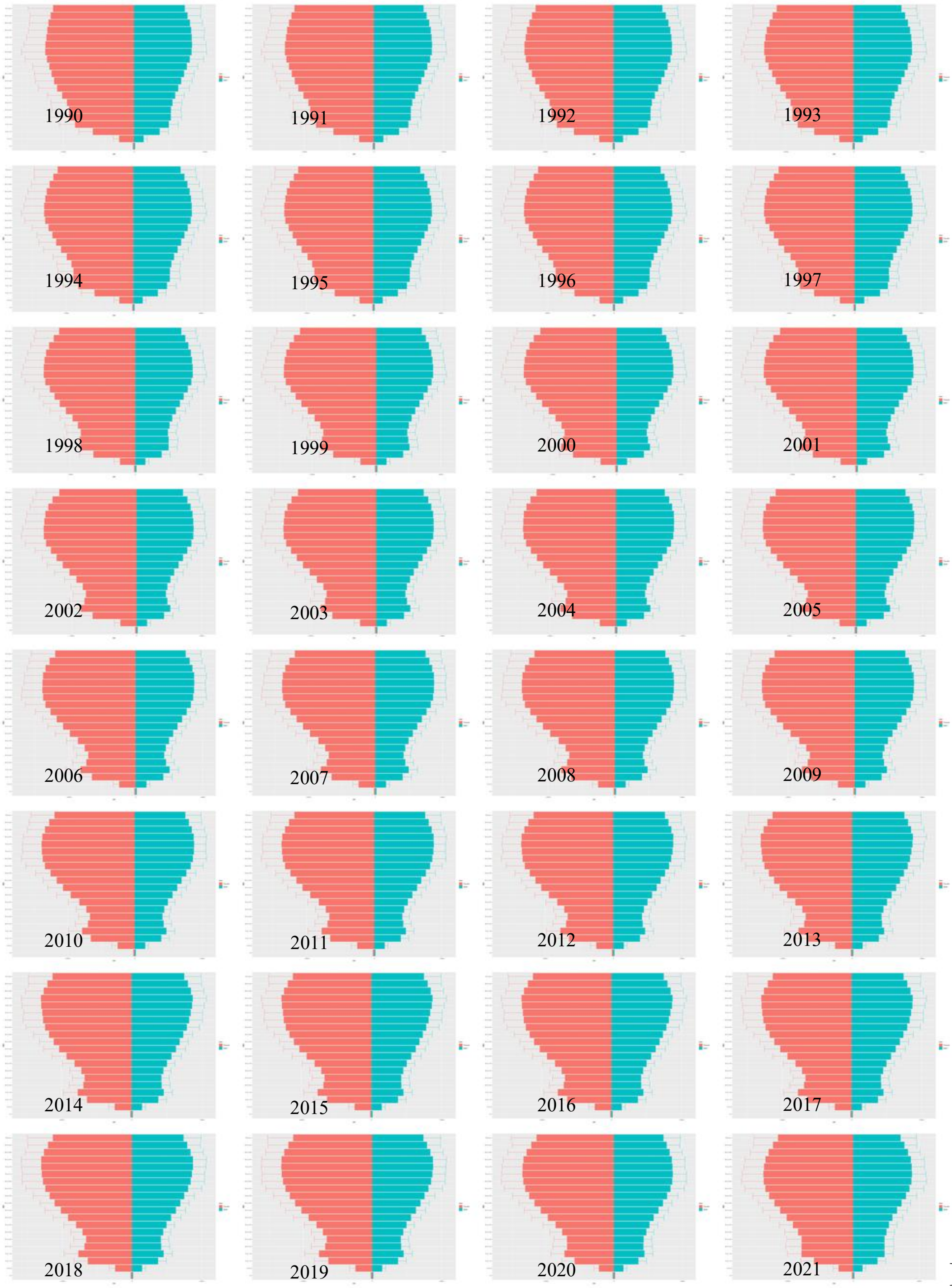
1990
1991
1992
1993
1994
1995
1996
1997
1998
1999
2000
2001
2002
2003
2004
2005
2006
2007
2008
2009
2010
2011
2012
2013
2014
2015
2016
2017
2018
2019
2020
2021

B

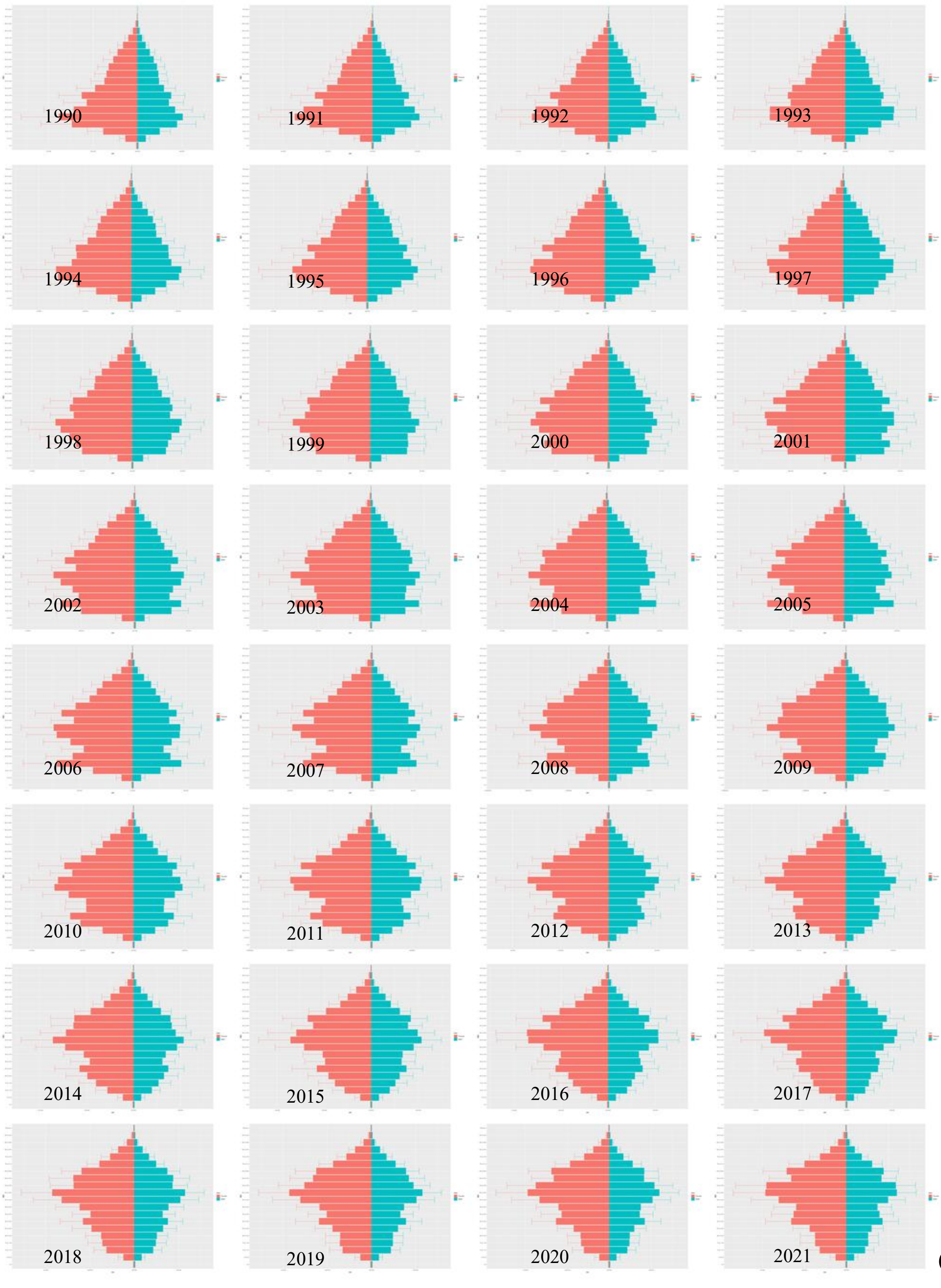
1990
1991
1992
1993
1994
1995
1996
1997
1998
1999
2000
2001
2002
2003
2004
2005
2006
2007
2008
2009
2010
2011
2012
2013
2014
2015
2016
2017
2018
2019
2020
2021

C

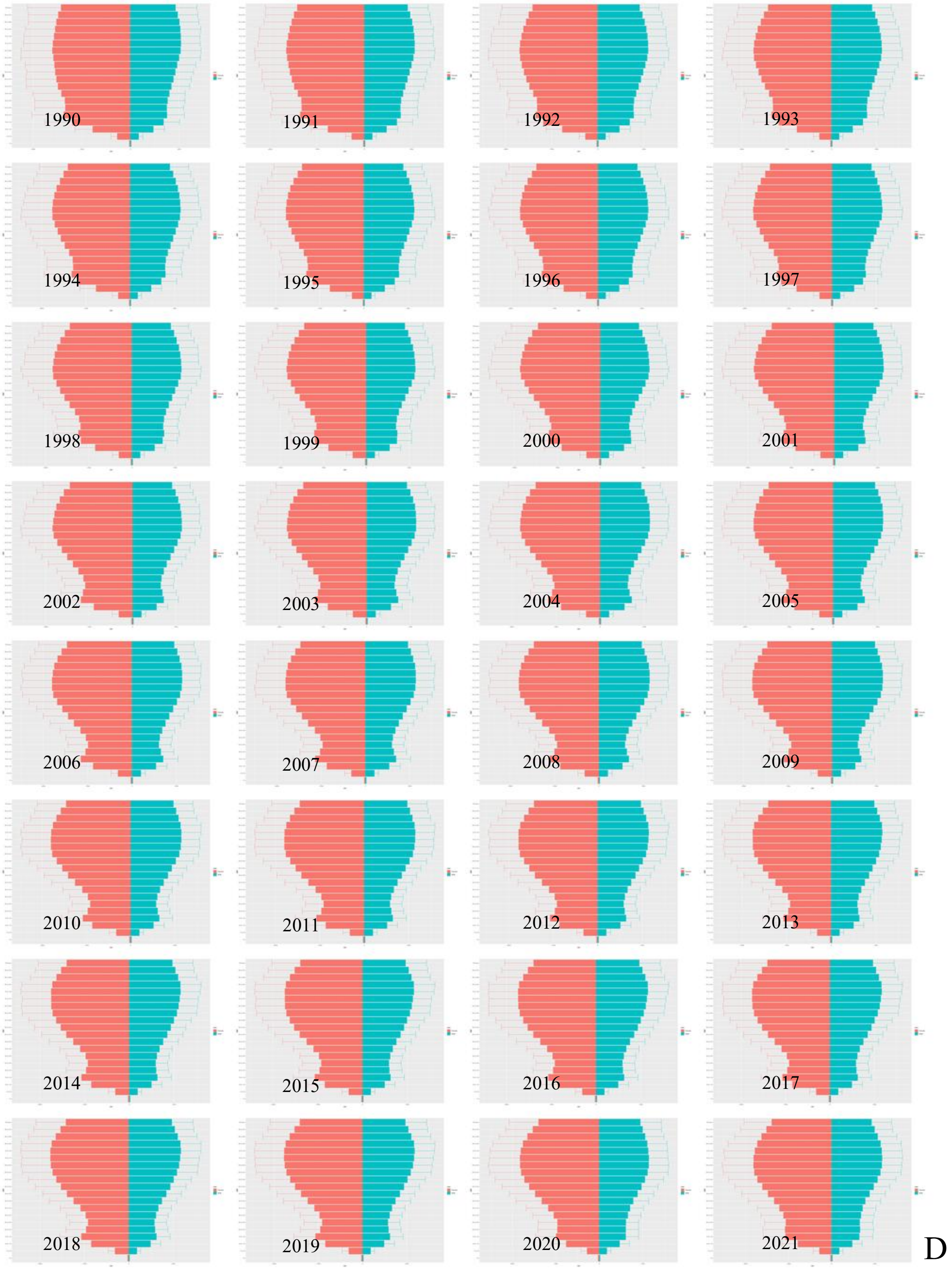

Fig. S61 (A) The prevalence cases of mental disorders in different ages from 1990 to 2021 in China; (B) The prevalence rates of mental disorders in different ages from 1990 to 2021 in China; (C) The years lived with disability of mental disorders in different ages from 1990 to 2021 in China; (D) The years lived with disability rates of mental disorders in different ages from 1990 to 2021 in China.

Notes: red for female, green for male; the ordinate from bottom to top is "<5", "5 to 9", "10 to 14", "15 to 19", "20 to 24", "25 to 29", "30 to 34", "35 to 39", "40 to 44", "45 to 49", "50 to 54", "55 to 59", "60 to 64", "65 to 69", "70 to 74", "75 to 79", "80 to 84", "85 to 89", "90 to 94", "95 plus".

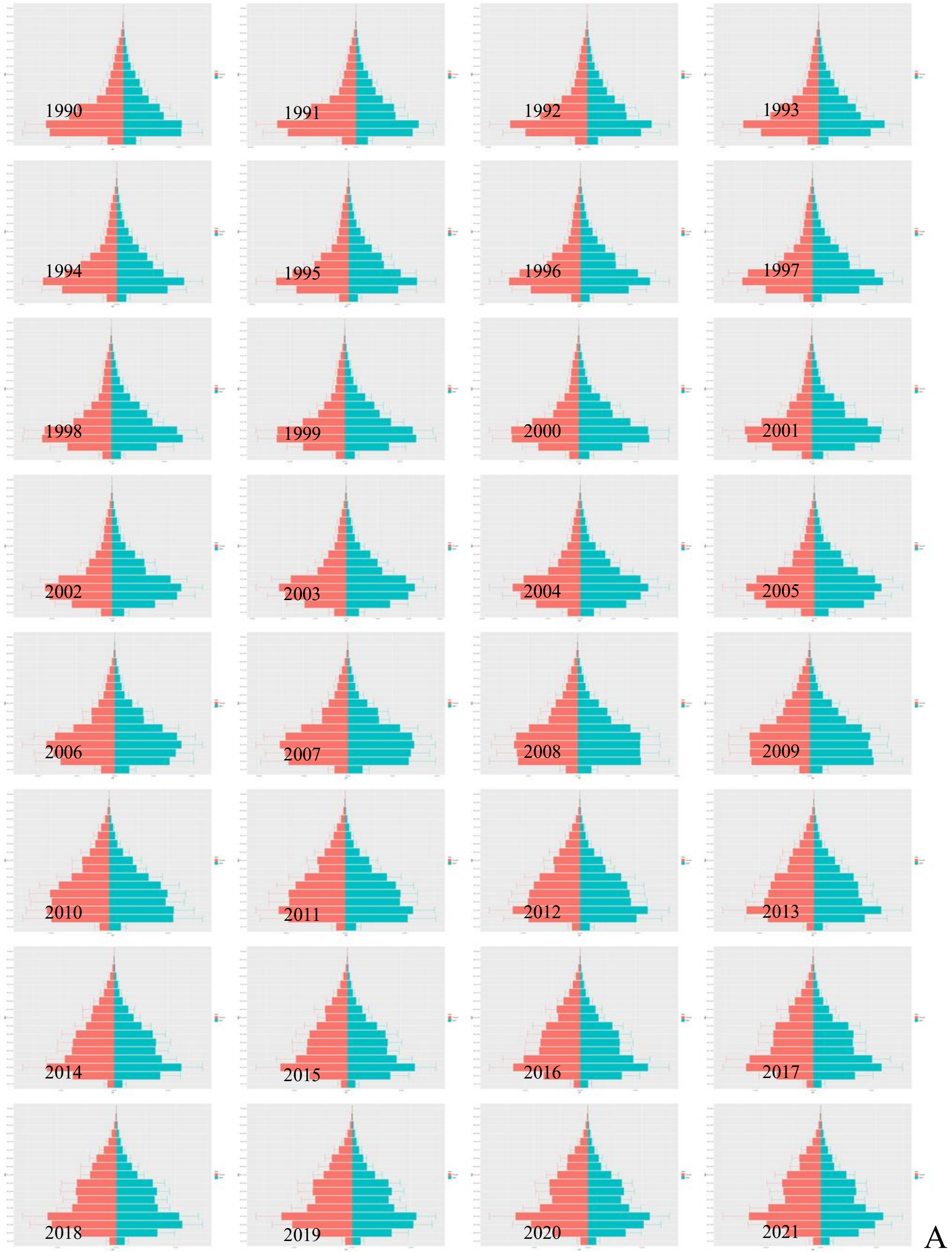
1990
1991
1992
1993
1994
1995
1996
1997
1998
1999
2000
2001
2002
2003
2004
2005
2006
2007
2008
2009
2010
2011
2012
2013
2014
2015
2016
2017
2018
2019
2020
2021
A

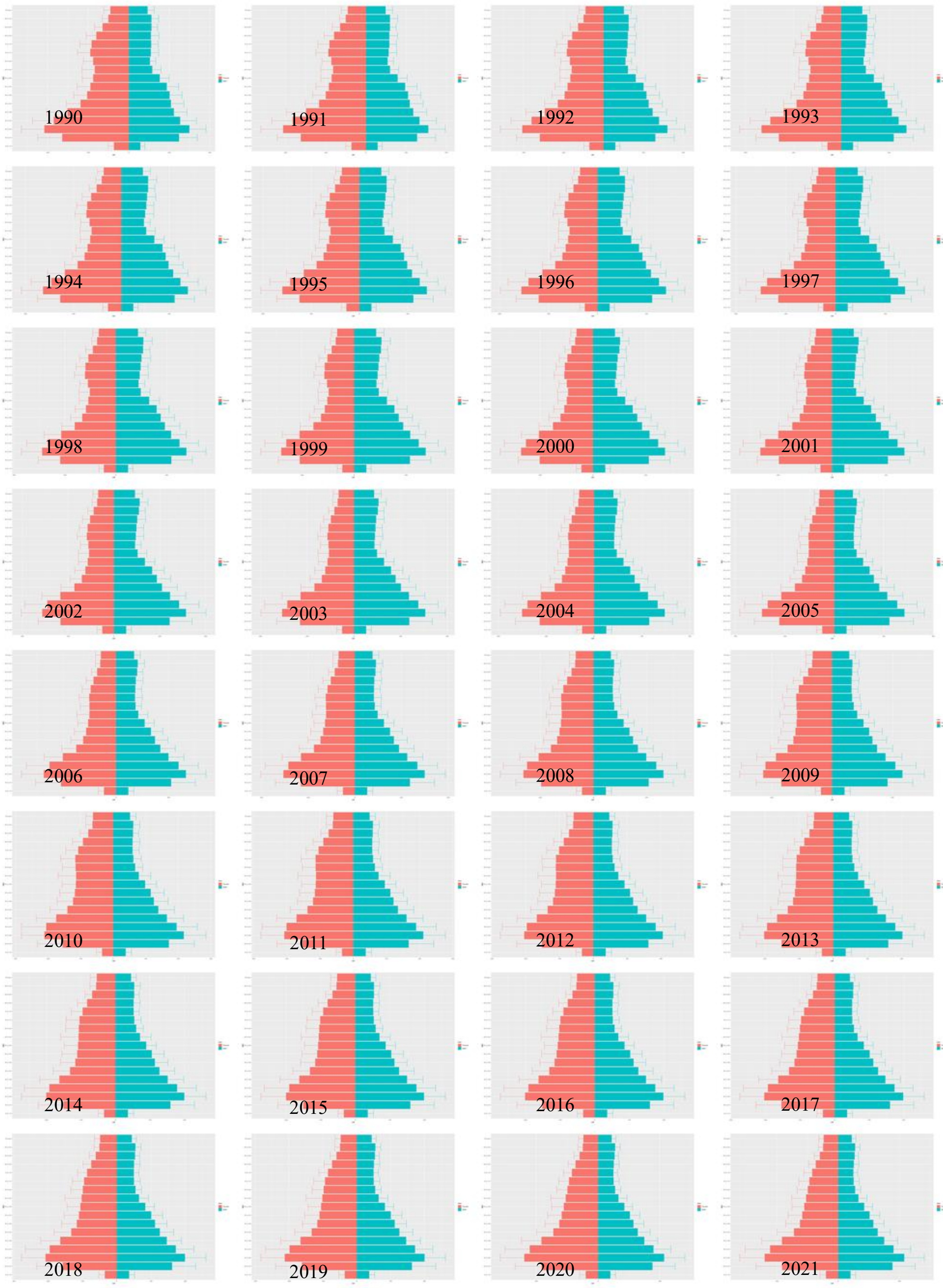
1990
1991
1992
1993
1994
1995
1996
1997
1998
1999
2000
2001
2002
2003
2004
2005
2006
2007
2008
2009
2010
2011
2012
2013
2014
2015
2016
2017
2018
2019
2020
2021

B

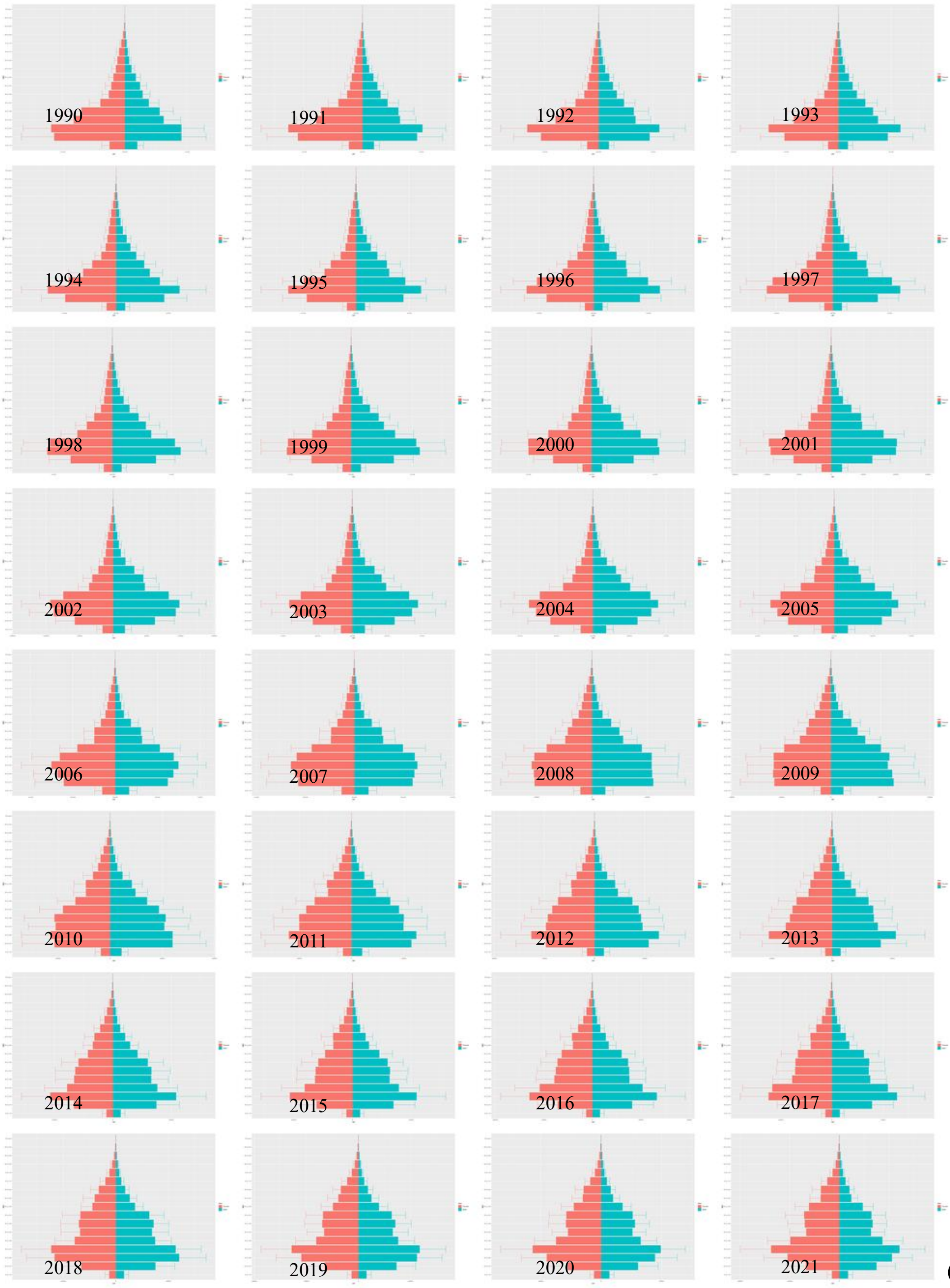
1990
1991
1992
1993
1994
1995
1996
1997
1998
1999
2000
2001
2002
2003
2004
2005
2006
2007
2008
2009
2010
2011
2012
2013
2014
2015
2016
2017
2018
2019
2020
2021

C

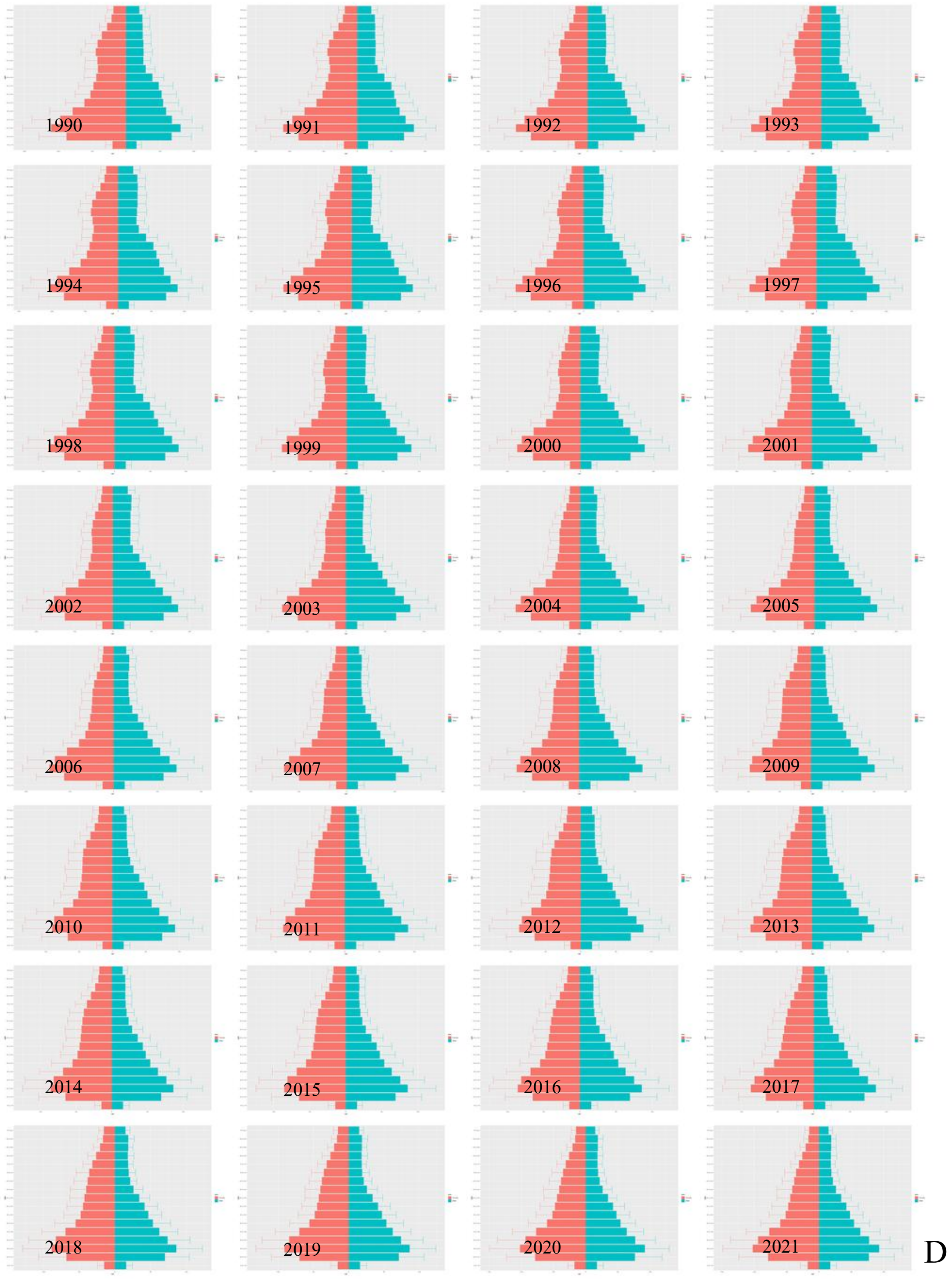

Fig. S62 (A) The prevalence cases of substance use disorders in different ages from 1990 to 2021 in China; (B) The prevalence rates of substance use disorders in different ages from 1990 to 2021 in China; (C) The years lived with disability of substance use disorders in different ages from 1990 to 2021 in China; (D) The years lived with disability rates of substance use disorders in different ages from 1990 to 2021 in China.

Notes: red for female, green for male; the ordinate from bottom to top is "15 to 19", "20 to 24", "25 to 29", "30 to 34", "35 to 39", "40 to 44", "45 to 49", "50 to 54", "55 to 59", "60 to 64", "65 to 69", "70 to 74", "75 to 79", "80 to 84", "85 to 89", "90 to 94", "95 plus".

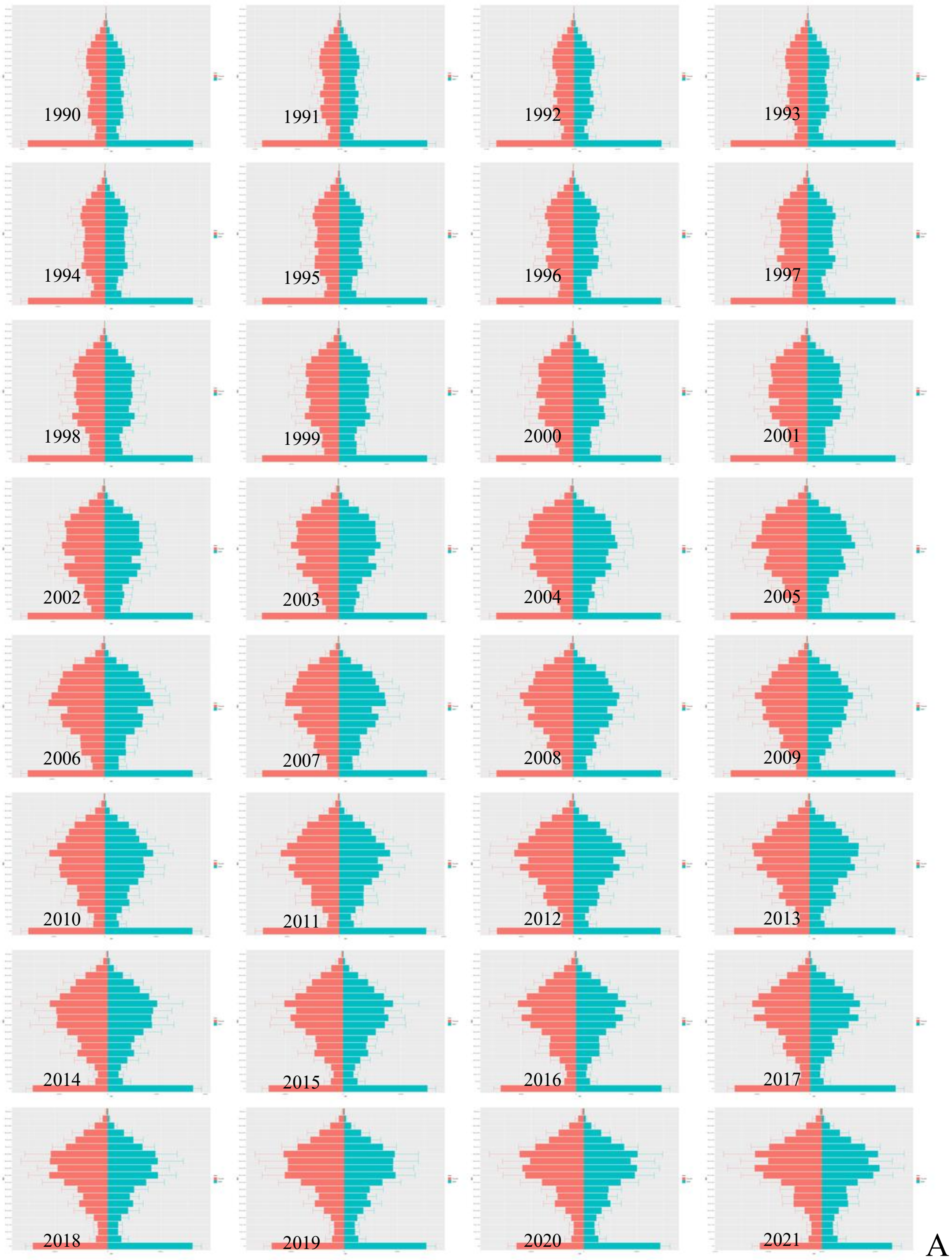
1990
1991
1992
1993
1994
1995
1996
1997
1998
1999
2000
2001
2002
2003
2004
2005
2006
2007
2008
2009
2010
2011
2012
2013
2014
2015
2016
2017
2018
2019
2020
2021
A

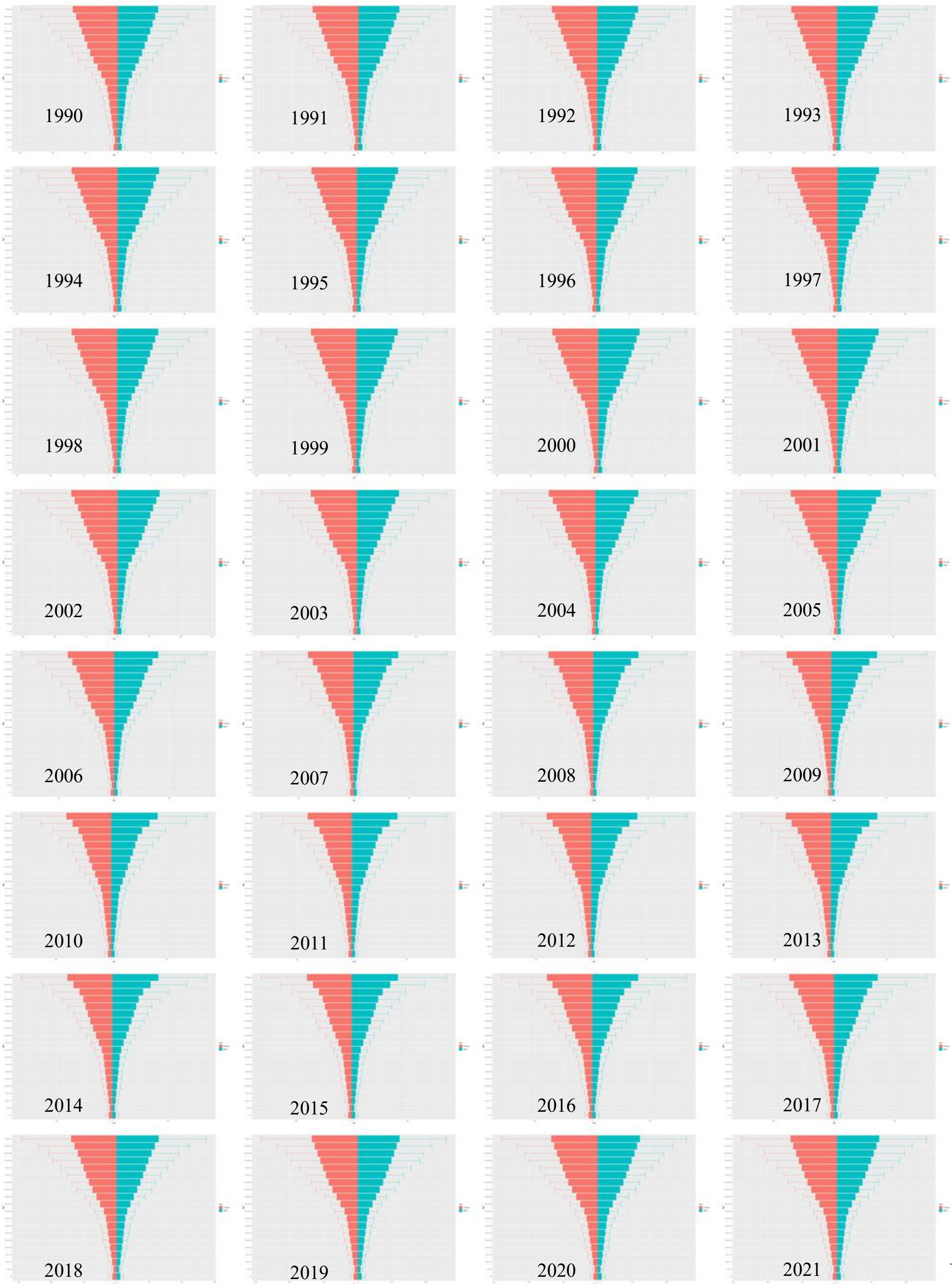
1990
1991
1992
1993
1994
1995
1996
1997
1998
1999
2000
2001
2002
2003
2004
2005
2006
2007
2008
2009
2010
2011
2012
2013
2014
2015
2016
2017
2018
2019
2020
2021

B

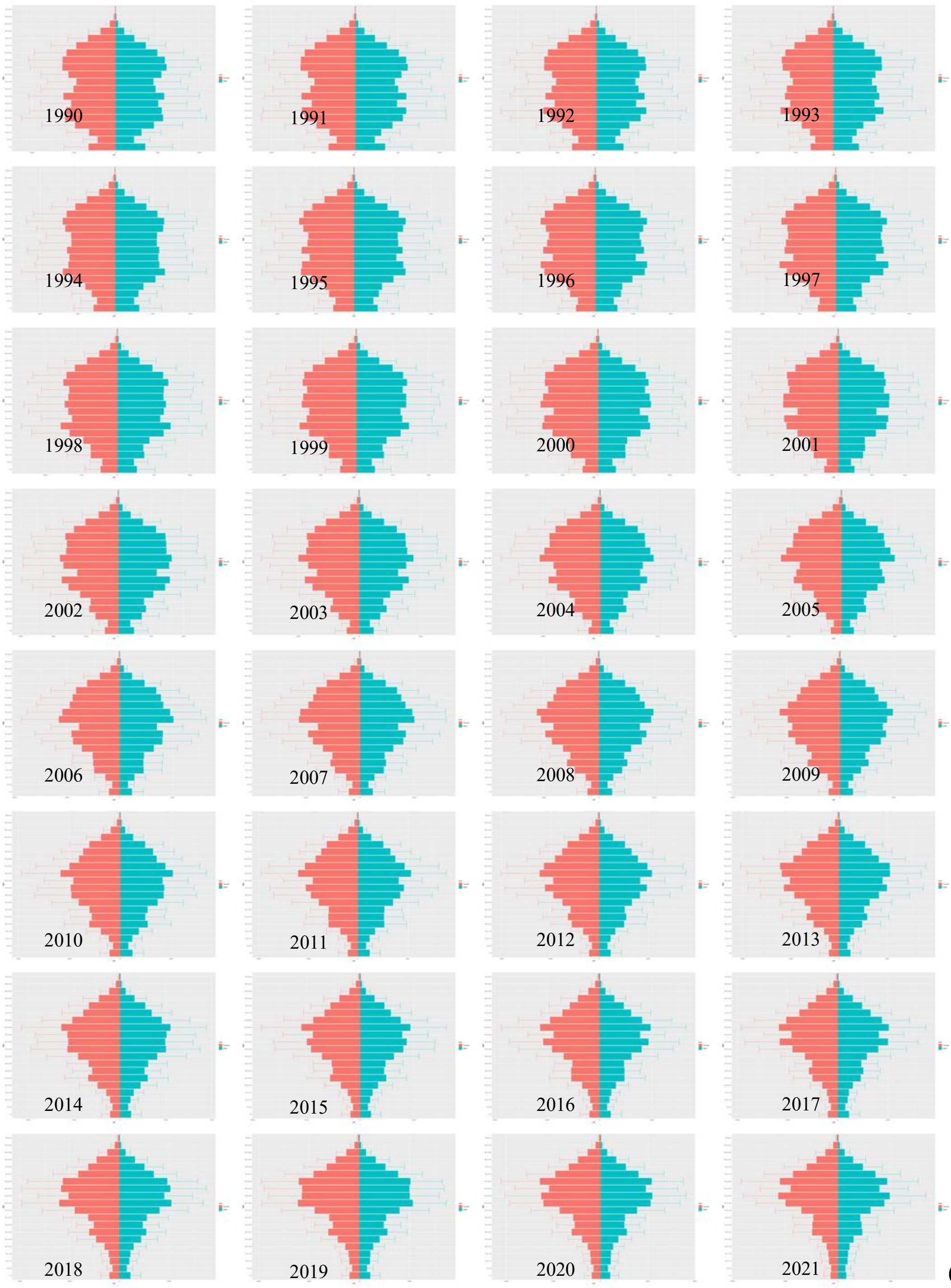
1990
1991
1992
1993
1994
1995
1996
1997
1998
1999
2000
2001
2002
2003
2004
2005
2006
2007
2008
2009
2010
2011
2012
2013
2014
2015
2016
2017
2018
2019
2020
2021
C

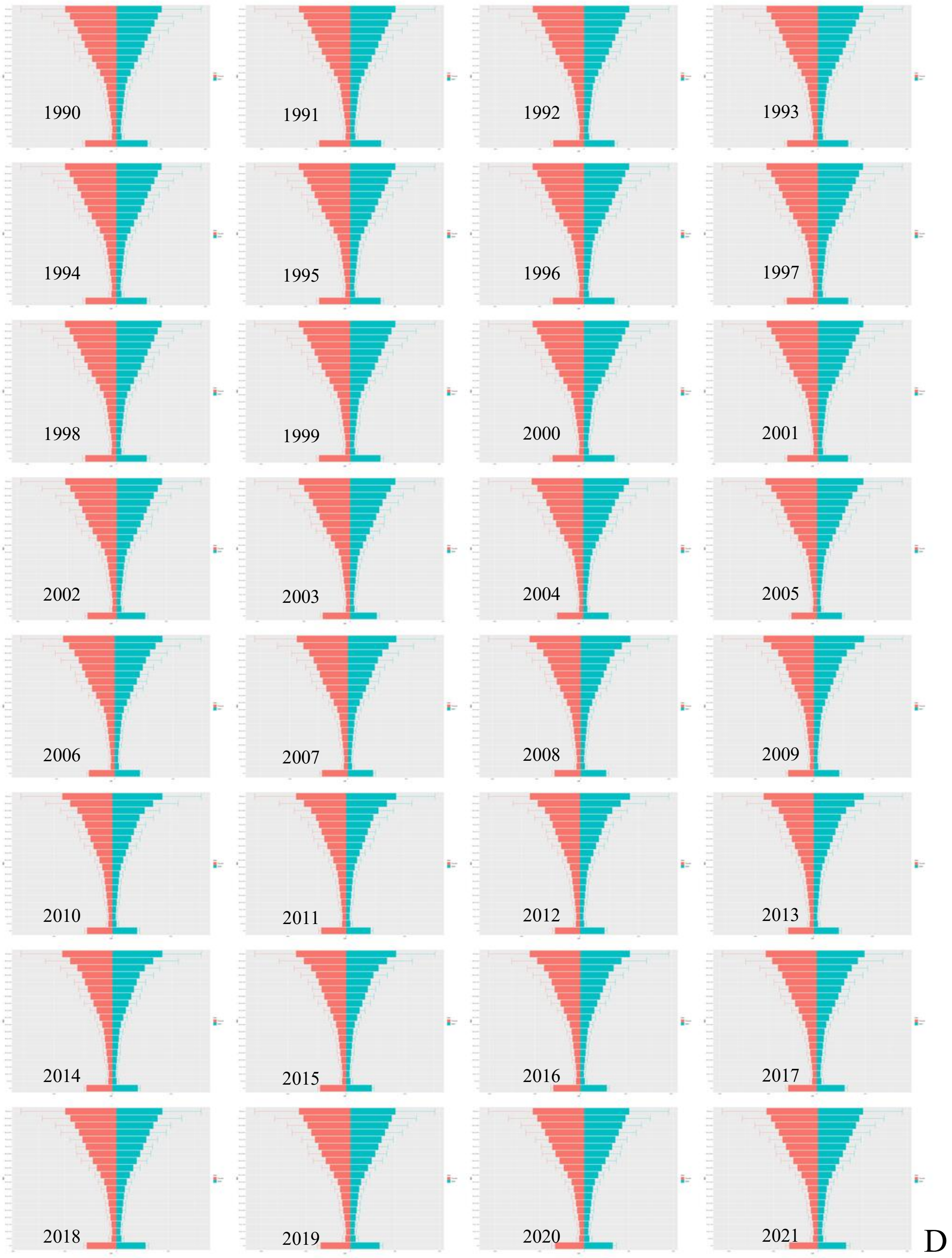

Fig. S63 (A) The prevalence cases of infectious disorders in different ages from 1990 to 2021 in China; (B) The prevalence rates of infectious disorders in different ages from 1990 to 2021 in China; (C) The years lived with disability of infectious disorders in different ages from 1990 to 2021 in China; (D) The years lived with disability rates of infectious disorders in different ages from 1990 to 2021 in China. Notes: red for female, green for male; the ordinate from bottom to top is "<5", "5 to 9", "10 to 14", "15 to 19", "20 to 24", "25 to 29", "30 to 34", "35 to 39", "40 to 44", "45 to 49", "50 to 54", "55 to 59", "60 to 64", "65 to 69", "70 to 74", "75 to 79", "80 to 84", "85 to 89", "90 to 94", "95 plus".

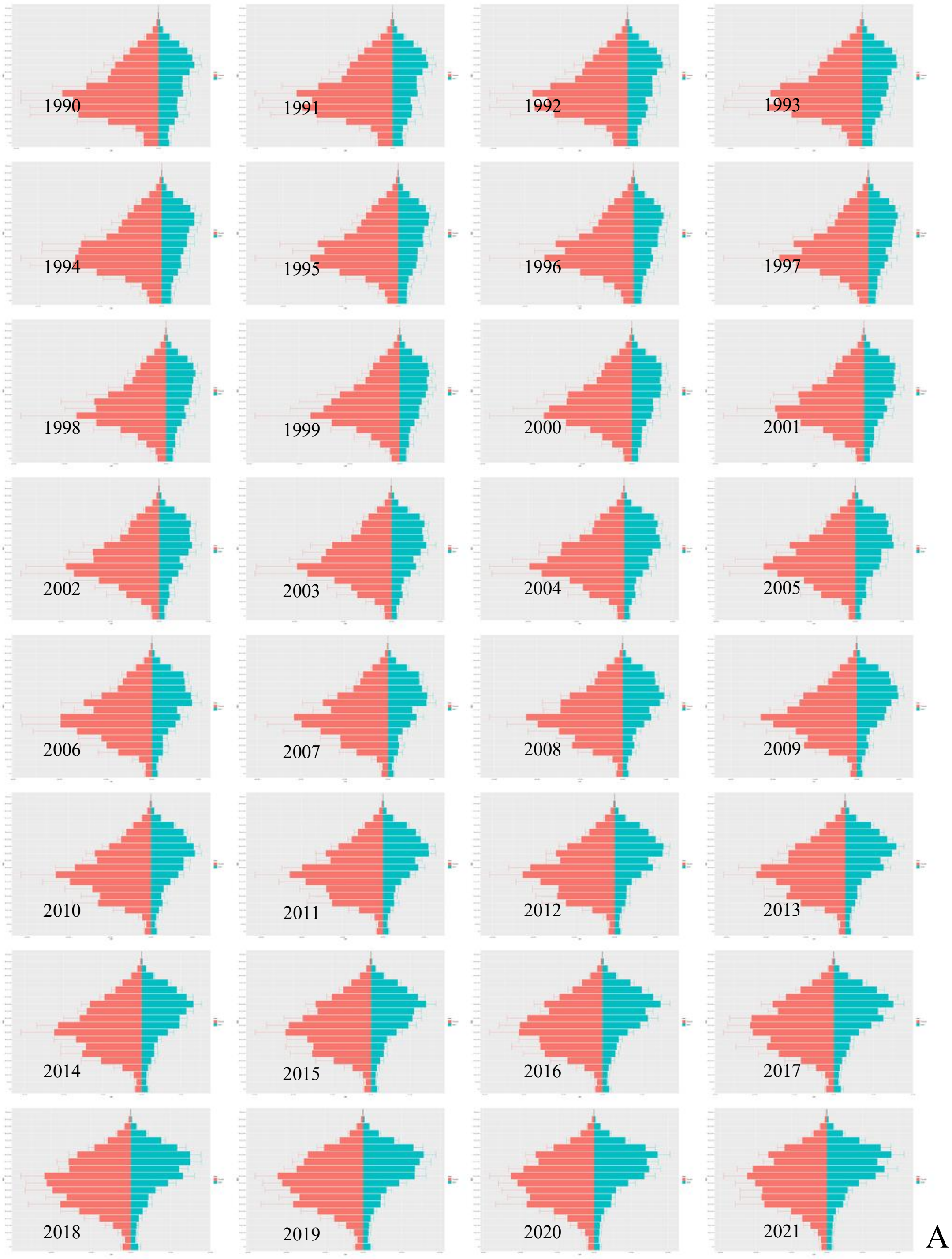
1990
1991
1992
1993
1994
1995
1996
1997
1998
1999
2000
2001
2002
2003
2004
2005
2006
2007
2008
2009
2010
2011
2012
2013
2014
2015
2016
2017
2018
2019
2020
2021
A

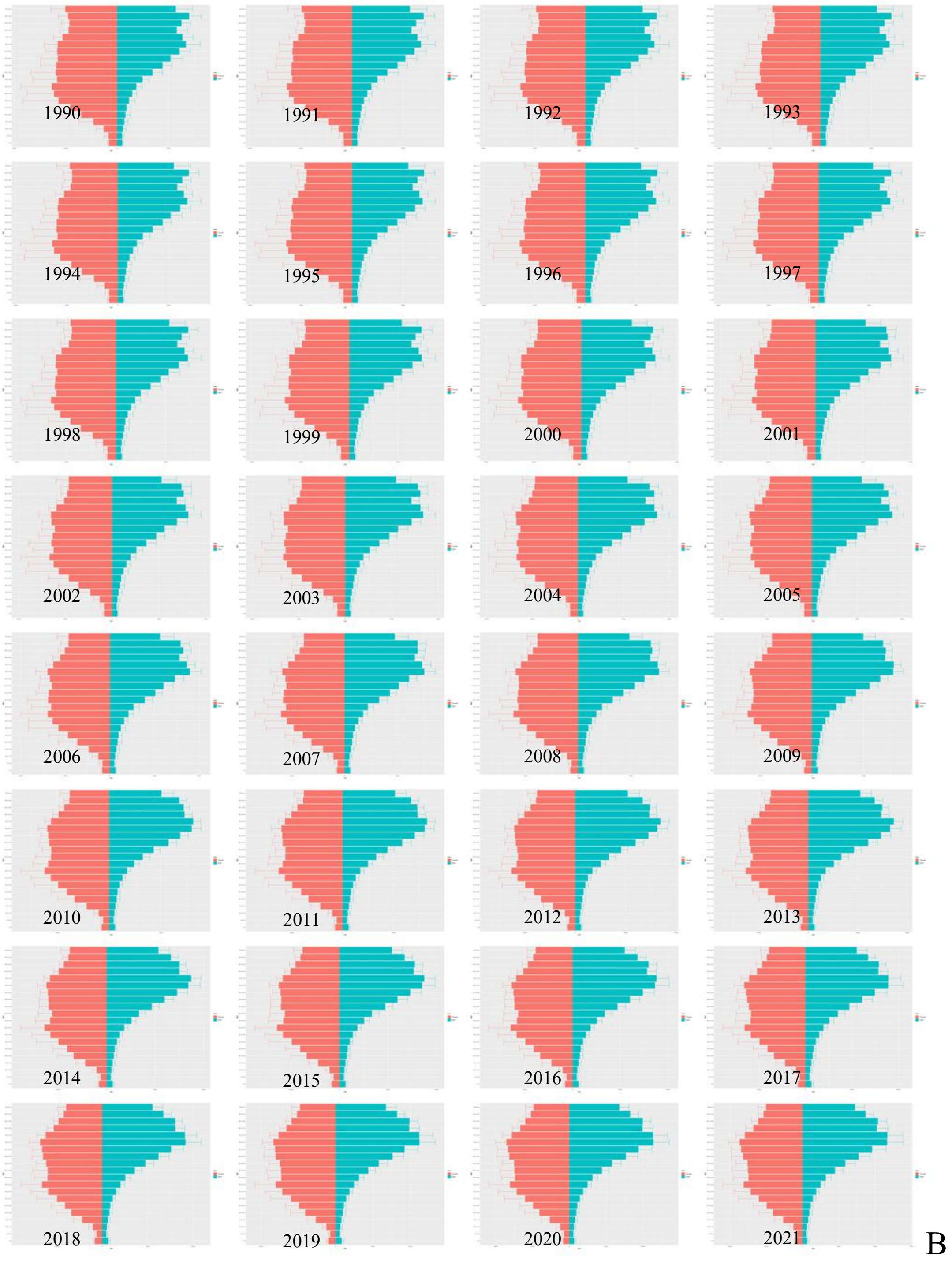
1990
1991
1992
1993
1994
1995
1996
1997
1998
1999
2000
2001
2002
2003
2004
2005
2006
2007
2008
2009
2010
2011
2012
2013
2014
2015
2016
2017
2018
2019
2020
2021
B

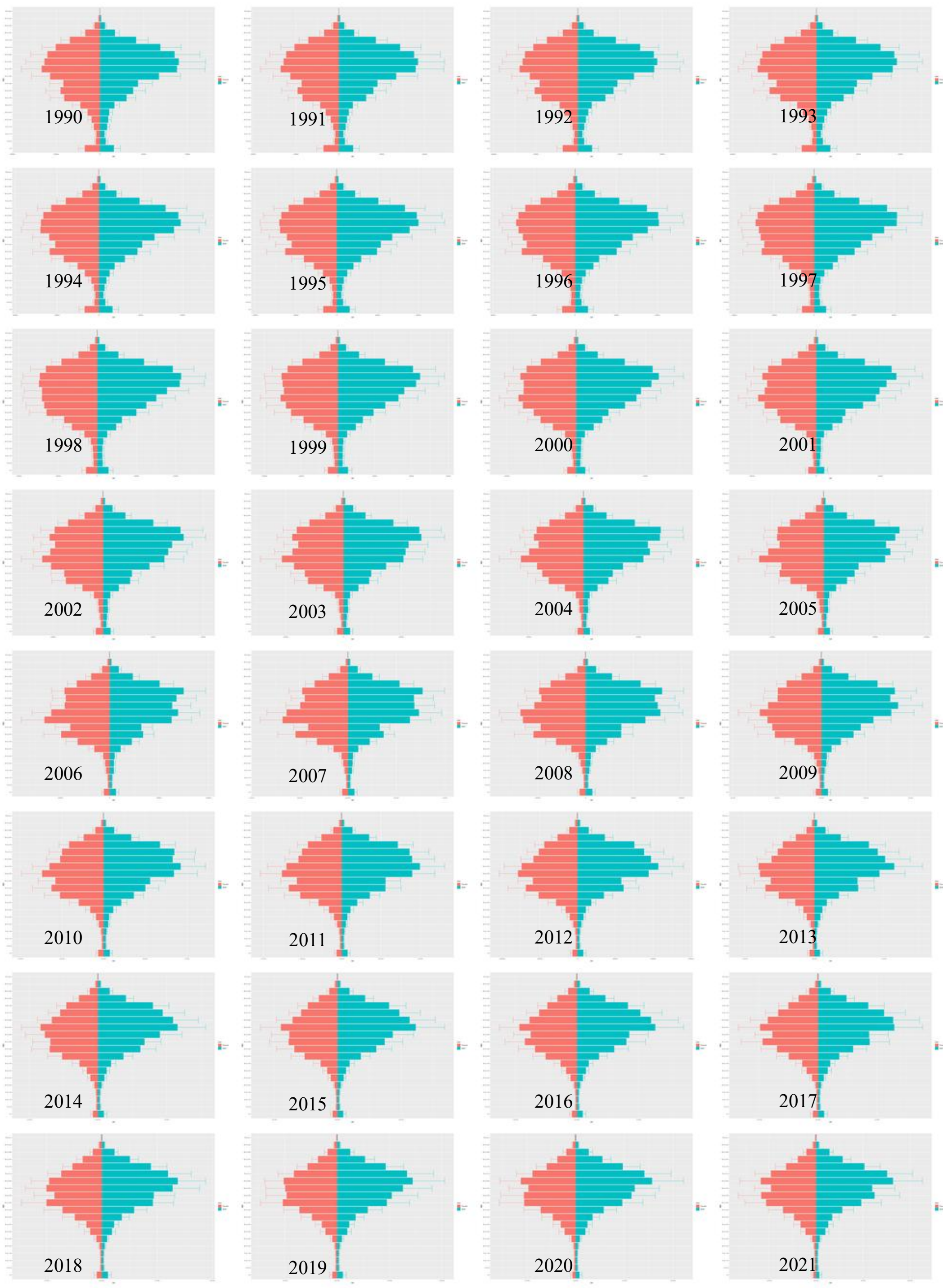
1990
1991
1992
1993
1994
1995
1996
1997
1998
1999
2000
2001
2002
2003
2004
2005
2006
2007
2008
2009
2010
2011
2012
2013
2014
2015
2016
2017
2018
2019
2020
2021

C

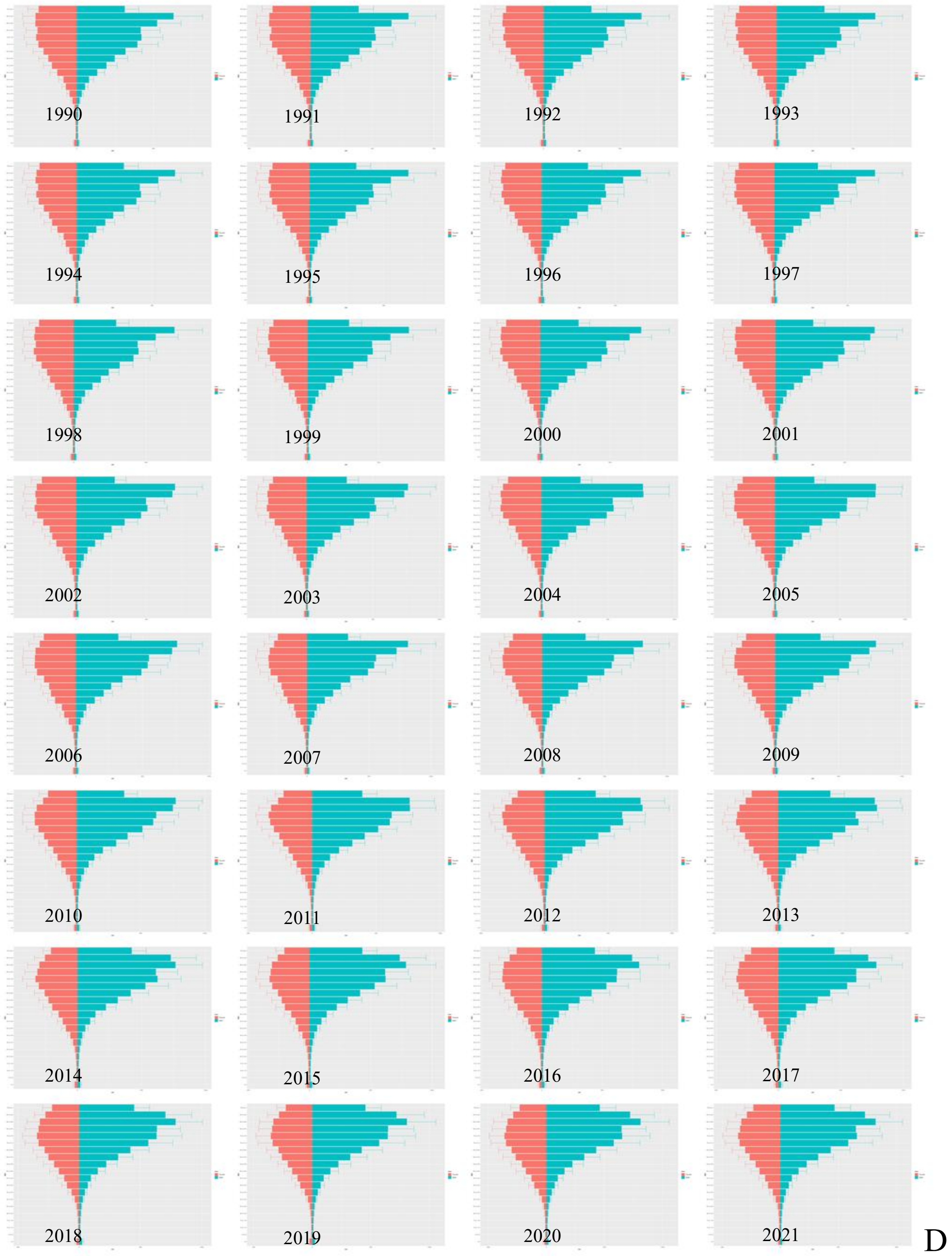

Fig. S64 (A) The prevalence cases of neoplasms in different ages from 1990 to 2021 in China; (B) The prevalence rates of neoplasms in different ages from 1990 to 2021 in China; (C) The years lived with disability of neoplasms in different ages from 1990 to 2021 in China; (D) The years lived with disability rates of neoplasms in different ages from 1990 to 2021 in China.

Notes: red for female, green for male; the ordinate from bottom to top is "<5", "5 to 9", "10 to 14", "15 to 19", "20 to 24", "25 to 29", "30 to 34", "35 to 39", "40 to 44", "45 to 49", "50 to 54", "55 to 59", "60 to 64", "65 to 69", "70 to 74", "75 to 79", "80 to 84", "85 to 89", "90 to 94", "95 plus".

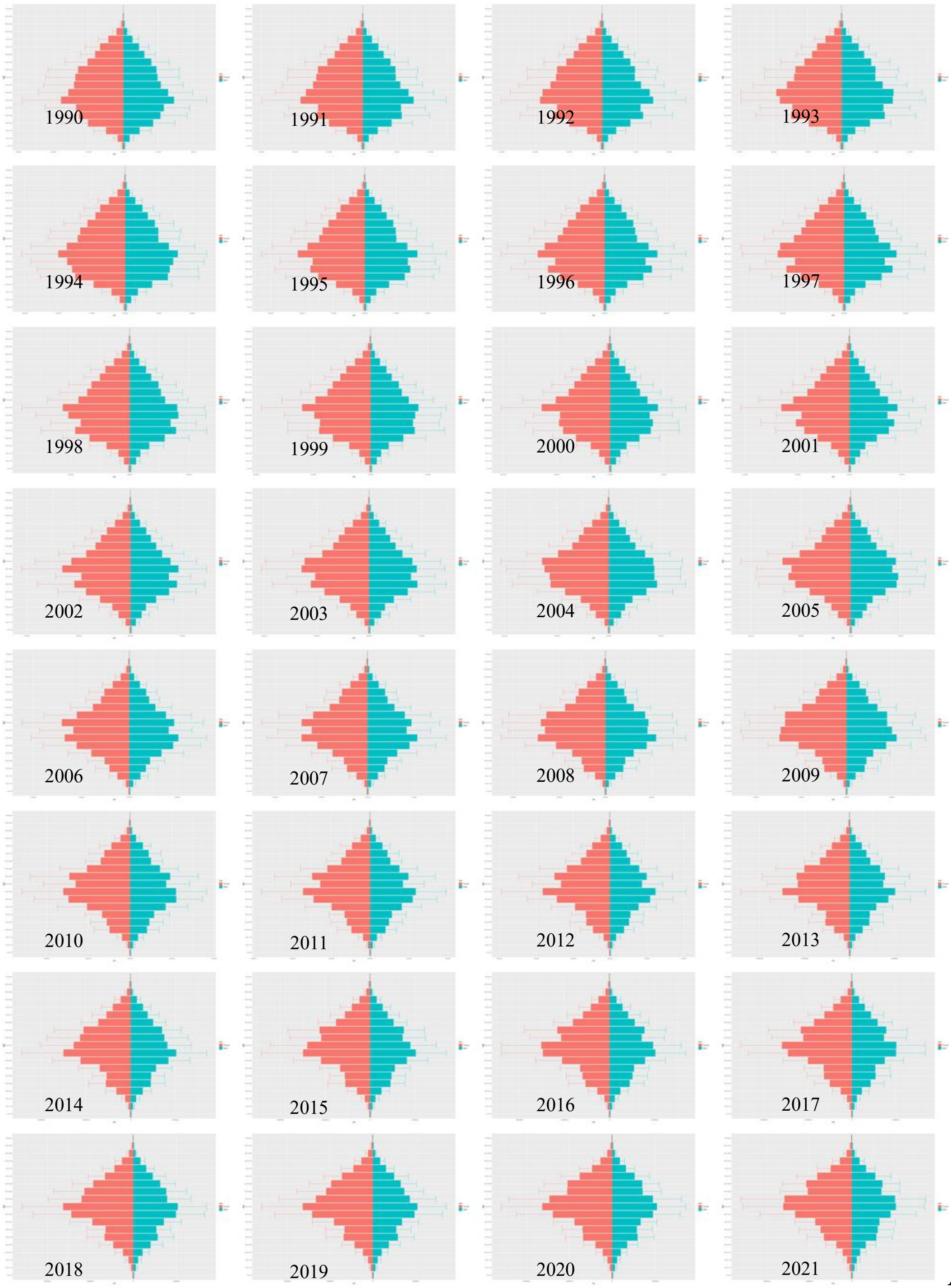
1990
1991
1992
1993
1994
1995
1996
1997
1998
1999
2000
2001
2002
2003
2004
2005
2006
2007
2008
2009
2010
2011
2012
2013
2014
2015
2016
2017
2018
2019
2020
2021

A

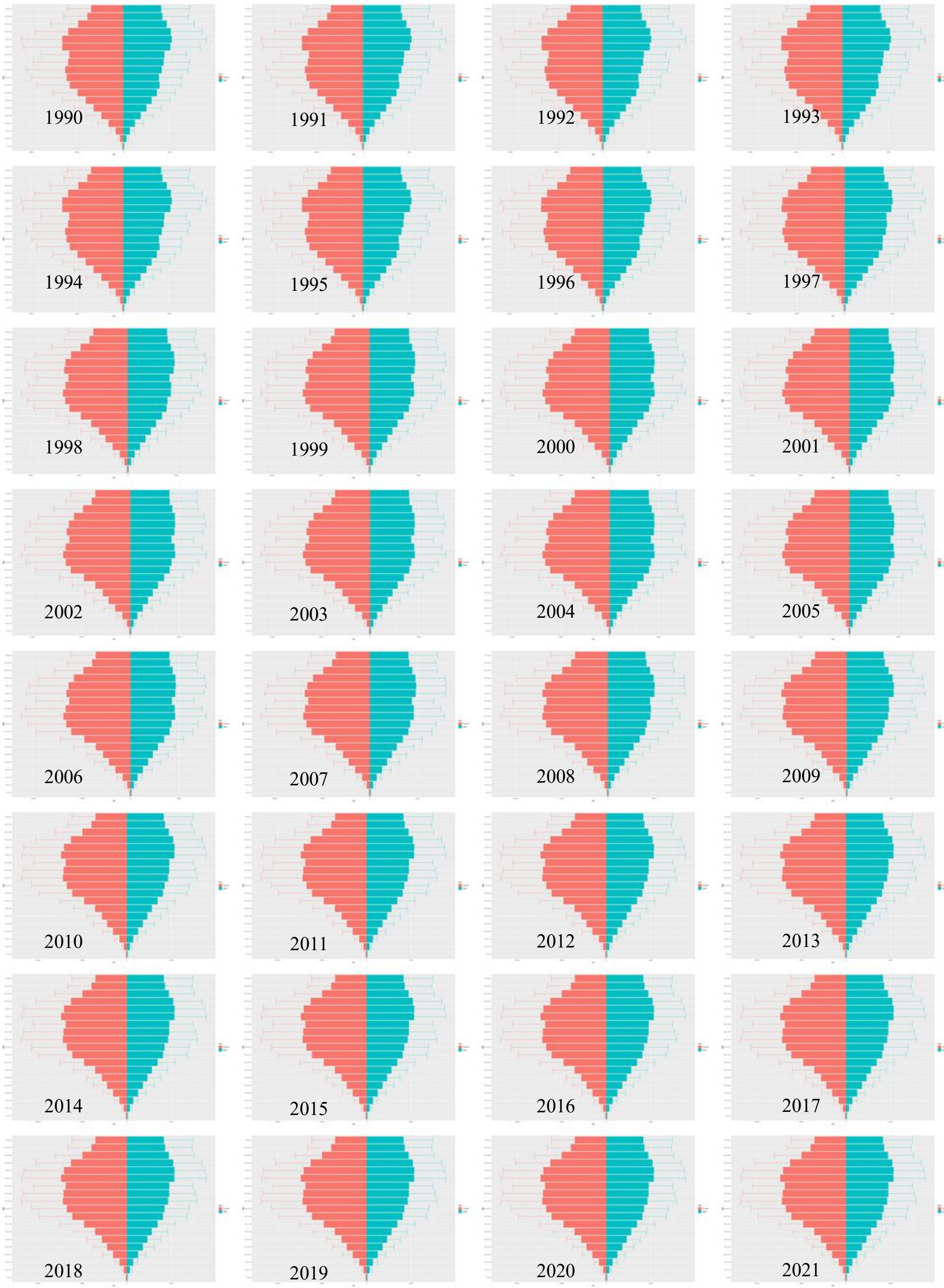
1990
1991
1992
1993
1994
1995
1996
1997
1998
1999
2000
2001
2002
2003
2004
2005
2006
2007
2008
2009
2010
2011
2012
2013
2014
2015
2016
2017
2018
2019
2020
2021

B

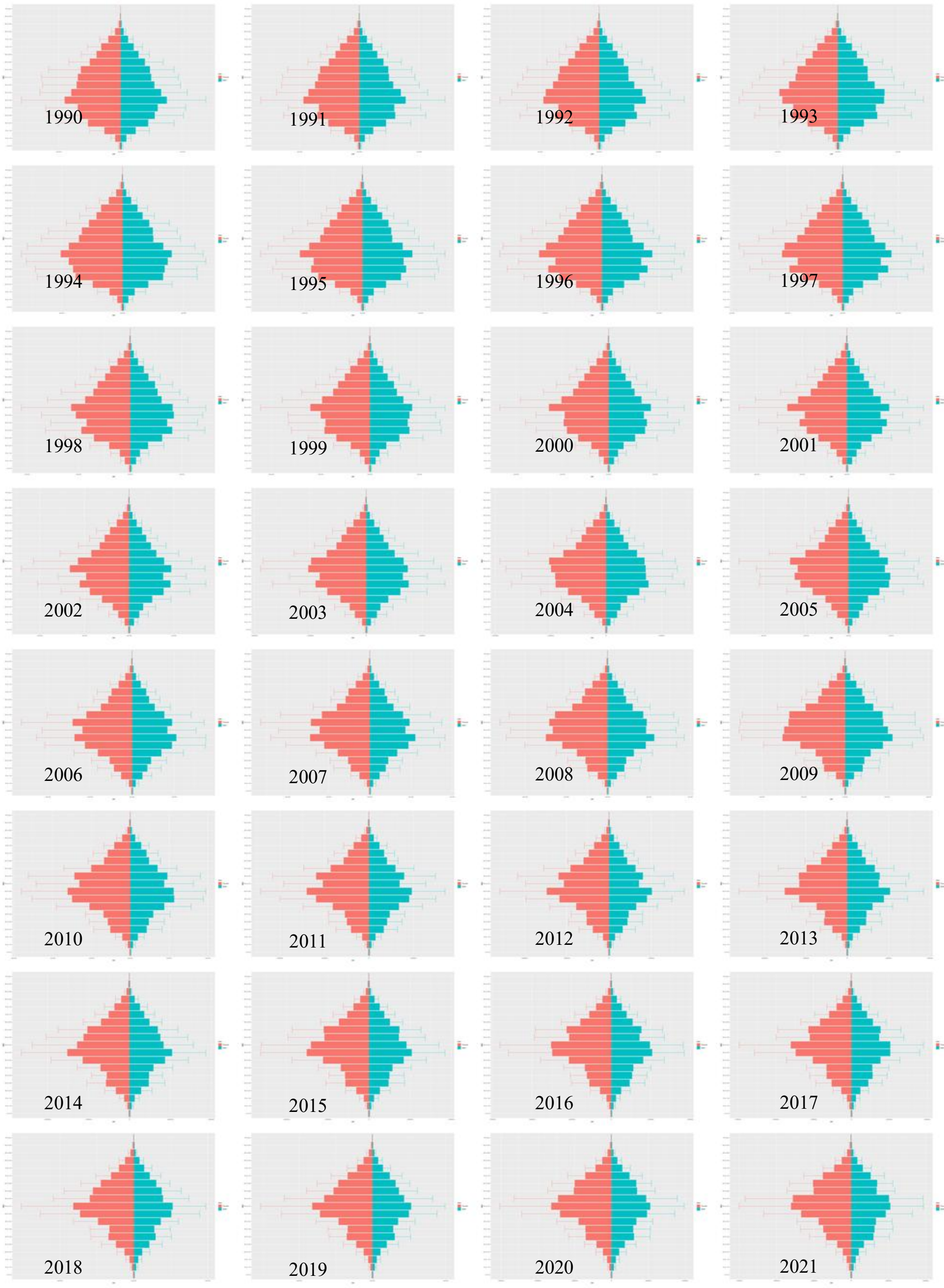
1990
1991
1992
1993
1994
1995
1996
1997
1998
1999
2000
2001
2002
2003
2004
2005
2006
2007
2008
2009
2010
2011
2012
2013
2014
2015
2016
2017
2018
2019
2020
2021

C

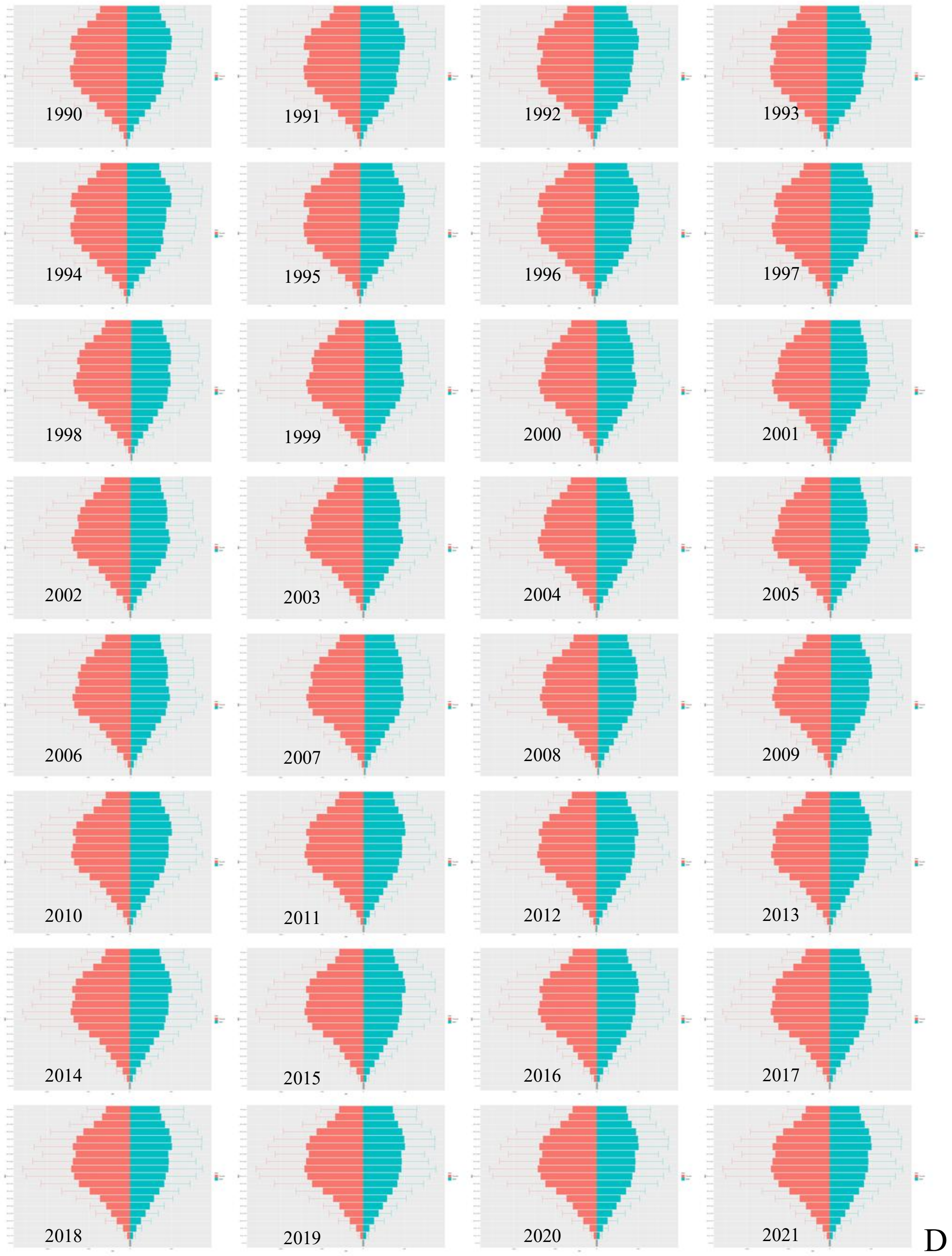

Fig. S65 (A) The prevalence cases of neck pain in different ages from 1990 to 2021 in China; (B) The prevalence rates of neck pain in different ages from 1990 to 2021 in China; (C) The years lived with disability of neck pain in different ages from 1990 to 2021 in China; (D) The years lived with disability rates of neck pain in different ages from 1990 to 2021 in China.

Notes: red for female, green for male; the ordinate from bottom to top is "5 to 9", "10 to 14", "15 to 19", "20 to 24", "25 to 29", "30 to 34", "35 to 39", "40 to 44", "45 to 49", "50 to 54", "55 to 59", "60 to 64", "65 to 69", "70 to 74", "75 to 79", "80 to 84", "85 to 89", "90 to 94", "95 plus".

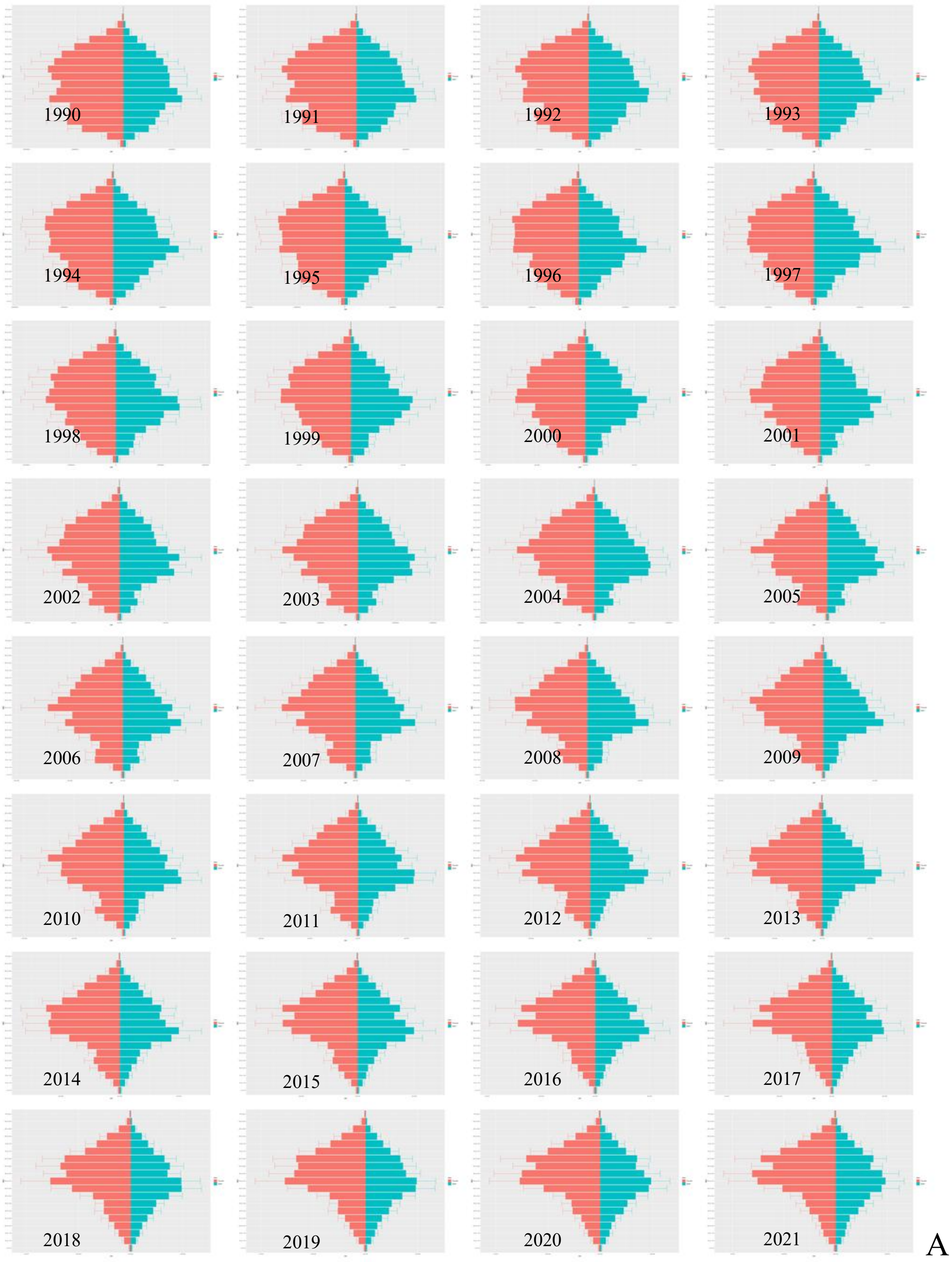
1990
1991
1992
1993
1994
1995
1996
1997
1998
1999
2000
2001
2002
2003
2004
2005
2006
2007
2008
2009
2010
2011
2012
2013
2014
2015
2016
2017
2018
2019
2020
2021
A

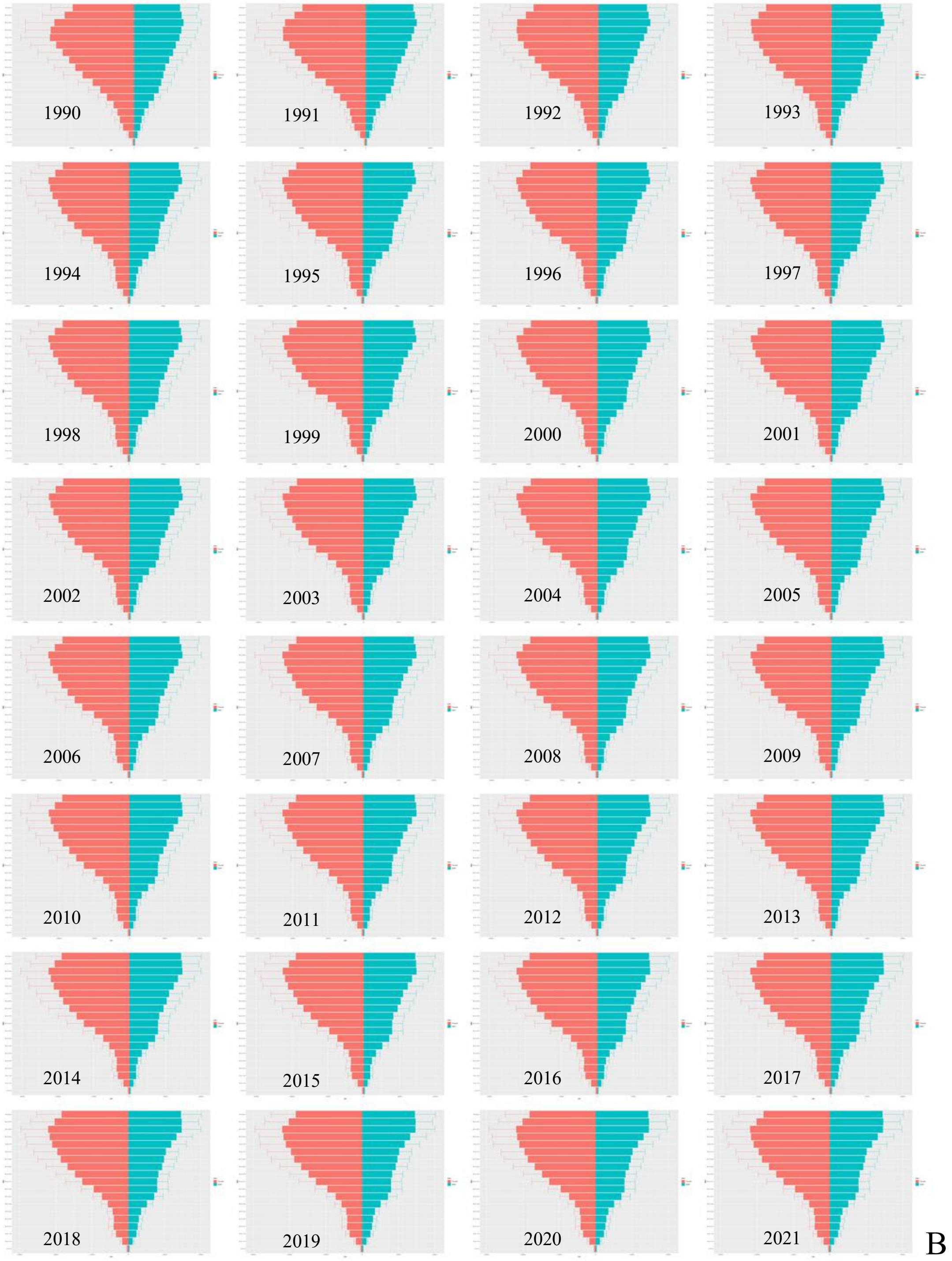
1990
1991
1992
1993
1994
1995
1996
1997
1998
1999
2000
2001
2002
2003
2004
2005
2006
2007
2008
2009
2010
2011
2012
2013
2014
2015
2016
2017
2018
2019
2020
2021
B

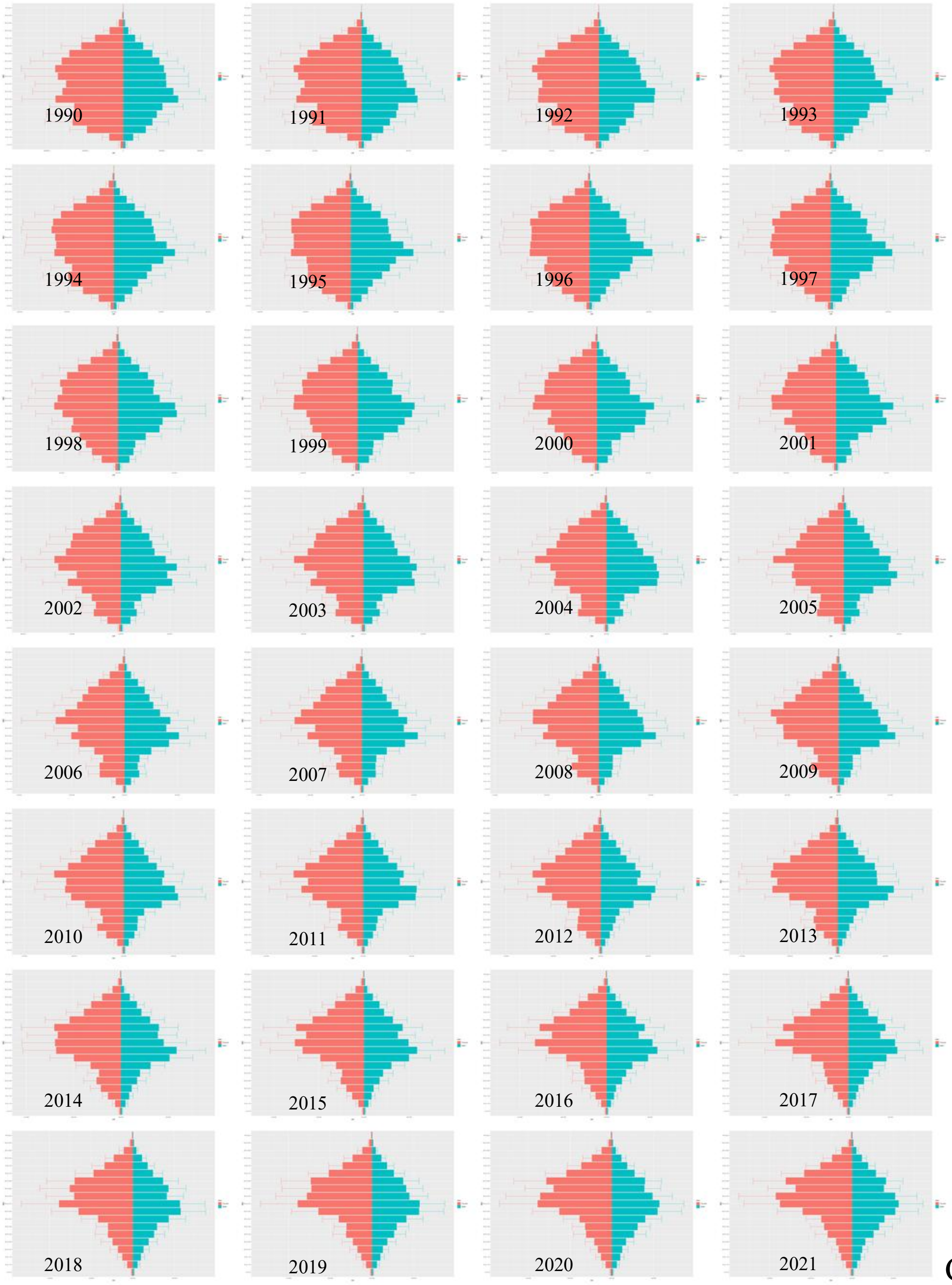
1990
1991
1992
1993
1994
1995
1996
1997
1998
1999
2000
2001
2002
2003
2004
2005
2006
2007
2008
2009
2010
2011
2012
2013
2014
2015
2016
2017
2018
2019
2020
2021

C

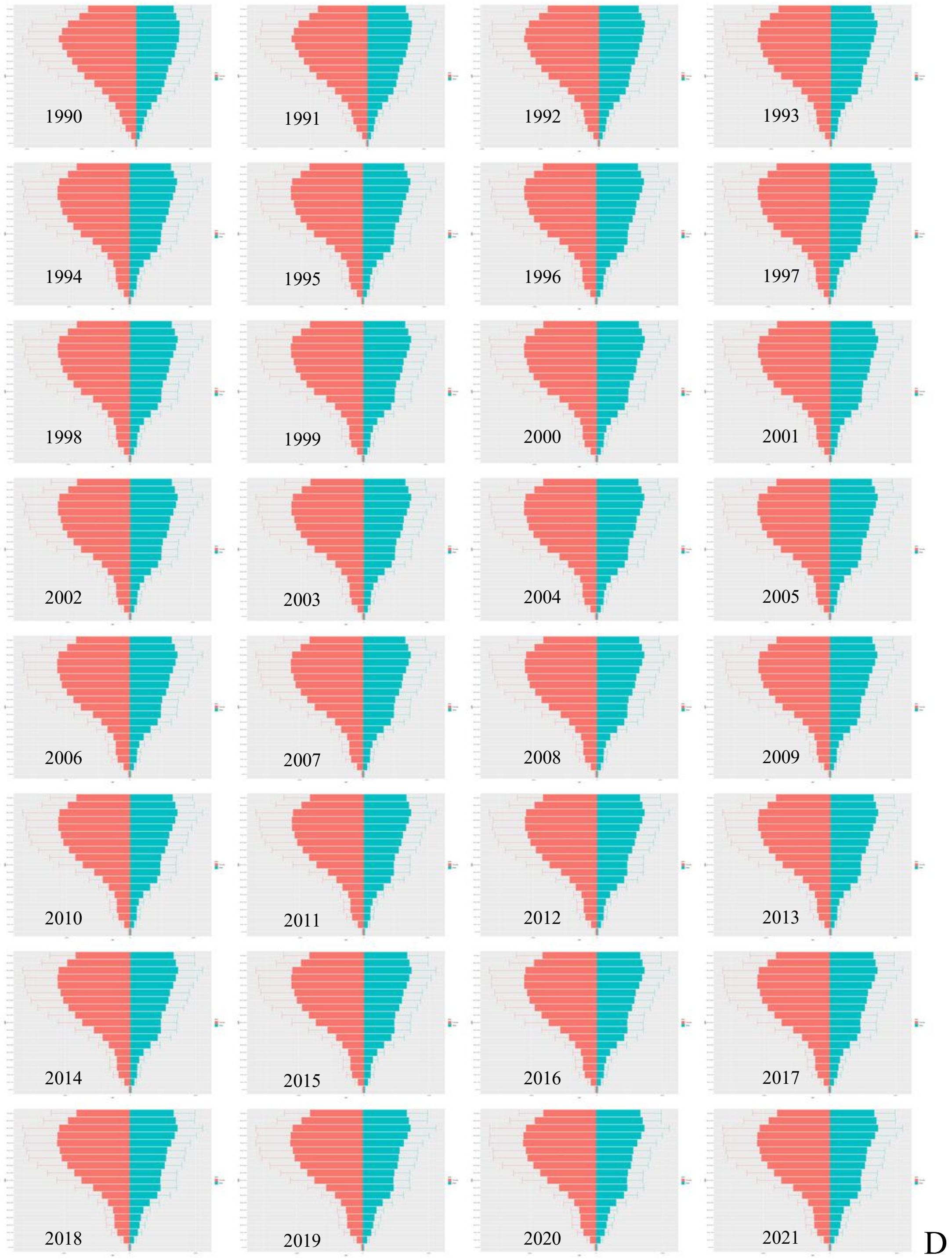

Fig. S66 (A) The prevalence cases of low back pain in different ages from 1990 to 2021 in China; (B) The prevalence rates of low back pain in different ages from 1990 to 2021 in China; (C) The years lived with disability of low back pain in different ages from 1990 to 2021 in China; (D) The years lived with disability rates of low back pain in different ages from 1990 to 2021 in China.

Notes: red for female, green for male; the ordinate from bottom to top is "5 to 9", "10 to 14", "15 to 19", "20 to 24", "25 to 29", "30 to 34", "35 to 39", "40 to 44", "45 to 49", "50 to 54", "55 to 59", "60 to 64", "65 to 69", "70 to 74", "75 to 79", "80 to 84", "85 to 89", "90 to 94", "95 plus".

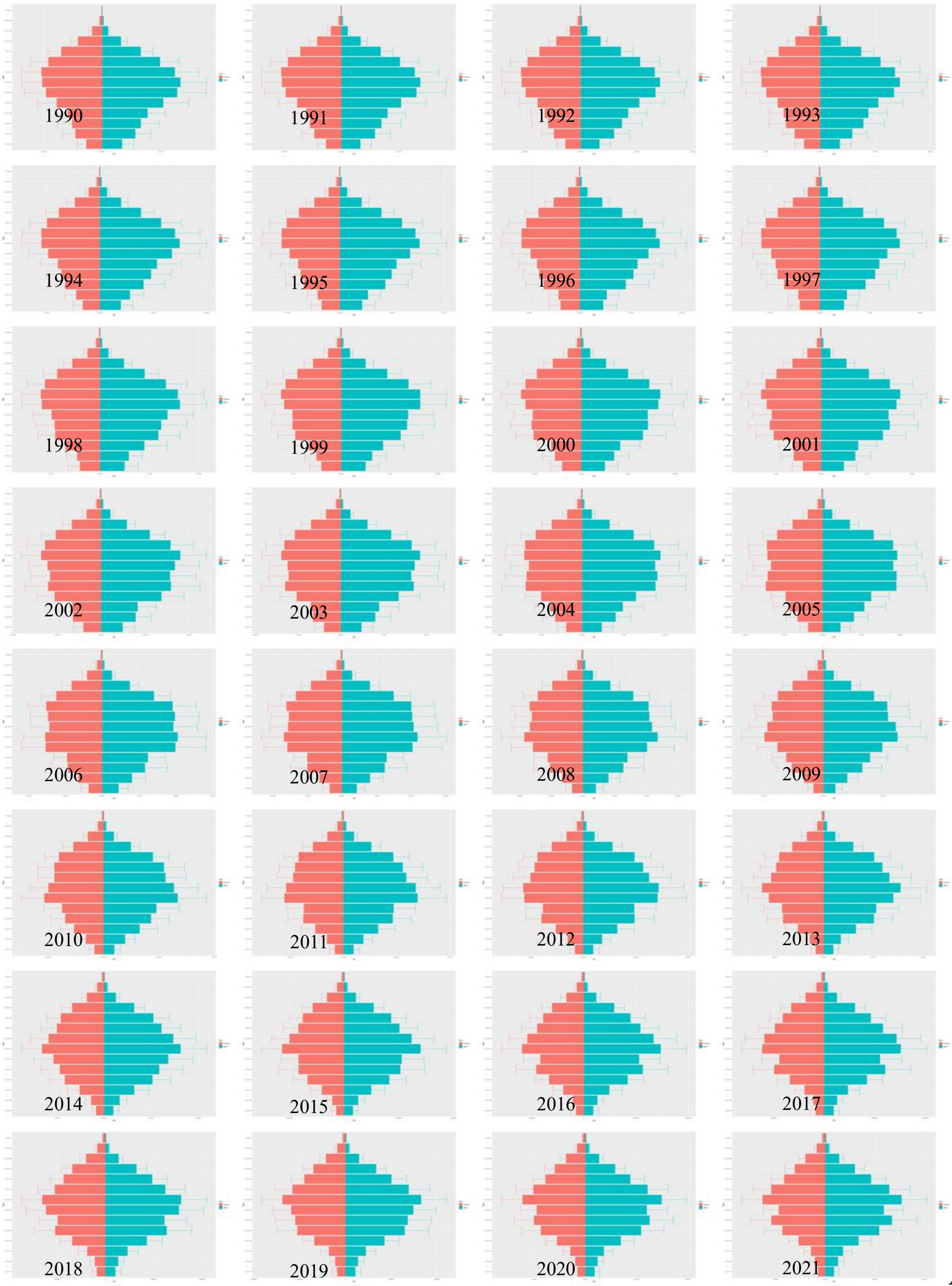
1990
1991
1992
1993
1994
1995
1996
1997
1998
1999
2000
2001
2002
2003
2004
2005
2006
2007
2008
2009
2010
2011
2012
2013
2014
2015
2016
2017
2018
2019
2020
2021

A

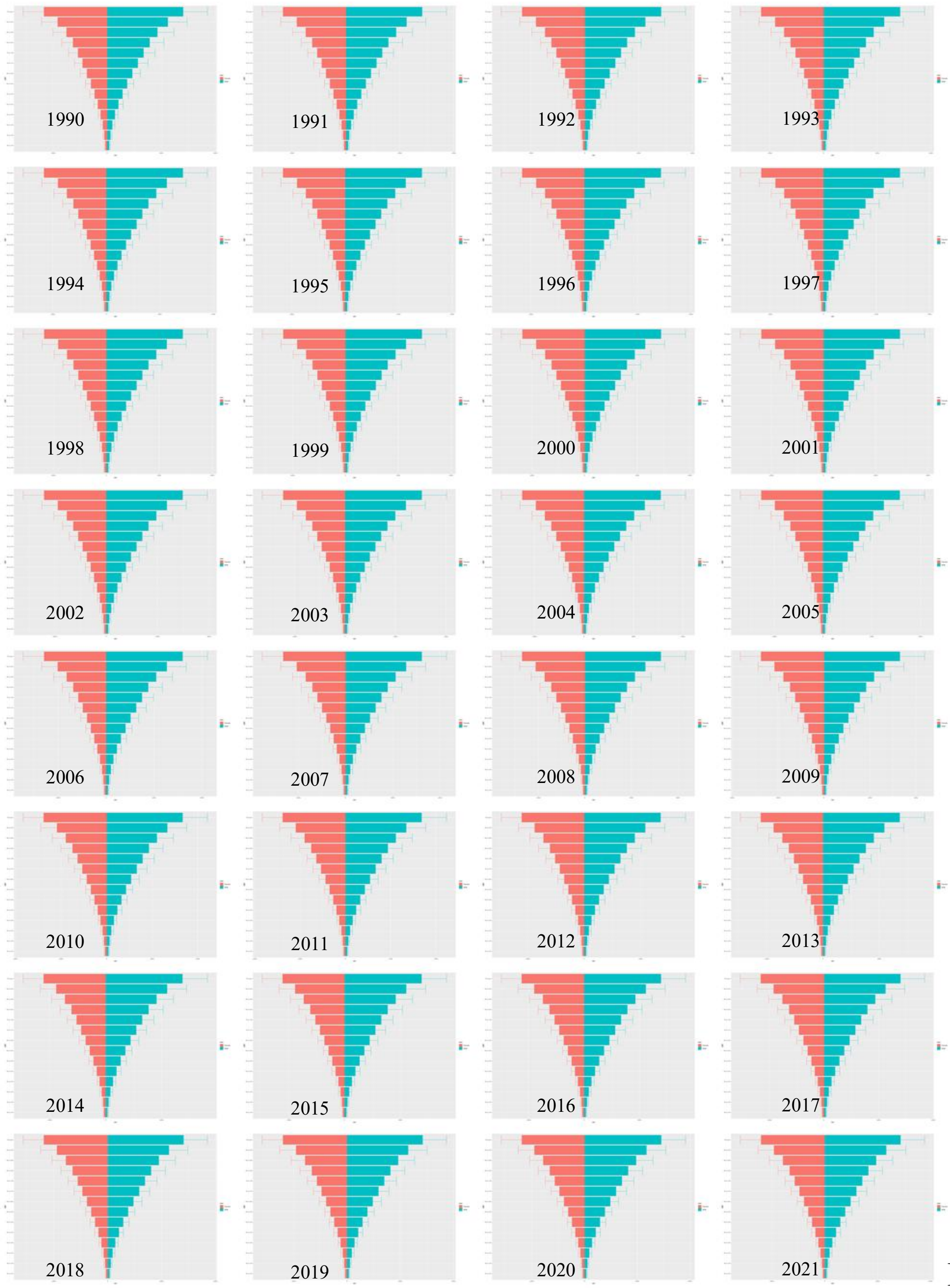
1990
1991
1992
1993
1994
1995
1996
1997
1998
1999
2000
2001
2002
2003
2004
2005
2006
2007
2008
2009
2010
2011
2012
2013
2014
2015
2016
2017
2018
2019
2020
2021

B

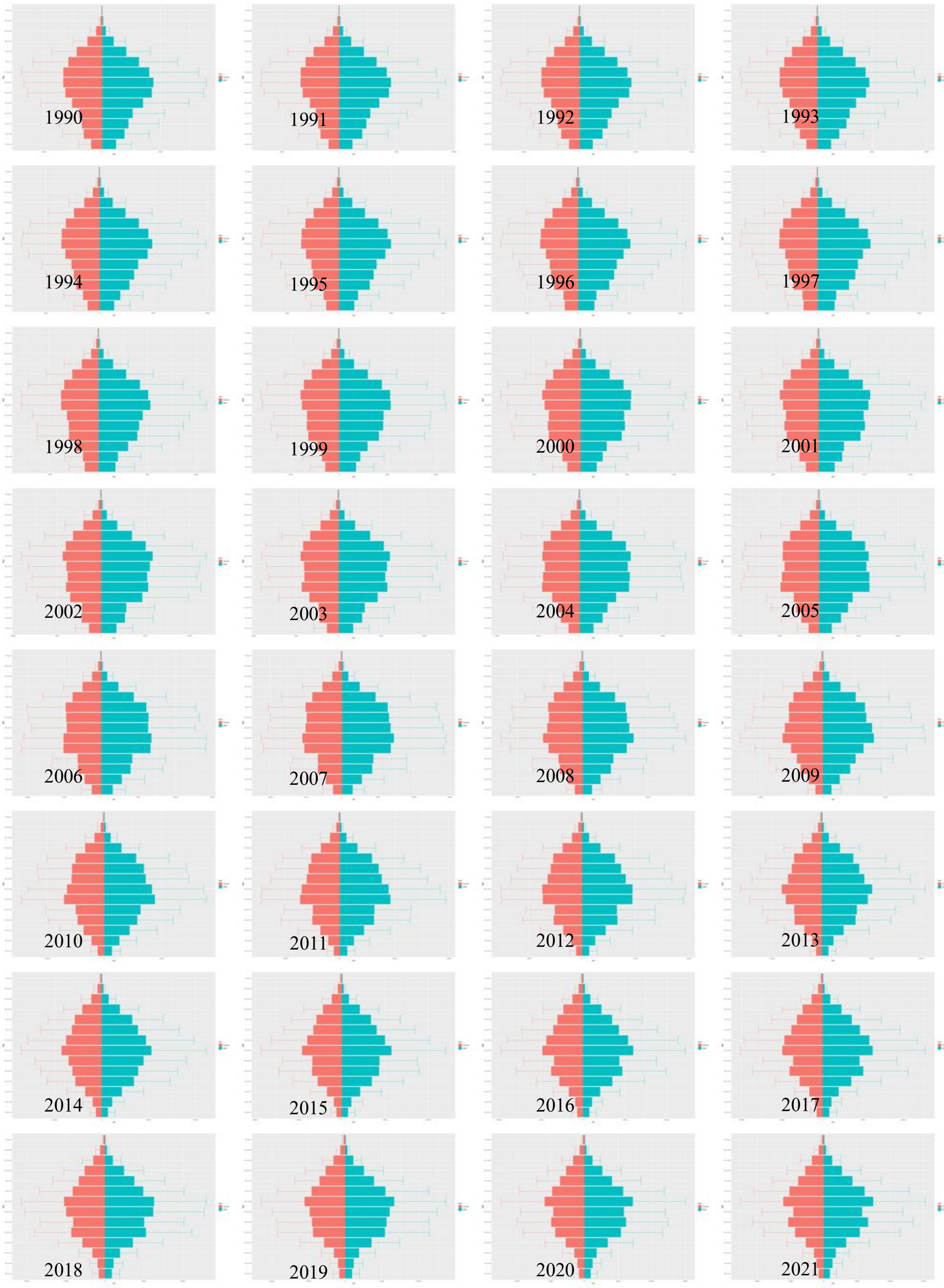
1990
1991
1992
1993
1994
1995
1996
1997
1998
1999
2000
2001
2002
2003
2004
2005
2006
2007
2008
2009
2010
2011
2012
2013
2014
2015
2016
2017
2018
2019
2020
2021

C

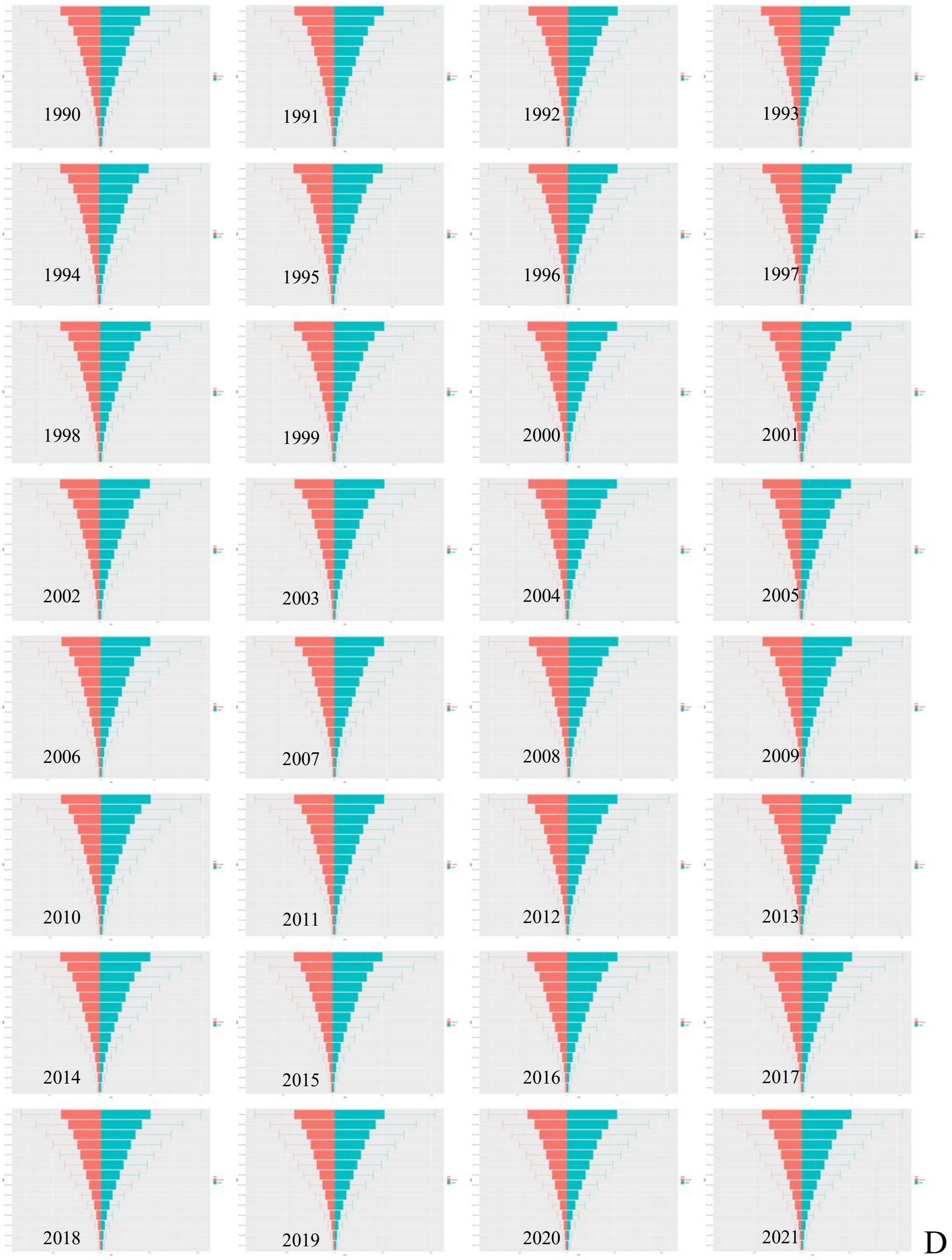

Fig. S67 (A) The prevalence cases of hip osteoarthritis in different ages from 1990 to 2021 in China; (B) The prevalence rates of hip osteoarthritis in different ages from 1990 to 2021 in China; (C) The years lived with disability of hip osteoarthritis in different ages from 1990 to 2021 in China; (D) The years lived with disability rates of hip osteoarthritis in different ages from 1990 to 2021 in China.

Notes: red for female, green for male; the ordinate from bottom to top is "30 to 34", "35 to 39", "40 to 44", "45 to 49", "50 to 54", "55 to 59", "60 to 64", "65 to 69", "70 to 74", "75 to 79", "80 to 84", "85 to 89", "90 to 94", "95 plus".

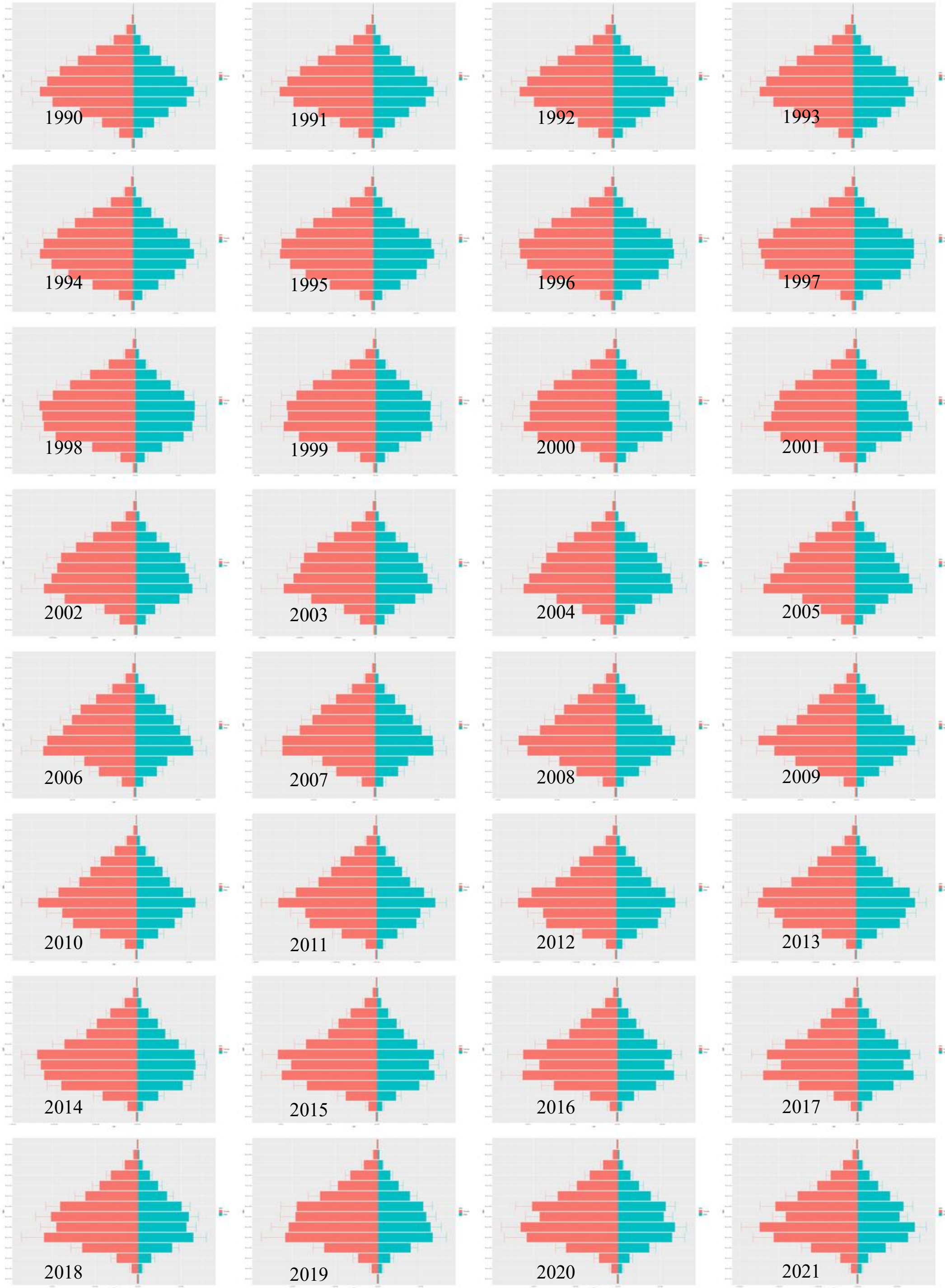
1990
1991
1992
1993
1994
1995
1996
1997
1998
1999
2000
2001
2002
2003
2004
2005
2006
2007
2008
2009
2010
2011
2012
2013
2014
2015
2016
2017
2018
2019
2020
2021

A

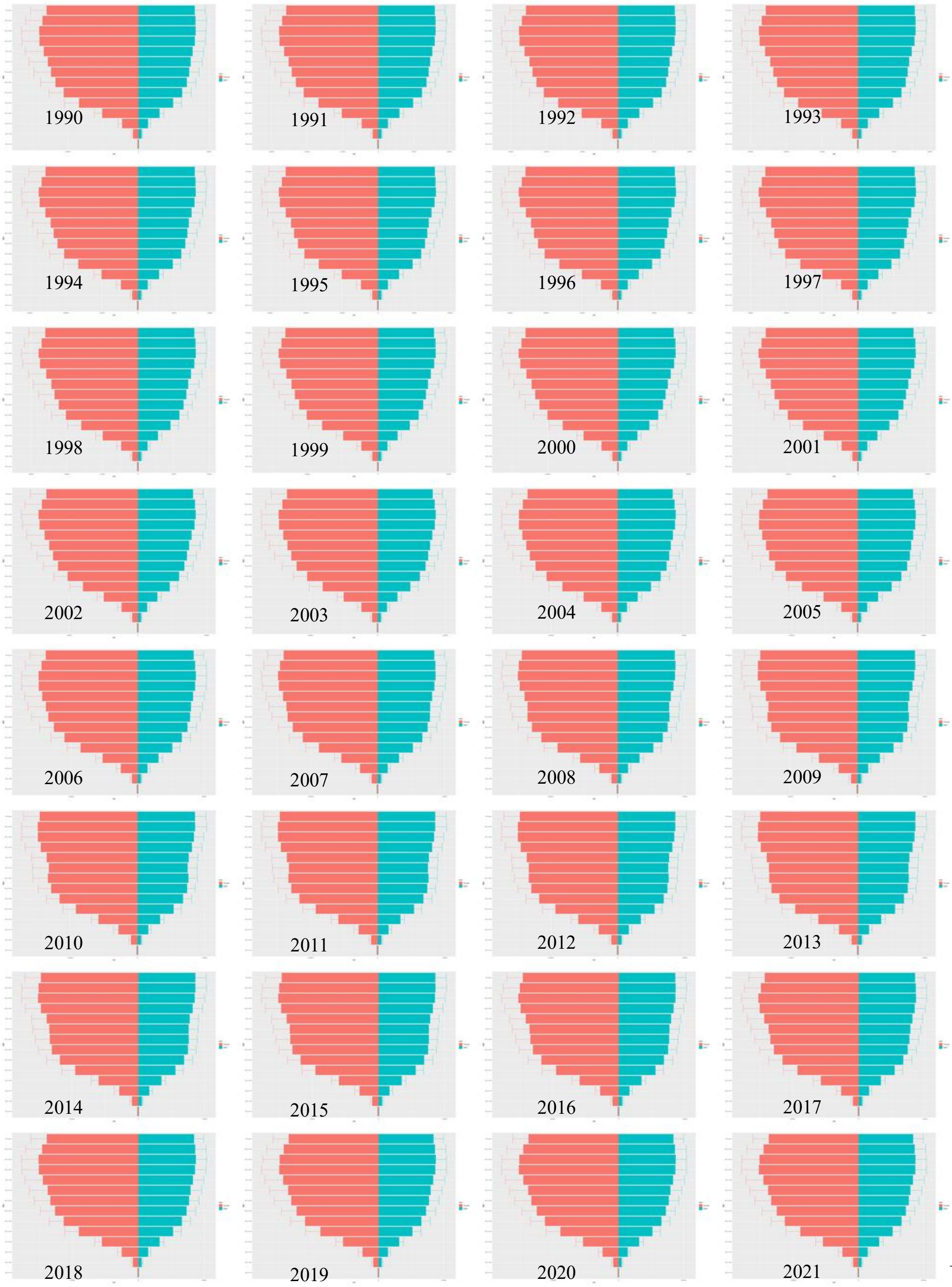
1990
1991
1992
1993
1994
1995
1996
1997
1998
1999
2000
2001
2002
2003
2004
2005
2006
2007
2008
2009
2010
2011
2012
2013
2014
2015
2016
2017
2018
2019
2020
2021

B

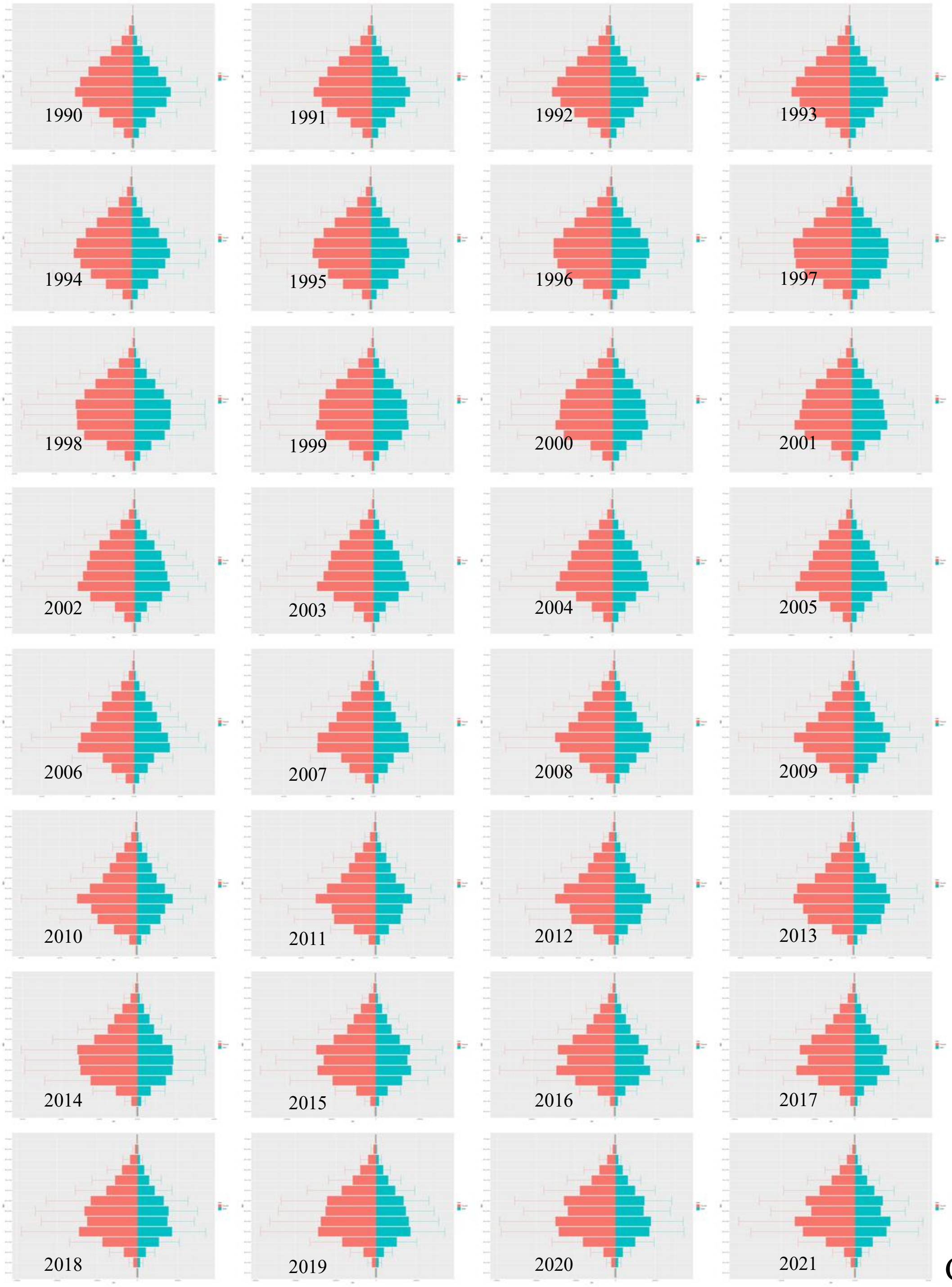
1990
1991
1992
1993
1994
1995
1996
1997
1998
1999
2000
2001
2002
2003
2004
2005
2006
2007
2008
2009
2010
2011
2012
2013
2014
2015
2016
2017
2018
2019
2020
2021
C

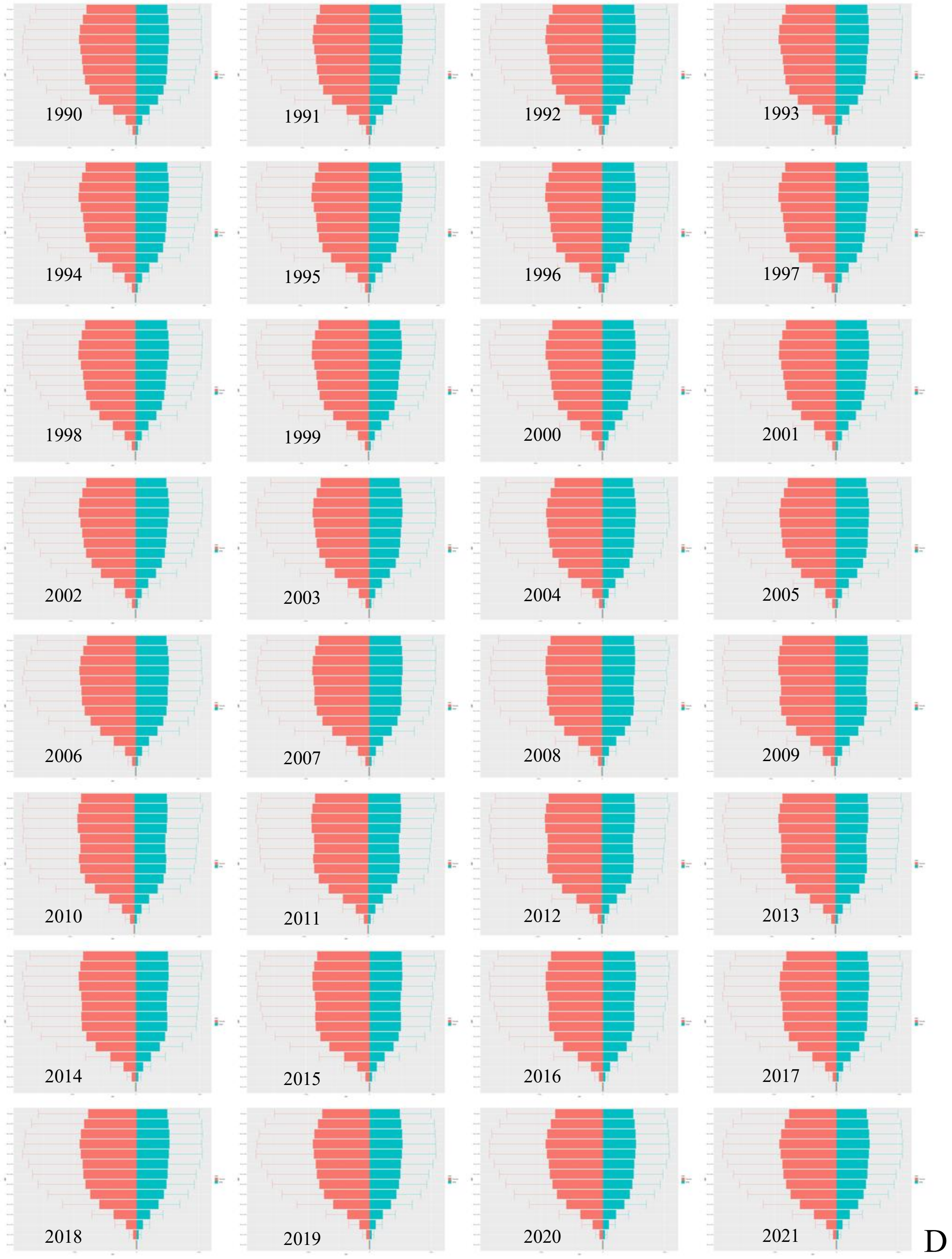

Fig. S68 (A) The prevalence cases of knee osteoarthritis in different ages from 1990 to 2021 in China; (B) The prevalence rates of knee osteoarthritis in different ages from 1990 to 2021 in China; (C) The years lived with disability of knee osteoarthritis in different ages from 1990 to 2021 in China; (D) The years lived with disability rates of knee osteoarthritis in different ages from 1990 to 2021 in China.

Notes: red for female, green for male; the ordinate from bottom to top is "35 to 39", "40 to 44", "45 to 49", "50 to 54", "55 to 59", "60 to 64", "65 to 69", "70 to 74", "75 to 79", "80 to 84", "85 to 89", "90 to 94", "95 plus".

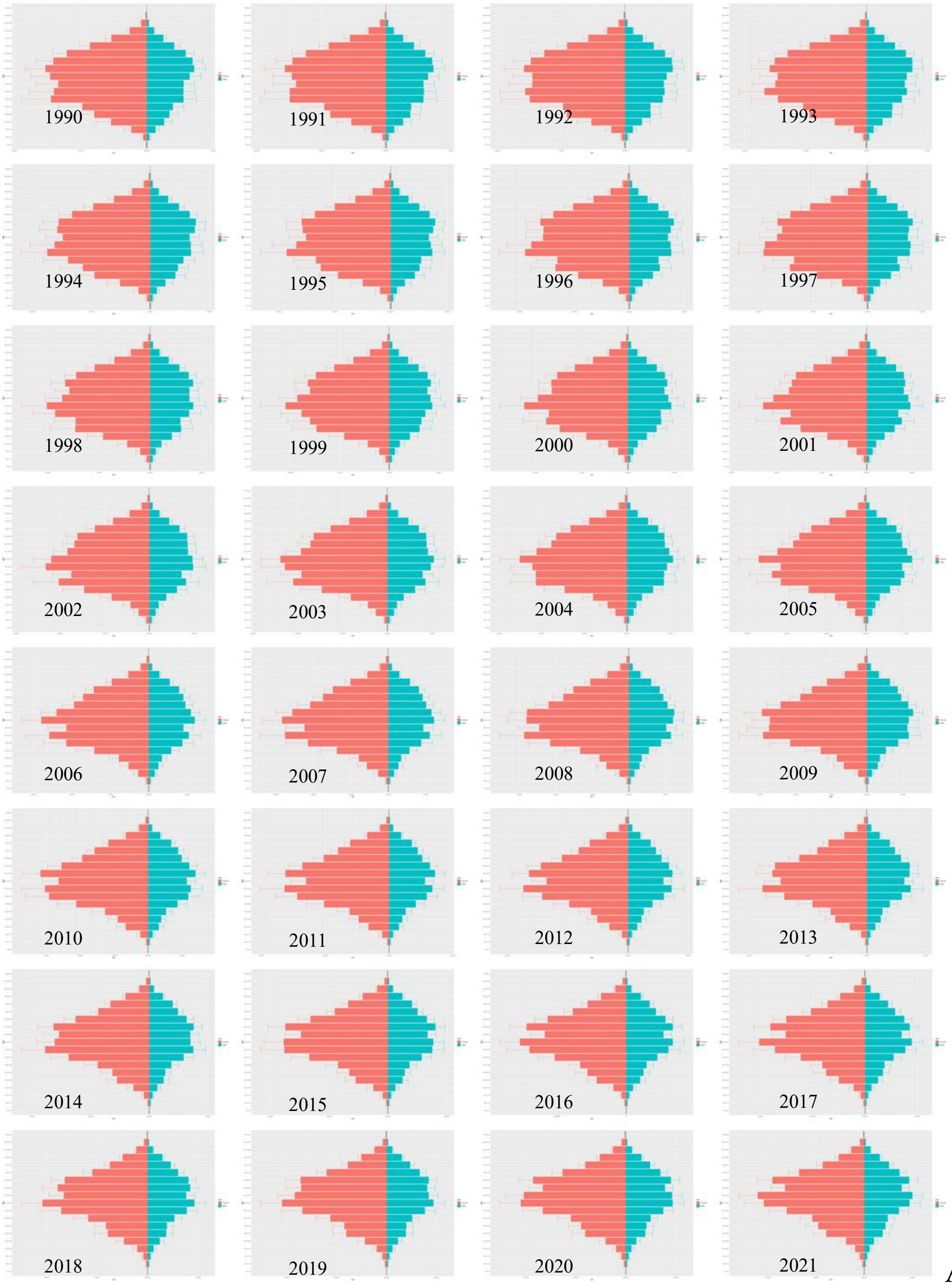
1990
1991
1992
1993
1994
1995
1996
1997
1998
1999
2000
2001
2002
2003
2004
2005
2006
2007
2008
2009
2010
2011
2012
2013
2014
2015
2016
2017
2018
2019
2020
2021
A

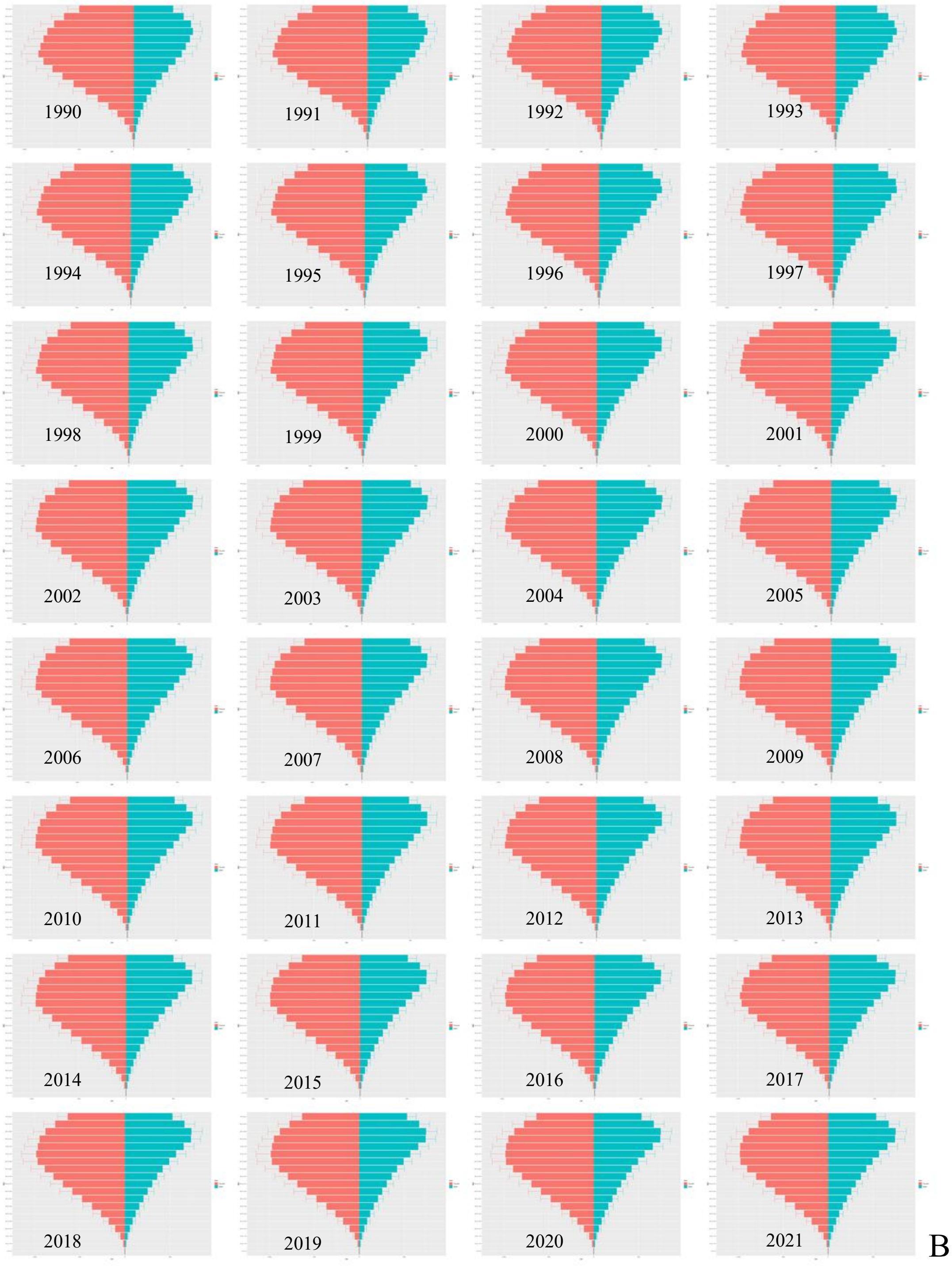
1990
1991
1992
1993
1994
1995
1996
1997
1998
1999
2000
2001
2002
2003
2004
2005
2006
2007
2008
2009
2010
2011
2012
2013
2014
2015
2016
2017
2018
2019
2020
2021
B

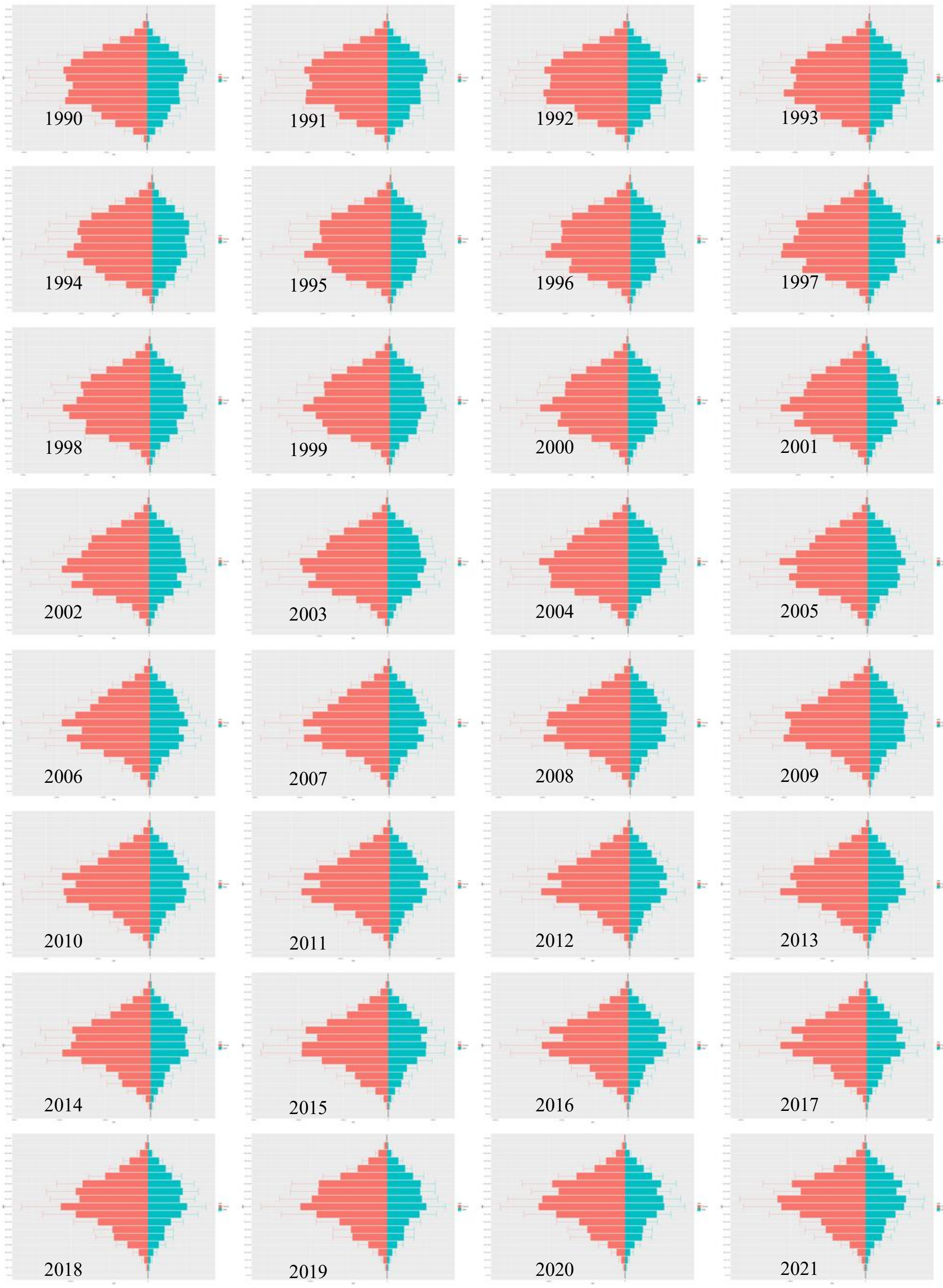
1990
1991
1992
1993
1994
1995
1996
1997
1998
1999
2000
2001
2002
2003
2004
2005
2006
2007
2008
2009
2010
2011
2012
2013
2014
2015
2016
2017
2018
2019
2020
2021
C

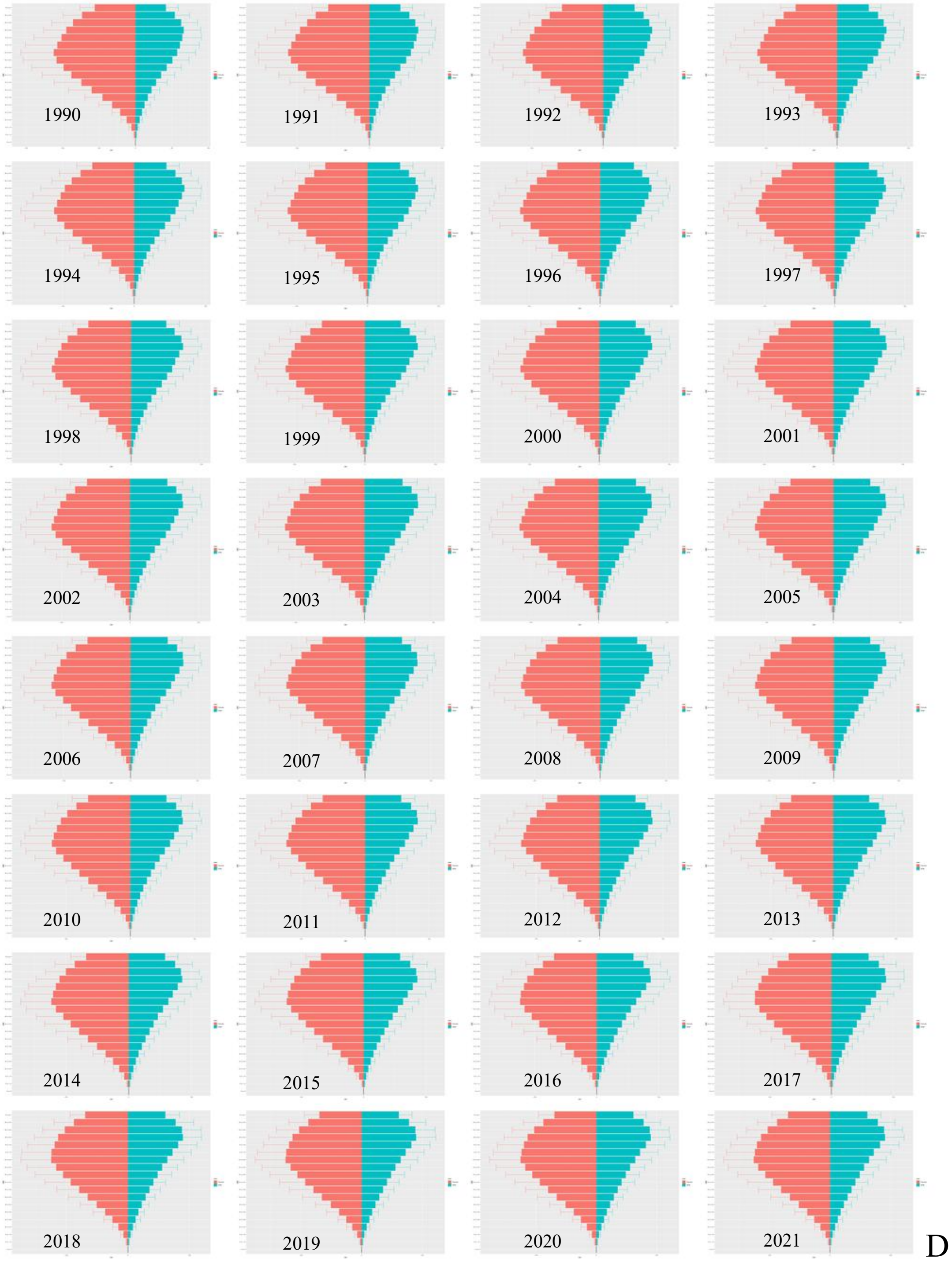

Fig. S69 (A) The prevalence cases of rheumatoid arthritis in different ages from 1990 to 2021 in China; (B) The prevalence rates of rheumatoid arthritis in different ages from 1990 to 2021 in China; (C) The years lived with disability of rheumatoid arthritis in different ages from 1990 to 2021 in China; (D) The years lived with disability rates of rheumatoid arthritis in different ages from 1990 to 2021 in China.
Notes: red for female, green for male; the ordinate from bottom to top is "5 to 9", "10 to 14", "15 to 19", "20 to 24", "25 to 29", "30 to 34", "35 to 39", "40 to 44", "45 to 49", "50 to 54", "55 to 59", "60 to 64", "65 to 69", "70 to 74", "75 to 79", "80 to 84", "85 to 89", "90 to 94", "95 plus".

1999 2000 2001

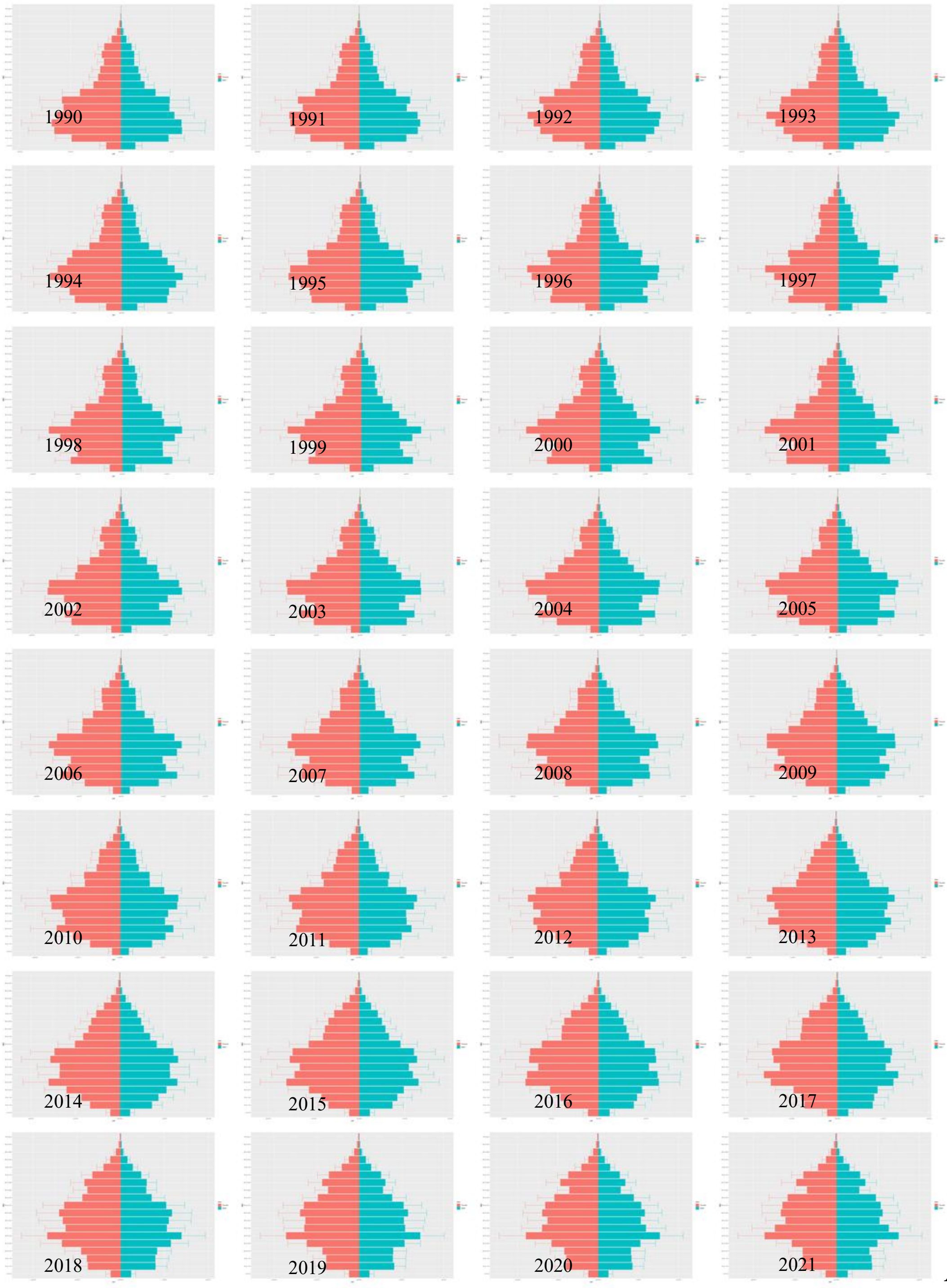
1990
1991
1992
1993
1994
1995
1996
1997
1998
1999
2000
2001
2002
2003
2004
2005
2006
2007
2008
2009
2010
2011
2012
2013
2014
2015
2016
2017
2018
2019
2020
2021
A

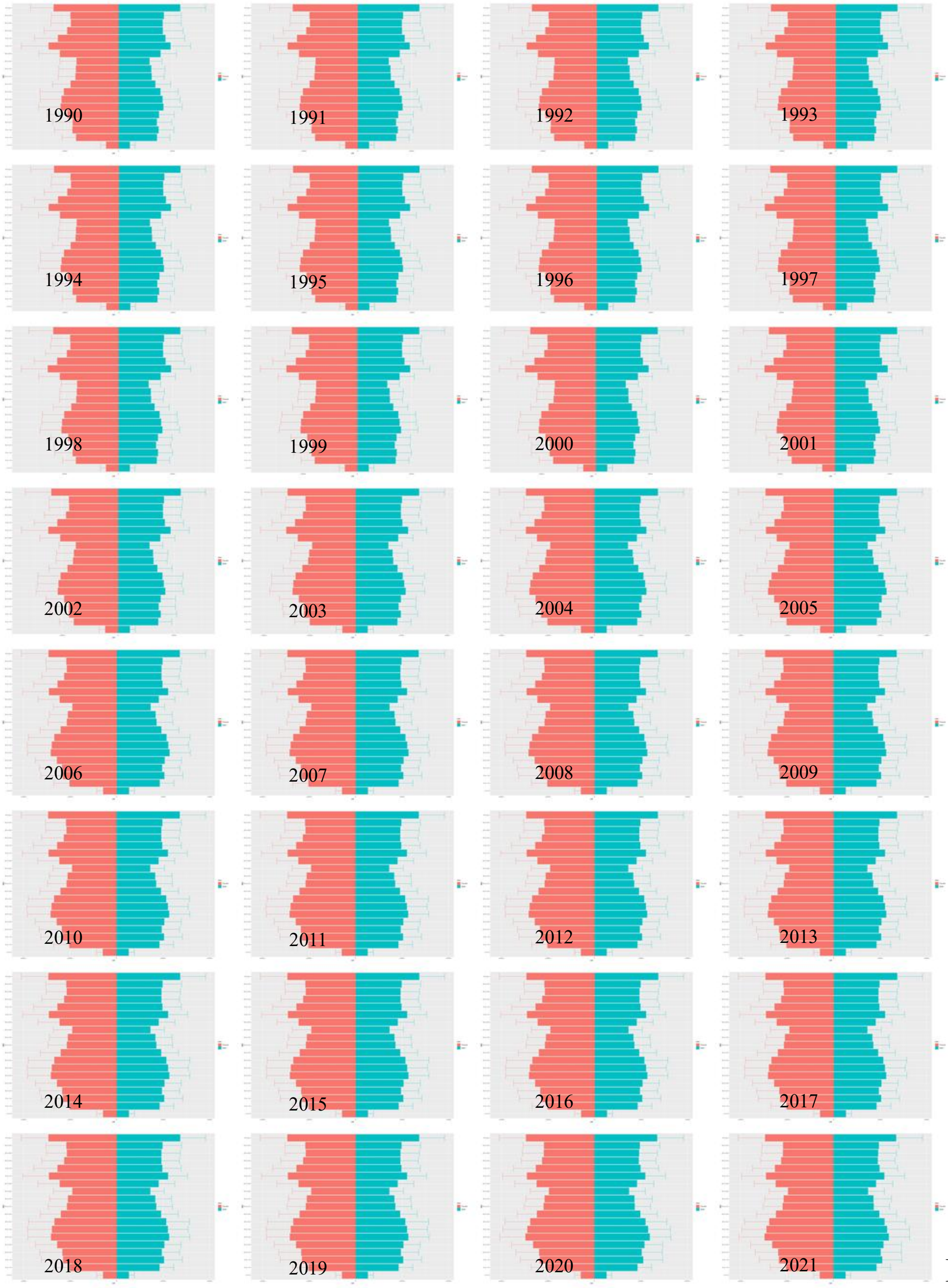
1990
1991
1992
1993
1994
1995
1996
1997
1998
1999
2000
2001
2002
2003
2004
2005
2006
2007
2008
2009
2010
2011
2012
2013
2014
2015
2016
2017
2018
2019
2020
2021

B

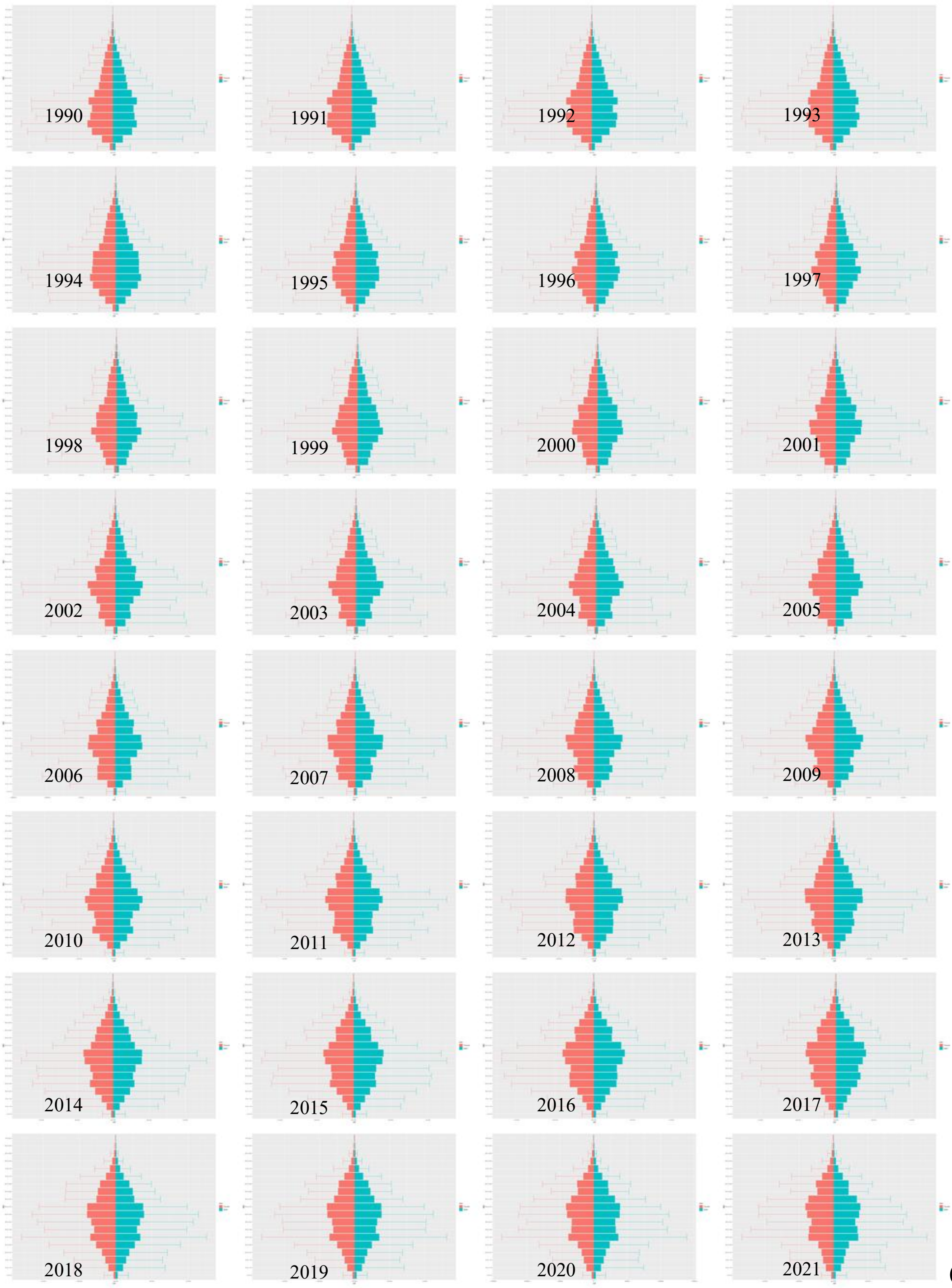
1990
1991
1992
1993
1994
1995
1996
1997
1998
1999
2000
2001
2002
2003
2004
2005
2006
2007
2008
2009
2010
2011
2012
2013
2014
2015
2016
2017
2018
2019
2020
2021
C

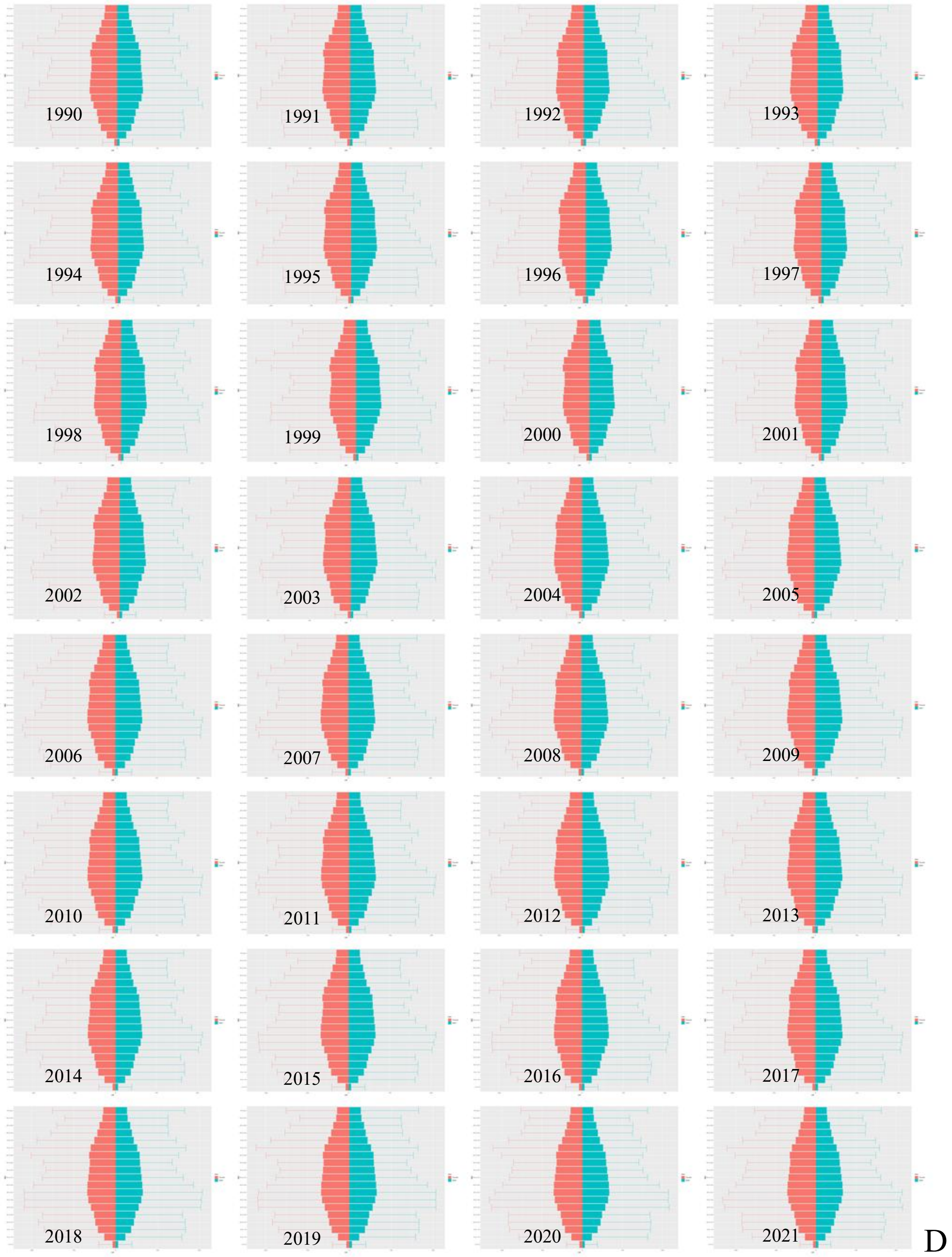

Fig. S70 (A) The prevalence cases of tension-type headache in different ages from 1990 to 2021 in China; (B) The prevalence rates of tension-type headache in different ages from 1990 to 2021 in China; (C) The years lived with disability of tension-type headache in different ages from 1990 to 2021 in China; (D) The years lived with disability rates of tension-type headache in different ages from 1990 to 2021 in China.

Notes: red for female, green for male; the ordinate from bottom to top is "5 to 9", "10 to 14", "15 to 19", "20 to 24", "25 to 29", "30 to 34", "35 to 39", "40 to 44", "45 to 49", "50 to 54", "55 to 59", "60 to 64", "65 to 69", "70 to 74", "75 to 79", "80 to 84", "85 to 89", "90 to 94", "95 plus".

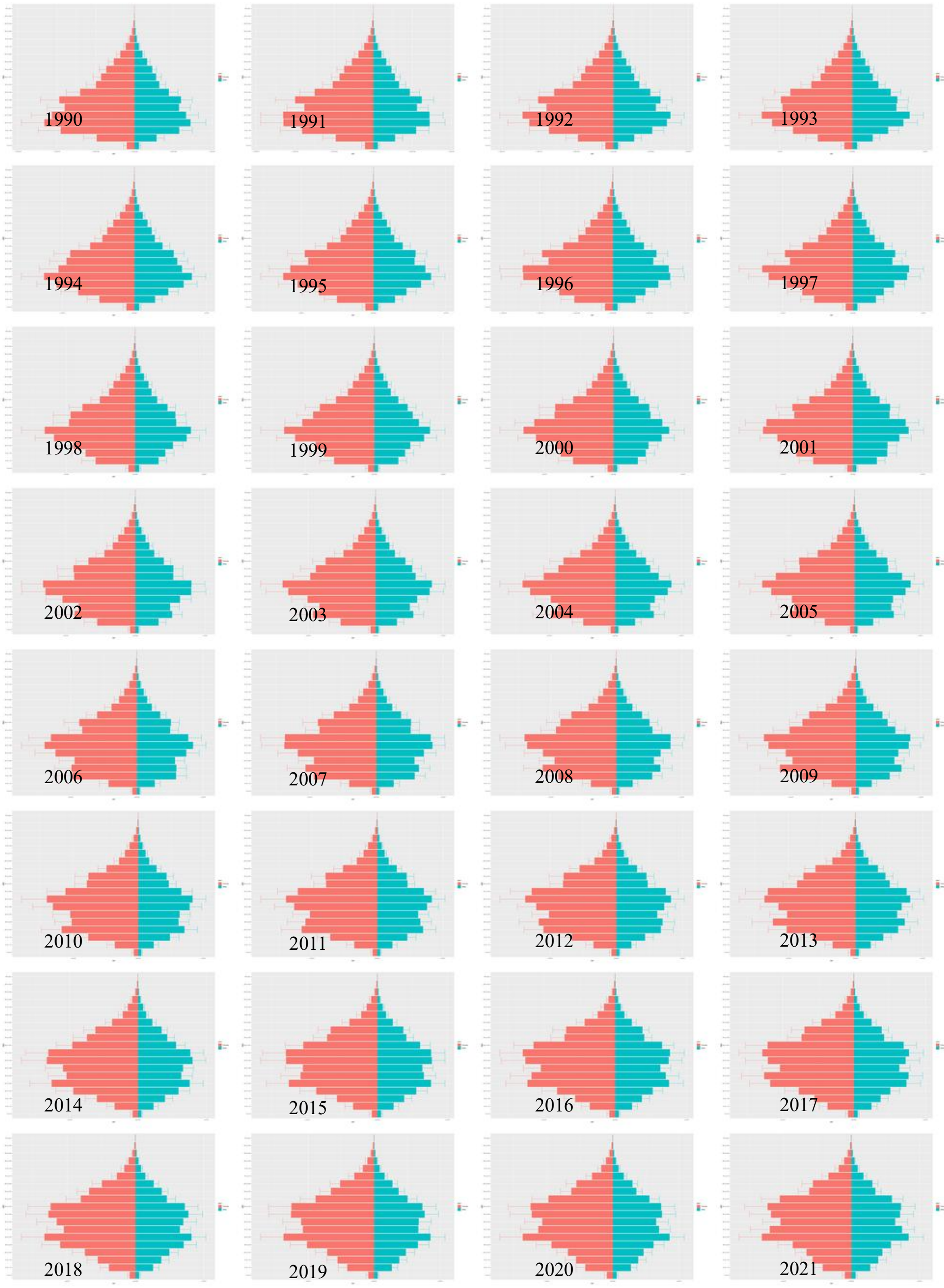
1990
1991
1992
1993
1994
1995
1996
1997
1998
1999
2000
2001
2002
2003
2004
2005
2006
2007
2008
2009
2010
2011
2012
2013
2014
2015
2016
2017
2018
2019
2020
2021

A

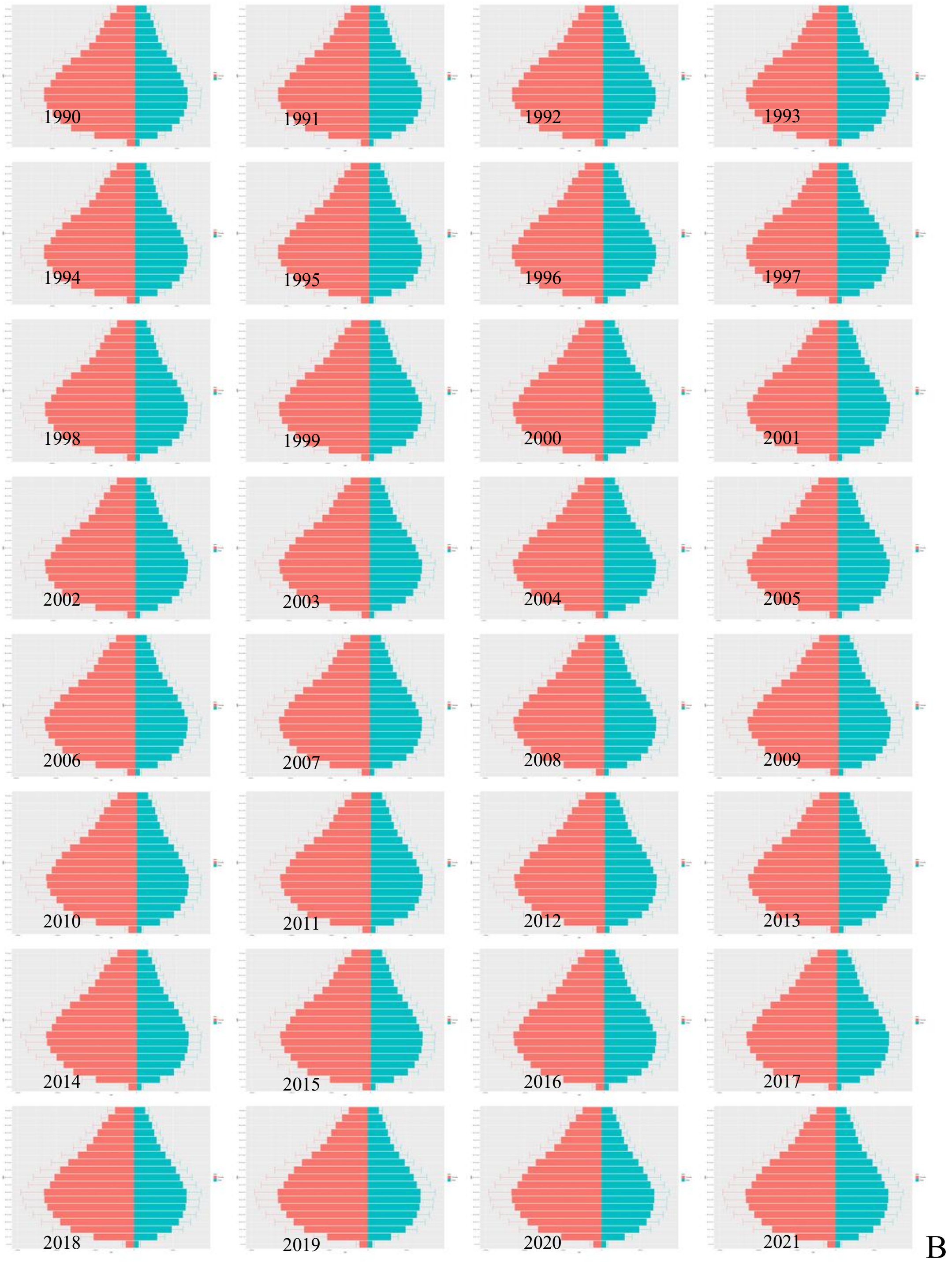
1990
1991
1992
1993
1994
1995
1996
1997
1998
1999
2000
2001
2002
2003
2004
2005
2006
2007
2008
2009
2010
2011
2012
2013
2014
2015
2016
2017
2018
2019
2020
2021
B

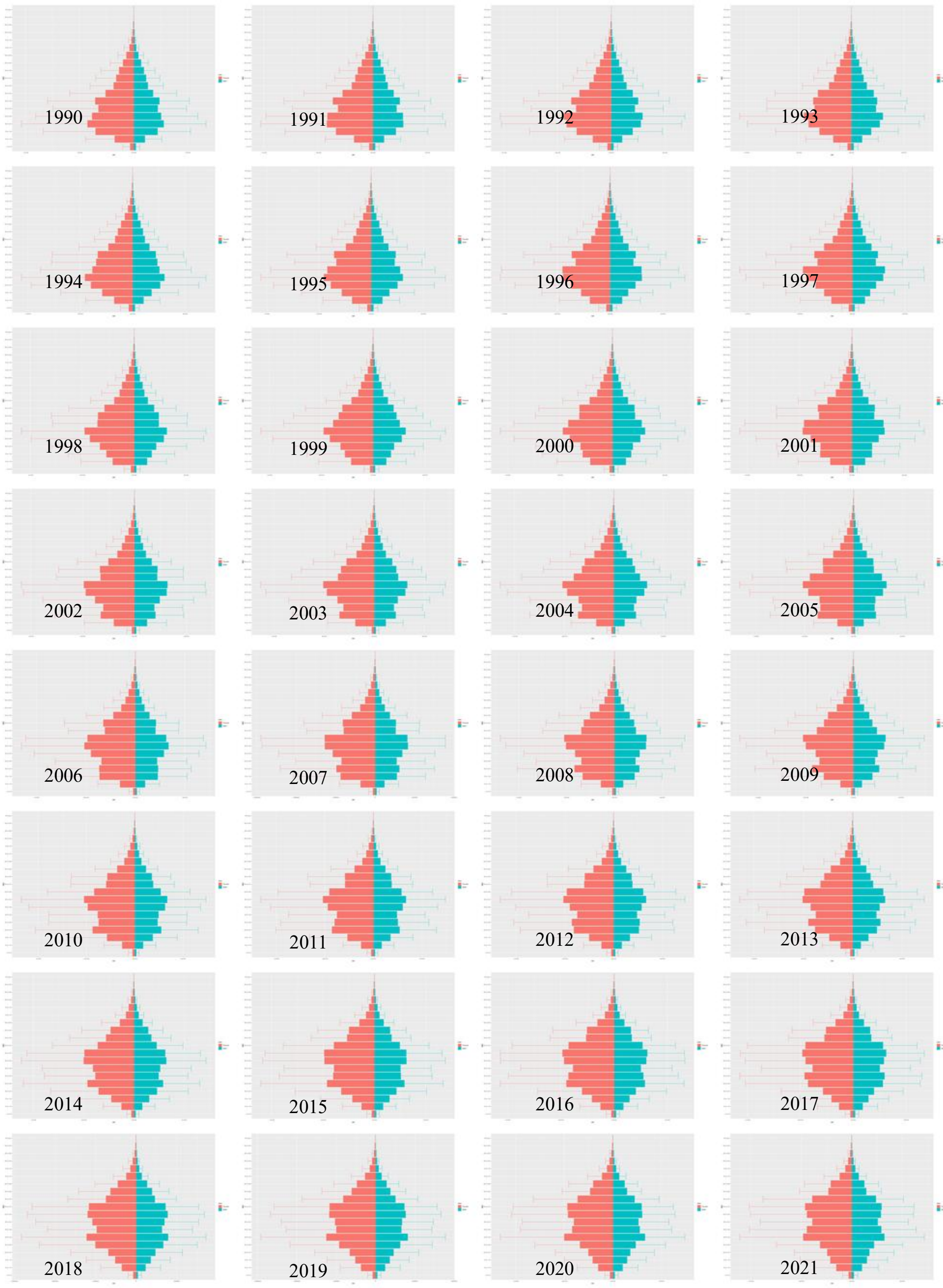
1990
1991
1992
1993
1994
1995
1996
1997
1998
1999
2000
2001
2002
2003
2004
2005
2006
2007
2008
2009
2010
2011
2012
2013
2014
2015
2016
2017
2018
2019
2020
2021

C

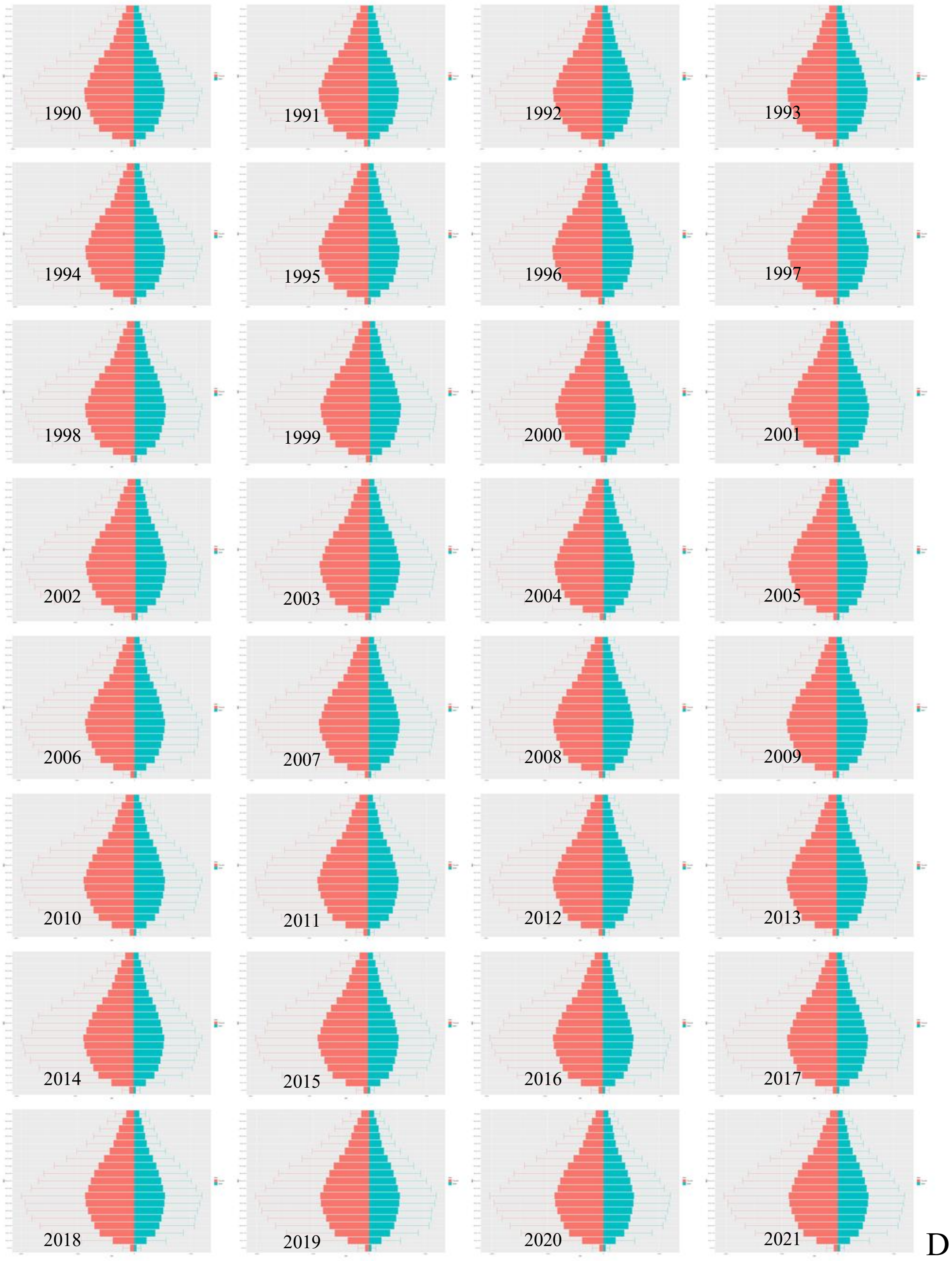

D

Fig. S71 (A) The prevalence cases of migraine in different ages from 1990 to 2021 in China; (B) The prevalence rates of migraine in different ages from 1990 to 2021 in China; (C) The years lived with disability of migraine in different ages from 1990 to 2021 in China; (D) The years lived with disability rates of migraine in different ages from 1990 to 2021 in China.

Notes: red for female, green for male; the ordinate from bottom to top is "5 to 9", "10 to 14", "15 to 19", "20 to 24", "25 to 29", "30 to 34", "35 to 39", "40 to 44", "45 to 49", "50 to 54", "55 to 59", "60 to 64", "65 to 69", "70 to 74", "75 to 79", "80 to 84", "85 to 89", "90 to 94", "95 plus".

1999 2000 2001

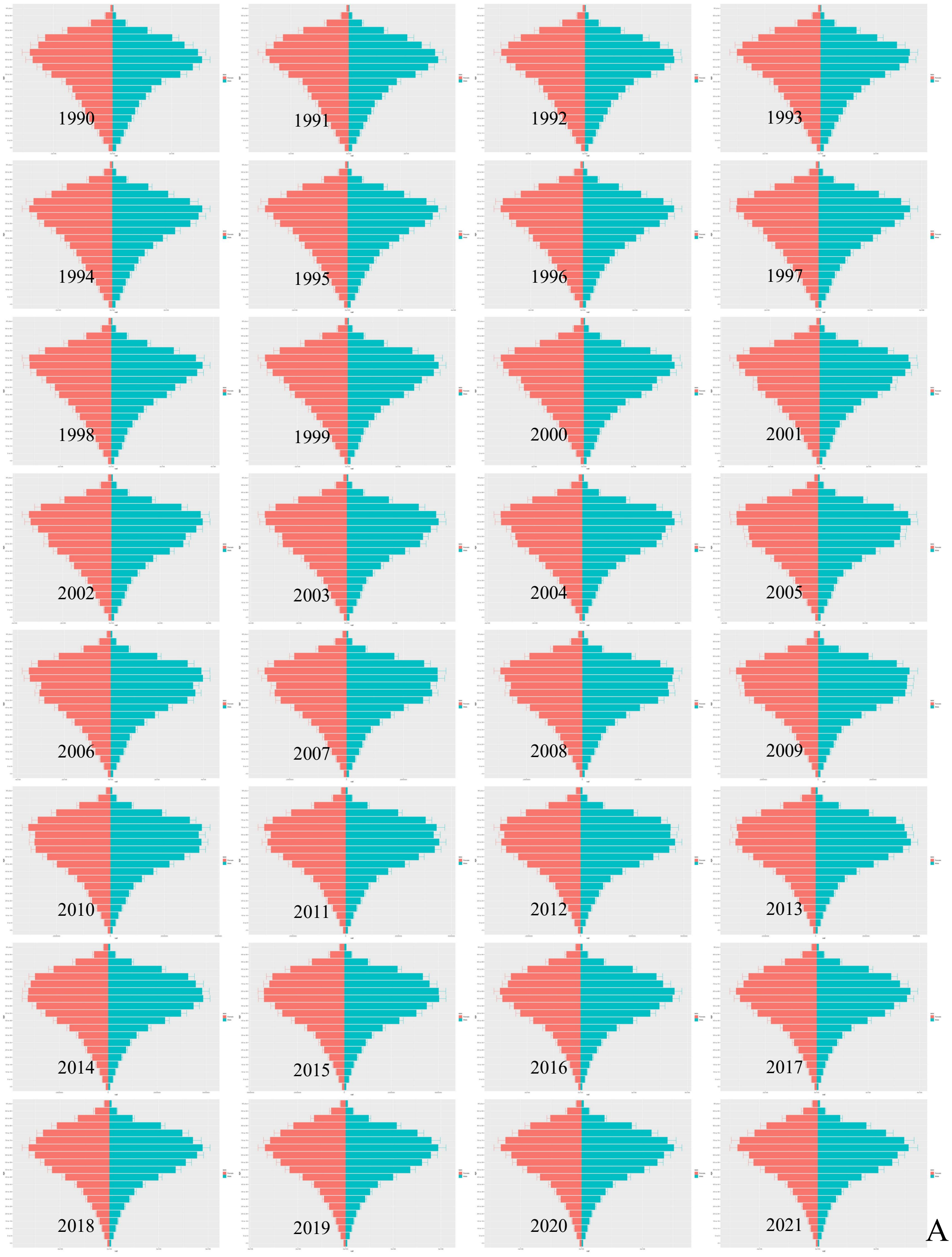
1990
1991
1992
1993
1994
1995
1996
1997
1998
1999
2000
2001
2002
2003
2004
2005
2006
2007
2008
2009
2010
2011
2012
2013
2014
2015
2016
2017
2018
2019
2020
2021

A

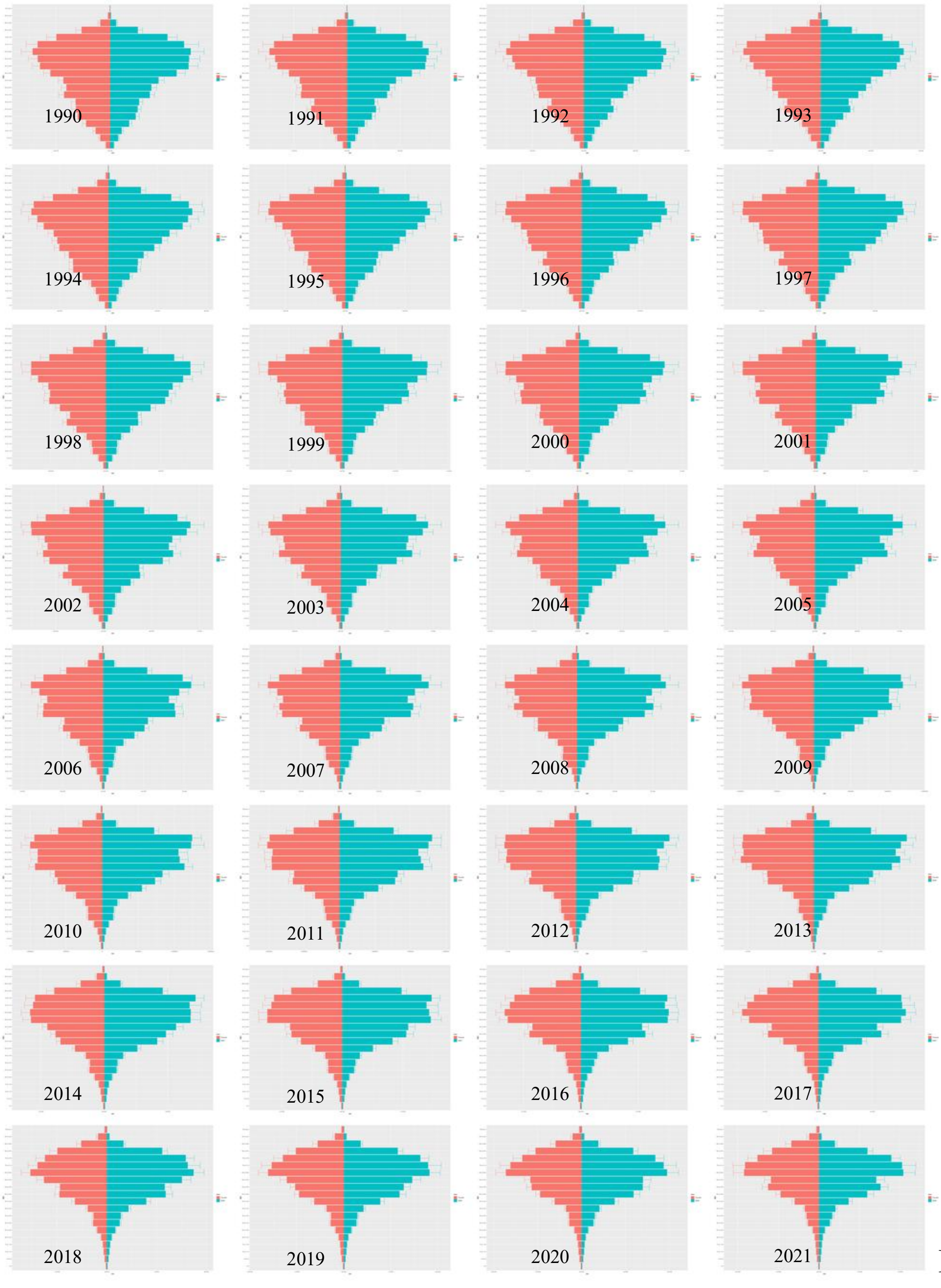
1990
1991
1992
1993
1994
1995
1996
1997
1998
1999
2000
2001
2002
2003
2004
2005
2006
2007
2008
2009
2010
2011
2012
2013
2014
2015
2016
2017
2018
2019
2020
2021

B

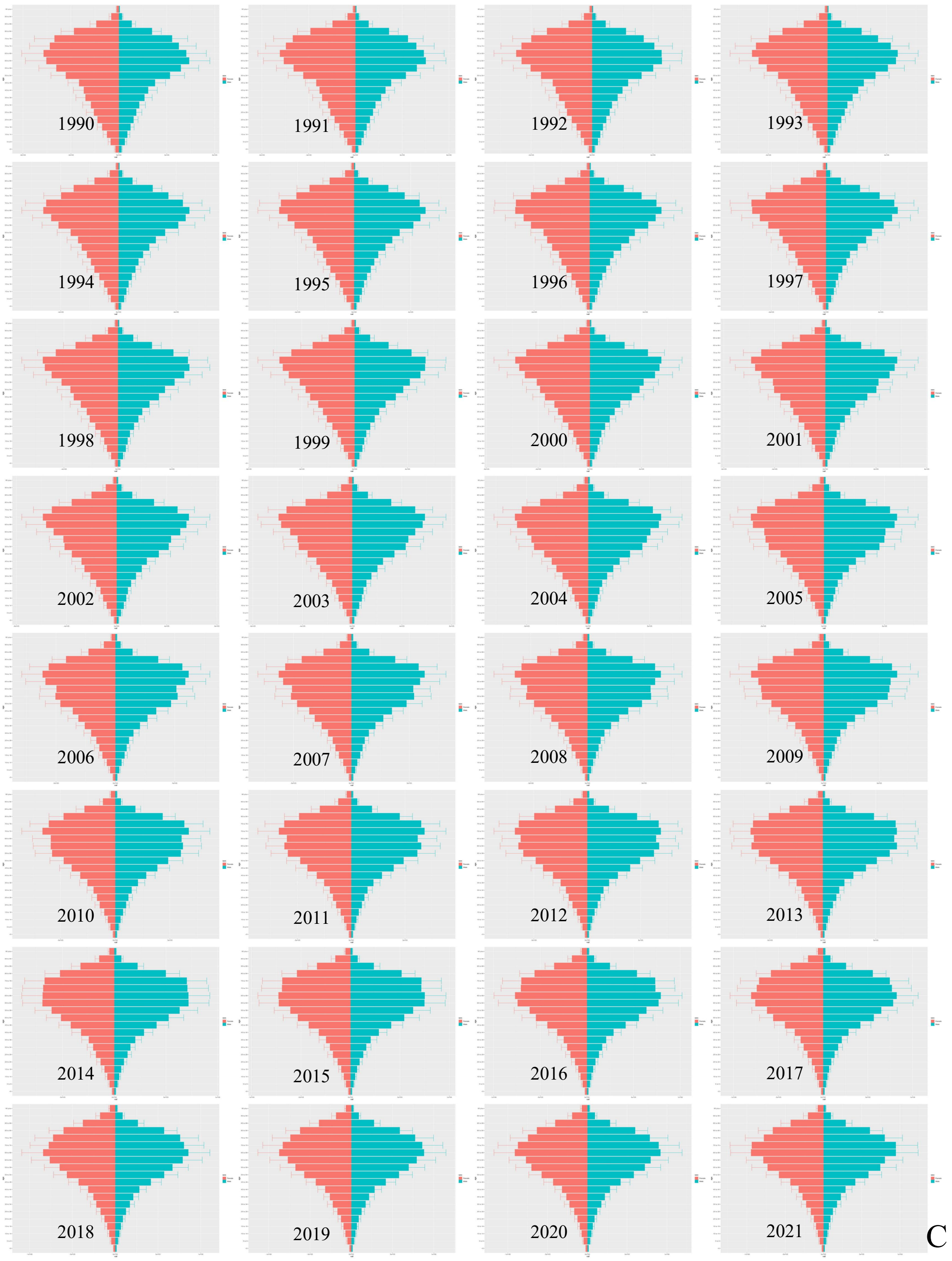
1990
1991
1992
1993
1994
1995
1996
1997
1998
1999
2000
2001
2002
2003
2004
2005
2006
2007
2008
2009
2010
2011
2012
2013
2014
2015
2016
2017
2018
2019
2020
2021
C

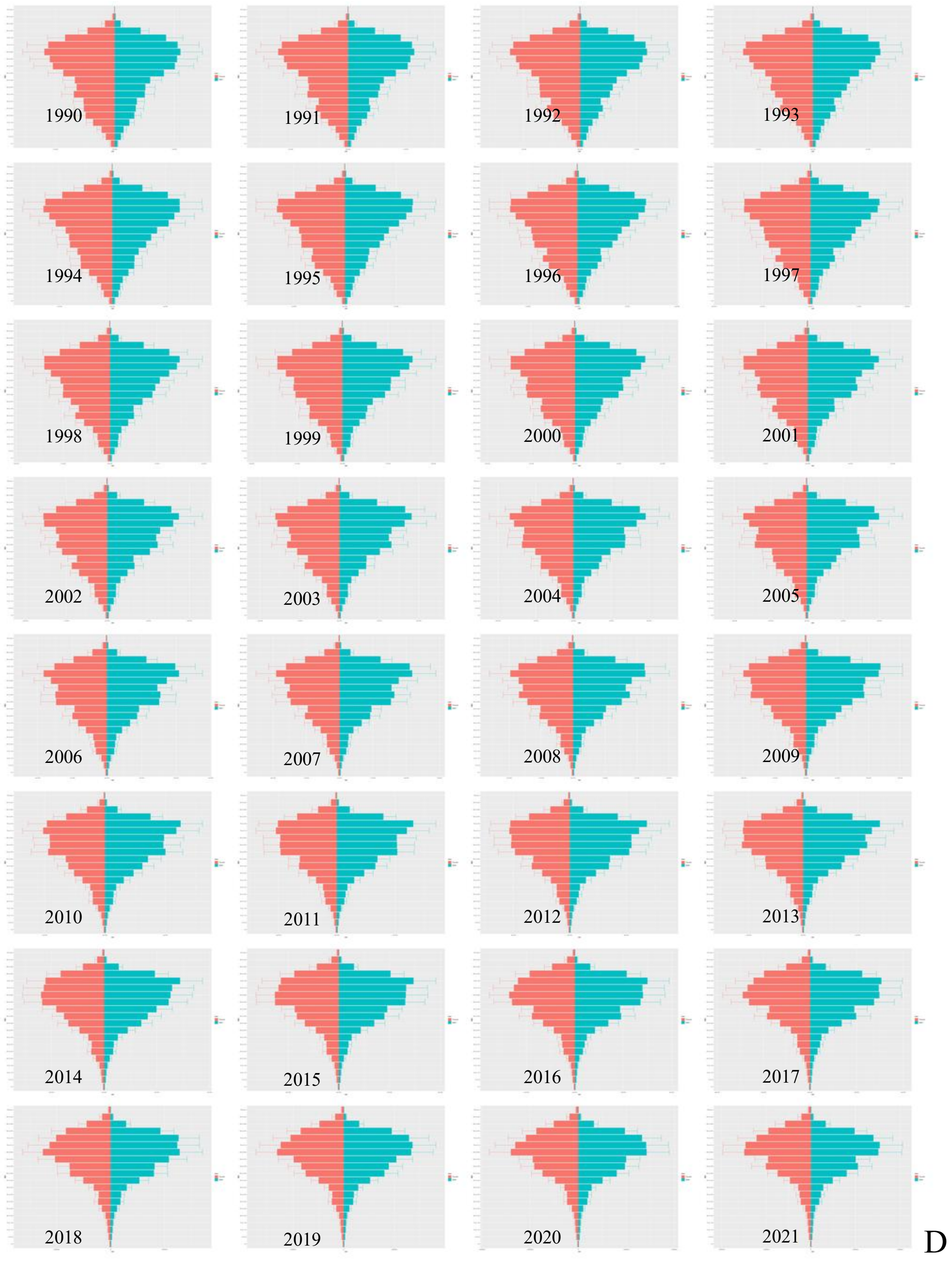

Fig. S72 (A) The prevalence cases of stroke in different ages from 1990 to 2021 in China; (B) The prevalence rates of stroke in different ages from 1990 to 2021 in China; (C) The years lived with disability of stroke in different ages from 1990 to 2021 in China; (D) The years lived with disability rates of stroke in different ages from 1990 to 2021 in China.

Notes: red for female, green for male; the ordinate from bottom to top is "<5", "5 to 9", "10 to 14", "15 to 19", "20 to 24", "25 to 29", "30 to 34", "35 to 39", "40 to 44", "45 to 49", "50 to 54", "55 to 59", "60 to 64", "65 to 69", "70 to 74", "75 to 79", "80 to 84", "85 to 89", "90 to 94", "95 plus".

1999 2000 2001

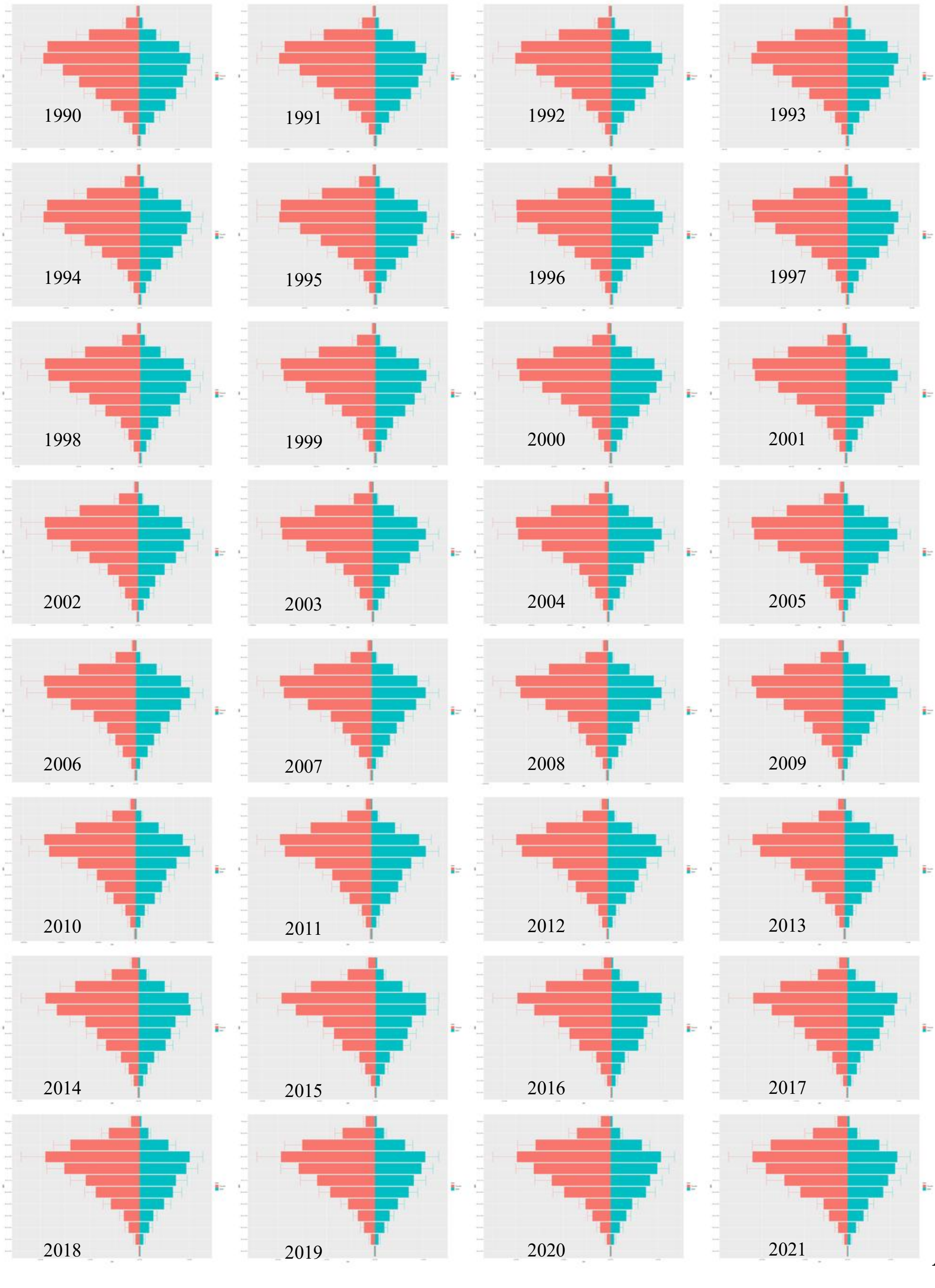
1990
1991
1992
1993
1994
1995
1996
1997
1998
1999
2000
2001
2002
2003
2004
2005
2006
2007
2008
2009
2010
2011
2012
2013
2014
2015
2016
2017
2018
2019
2020
2021

A

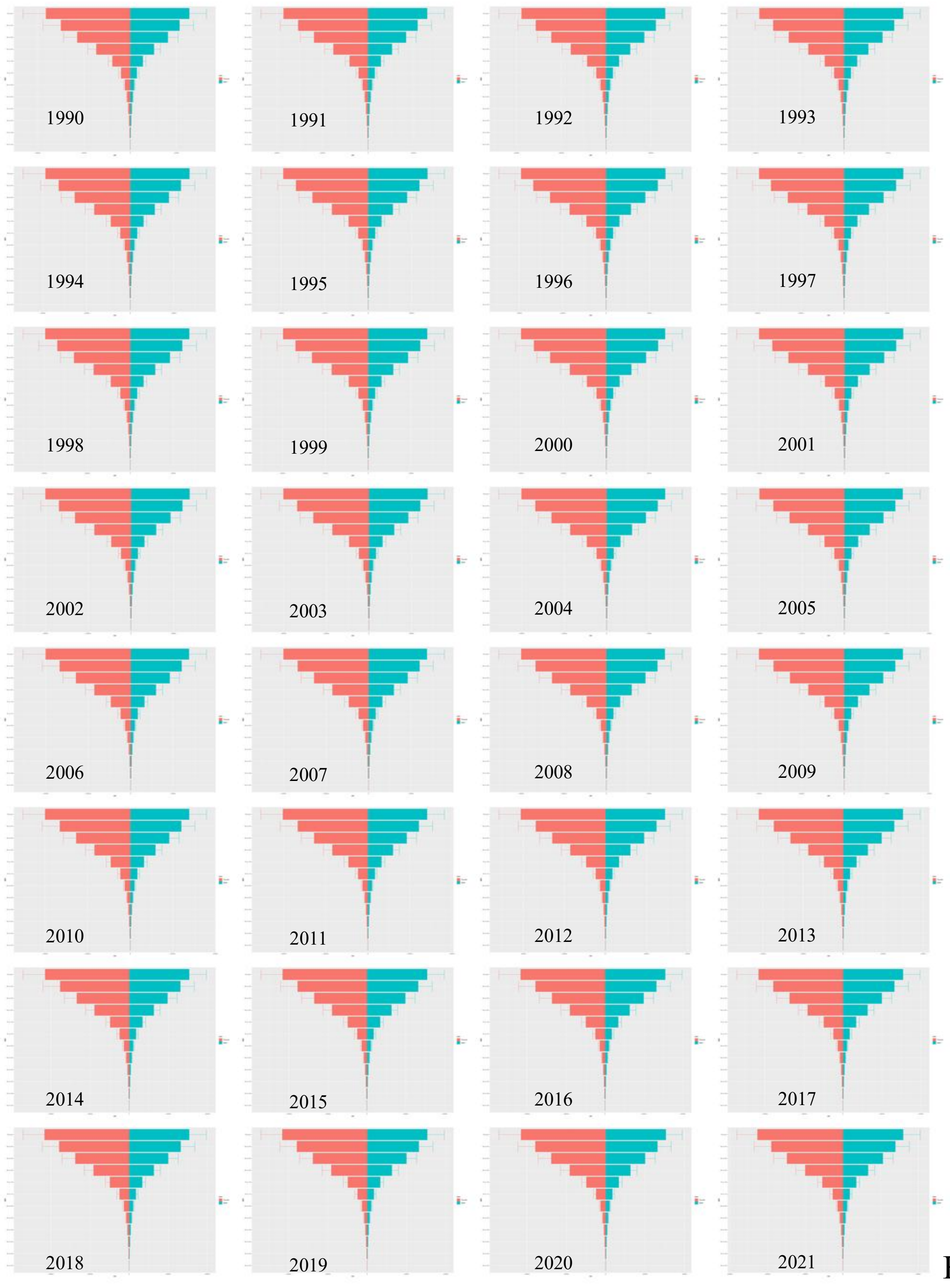
1990
1991
1992
1993
1994
1995
1996
1997
1998
1999
2000
2001
2002
2003
2004
2005
2006
2007
2008
2009
2010
2011
2012
2013
2014
2015
2016
2017
2018
2019
2020
2021

B

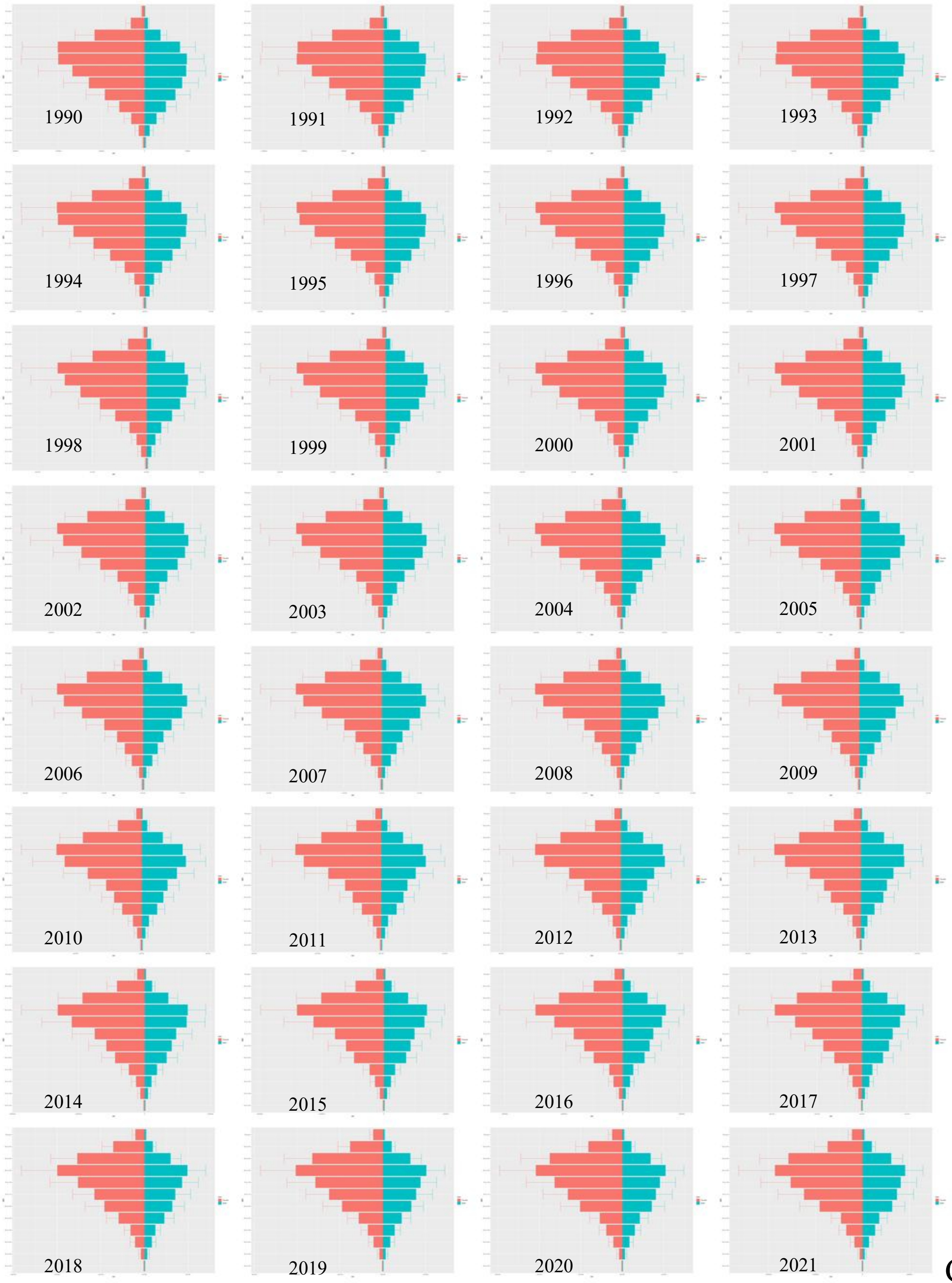
1990
1991
1992
1993
1994
1995
1996
1997
1998
1999
2000
2001
2002
2003
2004
2005
2006
2007
2008
2009
2010
2011
2012
2013
2014
2015
2016
2017
2018
2019
2020
2021
C

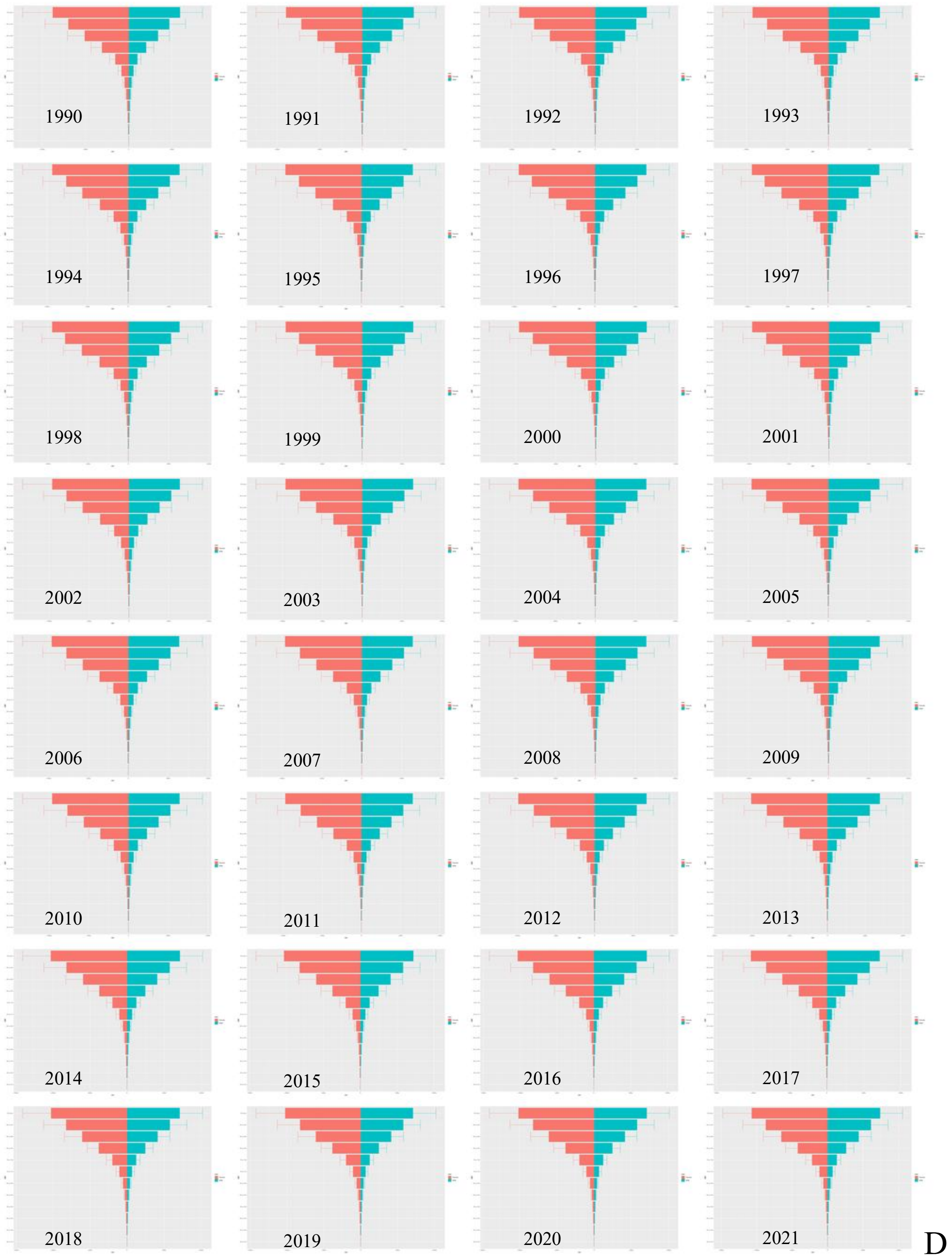

Fig. S73 (A) The prevalence cases of Alzheimer's disease and other dementias in different ages from 1990 to 2021 in China; (B) The prevalence rates of Alzheimer's disease and other dementias in different ages from 1990 to 2021 in China; (C) The years lived with disability of Alzheimer's disease and other dementias in different ages from 1990 to 2021 in China; (D) The years lived with disability rates of Alzheimer's disease and other dementias in different ages from 1990 to 2021 in China.

Notes: red for female, green for male; the ordinate from bottom to top is "40 to 44", "45 to 49", "50 to 54", "55 to 59", "60 to 64", "65 to 69", "70 to 74", "75 to 79", "80 to 84", "85 to 89", "90 to 94", "95 plus".

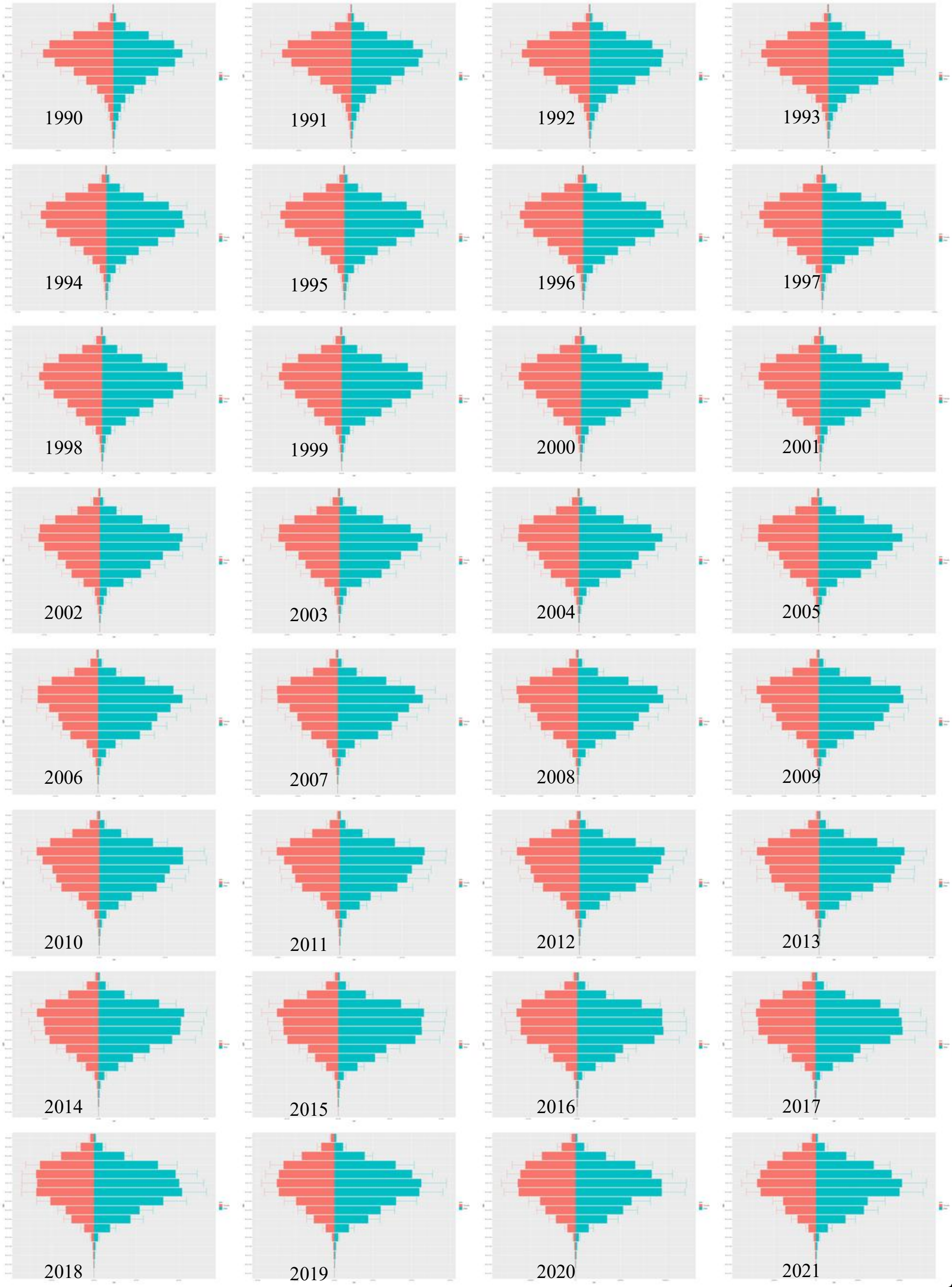
1990
1991
1992
1993
1994
1995
1996
1997
1998
1999
2000
2001
2002
2003
2004
2005
2006
2007
2008
2009
2010
2011
2012
2013
2014
2015
2016
2017
2018
2019
2020
2021

A

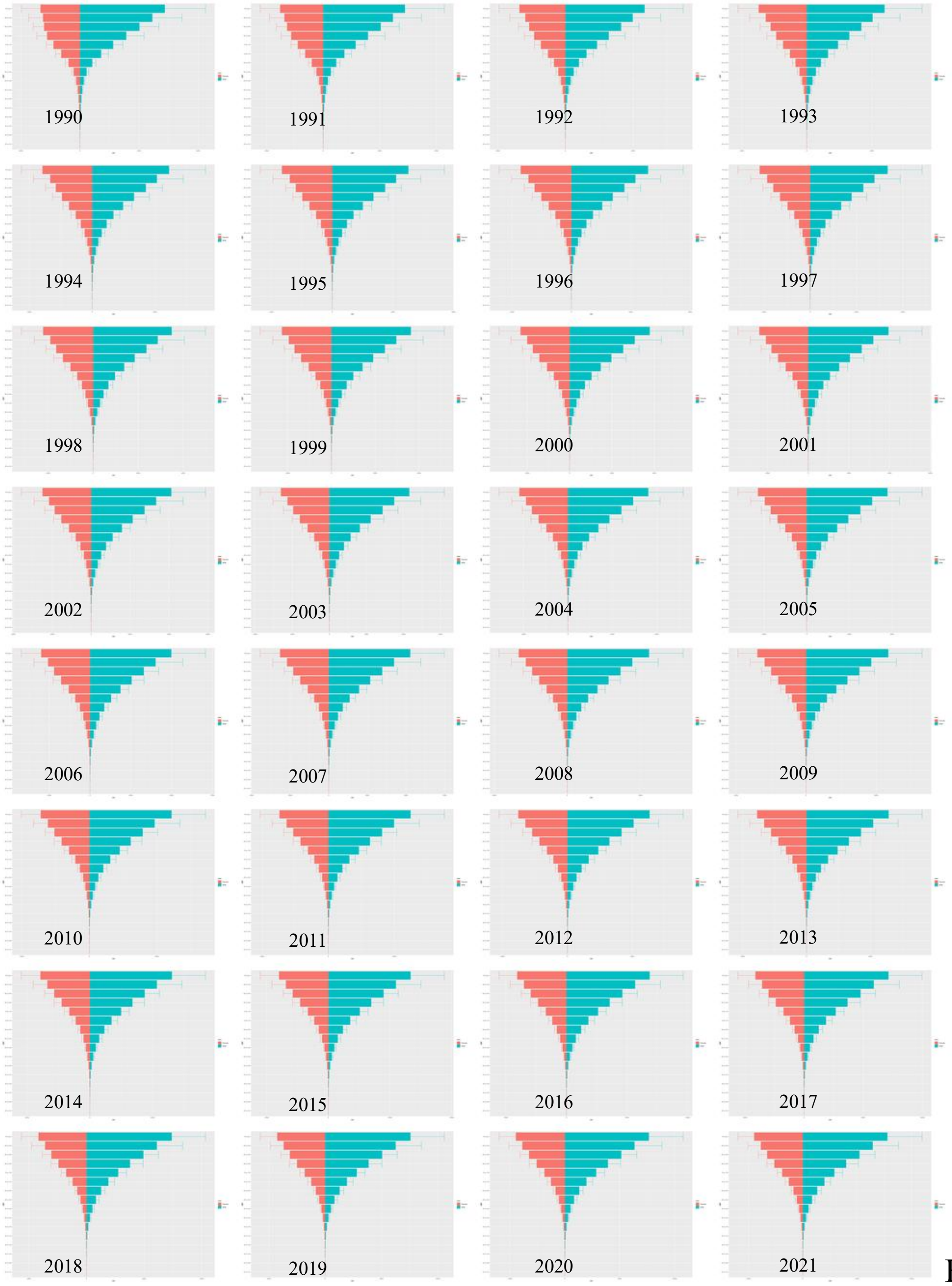
1990
1991
1992
1993
1994
1995
1996
1997
1998
1999
2000
2001
2002
2003
2004
2005
2006
2007
2008
2009
2010
2011
2012
2013
2014
2015
2016
2017
2018
2019
2020
2021

B

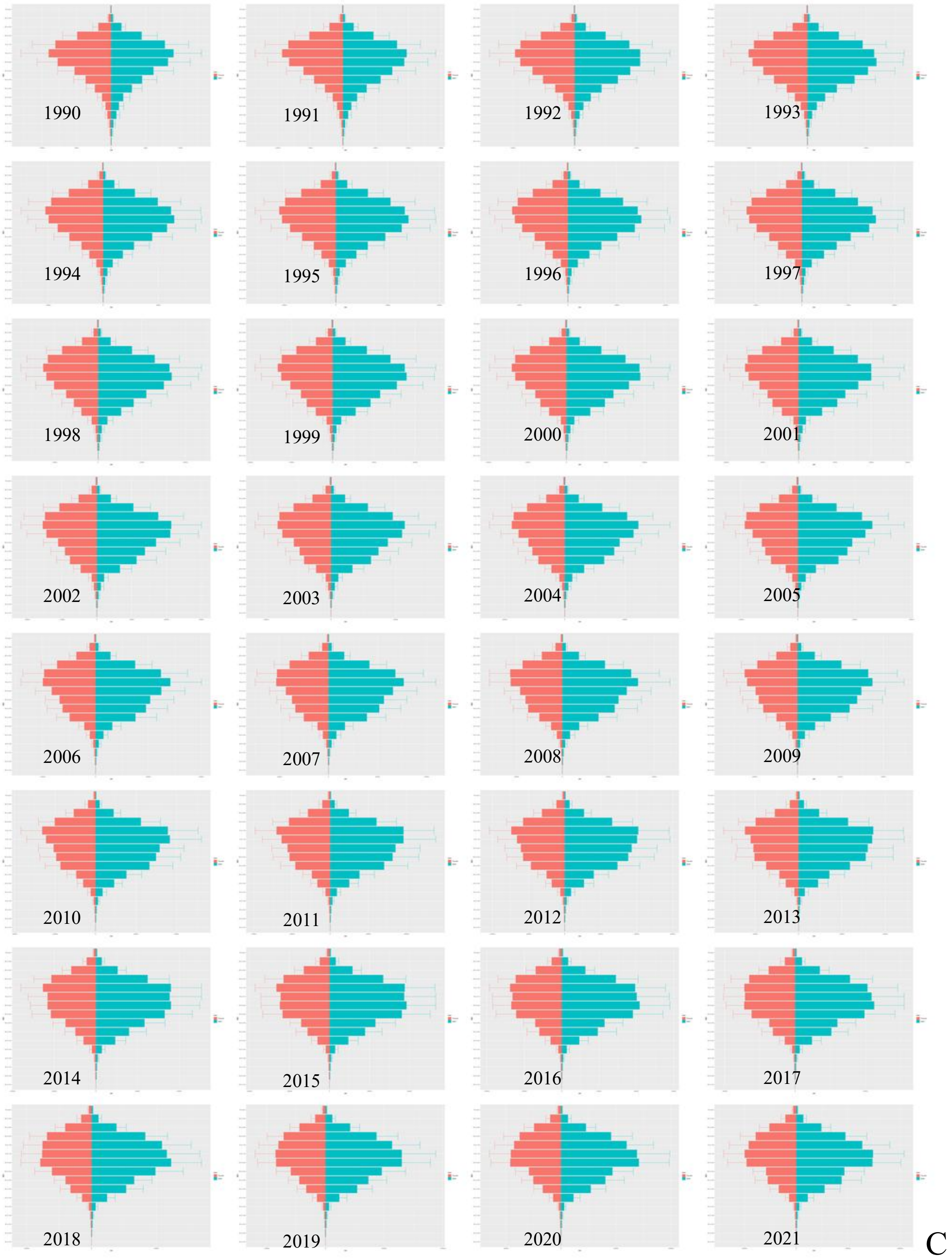
1990
1991
1992
1993
1994
1995
1996
1997
1998
1999
2000
2001
2002
2003
2004
2005
2006
2007
2008
2009
2010
2011
2012
2013
2014
2015
2016
2017
2018
2019
2020
2021
C

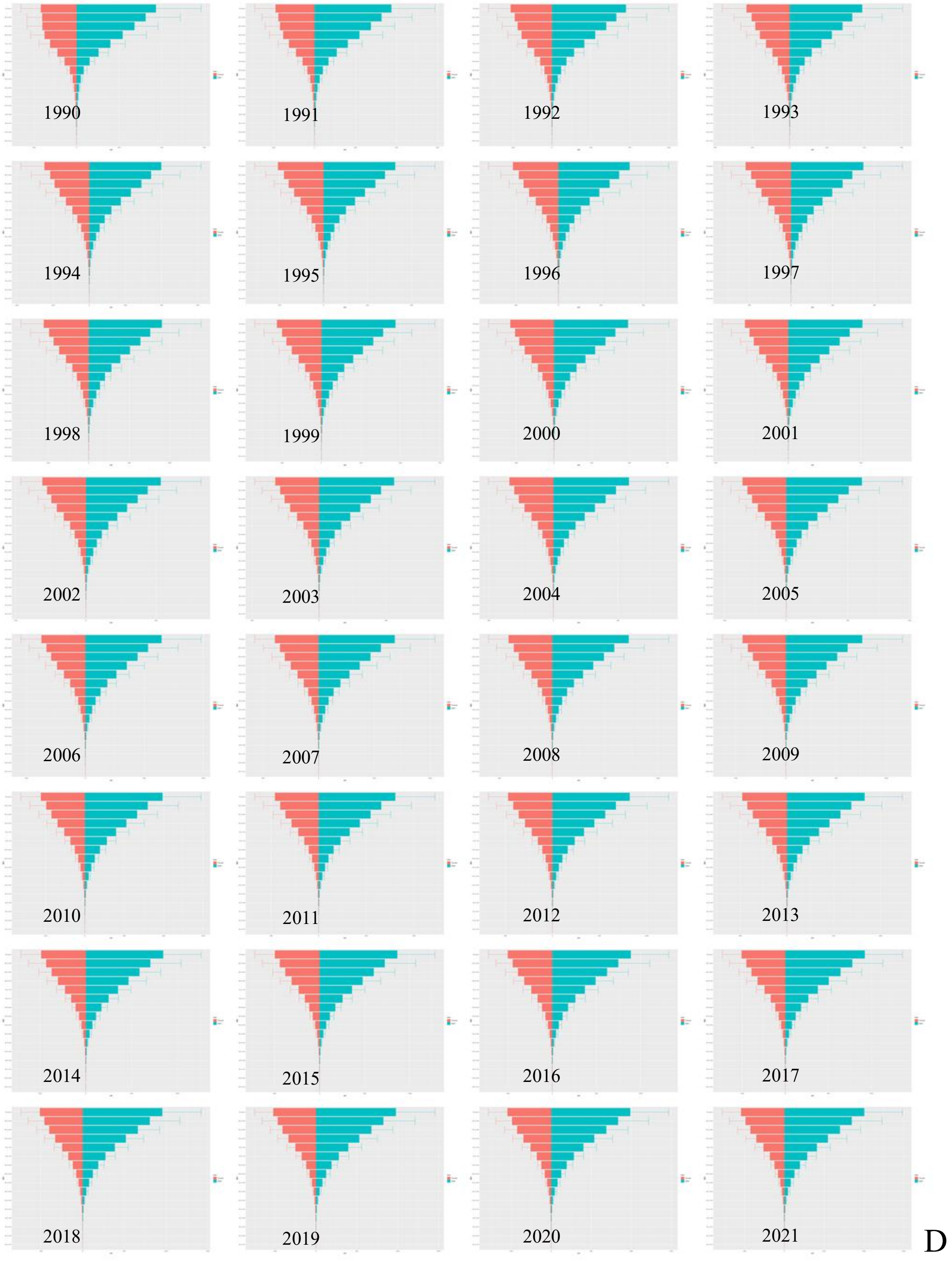

Fig. S74 (A) The prevalence cases of Parkinson's disease in different ages from 1990 to 2021 in China; (B) The prevalence rates of Parkinson's disease in different ages from 1990 to 2021 in China; (C) The years lived with disability of Parkinson's disease in different ages from 1990 to 2021 in China; (D) The years lived with disability rates of Parkinson's disease in different ages from 1990 to 2021 in China.

Notes: red for female, green for male; the ordinate from bottom to top is "20 to 24", "25 to 29", "30 to 34", "35 to 39", "40 to 44", "45 to 49", "50 to 54", "55 to 59", "60 to 64", "65 to 69", "70 to 74", "75 to 79", "80 to 84", "85 to 89", "90 to 94", "95 plus".

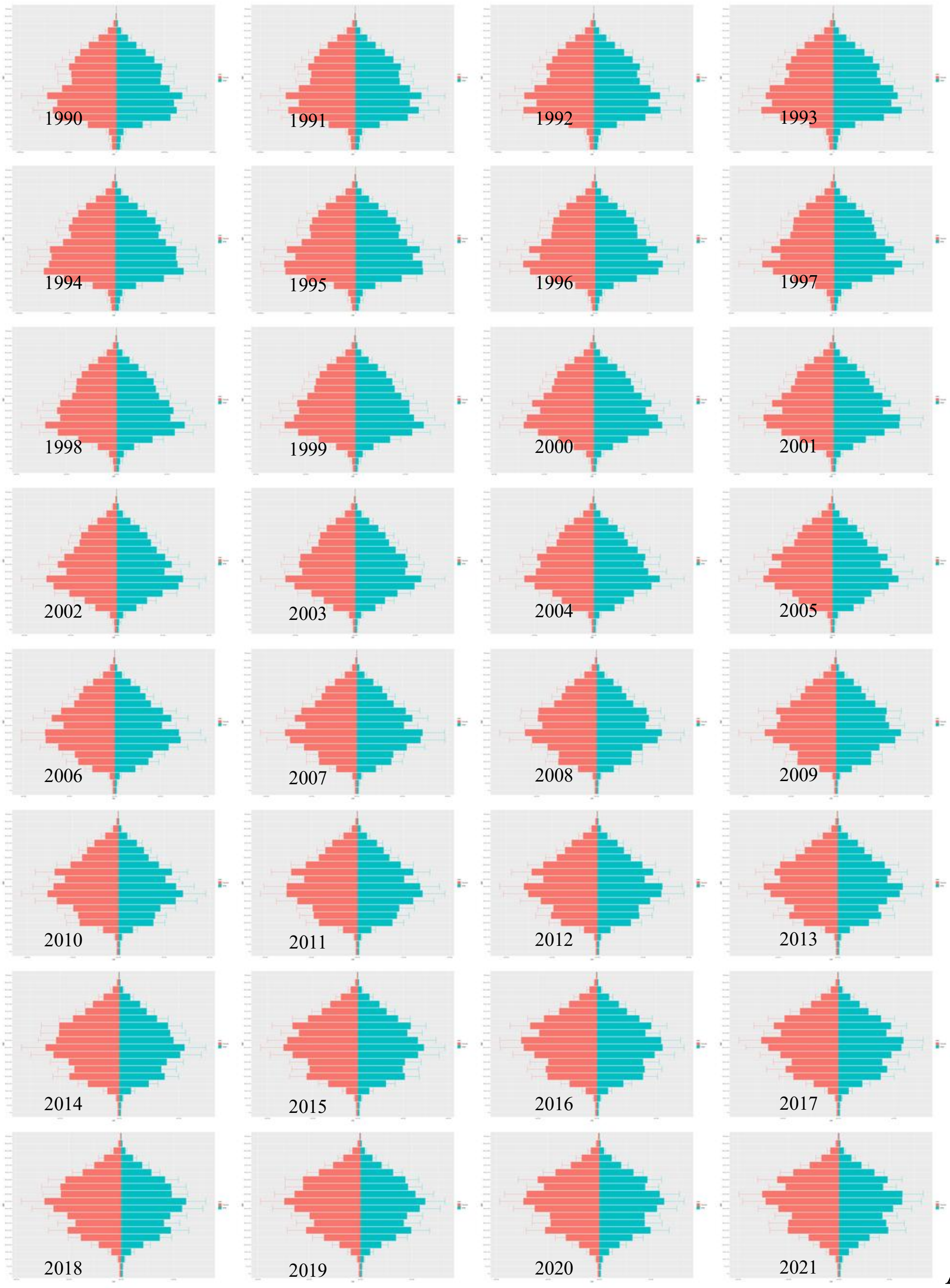
1990
1991
1992
1993
1994
1995
1996
1997
1998
1999
2000
2001
2002
2003
2004
2005
2006
2007
2008
2009
2010
2011
2012
2013
2014
2015
2016
2017
2018
2019
2020
2021

A

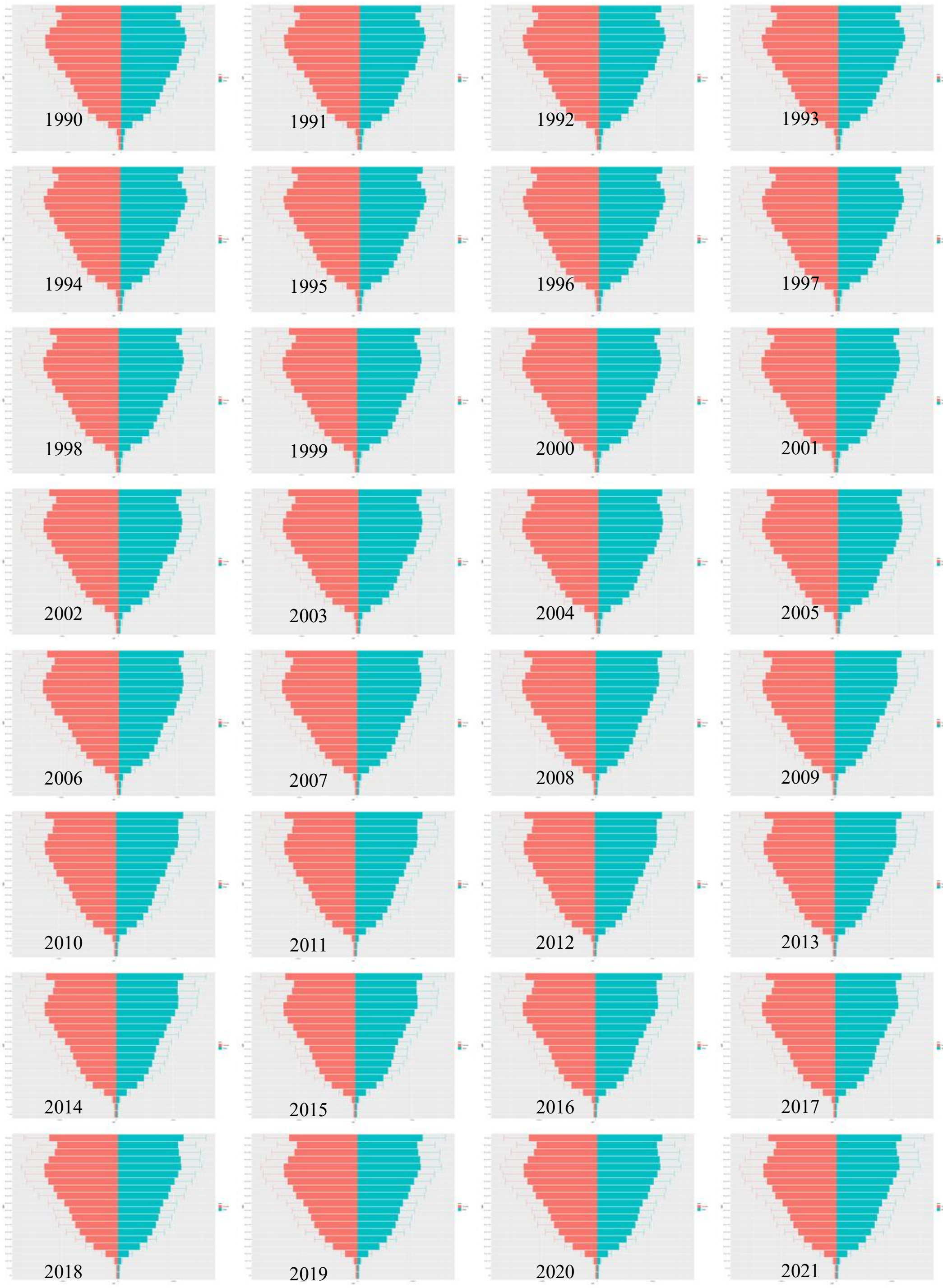
1990
1991
1992
1993
1994
1995
1996
1997
1998
1999
2000
2001
2002
2003
2004
2005
2006
2007
2008
2009
2010
2011
2012
2013
2014
2015
2016
2017
2018
2019
2020
2021
B

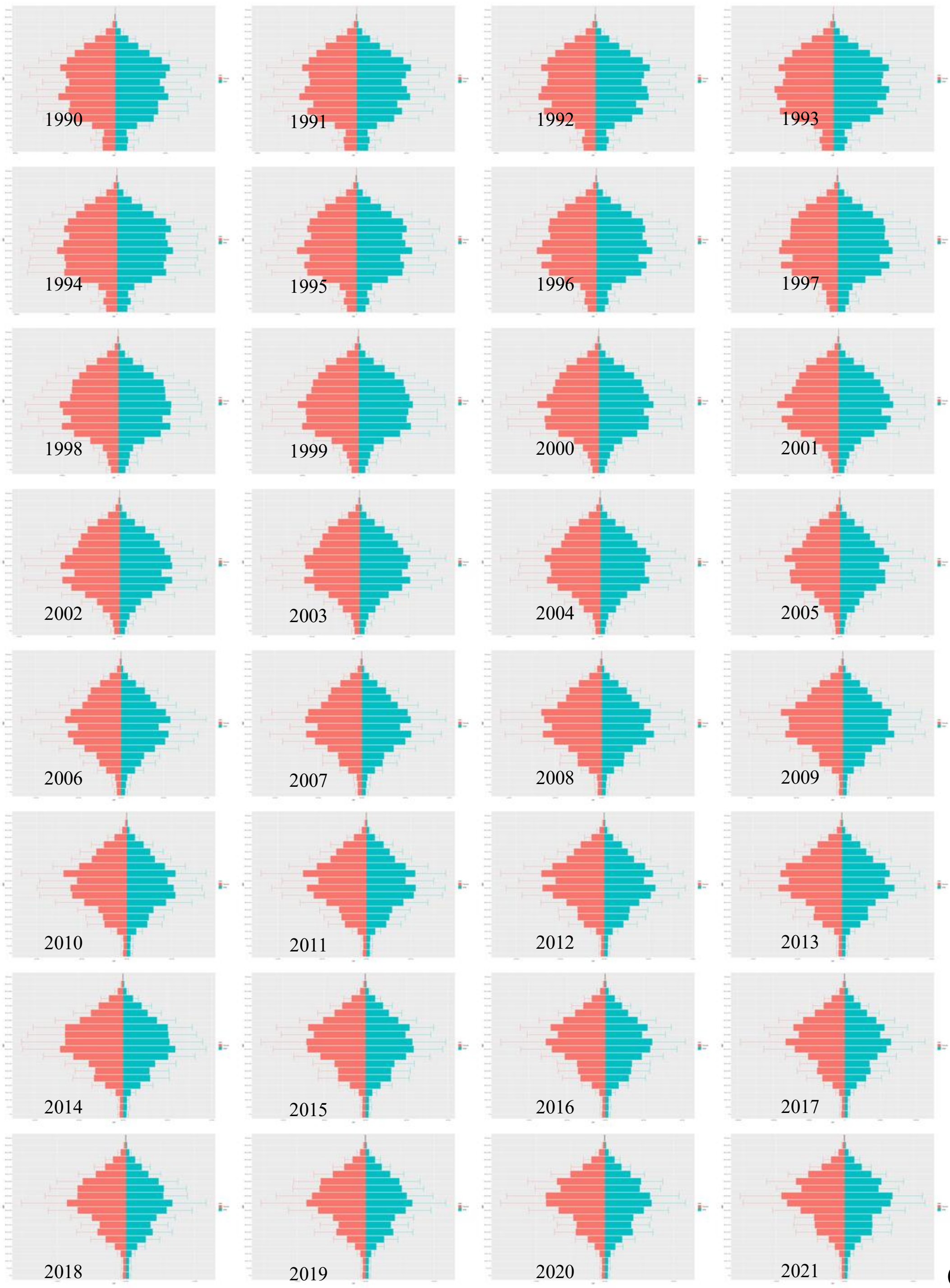
1990
1991
1992
1993
1994
1995
1996
1997
1998
1999
2000
2001
2002
2003
2004
2005
2006
2007
2008
2009
2010
2011
2012
2013
2014
2015
2016
2017
2018
2019
2020
2021

C

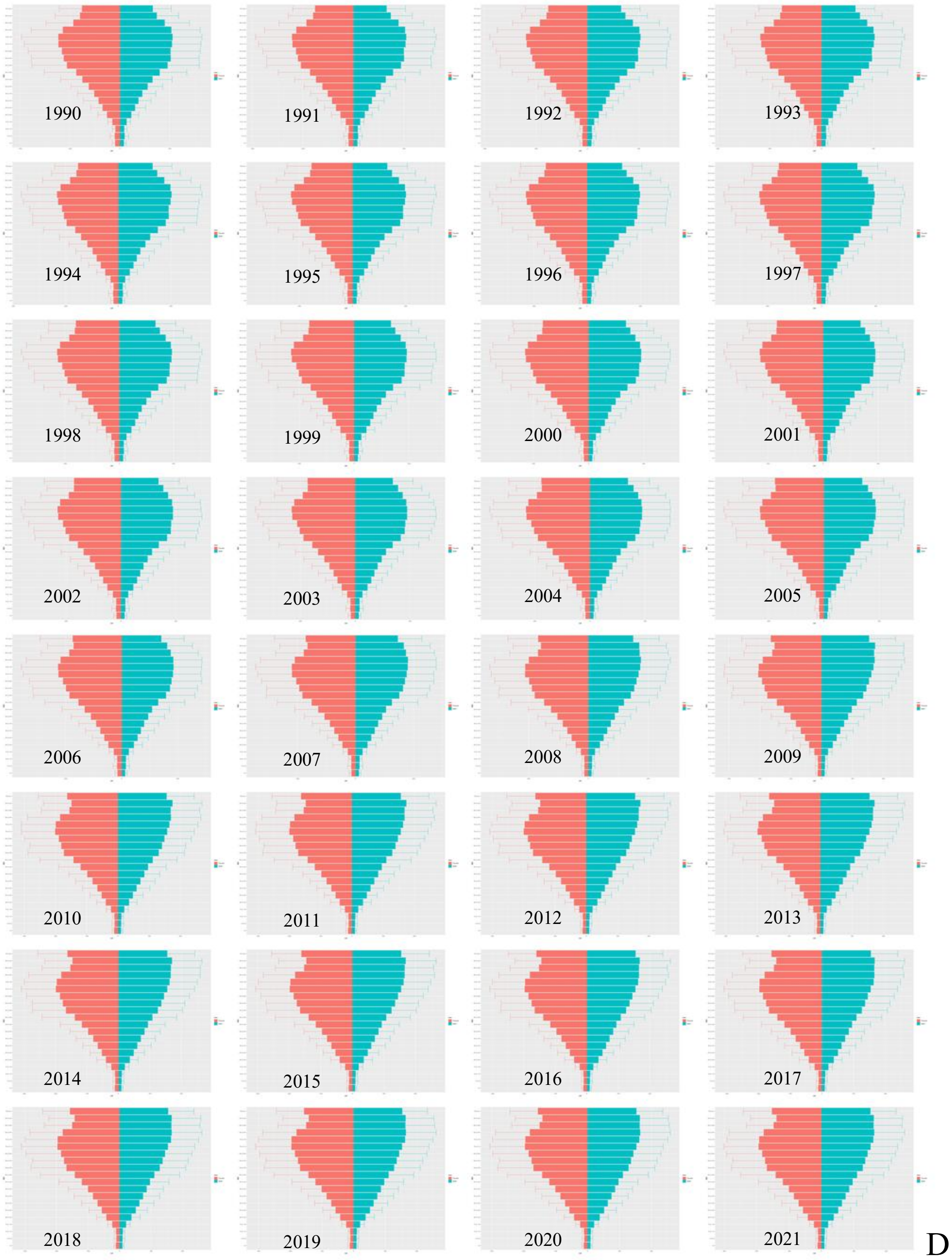

D

Fig. S75 (A) The prevalence cases of upper digestive system diseases in different ages from 1990 to 2021 in China; (B) The prevalence rates of upper digestive system diseases in different ages from 1990 to 2021 in China; (C) The years lived with disability of upper digestive system diseases in different ages from 1990 to 2021 in China; (D) The years lived with disability rates of upper digestive system diseases in different ages from 1990 to 2021 in China.

Notes: red for female, green for male; the ordinate from bottom to top is "<5", "5 to 9", "10 to 14", "15 to 19", "20 to 24", "25 to 29", "30 to 34", "35 to 39", "40 to 44", "45 to 49", "50 to 54", "55 to 59", "60 to 64", "65 to 69", "70 to 74", "75 to 79", "80 to 84", "85 to 89", "90 to 94", "95 plus".

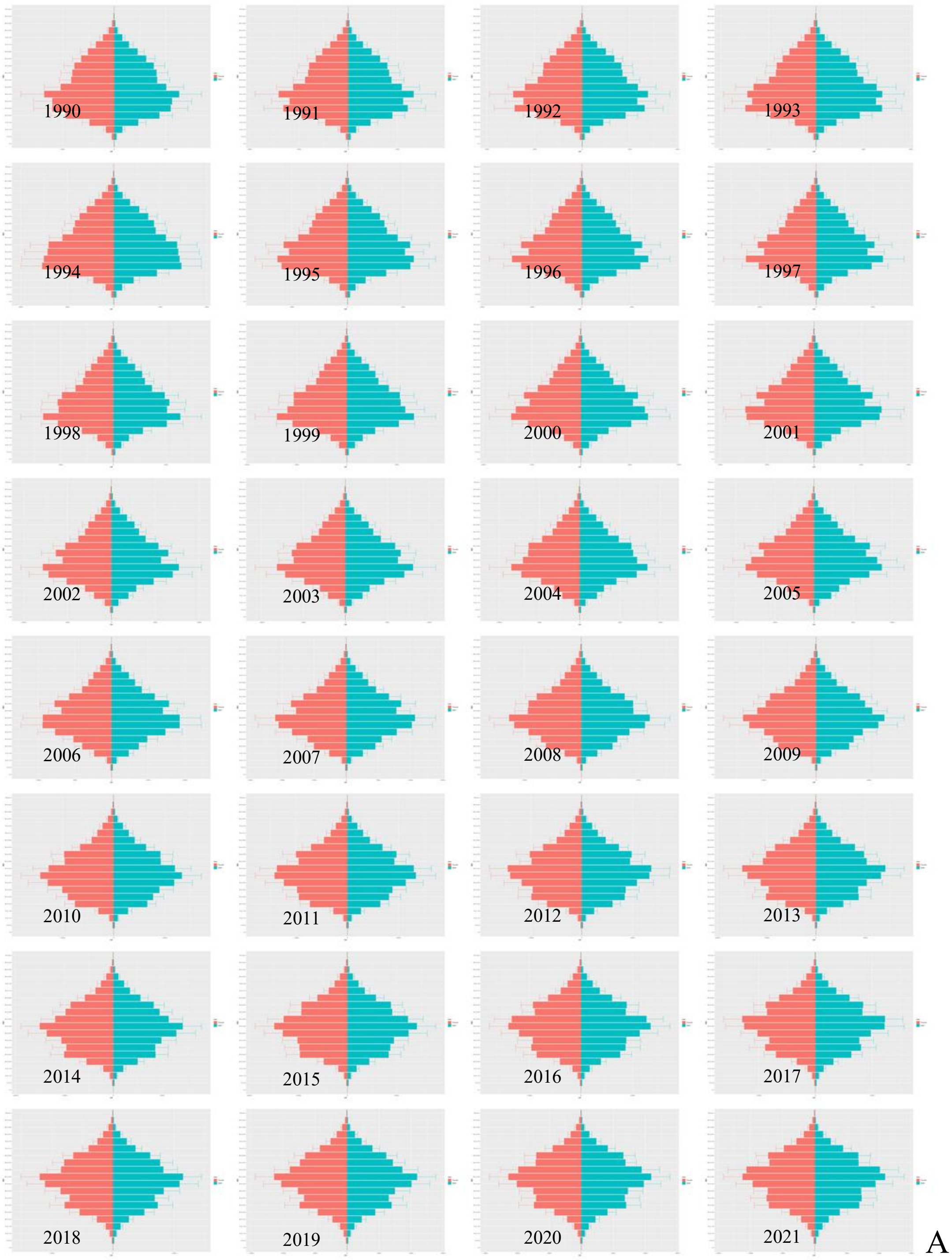
1990
1991
1992
1993
1994
1995
1996
1997
1998
1999
2000
2001
2002
2003
2004
2005
2006
2007
2008
2009
2010
2011
2012
2013
2014
2015
2016
2017
2018
2019
2020
2021
A

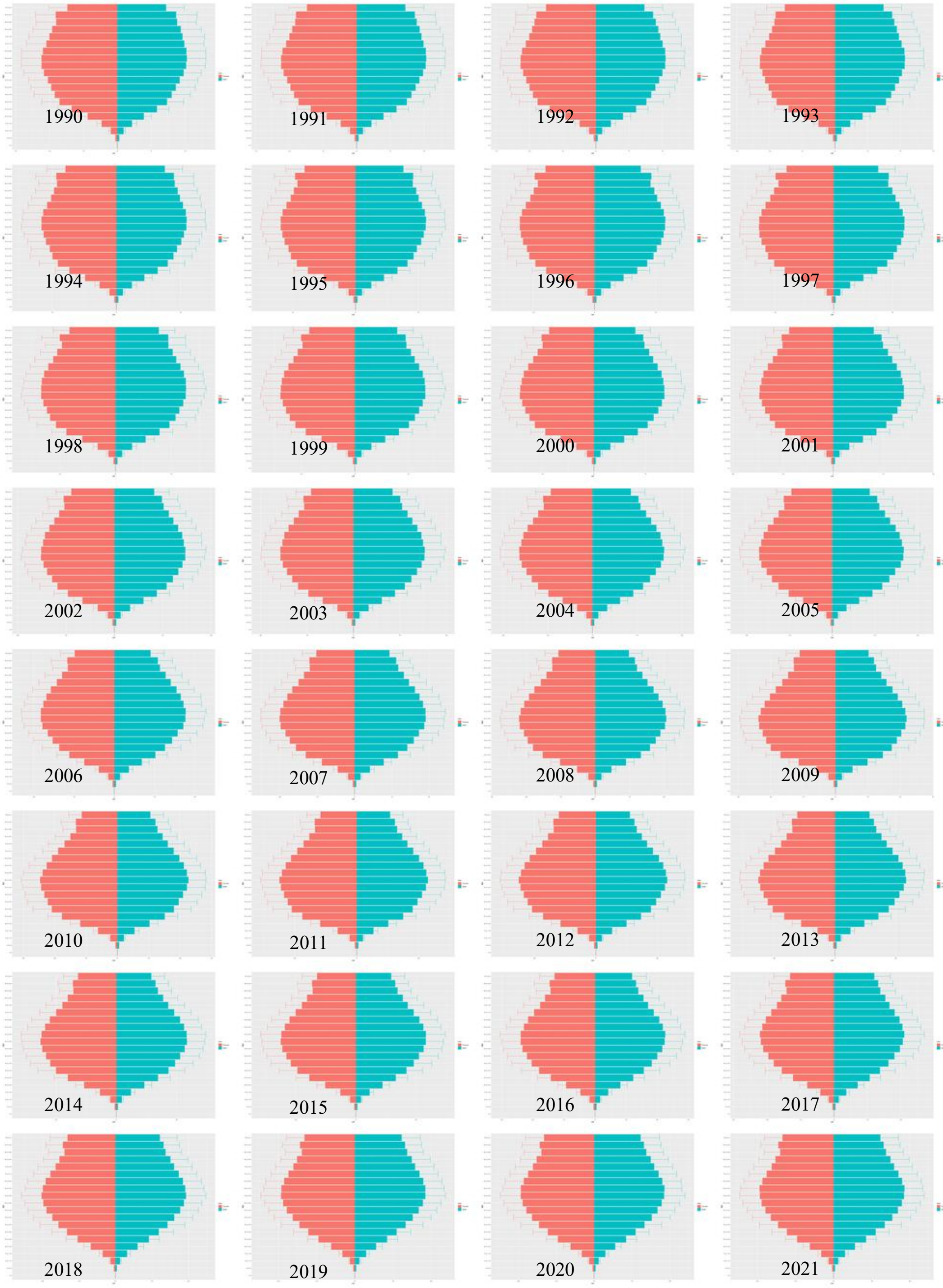
1990
1991
1992
1993
1994
1995
1996
1997
1998
1999
2000
2001
2002
2003
2004
2005
2006
2007
2008
2009
2010
2011
2012
2013
2014
2015
2016
2017
2018
2019
2020
2021
B

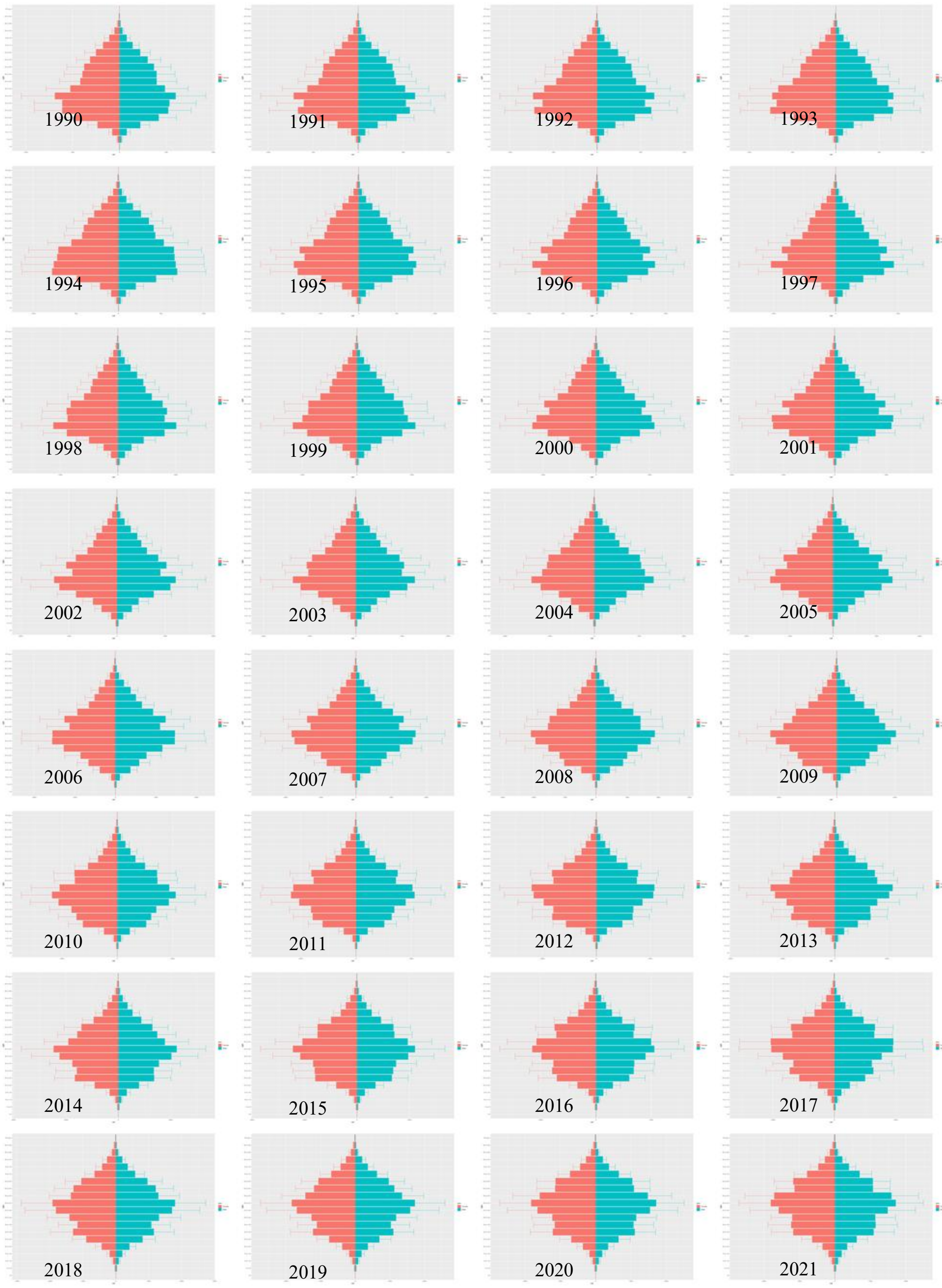
1990
1991
1992
1993
1994
1995
1996
1997
1998
1999
2000
2001
2002
2003
2004
2005
2006
2007
2008
2009
2010
2011
2012
2013
2014
2015
2016
2017
2018
2019
2020
2021

C

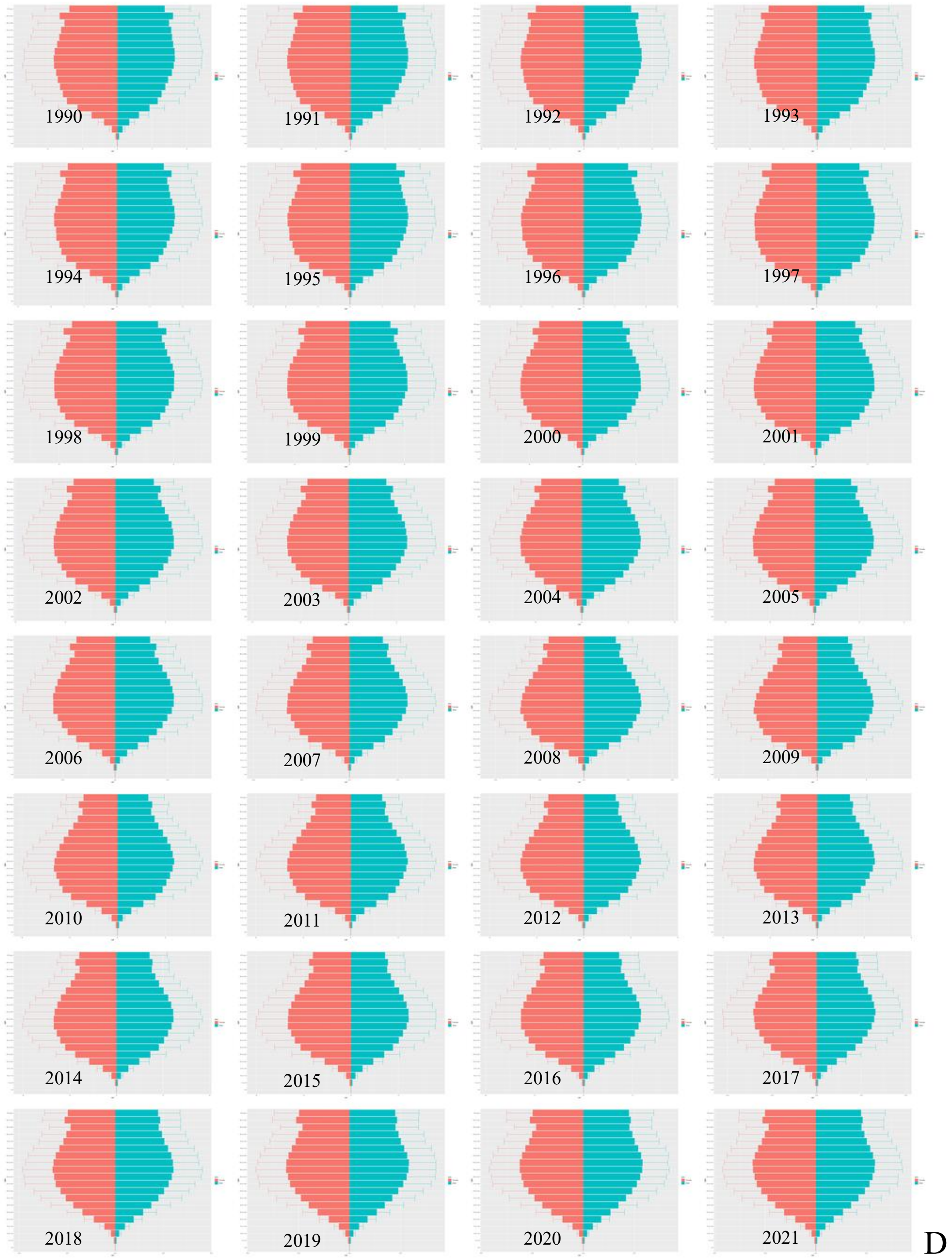

Fig. S76 (A) The prevalence cases of inflammatory bowel disease in different ages from 1990 to 2021 in China; (B) The prevalence rates of inflammatory bowel disease in different ages from 1990 to 2021 in China; (C) The years lived with disability of inflammatory bowel disease in different ages from 1990 to 2021 in China; (D) The years lived with disability rates of inflammatory bowel disease in different ages from 1990 to 2021 in China.

Notes: red for female, green for male; the ordinate from bottom to top is "<5", "5 to 9", "10 to 14", "15 to 19", "20 to 24", "25 to 29", "30 to 34", "35 to 39", "40 to 44", "45 to 49", "50 to 54", "55 to 59", "60 to 64", "65 to 69", "70 to 74", "75 to 79", "80 to 84", "85 to 89", "90 to 94", "95 plus".

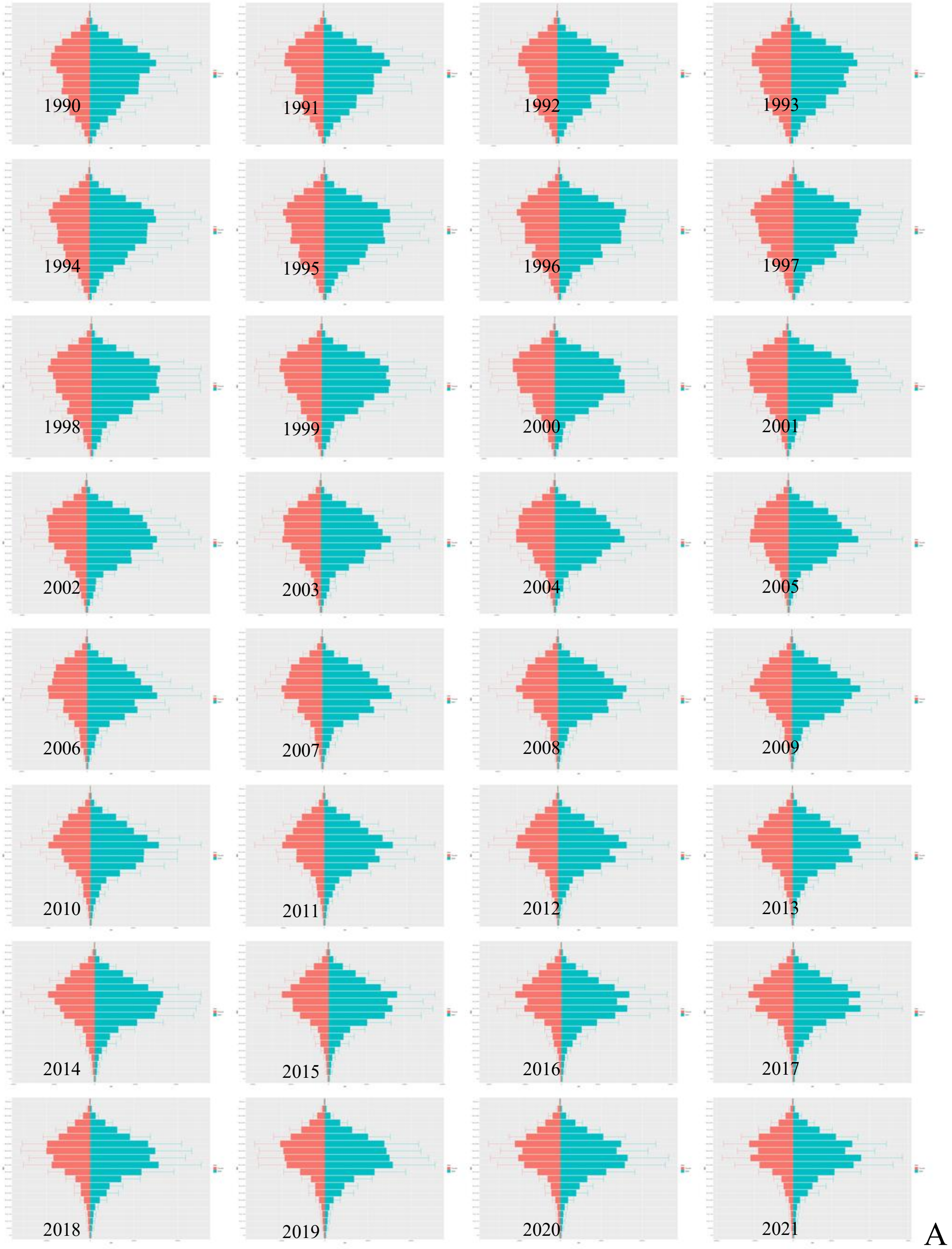
1990
1991
1992
1993
1994
1995
1996
1997
1998
1999
2000
2001
2002
2003
2004
2005
2006
2007
2008
2009
2010
2011
2012
2013
2014
2015
2016
2017
2018
2019
2020
2021
A

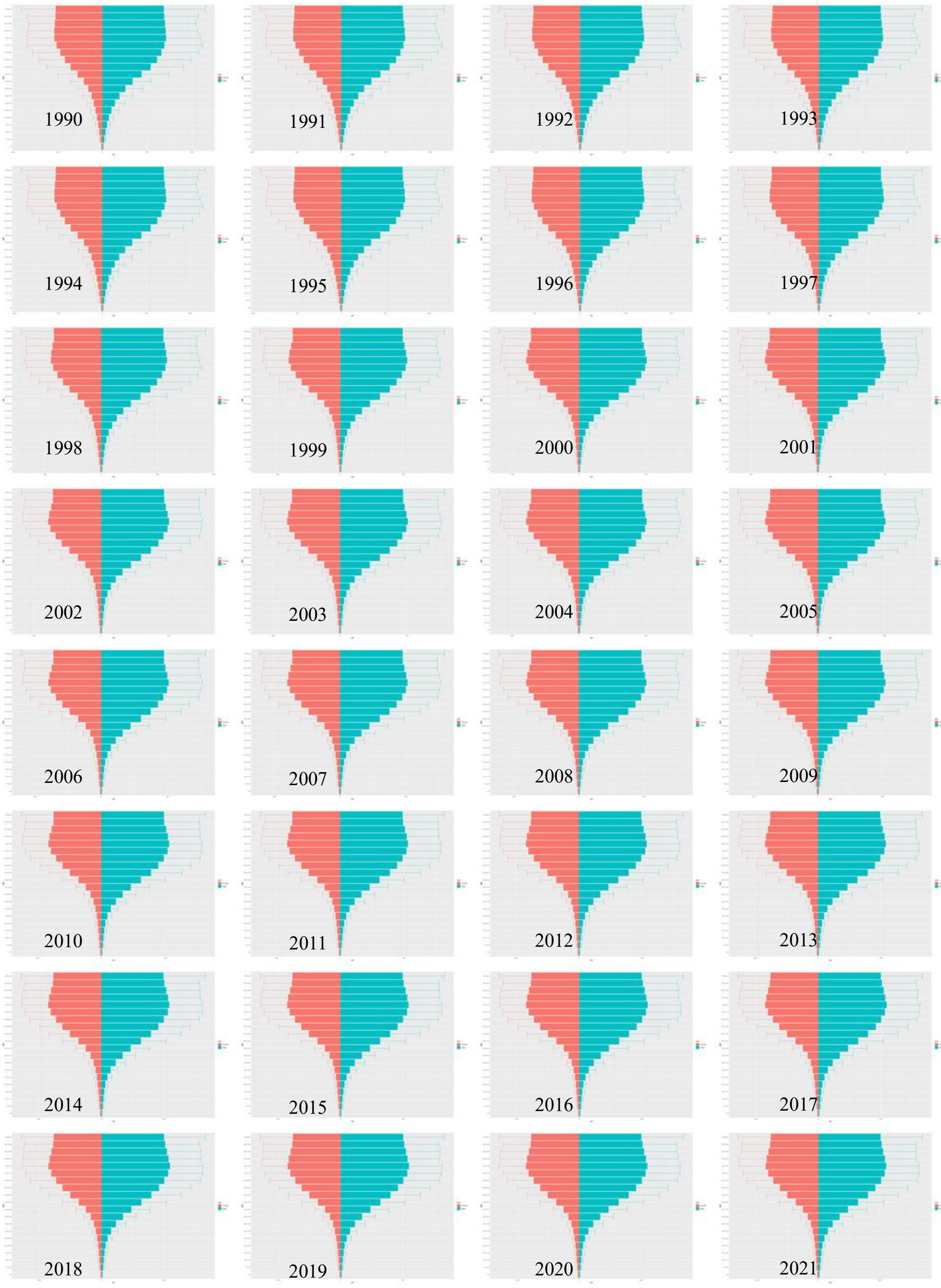
1990
1991
1992
1993
1994
1995
1996
1997
1998
1999
2000
2001
2002
2003
2004
2005
2006
2007
2008
2009
2010
2011
2012
2013
2014
2015
2016
2017
2018
2019
2020
2021
B

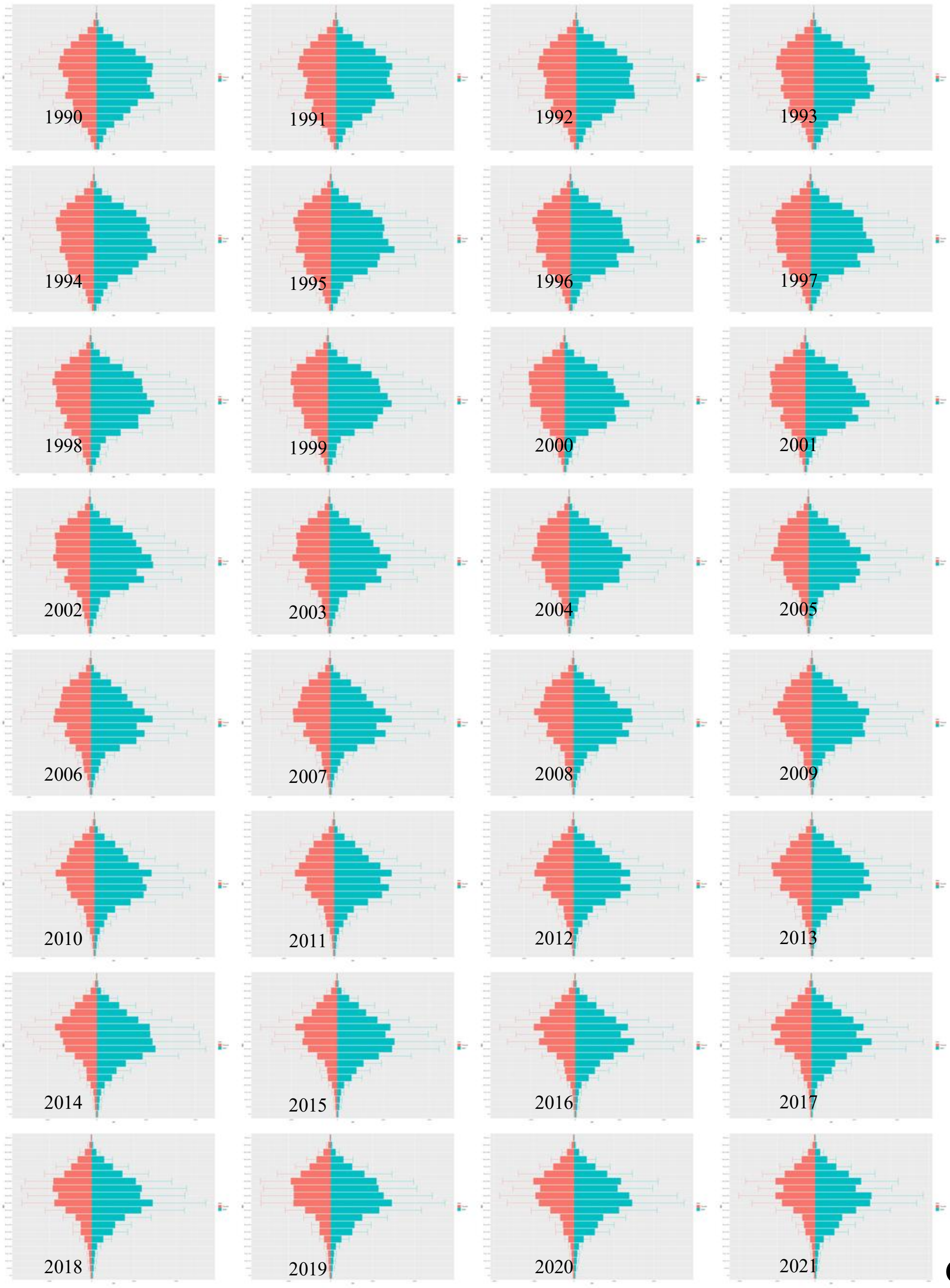
1990
1991
1992
1993
1994
1995
1996
1997
1998
1999
2000
2001
2002
2003
2004
2005
2006
2007
2008
2009
2010
2011
2012
2013
2014
2015
2016
2017
2018
2019
2020
2021

C

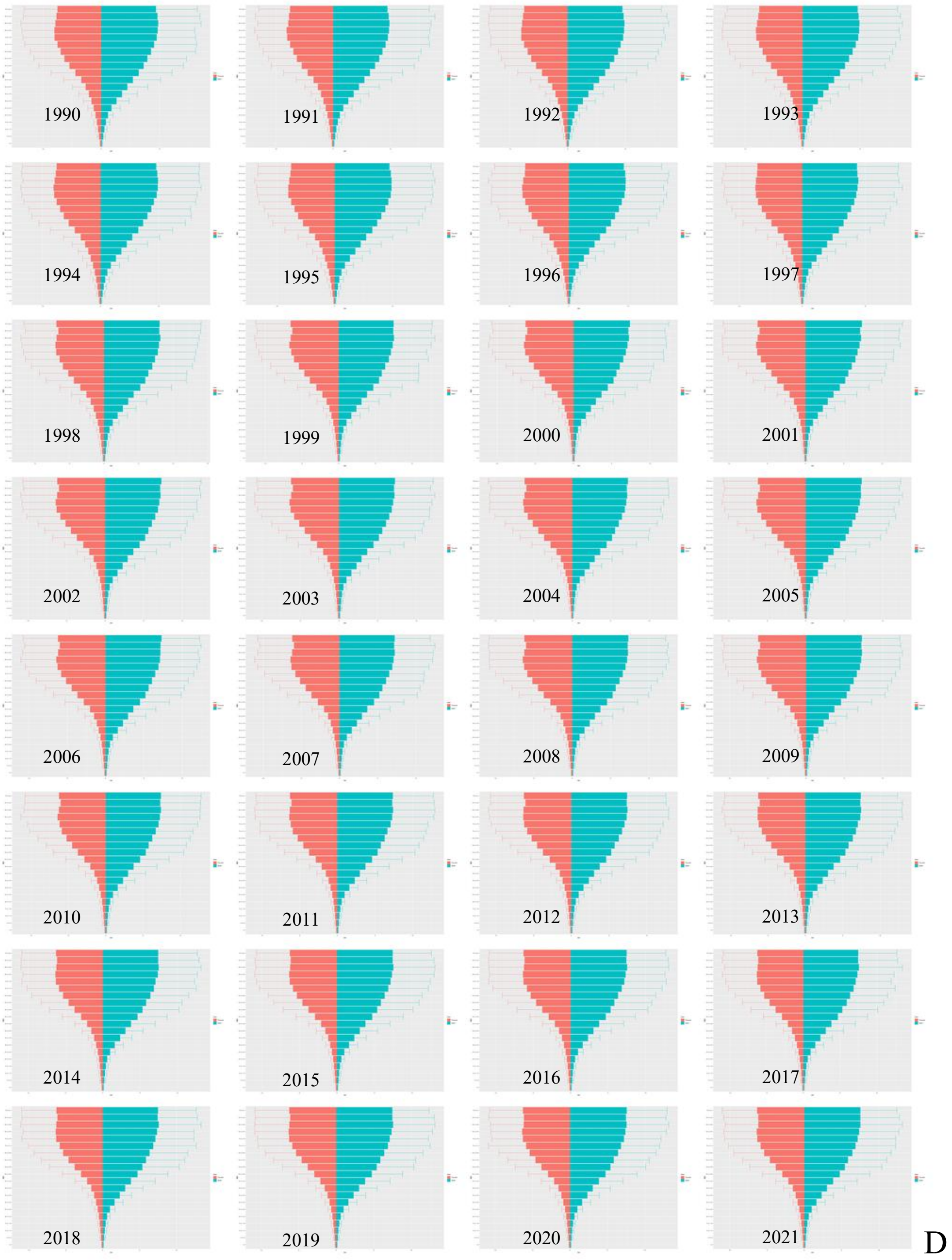

Fig. S77 (A) The prevalence cases of pancreatitis in different ages from 1990 to 2021 in China; (B) The prevalence rates of pancreatitis in different ages from 1990 to 2021 in China; (C) The years lived with disability of pancreatitis in different ages from 1990 to 2021 in China; (D) The years lived with disability rates of pancreatitis in different ages from 1990 to 2021 in China.

Notes: red for female, green for male; the ordinate from bottom to top is "<5", "5 to 9", "10 to 14", "15 to 19", "20 to 24", "25 to 29", "30 to 34", "35 to 39", "40 to 44", "45 to 49", "50 to 54", "55 to 59", "60 to 64", "65 to 69", "70 to 74", "75 to 79", "80 to 84", "85 to 89", "90 to 94", "95 plus".

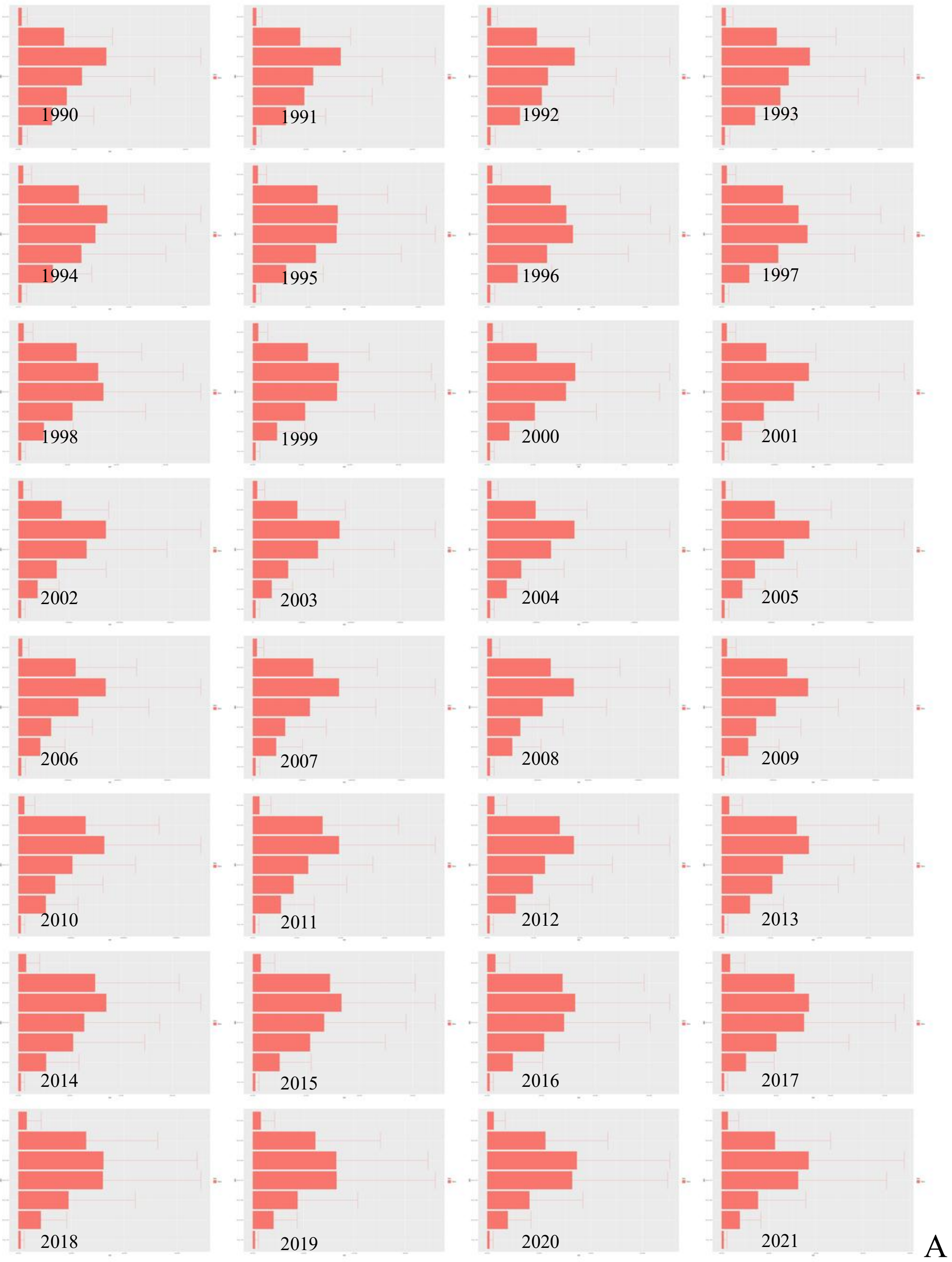
1990
1991
1992
1993
1994
1995
1996
1997
1998
1999
2000
2001
2002
2003
2004
2005
2006
2007
2008
2009
2010
2011
2012
2013
2014
2015
2016
2017
2018
2019
2020
2021
A

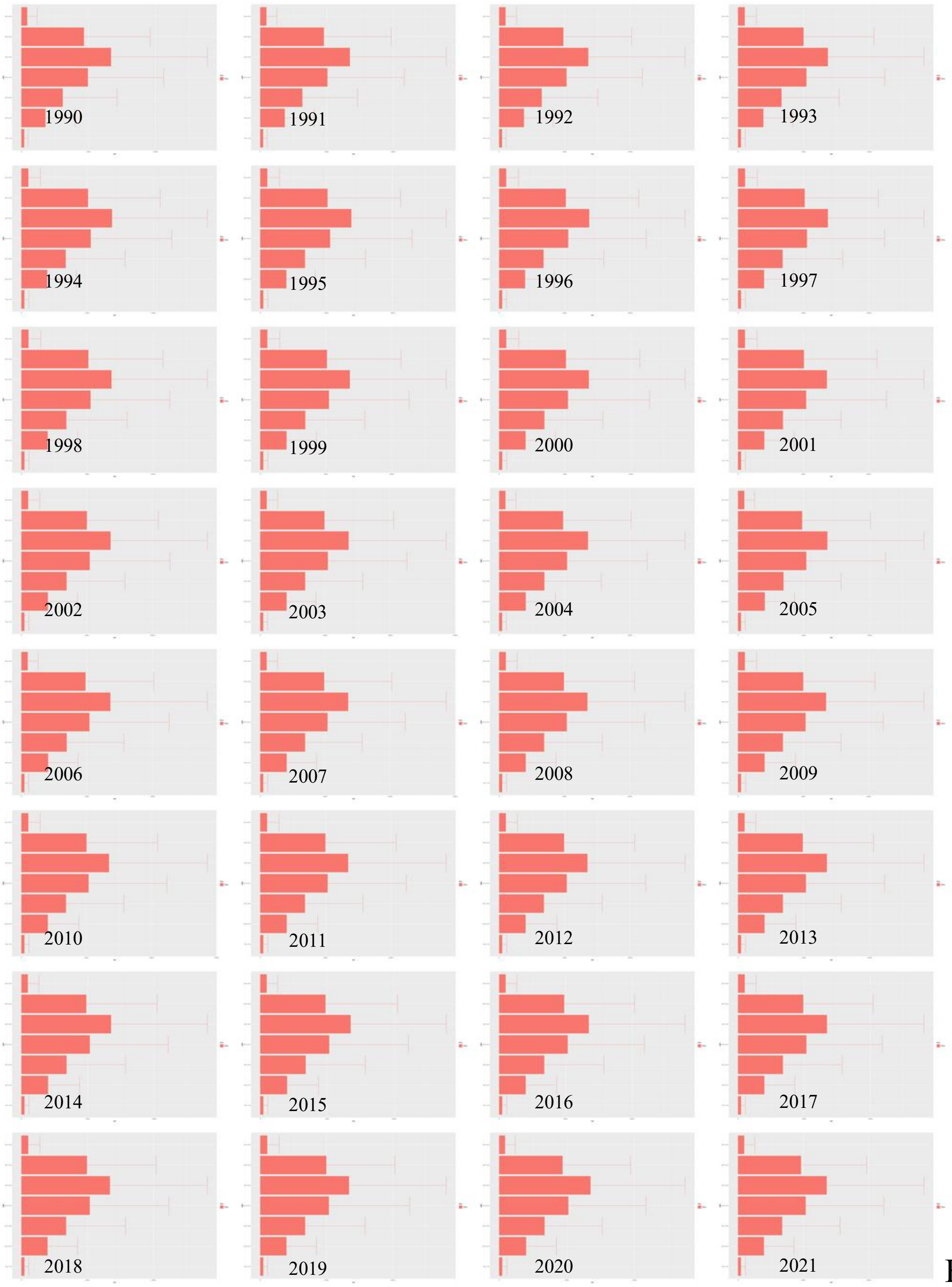
1990
1991
1992
1993
1994
1995
1996
1997
1998
1999
2000
2001
2002
2003
2004
2005
2006
2007
2008
2009
2010
2011
2012
2013
2014
2015
2016
2017
2018
2019
2020
2021
B

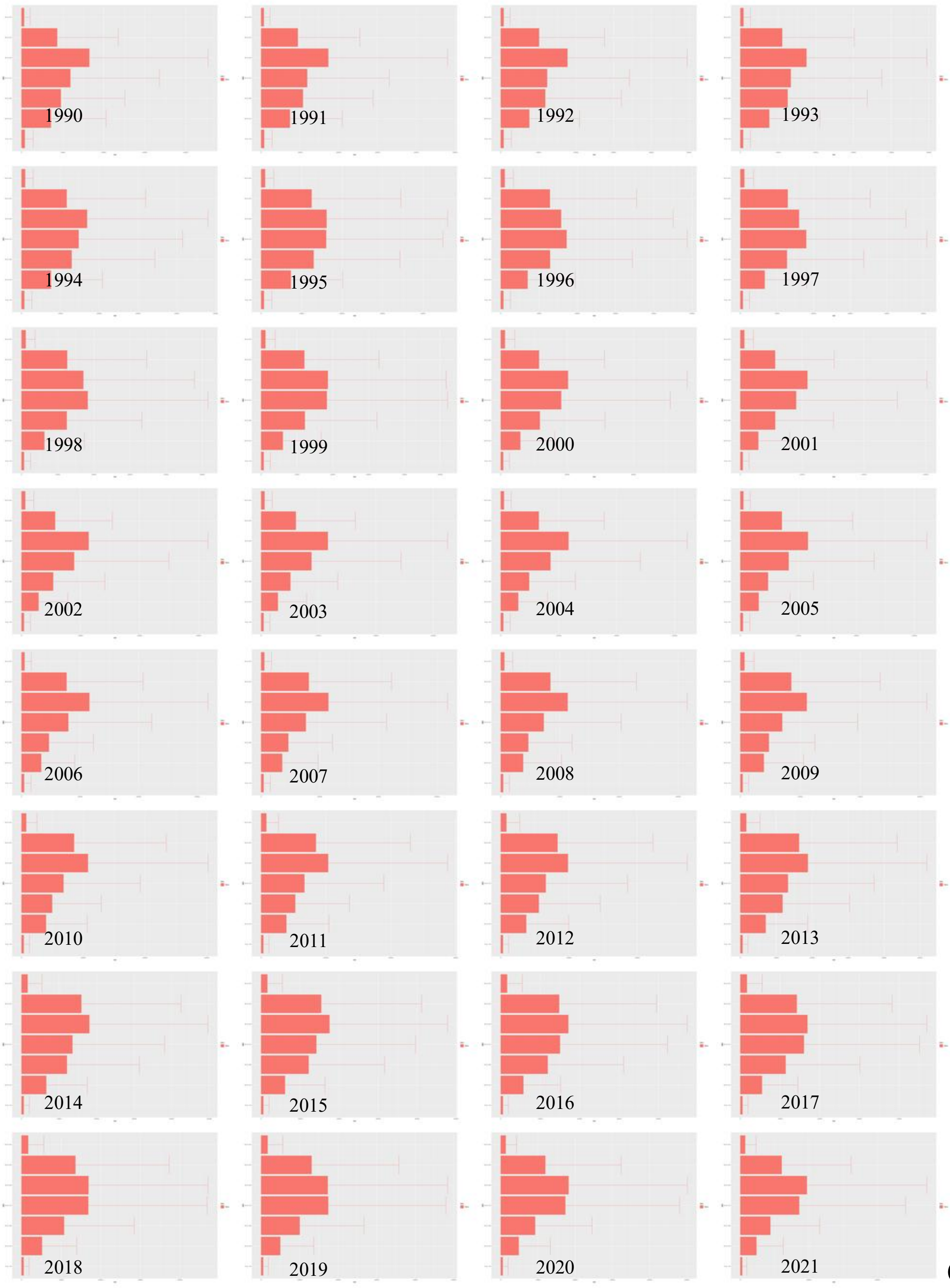
1990
1991
1992
1993
1994
1995
1996
1997
1998
1999
2000
2001
2002
2003
2004
2005
2006
2007
2008
2009
2010
2011
2012
2013
2014
2015
2016
2017
2018
2019
2020
2021

C

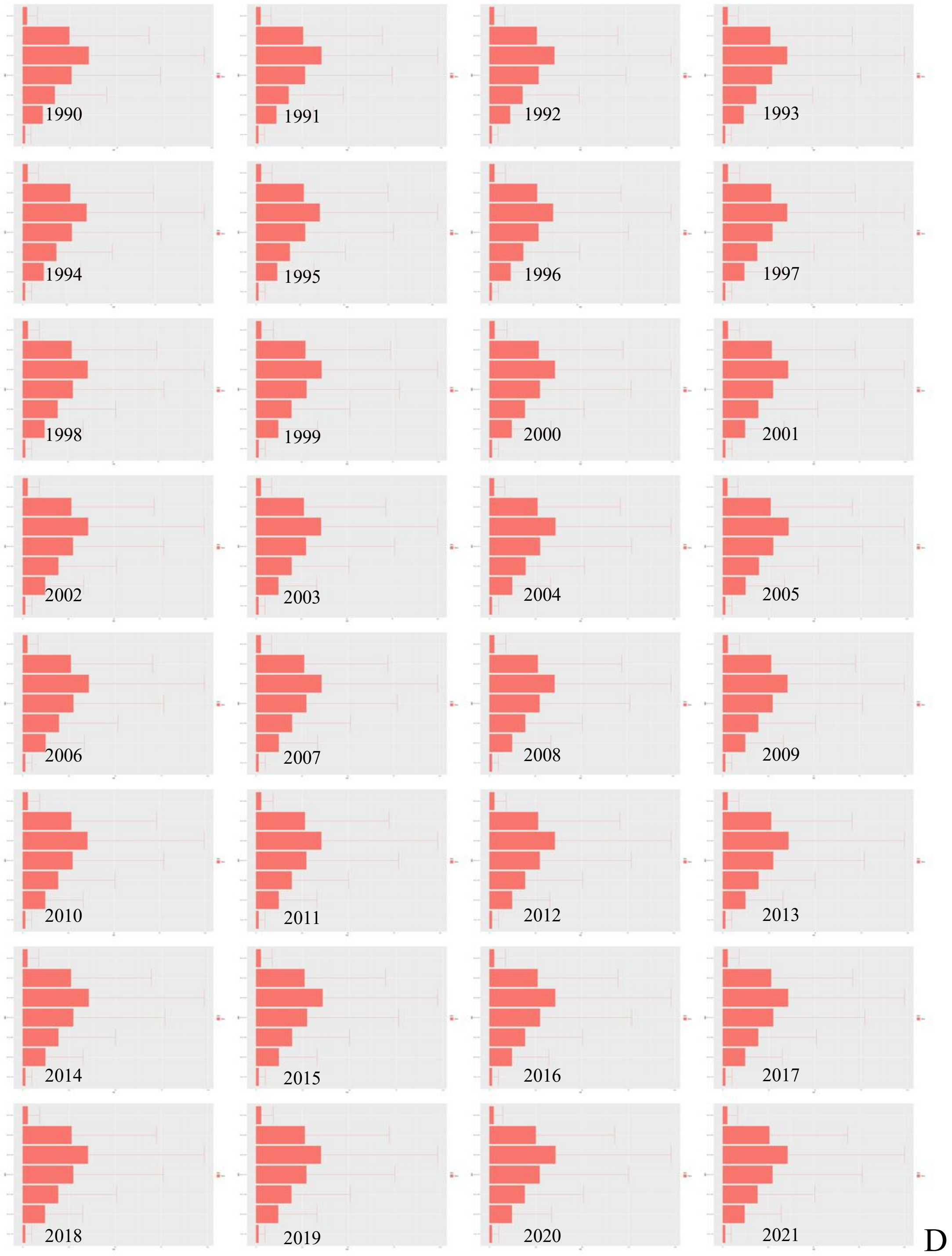

Fig. S78 (A) The prevalence cases of male infertility in different ages from 1990 to 2021 in China; (B) The prevalence rates of male infertility in different ages from 1990 to 2021 in China; (C) The years lived with disability of male infertility in different ages from 1990 to 2021 in China; (D) The years lived with disability rates of male infertility in different ages from 1990 to 2021 in China.

Notes: red for male; the ordinate from bottom to top is "15 to 19", "20 to 24", "25 to 29", "30 to 34", "35 to 39", "40 to 44", "45 to 49".

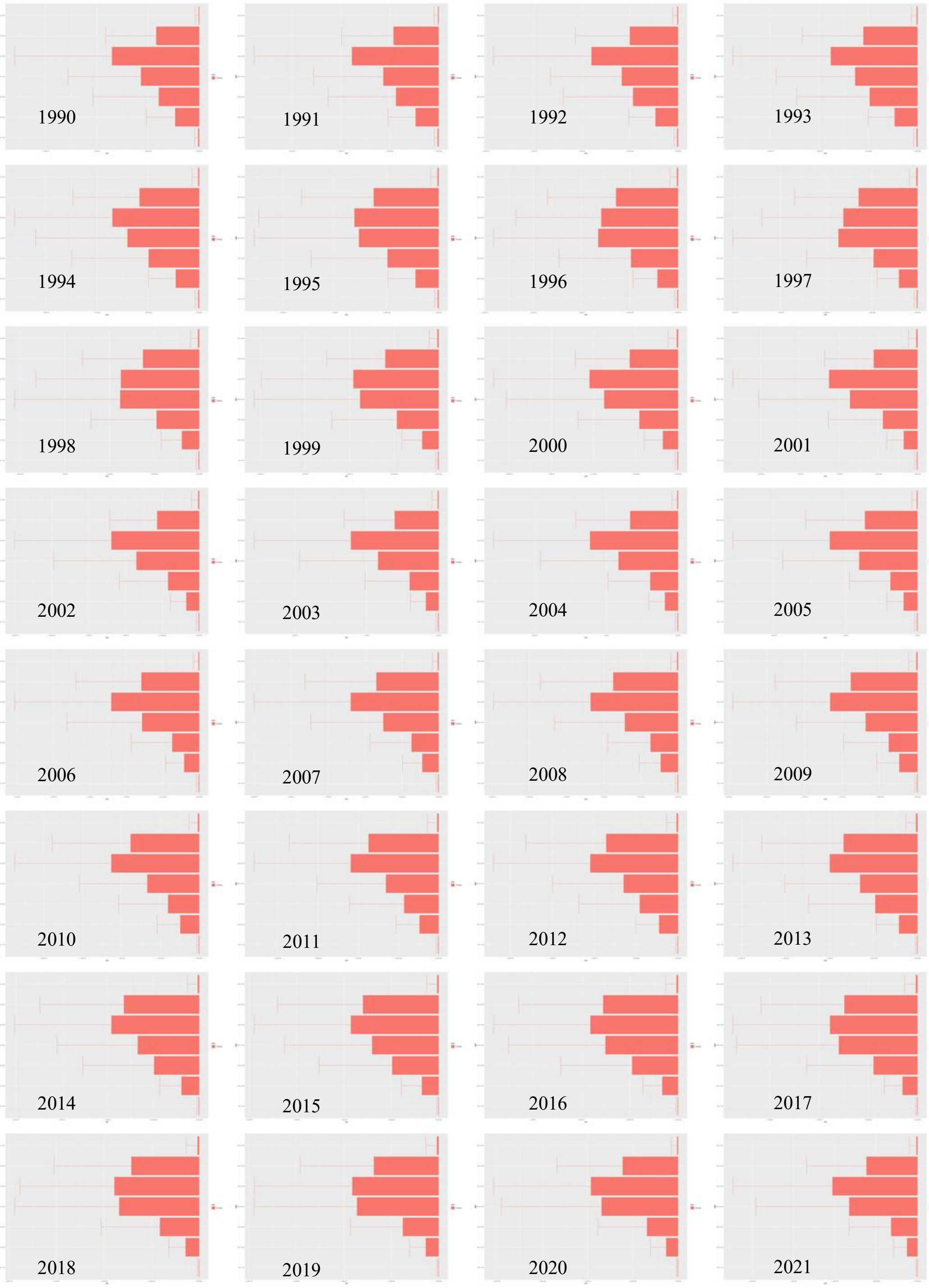
1990
1991
1992
1993
1994
1995
1996
1997
1998
1999
2000
2001
2002
2003
2004
2005
2006
2007
2008
2009
2010
2011
2012
2013
2014
2015
2016
2017
2018
2019
2020
2021

A

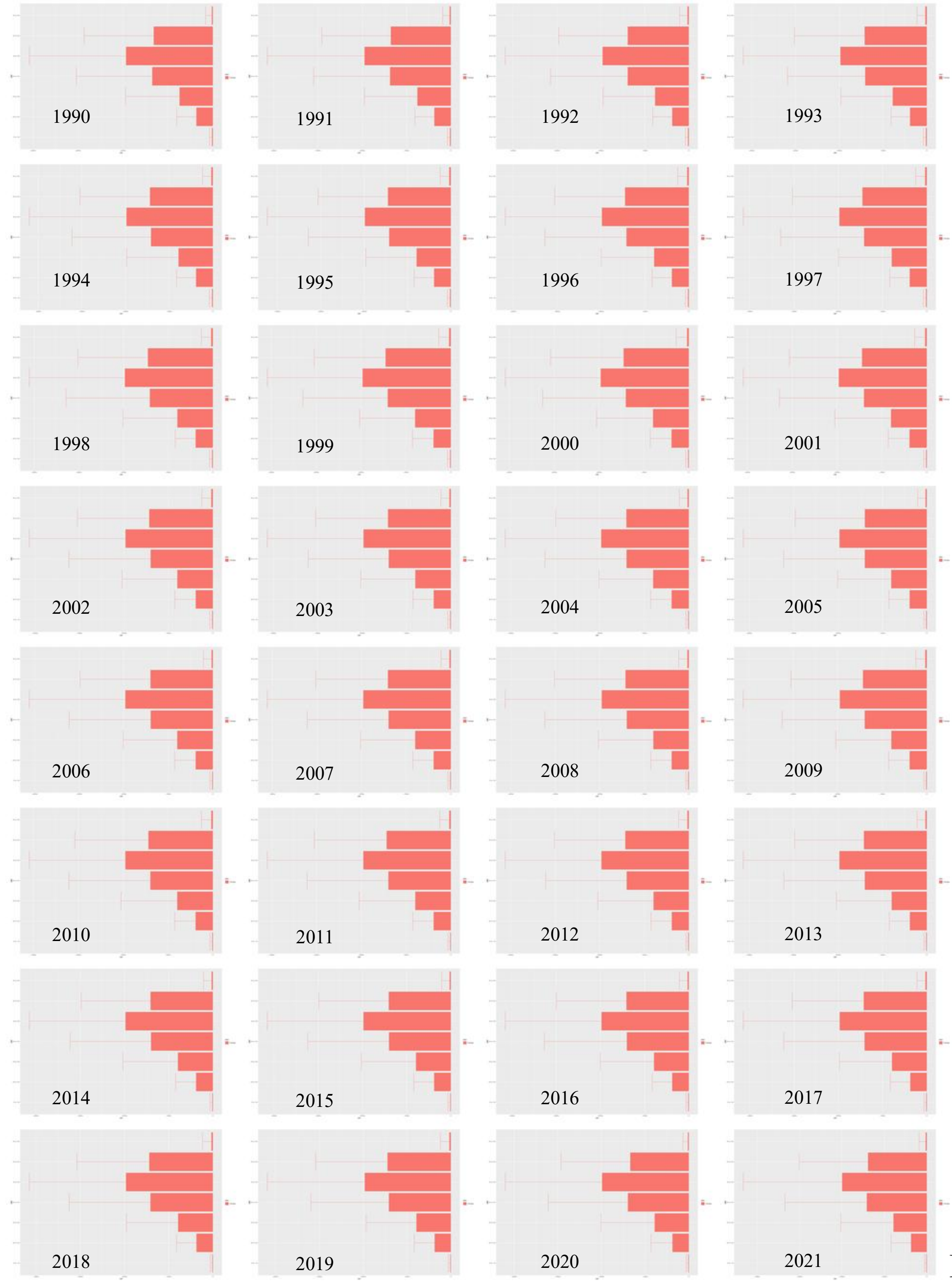
1990
1991
1992
1993
1994
1995
1996
1997
1998
1999
2000
2001
2002
2003
2004
2005
2006
2007
2008
2009
2010
2011
2012
2013
2014
2015
2016
2017
2018
2019
2020
2021

B

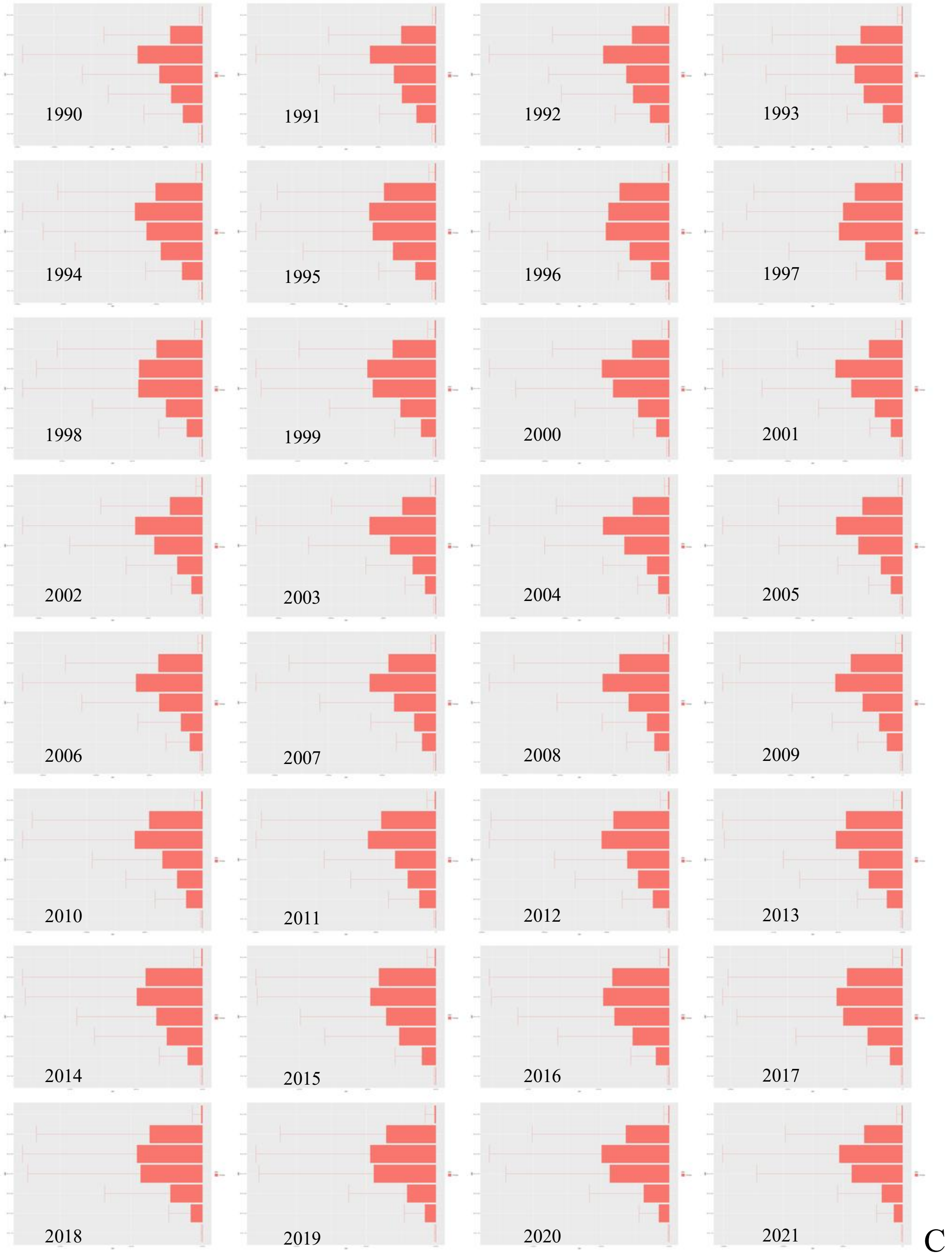
1990
1991
1992
1993
1994
1995
1996
1997
1998
1999
2000
2001
2002
2003
2004
2005
2006
2007
2008
2009
2010
2011
2012
2013
2014
2015
2016
2017
2018
2019
2020
2021
C

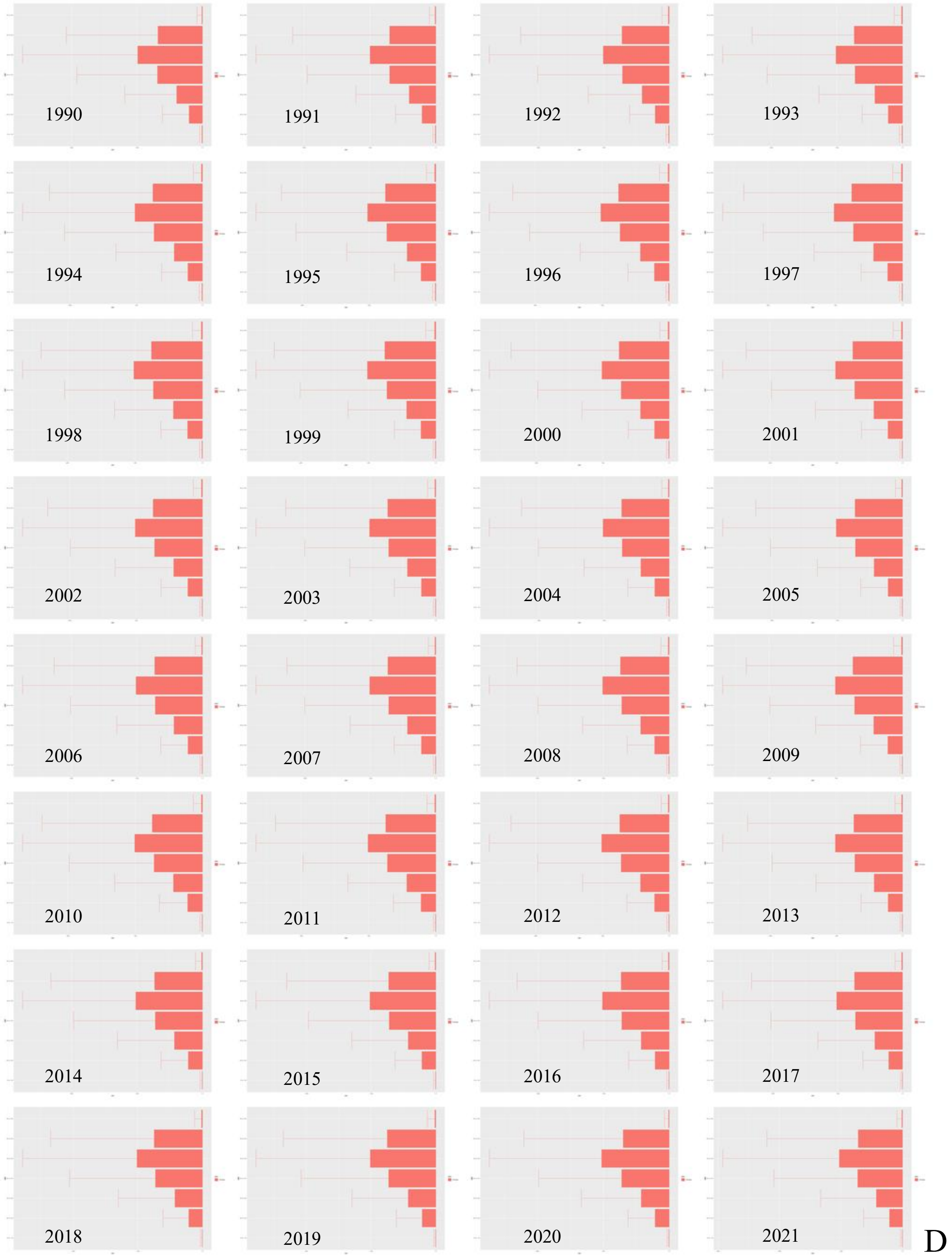

Fig. S79 (A) The prevalence cases of female infertility in different ages from 1990 to 2021 in China; (B) The prevalence rates of female infertility in different ages from 1990 to 2021 in China; (C) The years lived with disability of female infertility in different ages from 1990 to 2021 in China; (D) The years lived with disability rates of female infertility in different ages from 1990 to 2021 in China.

Notes: red for female; the ordinate from bottom to top is "<5", "5 to 9", "10 to 14", "15 to 19", "20 to 24", "25 to 29", "30 to 34", "35 to 39", "40 to 44", "45 to 49", "50 to 54", "55 to 59", "60 to 64", "65 to 69", "70 to 74", "75 to 79", "80 to 84", "85 to 89", "90 to 94", "95 plus".

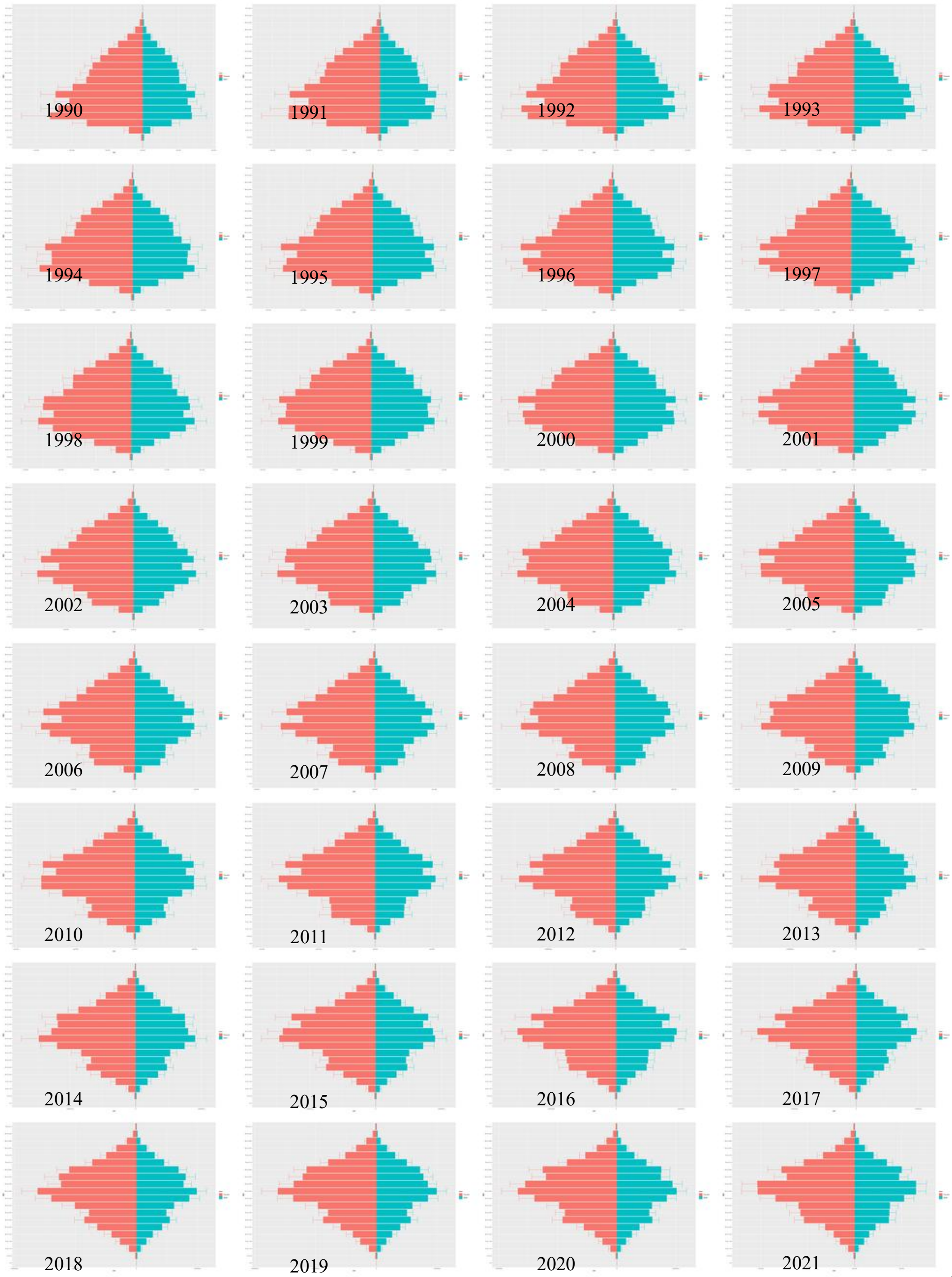
1990
1991
1992
1993
1994
1995
1996
1997
1998
1999
2000
2001
2002
2003
2004
2005
2006
2007
2008
2009
2010
2011
2012
2013
2014
2015
2016
2017
2018
2019
2020
2021

A

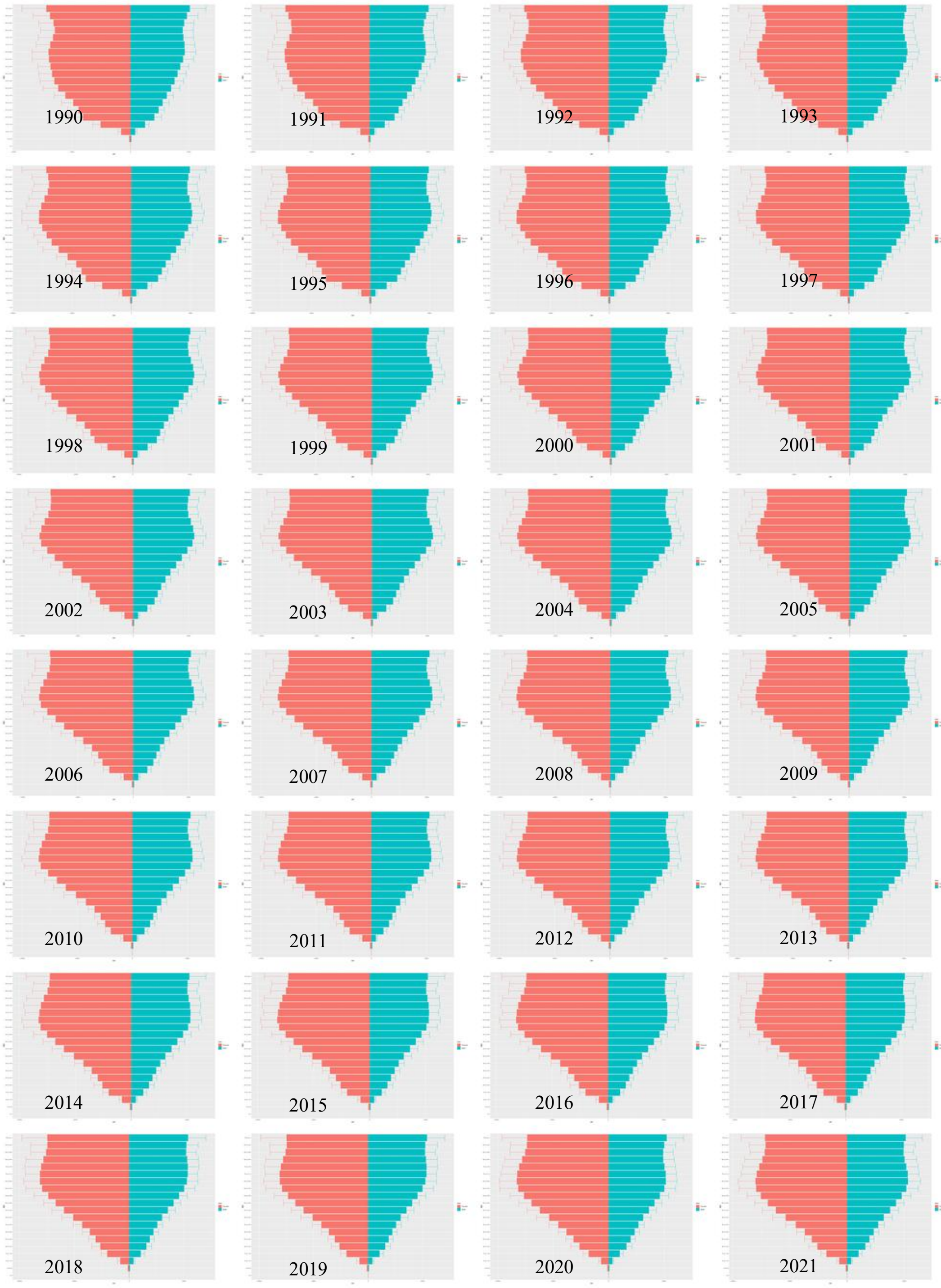
1990
1991
1992
1993
1994
1995
1996
1997
1998
1999
2000
2001
2002
2003
2004
2005
2006
2007
2008
2009
2010
2011
2012
2013
2014
2015
2016
2017
2018
2019
2020
2021
B

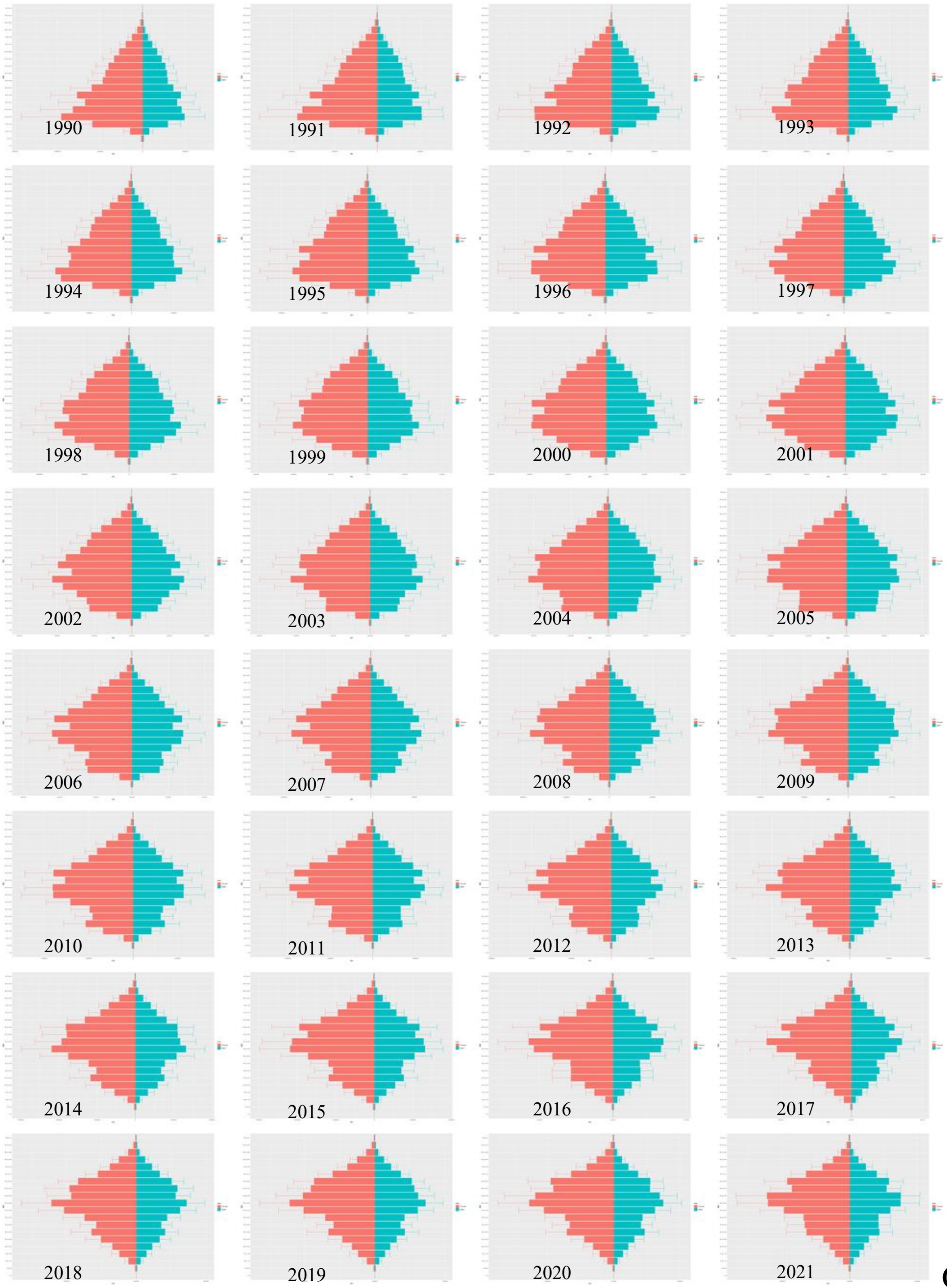
1990
1991
1992
1993
1994
1995
1996
1997
1998
1999
2000
2001
2002
2003
2004
2005
2006
2007
2008
2009
2010
2011
2012
2013
2014
2015
2016
2017
2018
2019
2020
2021

C

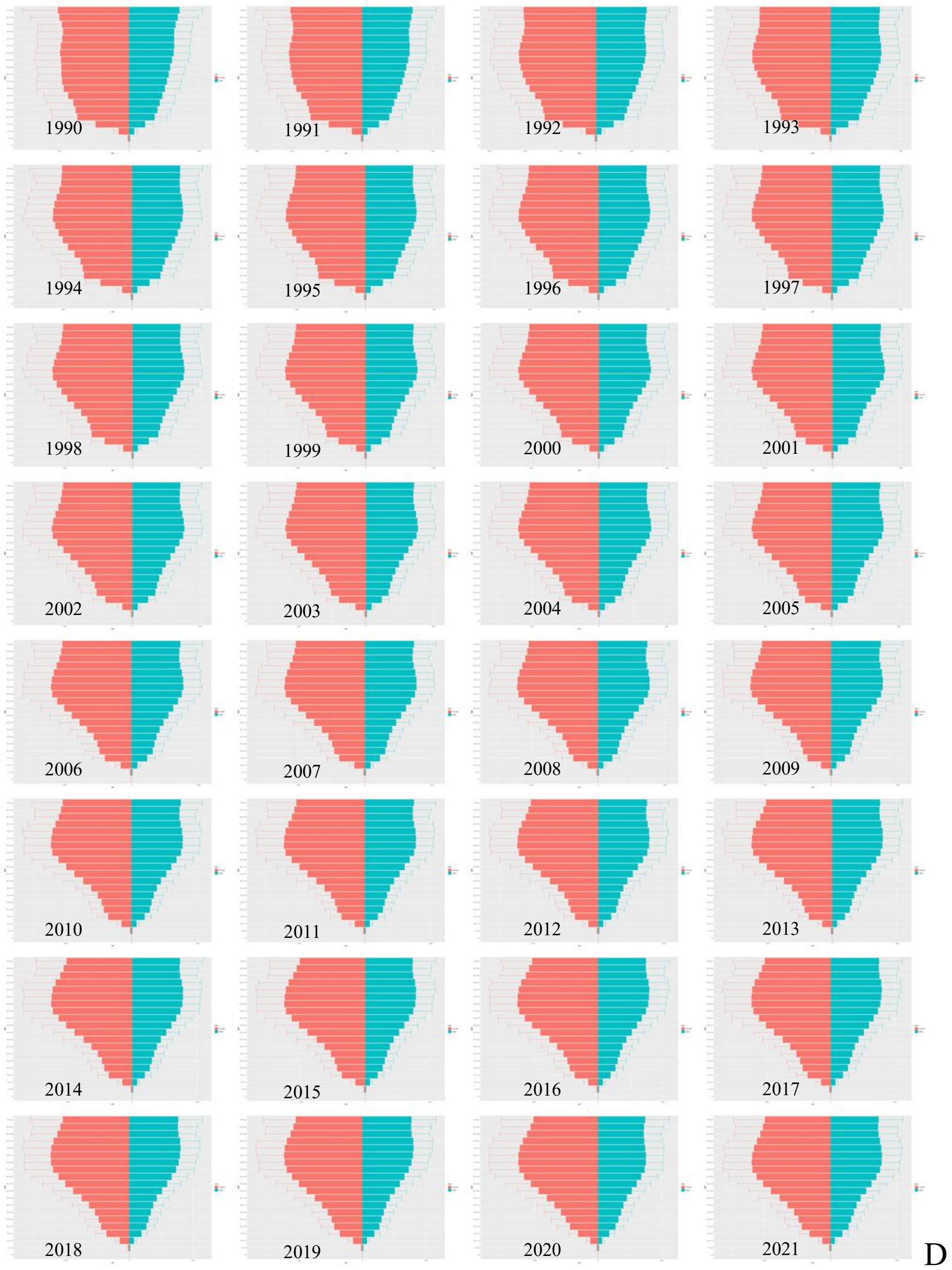

Fig. S80 (A) The prevalence cases of depressive disorders in different ages from 1990 to 2021 in China; (B) The prevalence rates of depressive disorders in different ages from 1990 to 2021 in China; (C) The years lived with disability of depressive disorders in different ages from 1990 to 2021 in China; (D) The years lived with disability rates of depressive disorders in different ages from 1990 to 2021 in China.

Notes: red for female, green for male; the ordinate from bottom to top is "<5", "5 to 9", "10 to 14", "15 to 19", "20 to 24", "25 to 29", "30 to 34", "35 to 39", "40 to 44", "45 to 49", "50 to 54", "55 to 59", "60 to 64", "65 to 69", "70 to 74", "75 to 79", "80 to 84", "85 to 89", "90 to 94", "95 plus".

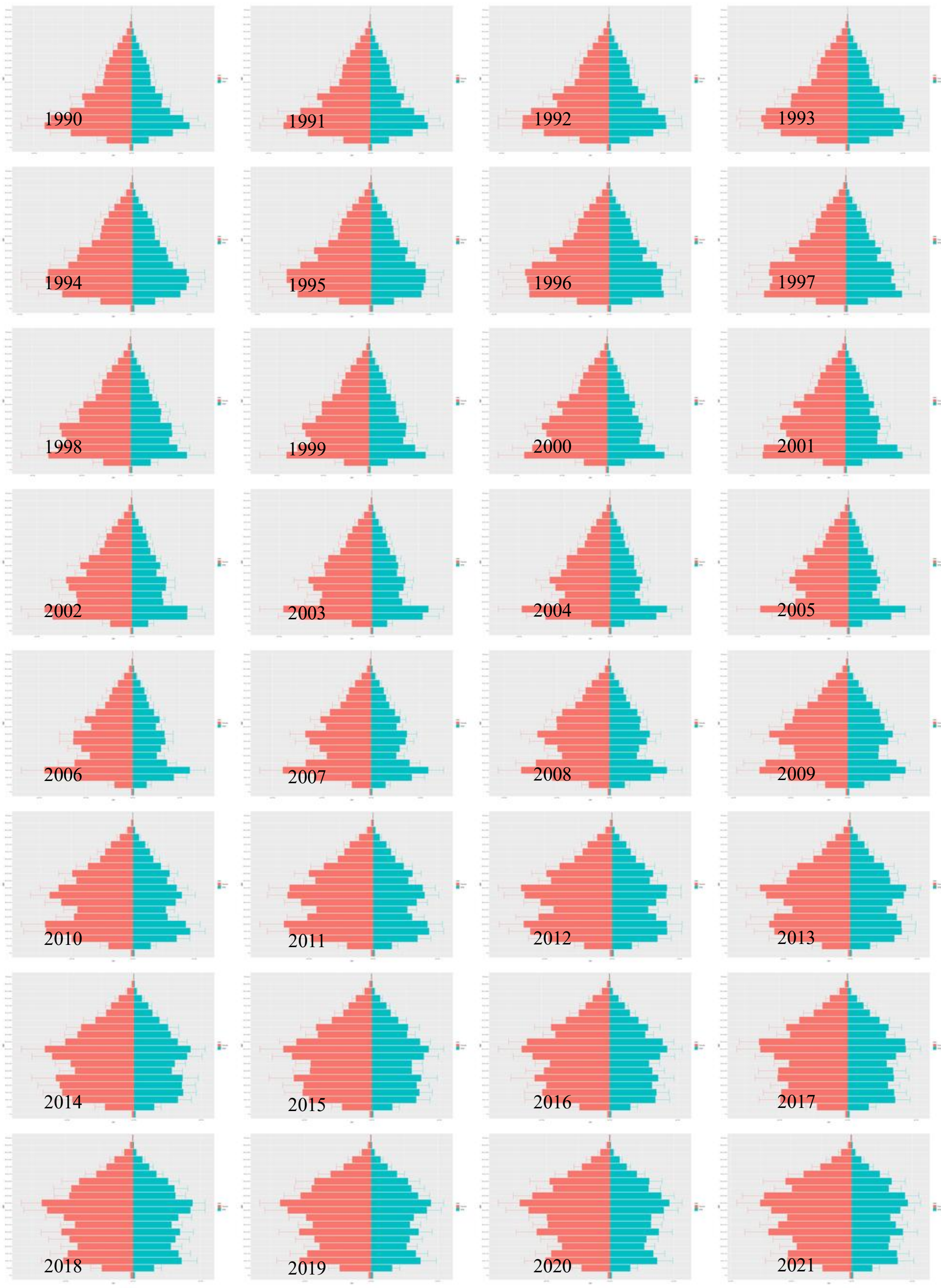
1990
1991
1992
1993
1994
1995
1996
1997
1998
1999
2000
2001
2002
2003
2004
2005
2006
2007
2008
2009
2010
2011
2012
2013
2014
2015
2016
2017
2018
2019
2020
2021

A

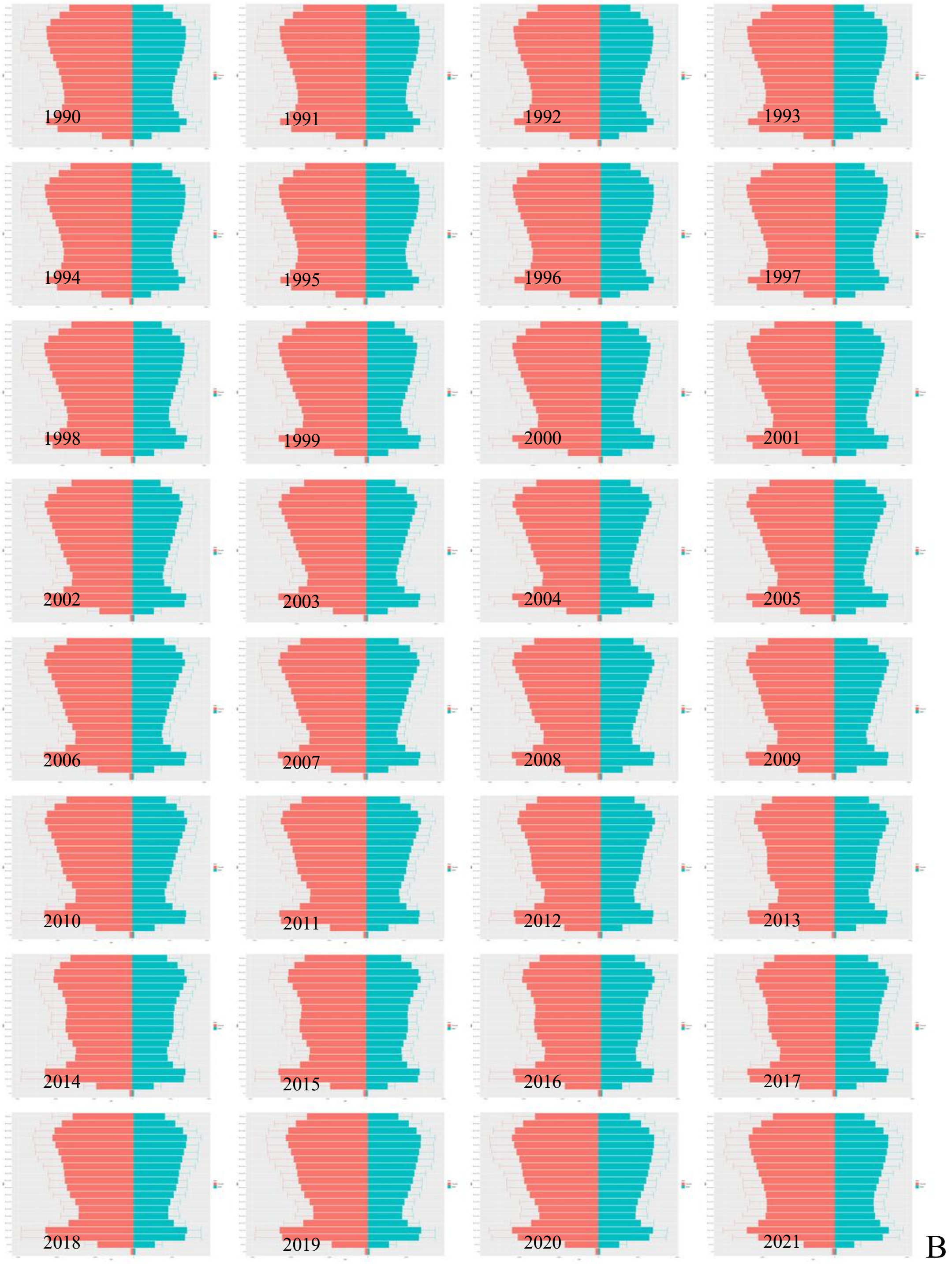
1990
1991
1992
1993
1994
1995
1996
1997
1998
1999
2000
2001
2002
2003
2004
2005
2006
2007
2008
2009
2010
2011
2012
2013
2014
2015
2016
2017
2018
2019
2020
2021
B

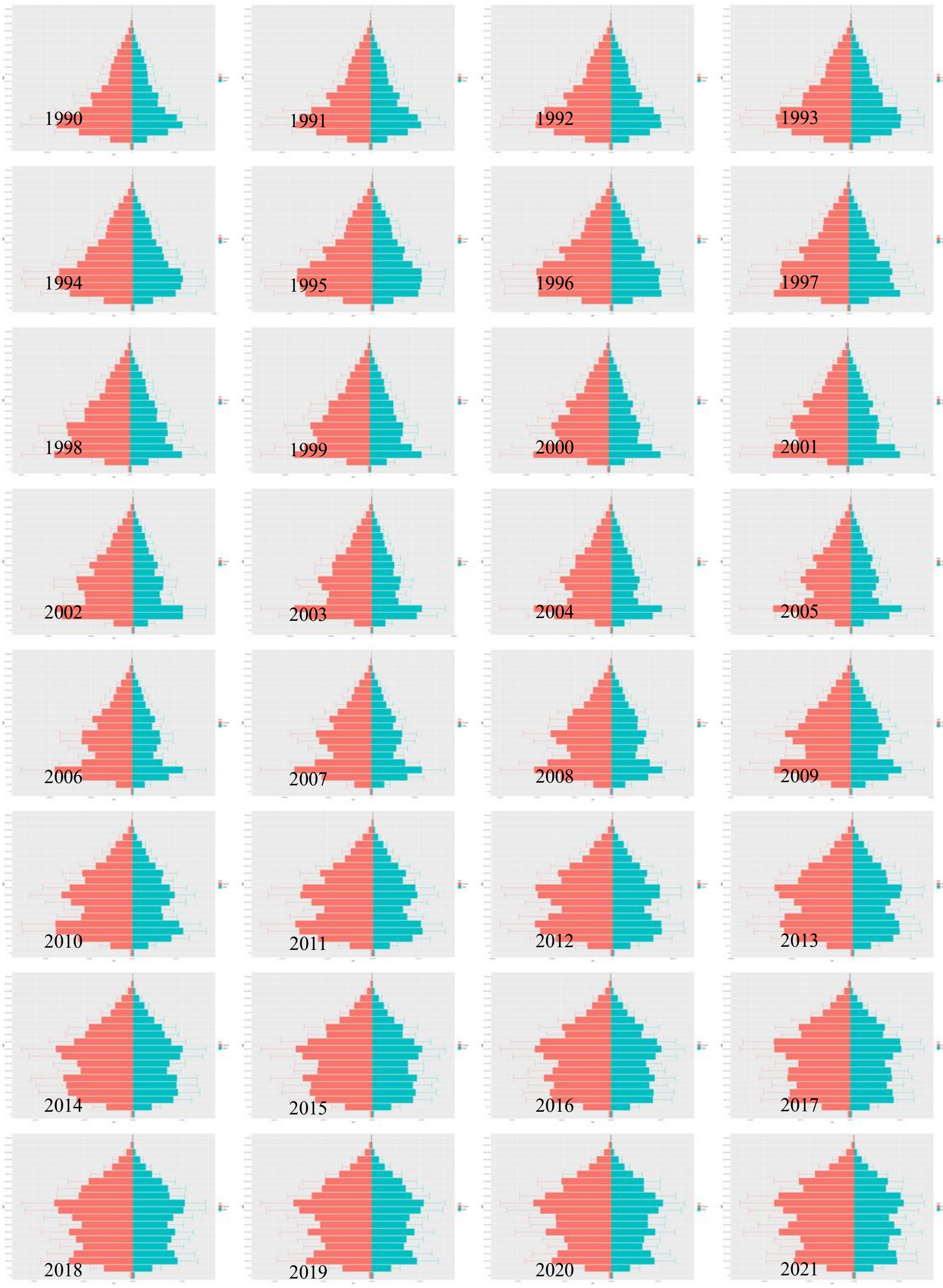
1990
1991
1992
1993
1994
1995
1996
1997
1998
1999
2000
2001
2002
2003
2004
2005
2006
2007
2008
2009
2010
2011
2012
2013
2014
2015
2016
2017
2018
2019
2020
2021

C

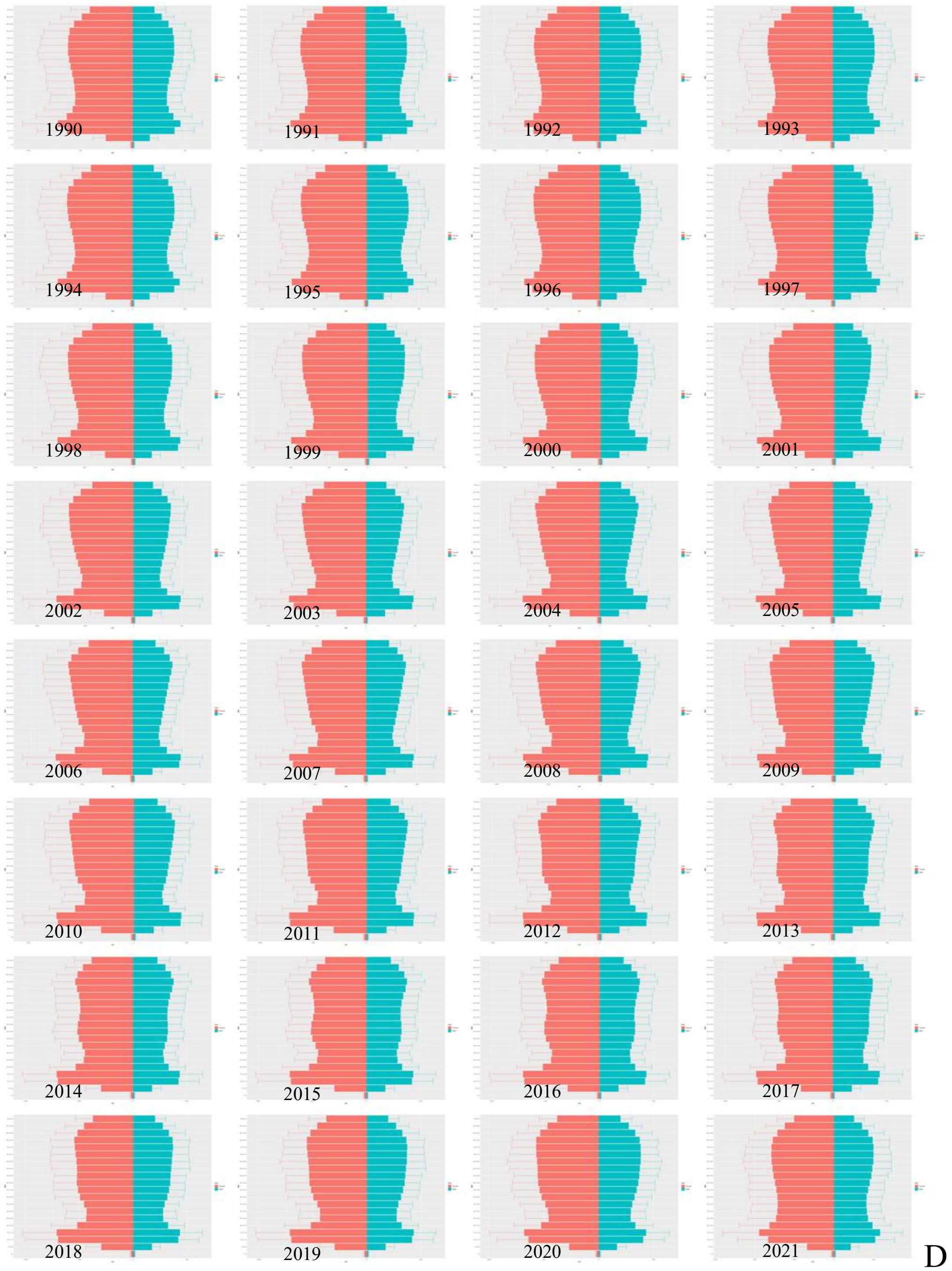

Fig. S81 (A) The prevalence cases of anxiety disorders in different ages from 1990 to 2021 in China; (B) The prevalence rates of anxiety disorders in different ages from 1990 to 2021 in China; (C) The years lived with disability of anxiety disorders in different ages from 1990 to 2021 in China; (D) The years lived with disability rates of anxiety disorders in different ages from 1990 to 2021 in China.

Notes: red for female, green for male; the ordinate from bottom to top is "<5", "5 to 9", "10 to 14", "15 to 19", "20 to 24", "25 to 29", "30 to 34", "35 to 39", "40 to 44", "45 to 49", "50 to 54", "55 to 59", "60 to 64", "65 to 69", "70 to 74", "75 to 79", "80 to 84", "85 to 89", "90 to 94", "95 plus".

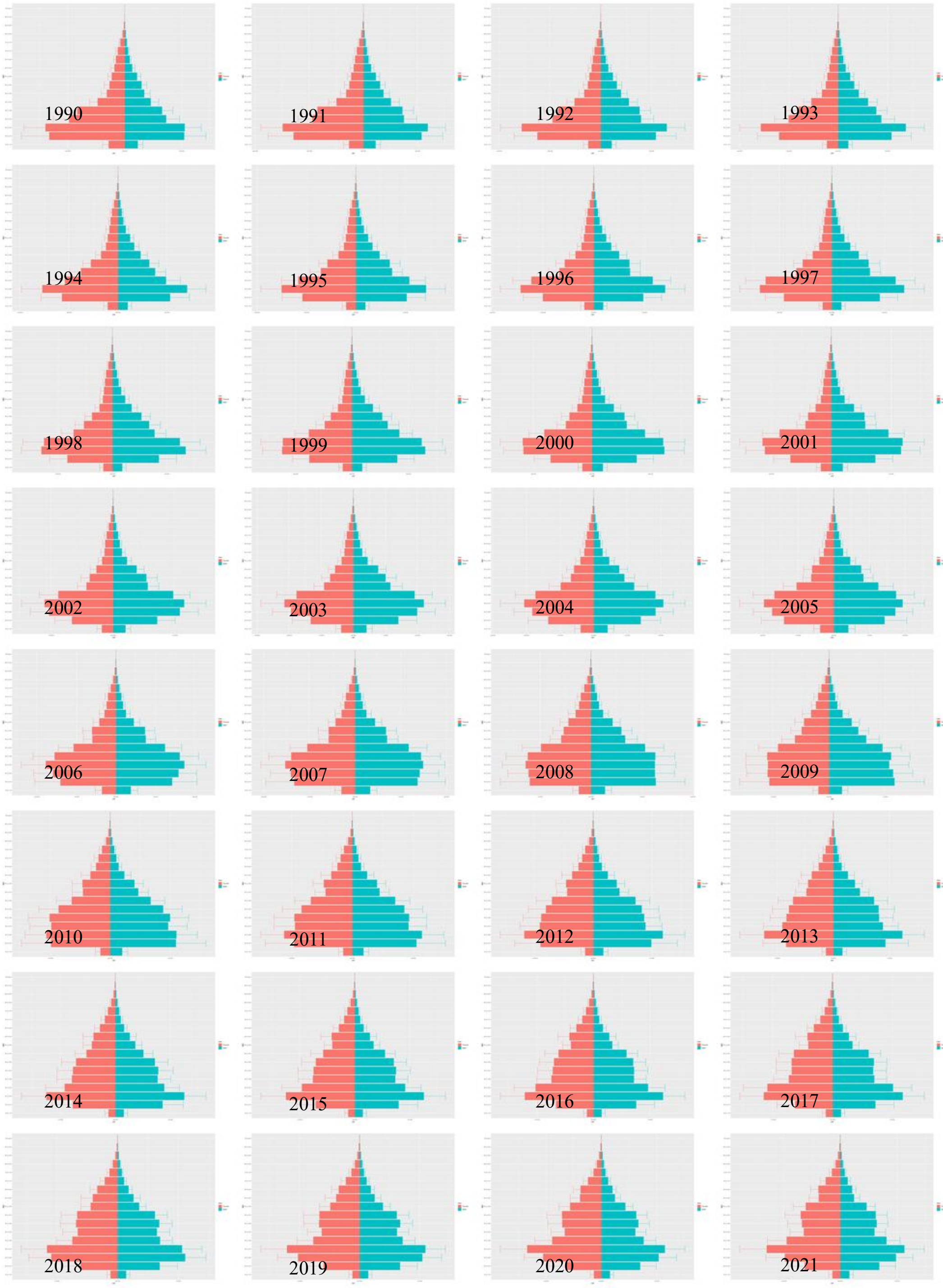
1990
1991
1992
1993
1994
1995
1996
1997
1998
1999
2000
2001
2002
2003
2004
2005
2006
2007
2008
2009
2010
2011
2012
2013
2014
2015
2016
2017
2018
2019
2020
2021

A

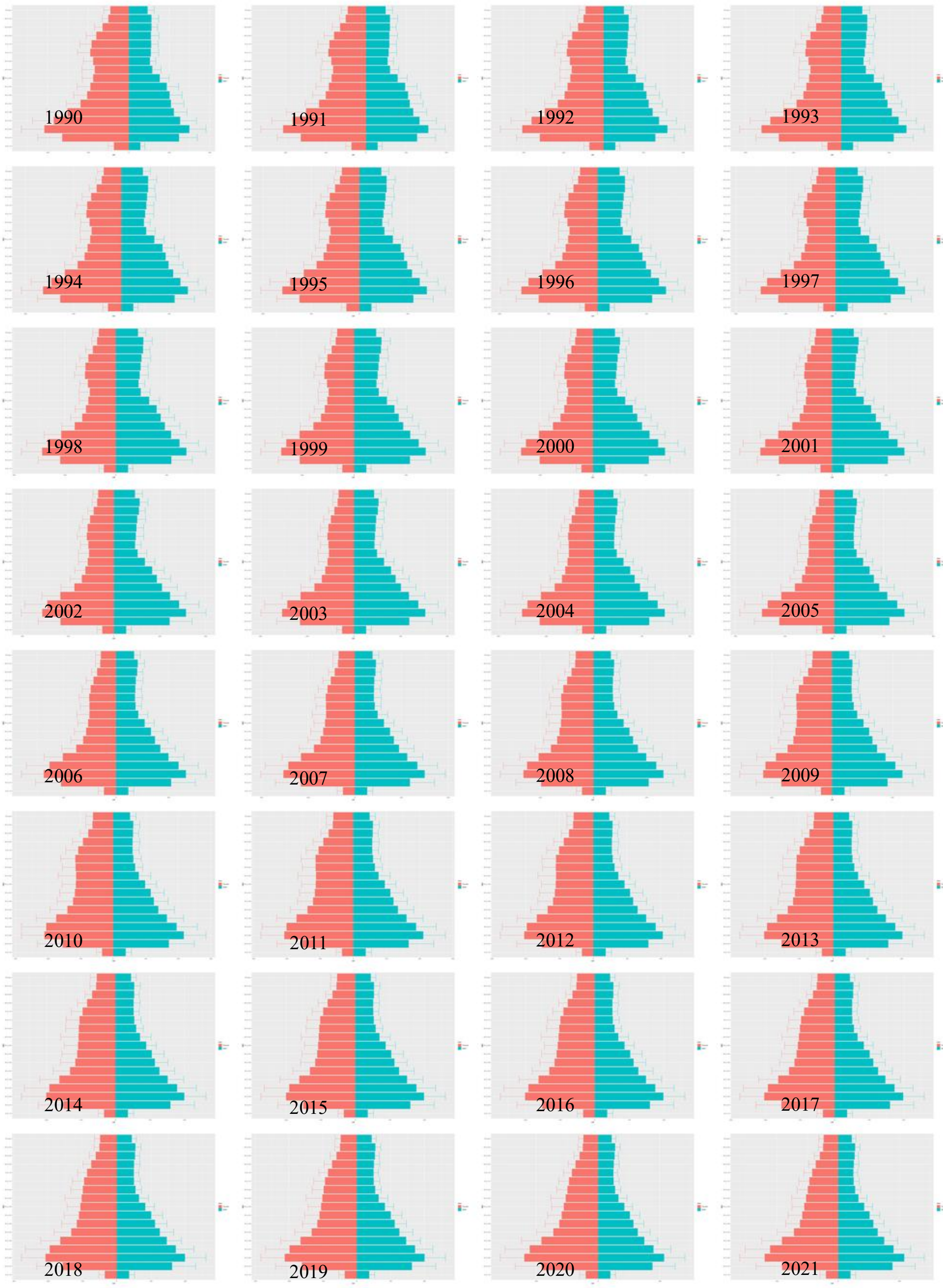
1990
1991
1992
1993
1994
1995
1996
1997
1998
1999
2000
2001
2002
2003
2004
2005
2006
2007
2008
2009
2010
2011
2012
2013
2014
2015
2016
2017
2018
2019
2020
2021

B

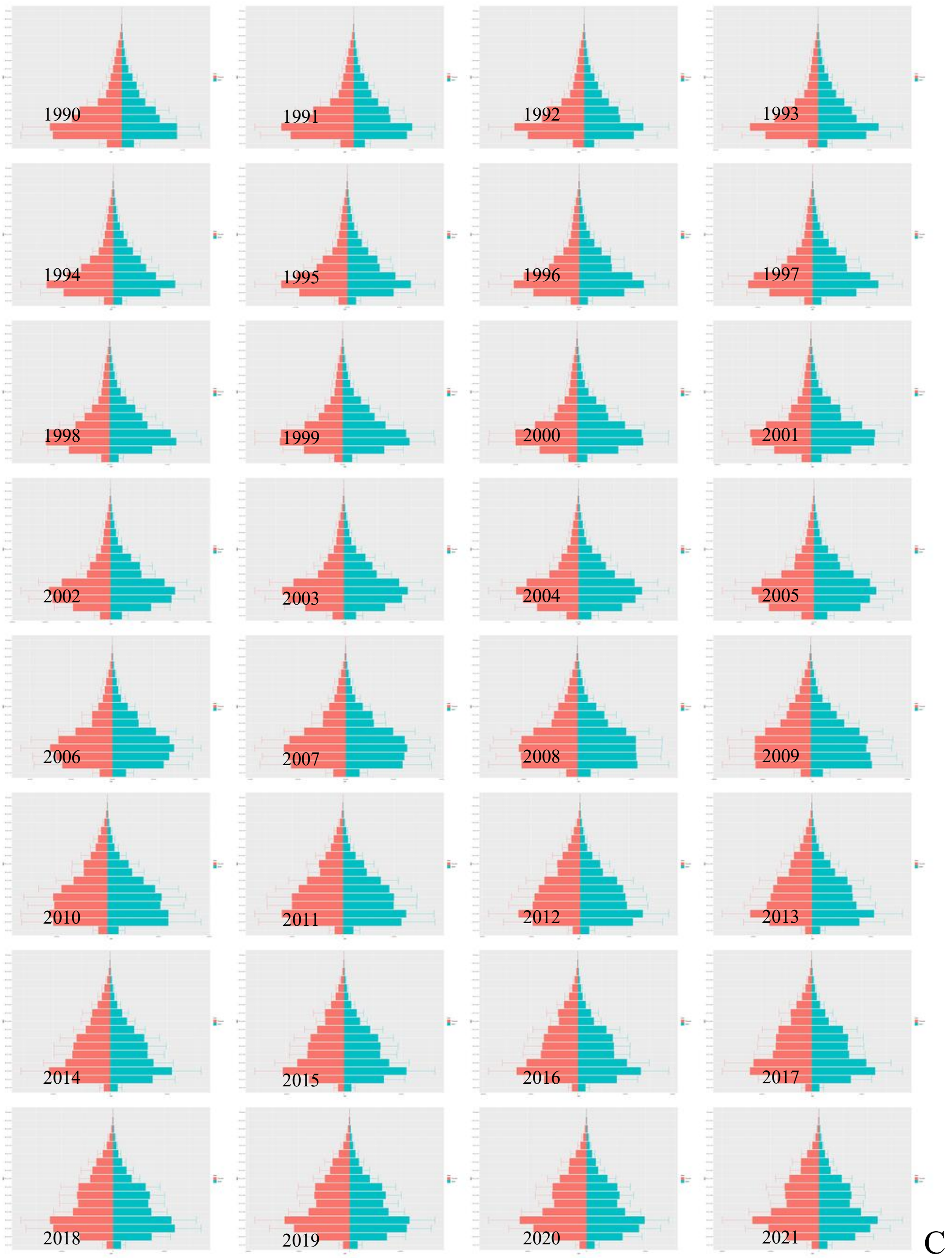
1990
1991
1992
1993
1994
1995
1996
1997
1998
1999
2000
2001
2002
2003
2004
2005
2006
2007
2008
2009
2010
2011
2012
2013
2014
2015
2016
2017
2018
2019
2020
2021
C

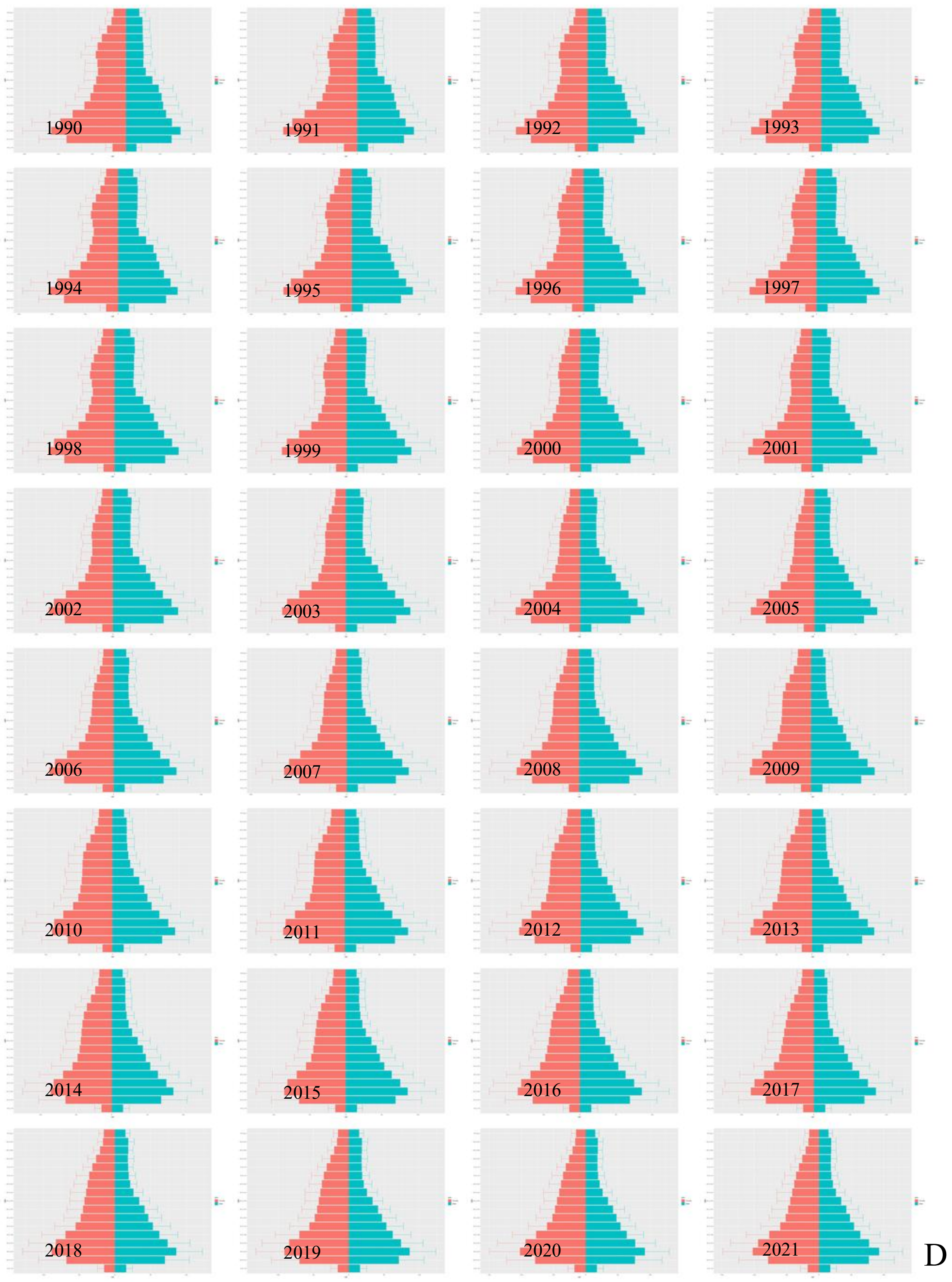

Fig. S82 (A) The prevalence cases of opioid use disorders in different ages from 1990 to 2021 in China; (B) The prevalence rates of opioid use disorders in different ages from 1990 to 2021 in China; (C) The years lived with disability of opioid use disorders in different ages from 1990 to 2021 in China; (D) The years lived with disability rates of opioid use disorders in different ages from 1990 to 2021 in China.

Notes: red for female, green for male; the ordinate from bottom to top is "<5", "5 to 9", "10 to 14", "15 to 19", "20 to 24", "25 to 29", "30 to 34", "35 to 39", "40 to 44", "45 to 49", "50 to 54", "55 to 59", "60 to 64", "65 to 69", "70 to 74", "75 to 79", "80 to 84", "85 to 89", "90 to 94", "95 plus".

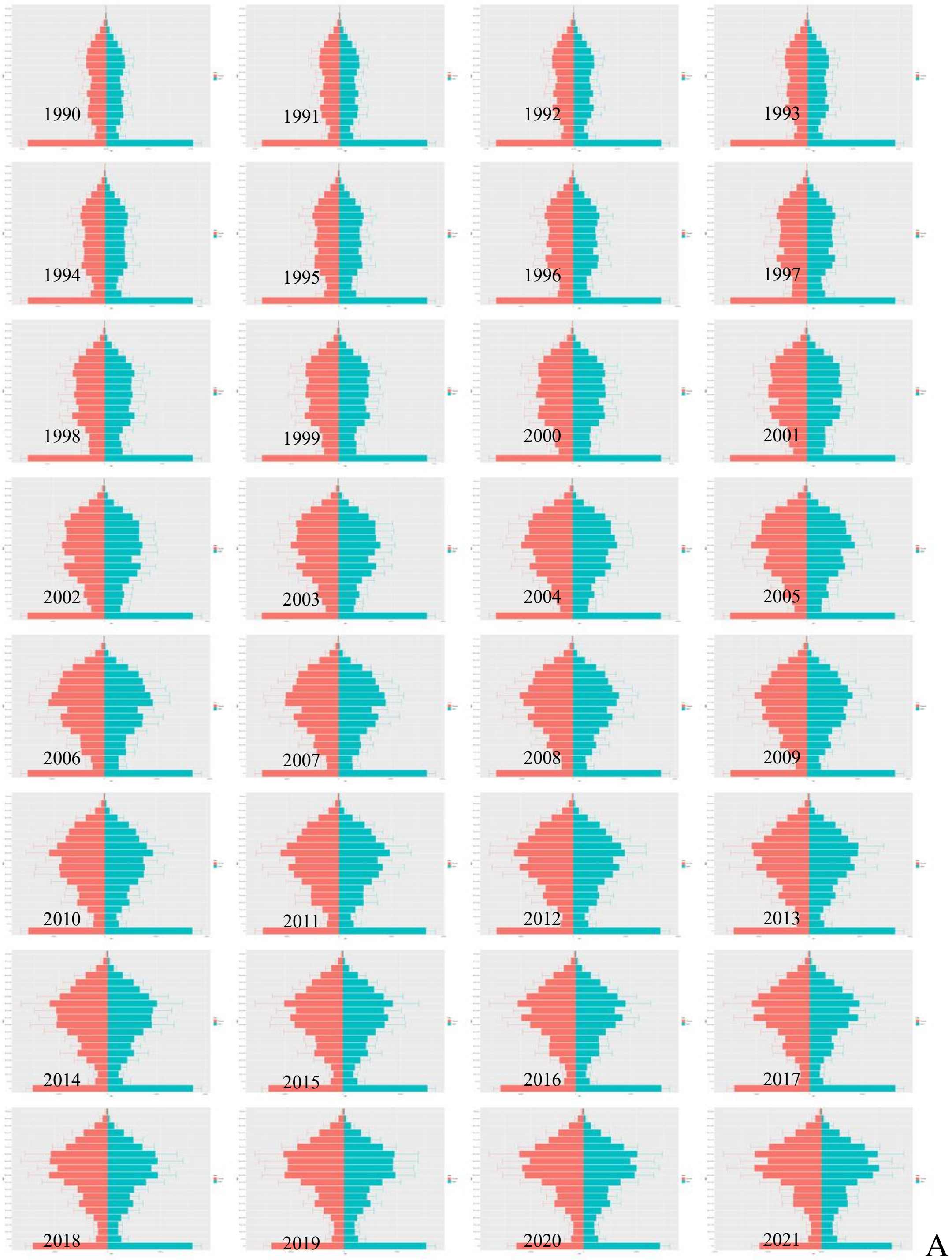
1990
1991
1992
1993
1994
1995
1996
1997
1998
1999
2000
2001
2002
2003
2004
2005
2006
2007
2008
2009
2010
2011
2012
2013
2014
2015
2016
2017
2018
2019
2020
2021
A

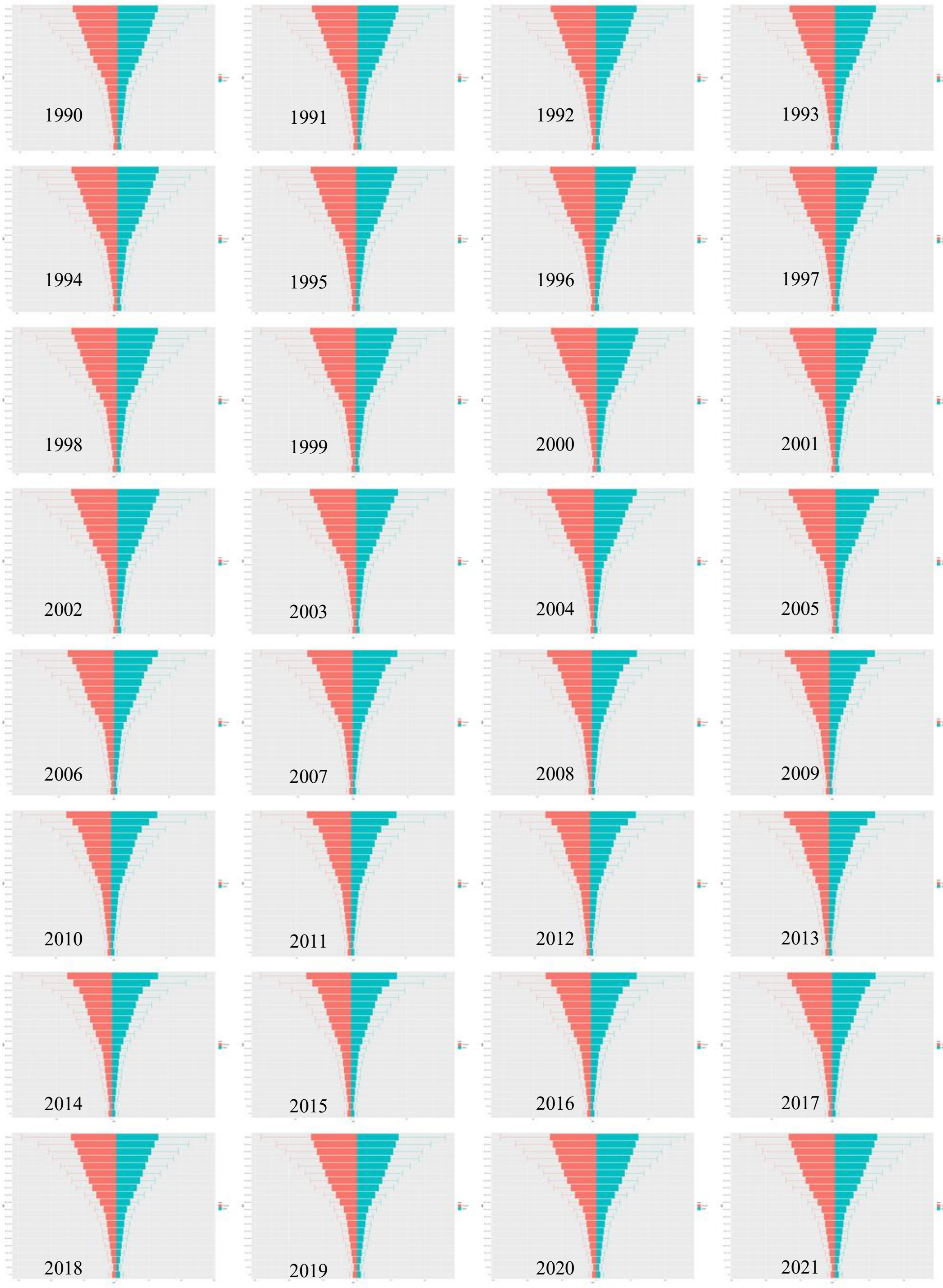
1990
1991
1992
1993
1994
1995
1996
1997
1998
1999
2000
2001
2002
2003
2004
2005
2006
2007
2008
2009
2010
2011
2012
2013
2014
2015
2016
2017
2018
2019
2020
2021
B

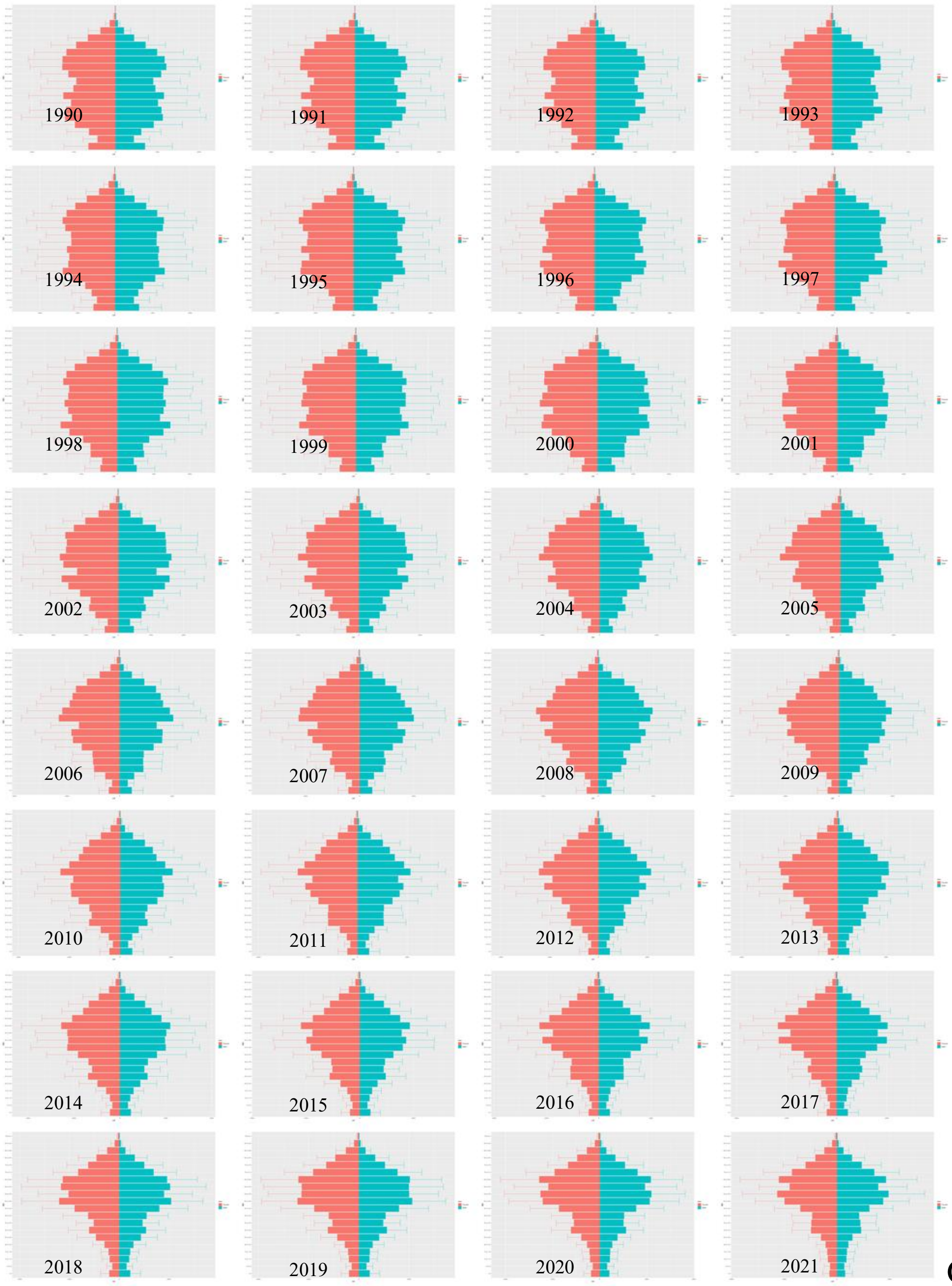
1990
1991
1992
1993
1994
1995
1996
1997
1998
1999
2000
2001
2002
2003
2004
2005
2006
2007
2008
2009
2010
2011
2012
2013
2014
2015
2016
2017
2018
2019
2020
2021

C

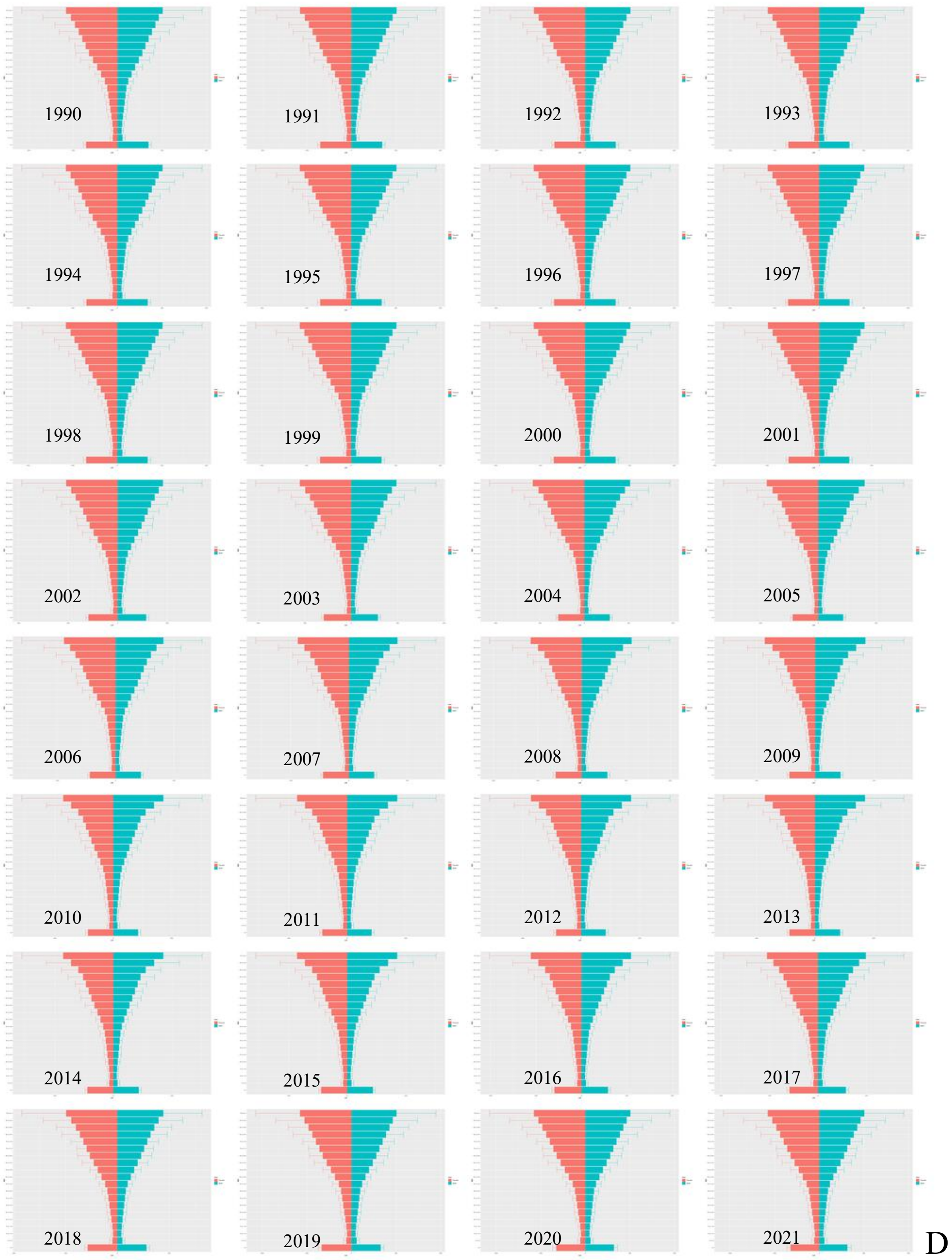

Fig. S83 (A) The prevalence cases of varicella and herpes zoster in different ages from 1990 to 2021 in China; (B) The prevalence rates of varicella and herpes zoster in different ages from 1990 to 2021 in China; (C) The years lived with disability of varicella and herpes zoster in different ages from 1990 to 2021 in China; (D) The years lived with disability rates of varicella and herpes zoster in different ages from 1990 to 2021 in China.

Notes: red for female, green for male; the ordinate from bottom to top is "<5", "5 to 9", "10 to 14", "15 to 19", "20 to 24", "25 to 29", "30 to 34", "35 to 39", "40 to 44", "45 to 49", "50 to 54", "55 to 59", "60 to 64", "65 to 69", "70 to 74", "75 to 79", "80 to 84", "85 to 89", "90 to 94", "95 plus".

A

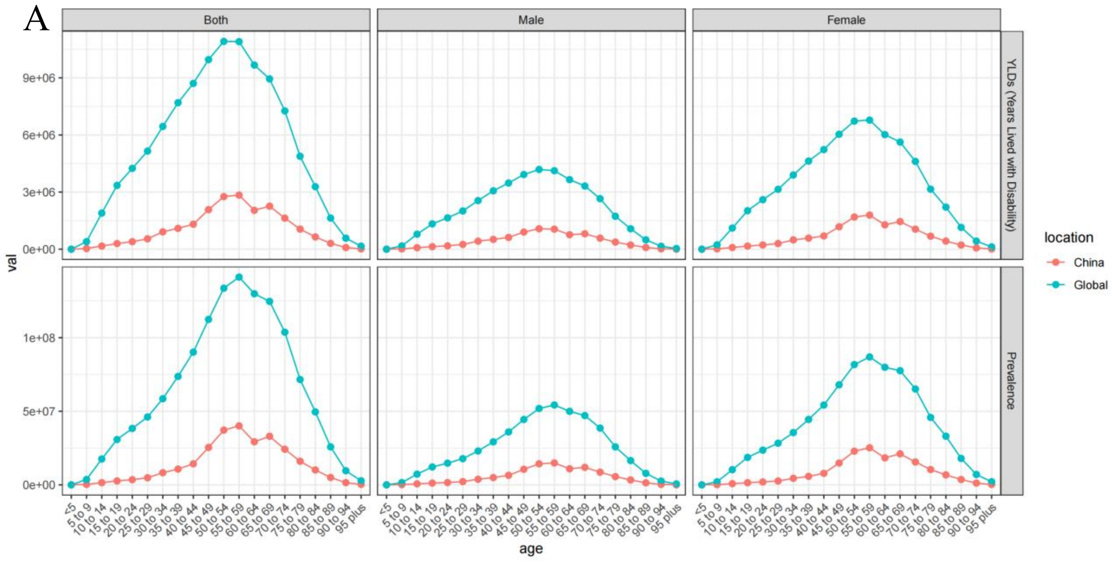

B

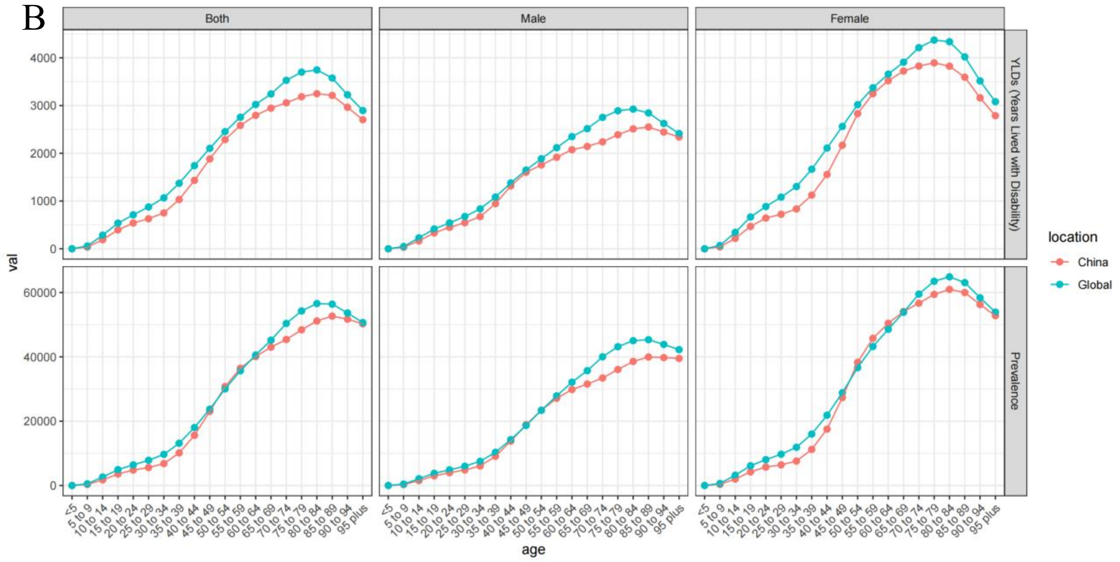

Fig. S84 (A) The number of prevalent cases and years lived with disability of musculoskeletal disorders in different ages in 2021; (B) The age-standardized rates of prevalent cases and years lived with disability of musculoskeletal disorders in different ages in 2021.

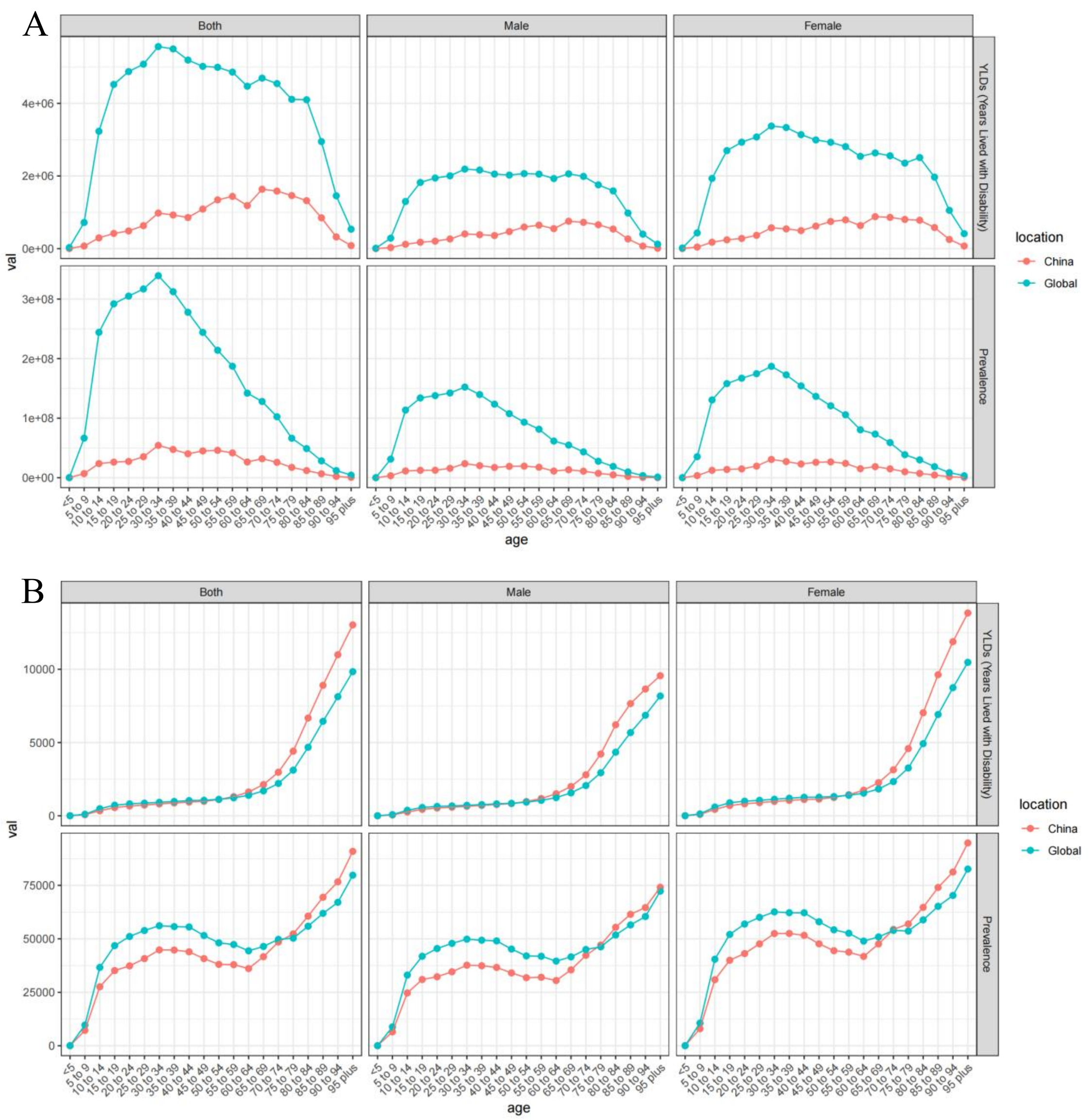

Fig. S85 (A) The number of prevalent cases and years lived with disability of neurological disorders in different ages in 2021; (B) The age-standardized rates of prevalent cases and years lived with disability of neurological disorders in different ages in 2021.

A

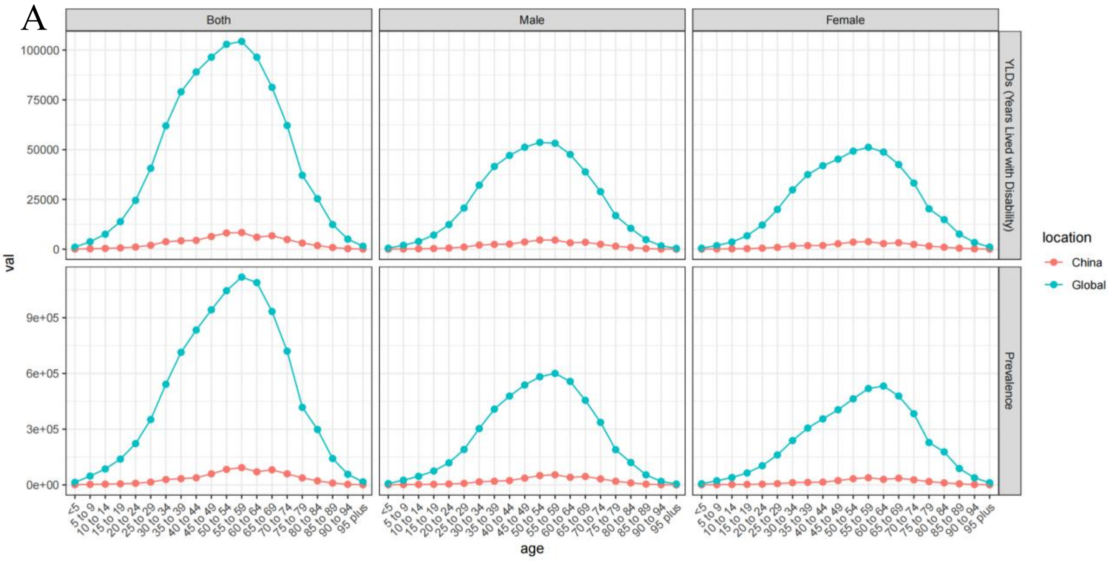

B

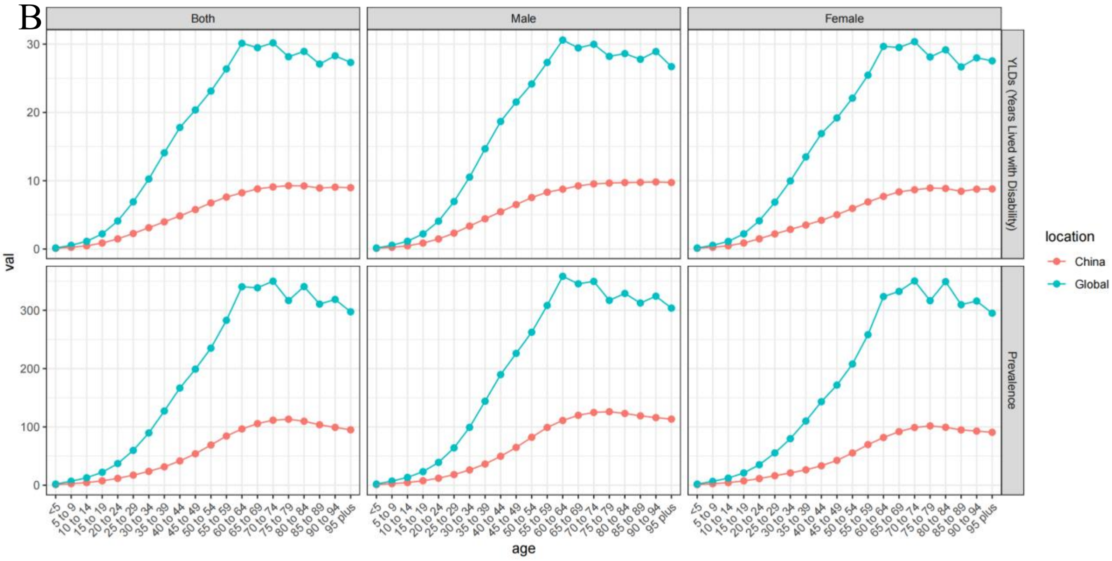

Fig. S86 (A) The number of prevalent cases and years lived with disability of digestive disorders in different ages in 2021; (B) The age-standardized rates of prevalent cases and years lived with disability of digestive disorders in different ages in 2021.

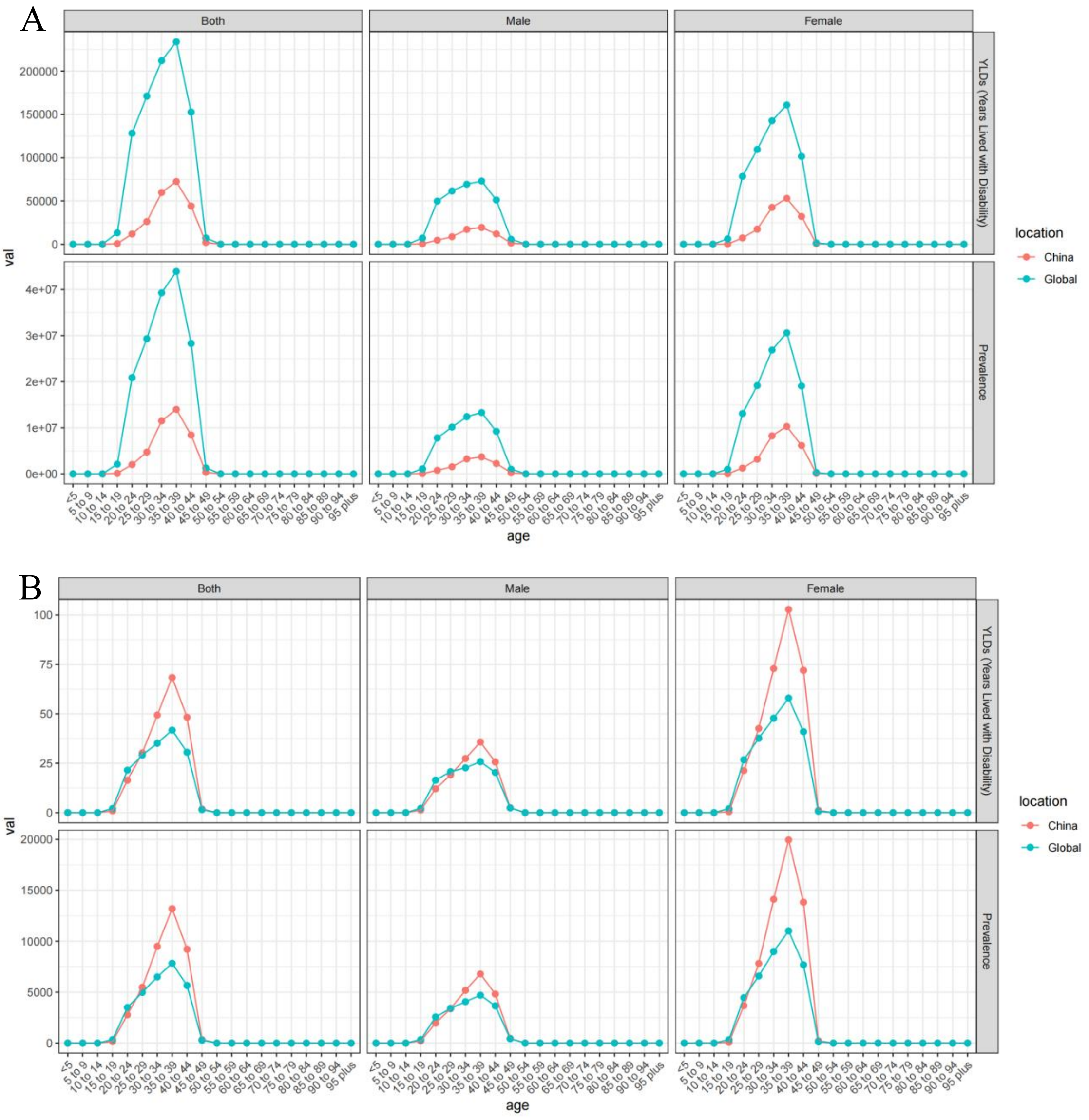

Fig. S87 (A) The number of prevalent cases and years lived with disability of genecological disorders in different ages in 2021; (B) The age-standardized rates of prevalent cases and years lived with disability of genecological disorders in different ages in 2021.

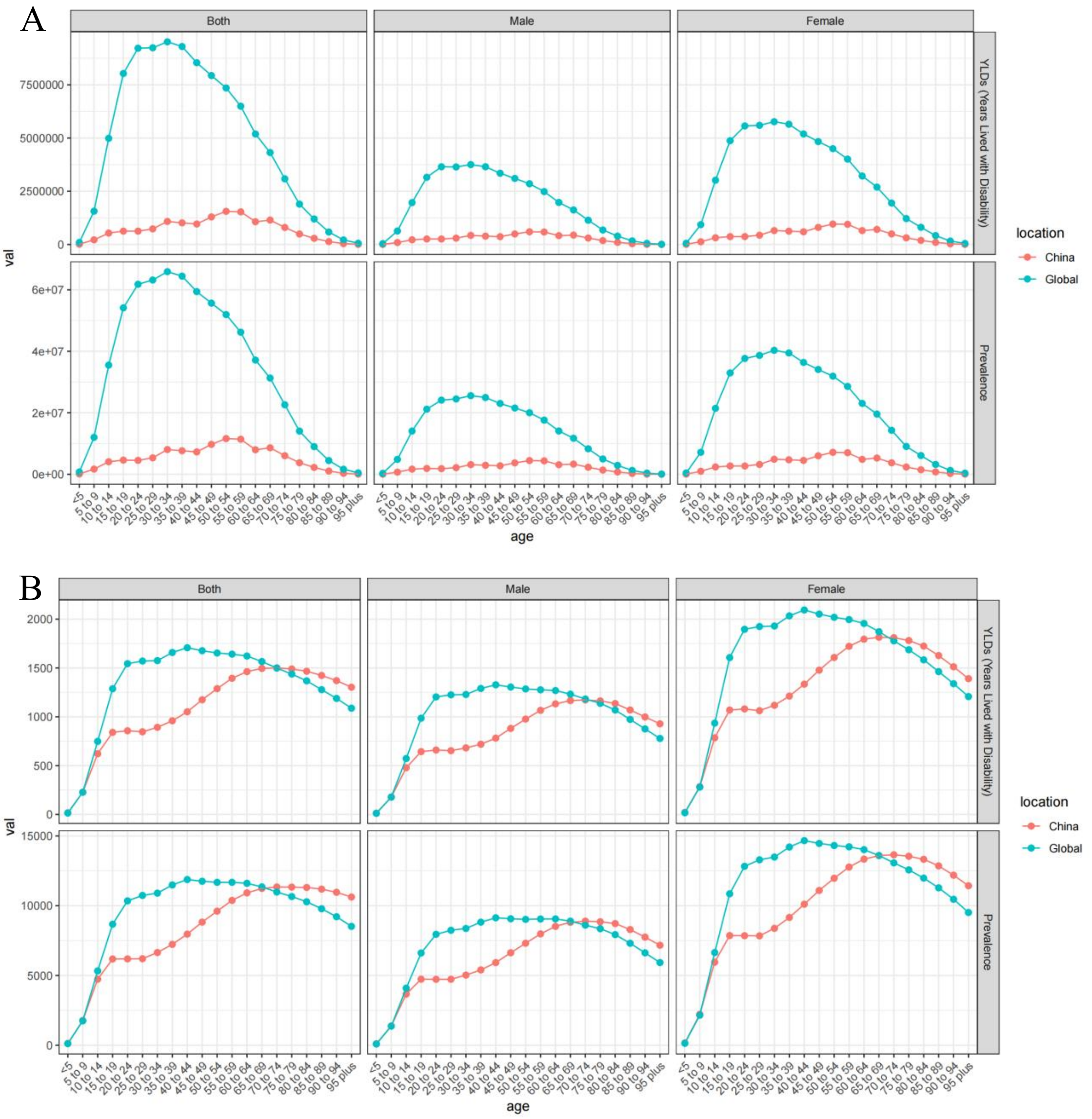

Fig. S88 (A) The number of prevalent cases and years lived with disability of mental disorders in different ages in 2021; (B) The age-standardized rates of prevalent cases and years lived with disability of mental disorders in different ages in 2021.

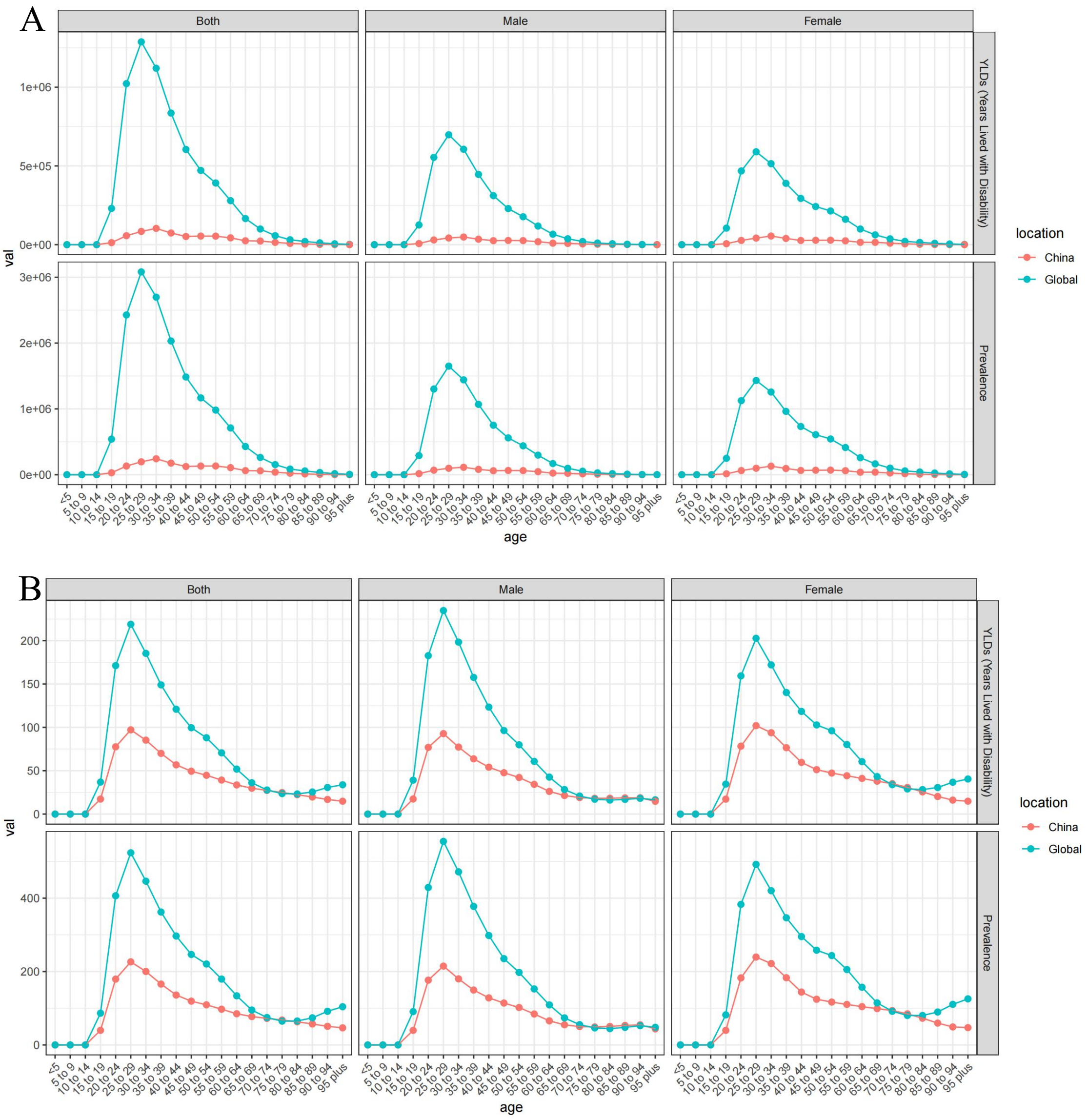

Fig. S89 (A) The number of prevalent cases and years lived with disability of substance use disorders in different ages in 2021; (B) The age-standardized rates of prevalent cases and years lived with disability of substance use disorders in different ages in 2021.

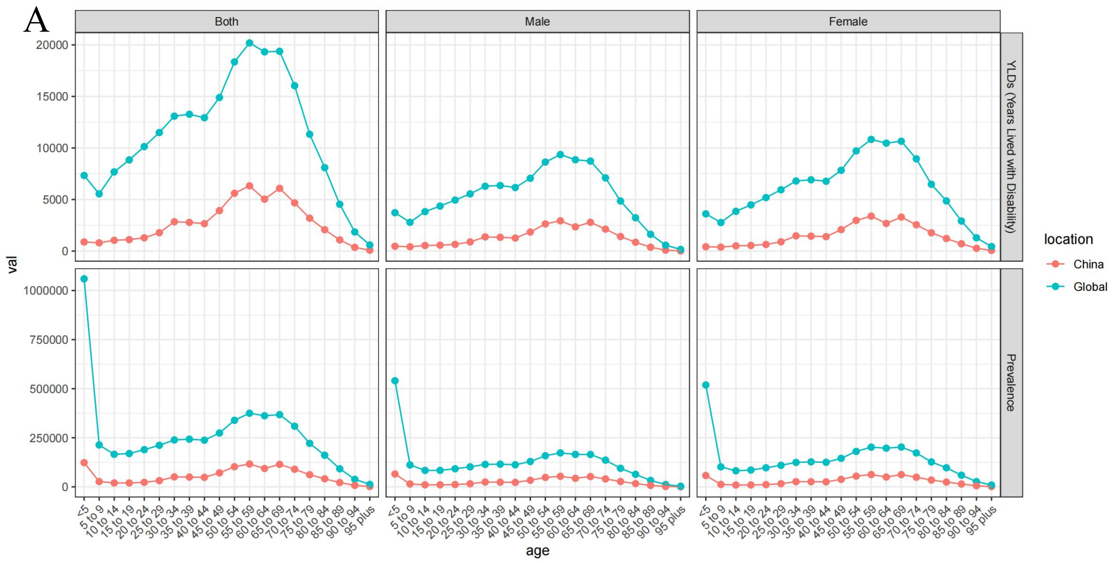

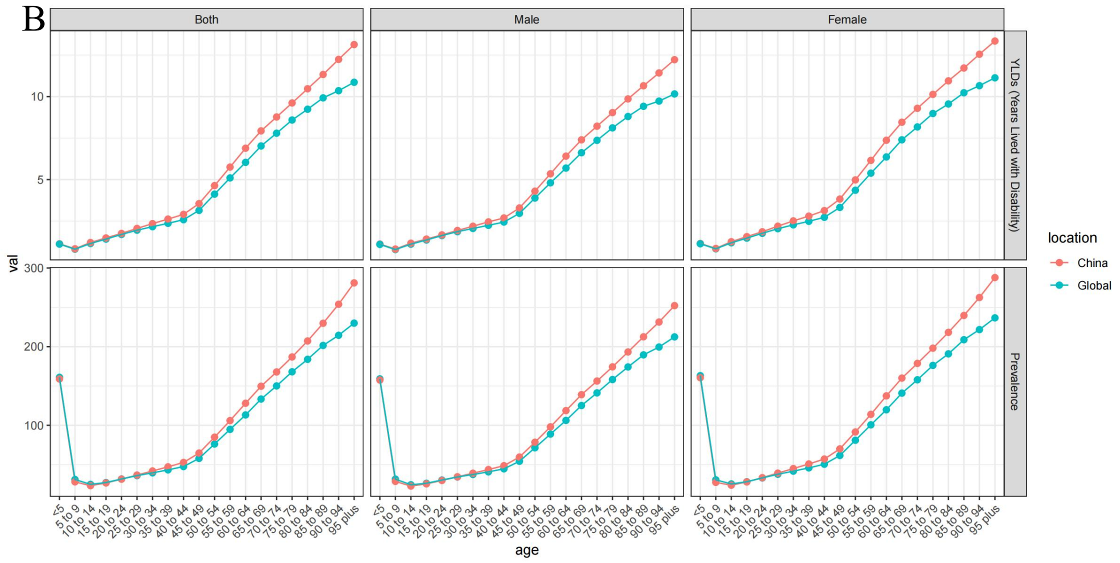

Fig. S90 (A) The number of prevalent cases and years lived with disability of infectious disorders in different ages in 2021; (B) The age-standardized rates of prevalent cases and years lived with disability of infectious disorders in different ages in 2021.

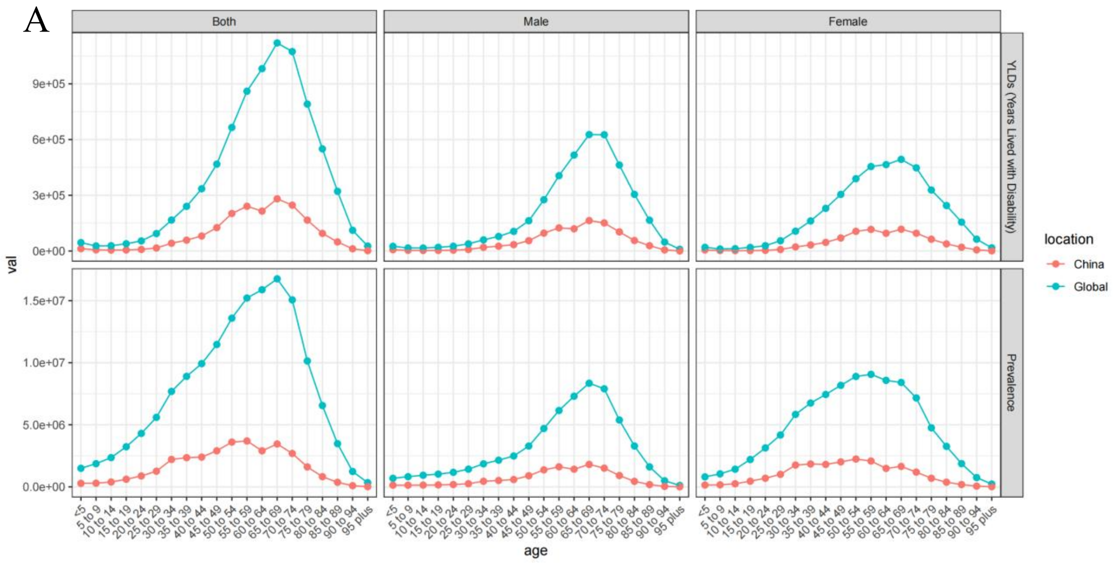

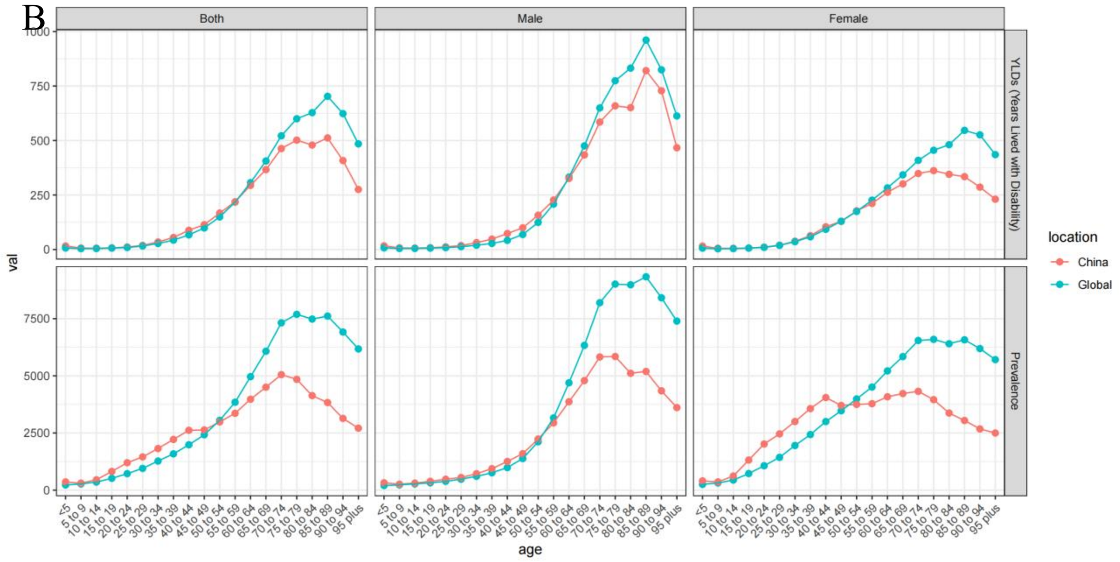

Fig. S91 (A) The number of prevalent cases and years lived with disability of neoplasms in different ages in 2021; (B) The age-standardized rates of prevalent cases and years lived with disability of neoplasms in different ages in 2021.

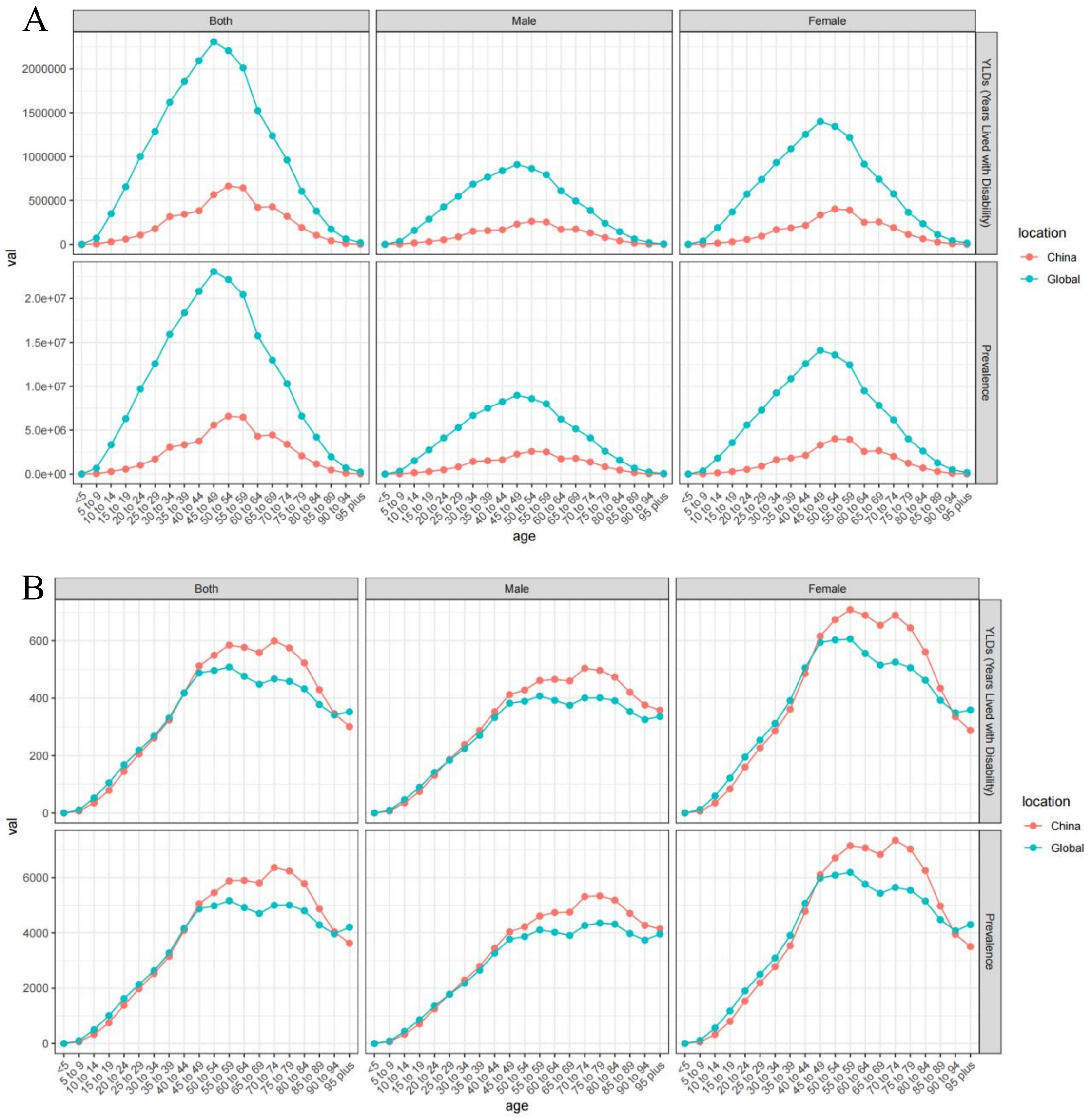

Fig. S92 (A) The number of prevalent cases and years lived with disability of neck pain in different ages in 2021; (B) The age-standardized rates of prevalent cases and years lived with disability of neck pain in different ages in 2021.

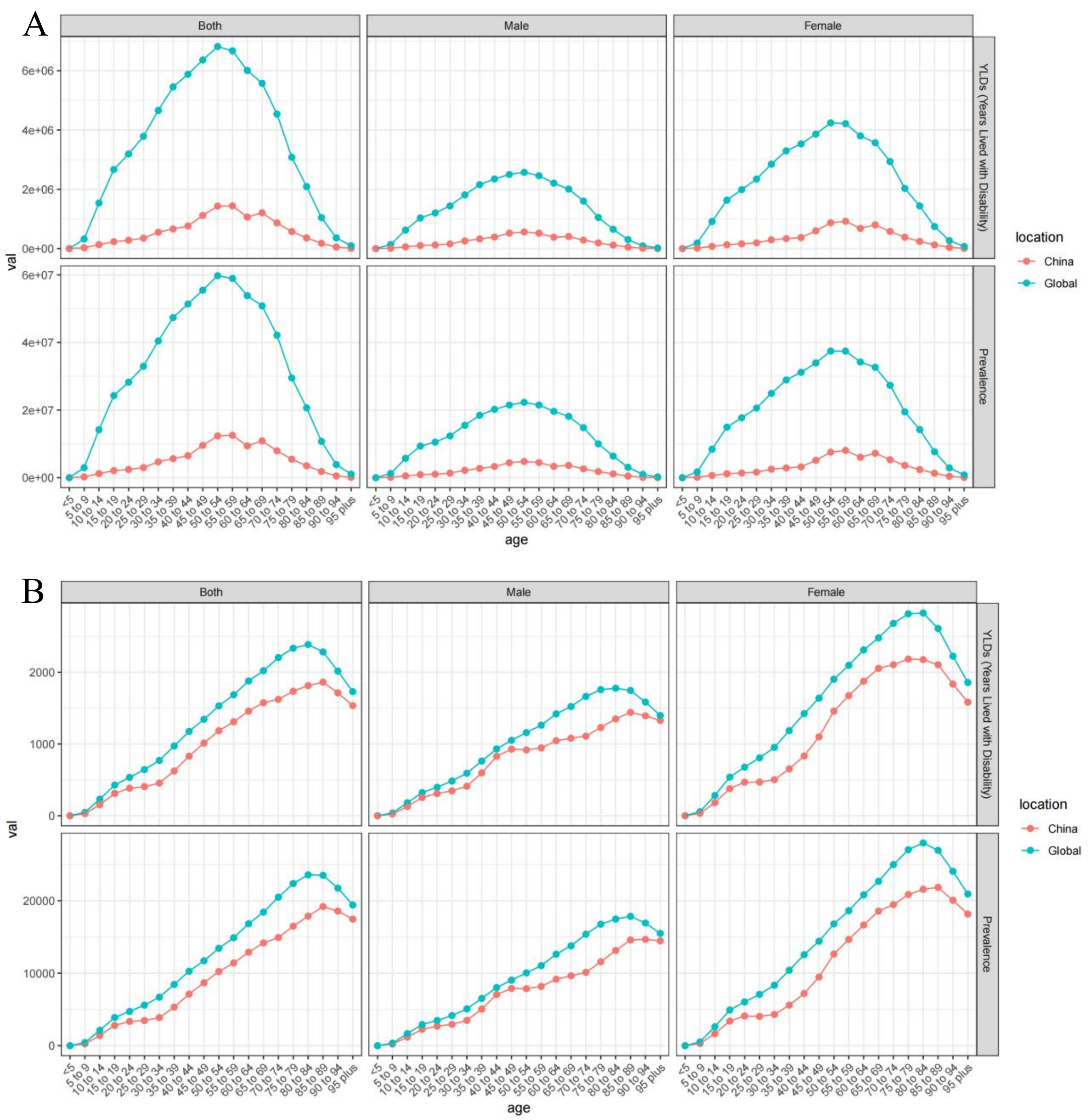

Fig. S93 (A) The number of prevalent cases and years lived with disability of low back pain in different ages in 2021; (B) The age-standardized rates of prevalent cases and years lived with disability of low back pain in different ages in 2021.

A

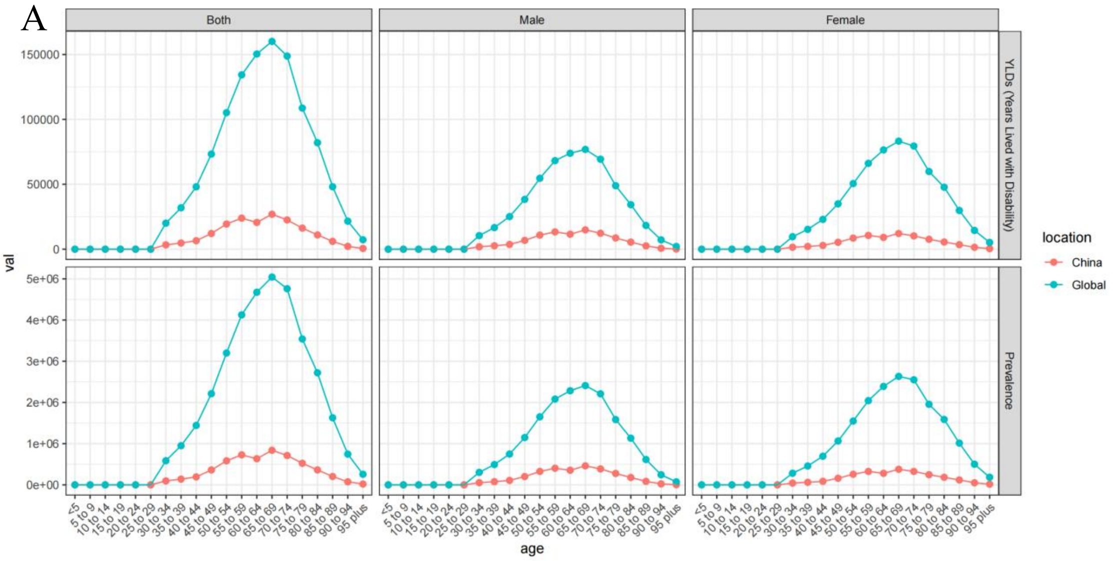

B

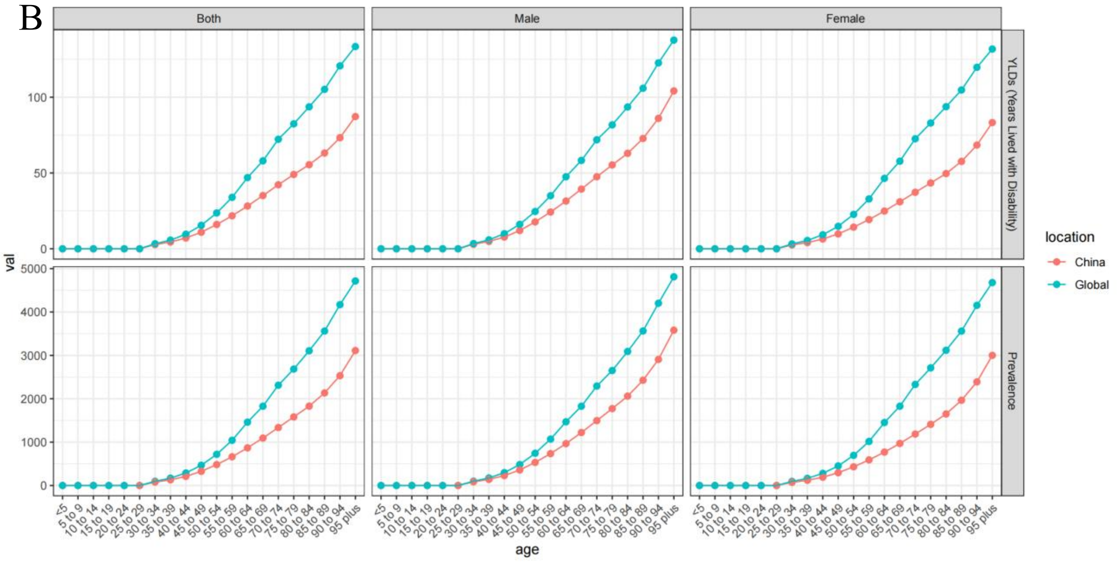

Fig. S94 (A) The number of prevalent cases and years lived with disability of hip osteoarthritis in different ages in 2021; (B) The age-standardized rates of prevalent cases and years lived with disability of hip osteoarthritis in different ages in 2021.

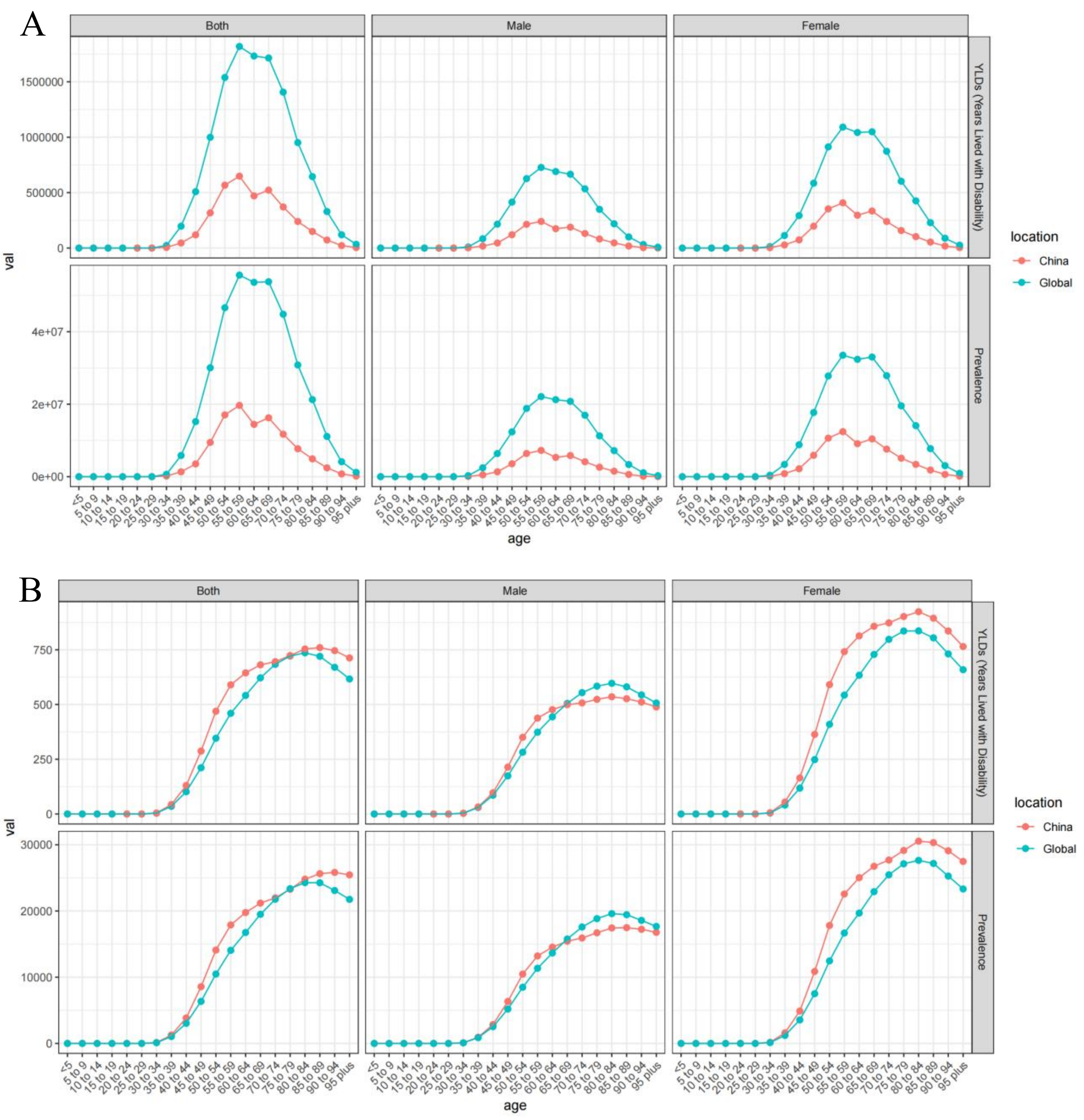

Fig. S95 (A) The number of prevalent cases and years lived with disability of knee osteoarthritis in different ages in 2021; (B) The age-standardized rates of prevalent cases and years lived with disability of knee osteoarthritis in different ages in 2021.

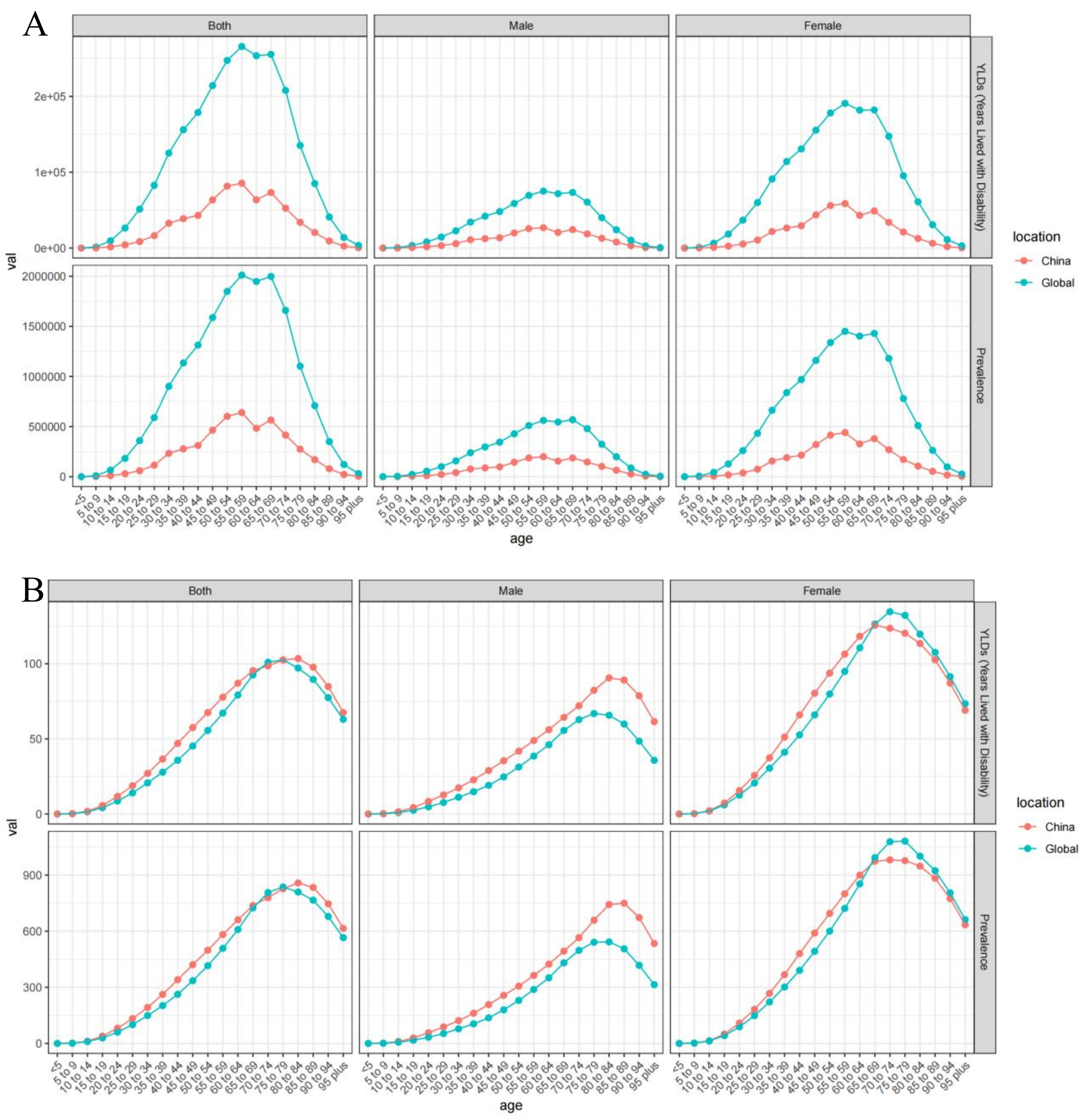

Fig. S96 (A) The number of prevalent cases and years lived with disability of rheumatoid arthritis in different ages in 2021; (B) The age-standardized rates of prevalent cases and years lived with disability of rheumatoid arthritis in different ages in 2021.

A

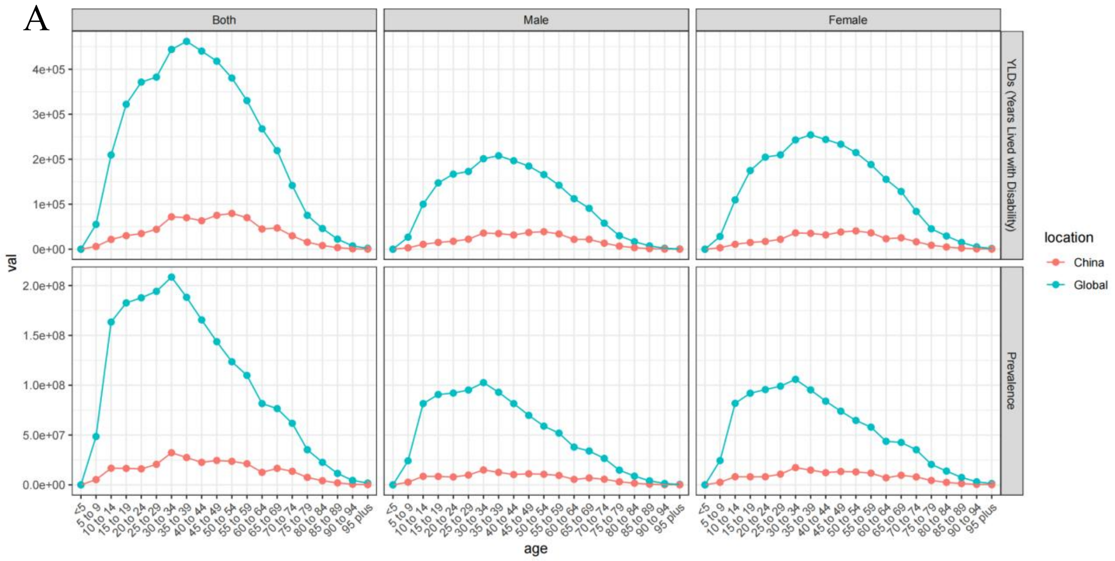

B

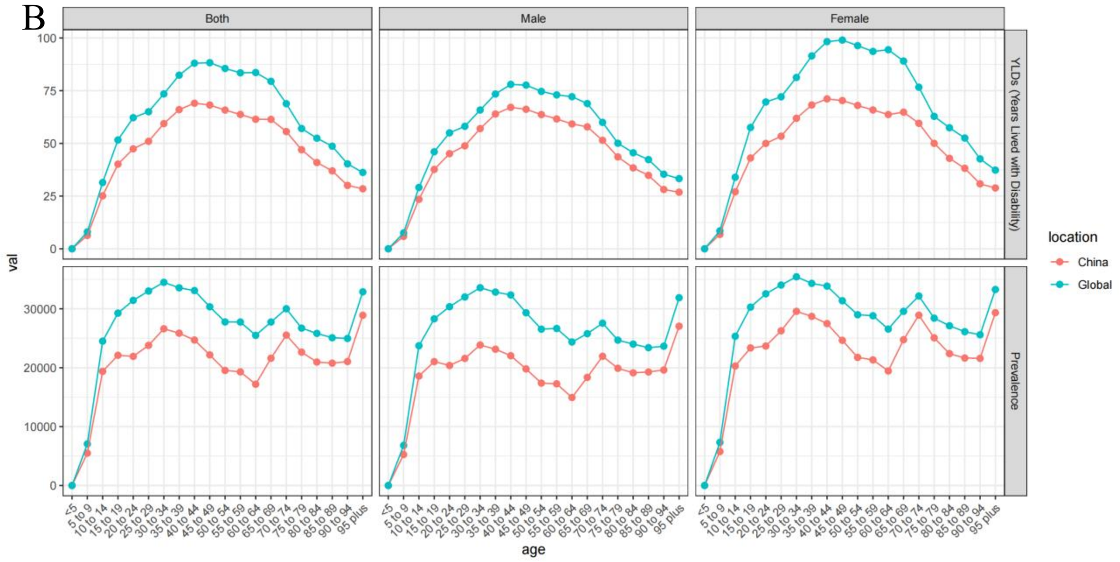

Fig. S97 (A) The number of prevalent cases and years lived with disability of tension-type headache in different ages in 2021; (B) The age-standardized rates of prevalent cases and years lived with disability of tension-type headache in different ages in 2021.

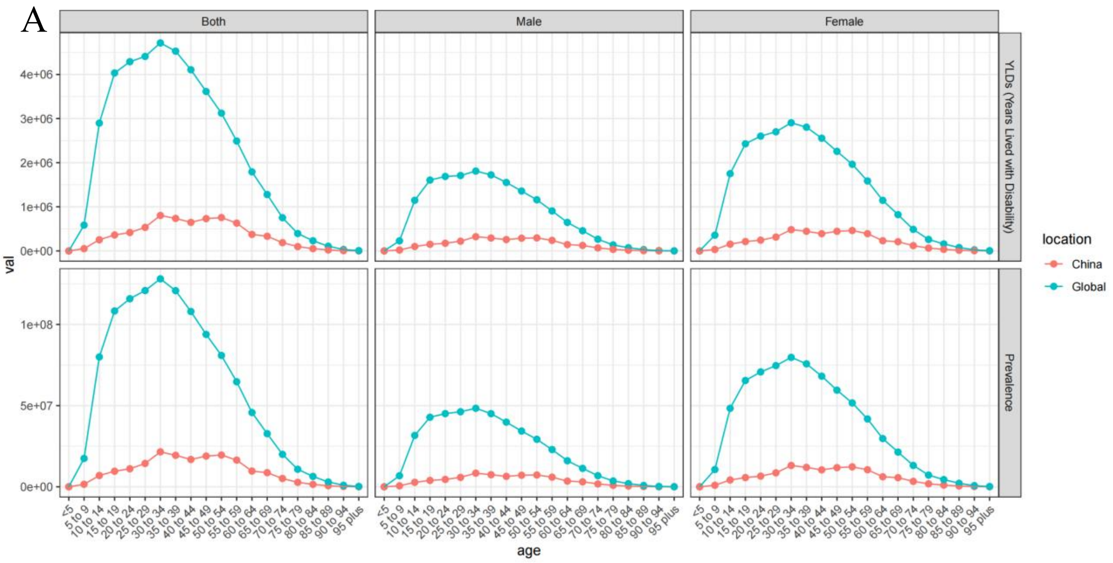

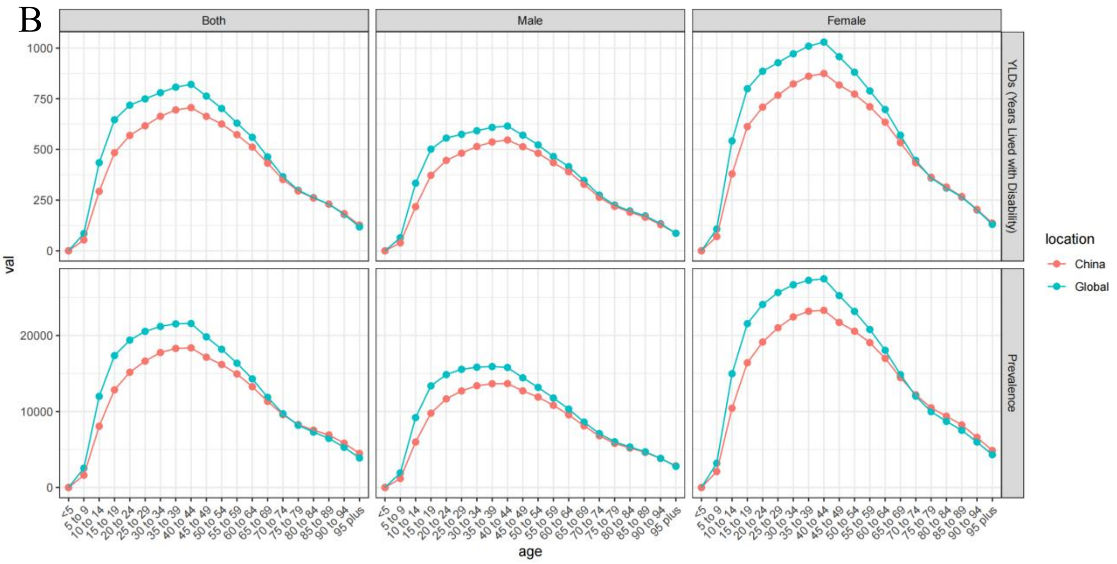

Fig. S98 (A) The number of prevalent cases and years lived with disability of migraine in different ages in 2021; (B) The age-standardized rates of prevalent cases and years lived with disability of migraine in different ages in 2021.

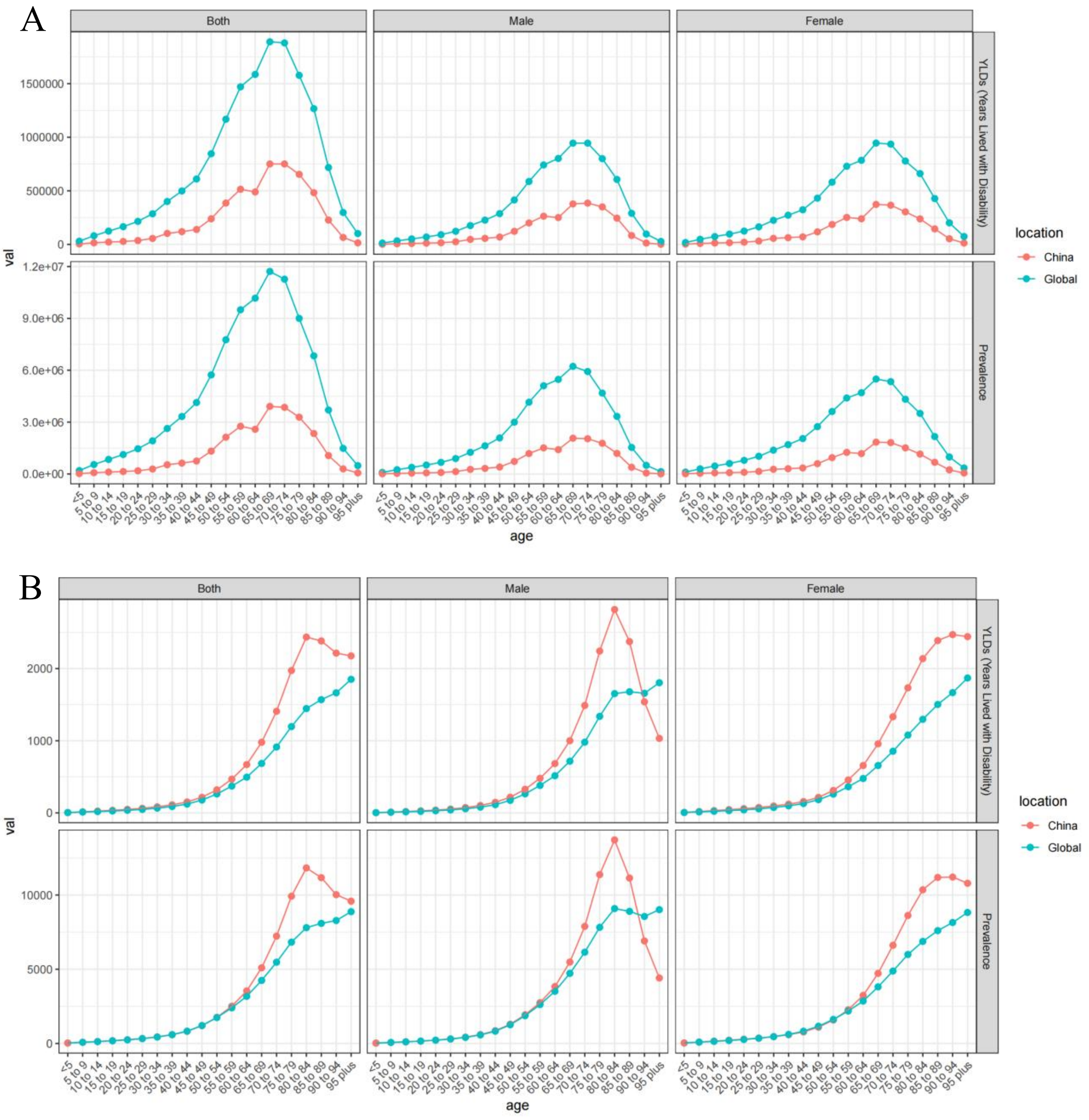

Fig. S99 (A) The number of prevalent cases and years lived with disability of stroke in different ages in 2021; (B) The age-standardized rates of prevalent cases and years lived with disability of stroke in different ages in 2021.

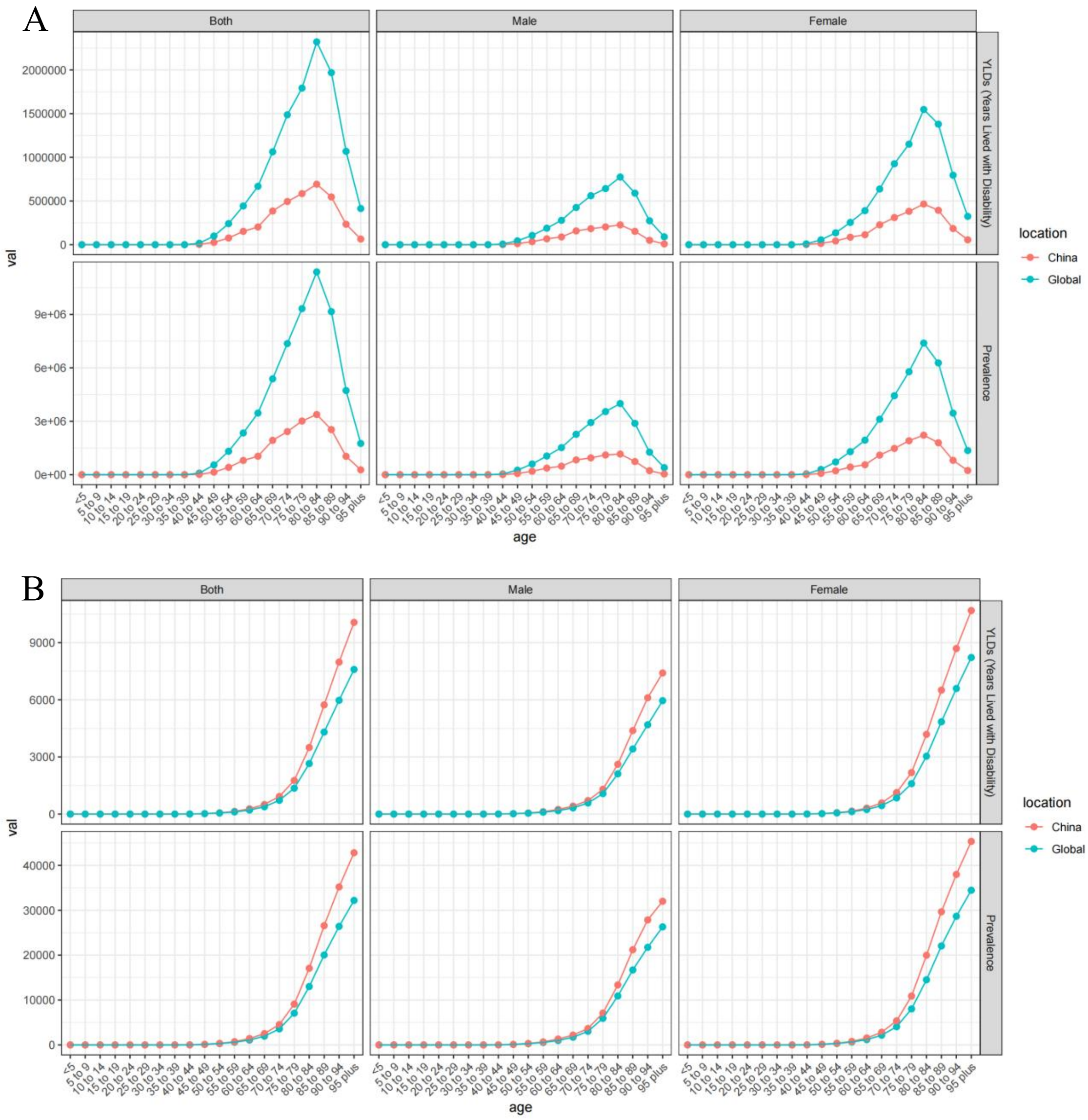

Fig. S100 (A) The number of prevalent cases and years lived with disability of Alzheimer's disease and other dementias in different ages in 2021; (B) The age-standardized rates of prevalent cases and years lived with disability of Alzheimer's disease and other dementias in different ages in 2021.

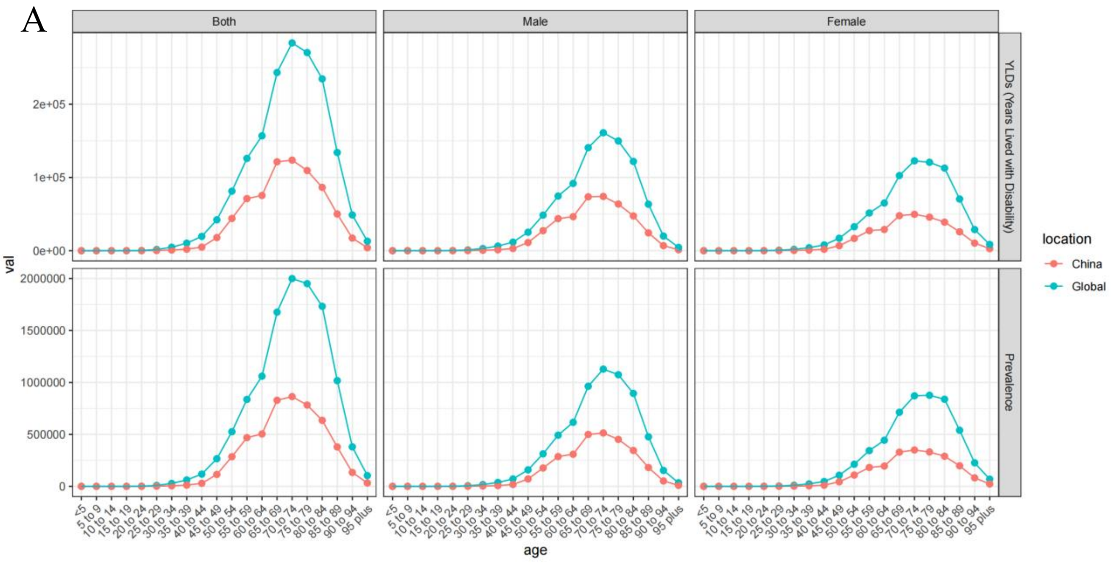

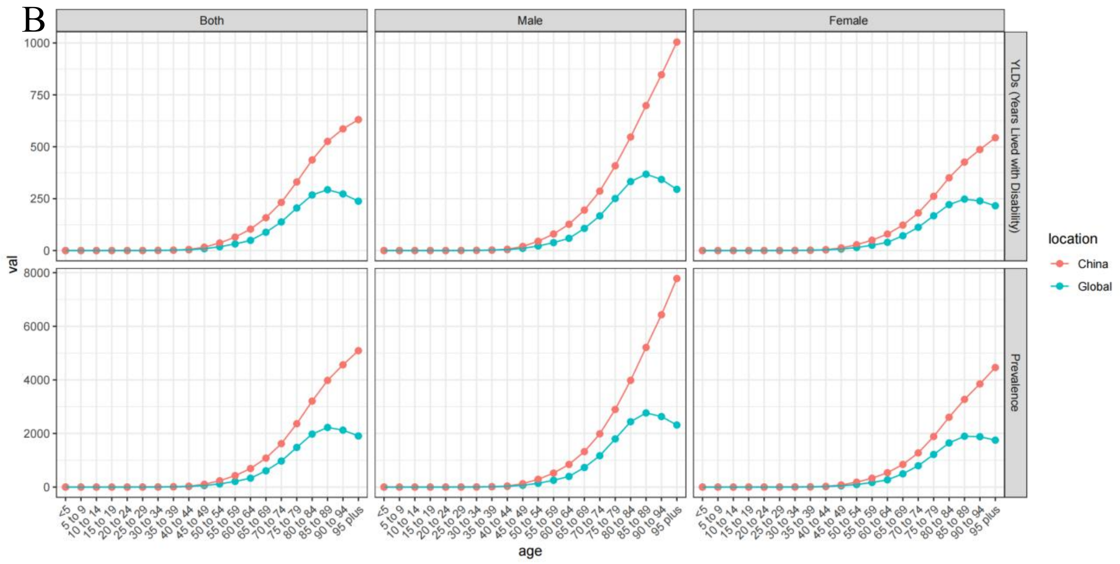

Fig. S101 (A) The number of prevalent cases and years lived with disability of Parkinson's disease in different ages in 2021; (B) The age-standardized rates of prevalent cases and years lived with disability of Parkinson's disease in different ages in 2021.

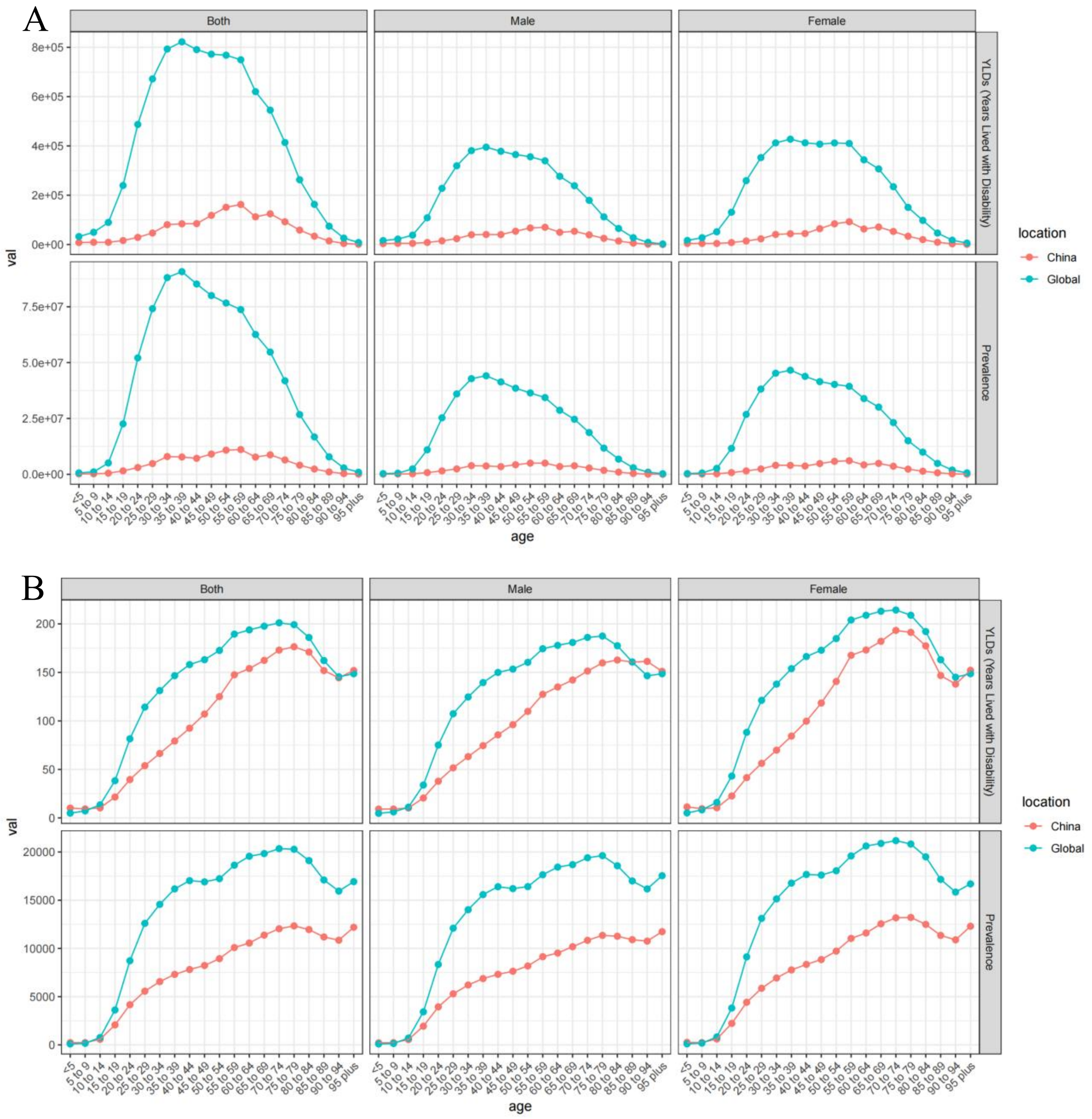

Fig. S102 (A) The number of prevalent cases and years lived with disability of upper digestive system diseases in different ages in 2021; (B) The age-standardized rates of prevalent cases and years lived with disability of upper digestive system diseases in different ages in 2021.

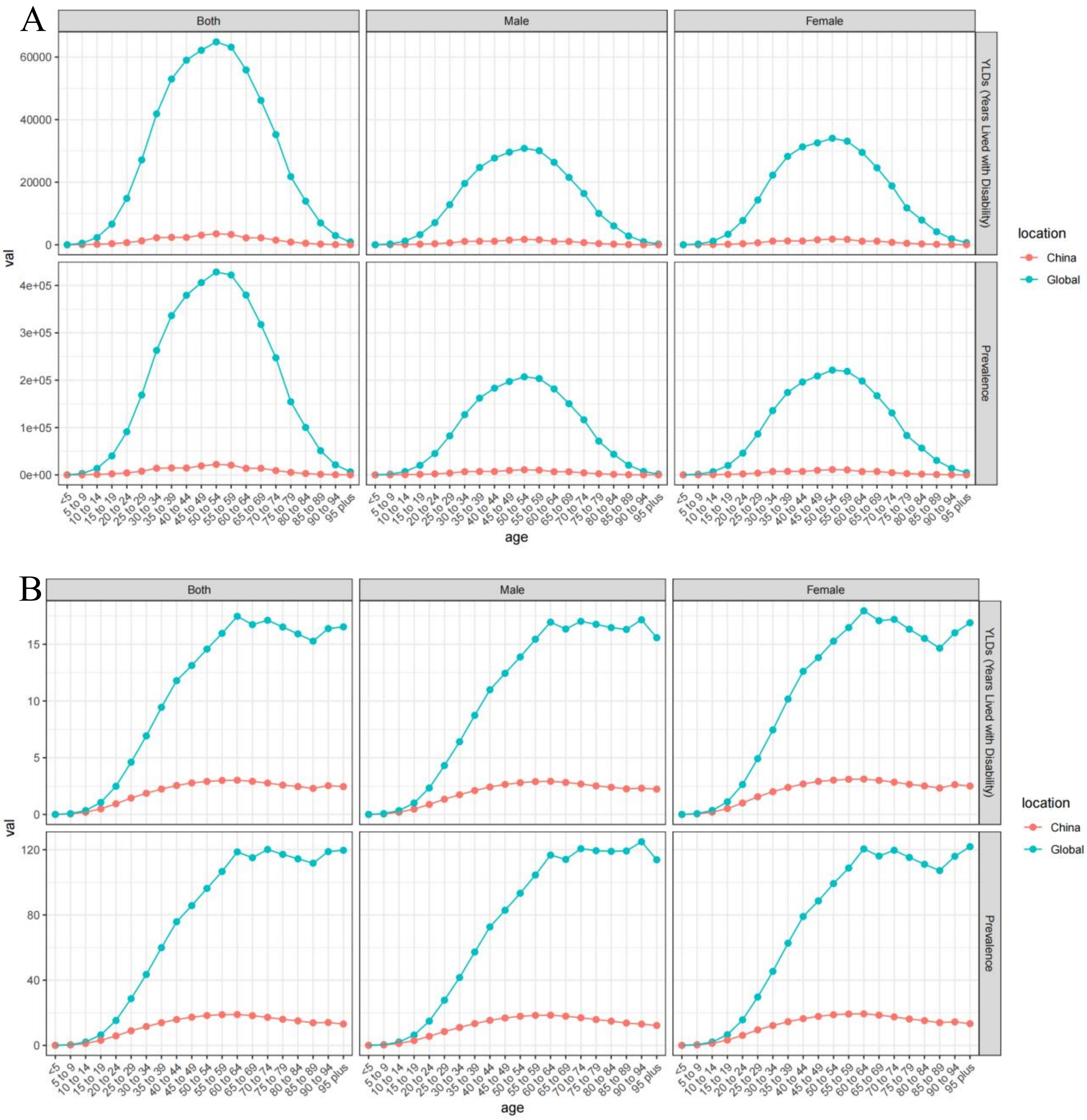

Fig. S103 (A) The number of prevalent cases and years lived with disability of inflammatory bowel disease in different ages in 2021; (B) The age-standardized rates of prevalent cases and years lived with disability of inflammatory bowel disease in different ages in 2021.

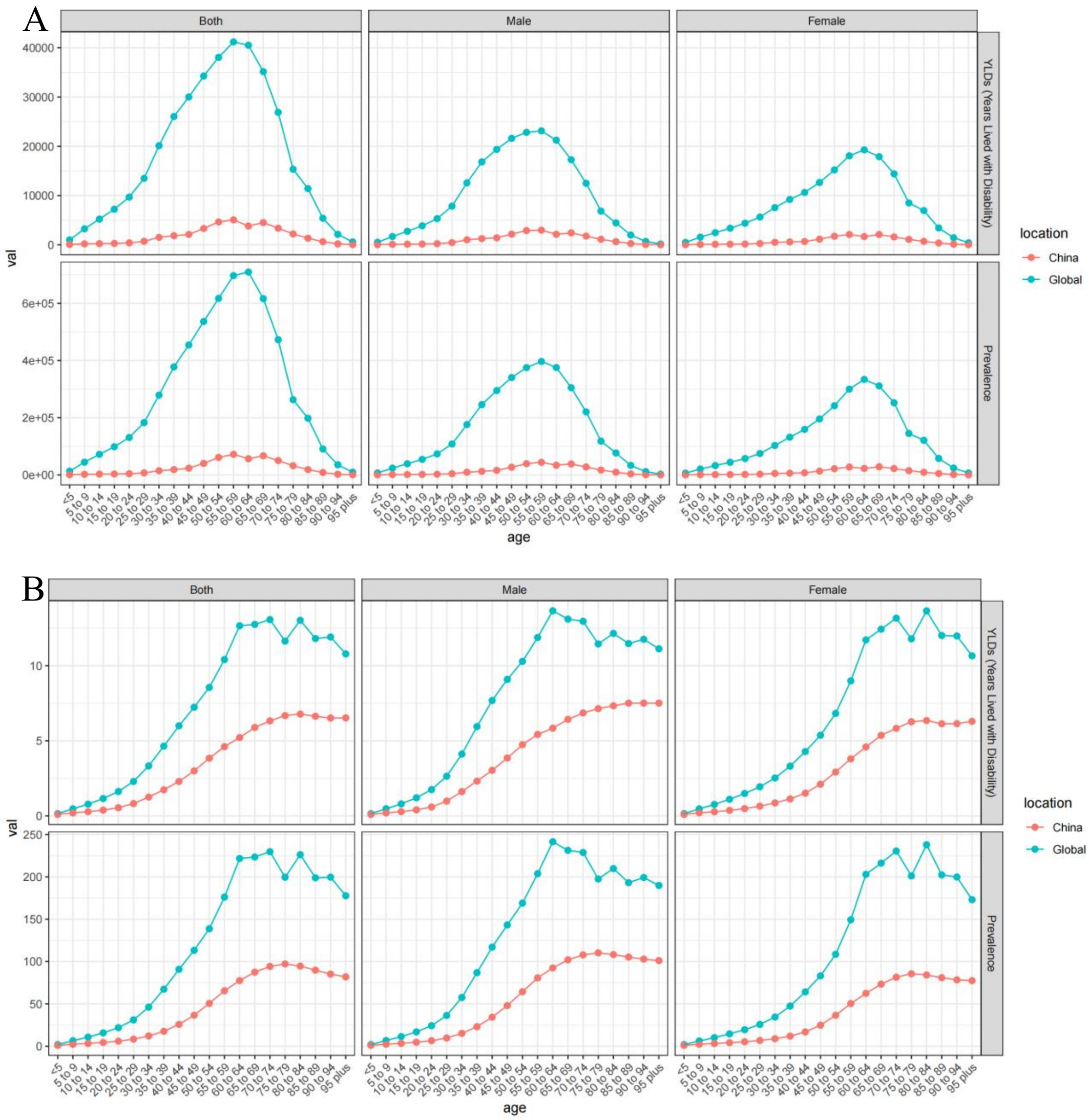

Fig. S104 (A) The number of prevalent cases and years lived with disability of pancreatitis in different ages in 2021; (B) The age-standardized rates of prevalent cases and years lived with disability of pancreatitis in different ages in 2021.

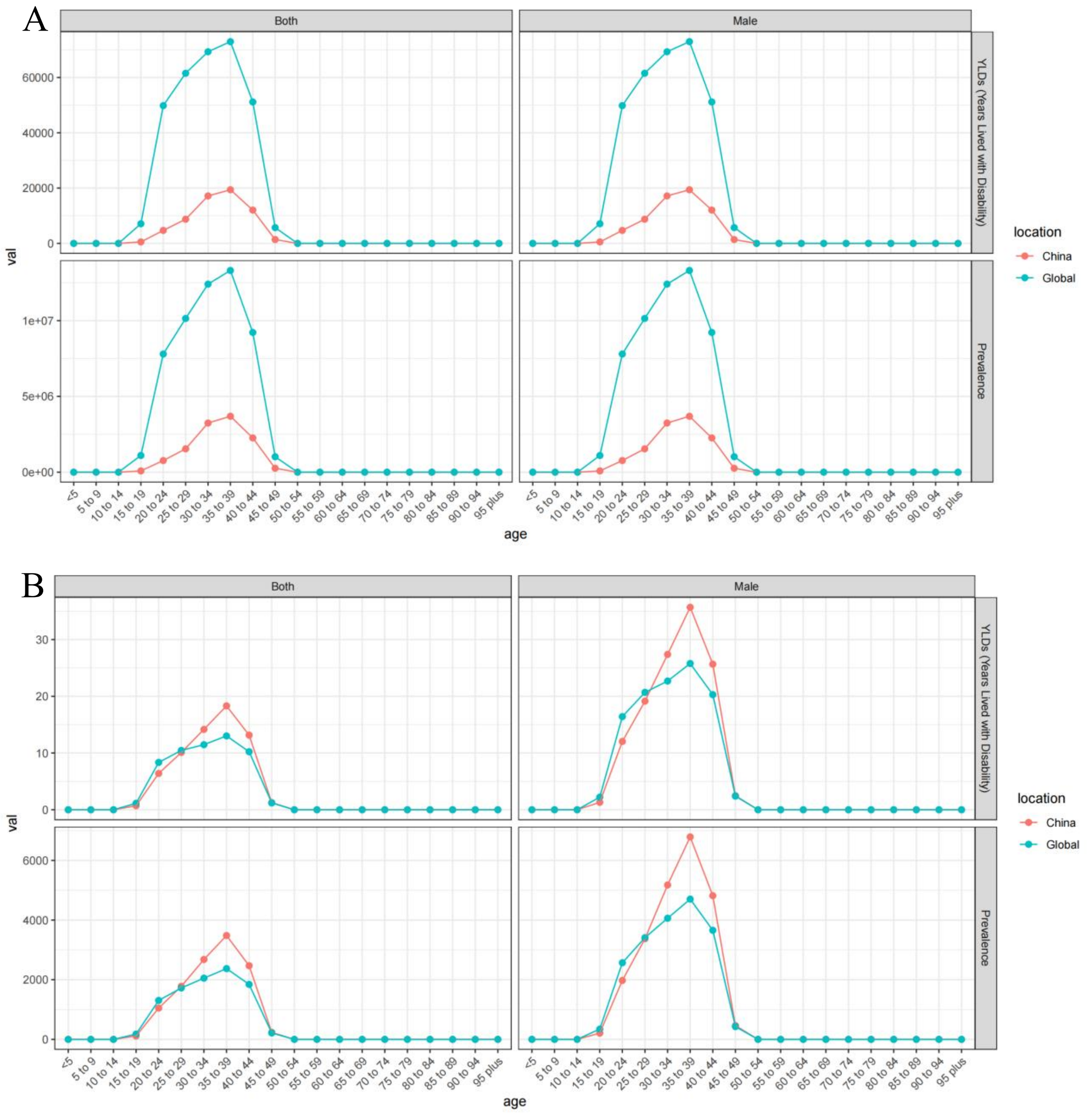

Fig. S105 (A) The number of prevalent cases and years lived with disability of male infertility in different ages in 2021; (B) The age-standardized rates of prevalent cases and years lived with disability of male infertility in different ages in 2021.

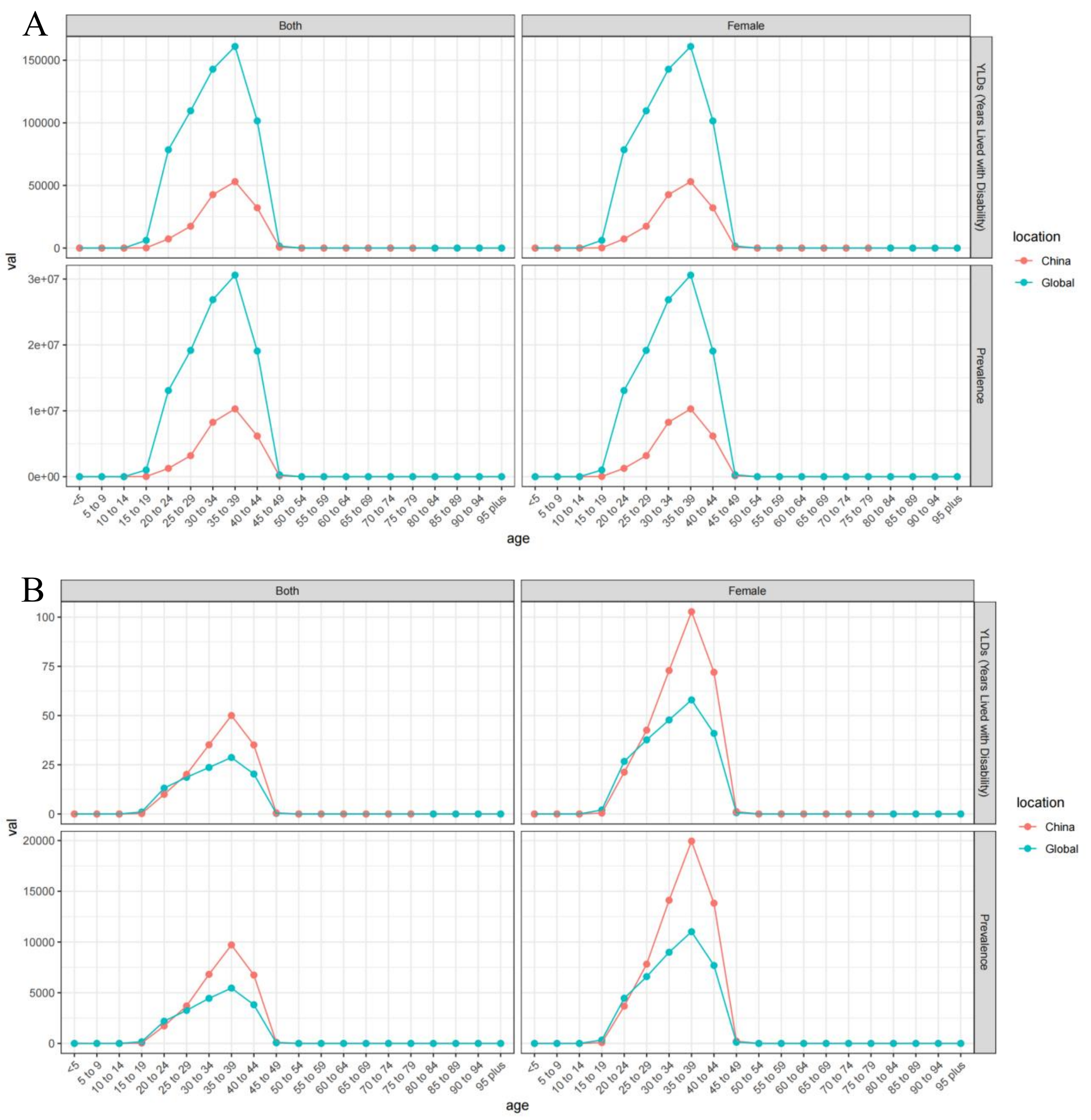

Fig. S106 (A) The number of prevalent cases and years lived with disability of female infertility in different ages in 2021; (B) The age-standardized rates of prevalent cases and years lived with disability of female infertility in different ages in 2021.

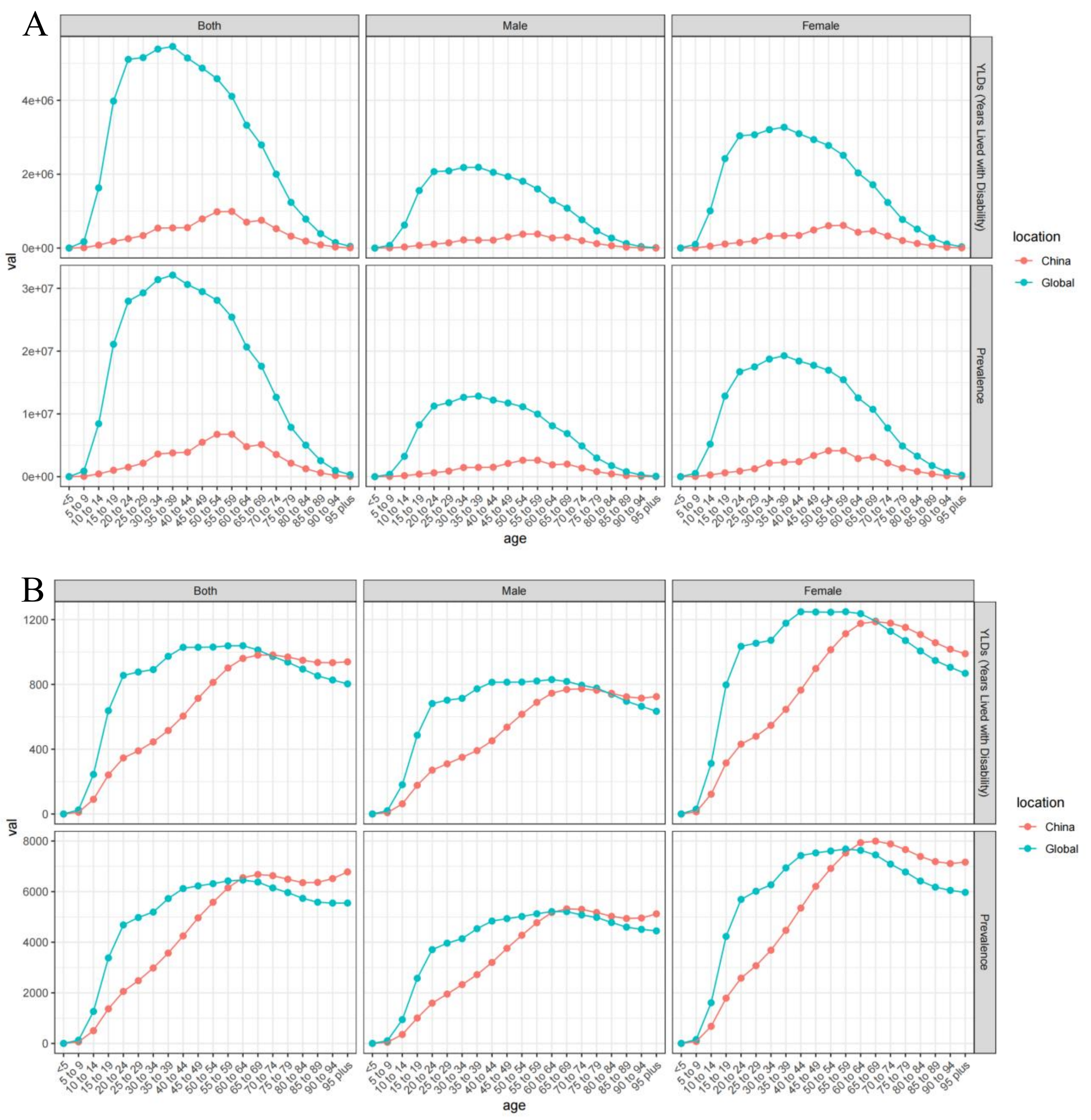

Fig. S107 (A) The number of prevalent cases and years lived with disability of depressive disorders in different ages in 2021; (B) The age-standardized rates of prevalent cases and years lived with disability of depressive disorders in different ages in 2021.

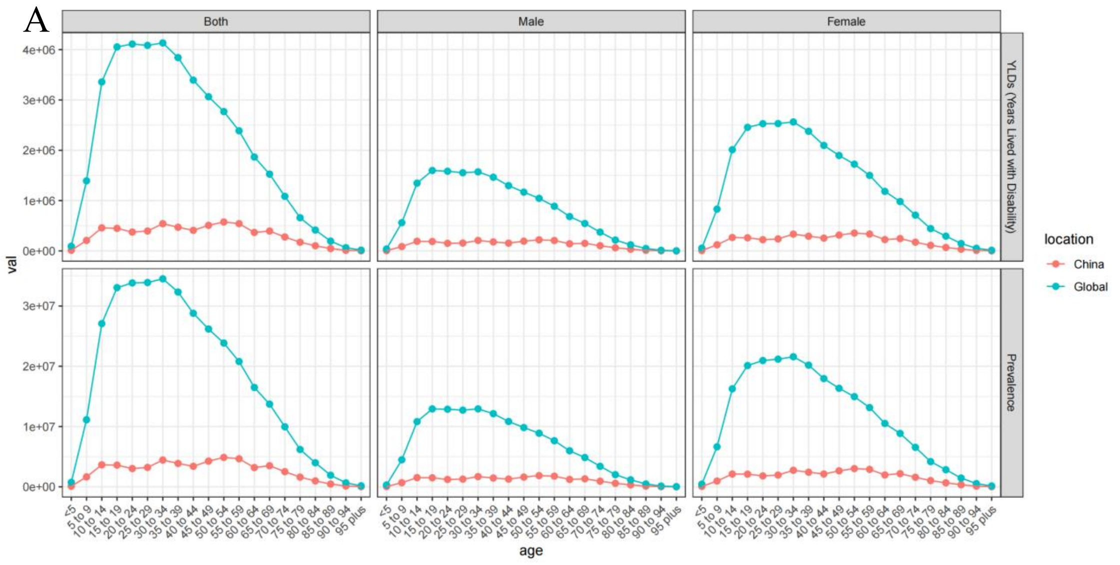

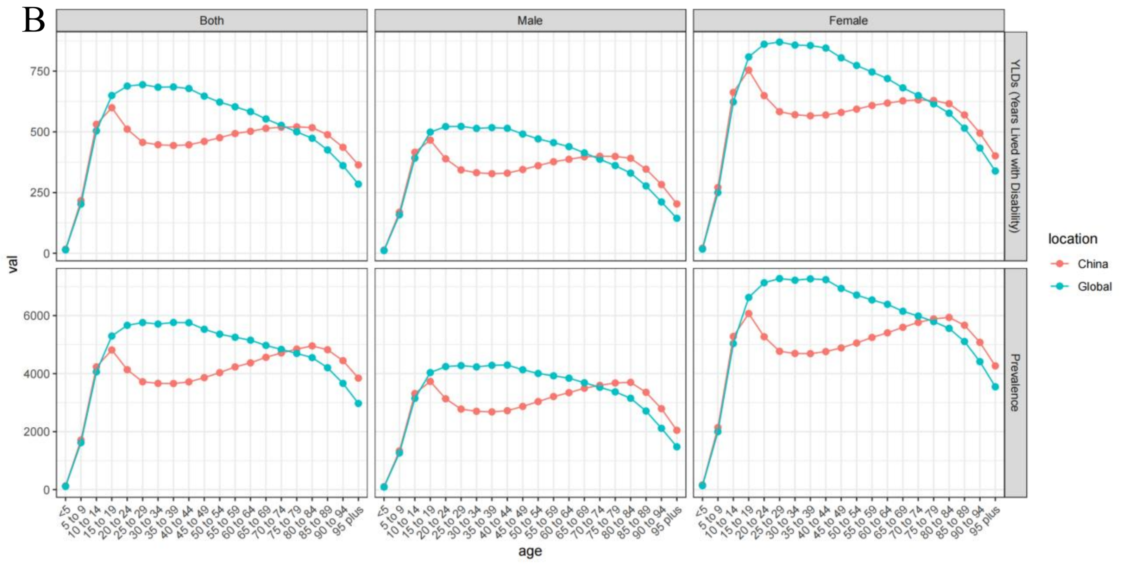

Fig. S108 (A) The number of prevalent cases and years lived with disability of anxiety disorders in different ages in 2021; (B) The age-standardized rates of prevalent cases and years lived with disability of anxiety disorders in different ages in 2021.

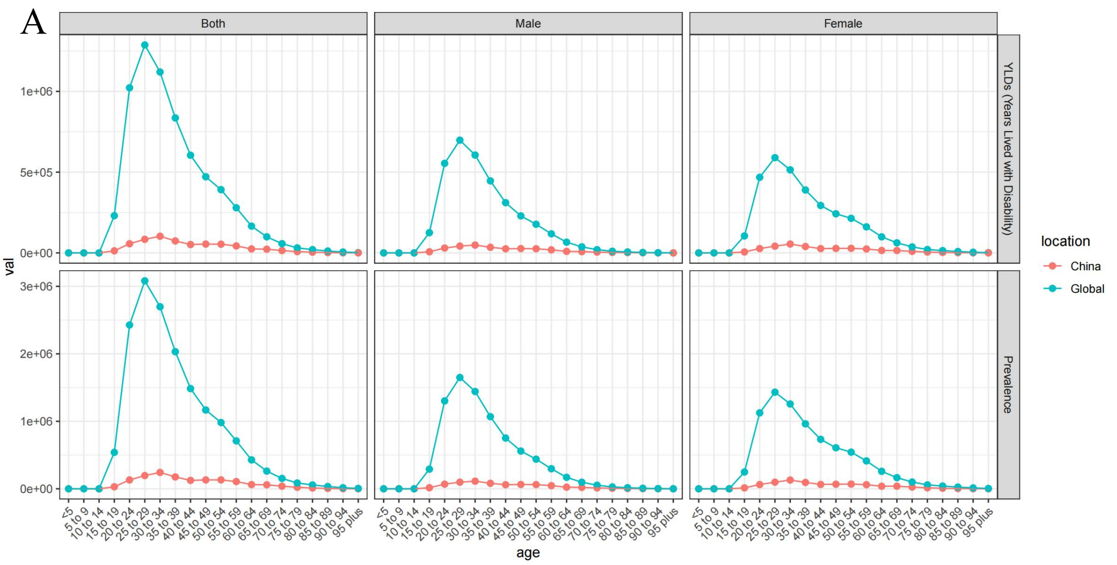

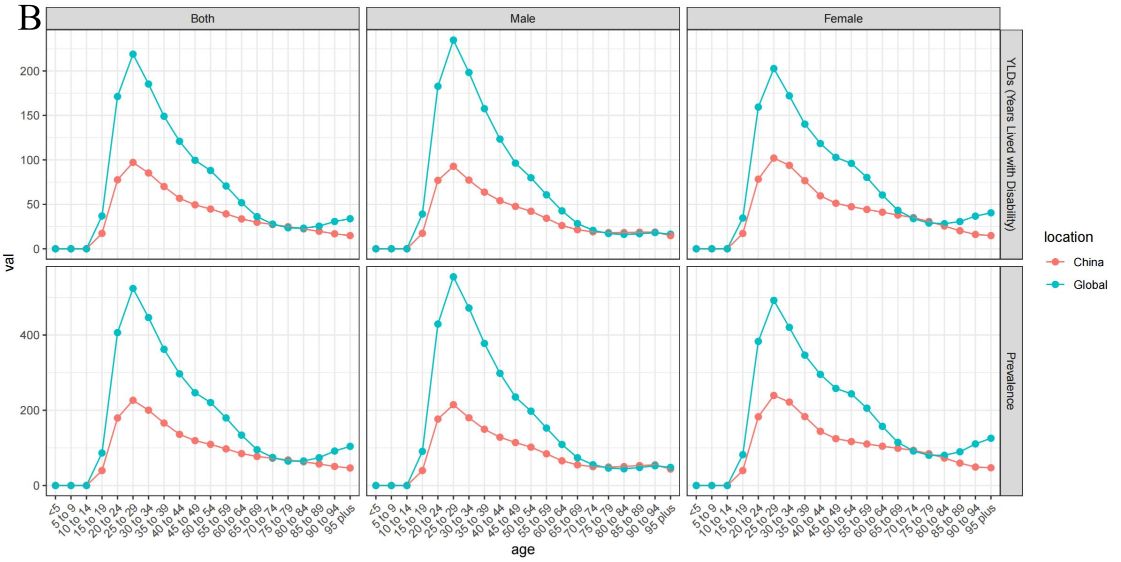

Fig. S109 (A) The number of prevalent cases and years lived with disability of opioid use disorders in different ages in 2021; (B) The age-standardized rates of prevalent cases and years lived with disability of opioid use disorders in different ages in 2021.

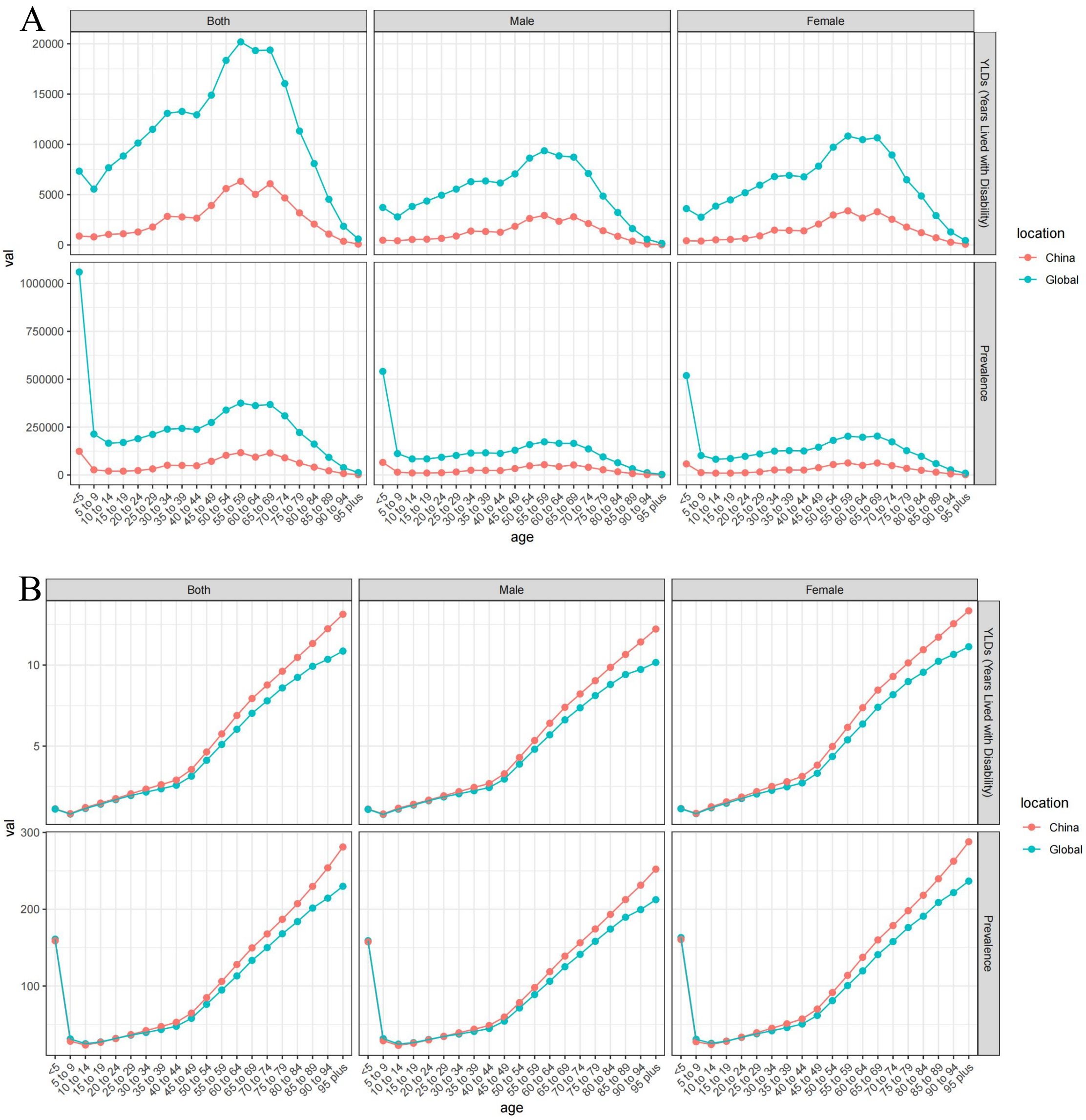

Fig. S110 (A) The number of prevalent cases and years lived with disability of varicella and herpes zoster in different ages in 2021; (B) The age-standardized rates of prevalent cases and years lived with disability of varicella and herpes zoster in different agesin different ages in 2021.

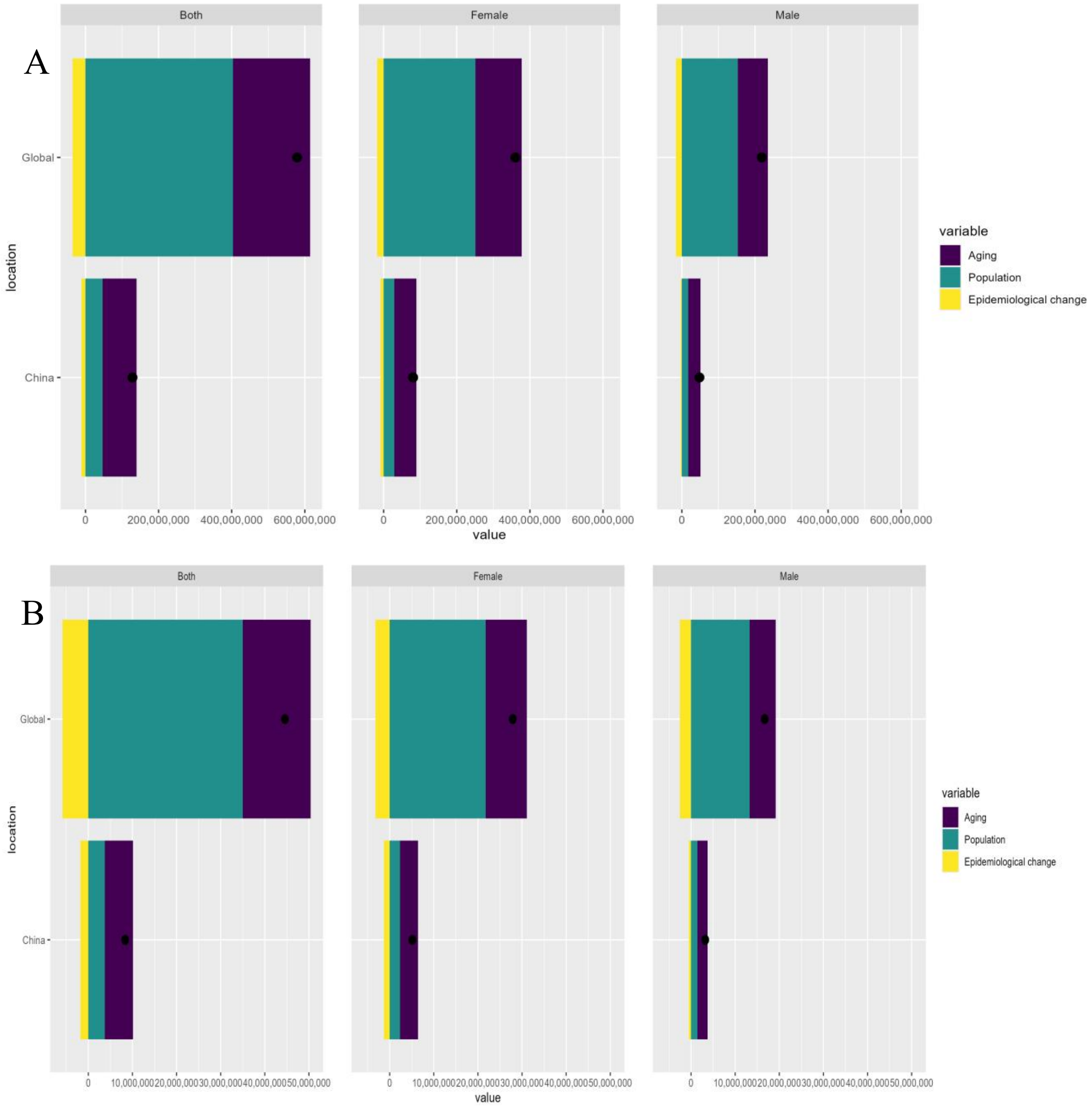

Fig. S111 (A) Decomposition analysis of musculoskeletal disorders change in prevalence from 1990 to 2021; (B) Decomposition analysis of musculoskeletal disorders change in years lived with disability from 1990 to 2021.

Notes: Black dots represent the total change contributed by all three components. A positive value for each component indicates a corresponding positive contribution, and a negative value indicates a corresponding negative contribution.

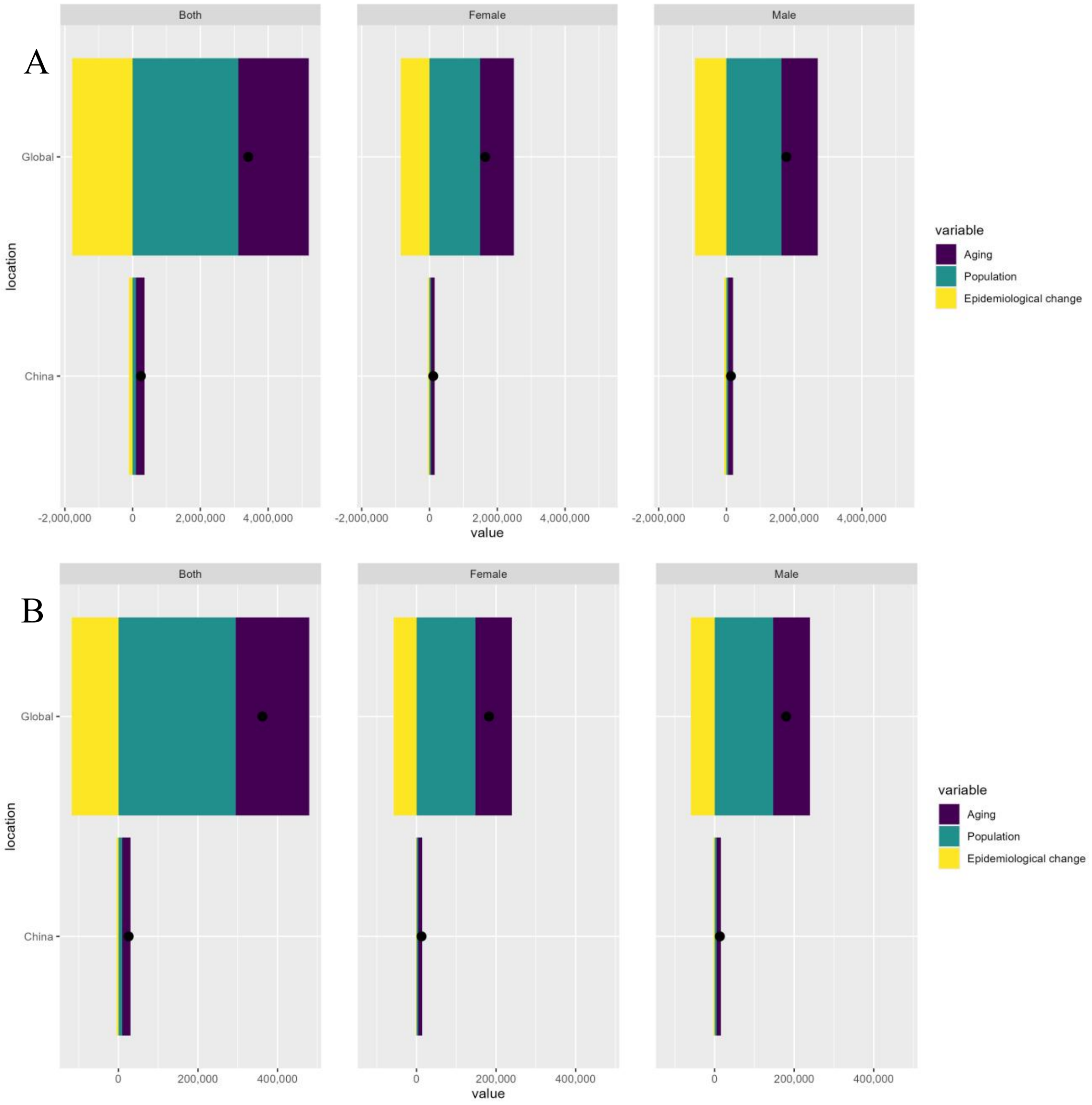

Fig. S112 (A) Decomposition analysis of digestive disorders change in prevalence from 1990 to 2021; (B) Decomposition analysis of digestive disorders change in years lived with disability from 1990 to 2021.

Notes: Black dots represent the total change contributed by all three components. A positive value for each component indicates a corresponding positive contribution, and a negative value indicates a corresponding negative contribution.

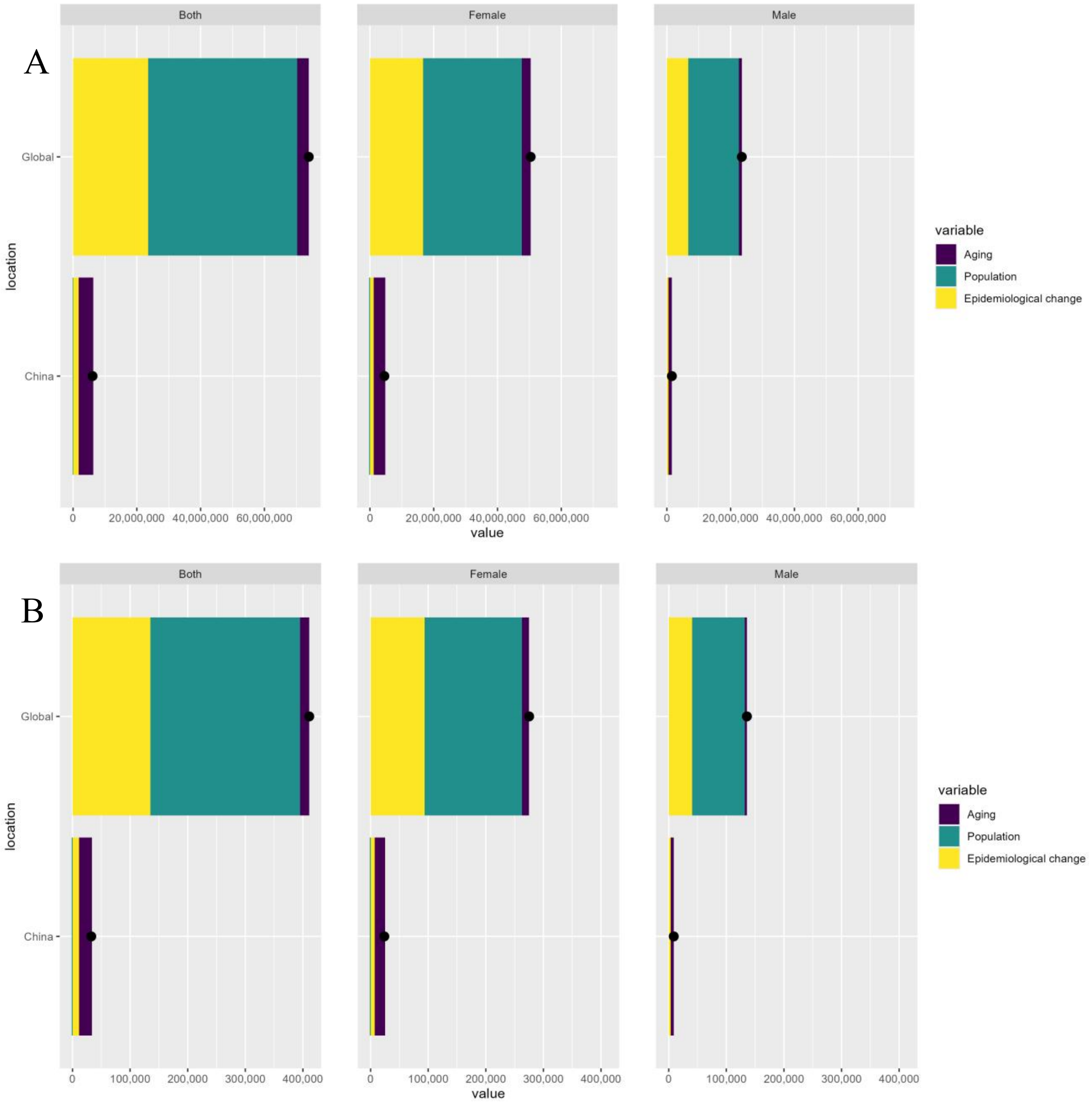

Fig. S113 (A) Decomposition analysis of genecological disorders disorders change in prevalence from 1990 to 2021; (B) Decomposition analysis of genecological disorders change in years lived with disability from 1990 to 2021.

Notes: Black dots represent the total change contributed by all three components. A positive value for each component indicates a corresponding positive contribution, and a negative value indicates a corresponding negative contribution.

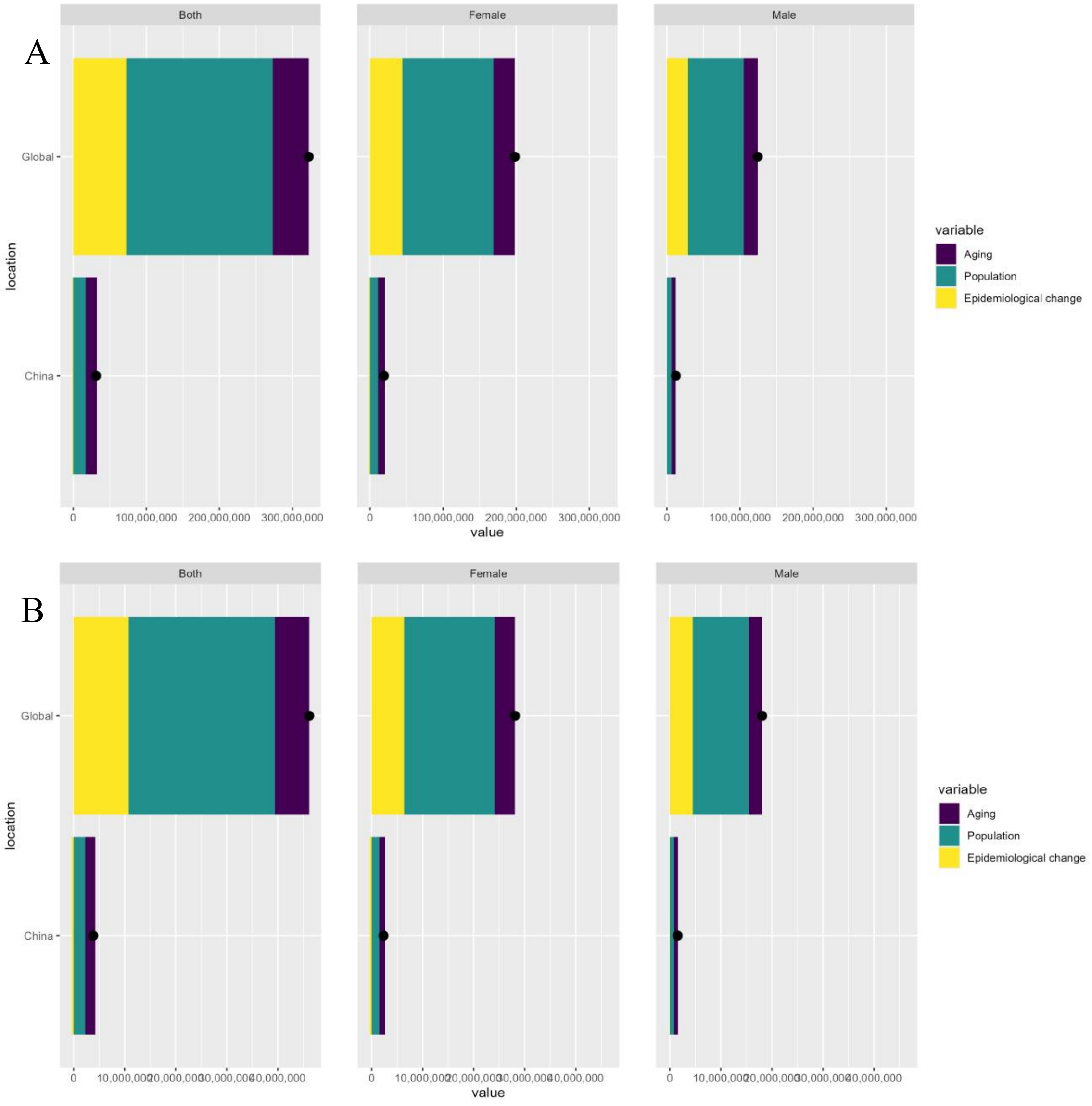

Fig. S114 (A) Decomposition analysis of mental disorders change in prevalence from 1990 to 2021; (B) Decomposition analysis of mental disorders change in years lived with disability from 1990 to 2021.
Notes: Black dots represent the total change contributed by all three components. A positive value for each component indicates a corresponding positive contribution, and a negative value indicates a corresponding negative contribution.

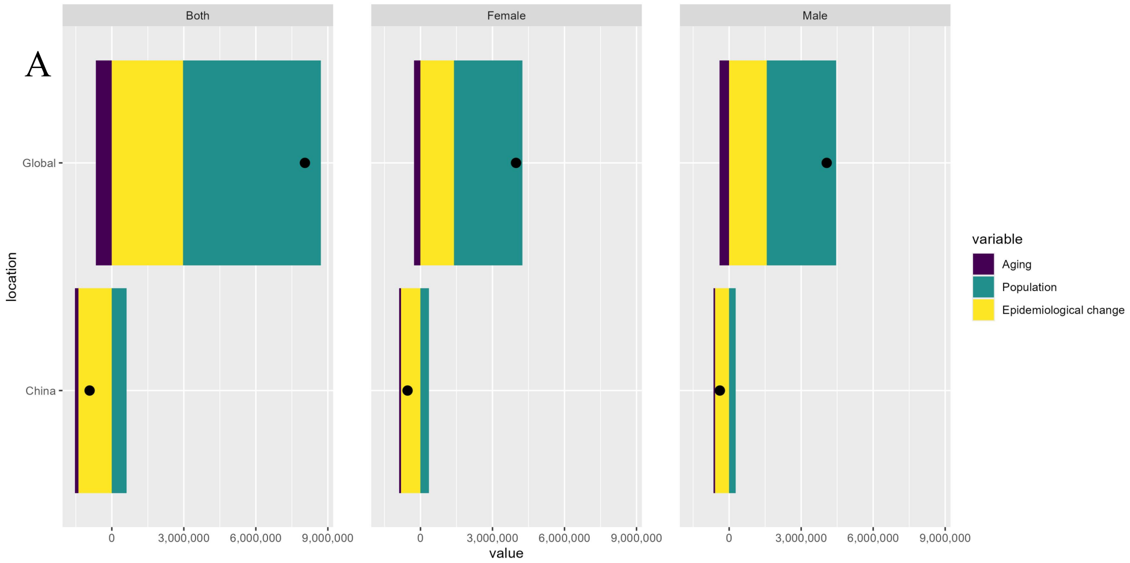

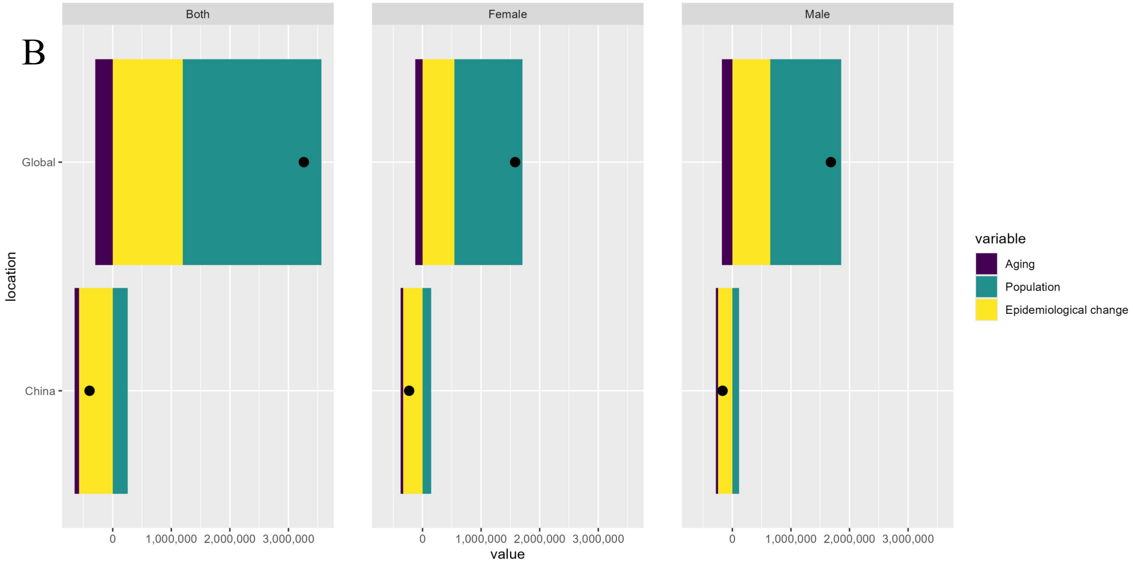

Fig. S115 (A) Decomposition analysis of substance use disorders change in prevalence from 1990 to 2021; (B) Decomposition analysis of substance use disorders change in years lived with disability from 1990 to 2021.

Notes: Black dots represent the total change contributed by all three components. A positive value for each component indicates a corresponding positive contribution, and a negative value indicates a corresponding negative contribution.

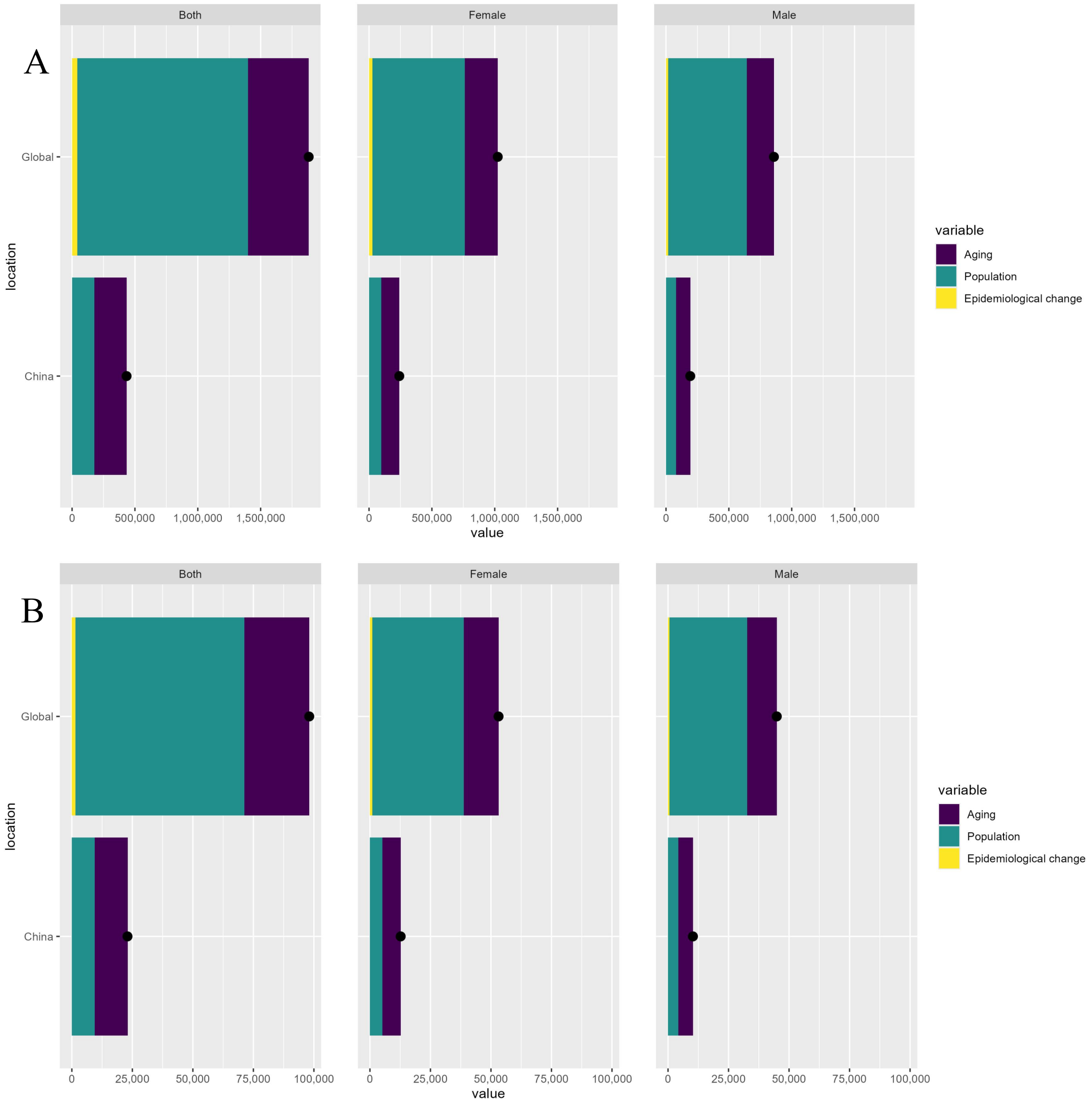

Fig. S116 (A) Decomposition analysis of infectious disorders change in prevalence from 1990 to 2021; (B) Decomposition analysis of infectious disorders change in years lived with disability from 1990 to 2021.

Notes: Black dots represent the total change contributed by all three components. A positive value for each component indicates a corresponding positive contribution, and a negative value indicates a corresponding negative contribution.

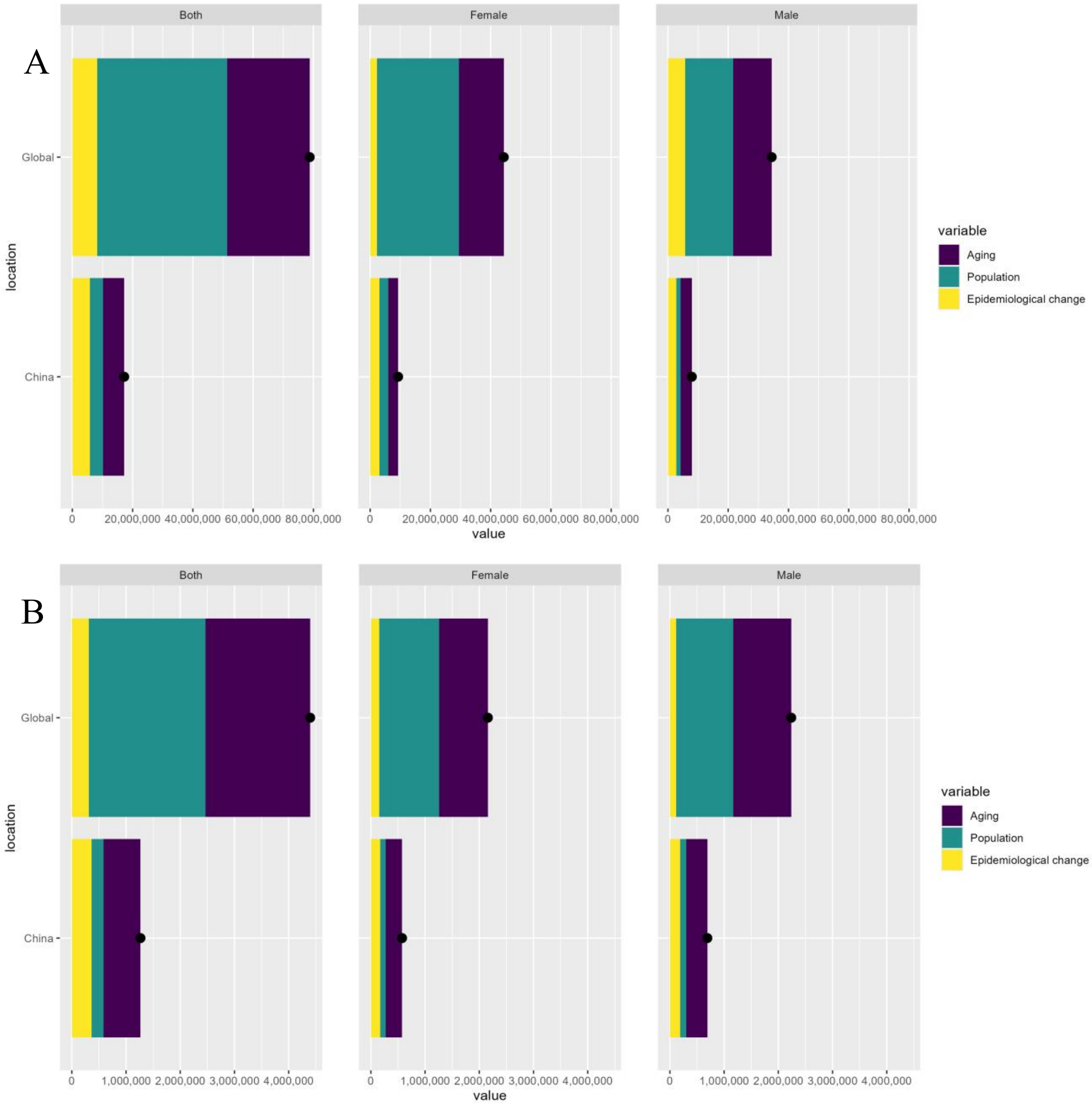

Fig. S117 (A) Decomposition analysis of neoplasms change in prevalence from 1990 to 2021; (B) Decomposition analysis of neoplasms change in years lived with disability from 1990 to 2021.

Notes: Black dots represent the total change contributed by all three components. A positive value for each component indicates a corresponding positive contribution, and a negative value indicates a corresponding negative contribution.

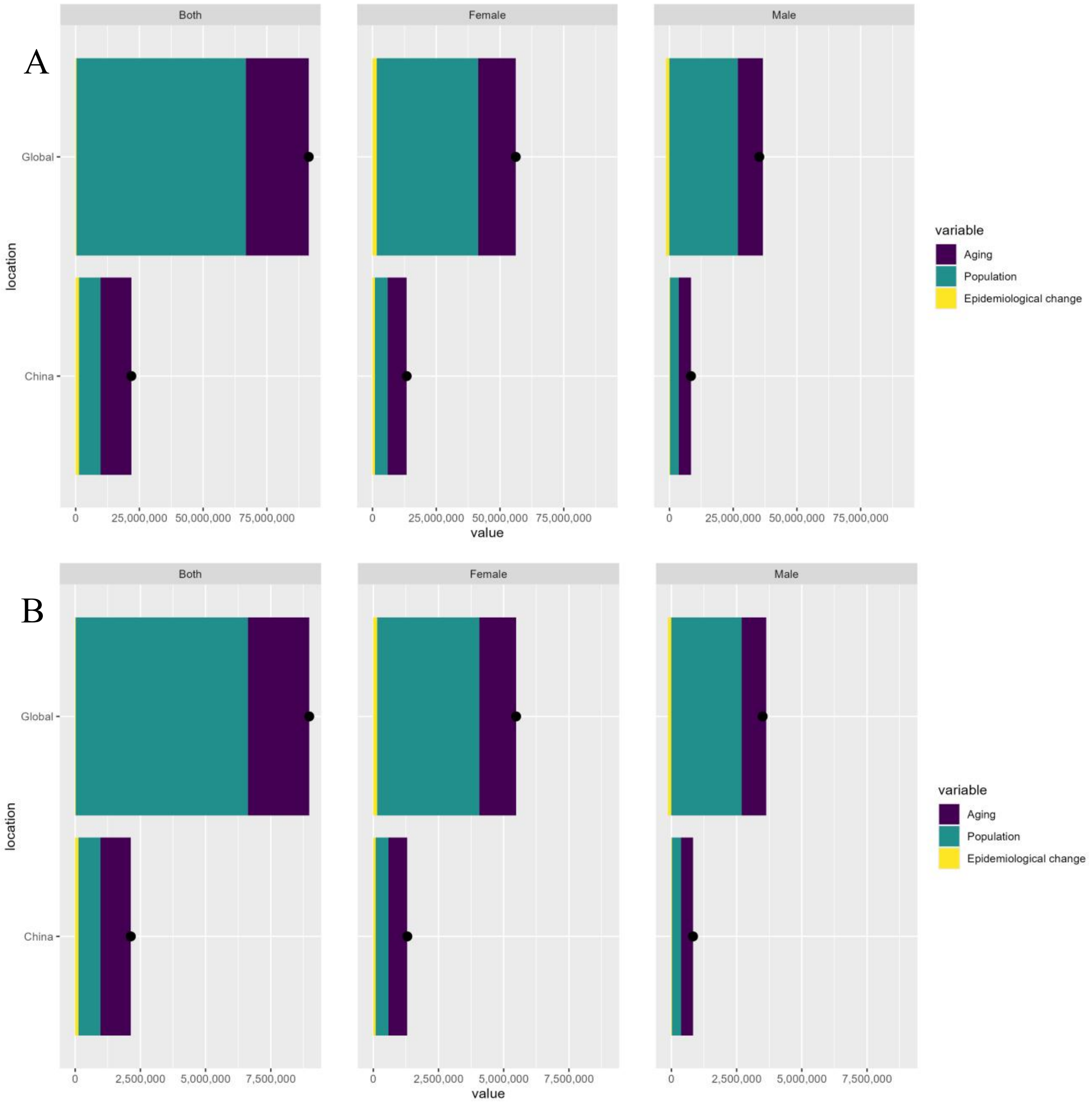

Fig. S118 (A) Decomposition analysis of neck pain change in prevalence from 1990 to 2021; (B) Decomposition analysis of neck pain change in years lived with disability from 1990 to 2021.

Notes: Black dots represent the total change contributed by all three components. A positive value for each component indicates a corresponding positive contribution, and a negative value indicates a corresponding negative contribution.

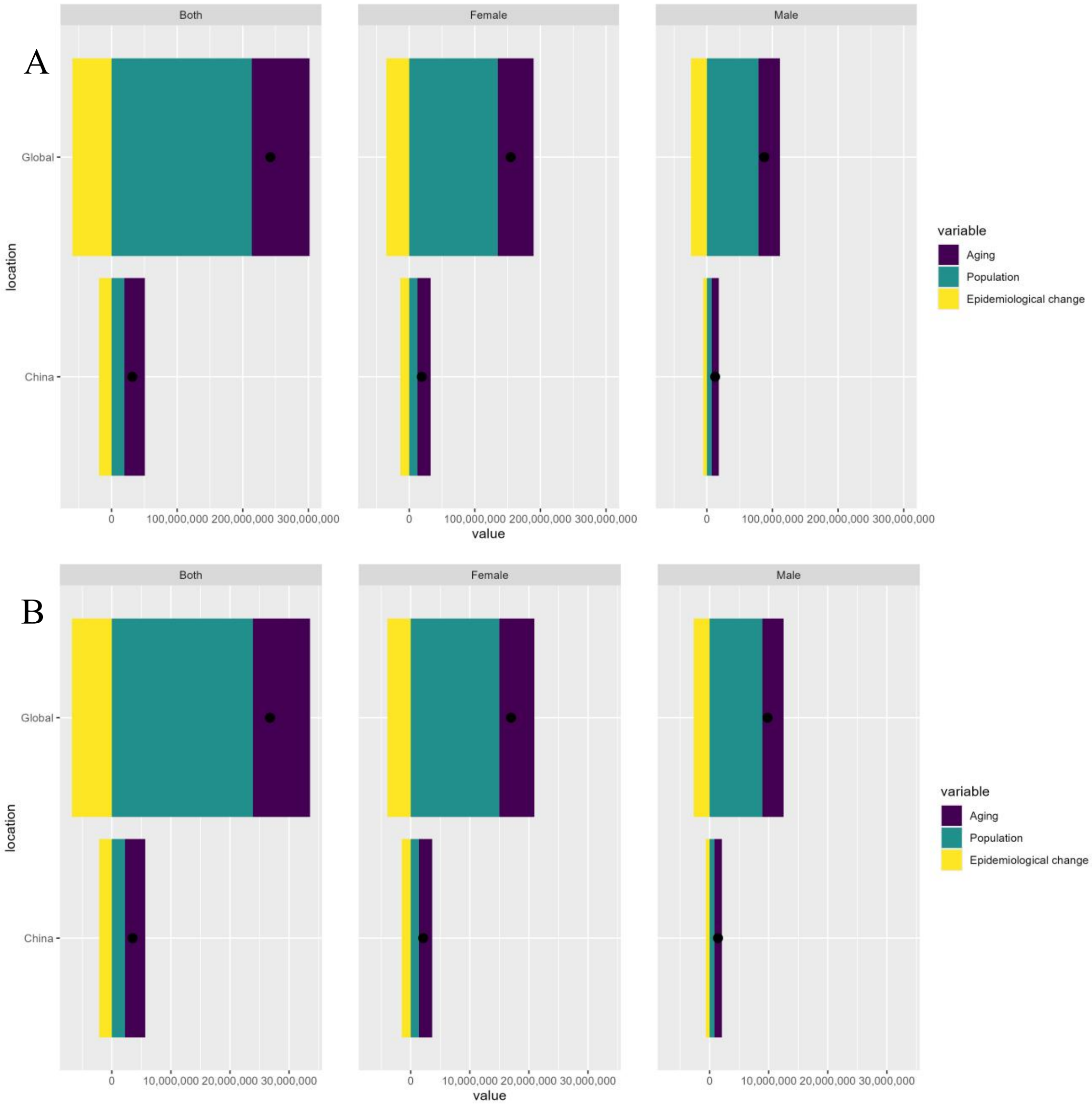

Fig. S119 (A) Decomposition analysis of low back pain change in prevalence from 1990 to 2021; (B) Decomposition analysis of low back pain change in years lived with disability from 1990 to 2021.

Notes: Black dots represent the total change contributed by all three components. A positive value for each component indicates a corresponding positive contribution, and a negative value indicates a corresponding negative contribution.

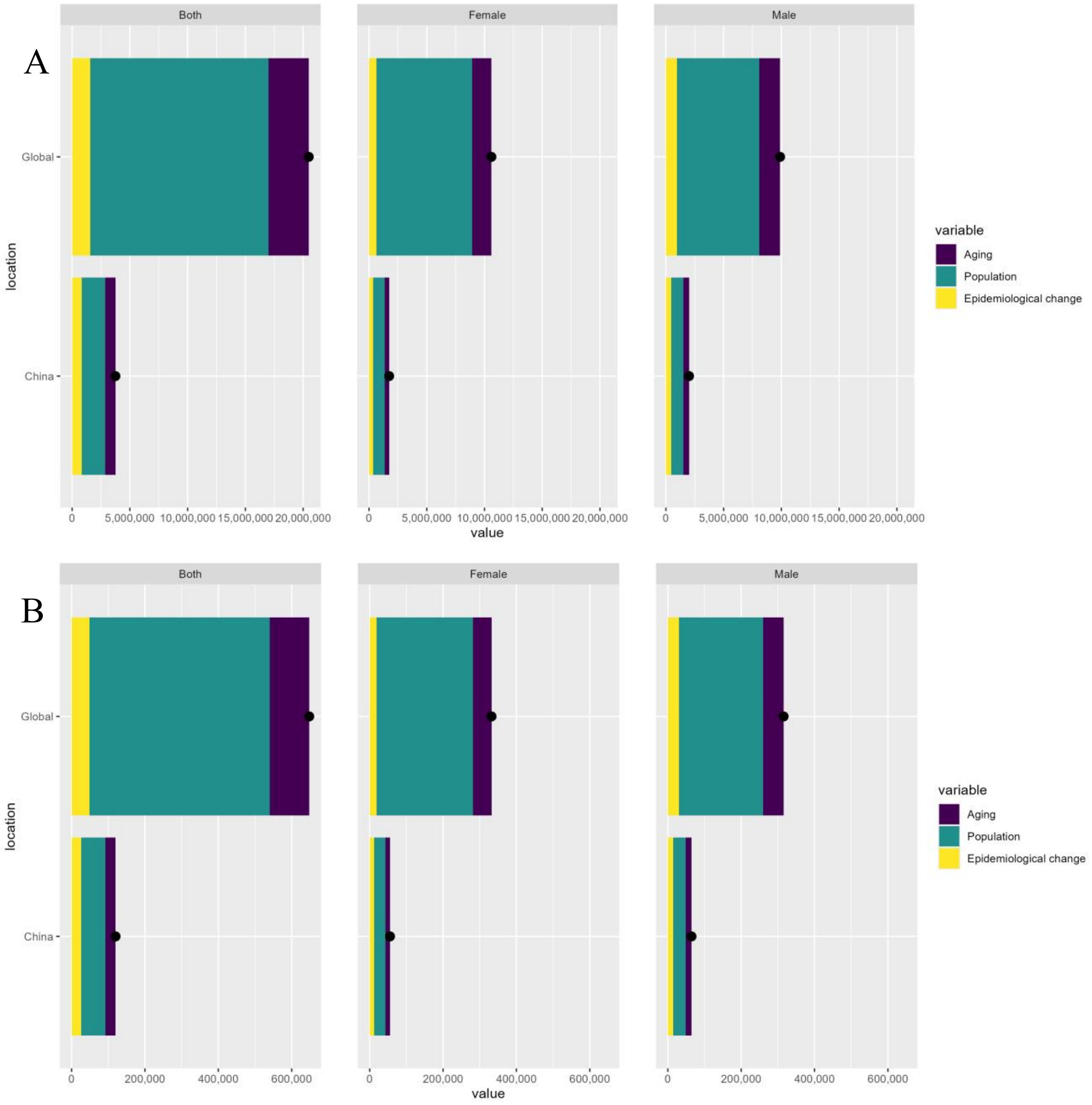

Fig. S120 (A) Decomposition analysis of hip osteoarthritis change in prevalence from 1990 to 2021; (B) Decomposition analysis of hip osteoarthritis change in years lived with disability from 1990 to 2021. Notes: Black dots represent the total change contributed by all three components. A positive value for each component indicates a corresponding positive contribution, and a negative value indicates a corresponding negative contribution.

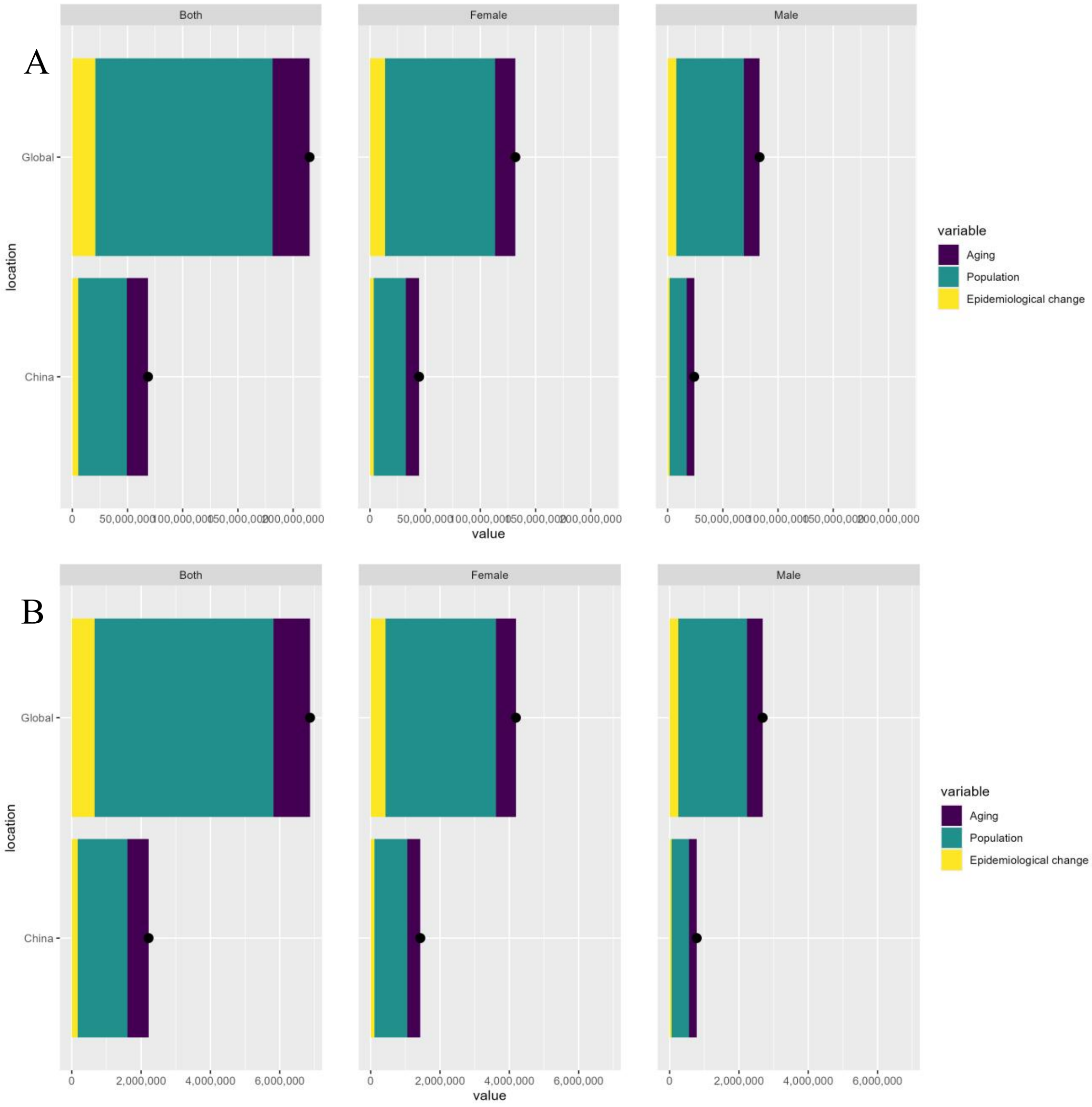

Fig. S121 (A) Decomposition analysis of knee osteoarthritis change in prevalence from 1990 to 2021; (B) Decomposition analysis of knee osteoarthritis change in years lived with disability from 1990 to 2021.

Notes: Black dots represent the total change contributed by all three components. A positive value for each component indicates a corresponding positive contribution, and a negative value indicates a corresponding negative contribution.

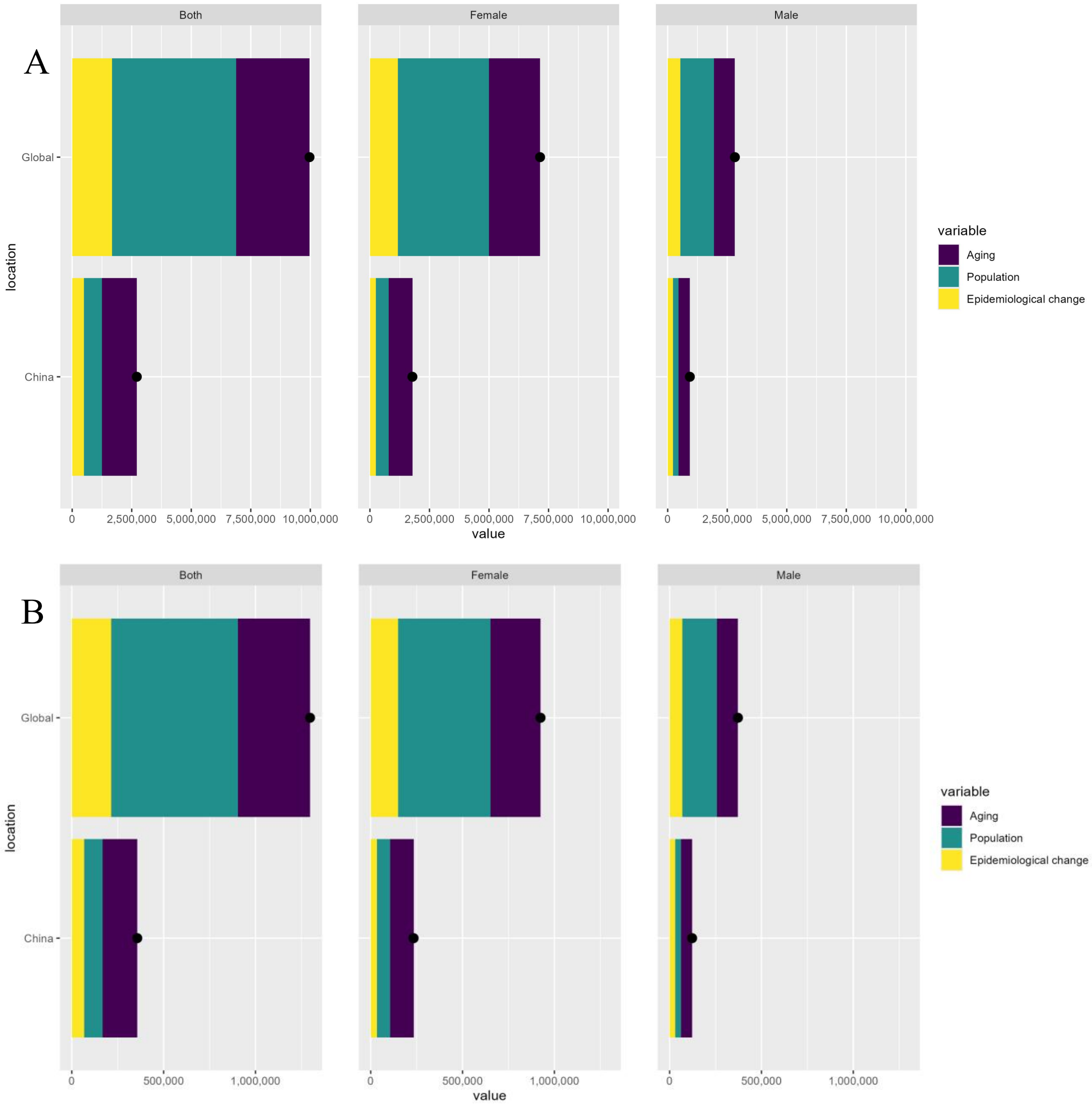

Fig. S122 (A) Decomposition analysis of rheumatoid arthritis change in prevalence from 1990 to 2021; (B) Decomposition analysis of rheumatoid arthritis change in years lived with disability from 1990 to 2021.

Notes: Black dots represent the total change contributed by all three components. A positive value for each component indicates a corresponding positive contribution, and a negative value indicates a corresponding negative contribution.

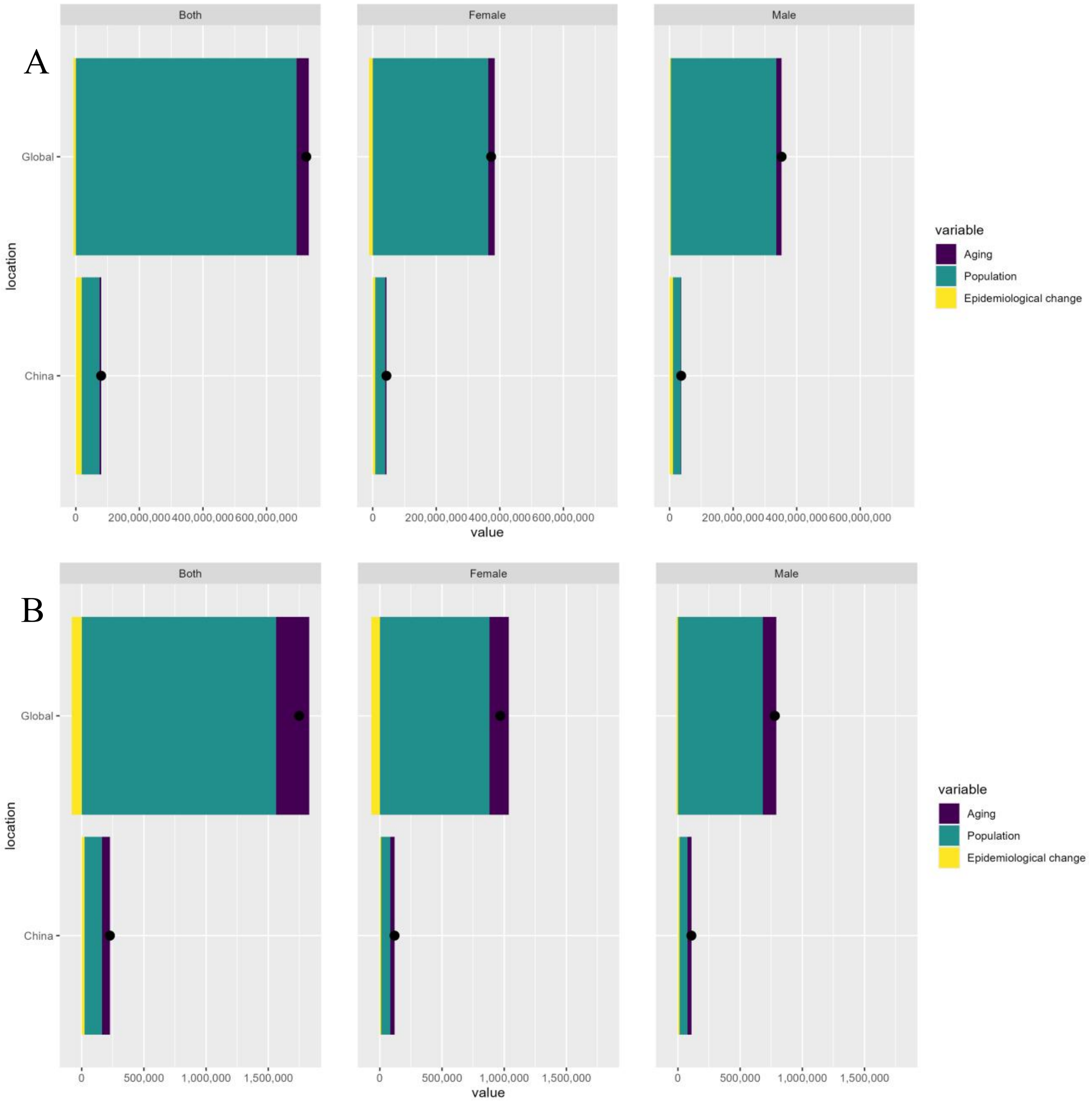

Fig. S123 (A) Decomposition analysis of tension-type headache change in prevalence from 1990 to 2021; (B) Decomposition analysis of tension-type headache change in years lived with disability from 1990 to 2021.

Notes: Black dots represent the total change contributed by all three components. A positive value for each component indicates a corresponding positive contribution, and a negative value indicates a corresponding negative contribution.

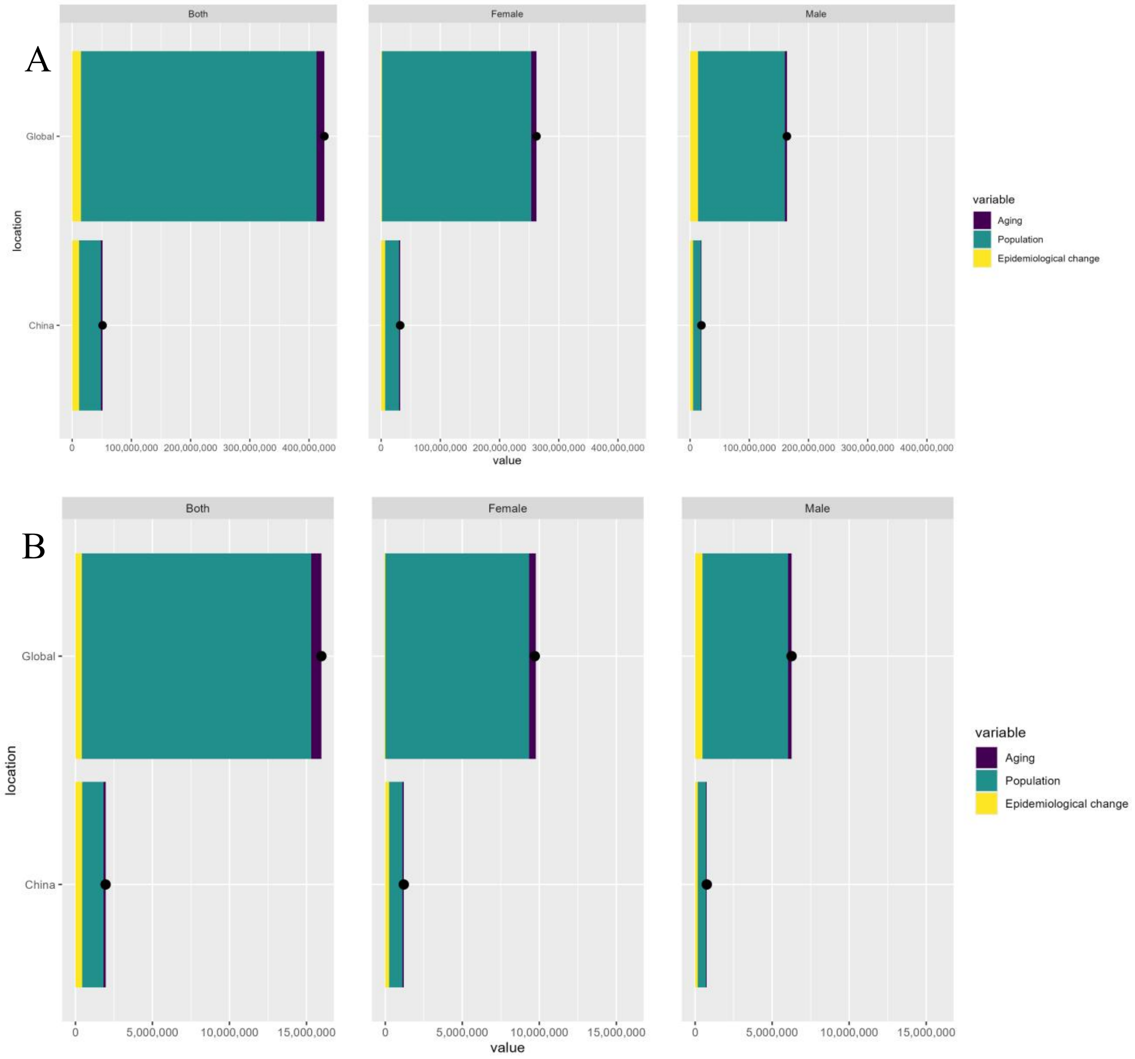

Fig. S124 (A) Decomposition analysis of migraine change in prevalence from 1990 to 2021; (B) Decomposition analysis of migraine change in years lived with disability from 1990 to 2021.

Notes: Black dots represent the total change contributed by all three components. A positive value for each component indicates a corresponding positive contribution, and a negative value indicates a corresponding negative contribution.

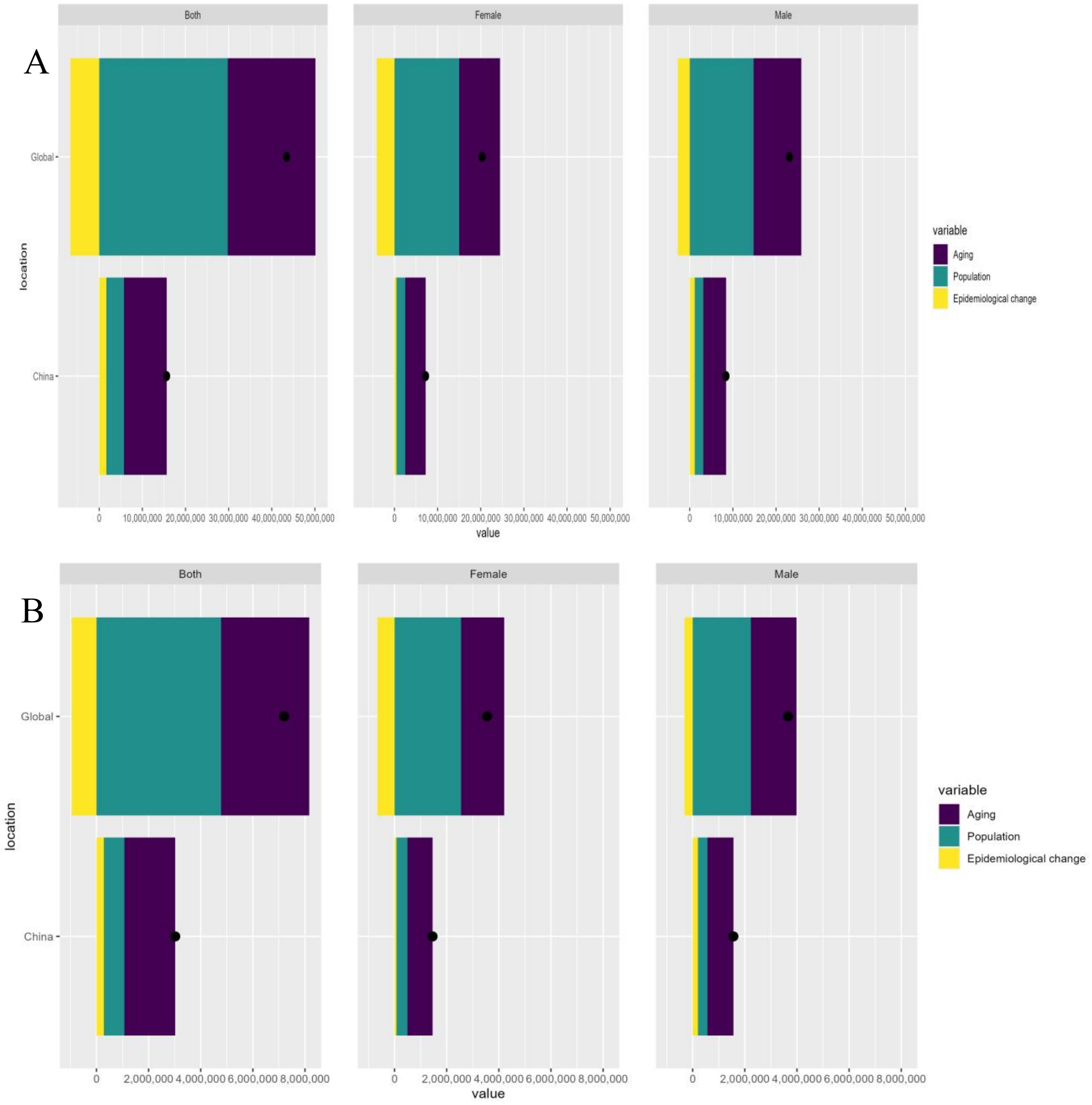

Fig. S125 (A) Decomposition analysis of stroke change in prevalence from 1990 to 2021; (B) Decomposition analysis of stroke change in years lived with disability from 1990 to 2021.

Notes: Black dots represent the total change contributed by all three components. A positive value for each component indicates a corresponding positive contribution, and a negative value indicates a corresponding negative contribution.

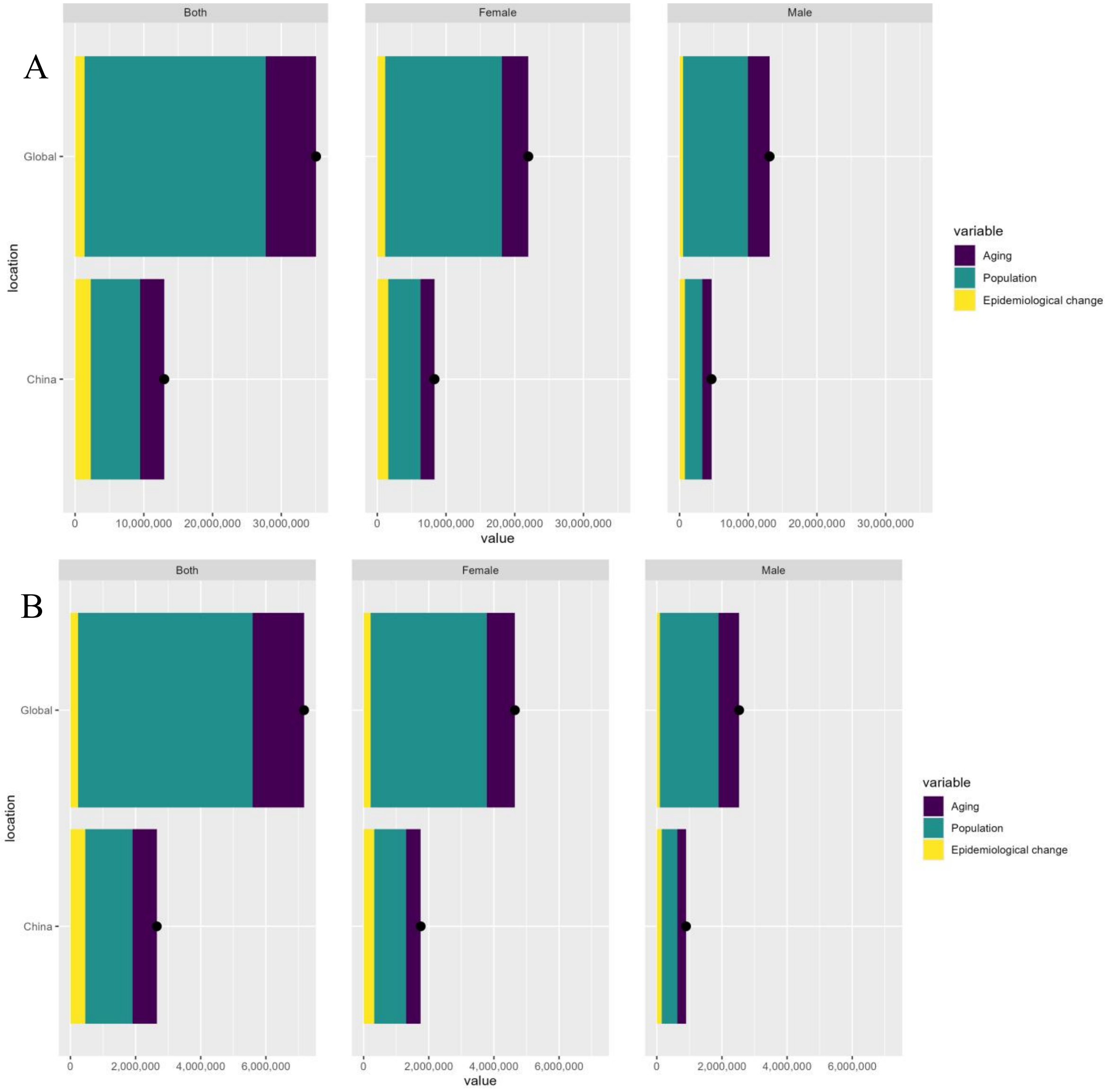

Fig. S126 (A) Decomposition analysis of Alzheimer's disease and other dementias change in prevalence from 1990 to 2021; (B) Decomposition analysis of Alzheimer's disease and other dementias change in years lived with disability from 1990 to 2021.

Notes: Black dots represent the total change contributed by all three components. A positive value for each component indicates a corresponding positive contribution, and a negative value indicates a corresponding negative contribution.

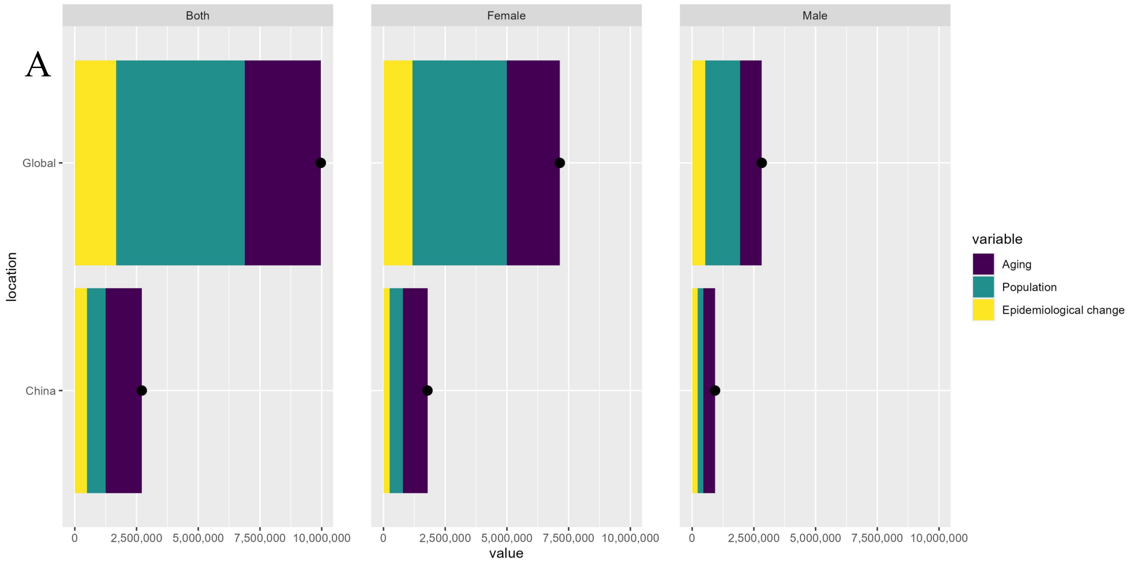

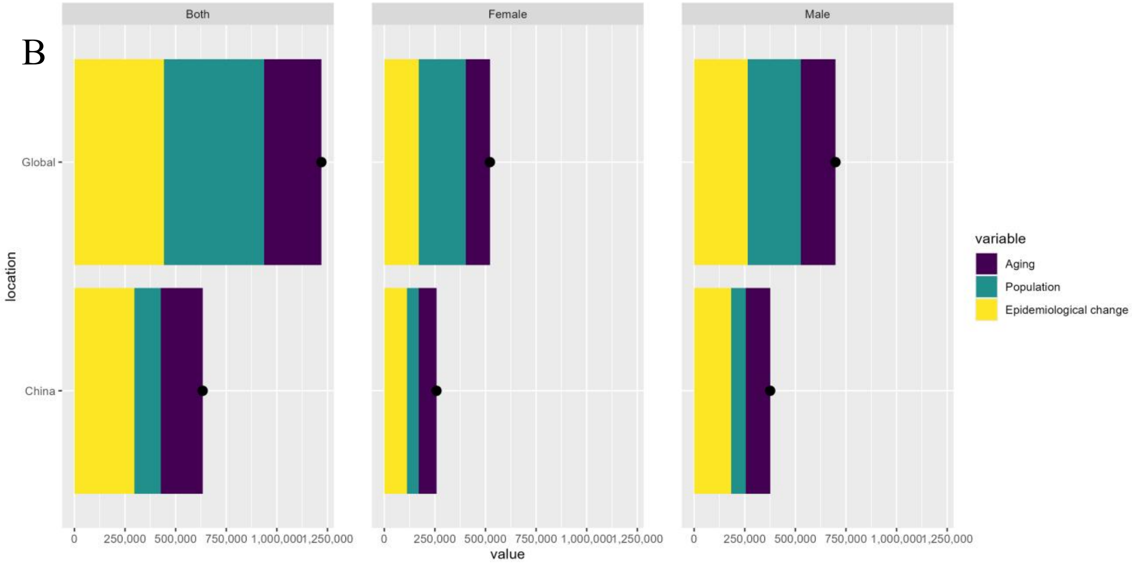

Fig. S127 (A) Decomposition analysis of Parkinson's disease change in prevalence from 1990 to 2021; (B) Decomposition analysis of Parkinson's disease change in years lived with disability from 1990 to 2021.

Notes: Black dots represent the total change contributed by all three components. A positive value for each component indicates a corresponding positive contribution, and a negative value indicates a corresponding negative contribution.

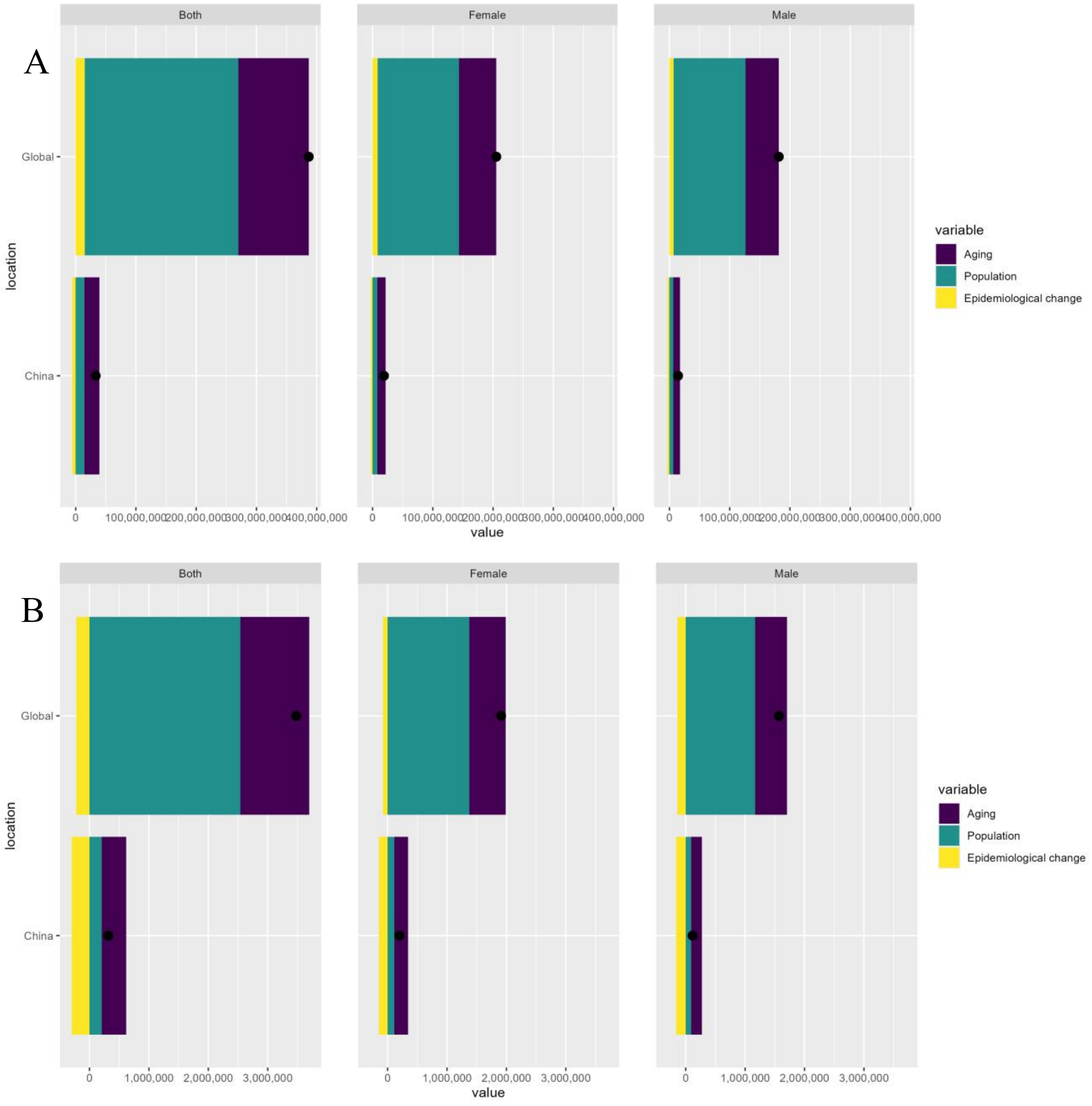

Fig. S128 (A) Decomposition analysis of upper digestive system diseases change in prevalence from 1990 to 2021; (B) Decomposition analysis of upper digestive system diseases change in years lived with disability from 1990 to 2021.

Notes: Black dots represent the total change contributed by all three components. A positive value for each component indicates a corresponding positive contribution, and a negative value indicates a corresponding negative contribution.

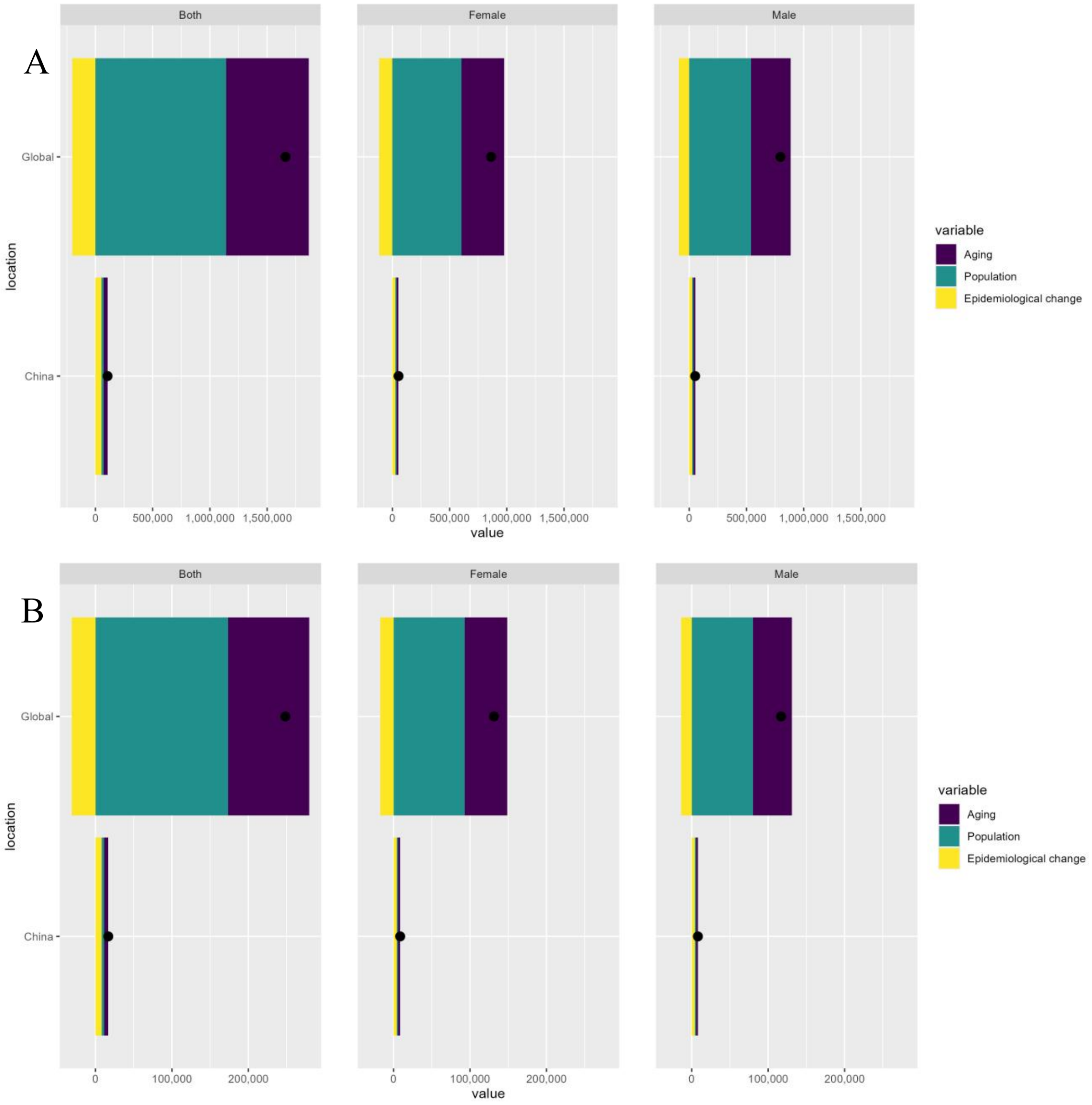

Fig. S129 (A) Decomposition analysis of inflammatory bowel disease change in prevalence from 1990 to 2021; (B) Decomposition analysis of inflammatory bowel disease change in years lived with disability from 1990 to 2021.

Notes: Black dots represent the total change contributed by all three components. A positive value for each component indicates a corresponding positive contribution, and a negative value indicates a corresponding negative contribution.

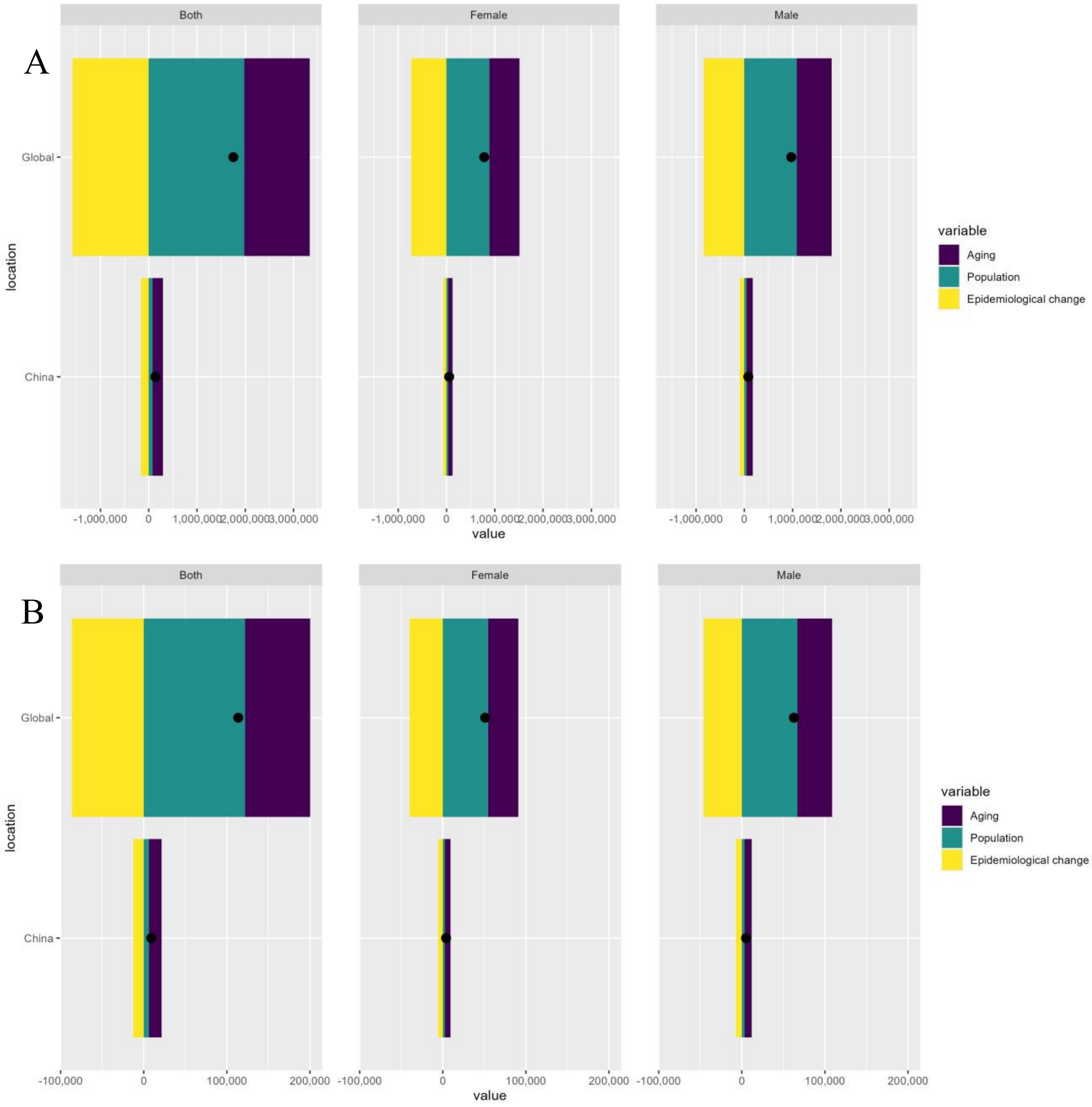

Fig. S130 (A) Decomposition analysis of pancreatitis change in prevalence from 1990 to 2021; (B) Decomposition analysis of pancreatitis change in years lived with disability from 1990 to 2021.

Notes: Black dots represent the total change contributed by all three components. A positive value for each component indicates a corresponding positive contribution, and a negative value indicates a corresponding negative contribution.

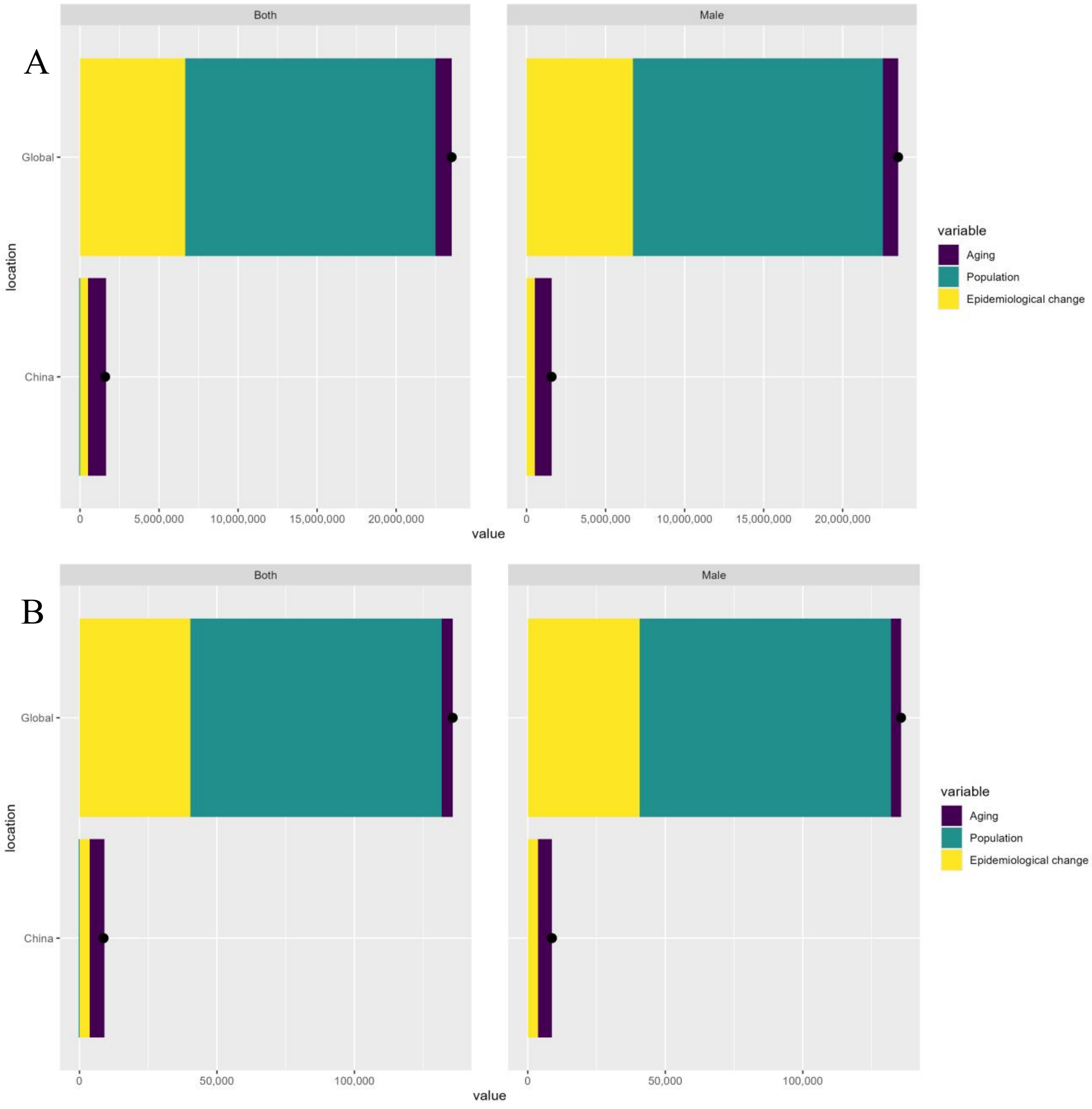

Fig. S131 (A) Decomposition analysis of male infertility change in prevalence from 1990 to 2021; (B) Decomposition analysis of male infertility change in years lived with disability from 1990 to 2021.

Notes: Black dots represent the total change contributed by all three components. A positive value for each component indicates a corresponding positive contribution, and a negative value indicates a corresponding negative contribution.

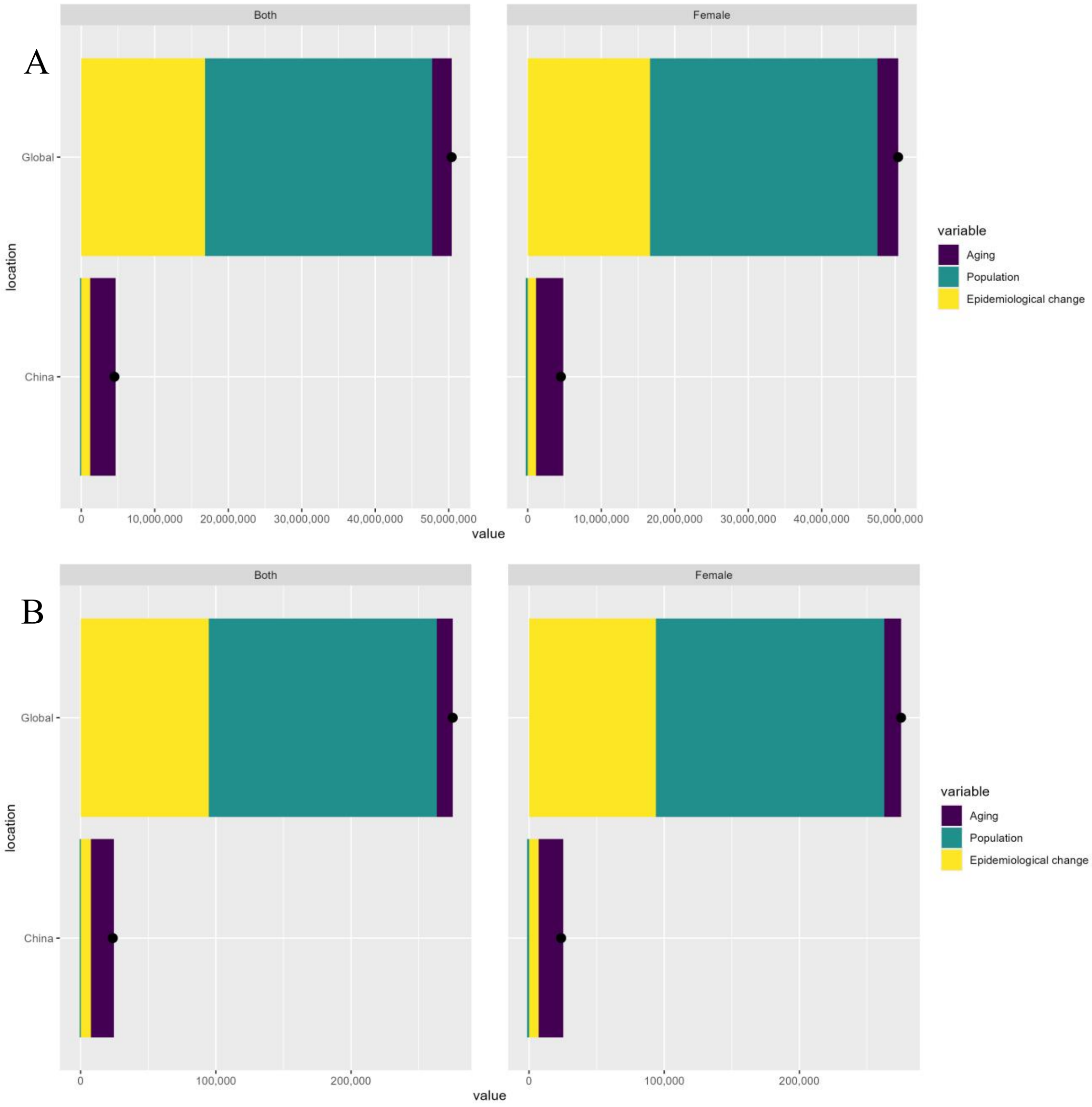

Fig. S132 (A) Decomposition analysis of female infertility change in prevalence from 1990 to 2021; (B) Decomposition analysis of female infertility change in years lived with disability from 1990 to 2021.

Notes: Black dots represent the total change contributed by all three components. A positive value for each component indicates a corresponding positive contribution, and a negative value indicates a corresponding negative contribution.

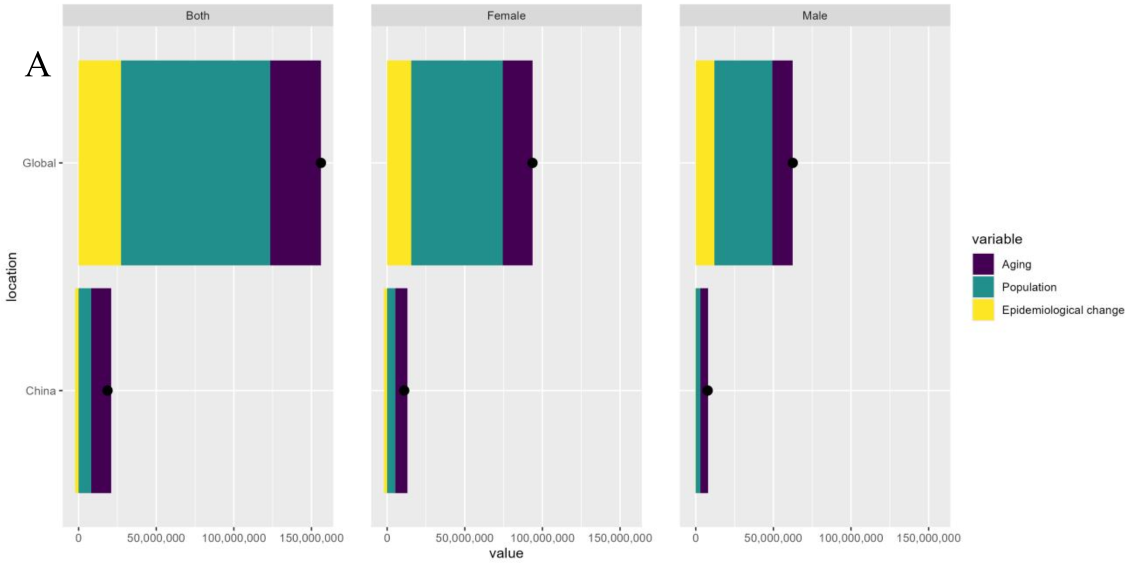

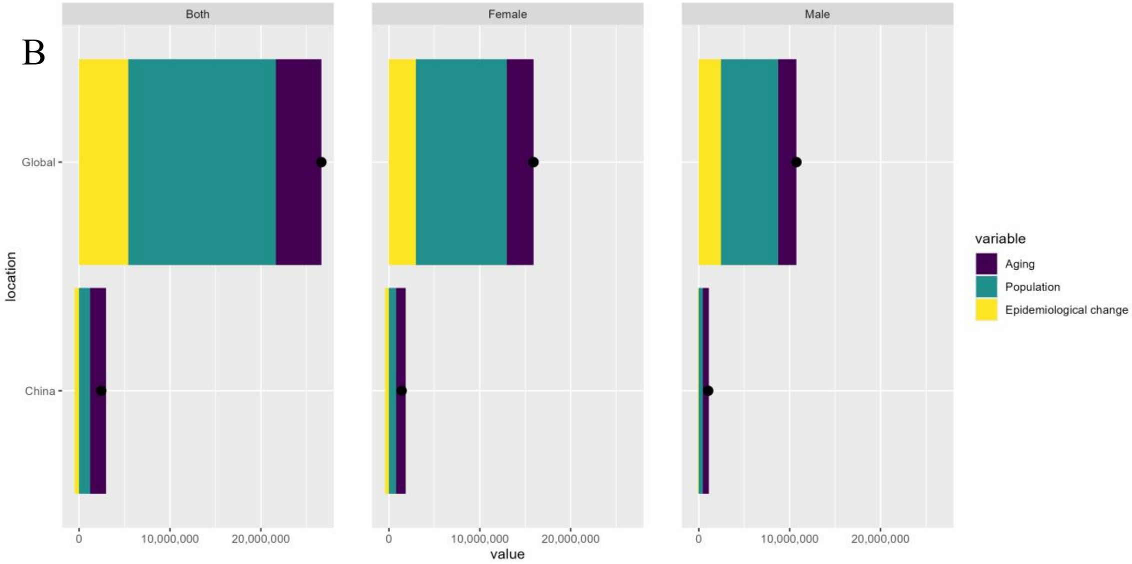

Fig. S133 (A) Decomposition analysis of depressive disorders change in prevalence from 1990 to 2021; (B) Decomposition analysis of depressive disorders change in years lived with disability from 1990 to 2021.

Notes: Black dots represent the total change contributed by all three components. A positive value for each component indicates a corresponding positive contribution, and a negative value indicates a corresponding negative contribution.

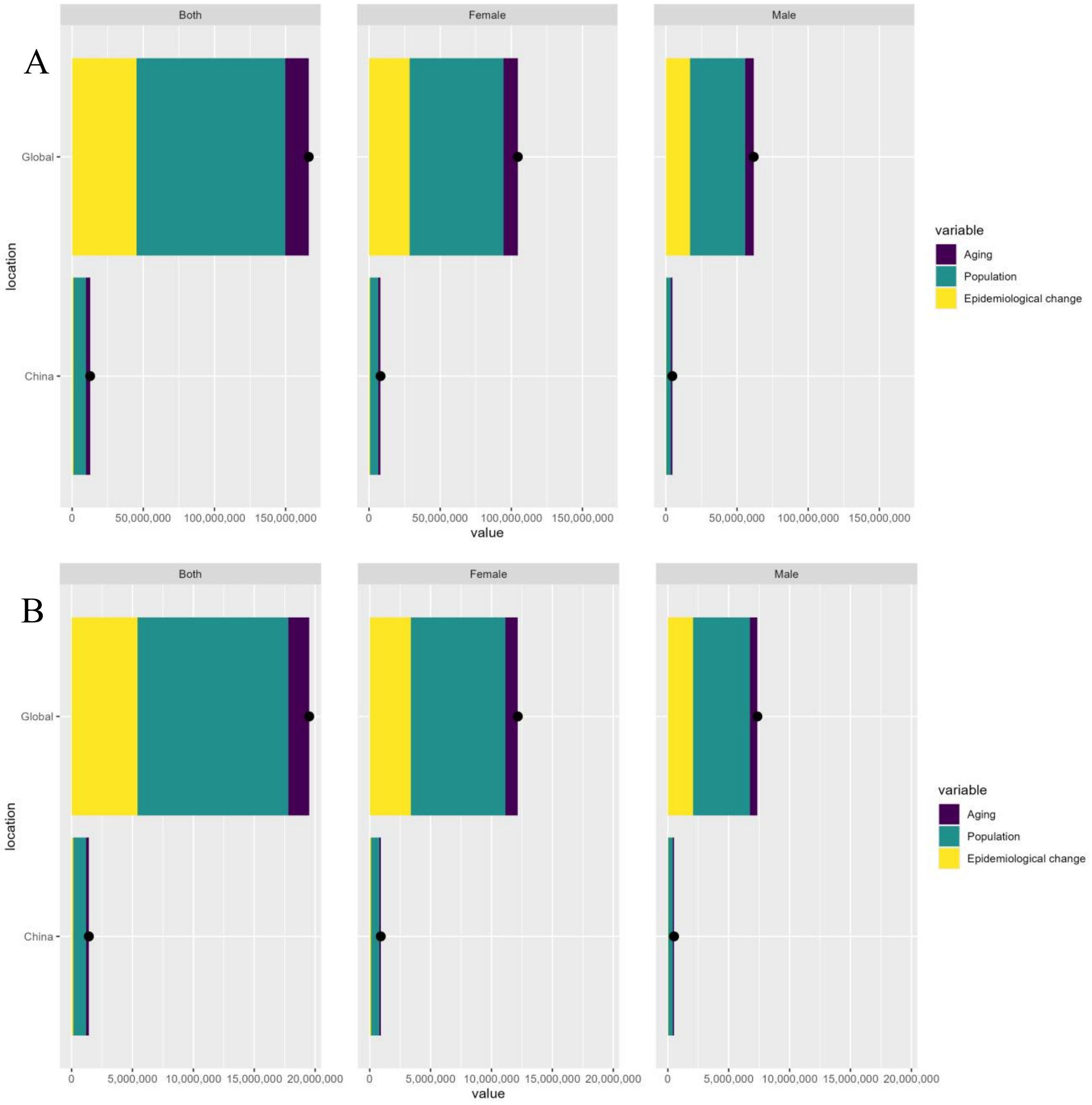

Fig. S134 (A) Decomposition analysis of anxiety disorders change in prevalence from 1990 to 2021; (B) Decomposition analysis of anxiety disorders change in years lived with disability from 1990 to 2021.
Notes: Black dots represent the total change contributed by all three components. A positive value for each component indicates a corresponding positive contribution, and a negative value indicates a corresponding negative contribution.

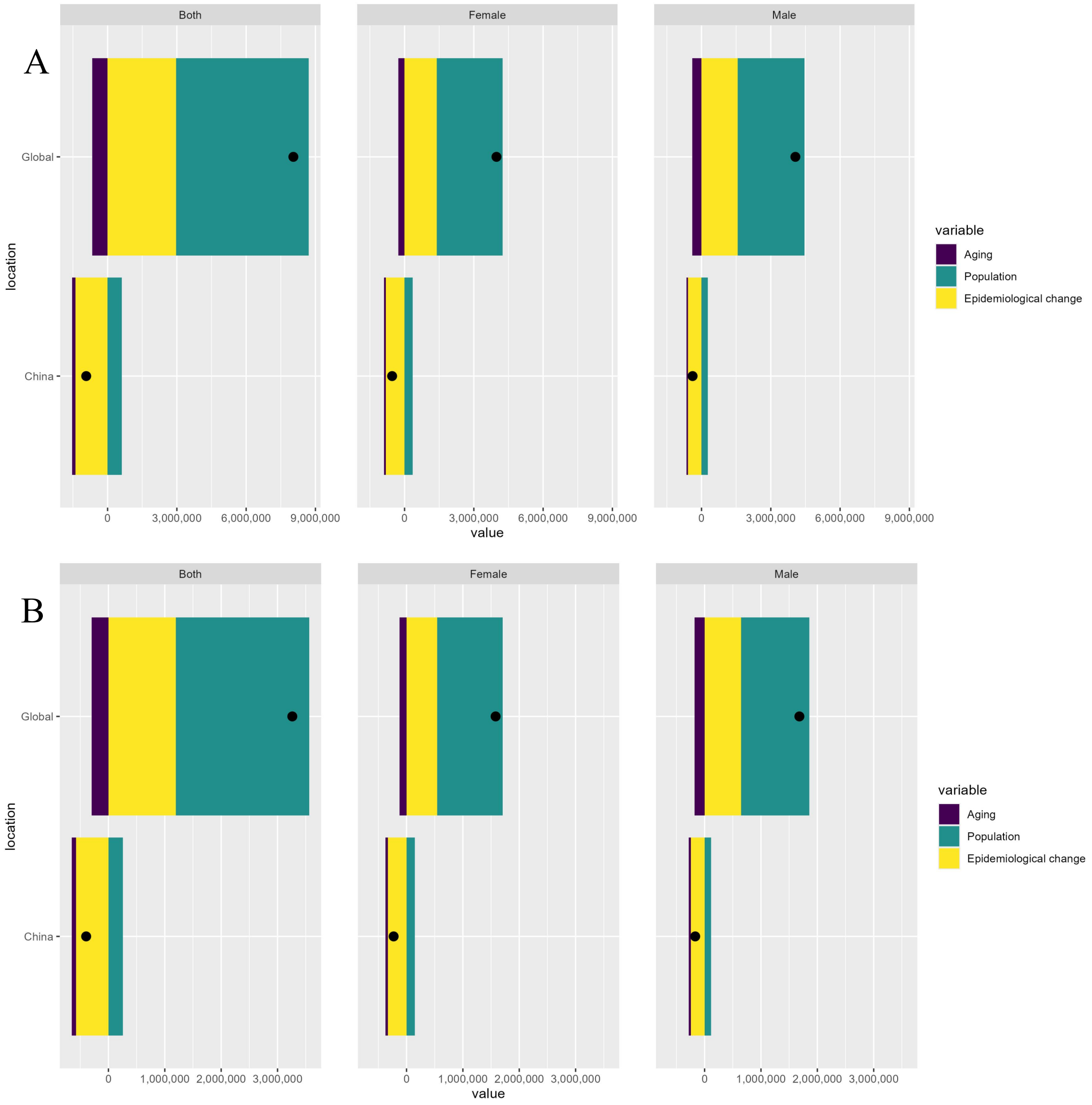

Fig. S135 (A) Decomposition analysis of opioid use disorders change in prevalence from 1990 to 2021; (B) Decomposition analysis of opioid use disorders change in years lived with disability from 1990 to 2021.

Notes: Black dots represent the total change contributed by all three components. A positive value for each component indicates a corresponding positive contribution, and a negative value indicates a corresponding negative contribution.

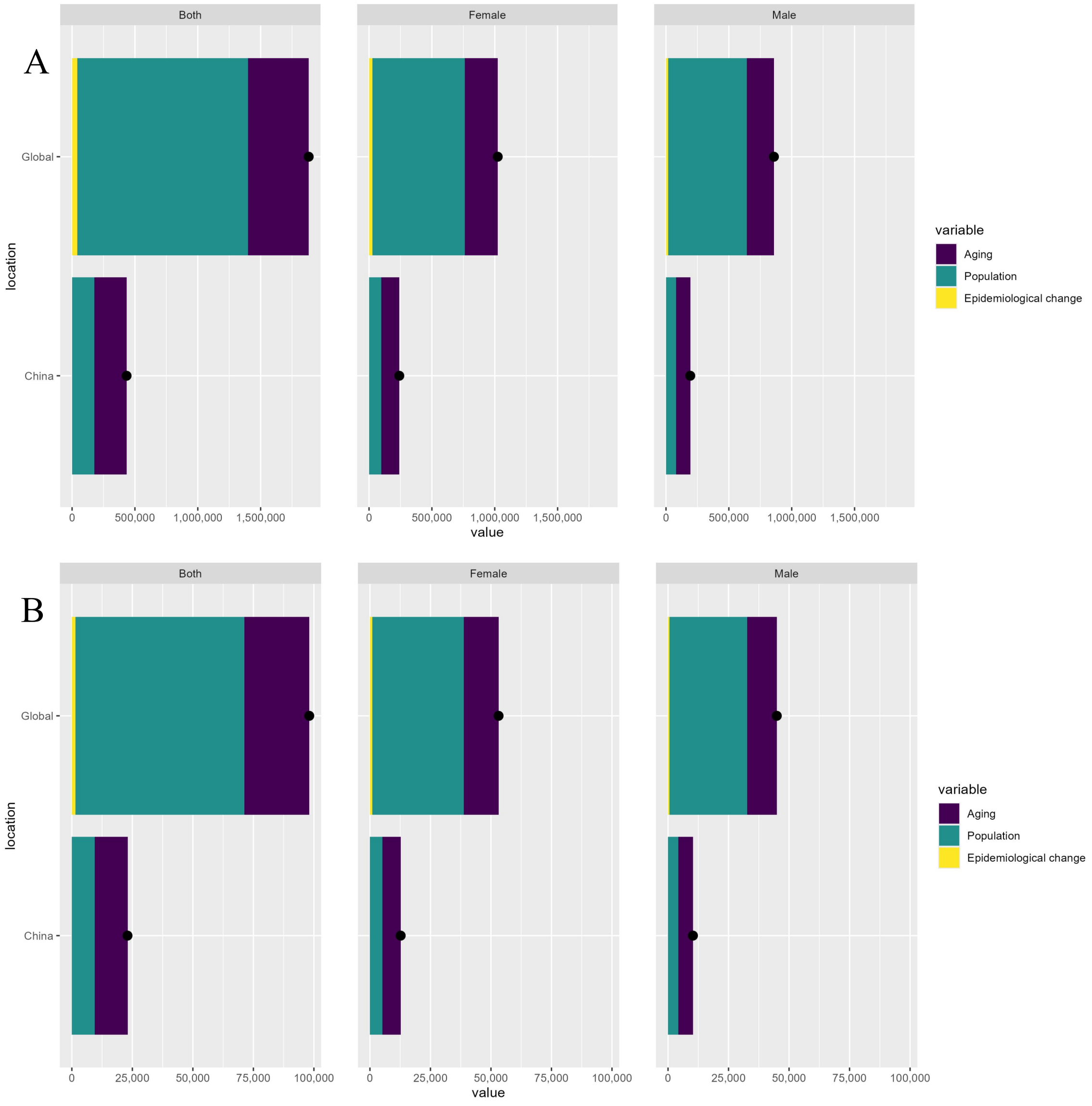

Fig. S136 (A) Decomposition analysis of varicella and herpes zoster change in prevalence from 1990 to 2021; (B) Decomposition analysis of varicella and herpes zoster change in years lived with disability from 1990 to 2021.

Notes: Black dots represent the total change contributed by all three components. A positive value for each component indicates a corresponding positive contribution, and a negative value indicates a corresponding negative contribution.

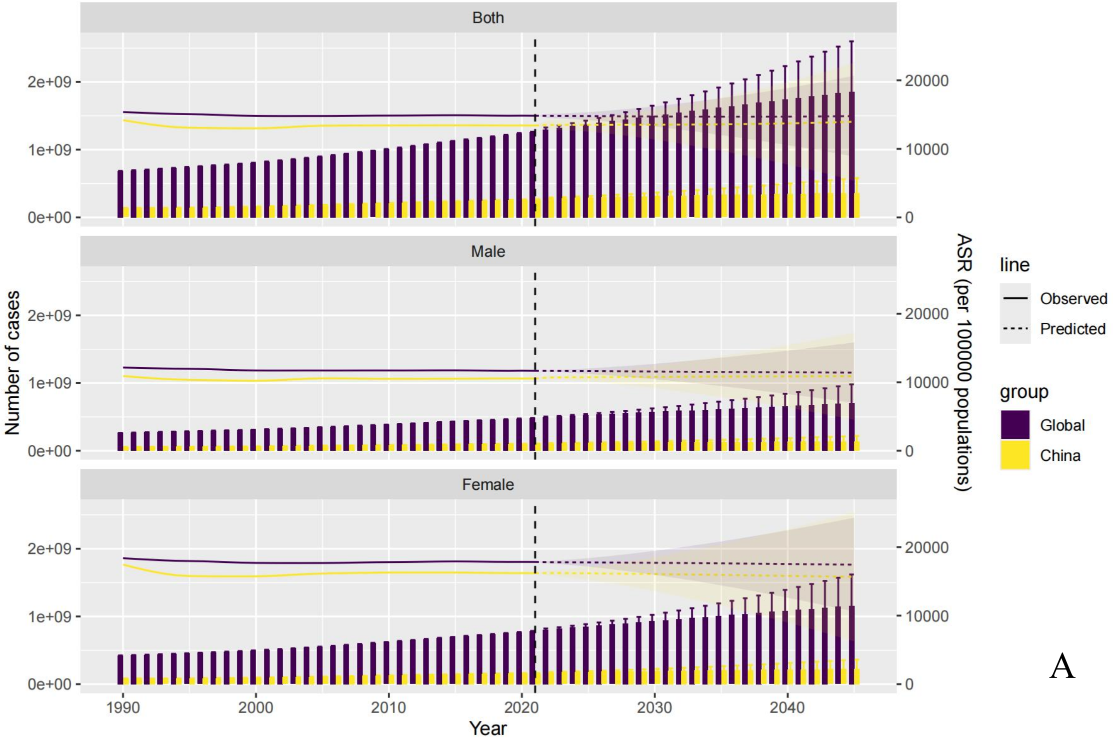

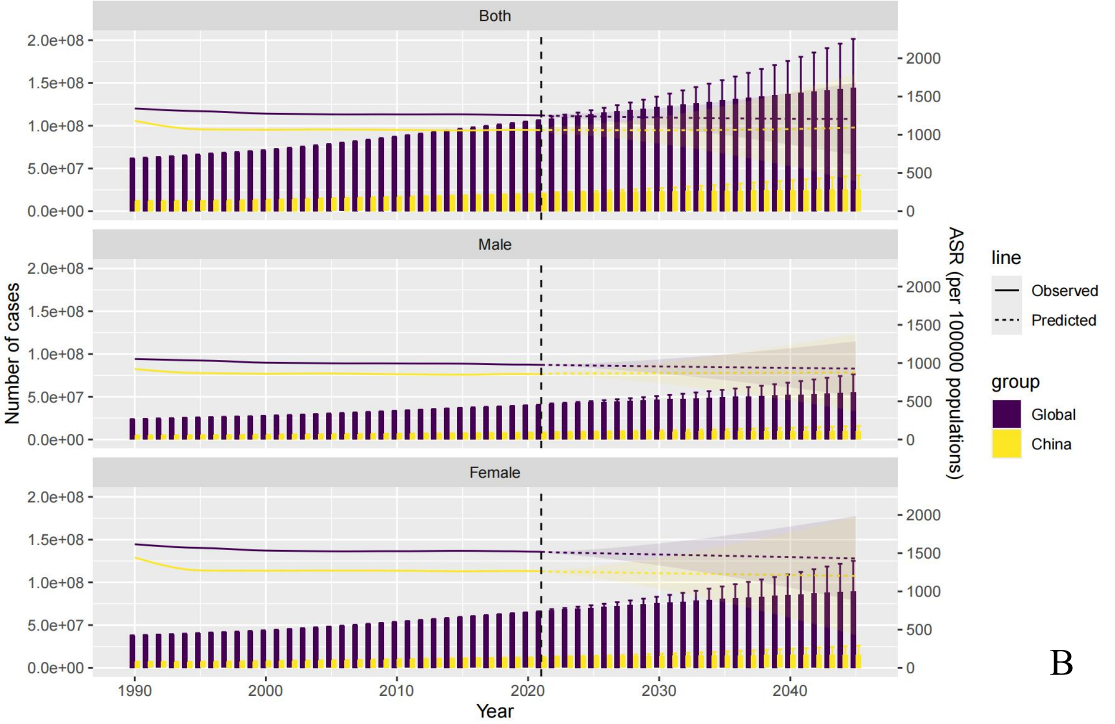

Fig. S137 (A) Future forecasts of Global Burden of Disease in musculoskeletal disorders burden based on prevalence using bayesian age-period-cohort model; (B) Future forecasts of Global Burden of Disease in musculoskeletal disorders burden based on years lived with disability using bayesian age-period-cohort model.

Notes: The line graphs show the change of age-standardized rates (ASR), and the bar graphs show the change of burden.

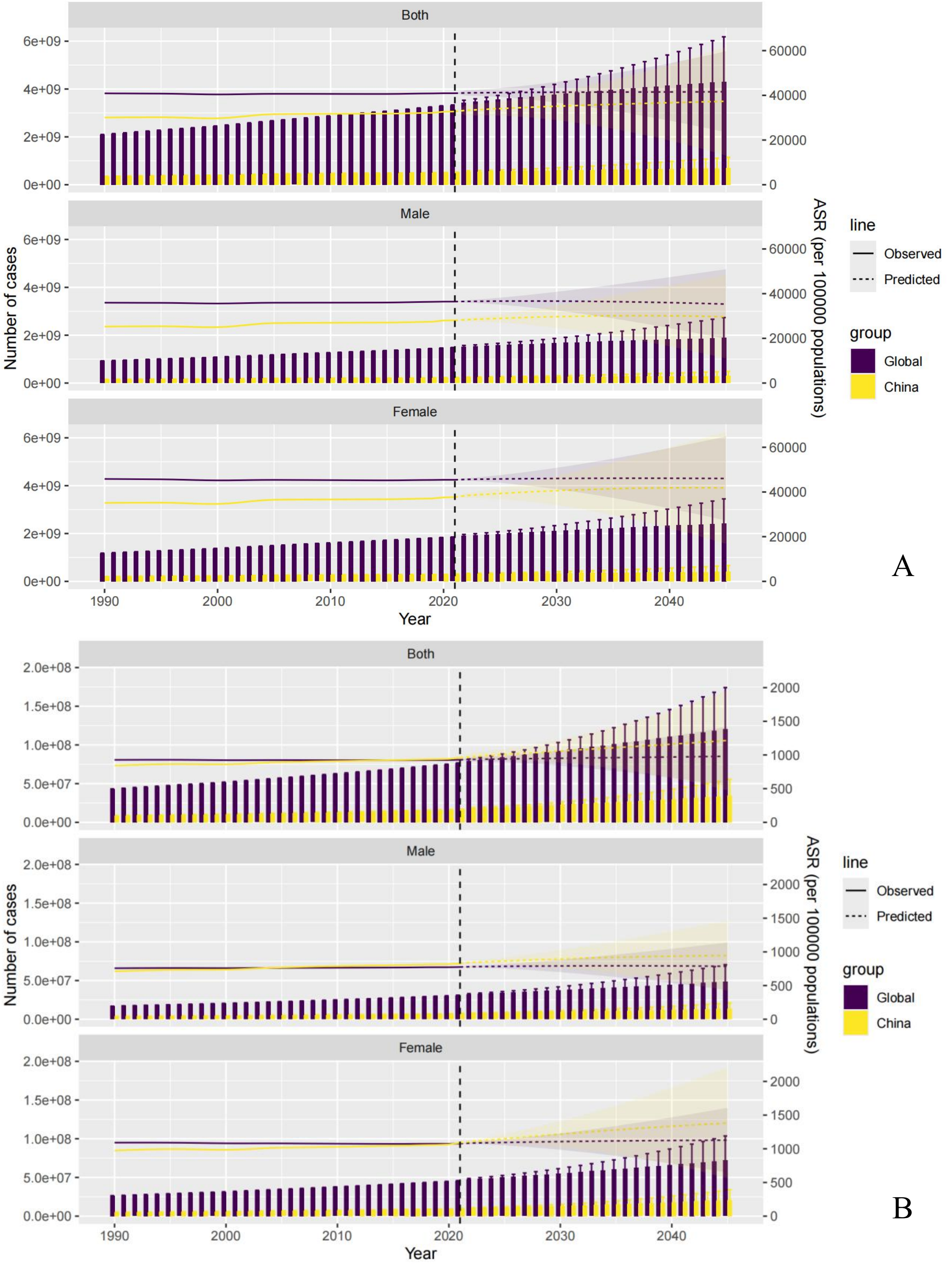

Fig. S138 (A) Future forecasts of Global Burden of Disease in neurological disorders burden based on prevalence using bayesian age-period-cohort model; (B) Future forecasts of Global Burden of Disease in neurological disorders burden based on years lived with disability using bayesian age-period-cohort model.

Notes: The line graphs show the change of age-standardized rates (ASR), and the bar graphs show the change of burden.

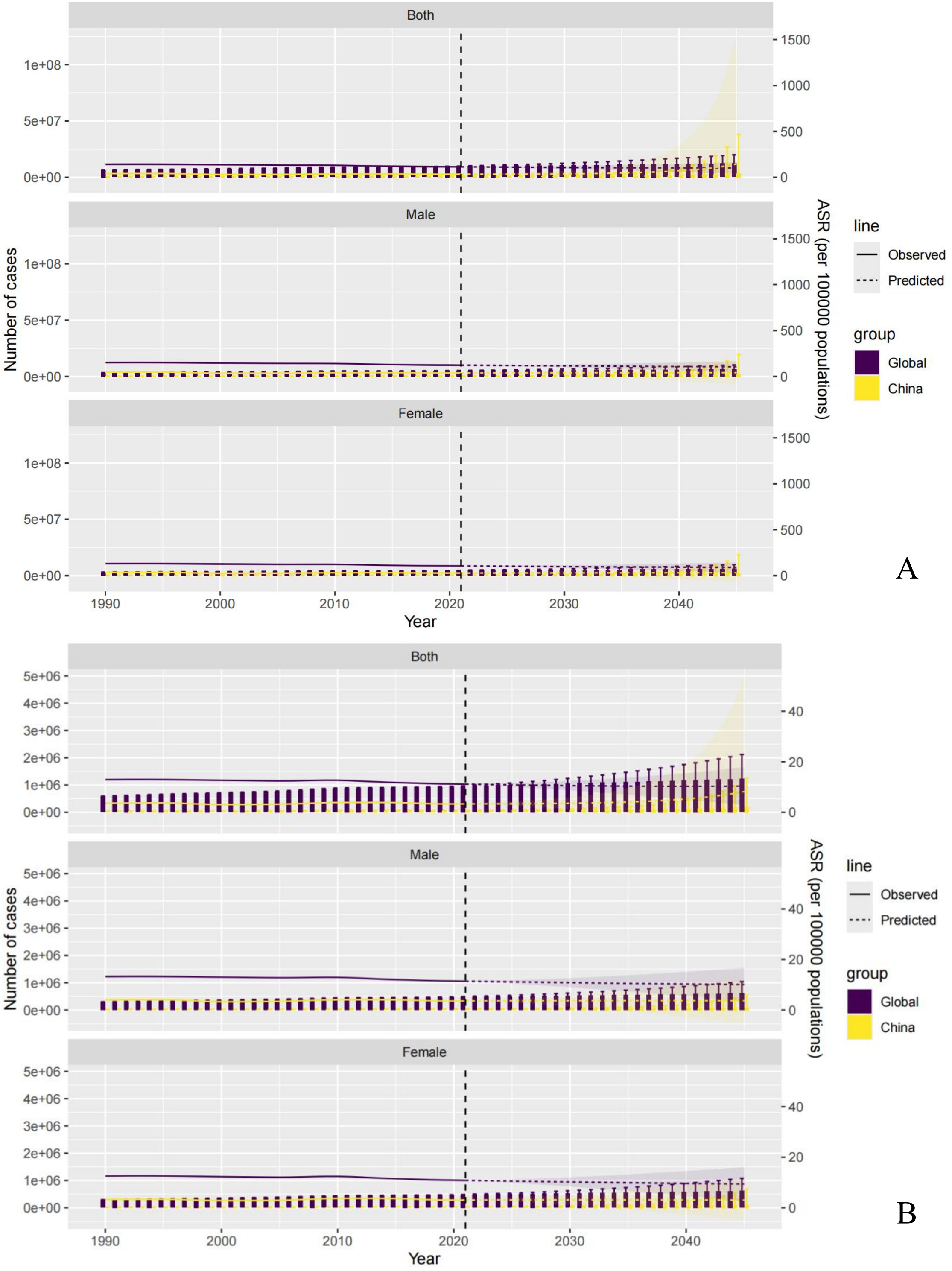

Fig. S139 (A) Future forecasts of Global Burden of Disease in digestive disorders burden based on prevalence using bayesian age-period-cohort model; (B) Future forecasts of Global Burden of Disease in digestive disorders burden based on years lived with disability using bayesian age-period-cohort model.

Notes: The line graphs show the change of age-standardized rates (ASR), and the bar graphs show the change of burden.

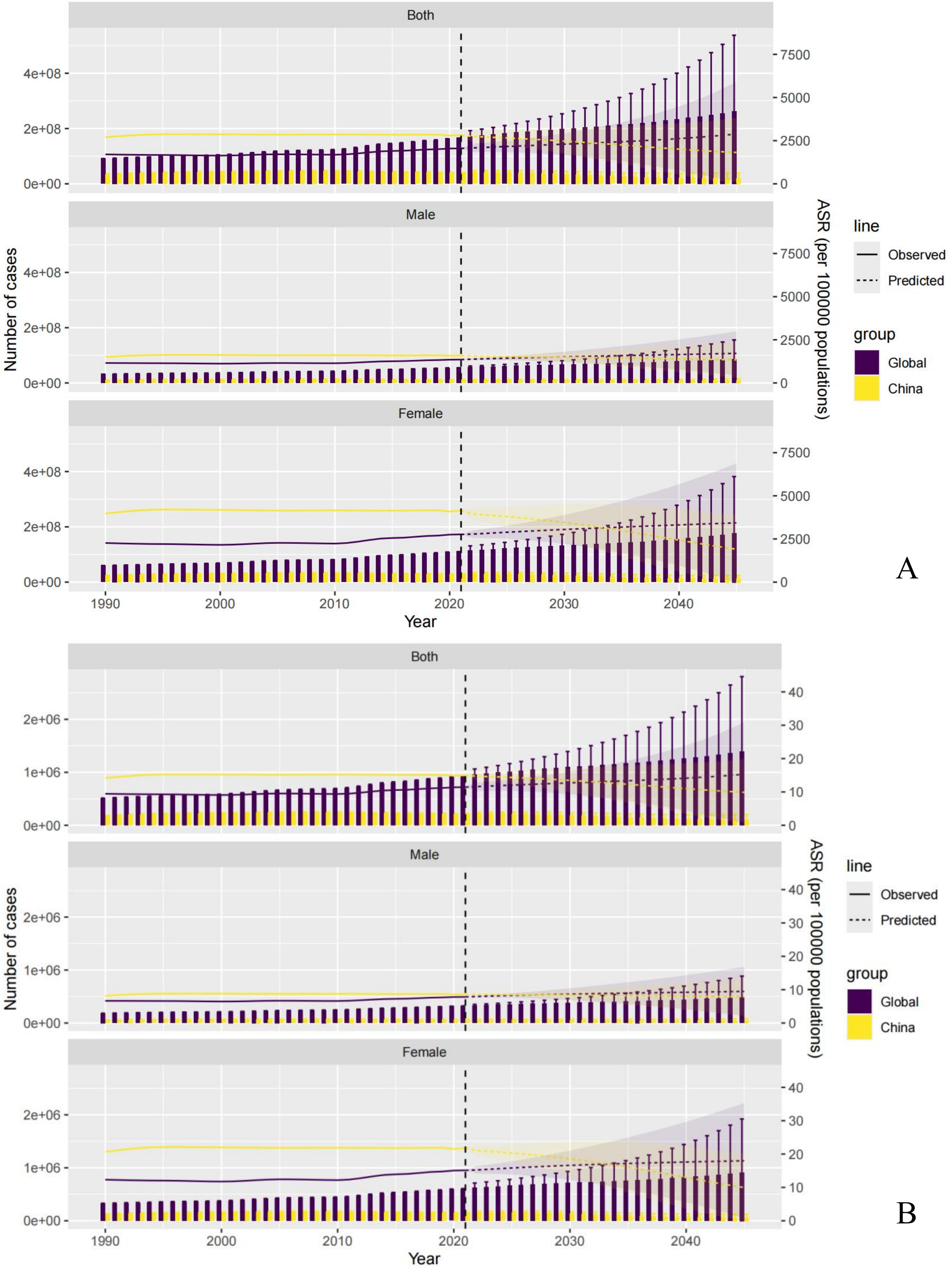

Fig. S140 (A) Future forecasts of Global Burden of Disease in genecological disorders burden based on prevalence using bayesian age-period-cohort model; (B) Future forecasts of Global Burden of Disease in genecological disorders burden based on years lived with disability using bayesian age-period-cohort model.

Notes: The line graphs show the change of age-standardized rates (ASR), and the bar graphs show the change of burden.

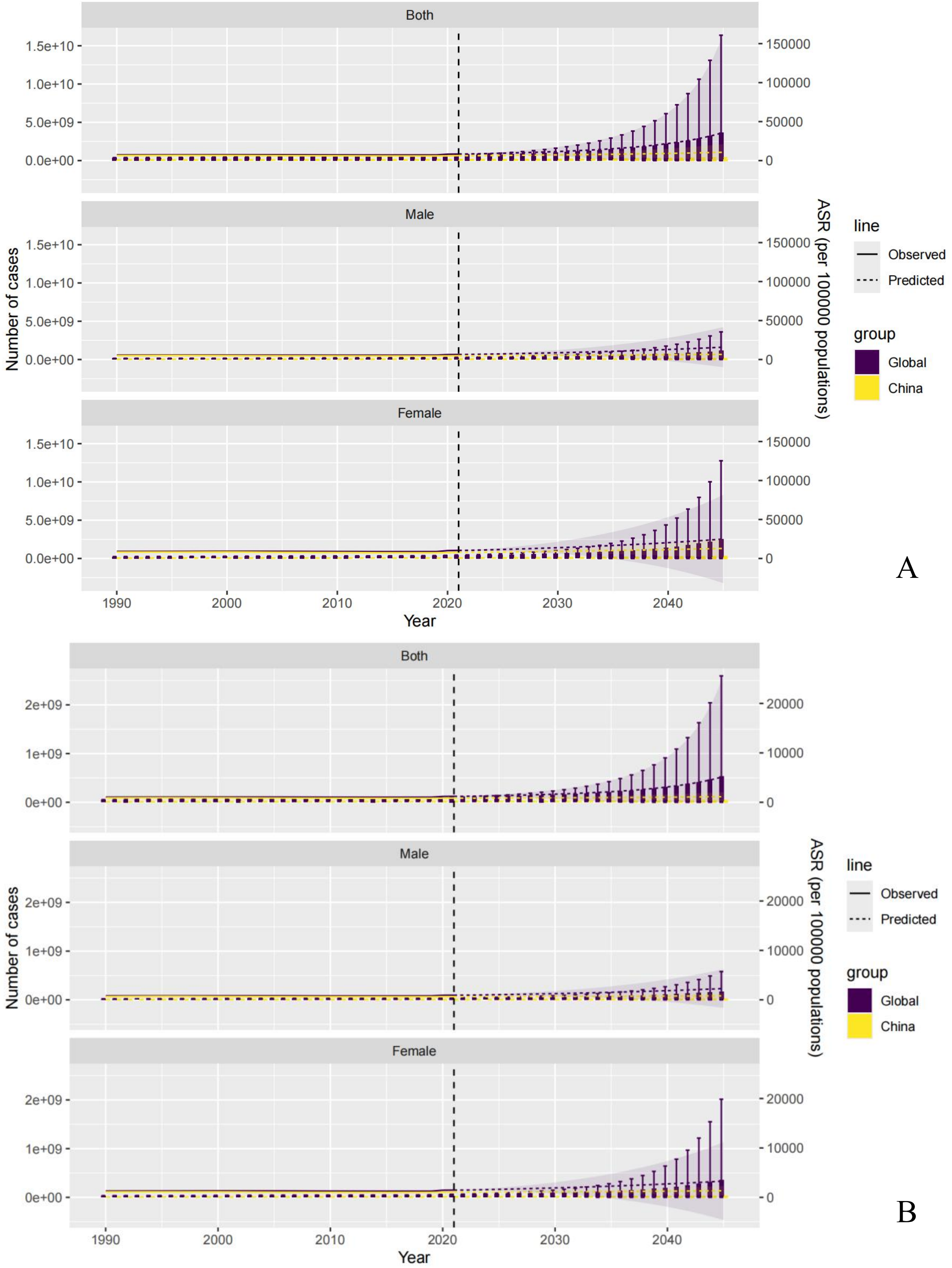

Fig. S141 (A) Future forecasts of Global Burden of Disease in mental disorders burden based on prevalence using bayesian age-period-cohort model; (B) Future forecasts of Global Burden of Disease in mental disorders burden based on years lived with disability using bayesian age-period-cohort model.

Notes: The line graphs show the change of age-standardized rates (ASR), and the bar graphs show the change of burden.

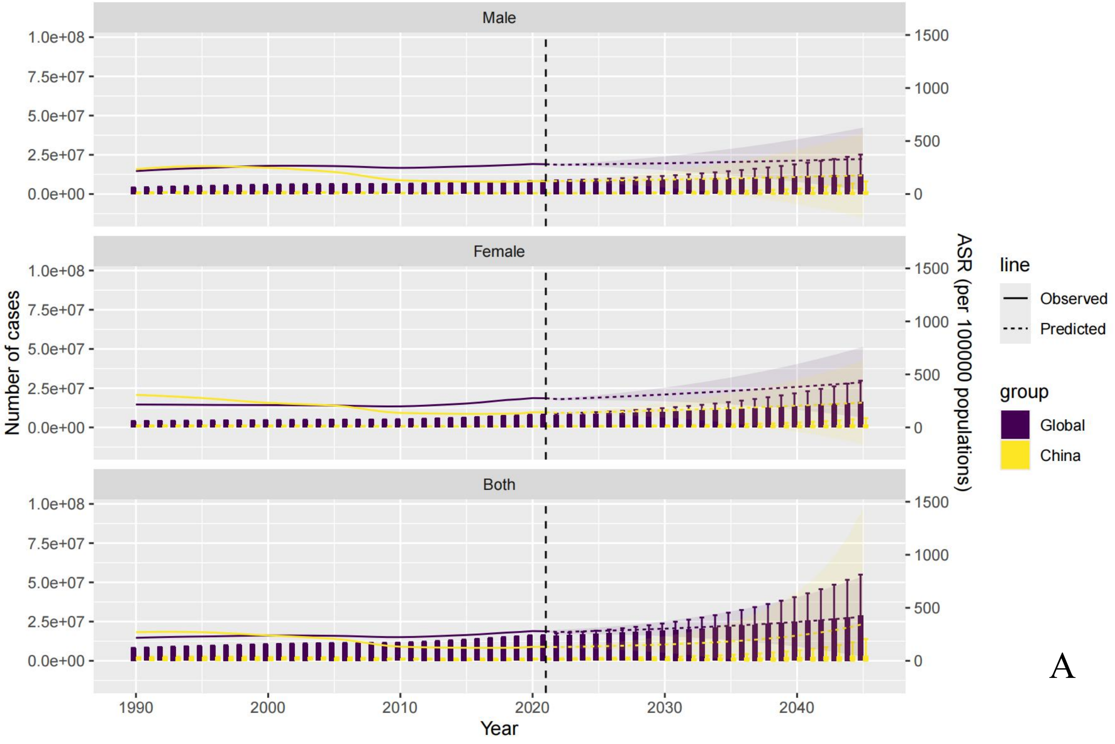

A

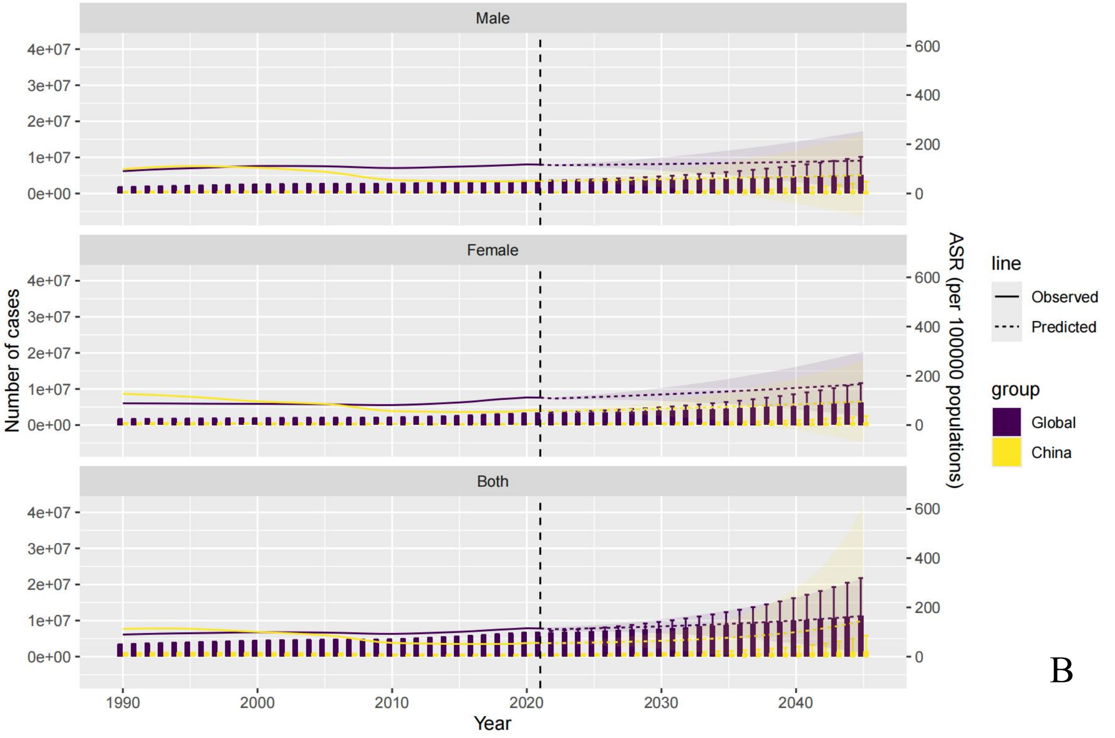

B

Fig. S142 (A) Future forecasts of Global Burden of Disease in substance use disorders burden based on prevalence using bayesian age-period-cohort model; (B) Future forecasts of Global Burden of Disease in substance use disorders burden based on years lived with disability using bayesian age-period-cohort model.

Notes: The line graphs show the change of age-standardized rates (ASR), and the bar graphs show the change of burden.

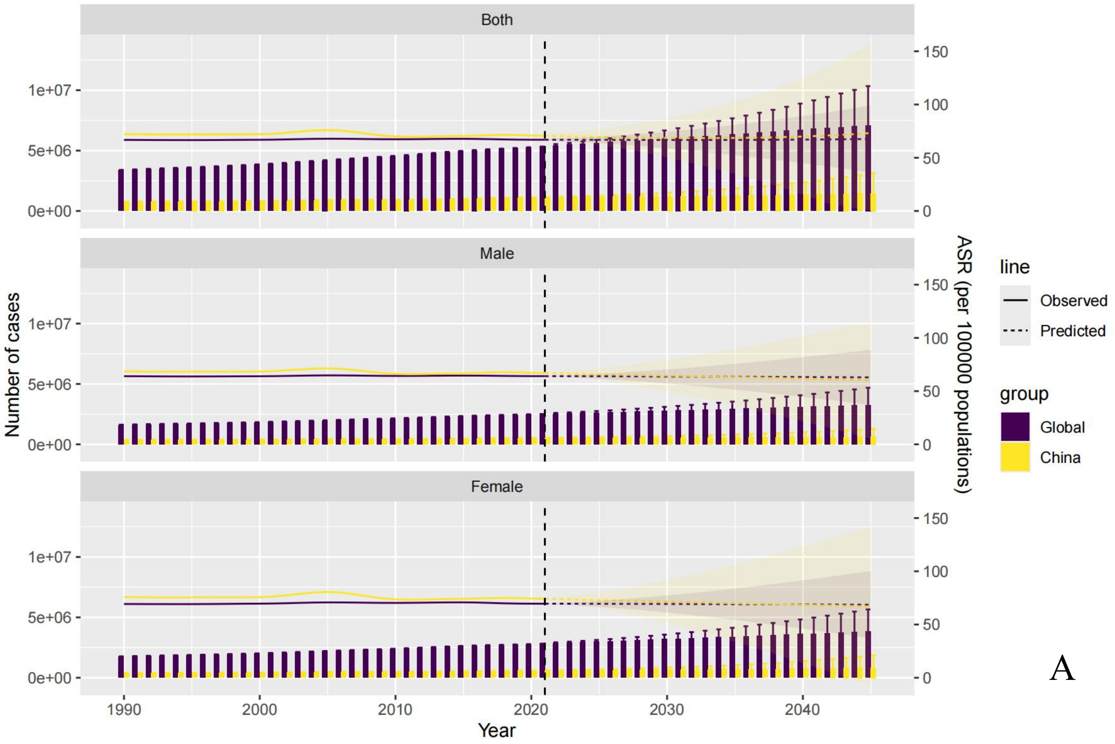

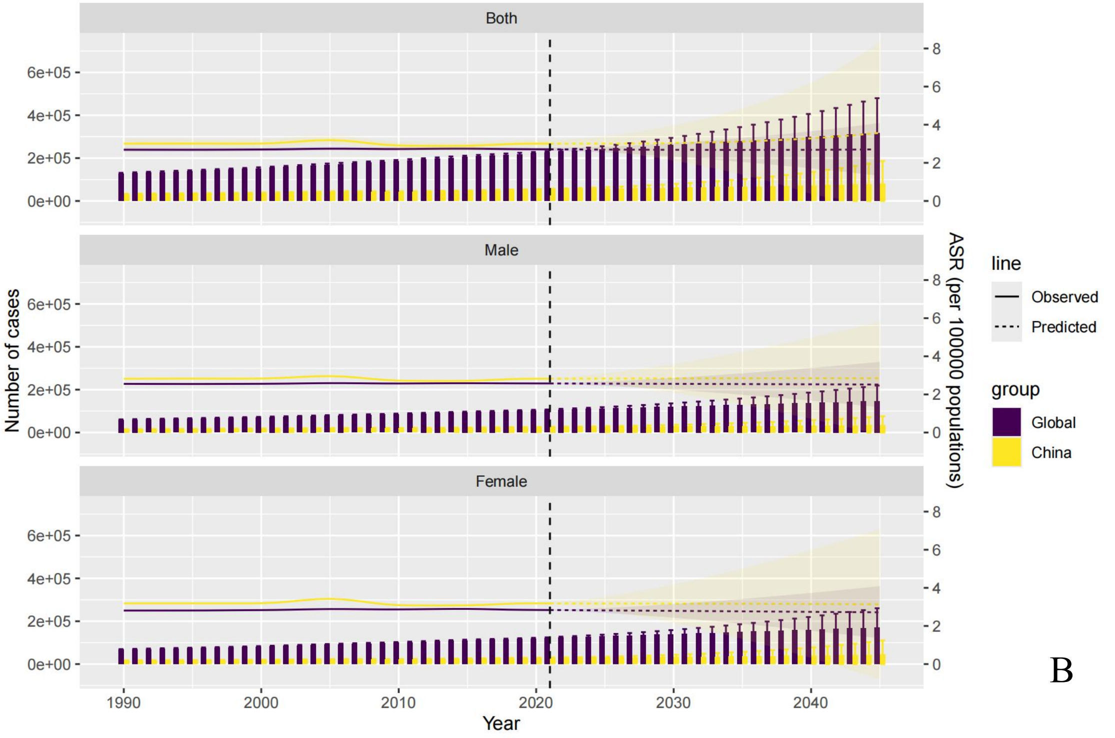

Fig. S143 (A) Future forecasts of Global Burden of Disease in infectious disorders burden based on prevalence using bayesian age-period-cohort model; (B) Future forecasts of Global Burden of Disease in infectious disorders burden based on years lived with disability using bayesian age-period-cohort model.

Notes: The line graphs show the change of age-standardized rates (ASR), and the bar graphs show the change of burden.

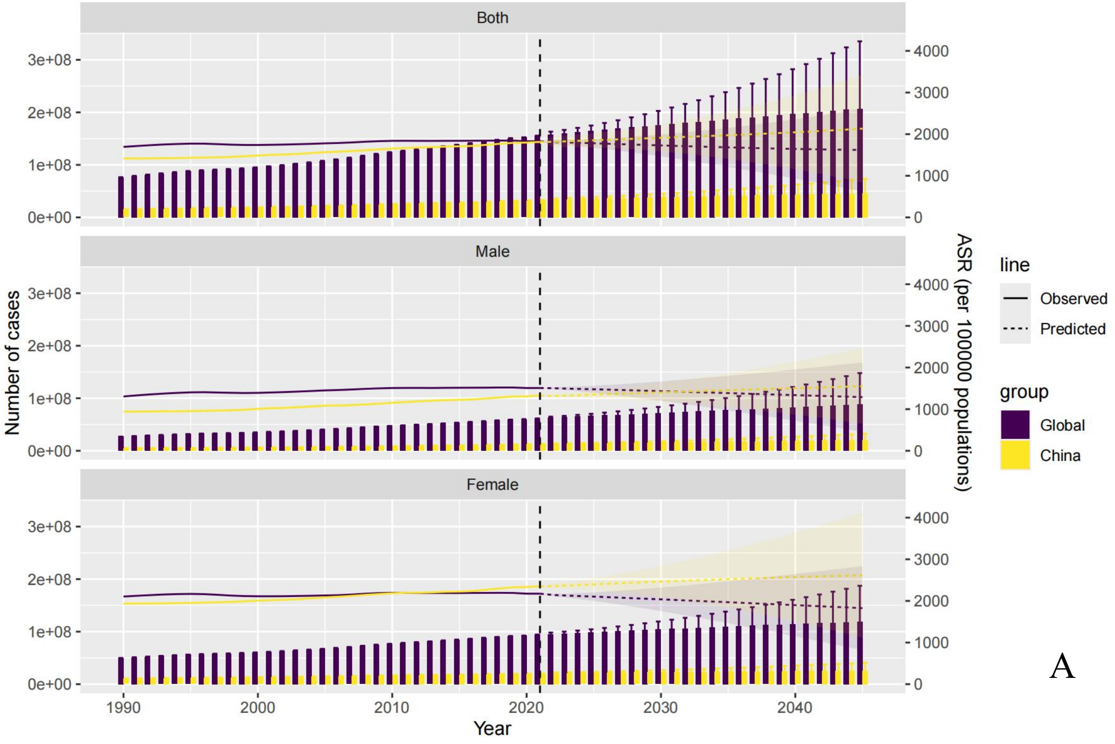

A

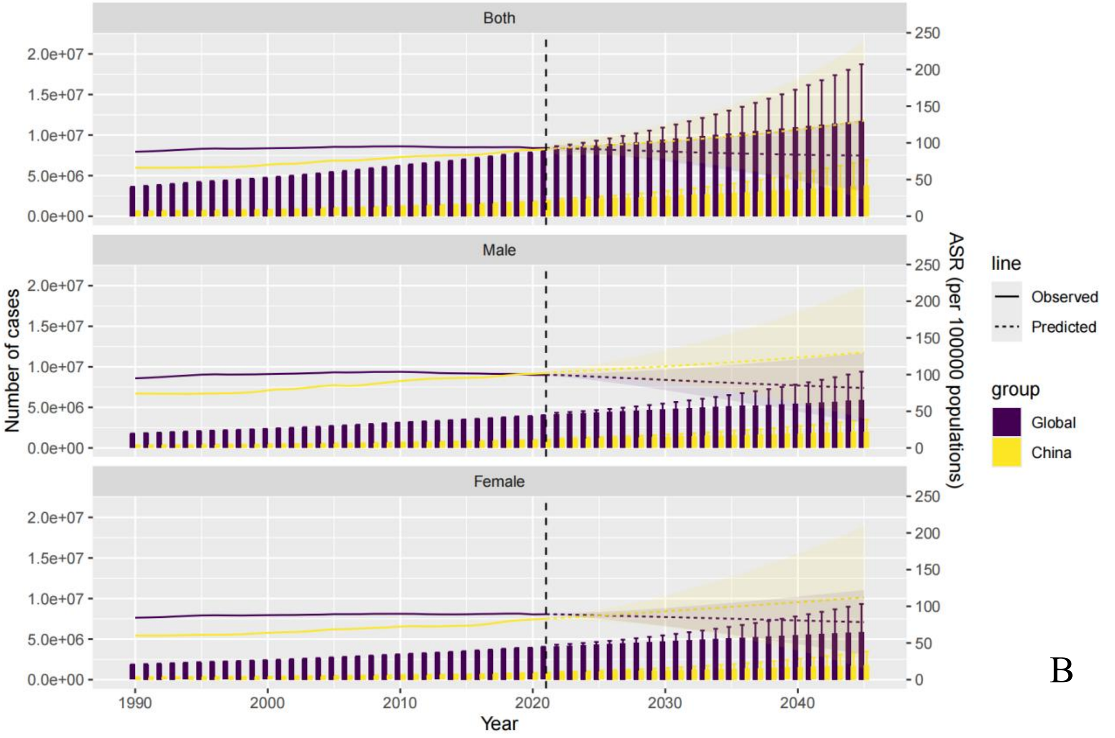

B

Fig. S144 (A) Future forecasts of Global Burden of Disease in neoplasms burden based on prevalence using bayesian age-period-cohort model; (B) Future forecasts of Global Burden of Disease in neoplasms burden based on years lived with disability using bayesian age-period-cohort model.

Notes: The line graphs show the change of age-standardized rates (ASR), and the bar graphs show the change of burden.

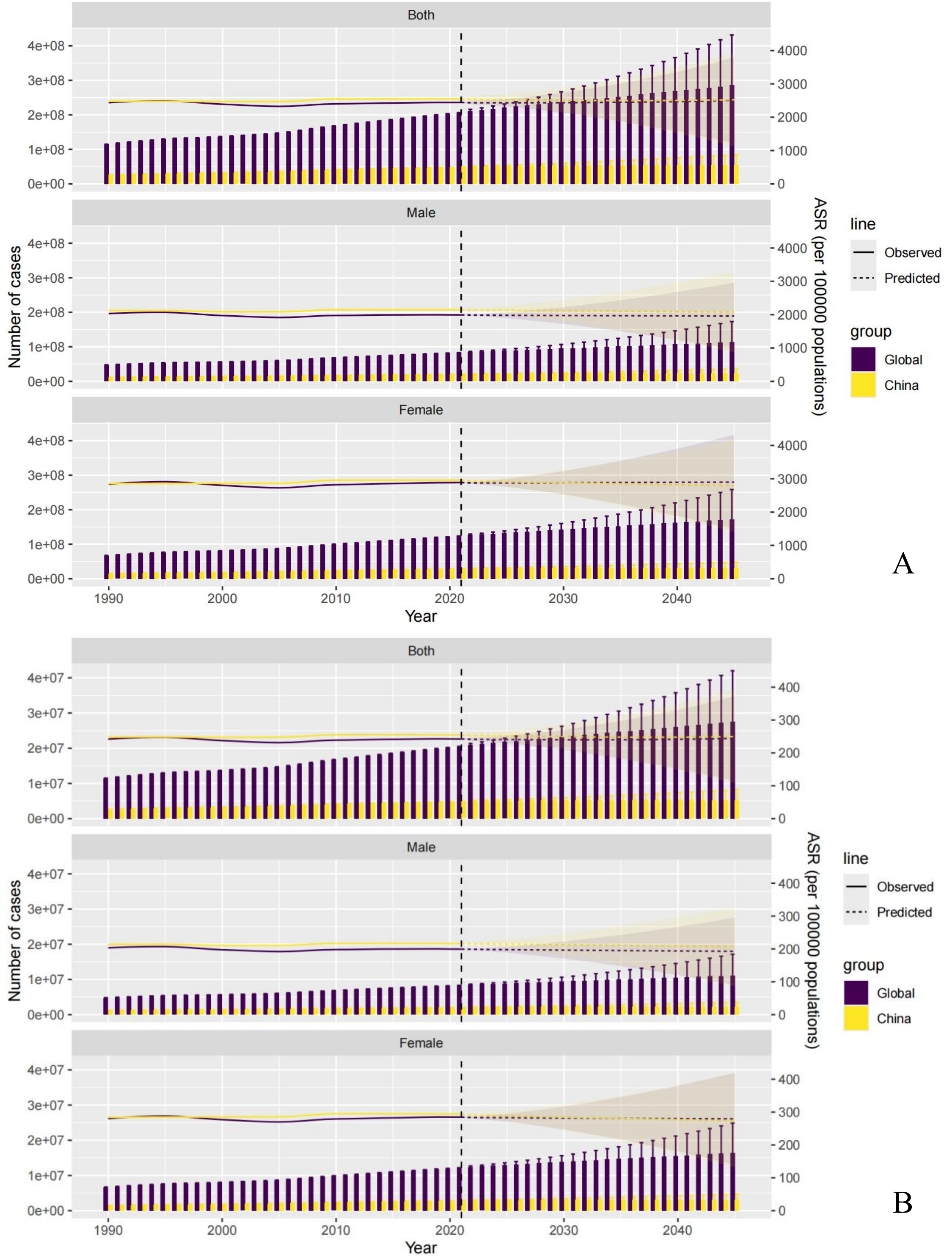

Fig. S145 (A) Future forecasts of Global Burden of Disease in neck pain burden based on prevalence using bayesian age-period-cohort model; (B) Future forecasts of Global Burden of Disease in neck pain burden based on years lived with disability using bayesian age-period-cohort model.

Notes: The line graphs show the change of age-standardized rates (ASR), and the bar graphs show the change of burden.

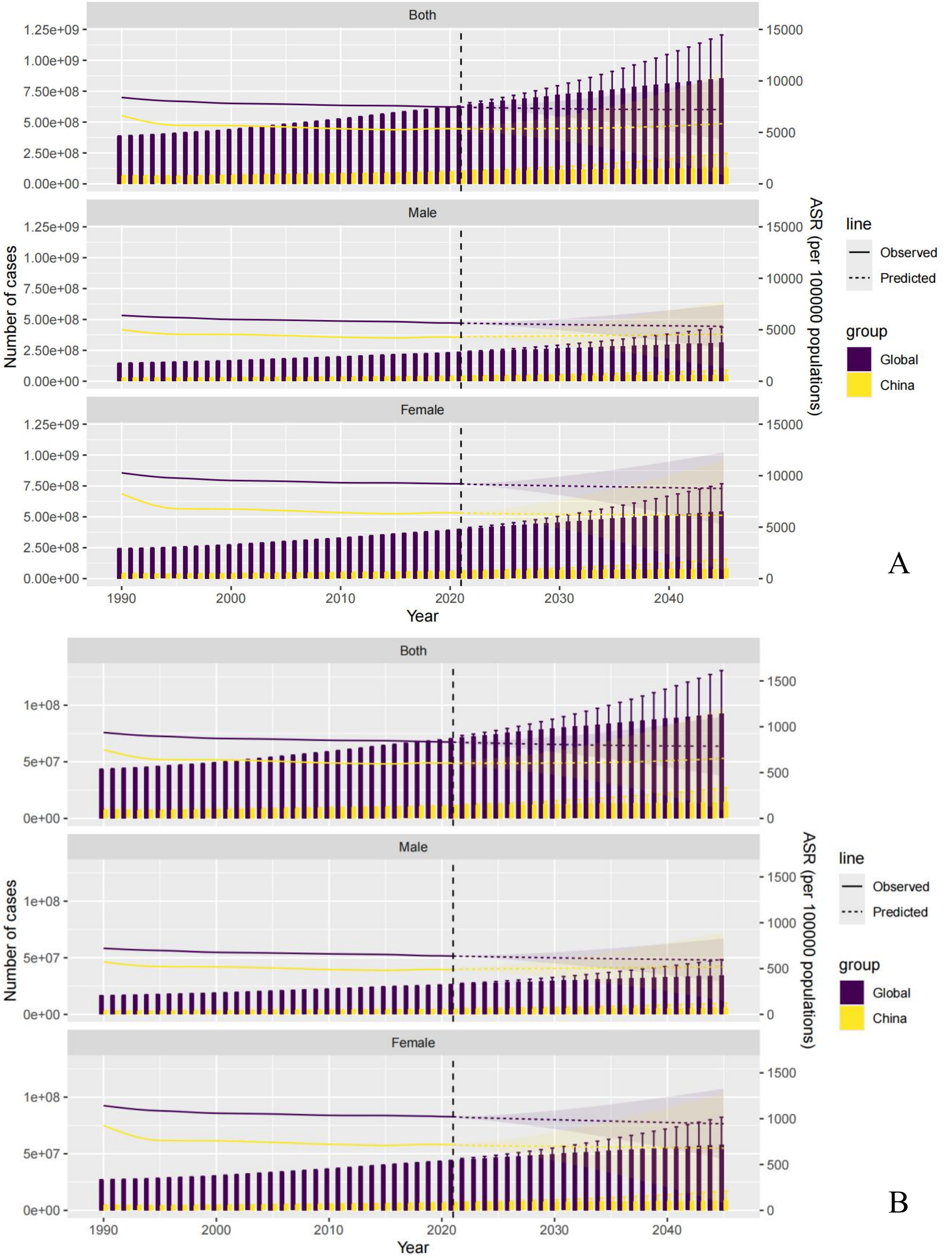

Fig. S146 (A) Future forecasts of Global Burden of Disease in low back pain burden based on prevalence using bayesian age-period-cohort model; (B) Future forecasts of Global Burden of Disease in low back pain burden based on years lived with disability using bayesian age-period-cohort model. Notes: The line graphs show the change of age-standardized rates (ASR), and the bar graphs show the change of burden.

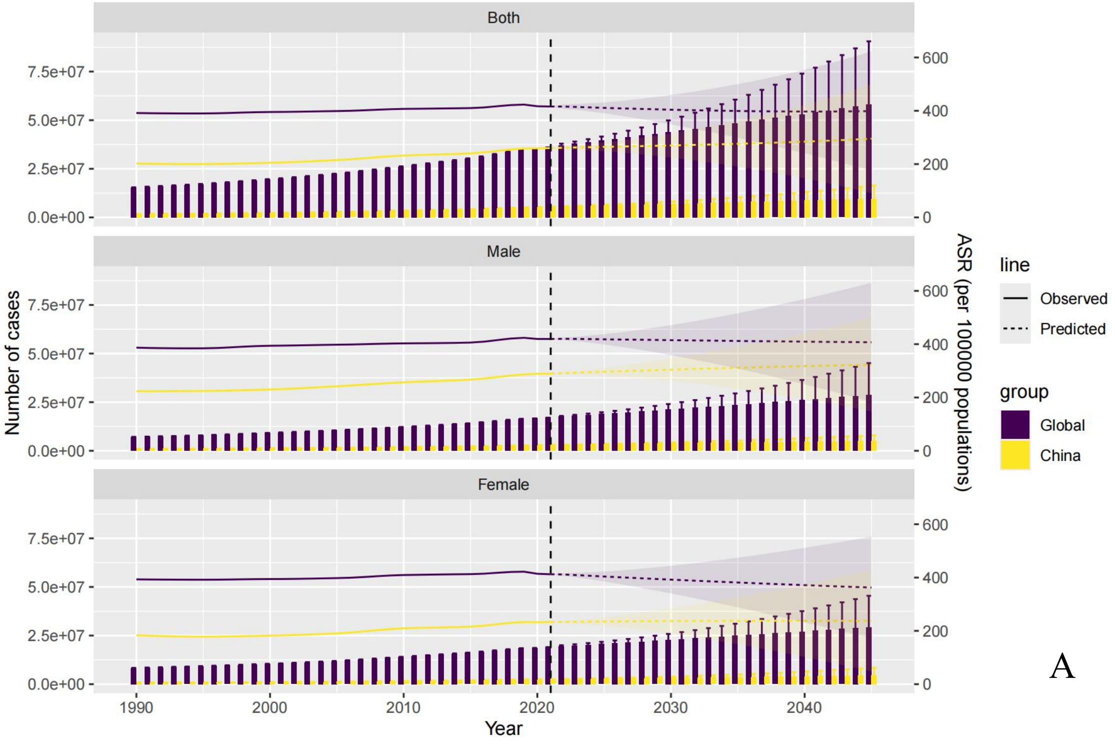

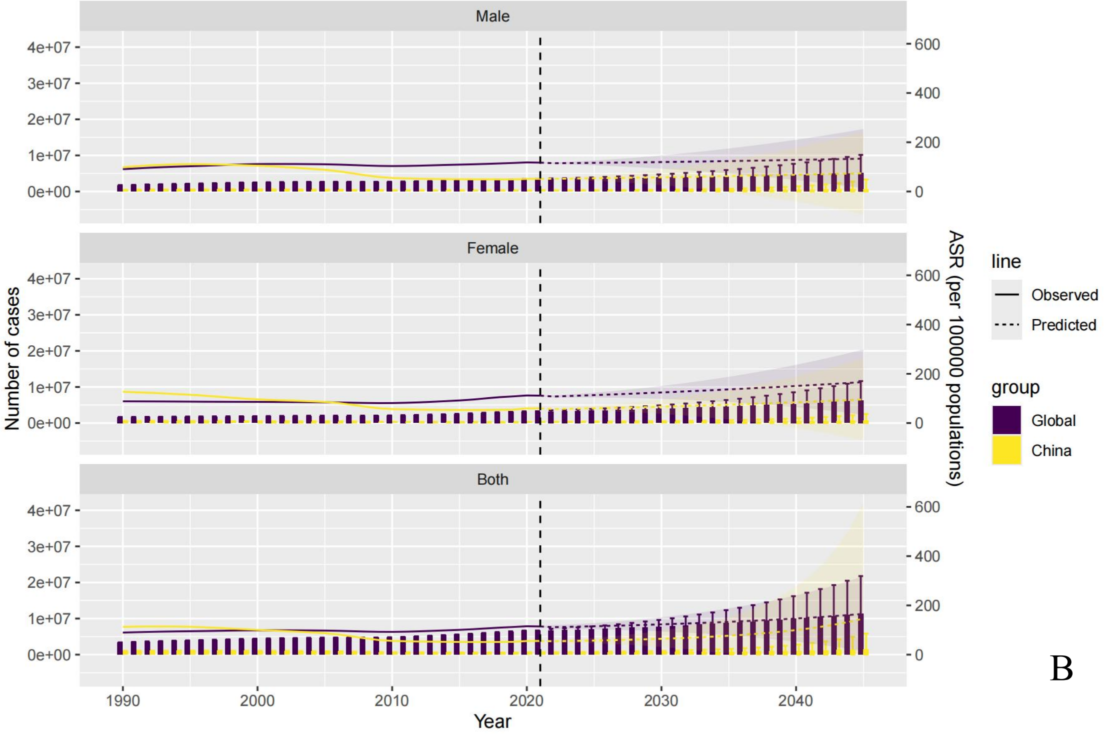

Fig. S147 (A) Future forecasts of Global Burden of Disease in hip osteoarthritis burden based on prevalence using bayesian age-period-cohort model; (B) Future forecasts of Global Burden of Disease in hip osteoarthritis burden based on years lived with disability using bayesian age-period-cohort model.

Notes: The line graphs show the change of age-standardized rates (ASR), and the bar graphs show the change of burden.

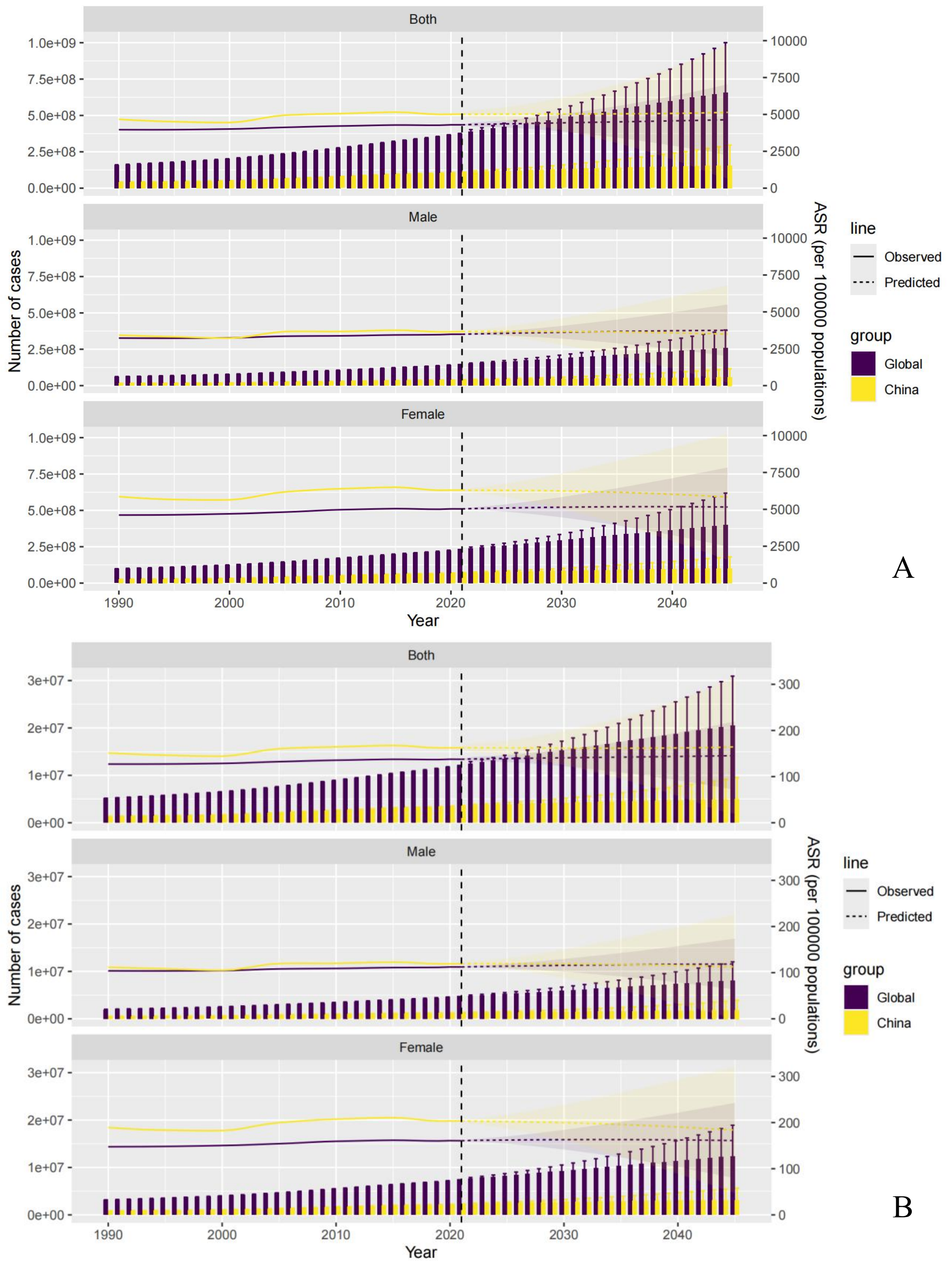

Fig. S148 (A) Future forecasts of Global Burden of Disease in knee osteoarthritis burden based on prevalence using bayesian age-period-cohort model; (B) Future forecasts of Global Burden of Disease in knee osteoarthritis burden based on years lived with disability using bayesian age-period-cohort model.

Notes: The line graphs show the change of age-standardized rates (ASR), and the bar graphs show the change of burden.

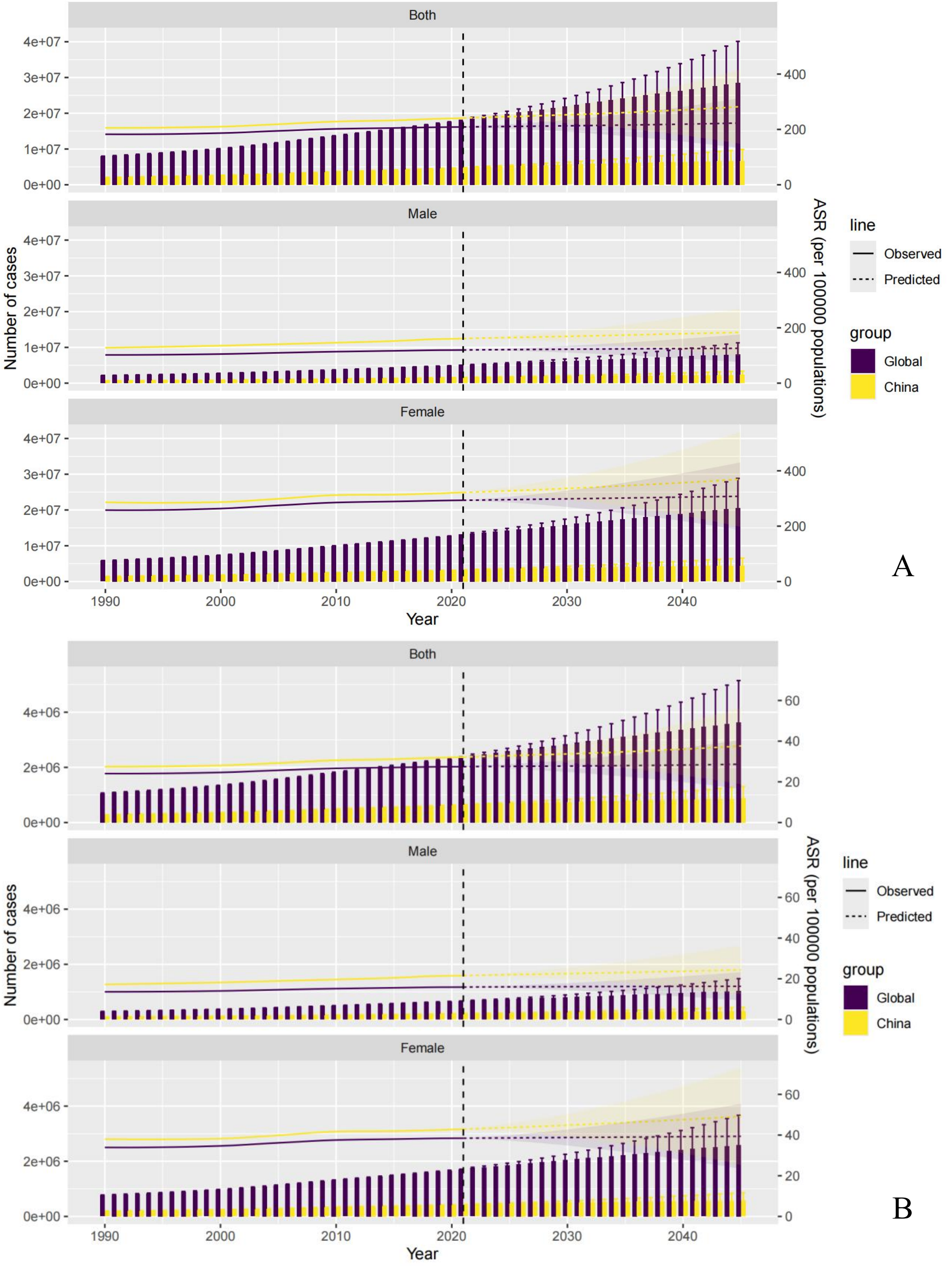

Fig. S149 (A) Future forecasts of Global Burden of Disease in rheumatoid arthritis burden based on prevalence using bayesian age-period-cohort model; (B) Future forecasts of Global Burden of Disease in rheumatoid arthritis burden based on years lived with disability using bayesian age-period-cohort model.

Notes: The line graphs show the change of age-standardized rates (ASR), and the bar graphs show the change of burden.

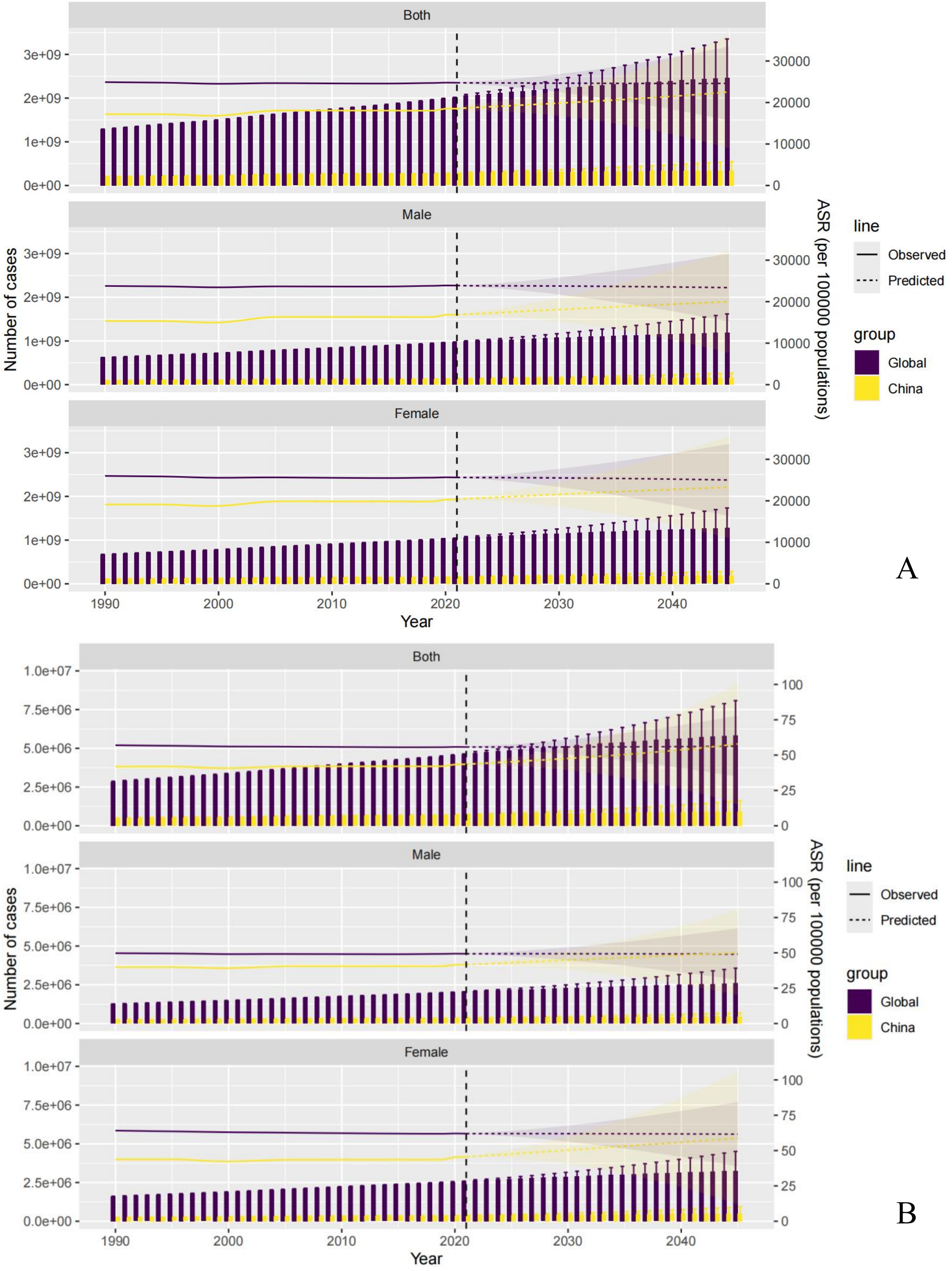

Fig. S150 (A) Future forecasts of Global Burden of Disease in tension-type headache burden based on prevalence using bayesian age-period-cohort model; (B) Future forecasts of Global Burden of Disease in tension-type headache burden based on years lived with disability using bayesian age-period-cohort model.

Notes: The line graphs show the change of age-standardized rates (ASR), and the bar graphs show the change of burden.

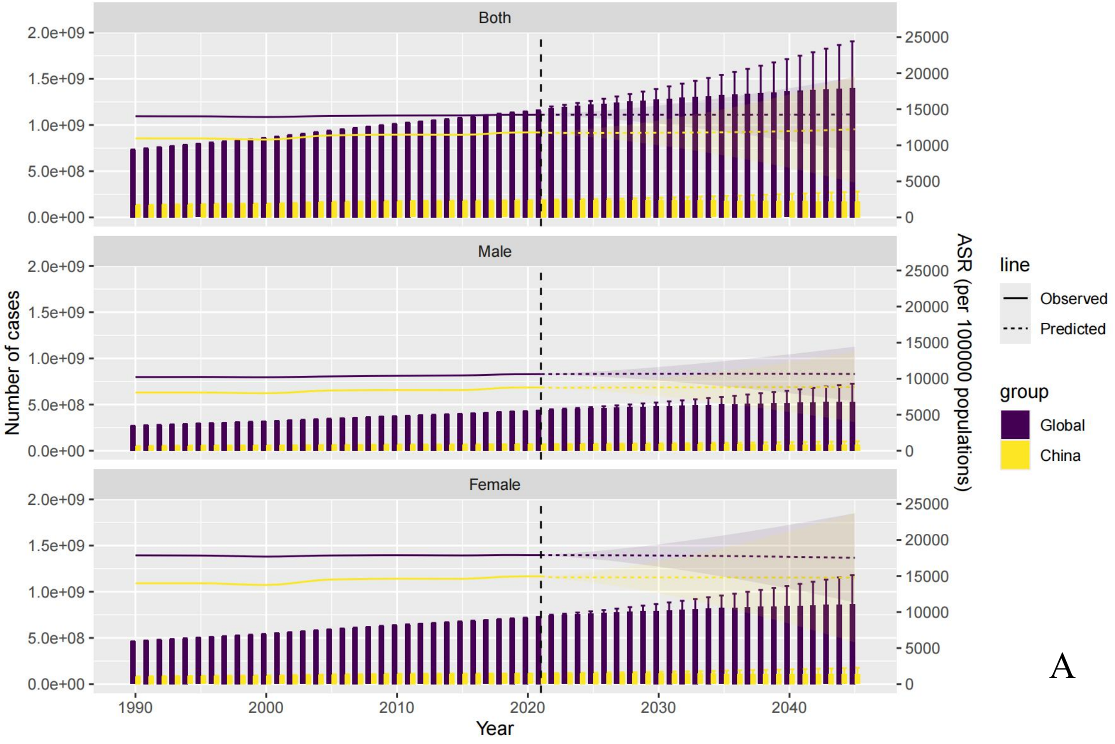

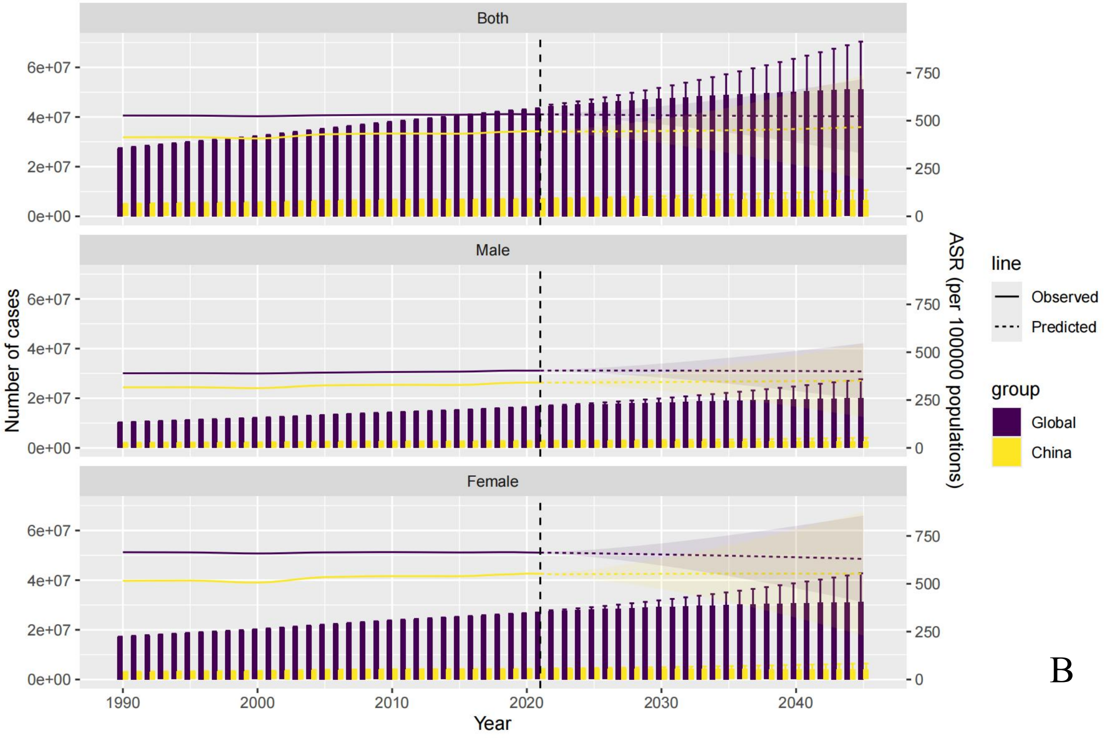

Fig. S151 (A) Future forecasts of Global Burden of Disease in migraine burden based on prevalence using bayesian age-period-cohort model; (B) Future forecasts of Global Burden of Disease in migraine burden based on years lived with disability using bayesian age-period-cohort model.

Notes: The line graphs show the change of age-standardized rates (ASR), and the bar graphs show the change of burden.

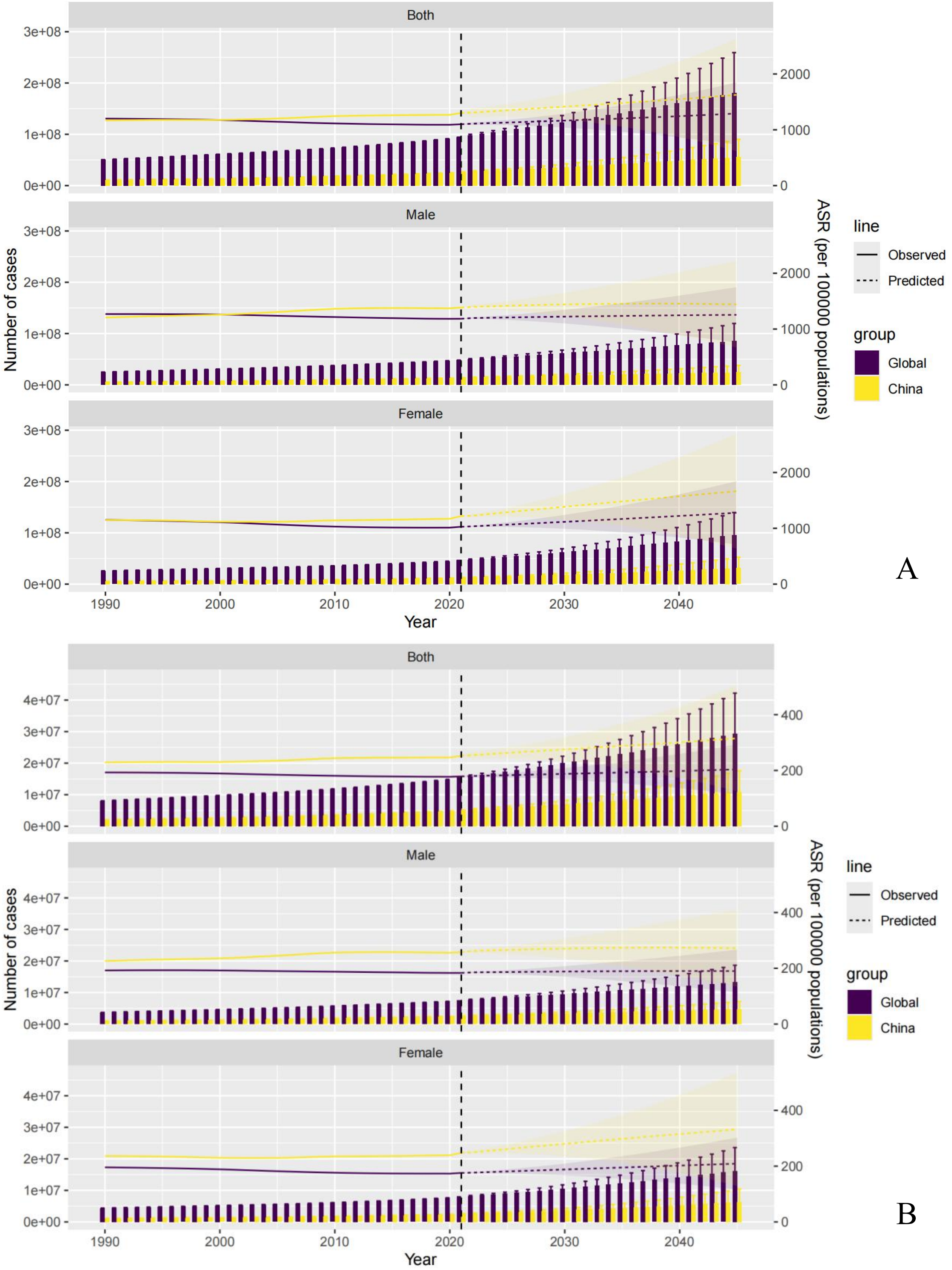

Fig. S152 (A) Future forecasts of Global Burden of Disease in stroke burden based on prevalence using bayesian age-period-cohort model; (B) Future forecasts of Global Burden of Disease in stroke burden based on years lived with disability using bayesian age-period-cohort model.

Notes: The line graphs show the change of age-standardized rates (ASR), and the bar graphs show the change of burden.

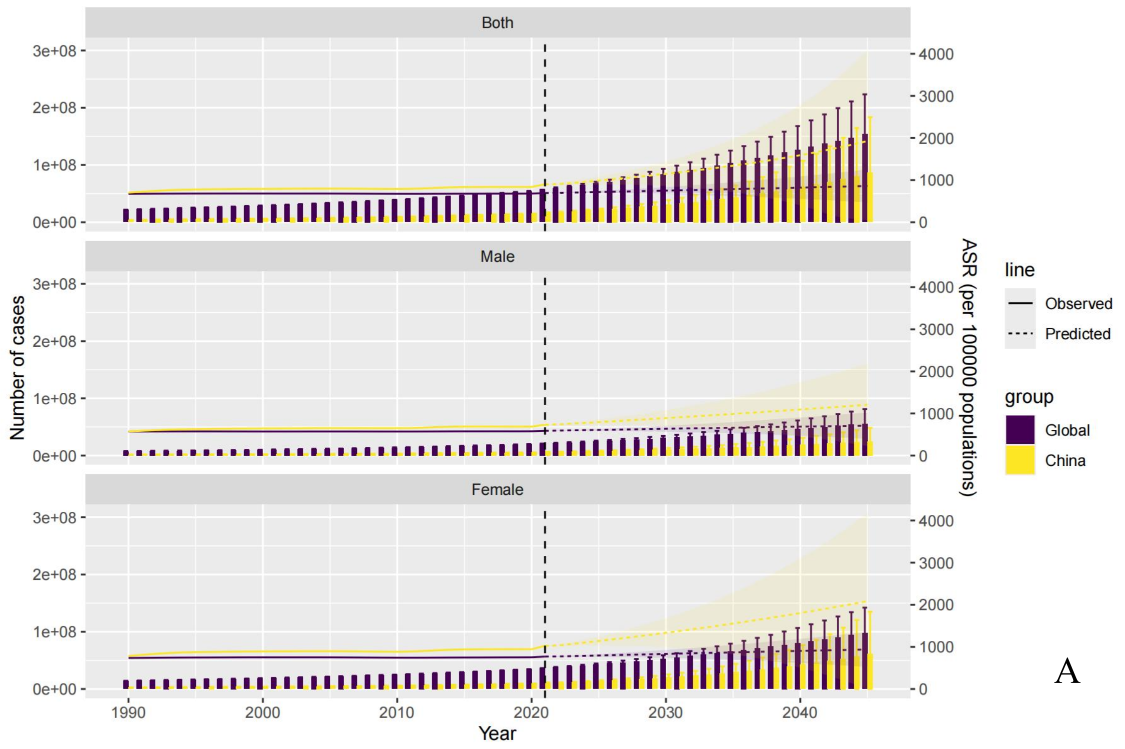

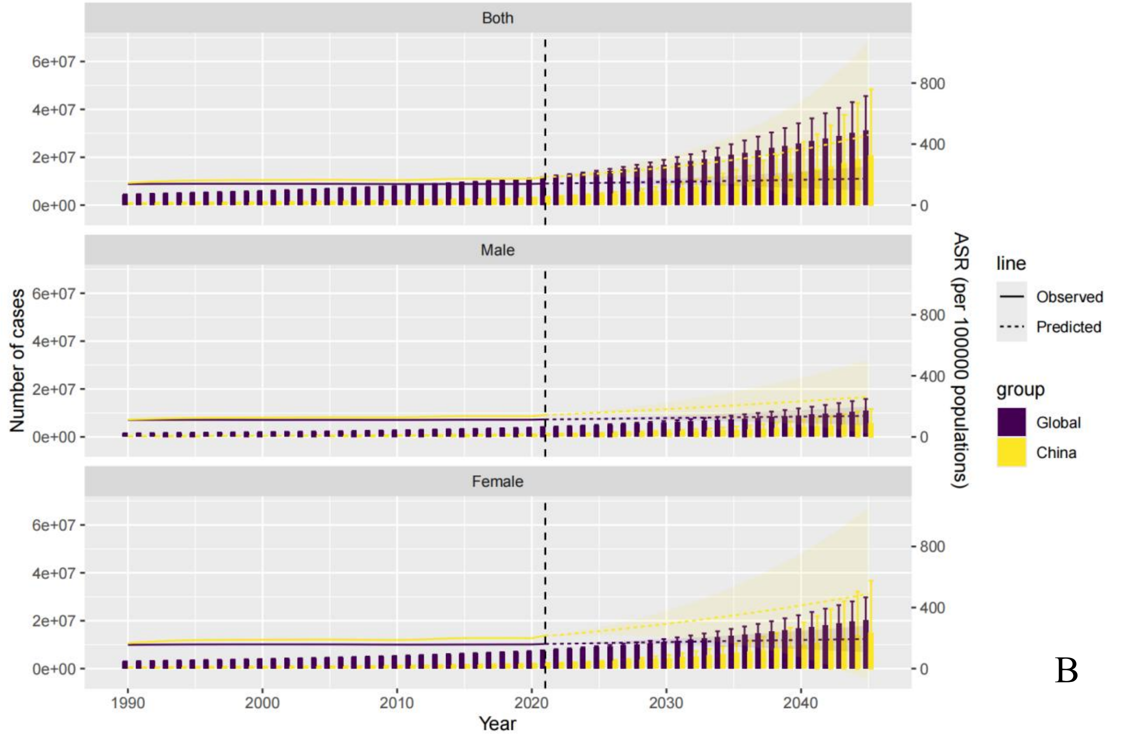

Fig. S153 (A) Future forecasts of Global Burden of Disease in Alzheimer's disease and other dementias burden based on prevalence using bayesian age-period-cohort model; (B) Future forecasts of Global Burden of Disease in Alzheimer's disease and other dementias burden based on years lived with disability using bayesian age-period-cohort model.

Notes: The line graphs show the change of age-standardized rates (ASR), and the bar graphs show the change of burden.

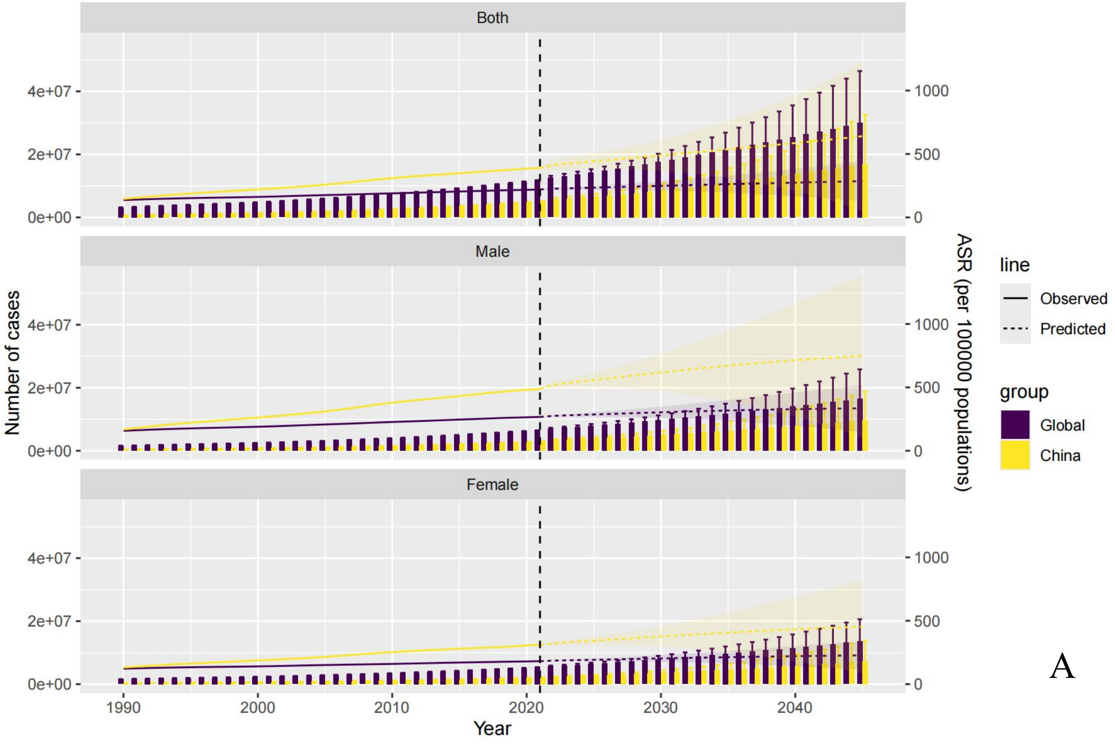

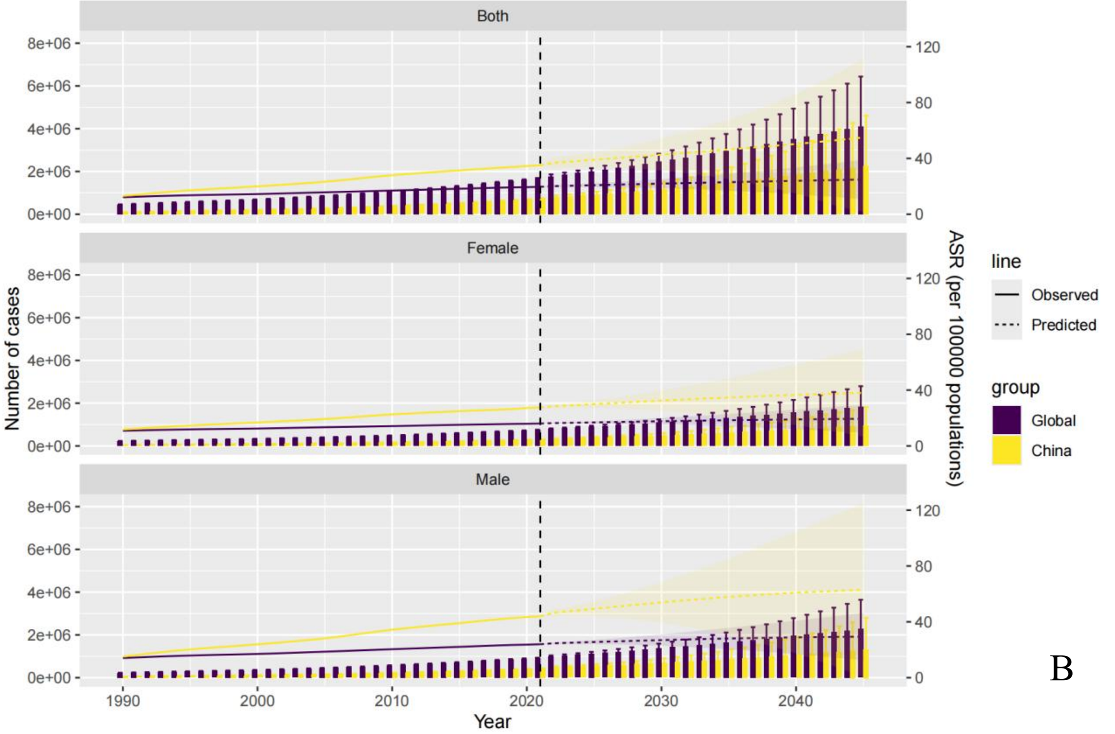

Fig. S154 (A) Future forecasts of Global Burden of Disease in Parkinson's disease burden based on prevalence using bayesian age-period-cohort model; (B) Future forecasts of Global Burden of Disease in Parkinson's disease burden based on years lived with disability using bayesian age-period-cohort model.

Notes: The line graphs show the change of age-standardized rates (ASR), and the bar graphs show the change of burden.

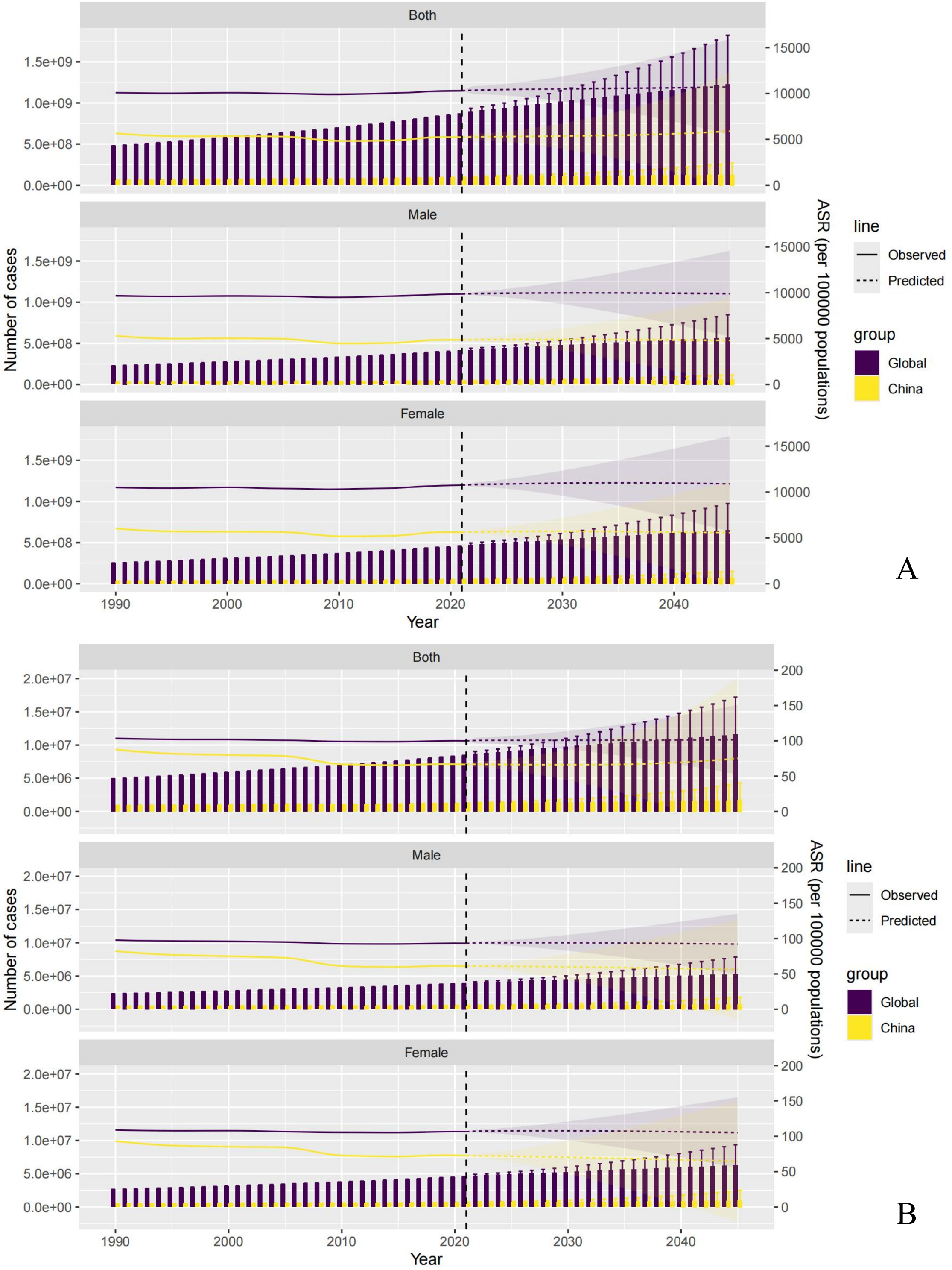

Fig. S155 (A) Future forecasts of Global Burden of Disease in upper digestive system diseases burden based on prevalence using bayesian age-period-cohort model; (B) Future forecasts of Global Burden of Disease in upper digestive system diseases burden based on years lived with disability using bayesian age-period-cohort model.

Notes: The line graphs show the change of age-standardized rates (ASR), and the bar graphs show the change of burden.

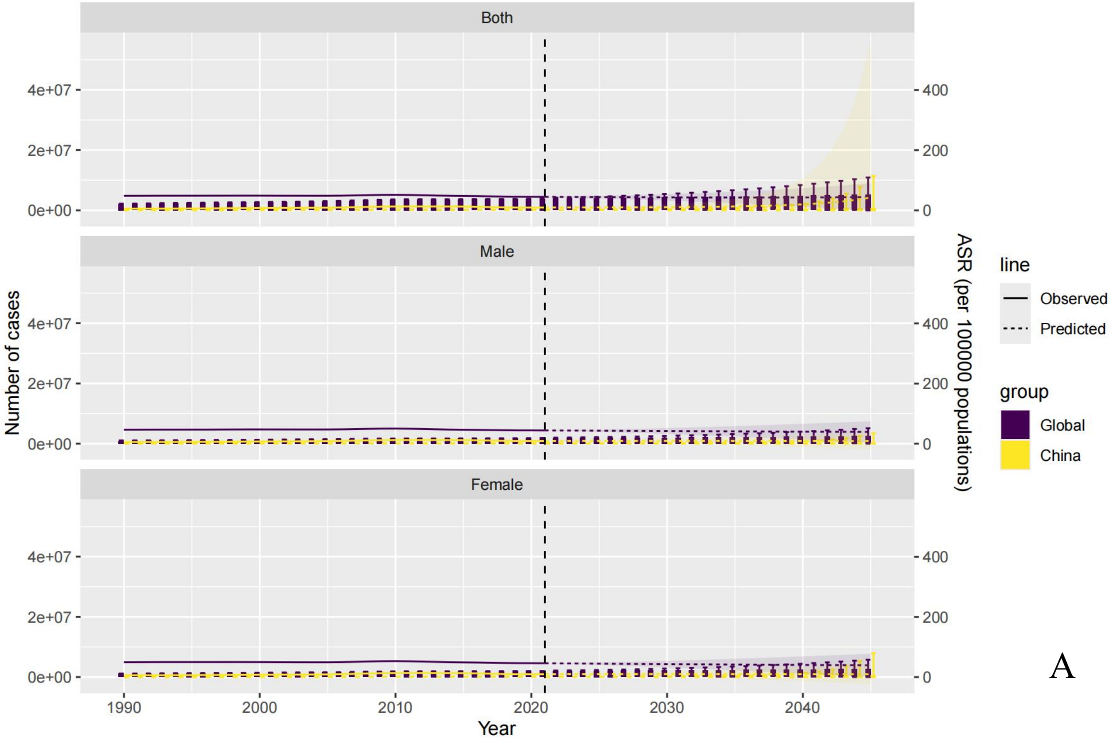

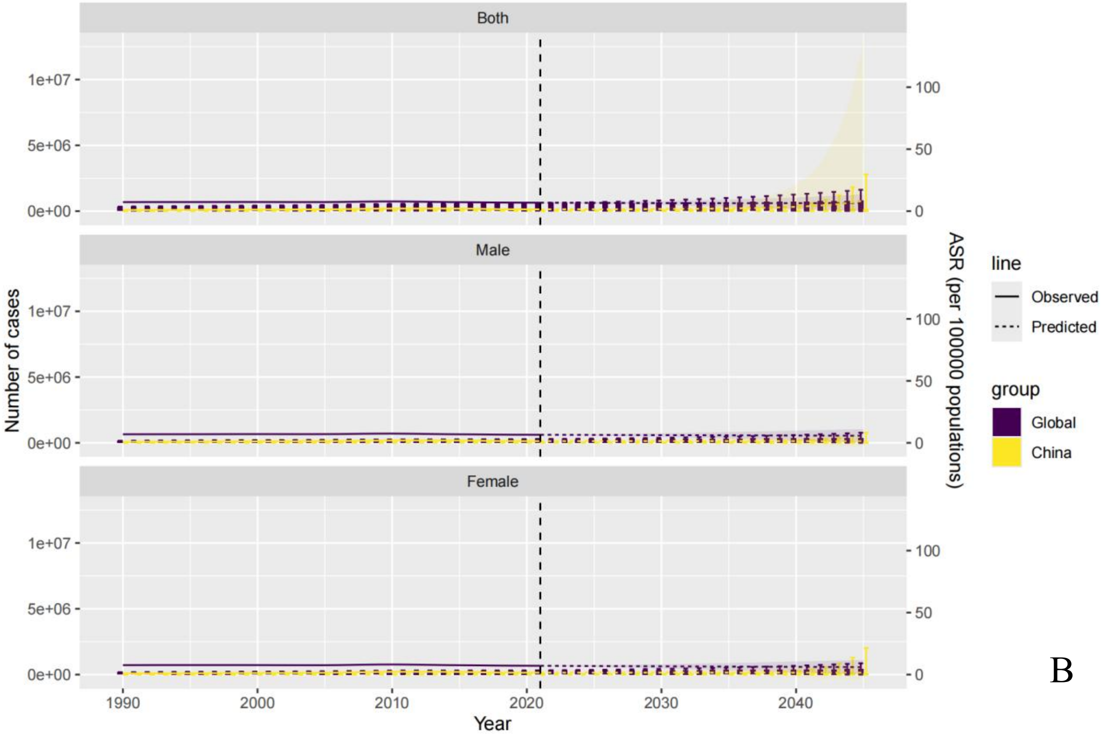

Fig. S156 (A) Future forecasts of Global Burden of Disease in inflammatory bowel disease burden based on prevalence using bayesian age-period-cohort model; (B) Future forecasts of Global Burden of Disease in inflammatory bowel disease burden based on years lived with disability using bayesian age-period-cohort model.

Notes: The line graphs show the change of age-standardized rates (ASR), and the bar graphs show the change of burden.

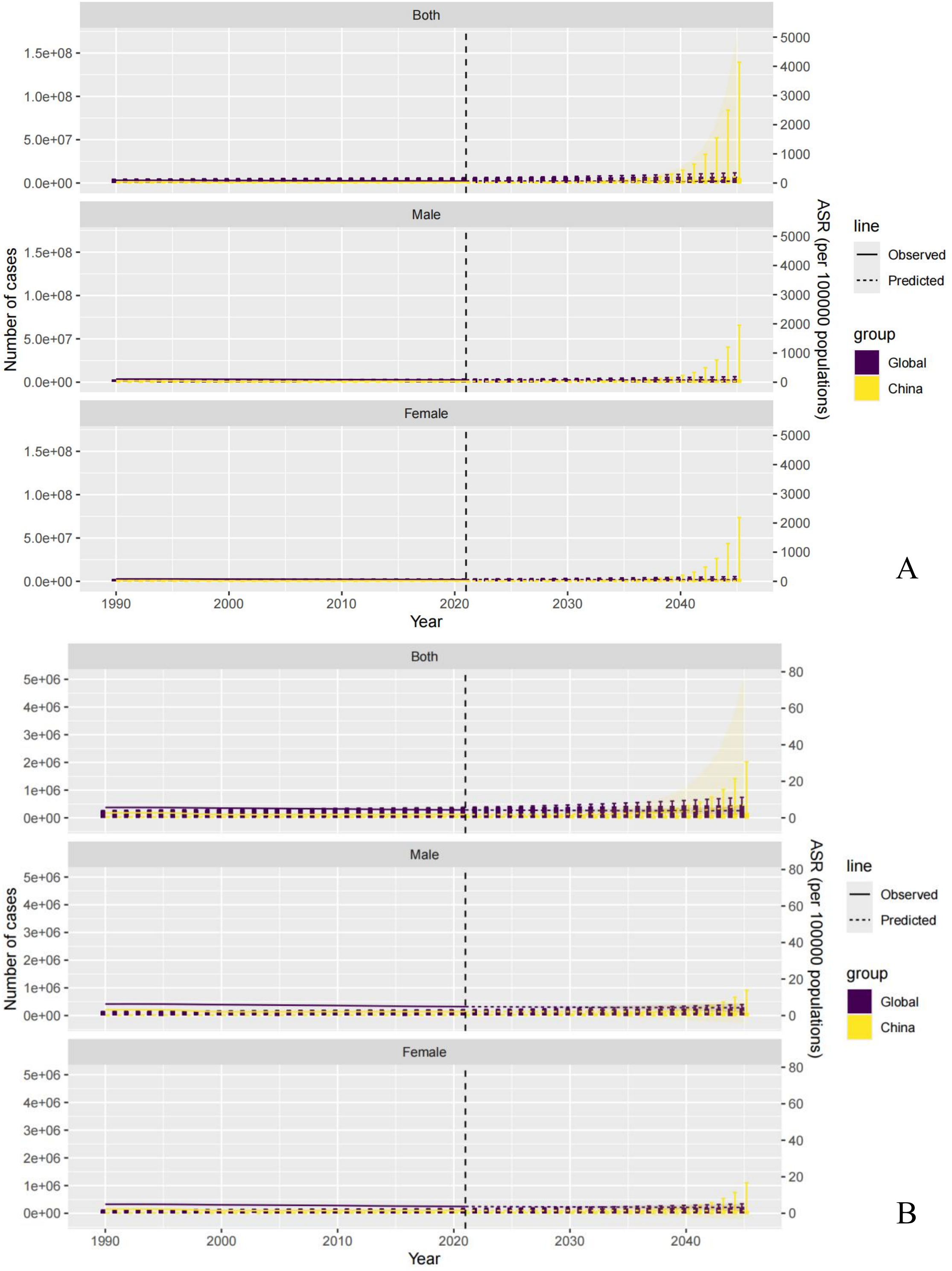

Fig. S157 (A) Future forecasts of Global Burden of Disease in pancreatitis burden based on prevalence using bayesian age-period-cohort model; (B) Future forecasts of Global Burden of Disease in pancreatitis burden based on years lived with disability using bayesian age-period-cohort model.

Notes: The line graphs show the change of age-standardized rates (ASR), and the bar graphs show the change of burden.

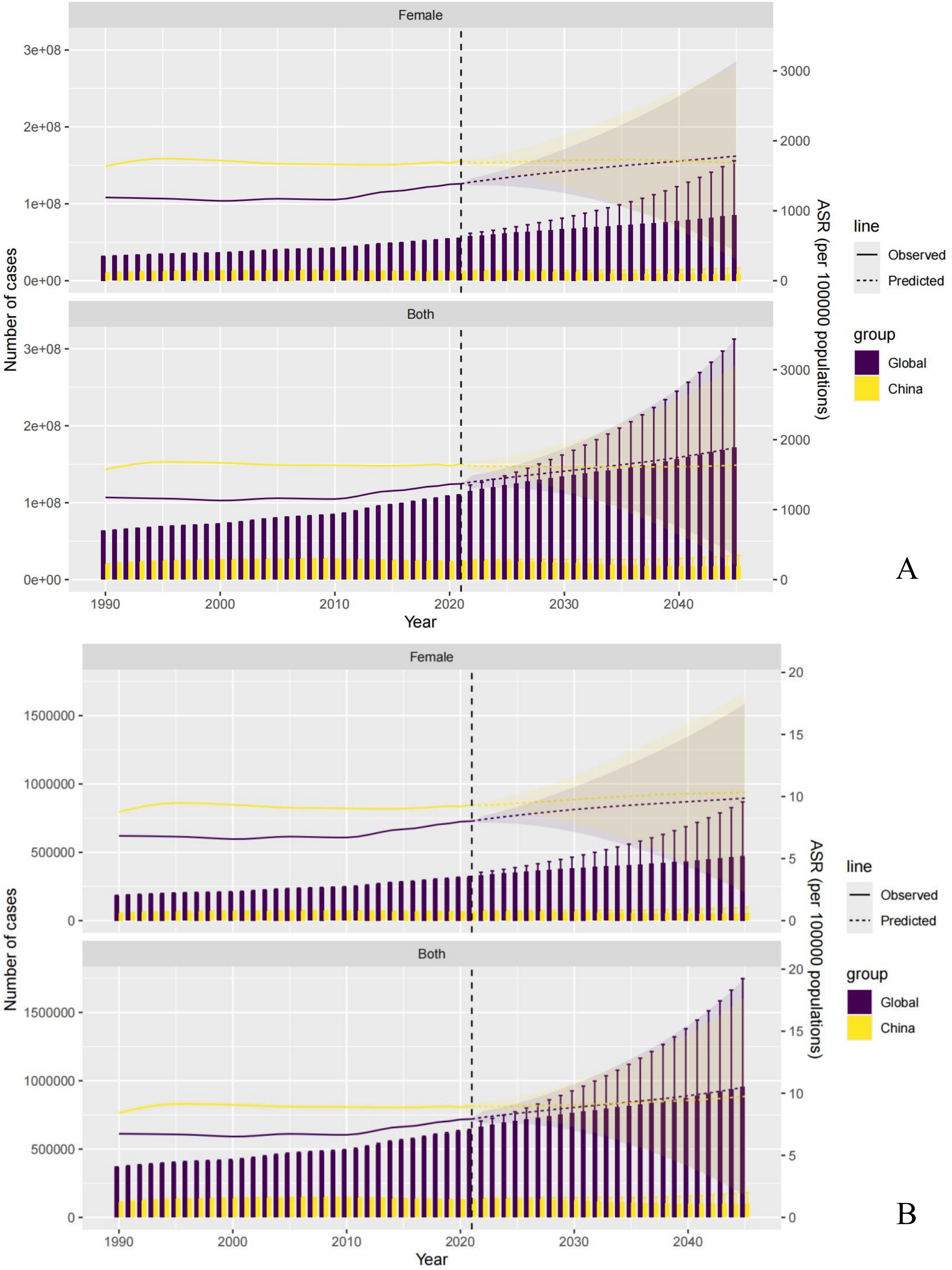

Fig. S158 (A) Future forecasts of Global Burden of Disease in male infertility burden based on prevalence using bayesian age-period-cohort model; (B) Future forecasts of Global Burden of Disease in male infertility burden based on years lived with disability using bayesian age-period-cohort model.
Notes: The line graphs show the change of age-standardized rates (ASR), and the bar graphs show the change of burden.

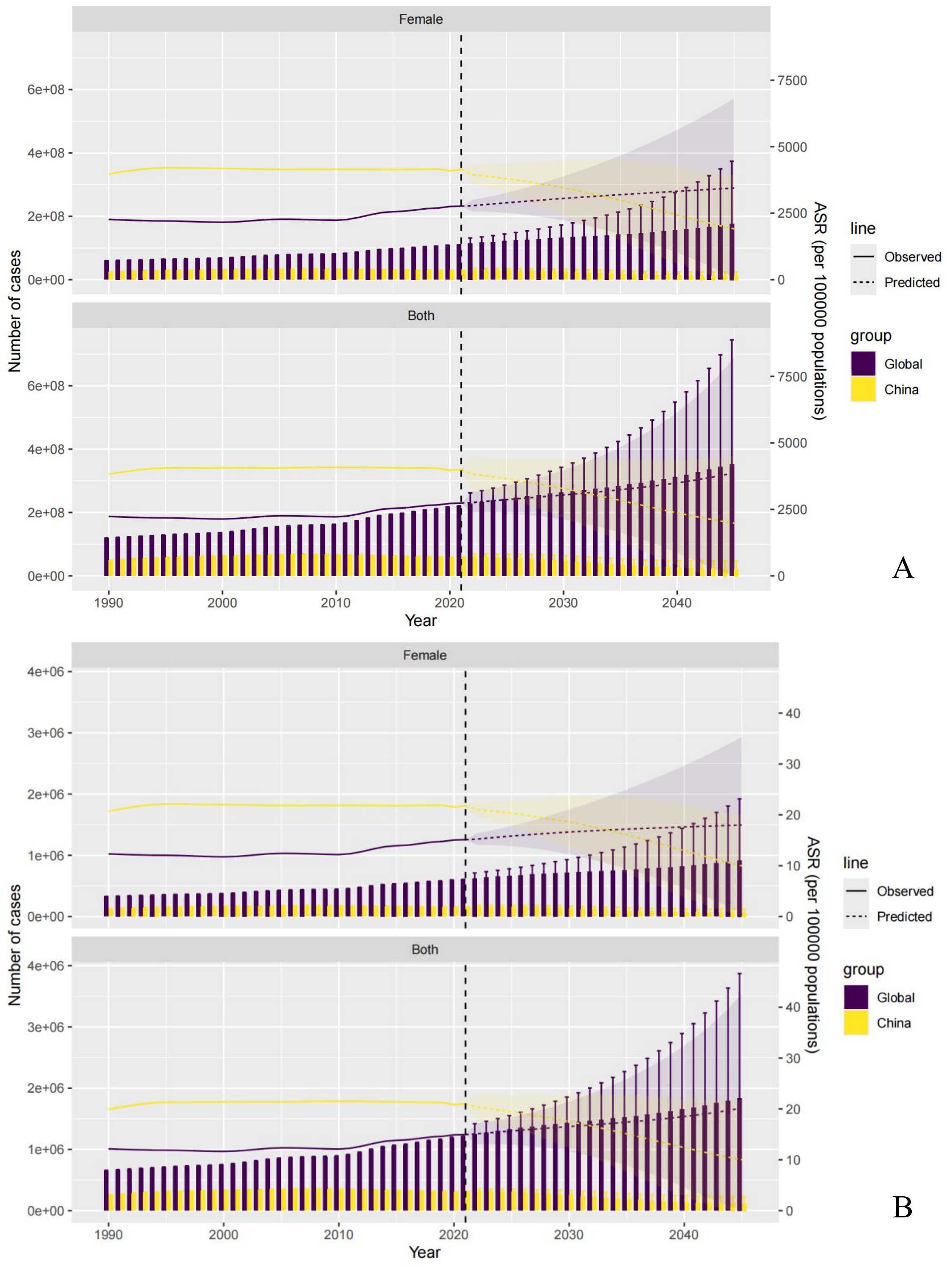

Fig. S159 (A) Future forecasts of Global Burden of Disease in female infertility burden based on prevalence using bayesian age-period-cohort model; (B) Future forecasts of Global Burden of Disease in female infertility burden based on years lived with disability using bayesian age-period-cohort model.

Notes: The line graphs show the change of age-standardized rates (ASR), and the bar graphs show the change of burden.

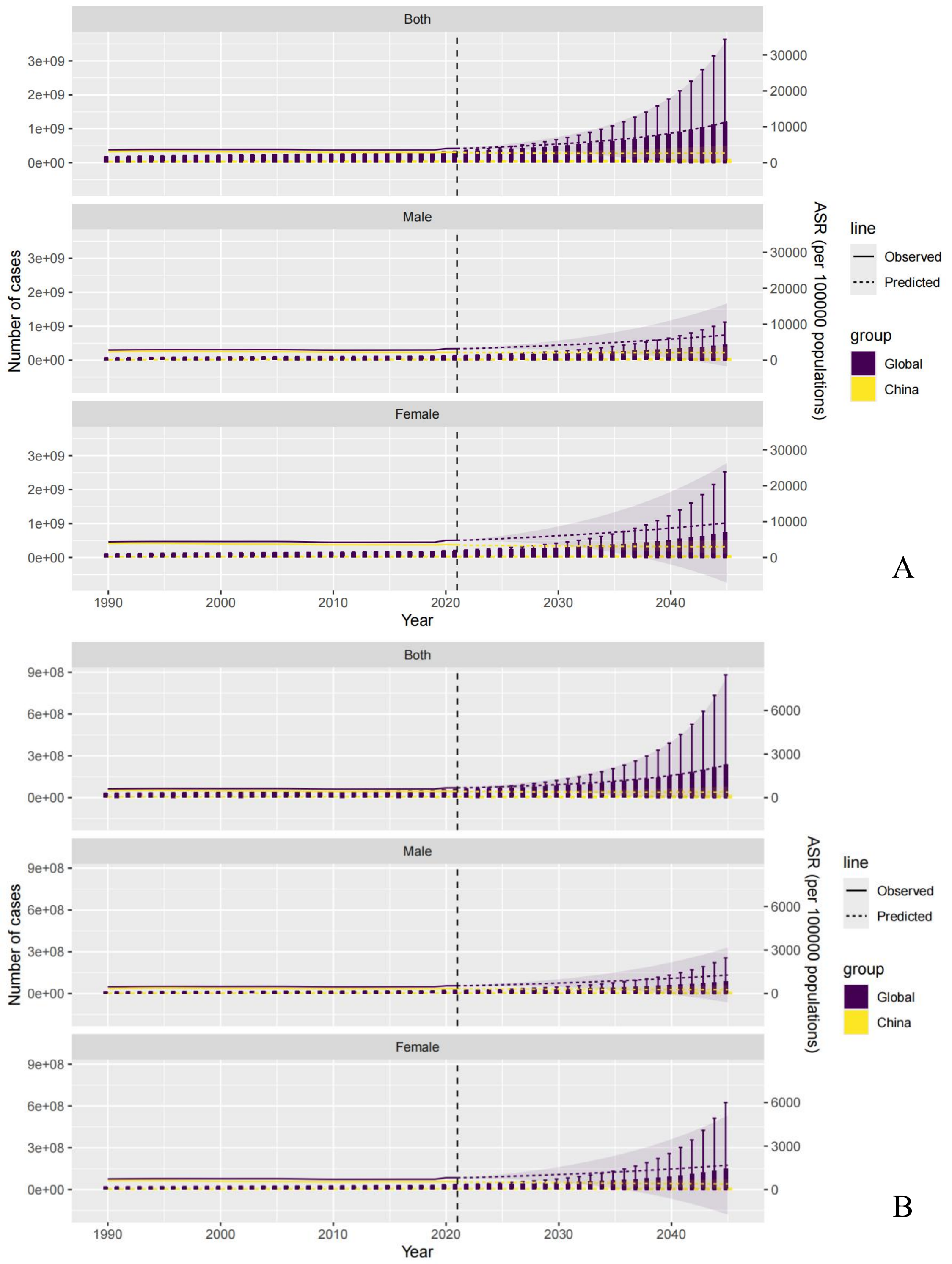

Fig. S160 (A) Future forecasts of Global Burden of Disease in depressive disorders burden based on prevalence using bayesian age-period-cohort model; (B) Future forecasts of Global Burden of Disease in depressive disorders burden based on years lived with disability using bayesian age-period-cohort model.

Notes: The line graphs show the change of age-standardized rates (ASR), and the bar graphs show the change of burden.

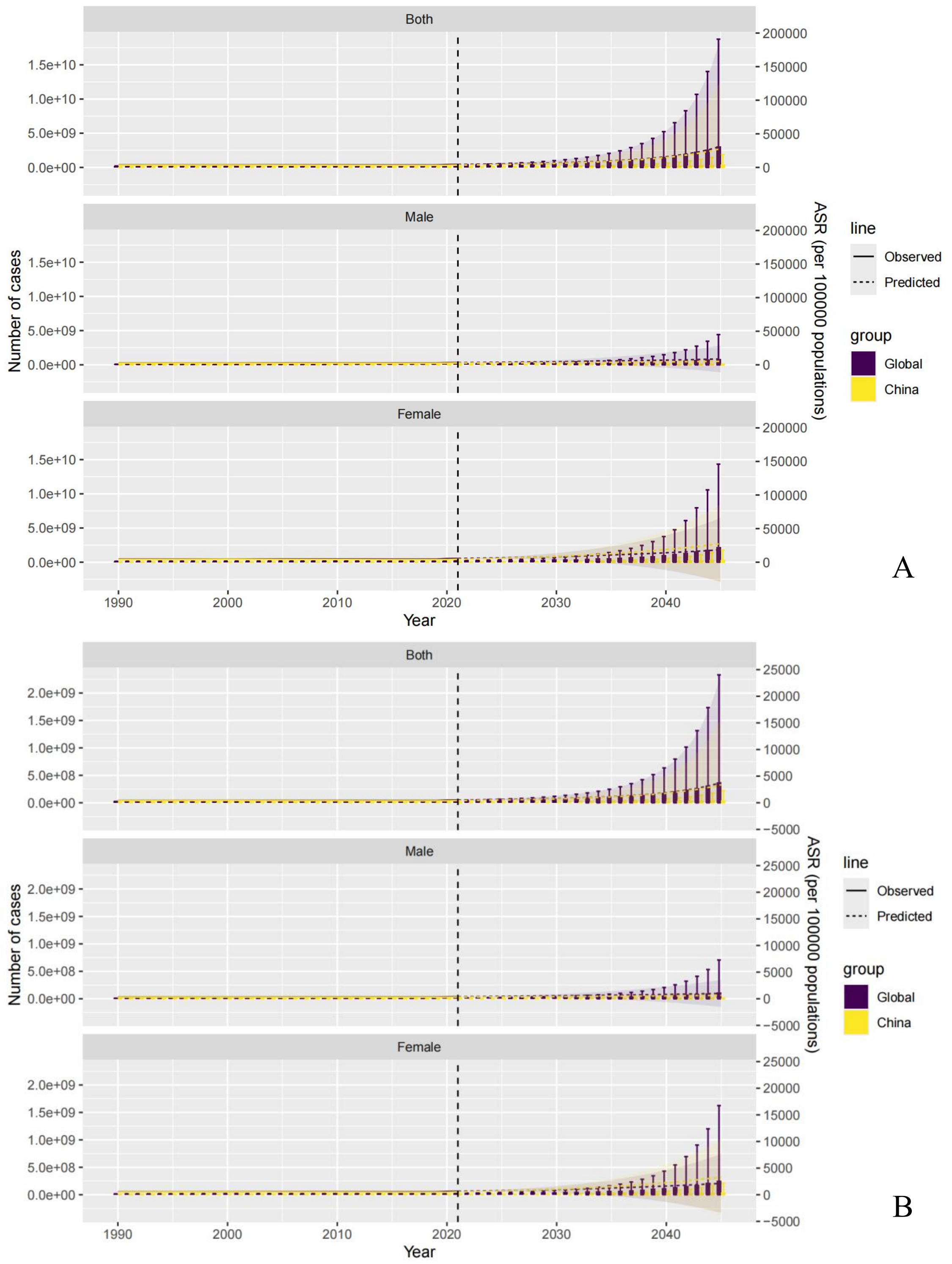

Fig. S161 (A) Future forecasts of Global Burden of Disease in anxiety disorders burden based on prevalence using bayesian age-period-cohort model; (B) Future forecasts of Global Burden of Disease in anxiety disorders burden based on years lived with disability using bayesian age-period-cohort model.

Notes: The line graphs show the change of age-standardized rates (ASR), and the bar graphs show the change of burden.

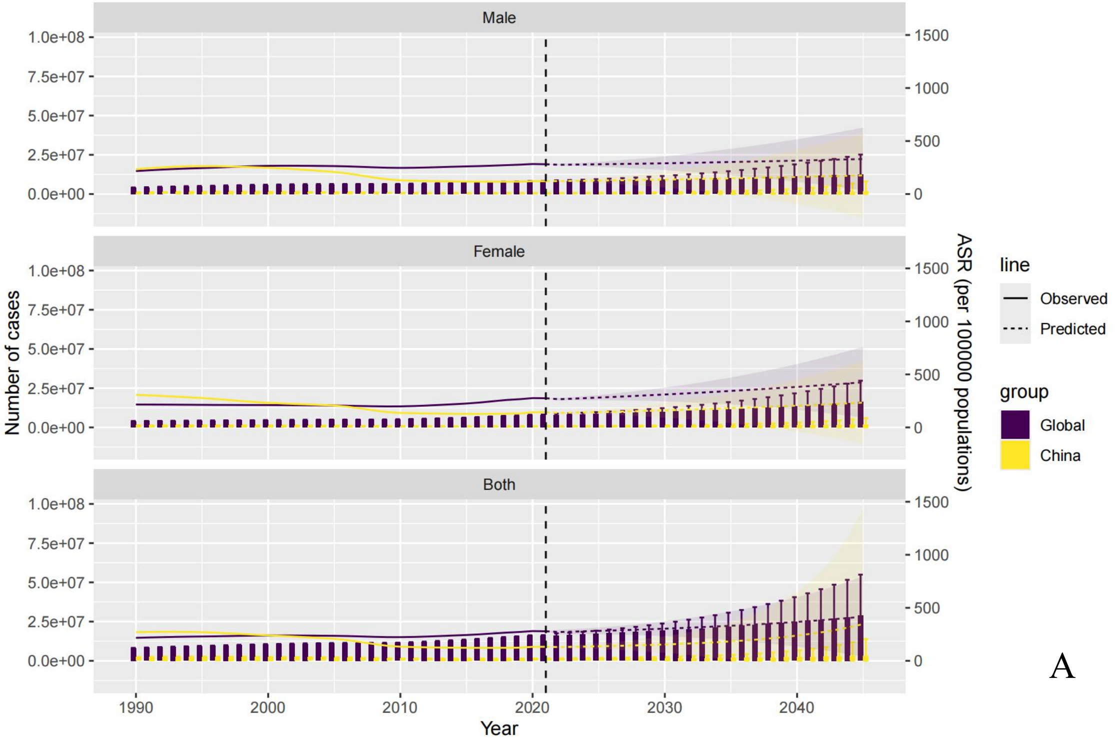

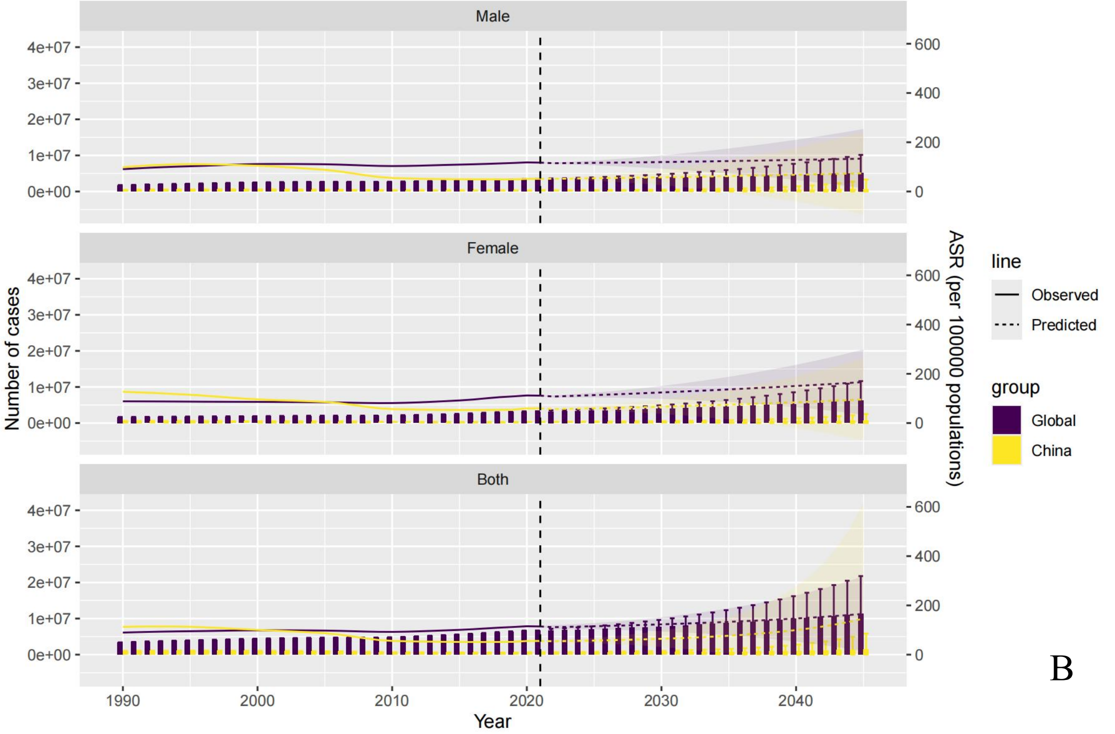

Fig. S162 (A) Future forecasts of Global Burden of Disease in opioid use disorders burden based on prevalence using bayesian age-period-cohort model; (B) Future forecasts of Global Burden of Disease in opioid use disorders burden based on years lived with disability using bayesian age-period-cohort model.

Notes: The line graphs show the change of age-standardized rates (ASR), and the bar graphs show the change of burden.

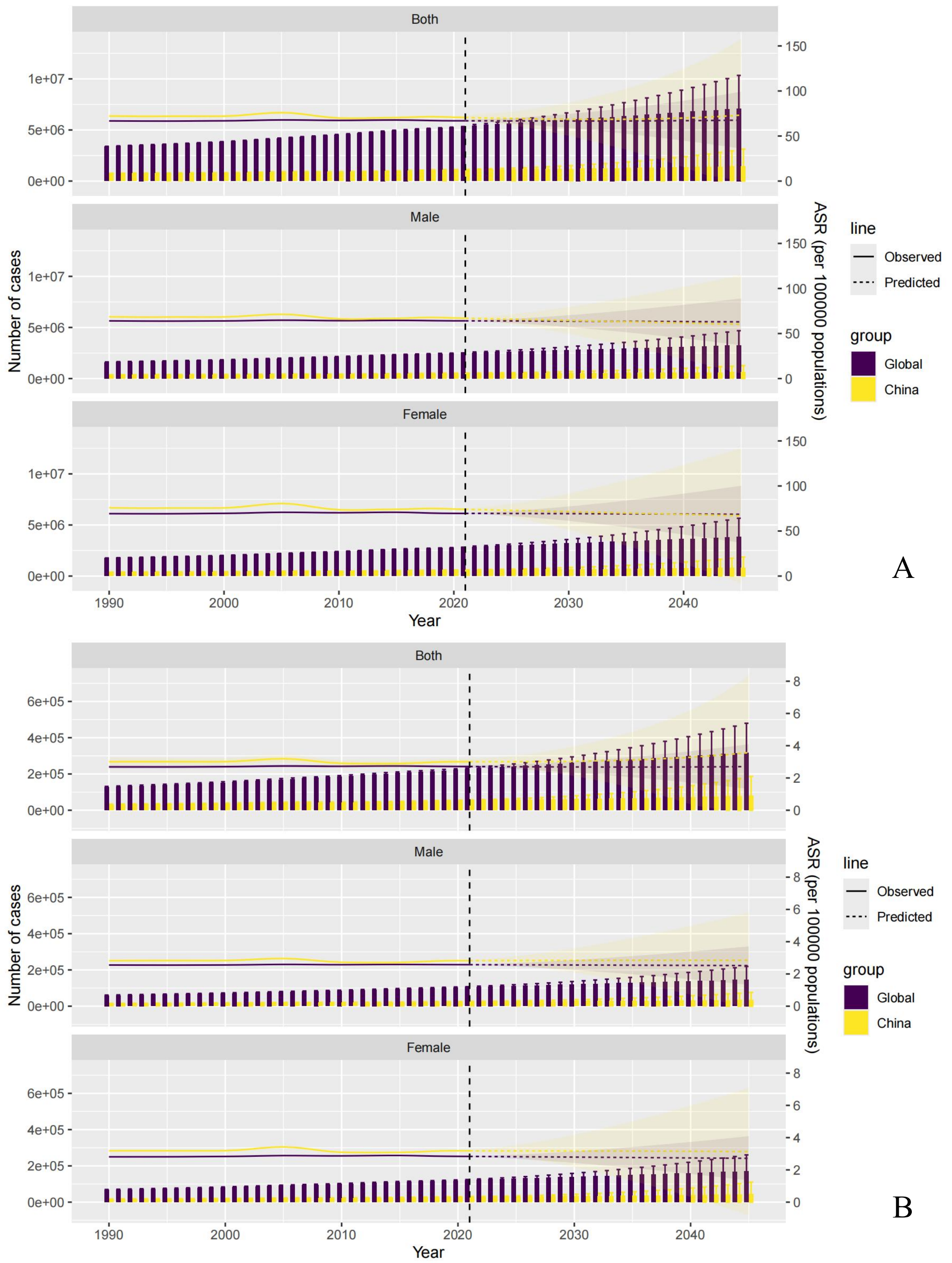

Fig. S163 (A) Future forecasts of Global Burden of Disease in varicella and herpes zoster burden based on prevalence using bayesian age-period-cohort model; (B) Future forecasts of Global Burden of Disease in varicella and herpes zoster burden based on years lived with disability using bayesian age-period-cohort model.

Notes: The line graphs show the change of age-standardized rates (ASR), and the bar graphs show the change of burden.
